# Supplementary material for: Identifying restrictions in the order of accumulation of mutations during tumor progression: effects of passengers, evolutionary models, and sampling
Source: BMC Bioinformatics. 2015 Feb 12;16:41. doi: 10.1186/s12859-015-0466-7 (PMC4339747; doi:10.1186/s12859-015-0466-7)
Supplement: Additional file 1 — Supplementary methods. Additional details about: a) experimental design; b) evolutionary models and their simulation; c) the true graphs used; d) methods and software for inferring restrictions (CBN, OT, DiP); e) computation of performance measures; f) multiple comparisons with the best (MCB); g) generalized linear mixed models; h) availability of code, scripts and full model fits. [file 12859_2015_466_MOESM1_ESM.pdf]

Additional file 1. Supplementary Methods.

Supplementary material for "Identifying Restrictions in the Order of Accumulation of Mutations during Tumor Progression: Effects of Passengers, Evolutionary Models, and Sampling"

Ramon Diaz-Uriarte
Dept. Biochemistry, Universidad Autónoma de Madrid
Instituto de Investigaciones Biomédicas "Alberto Sols" (UAM-CSIC)
Madrid, Spain

ramon.diaz@iib.uam.es

rdiaz02@gmail.com

http://ligarto.org/rdiaz

Contents

1 Experimental design 4

2 Evolutionary models and their simulation 8
- 2.1 Evolutionary models . . . 8
- 2.2 Simulation . . . 10
- 2.3 Examples of simulated cancer progression trajectories . . . 11

3 Graphs (oncogenetic trees and CBNs) used 13

4 Methods and software: CBN/DiP/OT 15

5 Measuring performance: performance measures 16

6 Overall ranking of Filtering, Method, and Sampling scheme 18

7 Multiple comparisons with the best (MCB) 19
- 7.1 Constructing the confidence sets summary tables . . . 20

8 Generalized linear mixed modeling (GLMM) of performance measures. 21

9 Why GLMs, MCB, and ranking? 23

10 Availability of scripts, code, and model fits 23

List of Tables

1 Factors considered and their levels or possible values, together with acronyms used through the text. The within-data set factors, Filtering and Method (see text), are shown in italics. All other factors are among-data set factors. Sampling scheme, used through the text, refers to when (S.Time) and how (S.Type) we sample. 7

2 Main parameters for each of the tumor progression models. j is the number of drivers with their dependencies met, and p the number of drivers with dependencies not met. In all cases $s = 0.1$. s_h is set to either 0 (so it has no effect) or ∞ (so fitness of that clone is 0). N: population size. $K = 2000$. $^+$ This is really $b_j = \max(0, (1+s)^j(1-s_h)^p)$. This is the same table as provided in the ms. 9

3 Proportion of simulations that result in cancer by Graph, sh, and evolutionary Model. 10

List of Figures

1 Inferring order restrictions. (a) Main steps in the analysis of patient data. (b) Main steps used in this paper for the generation (simulation) of data and its analysis. Terms in monospaced blue font are those in Table 1, and terms in italics, as in Table 1, correspond to within-data set factors. Numbers indicate the chronological order of the steps. In step 1, cancer development is simulated for the specified values of Model, sh, and True Graph. This simulation generates tumor cell data for the equivalent of a single patient in panel (a). In step 2, data for S.Size patients are sampled (cross-sectional sampling) according to the settings of S.Time and S.Type, producing a data set (a collection of genotypes: a matrix of subjects by genes). If the identity of the true drivers is not known, Filtering in step 3 removes from the data set the genes that do not meet certain frequency criteria. The data set is then passed on, in step 4, to one of the specified methods to infer the graph that encodes the order restrictions. This inferred graph is compared, in step 5, with the true graph (which was used in step 1 to generate the cancer cell data) yielding the four performance measures Diff, PFD, PND and FPF. The process illustrated here was repeated 20 times for all possible combinations of Model, sh, True Graph, S.Time, S.Type, S.Size. Every data set was subject to all Filtering procedures and analyzed with all six Methods. 5
2 Alternative schematic representation of the key steps of design and analysis, and illustration of the within- and among-data set factors. Terms in magenta are those in Table 1 of the manuscript (reproduced below as 1), and terms in italics, as in Table 1 of the manuscript (reproduced below as 1) , correspond to within-data set entries. Terms in blue denote the main steps, with numbers indicating chronological order (Filter and Method are both steps and entries in the table). The process illustrated here was repeated 20 times for all possible combinations of Model, sh, True Graph, S.Time, S.Type, S.Size. 6
3 Examples of three randomly chosen simulations that reach cancer for each model, all with graph 11-A and $sh = Inf$. Thick lines are the sum of all clones with the specified number of drivers, whereas thin lines, with dotted line type, show individual clones. For the Bozic and exp models, the y-axis is shown in log scale. 12
4 The six graphs (oncogenetic trees and CBNs) used. 14

1 Experimental design

The experimental design in this paper combines within- and among-data set factors. Method (Drivers Known scenario) or Filtering by Method combinations (Drivers Unknown scenario) were applied to a data set (Figure 1 of the ms. reproduced below as Figure 1 and Figure 2): a matrix of genes by subjects. Each simulation run, when we sample from it, produces the observed genotype of a subject, and a data set is made from the genotypes of multiple subjects (that are sampled in the same way). The same data set can be analyzed with different Methods (or different Filtering by Method combinations).

For other factors (e.g., Model) different settings of the factor (and reruns of the same settings with different seeds of the random number generator) produce different data sets. This allows us to use an experimental design with among- and within-data set factors so as to examine the effect of Method and Filtering, controlling for possible among-data set variation (as explained below), and so as to minimize computational costs.

For factors Model, sh,True Graph (= Number of Nodes * Conjunction), S.Size, S.Type, and S.Time, the among-data set factors, I used a full factorial design (thus, $4 * 2 * 3 * 2 * 3 * 3 * 2 = 864$ among-data set factor combinations). For every combination of the among-data set factors I used twenty independent replicate data sets. Each of the twenty replicate data sets was analyzed with every Method or every Filtering by Method combination (the within-data set factors) to infer a graph from the data (i.e., to try to infer the order restrictions among events). Therefore, a total of $864 * 6 = 5184$ or $864 * 6 * 4 = 20736$ factor combinations for the Drivers Known and Drivers Unknown scenarios, respectively, were examined. Every data set was obtained by independently sampling different (and independently generated) simulated tumor trajectories, according to the settings of the among-data set factors. For instance, if sample size was set to 1000, I simulated 1000 tumor progression trajectories as specified by the settings of Model, sh, and True Graph, and sampled each of those trajectories as specified by the settings of S.Time and S.Type (see Figure 1 and 2). This process was repeated at every one of the 864 among-data set factors (and, thus, the results shown here are based on 7.49 million simulated trajectories = $4*2*3*2*3*2*20*(1000+200+100)$).

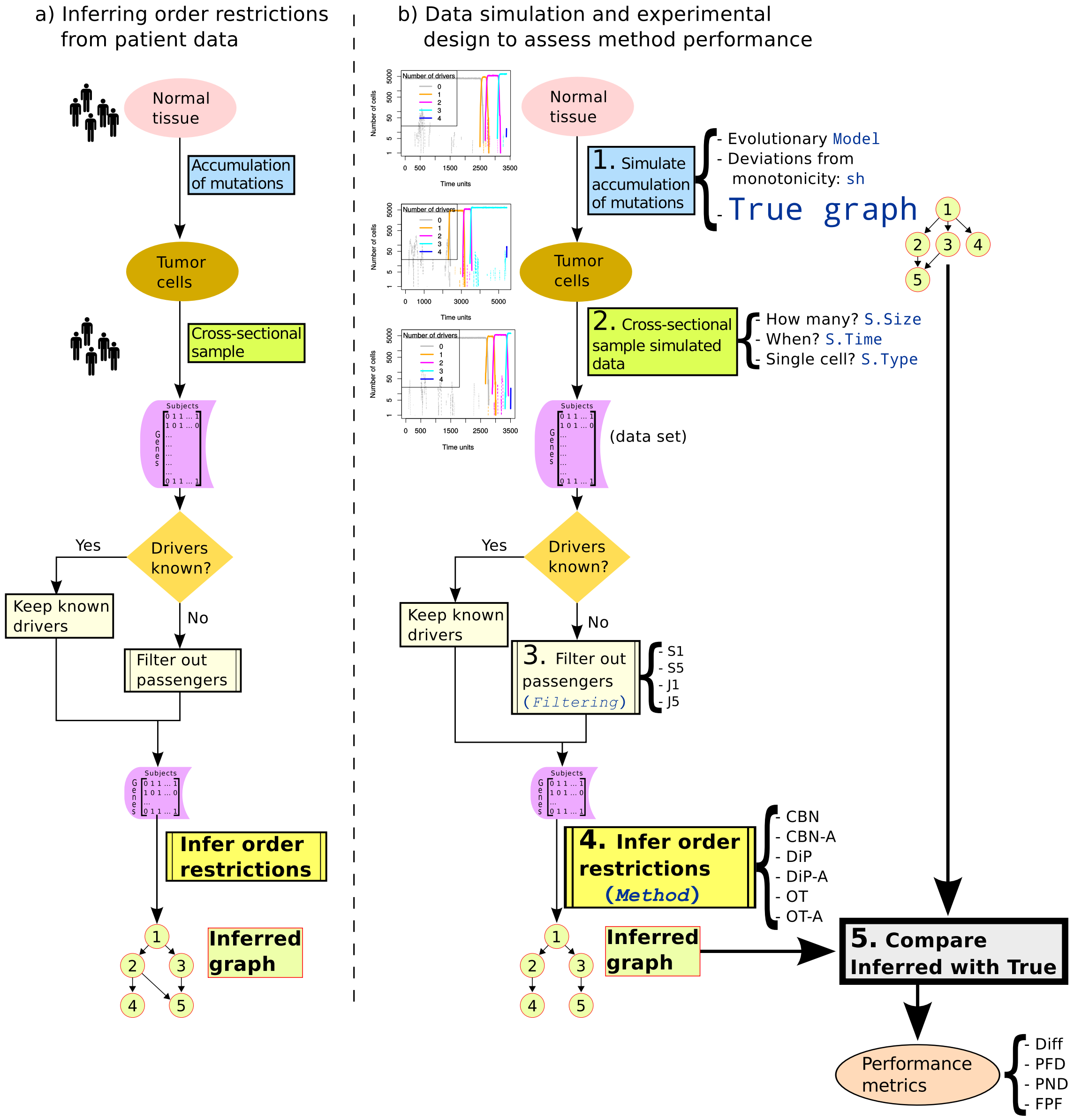

Figure 1: Inferring order restrictions. (a) Main steps in the analysis of patient data. (b) Main steps used in this paper for the generation (simulation) of data and its analysis. Terms in monospaced blue font are those in Table 1, and terms in italics, as in Table 1, correspond to within-data set factors. Numbers indicate the chronological order of the steps. In step 1, cancer development is simulated for the specified values of Model, sh, and True Graph. This simulation generates tumor cell data for the equivalent of a single patient in panel (a). In step 2, data for S.Size patients are sampled (cross-sectional sampling) according to the settings of S.Time and S.Type, producing a data set (a collection of genotypes: a matrix of subjects by genes). If the identity of the true drivers is not known, Filtering in step 3 removes from the data set the genes that do not meet certain frequency criteria. The data set is then passed on, in step 4, to one of the specified methods to infer the graph that encodes the order restrictions. This inferred graph is compared, in step 5, with the true graph (which was used in step 1 to generate the cancer cell data) yielding the four performance measures Diff, PFD, PND and FPF. The process illustrated here was repeated 20 times for all possible combinations of Model, sh, True Graph, S.Time, S.Type, S.Size. Every data set was subject to all Filtering procedures and analyzed with all six Methods.

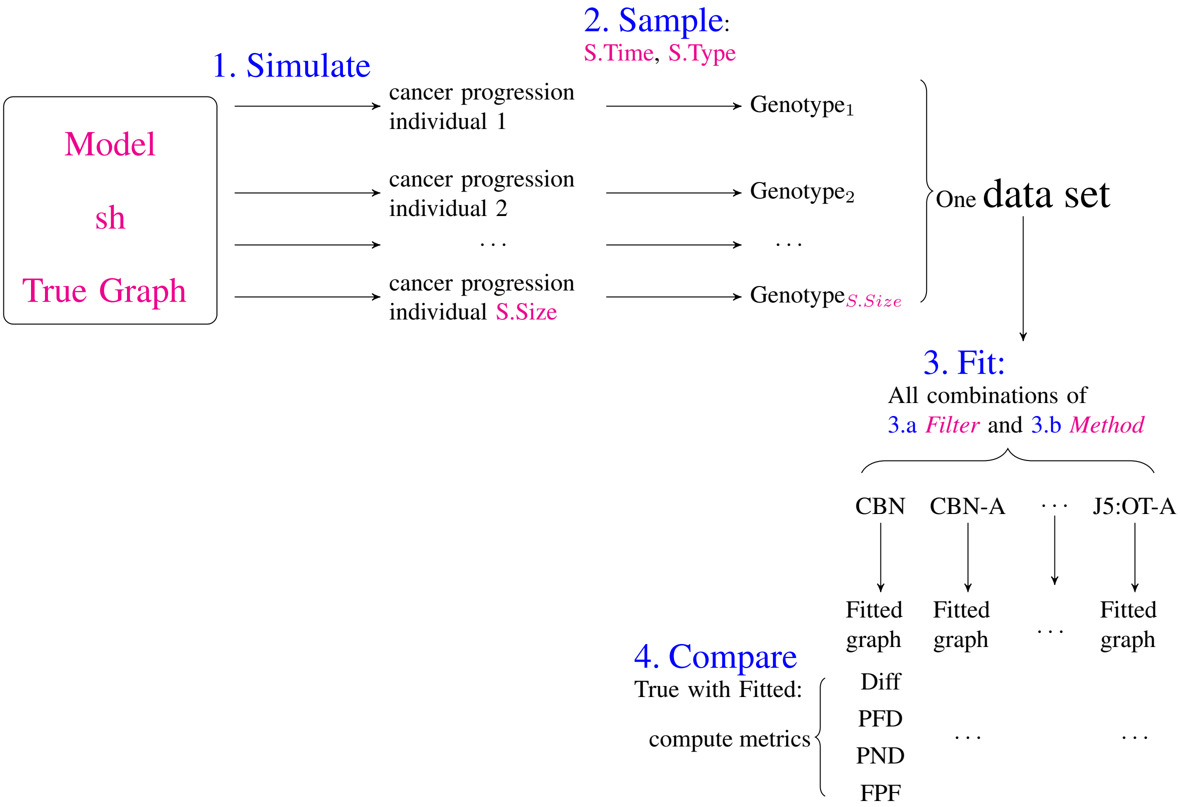

Figure 2: Alternative schematic representation of the key steps of design and analysis, and illustration of the within- and among-data set factors. Terms in magenta are those in Table 1 of the manuscript (reproduced below as 1), and terms in italics, as in Table 1 of the manuscript (reproduced below as 1) , correspond to within-data set entries. Terms in blue denote the main steps, with numbers indicating chronological order (Filter and Method are both steps and entries in the table). The process illustrated here was repeated 20 times for all possible combinations of Model, sh, True Graph, S.Time, S.Type, S.Size.

Table 1: Factors considered and their levels or possible values, together with acronyms used through the text. The within-data set factors, Filtering and Method (see text), are shown in italics. All other factors are among-data set factors. Sampling scheme, used through the text, refers to when (S.Time) and how (S.Type) we sample.

Factor	Description	Values
Model	Evolutionary model of cancer progression	exp, Bozic, McF_4, McF_6
sh	Penalization of deviations from monotonicity	0, Inf (for ∞)
True Graph	The true graph: the structure that encodes the order restrictions. All possible combinations of Number of nodes and Conjunction	11-A, 11-B, 9-A, 9-B, 7-A, 7-B
Number of nodes (NumNodes)	Number of genes or alterations	11, 9, 7
Conjunction	Whether or not the graph has conjunctions	Yes, No
Sample size (S.Size)	Number of samples used for reconstructing the graph	100, 200, 1000
Sampling time (S.Time)	When the sample is taken	Last, unif (for uniform)
Sampling type (S.Type)	How tissue is collected	singleC (for single cell), wholeT_0.5 (whole tumor, detection threshold=0.5), wholeT_0.01 (whole tumor, detection threshold=0.1)
Filtering	Method for selecting drivers, or filtering passengers, when the true drivers are not known	S1, S5, J1, J5 (for frequency of Single event and Joint frequency of events, with thresholds 1% and 5% respectively)
Method	Method for inferring the order restrictions	CBN, CBN-A, DiP, DiP-A, OT, OT-A

2 Evolutionary models and their simulation

2.1 Evolutionary models

As in the ms., Table 2 summarizes the main parameters of the models used. Below I provided details of the models and values of the parameters used.

The model we will call "Bozic" is based on Bozic *et al.* (2010), one of the first papers to explicitly model both drivers and passengers. When cancer develops (i.e., when drivers accumulate), this is a model that leads to exponential growth. Here I use the second continuous-time version of their model (see p. 5 of their supplementary material): birth rate is constant and equal to 1 and death rate is $d_j = (1-s)^j$, where s is the selection coefficient and j is the number of drivers. I set $s = 0.1$, a value within the range considered in table S1 of the supplementary material in Bozic *et al.* (2010). The mutation rate per gene per unit time, μ, is set to 10^{-6} which leads to each daughter cell having, at the start of the process, a probability of a change in at least one driver of about 10^{-5}, very similar to the value of $u = 3.4 * 10^{-5}$ given in p. 18546 in Bozic *et al.* (2010) and well within the range of values in their table S1. In Bozic *et al.* (2010) "The process is initiated with a single surviving founder cell with one driver mutation." Here, however, the simulation starts from a population without any mutated driver (or any mutated passenger): Tomasetti *et al.* (2013) show that by the time the first driver mutation appears passengers could have accumulated, and in the scenario where the identity of drivers is not know, it is crucial for us to allow for this effect (as it could make separation of drivers and passengers by simple frequency statistics harder). Simulations start with an initial population of size $N = 500$ (although, of course, the population size will be one for the first cell with one driver, as in Bozic *et al.* (2010)). Simulations are stopped when the population reaches a size of 10^9 cell, as in Beerenwinkel *et al.* (2007), or when 25 years (more precisely, $(1/4) * 25 * 365$ time units) have passed, whichever comes first; if population size does not reach 10^9 cells, that simulation is discarded. Detection size is larger than the one used in Bozic *et al.* (2010), for two reasons: first, Bozic *et al.* (2010) start the process from a cell that already has one driver mutation and, second, we want to give simulations a chance to accumulate, in at least some cases, a large number of drivers to avoid penalizing the graphs with 11 drivers. The 1/4 of the time expression reflects that in Bozic *et al.* (2010) events (birth or death) occur at a rate $1/T$ (see p. 6 of their supplementary material), where $T = 4$ is the number of days between cell divisions. With the above parameters, the number of simulations that reach completion ranges between 9 and 20%, depending on graph and sh (see Table 3).

I also use a second model with exponential growth, and refer to this model as "exp". In this model, death rate is constant and equal to 1, and birth rate, b_j, is $(1+s)^j$, where $s = 0.1$ is the selection coefficient and j is the number of drivers. The expression for b_j is the same as the one used by Datta *et al.* (2013) and Beerenwinkel *et al.* (2007) (but those authors use a Fisher-Wright model where the relative fitness of a cell depends on the fitness of the rest of the population). Mutation rate is proportional to growth rate, as in several of the models considered in Mather *et al.* (2012), so that fitter clones evolve faster, with a mutation rate per gene per unit time of $\mu = 10^{-7} b_j$, where the 10^{-7} is like the value used in Beerenwinkel *et al.* (2007), and within the range of values (per division) considered in McFarland *et al.* (2013).

The models denoted "McF_4" and "McF_6" are based on McFarland *et al.* (2013). This is a model that leads to logistic-like behavior, in contrast to the exponential and Bozic models. Here, birth rate, b_j depends on the number of drivers as given by $b_j = (1+s_d)^j$, and death rate increases with population size. In the absence of deleterious passenger effects, this model leads to periods of populations size stasis altered by fast increases in population size that correspond to the acquisition of a driver that sweeps through the population. The original model in McFarland *et al.* (2013) allows for the incorporation of deleterious effects of passenger mutations but here we will assume that passengers neutral (so $s_p = 0$ in their equation 1): this is done for the sake of simplicity and to avoid aliasing strong density dependence with passenger deleterious effects. The model used here uses their second form for death rate, $D = \log(1 + N/K)$, as it allows populations to grow to larger sample sizes. The fitness advantage of a driver, s_d, is set to 0.1, identical to the value used by

Model	Birth rate (b_j)	Death rate (d_j or D_N)	Mutation rate (per gene per unit time)	Cancer reached if
Bozic	1	$(1-s)^j(1+s_h)^p$	10^{-6}	$> 10^9$ cells
exp	$(1+s)^j(1-s_h)^p$ $^{(+)}$	1	$b_j * 10^{-7}$	$> 10^9$ cells
McF_4	$(1+s)^j/(1+s_h)^p$	$\log(1+N/K)$	$5 * 10^{-7}$	Number of drivers ≥ 4
McF_6	$(1+s)^j/(1+s_h)^p$	$\log(1+N/K)$	$5 * 10^{-7}$	Number of drivers ≥ 6

Table 2: Main parameters for each of the tumor progression models. j is the number of drivers with their dependencies met, and p the number of drivers with dependencies not met. In all cases $s = 0.1$. s_h is set to either 0 (so it has no effect) or ∞ (so fitness of that clone is 0). N: population size. $K = 2000$. $^+$ This is really $b_j = \max(0, (1+s)^j(1-s_h)^p)$. This is the same table as provided in the ms.

McFarland *et al.* (2013); this s_d is thus the same as the s of the Bozic model. Mutation rate per gene per unit of time is set to $5 * 10^{-7}$, a value whose magnitude is within the range used in their paper, although larger than their 10 possible activating mutations per gene 10^{-8}: McFarland *et al.* (2013) have a much larger number of potential drivers (70 vs. our maximum of 11), and thus to achieve comparable numbers of mutated drivers in our case, mutation rate should be higher. With our mutation rate mutation rate of $5 * 10^{-7}$, the probability that a daughter cell has one driver mutated is comparable to that of McFarland *et al.* (2013). The model in McFarland *et al.* (2013) only allows mutation events to occur during cell division, whereas in our simulations mutations are not restricted to occur only during division, and the rate is given per unit time; having mutations occur only at division would be extremely cumbersome when using the approach of Mather *et al.* (2012) (see section 2.2) followed here, and having mutations occur at a fixed rate per unit time is also common in other models of tumor progression (e.g., Durrett *et al.*, 2011, 2010). Fortunately, in the original model of McFarland *et al.* (2013) having mutations proportional to unit time, not generation, leads to the same results (C. D. McFarland, pers. comm.). Initial equilibrium population size, K, is set to 2000; this is double the default number used in McFarland *et al.* (2013), but well within the range of values they explored (100-10000; see their Supplementary material); since simulations are fairly expensive, we want to increase the probability of reaching cancer which, as shown by McFarland *et al.* (2013) (see their Figure S3), increases with the initial population size (recall that we discard any simulation that does not reach cancer). Simulations are stopped when the number of drivers in any genotype is larger or equal than a pre-specified threshold. I have used two versions of the model, McF_4, where the threshold is set at four, and McF_6, where the threshold is set at six, in both cases leading to numbers of drivers within the ranges shown in their Table 1. (Simulations under McF_4 could be obtained by running simulations as for McF_6 and discarding all the samples from the time when four drivers are detected; this is, however, computationally wasteful.) The criterion for stopping the simulations does not include population size, since it is really redundant given this model: the population sizes for a given number of drivers can be found by setting $B(d) = D(N)$. In the McF_4 model the average final population size is about 5500 to 5800, a value slightly above that of setting $B(3) = D(N)$, because we stop the simulations at the first sampling period when four drivers have been reached. That corresponds to the period during the driver sweep (see also Figure 2 in McFarland *et al.* (2013)) when the population is in transition from $D(N) = B(3)$ to $D(N) = B(4)$. In the McF_6 model the final average sizes are of about 8100 to 8500 (slightly above $D(N) = B(5)$). With the above parameters, the number of simulations that reach completion ranges between 70 and 98%, depending on graph and sh (see Table 3).

Note that the models used do allow for the presence of clonal interference (e.g., in Graph 7A between two clones, one with mutation in genes 1 and 2, and another with mutations in genes 1 and 3). Regardless, the set of models used here is a relatively limited one (e.g., Korolev *et al.*, 2014) but, as discussed in the ms. the purpose of using several models is not to exhaust the range of

Table 3: Proportion of simulations that result in cancer by Graph, sh, and evolutionary Model.

	Graph	sh	Bozic	exp	McF_4	McF_6
1	11-A	Inf	0.16	0.04	0.95	0.92
2	11-B	Inf	0.14	0.03	0.94	0.90
3	9-A	Inf	0.19	0.04	0.96	0.91
4	9-B	Inf	0.20	0.05	0.98	0.95
5	7-A	Inf	0.10	0.01	0.83	0.72
6	7-B	Inf	0.10	0.02	0.81	0.76
7	11-A	0	0.17	0.04	0.95	0.93
8	11-B	0	0.16	0.04	0.96	0.91
9	9-A	0	0.20	0.04	0.98	0.92
10	9-B	0	0.20	0.06	0.98	0.96
11	7-A	0	0.10	0.01	0.81	0.72
12	7-B	0	0.09	0.02	0.84	0.78

plausible models but to examine the impact of some major models in the quality of our inferences about restrictions.

2.2 Simulation

For the simulations, I have used the Binomial-Negative Binomial (BNB) algorithm of Mather *et al.* (2012). This method is closely related to the Gillespie algorithm and the next reaction method Gibson and Bruck (2000) (see also Zhu *et al.*, 2011, for an example of modeling a Moran model of cancer development), but can lead to significant speed improvements when mutation rates are much smaller than death and birth rates. Given the very large number of simulations used in this study using a fast procedure was crucial. This algorithm is exact when birth, death, and mutation rates are constant between consecutive mutations, as in the "exp" and "Bozic" models. For the McFarland model (McFarland *et al.*, 2013), where death rate is density dependent, the approach in Mather *et al.* (2012) does not provide an exact simulation, but can be used to provide a very accurate approximation, as discussed in section 2.6 and section E of the supplementary material in Mather *et al.* (2012). This involves updating the system with short enough time increments so that birth, date, and mutation rates can be considered constant between updates. When using the model of McFarland *et al.* (2013), the value that is affected is the death rate through its dependence on the ratio N/K. In the simulations reported here, I update the system every 0.05 time units, so that even during fast driver sweeps, the relative change in death rate between updates remains small (in a set of 2,300,000 random simulations that represent all graphs, the largest absolute change in the death rate between successive updates was 0.027, with a mean of the maxima of 0.016, indicating an accurate approximation as the largest change in death rate between any two updates was less than 3% of any death rate in the simulation). I have implemented the BNB algorithm of Mather *et al.* (2012) so that in step 6 of their Algorithm 5 (see section C.1, p. 5, of their supplementary material), when updating the birth and death parameters, we check if the dependencies specified in the graph of the oncogenetic model are met, and if they are not, they are adjusted according to the setting of s_h, s, p, j (see Table 2). For all models, to determine if stopping criteria are met and to provide samples for the uniform sampling scheme, the simulation process is sampled every 15 time units. Examples of simulated trajectories for each model are shown in Figure 3, in section 2.3. Since our study needs to examine the effect of passengers on the inference of oncogenetic models, we keep track of individual clones, where a clone is any of the possible combinations of individual passengers and drivers (in contrast to most other simulation methods which only need to keep track of number of drivers or, at most, identity of clones as defined just by driver).

2.3 Examples of simulated cancer progression trajectories

The figures below show simulated trajectories, for simulations that reach cancer, for each of the models used. These figures reproduce the qualitative behavior of original models. Our simulations restrict attention to just a few passengers (50 in the examples shown below), whereas the original models consider many more (5000 in McFarland *et al.* (2013)) which explains the smaller number of different clones compared to, for example, Fig. 2C in McFarland *et al.* (2013).

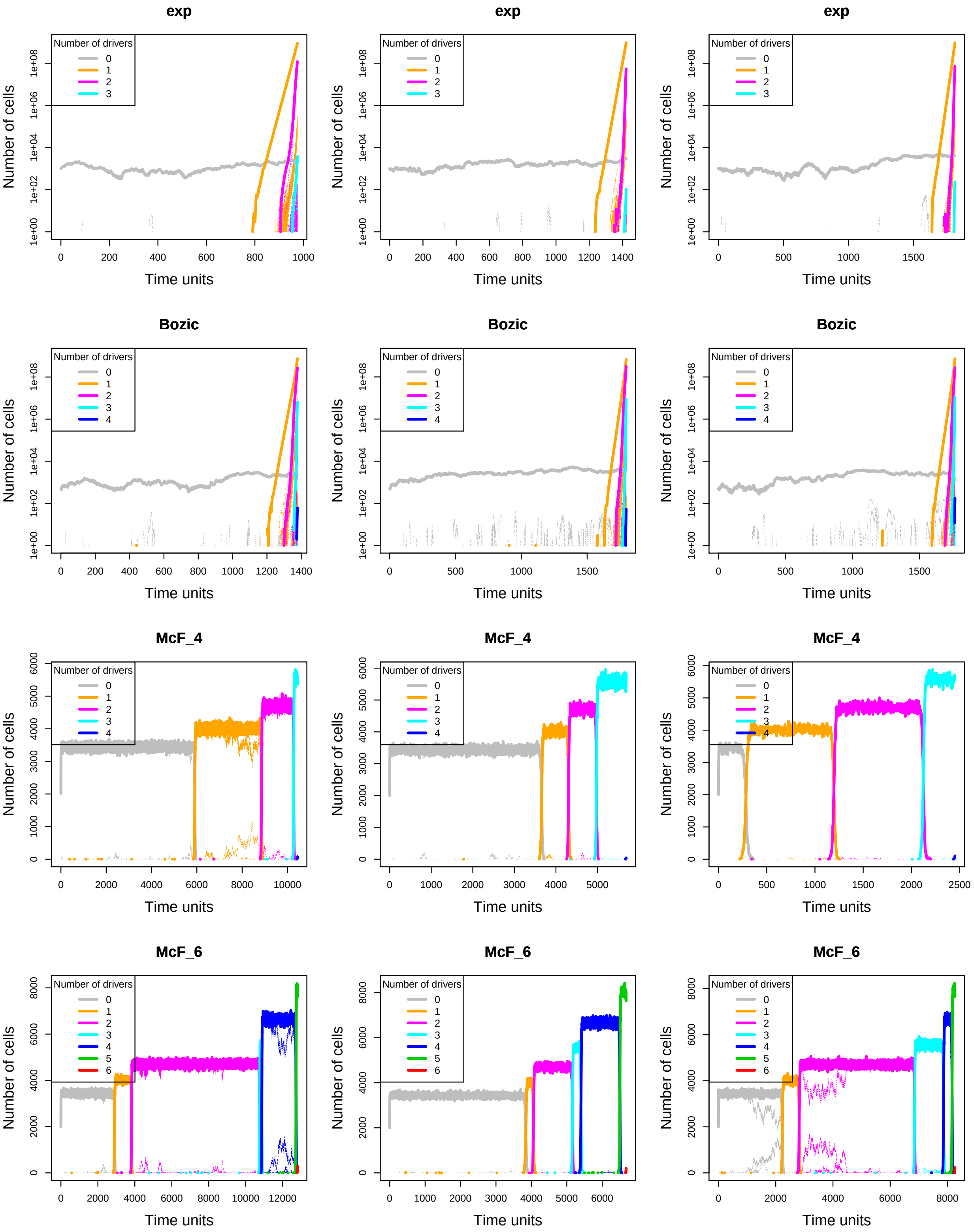

Figure 3: Examples of three randomly chosen simulations that reach cancer for each model, all with graph 11-A and $sh = Inf$. Thick lines are the sum of all clones with the specified number of drivers, whereas thin lines, with dotted line type, show individual clones. For the Bozic and exp models, the y-axis is shown in log scale.

3 Graphs (oncogenetic trees and CBNs) used

Graph 11-A is the same as Poset 2 in Gerstung *et al.* (2009) (their Figure 2A), and Graph 7-A is the same as the estimated graph for pancreatic cancer in Gerstung *et al.* (2011) (their Figure 2B). Graph 9 was created so as to contain an intermediate number of both nodes and conjunctions between the previous two graphs. In addition to the number of nodes and presence/absence and number of conjunctions, the six graphs used differ in other features (such as depth, total number of edges, existence or not of isolated nodes or subgraphs, existence or not of a single non-root parental node, indegree of conjunctions, and outdegree).

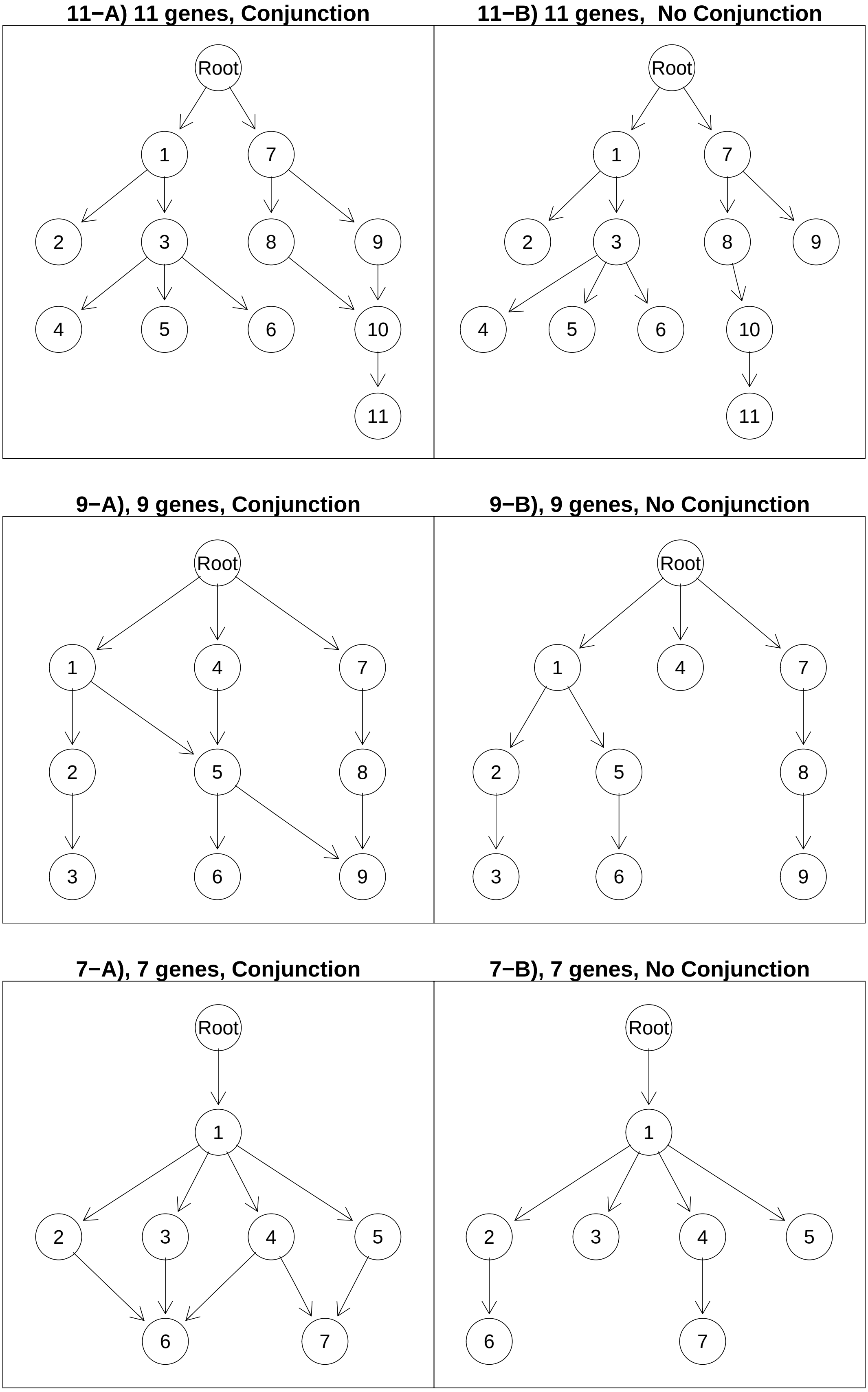

Figure 4: The six graphs (oncogenetic trees and CBNs) used.

4 Methods and software: CBN/DiP/OT

I used versions 0.3.2 and 0.3.3 of package Oncotree (Szabo and Pappas, 2013), downloaded from CRAN. Tree reconstruction requires no additional parameters with this method. The version of Rtreemix (Bogojeska, 2014) was 1.24.0. The results of Oncotree and Rtreemix were usually identical or very similar (see also plots in *Additional file 5*), but the implementation in package Oncotree incorporates a model for errors due to deviations from the graph of the oncogenetic model model (Szabo and Boucher, 2002) and is focused on single oncogenetic trees (whereas Rtreemix's emphasis are mixtures of trees).

For CBN I used version 0.1.04 of the software available from `http://www.bsse.ethz.ch/cbg/software/ct-cbn` (this is the latest version, as of May 2014, and was also the available one on April 2013). I wrote a wrapper to call their code from R, and I used the same default settings for temp (`-T = 1`) and steps (`-N =` number of nodes[2]) and started the simulated annealing search for the best poset from an initial linear poset as in Gerstung *et al.* (2011) (see their code in `example.py`, and their usage of their function `linear_poset`). The number of OpenMP threads of the `h-cbn` program was set to one: I was running as many different simultaneous processes as cores available in the computing cluster, and thus having multiple OpenMP threads would have lead to occasional (and unpredictable) increases in load with increases in total computing time from the cost of context switching (and running fewer processes would not have been compensated from the relatively small gains from the OpenMP parallelism in h-cbn). Some runs took extremely long to run; no run was allowed to run for more than 2 days.

For DiProg I used the code available from `https://bitbucket.org/farahani/diprog`. DiProg currently depends on IBM's ILOG CPLEX optimization library. I used version 12.6 of the library. This library not only is not open source, but has a severely restrictive license (although the authors of DiProg are working on making it work also with open source libraries —H. Farahani, pers. comm.). I obtained the library under the IBM Academic Initiative program. I set the maximum number of threads used by CPLEX to one (for reasons identical to the ones that lead me to use only one OpenMP thread with CBN). I used version 51465b398f9c of DiProg (from May 2014), with a minor fix where I prevented the python code from waiting unnecessarily idle until the maximum time (I removed the calls to function `WatcherThread` and set instead the maximum time directly via `c.parameters.timelimit.set()`, one of ILOG CPLEX parameters). I wrote a wrapper to call the python code from R and I run DiProg with option "MPN" (for monotone progression network —all our conjunctions are of monotone, not semimonotone type, *sensu* Farahani and Lagergren, 2013), examining solutions with $k = 1, 2, 3$ and kept the solution with the best BIC (which, in this case, it is the largest BIC, not the smallest one; see pp. 3 and 5 of Farahani and Lagergren, 2013). Each of the runs for each value of k was limited to use a maximum of 500 seconds and 2GB of RAM, but none of the runs ever got close to the limits (e.g., the maximum time of any run, over the three ks was 78 seconds). The other parameter of DiProg is ε. In Farahani and Lagergren (2013) they suggest comparing reconstructions with different values of ε against some known ground truth, and using the ε that leads to the smallest number of "bad edges" relative to the number of learned edges. It should be noted that this way of choosing ε might not always (or even frequently) be available for many users who are dealing with data for which very little is known. I run the code with two values of ε, 0.05 and 0.2 and the results reported here are those from 0.05, which overall lead to better reconstructions.

5 Measuring performance: performance measures

I measure performance using four different performance measures.

Difference between adjacency matrices This is a measure of the difference between the fitted and the true graph. Let A_T and A_F be the adjacency matrices for the true and inferred graphs respectively. Compute $A = A_T - A_F$ as their matrix difference (after, if necessary, adding the corresponding rows and columns filled with zeroes for any nodes present in one matrix and absent in the other). a_{ij} is the entry of A from row i and column j.

We define the difference between adjacency matrices as

$$\sum_i \sum_j |a_{ij}| \tag{1}$$

Note that our measure of dissimilarity is just the square of the "usual" Frobenius norm (Gentle, 2007).This performance measure is also the same as the "graph edit distance" of Hainke *et al.* (2012) (although the performance measure in Hainke *et al.*, 2012 is undefined if the number of nodes differs).

This is similar to the score used by Yin *et al.* (2006). In that paper, however, the authors use the maximum absolute row norm (the maximum difference in outgoing edges) of the matrix A and divide that norm by the number of (true) nodes (see their expression for S_{kl} in p. 15 and p. 14 for definition). Here, I use instead the total number of different connections since I want to give a larger score to a graph that misses more connections (even if the largest number of outgoing connections missed does not change). For instance, and referring to graph "11_B" a inferred graph that does not include the connection between nodes 3 and 4, and the connection between nodes 10 and 11, would have missed 2 connections, but a graph that included the connection between 3 and 4 (and not between 10 and 11) would have missed only one connection. The maximum absolute row sum, however, would be the same in both cases.

Note that the adjacency matrices include the root node, as Yin *et al.* (2006) do. This is in contrast to the **PFD**, **PND**, and **FPF** performance measure. This explains that in some cases **Diff** can be different even when the other three performance measure. are the same[1].

Finally, I do not scale Diff so that the numbers in the figures can be read directly as number of missed connections.

Proportion of false discoveries, PFD This is a measure of the proportion of false relations returned by a method.

Following Gerstung *et al.* (2009) and Gerstung *et al.* (2011), we define "relations" as the transitive closure of "cover relations" of the posets. For instance suppose a graph with $A \to B \to C$; the cover relations are $A \to B$ and $B \to C$, but we also include $A \to C$ in the relations; this is a biologically reasonable procedure, since C does depend on A (albeit indirectly). As in Gerstung *et al.* (2009) (see their p. 2811) and Gerstung *et al.* (2011), we do not include the root node when finding cover relations and their transitive closure (this is in contrast to what is done in the computation of **Difference between adjacency matrices**).

Now, similar to FPR in Gerstung *et al.* (2009) and Gerstung *et al.* (2011), we define

$$PFD = \frac{\#\text{ of relations in } F \text{ but not in } T}{\#\text{ of relations in } F} \tag{2}$$

[1] An extreme case, for instance, is the inference of tree 7-A, when there are passengers, under exp model, with sample size of 1000, S.Time=unif, S.Type = singleCell, and sh = 0; here only DiP and DiP-A infer trees, but these are trees that only have a root node from only node 1 hangs; in contrast, OT and CBN infer no tree whatsoever. PFD is therefore NA for all methods, PND is 1, FPF is 0, but Diff is 9 for DiP and 10 for all the other methods. DiP got right that node 1 does hang directly from root, whereas all other methods did not even infer that. However, when considering the transitive closure without the root, all of them have identical performance measures.

where F is the inferred graph and T is the true one.

The numerator is, therefore, the number of **false positives (FP)**. The denominator in our expression is not the same as the one in FPR Gerstung *et al.* (2009) and Gerstung *et al.* (2011). The one we use is easier to interpret directly as just the proportion of false relations out of the total number of relations returned by a method. And, therefore, we can interpret PFD directly as the proportion or fraction of false relations out of the total number of relations returned by a method. PFD as defined here is equivalent to $(1 - precision)$ or $(1 -$ positive predictive value$)$ (Davis and Goadrich, 2006; Pepe, 2003).

Note that the PFD is not defined when no edges are returned by the method (which is the reasonable thing to do, since when no edges are returned, we cannot compute the probability of inferring an incorrect edge). For those familiar with FDR control in multiple testing, this would be similar to the pFDR in Storey (2003) (see his section 6).

Proportion of Negative Discoveries, PND This is a measure of the proportion of relations not discovered. This is the same as FNR in Gerstung *et al.* (2009) and Gerstung *et al.* (2011) and is:

$$PND = \frac{\#\text{ of relations in } T \text{ but not in } F}{\#\text{ of relations in } T} \tag{3}$$

The numerator is the number of **false negatives (FN)**. PND is equivalent to $1 - recall$ or $1 - sensitivity$ (Davis and Goadrich, 2006; Pepe, 2003).

False positive fraction, FPF The proportion of falsely detected edges from those edges that are not present: $FPF = \frac{\#\text{ of relations in } F \text{ but not in } T}{\#\text{ of relations not in } T}$. The numerator is again the number of FP. The FPF is equivalent to $1 - specificity$ (Pepe, 2003). The relations not in T are computed as follows: for a set of candidate genes of size N, where N in our case is the number of drivers and the number of passengers (which is four times the number of drivers), the total possible cover relations over all possible graphs are $N * (N - 1)$, and we subtract from those the # relations in T. When Drivers are Known, that number is much smaller than when Drivers are Unknown (which explains that the FPFs are larger when Drivers are Known: as in common in document retrieval contexts, the cause is simply that the set of negative cases is much larger than that of positive cases). Regardless, it should be noted that FPF is often of minor value, to assess performance, compared to PND and PFD.

Some authors (e.g., Szabo and Boucher, 2008) use, as one of their performance measures, the probability of correct reconstruction (i.e., recovering the true graph) . This would not work for us as a general way of ranking methods as the (estimated) probability of correct reconstruction is zero for many combinations of methods and models and would, therefore, not allow us to differentiate between methods that, even if not recovering the exact true graph, have very different behavior in terms of how many edges or relationships they miss.

6 Overall ranking of Filtering, Method, and Sampling scheme

For each performance measure separately, I ranked the 36 combinations of Method by S.Time by S.Type (for the Drivers Known scenario) or the 144 combinations of Filtering by Method by S.Time by S.Type (for the Drivers Unknown scenario) in each of the 144 factor combinations defined by True Graph by Model by sh (see section "Deviations from monotonicity and genetic context dependence of driver status: *sh*"), by S.Size.

Thus, for each performance measure, there were 144 separate rankings (where we ranked 36 items in the Drivers Known scenario and 144 items in the Drivers Unknown scenario).

Ranking was done using the median (over the 20 replicates) of the performance measure. Then, for each performance measure, I obtained the average rank over subsets of the 144 combinations and the averaged ranks were then ranked to obtain the final rankings for each performance measure.Similar to Narendra *et al.* (2011), because the maximum and minimum ranks may differ between scenarios, the ranks were normalized before averaging (for every rank, the minimum rank for each scenario was subtracted, and this difference was divided by the range, the difference between maximum and minimum).

7 Multiple comparisons with the best (MCB)

The objective of the within-data set comparison is to find the best Method(s) (or Method by Filtering combination(s)), where best, for all performance measures, is "smaller". I have used the method of "multiple comparisons with the best (MCB)" (see Hsu, 1996; for a general, non-technical overview see pp. 67 and 68 of Hsu *et al.*, 2004). This method is closely connected to the "subset selection" procedures in the analysis of simulation experiments common in industrial settings (Allen, 2011; Goldsman and Nelson, 1998), as explained in Hsu (1996, p. 100 and ff.) and Hsu (1982, p. 462).

Briefly, we are interested in identifying the best method (where best, in our case, and for all measures, means smaller values —smaller number of errors). MCB compares each method against the best of the other methods (see p. 25 and 81-82 in Hsu, 1996). As explained in Hsu *et al.* (2004, p. 67 and 68), MCB asks, for each of the methods considered, "Is there sufficient evidence that this method is *not* the best?". For each of the methods, the probability of incorrectly answering "yes" is controlled at level α (using a multiple comparisons with a control procedure where each method is the control), and thus the set of methods for which the answer is "no" is a $100(1-\alpha)\%$ *confidence set* (called C) for the best method. Thus, paraphrasing Hsu (1982, p. 462), methods that are not contained in C can be *rejected* as methods that are not the best method.

In Hsu (1982) (see also Hsu, 1996, section 7.6, pp. 220 and ff.) a procedure for multiple comparisons with the best for block designs using Wilcoxon signed ranks is provided. This fits the design in this paper since each replicate data set in my design is a block. In addition, and because of the distribution of the four measures of performance, we want to use a nonparametric procedure (i.e., the one based on signed ranks) and not the normal means procedure.

Thus, separately for each performance measure and for each of the 864 among-data set combinations, I have used this procedure (procedure R_1, small-sample approximation, in p.463 of Hsu, 1982) to obtain the confidence set, C, for the best method; the probability of coverage has been set to (at least) 0.90. Before the analysis in each of the 864 among-data set combinations, Methods (or Method by Filter combinations) with more than 4 missing values (more than 20% of missing values) have been excluded (since methods that can not reliably return those statistics are unlikely to be of large interest, and because missing values decrease the sharpness of the procedure). This is the procedure I call "MCB" in *Additional file 7.*

The procedure in Hsu (1982), however, assumes that the error variables have an absolutely continuous distribution (see pp. 461 and 462 in Hsu, 1982) and, thus, that all pairwise differences (e.g., $Y_{ji}^{(\alpha)}(\delta)$ in p. 462 of Hsu, 1982) are distinct with probability one. Direct application of the procedures in section 4 of Hsu (1982) to cases where some, or all, values are identical between two or more treatments (Methods or Method by Filtering in our case) will fail: suppose two Methods (say, A and B) that are much better than all the others (i.e., have much smaller performance measures), but that have identical values for this performance measure. In this case, the set C (e.q. 4.2 in p. 463 of Hsu, 1982) will be empty because $\min_{j \neq i} V_{ij}$ (see e.q. 4.2) will be 0 even when i is A (or B), and this will happen at all levels of P^* (even those arbitrarily close to 1), and not just for small P^* (see "Remark" on p. 463 of Hsu, 1982). (Commented numerical examples of this phenomenon are provided in the code: see file `mcb-wilcox.R`). Thus, before applying this method, random noise from a uniform distribution $U(-1e^{-9}, 1e^{-9})$ was added to the data. Note that the added noise, as it can only take values between $\pm 1e^{-9}$, will not change the Wilcoxon signed rank statistic when all values are distinct between treatments (the smallest possible difference between two different values for any performance measure is $2.3e^{-7}$, for FPF), but when values are identical adding noise will have the effect of making the Wilcoxon signed rank statistic be symmetrically distributed around $n*(n+1)/4$, where n is the sample size. A second limitation of the approach in Hsu (1982) is due to its possible conservatism in the presence of missing data, were some Method(s) have fewer missing values than other (as we need to use a common v^*, in eq. 4.6, p. 463, of Hsu, 1982).

Given the above limitations, I have used a second procedure for multiple comparisons with the best, based directly on the exact p-values from the paired Wilcoxon tests, and with Pratt's method for handling zeros (induced by identical values), as implemented in the R package coin (Hothorn *et al.*, 2006, 2008). In those cases where all observations of two treatments have identical values,

the p-value is set to 1. This is the method I refer to as "MCB-2" in *Additional file 7*. This method simply follows the idea of constructing constrained MCB methods from one-sided multiple comparisons with a control (p. 115 in Hsu, 1996) and constructing a conservative method by setting the error rate of each individual comparison appropriately (p. 221 in Hsu, 1996), in our case to $1 - (1 - \alpha)^{1/(k-1)}$ (see also p. 463 in Hsu, 1982). Obviously, the MCB and MCB-2 procedures are identical in the absence of ties and missing values.

Finally, note that the MCB procedure is conducted for each combination of True Graph, Model, sh, S. Size, S. Time and S. Type. This is appropriate, as the procedure in Hsu (1982) assumes a no interaction model (see eq. 2.1 in Hsu, 1982 or eq. 7.57, p. 220 in Hsu, 1996), which is a reasonable assumption if we conduct the MCB for each combination of True Graph, Model, sh, S. Size, S. Time and S. Type (as the different blocks are simply different replicates of the same setting —i.e., identical runs that differ only in the random seed used).

In the main manuscript the tables show the results from "MCB-2", but the results from both procedures are very similar. *Additional file 7* shows the full results for both methods, and *Additional file 2* shows the summary tables for both methods, including the same tables as in the ms. but using MCB instead of MCB-2.

7.1 Constructing the confidence sets summary tables

Additional file 7 shows the confidence sets (i.e., the best method or methods) for every one of the 864 among-data set combinations. The summary tables shown in the ms and in *Additional file 2* simply count how often each of the confidence sets appear in different groupings of the among-data set combinations.

8 Generalized linear mixed modeling (GLMM) of performance measures.

Here I provide further details about the statistical modeling of performance measures.

Diff was modeled as a Poisson-distributed count, and PND, PFD, and FPF, were modeled as binomial data. Some factors, specially Number of Nodes but possibly also Model, might be regarded as random effects, but following standard recommendations in the literature (e.g., Collett, 2003; Hadfield, 2010; Gelman and Hill, 2007) for factors with few (less than, say, 10) levels I model them as fixed effects as otherwise, the estimation of the variance is very poor (in fact, in a Bayesian context, there is no true difference between fixed and random effects, only the relative weight we give to other levels to determine its value). Nevertheless, for the Drivers Known scenario, I rerun all the analysis using Graph (with six levels, so distinguishing between 11-A and 11-B, etc) as random effect, instead of using Conjunction, and Number of Nodes with the with three levels as 11, 9, 7 (and its interactions) as fixed effects and, as we would expect, it had no relevant effect on any of the other coefficients; the quality of the fit (judged by DIC and the CPO –see below) was obviously slightly smaller as, for simplicity, no random interactions of Graph with other terms were included.

Models were fitted using INLA Rue *et al.* (2009); Fong *et al.* (2010), a Bayesian approach that uses nested Laplace approximation as an alternative to Markov Chain Monte Carlo (MCMC), with the R package R-INLA. When using INLA, all models have been fitted with two different priors for the hyperparameter: the default one (Gamma(a, b), $a = 1, b = 0.00005$) and the one recommended in Fong *et al.* (2010) (Gamma(a, b), $a = 0.5, b = 0.0164$), which lead to the same conclusions regarding the fixed effects. For model validation I have used the cross-validated probability integral transform (PIT) (Held *et al.*, 2010), and a simple comparison of fitted vs. observed values.

I also fitted the additive and two-way interaction models for the Drivers Known scenario, and the additive model for the Drivers Unknown scenario, using the R package MCMCglmm Hadfield (2010). MCMCglmm differs from INLA not only on the use of MCMC (vs. Laplace approximations) but also on the priors and in the inclusion of an observation-level random effect. When using MCMCglmm (Hadfield, 2010) three chains, from overdispersed starting points, have been run in parallel and, after discarding the burn-in period (with variable number of iterations depending on the model), convergence has been assessed informally using trace plots and more formally with the Gelman-Rubin $\hat{R}$ statistic: chains have been run so that its value is < 1.1 for all parameters (Gelman and Hill, 2007, e.g., see section 16.4 in). All results are based on a total effective sample size of at least 1000 for each parameter estimate. Note that for the model for Diff in the Drivers Unknown is not clear if convergence has been reached. The complete set of models fitted is available from the supplementary material page (see section 10).

For model selection, in the INLA fits the mean logarithmic conditional predictive ordinate (CPO) (Roos and Held, 2011) leads to the same choices as using the DIC. I have fitted several models of increasing complexity, from models that include only main effects to models including up to all possible four-way interactions in the Drivers Known scenario and up to all three-way interactions in the Drivers Unknown scenario. In all cases, the DIC and the CPO indicated that larger models were to be preferred (e.g., models with four-way interactions preferred over those with three), which reflects the enormous sample size (20 observations per each cell of the fixed effects combinations), an effect that can also be seen in the very small standard deviations of effects (see model fits). The MCMCglmm fits also showed improved (i.e., smaller) DIC with larger model size. Thus, we will focus mainly on estimation, concentrating on factors that have a relevant effect (e.g., section 8.4.3 in Agresti, 2002). Most of the interactions in the three- and four-way interaction models, in addition to being extremely difficult to understand, affect factors not under user control and are of small magnitude. We will therefore be concerned with two-way interactions. All the fits are available from the supplementary material page (see section 10).

As explained in the text, I have used sum-to-zero contrasts (very similar to the "deviation coding" popular in psychology, except there the coefficients are ± 0.5 and 0 instead of ± 1 and 0). With sum-to-zero contrasts the missing parameter for a factor (or factor combination) is $-\sum$ rest of parameters for that factor, and the intercept is the overall mean. With these contrasts

each main effect parameter is to be interpreted as the (marginal) deviation of that level from the overall mean, and the interaction parameter as the deviation of the linear predictor of the cell mean (for that combination of levels) from the addition of the corresponding main effect parameters (e.g., section 3.5.2 in McCullagh and Nelder, 1989). For ease of interpretation of coefficients when using sum-to-zero contrasts I have used *contr.Sum* from the R package car (Fox and Weisberg, 2011).

When interpreting parameters for interaction terms it is important to remember the parameterization. Following section section 3.5.2 in McCullagh and Nelder (1989), suppose two factors, α and β (where α could be, say, Conjunction or no conjunction, and β could be S.Time), with two levels each. Denote their interaction by γ. We will thus have γ_{11}, γ_{12}, γ_{21}, γ_{22}. The constraints require that

$$\gamma_{11} + \gamma_{12} = 0, \qquad \gamma_{11} + \gamma_{21} = 0,$$
$$\gamma_{21} + \gamma_{22} = 0, \qquad \gamma_{12} + \gamma_{22} = 0$$

Thus, for example, a large γ_{11} (i.e., a large of level 1 of α and level 1 of β) means a small γ_{12} and a small γ_{21}. So when thinking about this large γ_{11} we can see that being β_1, having α_1 vs. α_2 leads to a larger value. Analogously, we can see that, being α_1, having β_1 leads to a larger value than having β_2. This can be generalized immediately to factors with more than two levels each.

When interpreting model fits, recall that these are generalized linear models and, therefore, for the binomial models the effect of having level i instead of level j of a variable is to change the odds ratio by $e^{\beta_i - \beta_j}$; likewise, for the Poisson models, $e^{\beta_i - \beta_j} = \frac{\mu_i}{\mu_j}$, where μ_i is the Poisson parameter (the mean) for level i. To ease interpretation, plots of the parameters from model fits show the exponential of the coefficient (so they can be directly read as changes in the odds ratio or the scale of the Poisson parameter).

9 Why GLMs, MCB, and ranking?

In this paper I have used three different sets of analyses that include GLMs, multiple comparisons with the best (MCB) for finding the best (set of) methods, and a simple ranking. Why three approaches? As is explained in the main text, they address different questions with approaches that use very different procedures and are based on very different assumptions. Given the complexity of the study, it is reassuring to see that all three methods (MCB, GLM, and simple overall ranking) lead to the same conclusion in those questions that all three methods can address (e.g., the overall superiority of OT, or the superiority of CBN with measure PND when we are dealing with graphs with conjunctions). Thus, one important reason for using three sets of approaches is **to check the internal consistency** of the methods and results.

More generally, however, the use of MCB and GLMs is somewhat analogous to comparing means of a factor (which we can do with multiple comparisons, of which MCB is a specific type) and trying to assess whether there is an interaction in an ANOVA (which we would do by fitting a linear model). Modeling and multiple comparisons are different objectives. And this is the reason why multiple comparisons procedures have not lead to the disappearance of GLMs or linear models.

The last point is especially relevant for our paper. Even if we could use MCB in our case (see next paragraph), it would not be the most appropriate procedure for some of our questions. Suppose we fit a model where we create an appropriate factor that encompasses all combinations of Method, Filter, Evolutionary model, etc. Assume there is statistical methodology to find the MCB in this case (see next paragraph). This might give us the set of best combinations of all those factors (including confidence intervals for the best). However, it would be extremely hard to use them to understand if interactions between the original factors exist (e.g., between Evolutionary Model and sh), or the relative importance of factors (is it really worth it to increase Sample Size?). In fact, using MCB would not be addressing the important questions here. For example, the question is not whether OT under the Bozic model is better than CBN under the McFarland model; what we care about here is whether Evolutionary Model makes much of a difference or not. Sure we can try to answer the second by answering the first, **but using a model (a GLM in this case) directly answers the relevant question.**

Moreover, no statistical methodology has been developed for MCB procedures for fitting a single model that is as complex as ours: with crossed between- and within-data set factors and using non-normal responses. In fact, as I explain above (section "Multiple comparisons with the best", p. 19) I used MCB by applying it over subsets of the data for which the design does allow the use of existing MCB methodology: "the MCB procedure is conducted for each combination of True Graph, Model, sh, S.Size, S.Time and S.Type. This is appropriate, as the procedure in Hsu (1982) assumes a no interaction model, which is a reasonable assumption if we conduct the MCB for each combination of True Graph, Model, sh, S.Size, S.Time and S.Type (as the different blocks are simply different replicates of the same setting —i.e., identical runs that differ only in the random seed used)". This assumption, however, would be violated otherwise (i.e., if we did not split by the above mentioned combinations as the no interaction assumption would be false). Again, for our purposes (finding the best method for **those** partitions) this is not a problem; but it would be if we wanted to apply the MCB in one sweep over the complete design.

10 Availability of scripts, code, and model fits

The code to reproduce all analysis, figures, and tables, as well as the output from all fitted statistical models is available as Additional File 9 (Code_scripts_model_fits.zip).

References

Agresti, A. (2002). *Categorical Data Analysis, 2nd ed.* Wiley, Hoboken, New Jersey, 2nd edition.

Allen, T. (2011). *Introduction to Discrete Event Simulation and Agent-Based Modeling*. Springer.

Beerenwinkel, N., Antal, T., Dingli, D., Traulsen, A., Kinzler, K. W., Velculescu, V. E., Vogelstein, B., and Nowak, M. A. (2007). Genetic progression and the waiting time to cancer. *PLoS computational biology*, **3**(11), e225.

Bogojeska, J. (2014). Rtreemix: Mutagenetic tree mixture models.

Bozic, I., Antal, T., Ohtsuki, H., Carter, H., Kim, D., Chen, S., Karchin, R., Kinzler, K. W., Vogelstein, B., and Nowak, M. A. (2010). Accumulation of driver and passenger mutations during tumor progression. *Proceedings of the National Academy of Sciences of the United States of America*, **107**, 18545–18550.

Collett, D. (2003). *Modelling binary data, 2nd ed.* Chapman and Hall/CRC, London.

Datta, R. S., Gutteridge, A., Swanton, C., Maley, C. C., and Graham, T. A. (2013). Modelling the evolution of genetic instability during tumour progression. *Evolutionary applications*, **6**(1), 20–33.

Davis, J. and Goadrich, M. (2006). The relationship between Precision-Recall and ROC curves. *Proceedings of the 23rd international conference on Machine learning - ICML '06*, pages 233–240.

Durrett, R., Foo, J., Leder, K., Mayberry, J., and Michor, F. (2010). Evolutionary dynamics of tumor progression with random fitness values. *Theoretical population biology*, **78**(1), 54–66.

Durrett, R., Foo, J., Leder, K., Mayberry, J., and Michor, F. (2011). Intratumor heterogeneity in evolutionary models of tumor progression. *Genetics*, **188**(2), 461–77.

Farahani, H. S. and Lagergren, J. (2013). Learning oncogenetic networks by reducing to mixed integer linear programming. *PloS one*, **8**(6), e65773.

Fong, Y., Rue, H. v., and Wakefield, J. (2010). Bayesian inference for generalized linear mixed models. *Biostatistics (Oxford, England)*, **11**(3), 397–412.

Fox, J. and Weisberg, S. (2011). *An R companion to applied regression, 2nd ed.* Sage, Thousand Oaks, CA.

Gelman, A. and Hill, J. (2007). *Data analysis using regression and multilevel hierarchical models*. Cambridge Univ Press.

Gentle, J. E. (2007). *Matrix Algebra*. Springer, New York.

Gerstung, M., Baudis, M., Moch, H., and Beerenwinkel, N. (2009). Quantifying cancer progression with conjunctive Bayesian networks. *Bioinformatics (Oxford, England)*, **25**(21), 2809–2815.

Gerstung, M., Eriksson, N., Lin, J., Vogelstein, B., and Beerenwinkel, N. (2011). The Temporal Order of Genetic and Pathway Alterations in Tumorigenesis. *PLoS ONE*, **6**(11), e27136.

Gibson, M. A. and Bruck, J. (2000). Efficient Exact Stochastic Simulation of Chemical Systems with Many Species and Many Channels. *The Journal of Physical Chemistry A*, **104**(9), 1876–1889.

Goldsman, D. and Nelson, B. (1998). Statistical screening, selection, and multiple comparison procedures in computer simulation. *Proceedings of the 30th conference on Winter simulation*, **1**(1994), 159–166.

Hadfield, J. (2010). MCMC Methods for Multi-response Generalized Linear Mixed Models: The MCMCglmm R Package. *Journal of Statistical Software*, **33**(2), 1–22.

Hainke, K., Rahnenführer, J., and Fried, R. (2012). Cumulative disease progression models for cross-sectional data: A review and comparison. *Biometrical journal. Biometrische Zeitschrift*, **54**(5), 617–40.

Held, L., Schrödle, B., and Rue, H. (2010). Posterior and cross-validatory predictive checks: a comparison of MCMC and INLA. In T. Kneib and G. Tutz, editors, *Statistical Modelling and Regression Structures*, chapter 6, pages 91–110. Physica-Verlag HD.

Hothorn, T., Hornik, K., van de Wiel, M. A., and Zeileis, A. (2006). A lego system for conditional inference. *The American Statistician*, **60**(3), 257–263.

Hothorn, T., Hornik, K., van de Wiel, M. A., and Zeileis, A. (2008). Implementing a class of permutation tests: the coin package. *Journal of Statistical Software*, **28**(8), 1–23.

Hsu, J. (1982). Simultaneous inference with respect to the best treatment in block designs. *Journal of the American Statistical Association*, **77**(378), 461–467.

Hsu, J., Qiu, P., Hin, L., Mutti, D., and Zadnik, K. (2004). Multiple comparisons with the best ROC curve. *Institute of Mathematical Statistics Lecture Notes-Monograph Series*, **47**, 65–75.

Hsu, J. C. (1996). *Multiple comparisons: theory and methods*. Chapman and Hall/CRC Press.

Korolev, K. S., Xavier, J. B., and Gore, J. (2014). Turning ecology and evolution against cancer. *Nature reviews Cancer*, **14**(5), 371–80.

Mather, W. H., Hasty, J., and Tsimring, L. S. (2012). Fast stochastic algorithm for simulating evolutionary population dynamics. *Bioinformatics (Oxford, England)*, **28**(9), 1230–1238.

McCullagh, P. and Nelder, J. (1989). *Generalized Linear Models, 2nd ed*. Chapman and Hall/CRC, London.

McFarland, C. D., Korolev, K. S., Kryukov, G. V., Sunyaev, S. R., and Mirny, L. A. (2013). Impact of deleterious passenger mutations on cancer progression. *Proceedings of the National Academy of Sciences of the United States of America*, **110**(8), 2910–5.

Narendra, V., Lytkin, N. I., Aliferis, C. F., and Statnikov, A. (2011). A comprehensive assessment of methods for de-novo reverse-engineering of genome-scale regulatory networks. *Genomics*, **97**(1), 7–18.

Pepe, M. (2003). *The statistical evaluation of medical tests for classification and prediction*. Oxford University Press, Oxford, UK.

Roos, M. and Held, L. (2011). Sensitivity analysis in Bayesian generalized linear mixed models for binary data. *Bayesian Analysis*, **6**(2), 259–278.

Rue, H. v., Martino, S., and Chopin, N. (2009). Approximate Bayesian inference for latent Gaussian models by using integrated nested Laplace approximations. *Journal of the Royal Statistical Society: Series B (Statistical Methodology)*, **71**(2), 319–392.

Storey, J. (2003). The positive false discovery rate: a bayesian interpretation and the q-value. *Annals of statistics*, **31**(6), 2013–2035.

Szabo, A. and Boucher, K. (2002). Estimating an oncogenetic tree when false negatives and positives are present. *Mathematical Biosciences*, **176**(2), 219–236.

Szabo, A. and Boucher, K. M. (2008). Oncogenetic trees. In W.-Y. Tan and L. Hanin, editors, *Handbook of cancer models with applications*, chapter 1, pages 1–24. World Scientific.

Szabo, A. and Pappas, L. (2013). Oncotree: Estimating oncogenetic trees.

Tomasetti, C., Vogelstein, B., and Parmigiani, G. (2013). Half or more of the somatic mutations in cancers of self-renewing tissues originate prior to tumor initiation. *Proceedings of the National Academy of Sciences of the United States of America*, **110**(6), 1999–2004.

Yin, J., Beerenwinkel, N., and Lengauer, T. (2006). Model Selection for Mixtures of Mutagenetic Trees. *Statistical Applications in Genetics and Molecular Biology*, **5**(17).

Zhu, T., Hu, Y., Ma, Z.-M., Zhang, D.-X., Li, T., and Yang, Z. (2011). Efficient simulation under a population genetics model of carcinogenesis. *Bioinformatics (Oxford, England)*, **27**(6), 837–43.
