# Supplementary material for: Identifying restrictions in the order of accumulation of mutations during tumor progression: effects of passengers, evolutionary models, and sampling
Source: BMC Bioinformatics. 2015 Feb 12;16:41. doi: 10.1186/s12859-015-0466-7 (PMC4339747; doi:10.1186/s12859-015-0466-7)
Supplement: Additional file 2 — Supplementary results. Additional results including: a) marginal plots of several two and three-way interactions; b) full tables of overall ranking of methods and filtering, including splits by S.Size; c) commented example of filtering and method effects; d) full tables of frequencies of confidence sets; e) full tables from the GLMM fits; f) tables comparing how often the augmented alternative is better; g) timings (execution time) of methods. [file 12859_2015_466_MOESM2_ESM.pdf]

Additional file 2. Supplementary Results.

Supplementary material for "Identifying Restrictions in the Order of Accumulation of Mutations during Tumor Progression: Effects of Passengers, Evolutionary Models, and Sampling"

Ramon Diaz-Uriarte
Dept. Biochemistry, Universidad Autónoma de Madrid
Instituto de Investigaciones Biomédicas "Alberto Sols" (UAM-CSIC)
Madrid, Spain

ramon.diaz@iib.uam.es

rdiaz02@gmail.com

http://ligarto.org/rdiaz

Contents

1 Results: marginal plots 7
1.1 Method by S.Time . . . 8
1.2 Method by S.Type . . . 9
1.3 Method by S.Size . . . 10
1.4 Model by S.Time . . . 11
1.5 Method by sh . . . 12
1.6 Model by Method by sh . . . 13
1.7 Filter by Method . . . 14
1.8 Filter by S.Type . . . 14
1.9 FN and FP: S.Time, Method, Model with Drivers Known . . . 15

2 Overall ranking 17
2.1 Overall ranking: Drivers Known . . . 17
2.1.1 Overall ranking: Drivers Known, conjunction . . . 18
2.1.2 Overall ranking: Drivers Known, no conjunction . . . 22
2.2 Overall ranking: Drivers Unknown . . . 26
2.2.1 Overall ranking: Drivers Unknown, conjunction . . . 29
2.2.2 Overall ranking: Drivers Unknown, no conjunction . . . 41

3 Commented example of filtering + method effects 53

4 Frequencies of confidence sets 55
4.1 Drivers Known, MCB . . . 55
4.2 Drivers Known, MCB-2 . . . 60
4.3 Drivers Unknown, MCB . . . 65
4.4 Drivers Unknown, MCB2-2 . . . 70
4.5 Summary frequencies of confidence sets with method MCB . . . 75

5 Model fits: tables 78
5.1 Drivers Known . . . 79
5.1.1 Fit for Diff, Drivers Known . . . 79

5.1.2 Fit for PFD, Drivers Known 82
5.1.3 Fit for PND, Drivers Known 85
5.1.4 Fit for FPF, Drivers Known 88
5.1.5 Fit for 'Inferred edges', Drivers Known 91
5.2 Drivers Unknown 94
5.2.1 Fit for Diff, Drivers Unknown 94
5.2.2 Fit for PFD, Drivers Unknown 98
5.2.3 Fit for PND, Drivers Unknown 102
5.2.4 Fit for FPF, Drivers Unknown 106
5.2.5 Fit for 'Inferred edges', Drivers Unknown 110

6 Choosing augmented alternatives 114

7 Timings of methods: additional results 115
7.1 Timing of BCBN 120

List of Tables

1 Overall ranking of all 36 combinations of method and sampling when Drivers are Known with respect to each performance performance measure. Methods have been ordered by their performance in the first performance measure. Best 5 methods are shown in bold. 17

2 Overall ranking of all 36 combinations of method and sampling when Drivers are Known with respect to each performance measure. Only graphs with Conjunction. Methods have been ordered by their performance in the first performance measure. Best 5 methods are shown in bold. 18

3 Overall ranking of all 36 combinations of method and sampling when Drivers are Known with respect to each performance measure. S.Size = 1000. Only graphs with Conjunction. Methods have been ordered by their performance in the first performance measure. Best 5 methods are shown in bold. 19

4 Overall ranking of all 36 combinations of method and sampling when Drivers are Known with respect to each performance measure. S.Size = 200. Only graphs with Conjunction. Methods have been ordered by their performance in the first performance measure. Best 5 methods are shown in bold. 20

5 Overall ranking of all 36 combinations of method and sampling when Drivers are Known with respect to each performance measure. S.Size = 100. Only graphs with Conjunction. Methods have been ordered by their performance in the first performance measure. Best 5 methods are shown in bold. 21

6 Overall ranking of all 36 combinations of method and sampling when Drivers are Known with respect to each performance measure. Only graphs without Conjunction. Methods have been ordered by their performance in the first performance measure. Best 5 methods are shown in bold. 22

7 Overall ranking of all 36 combinations of method and sampling when Drivers are Known with respect to each performance measure. S.Size = 1000. Only graphs without Conjunction. Methods have been ordered by their performance in the first performance measure. Best 5 methods are shown in bold. 23

8 Overall ranking of all 36 combinations of method and sampling when Drivers are Known with respect to each performance measure. S.Size = 200. Only graphs without Conjunction. Methods have been ordered by their performance in the first performance measure. Best 5 methods are shown in bold. 24

9 Overall ranking of all 36 combinations of method and sampling when Drivers are Known with respect to each performance measure. S.Size = 100. Only graphs without Conjunction. Methods have been ordered by their performance in the first performance measure. Best 5 methods are shown in bold. 25

10 Overall ranking of all 144 combinations of method, filtering, and sampling when Drivers are Unknown with respect to each performance performance measure. Methods have been ordered by their performance in the first performance measure. Best 10 methods are shown in bold. 26

11 Overall ranking of all 144 combinations of method, filtering, and sampling when Drivers are Unknown with respect to each performance measure. Only graphs with Conjunction. Methods have been ordered by their performance in the first performance measure. Best 10 methods are shown in bold. 29

12 Overall ranking of all 144 combinations of method, filtering, and sampling when Drivers are Unknown with respect to each performance measure. S.Size = 1000. Only graphs with Conjunction. Methods have been ordered by their performance in the first performance measure. Best 10 methods are shown in bold. 32

13 Overall ranking of all 144 combinations of method, filtering, and sampling when Drivers are Unknown with respect to each performance measure. S.Size = 200. Only graphs with Conjunction. Methods have been ordered by their performance in the first performance measure. Best 10 methods are shown in bold. 35
14 Overall ranking of all 144 combinations of method, filtering, and sampling when Drivers are Unknown with respect to each performance measure. S.Size = 100. Only graphs with Conjunction. Methods have been ordered by their performance in the first performance measure. Best 10 methods are shown in bold. 38
15 Overall ranking of all 144 combinations of method, filtering, and sampling when Drivers are Unknown with respect to each performance measure. Only graphs without Conjunction. Methods have been ordered by their performance in the first performance measure. Best 10 methods are shown in bold. 41
16 Overall ranking of all 144 combinations of method, filtering, and sampling when Drivers are Unknown with respect to each performance measure. S.Size = 1000. Only graphs without Conjunction. Methods have been ordered by their performance in the first performance measure. Best 10 methods are shown in bold. 44
17 Overall ranking of all 144 combinations of method, filtering, and sampling when Drivers are Unknown with respect to each performance measure. S.Size = 200. Only graphs without Conjunction. Methods have been ordered by their performance in the first performance measure. Best 10 methods are shown in bold. 47
18 Overall ranking of all 144 combinations of method, filtering, and sampling when Drivers are Unknown with respect to each performance measure. S.Size = 100. Only graphs without Conjunction. Methods have been ordered by their performance in the first performance measure. Best 10 methods are shown in bold. 50
19 Frequency of confidence sets (method MCB) for measure Diff when Drivers are Known. The table shows the 3 most common confidence set combinations (combinations not shown have a frequency less than 0.025). 56
20 Frequency of confidence sets (method MCB) for measure PFD when Drivers are Known. The table shows the 8 most common confidence set combinations (combinations not shown have a frequency less than 0.025). 57
21 Frequency of confidence sets (method MCB) for measure PND when Drivers are Known. The table shows the 8 most common confidence set combinations (combinations not shown have a frequency less than 0.025). 58
22 Frequency of confidence sets (method MCB) for measure FPF when Drivers are Known. The table shows the 8 most common confidence set combinations (combinations not shown have a frequency less than 0.025). 59
23 Frequency of confidence sets (method MCB-2) for measure Diff when Drivers are Known. The table shows the 4 most common confidence set combinations (combinations not shown have a frequency less than 0.025). 61
24 Frequency of confidence sets (method MCB-2) for measure PFD when Drivers are Known. The table shows the 8 most common confidence set combinations (combinations not shown have a frequency less than 0.025). 62
25 Frequency of confidence sets (method MCB-2) for measure PND when Drivers are Known. The table shows the 7 most common confidence set combinations (combinations not shown have a frequency less than 0.025). 63
26 Frequency of confidence sets (method MCB-2) for measure FPF when Drivers are Known. The table shows the 6 most common confidence set combinations (combinations not shown have a frequency less than 0.025). 64
27 Frequency of confidence sets (method MCB) for measure Diff when Drivers are Unknown. The table shows the 7 most common confidence set combinations (combinations not shown have a frequency less than 0.025). 66

28 Frequency of confidence sets (method MCB) for measure PFD when Drivers are Unknown. The table shows the 5 most common confidence set combinations (combinations not shown have a frequency less than 0.025). 67
29 Frequency of confidence sets (method MCB) for measure PND when Drivers are Unknown. The table shows the 8 most common confidence set combinations (combinations not shown have a frequency less than 0.025). 68
30 Frequency of confidence sets (method MCB) for measure FPF when Drivers are Unknown. The table shows the 6 most common confidence set combinations (combinations not shown have a frequency less than 0.025). 69
31 Frequency of confidence sets (method MCB-2) for measure Diff when Drivers are Unknown. The table shows the 9 most common confidence set combinations (combinations not shown have a frequency less than 0.025). 71
32 Frequency of confidence sets (method MCB-2) for measure PFD when Drivers are Unknown. The table shows the 6 most common confidence set combinations (combinations not shown have a frequency less than 0.025). 72
33 Frequency of confidence sets (method MCB-2) for measure PND when Drivers are Unknown. The table shows the 9 most common confidence set combinations (combinations not shown have a frequency less than 0.025). 73
34 Frequency of confidence sets (method MCB-2) for measure FPF when Drivers are Unknown. The table shows the 11 most common confidence set combinations (combinations not shown have a frequency less than 0.025). 74
35 Frequencies of confidence sets for all measures when Drivers are Known. The table shows the frequency of the most common confidence set combinations. Combinations not shown have a frequency less than 0.05 for all columns. Frequencies normalized by column total (N = 432). 76
36 Frequencies of confidence sets for all measures when Drivers are Unknown. The table shows the frequency of the most common confidence set combinations. Combinations not shown have a frequency less than 0.05 for all columns or are composed of more than 10 individual best methods. Frequencies normalized by column total (N = 432). 'A:B' denotes filtering with A and using method B. 77
37 Model fit for performance measure 'Diff' when there are no passengers. 79
38 Model fit for performance measure 'PFD' when there are no passengers. 82
39 Model fit for performance measure 'PND' when there are no passengers. 85
40 Model fit for performance measure 'FPF' when there are no passengers. 88
41 Model fit for performance measure 'Inferred edges' when there are no passengers. . . 91
42 Model fit for performance measure 'Diff' when there are passengers. 94
43 Model fit for performance measure 'PFD' when there are passengers. 98
44 Model fit for performance measure 'PND' when there are passengers. 102
45 Model fit for performance measure 'FPF' when there are passengers. 106
46 Model fit for performance measure 'Inferred edges' when there are passengers. 110
47 Comparison of augmented and non augmented alternatives in the Drivers Known scenario. 114
48 Comparison of augmented and non augmented alternatives when Drivers are Unknown and Filtering = S5. 114
49 Summary statistics of timings for the BCBN function (in seconds), using a total of 40 replicates per row shown. Those 40 replicates use four different models (Bozic and McF_4, with uniform and last S.Time), and the two graphs for each NumNodes. Not all 40 cases were always completed within 4 days —although some were allowed to run until completion or until crashes in the function. 120

List of Figures

1 Mean of each performance measure for the different combinations of factors when Drivers Known. 8
2 Mean of each performance measure for the different combinations of factors when Drivers Unknown. 8
3 Mean of each performance measure for the different combinations of factors when Drivers Known. 9
4 Mean of each performance measure for the different combinations of factors when Drivers Unknown. 9
5 Mean of each performance measure for the different combinations of factors when Drivers Known. 10
6 Mean of each performance measure for the different combinations of factors when Drivers Unknown. 10
7 Mean of each performance measure for the different combinations of factors when Drivers Known. 11
8 Mean of each performance measure for the different combinations of factors when Drivers Unknown. 11
9 Mean of each performance measure for the different combinations of factors when Drivers Known. 12
10 Mean of each performance measure for the different combinations of factors when Drivers Unknown. 12
11 Mean of each performance measure for the different combinations of factors when Drivers Known. 13
12 Mean of each performance measure for the different combinations of factors when Drivers Unknown. 13
13 Mean of each performance measure for the different combinations of factors when Drivers Unknown. 14
14 Mean of each performance measure for the different combinations of factors when Drivers Unknown. 14
15 Mean number of (a) false positive (FP) relations and (b) false negative (FN) relations, in the Drivers Known scenario, for the different combinations of Model, Method, and S.Time. 16
16 Example of effects of filtering and method. See text for details; these are the relevant figures from the corresponding *Additional file 1* and *Additional File 3*. 54
17 Mean execution time (in seconds) for each method, by S.Size and NumNodes of the original graph, in the Drivers Known scenario. 116
18 Mean execution time (in seconds) for each method, by S.Size, NumNodes of the original graph, and Filtering, in the Drivers Unknown scenario. 117
19 Median execution time (in seconds) for each method, by S.Size and NumNodes of the original graph, in the Drivers Known scenario. 118
20 Median execution time (in seconds) for each method, by S.Size, NumNodes of the original graph, and Filtering, in the Drivers Unknown scenario. 119

1 Results: marginal plots

The following plots show marginal means of the specified factors for both the Drivers Known and Drivers Unknown scenarios except for those related to Filtering (obviously, only for the Drivers Unknown scenario).

1.1 Method by S.Time

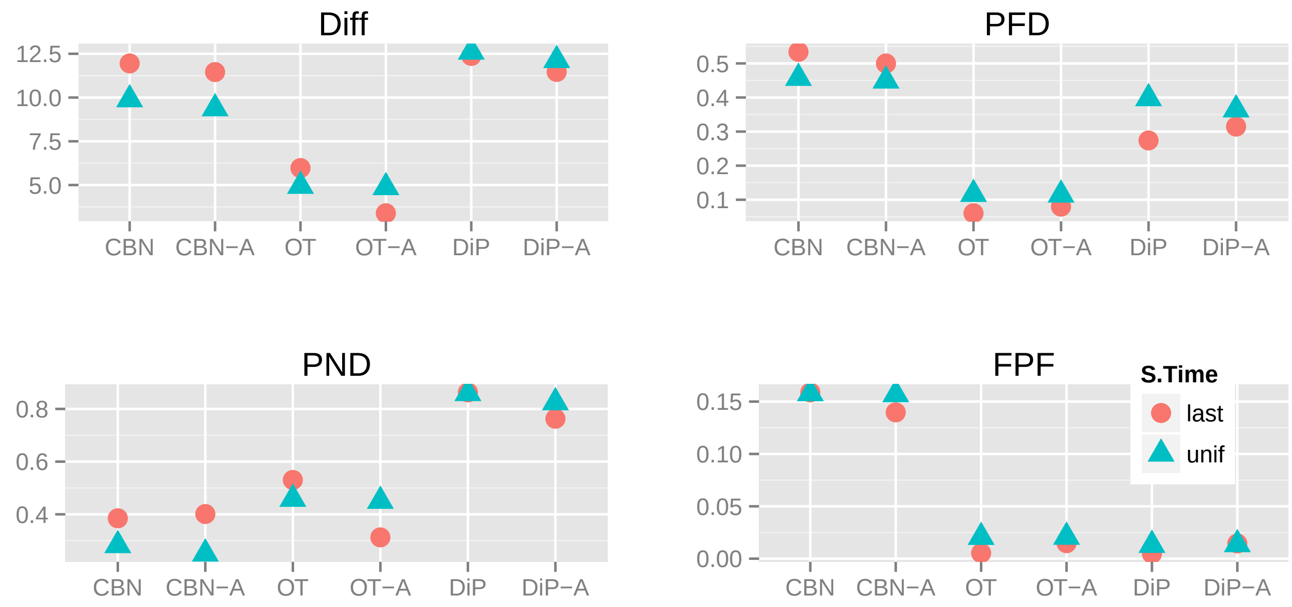

Figure 1: Mean of each performance measure for the different combinations of factors when Drivers Known.

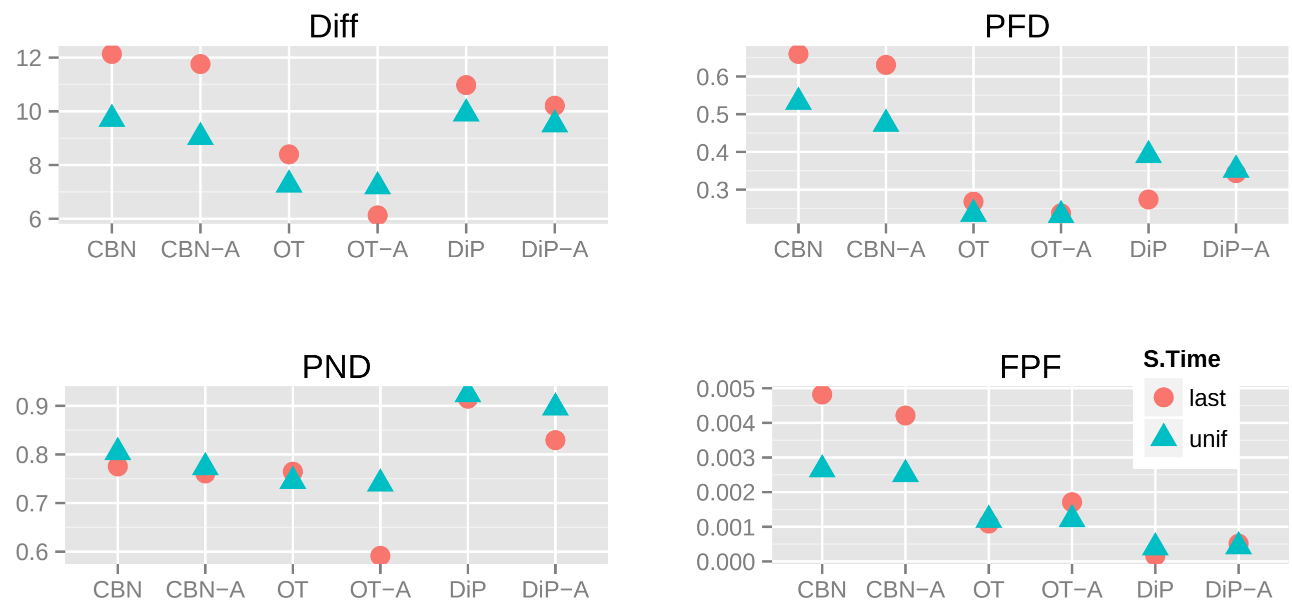

Figure 2: Mean of each performance measure for the different combinations of factors when Drivers Unknown.

1.2 Method by S.Type

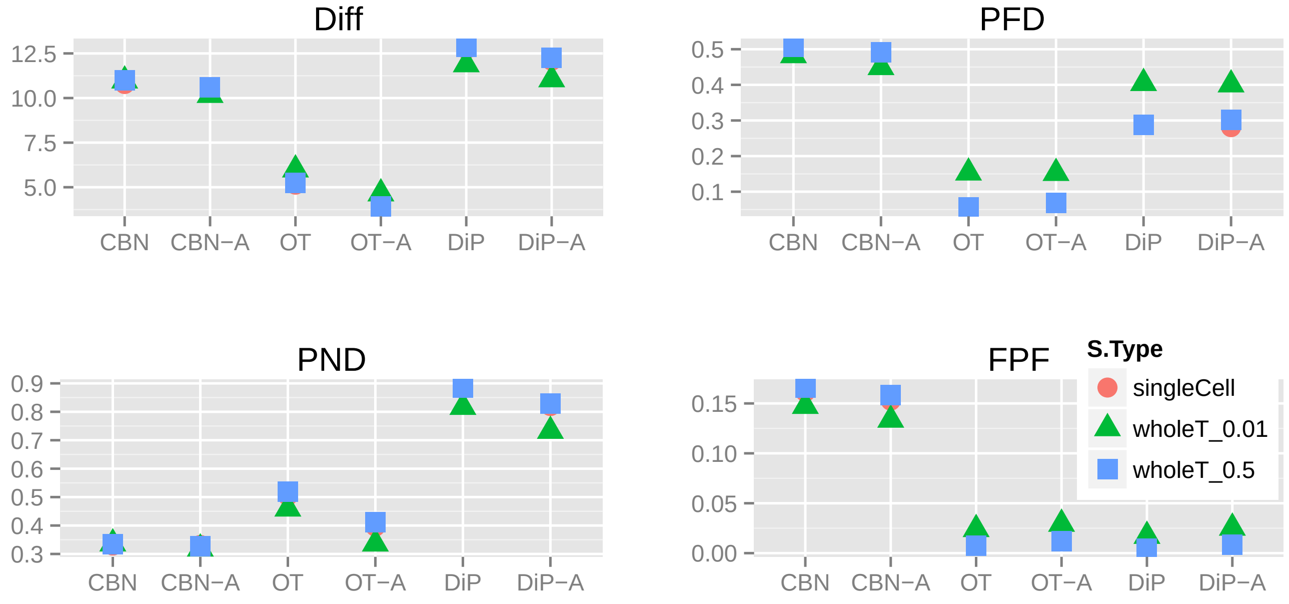

Figure 3: Mean of each performance measure for the different combinations of factors when Drivers Known.

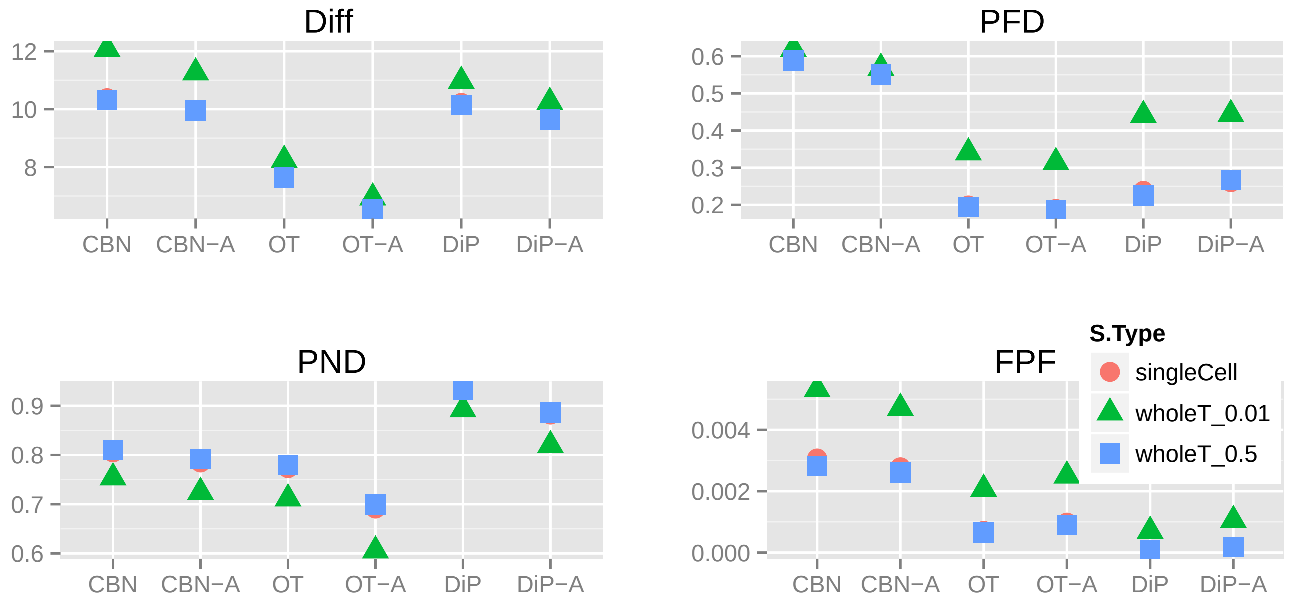

Figure 4: Mean of each performance measure for the different combinations of factors when Drivers Unknown.

1.3 Method by S.Size

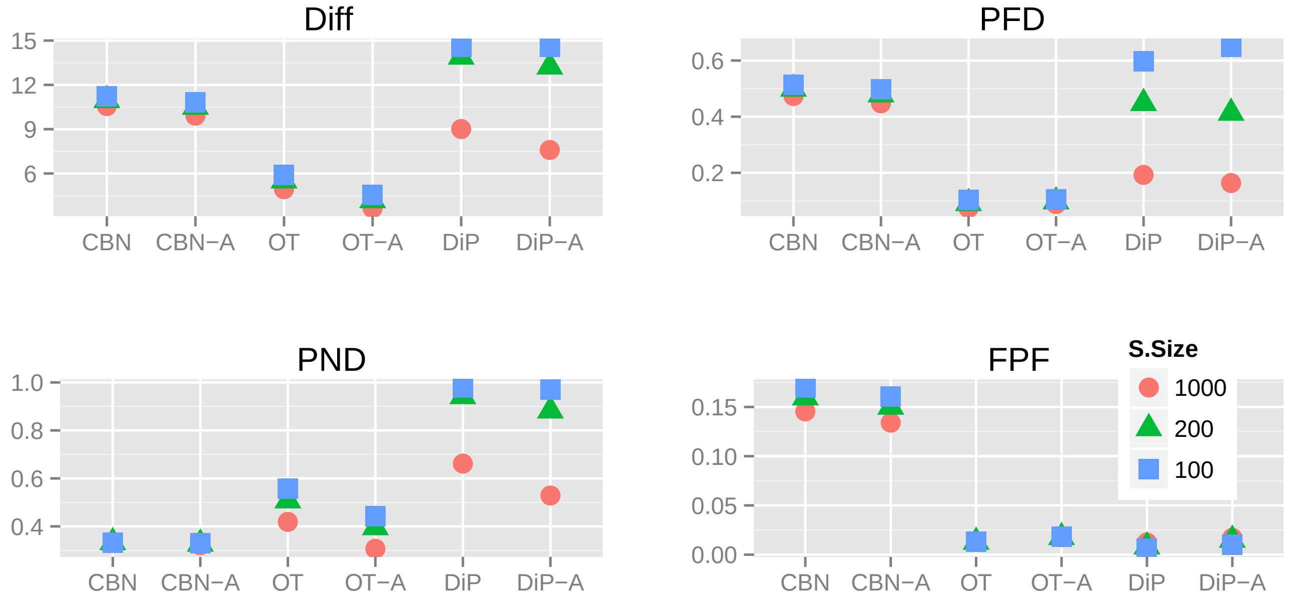

Figure 5: Mean of each performance measure for the different combinations of factors when Drivers Known.

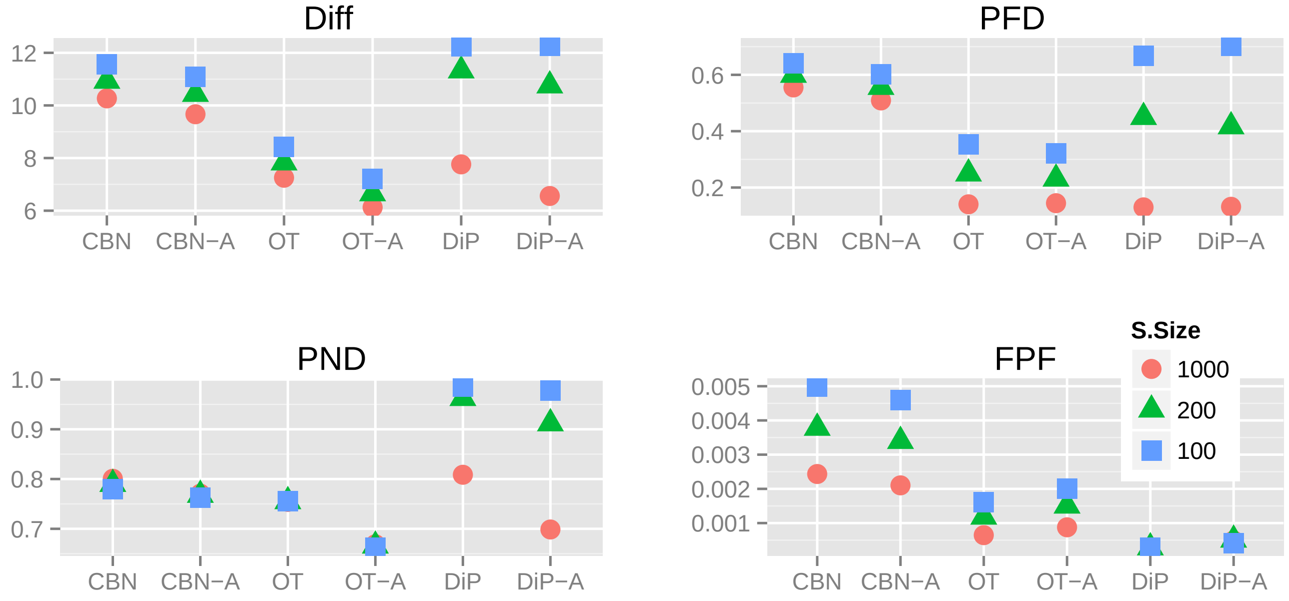

Figure 6: Mean of each performance measure for the different combinations of factors when Drivers Unknown.

1.4 Model by S.Time

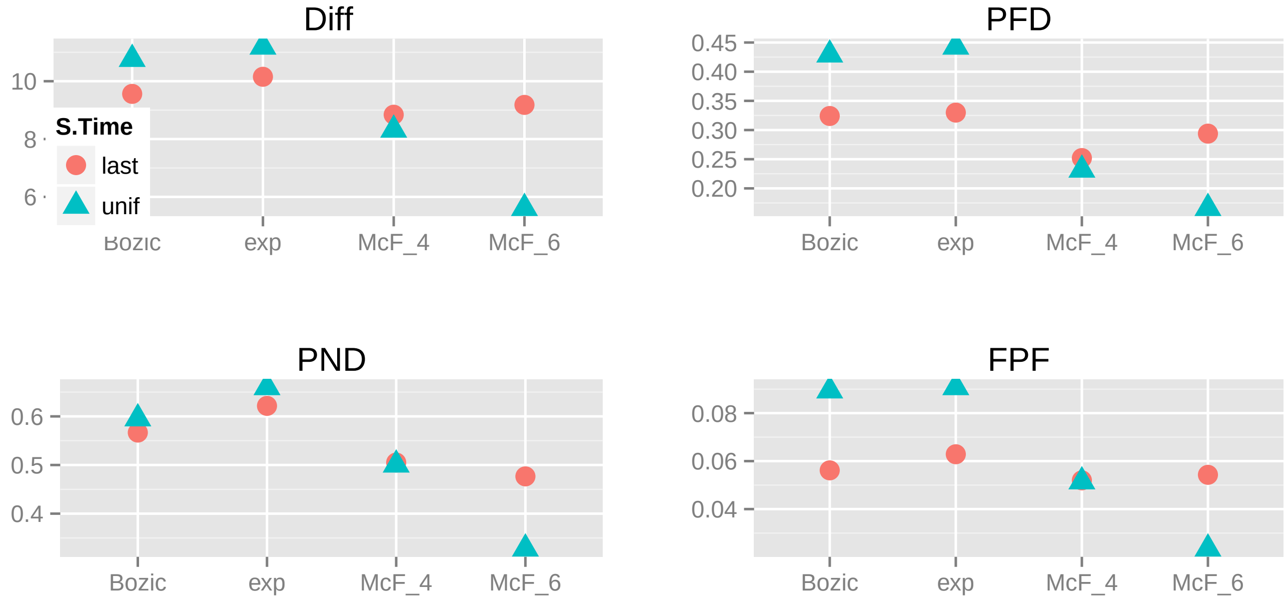

Figure 7: Mean of each performance measure for the different combinations of factors when Drivers Known.

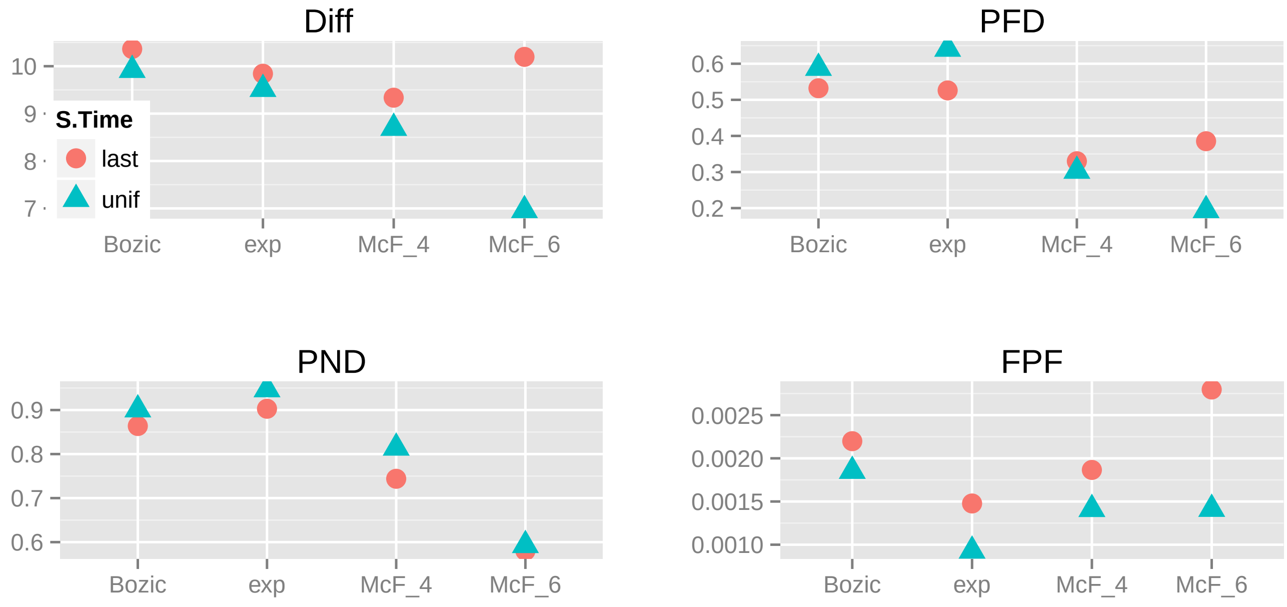

Figure 8: Mean of each performance measure for the different combinations of factors when Drivers Unknown.

1.5 Method by sh

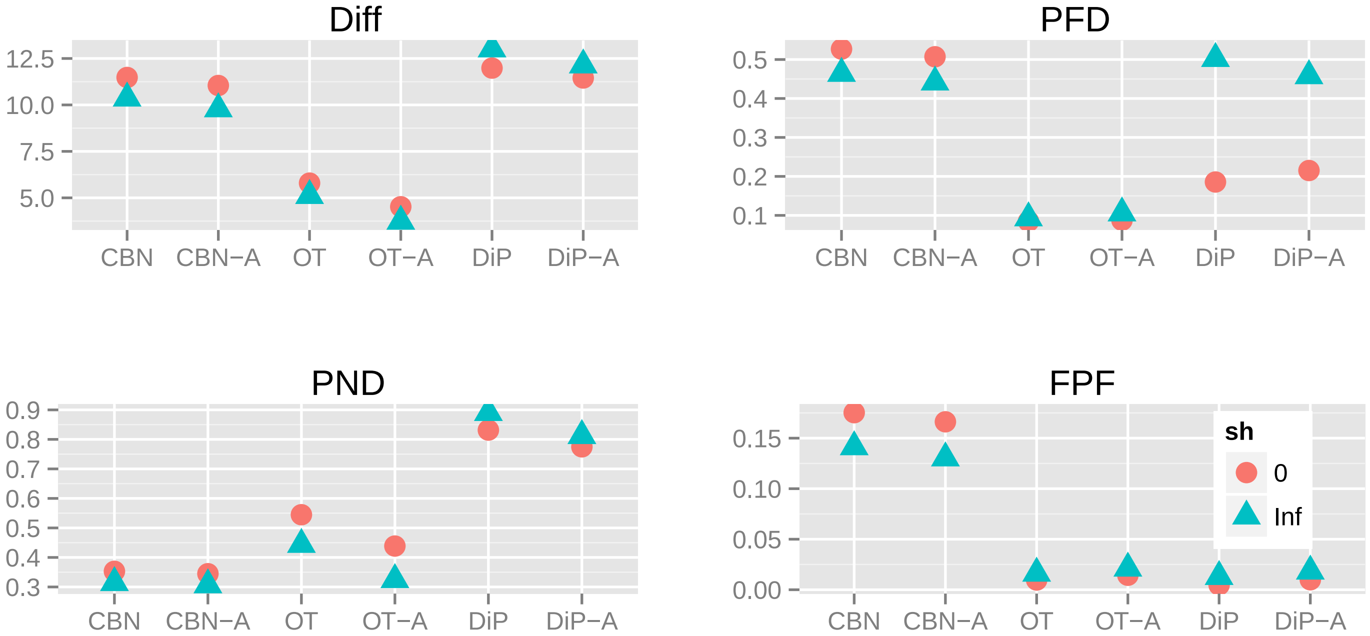

Figure 9: Mean of each performance measure for the different combinations of factors when Drivers Known.

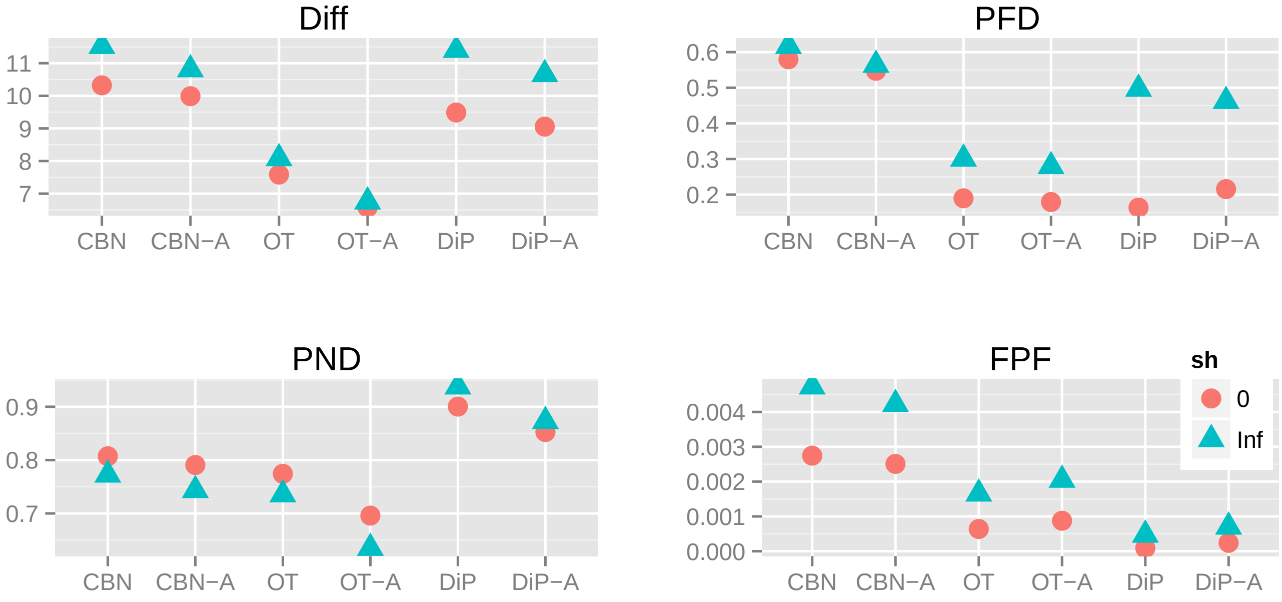

Figure 10: Mean of each performance measure for the different combinations of factors when Drivers Unknown.

1.6 Model by Method by sh

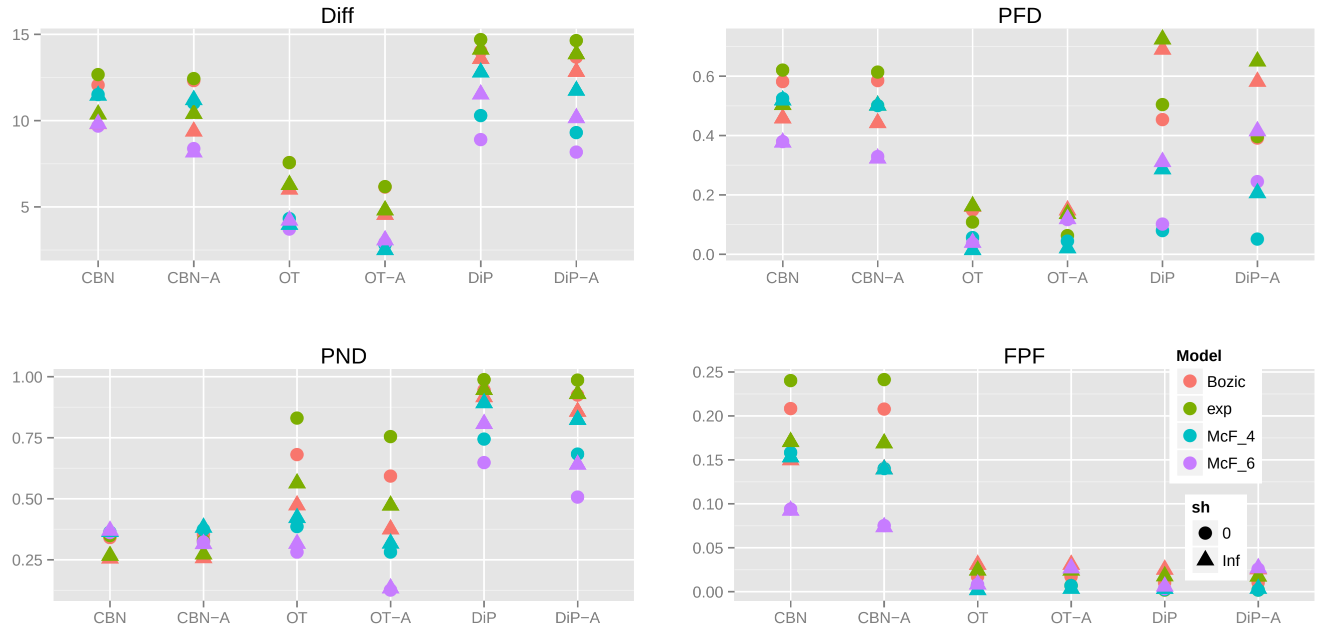

Figure 11: Mean of each performance measure for the different combinations of factors when Drivers Known.

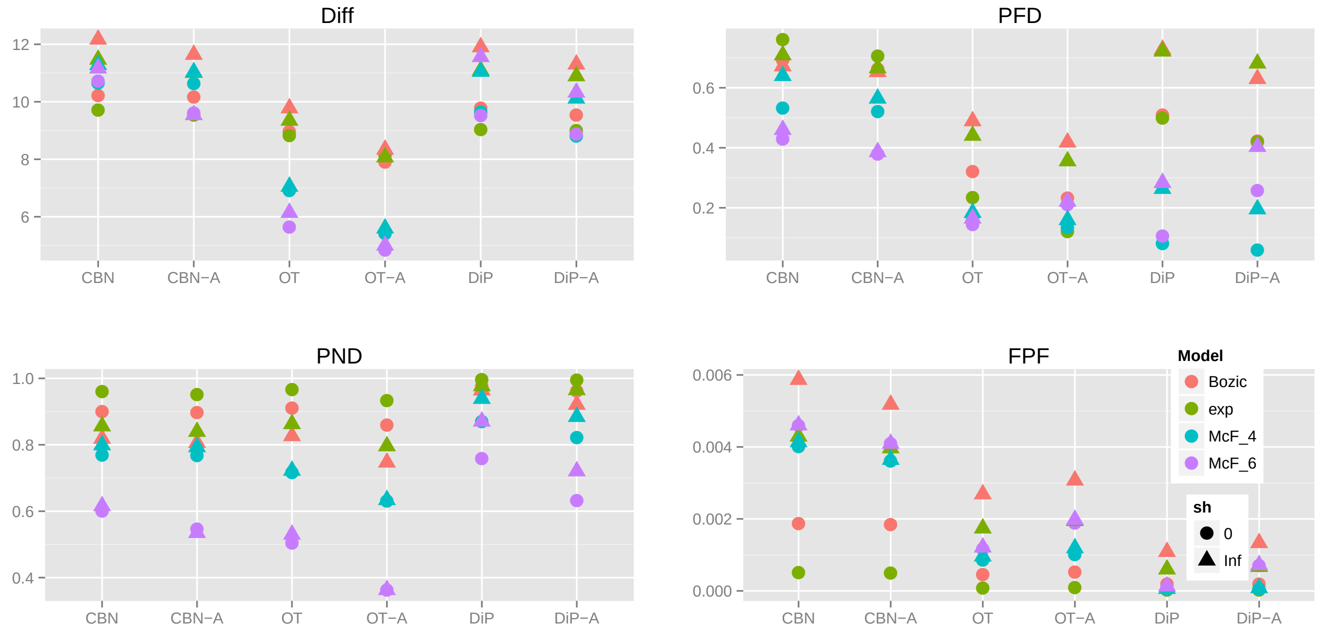

Figure 12: Mean of each performance measure for the different combinations of factors when Drivers Unknown.

1.7 Filter by Method

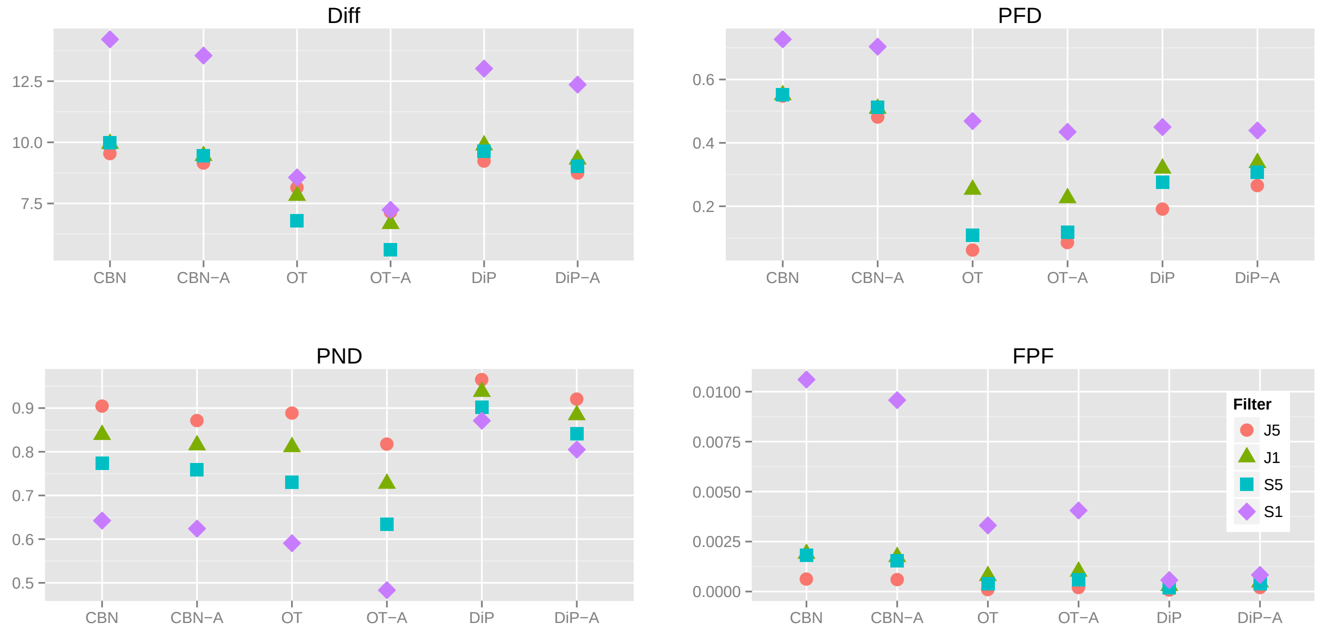

Figure 13: Mean of each performance measure for the different combinations of factors when Drivers Unknown.

1.8 Filter by S.Type

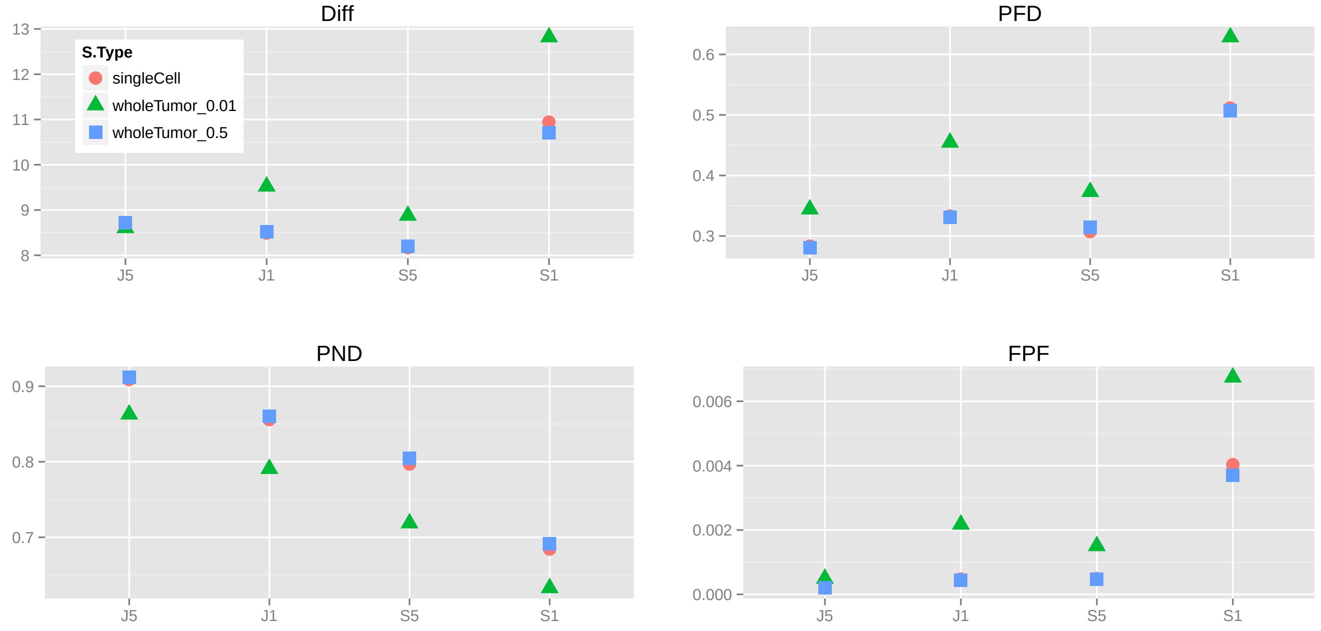

Figure 14: Mean of each performance measure for the different combinations of factors when Drivers Unknown.

1.9 FN and FP: S.Time, Method, Model with Drivers Known

The following figure shows that, even if there were three way interactions between Model, Method, and S.Time, they are of much smaller relevance than those between Model, Method, and sh, shown in the ms.

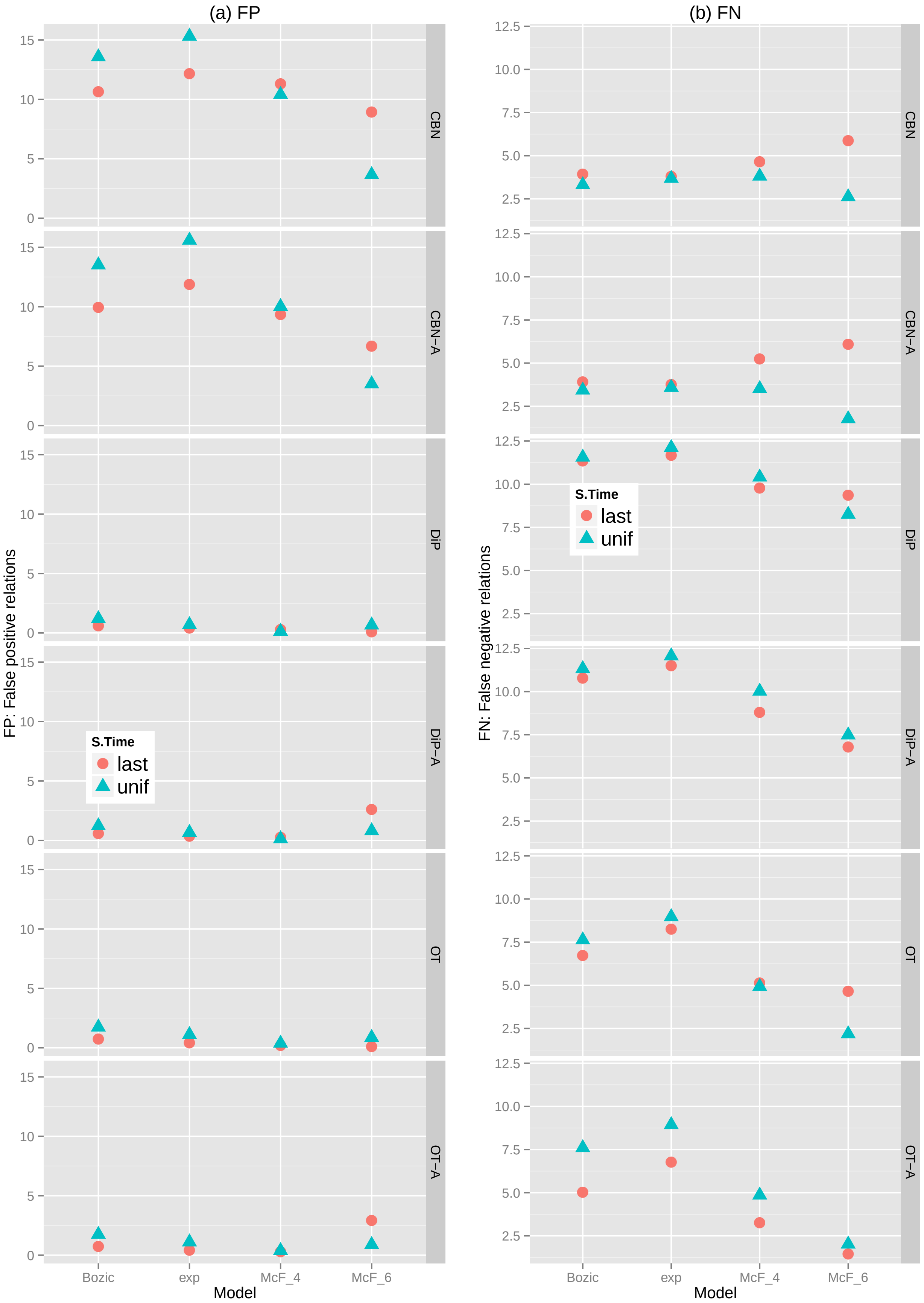

Figure 15: Mean number of (a) false positive (FP) relations and (b) false negative (FN) relations, in the Drivers Known scenario, for the different combinations of Model, Method, and S.Time.

2 Overall ranking

2.1 Overall ranking: Drivers Known

Table 1: Overall ranking of all 36 combinations of method and sampling when Drivers are Known with respect to each performance performance measure. Methods have been ordered by their performance in the first performance measure. Best 5 methods are shown in bold.

Method and sampling	Diff	PFD	PND	FPF
OT-A, last, singleC	**1**	**1**	8	13
OT-A, last, wholeT_0.5	**2**	**2**	9	14
OT-A, last, wholeT_0.01	**3**	6	**2**	22
OT-A, unif, singleC	**4**	8	19	10
OT, unif, singleC	**5**	7	20	9
OT-A, unif, wholeT_0.5	6	10	21	15.5
OT, unif, wholeT_0.5	7	9	22	15.5
OT-A, unif, wholeT_0.01	8	11	12	24
OT, unif, wholeT_0.01	9	12	14	23
OT, last, singleC	10	**5**	23	**2**
OT, last, wholeT_0.01	11	**3**	18	18
OT, last, wholeT_0.5	12	**4**	24	**1**
CBN-A, unif, wholeT_0.01	13	13	**1**	26
CBN-A, unif, wholeT_0.5	14	17	**4**	33
CBN-A, unif, singleC	15	15	**5**	29
CBN, unif, singleC	16	18	6	31
CBN, unif, wholeT_0.5	17	19	7	32
CBN, unif, wholeT_0.01	18	14	**3**	27
CBN-A, last, wholeT_0.01	19	16	15	25
CBN, last, singleC	20	23	10	35
DiP-A, last, wholeT_0.01	21	22	25	21
CBN, last, wholeT_0.01	22	20	13	28
CBN-A, last, singleC	23	21	17	30
CBN-A, last, wholeT_0.5	24	24	16	34
CBN, last, wholeT_0.5	25	25	11	36
DiP-A, unif, wholeT_0.01	26	27	26	20
DiP, last, wholeT_0.01	27	29	29	11
DiP-A, last, singleC	28	26	27	17
DiP, unif, wholeT_0.01	29	31	30	19
DiP-A, last, wholeT_0.5	30	33	28	12
DiP-A, unif, singleC	31	30	31	6
DiP-A, unif, wholeT_0.5	32	35	32	7
DiP, last, singleC	33	28	34	8
DiP, last, wholeT_0.5	34	34	36	**5**
DiP, unif, singleC	35	32	33	**3**
DiP, unif, wholeT_0.5	36	36	35	**4**

2.1.1 Overall ranking: Drivers Known, conjunction

Table 2: Overall ranking of all 36 combinations of method and sampling when Drivers are Known with respect to each performance measure. Only graphs with Conjunction. Methods have been ordered by their performance in the first performance measure. Best 5 methods are shown in bold.

Method and sampling	Diff	PFD	PND	FPF
OT-A, last, singleC	**1**	**2**	14	10
OT-A, last, wholeT_0.5	**2**	**1**	15	12
OT-A, last, wholeT_0.01	**3**	**3**	7	22
OT-A, unif, singleC	**4**	6	19	13
OT, unif, singleC	**5**	**5**	20	11
OT-A, unif, wholeT_0.5	6	10	21	15.5
OT, unif, wholeT_0.5	7	8	22	15.5
OT-A, unif, wholeT_0.01	8	11	16	24
OT, unif, wholeT_0.01	9	12	17	23
OT, last, singleC	10	9	23	**1**
OT, last, wholeT_0.01	11	**4**	18	18
OT, last, wholeT_0.5	12	7	24	**2**
CBN-A, unif, wholeT_0.01	13	13	**1**	26
CBN-A, unif, singleC	14	16	**2**	28
CBN-A, unif, wholeT_0.5	15	18	**3**	34
CBN, unif, singleC	16	17	**5**	29
CBN, unif, wholeT_0.01	17	14	**4**	27
CBN, unif, wholeT_0.5	18	19	6	32
CBN-A, last, wholeT_0.01	19	15	12	25
CBN, last, singleC	20	22	10	35
CBN, last, wholeT_0.01	21	20	9	30
CBN-A, last, singleC	22	21	13	31
CBN-A, last, wholeT_0.5	23	23	11	33
CBN, last, wholeT_0.5	24	24	8	36
DiP-A, last, wholeT_0.01	25	26	25	21
DiP-A, unif, wholeT_0.01	26	25	26	20
DiP, last, wholeT_0.01	27	29	30	9
DiP, unif, wholeT_0.01	28	32	29	19
DiP-A, last, singleC	29	27	27	17
DiP-A, last, wholeT_0.5	30	33	28	14
DiP-A, unif, singleC	31	28	31	**4**
DiP-A, unif, wholeT_0.5	32	34	32	7
DiP, last, wholeT_0.5	33	35	34	**5**
DiP, last, singleC	34	30	35	8
DiP, unif, singleC	35	31	33	**3**
DiP, unif, wholeT_0.5	36	36	36	6

Table 3: Overall ranking of all 36 combinations of method and sampling when Drivers are Known with respect to each performance measure. S.Size = 1000. Only graphs with Conjunction. Methods have been ordered by their performance in the first performance measure. Best 5 methods are shown in bold.

Method and sampling	Diff	PFD	PND	FPF
OT-A, last, singleC	**1**	**4**	9	12
OT-A, last, wholeT_0.5	**2**	**5**	16	13
OT-A, last, wholeT_0.01	**3**	9	7	18
OT-A, unif, singleC	**4**	**2**	20	7.5
OT, unif, singleC	**5**	**1**	21	7.5
OT, unif, wholeT_0.5	6.5	6	23.5	9.5
OT-A, unif, wholeT_0.5	6.5	7	23.5	9.5
OT-A, unif, wholeT_0.01	8	12	17	24
OT, last, singleC	9	8	22	**2**
OT, last, wholeT_0.01	10	**3**	19	11
OT, unif, wholeT_0.01	11	11	18	23
DiP-A, last, wholeT_0.01	12	13	15	21
OT, last, wholeT_0.5	13	10	26	**1**
CBN-A, unif, wholeT_0.01	14	23	**1**	26
DiP, last, wholeT_0.01	15	16	29	15
DiP-A, unif, wholeT_0.01	16	14	28	20
CBN-A, unif, singleC	17	27	**3**	25
CBN, unif, singleC	18	26	**4**	29
CBN-A, unif, wholeT_0.5	19	28	6	28
CBN-A, last, wholeT_0.01	20	30	14	27
DiP-A, last, singleC	21	19	27	19
DiP-A, last, wholeT_0.5	22	21	25	17
CBN, unif, wholeT_0.01	23	29	**2**	30
CBN, unif, wholeT_0.5	24	31	**5**	33
DiP-A, unif, singleC	25	15	31	**4**
DiP, unif, wholeT_0.01	26	18	30	22
CBN, last, singleC	27	34	8	34
CBN, last, wholeT_0.01	28	33	10	32
DiP-A, unif, wholeT_0.5	29	20	32	**5**
CBN-A, last, singleC	30	32	11	31
DiP, last, wholeT_0.5	31	25	34	14
DiP, unif, singleC	32	17	33	**3**
DiP, last, singleC	33	22	35	16
CBN-A, last, wholeT_0.5	34	35	13	35
CBN, last, wholeT_0.5	35	36	12	36
DiP, unif, wholeT_0.5	36	24	36	6

Table 4: Overall ranking of all 36 combinations of method and sampling when Drivers are Known with respect to each performance measure. S.Size = 200. Only graphs with Conjunction. Methods have been ordered by their performance in the first performance measure. Best 5 methods are shown in bold.

Method and sampling	Diff	PFD	PND	FPF
OT-A, last, singleC	**1**	**2**	14	10
OT-A, last, wholeT_0.5	**2**	**1**	15	14
OT-A, last, wholeT_0.01	**3**	**3**	10	24
OT-A, unif, singleC	**4**	11	19	16.5
OT, unif, singleC	**5**	7	20	16.5
OT, unif, wholeT_0.5	6.5	6	21	12.5
OT-A, unif, wholeT_0.5	6.5	8	22	12.5
OT-A, unif, wholeT_0.01	8	9	16	22.5
OT, unif, wholeT_0.01	9	12	17	22.5
OT, last, singleC	10	10	23	**1**
OT, last, wholeT_0.5	11	**4**	24	**2**
OT, last, wholeT_0.01	12	**5**	18	18
CBN-A, unif, wholeT_0.5	13	16	**1**	35
CBN-A, unif, singleC	14	15	**2**	32
CBN-A, unif, wholeT_0.01	15	13	**3**	29
CBN, unif, wholeT_0.5	16	19	**5**	31
CBN, unif, singleC	17	17	6	28
CBN, unif, wholeT_0.01	18	18	**4**	27
CBN-A, last, wholeT_0.01	19	14	11	25
CBN-A, last, singleC	20	22	13	26
CBN, last, singleC	21	23	9	34
CBN-A, last, wholeT_0.5	22	21	12	33
CBN, last, wholeT_0.01	23	20	7	30
CBN, last, wholeT_0.5	24	24	8	36
DiP-A, unif, wholeT_0.01	25	25	26	20
DiP-A, last, wholeT_0.01	26	27	25	21
DiP, unif, wholeT_0.01	27	31	29	19
DiP-A, last, singleC	28	26	27	15
DiP-A, last, wholeT_0.5	29	33	28	11
DiP, last, wholeT_0.01	30	30	32	9
DiP-A, unif, singleC	31	28	30	8
DiP-A, unif, wholeT_0.5	32	34	31	6.5
DiP, last, singleC	33	29	35	**5**
DiP, unif, singleC	34.5	32	33.5	**4**
DiP, unif, wholeT_0.5	34.5	36	33.5	6.5
DiP, last, wholeT_0.5	36	35	36	**3**

Table 5: Overall ranking of all 36 combinations of method and sampling when Drivers are Known with respect to each performance measure. S.Size = 100. Only graphs with Conjunction. Methods have been ordered by their performance in the first performance measure. Best 5 methods are shown in bold.

Method and sampling	Diff	PFD	PND	FPF
OT-A, last, singleC	**1**	**3**	14	13
OT-A, last, wholeT_0.5	**2**	**2**	15	12
OT-A, last, wholeT_0.01	**3**	**1**	10	22
OT, unif, singleC	**4**	9	22	14
OT-A, unif, singleC	**5**	10	20	15
OT-A, unif, wholeT_0.5	6	12	19	16.5
OT, unif, wholeT_0.5	7	11	21	16.5
OT-A, unif, wholeT_0.01	8	6	17	23.5
OT, last, wholeT_0.01	9	**4**	16	19
OT, last, wholeT_0.5	10	**5**	24	**2.5**
OT, last, singleC	11	7	23	**4**
OT, unif, wholeT_0.01	12	8	18	23.5
CBN-A, unif, singleC	13	16	**2**	30
CBN-A, unif, wholeT_0.01	14	14	**1**	28
CBN, unif, wholeT_0.01	15	13	**4**	26
CBN-A, unif, wholeT_0.5	16	19	**3**	35
CBN, unif, singleC	17	18	**5**	32
CBN, unif, wholeT_0.5	18	20	6	33
CBN-A, last, wholeT_0.01	19	15	13	25
CBN, last, wholeT_0.01	20	17	9	27
CBN-A, last, wholeT_0.5	21	22	8	29
CBN, last, singleC	22	21	11	34
CBN, last, wholeT_0.5	23	24	7	36
CBN-A, last, singleC	24	23	12	31
DiP-A, unif, wholeT_0.01	25	28	25	20
DiP, last, wholeT_0.01	26	31	28	9
DiP, unif, wholeT_0.01	27	32	27	18
DiP-A, last, wholeT_0.01	28	29	26	21
DiP, last, singleC	29	26	30	**1**
DiP, last, wholeT_0.5	30	34	32.5	**2.5**
DiP-A, last, singleC	31	25	29	7
DiP-A, unif, singleC	33.5	27	31	8
DiP, unif, singleC	33.5	30	35	6
DiP-A, unif, wholeT_0.5	33.5	35	35	11
DiP, unif, wholeT_0.5	33.5	36	35	10
DiP-A, last, wholeT_0.5	36	33	32.5	**5**

2.1.2 Overall ranking: Drivers Known, no conjunction

Table 6: Overall ranking of all 36 combinations of method and sampling when Drivers are Known with respect to each performance measure. Only graphs without Conjunction. Methods have been ordered by their performance in the first performance measure. Best 5 methods are shown in bold.

Method and sampling	Diff	PFD	PND	FPF
OT-A, last, singleC	**1**	**3**	**2**	15
OT-A, last, wholeT_0.5	**2**	**4**	**3**	14
OT-A, last, wholeT_0.01	**3**	6	**1**	22
OT-A, unif, singleC	**4**	9	12.5	9.5
OT, unif, singleC	**5**	7	12.5	9.5
OT, unif, wholeT_0.5	6.5	8	15.5	12.5
OT-A, unif, wholeT_0.5	6.5	10	15.5	12.5
OT-A, unif, wholeT_0.01	8	11	**5**	24
OT, unif, wholeT_0.01	9	12	7	23
OT, last, singleC	10	**1**	17	**2**
OT, last, wholeT_0.5	11	**2**	23	**1**
OT, last, wholeT_0.01	12	**5**	14	18
CBN-A, unif, wholeT_0.01	13	13	**4**	26
CBN-A, unif, wholeT_0.5	14	16	8	31
CBN-A, unif, singleC	15	15	9	29
CBN, unif, wholeT_0.5	16	18	11	32
CBN, unif, singleC	17	19	10	34
CBN, unif, wholeT_0.01	18	14	6	27
CBN-A, last, wholeT_0.01	19	17	20	25
DiP-A, last, wholeT_0.01	20	20	25	21
CBN, last, singleC	21	28	18	35
CBN-A, last, wholeT_0.5	22	25	24	33
CBN, last, wholeT_0.01	23	21	21	28
CBN-A, last, singleC	24	23	22	30
DiP-A, unif, wholeT_0.01	25	26	26	20
CBN, last, wholeT_0.5	26	29	19	36
DiP, last, wholeT_0.01	27	27	29	16
DiP-A, last, singleC	28	22	27	17
DiP, unif, wholeT_0.01	29	31	30	19
DiP-A, last, wholeT_0.5	30	33	28	11
DiP-A, unif, singleC	31	30	31	6
DiP, last, singleC	32	24	34	7
DiP-A, unif, wholeT_0.5	33	35	32	8
DiP, last, wholeT_0.5	34	34	36	**5**
DiP, unif, singleC	35	32	33	**3**
DiP, unif, wholeT_0.5	36	36	35	**4**

Table 7: Overall ranking of all 36 combinations of method and sampling when Drivers are Known with respect to each performance measure. S.Size = 1000. Only graphs without Conjunction. Methods have been ordered by their performance in the first performance measure. Best 5 methods are shown in bold.

Method and sampling	Diff	PFD	PND	FPF
OT-A, last, singleC	**1**	9	**2**	14
OT-A, last, wholeT_0.5	**2**	8	**3**	12
OT, unif, wholeT_0.5	**3.5**	**3**	7.5	7.5
OT-A, unif, wholeT_0.5	**3.5**	**4**	7.5	7.5
OT-A, last, wholeT_0.01	**5**	10	**1**	19
OT-A, unif, singleC	6	7	10.5	9.5
OT, unif, singleC	7	6	10.5	9.5
OT-A, unif, wholeT_0.01	8	11	**4**	24
DiP-A, last, wholeT_0.01	9	13	6	18
OT, unif, wholeT_0.01	10	12	**5**	23
OT, last, singleC	11	**2**	18	**2**
OT, last, wholeT_0.5	12	**1**	19	**1**
OT, last, wholeT_0.01	13	**5**	14	11
DiP, last, wholeT_0.01	14	14	28	13
DiP-A, last, singleC	15	17	21	20
DiP-A, last, wholeT_0.5	16	20	20	17
DiP-A, unif, wholeT_0.01	17	15	24	21
DiP-A, unif, singleC	18	19	30	**4**
DiP, last, singleC	19	16	35	16
DiP, unif, wholeT_0.01	20	18	31	22
CBN-A, unif, wholeT_0.5	21	27	12	29
CBN-A, unif, wholeT_0.01	22	24	9	26
DiP, last, wholeT_0.5	23	21	36	15
DiP-A, unif, wholeT_0.5	24	23	32	**5**
CBN-A, unif, singleC	25	28	13	28
CBN, unif, wholeT_0.5	26	29	17	30
DiP, unif, singleC	27	22	33	**3**
CBN-A, last, wholeT_0.01	28	30	25	25
DiP, unif, wholeT_0.5	29	25	34	6
CBN, unif, singleC	30	31	16	33
CBN, unif, wholeT_0.01	31	26	15	27
CBN-A, last, singleC	32	33	26	31
CBN, last, wholeT_0.5	33	34	22	36
CBN-A, last, wholeT_0.5	34	35	27	34
CBN, last, singleC	35	36	23	35
CBN, last, wholeT_0.01	36	32	29	32

Table 8: Overall ranking of all 36 combinations of method and sampling when Drivers are Known with respect to each performance measure. S.Size = 200. Only graphs without Conjunction. Methods have been ordered by their performance in the first performance measure. Best 5 methods are shown in bold.

Method and sampling	Diff	PFD	PND	FPF
OT-A, last, singleC	**1**	**2.5**	**2**	13.5
OT-A, last, wholeT_0.5	**2**	**2.5**	**4**	13.5
OT-A, last, wholeT_0.01	**3**	6	**1**	21
OT, unif, singleC	**4**	7	14.5	6.5
OT-A, unif, singleC	**5**	8	14.5	6.5
OT, unif, wholeT_0.5	6.5	10	22.5	11.5
OT-A, unif, wholeT_0.5	6.5	12	22.5	11.5
OT-A, unif, wholeT_0.01	8	9	10	23.5
OT, unif, wholeT_0.01	9	11	11	23.5
OT, last, singleC	10	**1**	18	**1.5**
OT, last, wholeT_0.5	11	**5**	24	**1.5**
OT, last, wholeT_0.01	12	**4**	12	17
CBN-A, unif, wholeT_0.01	13	13	**3**	30
CBN-A, unif, singleC	14	15	8	29
CBN-A, unif, wholeT_0.5	15	16	7	31
CBN, unif, singleC	16	17	6	32
CBN, unif, wholeT_0.5	17	19	9	34
CBN, unif, wholeT_0.01	18	14	**5**	27
CBN-A, last, wholeT_0.01	19	18	17	25
CBN, last, singleC	20	22	19	35
CBN, last, wholeT_0.01	21	20	16	26
CBN-A, last, wholeT_0.5	22	23	21	33
CBN, last, wholeT_0.5	23	24	13	36
CBN-A, last, singleC	24	21	20	28
DiP-A, last, wholeT_0.01	25	25	25	22
DiP-A, unif, wholeT_0.01	26	27	26	20
DiP-A, last, singleC	27	26	27	18
DiP, unif, wholeT_0.01	28	31	29	19
DiP-A, last, wholeT_0.5	29	32	28	15
DiP, last, wholeT_0.01	30	29	30	16
DiP-A, unif, singleC	31	30	31	9
DiP-A, unif, wholeT_0.5	32	35	32	10
DiP, last, singleC	33	28	33	8
DiP, last, wholeT_0.5	34	34	34	**3**
DiP, unif, singleC	35	33	36	**5**
DiP, unif, wholeT_0.5	36	36	35	**4**

Table 9: Overall ranking of all 36 combinations of method and sampling when Drivers are Known with respect to each performance measure. S.Size = 100. Only graphs without Conjunction. Methods have been ordered by their performance in the first performance measure. Best 5 methods are shown in bold.

Method and sampling	Diff	PFD	PND	FPF
OT-A, last, singleC	**1**	**1**	**2**	11
OT-A, last, wholeT_0.5	**2**	**2**	**5**	12
OT-A, last, wholeT_0.01	**3**	**5**	**1**	22
OT-A, unif, singleC	**4**	11	19.5	14.5
OT, unif, singleC	**5**	9	19.5	14.5
OT, unif, wholeT_0.5	6.5	10	22.5	16.5
OT-A, unif, wholeT_0.5	6.5	12	22.5	16.5
OT-A, unif, wholeT_0.01	8	7	10	23.5
OT, unif, wholeT_0.01	9	8	11	23.5
OT, last, wholeT_0.5	10	**4**	24	**2.5**
OT, last, singleC	11	**3**	21	**4**
OT, last, wholeT_0.01	12	6	15	18
CBN-A, unif, wholeT_0.5	13	18	8	32
CBN-A, unif, wholeT_0.01	14	13	**4**	26
CBN-A, unif, singleC	15	16	6	31
CBN, unif, wholeT_0.5	16	19	7	33
CBN, unif, singleC	17	20	9	34
CBN, unif, wholeT_0.01	18	14	**3**	29
CBN-A, last, wholeT_0.01	19	15	18	25
CBN, last, singleC	20	22	12	36
CBN-A, last, wholeT_0.5	21	21	17	28
CBN, last, wholeT_0.01	22	17	13	27
CBN-A, last, singleC	23	23	16	30
CBN, last, wholeT_0.5	24	24	14	35
DiP-A, unif, wholeT_0.01	25	30	25	20
DiP-A, last, wholeT_0.01	26	27	26	21
DiP, last, wholeT_0.01	27	31	28	10
DiP, unif, wholeT_0.01	28	32	27	19
DiP, last, singleC	29	26	32	**1**
DiP-A, unif, singleC	30	28	31	9
DiP-A, unif, wholeT_0.5	31	35	29	13
DiP, last, wholeT_0.5	32	34	35.5	**2.5**
DiP, unif, wholeT_0.5	33	36	34	8
DiP, unif, singleC	34	29	33	7
DiP-A, last, wholeT_0.5	35	33	35.5	**5**
DiP-A, last, singleC	36	25	30	6

2.2 Overall ranking: Drivers Unknown

Table 10: Overall ranking of all 144 combinations of method, filtering, and sampling when Drivers are Unknown with respect to each performance performance measure. Methods have been ordered by their performance in the first performance measure. Best 10 methods are shown in bold.

Method and sampling	Diff	PFD	PND	FPF
S5, OT-A, last, singleC	**1**	**8**	13	60
S5, OT-A, last, wholeT_0.5	**2**	**6**	23	59
S5, OT-A, last, wholeT_0.01	**3**	**4**	**4**	94
J5, OT-A, last, wholeT_0.01	**4**	**1**	29	71
J1, OT-A, last, singleC	**5**	14	37	76
S5, OT-A, unif, wholeT_0.01	**6**	17	33	70
J1, OT-A, last, wholeT_0.5	**7**	**7**	39	75
S5, OT, unif, singleC	**8**	41	58.5	25
S5, OT-A, unif, singleC	**9**	50	58.5	28
S5, OT, unif, wholeT_0.01	**10**	**9**	34	69
S5, OT, unif, wholeT_0.5	11	28	65	30.5
S5, OT-A, unif, wholeT_0.5	12	47	64	30.5
S1, OT-A, last, wholeT_0.5	13	31	**3**	116
J1, OT-A, last, wholeT_0.01	14	19	**9**	109
S1, OT-A, unif, wholeT_0.5	15	38	18	102
S1, OT, unif, wholeT_0.5	16	23	20	101
S1, OT-A, last, singleC	17	34	**2**	120
S1, OT, unif, singleC	18	24	12	106
S1, OT-A, unif, singleC	19	35	11	107
J1, OT-A, unif, singleC	20	30	63	40
J1, OT, unif, singleC	21	18	66	35
S5, OT, last, singleC	22	13	45	20
S5, OT, last, wholeT_0.5	23	**3**	52	17
J5, OT-A, last, singleC	24	33	55	46
J1, OT-A, unif, wholeT_0.5	25	26	69.5	45
J1, OT, unif, wholeT_0.5	26	11	69.5	44
J5, OT-A, last, wholeT_0.5	27	29	56	43
J1, OT-A, unif, wholeT_0.01	28	16	35	92
J1, OT, unif, wholeT_0.01	29	12	36	91
S1, OT-A, last, wholeT_0.01	30	49	**1**	134
S5, DiP-A, unif, wholeT_0.5	31	137	122	29
S5, DiP-A, unif, singleC	32	117	120	26
J5, OT-A, unif, wholeT_0.01	33	63	94	38.5
J5, OT, last, wholeT_0.01	34	**5**	61	37
S5, OT, last, wholeT_0.01	35	**2**	27	82
J5, OT, unif, wholeT_0.01	36	53	95	38.5
S5, CBN-A, unif, wholeT_0.01	37	15	38	93
S5, CBN-A, unif, singleC	38	43	71	83
S5, DiP, unif, wholeT_0.5	39	143	128	19
J1, OT, last, singleC	40	27	83	58
S5, DiP-A, unif, wholeT_0.01	41	106	86	67
J1, OT, last, wholeT_0.5	42	**10**	87	54
S5, DiP, unif, singleC	43	123	129	16
J1, CBN-A, unif, singleC	44	37	74	74
S5, CBN-A, unif, wholeT_0.5	45	45	73	84
J1, CBN-A, unif, wholeT_0.5	46	39	77	68
J5, DiP-A, unif, wholeT_0.01	47	129	133	33
J1, OT, last, wholeT_0.01	48	20	40	97
J5, DiP-A, unif, singleC	49	128	138	**9**
S5, DiP, unif, wholeT_0.01	50	120	98	66

Table 10: *(continued)*

Method and sampling	Diff	PFD	PND	FPF
J5, DiP, unif, wholeT_0.01	51	139	142	32
J5, OT, last, singleC	52	40	103	**4**
S1, OT, last, wholeT_0.5	53	32	28	110
J5, DiP-A, last, singleC	54	108	121	34
J5, DiP-A, unif, wholeT_0.5	55	141	141	11
J1, DiP-A, unif, singleC	56	118	127	27
S1, OT, last, singleC	57	44	25	111
J1, DiP-A, unif, wholeT_0.5	58	138	130	23
J5, DiP-A, last, wholeT_0.5	59	130	125	36
J5, DiP-A, last, wholeT_0.01	60	105	88	73
S5, CBN, unif, wholeT_0.01	61	22	46	104
J5, DiP, unif, singleC	62	132	143	**8**
J5, OT-A, unif, singleC	63	80	115	14.5
J5, DiP, unif, wholeT_0.5	64	144	144	**4**
J5, OT, unif, singleC	65	70	116	14.5
J1, CBN, unif, singleC	66	46	89	81
J5, OT, last, wholeT_0.5	67	21	105	**4**
S5, CBN, unif, singleC	68	52	82	88
J1, CBN-A, unif, wholeT_0.01	69	25	41	108
S5, DiP-A, last, wholeT_0.5	70	125	92	49
J1, CBN, unif, wholeT_0.5	71	36	93	77
S5, DiP-A, last, singleC	72	100	81	51
J5, OT-A, unif, wholeT_0.5	73	75	118	21.5
J1, DiP, unif, singleC	74	124	136	18
J5, OT, unif, wholeT_0.5	75	57	119	21.5
S5, CBN, unif, wholeT_0.5	76	48	90	87
J1, DiP-A, last, singleC	77	104	108	42
J1, DiP-A, last, wholeT_0.5	78	127	110	47
J1, DiP, unif, wholeT_0.5	79	142	137	13
J5, DiP, last, singleC	80	119	140	**4**
J5, DiP, last, wholeT_0.5	81	136	139	**4**
J1, DiP-A, unif, wholeT_0.01	82	103	96	80
S1, OT, last, wholeT_0.01	83	51	17	132
S5, DiP, last, wholeT_0.5	84	131	113	**10**
S1, OT-A, unif, wholeT_0.01	85	61	**5**	136
J5, CBN-A, unif, wholeT_0.01	86	73	100	85
J5, CBN-A, last, wholeT_0.01	87	55	50	114
J5, CBN-A, unif, singleC	88	85	124	56
J5, CBN-A, unif, wholeT_0.5	89	87	123	53
S1, OT, unif, wholeT_0.01	90	59	**6**	135
S5, DiP, last, singleC	91	109	112	12
J1, DiP, last, wholeT_0.5	92	133	135	**4**
J1, DiP, last, singleC	93	114	134	**4**
J1, CBN, unif, wholeT_0.01	94	42	49	113
J1, DiP, unif, wholeT_0.01	95	111	111	78
J5, DiP, last, wholeT_0.01	96	121	126	24
S5, DiP-A, last, wholeT_0.01	97	96	54	89
J5, CBN, unif, wholeT_0.01	98	90	109	86
J5, CBN-A, last, wholeT_0.5	99	93	80	103
J1, DiP-A, last, wholeT_0.01	100	101	67	90

Table 10: *(continued)*

Method and sampling	Diff	PFD	PND	FPF
J5, CBN, unif, singleC	101	99	131	62
J5, CBN-A, last, singleC	102	81	84	105
J5, CBN, unif, wholeT_0.5	103	84	132	61
J1, CBN-A, last, singleC	104	72	62	119
J5, CBN, last, singleC	105	78	99	99
J5, CBN, last, wholeT_0.5	106	76	101	96
J5, CBN, last, wholeT_0.01	107	67	60	112
S1, CBN-A, unif, wholeT_0.5	108	64	31	125
J1, CBN-A, last, wholeT_0.5	109	74	68	118
J1, CBN, last, singleC	110	66	72	117
S1, CBN-A, unif, singleC	111	60	24	128
S1, DiP-A, unif, wholeT_0.5	112	135	104	57
J1, CBN, last, wholeT_0.5	113	68	76	115
S1, CBN, unif, wholeT_0.5	114	56	32	126
S1, DiP-A, unif, singleC	115	115	97	55
S5, CBN, last, singleC	116	58	44	123
S5, CBN, last, wholeT_0.5	117	54	47	122
S1, DiP, unif, wholeT_0.5	118	140	117	50
S5, CBN-A, last, singleC	119	62	48	124
S1, CBN, unif, singleC	120	65	30	129
S5, CBN-A, last, wholeT_0.5	121	69	51	121
S5, DiP, last, wholeT_0.01	122	113	91	65
J1, DiP, last, wholeT_0.01	123	116	107	64
S1, DiP, unif, singleC	124	122	114	48
J1, CBN-A, last, wholeT_0.01	125	79	42	127
S5, CBN-A, last, wholeT_0.01	126	71	22	131
S1, DiP-A, last, wholeT_0.01	127	97	53	95
S1, DiP-A, last, wholeT_0.5	128	126	79	63
J1, CBN, last, wholeT_0.01	129	83	43	130
S1, DiP-A, last, singleC	130	102	75	72
S5, CBN, last, wholeT_0.01	131	77	26	133
S1, DiP-A, unif, wholeT_0.01	132	98	57	100
S1, DiP, last, wholeT_0.5	133	134	106	41
S1, DiP, last, wholeT_0.01	134	112	85	79
S1, DiP, last, singleC	135	107	102	52
S1, DiP, unif, wholeT_0.01	136	110	78	98
S1, CBN-A, last, wholeT_0.5	137	91	21	138
S1, CBN-A, unif, wholeT_0.01	138	82	**7**	143
S1, CBN-A, last, singleC	139	86	19	141
S1, CBN-A, last, wholeT_0.01	140	94	**10**	137
S1, CBN, last, wholeT_0.5	141	89	15	139
S1, CBN, last, singleC	142	92	16	142
S1, CBN, unif, wholeT_0.01	143	88	**8**	144
S1, CBN, last, wholeT_0.01	144	95	14	140

2.2.1 Overall ranking: Drivers Unknown, conjunction

Table 11: Overall ranking of all 144 combinations of method, filtering, and sampling when Drivers are Unknown with respect to each performance measure. Only graphs with Conjunction. Methods have been ordered by their performance in the first performance measure. Best 10 methods are shown in bold.

Method and sampling	Diff	PFD	PND	FPF
S5, OT-A, last, singleC	**1**	**7**	15	60
S5, OT-A, last, wholeT_0.5	**2**	**6**	23	59
J5, OT-A, last, wholeT_0.01	**3**	**1**	26	71
S5, OT-A, last, wholeT_0.01	**4**	**2**	**2**	94
S5, OT-A, unif, wholeT_0.01	**5**	17	33	69
S5, OT, unif, singleC	**6**	41	61.5	28
S5, OT-A, unif, singleC	**7**	50	61.5	31
J1, OT-A, last, singleC	**8**	15	38	80
J1, OT-A, last, wholeT_0.5	**9**	**10**	39	79
S5, OT-A, unif, wholeT_0.5	**10**	47	67	29.5
S5, OT, unif, wholeT_0.01	11	11	34	68
S5, OT, unif, wholeT_0.5	12	30	70	29.5
J1, OT-A, last, wholeT_0.01	13	21	**9**	109
S1, OT-A, unif, wholeT_0.5	14	40	17	102
S1, OT, unif, wholeT_0.5	15	23	18	101
S1, OT, unif, singleC	16	22	12	104
S1, OT-A, unif, singleC	17	34	11	106
S1, OT-A, last, wholeT_0.5	18	39	**3**	117
J1, OT, unif, singleC	19	18	63	40
J1, OT-A, unif, singleC	20	32	60	46
S1, OT-A, last, singleC	21	38	**4**	120
J5, OT-A, last, singleC	22	26	53	43.5
S5, OT, last, singleC	23	16	51	21
S5, OT, last, wholeT_0.5	24	**3**	55	16
J5, OT-A, last, wholeT_0.5	25	24	54	43.5
J1, OT-A, unif, wholeT_0.5	26	28	68.5	45
J1, OT, unif, wholeT_0.5	27	13	68.5	42
J1, OT-A, unif, wholeT_0.01	28	12	35	92
J1, OT, unif, wholeT_0.01	29	**9**	36	91
S5, CBN-A, unif, wholeT_0.01	30	**4**	37	89
J5, OT, last, wholeT_0.01	31	**5**	59	32
S5, DiP-A, unif, wholeT_0.5	32	136	125	20
S5, DiP-A, unif, singleC	33	118	124	23
J5, OT-A, unif, wholeT_0.01	34	60	92	36.5
S5, CBN-A, unif, singleC	35	29	66	77
S1, OT-A, last, wholeT_0.01	36	48	**1**	134
J5, OT, unif, wholeT_0.01	37	55	94	36.5
S5, OT, last, wholeT_0.01	38	**8**	29	83
S5, CBN-A, unif, wholeT_0.5	39	42	75	78
S5, DiP-A, unif, wholeT_0.01	40	102	86	64
S5, DiP, unif, wholeT_0.5	41	143	130	14
J1, CBN-A, unif, singleC	42	33	71	72
J1, CBN-A, unif, wholeT_0.5	43	37	74	70
S5, DiP, unif, singleC	44	124	131	22
J5, DiP-A, unif, wholeT_0.01	45	126	129	35
J1, OT, last, singleC	46	31	88	62
J1, OT, last, wholeT_0.5	47	14	89	61
J5, OT, last, singleC	48	35	101	**4**
J5, DiP-A, unif, singleC	49	125	139	10.5
S5, DiP, unif, wholeT_0.01	50	120	103	63

Table 11: *(continued)*

Method and sampling	Diff	PFD	PND	FPF
J5, DiP-A, last, singleC	51	105	112	34
J1, OT, last, wholeT_0.01	52	27	42	99
J1, DiP-A, unif, singleC	53	117	126	26
J5, DiP, unif, wholeT_0.01	54	138	142	33
J5, DiP-A, unif, wholeT_0.5	55	140	141	10.5
J1, DiP-A, unif, wholeT_0.5	56	133	128	27
J5, OT, last, wholeT_0.5	57	20	102	**4**
S5, CBN, unif, wholeT_0.01	58	19	47	97
J5, DiP-A, last, wholeT_0.5	59	130	113	39
J1, CBN, unif, singleC	60	46	81	84
J5, DiP, unif, singleC	61	131	143	**8**
J1, CBN-A, unif, wholeT_0.01	62	25	40	108
S5, CBN, unif, singleC	63	54	82	88
J5, DiP, unif, wholeT_0.5	64	144	144	**4**
J5, OT, unif, singleC	65.5	69	114.5	17.5
J5, OT-A, unif, singleC	65.5	80	114.5	17.5
J5, DiP-A, last, wholeT_0.01	67	107	83	73
J1, CBN, unif, wholeT_0.5	68	36	85	75
S5, CBN, unif, wholeT_0.5	69	45	91	87
J1, DiP, unif, singleC	70	123	136	19
J1, DiP, unif, wholeT_0.5	71	142	137	15
S5, DiP-A, last, wholeT_0.5	72	127	96	51
S5, DiP-A, last, singleC	73	100	87	50
J5, OT-A, unif, wholeT_0.5	74	77	117.5	24.5
J5, OT, unif, wholeT_0.5	75	59	117.5	24.5
S1, OT, last, wholeT_0.5	76	43	31	110
J1, DiP-A, last, singleC	77	104	106	47
J1, DiP-A, unif, wholeT_0.01	78	98	93	81
J5, DiP, last, singleC	79	115	140	**4**
J1, DiP-A, last, wholeT_0.5	80	128	108	53
S1, OT, last, singleC	81	49	30	113
J5, DiP, last, wholeT_0.5	82	137	138	**4**
S5, DiP, last, wholeT_0.5	83	134	120	**9**
S1, OT, last, wholeT_0.01	84	51	20	132
J5, CBN-A, unif, wholeT_0.5	85	89	123	52
J5, CBN-A, unif, singleC	86	85	121	56
J5, CBN-A, unif, wholeT_0.01	87	72	99	85
J1, CBN, unif, wholeT_0.01	88	44	43	111
S5, DiP, last, singleC	89	113	119	12
J1, DiP, unif, wholeT_0.01	90	109	109	76
J1, DiP, last, wholeT_0.5	91	132	134	**4**
J1, DiP, last, singleC	92	110	135	**4**
J5, CBN-A, last, wholeT_0.01	93	57	50	114
J5, DiP, last, wholeT_0.01	94	121	127	13
J5, CBN-A, last, wholeT_0.5	95	95	76	105
J5, CBN, unif, wholeT_0.01	96	91	104	86
J5, CBN, unif, wholeT_0.5	97	83	133	57
J5, CBN, unif, singleC	98	96	132	58
J5, CBN-A, last, singleC	99	78	78	107
J1, CBN-A, last, singleC	100	73	58	119

Table 11: *(continued)*

Method and sampling	Diff	PFD	PND	FPF
S1, CBN-A, unif, wholeT_0.5	101	63	28	126
S1, OT-A, unif, wholeT_0.01	102	65	**5**	136
J5, CBN, last, wholeT_0.01	103	71	64	112
S1, CBN-A, unif, singleC	104	58	22	127
S5, DiP-A, last, wholeT_0.01	105	99	56	90
J5, CBN, last, singleC	106	79	97	103
J5, CBN, last, wholeT_0.5	107	75	98	98
J1, CBN-A, last, wholeT_0.5	108	76	65	118
S1, OT, unif, wholeT_0.01	109	62	**6**	135
J1, CBN, last, singleC	110	70	73	116
J1, DiP-A, last, wholeT_0.01	111	106	72	93
S5, CBN-A, last, wholeT_0.5	112	67	48	122
S1, DiP-A, unif, wholeT_0.5	113	135	105	55
S1, CBN, unif, wholeT_0.5	114	56	32	124
S5, CBN, last, wholeT_0.5	115	52	46	121
S5, CBN, last, singleC	116	53	45	123
S5, CBN-A, last, singleC	117	61	44	125
J1, CBN, last, wholeT_0.5	118	68	77	115
S1, DiP-A, unif, singleC	119	112	100	48
S1, DiP, unif, wholeT_0.5	120	141	122	49
S1, CBN, unif, singleC	121	66	27	128
S5, DiP, last, wholeT_0.01	122	114	95	66
S1, DiP, unif, singleC	123	122	116	38
J1, DiP, last, wholeT_0.01	124	119	110	65
J1, CBN-A, last, wholeT_0.01	125	81	41	129
S5, CBN-A, last, wholeT_0.01	126	64	19	130
S1, DiP-A, last, wholeT_0.01	127	101	52	96
J1, CBN, last, wholeT_0.01	128	88	49	131
S1, DiP-A, last, wholeT_0.5	129	129	84	67
S1, DiP-A, last, singleC	130	103	80	74
S5, CBN, last, wholeT_0.01	131	74	25	133
S1, DiP-A, unif, wholeT_0.01	132	97	57	100
S1, DiP, last, wholeT_0.5	133	139	111	41
S1, DiP, last, wholeT_0.01	134	116	90	82
S1, DiP, last, singleC	135	111	107	54
S1, DiP, unif, wholeT_0.01	136	108	79	95
S1, CBN-A, unif, wholeT_0.01	137	82	**7**	143
S1, CBN-A, last, wholeT_0.5	138	92	24	140
S1, CBN-A, last, singleC	139	86	21	141
S1, CBN, last, wholeT_0.5	140	87	14	138
S1, CBN, last, singleC	141	90	16	142
S1, CBN-A, last, wholeT_0.01	142	93	**10**	137
S1, CBN, unif, wholeT_0.01	143	84	**8**	144
S1, CBN, last, wholeT_0.01	144	94	13	139

Table 12: Overall ranking of all 144 combinations of method, filtering, and sampling when Drivers are Unknown with respect to each performance measure. S.Size = 1000. Only graphs with Conjunction. Methods have been ordered by their performance in the first performance measure. Best 10 methods are shown in bold.

Method and sampling	Diff	PFD	PND	FPF
S1, OT, unif, singleC	**1.5**	**3**	18.5	50.5
S1, OT-A, unif, singleC	**1.5**	**6**	18.5	50.5
S1, OT, unif, wholeT_0.5	**3.5**	**4**	27.5	57.5
S1, OT-A, unif, wholeT_0.5	**3.5**	17	27.5	57.5
S5, OT-A, last, singleC	**5**	25	20	53.5
S5, OT-A, last, wholeT_0.5	**6**	18	30	53.5
S5, OT-A, last, wholeT_0.01	**7**	16	**3**	96
S5, DiP-A, last, wholeT_0.5	**8**	75	38	67
S5, DiP-A, unif, wholeT_0.01	**9**	47	50	66
S5, DiP-A, unif, wholeT_0.5	**10**	107	90	13
S1, DiP-A, unif, singleC	11	68	47	44
J1, OT-A, last, wholeT_0.01	12	28	12	104
S5, DiP-A, last, singleC	13	61	34	69
S5, DiP-A, unif, singleC	14	85	87	26
S1, OT-A, last, wholeT_0.5	15	36	**6**	99
J5, OT-A, last, wholeT_0.01	16	**9**	35	77
S1, OT-A, last, singleC	17	40	**4**	107
S5, DiP-A, last, wholeT_0.01	18	37	**8**	100
J5, DiP-A, last, wholeT_0.01	19	41	42	86
J1, OT-A, last, wholeT_0.5	20.5	11	51.5	48.5
J1, OT-A, last, singleC	20.5	15	51.5	48.5
S5, OT, unif, wholeT_0.01	22.5	**10**	45.5	72
S5, OT-A, unif, wholeT_0.01	22.5	27	45.5	72
S5, OT, unif, singleC	24.5	42	88.5	13
S5, OT-A, unif, singleC	24.5	65	88.5	13
S1, DiP-A, unif, wholeT_0.5	26	90	62	45
J1, DiP-A, last, wholeT_0.01	27	43	14	102
S5, OT, unif, wholeT_0.5	28.5	29	93.5	13
S5, OT-A, unif, wholeT_0.5	28.5	52	93.5	13
S5, DiP, unif, wholeT_0.01	30	72	67	72
S5, DiP, unif, wholeT_0.5	31	127	108	13
J1, OT, unif, wholeT_0.01	32.5	**1**	48.5	75.5
J1, OT-A, unif, wholeT_0.01	32.5	**2**	48.5	75.5
S5, DiP, last, wholeT_0.5	34	80	75	13
S5, DiP, unif, singleC	35	97	109	32.5
J1, DiP-A, unif, wholeT_0.01	36	20	58	81
J1, DiP-A, unif, singleC	37	84	98	32.5
S5, DiP, last, singleC	38	57	78	27
J1, DiP-A, last, singleC	39.5	60	69.5	61.5
J1, DiP-A, last, wholeT_0.5	39.5	76	69.5	61.5
S5, OT, last, singleC	41	26	64	13
S1, DiP, unif, singleC	42	86	72	39
S1, CBN-A, unif, wholeT_0.5	43	66	40	110
S5, OT, last, wholeT_0.5	44	**8**	74	13
J1, DiP-A, unif, wholeT_0.5	45	102	110	32.5
S1, CBN-A, unif, singleC	46	46	31	108
S1, DiP, unif, wholeT_0.5	47	122	79	52
J1, OT, unif, singleC	48.5	21	91.5	32.5
J1, OT-A, unif, singleC	48.5	33	91.5	32.5
J5, DiP-A, last, singleC	50	78	82.5	59.5

Table 12: *(continued)*

Method and sampling	Diff	PFD	PND	FPF
J1, DiP, unif, wholeT_0.01	51	34	80	80
J5, OT-A, last, singleC	52	38	76.5	46.5
J5, DiP-A, last, wholeT_0.5	53	92	82.5	59.5
S5, OT, last, wholeT_0.01	54	13	32	79
J1, DiP, unif, singleC	55	94	121	32.5
J5, OT-A, last, wholeT_0.5	56	32	76.5	46.5
S1, OT-A, last, wholeT_0.01	57	91	**1**	132
J1, OT, last, wholeT_0.01	58	23	57	87
J1, DiP, last, wholeT_0.5	59	77	116	13
J1, OT, unif, wholeT_0.5	60.5	12	100.5	32.5
J1, OT-A, unif, wholeT_0.5	60.5	31	100.5	32.5
S5, DiP, last, wholeT_0.01	62	64	43	84
J1, DiP, unif, wholeT_0.5	63	124	124	32.5
J5, OT, last, wholeT_0.01	64	**5**	85	38
J1, DiP, last, singleC	65	53	117	13
S1, CBN, unif, wholeT_0.5	66	45	44	105
S1, CBN, unif, singleC	67	70	37	113
S5, CBN-A, unif, wholeT_0.01	68	14	55	93
J5, DiP, last, wholeT_0.01	69	50	99	32.5
J1, CBN-A, unif, wholeT_0.01	70	19	53	97
S5, CBN-A, unif, singleC	71	48	96	88
J1, DiP, last, wholeT_0.01	72	51	73	74
J5, DiP-A, unif, wholeT_0.01	73	121	128	41.5
S5, CBN-A, unif, wholeT_0.5	74	54	102	85
S1, OT, last, wholeT_0.5	75	44	39	90
J1, OT, last, singleC	76	24	111	13
S1, OT, last, singleC	77	69	36	95
J5, DiP, last, wholeT_0.5	78	98	127	13
J5, DiP, last, singleC	79	79	130	13
S1, DiP-A, last, wholeT_0.01	80	56	**2**	118
J1, OT, last, wholeT_0.5	81	**7**	113	13
J5, OT, unif, wholeT_0.01	82.5	74	114.5	41.5
J5, OT-A, unif, wholeT_0.01	82.5	87	114.5	41.5
S1, DiP-A, last, wholeT_0.5	84	83	25	89
S1, DiP-A, last, singleC	85	55	16	92
J5, DiP-A, unif, singleC	86	134	133	13
J1, CBN-A, unif, singleC	87	49	106	68
J5, DiP-A, unif, wholeT_0.5	88	141	137	13
J5, DiP, unif, wholeT_0.01	89	143	140	41.5
J1, CBN-A, unif, wholeT_0.5	90	62	107	63
J5, OT, last, singleC	91	39	123	13
J5, DiP, unif, wholeT_0.5	92	144	142	13
J5, DiP, unif, singleC	93	142	141	13
S1, OT, last, wholeT_0.01	94	96	21	126
S5, CBN, unif, wholeT_0.01	95	35	65	109
J5, OT, last, wholeT_0.5	96	22	125	13
S1, DiP, last, wholeT_0.5	97	93	61	78
J1, CBN, unif, wholeT_0.01	98	30	59	111
J1, CBN, unif, singleC	99	71	118	83
S1, OT, unif, wholeT_0.01	100.5	110	**7**	135

Table 12: *(continued)*

Method and sampling	Diff	PFD	PND	FPF
S1, OT-A, unif, wholeT_0.01	100.5	112	**5**	136
S1, DiP, last, wholeT_0.01	102	59	33	103
J5, OT, unif, singleC	103.5	89	133	13
J5, OT-A, unif, singleC	103.5	114	133	13
S5, CBN, unif, singleC	105	88	112	101
S1, DiP-A, unif, wholeT_0.01	106	58	**10**	112
J1, CBN, unif, wholeT_0.5	107	63	120	70
J5, OT, unif, wholeT_0.5	108.5	81	137	13
J5, OT-A, unif, wholeT_0.5	108.5	99	137	13
S5, CBN, unif, wholeT_0.5	110	73	119	98
S1, DiP, last, singleC	111	67	56	82
J5, CBN-A, unif, singleC	112	118	135	55.5
J1, CBN-A, last, singleC	113	105	84	123
J5, CBN-A, last, wholeT_0.01	114	95	68	124
J1, CBN-A, last, wholeT_0.5	115	111	86	122
J5, CBN-A, unif, wholeT_0.01	116	113	126	91
J5, CBN-A, unif, wholeT_0.5	117	128	139	55.5
J5, CBN-A, last, wholeT_0.5	118	126	95	115
S5, CBN-A, last, wholeT_0.5	119	115	63	129
J1, CBN, last, singleC	120	103	105	120
J5, CBN-A, last, singleC	121	117	103	117
S1, DiP, unif, wholeT_0.01	122	82	26	114
J1, CBN, last, wholeT_0.5	123	104	104	121
S5, CBN-A, last, singleC	124	108	60	131
J5, CBN, last, wholeT_0.5	125	109	122	106
J5, CBN, last, wholeT_0.01	126	116	97	119
J5, CBN, unif, singleC	127	138	143	64.5
J5, CBN, last, singleC	128	131	129	116
S5, CBN, last, singleC	129	101	71	130
J1, CBN-A, last, wholeT_0.01	130	120	54	125
J5, CBN, unif, wholeT_0.5	131	119	144	64.5
J5, CBN, unif, wholeT_0.01	132	137	131	94
S5, CBN, last, wholeT_0.5	133	100	66	127
S5, CBN-A, last, wholeT_0.01	134	106	29	133
S1, CBN-A, last, wholeT_0.5	135	130	17	139
J1, CBN, last, wholeT_0.01	136	136	81	128
S1, CBN-A, unif, wholeT_0.01	137	132	**9**	143
S1, CBN-A, last, singleC	138	125	22	141
S5, CBN, last, wholeT_0.01	139	133	41	134
S1, CBN, last, wholeT_0.5	140	123	23	140
S1, CBN, last, singleC	141	129	24	142
S1, CBN, unif, wholeT_0.01	142	135	11	144
S1, CBN, last, wholeT_0.01	143	140	15	138
S1, CBN-A, last, wholeT_0.01	144	139	13	137

Table 13: Overall ranking of all 144 combinations of method, filtering, and sampling when Drivers are Unknown with respect to each performance measure. S.Size = 200. Only graphs with Conjunction. Methods have been ordered by their performance in the first performance measure. Best 10 methods are shown in bold.

Method and sampling	Diff	PFD	PND	FPF
S5, OT-A, last, singleC	**1**	11	15	51
S5, OT-A, last, wholeT_0.5	**2**	12	21	50
J5, OT-A, last, wholeT_0.01	**3**	**1**	24	71
J1, OT-A, last, wholeT_0.5	**4**	**6**	39	67
S5, OT-A, last, wholeT_0.01	**5**	**2**	**3**	98
S5, OT-A, unif, wholeT_0.01	**6**	17	33.5	68.5
J1, OT-A, last, singleC	**7**	14	38	75
S5, OT, unif, singleC	**8.5**	32	57.5	37.5
S5, OT-A, unif, singleC	**8.5**	47	57.5	37.5
S5, OT, unif, wholeT_0.01	**10**	13	33.5	68.5
S5, OT, unif, wholeT_0.5	11.5	28	68.5	33.5
S5, OT-A, unif, wholeT_0.5	11.5	49	68.5	33.5
J1, OT-A, last, wholeT_0.01	13	18	**9**	106
S1, OT-A, unif, wholeT_0.5	14	58	19	108
S1, OT, unif, wholeT_0.5	15	43	18	107
J1, OT, unif, singleC	16.5	23	59.5	29.5
J1, OT-A, unif, singleC	16.5	38	59.5	29.5
S1, OT-A, last, wholeT_0.5	18	48	**2**	124
J1, OT, unif, wholeT_0.5	19.5	16	65.5	31.5
J1, OT-A, unif, wholeT_0.5	19.5	30	65.5	31.5
J1, OT-A, unif, wholeT_0.01	21	**5**	35.5	88.5
J1, OT, unif, wholeT_0.01	22	**4**	35.5	88.5
J5, OT-A, last, singleC	23	25	53	42
J5, OT-A, last, wholeT_0.5	24	34	52	42
S5, OT, last, singleC	25	19	51	**6**
S1, OT, unif, singleC	26.5	51	12.5	112
S1, OT-A, unif, singleC	26.5	64	12.5	113
S5, OT, last, wholeT_0.5	28	**7**	54	**6**
S1, OT-A, last, singleC	29	44	**4**	123
S5, CBN-A, unif, wholeT_0.01	30	**3**	37	91
S1, OT-A, last, wholeT_0.01	31	39	**1**	136
J5, OT-A, unif, wholeT_0.01	32	62	85.5	44.5
J5, OT, last, wholeT_0.01	33	**10**	56	25
J5, OT, unif, wholeT_0.01	34	59	85.5	44.5
S5, CBN-A, unif, singleC	35	21	64	77
J1, CBN-A, unif, wholeT_0.5	36	35	70	66
S5, CBN-A, unif, wholeT_0.5	37	37	71	80
J1, CBN-A, unif, singleC	38	29	63	73
J1, OT, last, wholeT_0.5	39	**8**	81	39
S5, OT, last, wholeT_0.01	40	**9**	26	84
J1, OT, last, singleC	41	26	83	48
J5, DiP-A, unif, wholeT_0.01	42	115	119	40
J5, OT, last, singleC	43	33	91.5	**6**
S5, CBN, unif, wholeT_0.01	44	15	49	100
J5, DiP-A, unif, singleC	45	117	126	15
S5, DiP-A, unif, wholeT_0.5	46	134	121	18
J1, OT, last, wholeT_0.01	47	27	46	94
S5, CBN, unif, singleC	48	53	75	90
J5, OT, last, wholeT_0.5	49	20	91.5	**6**
J5, DiP, unif, wholeT_0.01	50	131	135	36

Table 13: *(continued)*

Method and sampling	Diff	PFD	PND	FPF
S5, DiP-A, unif, singleC	51	114	120	19.5
J1, CBN, unif, wholeT_0.5	52	24	76.5	78
J1, CBN-A, unif, wholeT_0.01	53	22	43	97
J5, DiP-A, unif, wholeT_0.5	54	136	125	15
S5, CBN, unif, wholeT_0.5	55	36	78	87
S5, DiP-A, unif, wholeT_0.01	56	101	90	64
J5, OT, unif, singleC	57.5	60	100.5	21.5
J5, OT-A, unif, singleC	57.5	77	100.5	21.5
J1, CBN, unif, singleC	59	40	76.5	79
S5, DiP, unif, wholeT_0.5	60	144	131.5	15
J5, OT, unif, wholeT_0.5	61.5	54	100.5	23.5
J5, OT-A, unif, wholeT_0.5	61.5	80	100.5	23.5
J5, DiP-A, last, singleC	63	104	110	42
J5, DiP, unif, singleC	64	129	139	12
S5, DiP, unif, singleC	65	125	131.5	19.5
J5, DiP, unif, wholeT_0.5	66	141	140	**6**
J1, DiP-A, unif, wholeT_0.5	67	133	124	27
J1, DiP-A, unif, singleC	68	111	123	27
S1, OT, last, wholeT_0.5	69	50	29	122
S5, DiP, unif, wholeT_0.01	70	118	106	65
J5, DiP-A, last, wholeT_0.5	71	127	117	49
J1, CBN, unif, wholeT_0.01	72	31	47	105
J1, DiP, unif, wholeT_0.5	73	143	136	17
S1, OT, last, singleC	74	52	31	117
J1, DiP, unif, singleC	75	124	138	27
S1, OT, last, wholeT_0.01	76	45	20	131
J5, CBN-A, unif, wholeT_0.5	77	93	104	52
J5, CBN-A, unif, wholeT_0.01	78	70	89	82
J5, CBN-A, unif, singleC	79	87	103	58
J1, DiP-A, unif, wholeT_0.01	80	102	93	83
J1, DiP-A, last, singleC	81	105	109	46
J1, DiP-A, last, wholeT_0.5	82	128	111	56
J5, CBN-A, last, wholeT_0.01	83	46	50	110
J5, DiP, last, singleC	84	119	143	**6**
J5, CBN, unif, wholeT_0.5	85	75	114	59
J5, CBN, unif, wholeT_0.01	86	88	96	86
J5, CBN, unif, singleC	87	91	115	60
J5, DiP-A, last, wholeT_0.01	88	107	94	76
J5, DiP, last, wholeT_0.5	89	137	144	**6**
J5, CBN-A, last, singleC	90	74	74	103
J5, CBN, last, wholeT_0.01	91	72	62	109
J1, DiP, unif, wholeT_0.01	92	116	108	81
J1, CBN-A, last, wholeT_0.5	93	73	55	115
J1, CBN-A, last, singleC	94	78	61	116
J5, CBN-A, last, wholeT_0.5	95	96	72	104
J5, CBN, last, wholeT_0.5	96	68	87	102
S5, DiP-A, last, singleC	97	99	98	47
J1, CBN, last, singleC	98	67	73	114
J5, CBN, last, singleC	99	69	84	101
S1, OT-A, unif, wholeT_0.01	100	66	**6**	135

Table 13: *(continued)*

Method and sampling	Diff	PFD	PND	FPF
S5, DiP-A, last, wholeT_0.5	101	126	107	57
S5, CBN, last, singleC	102	41	42	119
S1, OT, unif, wholeT_0.01	103	61	**7**	134
S5, CBN, last, wholeT_0.5	104	42	44	118
S5, CBN-A, last, singleC	105	57	41	121
J1, CBN, last, wholeT_0.5	106	55	67	111
J1, DiP, last, wholeT_0.5	107	138	141.5	**6**
S5, CBN-A, last, wholeT_0.5	108	63	45	120
J1, DiP, last, singleC	109	120	141.5	**6**
J5, DiP, last, wholeT_0.01	110	130	130	13
S1, CBN-A, unif, wholeT_0.5	111	76	28	126
S1, CBN, unif, wholeT_0.5	112	71	32	130
S5, DiP, last, wholeT_0.5	113	139	137	**6**
S1, CBN-A, unif, singleC	114	79	23	133
S5, DiP, last, singleC	115	121	133	**6**
J1, DiP-A, last, wholeT_0.01	116	108	88	93
S1, CBN, unif, singleC	117	82	30	132
S1, DiP-A, unif, wholeT_0.5	118	135	118	61
S5, DiP-A, last, wholeT_0.01	119	100	80	95
J1, CBN-A, last, wholeT_0.01	120	83	40	125
S1, DiP, unif, wholeT_0.5	121	142	127.5	53
S5, CBN-A, last, wholeT_0.01	122	56	16	127
J1, CBN, last, wholeT_0.01	123	86	48	128
J1, DiP, last, wholeT_0.01	124	122	122	63
S1, DiP-A, unif, singleC	125	110	116	62
S5, CBN, last, wholeT_0.01	126	65	17	129
S1, DiP, unif, singleC	127	123	127.5	54
S5, DiP, last, wholeT_0.01	128	109	112.5	70
S1, CBN-A, unif, wholeT_0.01	129	81	**5**	142
S1, CBN-A, last, wholeT_0.01	130	92	**10**	137
S1, DiP-A, last, wholeT_0.01	131	98	79	99
S1, CBN-A, last, wholeT_0.5	132	94	27	140
S1, DiP-A, unif, wholeT_0.01	133	97	82	96
S1, CBN-A, last, singleC	134	90	25	141
S1, DiP-A, last, wholeT_0.5	135	132	105	72
S1, DiP-A, last, singleC	136	103	97	85
S1, CBN, last, wholeT_0.5	137	89	11	138
S1, DiP, unif, wholeT_0.01	138	106	95	92
S1, CBN, last, singleC	139	95	22	144
S1, CBN, unif, wholeT_0.01	140	84	**8**	143
S1, CBN, last, wholeT_0.01	141	85	14	139
S1, DiP, last, singleC	142	112	129	55
S1, DiP, last, wholeT_0.5	143	140	134	35
S1, DiP, last, wholeT_0.01	144	113	112.5	74

Table 14: Overall ranking of all 144 combinations of method, filtering, and sampling when Drivers are Unknown with respect to each performance measure. S.Size = 100. Only graphs with Conjunction. Methods have been ordered by their performance in the first performance measure. Best 10 methods are shown in bold.

Method and sampling	Diff	PFD	PND	FPF
S5, OT-A, last, singleC	**1**	**3**	20	60
S5, OT-A, last, wholeT_0.5	**2**	**4**	22	61
J5, OT-A, last, wholeT_0.01	**3**	**1**	28	69
S5, OT, unif, singleC	**4**	57	61.5	40
S5, OT-A, unif, singleC	**5**	63	61.5	49
S5, OT-A, last, wholeT_0.01	**6**	**2**	**4**	99
S5, OT-A, unif, wholeT_0.5	**7**	54	68	43.5
S5, OT-A, unif, wholeT_0.01	**8**	13	33	71
S5, OT, unif, wholeT_0.5	**9**	37	69	43.5
J1, OT-A, last, singleC	**10**	17	40	92
S5, OT, unif, wholeT_0.01	11	**10**	35	70
J1, OT-A, last, wholeT_0.5	12	15	41	100
J1, OT, unif, singleC	13	14	58	62
J1, OT-A, unif, singleC	14	22	57	68
J5, OT-A, last, singleC	15	21	52	51.5
J5, OT-A, last, wholeT_0.5	16	16	54	51.5
J1, OT-A, unif, wholeT_0.5	17	28	64.5	66
J1, OT, unif, wholeT_0.5	18	11	64.5	64
S5, OT, last, wholeT_0.5	19	**5**	53	37
S5, OT, last, singleC	20	**8**	51	42
J5, OT-A, unif, wholeT_0.01	21	58	81	45.5
S5, CBN-A, unif, singleC	22	24	67	74
J1, OT-A, last, wholeT_0.01	23	25	**9**	121
S5, CBN-A, unif, wholeT_0.01	24	**7**	38	90
J1, CBN-A, unif, wholeT_0.5	25	19	59	78
J5, OT, last, wholeT_0.01	26	**9**	63	39
J5, OT, unif, wholeT_0.01	27	61	82	45.5
J1, CBN-A, unif, singleC	28	20	66	77
S1, OT-A, last, singleC	29	33	**3**	125
S1, OT-A, last, wholeT_0.5	30	29	**2**	123
S5, CBN-A, unif, wholeT_0.5	31	36	71	73
J5, OT, last, singleC	32	39	86	**7**
J1, CBN, unif, singleC	33	46	70	81
S1, OT-A, last, wholeT_0.01	34	18	**1**	130
S5, OT, last, wholeT_0.01	35	**6**	30	80
J5, DiP-A, unif, wholeT_0.01	36	126	107	48
J5, OT, last, wholeT_0.5	37	23	88	**7**
J1, OT-A, unif, wholeT_0.01	38	53	34	107
J1, OT, last, singleC	39	52	78	86
S5, CBN, unif, singleC	40	42	75	85
S5, CBN, unif, wholeT_0.01	41	12	47	95
J1, OT, last, wholeT_0.5	42	35	79	88
J5, OT, unif, singleC	43.5	86	91.5	22.5
J5, OT-A, unif, singleC	43.5	90	91.5	22.5
J5, DiP, unif, wholeT_0.01	45	128	119	41
J1, OT, unif, wholeT_0.01	46	48	36	103
S5, CBN, unif, wholeT_0.5	47	41	80	83
S1, OT-A, unif, wholeT_0.5	48	44	14	114
J5, DiP-A, unif, singleC	49.5	111	128	**7**
J5, DiP, unif, singleC	49.5	117	128	**7**

Table 14: *(continued)*

Method and sampling	Diff	PFD	PND	FPF
J5, OT-A, unif, wholeT_0.5	51	85	89.5	28.5
S1, OT, unif, wholeT_0.5	52	32	15	113
J1, CBN, unif, wholeT_0.5	53	26	73	82
J5, OT, unif, wholeT_0.5	54	67	89.5	28.5
S1, OT, unif, singleC	55	30	12	116
J5, DiP-A, unif, wholeT_0.5	56.5	137	139.5	**7**
J5, DiP, unif, wholeT_0.5	56.5	141	139.5	**7**
S1, OT-A, unif, singleC	58	40	11	118
S5, DiP-A, unif, wholeT_0.5	59.5	139	128	32
S5, DiP, unif, wholeT_0.5	59.5	143	128	26
S5, DiP-A, unif, singleC	61	112	128	24
S5, DiP, unif, singleC	62	118	128	19.5
J5, CBN-A, unif, wholeT_0.01	63	65	83	75
J5, CBN-A, unif, wholeT_0.5	64	92	93	47
J5, CBN-A, unif, singleC	65	93	94	54
J5, CBN, unif, wholeT_0.01	66	88	87	79
J5, DiP, last, wholeT_0.5	67	133	139.5	**7**
J5, CBN, unif, wholeT_0.5	68	95	95	56
J1, OT, last, wholeT_0.01	69	43	43	115
J5, CBN, unif, singleC	70	96	96	55
J5, DiP-A, last, wholeT_0.5	71	131	128	19.5
J5, DiP, last, singleC	72	102	139.5	**7**
J5, CBN-A, last, wholeT_0.5	73	94	74	97
J5, CBN-A, last, wholeT_0.01	74	51	50	102
S5, DiP-A, unif, wholeT_0.01	75	113	99	67
J5, DiP-A, last, singleC	76	97	139.5	19.5
J5, CBN-A, last, singleC	77	84	76	96
J5, CBN, last, wholeT_0.01	78	59	55	101
J5, CBN, last, singleC	79	77	84	98
J1, CBN-A, unif, wholeT_0.01	80	56	37	119
S1, OT, last, singleC	81	49	31	120
S1, OT, last, wholeT_0.5	82	38	32	117
J1, DiP-A, unif, wholeT_0.5	83.5	138	139.5	25
J1, DiP, unif, wholeT_0.5	83.5	142	139.5	14
J5, CBN, last, wholeT_0.5	85	72	85	93
S5, DiP, unif, wholeT_0.01	86	123	106	59
J1, DiP-A, unif, singleC	87.5	114	128	19.5
J1, DiP, unif, singleC	87.5	119	128	16
S1, OT, last, wholeT_0.01	89	27	27	127
J1, CBN-A, last, singleC	90	64	56	109
J1, CBN, unif, wholeT_0.01	91	74	42	122
J1, CBN, last, singleC	92	66	60	110
S5, CBN, last, wholeT_0.5	93	31	46	108
J1, CBN-A, last, wholeT_0.5	94	69	72	105
S5, CBN-A, last, wholeT_0.5	95	60	49	106
S5, CBN, last, singleC	96	34	44	111
J5, DiP, last, wholeT_0.01	97	127	115	**7**
S5, CBN-A, last, singleC	98	47	48	112
S1, OT-A, unif, wholeT_0.01	99	50	**6**	134
J5, DiP-A, last, wholeT_0.01	100	121	109	65

Table 14: *(continued)*

Method and sampling	Diff	PFD	PND	FPF
J1, CBN, last, wholeT_0.5	101	68	77	104
J1, DiP, last, singleC	102	103	139.5	**7**
J1, DiP-A, last, singleC	103	99	139.5	30.5
J1, DiP, last, wholeT_0.5	104	134	139.5	**7**
J1, DiP-A, last, wholeT_0.5	105	132	128	34
S1, OT, unif, wholeT_0.01	106	45	**8**	133
S5, DiP, last, wholeT_0.5	107	135	121	16
S5, DiP, last, singleC	108	106	113.5	**7**
S5, DiP-A, last, wholeT_0.5	109	129	117	27
S5, DiP-A, last, singleC	110	98	109	30.5
J1, DiP-A, unif, wholeT_0.01	111	110	104	76
J1, DiP, unif, wholeT_0.01	112	116	112	72
S5, CBN-A, last, wholeT_0.01	113	62	21	126
J1, CBN-A, last, wholeT_0.01	114	82	45	128
S1, CBN-A, unif, singleC	115	73	17	135
S1, CBN-A, unif, wholeT_0.5	116	75	25	131
S1, CBN, unif, wholeT_0.5	117	71	29	132
J1, CBN, last, wholeT_0.01	118	80	39	129
S5, CBN, last, wholeT_0.01	119	55	24	124
S5, DiP-A, last, wholeT_0.01	120	105	100	84
S5, DiP, last, wholeT_0.01	121	124	101.5	57
S1, CBN, unif, singleC	122	78	16	136
J1, DiP, last, wholeT_0.01	123	122	111	63
J1, DiP-A, last, wholeT_0.01	124	108	105	89
S1, DiP-A, unif, wholeT_0.5	125	140	128	53
S1, DiP, unif, wholeT_0.5	126	144	128	38
S1, DiP-A, unif, singleC	127	115	120	36
S1, DiP, unif, singleC	128	120	128	35
S1, CBN-A, unif, wholeT_0.01	129	70	**5**	142
S1, CBN-A, last, singleC	130	83	23	139
S1, CBN-A, last, wholeT_0.5	131	91	26	141
S1, CBN, last, wholeT_0.5	132	89	18	138
S1, CBN-A, last, wholeT_0.01	133	79	**10**	137
S1, CBN, last, singleC	134	87	13	144
S1, CBN, unif, wholeT_0.01	135	76	**7**	143
S1, DiP-A, unif, wholeT_0.01	136	101	97	94
S1, DiP, last, wholeT_0.5	137	136	117	16
S1, DiP, unif, wholeT_0.01	138	104	103	91
S1, DiP, last, singleC	139	109	113.5	**7**
S1, DiP-A, last, singleC	140	100	109	33
S1, DiP, last, wholeT_0.01	141	125	101.5	58
S1, DiP-A, last, wholeT_0.5	142	130	117	50
S1, DiP-A, last, wholeT_0.01	143	107	98	87
S1, CBN, last, wholeT_0.01	144	81	19	140

2.2.2 Overall ranking: Drivers Unknown, no conjunction

Table 15: Overall ranking of all 144 combinations of method, filtering, and sampling when Drivers are Unknown with respect to each performance measure. Only graphs without Conjunction. Methods have been ordered by their performance in the first performance measure. Best 10 methods are shown in bold.

Method and sampling	Diff	PFD	PND	FPF
S5, OT-A, last, singleC	**1**	13	13	60
S5, OT-A, last, wholeT_0.5	**2**	**7**	23	59
S5, OT-A, last, wholeT_0.01	**3**	**5**	**4**	94
J5, OT-A, last, wholeT_0.01	**4**	**2**	29	74
S1, OT-A, last, wholeT_0.5	**5**	27	**3**	116
J1, OT-A, last, singleC	**6**	12	37	75
J1, OT-A, last, wholeT_0.5	**7**	**6**	39	71
S5, OT-A, unif, singleC	**8**	49	55.5	20.5
S5, OT, unif, singleC	**9**	38	55.5	20.5
S5, OT-A, unif, wholeT_0.01	**10**	17	32.5	68.5
S5, OT, unif, wholeT_0.01	11	**10**	32.5	68.5
S1, OT-A, last, singleC	12	32	**2**	120
S5, OT, unif, wholeT_0.5	13	30	60.5	27.5
S5, OT-A, unif, wholeT_0.5	14	45	60.5	27.5
J1, OT-A, last, wholeT_0.01	15	19	**8**	108
S1, OT, unif, wholeT_0.5	16.5	22	20.5	101
S1, OT-A, unif, wholeT_0.5	16.5	37	20.5	102
S1, OT-A, unif, singleC	18	36	10.5	106
S1, OT, unif, singleC	19	24	10.5	105
S5, OT, last, singleC	20	**8**	42	19
J1, OT-A, unif, singleC	21	31	65	34.5
J1, OT, unif, singleC	22	16	67	34.5
S5, OT, last, wholeT_0.5	23	**3**	49	17
S1, OT-A, last, wholeT_0.01	24	50	**1**	134
J5, OT-A, last, singleC	25	41	59	47
J1, OT-A, unif, wholeT_0.5	26	28	69.5	45.5
J1, OT, unif, wholeT_0.5	27	11	69.5	45.5
J1, OT-A, unif, wholeT_0.01	28	18	35	92
J5, OT-A, last, wholeT_0.5	29	33	63	44
J1, OT, unif, wholeT_0.01	30	14	36	91
S5, DiP-A, unif, wholeT_0.5	31	137	119	31
S5, DiP-A, unif, singleC	32	117	118	26
J5, OT-A, unif, wholeT_0.01	33	67	94	40.5
S5, OT, last, wholeT_0.01	34	**1**	24	82
J5, OT, unif, wholeT_0.01	35	53	95	40.5
J5, OT, last, wholeT_0.01	36	**4**	62	50
J1, OT, last, singleC	37	25	82	53
J1, OT, last, wholeT_0.5	38	**9**	83	43
S5, DiP, unif, wholeT_0.5	39	141	127	25
S5, CBN-A, unif, wholeT_0.01	40	20	40	103
S5, DiP, unif, singleC	41	125	128	13
S5, DiP-A, unif, wholeT_0.01	42	112	86	70
S1, OT, last, wholeT_0.5	43	21	25	109
S1, OT, last, singleC	44	35	16	112
S5, CBN-A, unif, singleC	45	47	72	84
J5, DiP-A, unif, wholeT_0.01	46	133	135	33
J1, CBN-A, unif, singleC	47	40	79	76
J1, CBN-A, unif, wholeT_0.5	48	39	78	64
S5, CBN-A, unif, wholeT_0.5	49	46	75	85
J1, OT, last, wholeT_0.01	50	15	38	96

Table 15: *(continued)*

Method and sampling	Diff	PFD	PND	FPF
J5, DiP-A, unif, singleC	51	128	138	**9**
J5, DiP, unif, wholeT_0.01	52	138	142	32
S5, DiP, unif, wholeT_0.01	53	120	96	72
J5, DiP-A, unif, wholeT_0.5	54	143	141	11.5
J5, DiP-A, last, wholeT_0.01	55	105	89	73
J5, DiP-A, last, singleC	56	110	124	37
J1, DiP-A, unif, singleC	57	118	129	29
J5, OT, last, singleC	58	43	109	**4.5**
S5, DiP-A, last, wholeT_0.5	59	124	87	48
J5, DiP, unif, singleC	60	132	144	**4.5**
J5, OT-A, unif, singleC	61	79	116	14.5
J5, DiP-A, last, wholeT_0.5	62	129	126	36
J5, OT, unif, singleC	63	71	117	14.5
J5, DiP, unif, wholeT_0.5	64	144	143	**4.5**
J1, DiP-A, unif, wholeT_0.5	65	139	130	24
S5, CBN, unif, wholeT_0.01	66	26	46	107
J1, CBN, unif, singleC	67	44	91	81
S5, CBN, unif, singleC	68	51	84	88
S5, DiP-A, last, singleC	69	100	80	52
S1, OT-A, unif, wholeT_0.01	70	59	**5**	136
J1, CBN-A, unif, wholeT_0.01	71	29	44	110
J1, CBN, unif, wholeT_0.5	72	34	93	77
J1, DiP-A, last, wholeT_0.5	73	126	108	38
S1, OT, unif, wholeT_0.01	74	55	**6**	135
J5, OT, last, wholeT_0.5	75	23	112	**4.5**
J5, OT-A, unif, wholeT_0.5	76	75	120	22.5
J5, OT, unif, wholeT_0.5	77	54	121	22.5
J1, DiP-A, last, singleC	78	104	107	39
J1, DiP, unif, singleC	79	127	137	18
S5, CBN, unif, wholeT_0.5	80	48	88	90
J5, DiP, last, singleC	81	119	140	**4.5**
J1, DiP, unif, wholeT_0.5	82	142	136	16
J5, DiP, last, wholeT_0.5	83	136	139	**4.5**
J1, DiP-A, unif, wholeT_0.01	84	107	97	80
S1, OT, last, wholeT_0.01	85	52	14	132
J5, CBN-A, last, wholeT_0.01	86	56	51	111
S5, DiP, last, wholeT_0.5	87	130	110	**10**
J5, CBN-A, unif, wholeT_0.01	88	72	102	83
J1, DiP, last, wholeT_0.5	89	134	134	**4.5**
J5, CBN-A, unif, singleC	90	84	123	56
S5, DiP, last, singleC	91	106	106	11.5
J5, CBN-A, unif, wholeT_0.5	92	82	122	54
J1, DiP, last, singleC	93	115	132	**4.5**
S5, DiP-A, last, wholeT_0.01	94	89	53	87
J1, DiP-A, last, wholeT_0.01	95	97	64	89
J5, DiP, last, wholeT_0.01	96	121	125	30
J1, CBN, unif, wholeT_0.01	97	42	52	114
J1, DiP, unif, wholeT_0.01	98	113	114	79
J5, CBN, unif, wholeT_0.01	99	87	113	86
J5, CBN, unif, singleC	100	103	131	63

Table 15: *(continued)*

Method and sampling	Diff	PFD	PND	FPF
J5, CBN-A, last, wholeT_0.5	101	93	90	98
J5, CBN-A, last, singleC	102	83	92	100
J5, CBN, last, singleC	103	77	103	97
J5, CBN, last, wholeT_0.5	104	78	105	95
J5, CBN, unif, wholeT_0.5	105	86	133	62
J1, CBN-A, last, singleC	106	69	66	119
J5, CBN, last, wholeT_0.01	107	61	57	113
S1, CBN-A, unif, wholeT_0.5	108	66	31	126
J1, CBN-A, last, wholeT_0.5	109	74	73	117
S1, DiP-A, unif, wholeT_0.5	110	135	101	58
J1, CBN, last, singleC	111	65	68	118
S1, CBN-A, unif, singleC	112	64	27	130
J1, CBN, last, wholeT_0.5	113	68	74	115
S1, DiP, unif, wholeT_0.5	114	140	115	51
S1, DiP-A, unif, singleC	115	116	98	57
S1, CBN, unif, singleC	116	60	30	129
S1, CBN, unif, wholeT_0.5	117	58	34	127
J1, DiP, last, wholeT_0.01	118	114	104	67
S5, CBN, last, singleC	119	63	45	123
S5, DiP, last, wholeT_0.01	120	109	85	65
S5, CBN, last, wholeT_0.5	121	57	47	122
S5, CBN-A, last, singleC	122	62	48	124
S1, DiP, unif, singleC	123	122	111	55
S5, CBN-A, last, wholeT_0.5	124	70	54	121
J1, CBN-A, last, wholeT_0.01	125	76	43	125
S5, CBN-A, last, wholeT_0.01	126	73	26	131
S1, DiP-A, last, wholeT_0.01	127	91	50	93
S1, DiP-A, last, wholeT_0.5	128	123	76	61
J1, CBN, last, wholeT_0.01	129	80	41	128
S1, DiP-A, last, singleC	130	101	71	66
S1, DiP-A, unif, wholeT_0.01	131	98	58	104
S5, CBN, last, wholeT_0.01	132	85	28	133
S1, DiP, last, wholeT_0.5	133	131	100	42
S1, DiP, last, wholeT_0.01	134	108	81	78
S1, DiP, unif, wholeT_0.01	135	111	77	99
S1, DiP, last, singleC	136	102	99	49
S1, CBN-A, last, wholeT_0.5	137	95	22	138
S1, CBN-A, last, singleC	138	88	17	141
S1, CBN-A, last, wholeT_0.01	139	94	12	137
S1, CBN-A, unif, wholeT_0.01	140	81	**7**	143
S1, CBN, last, wholeT_0.5	141	92	19	139
S1, CBN, last, singleC	142	96	15	142
S1, CBN, unif, wholeT_0.01	143	90	**9**	144
S1, CBN, last, wholeT_0.01	144	99	18	140

Table 16: Overall ranking of all 144 combinations of method, filtering, and sampling when Drivers are Unknown with respect to each performance measure. S.Size = 1000. Only graphs without Conjunction. Methods have been ordered by their performance in the first performance measure. Best 10 methods are shown in bold.

Method and sampling	Diff	PFD	PND	FPF
S1, OT, unif, singleC	**1.5**	**4**	22.5	60.5
S1, OT-A, unif, singleC	**1.5**	11	22.5	60.5
S5, OT-A, last, singleC	**3**	27	17	63.5
S5, OT-A, last, wholeT_0.5	**4**	22	24	63.5
S1, OT, unif, wholeT_0.5	**5.5**	**5**	29.5	56.5
S1, OT-A, unif, wholeT_0.5	**5.5**	19	29.5	56.5
S1, OT-A, last, wholeT_0.5	**7**	25	**3**	101
S5, OT-A, last, wholeT_0.01	**8**	20	**5**	100
S5, DiP-A, last, wholeT_0.01	**9**	31	**8**	96
S5, DiP-A, last, singleC	**10**	57	28	63.5
S5, DiP-A, last, wholeT_0.5	11	74	35	63.5
S1, OT-A, last, singleC	12	44	**2**	106
J1, OT-A, last, wholeT_0.01	13	40	**10**	105
J1, DiP-A, last, wholeT_0.01	14	34	14	91
S1, DiP-A, unif, singleC	15	73	48	44
J5, DiP-A, last, wholeT_0.01	16	16	42	66
S5, DiP-A, unif, singleC	17	86	81	16.5
S5, DiP-A, unif, wholeT_0.5	18	119	84	16.5
J5, OT-A, last, wholeT_0.01	19	**6**	38	78
J1, OT-A, last, singleC	20	17	52	52.5
S5, OT, unif, wholeT_0.01	21.5	15	43.5	68.5
S5, OT-A, unif, wholeT_0.01	21.5	28	43.5	68.5
J1, OT-A, last, wholeT_0.5	23	12	53	52.5
S1, DiP-A, unif, wholeT_0.5	24	91	57	42.5
S5, OT, unif, singleC	25.5	47	79.5	16.5
S5, OT-A, unif, singleC	25.5	66	79.5	16.5
S5, DiP-A, unif, wholeT_0.01	27	70	51	71
S5, OT, unif, wholeT_0.5	28.5	33	82.5	16.5
S5, OT-A, unif, wholeT_0.5	28.5	58	82.5	16.5
S5, DiP, unif, wholeT_0.5	30	125	102	16.5
S5, DiP, unif, singleC	31	94	96	16.5
S5, DiP, last, singleC	32	35	60	16.5
S1, DiP, unif, wholeT_0.5	33	108	73	45
S5, OT, last, singleC	34	**9**	56	16.5
S5, DiP, last, wholeT_0.5	35	55	64	16.5
J1, DiP-A, last, singleC	36	50	70.5	52.5
J1, DiP-A, last, wholeT_0.5	37	68	70.5	52.5
S5, DiP, unif, wholeT_0.01	38	76	65	77
S1, DiP, unif, singleC	39	87	66	42.5
S1, OT-A, last, wholeT_0.01	40	92	**1**	133
J1, OT, unif, wholeT_0.01	41.5	**3**	54.5	81.5
J1, OT-A, unif, wholeT_0.01	41.5	**7**	54.5	81.5
S5, OT, last, wholeT_0.5	43	**2**	62	16.5
S1, DiP-A, last, wholeT_0.01	44	30	**4**	103
J1, DiP-A, unif, singleC	45	84	107	34.5
J1, DiP-A, unif, wholeT_0.5	46	118	111	16.5
S1, DiP-A, last, wholeT_0.5	47	79	15	86
J5, DiP-A, last, singleC	48	85	92	48.5
S5, OT, last, wholeT_0.01	49	**1**	31	80
S1, OT, last, singleC	50	45	16	93

Table 16: *(continued)*

Method and sampling	Diff	PFD	PND	FPF
S1, OT, last, wholeT_0.5	51	**10**	32	85
J1, OT, unif, singleC	52.5	21	98.5	34.5
J1, OT-A, unif, singleC	52.5	39	98.5	34.5
S5, DiP, last, wholeT_0.01	54	32	39	76
J1, DiP-A, unif, wholeT_0.01	55	38	74	83
J5, OT-A, last, singleC	56	61	86	48.5
J5, DiP-A, last, wholeT_0.5	57	100	95	48.5
J1, DiP, unif, singleC	58	93	119	34.5
S1, DiP-A, last, singleC	59	54	12	88
J1, DiP, last, singleC	60	62	108.5	16.5
J1, DiP, last, wholeT_0.01	61	41	61	74
J1, OT, unif, wholeT_0.5	62.5	18	103.5	16.5
J1, OT-A, unif, wholeT_0.5	62.5	36	103.5	16.5
J1, DiP, last, wholeT_0.5	64	80	108.5	16.5
J5, OT-A, last, wholeT_0.5	65	53	87	48.5
J1, DiP, unif, wholeT_0.5	66	123	120	16.5
J1, OT, last, wholeT_0.01	67	14	50	89
S1, CBN-A, unif, singleC	68	60	37	117
J5, DiP, last, wholeT_0.01	69	48	91	37
J1, OT, last, singleC	70	26	105.5	16.5
J1, DiP, unif, wholeT_0.01	71	49	88	84
S1, CBN, unif, singleC	72	52	45	116
J5, DiP-A, unif, wholeT_0.01	73	129	123	39.5
S1, OT-A, unif, wholeT_0.01	74	107	**6.5**	136
J1, OT, last, wholeT_0.5	75	13	105.5	16.5
S1, CBN-A, unif, wholeT_0.5	76	63	41	115
S1, OT, unif, wholeT_0.01	77	103	**6.5**	135
J5, OT, last, wholeT_0.01	78	**8**	85	46
J5, OT, unif, wholeT_0.01	79.5	77	117.5	39.5
J5, OT-A, unif, wholeT_0.01	79.5	89	117.5	39.5
J5, DiP-A, unif, singleC	81	130	133	16.5
J5, DiP, last, singleC	82	88	129.5	16.5
J5, DiP, unif, wholeT_0.01	83	143	140	39.5
J5, DiP-A, unif, wholeT_0.5	84	142	137	16.5
J5, DiP, last, wholeT_0.5	85	114	129.5	16.5
S1, DiP, last, wholeT_0.5	86	72	47	75
S1, CBN, unif, wholeT_0.5	87	65	49	120
J1, CBN-A, unif, wholeT_0.01	88	29	69	102
S5, CBN-A, unif, wholeT_0.01	89	64	59	111
S5, CBN-A, unif, wholeT_0.5	90	67	101	94
J5, DiP, unif, singleC	91	141	141	16.5
J1, CBN-A, unif, wholeT_0.5	92	56	115	55
S5, CBN-A, unif, singleC	93	81	94	92
J5, DiP, unif, wholeT_0.5	94	144	142	16.5
J1, CBN-A, unif, singleC	95	71	113	79
S1, DiP, last, wholeT_0.01	96	24	33	90
S1, OT, last, wholeT_0.01	97	96	19	130
S5, CBN, unif, wholeT_0.01	98	43	67	110
S1, DiP-A, unif, wholeT_0.01	99	46	13	109
J5, OT, last, singleC	100	59	127.5	16.5

Table 16: *(continued)*

Method and sampling	Diff	PFD	PND	FPF
S1, DiP, last, singleC	101	23	46	70
S5, CBN, unif, wholeT_0.5	102	69	114	97
S5, CBN, unif, singleC	103	78	110	98
J5, OT, unif, singleC	104.5	90	133	16.5
J5, OT-A, unif, singleC	104.5	109	133	16.5
J5, OT, last, wholeT_0.5	106	37	127.5	16.5
J1, CBN, unif, wholeT_0.5	107	51	121	67
J5, OT, unif, wholeT_0.5	108.5	82	137	16.5
J5, OT-A, unif, wholeT_0.5	108.5	97	137	16.5
J1, CBN, unif, wholeT_0.01	110	42	77	114
J5, CBN-A, last, wholeT_0.01	111	99	75	119
J1, CBN, unif, singleC	112	83	122	87
S1, DiP, unif, wholeT_0.01	113	75	34	113
J5, CBN-A, unif, singleC	114	111	135	58.5
J5, CBN-A, unif, wholeT_0.5	115	116	139	58.5
J5, CBN-A, unif, wholeT_0.01	116	117	125	95
J1, CBN-A, last, wholeT_0.01	117	105	58	125
J5, CBN-A, last, wholeT_0.5	118	134	116	108
J5, CBN-A, last, singleC	119	124	112	112
J1, CBN-A, last, wholeT_0.5	120	121	90	123
J5, CBN, last, singleC	121	110	124	107
J1, CBN-A, last, singleC	122	106	93	124
J5, CBN, last, wholeT_0.5	123	122	126	104
J1, CBN, last, wholeT_0.5	124	95	100	121
J1, CBN, last, singleC	125	98	97	122
J5, CBN, unif, singleC	126	140	143	72.5
J5, CBN, unif, wholeT_0.01	127	139	131	99
J5, CBN, last, wholeT_0.01	128	102	89	118
J5, CBN, unif, wholeT_0.5	129	136	144	72.5
S5, CBN, last, singleC	130	101	63	128
S5, CBN, last, wholeT_0.5	131	104	72	129
S5, CBN-A, last, singleC	132	113	76	131
S5, CBN-A, last, wholeT_0.01	133	120	36	132
S5, CBN-A, last, wholeT_0.5	134	115	78	127
J1, CBN, last, wholeT_0.01	135	112	68	126
S1, CBN-A, last, wholeT_0.01	136	138	18	137
S1, CBN-A, unif, wholeT_0.01	137	127	**9**	143
S1, CBN-A, last, wholeT_0.5	138	131	27	140
S1, CBN-A, last, singleC	139	126	25	141
S5, CBN, last, wholeT_0.01	140	132	40	134
S1, CBN, last, singleC	141	135	21	138
S1, CBN, unif, wholeT_0.01	142	133	11	144
S1, CBN, last, wholeT_0.5	143	128	20	142
S1, CBN, last, wholeT_0.01	144	137	26	139

Table 17: Overall ranking of all 144 combinations of method, filtering, and sampling when Drivers are Unknown with respect to each performance measure. S.Size = 200. Only graphs without Conjunction. Methods have been ordered by their performance in the first performance measure. Best 10 methods are shown in bold.

Method and sampling	Diff	PFD	PND	FPF
S5, OT-A, last, singleC	**1**	16	17	55.5
S5, OT-A, last, wholeT_0.5	**2**	**10**	27	55.5
J5, OT-A, last, wholeT_0.01	**3**	**1**	29	72
J1, OT-A, last, singleC	**4**	13	38	62
J1, OT-A, last, wholeT_0.5	**5**	**6**	40	53
S5, OT-A, last, wholeT_0.01	**6**	**5**	**3**	95
J1, OT-A, last, wholeT_0.01	**7**	15	**8**	103
S5, OT, unif, singleC	**8.5**	33	57.5	16.5
S5, OT-A, unif, singleC	**8.5**	49	57.5	16.5
S5, OT, unif, wholeT_0.01	10.5	17	31.5	65.5
S5, OT-A, unif, wholeT_0.01	10.5	21	31.5	65.5
S5, OT, unif, wholeT_0.5	12.5	27	55.5	28.5
S5, OT-A, unif, wholeT_0.5	12.5	45	55.5	28.5
S1, OT-A, last, wholeT_0.5	14	36	**4**	121
S1, OT, unif, wholeT_0.5	15.5	40	25.5	108
S1, OT-A, unif, wholeT_0.5	15.5	58	25.5	109
S1, OT-A, last, singleC	17	39	**2**	124
S1, OT, unif, singleC	18	43	11.5	113.5
S1, OT-A, unif, singleC	19	55	11.5	113.5
J1, OT, unif, singleC	20.5	19	61.5	22.5
J1, OT-A, unif, singleC	20.5	26	61.5	22.5
J1, OT-A, unif, wholeT_0.01	22	11	35	84.5
J1, OT, unif, wholeT_0.5	23.5	20	69.5	32.5
J1, OT-A, unif, wholeT_0.5	23.5	31	69.5	32.5
S1, OT-A, last, wholeT_0.01	25	42	**1**	132
J5, OT-A, last, singleC	26	35	53	45
J1, OT, unif, wholeT_0.01	27	**7**	36	84.5
S5, OT, last, singleC	28	**9**	45	**7.5**
S5, OT, last, wholeT_0.5	29	**3**	50	**7.5**
J5, OT-A, last, wholeT_0.5	30	29	59	41.5
J5, OT-A, unif, wholeT_0.01	31	66	84.5	43.5
J5, OT, unif, wholeT_0.01	32	59	84.5	43.5
S5, CBN-A, unif, wholeT_0.01	33	18	39	99
J1, CBN-A, unif, singleC	34	30	72	63
J1, OT, last, wholeT_0.5	35	12	77	26
J5, OT, last, wholeT_0.01	36	**4**	63	47
J1, OT, last, singleC	37	22	75	34
J1, CBN-A, unif, wholeT_0.5	38	34	74	58
S5, OT, last, wholeT_0.01	39	**2**	21	83
S5, CBN-A, unif, singleC	40	41	68	86
J1, OT, last, wholeT_0.01	41	**8**	41	88
J5, DiP-A, unif, wholeT_0.01	42	131	128	37.5
J1, CBN, unif, singleC	43	32	78	71
S5, DiP-A, unif, singleC	44	111	116	35
S5, CBN-A, unif, wholeT_0.5	45	46	66	89
J1, CBN-A, unif, wholeT_0.01	46	14	43	102
S5, DiP-A, unif, wholeT_0.5	47	135	118	40
S1, OT, last, wholeT_0.5	48	38	22	117
J5, OT, last, singleC	49	37	94	**7.5**
J5, DiP-A, unif, singleC	50	124	134	**7.5**

Table 17: *(continued)*

Method and sampling	Diff	PFD	PND	FPF
J5, DiP, unif, wholeT_0.01	51	132	138	37.5
S1, OT, last, singleC	52	44	19	118
J5, OT, unif, singleC	53.5	64	102.5	**7.5**
J5, OT-A, unif, singleC	53.5	80	102.5	**7.5**
J1, CBN, unif, wholeT_0.5	55	28	86	73
S5, DiP-A, unif, wholeT_0.01	56	106	95	75.5
J5, OT, unif, wholeT_0.5	57.5	56	102.5	24.5
J5, OT-A, unif, wholeT_0.5	57.5	75	102.5	24.5
J5, DiP-A, unif, wholeT_0.5	59	137	135	16.5
S5, CBN, unif, wholeT_0.01	60	23	44	105
J5, DiP, unif, wholeT_0.5	61	143	139	**7.5**
J5, DiP, unif, singleC	62	128	144	**7.5**
J5, OT, last, wholeT_0.5	63	24	99	**7.5**
J1, DiP-A, unif, singleC	64	114	122	31
S5, CBN, unif, singleC	65	53	79	90
S5, DiP, unif, wholeT_0.5	66	142	132	30
S5, DiP, unif, singleC	67	125	133	20
S1, OT-A, unif, wholeT_0.01	68	54	**5**	134
S1, OT, unif, wholeT_0.01	69	50	**6**	133
S5, CBN, unif, wholeT_0.5	70	51	76	94
J1, DiP-A, unif, wholeT_0.5	71	140	130	27
J5, CBN-A, unif, wholeT_0.01	72	71	89	82
J5, DiP-A, last, singleC	73	103	121	46
J5, CBN-A, last, wholeT_0.01	74	48	51	107
J1, DiP, unif, singleC	75	130	136	20
S5, DiP, unif, wholeT_0.01	76	118	108	75.5
J5, DiP-A, last, wholeT_0.5	77	126	125	41.5
J5, CBN-A, unif, wholeT_0.5	78	85	106	50
S1, OT, last, wholeT_0.01	79	47	14	130
J1, DiP-A, unif, wholeT_0.01	80	109	100	81
J5, CBN-A, unif, singleC	81	88	109	57
J1, DiP, unif, wholeT_0.5	82	144	137	20
J1, CBN, unif, wholeT_0.01	83	25	49	106
J5, CBN, unif, wholeT_0.01	84	76	98	87
J5, DiP, last, singleC	85	120	143	**7.5**
J1, DiP-A, last, wholeT_0.5	86	127	112	49
J5, DiP, last, wholeT_0.5	87	138	142	**7.5**
J1, DiP-A, last, singleC	88	104	113	48
J5, CBN, last, wholeT_0.5	89	82	91	93
J5, CBN, unif, singleC	90	93	117	64
J5, CBN, unif, wholeT_0.5	91	72	115	61
J1, CBN-A, last, wholeT_0.5	92	62	64	112
J5, CBN-A, last, wholeT_0.5	93	91	82	100
S5, DiP-A, last, wholeT_0.5	94	119	96	54
J5, DiP-A, last, wholeT_0.01	95	110	97	79
J5, CBN-A, last, singleC	96	77	81	101
J5, CBN, last, wholeT_0.01	97	60	54	110
J1, DiP, unif, wholeT_0.01	98	117	111	80
J5, CBN, last, singleC	99	84	90	98
J1, CBN, last, singleC	100	61	65	116

Table 17: *(continued)*

Method and sampling	Diff	PFD	PND	FPF
J1, CBN-A, last, singleC	101	70	60	115
S5, DiP-A, last, singleC	102	101	88	60
J1, CBN, last, wholeT_0.5	103	63	67	111
J1, DiP, last, wholeT_0.5	104	139	141	**7.5**
S1, CBN-A, unif, wholeT_0.5	105	73	33	128
J1, DiP, last, singleC	106	122	140	**7.5**
J5, DiP, last, wholeT_0.01	107	129	131	39
S1, CBN, unif, wholeT_0.5	108	67	34	127
S5, CBN, last, singleC	109	65	46	122
S5, CBN, last, wholeT_0.5	110	52	47	120
S5, DiP, last, wholeT_0.5	111	134	126	**7.5**
J1, DiP-A, last, wholeT_0.01	112	100	83	91
S5, CBN-A, last, singleC	113	57	42	123
S5, DiP, last, singleC	114	108	124	16.5
S5, CBN-A, last, wholeT_0.5	115	68	52	119
S1, CBN, unif, singleC	116	74	30	136
S1, CBN-A, unif, singleC	117	79	28	135
J1, CBN-A, last, wholeT_0.01	118	81	48	125
S5, DiP-A, last, wholeT_0.01	119	97	73	92
S5, CBN-A, last, wholeT_0.01	120	69	15	129
S1, DiP-A, unif, wholeT_0.5	121	136	114	69
J1, CBN, last, wholeT_0.01	122	78	37	126
J1, DiP, last, wholeT_0.01	123	121	120	67
S1, DiP, unif, wholeT_0.5	124	141	127	51
S5, CBN, last, wholeT_0.01	125	87	18	131
S5, DiP, last, wholeT_0.01	126	112	107	70
S1, DiP-A, unif, singleC	127	116	110	77
S1, DiP, unif, singleC	128	123	129	68
S1, DiP-A, last, wholeT_0.01	129	98	71	96
S1, CBN-A, last, wholeT_0.5	130	95	20	137
S1, CBN-A, last, singleC	131	89	13	141
S1, DiP-A, unif, wholeT_0.01	132	99	80	104
S1, CBN-A, last, wholeT_0.01	133	92	**10**	138
S1, DiP-A, last, wholeT_0.5	134	115	92	59
S1, CBN-A, unif, wholeT_0.01	135	83	**7**	142
S1, CBN, last, wholeT_0.5	136	90	23	139
S1, DiP, unif, wholeT_0.01	137	105	93	97
S1, DiP-A, last, singleC	138	102	87	74
S1, CBN, unif, wholeT_0.01	139	86	**9**	143
S1, DiP, last, wholeT_0.01	140	113	105	78
S1, CBN, last, singleC	141	94	24	144
S1, DiP, last, wholeT_0.5	142	133	123	36
S1, CBN, last, wholeT_0.01	143	96	16	140
S1, DiP, last, singleC	144	107	119	52

Table 18: Overall ranking of all 144 combinations of method, filtering, and sampling when Drivers are Unknown with respect to each performance measure. S.Size = 100. Only graphs without Conjunction. Methods have been ordered by their performance in the first performance measure. Best 10 methods are shown in bold.

Method and sampling	Diff	PFD	PND	FPF
S5, OT-A, last, singleC	**1**	**8**	16	63
S5, OT-A, last, wholeT_0.5	**2**	**5**	28	59
J5, OT-A, last, wholeT_0.01	**3**	**1**	31	69
S5, OT-A, last, wholeT_0.01	**4**	**3**	**4**	98
S5, OT-A, unif, singleC	**5**	57	54.5	31.5
J1, OT-A, last, singleC	**6**	15	38	91
S5, OT, unif, singleC	**7**	44	54.5	31.5
S5, OT-A, unif, wholeT_0.01	**8**	12	33.5	70.5
S5, OT, unif, wholeT_0.5	**9**	27	65.5	45.5
J1, OT-A, last, wholeT_0.5	**10**	11	40	85
S5, OT-A, unif, wholeT_0.5	11	55	65.5	45.5
S5, OT, unif, wholeT_0.01	12	**10**	33.5	70.5
S1, OT-A, last, wholeT_0.5	13	19	**3**	123
S1, OT-A, last, singleC	14	13	**2**	124
J1, OT-A, unif, singleC	15	30	59	57.5
J1, OT, unif, singleC	16	23	61	57.5
J1, OT-A, unif, wholeT_0.5	17	17	56.5	67.5
J1, OT, unif, wholeT_0.5	18	**9**	56.5	67.5
S5, OT, last, singleC	19	16	44	38
J5, OT-A, last, wholeT_0.5	20	24	62	44
J5, OT-A, last, singleC	21	33	60	49
J1, OT-A, last, wholeT_0.01	22	25	**8**	115
S5, OT, last, wholeT_0.5	23	**7**	49	30
S1, OT-A, last, wholeT_0.01	24	26	**1**	132
J5, OT-A, unif, wholeT_0.01	25	68	79	42.5
J5, OT, unif, wholeT_0.01	26	63	81	42.5
S1, OT, unif, wholeT_0.5	27.5	28	13.5	112
S1, OT-A, unif, wholeT_0.5	27.5	43	13.5	113
S5, CBN-A, unif, wholeT_0.01	29	**6**	37	99
J5, OT, last, wholeT_0.01	30	**4**	58	51
S5, CBN-A, unif, singleC	31	47	68	79
S5, CBN-A, unif, wholeT_0.5	32	48	71	78
S1, OT-A, unif, singleC	33	54	10.5	120
S1, OT, unif, singleC	34	40	10.5	118
J1, OT, last, singleC	35	37	75	76
S5, OT, last, wholeT_0.01	36	**2**	26	83
J1, CBN-A, unif, singleC	37	29	69	80
J1, OT-A, unif, wholeT_0.01	38	61	35	105
J1, CBN-A, unif, wholeT_0.5	39	39	67	75
J1, OT, last, wholeT_0.5	40	14	77	72
J5, OT-A, unif, singleC	41	89	89	23.5
J1, OT, unif, wholeT_0.01	42	53	36	103
J5, OT, unif, singleC	43	81	90	23.5
S5, CBN, unif, singleC	44	46	74	87
J5, DiP-A, unif, wholeT_0.01	45	127	111	34
J5, OT, last, singleC	46	41	86	**4.5**
J1, CBN, unif, singleC	47	38	76	89
J5, OT, last, wholeT_0.5	48	18	88	**4.5**
J5, OT-A, unif, wholeT_0.5	49	78	91	35.5
J5, OT, unif, wholeT_0.5	50	64	92	35.5

Table 18: *(continued)*

Method and sampling	Diff	PFD	PND	FPF
J5, DiP, unif, wholeT_0.01	51	128	128.5	33
J5, DiP-A, unif, singleC	52.5	111	140	**10**
J5, DiP, unif, singleC	52.5	119	140	**4.5**
J5, DiP-A, unif, wholeT_0.5	54	137	131	**10**
J1, CBN, unif, wholeT_0.5	55	32	72	82
J5, DiP, unif, wholeT_0.5	56	141	140	**4.5**
S5, CBN, unif, wholeT_0.5	57	52	78	86
S5, CBN, unif, wholeT_0.01	58	21	47	100
S1, OT, last, wholeT_0.5	59	22	25	114
S1, OT, last, singleC	60	20	23	119
J5, CBN-A, unif, wholeT_0.01	61	56	83	74
S5, DiP-A, unif, wholeT_0.5	62	139	117	50
S5, DiP, unif, wholeT_0.5	63	143	130	29
J5, CBN-A, last, wholeT_0.01	64	45	50	101
J5, CBN-A, unif, singleC	65	95	93	53
J5, CBN-A, unif, wholeT_0.5	66	92	94	47
S5, DiP-A, unif, singleC	67	115	119	37
J1, OT, last, wholeT_0.01	68	34	41	109
S5, DiP, unif, singleC	69	123	127	15
J5, DiP, last, singleC	70	104	140	**4.5**
J5, DiP, last, wholeT_0.5	71	133	140	**4.5**
J5, CBN, unif, wholeT_0.01	72	76	87	81
J5, CBN, unif, singleC	73	96	96	62
J5, DiP-A, last, wholeT_0.5	74	129	140	18
J5, DiP-A, last, singleC	75	98	135	18
J5, CBN, last, singleC	76	74	84	92
J5, CBN, unif, wholeT_0.5	77	83	98	56
J1, CBN-A, unif, wholeT_0.01	78	58	39	122
S1, OT-A, unif, wholeT_0.01	79	35	**5**	133
S1, OT, last, wholeT_0.01	80	31	22	128
J5, CBN-A, last, singleC	81	93	82	94
J1, CBN-A, last, singleC	82	59	63	106
S1, OT, unif, wholeT_0.01	83	36	**6**	130
J5, CBN-A, last, wholeT_0.5	84	86	80	96
J5, CBN, last, wholeT_0.5	85	71	85	95
S5, DiP-A, unif, wholeT_0.01	86	116	97	64
J5, CBN, last, wholeT_0.01	87	51	53	102
S5, DiP, unif, wholeT_0.01	88	125	102	61
J1, DiP-A, unif, wholeT_0.5	89	138	126	27
J1, DiP-A, unif, singleC	90.5	113	140	40
J1, DiP, unif, singleC	90.5	121	140	20
J1, CBN, unif, wholeT_0.01	92	67	51	121
J1, CBN, last, singleC	93	62	64	107
J1, DiP, unif, wholeT_0.5	94	142	133.5	16
J1, CBN-A, last, wholeT_0.5	95	69	73	108
J5, DiP-A, last, wholeT_0.01	96	118	108	66
S5, CBN, last, singleC	97	50	45	117
J1, CBN, last, wholeT_0.5	98	66	70	104
J5, DiP, last, wholeT_0.01	99	126	110	39
J1, DiP, last, singleC	100	106	132	**4.5**

Table 18: *(continued)*

Method and sampling	Diff	PFD	PND	FPF
S5, CBN, last, wholeT_0.5	101	42	46	111
S5, CBN-A, last, singleC	102	49	48	116
J1, DiP, last, wholeT_0.5	103	134	140	**4.5**
J1, DiP-A, last, singleC	104	101	128.5	21
J1, DiP-A, last, wholeT_0.5	105	131	133.5	18
S5, CBN-A, last, wholeT_0.5	106	60	52	110
J1, DiP-A, unif, wholeT_0.01	107	108	103	77
S5, DiP, last, wholeT_0.5	108	135	123.5	12.5
S5, DiP-A, last, wholeT_0.5	109	130	123.5	22
J1, DiP, unif, wholeT_0.01	110	112	107	73
S5, CBN-A, last, wholeT_0.01	111	65	27	125
S5, DiP, last, singleC	112	109	115.5	**10**
S5, DiP-A, last, singleC	113	100	112.5	25.5
J1, CBN-A, last, wholeT_0.01	114	75	43	126
J1, CBN, last, wholeT_0.01	115	94	42	129
S1, CBN-A, unif, wholeT_0.5	116	87	29	131
S1, CBN-A, unif, singleC	117	79	17	135
S5, CBN, last, wholeT_0.01	118	70	30	127
S1, CBN, unif, wholeT_0.5	119	77	32	134
J1, DiP, last, wholeT_0.01	120	120	109	65
S1, CBN, unif, singleC	121	82	21	136
J1, DiP-A, last, wholeT_0.01	122	105	106	90
S5, DiP-A, last, wholeT_0.01	123	97	99.5	84
S5, DiP, last, wholeT_0.01	124	122	104.5	55
S1, DiP-A, unif, wholeT_0.5	125	140	114	54
S1, DiP, unif, wholeT_0.5	126	144	121	48
S1, DiP-A, unif, singleC	127	117	118	52
S1, CBN-A, last, singleC	128	80	20	139
S1, DiP, unif, singleC	129	124	120	41
S1, CBN-A, last, wholeT_0.5	130	90	24	140
S1, CBN-A, unif, wholeT_0.01	131	72	**7**	141
S1, CBN, last, wholeT_0.5	132	88	19	138
S1, DiP-A, unif, wholeT_0.01	133	103	95	97
S1, CBN-A, last, wholeT_0.01	134	73	15	137
S1, DiP, unif, wholeT_0.01	135	107	101	93
S1, CBN, last, singleC	136	91	12	144
S1, DiP-A, last, wholeT_0.01	137	99	99.5	88
S1, DiP, last, wholeT_0.01	138	114	104.5	60
S1, CBN, unif, wholeT_0.01	139	85	**9**	143
S1, DiP, last, wholeT_0.5	140	136	123.5	12.5
S1, DiP, last, singleC	141	110	115.5	14
S1, DiP-A, last, wholeT_0.5	142	132	123.5	28
S1, CBN, last, wholeT_0.01	143	84	18	142
S1, DiP-A, last, singleC	144	102	112.5	25.5

3 Commented example of filtering + method effects

Any filtering scheme can lead to two different types of errors: a) true drivers being missed because they are considered passengers (i.e., being filtered out when they should not); b) passengers being considered as drivers (i.e., not being filtered out when they should). After the filtering step, all methods are applied. Now, different methods can perform very differently, even if they start from the same set of data. Of course, if a filtering method has, say, filtered out many true drivers, none of those will appear in the reconstructed graph. But what each method does with those genes that pass the filtering step depends strongly on method. Note that what we are interested is in the error of filtering **together** with the error introduced by the methods with the filtered data or, to put it differently, in how different methods are able to deal with the possible errors introduced by filtering, as we are interested in the reconstruction of the restrictions (not merely on telling passengers from drivers).

The following examples can illustrate how different methods deal with both types of error (I refer to the original figures, but have reproduced the relevant parts below):

We will start with the first error: true drivers being missed. If we go to *Additional File 3*, p. 23, we see several examples where filtering (J5 here) has eliminated many of the true drivers. (Note that, as explained in the Introduction to *Additional File 3*, across rows, i.e., across Method, the data set is the same, so those correspond to the same data being fitted by different methods). For instance, in the last row we see that the true drivers 3, 6, and 7 are missing from all graphs, because the filtering step removed them. However, OT-A (on the right) is able to correctly reconstruct that 2, 4, and 5 depend on 1 (compare with the original trees, shown in section 2 of *Additional File 1*). In contrast, OT eliminates 1 completely from the tree (so the dependence of 2, 4, and 5 is not correctly inferred); in fact, this is something that OT (not OT-A) often does when the frequency of an alteration is 1 (as explained in the ms. section "CBN-A, OT-A, DiP-A", where data augmentation is described). CBN (left most) makes 2 depend on 1, correctly, but is not able to make 4 and 5 depend on 1. CBN-A and DiP are unable to reconstruct the dependency of 2, 4, and 5 on 1, and DiP-A returns the same graph as OT-A. Thus, in this example, even if starting from a data set where 3, 6, and 7, were missing, OT-A and DiP-A are able to do as well as it is possible after this filtering, but the other methods make larger errors. So the total error is a combination of the error introduced by the filtering step **and** the error introduced by the method itself.

To illustrate the second type of error induced by filtering (passengers being considered as drivers) we will go to *Additional File 3*, p. 8, that shows S1, and we will focus on the first row. All methods start from a bad situation, since filtering has not excluded genes, 30, 42, 46, and 48 , and all those are passengers (filtering has also excluded the true drivers 6 and 10, but we will not focus on that here). Now, all methods will do somewhat poorly, since they will add at least one connection to those passenger genes (except in some exceptional cases where a gene might not be added to the graph, as we saw above). But, again, how poorly they do can differ strongly between methods. For instance, on the left, CBN makes 4 and 5 depend on 42, which itself is made to depend on 30, and that one on 46 and the later on 2; in this tree, thus, there are many dependence relations of true drivers that are wrong as some drivers are made to depend on passengers, and some drivers (e.g., 4) are made to depend, indirectly, on the incorrect driver (2). These errors will be reflected in large Diff and large PFD (note in particular that PFD uses the transitive closure of the relationships, which thus magnifies the effect of incorrectly placing passengers as parents of drivers). In contrast, on the right, in the OT-A graph, 30, 42, 46, and 48 are at the bottom of the graph, and none of the true drivers are dependent on the passengers. In fact, the graph for the true drivers is correct except for the absence of 6 and 10. Again, in this case, even if all methods start from a set of data that includes passengers, the total error made is a combination of the filtering step **and** the error introduced by the method.

These examples illustrate how different methods can perform differently with the same filtering choice. In a sense, different methods are not equal when they have to "make the best of a bad situation".

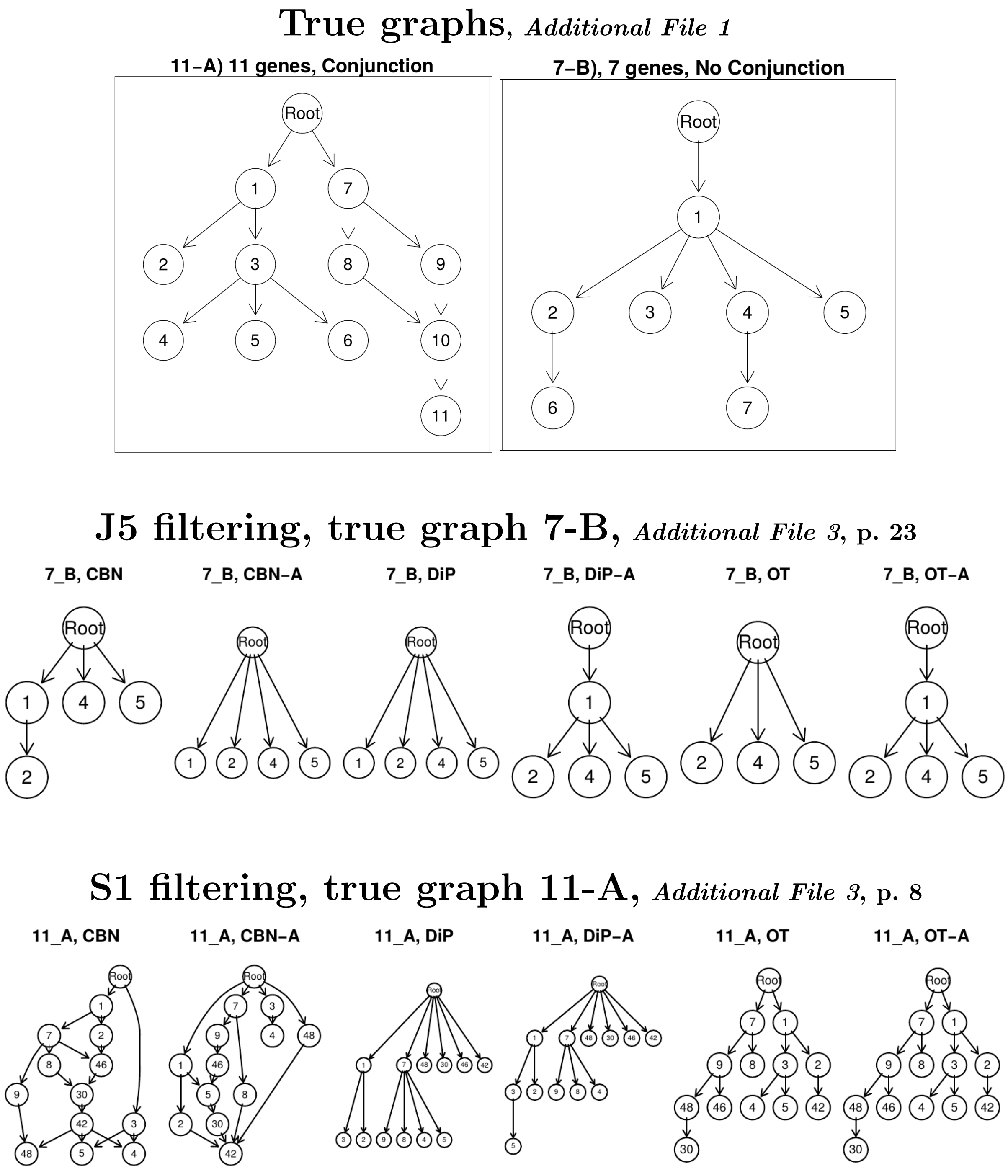

Figure 16: Example of effects of filtering and method. See text for details; these are the relevant figures from the corresponding *Additional file 1* and *Additional File 3*.

4 Frequencies of confidence sets

These tables show, grouped by sampling time, sampling type, and conjunction, the frequency of confidence sets, as shown in the file *Additional file 7*.

The total number of scenarios examined by row is 72 (3 * 4 * 2 * 3), and the overall total is 864 (3 * 2 * 4 * 2 * 3 * 3 * 2).

4.1 Drivers Known, MCB

Table 19: Frequency of confidence sets (method MCB) for measure Diff when Drivers are Known. The table shows the 3 most common confidence set combinations (combinations not shown have a frequency less than 0.025).

	Conjunction	S.Time	S.Type	OT, OT-A	OT-A	OT
1	Yes	last	singleC	36	23	11
2	Yes	last	wholeT_0.01	29	23	12
3	Yes	last	wholeT_0.5	34	24	10
4	Yes	unif	singleC	52	1	1
5	Yes	unif	wholeT_0.01	45	1	0
6	Yes	unif	wholeT_0.5	56	0	1
7	No	last	singleC	38	21	5
8	No	last	wholeT_0.01	32	20	6
9	No	last	wholeT_0.5	37	22	5
10	No	unif	singleC	55	1	1
11	No	unif	wholeT_0.01	56	1	1
12	No	unif	wholeT_0.5	56	1	1
13	OVERALL	-	-	526	138	54

Table 20: Frequency of confidence sets (method MCB) for measure PFD when Drivers are Known. The table shows the 8 most common confidence set combinations (combinations not shown have a frequency less than 0.025).

	Conjunction	S.Time	S.Type	OT, OT-A	DiP, DiP-A, OT, OT-A	OT-A	CBN, CBN-A	DiP-A, OT, OT-A	DiP, OT	OT	DiP, DiP-A
1	Yes	last	singleC	37	2	14	0	5	6	6	0
2	Yes	last	wholeT_0.01	31	3	10	0	4	7	7	5
3	Yes	last	wholeT_0.5	32	2	15	0	6	5	8	0
4	Yes	unif	singleC	41	8	0	8	0	0	1	3
5	Yes	unif	wholeT_0.01	30	9	0	9	2	0	1	5
6	Yes	unif	wholeT_0.5	38	5	0	16	2	0	0	3
7	No	last	singleC	43	6	5	0	3	7	6	0
8	No	last	wholeT_0.01	25	13	7	0	6	9	3	0
9	No	last	wholeT_0.5	39	4	6	0	10	7	5	0
10	No	unif	singleC	40	11	1	7	3	0	1	2
11	No	unif	wholeT_0.01	31	19	0	1	2	1	0	3
12	No	unif	wholeT_0.5	42	8	1	8	1	0	1	1
13	OVERALL	-	-	429	90	59	49	44	42	39	22

Table 21: Frequency of confidence sets (method MCB) for measure PND when Drivers are Known. The table shows the 8 most common confidence set combinations (combinations not shown have a frequency less than 0.025).

	Conjunction	S.Time	S.Type	CBN, CBN-A	OT, OT-A	OT-A	CBN, CBN-A, OT, OT-A	CBN-A	CBN, CBN-A, OT-A	DiP-A, OT-A	DiP, DiP-A, OT, OT-A
1	Yes	last	singleC	34	4	6	3	1	10	0	0
2	Yes	last	wholeT_0.01	24	6	13	5	7	3	1	0
3	Yes	last	wholeT_0.5	38	4	4	3	1	4	1	0
4	Yes	unif	singleC	59	0	0	2	6	1	0	0
5	Yes	unif	wholeT_0.01	52	0	0	8	5	1	0	0
6	Yes	unif	wholeT_0.5	57	0	0	5	7	0	0	0
7	No	last	singleC	11	16	18	10	3	1	7	2
8	No	last	wholeT_0.01	12	18	20	4	0	2	7	5
9	No	last	wholeT_0.5	15	15	17	6	1	2	6	2
10	No	unif	singleC	33	16	2	10	0	1	0	5
11	No	unif	wholeT_0.01	26	15	1	12	1	3	0	4
12	No	unif	wholeT_0.5	36	18	1	7	0	0	0	4
13	OVERALL	-	-	397	112	82	75	32	28	22	22

Table 22: Frequency of confidence sets (method MCB) for measure FPF when Drivers are Known. The table shows the 8 most common confidence set combinations (combinations not shown have a frequency less than 0.025).

	Conjunction	S.Time	S.Type	DiP, DiP-A, OT, OT-A	DiP, DiP-A	DiP, OT	OT, OT-A	DiP, DiP-A, OT	DiP-A, OT, OT-A	DiP, OT, OT-A	DiP, DiP-A, OT-A
1	Yes	last	singleC	39	1	9	7	4	4	3	3
2	Yes	last	wholeT_0.01	18	18	12	6	4	4	5	2
3	Yes	last	wholeT_0.5	41	3	12	4	1	3	6	2
4	Yes	unif	singleC	40	11	0	1	6	1	1	4
5	Yes	unif	wholeT_0.01	22	24	0	4	1	4	3	2
6	Yes	unif	wholeT_0.5	40	13	0	1	5	0	3	3
7	No	last	singleC	37	0	10	10	7	3	2	2
8	No	last	wholeT_0.01	24	12	13	2	3	2	6	2
9	No	last	wholeT_0.5	44	0	11	3	4	7	0	2
10	No	unif	singleC	44	13	0	1	3	0	2	1
11	No	unif	wholeT_0.01	23	20	1	4	5	5	1	3
12	No	unif	wholeT_0.5	42	13	0	3	2	0	0	4
13	OVERALL	-	-	414	128	68	46	45	33	32	30

4.2 Drivers Known, MCB-2

Table 23: Frequency of confidence sets (method MCB-2) for measure Diff when Drivers are Known. The table shows the 4 most common confidence set combinations (combinations not shown have a frequency less than 0.025).

	Conjunction	S.Time	S.Type	OT, OT-A	OT-A	OT	DiP, DiP-A, OT, OT-A
1	Yes	last	singleC	36	23	11	0
2	Yes	last	wholeT_0.01	31	23	10	1
3	Yes	last	wholeT_0.5	36	23	10	0
4	Yes	unif	singleC	54	0	0	3
5	Yes	unif	wholeT_0.01	48	0	0	2
6	Yes	unif	wholeT_0.5	56	0	0	3
7	No	last	singleC	37	21	5	0
8	No	last	wholeT_0.01	33	21	5	3
9	No	last	wholeT_0.5	38	21	5	0
10	No	unif	singleC	58	0	0	4
11	No	unif	wholeT_0.01	57	0	0	4
12	No	unif	wholeT_0.5	58	0	0	4
13	OVERALL	-	-	542	132	46	24

Table 24: Frequency of confidence sets (method MCB-2) for measure PFD when Drivers are Known. The table shows the 8 most common confidence set combinations (combinations not shown have a frequency less than 0.025).

	Conjunction	S.Time	S.Type	OT, OT-A	DiP, DiP-A, OT, OT-A	OT-A	CBN, CBN-A	DiP-A, OT, OT-A	DiP, OT	OT	DiP, DiP-A
1	Yes	last	singleC	35	2	15	0	7	6	6	0
2	Yes	last	wholeT_0.01	29	3	11	0	5	7	8	5
3	Yes	last	wholeT_0.5	33	2	15	0	7	5	7	0
4	Yes	unif	singleC	40	10	0	10	1	0	1	3
5	Yes	unif	wholeT_0.01	30	12	0	10	5	0	0	4
6	Yes	unif	wholeT_0.5	38	7	0	17	2	0	0	4
7	No	last	singleC	43	7	5	0	5	7	5	0
8	No	last	wholeT_0.01	26	15	6	0	5	9	3	1
9	No	last	wholeT_0.5	40	6	6	0	8	7	5	0
10	No	unif	singleC	42	12	0	8	2	0	0	2
11	No	unif	wholeT_0.01	31	21	0	1	2	0	0	2
12	No	unif	wholeT_0.5	45	8	0	9	0	0	0	2
13	OVERALL	-	-	432	105	58	55	49	41	35	23

Table 25: Frequency of confidence sets (method MCB-2) for measure PND when Drivers are Known. The table shows the 7 most common confidence set combinations (combinations not shown have a frequency less than 0.025).

	Conjunction	S.Time	S.Type	CBN, CBN-A	OT, OT-A	OT-A	CBN, CBN-A, OT, OT-A	CBN-A	DiP-A, OT-A	DiP, DiP-A, OT, OT-A
1	Yes	last	singleC	35	4	6	5	1	0	0
2	Yes	last	wholeT_0.01	25	5	14	5	7	1	0
3	Yes	last	wholeT_0.5	38	4	5	5	2	1	0
4	Yes	unif	singleC	58	0	0	4	6	0	0
5	Yes	unif	wholeT_0.01	52	0	0	8	5	0	0
6	Yes	unif	wholeT_0.5	57	0	0	3	7	0	0
7	No	last	singleC	11	15	18	10	3	7	2
8	No	last	wholeT_0.01	12	18	20	6	0	7	5
9	No	last	wholeT_0.5	15	15	17	8	1	7	2
10	No	unif	singleC	34	17	1	11	0	0	4
11	No	unif	wholeT_0.01	29	14	1	11	1	0	5
12	No	unif	wholeT_0.5	38	19	0	6	0	0	4
13	OVERALL	-	-	404	111	82	82	33	23	22

Table 26: Frequency of confidence sets (method MCB-2) for measure FPF when Drivers are Known. The table shows the 6 most common confidence set combinations (combinations not shown have a frequency less than 0.025).

	Conjunction	S.Time	S.Type	DiP, DiP-A, OT, OT-A	DiP, DiP-A	DiP, OT	OT, OT-A	DiP-A, OT, OT-A	DiP, OT, OT-A
1	Yes	last	singleC	48	0	11	7	3	2
2	Yes	last	wholeT_0.01	20	20	12	5	3	7
3	Yes	last	wholeT_0.5	48	2	12	4	2	4
4	Yes	unif	singleC	52	11	0	1	0	0
5	Yes	unif	wholeT_0.01	29	26	0	3	1	2
6	Yes	unif	wholeT_0.5	49	13	0	1	1	1
7	No	last	singleC	48	1	10	9	2	0
8	No	last	wholeT_0.01	29	15	12	2	2	4
9	No	last	wholeT_0.5	51	0	11	4	5	0
10	No	unif	singleC	47	15	0	1	1	1
11	No	unif	wholeT_0.01	32	24	0	4	3	0
12	No	unif	wholeT_0.5	46	14	0	2	0	2
13	OVERALL	-	-	499	141	68	43	23	23

4.3 Drivers Unknown, MCB

Table 27: Frequency of confidence sets (method MCB) for measure Diff when Drivers are Unknown. The table shows the 7 most common confidence set combinations (combinations not shown have a frequency less than 0.025).

	Conjunction	S.Time	S.Type	S5:OT, S5:OT-A	S1:OT, S1:OT-A	S5:OT-A	S5:DiP, S5:DiP-A, S5:OT, S5:OT-A	S5:OT	S1:OT, S1:OT-A, S5:OT, S5:OT-A	S1:DiP, S1:DiP-A, S1:OT, S1:OT-A, S5:DiP, S5:DiP-A, S5:OT, S5:OT-A
1	Yes	last	singleC	13	10	8	4	5	3	0
2	Yes	last	wholeT_0.01	11	1	6	1	9	3	1
3	Yes	last	wholeT_0.5	13	7	8	3	6	2	0
4	Yes	unif	singleC	2	6	0	2	0	0	5
5	Yes	unif	wholeT_0.01	4	1	0	3	0	0	0
6	Yes	unif	wholeT_0.5	3	5	0	2	0	1	7
7	No	last	singleC	11	12	10	5	6	6	0
8	No	last	wholeT_0.01	12	1	7	4	4	3	1
9	No	last	wholeT_0.5	10	11	11	4	4	5	1
10	No	unif	singleC	4	5	0	3	0	1	8
11	No	unif	wholeT_0.01	10	3	0	4	0	1	0
12	No	unif	wholeT_0.5	4	5	0	1	0	5	6
13	OVERALL	-	-	97	67	50	36	34	30	29

Table 28: Frequency of confidence sets (method MCB) for measure PFD when Drivers are Unknown. The table shows the 5 most common confidence set combinations (combinations not shown have a frequency less than 0.025).

	Conjunction	S.Time	S.Type	S1:OT, S1:OT-A	J5:OT, J5:OT-A, S5:OT, S5:OT-A	J1:OT, J1:OT-A, S5:OT, S5:OT-A	S1:CBN, S1:CBN-A, S5:CBN, S5:CBN-A	J1:DiP, J1:OT, J5:DiP, J5:OT, S1:DiP, S5:DiP, S5:OT
1	Yes	last	singleC	8	7	1	1	2
2	Yes	last	wholeT_0.01	1	5	2	0	3
3	Yes	last	wholeT_0.5	9	7	2	0	4
4	Yes	unif	singleC	5	0	3	5	0
5	Yes	unif	wholeT_0.01	2	1	0	0	0
6	Yes	unif	wholeT_0.5	5	0	3	6	0
7	No	last	singleC	8	5	3	0	5
8	No	last	wholeT_0.01	1	4	2	0	7
9	No	last	wholeT_0.5	9	5	2	0	3
10	No	unif	singleC	6	0	4	5	0
11	No	unif	wholeT_0.01	3	0	1	1	0
12	No	unif	wholeT_0.5	3	1	4	6	0
13	OVERALL	-	-	60	35	27	24	24

Table 29: Frequency of confidence sets (method MCB) for measure PND when Drivers are Unknown. The table shows the 8 most common confidence set combinations (combinations not shown have a frequency less than 0.025).

	Conjunction	S.Time	S.Type	S1:CBN, S1:CBN-A, S1:OT, S1:OT-A	S1:OT, S1:OT-A	S1:OT-A, S5:OT-A	S1:OT-A	S1:OT, S1:OT-A, S5:OT, S5:OT-A	S1:CBN-A, S1:OT, S1:OT-A	S1:CBN, S1:OT, S1:OT-A	S1:CBN, S1:CBN-A
1	Yes	last	singleC	11	13	4	8	3	2	4	1
2	Yes	last	wholeT_0.01	5	0	7	8	6	3	2	0
3	Yes	last	wholeT_0.5	12	17	7	6	0	3	0	0
4	Yes	unif	singleC	28	5	0	0	0	4	6	4
5	Yes	unif	wholeT_0.01	20	3	0	1	0	1	1	12
6	Yes	unif	wholeT_0.5	26	5	0	0	0	5	4	2
7	No	last	singleC	2	21	9	10	5	2	3	0
8	No	last	wholeT_0.01	3	2	13	2	10	1	1	0
9	No	last	wholeT_0.5	1	23	9	8	4	2	1	0
10	No	unif	singleC	15	20	0	0	3	5	4	0
11	No	unif	wholeT_0.01	19	18	0	0	1	1	1	5
12	No	unif	wholeT_0.5	14	24	0	0	3	2	3	0
13	OVERALL	-	-	156	151	49	43	35	31	30	24

Table 30: Frequency of confidence sets (method MCB) for measure FPF when Drivers are Unknown. The table shows the 6 most common confidence set combinations (combinations not shown have a frequency less than 0.025).

	Conjunction	S.Time	S.Type	J1:DiP, J1:DiP-A, J1:OT, J1:OT-A, J5:DiP, J5:DiP-A, J5:OT, J5:OT-A, S1:DiP, S1:DiP-A, S5:DiP, S5:DiP-A, S5:OT, S5:OT-A	J1:DiP, J1:OT, J5:DiP, J5:OT, S1:DiP, S5:DiP, S5:OT	J1:DiP, J1:DiP-A, J1:OT, J1:OT-A, J5:DiP, J5:DiP-A, J5:OT, J5:OT-A, S5:DiP, S5:DiP-A, S5:OT, S5:OT-A	J1:DiP, J1:DiP-A, J5:DiP, J5:DiP-A, J5:OT, J5:OT-A, S1:DiP, S1:DiP-A, S5:DiP, S5:DiP-A, S5:OT, S5:OT-A	J1:DiP, J1:DiP-A, J1:OT, J1:OT-A, J5:CBN, J5:CBN-A, J5:DiP, J5:DiP-A, J5:OT, J5:OT-A, S1:DiP, S1:DiP-A, S1:OT, S1:OT-A, S5:DiP, S5:DiP-A, S5:OT, S5:OT-A	J5:DiP, J5:DiP-A, J5:OT, J5:OT-A
1	Yes	last	singleC	3	5	6	6	4	0
2	Yes	last	wholeT_0.01	3	5	2	3	0	10
3	Yes	last	wholeT_0.5	9	8	4	9	2	0
4	Yes	unif	singleC	1	0	3	1	5	0
5	Yes	unif	wholeT_0.01	1	0	1	0	1	5
6	Yes	unif	wholeT_0.5	2	0	2	0	2	0
7	No	last	singleC	12	8	6	5	3	0
8	No	last	wholeT_0.01	4	6	2	2	0	5
9	No	last	wholeT_0.5	9	5	4	4	4	0
10	No	unif	singleC	3	0	3	0	2	0
11	No	unif	wholeT_0.01	1	0	2	2	2	2
12	No	unif	wholeT_0.5	3	0	1	0	2	0
13	OVERALL	-	-	51	37	36	32	27	22

4.4 Drivers Unknown, MCB2-2

Table 31: Frequency of confidence sets (method MCB-2) for measure Diff when Drivers are Unknown. The table shows the 9 most common confidence set combinations (combinations not shown have a frequency less than 0.025).

	Conjunction	S.Time	S.Type	S5:OT, S5:OT-A	S1:OT, S1:OT-A	S5:OT-A	S5:DiP, S5:DiP-A, S5:OT, S5:OT-A	S1:DiP, S1:DiP-A, S1:OT, S1:OT-A, S5:DiP, S5:DiP-A, S5:OT, S5:OT-A	S5:OT	S1:OT, S1:OT-A, S5:OT, S5:OT-A	S1:DiP, S1:DiP-A, S1:OT, S1:OT-A	S5:CBN-A, S5:OT, S5:OT-A
1	Yes	last	singleC	13	10	7	3	0	6	3	1	0
2	Yes	last	wholeT_0.01	11	1	5	2	1	9	3	0	0
3	Yes	last	wholeT_0.5	12	8	8	4	1	5	3	2	0
4	Yes	unif	singleC	2	6	0	2	5	0	0	4	6
5	Yes	unif	wholeT_0.01	5	1	0	3	0	0	0	2	5
6	Yes	unif	wholeT_0.5	2	5	0	3	8	0	1	1	1
7	No	last	singleC	12	12	10	5	0	5	7	1	0
8	No	last	wholeT_0.01	12	1	7	4	1	4	3	0	0
9	No	last	wholeT_0.5	10	11	11	4	1	4	5	0	0
10	No	unif	singleC	6	3	0	2	8	0	2	7	3
11	No	unif	wholeT_0.01	10	3	0	5	1	0	0	1	6
12	No	unif	wholeT_0.5	4	5	0	2	9	0	3	7	3
13	OVERALL	-	-	99	66	48	39	35	33	30	26	24

Table 32: Frequency of confidence sets (method MCB-2) for measure PFD when Drivers are Unknown. The table shows the 6 most common confidence set combinations (combinations not shown have a frequency less than 0.025).

	Conjunction	S.Time	S.Type	S1:OT, S1:OT-A	J5:OT, J5:OT-A, S5:OT, S5:OT-A	S1:CBN, S1:CBN-A, S5:CBN, S5:CBN-A	S5:OT, S5:OT-A	J1:OT, J1:OT-A, S5:OT, S5:OT-A	J1:DiP, J1:OT, J5:DiP, J5:OT, S1:DiP, S5:DiP, S5:OT
1	Yes	last	singleC	8	9	0	5	2	3
2	Yes	last	wholeT_0.01	1	7	0	1	1	5
3	Yes	last	wholeT_0.5	9	8	0	2	2	2
4	Yes	unif	singleC	6	0	6	1	1	0
5	Yes	unif	wholeT_0.01	3	0	0	6	1	0
6	Yes	unif	wholeT_0.5	5	0	7	0	3	0
7	No	last	singleC	8	6	0	3	3	5
8	No	last	wholeT_0.01	1	5	0	3	1	6
9	No	last	wholeT_0.5	9	4	0	2	2	1
10	No	unif	singleC	7	0	5	0	4	0
11	No	unif	wholeT_0.01	3	1	0	2	1	0
12	No	unif	wholeT_0.5	4	1	8	0	4	0
13	OVERALL	-	-	64	41	26	25	25	22

Table 33: Frequency of confidence sets (method MCB-2) for measure PND when Drivers are Unknown. The table shows the 9 most common confidence set combinations (combinations not shown have a frequency less than 0.025).

	Conjunction	S.Time	S.Type	S1:CBN, S1:CBN-A, S1:OT, S1:OT-A	S1:OT, S1:OT-A	S1:OT-A, S5:OT-A	S1:OT-A	J1:CBN, J1:CBN-A, J1:DiP, J1:DiP-A, J1:OT, J1:OT-A, J5:CBN, J5:CBN-A, J5:DiP, J5:DiP-A, J5:OT, J5:OT-A, S1:CBN, S1:CBN-A, S1:DiP, S1:DiP-A, S1:OT, S1:OT-A, S5:CBN, S5:CBN-A, S5:DiP, S5:DiP-A, S5:OT, S5:OT-A	S1:CBN-A, S1:OT, S1:OT-A	S1:OT, S1:OT-A, S5:OT, S5:OT-A	S1:CBN, S1:OT, S1:OT-A	S1:CBN, S1:CBN-A
1	Yes	last	singleC	12	18	6	7	0	2	2	1	0
2	Yes	last	wholeT_0.01	6	2	7	9	0	2	7	1	0
3	Yes	last	wholeT_0.5	11	15	5	8	1	3	0	3	1
4	Yes	unif	singleC	31	4	0	0	9	3	0	3	4
5	Yes	unif	wholeT_0.01	17	4	0	0	0	5	0	0	13
6	Yes	unif	wholeT_0.5	29	4	0	0	11	5	0	2	4
7	No	last	singleC	3	18	9	10	0	4	5	2	0
8	No	last	wholeT_0.01	2	3	11	2	0	2	10	2	0
9	No	last	wholeT_0.5	2	21	8	10	1	1	6	3	0
10	No	unif	singleC	18	17	0	0	8	7	4	4	0
11	No	unif	wholeT_0.01	19	17	1	0	1	2	1	2	5
12	No	unif	wholeT_0.5	16	20	0	0	10	2	3	6	0
13	OVERALL	-	-	166	143	47	46	41	38	38	29	27

Table 34: Frequency of confidence sets (method MCB-2) for measure FPF when Drivers are Unknown. The table shows the 11 most common confidence set combinations (combinations not shown have a frequency less than 0.025).

	Conjunction	S.Time	S.Type	J1:DiP, J1:DiP-A, J1:OT, J1:OT-A, J5:DiP, J5:DiP-A, J5:OT, J5:OT-A, S1:DiP, S1:DiP-A, S5:DiP, S5:DiP-A, S5:OT, S5:OT-A	J1:DiP, J1:DiP-A, J5:DiP, J5:DiP-A, J5:OT, J5:OT-A, S1:DiP, S1:DiP-A, S5:DiP, S5:DiP-A, S5:OT, S5:OT-A	J1:DiP, J1:DiP-A, J1:OT, J1:OT-A, J5:CBN, J5:CBN-A, J5:DiP, J5:DiP-A, J5:OT, J5:OT-A, S1:DiP, S1:DiP-A, S1:OT, S1:OT-A, S5:DiP, S5:DiP-A, S5:OT, S5:OT-A	J1:DiP, J1:OT, J5:DiP, J5:OT, S1:DiP, S5:DiP, S5:OT	J1:CBN, J1:CBN-A, J1:DiP, J1:DiP-A, J1:OT, J1:OT-A, J5:CBN, J5:CBN-A, J5:DiP, J5:DiP-A, J5:OT, J5:OT-A, S1:DiP, S1:DiP-A, S5:CBN, S5:CBN-A, S5:DiP, S5:DiP-A, S5:OT, S5:OT-A	J1:CBN, J1:CBN-A, J1:DiP, J1:DiP-A, J1:OT, J1:OT-A, J5:CBN, J5:CBN-A, J5:DiP, J5:DiP-A, J5:OT, J5:OT-A, S1:CBN, S1:CBN-A, S1:DiP, S1:DiP-A, S1:OT, S1:OT-A, S5:CBN, S5:CBN-A, S5:DiP, S5:DiP-A, S5:OT, S5:OT-A	J1:DiP, J1:DiP-A, J1:OT, J1:OT-A, J5:CBN, J5:CBN-A, J5:DiP, J5:DiP-A, J5:OT, J5:OT-A, S1:DiP, S1:DiP-A, S5:DiP, S5:DiP-A, S5:OT, S5:OT-A	J1:DiP, J1:DiP-A, J1:OT, J1:OT-A, J5:DiP, J5:DiP-A, J5:OT, J5:OT-A, S5:DiP, S5:DiP-A, S5:OT, S5:OT-A	J1:CBN, J1:CBN-A, J1:DiP, J1:DiP-A, J1:OT, J1:OT-A, J5:CBN, J5:CBN-A, J5:DiP, J5:DiP-A, J5:OT, J5:OT-A, S1:DiP, S1:DiP-A, S1:OT, S1:OT-A, S5:CBN, S5:CBN-A, S5:DiP, S5:DiP-A, S5:OT, S5:OT-A	J1:CBN, J1:CBN-A, J1:DiP, J1:DiP-A, J1:OT, J1:OT-A, J5:CBN, J5:CBN-A, J5:DiP, J5:DiP-A, J5:OT, J5:OT-A, S1:DiP, S1:DiP-A, S1:OT, S1:OT-A, S5:DiP, S5:DiP-A, S5:OT, S5:OT-A	J5:DiP, J5:DiP-A, J5:OT, J5:OT-A
1	Yes	last	singleC	10	9	4	7	0	0	5	5	3	1	0
2	Yes	last	wholeT_0.01	4	5	0	6	1	2	0	1	0	0	10
3	Yes	last	wholeT_0.5	13	10	7	6	1	0	1	3	3	0	0
4	Yes	unif	singleC	1	1	6	0	7	8	4	3	4	6	0
5	Yes	unif	wholeT_0.01	1	1	3	0	9	0	3	2	0	0	7
6	Yes	unif	wholeT_0.5	2	0	5	0	7	9	4	1	2	6	0
7	No	last	singleC	16	8	7	9	0	0	2	3	3	0	0
8	No	last	wholeT_0.01	9	8	0	8	1	2	0	1	0	0	5
9	No	last	wholeT_0.5	18	6	6	6	0	1	2	3	3	0	0
10	No	unif	singleC	5	2	6	0	7	6	5	2	5	4	0
11	No	unif	wholeT_0.01	2	2	4	0	5	0	2	3	0	0	3
12	No	unif	wholeT_0.5	4	2	4	0	4	7	4	2	4	8	0
13	OVERALL	-	-	85	54	52	42	42	35	32	29	27	25	25

4.5 Summary frequencies of confidence sets with method MCB

The following tables are the same as those shown in the ms. except the method used is MCB instead of MCB-2 (see *Additional file 1* for details).

**Confidence sets**	**Conjunction**				**No conjunction**			
	Diff	PFD	PND	FPF	Diff	PFD	PND	FPF
OT, OT-A	0.58	0.48	0.03	0.05	0.63	0.51	0.23	0.05
DiP, DiP-A, OT, OT-A	0.01	0.07	-	0.46	0.03	0.14	0.05	0.50
CBN, CBN-A	0.02	0.08	0.61	-	-	0.04	0.31	0.01
DiP, DiP-A	-	0.04	0.01	0.16	0.01	0.01	-	0.13
CBN, CBN-A, OT, OT-A	0.02	0.02	0.06	-	0.02	0.02	0.11	0.01
DiP-A, OT, OT-A	-	0.04	-	0.04	0.01	0.06	0.02	0.04
DiP, DiP-A, OT	-	0.01	-	0.05	-	-	-	0.06
OT-A	0.17	0.09	0.05	-	0.15	0.05	0.14	-
OT	0.08	0.05	-	0.01	0.04	0.04	-	-
DiP, OT	0.01	0.04	-	0.08	0.01	0.06	-	0.08
CBN-A	0.03	-	0.06	-	-	-	0.01	-

Table 35: Frequencies of confidence sets for all measures when Drivers are Known. The table shows the frequency of the most common confidence set combinations. Combinations not shown have a frequency less than 0.05 for all columns. Frequencies normalized by column total (N = 432).

Confidence sets	Conjunction				No conjunction			
	Diff	PFD	PND	FPF	Diff	PFD	PND	FPF
S1:OT-A, S5:OT-A	0.01	-	0.04	-	0.01	-	0.07	-
S1:OT, S1:OT-A	0.07	0.07	0.10	-	0.09	0.07	0.25	-
S1:OT-A	0.02	-	0.05	-	0.02	-	0.05	-
S5:OT-A	0.05	-	-	-	0.06	-	-	-
S5:OT, S5:OT-A	0.11	0.03	-	-	0.12	0.02	-	-
S1:OT, S1:OT-A, S5:OT, S5:OT-A	0.02	-	0.02	-	0.05	-	0.06	-
S1:CBN, S1:CBN-A, S1:OT, S1:OT-A	-	0.01	0.24	-	0.01	-	0.12	-

Table 36: Frequencies of confidence sets for all measures when Drivers are Unknown. The table shows the frequency of the most common confidence set combinations. Combinations not shown have a frequency less than 0.05 for all columns or are composed of more than 10 individual best methods. Frequencies normalized by column total (N = 432). 'A:B' denotes filtering with A and using method B.

5 Model fits: tables

We show here the coefficients for the fitted models. For performance measures "smaller is better" and thus the more negative the coefficient, the better. For "Inferred edges", the model fits a Poisson to the number of edges in the transitive closure of the cover relationships (whether correct or not), so larger coefficients denote large numbers of edges.

The models have been fitted using sum-to-zero contrasts (*contr.Sum*, from package Fox and Weisberg (2011)), so that the missing parameter for a factor (or factor combination) is $-\sum rest\ of\ parameters\ for\ that\ factor$, and the intercept is the overall mean. The fits shown for "Inferred edges" correspond to a model with two-way interactions. The fits for the performance measures correspond to the model in the main text: two-way interactions between all factors and thee-way interactions between the user-controllable factors (Method, Filter, Sample size, Sample time, Sample type); these are the models shown in the figures too.

We display here four columns of the columns that are standard in the summary output of R-INLA (Rue *et al.*, 2009; Martins *et al.*, 2013). These are all models that have been fitted with the default prior for the hyperparameters (Gamma(a, b), $a = 1, b = 0.00005$).

5.1 Drivers Known

5.1.1 Fit for Diff, Drivers Known

Table 37: Model fit for performance measure 'Diff' when there are no passengers.

Coefficient	mean	sd	0.025 quant.	0.975 quant.
(Intercept)	2.042	0.001	2.039	2.044
S.Size(100)	0.132	0.002	0.129	0.135
S.Size(1000)	−0.197	0.002	−0.201	−0.194
Method(CBN)	0.305	0.002	0.301	0.310
Method(CBN-A)	0.248	0.002	0.244	0.253
Method(OT)	−0.505	0.003	−0.512	−0.499
Method(OT-A)	−0.795	0.004	−0.802	−0.788
Method(DiP)	0.415	0.002	0.410	0.419
S.Time(last)	0.032	0.001	0.029	0.034
S.Type(singleC)	−0.014	0.002	−0.017	−0.011
S.Type(wholeT_0.01)	0.012	0.002	0.009	0.015
Model(Bozic)	0.159	0.002	0.155	0.163
Model(McF_4)	−0.113	0.002	−0.117	−0.109
Model(McF_6)	−0.256	0.002	−0.260	−0.251
Conjunction(No)	−0.173	0.001	−0.176	−0.171
sh(0)	0.025	0.001	0.022	0.027
NumNodes(7)	0.003	0.002	−0.001	0.006
NumNodes(9)	−0.136	0.002	−0.139	−0.133
S.Size(100):Method(CBN)	−0.101	0.003	−0.107	−0.095
S.Size(1000):Method(CBN)	0.159	0.003	0.152	0.165
S.Size(100):Method(CBN-A)	−0.086	0.003	−0.092	−0.079
S.Size(1000):Method(CBN-A)	0.136	0.003	0.130	0.143
S.Size(100):Method(OT)	−0.010	0.004	−0.017	−0.002
S.Size(1000):Method(OT)	0.039	0.004	0.031	0.047
S.Size(100):Method(OT-A)	0.001	0.005	−0.007	0.010
S.Size(1000):Method(OT-A)	0.030	0.005	0.020	0.039
S.Size(100):Method(DiP)	0.060	0.003	0.054	0.065
S.Size(1000):Method(DiP)	−0.132	0.003	−0.138	−0.126
S.Size(100):S.Time(last)	−0.004	0.001	−0.007	−0.001
S.Size(1000):S.Time(last)	0.003	0.002	−0.001	0.006
S.Size(100):S.Type(singleC)	−0.001	0.002	−0.005	0.003
S.Size(1000):S.Type(singleC)	0.002	0.002	−0.002	0.007
S.Size(100):S.Type(wholeT_0.01)	0.010	0.002	0.006	0.014
S.Size(1000):S.Type(wholeT_0.01)	−0.014	0.002	−0.018	−0.010
S.Size(100):Model(Bozic)	−0.060	0.002	−0.065	−0.055
S.Size(1000):Model(Bozic)	0.081	0.003	0.076	0.086
S.Size(100):Model(McF_4)	0.029	0.003	0.024	0.034
S.Size(1000):Model(McF_4)	−0.049	0.003	−0.055	−0.044
S.Size(100):Model(McF_6)	0.122	0.003	0.117	0.128
S.Size(1000):Model(McF_6)	−0.168	0.003	−0.174	−0.162
S.Size(100):Conjunction(No)	0.029	0.001	0.026	0.032
S.Size(1000):Conjunction(No)	−0.042	0.002	−0.045	−0.039
S.Size(100):sh(0)	0.000	0.001	−0.003	0.002
S.Size(1000):sh(0)	0.014	0.002	0.011	0.017
S.Size(100):NumNodes(7)	−0.043	0.002	−0.047	−0.039
S.Size(1000):NumNodes(7)	0.070	0.002	0.065	0.074
S.Size(100):NumNodes(9)	0.006	0.002	0.002	0.011
S.Size(1000):NumNodes(9)	−0.012	0.002	−0.016	−0.007
Method(CBN):S.Time(last)	0.078	0.002	0.073	0.082
Method(CBN-A):S.Time(last)	0.091	0.002	0.086	0.095
Method(OT):S.Time(last)	0.077	0.003	0.071	0.082
Method(OT-A):S.Time(last)	−0.186	0.003	−0.192	−0.179
Method(DiP):S.Time(last)	−0.023	0.002	−0.027	−0.019

Table 37: *(continued)*

Coefficient	mean	sd	0.025 quant.	0.975 quant.
Method(CBN):S.Type(singleC)	−0.001	0.003	−0.007	0.005
Method(CBN-A):S.Type(singleC)	0.019	0.003	0.013	0.025
Method(OT):S.Type(singleC)	−0.038	0.004	−0.046	−0.030
Method(OT-A):S.Type(singleC)	−0.057	0.005	−0.066	−0.048
Method(DiP):S.Type(singleC)	0.035	0.003	0.030	0.041
Method(CBN):S.Type(wholeT_0.01)	−0.003	0.003	−0.009	0.004
Method(CBN-A):S.Type(wholeT_0.01)	−0.031	0.003	−0.038	−0.025
Method(OT):S.Type(wholeT_0.01)	0.074	0.004	0.066	0.082
Method(OT-A):S.Type(wholeT_0.01)	0.107	0.005	0.098	0.116
Method(DiP):S.Type(wholeT_0.01)	−0.066	0.003	−0.072	−0.060
Method(CBN):Model(Bozic)	−0.145	0.004	−0.152	−0.137
Method(CBN-A):Model(Bozic)	−0.097	0.004	−0.105	−0.090
Method(OT):Model(Bozic)	0.123	0.005	0.113	0.132
Method(OT-A):Model(Bozic)	0.150	0.005	0.140	0.161
Method(DiP):Model(Bozic)	−0.028	0.004	−0.035	−0.021
Method(CBN):Model(McF_4)	0.181	0.004	0.173	0.188
Method(CBN-A):Model(McF_4)	0.201	0.004	0.194	0.209
Method(OT):Model(McF_4)	−0.148	0.006	−0.159	−0.137
Method(OT-A):Model(McF_4)	−0.279	0.007	−0.292	−0.266
Method(DiP):Model(McF_4)	0.037	0.004	0.030	0.045
Method(CBN):Model(McF_6)	0.108	0.004	0.100	0.116
Method(CBN-A):Model(McF_6)	−0.007	0.004	−0.015	0.002
Method(OT):Model(McF_6)	−0.079	0.006	−0.090	−0.067
Method(OT-A):Model(McF_6)	−0.009	0.006	−0.022	0.003
Method(DiP):Model(McF_6)	0.018	0.004	0.010	0.025
Method(CBN):Conjunction(No)	0.104	0.002	0.099	0.108
Method(CBN-A):Conjunction(No)	0.095	0.002	0.091	0.099
Method(OT):Conjunction(No)	−0.087	0.003	−0.093	−0.081
Method(OT-A):Conjunction(No)	−0.211	0.003	−0.218	−0.205
Method(DiP):Conjunction(No)	0.057	0.002	0.053	0.061
Method(CBN):sh(0)	0.022	0.002	0.018	0.026
Method(CBN-A):sh(0)	0.028	0.002	0.024	0.032
Method(OT):sh(0)	0.029	0.003	0.023	0.035
Method(OT-A):sh(0)	0.057	0.003	0.050	0.063
Method(DiP):sh(0)	−0.072	0.002	−0.076	−0.068
Method(CBN):NumNodes(7)	−0.156	0.003	−0.162	−0.150
Method(CBN-A):NumNodes(7)	−0.181	0.003	−0.188	−0.175
Method(OT):NumNodes(7)	0.455	0.004	0.447	0.462
Method(OT-A):NumNodes(7)	−0.071	0.005	−0.081	−0.062
Method(DiP):NumNodes(7)	0.016	0.003	0.010	0.022
Method(CBN):NumNodes(9)	0.057	0.003	0.051	0.064
Method(CBN-A):NumNodes(9)	0.078	0.003	0.071	0.084
Method(OT):NumNodes(9)	−0.190	0.005	−0.199	−0.181
Method(OT-A):NumNodes(9)	0.070	0.005	0.060	0.079
Method(DiP):NumNodes(9)	−0.032	0.003	−0.038	−0.026
S.Time(last):S.Type(singleC)	0.009	0.001	0.006	0.012
S.Time(last):S.Type(wholeT_0.01)	−0.017	0.001	−0.020	−0.014
S.Time(last):Model(Bozic)	−0.098	0.002	−0.101	−0.095
S.Time(last):Model(McF_4)	−0.020	0.002	−0.024	−0.016
S.Time(last):Model(McF_6)	0.205	0.002	0.201	0.209
S.Time(last):Conjunction(No)	−0.004	0.001	−0.006	−0.001
S.Time(last):sh(0)	0.015	0.001	0.013	0.017
S.Time(last):NumNodes(7)	0.053	0.002	0.050	0.056
S.Time(last):NumNodes(9)	−0.001	0.002	−0.004	0.002
S.Type(singleC):Model(Bozic)	−0.013	0.002	−0.017	−0.008
S.Type(wholeT_0.01):Model(Bozic)	0.013	0.002	0.008	0.017
S.Type(singleC):Model(McF_4)	0.009	0.003	0.004	0.014

Table 37: *(continued)*

Coefficient	mean	sd	0.025 quant.	0.975 quant.
S.Type(wholeT_0.01):Model(McF_4)	−0.011	0.003	−0.016	−0.006
S.Type(singleC):Model(McF_6)	0.001	0.003	−0.004	0.007
S.Type(wholeT_0.01):Model(McF_6)	−0.008	0.003	−0.013	−0.002
S.Type(singleC):Conjunction(No)	−0.001	0.001	−0.004	0.002
S.Type(wholeT_0.01):Conjunction(No)	0.004	0.001	0.001	0.006
S.Type(singleC):sh(0)	0.009	0.001	0.006	0.012
S.Type(wholeT_0.01):sh(0)	−0.021	0.001	−0.024	−0.018
S.Type(singleC):NumNodes(7)	−0.017	0.002	−0.022	−0.013
S.Type(wholeT_0.01):NumNodes(7)	0.038	0.002	0.034	0.042
S.Type(singleC):NumNodes(9)	0.012	0.002	0.008	0.016
S.Type(wholeT_0.01):NumNodes(9)	−0.022	0.002	−0.026	−0.017
Model(Bozic):Conjunction(No)	0.028	0.002	0.025	0.032
Model(McF_4):Conjunction(No)	−0.046	0.002	−0.050	−0.043
Model(McF_6):Conjunction(No)	−0.024	0.002	−0.028	−0.020
Model(Bozic):sh(0)	0.069	0.002	0.066	0.073
Model(McF_4):sh(0)	−0.053	0.002	−0.056	−0.049
Model(McF_6):sh(0)	−0.072	0.002	−0.076	−0.068
Model(Bozic):NumNodes(7)	−0.022	0.003	−0.027	−0.017
Model(McF_4):NumNodes(7)	0.076	0.003	0.071	0.081
Model(McF_6):NumNodes(7)	−0.009	0.003	−0.015	−0.004
Model(Bozic):NumNodes(9)	−0.009	0.003	−0.014	−0.004
Model(McF_4):NumNodes(9)	−0.043	0.003	−0.049	−0.038
Model(McF_6):NumNodes(9)	0.039	0.003	0.033	0.044
Conjunction(No):sh(0)	0.002	0.001	−0.001	0.004
Conjunction(No):NumNodes(7)	−0.038	0.002	−0.041	−0.035
Conjunction(No):NumNodes(9)	−0.027	0.002	−0.030	−0.024
sh(0):NumNodes(7)	−0.027	0.001	−0.030	−0.024
sh(0):NumNodes(9)	0.016	0.002	0.013	0.019

5.1.2 Fit for PFD, Drivers Known

Table 38: Model fit for performance measure 'PFD' when there are no passengers.

Coefficient	mean	sd	0.025 quant.	0.975 quant.
(Intercept)	−0.857	0.007	−0.871	−0.844
S.Size(100)	0.540	0.009	0.523	0.557
S.Size(1000)	−0.686	0.008	−0.701	−0.671
Method(CBN)	0.858	0.007	0.843	0.872
Method(CBN-A)	0.774	0.007	0.759	0.788
Method(OT)	−1.713	0.013	−1.739	−1.687
Method(OT-A)	−1.383	0.011	−1.405	−1.362
Method(DiP)	1.030	0.023	0.985	1.076
S.Time(last)	−0.122	0.005	−0.132	−0.112
S.Type(singleC)	−0.049	0.007	−0.063	−0.035
S.Type(wholeT_0.01)	0.090	0.006	0.078	0.103
Model(Bozic)	0.637	0.008	0.621	0.653
Model(McF_4)	−0.777	0.010	−0.797	−0.757
Model(McF_6)	−0.630	0.009	−0.647	−0.613
Conjunction(No)	0.031	0.005	0.022	0.040
sh(0)	−0.145	0.005	−0.155	−0.135
NumNodes(7)	−0.113	0.008	−0.129	−0.097
NumNodes(9)	−0.101	0.007	−0.115	−0.087
S.Size(100):Method(CBN)	−0.447	0.009	−0.465	−0.428
S.Size(1000):Method(CBN)	0.584	0.008	0.568	0.601
S.Size(100):Method(CBN-A)	−0.435	0.009	−0.454	−0.417
S.Size(1000):Method(CBN-A)	0.568	0.008	0.551	0.584
S.Size(100):Method(OT)	−0.308	0.015	−0.337	−0.279
S.Size(1000):Method(OT)	0.444	0.014	0.417	0.472
S.Size(100):Method(OT-A)	−0.410	0.013	−0.435	−0.386
S.Size(1000):Method(OT-A)	0.546	0.012	0.523	0.569
S.Size(100):Method(DiP)	0.891	0.031	0.831	0.952
S.Size(1000):Method(DiP)	−1.413	0.026	−1.465	−1.361
S.Size(100):S.Time(last)	−0.053	0.004	−0.061	−0.045
S.Size(1000):S.Time(last)	0.076	0.004	0.068	0.084
S.Size(100):S.Type(singleC)	0.011	0.006	0.000	0.023
S.Size(1000):S.Type(singleC)	−0.010	0.006	−0.021	0.002
S.Size(100):S.Type(wholeT_0.01)	−0.015	0.006	−0.026	−0.003
S.Size(1000):S.Type(wholeT_0.01)	0.013	0.006	0.002	0.024
S.Size(100):Model(Bozic)	−0.003	0.007	−0.017	0.010
S.Size(1000):Model(Bozic)	−0.002	0.007	−0.016	0.012
S.Size(100):Model(McF_4)	−0.070	0.007	−0.085	−0.056
S.Size(1000):Model(McF_4)	0.094	0.007	0.080	0.108
S.Size(100):Model(McF_6)	0.076	0.008	0.061	0.091
S.Size(1000):Model(McF_6)	−0.078	0.008	−0.093	−0.063
S.Size(100):Conjunction(No)	0.014	0.004	0.006	0.022
S.Size(1000):Conjunction(No)	−0.015	0.004	−0.023	−0.007
S.Size(100):sh(0)	−0.019	0.004	−0.027	−0.011
S.Size(1000):sh(0)	0.040	0.004	0.032	0.048
S.Size(100):NumNodes(7)	0.015	0.007	0.002	0.029
S.Size(1000):NumNodes(7)	0.000	0.007	−0.013	0.013
S.Size(100):NumNodes(9)	−0.019	0.006	−0.031	−0.008
S.Size(1000):NumNodes(9)	0.021	0.006	0.009	0.032
Method(CBN):S.Time(last)	0.273	0.005	0.262	0.284
Method(CBN-A):S.Time(last)	0.234	0.006	0.223	0.245
Method(OT):S.Time(last)	−0.286	0.011	−0.307	−0.265
Method(OT-A):S.Time(last)	−0.070	0.008	−0.086	−0.053
Method(DiP):S.Time(last)	−0.200	0.015	−0.229	−0.171
Method(CBN):S.Type(singleC)	0.056	0.008	0.040	0.071
Method(CBN-A):S.Type(singleC)	0.080	0.008	0.064	0.096

Table 38: *(continued)*

Coefficient	mean	sd	0.025 quant.	0.975 quant.
Method(OT):S.Type(singleC)	−0.281	0.016	−0.311	−0.250
Method(OT-A):S.Type(singleC)	−0.193	0.012	−0.217	−0.168
Method(DiP):S.Type(singleC)	0.260	0.022	0.218	0.303
Method(CBN):S.Type(wholeT_0.01)	−0.152	0.007	−0.167	−0.138
Method(CBN-A):S.Type(wholeT_0.01)	−0.190	0.007	−0.205	−0.175
Method(OT):S.Type(wholeT_0.01)	0.526	0.013	0.501	0.552
Method(OT-A):S.Type(wholeT_0.01)	0.376	0.011	0.354	0.397
Method(DiP):S.Type(wholeT_0.01)	−0.440	0.020	−0.480	−0.400
Method(CBN):Model(Bozic)	−0.474	0.009	−0.492	−0.456
Method(CBN-A):Model(Bozic)	−0.437	0.009	−0.455	−0.419
Method(OT):Model(Bozic)	0.182	0.015	0.151	0.212
Method(OT-A):Model(Bozic)	−0.111	0.014	−0.138	−0.083
Method(DiP):Model(Bozic)	0.626	0.024	0.578	0.674
Method(CBN):Model(McF_4)	0.888	0.011	0.866	0.909
Method(CBN-A):Model(McF_4)	0.867	0.011	0.845	0.888
Method(OT):Model(McF_4)	−0.157	0.022	−0.200	−0.115
Method(OT-A):Model(McF_4)	−0.349	0.020	−0.388	−0.310
Method(DiP):Model(McF_4)	−0.426	0.029	−0.482	−0.370
Method(CBN):Model(McF_6)	0.040	0.010	0.020	0.059
Method(CBN-A):Model(McF_6)	−0.037	0.010	−0.057	−0.017
Method(OT):Model(McF_6)	0.100	0.018	0.064	0.136
Method(OT-A):Model(McF_6)	0.882	0.014	0.855	0.908
Method(DiP):Model(McF_6)	−1.173	0.028	−1.229	−1.118
Method(CBN):Conjunction(No)	0.144	0.005	0.134	0.155
Method(CBN-A):Conjunction(No)	0.125	0.005	0.115	0.136
Method(OT):Conjunction(No)	−0.077	0.010	−0.096	−0.058
Method(OT-A):Conjunction(No)	−0.042	0.008	−0.058	−0.026
Method(DiP):Conjunction(No)	−0.071	0.014	−0.099	−0.043
Method(CBN):sh(0)	0.265	0.006	0.254	0.276
Method(CBN-A):sh(0)	0.275	0.006	0.263	0.286
Method(OT):sh(0)	0.163	0.010	0.143	0.183
Method(OT-A):sh(0)	0.145	0.008	0.129	0.161
Method(DiP):sh(0)	−0.602	0.016	−0.635	−0.570
Method(CBN):NumNodes(7)	−0.451	0.010	−0.470	−0.431
Method(CBN-A):NumNodes(7)	−0.483	0.010	−0.502	−0.464
Method(OT):NumNodes(7)	0.405	0.016	0.374	0.436
Method(OT-A):NumNodes(7)	−0.121	0.013	−0.147	−0.095
Method(DiP):NumNodes(7)	0.697	0.029	0.640	0.753
Method(CBN):NumNodes(9)	0.277	0.009	0.260	0.294
Method(CBN-A):NumNodes(9)	0.291	0.009	0.275	0.308
Method(OT):NumNodes(9)	−0.308	0.016	−0.340	−0.276
Method(OT-A):NumNodes(9)	0.113	0.013	0.088	0.137
Method(DiP):NumNodes(9)	−0.481	0.024	−0.529	−0.433
S.Time(last):S.Type(singleC)	0.016	0.004	0.008	0.024
S.Time(last):S.Type(wholeT_0.01)	−0.020	0.004	−0.028	−0.012
S.Time(last):Model(Bozic)	−0.266	0.005	−0.276	−0.257
S.Time(last):Model(McF_4)	−0.022	0.005	−0.032	−0.012
S.Time(last):Model(McF_6)	0.569	0.005	0.558	0.579
S.Time(last):Conjunction(No)	−0.004	0.003	−0.010	0.001
S.Time(last):sh(0)	0.033	0.003	0.027	0.038
S.Time(last):NumNodes(7)	0.001	0.005	−0.009	0.010
S.Time(last):NumNodes(9)	0.041	0.004	0.033	0.049
S.Type(singleC):Model(Bozic)	−0.004	0.007	−0.017	0.010
S.Type(wholeT_0.01):Model(Bozic)	−0.017	0.007	−0.030	−0.003
S.Type(singleC):Model(McF_4)	−0.002	0.007	−0.016	0.012
S.Type(wholeT_0.01):Model(McF_4)	0.049	0.007	0.035	0.064
S.Type(singleC):Model(McF_6)	0.003	0.008	−0.012	0.018

Table 38: *(continued)*

Coefficient	mean	sd	0.025 quant.	0.975 quant.
S.Type(wholeT_0.01):Model(McF_6)	−0.037	0.008	−0.052	−0.023
S.Type(singleC):Conjunction(No)	0.006	0.004	−0.002	0.014
S.Type(wholeT_0.01):Conjunction(No)	−0.004	0.004	−0.012	0.004
S.Type(singleC):sh(0)	0.005	0.004	−0.003	0.013
S.Type(wholeT_0.01):sh(0)	−0.020	0.004	−0.028	−0.012
S.Type(singleC):NumNodes(7)	−0.073	0.007	−0.086	−0.059
S.Type(wholeT_0.01):NumNodes(7)	0.107	0.007	0.094	0.121
S.Type(singleC):NumNodes(9)	0.036	0.006	0.025	0.048
S.Type(wholeT_0.01):NumNodes(9)	−0.041	0.006	−0.053	−0.029
Model(Bozic):Conjunction(No)	0.015	0.005	0.005	0.024
Model(McF_4):Conjunction(No)	−0.023	0.005	−0.033	−0.013
Model(McF_6):Conjunction(No)	−0.016	0.005	−0.027	−0.006
Model(Bozic):sh(0)	0.115	0.005	0.106	0.125
Model(McF_4):sh(0)	−0.107	0.005	−0.117	−0.097
Model(McF_6):sh(0)	−0.090	0.005	−0.100	−0.079
Model(Bozic):NumNodes(7)	0.069	0.008	0.053	0.084
Model(McF_4):NumNodes(7)	0.034	0.009	0.017	0.052
Model(McF_6):NumNodes(7)	−0.161	0.009	−0.179	−0.142
Model(Bozic):NumNodes(9)	−0.021	0.007	−0.035	−0.008
Model(McF_4):NumNodes(9)	−0.033	0.008	−0.048	−0.018
Model(McF_6):NumNodes(9)	0.046	0.008	0.030	0.062
Conjunction(No):sh(0)	−0.001	0.003	−0.007	0.004
Conjunction(No):NumNodes(7)	0.000	0.005	−0.010	0.009
Conjunction(No):NumNodes(9)	0.090	0.004	0.082	0.099
sh(0):NumNodes(7)	−0.061	0.005	−0.070	−0.052
sh(0):NumNodes(9)	0.030	0.004	0.022	0.038

5.1.3 Fit for PND, Drivers Known

Table 39: Model fit for performance measure 'PND' when there are no passengers.

Coefficient	mean	sd	0.025 quant.	0.975 quant.
(Intercept)	0.671	0.005	0.662	0.681
S.Size(100)	0.809	0.007	0.796	0.823
S.Size(1000)	−1.021	0.006	−1.032	−1.009
Method(CBN)	−1.466	0.006	−1.477	−1.455
Method(CBN-A)	−1.513	0.006	−1.525	−1.502
Method(OT)	−0.720	0.006	−0.732	−0.709
Method(OT-A)	−1.369	0.006	−1.382	−1.357
Method(DiP)	2.871	0.014	2.844	2.898
S.Time(last)	−0.023	0.004	−0.030	−0.015
S.Type(singleC)	0.132	0.006	0.121	0.143
S.Type(wholeT_0.01)	−0.331	0.006	−0.342	−0.321
Model(Bozic)	0.438	0.007	0.424	0.452
Model(McF_4)	−0.328	0.007	−0.342	−0.315
Model(McF_6)	−1.145	0.007	−1.159	−1.132
Conjunction(No)	−0.416	0.004	−0.424	−0.408
sh(0)	0.047	0.004	0.039	0.055
NumNodes(7)	0.350	0.006	0.338	0.361
NumNodes(9)	−0.007	0.006	−0.018	0.004
S.Size(100):Method(CBN)	−0.798	0.008	−0.813	−0.783
S.Size(1000):Method(CBN)	0.995	0.007	0.981	1.009
S.Size(100):Method(CBN-A)	−0.776	0.008	−0.791	−0.760
S.Size(1000):Method(CBN-A)	0.974	0.007	0.960	0.988
S.Size(100):Method(OT)	−0.457	0.008	−0.473	−0.442
S.Size(1000):Method(OT)	0.590	0.007	0.576	0.605
S.Size(100):Method(OT-A)	−0.425	0.008	−0.441	−0.409
S.Size(1000):Method(OT-A)	0.538	0.008	0.523	0.553
S.Size(100):Method(DiP)	1.044	0.017	1.010	1.078
S.Size(1000):Method(DiP)	−1.479	0.013	−1.504	−1.453
S.Size(100):S.Time(last)	−0.049	0.005	−0.059	−0.038
S.Size(1000):S.Time(last)	0.066	0.005	0.055	0.076
S.Size(100):S.Type(singleC)	0.017	0.008	0.002	0.032
S.Size(1000):S.Type(singleC)	−0.023	0.007	−0.038	−0.008
S.Size(100):S.Type(wholeT_0.01)	−0.032	0.008	−0.047	−0.017
S.Size(1000):S.Type(wholeT_0.01)	0.049	0.007	0.034	0.064
S.Size(100):Model(Bozic)	0.080	0.009	0.062	0.098
S.Size(1000):Model(Bozic)	−0.123	0.009	−0.141	−0.104
S.Size(100):Model(McF_4)	−0.058	0.009	−0.076	−0.040
S.Size(1000):Model(McF_4)	0.098	0.009	0.080	0.115
S.Size(100):Model(McF_6)	−0.069	0.009	−0.087	−0.050
S.Size(1000):Model(McF_6)	0.098	0.009	0.080	0.116
S.Size(100):Conjunction(No)	0.069	0.005	0.058	0.079
S.Size(1000):Conjunction(No)	−0.103	0.005	−0.113	−0.093
S.Size(100):sh(0)	−0.030	0.005	−0.041	−0.020
S.Size(1000):sh(0)	0.070	0.005	0.059	0.080
S.Size(100):NumNodes(7)	−0.084	0.008	−0.099	−0.068
S.Size(1000):NumNodes(7)	0.117	0.008	0.102	0.132
S.Size(100):NumNodes(9)	0.018	0.008	0.003	0.033
S.Size(1000):NumNodes(9)	−0.027	0.008	−0.041	−0.012
Method(CBN):S.Time(last)	0.297	0.005	0.288	0.307
Method(CBN-A):S.Time(last)	0.407	0.005	0.398	0.417
Method(OT):S.Time(last)	0.138	0.005	0.128	0.148
Method(OT-A):S.Time(last)	−0.315	0.005	−0.325	−0.305
Method(DiP):S.Time(last)	−0.073	0.008	−0.088	−0.058
Method(CBN):S.Type(singleC)	−0.143	0.007	−0.156	−0.130
Method(CBN-A):S.Type(singleC)	−0.103	0.007	−0.116	−0.089

Table 39: *(continued)*

Coefficient	mean	sd	0.025 quant.	0.975 quant.
Method(OT):S.Type(singleC)	−0.075	0.007	−0.089	−0.061
Method(OT-A):S.Type(singleC)	−0.048	0.007	−0.062	−0.033
Method(DiP):S.Type(singleC)	0.165	0.011	0.143	0.186
Method(CBN):S.Type(wholeT_0.01)	0.321	0.007	0.307	0.334
Method(CBN-A):S.Type(wholeT_0.01)	0.290	0.007	0.277	0.303
Method(OT):S.Type(wholeT_0.01)	0.140	0.007	0.127	0.154
Method(OT-A):S.Type(wholeT_0.01)	0.076	0.007	0.062	0.091
Method(DiP):S.Type(wholeT_0.01)	−0.369	0.010	−0.389	−0.348
Method(CBN):Model(Bozic)	−0.586	0.009	−0.603	−0.569
Method(CBN-A):Model(Bozic)	−0.539	0.009	−0.556	−0.522
Method(OT):Model(Bozic)	0.002	0.009	−0.015	0.020
Method(OT-A):Model(Bozic)	0.168	0.009	0.151	0.186
Method(DiP):Model(Bozic)	0.415	0.015	0.385	0.445
Method(CBN):Model(McF_4)	0.462	0.008	0.446	0.478
Method(CBN-A):Model(McF_4)	0.545	0.008	0.529	0.561
Method(OT):Model(McF_4)	−0.065	0.008	−0.081	−0.048
Method(OT-A):Model(McF_4)	−0.021	0.009	−0.039	−0.004
Method(DiP):Model(McF_4)	−0.466	0.013	−0.491	−0.441
Method(CBN):Model(McF_6)	1.261	0.009	1.244	1.278
Method(CBN-A):Model(McF_6)	1.130	0.009	1.113	1.147
Method(OT):Model(McF_6)	0.011	0.009	−0.007	0.028
Method(OT-A):Model(McF_6)	−0.426	0.010	−0.447	−0.406
Method(DiP):Model(McF_6)	−0.703	0.013	−0.729	−0.677
Method(CBN):Conjunction(No)	0.344	0.005	0.334	0.353
Method(CBN-A):Conjunction(No)	0.333	0.005	0.323	0.342
Method(OT):Conjunction(No)	−0.214	0.005	−0.224	−0.204
Method(OT-A):Conjunction(No)	−0.407	0.006	−0.418	−0.396
Method(DiP):Conjunction(No)	0.005	0.008	−0.010	0.020
Method(CBN):sh(0)	0.069	0.005	0.060	0.079
Method(CBN-A):sh(0)	0.061	0.005	0.052	0.071
Method(OT):sh(0)	0.214	0.005	0.204	0.224
Method(OT-A):sh(0)	0.260	0.005	0.250	0.270
Method(DiP):sh(0)	−0.396	0.008	−0.411	−0.381
Method(CBN):NumNodes(7)	0.112	0.007	0.097	0.126
Method(CBN-A):NumNodes(7)	−0.070	0.007	−0.085	−0.056
Method(OT):NumNodes(7)	0.332	0.008	0.317	0.347
Method(OT-A):NumNodes(7)	−0.580	0.008	−0.596	−0.564
Method(DiP):NumNodes(7)	0.634	0.014	0.606	0.661
Method(CBN):NumNodes(9)	−0.143	0.007	−0.157	−0.129
Method(CBN-A):NumNodes(9)	−0.049	0.007	−0.063	−0.035
Method(OT):NumNodes(9)	−0.046	0.007	−0.060	−0.032
Method(OT-A):NumNodes(9)	0.320	0.008	0.305	0.335
Method(DiP):NumNodes(9)	−0.279	0.011	−0.302	−0.257
S.Time(last):S.Type(singleC)	−0.015	0.005	−0.026	−0.005
S.Time(last):S.Type(wholeT_0.01)	0.040	0.005	0.029	0.050
S.Time(last):Model(Bozic)	−0.168	0.006	−0.180	−0.155
S.Time(last):Model(McF_4)	−0.040	0.006	−0.053	−0.028
S.Time(last):Model(McF_6)	0.449	0.007	0.436	0.462
S.Time(last):Conjunction(No)	0.010	0.004	0.003	0.017
S.Time(last):sh(0)	0.013	0.004	0.005	0.020
S.Time(last):NumNodes(7)	0.331	0.005	0.320	0.341
S.Time(last):NumNodes(9)	−0.141	0.005	−0.151	−0.130
S.Type(singleC):Model(Bozic)	0.054	0.009	0.036	0.072
S.Type(wholeT_0.01):Model(Bozic)	−0.155	0.009	−0.173	−0.137
S.Type(singleC):Model(McF_4)	−0.028	0.009	−0.045	−0.010
S.Type(wholeT_0.01):Model(McF_4)	0.089	0.009	0.072	0.107
S.Type(singleC):Model(McF_6)	−0.101	0.009	−0.119	−0.083

Table 39: *(continued)*

Coefficient	mean	sd	0.025 quant.	0.975 quant.
S.Type(wholeT_0.01):Model(McF_6)	0.239	0.009	0.221	0.257
S.Type(singleC):Conjunction(No)	0.015	0.005	0.005	0.025
S.Type(wholeT_0.01):Conjunction(No)	−0.026	0.005	−0.036	−0.016
S.Type(singleC):sh(0)	−0.004	0.005	−0.015	0.006
S.Type(wholeT_0.01):sh(0)	−0.019	0.005	−0.030	−0.009
S.Type(singleC):NumNodes(7)	−0.058	0.008	−0.073	−0.043
S.Type(wholeT_0.01):NumNodes(7)	0.125	0.008	0.110	0.140
S.Type(singleC):NumNodes(9)	0.035	0.007	0.021	0.050
S.Type(wholeT_0.01):NumNodes(9)	−0.073	0.007	−0.088	−0.059
Model(Bozic):Conjunction(No)	0.043	0.006	0.030	0.056
Model(McF_4):Conjunction(No)	−0.116	0.006	−0.129	−0.104
Model(McF_6):Conjunction(No)	−0.028	0.007	−0.040	−0.015
Model(Bozic):sh(0)	0.244	0.006	0.231	0.256
Model(McF_4):sh(0)	−0.300	0.006	−0.313	−0.288
Model(McF_6):sh(0)	−0.257	0.007	−0.270	−0.244
Model(Bozic):NumNodes(7)	−0.178	0.009	−0.196	−0.159
Model(McF_4):NumNodes(7)	0.250	0.009	0.232	0.268
Model(McF_6):NumNodes(7)	0.139	0.009	0.121	0.158
Model(Bozic):NumNodes(9)	0.034	0.009	0.016	0.052
Model(McF_4):NumNodes(9)	−0.229	0.009	−0.247	−0.212
Model(McF_6):NumNodes(9)	0.111	0.009	0.093	0.129
Conjunction(No):sh(0)	0.051	0.004	0.044	0.059
Conjunction(No):NumNodes(7)	0.015	0.005	0.004	0.026
Conjunction(No):NumNodes(9)	−0.174	0.005	−0.185	−0.164
sh(0):NumNodes(7)	−0.105	0.005	−0.116	−0.095
sh(0):NumNodes(9)	0.056	0.005	0.046	0.066

5.1.4 Fit for FPF, Drivers Known

Table 40: Model fit for performance measure 'FPF' when there are no passengers.

Coefficient	mean	sd	0.025 quant.	0.975 quant.
(Intercept)	−3.904	0.005	−3.914	−3.894
S.Size(100)	−0.093	0.006	−0.105	−0.082
S.Size(1000)	0.079	0.006	0.068	0.090
Method(CBN)	2.073	0.005	2.062	2.083
Method(CBN-A)	1.964	0.005	1.953	1.974
Method(OT)	−1.083	0.012	−1.106	−1.060
Method(OT-A)	−0.511	0.009	−0.529	−0.493
Method(DiP)	−1.487	0.014	−1.514	−1.459
S.Time(last)	−0.100	0.004	−0.108	−0.091
S.Type(singleC)	−0.194	0.006	−0.206	−0.182
S.Type(wholeT_0.01)	0.412	0.006	0.401	0.423
Model(Bozic)	0.450	0.007	0.437	0.464
Model(McF_4)	−0.609	0.009	−0.627	−0.590
Model(McF_6)	−0.011	0.008	−0.025	0.004
Conjunction(No)	0.016	0.004	0.008	0.024
sh(0)	−0.151	0.004	−0.159	−0.143
NumNodes(7)	0.071	0.006	0.059	0.083
NumNodes(9)	−0.237	0.006	−0.249	−0.225
S.Size(100):Method(CBN)	0.216	0.006	0.204	0.227
S.Size(1000):Method(CBN)	−0.221	0.006	−0.232	−0.211
S.Size(100):Method(CBN-A)	0.227	0.006	0.216	0.239
S.Size(1000):Method(CBN-A)	−0.242	0.006	−0.253	−0.231
S.Size(100):Method(OT)	0.090	0.012	0.067	0.113
S.Size(1000):Method(OT)	−0.061	0.012	−0.084	−0.038
S.Size(100):Method(OT-A)	0.083	0.010	0.063	0.102
S.Size(1000):Method(OT-A)	−0.038	0.010	−0.058	−0.019
S.Size(100):Method(DiP)	−0.320	0.015	−0.350	−0.290
S.Size(1000):Method(DiP)	0.349	0.013	0.324	0.374
S.Size(100):S.Time(last)	−0.055	0.004	−0.063	−0.047
S.Size(1000):S.Time(last)	0.074	0.004	0.066	0.082
S.Size(100):S.Type(singleC)	−0.001	0.006	−0.013	0.010
S.Size(1000):S.Type(singleC)	0.007	0.006	−0.005	0.018
S.Size(100):S.Type(wholeT_0.01)	0.012	0.006	0.001	0.024
S.Size(1000):S.Type(wholeT_0.01)	−0.019	0.006	−0.031	−0.008
S.Size(100):Model(Bozic)	−0.017	0.007	−0.031	−0.003
S.Size(1000):Model(Bozic)	0.042	0.007	0.028	0.056
S.Size(100):Model(McF_4)	−0.083	0.007	−0.097	−0.068
S.Size(1000):Model(McF_4)	0.110	0.007	0.095	0.124
S.Size(100):Model(McF_6)	0.117	0.008	0.102	0.132
S.Size(1000):Model(McF_6)	−0.180	0.008	−0.196	−0.164
S.Size(100):Conjunction(No)	0.007	0.004	−0.001	0.016
S.Size(1000):Conjunction(No)	−0.011	0.004	−0.020	−0.003
S.Size(100):sh(0)	−0.006	0.004	−0.014	0.002
S.Size(1000):sh(0)	0.015	0.004	0.006	0.023
S.Size(100):NumNodes(7)	0.052	0.007	0.039	0.065
S.Size(1000):NumNodes(7)	−0.049	0.007	−0.063	−0.036
S.Size(100):NumNodes(9)	−0.038	0.006	−0.050	−0.027
S.Size(1000):NumNodes(9)	0.045	0.006	0.033	0.057
Method(CBN):S.Time(last)	0.140	0.004	0.132	0.148
Method(CBN-A):S.Time(last)	0.075	0.004	0.067	0.084
Method(OT):S.Time(last)	−0.317	0.009	−0.335	−0.298
Method(OT-A):S.Time(last)	0.070	0.007	0.056	0.085
Method(DiP):S.Time(last)	−0.129	0.010	−0.149	−0.109
Method(CBN):S.Type(singleC)	0.217	0.006	0.205	0.229
Method(CBN-A):S.Type(singleC)	0.234	0.006	0.222	0.246

Table 40: *(continued)*

Coefficient	mean	sd	0.025 quant.	0.975 quant.
Method(OT):S.Type(singleC)	−0.177	0.014	−0.205	−0.149
Method(OT-A):S.Type(singleC)	−0.094	0.011	−0.116	−0.072
Method(DiP):S.Type(singleC)	−0.087	0.016	−0.118	−0.057
Method(CBN):S.Type(wholeT_0.01)	−0.509	0.005	−0.519	−0.498
Method(CBN-A):S.Type(wholeT_0.01)	−0.547	0.005	−0.557	−0.536
Method(OT):S.Type(wholeT_0.01)	0.336	0.011	0.314	0.358
Method(OT-A):S.Type(wholeT_0.01)	0.176	0.009	0.158	0.195
Method(DiP):S.Type(wholeT_0.01)	0.290	0.013	0.265	0.315
Method(CBN):Model(Bozic)	−0.197	0.007	−0.210	−0.184
Method(CBN-A):Model(Bozic)	−0.141	0.007	−0.154	−0.128
Method(OT):Model(Bozic)	0.209	0.013	0.183	0.235
Method(OT-A):Model(Bozic)	−0.080	0.012	−0.104	−0.056
Method(DiP):Model(Bozic)	0.252	0.015	0.223	0.281
Method(CBN):Model(McF_4)	0.661	0.009	0.643	0.679
Method(CBN-A):Model(McF_4)	0.627	0.009	0.609	0.645
Method(OT):Model(McF_4)	−0.140	0.020	−0.180	−0.100
Method(OT-A):Model(McF_4)	−0.376	0.019	−0.413	−0.339
Method(DiP):Model(McF_4)	−0.167	0.023	−0.212	−0.122
Method(CBN):Model(McF_6)	−0.720	0.008	−0.735	−0.705
Method(CBN-A):Model(McF_6)	−0.841	0.008	−0.857	−0.825
Method(OT):Model(McF_6)	−0.074	0.017	−0.107	−0.041
Method(OT-A):Model(McF_6)	0.746	0.012	0.722	0.769
Method(DiP):Model(McF_6)	−0.094	0.019	−0.131	−0.057
Method(CBN):Conjunction(No)	0.001	0.004	−0.006	0.009
Method(CBN-A):Conjunction(No)	−0.017	0.004	−0.025	−0.009
Method(OT):Conjunction(No)	−0.017	0.008	−0.033	0.000
Method(OT-A):Conjunction(No)	−0.007	0.007	−0.021	0.007
Method(DiP):Conjunction(No)	0.046	0.009	0.027	0.064
Method(CBN):sh(0)	0.276	0.004	0.268	0.284
Method(CBN-A):sh(0)	0.290	0.004	0.281	0.298
Method(OT):sh(0)	−0.060	0.009	−0.076	−0.043
Method(OT-A):sh(0)	−0.003	0.007	−0.017	0.011
Method(DiP):sh(0)	−0.345	0.010	−0.366	−0.325
Method(CBN):NumNodes(7)	−0.512	0.007	−0.526	−0.499
Method(CBN-A):NumNodes(7)	−0.515	0.007	−0.529	−0.501
Method(OT):NumNodes(7)	0.457	0.012	0.433	0.482
Method(OT-A):NumNodes(7)	0.123	0.011	0.101	0.145
Method(DiP):NumNodes(7)	0.236	0.015	0.207	0.265
Method(CBN):NumNodes(9)	0.341	0.006	0.329	0.354
Method(CBN-A):NumNodes(9)	0.336	0.007	0.323	0.349
Method(OT):NumNodes(9)	−0.360	0.015	−0.389	−0.331
Method(OT-A):NumNodes(9)	0.008	0.011	−0.013	0.030
Method(DiP):NumNodes(9)	−0.277	0.016	−0.309	−0.245
S.Time(last):S.Type(singleC)	0.029	0.004	0.021	0.038
S.Time(last):S.Type(wholeT_0.01)	−0.056	0.004	−0.065	−0.048
S.Time(last):Model(Bozic)	−0.241	0.005	−0.251	−0.231
S.Time(last):Model(McF_4)	−0.006	0.005	−0.017	0.004
S.Time(last):Model(McF_6)	0.460	0.006	0.449	0.472
S.Time(last):Conjunction(No)	−0.006	0.003	−0.012	0.000
S.Time(last):sh(0)	0.034	0.003	0.028	0.040
S.Time(last):NumNodes(7)	−0.088	0.005	−0.098	−0.079
S.Time(last):NumNodes(9)	0.072	0.004	0.064	0.081
S.Type(singleC):Model(Bozic)	−0.023	0.007	−0.037	−0.009
S.Type(wholeT_0.01):Model(Bozic)	0.034	0.007	0.020	0.048
S.Type(singleC):Model(McF_4)	0.003	0.007	−0.012	0.017
S.Type(wholeT_0.01):Model(McF_4)	0.025	0.007	0.010	0.039
S.Type(singleC):Model(McF_6)	0.039	0.008	0.023	0.054

Table 40: *(continued)*

Coefficient	mean	sd	0.025 quant.	0.975 quant.
S.Type(wholeT_0.01):Model(McF_6)	−0.115	0.008	−0.130	−0.099
S.Type(singleC):Conjunction(No)	0.005	0.004	−0.003	0.013
S.Type(wholeT_0.01):Conjunction(No)	−0.003	0.004	−0.011	0.005
S.Type(singleC):sh(0)	0.004	0.004	−0.005	0.012
S.Type(wholeT_0.01):sh(0)	−0.011	0.004	−0.019	−0.003
S.Type(singleC):NumNodes(7)	−0.060	0.007	−0.073	−0.046
S.Type(wholeT_0.01):NumNodes(7)	0.067	0.007	0.054	0.080
S.Type(singleC):NumNodes(9)	0.025	0.006	0.013	0.037
S.Type(wholeT_0.01):NumNodes(9)	−0.014	0.006	−0.026	−0.002
Model(Bozic):Conjunction(No)	0.027	0.005	0.017	0.036
Model(McF_4):Conjunction(No)	−0.013	0.005	−0.023	−0.003
Model(McF_6):Conjunction(No)	−0.052	0.006	−0.063	−0.041
Model(Bozic):sh(0)	0.070	0.005	0.060	0.080
Model(McF_4):sh(0)	−0.087	0.005	−0.097	−0.077
Model(McF_6):sh(0)	−0.023	0.006	−0.034	−0.012
Model(Bozic):NumNodes(7)	0.132	0.008	0.116	0.147
Model(McF_4):NumNodes(7)	−0.125	0.009	−0.142	−0.108
Model(McF_6):NumNodes(7)	−0.132	0.009	−0.150	−0.113
Model(Bozic):NumNodes(9)	−0.042	0.007	−0.056	−0.028
Model(McF_4):NumNodes(9)	0.061	0.008	0.046	0.076
Model(McF_6):NumNodes(9)	0.009	0.008	−0.007	0.025
Conjunction(No):sh(0)	−0.003	0.003	−0.009	0.003
Conjunction(No):NumNodes(7)	−0.029	0.005	−0.038	−0.019
Conjunction(No):NumNodes(9)	0.037	0.004	0.029	0.046
sh(0):NumNodes(7)	−0.022	0.005	−0.031	−0.013
sh(0):NumNodes(9)	0.007	0.004	−0.001	0.016

5.1.5 Fit for 'Inferred edges', Drivers Known

Table 41: Model fit for performance measure 'Inferred edges' when there are no passengers.

Coefficient	mean	sd	0.025 quant.	0.975 quant.
(Intercept)	1.662	0.002	1.658	1.666
S.Size(100)	−0.344	0.003	−0.350	−0.338
S.Size(1000)	0.403	0.002	0.398	0.407
Method(CBN)	1.138	0.003	1.133	1.143
Method(CBN-A)	1.105	0.003	1.100	1.110
Method(OT)	0.122	0.003	0.115	0.128
Method(OT-A)	0.348	0.003	0.342	0.354
Method(DiP)	−1.582	0.007	−1.596	−1.568
S.Time(last)	−0.023	0.002	−0.026	−0.020
S.Type(singleC)	−0.079	0.002	−0.084	−0.075
S.Type(wholeT_0.01)	0.183	0.002	0.178	0.187
Model(Bozic)	−0.042	0.003	−0.048	−0.037
Model(McF_4)	0.065	0.003	0.059	0.070
Model(McF_6)	0.326	0.003	0.321	0.331
Conjunction(No)	0.051	0.002	0.048	0.055
sh(0)	−0.017	0.002	−0.020	−0.014
NumNodes(7)	−0.458	0.003	−0.463	−0.453
NumNodes(9)	−0.057	0.002	−0.061	−0.052
S.Size(100):Method(CBN)	0.393	0.004	0.386	0.400
S.Size(1000):Method(CBN)	−0.459	0.003	−0.465	−0.454
S.Size(100):Method(CBN-A)	0.395	0.004	0.388	0.402
S.Size(1000):Method(CBN-A)	−0.464	0.003	−0.470	−0.459
S.Size(100):Method(OT)	0.228	0.004	0.219	0.237
S.Size(1000):Method(OT)	−0.262	0.004	−0.270	−0.255
S.Size(100):Method(OT-A)	0.246	0.004	0.238	0.255
S.Size(1000):Method(OT-A)	−0.279	0.004	−0.286	−0.272
S.Size(100):Method(DiP)	−0.621	0.010	−0.642	−0.601
S.Size(1000):Method(DiP)	0.819	0.007	0.806	0.832
S.Size(100):S.Time(last)	−0.005	0.002	−0.009	−0.002
S.Size(1000):S.Time(last)	0.007	0.002	0.004	0.011
S.Size(100):S.Type(singleC)	−0.008	0.002	−0.013	−0.004
S.Size(1000):S.Type(singleC)	0.017	0.002	0.012	0.022
S.Size(100):S.Type(wholeT_0.01)	0.020	0.002	0.015	0.024
S.Size(1000):S.Type(wholeT_0.01)	−0.036	0.002	−0.041	−0.032
S.Size(100):Model(Bozic)	−0.017	0.003	−0.022	−0.011
S.Size(1000):Model(Bozic)	0.036	0.003	0.030	0.042
S.Size(100):Model(McF_4)	−0.020	0.003	−0.026	−0.014
S.Size(1000):Model(McF_4)	0.031	0.003	0.025	0.036
S.Size(100):Model(McF_6)	0.052	0.003	0.046	0.058
S.Size(1000):Model(McF_6)	−0.093	0.003	−0.099	−0.087
S.Size(100):Conjunction(No)	0.002	0.002	−0.001	0.006
S.Size(1000):Conjunction(No)	−0.005	0.002	−0.009	−0.002
S.Size(100):sh(0)	−0.002	0.002	−0.005	0.001
S.Size(1000):sh(0)	−0.003	0.002	−0.006	0.000
S.Size(100):NumNodes(7)	0.022	0.003	0.017	0.028
S.Size(1000):NumNodes(7)	−0.025	0.003	−0.030	−0.020
S.Size(100):NumNodes(9)	−0.017	0.003	−0.022	−0.012
S.Size(1000):NumNodes(9)	0.023	0.002	0.018	0.027
Method(CBN):S.Time(last)	−0.043	0.002	−0.047	−0.039
Method(CBN-A):S.Time(last)	−0.088	0.002	−0.092	−0.084
Method(OT):S.Time(last)	−0.070	0.003	−0.075	−0.064
Method(OT-A):S.Time(last)	0.119	0.003	0.114	0.124
Method(DiP):S.Time(last)	−0.074	0.004	−0.083	−0.066
Method(CBN):S.Type(singleC)	0.101	0.003	0.095	0.107

Table 41: *(continued)*

Coefficient	mean	sd	0.025 quant.	0.975 quant.
Method(CBN-A):S.Type(singleC)	0.103	0.003	0.097	0.109
Method(OT):S.Type(singleC)	0.011	0.004	0.003	0.019
Method(OT-A):S.Type(singleC)	0.011	0.004	0.004	0.018
Method(DiP):S.Type(singleC)	−0.116	0.007	−0.129	−0.103
Method(CBN):S.Type(wholeT_0.01)	−0.240	0.003	−0.245	−0.234
Method(CBN-A):S.Type(wholeT_0.01)	−0.252	0.003	−0.257	−0.246
Method(OT):S.Type(wholeT_0.01)	−0.017	0.004	−0.025	−0.010
Method(OT-A):S.Type(wholeT_0.01)	−0.018	0.003	−0.025	−0.011
Method(DiP):S.Type(wholeT_0.01)	0.272	0.006	0.261	0.284
Method(CBN):Model(Bozic)	0.168	0.004	0.160	0.175
Method(CBN-A):Model(Bozic)	0.184	0.004	0.176	0.191
Method(OT):Model(Bozic)	−0.007	0.005	−0.017	0.003
Method(OT-A):Model(Bozic)	−0.058	0.005	−0.067	−0.048
Method(DiP):Model(Bozic)	−0.105	0.009	−0.121	−0.088
Method(CBN):Model(McF_4)	−0.088	0.004	−0.095	−0.082
Method(CBN-A):Model(McF_4)	−0.119	0.004	−0.127	−0.112
Method(OT):Model(McF_4)	0.043	0.005	0.033	0.052
Method(OT-A):Model(McF_4)	0.003	0.004	−0.006	0.012
Method(DiP):Model(McF_4)	0.090	0.008	0.075	0.105
Method(CBN):Model(McF_6)	−0.621	0.004	−0.628	−0.614
Method(CBN-A):Model(McF_6)	−0.647	0.004	−0.654	−0.640
Method(OT):Model(McF_6)	0.025	0.004	0.016	0.033
Method(OT-A):Model(McF_6)	0.137	0.004	0.129	0.144
Method(DiP):Model(McF_6)	0.405	0.007	0.391	0.418
Method(CBN):Conjunction(No)	−0.087	0.002	−0.091	−0.083
Method(CBN-A):Conjunction(No)	−0.097	0.002	−0.101	−0.093
Method(OT):Conjunction(No)	0.037	0.003	0.032	0.042
Method(OT-A):Conjunction(No)	0.032	0.003	0.027	0.037
Method(DiP):Conjunction(No)	0.069	0.004	0.061	0.078
Method(CBN):sh(0)	0.072	0.002	0.068	0.076
Method(CBN-A):sh(0)	0.078	0.002	0.074	0.082
Method(OT):sh(0)	−0.103	0.003	−0.108	−0.097
Method(OT-A):sh(0)	−0.095	0.003	−0.100	−0.090
Method(DiP):sh(0)	0.053	0.004	0.045	0.062
Method(CBN):NumNodes(7)	−0.200	0.004	−0.208	−0.193
Method(CBN-A):NumNodes(7)	−0.163	0.004	−0.170	−0.156
Method(OT):NumNodes(7)	0.007	0.005	−0.002	0.016
Method(OT-A):NumNodes(7)	0.297	0.004	0.289	0.305
Method(DiP):NumNodes(7)	−0.180	0.008	−0.196	−0.164
Method(CBN):NumNodes(9)	0.126	0.003	0.120	0.133
Method(CBN-A):NumNodes(9)	0.105	0.003	0.099	0.112
Method(OT):NumNodes(9)	−0.024	0.004	−0.032	−0.016
Method(OT-A):NumNodes(9)	−0.121	0.004	−0.128	−0.113
Method(DiP):NumNodes(9)	0.028	0.007	0.015	0.042
S.Time(last):S.Type(singleC)	0.016	0.002	0.013	0.019
S.Time(last):S.Type(wholeT_0.01)	−0.033	0.002	−0.036	−0.030
S.Time(last):Model(Bozic)	−0.026	0.002	−0.030	−0.021
S.Time(last):Model(McF_4)	0.024	0.002	0.020	0.028
S.Time(last):Model(McF_6)	0.008	0.002	0.004	0.012
S.Time(last):Conjunction(No)	−0.003	0.001	−0.005	0.000
S.Time(last):sh(0)	0.009	0.001	0.007	0.011
S.Time(last):NumNodes(7)	−0.133	0.002	−0.137	−0.129
S.Time(last):NumNodes(9)	0.067	0.002	0.064	0.071
S.Type(singleC):Model(Bozic)	−0.030	0.003	−0.036	−0.024
S.Type(wholeT_0.01):Model(Bozic)	0.067	0.003	0.061	0.072
S.Type(singleC):Model(McF_4)	0.017	0.003	0.011	0.022
S.Type(wholeT_0.01):Model(McF_4)	−0.033	0.003	−0.039	−0.027

Table 41: *(continued)*

Coefficient	mean	sd	0.025 quant.	0.975 quant.
S.Type(singleC):Model(McF_6)	0.044	0.003	0.038	0.050
S.Type(wholeT_0.01):Model(McF_6)	−0.107	0.003	−0.112	−0.101
S.Type(singleC):Conjunction(No)	0.002	0.002	−0.001	0.006
S.Type(wholeT_0.01):Conjunction(No)	−0.007	0.002	−0.010	−0.004
S.Type(singleC):sh(0)	0.003	0.002	0.000	0.006
S.Type(wholeT_0.01):sh(0)	−0.003	0.002	−0.006	0.001
S.Type(singleC):NumNodes(7)	−0.009	0.003	−0.014	−0.003
S.Type(wholeT_0.01):NumNodes(7)	0.005	0.003	0.000	0.010
S.Type(singleC):NumNodes(9)	0.002	0.002	−0.003	0.007
S.Type(wholeT_0.01):NumNodes(9)	0.007	0.002	0.002	0.011
Model(Bozic):Conjunction(No)	0.020	0.002	0.016	0.024
Model(McF_4):Conjunction(No)	0.018	0.002	0.014	0.022
Model(McF_6):Conjunction(No)	−0.067	0.002	−0.071	−0.063
Model(Bozic):sh(0)	−0.037	0.002	−0.041	−0.033
Model(McF_4):sh(0)	0.045	0.002	0.041	0.049
Model(McF_6):sh(0)	0.052	0.002	0.048	0.056
Model(Bozic):NumNodes(7)	0.078	0.003	0.071	0.084
Model(McF_4):NumNodes(7)	−0.118	0.003	−0.125	−0.112
Model(McF_6):NumNodes(7)	−0.026	0.003	−0.033	−0.019
Model(Bozic):NumNodes(9)	−0.036	0.003	−0.042	−0.030
Model(McF_4):NumNodes(9)	0.075	0.003	0.069	0.081
Model(McF_6):NumNodes(9)	−0.008	0.003	−0.014	−0.002
Conjunction(No):sh(0)	−0.003	0.001	−0.006	−0.001
Conjunction(No):NumNodes(7)	−0.014	0.002	−0.018	−0.010
Conjunction(No):NumNodes(9)	0.011	0.002	0.007	0.014
sh(0):NumNodes(7)	0.006	0.002	0.002	0.009
sh(0):NumNodes(9)	−0.005	0.002	−0.008	−0.001

5.2 Drivers Unknown

5.2.1 Fit for Diff, Drivers Unknown

Table 42: Model fit for performance measure 'Diff' when there are passengers.

Coefficient	mean	sd	0.025 quant.	0.975 quant.
(Intercept)	2.157	0.001	2.156	2.159
S.Size(100)	0.120	0.001	0.119	0.122
S.Size(1000)	−0.162	0.001	−0.164	−0.160
Filter(J5)	−0.043	0.001	−0.045	−0.041
Filter(J1)	−0.039	0.001	−0.041	−0.037
Filter(S5)	−0.105	0.001	−0.107	−0.103
Method(CBN)	0.187	0.001	0.185	0.189
Method(CBN-A)	0.133	0.001	0.131	0.135
Method(OT)	−0.167	0.001	−0.170	−0.165
Method(OT-A)	−0.338	0.001	−0.341	−0.336
Method(DiP)	0.128	0.001	0.126	0.131
S.Time(last)	0.061	0.001	0.060	0.063
S.Type(singleC)	−0.027	0.001	−0.029	−0.025
S.Type(wholeT_0.01)	0.054	0.001	0.052	0.056
Model(Bozic)	0.105	0.001	0.103	0.108
Model(McF_4)	−0.045	0.001	−0.047	−0.042
Model(McF_6)	−0.130	0.001	−0.132	−0.127
Conjunction(No)	−0.130	0.001	−0.131	−0.128
sh(0)	−0.046	0.001	−0.047	−0.045
NumNodes(7)	−0.004	0.001	−0.006	−0.002
NumNodes(9)	−0.094	0.001	−0.096	−0.092
S.Size(100):Filter(J5)	−0.074	0.001	−0.076	−0.071
S.Size(1000):Filter(J5)	0.092	0.001	0.089	0.095
S.Size(100):Filter(J1)	0.008	0.001	0.005	0.010
S.Size(1000):Filter(J1)	0.015	0.001	0.013	0.018
S.Size(100):Filter(S5)	−0.014	0.001	−0.017	−0.012
S.Size(1000):Filter(S5)	0.010	0.001	0.008	0.013
S.Size(100):Method(CBN)	−0.068	0.001	−0.071	−0.065
S.Size(1000):Method(CBN)	0.108	0.002	0.105	0.111
S.Size(100):Method(CBN-A)	−0.057	0.002	−0.060	−0.054
S.Size(1000):Method(CBN-A)	0.094	0.002	0.091	0.098
S.Size(100):Method(OT)	−0.033	0.002	−0.037	−0.030
S.Size(1000):Method(OT)	0.065	0.002	0.061	0.068
S.Size(100):Method(OT-A)	−0.026	0.002	−0.029	−0.022
S.Size(1000):Method(OT-A)	0.056	0.002	0.052	0.060
S.Size(100):Method(DiP)	0.054	0.002	0.051	0.057
S.Size(1000):Method(DiP)	−0.113	0.002	−0.116	−0.109
S.Size(100):S.Time(last)	−0.006	0.001	−0.008	−0.004
S.Size(1000):S.Time(last)	0.000	0.001	−0.002	0.002
S.Size(100):S.Type(singleC)	−0.001	0.001	−0.004	0.002
S.Size(1000):S.Type(singleC)	−0.006	0.001	−0.009	−0.003
S.Size(100):S.Type(wholeT_0.01)	0.001	0.001	−0.002	0.003
S.Size(1000):S.Type(wholeT_0.01)	0.007	0.001	0.004	0.009
S.Size(100):Model(Bozic)	−0.031	0.002	−0.034	−0.027
S.Size(1000):Model(Bozic)	0.056	0.002	0.052	0.059
S.Size(100):Model(McF_4)	0.015	0.002	0.012	0.019
S.Size(1000):Model(McF_4)	−0.033	0.002	−0.037	−0.030
S.Size(100):Model(McF_6)	0.084	0.002	0.081	0.088
S.Size(1000):Model(McF_6)	−0.124	0.002	−0.128	−0.120
S.Size(100):Conjunction(No)	0.015	0.001	0.013	0.017
S.Size(1000):Conjunction(No)	−0.022	0.001	−0.024	−0.020
S.Size(100):sh(0)	−0.005	0.001	−0.006	−0.003
S.Size(1000):sh(0)	0.017	0.001	0.015	0.019
S.Size(100):NumNodes(7)	−0.008	0.001	−0.010	−0.005
S.Size(1000):NumNodes(7)	0.013	0.001	0.010	0.016
S.Size(100):NumNodes(9)	−0.001	0.001	−0.004	0.002
S.Size(1000):NumNodes(9)	0.004	0.001	0.001	0.007
Filter(J5):Method(CBN)	−0.069	0.002	−0.073	−0.065

Table 42: *(continued)*

Coefficient	mean	sd	0.025 quant.	0.975 quant.
Filter(J1):Method(CBN)	−0.040	0.002	−0.044	−0.036
Filter(S5):Method(CBN)	0.028	0.002	0.024	0.031
Filter(J5):Method(CBN-A)	−0.063	0.002	−0.067	−0.059
Filter(J1):Method(CBN-A)	−0.042	0.002	−0.046	−0.038
Filter(S5):Method(CBN-A)	0.021	0.002	0.017	0.024
Filter(J5):Method(OT)	0.107	0.002	0.103	0.111
Filter(J1):Method(OT)	0.045	0.002	0.041	0.049
Filter(S5):Method(OT)	−0.042	0.002	−0.046	−0.037
Filter(J5):Method(OT-A)	0.120	0.002	0.115	0.124
Filter(J1):Method(OT-A)	0.045	0.002	0.041	0.050
Filter(S5):Method(OT-A)	−0.068	0.002	−0.072	−0.063
Filter(J5):Method(DiP)	−0.049	0.002	−0.053	−0.045
Filter(J1):Method(DiP)	−0.003	0.002	−0.006	0.001
Filter(S5):Method(DiP)	0.035	0.002	0.031	0.038
Filter(J5):S.Time(last)	−0.039	0.001	−0.041	−0.038
Filter(J1):S.Time(last)	0.008	0.001	0.006	0.009
Filter(S5):S.Time(last)	0.033	0.001	0.031	0.035
Filter(J5):S.Type(singleC)	0.027	0.001	0.025	0.030
Filter(J1):S.Type(singleC)	−0.012	0.001	−0.014	−0.009
Filter(S5):S.Type(singleC)	0.000	0.001	−0.003	0.002
Filter(J5):S.Type(wholeT_0.01)	−0.061	0.001	−0.064	−0.059
Filter(J1):S.Type(wholeT_0.01)	0.018	0.001	0.015	0.020
Filter(S5):S.Type(wholeT_0.01)	−0.005	0.001	−0.008	−0.003
Filter(J5):Model(Bozic)	−0.056	0.002	−0.059	−0.053
Filter(J1):Model(Bozic)	−0.001	0.002	−0.004	0.002
Filter(S5):Model(Bozic)	0.033	0.002	0.030	0.036
Filter(J5):Model(McF_4)	0.006	0.002	0.003	0.010
Filter(J1):Model(McF_4)	−0.011	0.002	−0.014	−0.008
Filter(S5):Model(McF_4)	−0.029	0.002	−0.033	−0.026
Filter(J5):Model(McF_6)	0.040	0.002	0.036	0.043
Filter(J1):Model(McF_6)	0.003	0.002	0.000	0.006
Filter(S5):Model(McF_6)	−0.041	0.002	−0.045	−0.038
Filter(J5):Conjunction(No)	0.002	0.001	0.000	0.003
Filter(J1):Conjunction(No)	−0.002	0.001	−0.004	0.000
Filter(S5):Conjunction(No)	−0.010	0.001	−0.012	−0.008
Filter(J5):sh(0)	0.047	0.001	0.045	0.049
Filter(J1):sh(0)	0.005	0.001	0.003	0.006
Filter(S5):sh(0)	0.004	0.001	0.002	0.005
Filter(J5):NumNodes(7)	−0.066	0.001	−0.068	−0.063
Filter(J1):NumNodes(7)	−0.023	0.001	−0.025	−0.020
Filter(S5):NumNodes(7)	0.008	0.001	0.005	0.010
Filter(J5):NumNodes(9)	0.033	0.001	0.031	0.036
Filter(J1):NumNodes(9)	−0.003	0.001	−0.005	0.000
Filter(S5):NumNodes(9)	−0.026	0.001	−0.029	−0.024
Method(CBN):S.Time(last)	0.054	0.001	0.052	0.056
Method(CBN-A):S.Time(last)	0.078	0.001	0.076	0.080
Method(OT):S.Time(last)	0.022	0.001	0.020	0.025
Method(OT-A):S.Time(last)	−0.123	0.001	−0.126	−0.121
Method(DiP):S.Time(last)	−0.009	0.001	−0.012	−0.007
Method(CBN):S.Type(singleC)	−0.019	0.002	−0.022	−0.016
Method(CBN-A):S.Type(singleC)	−0.011	0.002	−0.014	−0.008
Method(OT):S.Type(singleC)	0.004	0.002	0.001	0.007
Method(OT-A):S.Type(singleC)	0.004	0.002	0.000	0.008
Method(DiP):S.Type(singleC)	0.009	0.002	0.006	0.012
Method(CBN):S.Type(wholeT_0.01)	0.043	0.001	0.040	0.046
Method(CBN-A):S.Type(wholeT_0.01)	0.023	0.002	0.020	0.026
Method(OT):S.Type(wholeT_0.01)	−0.013	0.002	−0.017	−0.010
Method(OT-A):S.Type(wholeT_0.01)	−0.015	0.002	−0.019	−0.012
Method(DiP):S.Type(wholeT_0.01)	−0.014	0.002	−0.017	−0.011
Method(CBN):Model(Bozic)	−0.077	0.002	−0.081	−0.073
Method(CBN-A):Model(Bozic)	−0.050	0.002	−0.054	−0.047
Method(OT):Model(Bozic)	0.104	0.002	0.100	0.108

Table 42: *(continued)*

Coefficient	mean	sd	0.025 quant.	0.975 quant.
Method(OT-A):Model(Bozic)	0.122	0.002	0.117	0.126
Method(DiP):Model(Bozic)	−0.059	0.002	−0.063	−0.056
Method(CBN):Model(McF_4)	0.054	0.002	0.050	0.057
Method(CBN-A):Model(McF_4)	0.094	0.002	0.090	0.097
Method(OT):Model(McF_4)	−0.057	0.002	−0.061	−0.053
Method(OT-A):Model(McF_4)	−0.122	0.002	−0.127	−0.118
Method(DiP):Model(McF_4)	0.032	0.002	0.028	0.035
Method(CBN):Model(McF_6)	0.110	0.002	0.106	0.113
Method(CBN-A):Model(McF_6)	0.023	0.002	0.019	0.027
Method(OT):Model(McF_6)	−0.164	0.002	−0.169	−0.160
Method(OT-A):Model(McF_6)	−0.154	0.003	−0.159	−0.149
Method(DiP):Model(McF_6)	0.112	0.002	0.108	0.116
Method(CBN):Conjunction(No)	0.034	0.001	0.032	0.036
Method(CBN-A):Conjunction(No)	0.027	0.001	0.024	0.029
Method(OT):Conjunction(No)	−0.021	0.001	−0.024	−0.019
Method(OT-A):Conjunction(No)	−0.071	0.001	−0.074	−0.069
Method(DiP):Conjunction(No)	0.023	0.001	0.021	0.025
Method(CBN):sh(0)	−0.001	0.001	−0.004	0.001
Method(CBN-A):sh(0)	0.014	0.001	0.012	0.016
Method(OT):sh(0)	0.020	0.001	0.017	0.022
Method(OT-A):sh(0)	0.034	0.001	0.032	0.037
Method(DiP):sh(0)	−0.039	0.001	−0.041	−0.037
Method(CBN):NumNodes(7)	−0.039	0.002	−0.042	−0.036
Method(CBN-A):NumNodes(7)	−0.098	0.002	−0.102	−0.095
Method(OT):NumNodes(7)	0.210	0.002	0.207	0.213
Method(OT-A):NumNodes(7)	−0.150	0.002	−0.154	−0.146
Method(DiP):NumNodes(7)	0.084	0.002	0.081	0.087
Method(CBN):NumNodes(9)	0.023	0.002	0.020	0.026
Method(CBN-A):NumNodes(9)	0.075	0.002	0.072	0.078
Method(OT):NumNodes(9)	−0.087	0.002	−0.090	−0.083
Method(OT-A):NumNodes(9)	0.091	0.002	0.088	0.095
Method(DiP):NumNodes(9)	−0.079	0.002	−0.082	−0.076
S.Time(last):S.Type(singleC)	0.004	0.001	0.002	0.006
S.Time(last):S.Type(wholeT_0.01)	−0.010	0.001	−0.012	−0.008
S.Time(last):Model(Bozic)	−0.045	0.001	−0.047	−0.043
S.Time(last):Model(McF_4)	−0.036	0.001	−0.039	−0.034
S.Time(last):Model(McF_6)	0.129	0.001	0.126	0.131
S.Time(last):Conjunction(No)	0.003	0.001	0.001	0.004
S.Time(last):sh(0)	−0.007	0.001	−0.008	−0.005
S.Time(last):NumNodes(7)	0.035	0.001	0.033	0.037
S.Time(last):NumNodes(9)	0.008	0.001	0.006	0.010
S.Type(singleC):Model(Bozic)	−0.021	0.002	−0.024	−0.018
S.Type(wholeT_0.01):Model(Bozic)	0.039	0.002	0.035	0.042
S.Type(singleC):Model(McF_4)	0.012	0.002	0.009	0.016
S.Type(wholeT_0.01):Model(McF_4)	−0.022	0.002	−0.026	−0.019
S.Type(singleC):Model(McF_6)	0.010	0.002	0.006	0.013
S.Type(wholeT_0.01):Model(McF_6)	−0.023	0.002	−0.026	−0.019
S.Type(singleC):Conjunction(No)	−0.002	0.001	−0.004	0.000
S.Type(wholeT_0.01):Conjunction(No)	0.003	0.001	0.002	0.005
S.Type(singleC):sh(0)	0.019	0.001	0.017	0.021
S.Type(wholeT_0.01):sh(0)	−0.044	0.001	−0.046	−0.042
S.Type(singleC):NumNodes(7)	−0.021	0.001	−0.024	−0.018
S.Type(wholeT_0.01):NumNodes(7)	0.048	0.001	0.045	0.051
S.Type(singleC):NumNodes(9)	0.006	0.001	0.003	0.009
S.Type(wholeT_0.01):NumNodes(9)	−0.015	0.001	−0.018	−0.012
Model(Bozic):Conjunction(No)	0.014	0.001	0.012	0.017
Model(McF_4):Conjunction(No)	−0.015	0.001	−0.017	−0.012
Model(McF_6):Conjunction(No)	−0.013	0.001	−0.015	−0.010
Model(Bozic):sh(0)	−0.015	0.001	−0.017	−0.013
Model(McF_4):sh(0)	0.017	0.001	0.015	0.019
Model(McF_6):sh(0)	0.011	0.001	0.009	0.014
Model(Bozic):NumNodes(7)	−0.018	0.002	−0.022	−0.015

Table 42: *(continued)*

Coefficient	mean	sd	0.025 quant.	0.975 quant.
Model(McF_4):NumNodes(7)	0.053	0.002	0.049	0.056
Model(McF_6):NumNodes(7)	0.027	0.002	0.023	0.030
Model(Bozic):NumNodes(9)	0.000	0.002	−0.003	0.004
Model(McF_4):NumNodes(9)	−0.033	0.002	−0.037	−0.030
Model(McF_6):NumNodes(9)	0.010	0.002	0.007	0.014
Conjunction(No):sh(0)	−0.004	0.001	−0.006	−0.003
Conjunction(No):NumNodes(7)	−0.056	0.001	−0.058	−0.054
Conjunction(No):NumNodes(9)	−0.009	0.001	−0.011	−0.007
sh(0):NumNodes(7)	−0.012	0.001	−0.014	−0.010
sh(0):NumNodes(9)	0.002	0.001	0.000	0.004

5.2.2 Fit for PFD, Drivers Unknown

Table 43: Model fit for performance measure 'PFD' when there are passengers.

Coefficient	mean	sd	0.025 quant.	0.975 quant.
(Intercept)	−0.572	0.006	−0.585	−0.560
S.Size(100)	0.928	0.008	0.913	0.943
S.Size(1000)	−1.080	0.008	−1.095	−1.065
Filter(J5)	−0.565	0.009	−0.584	−0.547
Filter(J1)	−0.052	0.006	−0.065	−0.040
Filter(S5)	−0.437	0.006	−0.449	−0.425
Method(CBN)	0.879	0.006	0.867	0.891
Method(CBN-A)	0.705	0.006	0.693	0.716
Method(OT)	−0.999	0.008	−1.015	−0.983
Method(OT-A)	−0.762	0.007	−0.775	−0.748
Method(DiP)	0.180	0.018	0.145	0.214
S.Time(last)	0.129	0.005	0.118	0.139
S.Type(singleC)	−0.217	0.008	−0.232	−0.202
S.Type(wholeT_0.01)	0.459	0.007	0.446	0.473
Model(Bozic)	0.789	0.009	0.771	0.807
Model(McF_4)	−0.684	0.009	−0.703	−0.666
Model(McF_6)	−0.613	0.008	−0.630	−0.597
Conjunction(No)	−0.099	0.005	−0.109	−0.090
sh(0)	−0.454	0.005	−0.465	−0.443
NumNodes(7)	−0.403	0.008	−0.418	−0.388
NumNodes(9)	0.365	0.007	0.351	0.380
S.Size(100):Filter(J5)	−0.187	0.007	−0.200	−0.173
S.Size(1000):Filter(J5)	0.198	0.007	0.184	0.211
S.Size(100):Filter(J1)	0.376	0.005	0.366	0.386
S.Size(1000):Filter(J1)	−0.250	0.006	−0.261	−0.239
S.Size(100):Filter(S5)	−0.125	0.005	−0.135	−0.116
S.Size(1000):Filter(S5)	0.117	0.005	0.107	0.127
S.Size(100):Method(CBN)	−0.521	0.006	−0.533	−0.509
S.Size(1000):Method(CBN)	0.671	0.006	0.659	0.683
S.Size(100):Method(CBN-A)	−0.496	0.006	−0.508	−0.484
S.Size(1000):Method(CBN-A)	0.639	0.006	0.627	0.651
S.Size(100):Method(OT)	−0.271	0.007	−0.285	−0.257
S.Size(1000):Method(OT)	0.333	0.008	0.319	0.348
S.Size(100):Method(OT-A)	−0.382	0.006	−0.395	−0.369
S.Size(1000):Method(OT-A)	0.473	0.007	0.460	0.486
S.Size(100):Method(DiP)	1.086	0.021	1.046	1.127
S.Size(1000):Method(DiP)	−1.574	0.020	−1.613	−1.535
S.Size(100):S.Time(last)	−0.145	0.006	−0.157	−0.134
S.Size(1000):S.Time(last)	0.184	0.006	0.172	0.195
S.Size(100):S.Type(singleC)	0.070	0.008	0.054	0.086
S.Size(1000):S.Type(singleC)	−0.146	0.009	−0.163	−0.129
S.Size(100):S.Type(wholeT_0.01)	−0.187	0.008	−0.202	−0.171
S.Size(1000):S.Type(wholeT_0.01)	0.305	0.008	0.288	0.321
S.Size(100):Model(Bozic)	0.043	0.010	0.024	0.063
S.Size(1000):Model(Bozic)	0.009	0.011	−0.012	0.030
S.Size(100):Model(McF_4)	0.021	0.010	0.002	0.040
S.Size(1000):Model(McF_4)	−0.084	0.010	−0.104	−0.064
S.Size(100):Model(McF_6)	0.003	0.010	−0.016	0.022
S.Size(1000):Model(McF_6)	−0.095	0.010	−0.115	−0.076
S.Size(100):Conjunction(No)	−0.014	0.006	−0.025	−0.002
S.Size(1000):Conjunction(No)	0.017	0.006	0.005	0.029
S.Size(100):sh(0)	0.002	0.006	−0.009	0.014
S.Size(1000):sh(0)	0.041	0.006	0.029	0.053
S.Size(100):NumNodes(7)	0.161	0.008	0.145	0.177
S.Size(1000):NumNodes(7)	−0.187	0.009	−0.204	−0.170
S.Size(100):NumNodes(9)	−0.092	0.008	−0.108	−0.076
S.Size(1000):NumNodes(9)	0.098	0.008	0.081	0.115
Filter(J5):Method(CBN)	0.157	0.011	0.136	0.178
Filter(J1):Method(CBN)	−0.170	0.008	−0.186	−0.155
Filter(S5):Method(CBN)	0.052	0.007	0.038	0.066

Table 43: *(continued)*

Coefficient	mean	sd	0.025 quant.	0.975 quant.
Filter(J5):Method(CBN-A)	0.068	0.010	0.048	0.089
Filter(J1):Method(CBN-A)	−0.135	0.008	−0.150	−0.120
Filter(S5):Method(CBN-A)	0.052	0.007	0.037	0.066
Filter(J5):Method(OT)	−0.510	0.017	−0.543	−0.478
Filter(J1):Method(OT)	0.249	0.010	0.229	0.269
Filter(S5):Method(OT)	−0.236	0.011	−0.257	−0.215
Filter(J5):Method(OT-A)	−0.095	0.012	−0.119	−0.071
Filter(J1):Method(OT-A)	0.087	0.008	0.071	0.104
Filter(S5):Method(OT-A)	−0.136	0.008	−0.153	−0.120
Filter(J5):Method(DiP)	−0.004	0.031	−0.065	0.057
Filter(J1):Method(DiP)	−0.079	0.021	−0.121	−0.037
Filter(S5):Method(DiP)	0.101	0.020	0.062	0.139
Filter(J5):S.Time(last)	−0.166	0.006	−0.177	−0.155
Filter(J1):S.Time(last)	0.103	0.004	0.095	0.111
Filter(S5):S.Time(last)	0.184	0.004	0.177	0.192
Filter(J5):S.Type(singleC)	0.033	0.008	0.018	0.047
Filter(J1):S.Type(singleC)	−0.061	0.006	−0.072	−0.050
Filter(S5):S.Type(singleC)	−0.023	0.005	−0.034	−0.012
Filter(J5):S.Type(wholeT_0.01)	−0.100	0.007	−0.113	−0.087
Filter(J1):S.Type(wholeT_0.01)	0.131	0.005	0.122	0.141
Filter(S5):S.Type(wholeT_0.01)	0.021	0.005	0.011	0.030
Filter(J5):Model(Bozic)	−0.271	0.010	−0.290	−0.251
Filter(J1):Model(Bozic)	0.130	0.007	0.117	0.144
Filter(S5):Model(Bozic)	0.255	0.007	0.241	0.268
Filter(J5):Model(McF_4)	0.114	0.010	0.094	0.134
Filter(J1):Model(McF_4)	−0.066	0.007	−0.080	−0.051
Filter(S5):Model(McF_4)	−0.247	0.007	−0.260	−0.233
Filter(J5):Model(McF_6)	0.165	0.008	0.150	0.181
Filter(J1):Model(McF_6)	−0.077	0.006	−0.089	−0.066
Filter(S5):Model(McF_6)	−0.143	0.006	−0.155	−0.132
Filter(J5):Conjunction(No)	−0.009	0.005	−0.019	0.000
Filter(J1):Conjunction(No)	−0.026	0.004	−0.033	−0.019
Filter(S5):Conjunction(No)	0.051	0.003	0.044	0.058
Filter(J5):sh(0)	0.062	0.005	0.052	0.072
Filter(J1):sh(0)	−0.046	0.004	−0.054	−0.039
Filter(S5):sh(0)	0.037	0.004	0.029	0.044
Filter(J5):NumNodes(7)	−0.376	0.007	−0.390	−0.363
Filter(J1):NumNodes(7)	−0.052	0.005	−0.062	−0.042
Filter(S5):NumNodes(7)	−0.166	0.005	−0.177	−0.156
Filter(J5):NumNodes(9)	0.320	0.007	0.307	0.334
Filter(J1):NumNodes(9)	−0.038	0.005	−0.048	−0.027
Filter(S5):NumNodes(9)	−0.058	0.005	−0.068	−0.047
Method(CBN):S.Time(last)	0.264	0.004	0.256	0.272
Method(CBN-A):S.Time(last)	0.275	0.004	0.267	0.282
Method(OT):S.Time(last)	0.053	0.005	0.044	0.062
Method(OT-A):S.Time(last)	−0.003	0.004	−0.012	0.005
Method(DiP):S.Time(last)	−0.502	0.011	−0.524	−0.481
Method(CBN):S.Type(singleC)	0.047	0.006	0.036	0.059
Method(CBN-A):S.Type(singleC)	0.065	0.006	0.053	0.076
Method(OT):S.Type(singleC)	−0.131	0.007	−0.145	−0.117
Method(OT-A):S.Type(singleC)	−0.078	0.006	−0.090	−0.065
Method(DiP):S.Type(singleC)	0.091	0.018	0.057	0.126
Method(CBN):S.Type(wholeT_0.01)	−0.095	0.005	−0.105	−0.085
Method(CBN-A):S.Type(wholeT_0.01)	−0.159	0.005	−0.169	−0.148
Method(OT):S.Type(wholeT_0.01)	0.249	0.006	0.237	0.262
Method(OT-A):S.Type(wholeT_0.01)	0.147	0.006	0.136	0.158
Method(DiP):S.Type(wholeT_0.01)	−0.109	0.015	−0.138	−0.080
Method(CBN):Model(Bozic)	−0.439	0.007	−0.453	−0.425
Method(CBN-A):Model(Bozic)	−0.415	0.007	−0.429	−0.401
Method(OT):Model(Bozic)	0.002	0.008	−0.014	0.018
Method(OT-A):Model(Bozic)	−0.174	0.008	−0.189	−0.159
Method(DiP):Model(Bozic)	0.778	0.018	0.743	0.814

Table 43: *(continued)*

Coefficient	mean	sd	0.025 quant.	0.975 quant.
Method(CBN):Model(McF_4)	0.661	0.008	0.646	0.675
Method(CBN-A):Model(McF_4)	0.694	0.008	0.679	0.708
Method(OT):Model(McF_4)	0.155	0.009	0.138	0.173
Method(OT-A):Model(McF_4)	0.119	0.008	0.103	0.135
Method(DiP):Model(McF_4)	−0.664	0.022	−0.708	−0.620
Method(CBN):Model(McF_6)	0.165	0.006	0.152	0.177
Method(CBN-A):Model(McF_6)	0.095	0.006	0.083	0.108
Method(OT):Model(McF_6)	−0.017	0.008	−0.032	−0.002
Method(OT-A):Model(McF_6)	0.362	0.007	0.349	0.376
Method(DiP):Model(McF_6)	−0.965	0.020	−1.004	−0.927
Method(CBN):Conjunction(No)	0.085	0.004	0.077	0.092
Method(CBN-A):Conjunction(No)	0.073	0.004	0.066	0.080
Method(OT):Conjunction(No)	−0.037	0.004	−0.046	−0.028
Method(OT-A):Conjunction(No)	−0.005	0.004	−0.013	0.002
Method(DiP):Conjunction(No)	−0.061	0.010	−0.082	−0.041
Method(CBN):sh(0)	0.256	0.004	0.248	0.264
Method(CBN-A):sh(0)	0.295	0.004	0.287	0.303
Method(OT):sh(0)	0.117	0.005	0.107	0.127
Method(OT-A):sh(0)	0.159	0.005	0.150	0.168
Method(DiP):sh(0)	−0.697	0.013	−0.723	−0.672
Method(CBN):NumNodes(7)	−0.275	0.006	−0.286	−0.263
Method(CBN-A):NumNodes(7)	−0.383	0.006	−0.395	−0.372
Method(OT):NumNodes(7)	0.325	0.007	0.311	0.340
Method(OT-A):NumNodes(7)	−0.250	0.006	−0.262	−0.238
Method(DiP):NumNodes(7)	0.859	0.018	0.823	0.895
Method(CBN):NumNodes(9)	0.189	0.006	0.178	0.200
Method(CBN-A):NumNodes(9)	0.279	0.006	0.268	0.290
Method(OT):NumNodes(9)	−0.062	0.007	−0.075	−0.049
Method(OT-A):NumNodes(9)	0.183	0.006	0.171	0.195
Method(DiP):NumNodes(9)	−0.674	0.016	−0.705	−0.642
S.Time(last):S.Type(singleC)	0.087	0.006	0.075	0.099
S.Time(last):S.Type(wholeT_0.01)	−0.179	0.006	−0.191	−0.168
S.Time(last):Model(Bozic)	−0.150	0.007	−0.164	−0.135
S.Time(last):Model(McF_4)	−0.096	0.007	−0.110	−0.082
S.Time(last):Model(McF_6)	0.405	0.007	0.391	0.419
S.Time(last):Conjunction(No)	−0.027	0.004	−0.035	−0.019
S.Time(last):sh(0)	−0.062	0.004	−0.071	−0.054
S.Time(last):NumNodes(7)	0.174	0.006	0.162	0.185
S.Time(last):NumNodes(9)	−0.050	0.006	−0.062	−0.039
S.Type(singleC):Model(Bozic)	−0.031	0.011	−0.052	−0.010
S.Type(wholeT_0.01):Model(Bozic)	0.050	0.010	0.031	0.070
S.Type(singleC):Model(McF_4)	0.021	0.010	0.001	0.041
S.Type(wholeT_0.01):Model(McF_4)	−0.020	0.010	−0.039	−0.001
S.Type(singleC):Model(McF_6)	0.039	0.010	0.020	0.058
S.Type(wholeT_0.01):Model(McF_6)	−0.073	0.010	−0.091	−0.054
S.Type(singleC):Conjunction(No)	0.003	0.006	−0.009	0.014
S.Type(wholeT_0.01):Conjunction(No)	−0.001	0.006	−0.013	0.010
S.Type(singleC):sh(0)	0.062	0.006	0.050	0.074
S.Type(wholeT_0.01):sh(0)	−0.159	0.006	−0.170	−0.147
S.Type(singleC):NumNodes(7)	−0.141	0.009	−0.158	−0.124
S.Type(wholeT_0.01):NumNodes(7)	0.338	0.008	0.321	0.354
S.Type(singleC):NumNodes(9)	0.068	0.008	0.051	0.085
S.Type(wholeT_0.01):NumNodes(9)	−0.150	0.008	−0.165	−0.134
Model(Bozic):Conjunction(No)	−0.013	0.007	−0.028	0.001
Model(McF_4):Conjunction(No)	−0.051	0.007	−0.065	−0.037
Model(McF_6):Conjunction(No)	0.071	0.007	0.058	0.085
Model(Bozic):sh(0)	−0.154	0.007	−0.169	−0.139
Model(McF_4):sh(0)	0.151	0.007	0.137	0.165
Model(McF_6):sh(0)	0.226	0.007	0.212	0.239
Model(Bozic):NumNodes(7)	−0.085	0.010	−0.105	−0.065
Model(McF_4):NumNodes(7)	0.277	0.010	0.257	0.296
Model(McF_6):NumNodes(7)	0.427	0.010	0.408	0.446

Table 43: *(continued)*

Coefficient	mean	sd	0.025 quant.	0.975 quant.
Model(Bozic):NumNodes(9)	0.039	0.010	0.019	0.059
Model(McF_4):NumNodes(9)	−0.186	0.010	−0.205	−0.166
Model(McF_6):NumNodes(9)	−0.252	0.010	−0.272	−0.233
Conjunction(No):sh(0)	0.025	0.004	0.017	0.033
Conjunction(No):NumNodes(7)	−0.042	0.006	−0.054	−0.031
Conjunction(No):NumNodes(9)	0.002	0.006	−0.010	0.013
sh(0):NumNodes(7)	−0.150	0.006	−0.162	−0.138
sh(0):NumNodes(9)	0.091	0.006	0.079	0.102

5.2.3 Fit for PND, Drivers Unknown

Table 44: Model fit for performance measure 'PND' when there are passengers.

Coefficient	mean	sd	0.025 quant.	0.975 quant.
(Intercept)	2.465	0.004	2.457	2.473
S.Size(100)	0.464	0.005	0.454	0.474
S.Size(1000)	−0.558	0.005	−0.567	−0.549
Filter(J5)	1.150	0.004	1.142	1.159
Filter(J1)	0.182	0.003	0.175	0.188
Filter(S5)	−0.230	0.003	−0.236	−0.224
Method(CBN)	−0.515	0.004	−0.523	−0.508
Method(CBN-A)	−0.659	0.004	−0.667	−0.651
Method(OT)	−0.678	0.004	−0.686	−0.670
Method(OT-A)	−1.110	0.004	−1.118	−1.103
Method(DiP)	1.801	0.009	1.784	1.818
S.Time(last)	−0.383	0.003	−0.389	−0.376
S.Type(singleC)	0.247	0.005	0.238	0.256
S.Type(wholeT_0.01)	−0.610	0.005	−0.619	−0.601
Model(Bozic)	0.674	0.006	0.662	0.686
Model(McF_4)	−0.528	0.006	−0.539	−0.517
Model(McF_6)	−1.857	0.006	−1.868	−1.846
Conjunction(No)	−0.292	0.003	−0.299	−0.286
sh(0)	0.264	0.003	0.258	0.271
NumNodes(7)	−0.384	0.005	−0.393	−0.375
NumNodes(9)	0.319	0.005	0.310	0.329
S.Size(100):Filter(J5)	−0.024	0.004	−0.032	−0.015
S.Size(1000):Filter(J5)	0.065	0.004	0.057	0.073
S.Size(100):Filter(J1)	−0.012	0.004	−0.019	−0.004
S.Size(1000):Filter(J1)	0.029	0.004	0.022	0.036
S.Size(100):Filter(S5)	−0.019	0.003	−0.026	−0.012
S.Size(1000):Filter(S5)	0.010	0.003	0.003	0.016
S.Size(100):Method(CBN)	−0.608	0.005	−0.617	−0.599
S.Size(1000):Method(CBN)	0.716	0.004	0.707	0.724
S.Size(100):Method(CBN-A)	−0.570	0.005	−0.579	−0.561
S.Size(1000):Method(CBN-A)	0.669	0.004	0.661	0.677
S.Size(100):Method(OT)	−0.529	0.005	−0.538	−0.520
S.Size(1000):Method(OT)	0.630	0.004	0.622	0.638
S.Size(100):Method(OT-A)	−0.554	0.005	−0.563	−0.545
S.Size(1000):Method(OT-A)	0.655	0.004	0.646	0.663
S.Size(100):Method(DiP)	0.988	0.010	0.969	1.008
S.Size(1000):Method(DiP)	−1.346	0.007	−1.360	−1.332
S.Size(100):S.Time(last)	0.060	0.004	0.051	0.069
S.Size(1000):S.Time(last)	−0.065	0.004	−0.074	−0.057
S.Size(100):S.Type(singleC)	−0.025	0.006	−0.037	−0.013
S.Size(1000):S.Type(singleC)	0.016	0.006	0.003	0.028
S.Size(100):S.Type(wholeT_0.01)	0.040	0.006	0.028	0.052
S.Size(1000):S.Type(wholeT_0.01)	−0.020	0.006	−0.032	−0.008
S.Size(100):Model(Bozic)	−0.057	0.008	−0.073	−0.042
S.Size(1000):Model(Bozic)	0.058	0.008	0.042	0.073
S.Size(100):Model(McF_4)	0.043	0.007	0.028	0.058
S.Size(1000):Model(McF_4)	−0.046	0.007	−0.061	−0.032
S.Size(100):Model(McF_6)	0.130	0.007	0.116	0.145
S.Size(1000):Model(McF_6)	−0.109	0.007	−0.124	−0.095
S.Size(100):Conjunction(No)	0.003	0.004	−0.006	0.012
S.Size(1000):Conjunction(No)	−0.019	0.004	−0.027	−0.010
S.Size(100):sh(0)	−0.079	0.004	−0.088	−0.070
S.Size(1000):sh(0)	0.125	0.004	0.116	0.134
S.Size(100):NumNodes(7)	0.052	0.006	0.039	0.064
S.Size(1000):NumNodes(7)	−0.060	0.006	−0.073	−0.048
S.Size(100):NumNodes(9)	−0.036	0.006	−0.048	−0.023
S.Size(1000):NumNodes(9)	0.041	0.006	0.028	0.053
Filter(J5):Method(CBN)	0.002	0.006	−0.011	0.014
Filter(J1):Method(CBN)	0.005	0.005	−0.006	0.015
Filter(S5):Method(CBN)	0.047	0.005	0.037	0.057

Table 44: *(continued)*

Coefficient	mean	sd	0.025 quant.	0.975 quant.
Filter(J5):Method(CBN-A)	−0.137	0.006	−0.149	−0.125
Filter(J1):Method(CBN-A)	0.030	0.005	0.019	0.040
Filter(S5):Method(CBN-A)	0.112	0.005	0.102	0.122
Filter(J5):Method(OT)	0.048	0.006	0.036	0.060
Filter(J1):Method(OT)	0.045	0.005	0.035	0.055
Filter(S5):Method(OT)	0.014	0.005	0.005	0.024
Filter(J5):Method(OT-A)	0.189	0.006	0.177	0.200
Filter(J1):Method(OT-A)	0.095	0.005	0.085	0.105
Filter(S5):Method(OT-A)	−0.036	0.005	−0.046	−0.026
Filter(J5):Method(DiP)	−0.062	0.010	−0.081	−0.043
Filter(J1):Method(DiP)	−0.097	0.008	−0.113	−0.081
Filter(S5):Method(DiP)	−0.082	0.007	−0.096	−0.067
Filter(J5):S.Time(last)	−0.299	0.003	−0.305	−0.293
Filter(J1):S.Time(last)	0.112	0.003	0.107	0.117
Filter(S5):S.Time(last)	−0.094	0.002	−0.098	−0.089
Filter(J5):S.Type(singleC)	0.049	0.004	0.041	0.057
Filter(J1):S.Type(singleC)	0.024	0.004	0.017	0.031
Filter(S5):S.Type(singleC)	0.029	0.003	0.023	0.036
Filter(J5):S.Type(wholeT_0.01)	−0.103	0.004	−0.111	−0.095
Filter(J1):S.Type(wholeT_0.01)	−0.042	0.003	−0.049	−0.036
Filter(S5):S.Type(wholeT_0.01)	−0.082	0.003	−0.089	−0.076
Filter(J5):Model(Bozic)	−0.115	0.006	−0.127	−0.102
Filter(J1):Model(Bozic)	−0.112	0.005	−0.122	−0.102
Filter(S5):Model(Bozic)	0.169	0.005	0.159	0.179
Filter(J5):Model(McF_4)	0.001	0.005	−0.009	0.012
Filter(J1):Model(McF_4)	0.244	0.004	0.235	0.252
Filter(S5):Model(McF_4)	−0.171	0.004	−0.179	−0.163
Filter(J5):Model(McF_6)	−0.217	0.005	−0.226	−0.207
Filter(J1):Model(McF_6)	0.121	0.004	0.114	0.129
Filter(S5):Model(McF_6)	−0.123	0.004	−0.131	−0.115
Filter(J5):Conjunction(No)	0.146	0.003	0.140	0.151
Filter(J1):Conjunction(No)	0.060	0.002	0.055	0.065
Filter(S5):Conjunction(No)	−0.062	0.002	−0.066	−0.057
Filter(J5):sh(0)	0.115	0.003	0.109	0.121
Filter(J1):sh(0)	0.025	0.003	0.020	0.030
Filter(S5):sh(0)	0.020	0.002	0.015	0.024
Filter(J5):NumNodes(7)	−0.462	0.004	−0.471	−0.454
Filter(J1):NumNodes(7)	−0.177	0.004	−0.184	−0.170
Filter(S5):NumNodes(7)	0.165	0.004	0.159	0.172
Filter(J5):NumNodes(9)	0.230	0.004	0.221	0.239
Filter(J1):NumNodes(9)	0.050	0.004	0.042	0.057
Filter(S5):NumNodes(9)	−0.088	0.003	−0.095	−0.081
Method(CBN):S.Time(last)	0.101	0.003	0.095	0.107
Method(CBN-A):S.Time(last)	0.155	0.003	0.149	0.161
Method(OT):S.Time(last)	0.197	0.003	0.191	0.202
Method(OT-A):S.Time(last)	−0.295	0.003	−0.301	−0.289
Method(DiP):S.Time(last)	0.124	0.004	0.115	0.133
Method(CBN):S.Type(singleC)	−0.057	0.004	−0.065	−0.049
Method(CBN-A):S.Type(singleC)	−0.055	0.004	−0.064	−0.047
Method(OT):S.Type(singleC)	−0.043	0.004	−0.052	−0.035
Method(OT-A):S.Type(singleC)	−0.014	0.004	−0.023	−0.006
Method(DiP):S.Type(singleC)	0.067	0.006	0.054	0.080
Method(CBN):S.Type(wholeT_0.01)	0.139	0.004	0.131	0.147
Method(CBN-A):S.Type(wholeT_0.01)	0.116	0.004	0.108	0.124
Method(OT):S.Type(wholeT_0.01)	0.091	0.004	0.083	0.099
Method(OT-A):S.Type(wholeT_0.01)	0.033	0.004	0.025	0.041
Method(DiP):S.Type(wholeT_0.01)	−0.147	0.006	−0.159	−0.136
Method(CBN):Model(Bozic)	−0.215	0.006	−0.226	−0.203
Method(CBN-A):Model(Bozic)	−0.140	0.006	−0.151	−0.128
Method(OT):Model(Bozic)	0.035	0.006	0.023	0.046
Method(OT-A):Model(Bozic)	0.152	0.006	0.141	0.164
Method(DiP):Model(Bozic)	0.058	0.010	0.038	0.079

Table 44: *(continued)*

Coefficient	mean	sd	0.025 quant.	0.975 quant.
Method(CBN):Model(McF_4)	0.179	0.005	0.169	0.189
Method(CBN-A):Model(McF_4)	0.244	0.005	0.234	0.254
Method(OT):Model(McF_4)	−0.030	0.005	−0.040	−0.020
Method(OT-A):Model(McF_4)	0.008	0.005	−0.001	0.018
Method(DiP):Model(McF_4)	−0.182	0.008	−0.198	−0.167
Method(CBN):Model(McF_6)	0.390	0.005	0.381	0.400
Method(CBN-A):Model(McF_6)	0.267	0.005	0.257	0.276
Method(OT):Model(McF_6)	0.112	0.005	0.102	0.121
Method(OT-A):Model(McF_6)	−0.200	0.005	−0.210	−0.190
Method(DiP):Model(McF_6)	−0.031	0.008	−0.046	−0.016
Method(CBN):Conjunction(No)	0.096	0.003	0.090	0.102
Method(CBN-A):Conjunction(No)	0.092	0.003	0.086	0.097
Method(OT):Conjunction(No)	−0.043	0.003	−0.049	−0.037
Method(OT-A):Conjunction(No)	−0.141	0.003	−0.146	−0.135
Method(DiP):Conjunction(No)	−0.002	0.004	−0.010	0.007
Method(CBN):sh(0)	0.101	0.003	0.095	0.107
Method(CBN-A):sh(0)	0.129	0.003	0.123	0.134
Method(OT):sh(0)	0.121	0.003	0.115	0.127
Method(OT-A):sh(0)	0.139	0.003	0.134	0.145
Method(DiP):sh(0)	−0.346	0.004	−0.355	−0.337
Method(CBN):NumNodes(7)	0.012	0.004	0.003	0.021
Method(CBN-A):NumNodes(7)	−0.293	0.004	−0.302	−0.285
Method(OT):NumNodes(7)	0.302	0.004	0.293	0.311
Method(OT-A):NumNodes(7)	−0.620	0.004	−0.628	−0.611
Method(DiP):NumNodes(7)	0.864	0.008	0.849	0.879
Method(CBN):NumNodes(9)	−0.042	0.004	−0.051	−0.034
Method(CBN-A):NumNodes(9)	0.120	0.004	0.111	0.129
Method(OT):NumNodes(9)	−0.054	0.004	−0.062	−0.045
Method(OT-A):NumNodes(9)	0.275	0.004	0.266	0.283
Method(DiP):NumNodes(9)	−0.382	0.007	−0.395	−0.369
S.Time(last):S.Type(singleC)	−0.029	0.004	−0.038	−0.021
S.Time(last):S.Type(wholeT_0.01)	0.082	0.004	0.074	0.091
S.Time(last):Model(Bozic)	−0.071	0.006	−0.082	−0.061
S.Time(last):Model(McF_4)	0.052	0.005	0.041	0.062
S.Time(last):Model(McF_6)	0.324	0.005	0.314	0.334
S.Time(last):Conjunction(No)	−0.019	0.003	−0.025	−0.013
S.Time(last):sh(0)	−0.031	0.003	−0.037	−0.025
S.Time(last):NumNodes(7)	0.366	0.004	0.358	0.375
S.Time(last):NumNodes(9)	−0.134	0.005	−0.143	−0.126
S.Type(singleC):Model(Bozic)	0.113	0.008	0.098	0.129
S.Type(wholeT_0.01):Model(Bozic)	−0.336	0.008	−0.351	−0.321
S.Type(singleC):Model(McF_4)	−0.096	0.007	−0.111	−0.081
S.Type(wholeT_0.01):Model(McF_4)	0.272	0.007	0.258	0.286
S.Type(singleC):Model(McF_6)	−0.194	0.007	−0.208	−0.179
S.Type(wholeT_0.01):Model(McF_6)	0.469	0.007	0.454	0.483
S.Type(singleC):Conjunction(No)	0.015	0.004	0.006	0.023
S.Type(wholeT_0.01):Conjunction(No)	−0.032	0.004	−0.040	−0.023
S.Type(singleC):sh(0)	0.055	0.004	0.047	0.064
S.Type(wholeT_0.01):sh(0)	−0.179	0.004	−0.188	−0.171
S.Type(singleC):NumNodes(7)	−0.054	0.006	−0.066	−0.042
S.Type(wholeT_0.01):NumNodes(7)	0.098	0.006	0.086	0.110
S.Type(singleC):NumNodes(9)	0.045	0.006	0.033	0.058
S.Type(wholeT_0.01):NumNodes(9)	−0.088	0.006	−0.100	−0.076
Model(Bozic):Conjunction(No)	0.048	0.006	0.037	0.059
Model(McF_4):Conjunction(No)	−0.084	0.005	−0.094	−0.074
Model(McF_6):Conjunction(No)	−0.067	0.005	−0.077	−0.057
Model(Bozic):sh(0)	0.214	0.006	0.203	0.225
Model(McF_4):sh(0)	−0.415	0.005	−0.425	−0.405
Model(McF_6):sh(0)	−0.422	0.005	−0.432	−0.411
Model(Bozic):NumNodes(7)	−0.245	0.008	−0.261	−0.230
Model(McF_4):NumNodes(7)	0.249	0.007	0.234	0.264
Model(McF_6):NumNodes(7)	0.306	0.007	0.291	0.320

Table 44: *(continued)*

Coefficient	mean	sd	0.025 quant.	0.975 quant.
Model(Bozic):NumNodes(9)	0.117	0.008	0.101	0.133
Model(McF_4):NumNodes(9)	−0.195	0.008	−0.210	−0.180
Model(McF_6):NumNodes(9)	−0.145	0.007	−0.159	−0.130
Conjunction(No):sh(0)	0.019	0.003	0.013	0.025
Conjunction(No):NumNodes(7)	−0.073	0.004	−0.082	−0.064
Conjunction(No):NumNodes(9)	−0.128	0.004	−0.137	−0.119
sh(0):NumNodes(7)	−0.082	0.004	−0.091	−0.073
sh(0):NumNodes(9)	0.060	0.005	0.052	0.069

5.2.4 Fit for FPF, Drivers Unknown

Table 45: Model fit for performance measure 'FPF' when there are passengers.

Coefficient	mean	sd	0.025 quant.	0.975 quant.
(Intercept)	−8.314	0.005	−8.323	−8.304
S.Size(100)	0.432	0.006	0.421	0.443
S.Size(1000)	−0.502	0.006	−0.513	−0.490
Filter(J5)	−1.278	0.006	−1.290	−1.266
Filter(J1)	−0.181	0.004	−0.189	−0.174
Filter(S5)	−0.262	0.004	−0.270	−0.254
Method(CBN)	1.381	0.004	1.374	1.388
Method(CBN-A)	1.309	0.004	1.302	1.317
Method(OT)	−0.236	0.006	−0.248	−0.224
Method(OT-A)	0.185	0.005	0.176	0.195
Method(DiP)	−1.751	0.010	−1.772	−1.731
S.Time(last)	0.427	0.004	0.419	0.435
S.Type(singleC)	−0.494	0.006	−0.506	−0.482
S.Type(wholeT_0.01)	1.096	0.006	1.085	1.107
Model(Bozic)	0.315	0.007	0.302	0.329
Model(McF_4)	−0.339	0.008	−0.354	−0.324
Model(McF_6)	0.753	0.007	0.739	0.766
Conjunction(No)	−0.006	0.004	−0.014	0.002
sh(0)	−0.631	0.004	−0.639	−0.622
NumNodes(7)	0.112	0.006	0.101	0.124
NumNodes(9)	0.038	0.006	0.027	0.049
S.Size(100):Filter(J5)	−0.177	0.005	−0.186	−0.168
S.Size(1000):Filter(J5)	0.191	0.005	0.182	0.201
S.Size(100):Filter(J1)	0.399	0.003	0.393	0.405
S.Size(1000):Filter(J1)	−0.320	0.004	−0.328	−0.313
S.Size(100):Filter(S5)	−0.106	0.003	−0.112	−0.100
S.Size(1000):Filter(S5)	0.092	0.003	0.085	0.098
S.Size(100):Method(CBN)	0.126	0.003	0.121	0.131
S.Size(1000):Method(CBN)	−0.081	0.003	−0.087	−0.076
S.Size(100):Method(CBN-A)	0.145	0.003	0.140	0.150
S.Size(1000):Method(CBN-A)	−0.104	0.003	−0.109	−0.098
S.Size(100):Method(OT)	0.187	0.004	0.180	0.194
S.Size(1000):Method(OT)	−0.211	0.004	−0.219	−0.202
S.Size(100):Method(OT-A)	0.163	0.003	0.156	0.169
S.Size(1000):Method(OT-A)	−0.162	0.004	−0.170	−0.155
S.Size(100):Method(DiP)	−0.263	0.007	−0.276	−0.250
S.Size(1000):Method(DiP)	0.269	0.007	0.256	0.282
S.Size(100):S.Time(last)	−0.190	0.005	−0.200	−0.180
S.Size(1000):S.Time(last)	0.219	0.005	0.209	0.229
S.Size(100):S.Type(singleC)	0.103	0.007	0.089	0.117
S.Size(1000):S.Type(singleC)	−0.175	0.008	−0.189	−0.160
S.Size(100):S.Type(wholeT_0.01)	−0.240	0.007	−0.254	−0.227
S.Size(1000):S.Type(wholeT_0.01)	0.333	0.007	0.319	0.347
S.Size(100):Model(Bozic)	0.092	0.009	0.075	0.108
S.Size(1000):Model(Bozic)	−0.020	0.009	−0.038	−0.002
S.Size(100):Model(McF_4)	−0.004	0.009	−0.020	0.013
S.Size(1000):Model(McF_4)	−0.070	0.009	−0.087	−0.053
S.Size(100):Model(McF_6)	−0.075	0.008	−0.091	−0.058
S.Size(1000):Model(McF_6)	−0.078	0.009	−0.096	−0.061
S.Size(100):Conjunction(No)	−0.012	0.005	−0.021	−0.002
S.Size(1000):Conjunction(No)	0.018	0.005	0.008	0.029
S.Size(100):sh(0)	0.062	0.005	0.052	0.071
S.Size(1000):sh(0)	−0.023	0.005	−0.034	−0.013
S.Size(100):NumNodes(7)	0.148	0.007	0.134	0.162
S.Size(1000):NumNodes(7)	−0.197	0.008	−0.212	−0.182
S.Size(100):NumNodes(9)	−0.062	0.007	−0.075	−0.048
S.Size(1000):NumNodes(9)	0.081	0.007	0.067	0.096
Filter(J5):Method(CBN)	−0.002	0.007	−0.015	0.012
Filter(J1):Method(CBN)	−0.179	0.004	−0.187	−0.170
Filter(S5):Method(CBN)	0.036	0.004	0.027	0.044

Table 45: *(continued)*

Coefficient	mean	sd	0.025 quant.	0.975 quant.
Filter(J5):Method(CBN-A)	0.062	0.007	0.049	0.076
Filter(J1):Method(CBN-A)	−0.170	0.004	−0.179	−0.162
Filter(S5):Method(CBN-A)	−0.017	0.005	−0.026	−0.008
Filter(J5):Method(OT)	−0.559	0.014	−0.586	−0.532
Filter(J1):Method(OT)	0.229	0.007	0.216	0.243
Filter(S5):Method(OT)	−0.089	0.008	−0.104	−0.073
Filter(J5):Method(OT-A)	−0.151	0.009	−0.170	−0.133
Filter(J1):Method(OT-A)	0.076	0.006	0.065	0.087
Filter(S5):Method(OT-A)	−0.119	0.006	−0.131	−0.106
Filter(J5):Method(DiP)	0.219	0.017	0.186	0.251
Filter(J1):Method(DiP)	0.032	0.010	0.014	0.051
Filter(S5):Method(DiP)	0.092	0.011	0.071	0.113
Filter(J5):S.Time(last)	0.075	0.004	0.067	0.082
Filter(J1):S.Time(last)	−0.038	0.002	−0.043	−0.034
Filter(S5):S.Time(last)	0.284	0.003	0.279	0.289
Filter(J5):S.Type(singleC)	0.016	0.005	0.005	0.026
Filter(J1):S.Type(singleC)	−0.154	0.004	−0.161	−0.146
Filter(S5):S.Type(singleC)	−0.054	0.004	−0.062	−0.047
Filter(J5):S.Type(wholeT_0.01)	−0.048	0.005	−0.057	−0.039
Filter(J1):S.Type(wholeT_0.01)	0.321	0.003	0.315	0.327
Filter(S5):S.Type(wholeT_0.01)	0.091	0.003	0.085	0.098
Filter(J5):Model(Bozic)	−0.273	0.007	−0.285	−0.260
Filter(J1):Model(Bozic)	0.223	0.004	0.216	0.230
Filter(S5):Model(Bozic)	0.140	0.004	0.132	0.147
Filter(J5):Model(McF_4)	0.136	0.007	0.123	0.150
Filter(J1):Model(McF_4)	−0.342	0.005	−0.352	−0.333
Filter(S5):Model(McF_4)	−0.096	0.005	−0.105	−0.087
Filter(J5):Model(McF_6)	0.445	0.005	0.434	0.455
Filter(J1):Model(McF_6)	−0.044	0.004	−0.051	−0.037
Filter(S5):Model(McF_6)	−0.105	0.004	−0.113	−0.098
Filter(J5):Conjunction(No)	−0.029	0.003	−0.036	−0.023
Filter(J1):Conjunction(No)	−0.010	0.002	−0.014	−0.006
Filter(S5):Conjunction(No)	0.048	0.002	0.044	0.053
Filter(J5):sh(0)	0.027	0.004	0.020	0.034
Filter(J1):sh(0)	−0.115	0.003	−0.120	−0.110
Filter(S5):sh(0)	0.007	0.003	0.002	0.012
Filter(J5):NumNodes(7)	0.033	0.005	0.024	0.043
Filter(J1):NumNodes(7)	−0.001	0.003	−0.007	0.006
Filter(S5):NumNodes(7)	−0.246	0.004	−0.253	−0.239
Filter(J5):NumNodes(9)	0.066	0.005	0.057	0.075
Filter(J1):NumNodes(9)	−0.036	0.003	−0.042	−0.030
Filter(S5):NumNodes(9)	0.022	0.003	0.015	0.028
Method(CBN):S.Time(last)	0.220	0.002	0.216	0.224
Method(CBN-A):S.Time(last)	0.179	0.002	0.175	0.183
Method(OT):S.Time(last)	−0.023	0.003	−0.028	−0.018
Method(OT-A):S.Time(last)	0.153	0.003	0.148	0.158
Method(DiP):S.Time(last)	−0.483	0.005	−0.494	−0.473
Method(CBN):S.Type(singleC)	0.148	0.003	0.142	0.155
Method(CBN-A):S.Type(singleC)	0.154	0.003	0.147	0.161
Method(OT):S.Type(singleC)	0.002	0.005	−0.007	0.011
Method(OT-A):S.Type(singleC)	0.026	0.004	0.017	0.034
Method(DiP):S.Type(singleC)	−0.196	0.011	−0.218	−0.174
Method(CBN):S.Type(wholeT_0.01)	−0.291	0.003	−0.297	−0.286
Method(CBN-A):S.Type(wholeT_0.01)	−0.323	0.003	−0.328	−0.317
Method(OT):S.Type(wholeT_0.01)	−0.016	0.004	−0.023	−0.008
Method(OT-A):S.Type(wholeT_0.01)	−0.056	0.004	−0.063	−0.049
Method(DiP):S.Type(wholeT_0.01)	0.425	0.008	0.409	0.441
Method(CBN):Model(Bozic)	−0.263	0.003	−0.269	−0.256
Method(CBN-A):Model(Bozic)	−0.260	0.003	−0.266	−0.253
Method(OT):Model(Bozic)	−0.065	0.004	−0.073	−0.056
Method(OT-A):Model(Bozic)	−0.178	0.004	−0.186	−0.170
Method(DiP):Model(Bozic)	0.534	0.008	0.519	0.550

Table 45: *(continued)*

Coefficient	mean	sd	0.025 quant.	0.975 quant.
Method(CBN):Model(McF_4)	0.540	0.005	0.531	0.550
Method(CBN-A):Model(McF_4)	0.527	0.005	0.518	0.536
Method(OT):Model(McF_4)	0.261	0.006	0.249	0.273
Method(OT-A):Model(McF_4)	0.203	0.006	0.192	0.215
Method(DiP):Model(McF_4)	−0.583	0.015	−0.612	−0.553
Method(CBN):Model(McF_6)	−0.104	0.004	−0.112	−0.097
Method(CBN-A):Model(McF_6)	−0.139	0.004	−0.146	−0.131
Method(OT):Model(McF_6)	−0.169	0.005	−0.179	−0.159
Method(OT-A):Model(McF_6)	0.147	0.004	0.138	0.156
Method(DiP):Model(McF_6)	−0.395	0.011	−0.418	−0.373
Method(CBN):Conjunction(No)	0.022	0.002	0.019	0.026
Method(CBN-A):Conjunction(No)	0.012	0.002	0.009	0.016
Method(OT):Conjunction(No)	−0.016	0.003	−0.021	−0.010
Method(OT-A):Conjunction(No)	−0.014	0.002	−0.019	−0.009
Method(DiP):Conjunction(No)	0.022	0.005	0.012	0.031
Method(CBN):sh(0)	0.128	0.002	0.124	0.132
Method(CBN-A):sh(0)	0.148	0.002	0.143	0.152
Method(OT):sh(0)	−0.035	0.003	−0.041	−0.028
Method(OT-A):sh(0)	−0.012	0.003	−0.017	−0.006
Method(DiP):sh(0)	−0.181	0.007	−0.194	−0.168
Method(CBN):NumNodes(7)	−0.116	0.003	−0.121	−0.111
Method(CBN-A):NumNodes(7)	−0.095	0.003	−0.101	−0.090
Method(OT):NumNodes(7)	0.104	0.004	0.097	0.112
Method(OT-A):NumNodes(7)	0.116	0.003	0.110	0.123
Method(DiP):NumNodes(7)	0.034	0.007	0.021	0.048
Method(CBN):NumNodes(9)	0.110	0.003	0.105	0.115
Method(CBN-A):NumNodes(9)	0.118	0.003	0.113	0.123
Method(OT):NumNodes(9)	−0.011	0.004	−0.019	−0.004
Method(OT-A):NumNodes(9)	0.002	0.003	−0.005	0.008
Method(DiP):NumNodes(9)	−0.172	0.007	−0.186	−0.158
S.Time(last):S.Type(singleC)	0.132	0.005	0.122	0.142
S.Time(last):S.Type(wholeT_0.01)	−0.301	0.005	−0.311	−0.291
S.Time(last):Model(Bozic)	−0.089	0.006	−0.101	−0.077
S.Time(last):Model(McF_4)	−0.120	0.006	−0.132	−0.108
S.Time(last):Model(McF_6)	0.123	0.006	0.111	0.135
S.Time(last):Conjunction(No)	−0.017	0.004	−0.024	−0.010
S.Time(last):sh(0)	−0.034	0.004	−0.042	−0.027
S.Time(last):NumNodes(7)	−0.019	0.005	−0.029	−0.009
S.Time(last):NumNodes(9)	0.021	0.005	0.011	0.031
S.Type(singleC):Model(Bozic)	−0.057	0.009	−0.074	−0.039
S.Type(wholeT_0.01):Model(Bozic)	0.167	0.009	0.150	0.184
S.Type(singleC):Model(McF_4)	0.018	0.009	0.001	0.035
S.Type(wholeT_0.01):Model(McF_4)	−0.073	0.009	−0.090	−0.056
S.Type(singleC):Model(McF_6)	0.140	0.009	0.123	0.157
S.Type(wholeT_0.01):Model(McF_6)	−0.339	0.008	−0.356	−0.322
S.Type(singleC):Conjunction(No)	0.009	0.005	−0.001	0.019
S.Type(wholeT_0.01):Conjunction(No)	−0.009	0.005	−0.019	0.001
S.Type(singleC):sh(0)	0.000	0.005	−0.010	0.010
S.Type(wholeT_0.01):sh(0)	0.021	0.005	0.011	0.031
S.Type(singleC):NumNodes(7)	−0.091	0.007	−0.106	−0.077
S.Type(wholeT_0.01):NumNodes(7)	0.241	0.007	0.227	0.255
S.Type(singleC):NumNodes(9)	0.024	0.007	0.010	0.038
S.Type(wholeT_0.01):NumNodes(9)	−0.069	0.007	−0.083	−0.055
Model(Bozic):Conjunction(No)	0.003	0.006	−0.009	0.015
Model(McF_4):Conjunction(No)	0.022	0.006	0.010	0.034
Model(McF_6):Conjunction(No)	−0.017	0.006	−0.029	−0.005
Model(Bozic):sh(0)	−0.270	0.006	−0.282	−0.258
Model(McF_4):sh(0)	0.429	0.006	0.417	0.441
Model(McF_6):sh(0)	0.480	0.006	0.468	0.492
Model(Bozic):NumNodes(7)	0.066	0.009	0.048	0.083
Model(McF_4):NumNodes(7)	0.025	0.009	0.007	0.042
Model(McF_6):NumNodes(7)	0.246	0.009	0.229	0.263

Table 45: *(continued)*

Coefficient	mean	sd	0.025 quant.	0.975 quant.
Model(Bozic):NumNodes(9)	−0.030	0.009	−0.047	−0.013
Model(McF_4):NumNodes(9)	−0.020	0.009	−0.036	−0.003
Model(McF_6):NumNodes(9)	−0.127	0.009	−0.144	−0.111
Conjunction(No):sh(0)	0.011	0.004	0.004	0.018
Conjunction(No):NumNodes(7)	−0.015	0.005	−0.025	−0.005
Conjunction(No):NumNodes(9)	0.016	0.005	0.006	0.025
sh(0):NumNodes(7)	−0.079	0.005	−0.090	−0.069
sh(0):NumNodes(9)	0.034	0.005	0.024	0.044

5.2.5 Fit for 'Inferred edges', Drivers Unknown

Table 46: Model fit for performance measure 'Inferred edges' when there are passengers.

Coefficient	mean	sd	0.025 quant.	0.975 quant.
(Intercept)	0.673	0.003	0.668	0.678
S.Size(100)	−0.004	0.003	−0.011	0.002
S.Size(1000)	0.079	0.003	0.073	0.086
Filter(J5)	−1.076	0.003	−1.081	−1.070
Filter(J1)	−0.097	0.002	−0.101	−0.093
Filter(S5)	0.021	0.002	0.017	0.025
Method(CBN)	0.689	0.002	0.685	0.693
Method(CBN-A)	0.704	0.002	0.700	0.709
Method(OT)	0.153	0.002	0.148	0.158
Method(OT-A)	0.406	0.002	0.401	0.410
Method(DiP)	−1.217	0.004	−1.225	−1.209
S.Time(last)	0.301	0.002	0.296	0.305
S.Type(singleC)	−0.279	0.003	−0.286	−0.273
S.Type(wholeT_0.01)	0.653	0.003	0.647	0.659
Model(Bozic)	−0.167	0.004	−0.175	−0.159
Model(McF_4)	0.219	0.004	0.211	0.227
Model(McF_6)	1.042	0.004	1.034	1.049
Conjunction(No)	0.034	0.002	0.029	0.038
sh(0)	−0.395	0.002	−0.399	−0.390
NumNodes(7)	−0.098	0.003	−0.105	−0.092
NumNodes(9)	−0.140	0.003	−0.147	−0.134
S.Size(100):Filter(J5)	−0.114	0.003	−0.119	−0.108
S.Size(1000):Filter(J5)	0.115	0.003	0.110	0.121
S.Size(100):Filter(J1)	0.201	0.002	0.197	0.205
S.Size(1000):Filter(J1)	−0.130	0.002	−0.134	−0.126
S.Size(100):Filter(S5)	−0.078	0.002	−0.082	−0.074
S.Size(1000):Filter(S5)	0.071	0.002	0.067	0.075
S.Size(100):Method(CBN)	0.329	0.002	0.325	0.333
S.Size(1000):Method(CBN)	−0.381	0.002	−0.385	−0.378
S.Size(100):Method(CBN-A)	0.326	0.002	0.322	0.330
S.Size(1000):Method(CBN-A)	−0.376	0.002	−0.380	−0.372
S.Size(100):Method(OT)	0.240	0.002	0.235	0.245
S.Size(1000):Method(OT)	−0.294	0.002	−0.299	−0.289
S.Size(100):Method(OT-A)	0.245	0.002	0.240	0.249
S.Size(1000):Method(OT-A)	−0.294	0.002	−0.298	−0.290
S.Size(100):Method(DiP)	−0.529	0.005	−0.539	−0.519
S.Size(1000):Method(DiP)	0.740	0.004	0.733	0.748
S.Size(100):S.Time(last)	−0.094	0.003	−0.100	−0.088
S.Size(1000):S.Time(last)	0.088	0.003	0.082	0.094
S.Size(100):S.Type(singleC)	0.031	0.004	0.022	0.039
S.Size(1000):S.Type(singleC)	−0.044	0.004	−0.053	−0.035
S.Size(100):S.Type(wholeT_0.01)	−0.078	0.004	−0.086	−0.069
S.Size(1000):S.Type(wholeT_0.01)	0.073	0.004	0.065	0.082
S.Size(100):Model(Bozic)	0.147	0.005	0.137	0.158
S.Size(1000):Model(Bozic)	−0.139	0.005	−0.150	−0.128
S.Size(100):Model(McF_4)	−0.073	0.005	−0.083	−0.063
S.Size(1000):Model(McF_4)	0.063	0.005	0.053	0.073
S.Size(100):Model(McF_6)	−0.161	0.005	−0.171	−0.151
S.Size(1000):Model(McF_6)	0.102	0.005	0.092	0.112
S.Size(100):Conjunction(No)	−0.009	0.003	−0.014	−0.003
S.Size(1000):Conjunction(No)	0.012	0.003	0.006	0.018
S.Size(100):sh(0)	0.038	0.003	0.032	0.044
S.Size(1000):sh(0)	−0.021	0.003	−0.027	−0.015
S.Size(100):NumNodes(7)	0.012	0.004	0.004	0.021
S.Size(1000):NumNodes(7)	−0.019	0.004	−0.028	−0.011
S.Size(100):NumNodes(9)	0.007	0.004	−0.001	0.015
S.Size(1000):NumNodes(9)	−0.005	0.004	−0.014	0.003
Filter(J5):Method(CBN)	−0.140	0.004	−0.148	−0.132
Filter(J1):Method(CBN)	−0.113	0.003	−0.119	−0.108
Filter(S5):Method(CBN)	−0.049	0.003	−0.055	−0.044

Table 46: *(continued)*

Coefficient	mean	sd	0.025 quant.	0.975 quant.
Filter(J5):Method(CBN-A)	−0.013	0.004	−0.020	−0.005
Filter(J1):Method(CBN-A)	−0.119	0.003	−0.125	−0.113
Filter(S5):Method(CBN-A)	−0.098	0.003	−0.104	−0.093
Filter(J5):Method(OT)	−0.050	0.005	−0.059	−0.041
Filter(J1):Method(OT)	0.004	0.003	−0.002	0.011
Filter(S5):Method(OT)	0.008	0.003	0.001	0.014
Filter(J5):Method(OT-A)	0.048	0.004	0.040	0.056
Filter(J1):Method(OT-A)	−0.002	0.003	−0.008	0.004
Filter(S5):Method(OT-A)	−0.008	0.003	−0.014	−0.002
Filter(J5):Method(DiP)	0.000	0.007	−0.014	0.014
Filter(J1):Method(DiP)	0.115	0.005	0.105	0.126
Filter(S5):Method(DiP)	0.094	0.005	0.085	0.104
Filter(J5):S.Time(last)	0.154	0.002	0.150	0.158
Filter(J1):S.Time(last)	−0.054	0.001	−0.057	−0.052
Filter(S5):S.Time(last)	0.095	0.001	0.092	0.098
Filter(J5):S.Type(singleC)	−0.006	0.003	−0.012	−0.001
Filter(J1):S.Type(singleC)	−0.086	0.002	−0.091	−0.082
Filter(S5):S.Type(singleC)	−0.008	0.002	−0.012	−0.004
Filter(J5):S.Type(wholeT_0.01)	0.009	0.003	0.004	0.014
Filter(J1):S.Type(wholeT_0.01)	0.169	0.002	0.165	0.173
Filter(S5):S.Type(wholeT_0.01)	0.014	0.002	0.010	0.017
Filter(J5):Model(Bozic)	−0.168	0.004	−0.177	−0.160
Filter(J1):Model(Bozic)	0.154	0.003	0.148	0.159
Filter(S5):Model(Bozic)	−0.034	0.003	−0.039	−0.029
Filter(J5):Model(McF_4)	0.094	0.004	0.086	0.101
Filter(J1):Model(McF_4)	−0.291	0.003	−0.297	−0.285
Filter(S5):Model(McF_4)	0.059	0.003	0.054	0.064
Filter(J5):Model(McF_6)	0.428	0.003	0.422	0.434
Filter(J1):Model(McF_6)	−0.043	0.002	−0.047	−0.038
Filter(S5):Model(McF_6)	0.006	0.002	0.002	0.010
Filter(J5):Conjunction(No)	−0.019	0.002	−0.023	−0.016
Filter(J1):Conjunction(No)	−0.008	0.001	−0.011	−0.005
Filter(S5):Conjunction(No)	0.041	0.001	0.038	0.043
Filter(J5):sh(0)	−0.010	0.002	−0.013	−0.006
Filter(J1):sh(0)	−0.053	0.002	−0.056	−0.050
Filter(S5):sh(0)	0.013	0.001	0.010	0.016
Filter(J5):NumNodes(7)	0.208	0.003	0.203	0.213
Filter(J1):NumNodes(7)	0.018	0.002	0.014	0.022
Filter(S5):NumNodes(7)	−0.192	0.002	−0.196	−0.188
Filter(J5):NumNodes(9)	−0.111	0.003	−0.117	−0.106
Filter(J1):NumNodes(9)	−0.014	0.002	−0.018	−0.010
Filter(S5):NumNodes(9)	0.052	0.002	0.048	0.056
Method(CBN):S.Time(last)	0.133	0.001	0.131	0.136
Method(CBN-A):S.Time(last)	0.083	0.001	0.080	0.085
Method(OT):S.Time(last)	−0.107	0.002	−0.110	−0.104
Method(OT-A):S.Time(last)	0.111	0.002	0.108	0.114
Method(DiP):S.Time(last)	−0.260	0.003	−0.265	−0.255
Method(CBN):S.Type(singleC)	0.052	0.002	0.048	0.056
Method(CBN-A):S.Type(singleC)	0.062	0.002	0.058	0.066
Method(OT):S.Type(singleC)	0.015	0.003	0.010	0.020
Method(OT-A):S.Type(singleC)	0.024	0.002	0.020	0.029
Method(DiP):S.Type(singleC)	−0.081	0.004	−0.090	−0.072
Method(CBN):S.Type(wholeT_0.01)	−0.097	0.002	−0.100	−0.093
Method(CBN-A):S.Type(wholeT_0.01)	−0.126	0.002	−0.130	−0.122
Method(OT):S.Type(wholeT_0.01)	−0.035	0.002	−0.039	−0.031
Method(OT-A):S.Type(wholeT_0.01)	−0.055	0.002	−0.059	−0.051
Method(DiP):S.Type(wholeT_0.01)	0.166	0.004	0.159	0.174
Method(CBN):Model(Bozic)	0.009	0.002	0.004	0.014
Method(CBN-A):Model(Bozic)	−0.011	0.003	−0.015	−0.006
Method(OT):Model(Bozic)	−0.056	0.003	−0.062	−0.049
Method(OT-A):Model(Bozic)	−0.097	0.003	−0.103	−0.091
Method(DiP):Model(Bozic)	0.139	0.005	0.129	0.149

Table 46: *(continued)*

Coefficient	mean	sd	0.025 quant.	0.975 quant.
Method(CBN):Model(McF_4)	0.082	0.002	0.077	0.086
Method(CBN-A):Model(McF_4)	0.062	0.002	0.057	0.067
Method(OT):Model(McF_4)	0.064	0.003	0.058	0.070
Method(OT-A):Model(McF_4)	0.045	0.003	0.039	0.050
Method(DiP):Model(McF_4)	−0.144	0.005	−0.155	−0.134
Method(CBN):Model(McF_6)	−0.172	0.002	−0.177	−0.168
Method(CBN-A):Model(McF_6)	−0.167	0.002	−0.172	−0.163
Method(OT):Model(McF_6)	−0.019	0.003	−0.024	−0.013
Method(OT-A):Model(McF_6)	0.070	0.002	0.065	0.074
Method(DiP):Model(McF_6)	−0.034	0.004	−0.043	−0.026
Method(CBN):Conjunction(No)	−0.016	0.001	−0.019	−0.014
Method(CBN-A):Conjunction(No)	−0.025	0.001	−0.027	−0.022
Method(OT):Conjunction(No)	0.004	0.002	0.001	0.007
Method(OT-A):Conjunction(No)	0.003	0.001	0.000	0.006
Method(DiP):Conjunction(No)	0.035	0.003	0.030	0.041
Method(CBN):sh(0)	−0.026	0.001	−0.029	−0.023
Method(CBN-A):sh(0)	−0.014	0.001	−0.017	−0.011
Method(OT):sh(0)	−0.063	0.002	−0.066	−0.060
Method(OT-A):sh(0)	−0.052	0.002	−0.055	−0.049
Method(DiP):sh(0)	0.121	0.003	0.115	0.126
Method(CBN):NumNodes(7)	−0.024	0.002	−0.028	−0.020
Method(CBN-A):NumNodes(7)	0.043	0.002	0.039	0.046
Method(OT):NumNodes(7)	−0.028	0.002	−0.033	−0.024
Method(OT-A):NumNodes(7)	0.236	0.002	0.232	0.240
Method(DiP):NumNodes(7)	−0.314	0.004	−0.323	−0.305
Method(CBN):NumNodes(9)	0.083	0.002	0.080	0.087
Method(CBN-A):NumNodes(9)	0.058	0.002	0.054	0.062
Method(OT):NumNodes(9)	−0.032	0.002	−0.037	−0.028
Method(OT-A):NumNodes(9)	−0.097	0.002	−0.101	−0.092
Method(DiP):NumNodes(9)	0.049	0.004	0.041	0.057
S.Time(last):S.Type(singleC)	0.061	0.003	0.055	0.067
S.Time(last):S.Type(wholeT_0.01)	−0.152	0.003	−0.158	−0.146
S.Time(last):Model(Bozic)	0.012	0.004	0.004	0.019
S.Time(last):Model(McF_4)	−0.085	0.004	−0.093	−0.078
S.Time(last):Model(McF_6)	−0.111	0.004	−0.119	−0.104
S.Time(last):Conjunction(No)	−0.005	0.002	−0.009	−0.001
S.Time(last):sh(0)	−0.003	0.002	−0.007	0.001
S.Time(last):NumNodes(7)	−0.128	0.003	−0.134	−0.122
S.Time(last):NumNodes(9)	0.059	0.003	0.053	0.065
S.Type(singleC):Model(Bozic)	−0.101	0.005	−0.112	−0.091
S.Type(wholeT_0.01):Model(Bozic)	0.281	0.005	0.270	0.291
S.Type(singleC):Model(McF_4)	0.075	0.005	0.065	0.085
S.Type(wholeT_0.01):Model(McF_4)	−0.204	0.005	−0.214	−0.194
S.Type(singleC):Model(McF_6)	0.173	0.005	0.163	0.183
S.Type(wholeT_0.01):Model(McF_6)	−0.420	0.005	−0.430	−0.410
S.Type(singleC):Conjunction(No)	0.007	0.003	0.001	0.013
S.Type(wholeT_0.01):Conjunction(No)	−0.009	0.003	−0.015	−0.003
S.Type(singleC):sh(0)	−0.005	0.003	−0.011	0.001
S.Type(wholeT_0.01):sh(0)	0.043	0.003	0.037	0.049
S.Type(singleC):NumNodes(7)	−0.009	0.004	−0.017	0.000
S.Type(wholeT_0.01):NumNodes(7)	0.048	0.004	0.040	0.057
S.Type(singleC):NumNodes(9)	−0.011	0.004	−0.020	−0.002
S.Type(wholeT_0.01):NumNodes(9)	0.010	0.004	0.002	0.018
Model(Bozic):Conjunction(No)	0.002	0.004	−0.005	0.010
Model(McF_4):Conjunction(No)	0.032	0.004	0.025	0.039
Model(McF_6):Conjunction(No)	−0.031	0.004	−0.038	−0.024
Model(Bozic):sh(0)	−0.233	0.004	−0.240	−0.225
Model(McF_4):sh(0)	0.406	0.004	0.399	0.413
Model(McF_6):sh(0)	0.419	0.004	0.412	0.426
Model(Bozic):NumNodes(7)	0.107	0.005	0.096	0.117
Model(McF_4):NumNodes(7)	−0.045	0.005	−0.055	−0.034
Model(McF_6):NumNodes(7)	0.000	0.005	−0.010	0.010

Table 46: *(continued)*

Coefficient	mean	sd	0.025 quant.	0.975 quant.
Model(Bozic):NumNodes(9)	−0.041	0.005	−0.052	−0.031
Model(McF_4):NumNodes(9)	0.026	0.005	0.015	0.036
Model(McF_6):NumNodes(9)	−0.002	0.005	−0.012	0.008
Conjunction(No):sh(0)	0.007	0.002	0.003	0.011
Conjunction(No):NumNodes(7)	−0.005	0.003	−0.011	0.001
Conjunction(No):NumNodes(9)	0.016	0.003	0.010	0.022
sh(0):NumNodes(7)	0.009	0.003	0.003	0.015
sh(0):NumNodes(9)	−0.023	0.003	−0.029	−0.017

6 Choosing augmented alternatives

The tables below show how often the augmented or non-agumented alternatives are better. Using within-data set comparisons for each pair of methods (e.g., DiP and DiP-A) I count a case as better is Diff is smaller. Values in each cell are proportions. They need not sum to 1 over each two columns, as in many cases the Diff were equal.

	At least a gene freq.= 1		No gene freq.= 1	
	Augmented better	Augmented worse	Augmented better	Augmented worse
CBN	0.51	0.40	0.50	0.40
DiP	0.63	0.05	0.16	0.04
OT	0.94	0.06	0.14	0.07

Table 47: Comparison of augmented and non augmented alternatives in the Drivers Known scenario.

	At least a gene freq.= 1		No gene freq.= 1	
	Augmented better	Augmented worse	Augmented better	Augmented worse
CBN	0.48	0.30	0.37	0.26
DiP	0.63	0.05	0.12	0.04
OT	0.87	0.06	0.13	0.07

Table 48: Comparison of augmented and non augmented alternatives when Drivers are Unknown and Filtering = S5.

7 Timings of methods: additional results

Figures 17 to 20 show mean and median execution time of each Method under the different scenarios. Note the y-axis is in log_{10} scale; standard errors are not shown as the standard deviations are of the same order of magnitude, or smaller order of magnitude, as the means (i.e., there is no overlap whatsoever between families of methods).

As can be clearly seen, the OT methods are the fastest, generally by factors of two or three orders of magnitude. The speed differences between methods becomes larger as the number of nodes increases in the Drivers Known scenarios. In the Drivers Unknown scenarios the increase in NumNodes, however, has a much smaller effect, because the need to filter means that the actual nodes used in the reconstruction is often much smaller than the true number of nodes. In addition, the more stringent the criterion (e.g., J5 vs. S1) the smaller the nodes used in the reconstruction.

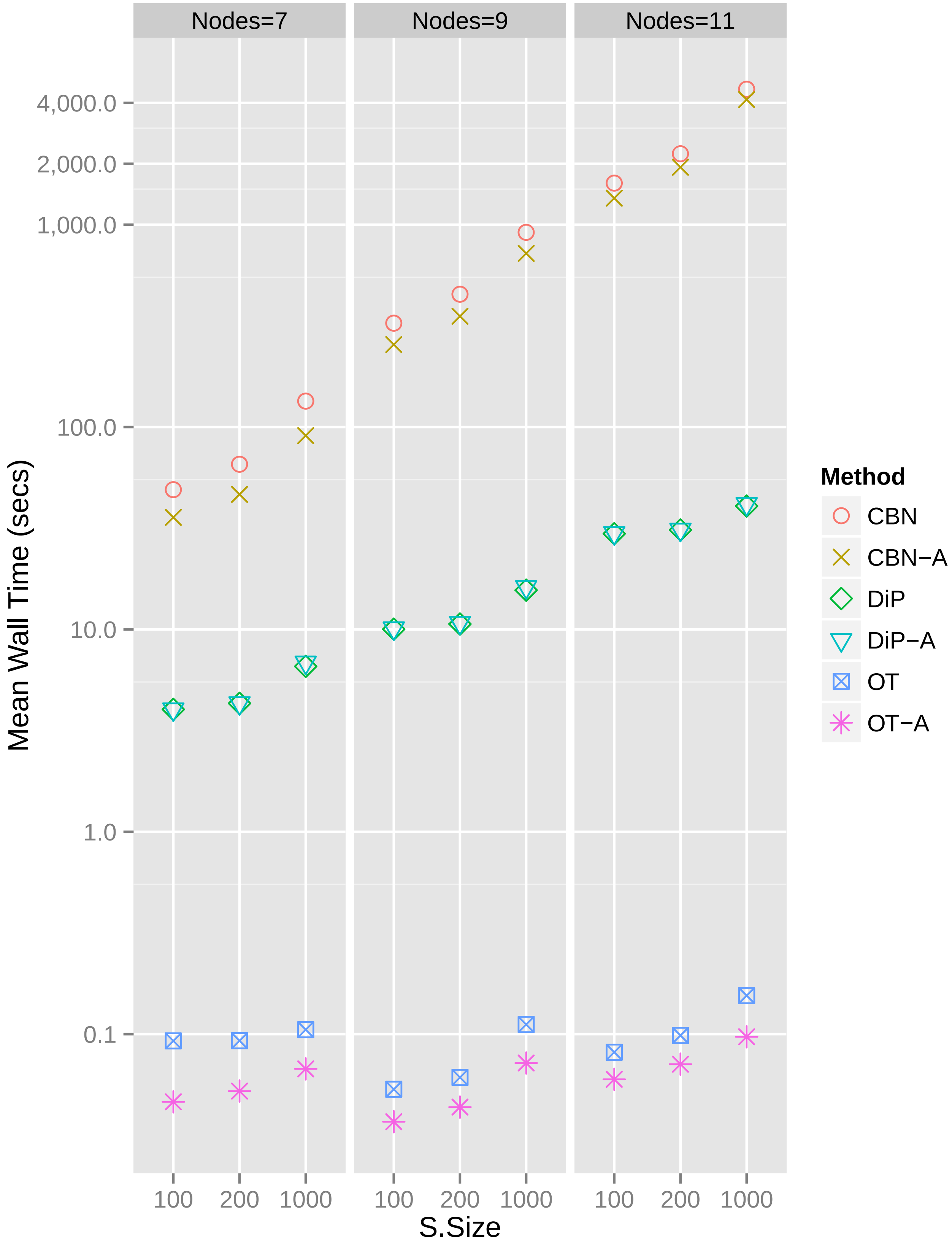

Figure 17: Mean execution time (in seconds) for each method, by S.Size and NumNodes of the original graph, in the Drivers Known scenario.

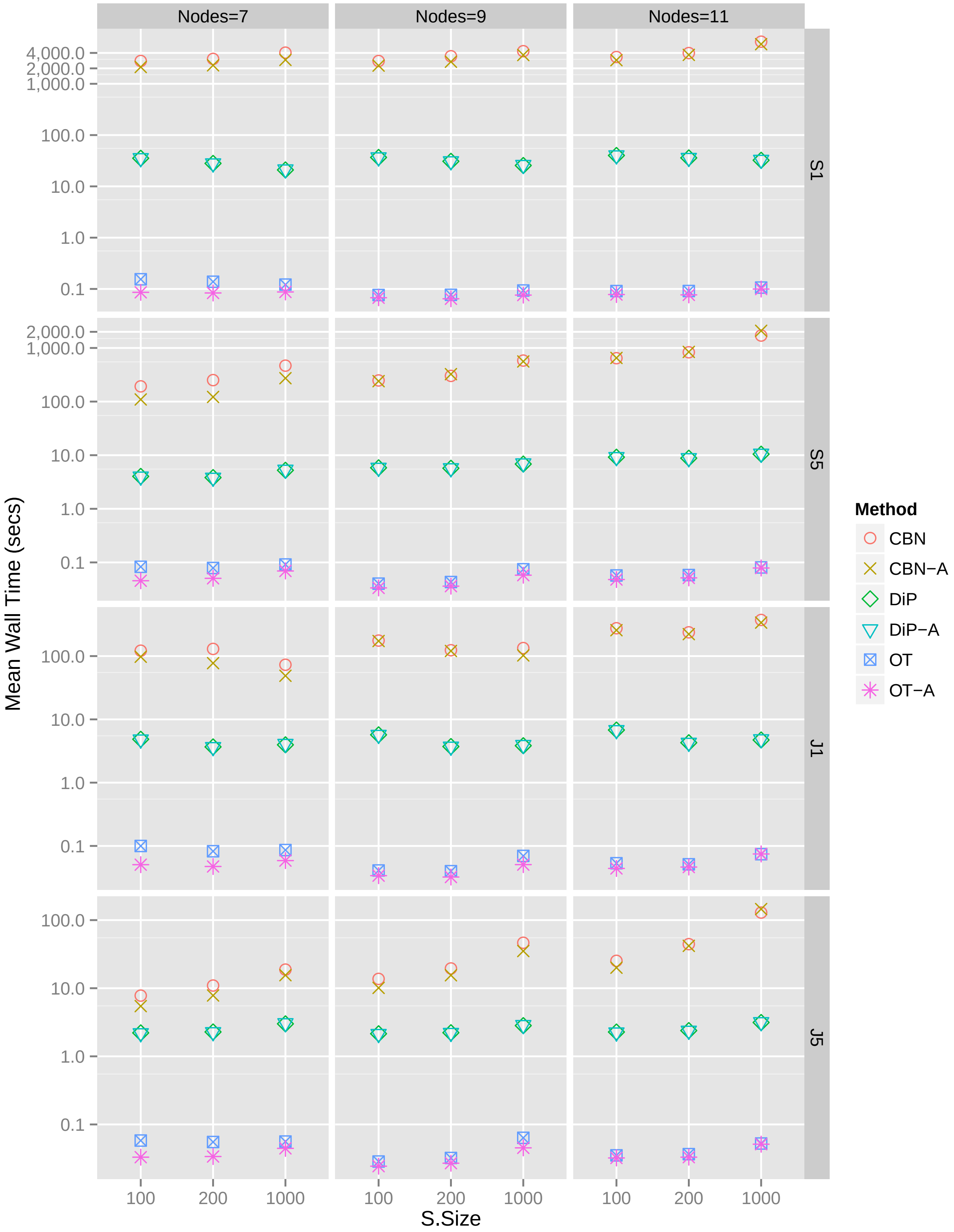

Figure 18: Mean execution time (in seconds) for each method, by S.Size, NumNodes of the original graph, and Filtering, in the Drivers Unknown scenario.

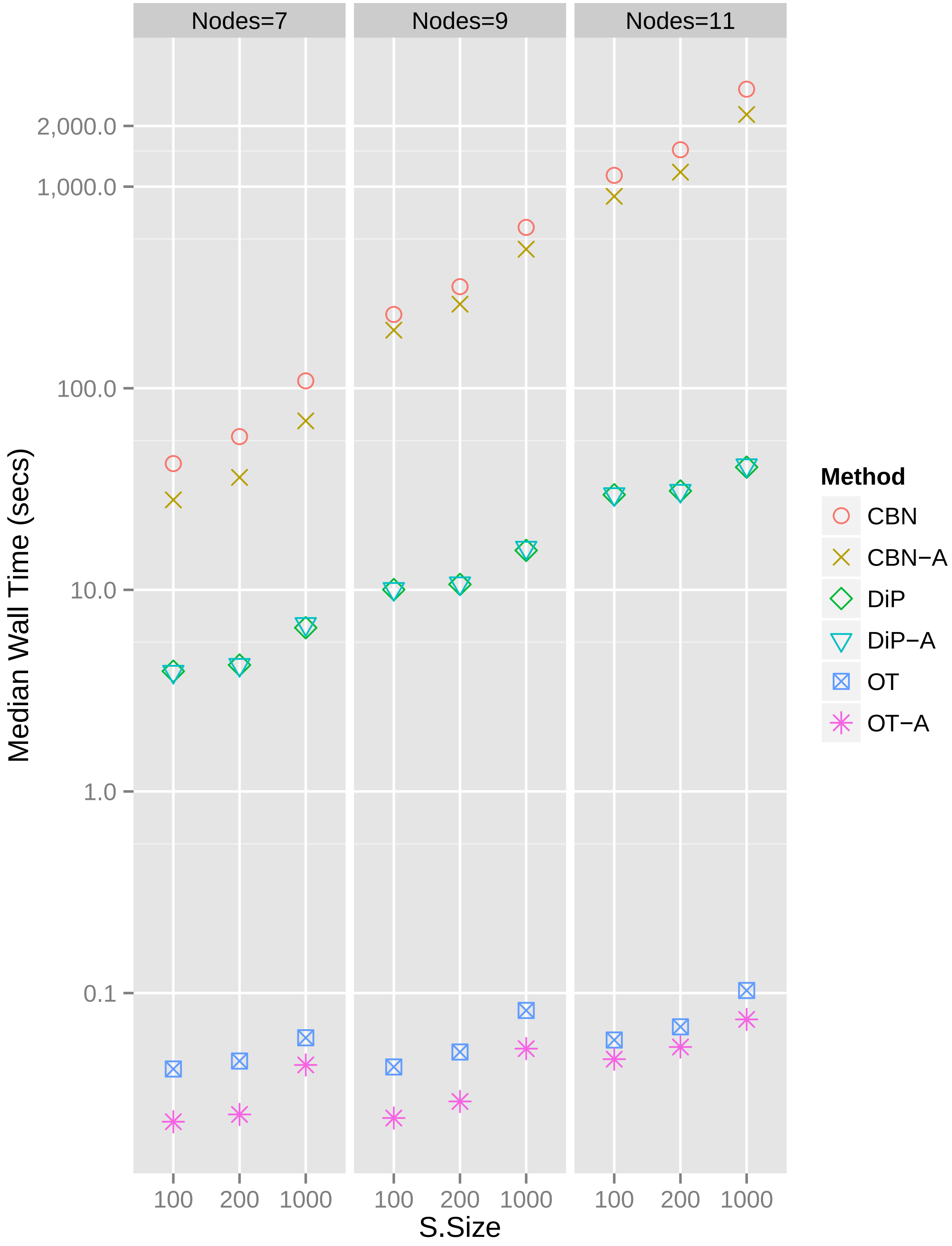

Figure 19: Median execution time (in seconds) for each method, by S.Size and NumNodes of the original graph, in the Drivers Known scenario.

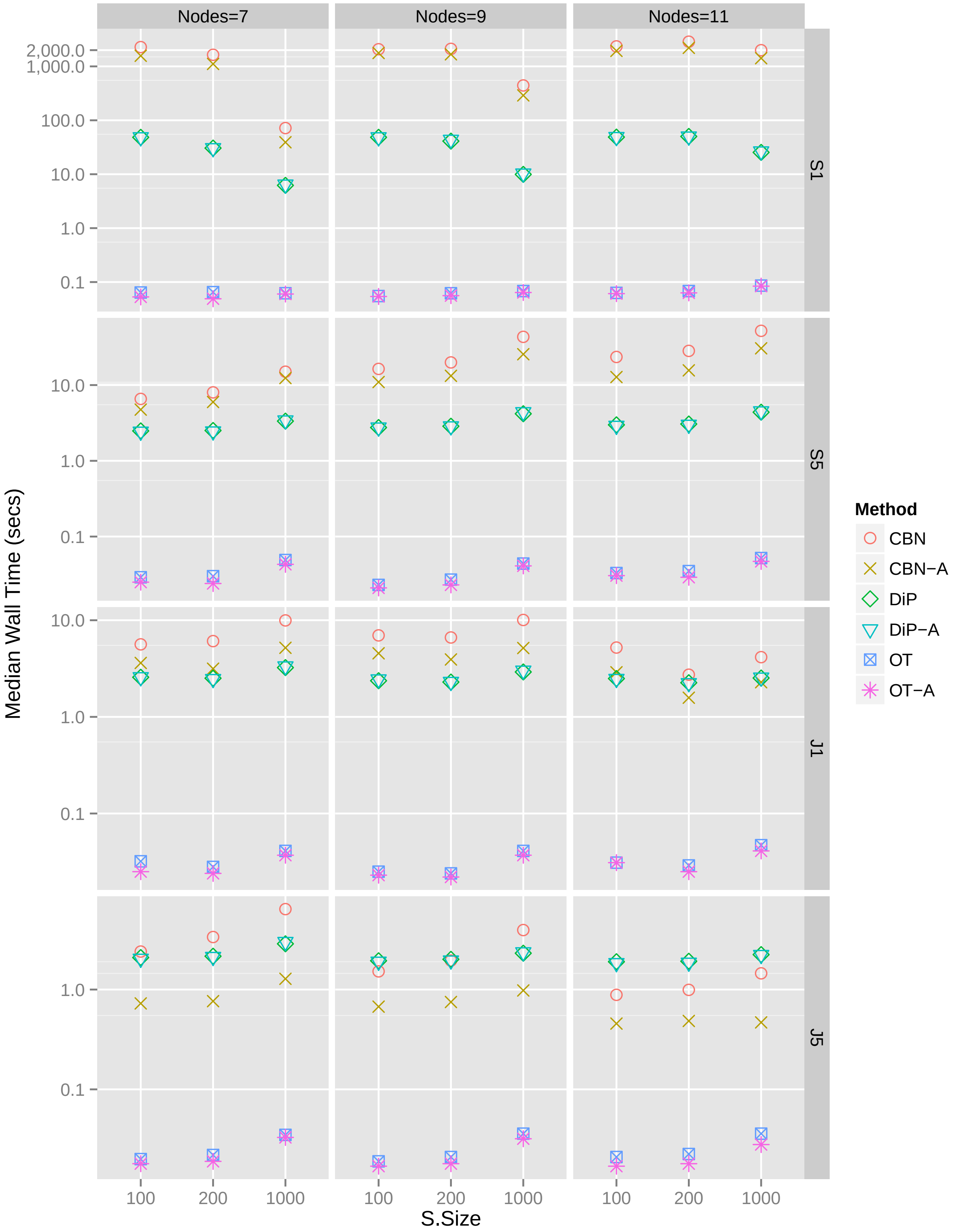

Figure 20: Median execution time (in seconds) for each method, by S.Size, NumNodes of the original graph, and Filtering, in the Drivers Unknown scenario.

7.1 Timing of BCBN

Below I show timings for the BCBN method of Sakoparnig and Beerenwinkel (2012). Complete code to obtain the timings as well as the data used and the output are provided in *Additional file 9* (directory "BCBN-timings").

Briefly, for the three NumNodes (7, 9, 11), and for SSize of 100, 200, and 1000, and always for cases without passengers, I simulated data using Bozic or McF_4, with STime set to "unif" or "last", and using both types of graphs (with and without conjunctions), with a total of five replicates for each combination, so there are a total of 40 cases for each combination of NumNodes and SSize. I then analyzed the data using BCBN, with package version 0.03, also included in *Additional file 9*(this is newer than the one available from the https://www1.ethz.ch/bsse/cbg/software/bayes-cbn, and includes some fixes for bugs that I reported during 2013). Table 49 shows a summary table of the timings. Overall, the median and mean time are 34000 and 40000 seconds (about 9.4 and 11 hours, respectively). Some runs were not completed after 4 days, and some not even after 8 days. In some cases, the procedure crashed irrecoverably (22 cases).

When timing the code, I used two idle machines, each with 64 cores, but launching only at most 15 processes per machine, so as to obtain the fastest possible times by having more cores than the total number of processes run: the code is hard-coded to be parallelized by the author and the runs use between one to four cores simultaneously, depending on the step (four MCMC chains are used).

Table 49: Summary statistics of timings for the BCBN function (in seconds), using a total of 40 replicates per row shown. Those 40 replicates use four different models (Bozic and McF_4, with uniform and last S.Time), and the two graphs for each NumNodes. Not all 40 cases were always completed within 4 days —although some were allowed to run until completion or until crashes in the function.

	NumNodes	SSize	Mean	Median	Min	Max
1	7	100	24372	25628	10260	34764
2	7	200	22897	22219	11748	32854
3	7	1000	26179	28974	9930	43995
4	9	100	29821	33909	11095	37111
5	9	200	27830	33688	12617	36688
6	9	1000	52846	34063	12886	223059
7	11	100	38952	37572	29471	90545
8	11	200	45233	37568	18100	147966
9	11	1000	116548	55192	34142	749946

References

Fox, J. and Weisberg, S. (2011). *An R companion to applied regression, 2nd ed*. Sage, Thousand Oaks, CA.

Martins, T., Simpson, D., Lindgren, F., and Rue, H. (2013). Bayesian computing with INLA: new features. *Computational Statistics & Data Analysis...*, **67**, 68–83.

Rue, H. v., Martino, S., and Chopin, N. (2009). Approximate Bayesian inference for latent Gaussian models by using integrated nested Laplace approximations. *Journal of the Royal Statistical Society: Series B (Statistical Methodology)*, **71**(2), 319–392.

Sakoparnig, T. and Beerenwinkel, N. (2012). Efficient sampling for Bayesian inference of conjunctive Bayesian networks. *Bioinformatics (Oxford, England)*, **28**(18), 2318–24.
