# Supplementary material for: Identifying restrictions in the order of accumulation of mutations during tumor progression: effects of passengers, evolutionary models, and sampling
Source: BMC Bioinformatics. 2015 Feb 12;16:41. doi: 10.1186/s12859-015-0466-7 (PMC4339747; doi:10.1186/s12859-015-0466-7)
Supplement: Additional file 3 — Understanding the different ranking compared to Hainke et al. A detailed examination of the possible reasons for the differences in results concerning OT and CBN between my results and those of Hainke et al. (2012). [file 12859_2015_466_MOESM3_ESM.pdf]

Additional file 3. Understanding the different ranking compared to Hainke et al.

Supplementary material for "Identifying Restrictions in the Order of Accumulation of Mutations during Tumor Progression: Effects of Passengers, Evolutionary Models, and Sampling"

Ramon Diaz-Uriarte
Dept. Biochemistry, Universidad Autónoma de Madrid
Instituto de Investigaciones Biomédicas "Alberto Sols" (UAM-CSIC)
Madrid, Spain

ramon.diaz@iib.uam.es

rdiaz02@gmail.com

http://ligarto.org/rdiaz

Contents

1 Possible reasons for differences in rankings 3

2 Comparison of Diff between Oncotree and Rtreemix 4

3 Difference in Diff between CBN and Oncotree/Rtreemix 7

List of Figures

1 Scatterplot of Diff performance measure computed with Rtreemix and Oncotree for the 5760 samples with Drivers Known and S. Type of single cell. Data have been jittered to minimize superposition of points. 5

2 Boxplots of the within dataset difference in Diff between Rtreemix and Oncotree for the 5760 samples with Drivers Known and S. Type of single cell. Each boxplot represents a total of 60 data sets (three sample sizes * 20 replicates per condition). . 6

3 Boxplots of the within dataset difference in Diff between CBN and Oncotree for the 5760 samples with Drivers Known and S. Type of single cell. Each boxplot represents a total of 60 data sets (three sample sizes * 20 replicates per condition). Values above the red horizontal line mean that CBN performs better. 8

4 Boxplots of the within dataset difference in Diff between CBN and Rtreemix for the 5760 samples with Drivers Known and S. Type of single cell. Each boxplot represents a total of 60 data sets (three sample sizes * 20 replicates per condition). Values above the red horizontal line mean that CBN performs better. 9

5 Boxplots of the within dataset difference in Diff between CBN and the OT-A method (fitted with the Oncotree package, after adding the 0.1 fraction of all zeroes) for the 5760 samples with Drivers Known and S. Type of single cell. Each boxplot represents a total of 60 data sets (three sample sizes * 20 replicates per condition). Values above the red horizontal line mean that CBN performs better. 10

1 Possible reasons for differences in rankings

There are several possible explanations for the differences between my results and those of Hainke *et al.* (2012), especially as reflected in their Table 4, p. 631, where OT is second to CBN in both the graph with conjunctions (last column of their table) and the tree without conjunctions (first column).

Differences in performance measures There are no differences in performance measure since the "ged" used in Hainke *et al.* (2012) is the same as the Diff we use here.

Differences in software for fitting oncogenetic tree models Hainke *et al.* (2012) fit oncogenetic trees using the Rtreemix package (Bogojeska, 2014) instead of Oncotree (Szabo and Pappas, 2013). To examine this difference as a possible cause, I reanalized all the data from the Drivers Known scenario (under the single cell sampling type) with Rtreemix and computed the Diff performance measure. results are shown in figures 1 and 2, in scetion 2. The two methods often, but not always, yield identical results. When they differ, though, there are no systematic deviations where Rtreemix consistently performs worse than Oncotree and, thus, usage of Rtreemix instead of Oncotree is not an explanation. I use the single cell sampling data since those are the ones that are likely to generate patterns of marginal and joint frequencies of mutations that are closest to those that can be obtain from the simulation procedures used in Hainke *et al.* (2012).

Differences in software for fitting CBN models I fitted CBN using the h-cbn program as described in Gerstung *et al.* (2011), which is an implementation of the version of CBN described in Gerstung *et al.* (2009), whereas Hainke *et al.* (2012) used a version of CBN that is no longer available, and that fits the approach described in Beerenwinkel *et al.* (2007) (K. Hainke, pers. comm.). It is possible, though unlikely, that the newer version of CBN shows worse performance (at least regarding Diff) than the older version. If that were the explanation, however, from a user's point of view the relevant results are the ones presented here, since they refer to the currently available software implementation of CBN.

Chance reversal of orderings Hainke *et al.* (2012) use a single graph for each of the scenarios, whereas I use three here, making it more likely that their results can be attributed to a chance reversal of orderings. This is not the most likely explanation, however, since the reversal affects both the CBN and the OT scenario of their Table 4.

Differences in graphs and results in Hainke et al. based on a single graph This is discussed in the ms. As can be seen in the figures in section 3, CBN outperforms OT in a small subset of scenarios for only graphs 7A and 7B, the two graphs with smaller number of nodes. Hainke *et al.* (2012) use for the oncogenetic graph scenario a single graph of five nodes and for the CBN scenario a single graph with four nodes. Note that the situations where CBN outperforms OT are essentially the same whether we use OT models fitted with the Oncotree package (Szabo and Pappas, 2013) or the Rtreemix package (Bogojeska, 2014). In addition, note that CBN outperforms OT precisely in those evolutionary scenarios (exp and Bozic, especially when S. Time = last) where clones with few drivers have not been swept out of the population.

2 Comparison of Diff between Oncotree and Rtreemix

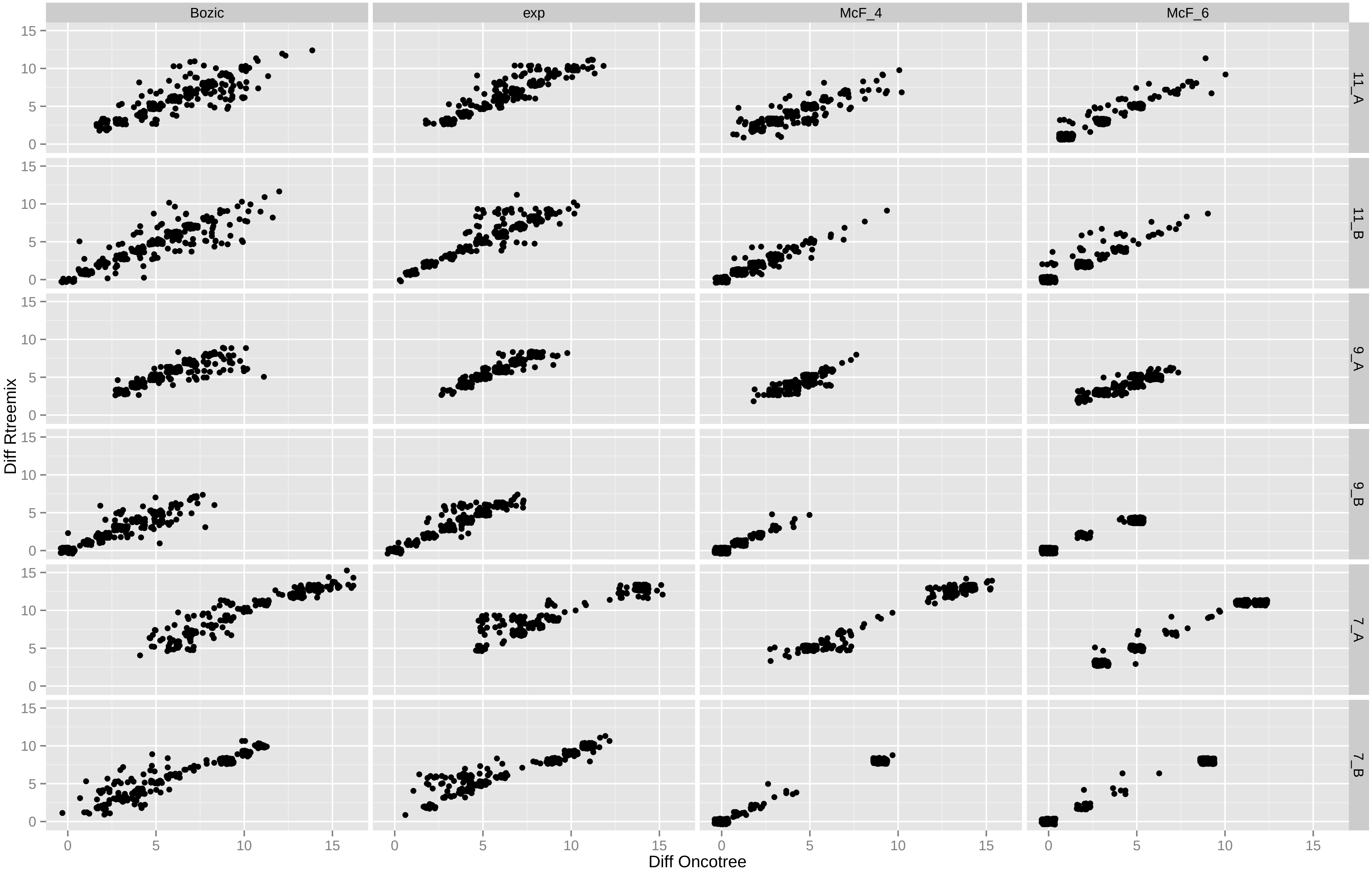

Figure 1: Scatterplot of Diff performance measure computed with Rtreemix and Oncotree for the 5760 samples with Drivers Known and S. Type of single cell. Data have been jittered to minimize superposition of points.

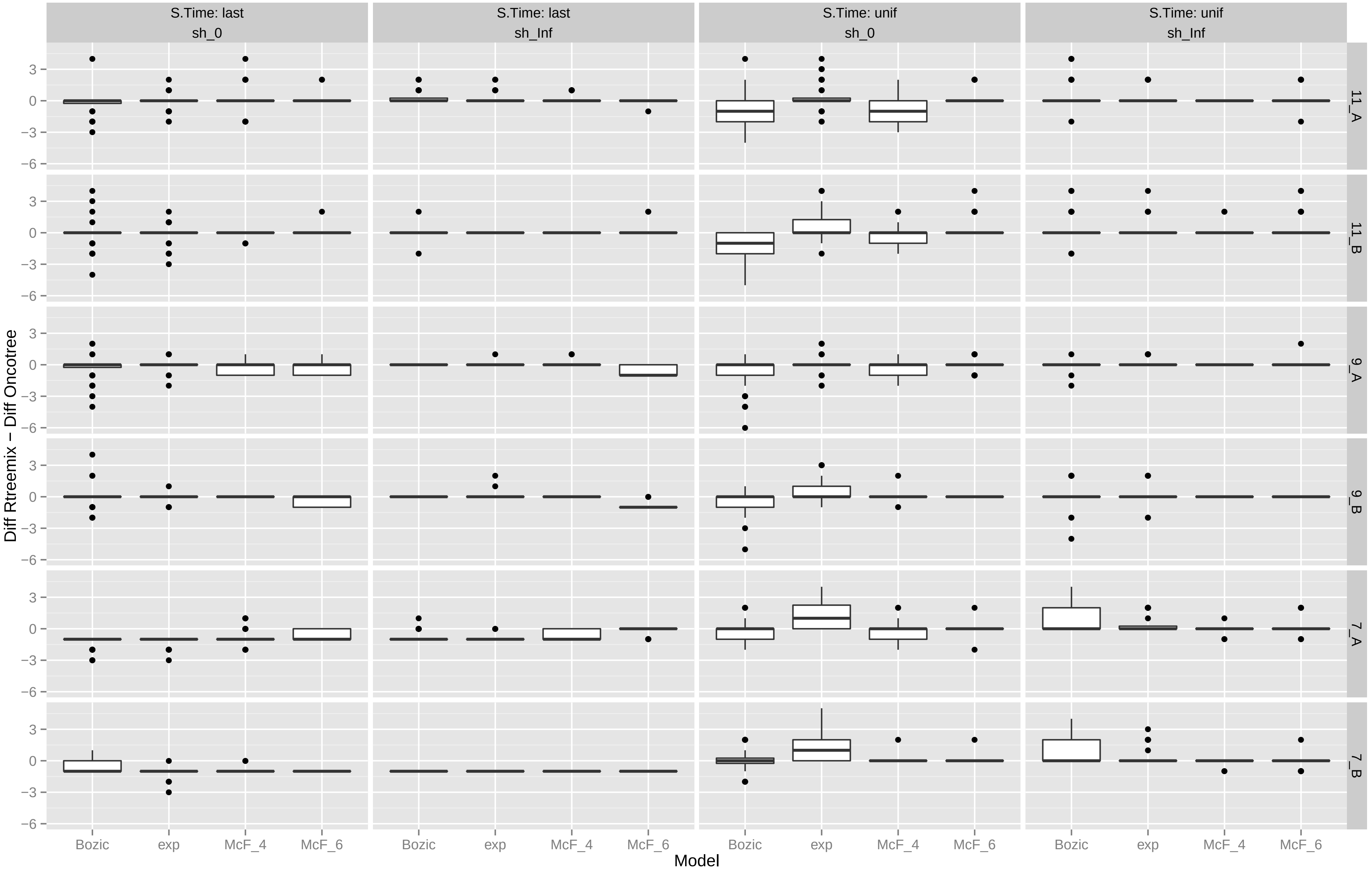

Figure 2: Boxplots of the within dataset difference in Diff between Rtreemix and Oncotree for the 5760 samples with Drivers Known and S. Type of single cell. Each boxplot represents a total of 60 data sets (three sample sizes * 20 replicates per condition).

3 Difference in Diff between CBN and Oncotree/Rtreemix

The next figures show the within dataset difference in Diff between CBN and OT when fitted with the Szabo and Pappas (2013) package, or between CBN and OT when fitted with the Bogojeska (2014) package.

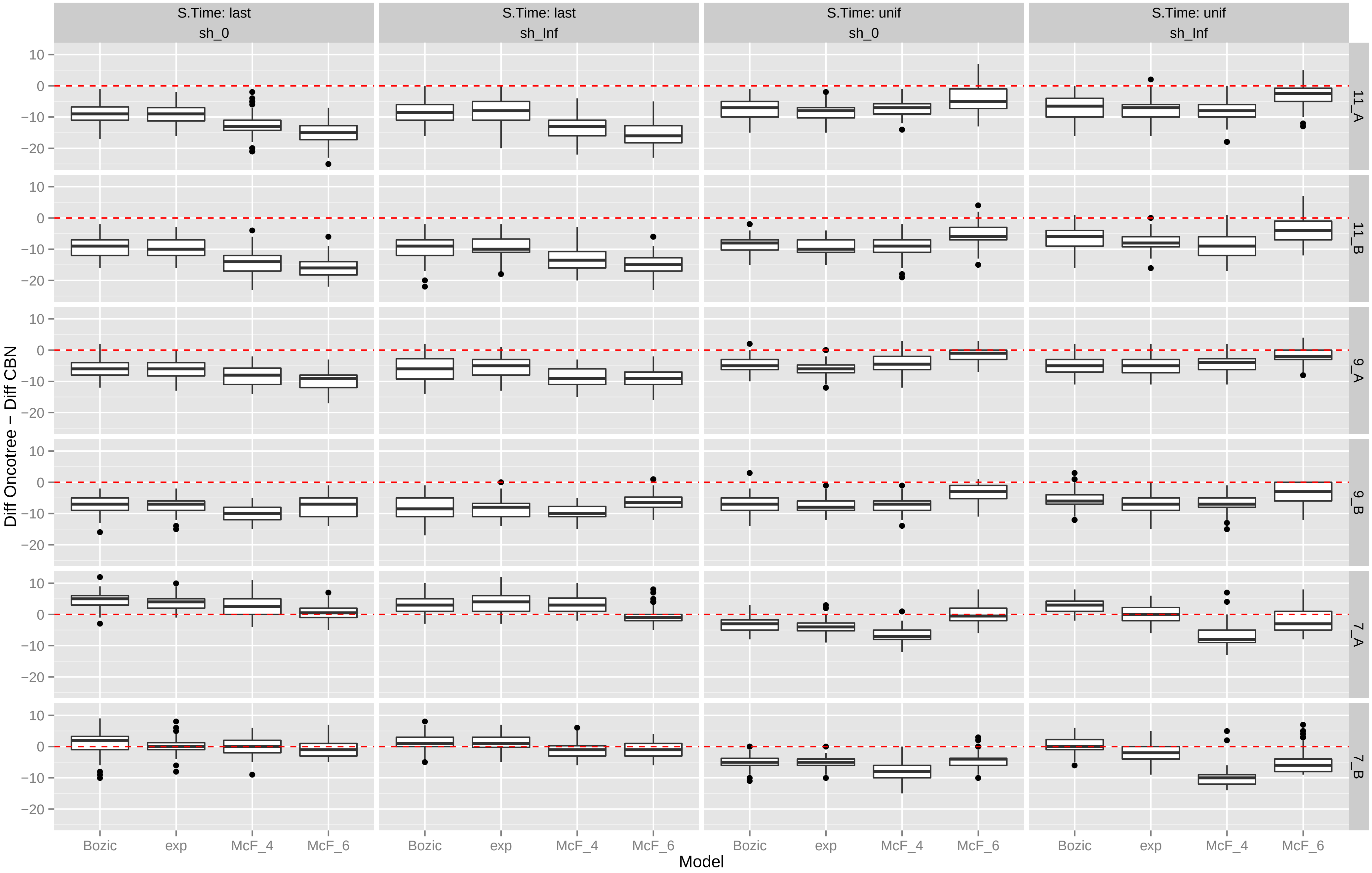

Figure 3: Boxplots of the within dataset difference in Diff between CBN and Oncotree for the 5760 samples with Drivers Known and S. Type of single cell. Each boxplot represents a total of 60 data sets (three sample sizes * 20 replicates per condition). Values above the red horizontal line mean that CBN performs better.

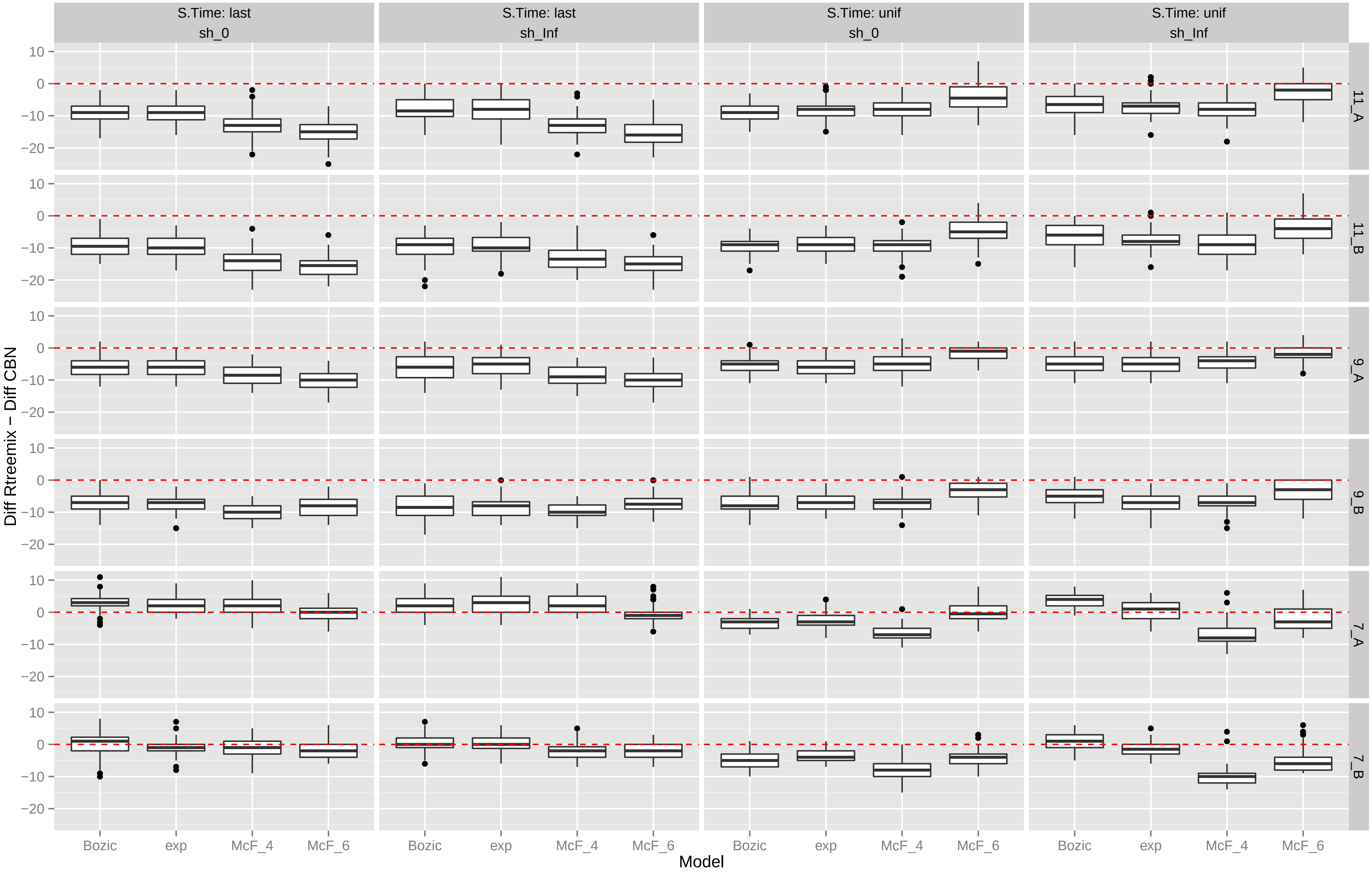

Figure 4: Boxplots of the within dataset difference in Diff between CBN and Rtreemix for the 5760 samples with Drivers Known and S. Type of single cell. Each boxplot represents a total of 60 data sets (three sample sizes * 20 replicates per condition). Values above the red horizontal line mean that CBN performs better.

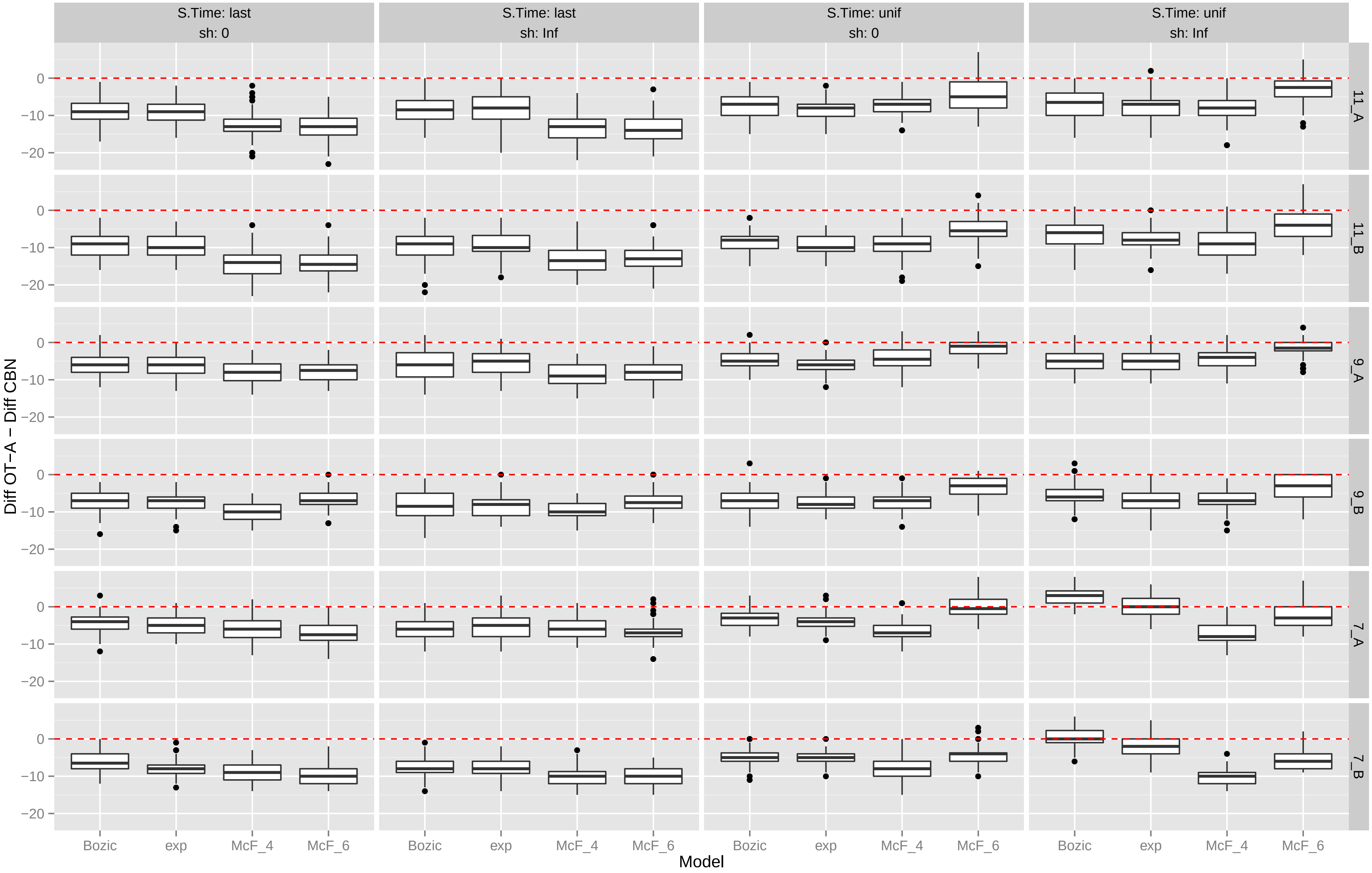

Figure 5: Boxplots of the within dataset difference in Diff between CBN and the OT-A method (fitted with the Oncotree package, after adding the 0.1 fraction of all zeroes) for the 5760 samples with Drivers Known and S. Type of single cell. Each boxplot represents a total of 60 data sets (three sample sizes * 20 replicates per condition). Values above the red horizontal line mean that CBN performs better.

References

Beerenwinkel, N., Eriksson, N., and Sturmfels, B. (2007). Conjunctive Bayesian networks. *Bernoulli*, **13**(4), 893–909.

Bogojeska, J. (2014). Rtreemix: Mutagenetic tree mixture models.

Gerstung, M., Baudis, M., Moch, H., and Beerenwinkel, N. (2009). Quantifying cancer progression with conjunctive Bayesian networks. *Bioinformatics (Oxford, England)*, **25**(21), 2809–2815.

Gerstung, M., Eriksson, N., Lin, J., Vogelstein, B., and Beerenwinkel, N. (2011). The Temporal Order of Genetic and Pathway Alterations in Tumorigenesis. *PLoS ONE*, **6**(11), e27136.

Hainke, K., Rahnenführer, J., and Fried, R. (2012). Cumulative disease progression models for cross-sectional data: A review and comparison. *Biometrical journal. Biometrische Zeitschrift*, **54**(5), 617–40.

Szabo, A. and Pappas, L. (2013). Oncotree: Estimating oncogenetic trees.
