# Supplementary material for: Identifying restrictions in the order of accumulation of mutations during tumor progression: effects of passengers, evolutionary models, and sampling
Source: BMC Bioinformatics. 2015 Feb 12;16:41. doi: 10.1186/s12859-015-0466-7 (PMC4339747; doi:10.1186/s12859-015-0466-7)
Supplement: Additional file 4 — Complete tables of confidence sets. All confidence sets for the 864 combinations of among-data set variables for both the Drivers Known and Drivers Unknown scenarios. [file 12859_2015_466_MOESM4_ESM.pdf]

Additional file 4. Complete tables of confidence sets.

Supplementary material for "Identifying Restrictions in the Order of Accumulation of Mutations during Tumor Progression: Effects of Passengers, Evolutionary Models, and Sampling"

Ramon Diaz-Uriarte
Dept. Biochemistry, Universidad Autónoma de Madrid
Instituto de Investigaciones Biomédicas "Alberto Sols" (UAM-CSIC)
Madrid, Spain

ramon.diaz@iib.uam.es

rdiaz02@gmail.com

http://ligarto.org/rdiaz

Contents

1 Introduction **3**

2 Drivers Known (MCB-2) **4**
2.1 Confidence sets (MCB-2), Diff, Drivers Known 5
2.2 Confidence sets (MCB-2), PFD, Drivers Known 27
2.3 Confidence sets (MCB-2), PND, Drivers Known 49
2.4 Confidence sets (MCB-2), FPF, Drivers Known 71

3 Drivers Unknown (MCB-2) **93**
3.1 Confidence sets (MCB-2), Diff, Drivers Unknown 94
3.2 Confidence sets (MCB-2), PFD, Drivers Unknown 124
3.3 Confidence sets (MCB-2), PND, Drivers Unknown 168
3.4 Confidence sets (MCB-2), FPF, Drivers Unknown 196

4 Drivers Known (MCB) **276**
4.1 Confidence sets (MCB), Diff, Drivers Known 277
4.2 Confidence sets (MCB), PFD, Drivers Known 299
4.3 Confidence sets (MCB), PND, Drivers Known 321
4.4 Confidence sets (MCB), FPF, Drivers Known 343

5 Drivers Unknown (MCB) **365**
5.1 Confidence sets (MCB), Diff, Drivers Unknown 366
5.2 Confidence sets (MCB), PFD, Drivers Unknown 396
5.3 Confidence sets (MCB), PND, Drivers Unknown 441
5.4 Confidence sets (MCB), FPF, Drivers Unknown 472

List of Tables

1 Confidence sets (method MCB-2) when Drivers are Known for measure Diff. 5
2 Confidence sets (method MCB-2) when Drivers are Known for measure PFD. 27
3 Confidence sets (method MCB-2) when Drivers are Known for measure PND. 49
4 Confidence sets (method MCB-2) when Drivers are Known for measure FPF. 71
5 Confidence sets (method MCB-2) when Drivers are Unknown for measure Diff. . . . 94
6 Confidence sets (method MCB-2) when Drivers are Unknown for measure PFD. . . . 124
7 Confidence sets (method MCB-2) when Drivers are Unknown for measure PND. . . 168
8 Confidence sets (method MCB-2) when Drivers are Unknown for measure FPF. . . . 196
9 Confidence sets (method MCB) when Drivers are Known for measure Diff. 277
10 Confidence sets (method MCB) when Drivers are Known for measure PFD. 299
11 Confidence sets (method MCB) when Drivers are Known for measure PND. 321
12 Confidence sets (method MCB) when Drivers are Known for measure FPF. 343
13 Confidence sets (method MCB) when Drivers are Unknown for measure Diff. 366
14 Confidence sets (method MCB) when Drivers are Unknown for measure PFD. 396
15 Confidence sets (method MCB) when Drivers are Unknown for measure PND. . . . 441
16 Confidence sets (method MCB) when Drivers are Unknown for measure FPF. 472

1 Introduction

This file shows the confidence sets for the 864 combinations of among-data set variables. Therefore, each value is based on the comparison of 20 within-data set replicates.

The meaning of the column headings is as used in the rest of the ms. The column denoted “Drivers” refers to the true number of drivers (this information is redundant given the name of the Graph, but allows for easily scanning the large tables). The last column gives the confidence set, obtained from using the “multiple comparisons with the best” procedure, in two different versions (see details in manuscript and Additional File 1); the two methods are called MCB and MCB-2.

2 Drivers Known (MCB-2)

2.1 Confidence sets (MCB-2), Diff, Drivers Known

Table 1: Confidence sets (method MCB-2) when Drivers are Known for measure Diff.

	Conjunction	Drivers	Tree	S.Size	Model	sh	S.Time	S.Type	Best method(s)
1	Yes	11	11-A	1000	Bozic	0	last	singleC	OT, OT-A
2	Yes	11	11-A	1000	Bozic	0	last	wholeT_0.01	CBN-A, DiP, DiP-A, OT, OT-A
3	Yes	11	11-A	1000	Bozic	0	last	wholeT_0.5	OT, OT-A
4	Yes	11	11-A	1000	Bozic	0	unif	singleC	OT, OT-A
5	Yes	11	11-A	1000	Bozic	0	unif	wholeT_0.01	CBN-A, OT, OT-A
6	Yes	11	11-A	1000	Bozic	0	unif	wholeT_0.5	OT, OT-A
7	Yes	11	11-A	1000	Bozic	Inf	last	singleC	OT, OT-A
8	Yes	11	11-A	1000	Bozic	Inf	last	wholeT_0.01	CBN-A, DiP, DiP-A, OT, OT-A
9	Yes	11	11-A	1000	Bozic	Inf	last	wholeT_0.5	OT, OT-A
10	Yes	11	11-A	1000	Bozic	Inf	unif	singleC	OT, OT-A
11	Yes	11	11-A	1000	Bozic	Inf	unif	wholeT_0.01	CBN-A, OT, OT-A
12	Yes	11	11-A	1000	Bozic	Inf	unif	wholeT_0.5	OT, OT-A
13	Yes	11	11-A	1000	exp	0	last	singleC	OT, OT-A
14	Yes	11	11-A	1000	exp	0	last	wholeT_0.01	OT, OT-A
15	Yes	11	11-A	1000	exp	0	last	wholeT_0.5	OT, OT-A
16	Yes	11	11-A	1000	exp	0	unif	singleC	OT, OT-A
17	Yes	11	11-A	1000	exp	0	unif	wholeT_0.01	OT, OT-A
18	Yes	11	11-A	1000	exp	0	unif	wholeT_0.5	OT, OT-A
19	Yes	11	11-A	1000	exp	Inf	last	singleC	OT, OT-A
20	Yes	11	11-A	1000	exp	Inf	last	wholeT_0.01	OT, OT-A
21	Yes	11	11-A	1000	exp	Inf	last	wholeT_0.5	OT, OT-A
22	Yes	11	11-A	1000	exp	Inf	unif	singleC	OT, OT-A
23	Yes	11	11-A	1000	exp	Inf	unif	wholeT_0.01	CBN, OT, OT-A
24	Yes	11	11-A	1000	exp	Inf	unif	wholeT_0.5	OT, OT-A
25	Yes	11	11-A	1000	McF_4	0	last	singleC	OT, OT-A
26	Yes	11	11-A	1000	McF_4	0	last	wholeT_0.01	OT, OT-A
27	Yes	11	11-A	1000	McF_4	0	last	wholeT_0.5	OT, OT-A
28	Yes	11	11-A	1000	McF_4	0	unif	singleC	OT, OT-A
29	Yes	11	11-A	1000	McF_4	0	unif	wholeT_0.01	OT, OT-A
30	Yes	11	11-A	1000	McF_4	0	unif	wholeT_0.5	OT, OT-A
31	Yes	11	11-A	1000	McF_4	Inf	last	singleC	OT, OT-A
32	Yes	11	11-A	1000	McF_4	Inf	last	wholeT_0.01	OT, OT-A
33	Yes	11	11-A	1000	McF_4	Inf	last	wholeT_0.5	OT, OT-A
34	Yes	11	11-A	1000	McF_4	Inf	unif	singleC	OT, OT-A
35	Yes	11	11-A	1000	McF_4	Inf	unif	wholeT_0.01	OT, OT-A
36	Yes	11	11-A	1000	McF_4	Inf	unif	wholeT_0.5	OT, OT-A
37	Yes	11	11-A	1000	McF_6	0	last	singleC	DiP

Table 1: *(continued)*

	Conjunction	Drivers	Tree	S.Size	Model	sh	S.Time	S.Type	Best method(s)
38	Yes	11	11-A	1000	McF_6	0	last	wholeT_0.01	DiP
39	Yes	11	11-A	1000	McF_6	0	last	wholeT_0.5	DiP
40	Yes	11	11-A	1000	McF_6	0	unif	singleC	DiP, DiP-A, OT, OT-A
41	Yes	11	11-A	1000	McF_6	0	unif	wholeT_0.01	DiP, DiP-A
42	Yes	11	11-A	1000	McF_6	0	unif	wholeT_0.5	DiP, DiP-A, OT, OT-A
43	Yes	11	11-A	1000	McF_6	Inf	last	singleC	OT
44	Yes	11	11-A	1000	McF_6	Inf	last	wholeT_0.01	OT
45	Yes	11	11-A	1000	McF_6	Inf	last	wholeT_0.5	OT
46	Yes	11	11-A	1000	McF_6	Inf	unif	singleC	DiP, DiP-A, OT, OT-A
47	Yes	11	11-A	1000	McF_6	Inf	unif	wholeT_0.01	DiP, DiP-A, OT, OT-A
48	Yes	11	11-A	1000	McF_6	Inf	unif	wholeT_0.5	DiP, DiP-A, OT, OT-A
49	Yes	11	11-A	200	Bozic	0	last	singleC	OT, OT-A
50	Yes	11	11-A	200	Bozic	0	last	wholeT_0.01	OT, OT-A
51	Yes	11	11-A	200	Bozic	0	last	wholeT_0.5	OT, OT-A
52	Yes	11	11-A	200	Bozic	0	unif	singleC	OT, OT-A
53	Yes	11	11-A	200	Bozic	0	unif	wholeT_0.01	OT, OT-A
54	Yes	11	11-A	200	Bozic	0	unif	wholeT_0.5	OT, OT-A
55	Yes	11	11-A	200	Bozic	Inf	last	singleC	OT, OT-A
56	Yes	11	11-A	200	Bozic	Inf	last	wholeT_0.01	OT, OT-A
57	Yes	11	11-A	200	Bozic	Inf	last	wholeT_0.5	OT, OT-A
58	Yes	11	11-A	200	Bozic	Inf	unif	singleC	OT, OT-A
59	Yes	11	11-A	200	Bozic	Inf	unif	wholeT_0.01	OT, OT-A
60	Yes	11	11-A	200	Bozic	Inf	unif	wholeT_0.5	OT, OT-A
61	Yes	11	11-A	200	exp	0	last	singleC	OT, OT-A
62	Yes	11	11-A	200	exp	0	last	wholeT_0.01	OT, OT-A
63	Yes	11	11-A	200	exp	0	last	wholeT_0.5	OT, OT-A
64	Yes	11	11-A	200	exp	0	unif	singleC	OT, OT-A
65	Yes	11	11-A	200	exp	0	unif	wholeT_0.01	OT, OT-A
66	Yes	11	11-A	200	exp	0	unif	wholeT_0.5	OT, OT-A
67	Yes	11	11-A	200	exp	Inf	last	singleC	OT, OT-A
68	Yes	11	11-A	200	exp	Inf	last	wholeT_0.01	CBN-A, OT, OT-A
69	Yes	11	11-A	200	exp	Inf	last	wholeT_0.5	OT, OT-A
70	Yes	11	11-A	200	exp	Inf	unif	singleC	OT, OT-A
71	Yes	11	11-A	200	exp	Inf	unif	wholeT_0.01	OT, OT-A
72	Yes	11	11-A	200	exp	Inf	unif	wholeT_0.5	OT, OT-A
73	Yes	11	11-A	200	McF_4	0	last	singleC	OT, OT-A
74	Yes	11	11-A	200	McF_4	0	last	wholeT_0.01	OT, OT-A
75	Yes	11	11-A	200	McF_4	0	last	wholeT_0.5	OT, OT-A
76	Yes	11	11-A	200	McF_4	0	unif	singleC	OT, OT-A
77	Yes	11	11-A	200	McF_4	0	unif	wholeT_0.01	OT, OT-A

Table 1: *(continued)*

	Conjunction	Drivers	Tree	S.Size	Model	sh	S.Time	S.Type	Best method(s)
78	Yes	11	11-A	200	McF_4	0	unif	wholeT_0.5	OT, OT-A
79	Yes	11	11-A	200	McF_4	Inf	last	singleC	OT, OT-A
80	Yes	11	11-A	200	McF_4	Inf	last	wholeT_0.01	OT, OT-A
81	Yes	11	11-A	200	McF_4	Inf	last	wholeT_0.5	OT, OT-A
82	Yes	11	11-A	200	McF_4	Inf	unif	singleC	OT, OT-A
83	Yes	11	11-A	200	McF_4	Inf	unif	wholeT_0.01	OT, OT-A
84	Yes	11	11-A	200	McF_4	Inf	unif	wholeT_0.5	OT, OT-A
85	Yes	11	11-A	200	McF_6	0	last	singleC	OT
86	Yes	11	11-A	200	McF_6	0	last	wholeT_0.01	OT
87	Yes	11	11-A	200	McF_6	0	last	wholeT_0.5	OT
88	Yes	11	11-A	200	McF_6	0	unif	singleC	OT, OT-A
89	Yes	11	11-A	200	McF_6	0	unif	wholeT_0.01	OT, OT-A
90	Yes	11	11-A	200	McF_6	0	unif	wholeT_0.5	OT, OT-A
91	Yes	11	11-A	200	McF_6	Inf	last	singleC	OT
92	Yes	11	11-A	200	McF_6	Inf	last	wholeT_0.01	OT, OT-A
93	Yes	11	11-A	200	McF_6	Inf	last	wholeT_0.5	OT
94	Yes	11	11-A	200	McF_6	Inf	unif	singleC	CBN, CBN-A, OT, OT-A
95	Yes	11	11-A	200	McF_6	Inf	unif	wholeT_0.01	OT, OT-A
96	Yes	11	11-A	200	McF_6	Inf	unif	wholeT_0.5	CBN-A, OT, OT-A
97	Yes	11	11-A	100	Bozic	0	last	singleC	OT, OT-A
98	Yes	11	11-A	100	Bozic	0	last	wholeT_0.01	OT, OT-A
99	Yes	11	11-A	100	Bozic	0	last	wholeT_0.5	OT, OT-A
100	Yes	11	11-A	100	Bozic	0	unif	singleC	OT, OT-A
101	Yes	11	11-A	100	Bozic	0	unif	wholeT_0.01	OT, OT-A
102	Yes	11	11-A	100	Bozic	0	unif	wholeT_0.5	OT, OT-A
103	Yes	11	11-A	100	Bozic	Inf	last	singleC	OT, OT-A
104	Yes	11	11-A	100	Bozic	Inf	last	wholeT_0.01	OT, OT-A
105	Yes	11	11-A	100	Bozic	Inf	last	wholeT_0.5	OT, OT-A
106	Yes	11	11-A	100	Bozic	Inf	unif	singleC	OT, OT-A
107	Yes	11	11-A	100	Bozic	Inf	unif	wholeT_0.01	OT, OT-A
108	Yes	11	11-A	100	Bozic	Inf	unif	wholeT_0.5	OT, OT-A
109	Yes	11	11-A	100	exp	0	last	singleC	OT, OT-A
110	Yes	11	11-A	100	exp	0	last	wholeT_0.01	OT, OT-A
111	Yes	11	11-A	100	exp	0	last	wholeT_0.5	OT, OT-A
112	Yes	11	11-A	100	exp	0	unif	singleC	OT, OT-A
113	Yes	11	11-A	100	exp	0	unif	wholeT_0.01	OT, OT-A
114	Yes	11	11-A	100	exp	0	unif	wholeT_0.5	OT, OT-A
115	Yes	11	11-A	100	exp	Inf	last	singleC	OT, OT-A
116	Yes	11	11-A	100	exp	Inf	last	wholeT_0.01	OT, OT-A
117	Yes	11	11-A	100	exp	Inf	last	wholeT_0.5	OT, OT-A

Table 1: *(continued)*

	Conjunction	Drivers	Tree	S.Size	Model	sh	S.Time	S.Type	Best method(s)
118	Yes	11	11-A	100	exp	Inf	unif	singleC	OT, OT-A
119	Yes	11	11-A	100	exp	Inf	unif	wholeT_0.01	OT, OT-A
120	Yes	11	11-A	100	exp	Inf	unif	wholeT_0.5	OT, OT-A
121	Yes	11	11-A	100	McF_4	0	last	singleC	OT, OT-A
122	Yes	11	11-A	100	McF_4	0	last	wholeT_0.01	OT, OT-A
123	Yes	11	11-A	100	McF_4	0	last	wholeT_0.5	OT, OT-A
124	Yes	11	11-A	100	McF_4	0	unif	singleC	OT, OT-A
125	Yes	11	11-A	100	McF_4	0	unif	wholeT_0.01	OT, OT-A
126	Yes	11	11-A	100	McF_4	0	unif	wholeT_0.5	OT, OT-A
127	Yes	11	11-A	100	McF_4	Inf	last	singleC	OT, OT-A
128	Yes	11	11-A	100	McF_4	Inf	last	wholeT_0.01	OT, OT-A
129	Yes	11	11-A	100	McF_4	Inf	last	wholeT_0.5	OT, OT-A
130	Yes	11	11-A	100	McF_4	Inf	unif	singleC	OT, OT-A
131	Yes	11	11-A	100	McF_4	Inf	unif	wholeT_0.01	OT, OT-A
132	Yes	11	11-A	100	McF_4	Inf	unif	wholeT_0.5	OT, OT-A
133	Yes	11	11-A	100	McF_6	0	last	singleC	OT
134	Yes	11	11-A	100	McF_6	0	last	wholeT_0.01	OT, OT-A
135	Yes	11	11-A	100	McF_6	0	last	wholeT_0.5	OT
136	Yes	11	11-A	100	McF_6	0	unif	singleC	OT, OT-A
137	Yes	11	11-A	100	McF_6	0	unif	wholeT_0.01	OT, OT-A
138	Yes	11	11-A	100	McF_6	0	unif	wholeT_0.5	OT, OT-A
139	Yes	11	11-A	100	McF_6	Inf	last	singleC	OT
140	Yes	11	11-A	100	McF_6	Inf	last	wholeT_0.01	OT, OT-A
141	Yes	11	11-A	100	McF_6	Inf	last	wholeT_0.5	OT
142	Yes	11	11-A	100	McF_6	Inf	unif	singleC	OT, OT-A
143	Yes	11	11-A	100	McF_6	Inf	unif	wholeT_0.01	CBN-A, OT, OT-A
144	Yes	11	11-A	100	McF_6	Inf	unif	wholeT_0.5	OT, OT-A
145	Yes	9	9-A	1000	Bozic	0	last	singleC	OT, OT-A
146	Yes	9	9-A	1000	Bozic	0	last	wholeT_0.01	OT, OT-A
147	Yes	9	9-A	1000	Bozic	0	last	wholeT_0.5	OT, OT-A
148	Yes	9	9-A	1000	Bozic	0	unif	singleC	OT, OT-A
149	Yes	9	9-A	1000	Bozic	0	unif	wholeT_0.01	OT, OT-A
150	Yes	9	9-A	1000	Bozic	0	unif	wholeT_0.5	OT, OT-A
151	Yes	9	9-A	1000	Bozic	Inf	last	singleC	OT, OT-A
152	Yes	9	9-A	1000	Bozic	Inf	last	wholeT_0.01	OT, OT-A
153	Yes	9	9-A	1000	Bozic	Inf	last	wholeT_0.5	OT, OT-A
154	Yes	9	9-A	1000	Bozic	Inf	unif	singleC	OT, OT-A
155	Yes	9	9-A	1000	Bozic	Inf	unif	wholeT_0.01	CBN-A, OT, OT-A
156	Yes	9	9-A	1000	Bozic	Inf	unif	wholeT_0.5	OT, OT-A
157	Yes	9	9-A	1000	exp	0	last	singleC	OT, OT-A

Table 1: *(continued)*

	Conjunction	Drivers	Tree	S.Size	Model	sh	S.Time	S.Type	Best method(s)
158	Yes	9	9-A	1000	exp	0	last	wholeT_0.01	OT, OT-A
159	Yes	9	9-A	1000	exp	0	last	wholeT_0.5	OT, OT-A
160	Yes	9	9-A	1000	exp	0	unif	singleC	OT, OT-A
161	Yes	9	9-A	1000	exp	0	unif	wholeT_0.01	OT, OT-A
162	Yes	9	9-A	1000	exp	0	unif	wholeT_0.5	OT, OT-A
163	Yes	9	9-A	1000	exp	Inf	last	singleC	OT, OT-A
164	Yes	9	9-A	1000	exp	Inf	last	wholeT_0.01	CBN, OT, OT-A
165	Yes	9	9-A	1000	exp	Inf	last	wholeT_0.5	OT, OT-A
166	Yes	9	9-A	1000	exp	Inf	unif	singleC	OT, OT-A
167	Yes	9	9-A	1000	exp	Inf	unif	wholeT_0.01	OT, OT-A
168	Yes	9	9-A	1000	exp	Inf	unif	wholeT_0.5	OT, OT-A
169	Yes	9	9-A	1000	McF_4	0	last	singleC	OT, OT-A
170	Yes	9	9-A	1000	McF_4	0	last	wholeT_0.01	DiP, DiP-A, OT, OT-A
171	Yes	9	9-A	1000	McF_4	0	last	wholeT_0.5	OT, OT-A
172	Yes	9	9-A	1000	McF_4	0	unif	singleC	OT, OT-A
173	Yes	9	9-A	1000	McF_4	0	unif	wholeT_0.01	OT, OT-A
174	Yes	9	9-A	1000	McF_4	0	unif	wholeT_0.5	OT, OT-A
175	Yes	9	9-A	1000	McF_4	Inf	last	singleC	OT, OT-A
176	Yes	9	9-A	1000	McF_4	Inf	last	wholeT_0.01	OT
177	Yes	9	9-A	1000	McF_4	Inf	last	wholeT_0.5	OT, OT-A
178	Yes	9	9-A	1000	McF_4	Inf	unif	singleC	OT, OT-A
179	Yes	9	9-A	1000	McF_4	Inf	unif	wholeT_0.01	OT, OT-A
180	Yes	9	9-A	1000	McF_4	Inf	unif	wholeT_0.5	OT, OT-A
181	Yes	9	9-A	1000	McF_6	0	last	singleC	OT
182	Yes	9	9-A	1000	McF_6	0	last	wholeT_0.01	DiP, OT
183	Yes	9	9-A	1000	McF_6	0	last	wholeT_0.5	OT
184	Yes	9	9-A	1000	McF_6	0	unif	singleC	DiP, DiP-A, OT, OT-A
185	Yes	9	9-A	1000	McF_6	0	unif	wholeT_0.01	DiP, DiP-A, OT, OT-A
186	Yes	9	9-A	1000	McF_6	0	unif	wholeT_0.5	DiP, DiP-A, OT, OT-A
187	Yes	9	9-A	1000	McF_6	Inf	last	singleC	OT
188	Yes	9	9-A	1000	McF_6	Inf	last	wholeT_0.01	OT
189	Yes	9	9-A	1000	McF_6	Inf	last	wholeT_0.5	DiP, OT
190	Yes	9	9-A	1000	McF_6	Inf	unif	singleC	CBN, OT, OT-A
191	Yes	9	9-A	1000	McF_6	Inf	unif	wholeT_0.01	CBN-A, OT, OT-A
192	Yes	9	9-A	1000	McF_6	Inf	unif	wholeT_0.5	OT, OT-A
193	Yes	9	9-A	200	Bozic	0	last	singleC	OT, OT-A
194	Yes	9	9-A	200	Bozic	0	last	wholeT_0.01	OT, OT-A
195	Yes	9	9-A	200	Bozic	0	last	wholeT_0.5	OT, OT-A
196	Yes	9	9-A	200	Bozic	0	unif	singleC	OT, OT-A
197	Yes	9	9-A	200	Bozic	0	unif	wholeT_0.01	OT, OT-A

Table 1: *(continued)*

	Conjunction	Drivers	Tree	S.Size	Model	sh	S.Time	S.Type	Best method(s)
198	Yes	9	9-A	200	Bozic	0	unif	wholeT_0.5	OT, OT-A
199	Yes	9	9-A	200	Bozic	Inf	last	singleC	OT, OT-A
200	Yes	9	9-A	200	Bozic	Inf	last	wholeT_0.01	OT, OT-A
201	Yes	9	9-A	200	Bozic	Inf	last	wholeT_0.5	OT, OT-A
202	Yes	9	9-A	200	Bozic	Inf	unif	singleC	OT, OT-A
203	Yes	9	9-A	200	Bozic	Inf	unif	wholeT_0.01	OT, OT-A
204	Yes	9	9-A	200	Bozic	Inf	unif	wholeT_0.5	OT, OT-A
205	Yes	9	9-A	200	exp	0	last	singleC	OT, OT-A
206	Yes	9	9-A	200	exp	0	last	wholeT_0.01	OT, OT-A
207	Yes	9	9-A	200	exp	0	last	wholeT_0.5	OT, OT-A
208	Yes	9	9-A	200	exp	0	unif	singleC	OT, OT-A
209	Yes	9	9-A	200	exp	0	unif	wholeT_0.01	OT, OT-A
210	Yes	9	9-A	200	exp	0	unif	wholeT_0.5	OT, OT-A
211	Yes	9	9-A	200	exp	Inf	last	singleC	OT, OT-A
212	Yes	9	9-A	200	exp	Inf	last	wholeT_0.01	OT, OT-A
213	Yes	9	9-A	200	exp	Inf	last	wholeT_0.5	OT, OT-A
214	Yes	9	9-A	200	exp	Inf	unif	singleC	OT, OT-A
215	Yes	9	9-A	200	exp	Inf	unif	wholeT_0.01	OT, OT-A
216	Yes	9	9-A	200	exp	Inf	unif	wholeT_0.5	OT, OT-A
217	Yes	9	9-A	200	McF_4	0	last	singleC	OT, OT-A
218	Yes	9	9-A	200	McF_4	0	last	wholeT_0.01	OT, OT-A
219	Yes	9	9-A	200	McF_4	0	last	wholeT_0.5	OT, OT-A
220	Yes	9	9-A	200	McF_4	0	unif	singleC	OT, OT-A
221	Yes	9	9-A	200	McF_4	0	unif	wholeT_0.01	OT, OT-A
222	Yes	9	9-A	200	McF_4	0	unif	wholeT_0.5	OT, OT-A
223	Yes	9	9-A	200	McF_4	Inf	last	singleC	OT, OT-A
224	Yes	9	9-A	200	McF_4	Inf	last	wholeT_0.01	OT
225	Yes	9	9-A	200	McF_4	Inf	last	wholeT_0.5	OT, OT-A
226	Yes	9	9-A	200	McF_4	Inf	unif	singleC	OT, OT-A
227	Yes	9	9-A	200	McF_4	Inf	unif	wholeT_0.01	OT, OT-A
228	Yes	9	9-A	200	McF_4	Inf	unif	wholeT_0.5	OT, OT-A
229	Yes	9	9-A	200	McF_6	0	last	singleC	OT
230	Yes	9	9-A	200	McF_6	0	last	wholeT_0.01	OT
231	Yes	9	9-A	200	McF_6	0	last	wholeT_0.5	OT
232	Yes	9	9-A	200	McF_6	0	unif	singleC	CBN, OT, OT-A
233	Yes	9	9-A	200	McF_6	0	unif	wholeT_0.01	OT, OT-A
234	Yes	9	9-A	200	McF_6	0	unif	wholeT_0.5	OT, OT-A
235	Yes	9	9-A	200	McF_6	Inf	last	singleC	OT
236	Yes	9	9-A	200	McF_6	Inf	last	wholeT_0.01	OT
237	Yes	9	9-A	200	McF_6	Inf	last	wholeT_0.5	OT

Table 1: *(continued)*

	Conjunction	Drivers	Tree	S.Size	Model	sh	S.Time	S.Type	Best method(s)
238	Yes	9	9-A	200	McF_6	Inf	unif	singleC	OT, OT-A
239	Yes	9	9-A	200	McF_6	Inf	unif	wholeT_0.01	OT, OT-A
240	Yes	9	9-A	200	McF_6	Inf	unif	wholeT_0.5	OT, OT-A
241	Yes	9	9-A	100	Bozic	0	last	singleC	OT, OT-A
242	Yes	9	9-A	100	Bozic	0	last	wholeT_0.01	OT, OT-A
243	Yes	9	9-A	100	Bozic	0	last	wholeT_0.5	OT, OT-A
244	Yes	9	9-A	100	Bozic	0	unif	singleC	OT, OT-A
245	Yes	9	9-A	100	Bozic	0	unif	wholeT_0.01	OT, OT-A
246	Yes	9	9-A	100	Bozic	0	unif	wholeT_0.5	OT, OT-A
247	Yes	9	9-A	100	Bozic	Inf	last	singleC	OT, OT-A
248	Yes	9	9-A	100	Bozic	Inf	last	wholeT_0.01	OT, OT-A
249	Yes	9	9-A	100	Bozic	Inf	last	wholeT_0.5	OT, OT-A
250	Yes	9	9-A	100	Bozic	Inf	unif	singleC	OT, OT-A
251	Yes	9	9-A	100	Bozic	Inf	unif	wholeT_0.01	OT, OT-A
252	Yes	9	9-A	100	Bozic	Inf	unif	wholeT_0.5	OT, OT-A
253	Yes	9	9-A	100	exp	0	last	singleC	OT, OT-A
254	Yes	9	9-A	100	exp	0	last	wholeT_0.01	OT, OT-A
255	Yes	9	9-A	100	exp	0	last	wholeT_0.5	OT, OT-A
256	Yes	9	9-A	100	exp	0	unif	singleC	OT, OT-A
257	Yes	9	9-A	100	exp	0	unif	wholeT_0.01	OT, OT-A
258	Yes	9	9-A	100	exp	0	unif	wholeT_0.5	OT, OT-A
259	Yes	9	9-A	100	exp	Inf	last	singleC	OT, OT-A
260	Yes	9	9-A	100	exp	Inf	last	wholeT_0.01	OT, OT-A
261	Yes	9	9-A	100	exp	Inf	last	wholeT_0.5	OT, OT-A
262	Yes	9	9-A	100	exp	Inf	unif	singleC	OT, OT-A
263	Yes	9	9-A	100	exp	Inf	unif	wholeT_0.01	OT, OT-A
264	Yes	9	9-A	100	exp	Inf	unif	wholeT_0.5	OT, OT-A
265	Yes	9	9-A	100	McF_4	0	last	singleC	OT, OT-A
266	Yes	9	9-A	100	McF_4	0	last	wholeT_0.01	OT, OT-A
267	Yes	9	9-A	100	McF_4	0	last	wholeT_0.5	OT, OT-A
268	Yes	9	9-A	100	McF_4	0	unif	singleC	OT, OT-A
269	Yes	9	9-A	100	McF_4	0	unif	wholeT_0.01	OT, OT-A
270	Yes	9	9-A	100	McF_4	0	unif	wholeT_0.5	OT, OT-A
271	Yes	9	9-A	100	McF_4	Inf	last	singleC	OT, OT-A
272	Yes	9	9-A	100	McF_4	Inf	last	wholeT_0.01	OT
273	Yes	9	9-A	100	McF_4	Inf	last	wholeT_0.5	OT, OT-A
274	Yes	9	9-A	100	McF_4	Inf	unif	singleC	OT, OT-A
275	Yes	9	9-A	100	McF_4	Inf	unif	wholeT_0.01	OT, OT-A
276	Yes	9	9-A	100	McF_4	Inf	unif	wholeT_0.5	OT, OT-A
277	Yes	9	9-A	100	McF_6	0	last	singleC	OT

Table 1: *(continued)*

	Conjunction	Drivers	Tree	S.Size	Model	sh	S.Time	S.Type	Best method(s)
278	Yes	9	9-A	100	McF_6	0	last	wholeT_0.01	OT
279	Yes	9	9-A	100	McF_6	0	last	wholeT_0.5	OT
280	Yes	9	9-A	100	McF_6	0	unif	singleC	OT, OT-A
281	Yes	9	9-A	100	McF_6	0	unif	wholeT_0.01	OT, OT-A
282	Yes	9	9-A	100	McF_6	0	unif	wholeT_0.5	OT, OT-A
283	Yes	9	9-A	100	McF_6	Inf	last	singleC	OT
284	Yes	9	9-A	100	McF_6	Inf	last	wholeT_0.01	OT
285	Yes	9	9-A	100	McF_6	Inf	last	wholeT_0.5	OT
286	Yes	9	9-A	100	McF_6	Inf	unif	singleC	OT, OT-A
287	Yes	9	9-A	100	McF_6	Inf	unif	wholeT_0.01	CBN, OT, OT-A
288	Yes	9	9-A	100	McF_6	Inf	unif	wholeT_0.5	OT, OT-A
289	Yes	7	7-A	1000	Bozic	0	last	singleC	OT-A
290	Yes	7	7-A	1000	Bozic	0	last	wholeT_0.01	OT-A
291	Yes	7	7-A	1000	Bozic	0	last	wholeT_0.5	OT-A
292	Yes	7	7-A	1000	Bozic	0	unif	singleC	OT, OT-A
293	Yes	7	7-A	1000	Bozic	0	unif	wholeT_0.01	CBN, CBN-A
294	Yes	7	7-A	1000	Bozic	0	unif	wholeT_0.5	OT, OT-A
295	Yes	7	7-A	1000	Bozic	Inf	last	singleC	OT-A
296	Yes	7	7-A	1000	Bozic	Inf	last	wholeT_0.01	OT-A
297	Yes	7	7-A	1000	Bozic	Inf	last	wholeT_0.5	OT-A
298	Yes	7	7-A	1000	Bozic	Inf	unif	singleC	CBN, CBN-A
299	Yes	7	7-A	1000	Bozic	Inf	unif	wholeT_0.01	OT, OT-A
300	Yes	7	7-A	1000	Bozic	Inf	unif	wholeT_0.5	CBN, CBN-A
301	Yes	7	7-A	1000	exp	0	last	singleC	OT-A
302	Yes	7	7-A	1000	exp	0	last	wholeT_0.01	OT-A
303	Yes	7	7-A	1000	exp	0	last	wholeT_0.5	OT-A
304	Yes	7	7-A	1000	exp	0	unif	singleC	OT, OT-A
305	Yes	7	7-A	1000	exp	0	unif	wholeT_0.01	CBN, CBN-A, OT-A
306	Yes	7	7-A	1000	exp	0	unif	wholeT_0.5	OT, OT-A
307	Yes	7	7-A	1000	exp	Inf	last	singleC	OT-A
308	Yes	7	7-A	1000	exp	Inf	last	wholeT_0.01	OT-A
309	Yes	7	7-A	1000	exp	Inf	last	wholeT_0.5	OT-A
310	Yes	7	7-A	1000	exp	Inf	unif	singleC	CBN, CBN-A, OT, OT-A
311	Yes	7	7-A	1000	exp	Inf	unif	wholeT_0.01	CBN, OT, OT-A
312	Yes	7	7-A	1000	exp	Inf	unif	wholeT_0.5	CBN, CBN-A
313	Yes	7	7-A	1000	McF_4	0	last	singleC	OT-A
314	Yes	7	7-A	1000	McF_4	0	last	wholeT_0.01	OT-A
315	Yes	7	7-A	1000	McF_4	0	last	wholeT_0.5	OT-A
316	Yes	7	7-A	1000	McF_4	0	unif	singleC	OT, OT-A
317	Yes	7	7-A	1000	McF_4	0	unif	wholeT_0.01	OT, OT-A

Table 1: *(continued)*

	Conjunction	Drivers	Tree	S.Size	Model	sh	S.Time	S.Type	Best method(s)
318	Yes	7	7-A	1000	McF_4	0	unif	wholeT_0.5	OT, OT-A
319	Yes	7	7-A	1000	McF_4	Inf	last	singleC	OT-A
320	Yes	7	7-A	1000	McF_4	Inf	last	wholeT_0.01	OT-A
321	Yes	7	7-A	1000	McF_4	Inf	last	wholeT_0.5	OT-A
322	Yes	7	7-A	1000	McF_4	Inf	unif	singleC	OT, OT-A
323	Yes	7	7-A	1000	McF_4	Inf	unif	wholeT_0.01	OT, OT-A
324	Yes	7	7-A	1000	McF_4	Inf	unif	wholeT_0.5	OT, OT-A
325	Yes	7	7-A	1000	McF_6	0	last	singleC	OT-A
326	Yes	7	7-A	1000	McF_6	0	last	wholeT_0.01	OT-A
327	Yes	7	7-A	1000	McF_6	0	last	wholeT_0.5	OT-A
328	Yes	7	7-A	1000	McF_6	0	unif	singleC	CBN-A
329	Yes	7	7-A	1000	McF_6	0	unif	wholeT_0.01	CBN-A, DiP, DiP-A
330	Yes	7	7-A	1000	McF_6	0	unif	wholeT_0.5	CBN-A
331	Yes	7	7-A	1000	McF_6	Inf	last	singleC	DiP-A, OT-A
332	Yes	7	7-A	1000	McF_6	Inf	last	wholeT_0.01	DiP-A, OT-A
333	Yes	7	7-A	1000	McF_6	Inf	last	wholeT_0.5	DiP-A, OT-A
334	Yes	7	7-A	1000	McF_6	Inf	unif	singleC	CBN-A
335	Yes	7	7-A	1000	McF_6	Inf	unif	wholeT_0.01	CBN-A
336	Yes	7	7-A	1000	McF_6	Inf	unif	wholeT_0.5	CBN-A
337	Yes	7	7-A	200	Bozic	0	last	singleC	OT-A
338	Yes	7	7-A	200	Bozic	0	last	wholeT_0.01	OT-A
339	Yes	7	7-A	200	Bozic	0	last	wholeT_0.5	OT-A
340	Yes	7	7-A	200	Bozic	0	unif	singleC	OT, OT-A
341	Yes	7	7-A	200	Bozic	0	unif	wholeT_0.01	CBN, CBN-A
342	Yes	7	7-A	200	Bozic	0	unif	wholeT_0.5	OT, OT-A
343	Yes	7	7-A	200	Bozic	Inf	last	singleC	OT-A
344	Yes	7	7-A	200	Bozic	Inf	last	wholeT_0.01	OT-A
345	Yes	7	7-A	200	Bozic	Inf	last	wholeT_0.5	OT-A
346	Yes	7	7-A	200	Bozic	Inf	unif	singleC	CBN, CBN-A
347	Yes	7	7-A	200	Bozic	Inf	unif	wholeT_0.01	OT, OT-A
348	Yes	7	7-A	200	Bozic	Inf	unif	wholeT_0.5	CBN, CBN-A
349	Yes	7	7-A	200	exp	0	last	singleC	OT-A
350	Yes	7	7-A	200	exp	0	last	wholeT_0.01	OT-A
351	Yes	7	7-A	200	exp	0	last	wholeT_0.5	OT-A
352	Yes	7	7-A	200	exp	0	unif	singleC	OT, OT-A
353	Yes	7	7-A	200	exp	0	unif	wholeT_0.01	CBN, CBN-A, OT, OT-A
354	Yes	7	7-A	200	exp	0	unif	wholeT_0.5	OT, OT-A
355	Yes	7	7-A	200	exp	Inf	last	singleC	OT-A
356	Yes	7	7-A	200	exp	Inf	last	wholeT_0.01	OT-A
357	Yes	7	7-A	200	exp	Inf	last	wholeT_0.5	OT-A

Table 1: *(continued)*

	Conjunction	Drivers	Tree	S.Size	Model	sh	S.Time	S.Type	Best method(s)
358	Yes	7	7-A	200	exp	Inf	unif	singleC	CBN, CBN-A, OT, OT-A
359	Yes	7	7-A	200	exp	Inf	unif	wholeT_0.01	OT, OT-A
360	Yes	7	7-A	200	exp	Inf	unif	wholeT_0.5	CBN, CBN-A, OT, OT-A
361	Yes	7	7-A	200	McF_4	0	last	singleC	OT-A
362	Yes	7	7-A	200	McF_4	0	last	wholeT_0.01	OT-A
363	Yes	7	7-A	200	McF_4	0	last	wholeT_0.5	OT-A
364	Yes	7	7-A	200	McF_4	0	unif	singleC	OT, OT-A
365	Yes	7	7-A	200	McF_4	0	unif	wholeT_0.01	OT, OT-A
366	Yes	7	7-A	200	McF_4	0	unif	wholeT_0.5	OT, OT-A
367	Yes	7	7-A	200	McF_4	Inf	last	singleC	OT-A
368	Yes	7	7-A	200	McF_4	Inf	last	wholeT_0.01	OT-A
369	Yes	7	7-A	200	McF_4	Inf	last	wholeT_0.5	OT-A
370	Yes	7	7-A	200	McF_4	Inf	unif	singleC	OT, OT-A
371	Yes	7	7-A	200	McF_4	Inf	unif	wholeT_0.01	OT, OT-A
372	Yes	7	7-A	200	McF_4	Inf	unif	wholeT_0.5	OT, OT-A
373	Yes	7	7-A	200	McF_6	0	last	singleC	OT-A
374	Yes	7	7-A	200	McF_6	0	last	wholeT_0.01	OT-A
375	Yes	7	7-A	200	McF_6	0	last	wholeT_0.5	OT-A
376	Yes	7	7-A	200	McF_6	0	unif	singleC	CBN-A
377	Yes	7	7-A	200	McF_6	0	unif	wholeT_0.01	CBN-A, OT, OT-A
378	Yes	7	7-A	200	McF_6	0	unif	wholeT_0.5	CBN-A
379	Yes	7	7-A	200	McF_6	Inf	last	singleC	OT-A
380	Yes	7	7-A	200	McF_6	Inf	last	wholeT_0.01	OT-A
381	Yes	7	7-A	200	McF_6	Inf	last	wholeT_0.5	OT-A
382	Yes	7	7-A	200	McF_6	Inf	unif	singleC	CBN-A
383	Yes	7	7-A	200	McF_6	Inf	unif	wholeT_0.01	CBN-A
384	Yes	7	7-A	200	McF_6	Inf	unif	wholeT_0.5	CBN-A
385	Yes	7	7-A	100	Bozic	0	last	singleC	OT-A
386	Yes	7	7-A	100	Bozic	0	last	wholeT_0.01	OT-A
387	Yes	7	7-A	100	Bozic	0	last	wholeT_0.5	OT-A
388	Yes	7	7-A	100	Bozic	0	unif	singleC	OT, OT-A
389	Yes	7	7-A	100	Bozic	0	unif	wholeT_0.01	CBN, CBN-A, OT, OT-A
390	Yes	7	7-A	100	Bozic	0	unif	wholeT_0.5	OT, OT-A
391	Yes	7	7-A	100	Bozic	Inf	last	singleC	OT-A
392	Yes	7	7-A	100	Bozic	Inf	last	wholeT_0.01	OT-A
393	Yes	7	7-A	100	Bozic	Inf	last	wholeT_0.5	OT-A
394	Yes	7	7-A	100	Bozic	Inf	unif	singleC	CBN, CBN-A
395	Yes	7	7-A	100	Bozic	Inf	unif	wholeT_0.01	CBN, CBN-A, OT, OT-A
396	Yes	7	7-A	100	Bozic	Inf	unif	wholeT_0.5	CBN, CBN-A
397	Yes	7	7-A	100	exp	0	last	singleC	OT-A

Table 1: *(continued)*

	Conjunction	Drivers	Tree	S.Size	Model	sh	S.Time	S.Type	Best method(s)
398	Yes	7	7-A	100	exp	0	last	wholeT_0.01	OT-A
399	Yes	7	7-A	100	exp	0	last	wholeT_0.5	OT-A
400	Yes	7	7-A	100	exp	0	unif	singleC	OT, OT-A
401	Yes	7	7-A	100	exp	0	unif	wholeT_0.01	CBN, OT, OT-A
402	Yes	7	7-A	100	exp	0	unif	wholeT_0.5	OT, OT-A
403	Yes	7	7-A	100	exp	Inf	last	singleC	OT-A
404	Yes	7	7-A	100	exp	Inf	last	wholeT_0.01	OT-A
405	Yes	7	7-A	100	exp	Inf	last	wholeT_0.5	OT-A
406	Yes	7	7-A	100	exp	Inf	unif	singleC	CBN-A, OT, OT-A
407	Yes	7	7-A	100	exp	Inf	unif	wholeT_0.01	OT, OT-A
408	Yes	7	7-A	100	exp	Inf	unif	wholeT_0.5	CBN, OT, OT-A
409	Yes	7	7-A	100	McF_4	0	last	singleC	OT-A
410	Yes	7	7-A	100	McF_4	0	last	wholeT_0.01	OT-A
411	Yes	7	7-A	100	McF_4	0	last	wholeT_0.5	OT-A
412	Yes	7	7-A	100	McF_4	0	unif	singleC	OT, OT-A
413	Yes	7	7-A	100	McF_4	0	unif	wholeT_0.01	OT, OT-A
414	Yes	7	7-A	100	McF_4	0	unif	wholeT_0.5	OT, OT-A
415	Yes	7	7-A	100	McF_4	Inf	last	singleC	OT-A
416	Yes	7	7-A	100	McF_4	Inf	last	wholeT_0.01	OT-A
417	Yes	7	7-A	100	McF_4	Inf	last	wholeT_0.5	OT-A
418	Yes	7	7-A	100	McF_4	Inf	unif	singleC	OT, OT-A
419	Yes	7	7-A	100	McF_4	Inf	unif	wholeT_0.01	OT, OT-A
420	Yes	7	7-A	100	McF_4	Inf	unif	wholeT_0.5	OT, OT-A
421	Yes	7	7-A	100	McF_6	0	last	singleC	OT-A
422	Yes	7	7-A	100	McF_6	0	last	wholeT_0.01	OT-A
423	Yes	7	7-A	100	McF_6	0	last	wholeT_0.5	OT-A
424	Yes	7	7-A	100	McF_6	0	unif	singleC	CBN, CBN-A
425	Yes	7	7-A	100	McF_6	0	unif	wholeT_0.01	CBN, CBN-A, OT, OT-A
426	Yes	7	7-A	100	McF_6	0	unif	wholeT_0.5	CBN-A
427	Yes	7	7-A	100	McF_6	Inf	last	singleC	OT-A
428	Yes	7	7-A	100	McF_6	Inf	last	wholeT_0.01	OT-A
429	Yes	7	7-A	100	McF_6	Inf	last	wholeT_0.5	OT-A
430	Yes	7	7-A	100	McF_6	Inf	unif	singleC	CBN-A
431	Yes	7	7-A	100	McF_6	Inf	unif	wholeT_0.01	CBN-A
432	Yes	7	7-A	100	McF_6	Inf	unif	wholeT_0.5	CBN-A
433	No	11	11-B	1000	Bozic	0	last	singleC	OT, OT-A
434	No	11	11-B	1000	Bozic	0	last	wholeT_0.01	OT, OT-A
435	No	11	11-B	1000	Bozic	0	last	wholeT_0.5	OT, OT-A
436	No	11	11-B	1000	Bozic	0	unif	singleC	OT, OT-A
437	No	11	11-B	1000	Bozic	0	unif	wholeT_0.01	OT, OT-A

Table 1: *(continued)*

	Conjunction	Drivers	Tree	S.Size	Model	sh	S.Time	S.Type	Best method(s)
438	No	11	11-B	1000	Bozic	0	unif	wholeT_0.5	OT, OT-A
439	No	11	11-B	1000	Bozic	Inf	last	singleC	OT, OT-A
440	No	11	11-B	1000	Bozic	Inf	last	wholeT_0.01	DiP-A
441	No	11	11-B	1000	Bozic	Inf	last	wholeT_0.5	OT, OT-A
442	No	11	11-B	1000	Bozic	Inf	unif	singleC	OT, OT-A
443	No	11	11-B	1000	Bozic	Inf	unif	wholeT_0.01	OT, OT-A
444	No	11	11-B	1000	Bozic	Inf	unif	wholeT_0.5	OT, OT-A
445	No	11	11-B	1000	exp	0	last	singleC	OT, OT-A
446	No	11	11-B	1000	exp	0	last	wholeT_0.01	OT, OT-A
447	No	11	11-B	1000	exp	0	last	wholeT_0.5	OT, OT-A
448	No	11	11-B	1000	exp	0	unif	singleC	OT, OT-A
449	No	11	11-B	1000	exp	0	unif	wholeT_0.01	OT, OT-A
450	No	11	11-B	1000	exp	0	unif	wholeT_0.5	OT, OT-A
451	No	11	11-B	1000	exp	Inf	last	singleC	OT, OT-A
452	No	11	11-B	1000	exp	Inf	last	wholeT_0.01	OT, OT-A
453	No	11	11-B	1000	exp	Inf	last	wholeT_0.5	OT, OT-A
454	No	11	11-B	1000	exp	Inf	unif	singleC	OT, OT-A
455	No	11	11-B	1000	exp	Inf	unif	wholeT_0.01	OT, OT-A
456	No	11	11-B	1000	exp	Inf	unif	wholeT_0.5	OT, OT-A
457	No	11	11-B	1000	McF_4	0	last	singleC	OT, OT-A
458	No	11	11-B	1000	McF_4	0	last	wholeT_0.01	DiP, DiP-A, OT, OT-A
459	No	11	11-B	1000	McF_4	0	last	wholeT_0.5	OT, OT-A
460	No	11	11-B	1000	McF_4	0	unif	singleC	OT, OT-A
461	No	11	11-B	1000	McF_4	0	unif	wholeT_0.01	OT, OT-A
462	No	11	11-B	1000	McF_4	0	unif	wholeT_0.5	OT, OT-A
463	No	11	11-B	1000	McF_4	Inf	last	singleC	OT, OT-A
464	No	11	11-B	1000	McF_4	Inf	last	wholeT_0.01	OT, OT-A
465	No	11	11-B	1000	McF_4	Inf	last	wholeT_0.5	OT, OT-A
466	No	11	11-B	1000	McF_4	Inf	unif	singleC	OT, OT-A
467	No	11	11-B	1000	McF_4	Inf	unif	wholeT_0.01	OT, OT-A
468	No	11	11-B	1000	McF_4	Inf	unif	wholeT_0.5	OT, OT-A
469	No	11	11-B	1000	McF_6	0	last	singleC	DiP, OT
470	No	11	11-B	1000	McF_6	0	last	wholeT_0.01	DiP, OT
471	No	11	11-B	1000	McF_6	0	last	wholeT_0.5	DiP, OT
472	No	11	11-B	1000	McF_6	0	unif	singleC	DiP, DiP-A, OT, OT-A
473	No	11	11-B	1000	McF_6	0	unif	wholeT_0.01	DiP, DiP-A, OT, OT-A
474	No	11	11-B	1000	McF_6	0	unif	wholeT_0.5	DiP, DiP-A
475	No	11	11-B	1000	McF_6	Inf	last	singleC	DiP, OT
476	No	11	11-B	1000	McF_6	Inf	last	wholeT_0.01	OT
477	No	11	11-B	1000	McF_6	Inf	last	wholeT_0.5	DiP, OT

Table 1: *(continued)*

	Conjunction	Drivers	Tree	S.Size	Model	sh	S.Time	S.Type	Best method(s)
478	No	11	11-B	1000	McF_6	Inf	unif	singleC	DiP, DiP-A
479	No	11	11-B	1000	McF_6	Inf	unif	wholeT_0.01	DiP, DiP-A
480	No	11	11-B	1000	McF_6	Inf	unif	wholeT_0.5	DiP, DiP-A, OT, OT-A
481	No	11	11-B	200	Bozic	0	last	singleC	OT, OT-A
482	No	11	11-B	200	Bozic	0	last	wholeT_0.01	OT, OT-A
483	No	11	11-B	200	Bozic	0	last	wholeT_0.5	OT, OT-A
484	No	11	11-B	200	Bozic	0	unif	singleC	OT, OT-A
485	No	11	11-B	200	Bozic	0	unif	wholeT_0.01	OT, OT-A
486	No	11	11-B	200	Bozic	0	unif	wholeT_0.5	OT, OT-A
487	No	11	11-B	200	Bozic	Inf	last	singleC	OT, OT-A
488	No	11	11-B	200	Bozic	Inf	last	wholeT_0.01	OT, OT-A
489	No	11	11-B	200	Bozic	Inf	last	wholeT_0.5	OT, OT-A
490	No	11	11-B	200	Bozic	Inf	unif	singleC	OT, OT-A
491	No	11	11-B	200	Bozic	Inf	unif	wholeT_0.01	OT, OT-A
492	No	11	11-B	200	Bozic	Inf	unif	wholeT_0.5	OT, OT-A
493	No	11	11-B	200	exp	0	last	singleC	OT, OT-A
494	No	11	11-B	200	exp	0	last	wholeT_0.01	OT, OT-A
495	No	11	11-B	200	exp	0	last	wholeT_0.5	OT, OT-A
496	No	11	11-B	200	exp	0	unif	singleC	OT, OT-A
497	No	11	11-B	200	exp	0	unif	wholeT_0.01	OT, OT-A
498	No	11	11-B	200	exp	0	unif	wholeT_0.5	OT, OT-A
499	No	11	11-B	200	exp	Inf	last	singleC	OT, OT-A
500	No	11	11-B	200	exp	Inf	last	wholeT_0.01	OT, OT-A
501	No	11	11-B	200	exp	Inf	last	wholeT_0.5	OT, OT-A
502	No	11	11-B	200	exp	Inf	unif	singleC	OT, OT-A
503	No	11	11-B	200	exp	Inf	unif	wholeT_0.01	OT, OT-A
504	No	11	11-B	200	exp	Inf	unif	wholeT_0.5	OT, OT-A
505	No	11	11-B	200	McF_4	0	last	singleC	OT, OT-A
506	No	11	11-B	200	McF_4	0	last	wholeT_0.01	OT, OT-A
507	No	11	11-B	200	McF_4	0	last	wholeT_0.5	OT, OT-A
508	No	11	11-B	200	McF_4	0	unif	singleC	OT, OT-A
509	No	11	11-B	200	McF_4	0	unif	wholeT_0.01	OT, OT-A
510	No	11	11-B	200	McF_4	0	unif	wholeT_0.5	OT, OT-A
511	No	11	11-B	200	McF_4	Inf	last	singleC	OT, OT-A
512	No	11	11-B	200	McF_4	Inf	last	wholeT_0.01	OT, OT-A
513	No	11	11-B	200	McF_4	Inf	last	wholeT_0.5	OT, OT-A
514	No	11	11-B	200	McF_4	Inf	unif	singleC	OT, OT-A
515	No	11	11-B	200	McF_4	Inf	unif	wholeT_0.01	OT, OT-A
516	No	11	11-B	200	McF_4	Inf	unif	wholeT_0.5	OT, OT-A
517	No	11	11-B	200	McF_6	0	last	singleC	OT

Table 1: *(continued)*

	Conjunction	Drivers	Tree	S.Size	Model	sh	S.Time	S.Type	Best method(s)
518	No	11	11-B	200	McF_6	0	last	wholeT_0.01	OT
519	No	11	11-B	200	McF_6	0	last	wholeT_0.5	OT
520	No	11	11-B	200	McF_6	0	unif	singleC	OT, OT-A
521	No	11	11-B	200	McF_6	0	unif	wholeT_0.01	OT, OT-A
522	No	11	11-B	200	McF_6	0	unif	wholeT_0.5	OT, OT-A
523	No	11	11-B	200	McF_6	Inf	last	singleC	OT
524	No	11	11-B	200	McF_6	Inf	last	wholeT_0.01	OT
525	No	11	11-B	200	McF_6	Inf	last	wholeT_0.5	OT
526	No	11	11-B	200	McF_6	Inf	unif	singleC	OT, OT-A
527	No	11	11-B	200	McF_6	Inf	unif	wholeT_0.01	OT, OT-A
528	No	11	11-B	200	McF_6	Inf	unif	wholeT_0.5	OT, OT-A
529	No	11	11-B	100	Bozic	0	last	singleC	OT, OT-A
530	No	11	11-B	100	Bozic	0	last	wholeT_0.01	OT, OT-A
531	No	11	11-B	100	Bozic	0	last	wholeT_0.5	OT, OT-A
532	No	11	11-B	100	Bozic	0	unif	singleC	OT, OT-A
533	No	11	11-B	100	Bozic	0	unif	wholeT_0.01	OT, OT-A
534	No	11	11-B	100	Bozic	0	unif	wholeT_0.5	OT, OT-A
535	No	11	11-B	100	Bozic	Inf	last	singleC	OT, OT-A
536	No	11	11-B	100	Bozic	Inf	last	wholeT_0.01	OT, OT-A
537	No	11	11-B	100	Bozic	Inf	last	wholeT_0.5	OT, OT-A
538	No	11	11-B	100	Bozic	Inf	unif	singleC	OT, OT-A
539	No	11	11-B	100	Bozic	Inf	unif	wholeT_0.01	OT, OT-A
540	No	11	11-B	100	Bozic	Inf	unif	wholeT_0.5	OT, OT-A
541	No	11	11-B	100	exp	0	last	singleC	OT, OT-A
542	No	11	11-B	100	exp	0	last	wholeT_0.01	OT, OT-A
543	No	11	11-B	100	exp	0	last	wholeT_0.5	OT, OT-A
544	No	11	11-B	100	exp	0	unif	singleC	OT, OT-A
545	No	11	11-B	100	exp	0	unif	wholeT_0.01	OT, OT-A
546	No	11	11-B	100	exp	0	unif	wholeT_0.5	OT, OT-A
547	No	11	11-B	100	exp	Inf	last	singleC	OT, OT-A
548	No	11	11-B	100	exp	Inf	last	wholeT_0.01	OT, OT-A
549	No	11	11-B	100	exp	Inf	last	wholeT_0.5	OT, OT-A
550	No	11	11-B	100	exp	Inf	unif	singleC	OT, OT-A
551	No	11	11-B	100	exp	Inf	unif	wholeT_0.01	OT, OT-A
552	No	11	11-B	100	exp	Inf	unif	wholeT_0.5	OT, OT-A
553	No	11	11-B	100	McF_4	0	last	singleC	OT, OT-A
554	No	11	11-B	100	McF_4	0	last	wholeT_0.01	OT, OT-A
555	No	11	11-B	100	McF_4	0	last	wholeT_0.5	OT, OT-A
556	No	11	11-B	100	McF_4	0	unif	singleC	OT, OT-A
557	No	11	11-B	100	McF_4	0	unif	wholeT_0.01	OT, OT-A

Table 1: *(continued)*

	Conjunction	Drivers	Tree	S.Size	Model	sh	S.Time	S.Type	Best method(s)
558	No	11	11-B	100	McF_4	0	unif	wholeT_0.5	OT, OT-A
559	No	11	11-B	100	McF_4	Inf	last	singleC	OT, OT-A
560	No	11	11-B	100	McF_4	Inf	last	wholeT_0.01	OT, OT-A
561	No	11	11-B	100	McF_4	Inf	last	wholeT_0.5	OT, OT-A
562	No	11	11-B	100	McF_4	Inf	unif	singleC	OT, OT-A
563	No	11	11-B	100	McF_4	Inf	unif	wholeT_0.01	OT, OT-A
564	No	11	11-B	100	McF_4	Inf	unif	wholeT_0.5	OT, OT-A
565	No	11	11-B	100	McF_6	0	last	singleC	OT
566	No	11	11-B	100	McF_6	0	last	wholeT_0.01	OT
567	No	11	11-B	100	McF_6	0	last	wholeT_0.5	OT
568	No	11	11-B	100	McF_6	0	unif	singleC	OT, OT-A
569	No	11	11-B	100	McF_6	0	unif	wholeT_0.01	OT, OT-A
570	No	11	11-B	100	McF_6	0	unif	wholeT_0.5	OT, OT-A
571	No	11	11-B	100	McF_6	Inf	last	singleC	OT
572	No	11	11-B	100	McF_6	Inf	last	wholeT_0.01	OT, OT-A
573	No	11	11-B	100	McF_6	Inf	last	wholeT_0.5	OT
574	No	11	11-B	100	McF_6	Inf	unif	singleC	OT, OT-A
575	No	11	11-B	100	McF_6	Inf	unif	wholeT_0.01	OT, OT-A
576	No	11	11-B	100	McF_6	Inf	unif	wholeT_0.5	OT, OT-A
577	No	9	9-B	1000	Bozic	0	last	singleC	OT, OT-A
578	No	9	9-B	1000	Bozic	0	last	wholeT_0.01	OT, OT-A
579	No	9	9-B	1000	Bozic	0	last	wholeT_0.5	OT, OT-A
580	No	9	9-B	1000	Bozic	0	unif	singleC	OT, OT-A
581	No	9	9-B	1000	Bozic	0	unif	wholeT_0.01	OT, OT-A
582	No	9	9-B	1000	Bozic	0	unif	wholeT_0.5	OT, OT-A
583	No	9	9-B	1000	Bozic	Inf	last	singleC	OT, OT-A
584	No	9	9-B	1000	Bozic	Inf	last	wholeT_0.01	DiP, DiP-A
585	No	9	9-B	1000	Bozic	Inf	last	wholeT_0.5	OT, OT-A
586	No	9	9-B	1000	Bozic	Inf	unif	singleC	OT, OT-A
587	No	9	9-B	1000	Bozic	Inf	unif	wholeT_0.01	OT, OT-A
588	No	9	9-B	1000	Bozic	Inf	unif	wholeT_0.5	OT, OT-A
589	No	9	9-B	1000	exp	0	last	singleC	OT, OT-A
590	No	9	9-B	1000	exp	0	last	wholeT_0.01	OT, OT-A
591	No	9	9-B	1000	exp	0	last	wholeT_0.5	OT, OT-A
592	No	9	9-B	1000	exp	0	unif	singleC	OT, OT-A
593	No	9	9-B	1000	exp	0	unif	wholeT_0.01	OT, OT-A
594	No	9	9-B	1000	exp	0	unif	wholeT_0.5	OT, OT-A
595	No	9	9-B	1000	exp	Inf	last	singleC	OT, OT-A
596	No	9	9-B	1000	exp	Inf	last	wholeT_0.01	CBN-A, DiP-A, OT, OT-A
597	No	9	9-B	1000	exp	Inf	last	wholeT_0.5	OT, OT-A

Table 1: *(continued)*

	Conjunction	Drivers	Tree	S.Size	Model	sh	S.Time	S.Type	Best method(s)
598	No	9	9-B	1000	exp	Inf	unif	singleC	OT, OT-A
599	No	9	9-B	1000	exp	Inf	unif	wholeT_0.01	OT, OT-A
600	No	9	9-B	1000	exp	Inf	unif	wholeT_0.5	OT, OT-A
601	No	9	9-B	1000	McF_4	0	last	singleC	DiP-A, OT, OT-A
602	No	9	9-B	1000	McF_4	0	last	wholeT_0.01	DiP, DiP-A, OT, OT-A
603	No	9	9-B	1000	McF_4	0	last	wholeT_0.5	OT, OT-A
604	No	9	9-B	1000	McF_4	0	unif	singleC	OT, OT-A
605	No	9	9-B	1000	McF_4	0	unif	wholeT_0.01	OT, OT-A
606	No	9	9-B	1000	McF_4	0	unif	wholeT_0.5	OT, OT-A
607	No	9	9-B	1000	McF_4	Inf	last	singleC	OT, OT-A
608	No	9	9-B	1000	McF_4	Inf	last	wholeT_0.01	DiP, DiP-A, OT, OT-A
609	No	9	9-B	1000	McF_4	Inf	last	wholeT_0.5	OT, OT-A
610	No	9	9-B	1000	McF_4	Inf	unif	singleC	OT, OT-A
611	No	9	9-B	1000	McF_4	Inf	unif	wholeT_0.01	OT, OT-A
612	No	9	9-B	1000	McF_4	Inf	unif	wholeT_0.5	OT, OT-A
613	No	9	9-B	1000	McF_6	0	last	singleC	OT
614	No	9	9-B	1000	McF_6	0	last	wholeT_0.01	OT
615	No	9	9-B	1000	McF_6	0	last	wholeT_0.5	OT
616	No	9	9-B	1000	McF_6	0	unif	singleC	DiP, DiP-A, OT, OT-A
617	No	9	9-B	1000	McF_6	0	unif	wholeT_0.01	DiP, DiP-A, OT, OT-A
618	No	9	9-B	1000	McF_6	0	unif	wholeT_0.5	DiP, DiP-A, OT, OT-A
619	No	9	9-B	1000	McF_6	Inf	last	singleC	DiP, DiP-A, OT-A
620	No	9	9-B	1000	McF_6	Inf	last	wholeT_0.01	DiP, DiP-A, OT-A
621	No	9	9-B	1000	McF_6	Inf	last	wholeT_0.5	DiP, DiP-A, OT-A
622	No	9	9-B	1000	McF_6	Inf	unif	singleC	DiP, DiP-A, OT, OT-A
623	No	9	9-B	1000	McF_6	Inf	unif	wholeT_0.01	DiP, DiP-A, OT, OT-A
624	No	9	9-B	1000	McF_6	Inf	unif	wholeT_0.5	DiP, DiP-A, OT, OT-A
625	No	9	9-B	200	Bozic	0	last	singleC	OT, OT-A
626	No	9	9-B	200	Bozic	0	last	wholeT_0.01	OT, OT-A
627	No	9	9-B	200	Bozic	0	last	wholeT_0.5	OT, OT-A
628	No	9	9-B	200	Bozic	0	unif	singleC	OT, OT-A
629	No	9	9-B	200	Bozic	0	unif	wholeT_0.01	OT, OT-A
630	No	9	9-B	200	Bozic	0	unif	wholeT_0.5	OT, OT-A
631	No	9	9-B	200	Bozic	Inf	last	singleC	OT, OT-A
632	No	9	9-B	200	Bozic	Inf	last	wholeT_0.01	OT, OT-A
633	No	9	9-B	200	Bozic	Inf	last	wholeT_0.5	OT, OT-A
634	No	9	9-B	200	Bozic	Inf	unif	singleC	OT, OT-A
635	No	9	9-B	200	Bozic	Inf	unif	wholeT_0.01	OT, OT-A
636	No	9	9-B	200	Bozic	Inf	unif	wholeT_0.5	OT, OT-A
637	No	9	9-B	200	exp	0	last	singleC	OT, OT-A

Table 1: *(continued)*

	Conjunction	Drivers	Tree	S.Size	Model	sh	S.Time	S.Type	Best method(s)
638	No	9	9-B	200	exp	0	last	wholeT_0.01	OT, OT-A
639	No	9	9-B	200	exp	0	last	wholeT_0.5	OT, OT-A
640	No	9	9-B	200	exp	0	unif	singleC	OT, OT-A
641	No	9	9-B	200	exp	0	unif	wholeT_0.01	OT, OT-A
642	No	9	9-B	200	exp	0	unif	wholeT_0.5	OT, OT-A
643	No	9	9-B	200	exp	Inf	last	singleC	OT, OT-A
644	No	9	9-B	200	exp	Inf	last	wholeT_0.01	OT, OT-A
645	No	9	9-B	200	exp	Inf	last	wholeT_0.5	OT, OT-A
646	No	9	9-B	200	exp	Inf	unif	singleC	OT, OT-A
647	No	9	9-B	200	exp	Inf	unif	wholeT_0.01	OT, OT-A
648	No	9	9-B	200	exp	Inf	unif	wholeT_0.5	OT, OT-A
649	No	9	9-B	200	McF_4	0	last	singleC	OT, OT-A
650	No	9	9-B	200	McF_4	0	last	wholeT_0.01	OT, OT-A
651	No	9	9-B	200	McF_4	0	last	wholeT_0.5	OT, OT-A
652	No	9	9-B	200	McF_4	0	unif	singleC	OT, OT-A
653	No	9	9-B	200	McF_4	0	unif	wholeT_0.01	OT, OT-A
654	No	9	9-B	200	McF_4	0	unif	wholeT_0.5	OT, OT-A
655	No	9	9-B	200	McF_4	Inf	last	singleC	OT, OT-A
656	No	9	9-B	200	McF_4	Inf	last	wholeT_0.01	OT, OT-A
657	No	9	9-B	200	McF_4	Inf	last	wholeT_0.5	OT, OT-A
658	No	9	9-B	200	McF_4	Inf	unif	singleC	OT, OT-A
659	No	9	9-B	200	McF_4	Inf	unif	wholeT_0.01	OT, OT-A
660	No	9	9-B	200	McF_4	Inf	unif	wholeT_0.5	OT, OT-A
661	No	9	9-B	200	McF_6	0	last	singleC	OT, OT-A
662	No	9	9-B	200	McF_6	0	last	wholeT_0.01	OT, OT-A
663	No	9	9-B	200	McF_6	0	last	wholeT_0.5	OT, OT-A
664	No	9	9-B	200	McF_6	0	unif	singleC	OT, OT-A
665	No	9	9-B	200	McF_6	0	unif	wholeT_0.01	OT, OT-A
666	No	9	9-B	200	McF_6	0	unif	wholeT_0.5	OT, OT-A
667	No	9	9-B	200	McF_6	Inf	last	singleC	OT-A
668	No	9	9-B	200	McF_6	Inf	last	wholeT_0.01	OT-A
669	No	9	9-B	200	McF_6	Inf	last	wholeT_0.5	OT-A
670	No	9	9-B	200	McF_6	Inf	unif	singleC	OT, OT-A
671	No	9	9-B	200	McF_6	Inf	unif	wholeT_0.01	OT, OT-A
672	No	9	9-B	200	McF_6	Inf	unif	wholeT_0.5	OT, OT-A
673	No	9	9-B	100	Bozic	0	last	singleC	OT, OT-A
674	No	9	9-B	100	Bozic	0	last	wholeT_0.01	OT, OT-A
675	No	9	9-B	100	Bozic	0	last	wholeT_0.5	OT, OT-A
676	No	9	9-B	100	Bozic	0	unif	singleC	OT, OT-A
677	No	9	9-B	100	Bozic	0	unif	wholeT_0.01	OT, OT-A

Table 1: *(continued)*

	Conjunction	Drivers	Tree	S.Size	Model	sh	S.Time	S.Type	Best method(s)
678	No	9	9-B	100	Bozic	0	unif	wholeT_0.5	OT, OT-A
679	No	9	9-B	100	Bozic	Inf	last	singleC	OT, OT-A
680	No	9	9-B	100	Bozic	Inf	last	wholeT_0.01	OT, OT-A
681	No	9	9-B	100	Bozic	Inf	last	wholeT_0.5	OT, OT-A
682	No	9	9-B	100	Bozic	Inf	unif	singleC	OT, OT-A
683	No	9	9-B	100	Bozic	Inf	unif	wholeT_0.01	OT, OT-A
684	No	9	9-B	100	Bozic	Inf	unif	wholeT_0.5	OT, OT-A
685	No	9	9-B	100	exp	0	last	singleC	OT, OT-A
686	No	9	9-B	100	exp	0	last	wholeT_0.01	OT, OT-A
687	No	9	9-B	100	exp	0	last	wholeT_0.5	OT, OT-A
688	No	9	9-B	100	exp	0	unif	singleC	OT, OT-A
689	No	9	9-B	100	exp	0	unif	wholeT_0.01	OT, OT-A
690	No	9	9-B	100	exp	0	unif	wholeT_0.5	OT, OT-A
691	No	9	9-B	100	exp	Inf	last	singleC	OT, OT-A
692	No	9	9-B	100	exp	Inf	last	wholeT_0.01	OT, OT-A
693	No	9	9-B	100	exp	Inf	last	wholeT_0.5	OT, OT-A
694	No	9	9-B	100	exp	Inf	unif	singleC	OT, OT-A
695	No	9	9-B	100	exp	Inf	unif	wholeT_0.01	OT, OT-A
696	No	9	9-B	100	exp	Inf	unif	wholeT_0.5	OT, OT-A
697	No	9	9-B	100	McF_4	0	last	singleC	OT, OT-A
698	No	9	9-B	100	McF_4	0	last	wholeT_0.01	OT, OT-A
699	No	9	9-B	100	McF_4	0	last	wholeT_0.5	OT, OT-A
700	No	9	9-B	100	McF_4	0	unif	singleC	OT, OT-A
701	No	9	9-B	100	McF_4	0	unif	wholeT_0.01	OT, OT-A
702	No	9	9-B	100	McF_4	0	unif	wholeT_0.5	OT, OT-A
703	No	9	9-B	100	McF_4	Inf	last	singleC	OT, OT-A
704	No	9	9-B	100	McF_4	Inf	last	wholeT_0.01	OT, OT-A
705	No	9	9-B	100	McF_4	Inf	last	wholeT_0.5	OT, OT-A
706	No	9	9-B	100	McF_4	Inf	unif	singleC	OT, OT-A
707	No	9	9-B	100	McF_4	Inf	unif	wholeT_0.01	OT, OT-A
708	No	9	9-B	100	McF_4	Inf	unif	wholeT_0.5	OT, OT-A
709	No	9	9-B	100	McF_6	0	last	singleC	OT, OT-A
710	No	9	9-B	100	McF_6	0	last	wholeT_0.01	OT, OT-A
711	No	9	9-B	100	McF_6	0	last	wholeT_0.5	OT, OT-A
712	No	9	9-B	100	McF_6	0	unif	singleC	OT, OT-A
713	No	9	9-B	100	McF_6	0	unif	wholeT_0.01	OT, OT-A
714	No	9	9-B	100	McF_6	0	unif	wholeT_0.5	OT, OT-A
715	No	9	9-B	100	McF_6	Inf	last	singleC	OT-A
716	No	9	9-B	100	McF_6	Inf	last	wholeT_0.01	OT-A
717	No	9	9-B	100	McF_6	Inf	last	wholeT_0.5	OT-A

Table 1: *(continued)*

	Conjunction	Drivers	Tree	S.Size	Model	sh	S.Time	S.Type	Best method(s)
718	No	9	9-B	100	McF_6	Inf	unif	singleC	OT, OT-A
719	No	9	9-B	100	McF_6	Inf	unif	wholeT_0.01	OT, OT-A
720	No	9	9-B	100	McF_6	Inf	unif	wholeT_0.5	OT, OT-A
721	No	7	7-B	1000	Bozic	0	last	singleC	OT-A
722	No	7	7-B	1000	Bozic	0	last	wholeT_0.01	OT-A
723	No	7	7-B	1000	Bozic	0	last	wholeT_0.5	OT-A
724	No	7	7-B	1000	Bozic	0	unif	singleC	OT, OT-A
725	No	7	7-B	1000	Bozic	0	unif	wholeT_0.01	CBN-A, OT, OT-A
726	No	7	7-B	1000	Bozic	0	unif	wholeT_0.5	OT, OT-A
727	No	7	7-B	1000	Bozic	Inf	last	singleC	DiP-A, OT-A
728	No	7	7-B	1000	Bozic	Inf	last	wholeT_0.01	DiP-A, OT-A
729	No	7	7-B	1000	Bozic	Inf	last	wholeT_0.5	DiP-A, OT-A
730	No	7	7-B	1000	Bozic	Inf	unif	singleC	CBN, CBN-A, OT, OT-A
731	No	7	7-B	1000	Bozic	Inf	unif	wholeT_0.01	OT, OT-A
732	No	7	7-B	1000	Bozic	Inf	unif	wholeT_0.5	CBN, CBN-A
733	No	7	7-B	1000	exp	0	last	singleC	OT-A
734	No	7	7-B	1000	exp	0	last	wholeT_0.01	OT-A
735	No	7	7-B	1000	exp	0	last	wholeT_0.5	OT-A
736	No	7	7-B	1000	exp	0	unif	singleC	OT, OT-A
737	No	7	7-B	1000	exp	0	unif	wholeT_0.01	CBN, CBN-A, OT, OT-A
738	No	7	7-B	1000	exp	0	unif	wholeT_0.5	OT, OT-A
739	No	7	7-B	1000	exp	Inf	last	singleC	OT-A
740	No	7	7-B	1000	exp	Inf	last	wholeT_0.01	OT-A
741	No	7	7-B	1000	exp	Inf	last	wholeT_0.5	OT-A
742	No	7	7-B	1000	exp	Inf	unif	singleC	CBN, OT, OT-A
743	No	7	7-B	1000	exp	Inf	unif	wholeT_0.01	OT, OT-A
744	No	7	7-B	1000	exp	Inf	unif	wholeT_0.5	OT, OT-A
745	No	7	7-B	1000	McF_4	0	last	singleC	DiP-A, OT-A
746	No	7	7-B	1000	McF_4	0	last	wholeT_0.01	DiP-A, OT-A
747	No	7	7-B	1000	McF_4	0	last	wholeT_0.5	DiP-A, OT-A
748	No	7	7-B	1000	McF_4	0	unif	singleC	DiP-A, OT, OT-A
749	No	7	7-B	1000	McF_4	0	unif	wholeT_0.01	DiP-A, OT, OT-A
750	No	7	7-B	1000	McF_4	0	unif	wholeT_0.5	DiP-A, OT, OT-A
751	No	7	7-B	1000	McF_4	Inf	last	singleC	DiP-A, OT-A
752	No	7	7-B	1000	McF_4	Inf	last	wholeT_0.01	DiP-A, OT-A
753	No	7	7-B	1000	McF_4	Inf	last	wholeT_0.5	DiP-A, OT-A
754	No	7	7-B	1000	McF_4	Inf	unif	singleC	OT, OT-A
755	No	7	7-B	1000	McF_4	Inf	unif	wholeT_0.01	OT, OT-A
756	No	7	7-B	1000	McF_4	Inf	unif	wholeT_0.5	OT, OT-A
757	No	7	7-B	1000	McF_6	0	last	singleC	DiP-A, OT-A

Table 1: *(continued)*

	Conjunction	Drivers	Tree	S.Size	Model	sh	S.Time	S.Type	Best method(s)
758	No	7	7-B	1000	McF_6	0	last	wholeT_0.01	DiP-A, OT-A
759	No	7	7-B	1000	McF_6	0	last	wholeT_0.5	DiP-A, OT-A
760	No	7	7-B	1000	McF_6	0	unif	singleC	DiP, DiP-A, OT, OT-A
761	No	7	7-B	1000	McF_6	0	unif	wholeT_0.01	DiP, DiP-A, OT, OT-A
762	No	7	7-B	1000	McF_6	0	unif	wholeT_0.5	DiP, DiP-A, OT, OT-A
763	No	7	7-B	1000	McF_6	Inf	last	singleC	DiP-A, OT-A
764	No	7	7-B	1000	McF_6	Inf	last	wholeT_0.01	DiP-A, OT-A
765	No	7	7-B	1000	McF_6	Inf	last	wholeT_0.5	DiP-A, OT-A
766	No	7	7-B	1000	McF_6	Inf	unif	singleC	DiP-A, OT, OT-A
767	No	7	7-B	1000	McF_6	Inf	unif	wholeT_0.01	DiP-A, OT, OT-A
768	No	7	7-B	1000	McF_6	Inf	unif	wholeT_0.5	DiP-A, OT, OT-A
769	No	7	7-B	200	Bozic	0	last	singleC	OT-A
770	No	7	7-B	200	Bozic	0	last	wholeT_0.01	OT-A
771	No	7	7-B	200	Bozic	0	last	wholeT_0.5	OT-A
772	No	7	7-B	200	Bozic	0	unif	singleC	OT, OT-A
773	No	7	7-B	200	Bozic	0	unif	wholeT_0.01	CBN, CBN-A, OT, OT-A
774	No	7	7-B	200	Bozic	0	unif	wholeT_0.5	OT, OT-A
775	No	7	7-B	200	Bozic	Inf	last	singleC	OT-A
776	No	7	7-B	200	Bozic	Inf	last	wholeT_0.01	OT-A
777	No	7	7-B	200	Bozic	Inf	last	wholeT_0.5	OT-A
778	No	7	7-B	200	Bozic	Inf	unif	singleC	CBN, CBN-A, OT, OT-A
779	No	7	7-B	200	Bozic	Inf	unif	wholeT_0.01	OT, OT-A
780	No	7	7-B	200	Bozic	Inf	unif	wholeT_0.5	CBN, CBN-A, OT, OT-A
781	No	7	7-B	200	exp	0	last	singleC	OT-A
782	No	7	7-B	200	exp	0	last	wholeT_0.01	OT-A
783	No	7	7-B	200	exp	0	last	wholeT_0.5	OT-A
784	No	7	7-B	200	exp	0	unif	singleC	OT, OT-A
785	No	7	7-B	200	exp	0	unif	wholeT_0.01	OT, OT-A
786	No	7	7-B	200	exp	0	unif	wholeT_0.5	OT, OT-A
787	No	7	7-B	200	exp	Inf	last	singleC	OT-A
788	No	7	7-B	200	exp	Inf	last	wholeT_0.01	OT-A
789	No	7	7-B	200	exp	Inf	last	wholeT_0.5	OT-A
790	No	7	7-B	200	exp	Inf	unif	singleC	CBN, CBN-A, OT, OT-A
791	No	7	7-B	200	exp	Inf	unif	wholeT_0.01	OT, OT-A
792	No	7	7-B	200	exp	Inf	unif	wholeT_0.5	OT, OT-A
793	No	7	7-B	200	McF_4	0	last	singleC	OT-A
794	No	7	7-B	200	McF_4	0	last	wholeT_0.01	OT-A
795	No	7	7-B	200	McF_4	0	last	wholeT_0.5	OT-A
796	No	7	7-B	200	McF_4	0	unif	singleC	OT, OT-A
797	No	7	7-B	200	McF_4	0	unif	wholeT_0.01	OT, OT-A

Table 1: *(continued)*

	Conjunction	Drivers	Tree	S.Size	Model	sh	S.Time	S.Type	Best method(s)
798	No	7	7-B	200	McF_4	0	unif	wholeT_0.5	OT, OT-A
799	No	7	7-B	200	McF_4	Inf	last	singleC	OT-A
800	No	7	7-B	200	McF_4	Inf	last	wholeT_0.01	OT-A
801	No	7	7-B	200	McF_4	Inf	last	wholeT_0.5	OT-A
802	No	7	7-B	200	McF_4	Inf	unif	singleC	OT, OT-A
803	No	7	7-B	200	McF_4	Inf	unif	wholeT_0.01	OT, OT-A
804	No	7	7-B	200	McF_4	Inf	unif	wholeT_0.5	OT, OT-A
805	No	7	7-B	200	McF_6	0	last	singleC	OT-A
806	No	7	7-B	200	McF_6	0	last	wholeT_0.01	OT-A
807	No	7	7-B	200	McF_6	0	last	wholeT_0.5	OT-A
808	No	7	7-B	200	McF_6	0	unif	singleC	OT, OT-A
809	No	7	7-B	200	McF_6	0	unif	wholeT_0.01	OT, OT-A
810	No	7	7-B	200	McF_6	0	unif	wholeT_0.5	CBN-A, OT, OT-A
811	No	7	7-B	200	McF_6	Inf	last	singleC	OT-A
812	No	7	7-B	200	McF_6	Inf	last	wholeT_0.01	OT-A
813	No	7	7-B	200	McF_6	Inf	last	wholeT_0.5	OT-A
814	No	7	7-B	200	McF_6	Inf	unif	singleC	OT, OT-A
815	No	7	7-B	200	McF_6	Inf	unif	wholeT_0.01	CBN-A, OT, OT-A
816	No	7	7-B	200	McF_6	Inf	unif	wholeT_0.5	CBN-A, OT, OT-A
817	No	7	7-B	100	Bozic	0	last	singleC	OT-A
818	No	7	7-B	100	Bozic	0	last	wholeT_0.01	OT-A
819	No	7	7-B	100	Bozic	0	last	wholeT_0.5	OT-A
820	No	7	7-B	100	Bozic	0	unif	singleC	OT, OT-A
821	No	7	7-B	100	Bozic	0	unif	wholeT_0.01	CBN-A, OT, OT-A
822	No	7	7-B	100	Bozic	0	unif	wholeT_0.5	OT, OT-A
823	No	7	7-B	100	Bozic	Inf	last	singleC	OT-A
824	No	7	7-B	100	Bozic	Inf	last	wholeT_0.01	OT-A
825	No	7	7-B	100	Bozic	Inf	last	wholeT_0.5	OT-A
826	No	7	7-B	100	Bozic	Inf	unif	singleC	CBN, CBN-A, OT, OT-A
827	No	7	7-B	100	Bozic	Inf	unif	wholeT_0.01	OT, OT-A
828	No	7	7-B	100	Bozic	Inf	unif	wholeT_0.5	CBN, CBN-A, OT, OT-A
829	No	7	7-B	100	exp	0	last	singleC	OT-A
830	No	7	7-B	100	exp	0	last	wholeT_0.01	OT-A
831	No	7	7-B	100	exp	0	last	wholeT_0.5	OT-A
832	No	7	7-B	100	exp	0	unif	singleC	OT, OT-A
833	No	7	7-B	100	exp	0	unif	wholeT_0.01	OT, OT-A
834	No	7	7-B	100	exp	0	unif	wholeT_0.5	OT, OT-A
835	No	7	7-B	100	exp	Inf	last	singleC	OT-A
836	No	7	7-B	100	exp	Inf	last	wholeT_0.01	OT-A
837	No	7	7-B	100	exp	Inf	last	wholeT_0.5	OT-A

Table 1: *(continued)*

	Conjunction	Drivers	Tree	S.Size	Model	sh	S.Time	S.Type	Best method(s)
838	No	7	7-B	100	exp	Inf	unif	singleC	OT, OT-A
839	No	7	7-B	100	exp	Inf	unif	wholeT_0.01	CBN, OT, OT-A
840	No	7	7-B	100	exp	Inf	unif	wholeT_0.5	OT, OT-A
841	No	7	7-B	100	McF_4	0	last	singleC	OT-A
842	No	7	7-B	100	McF_4	0	last	wholeT_0.01	OT-A
843	No	7	7-B	100	McF_4	0	last	wholeT_0.5	OT-A
844	No	7	7-B	100	McF_4	0	unif	singleC	OT, OT-A
845	No	7	7-B	100	McF_4	0	unif	wholeT_0.01	OT, OT-A
846	No	7	7-B	100	McF_4	0	unif	wholeT_0.5	OT, OT-A
847	No	7	7-B	100	McF_4	Inf	last	singleC	OT-A
848	No	7	7-B	100	McF_4	Inf	last	wholeT_0.01	OT-A
849	No	7	7-B	100	McF_4	Inf	last	wholeT_0.5	OT-A
850	No	7	7-B	100	McF_4	Inf	unif	singleC	OT, OT-A
851	No	7	7-B	100	McF_4	Inf	unif	wholeT_0.01	OT, OT-A
852	No	7	7-B	100	McF_4	Inf	unif	wholeT_0.5	OT, OT-A
853	No	7	7-B	100	McF_6	0	last	singleC	OT-A
854	No	7	7-B	100	McF_6	0	last	wholeT_0.01	OT-A
855	No	7	7-B	100	McF_6	0	last	wholeT_0.5	OT-A
856	No	7	7-B	100	McF_6	0	unif	singleC	CBN-A, OT, OT-A
857	No	7	7-B	100	McF_6	0	unif	wholeT_0.01	CBN-A, OT, OT-A
858	No	7	7-B	100	McF_6	0	unif	wholeT_0.5	CBN-A, OT, OT-A
859	No	7	7-B	100	McF_6	Inf	last	singleC	OT-A
860	No	7	7-B	100	McF_6	Inf	last	wholeT_0.01	OT-A
861	No	7	7-B	100	McF_6	Inf	last	wholeT_0.5	OT-A
862	No	7	7-B	100	McF_6	Inf	unif	singleC	CBN-A, OT-A
863	No	7	7-B	100	McF_6	Inf	unif	wholeT_0.01	CBN-A, OT-A
864	No	7	7-B	100	McF_6	Inf	unif	wholeT_0.5	CBN-A, OT, OT-A

2.2 Confidence sets (MCB-2), PFD, Drivers Known

Table 2: Confidence sets (method MCB-2) when Drivers are Known for measure PFD.

	Conjunction	Drivers	Tree	S.Size	Model	sh	S.Time	S.Type	Best method(s)
1	Yes	11	11-A	1000	Bozic	0	last	singleC	OT, OT-A
2	Yes	11	11-A	1000	Bozic	0	last	wholeT_0.01	DiP, DiP-A
3	Yes	11	11-A	1000	Bozic	0	last	wholeT_0.5	OT, OT-A
4	Yes	11	11-A	1000	Bozic	0	unif	singleC	DiP, DiP-A
5	Yes	11	11-A	1000	Bozic	0	unif	wholeT_0.01	DiP-A, OT, OT-A
6	Yes	11	11-A	1000	Bozic	0	unif	wholeT_0.5	OT, OT-A
7	Yes	11	11-A	1000	Bozic	Inf	last	singleC	OT, OT-A
8	Yes	11	11-A	1000	Bozic	Inf	last	wholeT_0.01	DiP, DiP-A
9	Yes	11	11-A	1000	Bozic	Inf	last	wholeT_0.5	OT, OT-A
10	Yes	11	11-A	1000	Bozic	Inf	unif	singleC	OT, OT-A
11	Yes	11	11-A	1000	Bozic	Inf	unif	wholeT_0.01	OT, OT-A
12	Yes	11	11-A	1000	Bozic	Inf	unif	wholeT_0.5	OT, OT-A
13	Yes	11	11-A	1000	exp	0	last	singleC	OT, OT-A
14	Yes	11	11-A	1000	exp	0	last	wholeT_0.01	DiP-A, OT, OT-A
15	Yes	11	11-A	1000	exp	0	last	wholeT_0.5	OT, OT-A
16	Yes	11	11-A	1000	exp	0	unif	singleC	OT, OT-A
17	Yes	11	11-A	1000	exp	0	unif	wholeT_0.01	DiP-A
18	Yes	11	11-A	1000	exp	0	unif	wholeT_0.5	OT, OT-A
19	Yes	11	11-A	1000	exp	Inf	last	singleC	OT, OT-A
20	Yes	11	11-A	1000	exp	Inf	last	wholeT_0.01	OT, OT-A
21	Yes	11	11-A	1000	exp	Inf	last	wholeT_0.5	OT, OT-A
22	Yes	11	11-A	1000	exp	Inf	unif	singleC	OT, OT-A
23	Yes	11	11-A	1000	exp	Inf	unif	wholeT_0.01	OT, OT-A
24	Yes	11	11-A	1000	exp	Inf	unif	wholeT_0.5	OT, OT-A
25	Yes	11	11-A	1000	McF_4	0	last	singleC	DiP-A, OT, OT-A
26	Yes	11	11-A	1000	McF_4	0	last	wholeT_0.01	DiP-A, OT, OT-A
27	Yes	11	11-A	1000	McF_4	0	last	wholeT_0.5	DiP-A, OT, OT-A
28	Yes	11	11-A	1000	McF_4	0	unif	singleC	DiP, DiP-A, OT, OT-A
29	Yes	11	11-A	1000	McF_4	0	unif	wholeT_0.01	DiP-A
30	Yes	11	11-A	1000	McF_4	0	unif	wholeT_0.5	DiP, DiP-A
31	Yes	11	11-A	1000	McF_4	Inf	last	singleC	OT, OT-A
32	Yes	11	11-A	1000	McF_4	Inf	last	wholeT_0.01	OT, OT-A
33	Yes	11	11-A	1000	McF_4	Inf	last	wholeT_0.5	DiP-A, OT, OT-A
34	Yes	11	11-A	1000	McF_4	Inf	unif	singleC	DiP, DiP-A, OT, OT-A
35	Yes	11	11-A	1000	McF_4	Inf	unif	wholeT_0.01	DiP-A, OT, OT-A
36	Yes	11	11-A	1000	McF_4	Inf	unif	wholeT_0.5	DiP, DiP-A, OT, OT-A
37	Yes	11	11-A	1000	McF_6	0	last	singleC	DiP, OT

Table 2: *(continued)*

	Conjunction	Drivers	Tree	S.Size	Model	sh	S.Time	S.Type	Best method(s)
38	Yes	11	11-A	1000	McF_6	0	last	wholeT_0.01	DiP, OT
39	Yes	11	11-A	1000	McF_6	0	last	wholeT_0.5	DiP, OT
40	Yes	11	11-A	1000	McF_6	0	unif	singleC	DiP, DiP-A, OT, OT-A
41	Yes	11	11-A	1000	McF_6	0	unif	wholeT_0.01	DiP, DiP-A, OT, OT-A
42	Yes	11	11-A	1000	McF_6	0	unif	wholeT_0.5	DiP, DiP-A
43	Yes	11	11-A	1000	McF_6	Inf	last	singleC	DiP, OT
44	Yes	11	11-A	1000	McF_6	Inf	last	wholeT_0.01	DiP, OT
45	Yes	11	11-A	1000	McF_6	Inf	last	wholeT_0.5	DiP, OT
46	Yes	11	11-A	1000	McF_6	Inf	unif	singleC	DiP, DiP-A
47	Yes	11	11-A	1000	McF_6	Inf	unif	wholeT_0.01	DiP, DiP-A
48	Yes	11	11-A	1000	McF_6	Inf	unif	wholeT_0.5	DiP, DiP-A
49	Yes	11	11-A	200	Bozic	0	last	singleC	OT, OT-A
50	Yes	11	11-A	200	Bozic	0	last	wholeT_0.01	OT, OT-A
51	Yes	11	11-A	200	Bozic	0	last	wholeT_0.5	OT, OT-A
52	Yes	11	11-A	200	Bozic	0	unif	singleC	OT, OT-A
53	Yes	11	11-A	200	Bozic	0	unif	wholeT_0.01	DiP, DiP-A, OT, OT-A
54	Yes	11	11-A	200	Bozic	0	unif	wholeT_0.5	OT, OT-A
55	Yes	11	11-A	200	Bozic	Inf	last	singleC	OT, OT-A
56	Yes	11	11-A	200	Bozic	Inf	last	wholeT_0.01	OT, OT-A
57	Yes	11	11-A	200	Bozic	Inf	last	wholeT_0.5	OT, OT-A
58	Yes	11	11-A	200	Bozic	Inf	unif	singleC	OT, OT-A
59	Yes	11	11-A	200	Bozic	Inf	unif	wholeT_0.01	OT, OT-A
60	Yes	11	11-A	200	Bozic	Inf	unif	wholeT_0.5	OT, OT-A
61	Yes	11	11-A	200	exp	0	last	singleC	OT, OT-A
62	Yes	11	11-A	200	exp	0	last	wholeT_0.01	OT, OT-A
63	Yes	11	11-A	200	exp	0	last	wholeT_0.5	OT, OT-A
64	Yes	11	11-A	200	exp	0	unif	singleC	OT, OT-A
65	Yes	11	11-A	200	exp	0	unif	wholeT_0.01	OT, OT-A
66	Yes	11	11-A	200	exp	0	unif	wholeT_0.5	CBN, CBN-A
67	Yes	11	11-A	200	exp	Inf	last	singleC	OT, OT-A
68	Yes	11	11-A	200	exp	Inf	last	wholeT_0.01	OT, OT-A
69	Yes	11	11-A	200	exp	Inf	last	wholeT_0.5	OT, OT-A
70	Yes	11	11-A	200	exp	Inf	unif	singleC	OT, OT-A
71	Yes	11	11-A	200	exp	Inf	unif	wholeT_0.01	OT, OT-A
72	Yes	11	11-A	200	exp	Inf	unif	wholeT_0.5	OT, OT-A
73	Yes	11	11-A	200	McF_4	0	last	singleC	DiP-A, OT, OT-A
74	Yes	11	11-A	200	McF_4	0	last	wholeT_0.01	DiP-A, OT, OT-A
75	Yes	11	11-A	200	McF_4	0	last	wholeT_0.5	DiP-A, OT, OT-A
76	Yes	11	11-A	200	McF_4	0	unif	singleC	OT, OT-A
77	Yes	11	11-A	200	McF_4	0	unif	wholeT_0.01	DiP, DiP-A, OT, OT-A

Table 2: *(continued)*

	Conjunction	Drivers	Tree	S.Size	Model	sh	S.Time	S.Type	Best method(s)
78	Yes	11	11-A	200	McF_4	0	unif	wholeT_0.5	OT, OT-A
79	Yes	11	11-A	200	McF_4	Inf	last	singleC	OT, OT-A
80	Yes	11	11-A	200	McF_4	Inf	last	wholeT_0.01	OT, OT-A
81	Yes	11	11-A	200	McF_4	Inf	last	wholeT_0.5	OT, OT-A
82	Yes	11	11-A	200	McF_4	Inf	unif	singleC	OT, OT-A
83	Yes	11	11-A	200	McF_4	Inf	unif	wholeT_0.01	OT, OT-A
84	Yes	11	11-A	200	McF_4	Inf	unif	wholeT_0.5	OT, OT-A
85	Yes	11	11-A	200	McF_6	0	last	singleC	DiP, OT
86	Yes	11	11-A	200	McF_6	0	last	wholeT_0.01	DiP, OT
87	Yes	11	11-A	200	McF_6	0	last	wholeT_0.5	DiP, OT
88	Yes	11	11-A	200	McF_6	0	unif	singleC	OT, OT-A
89	Yes	11	11-A	200	McF_6	0	unif	wholeT_0.01	DiP, DiP-A, OT, OT-A
90	Yes	11	11-A	200	McF_6	0	unif	wholeT_0.5	OT, OT-A
91	Yes	11	11-A	200	McF_6	Inf	last	singleC	OT
92	Yes	11	11-A	200	McF_6	Inf	last	wholeT_0.01	OT
93	Yes	11	11-A	200	McF_6	Inf	last	wholeT_0.5	OT
94	Yes	11	11-A	200	McF_6	Inf	unif	singleC	OT, OT-A
95	Yes	11	11-A	200	McF_6	Inf	unif	wholeT_0.01	OT, OT-A
96	Yes	11	11-A	200	McF_6	Inf	unif	wholeT_0.5	OT, OT-A
97	Yes	11	11-A	100	Bozic	0	last	singleC	OT, OT-A
98	Yes	11	11-A	100	Bozic	0	last	wholeT_0.01	OT, OT-A
99	Yes	11	11-A	100	Bozic	0	last	wholeT_0.5	OT, OT-A
100	Yes	11	11-A	100	Bozic	0	unif	singleC	OT, OT-A
101	Yes	11	11-A	100	Bozic	0	unif	wholeT_0.01	OT, OT-A
102	Yes	11	11-A	100	Bozic	0	unif	wholeT_0.5	CBN, CBN-A
103	Yes	11	11-A	100	Bozic	Inf	last	singleC	OT, OT-A
104	Yes	11	11-A	100	Bozic	Inf	last	wholeT_0.01	OT, OT-A
105	Yes	11	11-A	100	Bozic	Inf	last	wholeT_0.5	OT, OT-A
106	Yes	11	11-A	100	Bozic	Inf	unif	singleC	OT, OT-A
107	Yes	11	11-A	100	Bozic	Inf	unif	wholeT_0.01	OT, OT-A
108	Yes	11	11-A	100	Bozic	Inf	unif	wholeT_0.5	OT, OT-A
109	Yes	11	11-A	100	exp	0	last	singleC	OT, OT-A
110	Yes	11	11-A	100	exp	0	last	wholeT_0.01	OT, OT-A
111	Yes	11	11-A	100	exp	0	last	wholeT_0.5	OT, OT-A
112	Yes	11	11-A	100	exp	0	unif	singleC	CBN, CBN-A
113	Yes	11	11-A	100	exp	0	unif	wholeT_0.01	OT, OT-A
114	Yes	11	11-A	100	exp	0	unif	wholeT_0.5	CBN, CBN-A
115	Yes	11	11-A	100	exp	Inf	last	singleC	OT, OT-A
116	Yes	11	11-A	100	exp	Inf	last	wholeT_0.01	OT, OT-A
117	Yes	11	11-A	100	exp	Inf	last	wholeT_0.5	OT, OT-A

Table 2: *(continued)*

	Conjunction	Drivers	Tree	S.Size	Model	sh	S.Time	S.Type	Best method(s)
118	Yes	11	11-A	100	exp	Inf	unif	singleC	OT, OT-A
119	Yes	11	11-A	100	exp	Inf	unif	wholeT_0.01	OT, OT-A
120	Yes	11	11-A	100	exp	Inf	unif	wholeT_0.5	OT, OT-A
121	Yes	11	11-A	100	McF_4	0	last	singleC	OT, OT-A
122	Yes	11	11-A	100	McF_4	0	last	wholeT_0.01	DiP, DiP-A, OT, OT-A
123	Yes	11	11-A	100	McF_4	0	last	wholeT_0.5	OT, OT-A
124	Yes	11	11-A	100	McF_4	0	unif	singleC	OT, OT-A
125	Yes	11	11-A	100	McF_4	0	unif	wholeT_0.01	DiP, DiP-A, OT, OT-A
126	Yes	11	11-A	100	McF_4	0	unif	wholeT_0.5	OT, OT-A
127	Yes	11	11-A	100	McF_4	Inf	last	singleC	OT, OT-A
128	Yes	11	11-A	100	McF_4	Inf	last	wholeT_0.01	OT, OT-A
129	Yes	11	11-A	100	McF_4	Inf	last	wholeT_0.5	OT, OT-A
130	Yes	11	11-A	100	McF_4	Inf	unif	singleC	OT, OT-A
131	Yes	11	11-A	100	McF_4	Inf	unif	wholeT_0.01	OT, OT-A
132	Yes	11	11-A	100	McF_4	Inf	unif	wholeT_0.5	OT, OT-A
133	Yes	11	11-A	100	McF_6	0	last	singleC	DiP, OT
134	Yes	11	11-A	100	McF_6	0	last	wholeT_0.01	DiP, OT
135	Yes	11	11-A	100	McF_6	0	last	wholeT_0.5	OT
136	Yes	11	11-A	100	McF_6	0	unif	singleC	OT, OT-A
137	Yes	11	11-A	100	McF_6	0	unif	wholeT_0.01	DiP, DiP-A, OT, OT-A
138	Yes	11	11-A	100	McF_6	0	unif	wholeT_0.5	OT, OT-A
139	Yes	11	11-A	100	McF_6	Inf	last	singleC	OT
140	Yes	11	11-A	100	McF_6	Inf	last	wholeT_0.01	OT
141	Yes	11	11-A	100	McF_6	Inf	last	wholeT_0.5	OT
142	Yes	11	11-A	100	McF_6	Inf	unif	singleC	OT, OT-A
143	Yes	11	11-A	100	McF_6	Inf	unif	wholeT_0.01	OT, OT-A
144	Yes	11	11-A	100	McF_6	Inf	unif	wholeT_0.5	OT, OT-A
145	Yes	9	9-A	1000	Bozic	0	last	singleC	OT, OT-A
146	Yes	9	9-A	1000	Bozic	0	last	wholeT_0.01	DiP, DiP-A
147	Yes	9	9-A	1000	Bozic	0	last	wholeT_0.5	OT, OT-A
148	Yes	9	9-A	1000	Bozic	0	unif	singleC	OT, OT-A
149	Yes	9	9-A	1000	Bozic	0	unif	wholeT_0.01	DiP, DiP-A
150	Yes	9	9-A	1000	Bozic	0	unif	wholeT_0.5	OT, OT-A
151	Yes	9	9-A	1000	Bozic	Inf	last	singleC	OT, OT-A
152	Yes	9	9-A	1000	Bozic	Inf	last	wholeT_0.01	DiP, DiP-A, OT, OT-A
153	Yes	9	9-A	1000	Bozic	Inf	last	wholeT_0.5	OT, OT-A
154	Yes	9	9-A	1000	Bozic	Inf	unif	singleC	DiP, DiP-A, OT, OT-A
155	Yes	9	9-A	1000	Bozic	Inf	unif	wholeT_0.01	DiP, DiP-A, OT, OT-A
156	Yes	9	9-A	1000	Bozic	Inf	unif	wholeT_0.5	DiP-A, OT, OT-A
157	Yes	9	9-A	1000	exp	0	last	singleC	OT, OT-A

Table 2: *(continued)*

	Conjunction	Drivers	Tree	S.Size	Model	sh	S.Time	S.Type	Best method(s)
158	Yes	9	9-A	1000	exp	0	last	wholeT_0.01	DiP-A, OT, OT-A
159	Yes	9	9-A	1000	exp	0	last	wholeT_0.5	OT, OT-A
160	Yes	9	9-A	1000	exp	0	unif	singleC	OT, OT-A
161	Yes	9	9-A	1000	exp	0	unif	wholeT_0.01	OT, OT-A
162	Yes	9	9-A	1000	exp	0	unif	wholeT_0.5	CBN, CBN-A
163	Yes	9	9-A	1000	exp	Inf	last	singleC	OT, OT-A
164	Yes	9	9-A	1000	exp	Inf	last	wholeT_0.01	CBN, DiP, DiP-A, OT, OT-A
165	Yes	9	9-A	1000	exp	Inf	last	wholeT_0.5	OT, OT-A
166	Yes	9	9-A	1000	exp	Inf	unif	singleC	OT, OT-A
167	Yes	9	9-A	1000	exp	Inf	unif	wholeT_0.01	OT, OT-A
168	Yes	9	9-A	1000	exp	Inf	unif	wholeT_0.5	OT, OT-A
169	Yes	9	9-A	1000	McF_4	0	last	singleC	DiP, DiP-A, OT, OT-A
170	Yes	9	9-A	1000	McF_4	0	last	wholeT_0.01	DiP, DiP-A, OT, OT-A
171	Yes	9	9-A	1000	McF_4	0	last	wholeT_0.5	DiP, DiP-A, OT, OT-A
172	Yes	9	9-A	1000	McF_4	0	unif	singleC	DiP, DiP-A, OT, OT-A
173	Yes	9	9-A	1000	McF_4	0	unif	wholeT_0.01	DiP, DiP-A
174	Yes	9	9-A	1000	McF_4	0	unif	wholeT_0.5	DiP, DiP-A, OT, OT-A
175	Yes	9	9-A	1000	McF_4	Inf	last	singleC	OT, OT-A
176	Yes	9	9-A	1000	McF_4	Inf	last	wholeT_0.01	DiP, OT
177	Yes	9	9-A	1000	McF_4	Inf	last	wholeT_0.5	DiP, DiP-A, OT, OT-A
178	Yes	9	9-A	1000	McF_4	Inf	unif	singleC	DiP, DiP-A, OT, OT-A
179	Yes	9	9-A	1000	McF_4	Inf	unif	wholeT_0.01	OT, OT-A
180	Yes	9	9-A	1000	McF_4	Inf	unif	wholeT_0.5	DiP, DiP-A, OT, OT-A
181	Yes	9	9-A	1000	McF_6	0	last	singleC	OT
182	Yes	9	9-A	1000	McF_6	0	last	wholeT_0.01	DiP, OT
183	Yes	9	9-A	1000	McF_6	0	last	wholeT_0.5	DiP, OT
184	Yes	9	9-A	1000	McF_6	0	unif	singleC	DiP, DiP-A, OT, OT-A
185	Yes	9	9-A	1000	McF_6	0	unif	wholeT_0.01	DiP, DiP-A, OT, OT-A
186	Yes	9	9-A	1000	McF_6	0	unif	wholeT_0.5	DiP, DiP-A, OT, OT-A
187	Yes	9	9-A	1000	McF_6	Inf	last	singleC	DiP, OT
188	Yes	9	9-A	1000	McF_6	Inf	last	wholeT_0.01	OT
189	Yes	9	9-A	1000	McF_6	Inf	last	wholeT_0.5	DiP, OT
190	Yes	9	9-A	1000	McF_6	Inf	unif	singleC	DiP, DiP-A, OT, OT-A
191	Yes	9	9-A	1000	McF_6	Inf	unif	wholeT_0.01	DiP, DiP-A, OT, OT-A
192	Yes	9	9-A	1000	McF_6	Inf	unif	wholeT_0.5	DiP, DiP-A, OT, OT-A
193	Yes	9	9-A	200	Bozic	0	last	singleC	OT, OT-A
194	Yes	9	9-A	200	Bozic	0	last	wholeT_0.01	OT, OT-A
195	Yes	9	9-A	200	Bozic	0	last	wholeT_0.5	OT, OT-A
196	Yes	9	9-A	200	Bozic	0	unif	singleC	CBN
197	Yes	9	9-A	200	Bozic	0	unif	wholeT_0.01	DiP-A, OT, OT-A

Table 2: *(continued)*

	Conjunction	Drivers	Tree	S.Size	Model	sh	S.Time	S.Type	Best method(s)
198	Yes	9	9-A	200	Bozic	0	unif	wholeT_0.5	CBN, CBN-A
199	Yes	9	9-A	200	Bozic	Inf	last	singleC	OT, OT-A
200	Yes	9	9-A	200	Bozic	Inf	last	wholeT_0.01	OT, OT-A
201	Yes	9	9-A	200	Bozic	Inf	last	wholeT_0.5	OT, OT-A
202	Yes	9	9-A	200	Bozic	Inf	unif	singleC	OT, OT-A
203	Yes	9	9-A	200	Bozic	Inf	unif	wholeT_0.01	OT, OT-A
204	Yes	9	9-A	200	Bozic	Inf	unif	wholeT_0.5	OT, OT-A
205	Yes	9	9-A	200	exp	0	last	singleC	OT, OT-A
206	Yes	9	9-A	200	exp	0	last	wholeT_0.01	OT, OT-A
207	Yes	9	9-A	200	exp	0	last	wholeT_0.5	OT, OT-A
208	Yes	9	9-A	200	exp	0	unif	singleC	CBN-A
209	Yes	9	9-A	200	exp	0	unif	wholeT_0.01	OT, OT-A
210	Yes	9	9-A	200	exp	0	unif	wholeT_0.5	CBN, CBN-A
211	Yes	9	9-A	200	exp	Inf	last	singleC	OT, OT-A
212	Yes	9	9-A	200	exp	Inf	last	wholeT_0.01	OT, OT-A
213	Yes	9	9-A	200	exp	Inf	last	wholeT_0.5	OT, OT-A
214	Yes	9	9-A	200	exp	Inf	unif	singleC	OT, OT-A
215	Yes	9	9-A	200	exp	Inf	unif	wholeT_0.01	CBN-A, OT, OT-A
216	Yes	9	9-A	200	exp	Inf	unif	wholeT_0.5	OT, OT-A
217	Yes	9	9-A	200	McF_4	0	last	singleC	DiP-A, OT, OT-A
218	Yes	9	9-A	200	McF_4	0	last	wholeT_0.01	DiP, DiP-A
219	Yes	9	9-A	200	McF_4	0	last	wholeT_0.5	DiP-A, OT, OT-A
220	Yes	9	9-A	200	McF_4	0	unif	singleC	OT, OT-A
221	Yes	9	9-A	200	McF_4	0	unif	wholeT_0.01	DiP, DiP-A
222	Yes	9	9-A	200	McF_4	0	unif	wholeT_0.5	OT, OT-A
223	Yes	9	9-A	200	McF_4	Inf	last	singleC	OT, OT-A
224	Yes	9	9-A	200	McF_4	Inf	last	wholeT_0.01	OT
225	Yes	9	9-A	200	McF_4	Inf	last	wholeT_0.5	OT, OT-A
226	Yes	9	9-A	200	McF_4	Inf	unif	singleC	OT, OT-A
227	Yes	9	9-A	200	McF_4	Inf	unif	wholeT_0.01	OT, OT-A
228	Yes	9	9-A	200	McF_4	Inf	unif	wholeT_0.5	OT, OT-A
229	Yes	9	9-A	200	McF_6	0	last	singleC	DiP, OT
230	Yes	9	9-A	200	McF_6	0	last	wholeT_0.01	DiP, OT
231	Yes	9	9-A	200	McF_6	0	last	wholeT_0.5	OT
232	Yes	9	9-A	200	McF_6	0	unif	singleC	DiP, DiP-A, OT, OT-A
233	Yes	9	9-A	200	McF_6	0	unif	wholeT_0.01	DiP, DiP-A, OT, OT-A
234	Yes	9	9-A	200	McF_6	0	unif	wholeT_0.5	DiP, DiP-A, OT, OT-A
235	Yes	9	9-A	200	McF_6	Inf	last	singleC	OT
236	Yes	9	9-A	200	McF_6	Inf	last	wholeT_0.01	OT
237	Yes	9	9-A	200	McF_6	Inf	last	wholeT_0.5	OT

Table 2: *(continued)*

	Conjunction	Drivers	Tree	S.Size	Model	sh	S.Time	S.Type	Best method(s)
238	Yes	9	9-A	200	McF_6	Inf	unif	singleC	DiP, DiP-A, OT, OT-A
239	Yes	9	9-A	200	McF_6	Inf	unif	wholeT_0.01	DiP, DiP-A, OT, OT-A
240	Yes	9	9-A	200	McF_6	Inf	unif	wholeT_0.5	DiP, DiP-A, OT, OT-A
241	Yes	9	9-A	100	Bozic	0	last	singleC	OT, OT-A
242	Yes	9	9-A	100	Bozic	0	last	wholeT_0.01	OT, OT-A
243	Yes	9	9-A	100	Bozic	0	last	wholeT_0.5	OT, OT-A
244	Yes	9	9-A	100	Bozic	0	unif	singleC	CBN, CBN-A
245	Yes	9	9-A	100	Bozic	0	unif	wholeT_0.01	OT, OT-A
246	Yes	9	9-A	100	Bozic	0	unif	wholeT_0.5	CBN, CBN-A
247	Yes	9	9-A	100	Bozic	Inf	last	singleC	OT, OT-A
248	Yes	9	9-A	100	Bozic	Inf	last	wholeT_0.01	OT, OT-A
249	Yes	9	9-A	100	Bozic	Inf	last	wholeT_0.5	OT, OT-A
250	Yes	9	9-A	100	Bozic	Inf	unif	singleC	OT, OT-A
251	Yes	9	9-A	100	Bozic	Inf	unif	wholeT_0.01	OT, OT-A
252	Yes	9	9-A	100	Bozic	Inf	unif	wholeT_0.5	OT, OT-A
253	Yes	9	9-A	100	exp	0	last	singleC	OT, OT-A
254	Yes	9	9-A	100	exp	0	last	wholeT_0.01	OT, OT-A
255	Yes	9	9-A	100	exp	0	last	wholeT_0.5	OT, OT-A
256	Yes	9	9-A	100	exp	0	unif	singleC	CBN, CBN-A
257	Yes	9	9-A	100	exp	0	unif	wholeT_0.01	OT, OT-A
258	Yes	9	9-A	100	exp	0	unif	wholeT_0.5	CBN, CBN-A
259	Yes	9	9-A	100	exp	Inf	last	singleC	OT, OT-A
260	Yes	9	9-A	100	exp	Inf	last	wholeT_0.01	OT, OT-A
261	Yes	9	9-A	100	exp	Inf	last	wholeT_0.5	OT, OT-A
262	Yes	9	9-A	100	exp	Inf	unif	singleC	OT, OT-A
263	Yes	9	9-A	100	exp	Inf	unif	wholeT_0.01	OT, OT-A
264	Yes	9	9-A	100	exp	Inf	unif	wholeT_0.5	OT, OT-A
265	Yes	9	9-A	100	McF_4	0	last	singleC	OT, OT-A
266	Yes	9	9-A	100	McF_4	0	last	wholeT_0.01	DiP, DiP-A
267	Yes	9	9-A	100	McF_4	0	last	wholeT_0.5	OT, OT-A
268	Yes	9	9-A	100	McF_4	0	unif	singleC	OT, OT-A
269	Yes	9	9-A	100	McF_4	0	unif	wholeT_0.01	OT, OT-A
270	Yes	9	9-A	100	McF_4	0	unif	wholeT_0.5	OT, OT-A
271	Yes	9	9-A	100	McF_4	Inf	last	singleC	OT, OT-A
272	Yes	9	9-A	100	McF_4	Inf	last	wholeT_0.01	OT
273	Yes	9	9-A	100	McF_4	Inf	last	wholeT_0.5	OT, OT-A
274	Yes	9	9-A	100	McF_4	Inf	unif	singleC	OT, OT-A
275	Yes	9	9-A	100	McF_4	Inf	unif	wholeT_0.01	OT, OT-A
276	Yes	9	9-A	100	McF_4	Inf	unif	wholeT_0.5	OT, OT-A
277	Yes	9	9-A	100	McF_6	0	last	singleC	OT

Table 2: *(continued)*

	Conjunction	Drivers	Tree	S.Size	Model	sh	S.Time	S.Type	Best method(s)
278	Yes	9	9-A	100	McF_6	0	last	wholeT_0.01	OT
279	Yes	9	9-A	100	McF_6	0	last	wholeT_0.5	OT
280	Yes	9	9-A	100	McF_6	0	unif	singleC	OT
281	Yes	9	9-A	100	McF_6	0	unif	wholeT_0.01	DiP, DiP-A, OT, OT-A
282	Yes	9	9-A	100	McF_6	0	unif	wholeT_0.5	OT, OT-A
283	Yes	9	9-A	100	McF_6	Inf	last	singleC	OT
284	Yes	9	9-A	100	McF_6	Inf	last	wholeT_0.01	OT
285	Yes	9	9-A	100	McF_6	Inf	last	wholeT_0.5	OT
286	Yes	9	9-A	100	McF_6	Inf	unif	singleC	OT, OT-A
287	Yes	9	9-A	100	McF_6	Inf	unif	wholeT_0.01	OT, OT-A
288	Yes	9	9-A	100	McF_6	Inf	unif	wholeT_0.5	OT, OT-A
289	Yes	7	7-A	1000	Bozic	0	last	singleC	OT-A
290	Yes	7	7-A	1000	Bozic	0	last	wholeT_0.01	DiP-A
291	Yes	7	7-A	1000	Bozic	0	last	wholeT_0.5	OT-A
292	Yes	7	7-A	1000	Bozic	0	unif	singleC	OT, OT-A
293	Yes	7	7-A	1000	Bozic	0	unif	wholeT_0.01	CBN, CBN-A
294	Yes	7	7-A	1000	Bozic	0	unif	wholeT_0.5	OT, OT-A
295	Yes	7	7-A	1000	Bozic	Inf	last	singleC	DiP-A, OT, OT-A
296	Yes	7	7-A	1000	Bozic	Inf	last	wholeT_0.01	DiP-A, OT, OT-A
297	Yes	7	7-A	1000	Bozic	Inf	last	wholeT_0.5	DiP-A, OT, OT-A
298	Yes	7	7-A	1000	Bozic	Inf	unif	singleC	CBN, CBN-A
299	Yes	7	7-A	1000	Bozic	Inf	unif	wholeT_0.01	CBN, CBN-A, OT, OT-A
300	Yes	7	7-A	1000	Bozic	Inf	unif	wholeT_0.5	CBN, CBN-A
301	Yes	7	7-A	1000	exp	0	last	singleC	OT-A
302	Yes	7	7-A	1000	exp	0	last	wholeT_0.01	OT-A
303	Yes	7	7-A	1000	exp	0	last	wholeT_0.5	OT-A
304	Yes	7	7-A	1000	exp	0	unif	singleC	OT, OT-A
305	Yes	7	7-A	1000	exp	0	unif	wholeT_0.01	CBN, CBN-A
306	Yes	7	7-A	1000	exp	0	unif	wholeT_0.5	OT, OT-A
307	Yes	7	7-A	1000	exp	Inf	last	singleC	OT-A
308	Yes	7	7-A	1000	exp	Inf	last	wholeT_0.01	DiP-A, OT-A
309	Yes	7	7-A	1000	exp	Inf	last	wholeT_0.5	OT-A
310	Yes	7	7-A	1000	exp	Inf	unif	singleC	CBN, CBN-A, OT, OT-A
311	Yes	7	7-A	1000	exp	Inf	unif	wholeT_0.01	CBN, CBN-A
312	Yes	7	7-A	1000	exp	Inf	unif	wholeT_0.5	CBN, CBN-A
313	Yes	7	7-A	1000	McF_4	0	last	singleC	DiP-A, OT, OT-A
314	Yes	7	7-A	1000	McF_4	0	last	wholeT_0.01	OT, OT-A
315	Yes	7	7-A	1000	McF_4	0	last	wholeT_0.5	DiP-A, OT, OT-A
316	Yes	7	7-A	1000	McF_4	0	unif	singleC	DiP, DiP-A
317	Yes	7	7-A	1000	McF_4	0	unif	wholeT_0.01	DiP, DiP-A, OT

Table 2: *(continued)*

	Conjunction	Drivers	Tree	S.Size	Model	sh	S.Time	S.Type	Best method(s)
318	Yes	7	7-A	1000	McF_4	0	unif	wholeT_0.5	DiP, DiP-A
319	Yes	7	7-A	1000	McF_4	Inf	last	singleC	DiP-A, OT, OT-A
320	Yes	7	7-A	1000	McF_4	Inf	last	wholeT_0.01	OT, OT-A
321	Yes	7	7-A	1000	McF_4	Inf	last	wholeT_0.5	DiP-A, OT-A
322	Yes	7	7-A	1000	McF_4	Inf	unif	singleC	DiP-A, OT, OT-A
323	Yes	7	7-A	1000	McF_4	Inf	unif	wholeT_0.01	DiP-A, OT, OT-A
324	Yes	7	7-A	1000	McF_4	Inf	unif	wholeT_0.5	DiP-A, OT, OT-A
325	Yes	7	7-A	1000	McF_6	0	last	singleC	DiP, OT, OT-A
326	Yes	7	7-A	1000	McF_6	0	last	wholeT_0.01	DiP, OT, OT-A
327	Yes	7	7-A	1000	McF_6	0	last	wholeT_0.5	DiP, OT, OT-A
328	Yes	7	7-A	1000	McF_6	0	unif	singleC	CBN, CBN-A, DiP, DiP-A, OT, OT-A
329	Yes	7	7-A	1000	McF_6	0	unif	wholeT_0.01	CBN, CBN-A, DiP, DiP-A, OT, OT-A
330	Yes	7	7-A	1000	McF_6	0	unif	wholeT_0.5	CBN, CBN-A, DiP, DiP-A, OT, OT-A
331	Yes	7	7-A	1000	McF_6	Inf	last	singleC	DiP, DiP-A, OT, OT-A
332	Yes	7	7-A	1000	McF_6	Inf	last	wholeT_0.01	OT, OT-A
333	Yes	7	7-A	1000	McF_6	Inf	last	wholeT_0.5	DiP, OT, OT-A
334	Yes	7	7-A	1000	McF_6	Inf	unif	singleC	CBN, CBN-A, DiP, DiP-A, OT, OT-A
335	Yes	7	7-A	1000	McF_6	Inf	unif	wholeT_0.01	CBN, CBN-A, OT, OT-A
336	Yes	7	7-A	1000	McF_6	Inf	unif	wholeT_0.5	CBN, CBN-A, DiP, DiP-A, OT, OT-A
337	Yes	7	7-A	200	Bozic	0	last	singleC	OT-A
338	Yes	7	7-A	200	Bozic	0	last	wholeT_0.01	OT-A
339	Yes	7	7-A	200	Bozic	0	last	wholeT_0.5	OT-A
340	Yes	7	7-A	200	Bozic	0	unif	singleC	OT, OT-A
341	Yes	7	7-A	200	Bozic	0	unif	wholeT_0.01	CBN, CBN-A
342	Yes	7	7-A	200	Bozic	0	unif	wholeT_0.5	OT, OT-A
343	Yes	7	7-A	200	Bozic	Inf	last	singleC	OT-A
344	Yes	7	7-A	200	Bozic	Inf	last	wholeT_0.01	OT-A
345	Yes	7	7-A	200	Bozic	Inf	last	wholeT_0.5	OT-A
346	Yes	7	7-A	200	Bozic	Inf	unif	singleC	CBN, CBN-A
347	Yes	7	7-A	200	Bozic	Inf	unif	wholeT_0.01	CBN, CBN-A
348	Yes	7	7-A	200	Bozic	Inf	unif	wholeT_0.5	CBN, CBN-A
349	Yes	7	7-A	200	exp	0	last	singleC	OT-A
350	Yes	7	7-A	200	exp	0	last	wholeT_0.01	OT-A
351	Yes	7	7-A	200	exp	0	last	wholeT_0.5	OT-A
352	Yes	7	7-A	200	exp	0	unif	singleC	CBN, CBN-A
353	Yes	7	7-A	200	exp	0	unif	wholeT_0.01	CBN, CBN-A, OT, OT-A
354	Yes	7	7-A	200	exp	0	unif	wholeT_0.5	CBN, CBN-A
355	Yes	7	7-A	200	exp	Inf	last	singleC	OT-A
356	Yes	7	7-A	200	exp	Inf	last	wholeT_0.01	OT-A
357	Yes	7	7-A	200	exp	Inf	last	wholeT_0.5	OT-A

Table 2: *(continued)*

	Conjunction	Drivers	Tree	S.Size	Model	sh	S.Time	S.Type	Best method(s)
358	Yes	7	7-A	200	exp	Inf	unif	singleC	CBN, CBN-A, OT, OT-A
359	Yes	7	7-A	200	exp	Inf	unif	wholeT_0.01	CBN, CBN-A
360	Yes	7	7-A	200	exp	Inf	unif	wholeT_0.5	CBN, CBN-A, OT, OT-A
361	Yes	7	7-A	200	McF_4	0	last	singleC	OT-A
362	Yes	7	7-A	200	McF_4	0	last	wholeT_0.01	OT-A
363	Yes	7	7-A	200	McF_4	0	last	wholeT_0.5	OT-A
364	Yes	7	7-A	200	McF_4	0	unif	singleC	OT, OT-A
365	Yes	7	7-A	200	McF_4	0	unif	wholeT_0.01	DiP-A, OT, OT-A
366	Yes	7	7-A	200	McF_4	0	unif	wholeT_0.5	OT, OT-A
367	Yes	7	7-A	200	McF_4	Inf	last	singleC	OT-A
368	Yes	7	7-A	200	McF_4	Inf	last	wholeT_0.01	OT, OT-A
369	Yes	7	7-A	200	McF_4	Inf	last	wholeT_0.5	OT-A
370	Yes	7	7-A	200	McF_4	Inf	unif	singleC	OT, OT-A
371	Yes	7	7-A	200	McF_4	Inf	unif	wholeT_0.01	OT, OT-A
372	Yes	7	7-A	200	McF_4	Inf	unif	wholeT_0.5	OT, OT-A
373	Yes	7	7-A	200	McF_6	0	last	singleC	OT, OT-A
374	Yes	7	7-A	200	McF_6	0	last	wholeT_0.01	OT, OT-A
375	Yes	7	7-A	200	McF_6	0	last	wholeT_0.5	OT, OT-A
376	Yes	7	7-A	200	McF_6	0	unif	singleC	CBN, CBN-A, DiP-A
377	Yes	7	7-A	200	McF_6	0	unif	wholeT_0.01	CBN, CBN-A, DiP, DiP-A, OT, OT-A
378	Yes	7	7-A	200	McF_6	0	unif	wholeT_0.5	CBN, CBN-A
379	Yes	7	7-A	200	McF_6	Inf	last	singleC	DiP-A, OT, OT-A
380	Yes	7	7-A	200	McF_6	Inf	last	wholeT_0.01	OT, OT-A
381	Yes	7	7-A	200	McF_6	Inf	last	wholeT_0.5	DiP-A, OT, OT-A
382	Yes	7	7-A	200	McF_6	Inf	unif	singleC	CBN, CBN-A
383	Yes	7	7-A	200	McF_6	Inf	unif	wholeT_0.01	CBN, CBN-A
384	Yes	7	7-A	200	McF_6	Inf	unif	wholeT_0.5	CBN, CBN-A
385	Yes	7	7-A	100	Bozic	0	last	singleC	OT-A
386	Yes	7	7-A	100	Bozic	0	last	wholeT_0.01	OT-A
387	Yes	7	7-A	100	Bozic	0	last	wholeT_0.5	OT-A
388	Yes	7	7-A	100	Bozic	0	unif	singleC	OT, OT-A
389	Yes	7	7-A	100	Bozic	0	unif	wholeT_0.01	CBN, CBN-A
390	Yes	7	7-A	100	Bozic	0	unif	wholeT_0.5	OT, OT-A
391	Yes	7	7-A	100	Bozic	Inf	last	singleC	OT-A
392	Yes	7	7-A	100	Bozic	Inf	last	wholeT_0.01	OT-A
393	Yes	7	7-A	100	Bozic	Inf	last	wholeT_0.5	OT-A
394	Yes	7	7-A	100	Bozic	Inf	unif	singleC	CBN, CBN-A
395	Yes	7	7-A	100	Bozic	Inf	unif	wholeT_0.01	CBN, CBN-A
396	Yes	7	7-A	100	Bozic	Inf	unif	wholeT_0.5	CBN, CBN-A, OT, OT-A
397	Yes	7	7-A	100	exp	0	last	singleC	OT-A

Table 2: *(continued)*

	Conjunction	Drivers	Tree	S.Size	Model	sh	S.Time	S.Type	Best method(s)
398	Yes	7	7-A	100	exp	0	last	wholeT_0.01	OT-A
399	Yes	7	7-A	100	exp	0	last	wholeT_0.5	OT-A
400	Yes	7	7-A	100	exp	0	unif	singleC	OT, OT-A
401	Yes	7	7-A	100	exp	0	unif	wholeT_0.01	OT, OT-A
402	Yes	7	7-A	100	exp	0	unif	wholeT_0.5	CBN, CBN-A
403	Yes	7	7-A	100	exp	Inf	last	singleC	OT-A
404	Yes	7	7-A	100	exp	Inf	last	wholeT_0.01	OT-A
405	Yes	7	7-A	100	exp	Inf	last	wholeT_0.5	OT-A
406	Yes	7	7-A	100	exp	Inf	unif	singleC	OT, OT-A
407	Yes	7	7-A	100	exp	Inf	unif	wholeT_0.01	CBN, CBN-A, OT, OT-A
408	Yes	7	7-A	100	exp	Inf	unif	wholeT_0.5	OT, OT-A
409	Yes	7	7-A	100	McF_4	0	last	singleC	OT-A
410	Yes	7	7-A	100	McF_4	0	last	wholeT_0.01	OT-A
411	Yes	7	7-A	100	McF_4	0	last	wholeT_0.5	OT-A
412	Yes	7	7-A	100	McF_4	0	unif	singleC	OT, OT-A
413	Yes	7	7-A	100	McF_4	0	unif	wholeT_0.01	OT, OT-A
414	Yes	7	7-A	100	McF_4	0	unif	wholeT_0.5	OT, OT-A
415	Yes	7	7-A	100	McF_4	Inf	last	singleC	OT-A
416	Yes	7	7-A	100	McF_4	Inf	last	wholeT_0.01	OT, OT-A
417	Yes	7	7-A	100	McF_4	Inf	last	wholeT_0.5	OT-A
418	Yes	7	7-A	100	McF_4	Inf	unif	singleC	OT, OT-A
419	Yes	7	7-A	100	McF_4	Inf	unif	wholeT_0.01	OT, OT-A
420	Yes	7	7-A	100	McF_4	Inf	unif	wholeT_0.5	OT, OT-A
421	Yes	7	7-A	100	McF_6	0	last	singleC	OT, OT-A
422	Yes	7	7-A	100	McF_6	0	last	wholeT_0.01	OT, OT-A
423	Yes	7	7-A	100	McF_6	0	last	wholeT_0.5	OT, OT-A
424	Yes	7	7-A	100	McF_6	0	unif	singleC	CBN, CBN-A
425	Yes	7	7-A	100	McF_6	0	unif	wholeT_0.01	CBN, CBN-A, DiP-A
426	Yes	7	7-A	100	McF_6	0	unif	wholeT_0.5	CBN, CBN-A
427	Yes	7	7-A	100	McF_6	Inf	last	singleC	OT, OT-A
428	Yes	7	7-A	100	McF_6	Inf	last	wholeT_0.01	OT, OT-A
429	Yes	7	7-A	100	McF_6	Inf	last	wholeT_0.5	OT, OT-A
430	Yes	7	7-A	100	McF_6	Inf	unif	singleC	CBN, CBN-A
431	Yes	7	7-A	100	McF_6	Inf	unif	wholeT_0.01	CBN, CBN-A
432	Yes	7	7-A	100	McF_6	Inf	unif	wholeT_0.5	CBN, CBN-A
433	No	11	11-B	1000	Bozic	0	last	singleC	OT, OT-A
434	No	11	11-B	1000	Bozic	0	last	wholeT_0.01	DiP, DiP-A, OT, OT-A
435	No	11	11-B	1000	Bozic	0	last	wholeT_0.5	OT, OT-A
436	No	11	11-B	1000	Bozic	0	unif	singleC	DiP, DiP-A, OT, OT-A
437	No	11	11-B	1000	Bozic	0	unif	wholeT_0.01	DiP, DiP-A, OT, OT-A

Table 2: *(continued)*

	Conjunction	Drivers	Tree	S.Size	Model	sh	S.Time	S.Type	Best method(s)
438	No	11	11-B	1000	Bozic	0	unif	wholeT_0.5	OT, OT-A
439	No	11	11-B	1000	Bozic	Inf	last	singleC	OT, OT-A
440	No	11	11-B	1000	Bozic	Inf	last	wholeT_0.01	DiP-A
441	No	11	11-B	1000	Bozic	Inf	last	wholeT_0.5	OT, OT-A
442	No	11	11-B	1000	Bozic	Inf	unif	singleC	DiP-A, OT, OT-A
443	No	11	11-B	1000	Bozic	Inf	unif	wholeT_0.01	OT, OT-A
444	No	11	11-B	1000	Bozic	Inf	unif	wholeT_0.5	OT, OT-A
445	No	11	11-B	1000	exp	0	last	singleC	OT, OT-A
446	No	11	11-B	1000	exp	0	last	wholeT_0.01	DiP-A, OT, OT-A
447	No	11	11-B	1000	exp	0	last	wholeT_0.5	OT, OT-A
448	No	11	11-B	1000	exp	0	unif	singleC	OT, OT-A
449	No	11	11-B	1000	exp	0	unif	wholeT_0.01	DiP, DiP-A, OT, OT-A
450	No	11	11-B	1000	exp	0	unif	wholeT_0.5	OT, OT-A
451	No	11	11-B	1000	exp	Inf	last	singleC	OT, OT-A
452	No	11	11-B	1000	exp	Inf	last	wholeT_0.01	OT, OT-A
453	No	11	11-B	1000	exp	Inf	last	wholeT_0.5	OT, OT-A
454	No	11	11-B	1000	exp	Inf	unif	singleC	OT, OT-A
455	No	11	11-B	1000	exp	Inf	unif	wholeT_0.01	OT, OT-A
456	No	11	11-B	1000	exp	Inf	unif	wholeT_0.5	OT, OT-A
457	No	11	11-B	1000	McF_4	0	last	singleC	DiP, DiP-A, OT, OT-A
458	No	11	11-B	1000	McF_4	0	last	wholeT_0.01	DiP, DiP-A, OT, OT-A
459	No	11	11-B	1000	McF_4	0	last	wholeT_0.5	DiP-A, OT, OT-A
460	No	11	11-B	1000	McF_4	0	unif	singleC	DiP, DiP-A
461	No	11	11-B	1000	McF_4	0	unif	wholeT_0.01	DiP-A, OT, OT-A
462	No	11	11-B	1000	McF_4	0	unif	wholeT_0.5	DiP, DiP-A, OT, OT-A
463	No	11	11-B	1000	McF_4	Inf	last	singleC	OT, OT-A
464	No	11	11-B	1000	McF_4	Inf	last	wholeT_0.01	DiP, DiP-A, OT, OT-A
465	No	11	11-B	1000	McF_4	Inf	last	wholeT_0.5	DiP-A, OT, OT-A
466	No	11	11-B	1000	McF_4	Inf	unif	singleC	DiP, DiP-A, OT, OT-A
467	No	11	11-B	1000	McF_4	Inf	unif	wholeT_0.01	OT, OT-A
468	No	11	11-B	1000	McF_4	Inf	unif	wholeT_0.5	DiP, DiP-A, OT, OT-A
469	No	11	11-B	1000	McF_6	0	last	singleC	DiP, OT
470	No	11	11-B	1000	McF_6	0	last	wholeT_0.01	DiP, OT
471	No	11	11-B	1000	McF_6	0	last	wholeT_0.5	DiP, OT
472	No	11	11-B	1000	McF_6	0	unif	singleC	DiP, DiP-A, OT, OT-A
473	No	11	11-B	1000	McF_6	0	unif	wholeT_0.01	DiP, DiP-A, OT, OT-A
474	No	11	11-B	1000	McF_6	0	unif	wholeT_0.5	DiP, DiP-A
475	No	11	11-B	1000	McF_6	Inf	last	singleC	DiP, OT
476	No	11	11-B	1000	McF_6	Inf	last	wholeT_0.01	DiP, OT
477	No	11	11-B	1000	McF_6	Inf	last	wholeT_0.5	DiP, OT

Table 2: *(continued)*

	Conjunction	Drivers	Tree	S.Size	Model	sh	S.Time	S.Type	Best method(s)
478	No	11	11-B	1000	McF_6	Inf	unif	singleC	DiP, DiP-A
479	No	11	11-B	1000	McF_6	Inf	unif	wholeT_0.01	DiP, DiP-A, OT, OT-A
480	No	11	11-B	1000	McF_6	Inf	unif	wholeT_0.5	DiP, DiP-A
481	No	11	11-B	200	Bozic	0	last	singleC	OT, OT-A
482	No	11	11-B	200	Bozic	0	last	wholeT_0.01	OT, OT-A
483	No	11	11-B	200	Bozic	0	last	wholeT_0.5	OT, OT-A
484	No	11	11-B	200	Bozic	0	unif	singleC	OT, OT-A
485	No	11	11-B	200	Bozic	0	unif	wholeT_0.01	DiP, DiP-A, OT, OT-A
486	No	11	11-B	200	Bozic	0	unif	wholeT_0.5	OT, OT-A
487	No	11	11-B	200	Bozic	Inf	last	singleC	OT, OT-A
488	No	11	11-B	200	Bozic	Inf	last	wholeT_0.01	OT, OT-A
489	No	11	11-B	200	Bozic	Inf	last	wholeT_0.5	OT, OT-A
490	No	11	11-B	200	Bozic	Inf	unif	singleC	OT, OT-A
491	No	11	11-B	200	Bozic	Inf	unif	wholeT_0.01	OT, OT-A
492	No	11	11-B	200	Bozic	Inf	unif	wholeT_0.5	OT, OT-A
493	No	11	11-B	200	exp	0	last	singleC	OT, OT-A
494	No	11	11-B	200	exp	0	last	wholeT_0.01	OT, OT-A
495	No	11	11-B	200	exp	0	last	wholeT_0.5	OT, OT-A
496	No	11	11-B	200	exp	0	unif	singleC	CBN, CBN-A
497	No	11	11-B	200	exp	0	unif	wholeT_0.01	OT, OT-A
498	No	11	11-B	200	exp	0	unif	wholeT_0.5	CBN-A
499	No	11	11-B	200	exp	Inf	last	singleC	OT, OT-A
500	No	11	11-B	200	exp	Inf	last	wholeT_0.01	OT, OT-A
501	No	11	11-B	200	exp	Inf	last	wholeT_0.5	OT, OT-A
502	No	11	11-B	200	exp	Inf	unif	singleC	OT, OT-A
503	No	11	11-B	200	exp	Inf	unif	wholeT_0.01	OT, OT-A
504	No	11	11-B	200	exp	Inf	unif	wholeT_0.5	OT, OT-A
505	No	11	11-B	200	McF_4	0	last	singleC	DiP-A, OT, OT-A
506	No	11	11-B	200	McF_4	0	last	wholeT_0.01	DiP, DiP-A, OT, OT-A
507	No	11	11-B	200	McF_4	0	last	wholeT_0.5	DiP-A, OT, OT-A
508	No	11	11-B	200	McF_4	0	unif	singleC	DiP, DiP-A, OT, OT-A
509	No	11	11-B	200	McF_4	0	unif	wholeT_0.01	DiP, DiP-A
510	No	11	11-B	200	McF_4	0	unif	wholeT_0.5	OT, OT-A
511	No	11	11-B	200	McF_4	Inf	last	singleC	OT, OT-A
512	No	11	11-B	200	McF_4	Inf	last	wholeT_0.01	OT, OT-A
513	No	11	11-B	200	McF_4	Inf	last	wholeT_0.5	OT, OT-A
514	No	11	11-B	200	McF_4	Inf	unif	singleC	OT, OT-A
515	No	11	11-B	200	McF_4	Inf	unif	wholeT_0.01	OT, OT-A
516	No	11	11-B	200	McF_4	Inf	unif	wholeT_0.5	OT, OT-A
517	No	11	11-B	200	McF_6	0	last	singleC	DiP, OT

Table 2: *(continued)*

	Conjunction	Drivers	Tree	S.Size	Model	sh	S.Time	S.Type	Best method(s)
518	No	11	11-B	200	McF_6	0	last	wholeT_0.01	DiP, OT
519	No	11	11-B	200	McF_6	0	last	wholeT_0.5	DiP, OT
520	No	11	11-B	200	McF_6	0	unif	singleC	OT, OT-A
521	No	11	11-B	200	McF_6	0	unif	wholeT_0.01	DiP, DiP-A, OT, OT-A
522	No	11	11-B	200	McF_6	0	unif	wholeT_0.5	OT, OT-A
523	No	11	11-B	200	McF_6	Inf	last	singleC	OT
524	No	11	11-B	200	McF_6	Inf	last	wholeT_0.01	DiP, OT
525	No	11	11-B	200	McF_6	Inf	last	wholeT_0.5	OT
526	No	11	11-B	200	McF_6	Inf	unif	singleC	OT, OT-A
527	No	11	11-B	200	McF_6	Inf	unif	wholeT_0.01	OT, OT-A
528	No	11	11-B	200	McF_6	Inf	unif	wholeT_0.5	OT, OT-A
529	No	11	11-B	100	Bozic	0	last	singleC	OT, OT-A
530	No	11	11-B	100	Bozic	0	last	wholeT_0.01	OT, OT-A
531	No	11	11-B	100	Bozic	0	last	wholeT_0.5	OT, OT-A
532	No	11	11-B	100	Bozic	0	unif	singleC	OT, OT-A
533	No	11	11-B	100	Bozic	0	unif	wholeT_0.01	DiP, DiP-A, OT, OT-A
534	No	11	11-B	100	Bozic	0	unif	wholeT_0.5	OT, OT-A
535	No	11	11-B	100	Bozic	Inf	last	singleC	OT, OT-A
536	No	11	11-B	100	Bozic	Inf	last	wholeT_0.01	OT, OT-A
537	No	11	11-B	100	Bozic	Inf	last	wholeT_0.5	OT, OT-A
538	No	11	11-B	100	Bozic	Inf	unif	singleC	OT, OT-A
539	No	11	11-B	100	Bozic	Inf	unif	wholeT_0.01	OT, OT-A
540	No	11	11-B	100	Bozic	Inf	unif	wholeT_0.5	OT, OT-A
541	No	11	11-B	100	exp	0	last	singleC	OT, OT-A
542	No	11	11-B	100	exp	0	last	wholeT_0.01	OT, OT-A
543	No	11	11-B	100	exp	0	last	wholeT_0.5	OT, OT-A
544	No	11	11-B	100	exp	0	unif	singleC	CBN, CBN-A
545	No	11	11-B	100	exp	0	unif	wholeT_0.01	OT, OT-A
546	No	11	11-B	100	exp	0	unif	wholeT_0.5	CBN, CBN-A
547	No	11	11-B	100	exp	Inf	last	singleC	OT, OT-A
548	No	11	11-B	100	exp	Inf	last	wholeT_0.01	OT, OT-A
549	No	11	11-B	100	exp	Inf	last	wholeT_0.5	OT, OT-A
550	No	11	11-B	100	exp	Inf	unif	singleC	OT, OT-A
551	No	11	11-B	100	exp	Inf	unif	wholeT_0.01	OT, OT-A
552	No	11	11-B	100	exp	Inf	unif	wholeT_0.5	OT, OT-A
553	No	11	11-B	100	McF_4	0	last	singleC	OT, OT-A
554	No	11	11-B	100	McF_4	0	last	wholeT_0.01	DiP, DiP-A, OT, OT-A
555	No	11	11-B	100	McF_4	0	last	wholeT_0.5	OT, OT-A
556	No	11	11-B	100	McF_4	0	unif	singleC	OT, OT-A
557	No	11	11-B	100	McF_4	0	unif	wholeT_0.01	DiP, DiP-A

Table 2: *(continued)*

	Conjunction	Drivers	Tree	S.Size	Model	sh	S.Time	S.Type	Best method(s)
558	No	11	11-B	100	McF_4	0	unif	wholeT_0.5	OT, OT-A
559	No	11	11-B	100	McF_4	Inf	last	singleC	OT, OT-A
560	No	11	11-B	100	McF_4	Inf	last	wholeT_0.01	OT, OT-A
561	No	11	11-B	100	McF_4	Inf	last	wholeT_0.5	OT, OT-A
562	No	11	11-B	100	McF_4	Inf	unif	singleC	OT, OT-A
563	No	11	11-B	100	McF_4	Inf	unif	wholeT_0.01	OT, OT-A
564	No	11	11-B	100	McF_4	Inf	unif	wholeT_0.5	OT, OT-A
565	No	11	11-B	100	McF_6	0	last	singleC	OT
566	No	11	11-B	100	McF_6	0	last	wholeT_0.01	DiP, OT
567	No	11	11-B	100	McF_6	0	last	wholeT_0.5	OT
568	No	11	11-B	100	McF_6	0	unif	singleC	OT, OT-A
569	No	11	11-B	100	McF_6	0	unif	wholeT_0.01	DiP, DiP-A, OT, OT-A
570	No	11	11-B	100	McF_6	0	unif	wholeT_0.5	OT, OT-A
571	No	11	11-B	100	McF_6	Inf	last	singleC	OT
572	No	11	11-B	100	McF_6	Inf	last	wholeT_0.01	OT
573	No	11	11-B	100	McF_6	Inf	last	wholeT_0.5	OT
574	No	11	11-B	100	McF_6	Inf	unif	singleC	OT, OT-A
575	No	11	11-B	100	McF_6	Inf	unif	wholeT_0.01	OT, OT-A
576	No	11	11-B	100	McF_6	Inf	unif	wholeT_0.5	OT, OT-A
577	No	9	9-B	1000	Bozic	0	last	singleC	OT, OT-A
578	No	9	9-B	1000	Bozic	0	last	wholeT_0.01	DiP, DiP-A, OT, OT-A
579	No	9	9-B	1000	Bozic	0	last	wholeT_0.5	OT, OT-A
580	No	9	9-B	1000	Bozic	0	unif	singleC	OT, OT-A
581	No	9	9-B	1000	Bozic	0	unif	wholeT_0.01	DiP, DiP-A, OT, OT-A
582	No	9	9-B	1000	Bozic	0	unif	wholeT_0.5	OT, OT-A
583	No	9	9-B	1000	Bozic	Inf	last	singleC	OT, OT-A
584	No	9	9-B	1000	Bozic	Inf	last	wholeT_0.01	DiP, DiP-A
585	No	9	9-B	1000	Bozic	Inf	last	wholeT_0.5	OT, OT-A
586	No	9	9-B	1000	Bozic	Inf	unif	singleC	DiP, DiP-A, OT, OT-A
587	No	9	9-B	1000	Bozic	Inf	unif	wholeT_0.01	OT, OT-A
588	No	9	9-B	1000	Bozic	Inf	unif	wholeT_0.5	OT, OT-A
589	No	9	9-B	1000	exp	0	last	singleC	OT, OT-A
590	No	9	9-B	1000	exp	0	last	wholeT_0.01	DiP-A, OT, OT-A
591	No	9	9-B	1000	exp	0	last	wholeT_0.5	OT, OT-A
592	No	9	9-B	1000	exp	0	unif	singleC	OT, OT-A
593	No	9	9-B	1000	exp	0	unif	wholeT_0.01	DiP, DiP-A, OT, OT-A
594	No	9	9-B	1000	exp	0	unif	wholeT_0.5	OT, OT-A
595	No	9	9-B	1000	exp	Inf	last	singleC	DiP, DiP-A, OT, OT-A
596	No	9	9-B	1000	exp	Inf	last	wholeT_0.01	CBN-A, DiP, DiP-A, OT, OT-A
597	No	9	9-B	1000	exp	Inf	last	wholeT_0.5	DiP, DiP-A, OT, OT-A

Table 2: *(continued)*

	Conjunction	Drivers	Tree	S.Size	Model	sh	S.Time	S.Type	Best method(s)
598	No	9	9-B	1000	exp	Inf	unif	singleC	OT, OT-A
599	No	9	9-B	1000	exp	Inf	unif	wholeT_0.01	OT, OT-A
600	No	9	9-B	1000	exp	Inf	unif	wholeT_0.5	OT, OT-A
601	No	9	9-B	1000	McF_4	0	last	singleC	DiP-A, OT, OT-A
602	No	9	9-B	1000	McF_4	0	last	wholeT_0.01	DiP, DiP-A, OT, OT-A
603	No	9	9-B	1000	McF_4	0	last	wholeT_0.5	OT, OT-A
604	No	9	9-B	1000	McF_4	0	unif	singleC	DiP, DiP-A, OT, OT-A
605	No	9	9-B	1000	McF_4	0	unif	wholeT_0.01	DiP, DiP-A, OT, OT-A
606	No	9	9-B	1000	McF_4	0	unif	wholeT_0.5	DiP, DiP-A, OT, OT-A
607	No	9	9-B	1000	McF_4	Inf	last	singleC	OT, OT-A
608	No	9	9-B	1000	McF_4	Inf	last	wholeT_0.01	DiP, DiP-A, OT, OT-A
609	No	9	9-B	1000	McF_4	Inf	last	wholeT_0.5	DiP, DiP-A, OT, OT-A
610	No	9	9-B	1000	McF_4	Inf	unif	singleC	DiP, DiP-A, OT, OT-A
611	No	9	9-B	1000	McF_4	Inf	unif	wholeT_0.01	DiP-A, OT, OT-A
612	No	9	9-B	1000	McF_4	Inf	unif	wholeT_0.5	DiP, DiP-A, OT, OT-A
613	No	9	9-B	1000	McF_6	0	last	singleC	DiP, OT
614	No	9	9-B	1000	McF_6	0	last	wholeT_0.01	DiP, OT
615	No	9	9-B	1000	McF_6	0	last	wholeT_0.5	DiP, OT
616	No	9	9-B	1000	McF_6	0	unif	singleC	DiP, DiP-A, OT, OT-A
617	No	9	9-B	1000	McF_6	0	unif	wholeT_0.01	DiP, DiP-A, OT, OT-A
618	No	9	9-B	1000	McF_6	0	unif	wholeT_0.5	DiP, DiP-A, OT, OT-A
619	No	9	9-B	1000	McF_6	Inf	last	singleC	DiP, OT
620	No	9	9-B	1000	McF_6	Inf	last	wholeT_0.01	DiP, OT
621	No	9	9-B	1000	McF_6	Inf	last	wholeT_0.5	DiP, OT
622	No	9	9-B	1000	McF_6	Inf	unif	singleC	DiP, DiP-A, OT, OT-A
623	No	9	9-B	1000	McF_6	Inf	unif	wholeT_0.01	DiP, DiP-A, OT, OT-A
624	No	9	9-B	1000	McF_6	Inf	unif	wholeT_0.5	DiP, DiP-A, OT, OT-A
625	No	9	9-B	200	Bozic	0	last	singleC	OT, OT-A
626	No	9	9-B	200	Bozic	0	last	wholeT_0.01	OT, OT-A
627	No	9	9-B	200	Bozic	0	last	wholeT_0.5	OT, OT-A
628	No	9	9-B	200	Bozic	0	unif	singleC	OT, OT-A
629	No	9	9-B	200	Bozic	0	unif	wholeT_0.01	DiP, DiP-A, OT, OT-A
630	No	9	9-B	200	Bozic	0	unif	wholeT_0.5	CBN, CBN-A
631	No	9	9-B	200	Bozic	Inf	last	singleC	OT, OT-A
632	No	9	9-B	200	Bozic	Inf	last	wholeT_0.01	OT, OT-A
633	No	9	9-B	200	Bozic	Inf	last	wholeT_0.5	OT, OT-A
634	No	9	9-B	200	Bozic	Inf	unif	singleC	OT, OT-A
635	No	9	9-B	200	Bozic	Inf	unif	wholeT_0.01	OT, OT-A
636	No	9	9-B	200	Bozic	Inf	unif	wholeT_0.5	OT, OT-A
637	No	9	9-B	200	exp	0	last	singleC	OT, OT-A

Table 2: *(continued)*

	Conjunction	Drivers	Tree	S.Size	Model	sh	S.Time	S.Type	Best method(s)
638	No	9	9-B	200	exp	0	last	wholeT_0.01	OT, OT-A
639	No	9	9-B	200	exp	0	last	wholeT_0.5	OT, OT-A
640	No	9	9-B	200	exp	0	unif	singleC	CBN, CBN-A
641	No	9	9-B	200	exp	0	unif	wholeT_0.01	OT, OT-A
642	No	9	9-B	200	exp	0	unif	wholeT_0.5	CBN, CBN-A
643	No	9	9-B	200	exp	Inf	last	singleC	OT, OT-A
644	No	9	9-B	200	exp	Inf	last	wholeT_0.01	OT, OT-A
645	No	9	9-B	200	exp	Inf	last	wholeT_0.5	OT, OT-A
646	No	9	9-B	200	exp	Inf	unif	singleC	OT, OT-A
647	No	9	9-B	200	exp	Inf	unif	wholeT_0.01	OT, OT-A
648	No	9	9-B	200	exp	Inf	unif	wholeT_0.5	OT, OT-A
649	No	9	9-B	200	McF_4	0	last	singleC	OT, OT-A
650	No	9	9-B	200	McF_4	0	last	wholeT_0.01	DiP, DiP-A, OT, OT-A
651	No	9	9-B	200	McF_4	0	last	wholeT_0.5	DiP-A, OT, OT-A
652	No	9	9-B	200	McF_4	0	unif	singleC	OT, OT-A
653	No	9	9-B	200	McF_4	0	unif	wholeT_0.01	DiP, DiP-A, OT, OT-A
654	No	9	9-B	200	McF_4	0	unif	wholeT_0.5	OT, OT-A
655	No	9	9-B	200	McF_4	Inf	last	singleC	OT, OT-A
656	No	9	9-B	200	McF_4	Inf	last	wholeT_0.01	OT, OT-A
657	No	9	9-B	200	McF_4	Inf	last	wholeT_0.5	DiP-A, OT, OT-A
658	No	9	9-B	200	McF_4	Inf	unif	singleC	OT, OT-A
659	No	9	9-B	200	McF_4	Inf	unif	wholeT_0.01	OT, OT-A
660	No	9	9-B	200	McF_4	Inf	unif	wholeT_0.5	OT, OT-A
661	No	9	9-B	200	McF_6	0	last	singleC	DiP, OT
662	No	9	9-B	200	McF_6	0	last	wholeT_0.01	DiP, OT
663	No	9	9-B	200	McF_6	0	last	wholeT_0.5	DiP, OT
664	No	9	9-B	200	McF_6	0	unif	singleC	DiP, OT, OT-A
665	No	9	9-B	200	McF_6	0	unif	wholeT_0.01	DiP, DiP-A, OT, OT-A
666	No	9	9-B	200	McF_6	0	unif	wholeT_0.5	DiP, OT, OT-A
667	No	9	9-B	200	McF_6	Inf	last	singleC	DiP, OT
668	No	9	9-B	200	McF_6	Inf	last	wholeT_0.01	DiP, OT
669	No	9	9-B	200	McF_6	Inf	last	wholeT_0.5	DiP, OT
670	No	9	9-B	200	McF_6	Inf	unif	singleC	OT, OT-A
671	No	9	9-B	200	McF_6	Inf	unif	wholeT_0.01	OT, OT-A
672	No	9	9-B	200	McF_6	Inf	unif	wholeT_0.5	OT, OT-A
673	No	9	9-B	100	Bozic	0	last	singleC	OT, OT-A
674	No	9	9-B	100	Bozic	0	last	wholeT_0.01	OT, OT-A
675	No	9	9-B	100	Bozic	0	last	wholeT_0.5	OT, OT-A
676	No	9	9-B	100	Bozic	0	unif	singleC	CBN, CBN-A
677	No	9	9-B	100	Bozic	0	unif	wholeT_0.01	OT, OT-A

Table 2: *(continued)*

	Conjunction	Drivers	Tree	S.Size	Model	sh	S.Time	S.Type	Best method(s)
678	No	9	9-B	100	Bozic	0	unif	wholeT_0.5	CBN, CBN-A
679	No	9	9-B	100	Bozic	Inf	last	singleC	OT, OT-A
680	No	9	9-B	100	Bozic	Inf	last	wholeT_0.01	OT, OT-A
681	No	9	9-B	100	Bozic	Inf	last	wholeT_0.5	OT, OT-A
682	No	9	9-B	100	Bozic	Inf	unif	singleC	OT, OT-A
683	No	9	9-B	100	Bozic	Inf	unif	wholeT_0.01	OT, OT-A
684	No	9	9-B	100	Bozic	Inf	unif	wholeT_0.5	OT, OT-A
685	No	9	9-B	100	exp	0	last	singleC	OT, OT-A
686	No	9	9-B	100	exp	0	last	wholeT_0.01	OT, OT-A
687	No	9	9-B	100	exp	0	last	wholeT_0.5	OT, OT-A
688	No	9	9-B	100	exp	0	unif	singleC	CBN, CBN-A
689	No	9	9-B	100	exp	0	unif	wholeT_0.01	OT, OT-A
690	No	9	9-B	100	exp	0	unif	wholeT_0.5	CBN, CBN-A
691	No	9	9-B	100	exp	Inf	last	singleC	OT, OT-A
692	No	9	9-B	100	exp	Inf	last	wholeT_0.01	OT, OT-A
693	No	9	9-B	100	exp	Inf	last	wholeT_0.5	OT, OT-A
694	No	9	9-B	100	exp	Inf	unif	singleC	OT, OT-A
695	No	9	9-B	100	exp	Inf	unif	wholeT_0.01	OT, OT-A
696	No	9	9-B	100	exp	Inf	unif	wholeT_0.5	OT, OT-A
697	No	9	9-B	100	McF_4	0	last	singleC	OT, OT-A
698	No	9	9-B	100	McF_4	0	last	wholeT_0.01	DiP, DiP-A, OT, OT-A
699	No	9	9-B	100	McF_4	0	last	wholeT_0.5	OT, OT-A
700	No	9	9-B	100	McF_4	0	unif	singleC	OT, OT-A
701	No	9	9-B	100	McF_4	0	unif	wholeT_0.01	DiP, DiP-A, OT, OT-A
702	No	9	9-B	100	McF_4	0	unif	wholeT_0.5	OT, OT-A
703	No	9	9-B	100	McF_4	Inf	last	singleC	OT, OT-A
704	No	9	9-B	100	McF_4	Inf	last	wholeT_0.01	OT, OT-A
705	No	9	9-B	100	McF_4	Inf	last	wholeT_0.5	OT, OT-A
706	No	9	9-B	100	McF_4	Inf	unif	singleC	OT, OT-A
707	No	9	9-B	100	McF_4	Inf	unif	wholeT_0.01	OT, OT-A
708	No	9	9-B	100	McF_4	Inf	unif	wholeT_0.5	OT, OT-A
709	No	9	9-B	100	McF_6	0	last	singleC	OT
710	No	9	9-B	100	McF_6	0	last	wholeT_0.01	OT
711	No	9	9-B	100	McF_6	0	last	wholeT_0.5	OT
712	No	9	9-B	100	McF_6	0	unif	singleC	OT, OT-A
713	No	9	9-B	100	McF_6	0	unif	wholeT_0.01	DiP, DiP-A, OT, OT-A
714	No	9	9-B	100	McF_6	0	unif	wholeT_0.5	OT, OT-A
715	No	9	9-B	100	McF_6	Inf	last	singleC	OT
716	No	9	9-B	100	McF_6	Inf	last	wholeT_0.01	OT
717	No	9	9-B	100	McF_6	Inf	last	wholeT_0.5	OT

Table 2: *(continued)*

	Conjunction	Drivers	Tree	S.Size	Model	sh	S.Time	S.Type	Best method(s)
718	No	9	9-B	100	McF_6	Inf	unif	singleC	OT, OT-A
719	No	9	9-B	100	McF_6	Inf	unif	wholeT_0.01	OT, OT-A
720	No	9	9-B	100	McF_6	Inf	unif	wholeT_0.5	OT, OT-A
721	No	7	7-B	1000	Bozic	0	last	singleC	DiP-A, OT, OT-A
722	No	7	7-B	1000	Bozic	0	last	wholeT_0.01	DiP-A, OT, OT-A
723	No	7	7-B	1000	Bozic	0	last	wholeT_0.5	OT, OT-A
724	No	7	7-B	1000	Bozic	0	unif	singleC	DiP, DiP-A, OT, OT-A
725	No	7	7-B	1000	Bozic	0	unif	wholeT_0.01	CBN-A, DiP, DiP-A, OT, OT-A
726	No	7	7-B	1000	Bozic	0	unif	wholeT_0.5	OT, OT-A
727	No	7	7-B	1000	Bozic	Inf	last	singleC	DiP, DiP-A, OT, OT-A
728	No	7	7-B	1000	Bozic	Inf	last	wholeT_0.01	CBN, CBN-A, DiP, DiP-A, OT, OT-A
729	No	7	7-B	1000	Bozic	Inf	last	wholeT_0.5	DiP-A, OT, OT-A
730	No	7	7-B	1000	Bozic	Inf	unif	singleC	CBN, CBN-A
731	No	7	7-B	1000	Bozic	Inf	unif	wholeT_0.01	CBN, CBN-A, OT, OT-A
732	No	7	7-B	1000	Bozic	Inf	unif	wholeT_0.5	CBN, CBN-A
733	No	7	7-B	1000	exp	0	last	singleC	OT-A
734	No	7	7-B	1000	exp	0	last	wholeT_0.01	OT-A
735	No	7	7-B	1000	exp	0	last	wholeT_0.5	OT-A
736	No	7	7-B	1000	exp	0	unif	singleC	OT, OT-A
737	No	7	7-B	1000	exp	0	unif	wholeT_0.01	CBN, CBN-A, OT, OT-A
738	No	7	7-B	1000	exp	0	unif	wholeT_0.5	OT, OT-A
739	No	7	7-B	1000	exp	Inf	last	singleC	OT, OT-A
740	No	7	7-B	1000	exp	Inf	last	wholeT_0.01	CBN, CBN-A, OT, OT-A
741	No	7	7-B	1000	exp	Inf	last	wholeT_0.5	OT, OT-A
742	No	7	7-B	1000	exp	Inf	unif	singleC	OT, OT-A
743	No	7	7-B	1000	exp	Inf	unif	wholeT_0.01	CBN-A, OT, OT-A
744	No	7	7-B	1000	exp	Inf	unif	wholeT_0.5	OT, OT-A
745	No	7	7-B	1000	McF_4	0	last	singleC	DiP, DiP-A, OT, OT-A
746	No	7	7-B	1000	McF_4	0	last	wholeT_0.01	DiP, DiP-A, OT, OT-A
747	No	7	7-B	1000	McF_4	0	last	wholeT_0.5	DiP, DiP-A, OT, OT-A
748	No	7	7-B	1000	McF_4	0	unif	singleC	DiP, DiP-A, OT, OT-A
749	No	7	7-B	1000	McF_4	0	unif	wholeT_0.01	DiP, DiP-A, OT, OT-A
750	No	7	7-B	1000	McF_4	0	unif	wholeT_0.5	DiP, DiP-A, OT, OT-A
751	No	7	7-B	1000	McF_4	Inf	last	singleC	DiP, DiP-A, OT, OT-A
752	No	7	7-B	1000	McF_4	Inf	last	wholeT_0.01	DiP, DiP-A, OT, OT-A
753	No	7	7-B	1000	McF_4	Inf	last	wholeT_0.5	DiP, DiP-A, OT, OT-A
754	No	7	7-B	1000	McF_4	Inf	unif	singleC	DiP, DiP-A, OT, OT-A
755	No	7	7-B	1000	McF_4	Inf	unif	wholeT_0.01	DiP, DiP-A, OT, OT-A
756	No	7	7-B	1000	McF_4	Inf	unif	wholeT_0.5	DiP, DiP-A, OT, OT-A
757	No	7	7-B	1000	McF_6	0	last	singleC	DiP, DiP-A, OT, OT-A

Table 2: *(continued)*

	Conjunction	Drivers	Tree	S.Size	Model	sh	S.Time	S.Type	Best method(s)
758	No	7	7-B	1000	McF_6	0	last	wholeT_0.01	DiP, DiP-A, OT, OT-A
759	No	7	7-B	1000	McF_6	0	last	wholeT_0.5	DiP, DiP-A, OT, OT-A
760	No	7	7-B	1000	McF_6	0	unif	singleC	CBN, CBN-A, DiP, DiP-A, OT, OT-A
761	No	7	7-B	1000	McF_6	0	unif	wholeT_0.01	CBN, CBN-A, DiP, DiP-A, OT, OT-A
762	No	7	7-B	1000	McF_6	0	unif	wholeT_0.5	CBN, CBN-A, DiP, DiP-A, OT, OT-A
763	No	7	7-B	1000	McF_6	Inf	last	singleC	DiP, DiP-A, OT, OT-A
764	No	7	7-B	1000	McF_6	Inf	last	wholeT_0.01	DiP-A, OT, OT-A
765	No	7	7-B	1000	McF_6	Inf	last	wholeT_0.5	DiP, DiP-A, OT, OT-A
766	No	7	7-B	1000	McF_6	Inf	unif	singleC	CBN, CBN-A, DiP, DiP-A, OT, OT-A
767	No	7	7-B	1000	McF_6	Inf	unif	wholeT_0.01	CBN, CBN-A, DiP, DiP-A, OT, OT-A
768	No	7	7-B	1000	McF_6	Inf	unif	wholeT_0.5	CBN, CBN-A, DiP, DiP-A, OT, OT-A
769	No	7	7-B	200	Bozic	0	last	singleC	OT-A
770	No	7	7-B	200	Bozic	0	last	wholeT_0.01	OT-A
771	No	7	7-B	200	Bozic	0	last	wholeT_0.5	OT-A
772	No	7	7-B	200	Bozic	0	unif	singleC	OT, OT-A
773	No	7	7-B	200	Bozic	0	unif	wholeT_0.01	CBN, CBN-A, OT, OT-A
774	No	7	7-B	200	Bozic	0	unif	wholeT_0.5	OT, OT-A
775	No	7	7-B	200	Bozic	Inf	last	singleC	OT, OT-A
776	No	7	7-B	200	Bozic	Inf	last	wholeT_0.01	CBN, CBN-A, OT, OT-A
777	No	7	7-B	200	Bozic	Inf	last	wholeT_0.5	OT, OT-A
778	No	7	7-B	200	Bozic	Inf	unif	singleC	CBN, CBN-A, OT, OT-A
779	No	7	7-B	200	Bozic	Inf	unif	wholeT_0.01	CBN, CBN-A, OT, OT-A
780	No	7	7-B	200	Bozic	Inf	unif	wholeT_0.5	CBN, CBN-A, OT, OT-A
781	No	7	7-B	200	exp	0	last	singleC	OT-A
782	No	7	7-B	200	exp	0	last	wholeT_0.01	OT-A
783	No	7	7-B	200	exp	0	last	wholeT_0.5	OT-A
784	No	7	7-B	200	exp	0	unif	singleC	OT, OT-A
785	No	7	7-B	200	exp	0	unif	wholeT_0.01	OT, OT-A
786	No	7	7-B	200	exp	0	unif	wholeT_0.5	OT, OT-A
787	No	7	7-B	200	exp	Inf	last	singleC	OT, OT-A
788	No	7	7-B	200	exp	Inf	last	wholeT_0.01	CBN-A, OT-A
789	No	7	7-B	200	exp	Inf	last	wholeT_0.5	OT, OT-A
790	No	7	7-B	200	exp	Inf	unif	singleC	OT, OT-A
791	No	7	7-B	200	exp	Inf	unif	wholeT_0.01	CBN, CBN-A, OT, OT-A
792	No	7	7-B	200	exp	Inf	unif	wholeT_0.5	OT, OT-A
793	No	7	7-B	200	McF_4	0	last	singleC	OT, OT-A
794	No	7	7-B	200	McF_4	0	last	wholeT_0.01	DiP, DiP-A, OT, OT-A
795	No	7	7-B	200	McF_4	0	last	wholeT_0.5	OT, OT-A
796	No	7	7-B	200	McF_4	0	unif	singleC	DiP-A, OT, OT-A
797	No	7	7-B	200	McF_4	0	unif	wholeT_0.01	DiP, DiP-A, OT, OT-A

Table 2: *(continued)*

	Conjunction	Drivers	Tree	S.Size	Model	sh	S.Time	S.Type	Best method(s)
798	No	7	7-B	200	McF_4	0	unif	wholeT_0.5	OT, OT-A
799	No	7	7-B	200	McF_4	Inf	last	singleC	OT, OT-A
800	No	7	7-B	200	McF_4	Inf	last	wholeT_0.01	OT, OT-A
801	No	7	7-B	200	McF_4	Inf	last	wholeT_0.5	OT, OT-A
802	No	7	7-B	200	McF_4	Inf	unif	singleC	OT, OT-A
803	No	7	7-B	200	McF_4	Inf	unif	wholeT_0.01	OT, OT-A
804	No	7	7-B	200	McF_4	Inf	unif	wholeT_0.5	OT, OT-A
805	No	7	7-B	200	McF_6	0	last	singleC	DiP-A, OT, OT-A
806	No	7	7-B	200	McF_6	0	last	wholeT_0.01	DiP, DiP-A, OT, OT-A
807	No	7	7-B	200	McF_6	0	last	wholeT_0.5	DiP-A, OT, OT-A
808	No	7	7-B	200	McF_6	0	unif	singleC	CBN, CBN-A, DiP, DiP-A, OT, OT-A
809	No	7	7-B	200	McF_6	0	unif	wholeT_0.01	CBN, CBN-A, DiP, DiP-A, OT, OT-A
810	No	7	7-B	200	McF_6	0	unif	wholeT_0.5	CBN, CBN-A, DiP, OT, OT-A
811	No	7	7-B	200	McF_6	Inf	last	singleC	DiP-A, OT, OT-A
812	No	7	7-B	200	McF_6	Inf	last	wholeT_0.01	OT, OT-A
813	No	7	7-B	200	McF_6	Inf	last	wholeT_0.5	DiP-A, OT, OT-A
814	No	7	7-B	200	McF_6	Inf	unif	singleC	CBN, CBN-A
815	No	7	7-B	200	McF_6	Inf	unif	wholeT_0.01	CBN, CBN-A, DiP-A
816	No	7	7-B	200	McF_6	Inf	unif	wholeT_0.5	CBN, CBN-A, DiP
817	No	7	7-B	100	Bozic	0	last	singleC	OT-A
818	No	7	7-B	100	Bozic	0	last	wholeT_0.01	OT-A
819	No	7	7-B	100	Bozic	0	last	wholeT_0.5	OT-A
820	No	7	7-B	100	Bozic	0	unif	singleC	OT, OT-A
821	No	7	7-B	100	Bozic	0	unif	wholeT_0.01	CBN-A, OT, OT-A
822	No	7	7-B	100	Bozic	0	unif	wholeT_0.5	OT, OT-A
823	No	7	7-B	100	Bozic	Inf	last	singleC	OT, OT-A
824	No	7	7-B	100	Bozic	Inf	last	wholeT_0.01	CBN-A, OT-A
825	No	7	7-B	100	Bozic	Inf	last	wholeT_0.5	OT, OT-A
826	No	7	7-B	100	Bozic	Inf	unif	singleC	OT, OT-A
827	No	7	7-B	100	Bozic	Inf	unif	wholeT_0.01	CBN-A, OT, OT-A
828	No	7	7-B	100	Bozic	Inf	unif	wholeT_0.5	CBN-A, OT, OT-A
829	No	7	7-B	100	exp	0	last	singleC	OT-A
830	No	7	7-B	100	exp	0	last	wholeT_0.01	OT-A
831	No	7	7-B	100	exp	0	last	wholeT_0.5	OT-A
832	No	7	7-B	100	exp	0	unif	singleC	OT, OT-A
833	No	7	7-B	100	exp	0	unif	wholeT_0.01	OT, OT-A
834	No	7	7-B	100	exp	0	unif	wholeT_0.5	CBN, CBN-A
835	No	7	7-B	100	exp	Inf	last	singleC	OT, OT-A
836	No	7	7-B	100	exp	Inf	last	wholeT_0.01	OT-A
837	No	7	7-B	100	exp	Inf	last	wholeT_0.5	OT-A

Table 2: *(continued)*

	Conjunction	Drivers	Tree	S.Size	Model	sh	S.Time	S.Type	Best method(s)
838	No	7	7-B	100	exp	Inf	unif	singleC	OT, OT-A
839	No	7	7-B	100	exp	Inf	unif	wholeT_0.01	CBN, CBN-A, OT, OT-A
840	No	7	7-B	100	exp	Inf	unif	wholeT_0.5	OT, OT-A
841	No	7	7-B	100	McF_4	0	last	singleC	OT, OT-A
842	No	7	7-B	100	McF_4	0	last	wholeT_0.01	DiP-A, OT, OT-A
843	No	7	7-B	100	McF_4	0	last	wholeT_0.5	OT, OT-A
844	No	7	7-B	100	McF_4	0	unif	singleC	OT, OT-A
845	No	7	7-B	100	McF_4	0	unif	wholeT_0.01	OT, OT-A
846	No	7	7-B	100	McF_4	0	unif	wholeT_0.5	OT, OT-A
847	No	7	7-B	100	McF_4	Inf	last	singleC	OT, OT-A
848	No	7	7-B	100	McF_4	Inf	last	wholeT_0.01	OT, OT-A
849	No	7	7-B	100	McF_4	Inf	last	wholeT_0.5	OT, OT-A
850	No	7	7-B	100	McF_4	Inf	unif	singleC	OT, OT-A
851	No	7	7-B	100	McF_4	Inf	unif	wholeT_0.01	OT, OT-A
852	No	7	7-B	100	McF_4	Inf	unif	wholeT_0.5	OT, OT-A
853	No	7	7-B	100	McF_6	0	last	singleC	OT, OT-A
854	No	7	7-B	100	McF_6	0	last	wholeT_0.01	OT, OT-A
855	No	7	7-B	100	McF_6	0	last	wholeT_0.5	OT, OT-A
856	No	7	7-B	100	McF_6	0	unif	singleC	CBN, CBN-A, OT, OT-A
857	No	7	7-B	100	McF_6	0	unif	wholeT_0.01	CBN, CBN-A, DiP, DiP-A
858	No	7	7-B	100	McF_6	0	unif	wholeT_0.5	CBN, CBN-A
859	No	7	7-B	100	McF_6	Inf	last	singleC	OT, OT-A
860	No	7	7-B	100	McF_6	Inf	last	wholeT_0.01	OT, OT-A
861	No	7	7-B	100	McF_6	Inf	last	wholeT_0.5	OT, OT-A
862	No	7	7-B	100	McF_6	Inf	unif	singleC	CBN, CBN-A
863	No	7	7-B	100	McF_6	Inf	unif	wholeT_0.01	CBN, CBN-A
864	No	7	7-B	100	McF_6	Inf	unif	wholeT_0.5	CBN, CBN-A

2.3 Confidence sets (MCB-2), PND, Drivers Known

Table 3: Confidence sets (method MCB-2) when Drivers are Known for measure PND.

	Conjunction	Drivers	Tree	S.Size	Model	sh	S.Time	S.Type	Best method(s)
1	Yes	11	11-A	1000	Bozic	0	last	singleC	CBN, CBN-A
2	Yes	11	11-A	1000	Bozic	0	last	wholeT_0.01	CBN, CBN-A, OT, OT-A
3	Yes	11	11-A	1000	Bozic	0	last	wholeT_0.5	CBN, CBN-A, OT, OT-A
4	Yes	11	11-A	1000	Bozic	0	unif	singleC	CBN, CBN-A
5	Yes	11	11-A	1000	Bozic	0	unif	wholeT_0.01	CBN, CBN-A
6	Yes	11	11-A	1000	Bozic	0	unif	wholeT_0.5	CBN, CBN-A
7	Yes	11	11-A	1000	Bozic	Inf	last	singleC	CBN, CBN-A
8	Yes	11	11-A	1000	Bozic	Inf	last	wholeT_0.01	CBN, CBN-A
9	Yes	11	11-A	1000	Bozic	Inf	last	wholeT_0.5	CBN, CBN-A
10	Yes	11	11-A	1000	Bozic	Inf	unif	singleC	CBN, CBN-A
11	Yes	11	11-A	1000	Bozic	Inf	unif	wholeT_0.01	CBN, CBN-A
12	Yes	11	11-A	1000	Bozic	Inf	unif	wholeT_0.5	CBN, CBN-A
13	Yes	11	11-A	1000	exp	0	last	singleC	CBN, CBN-A
14	Yes	11	11-A	1000	exp	0	last	wholeT_0.01	CBN, CBN-A
15	Yes	11	11-A	1000	exp	0	last	wholeT_0.5	CBN, CBN-A
16	Yes	11	11-A	1000	exp	0	unif	singleC	CBN, CBN-A
17	Yes	11	11-A	1000	exp	0	unif	wholeT_0.01	CBN, CBN-A
18	Yes	11	11-A	1000	exp	0	unif	wholeT_0.5	CBN, CBN-A
19	Yes	11	11-A	1000	exp	Inf	last	singleC	CBN, CBN-A
20	Yes	11	11-A	1000	exp	Inf	last	wholeT_0.01	CBN, CBN-A
21	Yes	11	11-A	1000	exp	Inf	last	wholeT_0.5	CBN, CBN-A
22	Yes	11	11-A	1000	exp	Inf	unif	singleC	CBN, CBN-A
23	Yes	11	11-A	1000	exp	Inf	unif	wholeT_0.01	CBN
24	Yes	11	11-A	1000	exp	Inf	unif	wholeT_0.5	CBN, CBN-A
25	Yes	11	11-A	1000	McF_4	0	last	singleC	CBN-A, OT, OT-A
26	Yes	11	11-A	1000	McF_4	0	last	wholeT_0.01	OT, OT-A
27	Yes	11	11-A	1000	McF_4	0	last	wholeT_0.5	CBN, CBN-A, OT, OT-A
28	Yes	11	11-A	1000	McF_4	0	unif	singleC	CBN, CBN-A
29	Yes	11	11-A	1000	McF_4	0	unif	wholeT_0.01	CBN, CBN-A
30	Yes	11	11-A	1000	McF_4	0	unif	wholeT_0.5	CBN, CBN-A
31	Yes	11	11-A	1000	McF_4	Inf	last	singleC	CBN, CBN-A, OT, OT-A
32	Yes	11	11-A	1000	McF_4	Inf	last	wholeT_0.01	CBN, CBN-A, OT, OT-A
33	Yes	11	11-A	1000	McF_4	Inf	last	wholeT_0.5	CBN, CBN-A
34	Yes	11	11-A	1000	McF_4	Inf	unif	singleC	CBN, CBN-A
35	Yes	11	11-A	1000	McF_4	Inf	unif	wholeT_0.01	CBN, CBN-A
36	Yes	11	11-A	1000	McF_4	Inf	unif	wholeT_0.5	CBN, CBN-A
37	Yes	11	11-A	1000	McF_6	0	last	singleC	DiP, DiP-A

Table 3: *(continued)*

	Conjunction	Drivers	Tree	S.Size	Model	sh	S.Time	S.Type	Best method(s)
38	Yes	11	11-A	1000	McF_6	0	last	wholeT_0.01	DiP, DiP-A
39	Yes	11	11-A	1000	McF_6	0	last	wholeT_0.5	DiP, DiP-A
40	Yes	11	11-A	1000	McF_6	0	unif	singleC	CBN, CBN-A, DiP, DiP-A, OT, OT-A
41	Yes	11	11-A	1000	McF_6	0	unif	wholeT_0.01	CBN, DiP, DiP-A
42	Yes	11	11-A	1000	McF_6	0	unif	wholeT_0.5	CBN, CBN-A, DiP, DiP-A, OT, OT-A
43	Yes	11	11-A	1000	McF_6	Inf	last	singleC	DiP-A, OT, OT-A
44	Yes	11	11-A	1000	McF_6	Inf	last	wholeT_0.01	DiP-A
45	Yes	11	11-A	1000	McF_6	Inf	last	wholeT_0.5	DiP-A, OT, OT-A
46	Yes	11	11-A	1000	McF_6	Inf	unif	singleC	CBN, OT, OT-A
47	Yes	11	11-A	1000	McF_6	Inf	unif	wholeT_0.01	CBN, CBN-A, OT, OT-A
48	Yes	11	11-A	1000	McF_6	Inf	unif	wholeT_0.5	CBN, OT, OT-A
49	Yes	11	11-A	200	Bozic	0	last	singleC	CBN, CBN-A
50	Yes	11	11-A	200	Bozic	0	last	wholeT_0.01	CBN, CBN-A
51	Yes	11	11-A	200	Bozic	0	last	wholeT_0.5	CBN-A
52	Yes	11	11-A	200	Bozic	0	unif	singleC	CBN, CBN-A
53	Yes	11	11-A	200	Bozic	0	unif	wholeT_0.01	CBN, CBN-A
54	Yes	11	11-A	200	Bozic	0	unif	wholeT_0.5	CBN, CBN-A
55	Yes	11	11-A	200	Bozic	Inf	last	singleC	CBN, CBN-A
56	Yes	11	11-A	200	Bozic	Inf	last	wholeT_0.01	CBN, CBN-A
57	Yes	11	11-A	200	Bozic	Inf	last	wholeT_0.5	CBN, CBN-A
58	Yes	11	11-A	200	Bozic	Inf	unif	singleC	CBN, CBN-A
59	Yes	11	11-A	200	Bozic	Inf	unif	wholeT_0.01	CBN, CBN-A
60	Yes	11	11-A	200	Bozic	Inf	unif	wholeT_0.5	CBN, CBN-A
61	Yes	11	11-A	200	exp	0	last	singleC	CBN, CBN-A
62	Yes	11	11-A	200	exp	0	last	wholeT_0.01	CBN, CBN-A
63	Yes	11	11-A	200	exp	0	last	wholeT_0.5	CBN, CBN-A
64	Yes	11	11-A	200	exp	0	unif	singleC	CBN, CBN-A
65	Yes	11	11-A	200	exp	0	unif	wholeT_0.01	CBN, CBN-A
66	Yes	11	11-A	200	exp	0	unif	wholeT_0.5	CBN, CBN-A
67	Yes	11	11-A	200	exp	Inf	last	singleC	CBN, CBN-A
68	Yes	11	11-A	200	exp	Inf	last	wholeT_0.01	CBN-A
69	Yes	11	11-A	200	exp	Inf	last	wholeT_0.5	CBN, CBN-A
70	Yes	11	11-A	200	exp	Inf	unif	singleC	CBN, CBN-A
71	Yes	11	11-A	200	exp	Inf	unif	wholeT_0.01	CBN, CBN-A
72	Yes	11	11-A	200	exp	Inf	unif	wholeT_0.5	CBN, CBN-A
73	Yes	11	11-A	200	McF_4	0	last	singleC	CBN, CBN-A, OT, OT-A
74	Yes	11	11-A	200	McF_4	0	last	wholeT_0.01	CBN-A, OT, OT-A
75	Yes	11	11-A	200	McF_4	0	last	wholeT_0.5	CBN, CBN-A
76	Yes	11	11-A	200	McF_4	0	unif	singleC	CBN, CBN-A
77	Yes	11	11-A	200	McF_4	0	unif	wholeT_0.01	CBN, CBN-A

Table 3: *(continued)*

	Conjunction	Drivers	Tree	S.Size	Model	sh	S.Time	S.Type	Best method(s)
78	Yes	11	11-A	200	McF_4	0	unif	wholeT_0.5	CBN, CBN-A
79	Yes	11	11-A	200	McF_4	Inf	last	singleC	CBN, CBN-A
80	Yes	11	11-A	200	McF_4	Inf	last	wholeT_0.01	CBN, CBN-A, OT, OT-A
81	Yes	11	11-A	200	McF_4	Inf	last	wholeT_0.5	CBN, CBN-A, OT, OT-A
82	Yes	11	11-A	200	McF_4	Inf	unif	singleC	CBN, CBN-A
83	Yes	11	11-A	200	McF_4	Inf	unif	wholeT_0.01	CBN, CBN-A
84	Yes	11	11-A	200	McF_4	Inf	unif	wholeT_0.5	CBN, CBN-A
85	Yes	11	11-A	200	McF_6	0	last	singleC	OT, OT-A
86	Yes	11	11-A	200	McF_6	0	last	wholeT_0.01	OT, OT-A
87	Yes	11	11-A	200	McF_6	0	last	wholeT_0.5	OT, OT-A
88	Yes	11	11-A	200	McF_6	0	unif	singleC	CBN-A, OT, OT-A
89	Yes	11	11-A	200	McF_6	0	unif	wholeT_0.01	CBN, CBN-A, OT, OT-A
90	Yes	11	11-A	200	McF_6	0	unif	wholeT_0.5	CBN, CBN-A, OT, OT-A
91	Yes	11	11-A	200	McF_6	Inf	last	singleC	OT, OT-A
92	Yes	11	11-A	200	McF_6	Inf	last	wholeT_0.01	OT, OT-A
93	Yes	11	11-A	200	McF_6	Inf	last	wholeT_0.5	OT, OT-A
94	Yes	11	11-A	200	McF_6	Inf	unif	singleC	CBN, CBN-A, OT, OT-A
95	Yes	11	11-A	200	McF_6	Inf	unif	wholeT_0.01	CBN, CBN-A, OT, OT-A
96	Yes	11	11-A	200	McF_6	Inf	unif	wholeT_0.5	CBN, OT, OT-A
97	Yes	11	11-A	100	Bozic	0	last	singleC	CBN, CBN-A
98	Yes	11	11-A	100	Bozic	0	last	wholeT_0.01	CBN, CBN-A
99	Yes	11	11-A	100	Bozic	0	last	wholeT_0.5	CBN, CBN-A
100	Yes	11	11-A	100	Bozic	0	unif	singleC	CBN, CBN-A
101	Yes	11	11-A	100	Bozic	0	unif	wholeT_0.01	CBN, CBN-A
102	Yes	11	11-A	100	Bozic	0	unif	wholeT_0.5	CBN, CBN-A
103	Yes	11	11-A	100	Bozic	Inf	last	singleC	CBN, CBN-A
104	Yes	11	11-A	100	Bozic	Inf	last	wholeT_0.01	CBN, CBN-A
105	Yes	11	11-A	100	Bozic	Inf	last	wholeT_0.5	CBN, CBN-A
106	Yes	11	11-A	100	Bozic	Inf	unif	singleC	CBN, CBN-A
107	Yes	11	11-A	100	Bozic	Inf	unif	wholeT_0.01	CBN, CBN-A
108	Yes	11	11-A	100	Bozic	Inf	unif	wholeT_0.5	CBN, CBN-A
109	Yes	11	11-A	100	exp	0	last	singleC	CBN, CBN-A
110	Yes	11	11-A	100	exp	0	last	wholeT_0.01	CBN, CBN-A
111	Yes	11	11-A	100	exp	0	last	wholeT_0.5	CBN, CBN-A
112	Yes	11	11-A	100	exp	0	unif	singleC	CBN, CBN-A
113	Yes	11	11-A	100	exp	0	unif	wholeT_0.01	CBN, CBN-A
114	Yes	11	11-A	100	exp	0	unif	wholeT_0.5	CBN, CBN-A
115	Yes	11	11-A	100	exp	Inf	last	singleC	CBN, CBN-A
116	Yes	11	11-A	100	exp	Inf	last	wholeT_0.01	CBN, CBN-A
117	Yes	11	11-A	100	exp	Inf	last	wholeT_0.5	CBN, CBN-A

Table 3: *(continued)*

	Conjunction	Drivers	Tree	S.Size	Model	sh	S.Time	S.Type	Best method(s)
118	Yes	11	11-A	100	exp	Inf	unif	singleC	CBN, CBN-A
119	Yes	11	11-A	100	exp	Inf	unif	wholeT_0.01	CBN, CBN-A
120	Yes	11	11-A	100	exp	Inf	unif	wholeT_0.5	CBN, CBN-A
121	Yes	11	11-A	100	McF_4	0	last	singleC	CBN, CBN-A
122	Yes	11	11-A	100	McF_4	0	last	wholeT_0.01	OT, OT-A
123	Yes	11	11-A	100	McF_4	0	last	wholeT_0.5	CBN, CBN-A, OT, OT-A
124	Yes	11	11-A	100	McF_4	0	unif	singleC	CBN, CBN-A
125	Yes	11	11-A	100	McF_4	0	unif	wholeT_0.01	CBN
126	Yes	11	11-A	100	McF_4	0	unif	wholeT_0.5	CBN, CBN-A
127	Yes	11	11-A	100	McF_4	Inf	last	singleC	CBN, CBN-A, OT, OT-A
128	Yes	11	11-A	100	McF_4	Inf	last	wholeT_0.01	CBN, CBN-A, OT, OT-A
129	Yes	11	11-A	100	McF_4	Inf	last	wholeT_0.5	CBN, CBN-A
130	Yes	11	11-A	100	McF_4	Inf	unif	singleC	CBN, CBN-A
131	Yes	11	11-A	100	McF_4	Inf	unif	wholeT_0.01	CBN, CBN-A
132	Yes	11	11-A	100	McF_4	Inf	unif	wholeT_0.5	CBN, CBN-A
133	Yes	11	11-A	100	McF_6	0	last	singleC	OT, OT-A
134	Yes	11	11-A	100	McF_6	0	last	wholeT_0.01	OT, OT-A
135	Yes	11	11-A	100	McF_6	0	last	wholeT_0.5	OT, OT-A
136	Yes	11	11-A	100	McF_6	0	unif	singleC	CBN, CBN-A, OT, OT-A
137	Yes	11	11-A	100	McF_6	0	unif	wholeT_0.01	CBN, CBN-A, OT, OT-A
138	Yes	11	11-A	100	McF_6	0	unif	wholeT_0.5	CBN, CBN-A, OT, OT-A
139	Yes	11	11-A	100	McF_6	Inf	last	singleC	OT, OT-A
140	Yes	11	11-A	100	McF_6	Inf	last	wholeT_0.01	OT-A
141	Yes	11	11-A	100	McF_6	Inf	last	wholeT_0.5	OT, OT-A
142	Yes	11	11-A	100	McF_6	Inf	unif	singleC	CBN, CBN-A, OT, OT-A
143	Yes	11	11-A	100	McF_6	Inf	unif	wholeT_0.01	CBN, CBN-A, OT, OT-A
144	Yes	11	11-A	100	McF_6	Inf	unif	wholeT_0.5	CBN-A, OT, OT-A
145	Yes	9	9-A	1000	Bozic	0	last	singleC	CBN, CBN-A
146	Yes	9	9-A	1000	Bozic	0	last	wholeT_0.01	CBN, CBN-A
147	Yes	9	9-A	1000	Bozic	0	last	wholeT_0.5	CBN, CBN-A
148	Yes	9	9-A	1000	Bozic	0	unif	singleC	CBN, CBN-A
149	Yes	9	9-A	1000	Bozic	0	unif	wholeT_0.01	CBN, CBN-A
150	Yes	9	9-A	1000	Bozic	0	unif	wholeT_0.5	CBN, CBN-A
151	Yes	9	9-A	1000	Bozic	Inf	last	singleC	CBN, CBN-A
152	Yes	9	9-A	1000	Bozic	Inf	last	wholeT_0.01	CBN, CBN-A
153	Yes	9	9-A	1000	Bozic	Inf	last	wholeT_0.5	CBN, CBN-A
154	Yes	9	9-A	1000	Bozic	Inf	unif	singleC	CBN, CBN-A
155	Yes	9	9-A	1000	Bozic	Inf	unif	wholeT_0.01	CBN, CBN-A
156	Yes	9	9-A	1000	Bozic	Inf	unif	wholeT_0.5	CBN
157	Yes	9	9-A	1000	exp	0	last	singleC	CBN, CBN-A

Table 3: *(continued)*

	Conjunction	Drivers	Tree	S.Size	Model	sh	S.Time	S.Type	Best method(s)
158	Yes	9	9-A	1000	exp	0	last	wholeT_0.01	CBN, CBN-A
159	Yes	9	9-A	1000	exp	0	last	wholeT_0.5	CBN, CBN-A
160	Yes	9	9-A	1000	exp	0	unif	singleC	CBN, CBN-A
161	Yes	9	9-A	1000	exp	0	unif	wholeT_0.01	CBN, CBN-A
162	Yes	9	9-A	1000	exp	0	unif	wholeT_0.5	CBN, CBN-A
163	Yes	9	9-A	1000	exp	Inf	last	singleC	CBN, CBN-A
164	Yes	9	9-A	1000	exp	Inf	last	wholeT_0.01	CBN, CBN-A
165	Yes	9	9-A	1000	exp	Inf	last	wholeT_0.5	CBN, CBN-A
166	Yes	9	9-A	1000	exp	Inf	unif	singleC	CBN, CBN-A
167	Yes	9	9-A	1000	exp	Inf	unif	wholeT_0.01	CBN, CBN-A
168	Yes	9	9-A	1000	exp	Inf	unif	wholeT_0.5	CBN, CBN-A
169	Yes	9	9-A	1000	McF_4	0	last	singleC	CBN, CBN-A, OT, OT-A
170	Yes	9	9-A	1000	McF_4	0	last	wholeT_0.01	CBN
171	Yes	9	9-A	1000	McF_4	0	last	wholeT_0.5	CBN, CBN-A, OT, OT-A
172	Yes	9	9-A	1000	McF_4	0	unif	singleC	CBN, CBN-A
173	Yes	9	9-A	1000	McF_4	0	unif	wholeT_0.01	CBN, CBN-A
174	Yes	9	9-A	1000	McF_4	0	unif	wholeT_0.5	CBN, CBN-A
175	Yes	9	9-A	1000	McF_4	Inf	last	singleC	CBN, CBN-A
176	Yes	9	9-A	1000	McF_4	Inf	last	wholeT_0.01	CBN, CBN-A, OT-A
177	Yes	9	9-A	1000	McF_4	Inf	last	wholeT_0.5	CBN, CBN-A
178	Yes	9	9-A	1000	McF_4	Inf	unif	singleC	CBN, CBN-A
179	Yes	9	9-A	1000	McF_4	Inf	unif	wholeT_0.01	CBN, CBN-A
180	Yes	9	9-A	1000	McF_4	Inf	unif	wholeT_0.5	CBN, CBN-A
181	Yes	9	9-A	1000	McF_6	0	last	singleC	OT-A
182	Yes	9	9-A	1000	McF_6	0	last	wholeT_0.01	DiP-A
183	Yes	9	9-A	1000	McF_6	0	last	wholeT_0.5	OT-A
184	Yes	9	9-A	1000	McF_6	0	unif	singleC	CBN, CBN-A
185	Yes	9	9-A	1000	McF_6	0	unif	wholeT_0.01	CBN, CBN-A
186	Yes	9	9-A	1000	McF_6	0	unif	wholeT_0.5	CBN, CBN-A
187	Yes	9	9-A	1000	McF_6	Inf	last	singleC	OT-A
188	Yes	9	9-A	1000	McF_6	Inf	last	wholeT_0.01	OT-A
189	Yes	9	9-A	1000	McF_6	Inf	last	wholeT_0.5	DiP-A, OT-A
190	Yes	9	9-A	1000	McF_6	Inf	unif	singleC	CBN, CBN-A
191	Yes	9	9-A	1000	McF_6	Inf	unif	wholeT_0.01	CBN, CBN-A
192	Yes	9	9-A	1000	McF_6	Inf	unif	wholeT_0.5	CBN, CBN-A
193	Yes	9	9-A	200	Bozic	0	last	singleC	CBN, CBN-A
194	Yes	9	9-A	200	Bozic	0	last	wholeT_0.01	CBN, CBN-A
195	Yes	9	9-A	200	Bozic	0	last	wholeT_0.5	CBN, CBN-A
196	Yes	9	9-A	200	Bozic	0	unif	singleC	CBN, CBN-A
197	Yes	9	9-A	200	Bozic	0	unif	wholeT_0.01	CBN, CBN-A

Table 3: *(continued)*

	Conjunction	Drivers	Tree	S.Size	Model	sh	S.Time	S.Type	Best method(s)
198	Yes	9	9-A	200	Bozic	0	unif	wholeT_0.5	CBN, CBN-A
199	Yes	9	9-A	200	Bozic	Inf	last	singleC	CBN, CBN-A
200	Yes	9	9-A	200	Bozic	Inf	last	wholeT_0.01	CBN, CBN-A
201	Yes	9	9-A	200	Bozic	Inf	last	wholeT_0.5	CBN, CBN-A
202	Yes	9	9-A	200	Bozic	Inf	unif	singleC	CBN, CBN-A
203	Yes	9	9-A	200	Bozic	Inf	unif	wholeT_0.01	CBN, CBN-A
204	Yes	9	9-A	200	Bozic	Inf	unif	wholeT_0.5	CBN, CBN-A
205	Yes	9	9-A	200	exp	0	last	singleC	CBN, CBN-A
206	Yes	9	9-A	200	exp	0	last	wholeT_0.01	CBN, CBN-A
207	Yes	9	9-A	200	exp	0	last	wholeT_0.5	CBN, CBN-A
208	Yes	9	9-A	200	exp	0	unif	singleC	CBN, CBN-A
209	Yes	9	9-A	200	exp	0	unif	wholeT_0.01	CBN, CBN-A
210	Yes	9	9-A	200	exp	0	unif	wholeT_0.5	CBN, CBN-A
211	Yes	9	9-A	200	exp	Inf	last	singleC	CBN, CBN-A
212	Yes	9	9-A	200	exp	Inf	last	wholeT_0.01	CBN, CBN-A
213	Yes	9	9-A	200	exp	Inf	last	wholeT_0.5	CBN, CBN-A
214	Yes	9	9-A	200	exp	Inf	unif	singleC	CBN, CBN-A
215	Yes	9	9-A	200	exp	Inf	unif	wholeT_0.01	CBN, CBN-A
216	Yes	9	9-A	200	exp	Inf	unif	wholeT_0.5	CBN, CBN-A
217	Yes	9	9-A	200	McF_4	0	last	singleC	CBN, CBN-A, OT, OT-A
218	Yes	9	9-A	200	McF_4	0	last	wholeT_0.01	CBN, CBN-A, OT, OT-A
219	Yes	9	9-A	200	McF_4	0	last	wholeT_0.5	CBN, CBN-A
220	Yes	9	9-A	200	McF_4	0	unif	singleC	CBN, CBN-A
221	Yes	9	9-A	200	McF_4	0	unif	wholeT_0.01	CBN, CBN-A
222	Yes	9	9-A	200	McF_4	0	unif	wholeT_0.5	CBN, CBN-A
223	Yes	9	9-A	200	McF_4	Inf	last	singleC	CBN, CBN-A
224	Yes	9	9-A	200	McF_4	Inf	last	wholeT_0.01	CBN, OT-A
225	Yes	9	9-A	200	McF_4	Inf	last	wholeT_0.5	CBN, CBN-A
226	Yes	9	9-A	200	McF_4	Inf	unif	singleC	CBN, CBN-A
227	Yes	9	9-A	200	McF_4	Inf	unif	wholeT_0.01	CBN, CBN-A
228	Yes	9	9-A	200	McF_4	Inf	unif	wholeT_0.5	CBN, CBN-A
229	Yes	9	9-A	200	McF_6	0	last	singleC	OT-A
230	Yes	9	9-A	200	McF_6	0	last	wholeT_0.01	OT-A
231	Yes	9	9-A	200	McF_6	0	last	wholeT_0.5	OT-A
232	Yes	9	9-A	200	McF_6	0	unif	singleC	CBN, CBN-A
233	Yes	9	9-A	200	McF_6	0	unif	wholeT_0.01	CBN, CBN-A
234	Yes	9	9-A	200	McF_6	0	unif	wholeT_0.5	CBN, CBN-A
235	Yes	9	9-A	200	McF_6	Inf	last	singleC	OT-A
236	Yes	9	9-A	200	McF_6	Inf	last	wholeT_0.01	OT-A
237	Yes	9	9-A	200	McF_6	Inf	last	wholeT_0.5	OT-A

Table 3: *(continued)*

	Conjunction	Drivers	Tree	S.Size	Model	sh	S.Time	S.Type	Best method(s)
238	Yes	9	9-A	200	McF_6	Inf	unif	singleC	CBN, CBN-A
239	Yes	9	9-A	200	McF_6	Inf	unif	wholeT_0.01	CBN, CBN-A
240	Yes	9	9-A	200	McF_6	Inf	unif	wholeT_0.5	CBN, CBN-A
241	Yes	9	9-A	100	Bozic	0	last	singleC	CBN, CBN-A
242	Yes	9	9-A	100	Bozic	0	last	wholeT_0.01	CBN, CBN-A
243	Yes	9	9-A	100	Bozic	0	last	wholeT_0.5	CBN, CBN-A
244	Yes	9	9-A	100	Bozic	0	unif	singleC	CBN, CBN-A
245	Yes	9	9-A	100	Bozic	0	unif	wholeT_0.01	CBN, CBN-A
246	Yes	9	9-A	100	Bozic	0	unif	wholeT_0.5	CBN, CBN-A
247	Yes	9	9-A	100	Bozic	Inf	last	singleC	CBN, CBN-A
248	Yes	9	9-A	100	Bozic	Inf	last	wholeT_0.01	CBN, CBN-A
249	Yes	9	9-A	100	Bozic	Inf	last	wholeT_0.5	CBN, CBN-A
250	Yes	9	9-A	100	Bozic	Inf	unif	singleC	CBN, CBN-A
251	Yes	9	9-A	100	Bozic	Inf	unif	wholeT_0.01	CBN, CBN-A
252	Yes	9	9-A	100	Bozic	Inf	unif	wholeT_0.5	CBN, CBN-A
253	Yes	9	9-A	100	exp	0	last	singleC	CBN
254	Yes	9	9-A	100	exp	0	last	wholeT_0.01	CBN, CBN-A
255	Yes	9	9-A	100	exp	0	last	wholeT_0.5	CBN, CBN-A
256	Yes	9	9-A	100	exp	0	unif	singleC	CBN, CBN-A
257	Yes	9	9-A	100	exp	0	unif	wholeT_0.01	CBN, CBN-A
258	Yes	9	9-A	100	exp	0	unif	wholeT_0.5	CBN, CBN-A
259	Yes	9	9-A	100	exp	Inf	last	singleC	CBN, CBN-A
260	Yes	9	9-A	100	exp	Inf	last	wholeT_0.01	CBN, CBN-A
261	Yes	9	9-A	100	exp	Inf	last	wholeT_0.5	CBN, CBN-A
262	Yes	9	9-A	100	exp	Inf	unif	singleC	CBN, CBN-A
263	Yes	9	9-A	100	exp	Inf	unif	wholeT_0.01	CBN, CBN-A
264	Yes	9	9-A	100	exp	Inf	unif	wholeT_0.5	CBN, CBN-A
265	Yes	9	9-A	100	McF_4	0	last	singleC	CBN, CBN-A
266	Yes	9	9-A	100	McF_4	0	last	wholeT_0.01	CBN, CBN-A
267	Yes	9	9-A	100	McF_4	0	last	wholeT_0.5	CBN, CBN-A
268	Yes	9	9-A	100	McF_4	0	unif	singleC	CBN
269	Yes	9	9-A	100	McF_4	0	unif	wholeT_0.01	CBN, CBN-A
270	Yes	9	9-A	100	McF_4	0	unif	wholeT_0.5	CBN, CBN-A
271	Yes	9	9-A	100	McF_4	Inf	last	singleC	CBN, CBN-A
272	Yes	9	9-A	100	McF_4	Inf	last	wholeT_0.01	CBN
273	Yes	9	9-A	100	McF_4	Inf	last	wholeT_0.5	CBN, CBN-A
274	Yes	9	9-A	100	McF_4	Inf	unif	singleC	CBN, CBN-A
275	Yes	9	9-A	100	McF_4	Inf	unif	wholeT_0.01	CBN, CBN-A
276	Yes	9	9-A	100	McF_4	Inf	unif	wholeT_0.5	CBN, CBN-A
277	Yes	9	9-A	100	McF_6	0	last	singleC	OT-A

Table 3: *(continued)*

	Conjunction	Drivers	Tree	S.Size	Model	sh	S.Time	S.Type	Best method(s)
278	Yes	9	9-A	100	McF_6	0	last	wholeT_0.01	OT-A
279	Yes	9	9-A	100	McF_6	0	last	wholeT_0.5	OT-A
280	Yes	9	9-A	100	McF_6	0	unif	singleC	CBN, CBN-A
281	Yes	9	9-A	100	McF_6	0	unif	wholeT_0.01	CBN, CBN-A
282	Yes	9	9-A	100	McF_6	0	unif	wholeT_0.5	CBN, CBN-A
283	Yes	9	9-A	100	McF_6	Inf	last	singleC	OT-A
284	Yes	9	9-A	100	McF_6	Inf	last	wholeT_0.01	OT-A
285	Yes	9	9-A	100	McF_6	Inf	last	wholeT_0.5	CBN, OT-A
286	Yes	9	9-A	100	McF_6	Inf	unif	singleC	CBN, CBN-A
287	Yes	9	9-A	100	McF_6	Inf	unif	wholeT_0.01	CBN-A
288	Yes	9	9-A	100	McF_6	Inf	unif	wholeT_0.5	CBN, CBN-A
289	Yes	7	7-A	1000	Bozic	0	last	singleC	CBN, CBN-A, OT-A
290	Yes	7	7-A	1000	Bozic	0	last	wholeT_0.01	OT-A
291	Yes	7	7-A	1000	Bozic	0	last	wholeT_0.5	CBN, CBN-A, OT-A
292	Yes	7	7-A	1000	Bozic	0	unif	singleC	CBN, CBN-A
293	Yes	7	7-A	1000	Bozic	0	unif	wholeT_0.01	CBN, CBN-A
294	Yes	7	7-A	1000	Bozic	0	unif	wholeT_0.5	CBN, CBN-A
295	Yes	7	7-A	1000	Bozic	Inf	last	singleC	CBN, OT-A
296	Yes	7	7-A	1000	Bozic	Inf	last	wholeT_0.01	CBN-A
297	Yes	7	7-A	1000	Bozic	Inf	last	wholeT_0.5	OT-A
298	Yes	7	7-A	1000	Bozic	Inf	unif	singleC	CBN, CBN-A
299	Yes	7	7-A	1000	Bozic	Inf	unif	wholeT_0.01	CBN, CBN-A
300	Yes	7	7-A	1000	Bozic	Inf	unif	wholeT_0.5	CBN, CBN-A
301	Yes	7	7-A	1000	exp	0	last	singleC	CBN-A
302	Yes	7	7-A	1000	exp	0	last	wholeT_0.01	CBN, CBN-A, OT-A
303	Yes	7	7-A	1000	exp	0	last	wholeT_0.5	CBN, CBN-A
304	Yes	7	7-A	1000	exp	0	unif	singleC	CBN, CBN-A
305	Yes	7	7-A	1000	exp	0	unif	wholeT_0.01	CBN, CBN-A
306	Yes	7	7-A	1000	exp	0	unif	wholeT_0.5	CBN, CBN-A
307	Yes	7	7-A	1000	exp	Inf	last	singleC	CBN, CBN-A, OT-A
308	Yes	7	7-A	1000	exp	Inf	last	wholeT_0.01	CBN-A
309	Yes	7	7-A	1000	exp	Inf	last	wholeT_0.5	CBN, CBN-A
310	Yes	7	7-A	1000	exp	Inf	unif	singleC	CBN, CBN-A
311	Yes	7	7-A	1000	exp	Inf	unif	wholeT_0.01	CBN, CBN-A
312	Yes	7	7-A	1000	exp	Inf	unif	wholeT_0.5	CBN, CBN-A
313	Yes	7	7-A	1000	McF_4	0	last	singleC	CBN, OT-A
314	Yes	7	7-A	1000	McF_4	0	last	wholeT_0.01	OT-A
315	Yes	7	7-A	1000	McF_4	0	last	wholeT_0.5	CBN, OT-A
316	Yes	7	7-A	1000	McF_4	0	unif	singleC	CBN, CBN-A, OT, OT-A
317	Yes	7	7-A	1000	McF_4	0	unif	wholeT_0.01	CBN-A, DiP, DiP-A, OT, OT-A

Table 3: *(continued)*

	Conjunction	Drivers	Tree	S.Size	Model	sh	S.Time	S.Type	Best method(s)
318	Yes	7	7-A	1000	McF_4	0	unif	wholeT_0.5	CBN, CBN-A, OT, OT-A
319	Yes	7	7-A	1000	McF_4	Inf	last	singleC	CBN, OT-A
320	Yes	7	7-A	1000	McF_4	Inf	last	wholeT_0.01	CBN, OT-A
321	Yes	7	7-A	1000	McF_4	Inf	last	wholeT_0.5	CBN
322	Yes	7	7-A	1000	McF_4	Inf	unif	singleC	CBN, CBN-A
323	Yes	7	7-A	1000	McF_4	Inf	unif	wholeT_0.01	CBN, CBN-A, OT, OT-A
324	Yes	7	7-A	1000	McF_4	Inf	unif	wholeT_0.5	CBN, CBN-A
325	Yes	7	7-A	1000	McF_6	0	last	singleC	DiP-A
326	Yes	7	7-A	1000	McF_6	0	last	wholeT_0.01	DiP-A, OT-A
327	Yes	7	7-A	1000	McF_6	0	last	wholeT_0.5	DiP-A
328	Yes	7	7-A	1000	McF_6	0	unif	singleC	CBN-A
329	Yes	7	7-A	1000	McF_6	0	unif	wholeT_0.01	CBN-A, DiP, DiP-A
330	Yes	7	7-A	1000	McF_6	0	unif	wholeT_0.5	CBN-A
331	Yes	7	7-A	1000	McF_6	Inf	last	singleC	CBN-A, DiP-A
332	Yes	7	7-A	1000	McF_6	Inf	last	wholeT_0.01	DiP-A
333	Yes	7	7-A	1000	McF_6	Inf	last	wholeT_0.5	CBN-A, DiP-A
334	Yes	7	7-A	1000	McF_6	Inf	unif	singleC	CBN-A
335	Yes	7	7-A	1000	McF_6	Inf	unif	wholeT_0.01	CBN-A
336	Yes	7	7-A	1000	McF_6	Inf	unif	wholeT_0.5	CBN-A
337	Yes	7	7-A	200	Bozic	0	last	singleC	CBN, CBN-A
338	Yes	7	7-A	200	Bozic	0	last	wholeT_0.01	CBN, OT-A
339	Yes	7	7-A	200	Bozic	0	last	wholeT_0.5	CBN, CBN-A
340	Yes	7	7-A	200	Bozic	0	unif	singleC	CBN, CBN-A
341	Yes	7	7-A	200	Bozic	0	unif	wholeT_0.01	CBN, CBN-A
342	Yes	7	7-A	200	Bozic	0	unif	wholeT_0.5	CBN, CBN-A
343	Yes	7	7-A	200	Bozic	Inf	last	singleC	CBN, CBN-A, OT-A
344	Yes	7	7-A	200	Bozic	Inf	last	wholeT_0.01	CBN-A
345	Yes	7	7-A	200	Bozic	Inf	last	wholeT_0.5	CBN, OT-A
346	Yes	7	7-A	200	Bozic	Inf	unif	singleC	CBN, CBN-A
347	Yes	7	7-A	200	Bozic	Inf	unif	wholeT_0.01	CBN, CBN-A
348	Yes	7	7-A	200	Bozic	Inf	unif	wholeT_0.5	CBN, CBN-A
349	Yes	7	7-A	200	exp	0	last	singleC	CBN, CBN-A
350	Yes	7	7-A	200	exp	0	last	wholeT_0.01	CBN, CBN-A
351	Yes	7	7-A	200	exp	0	last	wholeT_0.5	CBN, CBN-A
352	Yes	7	7-A	200	exp	0	unif	singleC	CBN, CBN-A
353	Yes	7	7-A	200	exp	0	unif	wholeT_0.01	CBN, CBN-A
354	Yes	7	7-A	200	exp	0	unif	wholeT_0.5	CBN, CBN-A
355	Yes	7	7-A	200	exp	Inf	last	singleC	CBN
356	Yes	7	7-A	200	exp	Inf	last	wholeT_0.01	CBN-A
357	Yes	7	7-A	200	exp	Inf	last	wholeT_0.5	CBN

Table 3: *(continued)*

	Conjunction	Drivers	Tree	S.Size	Model	sh	S.Time	S.Type	Best method(s)
358	Yes	7	7-A	200	exp	Inf	unif	singleC	CBN, CBN-A
359	Yes	7	7-A	200	exp	Inf	unif	wholeT_0.01	CBN-A
360	Yes	7	7-A	200	exp	Inf	unif	wholeT_0.5	CBN, CBN-A
361	Yes	7	7-A	200	McF_4	0	last	singleC	CBN, OT-A
362	Yes	7	7-A	200	McF_4	0	last	wholeT_0.01	OT-A
363	Yes	7	7-A	200	McF_4	0	last	wholeT_0.5	CBN, OT-A
364	Yes	7	7-A	200	McF_4	0	unif	singleC	CBN, CBN-A
365	Yes	7	7-A	200	McF_4	0	unif	wholeT_0.01	CBN, CBN-A, OT, OT-A
366	Yes	7	7-A	200	McF_4	0	unif	wholeT_0.5	CBN, CBN-A
367	Yes	7	7-A	200	McF_4	Inf	last	singleC	CBN
368	Yes	7	7-A	200	McF_4	Inf	last	wholeT_0.01	CBN, OT-A
369	Yes	7	7-A	200	McF_4	Inf	last	wholeT_0.5	CBN, CBN-A
370	Yes	7	7-A	200	McF_4	Inf	unif	singleC	CBN, CBN-A
371	Yes	7	7-A	200	McF_4	Inf	unif	wholeT_0.01	CBN, CBN-A
372	Yes	7	7-A	200	McF_4	Inf	unif	wholeT_0.5	CBN, CBN-A
373	Yes	7	7-A	200	McF_6	0	last	singleC	CBN, CBN-A, OT-A
374	Yes	7	7-A	200	McF_6	0	last	wholeT_0.01	OT-A
375	Yes	7	7-A	200	McF_6	0	last	wholeT_0.5	CBN-A, OT-A
376	Yes	7	7-A	200	McF_6	0	unif	singleC	CBN-A
377	Yes	7	7-A	200	McF_6	0	unif	wholeT_0.01	CBN-A, OT, OT-A
378	Yes	7	7-A	200	McF_6	0	unif	wholeT_0.5	CBN-A
379	Yes	7	7-A	200	McF_6	Inf	last	singleC	CBN, CBN-A, OT-A
380	Yes	7	7-A	200	McF_6	Inf	last	wholeT_0.01	OT-A
381	Yes	7	7-A	200	McF_6	Inf	last	wholeT_0.5	CBN, CBN-A, OT-A
382	Yes	7	7-A	200	McF_6	Inf	unif	singleC	CBN-A
383	Yes	7	7-A	200	McF_6	Inf	unif	wholeT_0.01	CBN-A
384	Yes	7	7-A	200	McF_6	Inf	unif	wholeT_0.5	CBN-A
385	Yes	7	7-A	100	Bozic	0	last	singleC	CBN, CBN-A
386	Yes	7	7-A	100	Bozic	0	last	wholeT_0.01	CBN, OT-A
387	Yes	7	7-A	100	Bozic	0	last	wholeT_0.5	CBN, CBN-A
388	Yes	7	7-A	100	Bozic	0	unif	singleC	CBN, CBN-A
389	Yes	7	7-A	100	Bozic	0	unif	wholeT_0.01	CBN, CBN-A
390	Yes	7	7-A	100	Bozic	0	unif	wholeT_0.5	CBN, CBN-A
391	Yes	7	7-A	100	Bozic	Inf	last	singleC	CBN, CBN-A, OT-A
392	Yes	7	7-A	100	Bozic	Inf	last	wholeT_0.01	CBN-A
393	Yes	7	7-A	100	Bozic	Inf	last	wholeT_0.5	CBN, CBN-A
394	Yes	7	7-A	100	Bozic	Inf	unif	singleC	CBN, CBN-A
395	Yes	7	7-A	100	Bozic	Inf	unif	wholeT_0.01	CBN, CBN-A
396	Yes	7	7-A	100	Bozic	Inf	unif	wholeT_0.5	CBN, CBN-A
397	Yes	7	7-A	100	exp	0	last	singleC	CBN, CBN-A

Table 3: *(continued)*

	Conjunction	Drivers	Tree	S.Size	Model	sh	S.Time	S.Type	Best method(s)
398	Yes	7	7-A	100	exp	0	last	wholeT_0.01	CBN, CBN-A
399	Yes	7	7-A	100	exp	0	last	wholeT_0.5	CBN-A
400	Yes	7	7-A	100	exp	0	unif	singleC	CBN, CBN-A
401	Yes	7	7-A	100	exp	0	unif	wholeT_0.01	CBN, CBN-A
402	Yes	7	7-A	100	exp	0	unif	wholeT_0.5	CBN, CBN-A
403	Yes	7	7-A	100	exp	Inf	last	singleC	CBN, CBN-A
404	Yes	7	7-A	100	exp	Inf	last	wholeT_0.01	CBN-A
405	Yes	7	7-A	100	exp	Inf	last	wholeT_0.5	CBN
406	Yes	7	7-A	100	exp	Inf	unif	singleC	CBN, CBN-A
407	Yes	7	7-A	100	exp	Inf	unif	wholeT_0.01	CBN, CBN-A
408	Yes	7	7-A	100	exp	Inf	unif	wholeT_0.5	CBN, CBN-A
409	Yes	7	7-A	100	McF_4	0	last	singleC	CBN, CBN-A, OT-A
410	Yes	7	7-A	100	McF_4	0	last	wholeT_0.01	OT-A
411	Yes	7	7-A	100	McF_4	0	last	wholeT_0.5	CBN, OT-A
412	Yes	7	7-A	100	McF_4	0	unif	singleC	CBN-A
413	Yes	7	7-A	100	McF_4	0	unif	wholeT_0.01	CBN, CBN-A, OT-A
414	Yes	7	7-A	100	McF_4	0	unif	wholeT_0.5	CBN-A
415	Yes	7	7-A	100	McF_4	Inf	last	singleC	CBN, CBN-A
416	Yes	7	7-A	100	McF_4	Inf	last	wholeT_0.01	CBN, CBN-A, OT-A
417	Yes	7	7-A	100	McF_4	Inf	last	wholeT_0.5	CBN, CBN-A
418	Yes	7	7-A	100	McF_4	Inf	unif	singleC	CBN, CBN-A
419	Yes	7	7-A	100	McF_4	Inf	unif	wholeT_0.01	CBN, CBN-A
420	Yes	7	7-A	100	McF_4	Inf	unif	wholeT_0.5	CBN, CBN-A
421	Yes	7	7-A	100	McF_6	0	last	singleC	CBN, CBN-A, OT-A
422	Yes	7	7-A	100	McF_6	0	last	wholeT_0.01	OT-A
423	Yes	7	7-A	100	McF_6	0	last	wholeT_0.5	CBN-A, OT-A
424	Yes	7	7-A	100	McF_6	0	unif	singleC	CBN, CBN-A
425	Yes	7	7-A	100	McF_6	0	unif	wholeT_0.01	CBN, CBN-A, OT, OT-A
426	Yes	7	7-A	100	McF_6	0	unif	wholeT_0.5	CBN-A
427	Yes	7	7-A	100	McF_6	Inf	last	singleC	CBN, CBN-A, OT-A
428	Yes	7	7-A	100	McF_6	Inf	last	wholeT_0.01	OT-A
429	Yes	7	7-A	100	McF_6	Inf	last	wholeT_0.5	CBN-A, OT-A
430	Yes	7	7-A	100	McF_6	Inf	unif	singleC	CBN-A
431	Yes	7	7-A	100	McF_6	Inf	unif	wholeT_0.01	CBN-A
432	Yes	7	7-A	100	McF_6	Inf	unif	wholeT_0.5	CBN-A
433	No	11	11-B	1000	Bozic	0	last	singleC	CBN, CBN-A, OT, OT-A
434	No	11	11-B	1000	Bozic	0	last	wholeT_0.01	OT, OT-A
435	No	11	11-B	1000	Bozic	0	last	wholeT_0.5	CBN, CBN-A
436	No	11	11-B	1000	Bozic	0	unif	singleC	CBN, CBN-A
437	No	11	11-B	1000	Bozic	0	unif	wholeT_0.01	CBN, CBN-A

Table 3: *(continued)*

	Conjunction	Drivers	Tree	S.Size	Model	sh	S.Time	S.Type	Best method(s)
438	No	11	11-B	1000	Bozic	0	unif	wholeT_0.5	CBN, CBN-A
439	No	11	11-B	1000	Bozic	Inf	last	singleC	CBN-A, OT, OT-A
440	No	11	11-B	1000	Bozic	Inf	last	wholeT_0.01	CBN, CBN-A, OT, OT-A
441	No	11	11-B	1000	Bozic	Inf	last	wholeT_0.5	CBN, CBN-A, OT, OT-A
442	No	11	11-B	1000	Bozic	Inf	unif	singleC	CBN, CBN-A
443	No	11	11-B	1000	Bozic	Inf	unif	wholeT_0.01	CBN, CBN-A, OT, OT-A
444	No	11	11-B	1000	Bozic	Inf	unif	wholeT_0.5	CBN, CBN-A
445	No	11	11-B	1000	exp	0	last	singleC	CBN, CBN-A
446	No	11	11-B	1000	exp	0	last	wholeT_0.01	CBN, CBN-A
447	No	11	11-B	1000	exp	0	last	wholeT_0.5	CBN, CBN-A
448	No	11	11-B	1000	exp	0	unif	singleC	CBN, CBN-A
449	No	11	11-B	1000	exp	0	unif	wholeT_0.01	CBN, CBN-A
450	No	11	11-B	1000	exp	0	unif	wholeT_0.5	CBN, CBN-A
451	No	11	11-B	1000	exp	Inf	last	singleC	CBN, CBN-A, OT, OT-A
452	No	11	11-B	1000	exp	Inf	last	wholeT_0.01	CBN, CBN-A, OT, OT-A
453	No	11	11-B	1000	exp	Inf	last	wholeT_0.5	CBN-A, OT, OT-A
454	No	11	11-B	1000	exp	Inf	unif	singleC	CBN, CBN-A
455	No	11	11-B	1000	exp	Inf	unif	wholeT_0.01	CBN, CBN-A
456	No	11	11-B	1000	exp	Inf	unif	wholeT_0.5	CBN, CBN-A
457	No	11	11-B	1000	McF_4	0	last	singleC	OT, OT-A
458	No	11	11-B	1000	McF_4	0	last	wholeT_0.01	DiP, DiP-A, OT, OT-A
459	No	11	11-B	1000	McF_4	0	last	wholeT_0.5	OT, OT-A
460	No	11	11-B	1000	McF_4	0	unif	singleC	OT, OT-A
461	No	11	11-B	1000	McF_4	0	unif	wholeT_0.01	CBN-A, OT, OT-A
462	No	11	11-B	1000	McF_4	0	unif	wholeT_0.5	CBN, OT, OT-A
463	No	11	11-B	1000	McF_4	Inf	last	singleC	OT, OT-A
464	No	11	11-B	1000	McF_4	Inf	last	wholeT_0.01	OT, OT-A
465	No	11	11-B	1000	McF_4	Inf	last	wholeT_0.5	CBN-A, DiP-A, OT, OT-A
466	No	11	11-B	1000	McF_4	Inf	unif	singleC	CBN, CBN-A, OT, OT-A
467	No	11	11-B	1000	McF_4	Inf	unif	wholeT_0.01	CBN, CBN-A, OT, OT-A
468	No	11	11-B	1000	McF_4	Inf	unif	wholeT_0.5	CBN, CBN-A, OT, OT-A
469	No	11	11-B	1000	McF_6	0	last	singleC	DiP, DiP-A, OT, OT-A
470	No	11	11-B	1000	McF_6	0	last	wholeT_0.01	DiP, DiP-A, OT, OT-A
471	No	11	11-B	1000	McF_6	0	last	wholeT_0.5	DiP, DiP-A, OT, OT-A
472	No	11	11-B	1000	McF_6	0	unif	singleC	DiP, DiP-A, OT, OT-A
473	No	11	11-B	1000	McF_6	0	unif	wholeT_0.01	DiP, DiP-A, OT, OT-A
474	No	11	11-B	1000	McF_6	0	unif	wholeT_0.5	DiP, DiP-A, OT, OT-A
475	No	11	11-B	1000	McF_6	Inf	last	singleC	DiP, DiP-A, OT, OT-A
476	No	11	11-B	1000	McF_6	Inf	last	wholeT_0.01	DiP-A, OT, OT-A
477	No	11	11-B	1000	McF_6	Inf	last	wholeT_0.5	DiP, DiP-A, OT, OT-A

Table 3: *(continued)*

	Conjunction	Drivers	Tree	S.Size	Model	sh	S.Time	S.Type	Best method(s)
478	No	11	11-B	1000	McF_6	Inf	unif	singleC	DiP, DiP-A, OT, OT-A
479	No	11	11-B	1000	McF_6	Inf	unif	wholeT_0.01	DiP, DiP-A, OT, OT-A
480	No	11	11-B	1000	McF_6	Inf	unif	wholeT_0.5	DiP, DiP-A, OT, OT-A
481	No	11	11-B	200	Bozic	0	last	singleC	CBN, CBN-A
482	No	11	11-B	200	Bozic	0	last	wholeT_0.01	CBN, CBN-A
483	No	11	11-B	200	Bozic	0	last	wholeT_0.5	CBN, CBN-A
484	No	11	11-B	200	Bozic	0	unif	singleC	CBN, CBN-A
485	No	11	11-B	200	Bozic	0	unif	wholeT_0.01	CBN, CBN-A
486	No	11	11-B	200	Bozic	0	unif	wholeT_0.5	CBN, CBN-A
487	No	11	11-B	200	Bozic	Inf	last	singleC	CBN, CBN-A, OT, OT-A
488	No	11	11-B	200	Bozic	Inf	last	wholeT_0.01	CBN, CBN-A, OT, OT-A
489	No	11	11-B	200	Bozic	Inf	last	wholeT_0.5	CBN, CBN-A, OT, OT-A
490	No	11	11-B	200	Bozic	Inf	unif	singleC	CBN, CBN-A
491	No	11	11-B	200	Bozic	Inf	unif	wholeT_0.01	CBN, CBN-A
492	No	11	11-B	200	Bozic	Inf	unif	wholeT_0.5	CBN, CBN-A
493	No	11	11-B	200	exp	0	last	singleC	CBN, CBN-A
494	No	11	11-B	200	exp	0	last	wholeT_0.01	CBN, CBN-A
495	No	11	11-B	200	exp	0	last	wholeT_0.5	CBN, CBN-A
496	No	11	11-B	200	exp	0	unif	singleC	CBN, CBN-A
497	No	11	11-B	200	exp	0	unif	wholeT_0.01	CBN, CBN-A
498	No	11	11-B	200	exp	0	unif	wholeT_0.5	CBN, CBN-A
499	No	11	11-B	200	exp	Inf	last	singleC	CBN, CBN-A, OT, OT-A
500	No	11	11-B	200	exp	Inf	last	wholeT_0.01	CBN, CBN-A
501	No	11	11-B	200	exp	Inf	last	wholeT_0.5	CBN, CBN-A, OT, OT-A
502	No	11	11-B	200	exp	Inf	unif	singleC	CBN, CBN-A
503	No	11	11-B	200	exp	Inf	unif	wholeT_0.01	CBN, CBN-A
504	No	11	11-B	200	exp	Inf	unif	wholeT_0.5	CBN, CBN-A
505	No	11	11-B	200	McF_4	0	last	singleC	OT, OT-A
506	No	11	11-B	200	McF_4	0	last	wholeT_0.01	OT, OT-A
507	No	11	11-B	200	McF_4	0	last	wholeT_0.5	OT, OT-A
508	No	11	11-B	200	McF_4	0	unif	singleC	CBN, CBN-A
509	No	11	11-B	200	McF_4	0	unif	wholeT_0.01	CBN, CBN-A
510	No	11	11-B	200	McF_4	0	unif	wholeT_0.5	CBN, CBN-A
511	No	11	11-B	200	McF_4	Inf	last	singleC	CBN, CBN-A, OT, OT-A
512	No	11	11-B	200	McF_4	Inf	last	wholeT_0.01	OT, OT-A
513	No	11	11-B	200	McF_4	Inf	last	wholeT_0.5	CBN, CBN-A, OT, OT-A
514	No	11	11-B	200	McF_4	Inf	unif	singleC	CBN, CBN-A
515	No	11	11-B	200	McF_4	Inf	unif	wholeT_0.01	CBN, CBN-A
516	No	11	11-B	200	McF_4	Inf	unif	wholeT_0.5	CBN, CBN-A
517	No	11	11-B	200	McF_6	0	last	singleC	OT, OT-A

Table 3: *(continued)*

	Conjunction	Drivers	Tree	S.Size	Model	sh	S.Time	S.Type	Best method(s)
518	No	11	11-B	200	McF_6	0	last	wholeT_0.01	OT, OT-A
519	No	11	11-B	200	McF_6	0	last	wholeT_0.5	OT, OT-A
520	No	11	11-B	200	McF_6	0	unif	singleC	OT, OT-A
521	No	11	11-B	200	McF_6	0	unif	wholeT_0.01	CBN-A, OT, OT-A
522	No	11	11-B	200	McF_6	0	unif	wholeT_0.5	OT, OT-A
523	No	11	11-B	200	McF_6	Inf	last	singleC	OT, OT-A
524	No	11	11-B	200	McF_6	Inf	last	wholeT_0.01	OT, OT-A
525	No	11	11-B	200	McF_6	Inf	last	wholeT_0.5	OT, OT-A
526	No	11	11-B	200	McF_6	Inf	unif	singleC	OT, OT-A
527	No	11	11-B	200	McF_6	Inf	unif	wholeT_0.01	OT, OT-A
528	No	11	11-B	200	McF_6	Inf	unif	wholeT_0.5	OT, OT-A
529	No	11	11-B	100	Bozic	0	last	singleC	CBN, CBN-A
530	No	11	11-B	100	Bozic	0	last	wholeT_0.01	CBN, CBN-A
531	No	11	11-B	100	Bozic	0	last	wholeT_0.5	CBN, CBN-A
532	No	11	11-B	100	Bozic	0	unif	singleC	CBN, CBN-A
533	No	11	11-B	100	Bozic	0	unif	wholeT_0.01	CBN, CBN-A
534	No	11	11-B	100	Bozic	0	unif	wholeT_0.5	CBN, CBN-A
535	No	11	11-B	100	Bozic	Inf	last	singleC	CBN, CBN-A, OT, OT-A
536	No	11	11-B	100	Bozic	Inf	last	wholeT_0.01	CBN, CBN-A, OT, OT-A
537	No	11	11-B	100	Bozic	Inf	last	wholeT_0.5	CBN, CBN-A, OT, OT-A
538	No	11	11-B	100	Bozic	Inf	unif	singleC	CBN, CBN-A
539	No	11	11-B	100	Bozic	Inf	unif	wholeT_0.01	CBN, CBN-A
540	No	11	11-B	100	Bozic	Inf	unif	wholeT_0.5	CBN, CBN-A
541	No	11	11-B	100	exp	0	last	singleC	CBN, CBN-A
542	No	11	11-B	100	exp	0	last	wholeT_0.01	CBN, CBN-A
543	No	11	11-B	100	exp	0	last	wholeT_0.5	CBN, CBN-A
544	No	11	11-B	100	exp	0	unif	singleC	CBN, CBN-A
545	No	11	11-B	100	exp	0	unif	wholeT_0.01	CBN, CBN-A
546	No	11	11-B	100	exp	0	unif	wholeT_0.5	CBN, CBN-A
547	No	11	11-B	100	exp	Inf	last	singleC	CBN, CBN-A
548	No	11	11-B	100	exp	Inf	last	wholeT_0.01	CBN, CBN-A
549	No	11	11-B	100	exp	Inf	last	wholeT_0.5	CBN, CBN-A
550	No	11	11-B	100	exp	Inf	unif	singleC	CBN, CBN-A
551	No	11	11-B	100	exp	Inf	unif	wholeT_0.01	CBN, CBN-A
552	No	11	11-B	100	exp	Inf	unif	wholeT_0.5	CBN, CBN-A
553	No	11	11-B	100	McF_4	0	last	singleC	CBN-A, OT, OT-A
554	No	11	11-B	100	McF_4	0	last	wholeT_0.01	OT, OT-A
555	No	11	11-B	100	McF_4	0	last	wholeT_0.5	CBN-A, OT, OT-A
556	No	11	11-B	100	McF_4	0	unif	singleC	CBN, CBN-A
557	No	11	11-B	100	McF_4	0	unif	wholeT_0.01	CBN, CBN-A

Table 3: *(continued)*

	Conjunction	Drivers	Tree	S.Size	Model	sh	S.Time	S.Type	Best method(s)
558	No	11	11-B	100	McF_4	0	unif	wholeT_0.5	CBN, CBN-A
559	No	11	11-B	100	McF_4	Inf	last	singleC	CBN, CBN-A, OT, OT-A
560	No	11	11-B	100	McF_4	Inf	last	wholeT_0.01	OT, OT-A
561	No	11	11-B	100	McF_4	Inf	last	wholeT_0.5	CBN-A, OT, OT-A
562	No	11	11-B	100	McF_4	Inf	unif	singleC	CBN, CBN-A
563	No	11	11-B	100	McF_4	Inf	unif	wholeT_0.01	CBN, CBN-A
564	No	11	11-B	100	McF_4	Inf	unif	wholeT_0.5	CBN, CBN-A
565	No	11	11-B	100	McF_6	0	last	singleC	OT, OT-A
566	No	11	11-B	100	McF_6	0	last	wholeT_0.01	OT, OT-A
567	No	11	11-B	100	McF_6	0	last	wholeT_0.5	OT, OT-A
568	No	11	11-B	100	McF_6	0	unif	singleC	CBN, CBN-A, OT, OT-A
569	No	11	11-B	100	McF_6	0	unif	wholeT_0.01	OT, OT-A
570	No	11	11-B	100	McF_6	0	unif	wholeT_0.5	OT, OT-A
571	No	11	11-B	100	McF_6	Inf	last	singleC	OT, OT-A
572	No	11	11-B	100	McF_6	Inf	last	wholeT_0.01	OT, OT-A
573	No	11	11-B	100	McF_6	Inf	last	wholeT_0.5	OT, OT-A
574	No	11	11-B	100	McF_6	Inf	unif	singleC	CBN, CBN-A, OT, OT-A
575	No	11	11-B	100	McF_6	Inf	unif	wholeT_0.01	CBN, CBN-A, OT, OT-A
576	No	11	11-B	100	McF_6	Inf	unif	wholeT_0.5	CBN, CBN-A, OT, OT-A
577	No	9	9-B	1000	Bozic	0	last	singleC	CBN, CBN-A, OT, OT-A
578	No	9	9-B	1000	Bozic	0	last	wholeT_0.01	OT, OT-A
579	No	9	9-B	1000	Bozic	0	last	wholeT_0.5	CBN, CBN-A, OT, OT-A
580	No	9	9-B	1000	Bozic	0	unif	singleC	CBN, CBN-A
581	No	9	9-B	1000	Bozic	0	unif	wholeT_0.01	CBN, CBN-A
582	No	9	9-B	1000	Bozic	0	unif	wholeT_0.5	CBN, CBN-A
583	No	9	9-B	1000	Bozic	Inf	last	singleC	OT, OT-A
584	No	9	9-B	1000	Bozic	Inf	last	wholeT_0.01	DiP, DiP-A, OT, OT-A
585	No	9	9-B	1000	Bozic	Inf	last	wholeT_0.5	OT, OT-A
586	No	9	9-B	1000	Bozic	Inf	unif	singleC	CBN, OT, OT-A
587	No	9	9-B	1000	Bozic	Inf	unif	wholeT_0.01	CBN-A, OT, OT-A
588	No	9	9-B	1000	Bozic	Inf	unif	wholeT_0.5	CBN, CBN-A, OT, OT-A
589	No	9	9-B	1000	exp	0	last	singleC	CBN, CBN-A
590	No	9	9-B	1000	exp	0	last	wholeT_0.01	CBN, CBN-A
591	No	9	9-B	1000	exp	0	last	wholeT_0.5	CBN, CBN-A
592	No	9	9-B	1000	exp	0	unif	singleC	CBN, CBN-A
593	No	9	9-B	1000	exp	0	unif	wholeT_0.01	CBN, CBN-A
594	No	9	9-B	1000	exp	0	unif	wholeT_0.5	CBN, CBN-A
595	No	9	9-B	1000	exp	Inf	last	singleC	OT, OT-A
596	No	9	9-B	1000	exp	Inf	last	wholeT_0.01	OT, OT-A
597	No	9	9-B	1000	exp	Inf	last	wholeT_0.5	OT, OT-A

Table 3: *(continued)*

	Conjunction	Drivers	Tree	S.Size	Model	sh	S.Time	S.Type	Best method(s)
598	No	9	9-B	1000	exp	Inf	unif	singleC	CBN, CBN-A, OT, OT-A
599	No	9	9-B	1000	exp	Inf	unif	wholeT_0.01	CBN, CBN-A, OT, OT-A
600	No	9	9-B	1000	exp	Inf	unif	wholeT_0.5	CBN, CBN-A
601	No	9	9-B	1000	McF_4	0	last	singleC	DiP-A, OT, OT-A
602	No	9	9-B	1000	McF_4	0	last	wholeT_0.01	DiP, DiP-A, OT, OT-A
603	No	9	9-B	1000	McF_4	0	last	wholeT_0.5	OT, OT-A
604	No	9	9-B	1000	McF_4	0	unif	singleC	OT, OT-A
605	No	9	9-B	1000	McF_4	0	unif	wholeT_0.01	OT, OT-A
606	No	9	9-B	1000	McF_4	0	unif	wholeT_0.5	OT, OT-A
607	No	9	9-B	1000	McF_4	Inf	last	singleC	OT, OT-A
608	No	9	9-B	1000	McF_4	Inf	last	wholeT_0.01	DiP, DiP-A, OT, OT-A
609	No	9	9-B	1000	McF_4	Inf	last	wholeT_0.5	OT, OT-A
610	No	9	9-B	1000	McF_4	Inf	unif	singleC	OT, OT-A
611	No	9	9-B	1000	McF_4	Inf	unif	wholeT_0.01	OT, OT-A
612	No	9	9-B	1000	McF_4	Inf	unif	wholeT_0.5	OT, OT-A
613	No	9	9-B	1000	McF_6	0	last	singleC	DiP-A, OT-A
614	No	9	9-B	1000	McF_6	0	last	wholeT_0.01	DiP-A, OT-A
615	No	9	9-B	1000	McF_6	0	last	wholeT_0.5	DiP-A, OT-A
616	No	9	9-B	1000	McF_6	0	unif	singleC	DiP, DiP-A, OT, OT-A
617	No	9	9-B	1000	McF_6	0	unif	wholeT_0.01	DiP, DiP-A, OT, OT-A
618	No	9	9-B	1000	McF_6	0	unif	wholeT_0.5	CBN, CBN-A, DiP, DiP-A, OT, OT-A
619	No	9	9-B	1000	McF_6	Inf	last	singleC	DiP-A, OT-A
620	No	9	9-B	1000	McF_6	Inf	last	wholeT_0.01	DiP-A, OT-A
621	No	9	9-B	1000	McF_6	Inf	last	wholeT_0.5	DiP-A, OT-A
622	No	9	9-B	1000	McF_6	Inf	unif	singleC	CBN-A, DiP, DiP-A, OT, OT-A
623	No	9	9-B	1000	McF_6	Inf	unif	wholeT_0.01	DiP, DiP-A, OT, OT-A
624	No	9	9-B	1000	McF_6	Inf	unif	wholeT_0.5	DiP, DiP-A, OT, OT-A
625	No	9	9-B	200	Bozic	0	last	singleC	CBN, CBN-A, OT, OT-A
626	No	9	9-B	200	Bozic	0	last	wholeT_0.01	CBN, CBN-A, OT, OT-A
627	No	9	9-B	200	Bozic	0	last	wholeT_0.5	CBN, CBN-A
628	No	9	9-B	200	Bozic	0	unif	singleC	CBN, CBN-A
629	No	9	9-B	200	Bozic	0	unif	wholeT_0.01	CBN, CBN-A
630	No	9	9-B	200	Bozic	0	unif	wholeT_0.5	CBN, CBN-A
631	No	9	9-B	200	Bozic	Inf	last	singleC	OT, OT-A
632	No	9	9-B	200	Bozic	Inf	last	wholeT_0.01	CBN, OT, OT-A
633	No	9	9-B	200	Bozic	Inf	last	wholeT_0.5	OT, OT-A
634	No	9	9-B	200	Bozic	Inf	unif	singleC	CBN, CBN-A
635	No	9	9-B	200	Bozic	Inf	unif	wholeT_0.01	CBN-A
636	No	9	9-B	200	Bozic	Inf	unif	wholeT_0.5	CBN, CBN-A
637	No	9	9-B	200	exp	0	last	singleC	CBN, CBN-A

Table 3: *(continued)*

	Conjunction	Drivers	Tree	S.Size	Model	sh	S.Time	S.Type	Best method(s)
638	No	9	9-B	200	exp	0	last	wholeT_0.01	CBN, CBN-A
639	No	9	9-B	200	exp	0	last	wholeT_0.5	CBN, CBN-A
640	No	9	9-B	200	exp	0	unif	singleC	CBN, CBN-A
641	No	9	9-B	200	exp	0	unif	wholeT_0.01	CBN, CBN-A
642	No	9	9-B	200	exp	0	unif	wholeT_0.5	CBN, CBN-A
643	No	9	9-B	200	exp	Inf	last	singleC	CBN, CBN-A, OT, OT-A
644	No	9	9-B	200	exp	Inf	last	wholeT_0.01	OT, OT-A
645	No	9	9-B	200	exp	Inf	last	wholeT_0.5	CBN, CBN-A, OT, OT-A
646	No	9	9-B	200	exp	Inf	unif	singleC	CBN, CBN-A
647	No	9	9-B	200	exp	Inf	unif	wholeT_0.01	CBN, CBN-A
648	No	9	9-B	200	exp	Inf	unif	wholeT_0.5	CBN, CBN-A
649	No	9	9-B	200	McF_4	0	last	singleC	OT, OT-A
650	No	9	9-B	200	McF_4	0	last	wholeT_0.01	OT, OT-A
651	No	9	9-B	200	McF_4	0	last	wholeT_0.5	OT, OT-A
652	No	9	9-B	200	McF_4	0	unif	singleC	CBN, CBN-A, OT, OT-A
653	No	9	9-B	200	McF_4	0	unif	wholeT_0.01	CBN, OT, OT-A
654	No	9	9-B	200	McF_4	0	unif	wholeT_0.5	CBN, CBN-A, OT, OT-A
655	No	9	9-B	200	McF_4	Inf	last	singleC	OT, OT-A
656	No	9	9-B	200	McF_4	Inf	last	wholeT_0.01	OT, OT-A
657	No	9	9-B	200	McF_4	Inf	last	wholeT_0.5	OT, OT-A
658	No	9	9-B	200	McF_4	Inf	unif	singleC	CBN, CBN-A, OT, OT-A
659	No	9	9-B	200	McF_4	Inf	unif	wholeT_0.01	CBN, CBN-A, OT, OT-A
660	No	9	9-B	200	McF_4	Inf	unif	wholeT_0.5	CBN, CBN-A, OT, OT-A
661	No	9	9-B	200	McF_6	0	last	singleC	OT-A
662	No	9	9-B	200	McF_6	0	last	wholeT_0.01	OT-A
663	No	9	9-B	200	McF_6	0	last	wholeT_0.5	OT-A
664	No	9	9-B	200	McF_6	0	unif	singleC	CBN-A, OT, OT-A
665	No	9	9-B	200	McF_6	0	unif	wholeT_0.01	OT, OT-A
666	No	9	9-B	200	McF_6	0	unif	wholeT_0.5	OT, OT-A
667	No	9	9-B	200	McF_6	Inf	last	singleC	OT-A
668	No	9	9-B	200	McF_6	Inf	last	wholeT_0.01	OT-A
669	No	9	9-B	200	McF_6	Inf	last	wholeT_0.5	OT-A
670	No	9	9-B	200	McF_6	Inf	unif	singleC	OT, OT-A
671	No	9	9-B	200	McF_6	Inf	unif	wholeT_0.01	OT, OT-A
672	No	9	9-B	200	McF_6	Inf	unif	wholeT_0.5	OT, OT-A
673	No	9	9-B	100	Bozic	0	last	singleC	CBN, CBN-A
674	No	9	9-B	100	Bozic	0	last	wholeT_0.01	CBN, CBN-A
675	No	9	9-B	100	Bozic	0	last	wholeT_0.5	CBN, CBN-A
676	No	9	9-B	100	Bozic	0	unif	singleC	CBN, CBN-A
677	No	9	9-B	100	Bozic	0	unif	wholeT_0.01	CBN, CBN-A

Table 3: *(continued)*

	Conjunction	Drivers	Tree	S.Size	Model	sh	S.Time	S.Type	Best method(s)
678	No	9	9-B	100	Bozic	0	unif	wholeT_0.5	CBN, CBN-A
679	No	9	9-B	100	Bozic	Inf	last	singleC	CBN-A, OT, OT-A
680	No	9	9-B	100	Bozic	Inf	last	wholeT_0.01	OT, OT-A
681	No	9	9-B	100	Bozic	Inf	last	wholeT_0.5	CBN, CBN-A, OT, OT-A
682	No	9	9-B	100	Bozic	Inf	unif	singleC	CBN, CBN-A
683	No	9	9-B	100	Bozic	Inf	unif	wholeT_0.01	CBN, CBN-A
684	No	9	9-B	100	Bozic	Inf	unif	wholeT_0.5	CBN, CBN-A
685	No	9	9-B	100	exp	0	last	singleC	CBN, CBN-A
686	No	9	9-B	100	exp	0	last	wholeT_0.01	CBN, CBN-A
687	No	9	9-B	100	exp	0	last	wholeT_0.5	CBN, CBN-A
688	No	9	9-B	100	exp	0	unif	singleC	CBN, CBN-A
689	No	9	9-B	100	exp	0	unif	wholeT_0.01	CBN, CBN-A
690	No	9	9-B	100	exp	0	unif	wholeT_0.5	CBN, CBN-A
691	No	9	9-B	100	exp	Inf	last	singleC	CBN, CBN-A
692	No	9	9-B	100	exp	Inf	last	wholeT_0.01	CBN, CBN-A, OT, OT-A
693	No	9	9-B	100	exp	Inf	last	wholeT_0.5	CBN, CBN-A
694	No	9	9-B	100	exp	Inf	unif	singleC	CBN, CBN-A
695	No	9	9-B	100	exp	Inf	unif	wholeT_0.01	CBN, CBN-A
696	No	9	9-B	100	exp	Inf	unif	wholeT_0.5	CBN, CBN-A
697	No	9	9-B	100	McF_4	0	last	singleC	OT, OT-A
698	No	9	9-B	100	McF_4	0	last	wholeT_0.01	OT, OT-A
699	No	9	9-B	100	McF_4	0	last	wholeT_0.5	OT, OT-A
700	No	9	9-B	100	McF_4	0	unif	singleC	CBN, CBN-A, OT, OT-A
701	No	9	9-B	100	McF_4	0	unif	wholeT_0.01	CBN, CBN-A, OT, OT-A
702	No	9	9-B	100	McF_4	0	unif	wholeT_0.5	CBN, CBN-A
703	No	9	9-B	100	McF_4	Inf	last	singleC	OT, OT-A
704	No	9	9-B	100	McF_4	Inf	last	wholeT_0.01	OT, OT-A
705	No	9	9-B	100	McF_4	Inf	last	wholeT_0.5	OT, OT-A
706	No	9	9-B	100	McF_4	Inf	unif	singleC	CBN, CBN-A, OT, OT-A
707	No	9	9-B	100	McF_4	Inf	unif	wholeT_0.01	CBN, CBN-A, OT, OT-A
708	No	9	9-B	100	McF_4	Inf	unif	wholeT_0.5	CBN, CBN-A
709	No	9	9-B	100	McF_6	0	last	singleC	OT-A
710	No	9	9-B	100	McF_6	0	last	wholeT_0.01	OT-A
711	No	9	9-B	100	McF_6	0	last	wholeT_0.5	OT-A
712	No	9	9-B	100	McF_6	0	unif	singleC	OT, OT-A
713	No	9	9-B	100	McF_6	0	unif	wholeT_0.01	CBN, OT, OT-A
714	No	9	9-B	100	McF_6	0	unif	wholeT_0.5	OT, OT-A
715	No	9	9-B	100	McF_6	Inf	last	singleC	OT-A
716	No	9	9-B	100	McF_6	Inf	last	wholeT_0.01	OT-A
717	No	9	9-B	100	McF_6	Inf	last	wholeT_0.5	OT-A

Table 3: *(continued)*

	Conjunction	Drivers	Tree	S.Size	Model	sh	S.Time	S.Type	Best method(s)
718	No	9	9-B	100	McF_6	Inf	unif	singleC	OT, OT-A
719	No	9	9-B	100	McF_6	Inf	unif	wholeT_0.01	CBN, OT, OT-A
720	No	9	9-B	100	McF_6	Inf	unif	wholeT_0.5	OT, OT-A
721	No	7	7-B	1000	Bozic	0	last	singleC	OT-A
722	No	7	7-B	1000	Bozic	0	last	wholeT_0.01	OT-A
723	No	7	7-B	1000	Bozic	0	last	wholeT_0.5	OT-A
724	No	7	7-B	1000	Bozic	0	unif	singleC	CBN, CBN-A, OT, OT-A
725	No	7	7-B	1000	Bozic	0	unif	wholeT_0.01	CBN, CBN-A, DiP-A, OT, OT-A
726	No	7	7-B	1000	Bozic	0	unif	wholeT_0.5	CBN, CBN-A, OT, OT-A
727	No	7	7-B	1000	Bozic	Inf	last	singleC	DiP-A, OT-A
728	No	7	7-B	1000	Bozic	Inf	last	wholeT_0.01	DiP-A, OT-A
729	No	7	7-B	1000	Bozic	Inf	last	wholeT_0.5	DiP-A, OT-A
730	No	7	7-B	1000	Bozic	Inf	unif	singleC	OT, OT-A
731	No	7	7-B	1000	Bozic	Inf	unif	wholeT_0.01	CBN, CBN-A, OT, OT-A
732	No	7	7-B	1000	Bozic	Inf	unif	wholeT_0.5	OT, OT-A
733	No	7	7-B	1000	exp	0	last	singleC	CBN-A
734	No	7	7-B	1000	exp	0	last	wholeT_0.01	CBN, OT-A
735	No	7	7-B	1000	exp	0	last	wholeT_0.5	CBN-A
736	No	7	7-B	1000	exp	0	unif	singleC	CBN, CBN-A
737	No	7	7-B	1000	exp	0	unif	wholeT_0.01	CBN, CBN-A, OT-A
738	No	7	7-B	1000	exp	0	unif	wholeT_0.5	CBN, CBN-A
739	No	7	7-B	1000	exp	Inf	last	singleC	OT-A
740	No	7	7-B	1000	exp	Inf	last	wholeT_0.01	OT-A
741	No	7	7-B	1000	exp	Inf	last	wholeT_0.5	OT-A
742	No	7	7-B	1000	exp	Inf	unif	singleC	CBN, CBN-A, OT, OT-A
743	No	7	7-B	1000	exp	Inf	unif	wholeT_0.01	CBN-A, OT, OT-A
744	No	7	7-B	1000	exp	Inf	unif	wholeT_0.5	CBN, CBN-A
745	No	7	7-B	1000	McF_4	0	last	singleC	DiP-A, OT-A
746	No	7	7-B	1000	McF_4	0	last	wholeT_0.01	DiP-A, OT-A
747	No	7	7-B	1000	McF_4	0	last	wholeT_0.5	DiP-A, OT-A
748	No	7	7-B	1000	McF_4	0	unif	singleC	DiP-A, OT, OT-A
749	No	7	7-B	1000	McF_4	0	unif	wholeT_0.01	DiP-A, OT, OT-A
750	No	7	7-B	1000	McF_4	0	unif	wholeT_0.5	DiP-A, OT, OT-A
751	No	7	7-B	1000	McF_4	Inf	last	singleC	DiP-A, OT-A
752	No	7	7-B	1000	McF_4	Inf	last	wholeT_0.01	DiP-A, OT-A
753	No	7	7-B	1000	McF_4	Inf	last	wholeT_0.5	DiP-A, OT-A
754	No	7	7-B	1000	McF_4	Inf	unif	singleC	OT, OT-A
755	No	7	7-B	1000	McF_4	Inf	unif	wholeT_0.01	OT, OT-A
756	No	7	7-B	1000	McF_4	Inf	unif	wholeT_0.5	OT, OT-A
757	No	7	7-B	1000	McF_6	0	last	singleC	DiP-A, OT-A

Table 3: *(continued)*

	Conjunction	Drivers	Tree	S.Size	Model	sh	S.Time	S.Type	Best method(s)
758	No	7	7-B	1000	McF_6	0	last	wholeT_0.01	DiP-A, OT-A
759	No	7	7-B	1000	McF_6	0	last	wholeT_0.5	DiP-A, OT-A
760	No	7	7-B	1000	McF_6	0	unif	singleC	DiP, DiP-A, OT, OT-A
761	No	7	7-B	1000	McF_6	0	unif	wholeT_0.01	DiP, DiP-A, OT, OT-A
762	No	7	7-B	1000	McF_6	0	unif	wholeT_0.5	DiP, DiP-A, OT, OT-A
763	No	7	7-B	1000	McF_6	Inf	last	singleC	DiP-A, OT-A
764	No	7	7-B	1000	McF_6	Inf	last	wholeT_0.01	DiP-A, OT-A
765	No	7	7-B	1000	McF_6	Inf	last	wholeT_0.5	DiP-A, OT-A
766	No	7	7-B	1000	McF_6	Inf	unif	singleC	DiP-A, OT, OT-A
767	No	7	7-B	1000	McF_6	Inf	unif	wholeT_0.01	DiP-A, OT, OT-A
768	No	7	7-B	1000	McF_6	Inf	unif	wholeT_0.5	DiP-A, OT, OT-A
769	No	7	7-B	200	Bozic	0	last	singleC	CBN, OT-A
770	No	7	7-B	200	Bozic	0	last	wholeT_0.01	OT-A
771	No	7	7-B	200	Bozic	0	last	wholeT_0.5	CBN, CBN-A, OT-A
772	No	7	7-B	200	Bozic	0	unif	singleC	CBN, CBN-A
773	No	7	7-B	200	Bozic	0	unif	wholeT_0.01	CBN, CBN-A, OT, OT-A
774	No	7	7-B	200	Bozic	0	unif	wholeT_0.5	CBN, CBN-A
775	No	7	7-B	200	Bozic	Inf	last	singleC	OT-A
776	No	7	7-B	200	Bozic	Inf	last	wholeT_0.01	OT-A
777	No	7	7-B	200	Bozic	Inf	last	wholeT_0.5	OT-A
778	No	7	7-B	200	Bozic	Inf	unif	singleC	CBN, CBN-A, OT, OT-A
779	No	7	7-B	200	Bozic	Inf	unif	wholeT_0.01	CBN, CBN-A, OT, OT-A
780	No	7	7-B	200	Bozic	Inf	unif	wholeT_0.5	CBN, CBN-A
781	No	7	7-B	200	exp	0	last	singleC	CBN-A
782	No	7	7-B	200	exp	0	last	wholeT_0.01	CBN, CBN-A, OT-A
783	No	7	7-B	200	exp	0	last	wholeT_0.5	CBN, CBN-A
784	No	7	7-B	200	exp	0	unif	singleC	CBN, CBN-A
785	No	7	7-B	200	exp	0	unif	wholeT_0.01	CBN, CBN-A
786	No	7	7-B	200	exp	0	unif	wholeT_0.5	CBN
787	No	7	7-B	200	exp	Inf	last	singleC	OT-A
788	No	7	7-B	200	exp	Inf	last	wholeT_0.01	OT-A
789	No	7	7-B	200	exp	Inf	last	wholeT_0.5	OT-A
790	No	7	7-B	200	exp	Inf	unif	singleC	CBN, CBN-A
791	No	7	7-B	200	exp	Inf	unif	wholeT_0.01	CBN, CBN-A
792	No	7	7-B	200	exp	Inf	unif	wholeT_0.5	CBN, CBN-A
793	No	7	7-B	200	McF_4	0	last	singleC	OT-A
794	No	7	7-B	200	McF_4	0	last	wholeT_0.01	OT-A
795	No	7	7-B	200	McF_4	0	last	wholeT_0.5	OT-A
796	No	7	7-B	200	McF_4	0	unif	singleC	OT, OT-A
797	No	7	7-B	200	McF_4	0	unif	wholeT_0.01	OT, OT-A

Table 3: *(continued)*

	Conjunction	Drivers	Tree	S.Size	Model	sh	S.Time	S.Type	Best method(s)
798	No	7	7-B	200	McF_4	0	unif	wholeT_0.5	OT, OT-A
799	No	7	7-B	200	McF_4	Inf	last	singleC	OT-A
800	No	7	7-B	200	McF_4	Inf	last	wholeT_0.01	OT-A
801	No	7	7-B	200	McF_4	Inf	last	wholeT_0.5	OT-A
802	No	7	7-B	200	McF_4	Inf	unif	singleC	OT, OT-A
803	No	7	7-B	200	McF_4	Inf	unif	wholeT_0.01	OT, OT-A
804	No	7	7-B	200	McF_4	Inf	unif	wholeT_0.5	OT, OT-A
805	No	7	7-B	200	McF_6	0	last	singleC	OT-A
806	No	7	7-B	200	McF_6	0	last	wholeT_0.01	OT-A
807	No	7	7-B	200	McF_6	0	last	wholeT_0.5	OT-A
808	No	7	7-B	200	McF_6	0	unif	singleC	OT, OT-A
809	No	7	7-B	200	McF_6	0	unif	wholeT_0.01	OT, OT-A
810	No	7	7-B	200	McF_6	0	unif	wholeT_0.5	OT, OT-A
811	No	7	7-B	200	McF_6	Inf	last	singleC	OT-A
812	No	7	7-B	200	McF_6	Inf	last	wholeT_0.01	OT-A
813	No	7	7-B	200	McF_6	Inf	last	wholeT_0.5	OT-A
814	No	7	7-B	200	McF_6	Inf	unif	singleC	OT, OT-A
815	No	7	7-B	200	McF_6	Inf	unif	wholeT_0.01	OT, OT-A
816	No	7	7-B	200	McF_6	Inf	unif	wholeT_0.5	OT, OT-A
817	No	7	7-B	100	Bozic	0	last	singleC	CBN, CBN-A, OT-A
818	No	7	7-B	100	Bozic	0	last	wholeT_0.01	OT-A
819	No	7	7-B	100	Bozic	0	last	wholeT_0.5	CBN, CBN-A, OT-A
820	No	7	7-B	100	Bozic	0	unif	singleC	CBN, CBN-A
821	No	7	7-B	100	Bozic	0	unif	wholeT_0.01	CBN, CBN-A, OT, OT-A
822	No	7	7-B	100	Bozic	0	unif	wholeT_0.5	CBN, CBN-A
823	No	7	7-B	100	Bozic	Inf	last	singleC	OT-A
824	No	7	7-B	100	Bozic	Inf	last	wholeT_0.01	OT-A
825	No	7	7-B	100	Bozic	Inf	last	wholeT_0.5	OT-A
826	No	7	7-B	100	Bozic	Inf	unif	singleC	CBN, CBN-A
827	No	7	7-B	100	Bozic	Inf	unif	wholeT_0.01	CBN, CBN-A
828	No	7	7-B	100	Bozic	Inf	unif	wholeT_0.5	CBN, CBN-A
829	No	7	7-B	100	exp	0	last	singleC	CBN-A
830	No	7	7-B	100	exp	0	last	wholeT_0.01	CBN, CBN-A
831	No	7	7-B	100	exp	0	last	wholeT_0.5	CBN, CBN-A
832	No	7	7-B	100	exp	0	unif	singleC	CBN, CBN-A
833	No	7	7-B	100	exp	0	unif	wholeT_0.01	CBN, CBN-A
834	No	7	7-B	100	exp	0	unif	wholeT_0.5	CBN, CBN-A
835	No	7	7-B	100	exp	Inf	last	singleC	OT-A
836	No	7	7-B	100	exp	Inf	last	wholeT_0.01	OT-A
837	No	7	7-B	100	exp	Inf	last	wholeT_0.5	CBN, OT-A

Table 3: *(continued)*

	Conjunction	Drivers	Tree	S.Size	Model	sh	S.Time	S.Type	Best method(s)
838	No	7	7-B	100	exp	Inf	unif	singleC	CBN, CBN-A
839	No	7	7-B	100	exp	Inf	unif	wholeT_0.01	CBN, CBN-A
840	No	7	7-B	100	exp	Inf	unif	wholeT_0.5	CBN, CBN-A
841	No	7	7-B	100	McF_4	0	last	singleC	OT-A
842	No	7	7-B	100	McF_4	0	last	wholeT_0.01	OT-A
843	No	7	7-B	100	McF_4	0	last	wholeT_0.5	OT-A
844	No	7	7-B	100	McF_4	0	unif	singleC	OT, OT-A
845	No	7	7-B	100	McF_4	0	unif	wholeT_0.01	OT, OT-A
846	No	7	7-B	100	McF_4	0	unif	wholeT_0.5	OT, OT-A
847	No	7	7-B	100	McF_4	Inf	last	singleC	OT-A
848	No	7	7-B	100	McF_4	Inf	last	wholeT_0.01	OT-A
849	No	7	7-B	100	McF_4	Inf	last	wholeT_0.5	OT-A
850	No	7	7-B	100	McF_4	Inf	unif	singleC	OT, OT-A
851	No	7	7-B	100	McF_4	Inf	unif	wholeT_0.01	OT, OT-A
852	No	7	7-B	100	McF_4	Inf	unif	wholeT_0.5	OT, OT-A
853	No	7	7-B	100	McF_6	0	last	singleC	OT-A
854	No	7	7-B	100	McF_6	0	last	wholeT_0.01	OT-A
855	No	7	7-B	100	McF_6	0	last	wholeT_0.5	OT-A
856	No	7	7-B	100	McF_6	0	unif	singleC	OT, OT-A
857	No	7	7-B	100	McF_6	0	unif	wholeT_0.01	OT, OT-A
858	No	7	7-B	100	McF_6	0	unif	wholeT_0.5	OT, OT-A
859	No	7	7-B	100	McF_6	Inf	last	singleC	OT-A
860	No	7	7-B	100	McF_6	Inf	last	wholeT_0.01	OT-A
861	No	7	7-B	100	McF_6	Inf	last	wholeT_0.5	OT-A
862	No	7	7-B	100	McF_6	Inf	unif	singleC	OT-A
863	No	7	7-B	100	McF_6	Inf	unif	wholeT_0.01	OT-A
864	No	7	7-B	100	McF_6	Inf	unif	wholeT_0.5	OT, OT-A

2.4 Confidence sets (MCB-2), FPF, Drivers Known

Table 4: Confidence sets (method MCB-2) when Drivers are Known for measure FPF.

	Conjunction	Drivers	Tree	S.Size	Model	sh	S.Time	S.Type	Best method(s)
1	Yes	11	11-A	1000	Bozic	0	last	singleC	DiP-A, OT, OT-A
2	Yes	11	11-A	1000	Bozic	0	last	wholeT_0.01	DiP, DiP-A
3	Yes	11	11-A	1000	Bozic	0	last	wholeT_0.5	DiP, DiP-A, OT, OT-A
4	Yes	11	11-A	1000	Bozic	0	unif	singleC	DiP, DiP-A
5	Yes	11	11-A	1000	Bozic	0	unif	wholeT_0.01	DiP-A
6	Yes	11	11-A	1000	Bozic	0	unif	wholeT_0.5	DiP, DiP-A
7	Yes	11	11-A	1000	Bozic	Inf	last	singleC	OT, OT-A
8	Yes	11	11-A	1000	Bozic	Inf	last	wholeT_0.01	DiP, DiP-A
9	Yes	11	11-A	1000	Bozic	Inf	last	wholeT_0.5	OT, OT-A
10	Yes	11	11-A	1000	Bozic	Inf	unif	singleC	DiP, DiP-A, OT, OT-A
11	Yes	11	11-A	1000	Bozic	Inf	unif	wholeT_0.01	DiP, DiP-A, OT, OT-A
12	Yes	11	11-A	1000	Bozic	Inf	unif	wholeT_0.5	DiP, DiP-A, OT, OT-A
13	Yes	11	11-A	1000	exp	0	last	singleC	DiP, DiP-A, OT, OT-A
14	Yes	11	11-A	1000	exp	0	last	wholeT_0.01	DiP-A, OT, OT-A
15	Yes	11	11-A	1000	exp	0	last	wholeT_0.5	DiP, DiP-A, OT, OT-A
16	Yes	11	11-A	1000	exp	0	unif	singleC	DiP, DiP-A, OT, OT-A
17	Yes	11	11-A	1000	exp	0	unif	wholeT_0.01	DiP, DiP-A
18	Yes	11	11-A	1000	exp	0	unif	wholeT_0.5	DiP, DiP-A, OT, OT-A
19	Yes	11	11-A	1000	exp	Inf	last	singleC	DiP-A, OT, OT-A
20	Yes	11	11-A	1000	exp	Inf	last	wholeT_0.01	OT, OT-A
21	Yes	11	11-A	1000	exp	Inf	last	wholeT_0.5	DiP, DiP-A, OT, OT-A
22	Yes	11	11-A	1000	exp	Inf	unif	singleC	DiP, DiP-A, OT, OT-A
23	Yes	11	11-A	1000	exp	Inf	unif	wholeT_0.01	DiP, DiP-A, OT, OT-A
24	Yes	11	11-A	1000	exp	Inf	unif	wholeT_0.5	DiP-A, OT, OT-A
25	Yes	11	11-A	1000	McF_4	0	last	singleC	DiP-A, OT, OT-A
26	Yes	11	11-A	1000	McF_4	0	last	wholeT_0.01	DiP-A, OT, OT-A
27	Yes	11	11-A	1000	McF_4	0	last	wholeT_0.5	DiP-A, OT, OT-A
28	Yes	11	11-A	1000	McF_4	0	unif	singleC	DiP, DiP-A, OT, OT-A
29	Yes	11	11-A	1000	McF_4	0	unif	wholeT_0.01	DiP, DiP-A
30	Yes	11	11-A	1000	McF_4	0	unif	wholeT_0.5	DiP, DiP-A
31	Yes	11	11-A	1000	McF_4	Inf	last	singleC	OT, OT-A
32	Yes	11	11-A	1000	McF_4	Inf	last	wholeT_0.01	OT, OT-A
33	Yes	11	11-A	1000	McF_4	Inf	last	wholeT_0.5	DiP-A, OT, OT-A
34	Yes	11	11-A	1000	McF_4	Inf	unif	singleC	DiP, DiP-A, OT, OT-A
35	Yes	11	11-A	1000	McF_4	Inf	unif	wholeT_0.01	DiP, DiP-A, OT, OT-A
36	Yes	11	11-A	1000	McF_4	Inf	unif	wholeT_0.5	DiP, DiP-A, OT, OT-A
37	Yes	11	11-A	1000	McF_6	0	last	singleC	DiP, OT

Table 4: *(continued)*

	Conjunction	Drivers	Tree	S.Size	Model	sh	S.Time	S.Type	Best method(s)
38	Yes	11	11-A	1000	McF_6	0	last	wholeT_0.01	DiP, OT
39	Yes	11	11-A	1000	McF_6	0	last	wholeT_0.5	DiP, OT
40	Yes	11	11-A	1000	McF_6	0	unif	singleC	DiP, DiP-A, OT, OT-A
41	Yes	11	11-A	1000	McF_6	0	unif	wholeT_0.01	DiP, DiP-A, OT, OT-A
42	Yes	11	11-A	1000	McF_6	0	unif	wholeT_0.5	DiP, DiP-A
43	Yes	11	11-A	1000	McF_6	Inf	last	singleC	DiP, OT
44	Yes	11	11-A	1000	McF_6	Inf	last	wholeT_0.01	DiP, OT
45	Yes	11	11-A	1000	McF_6	Inf	last	wholeT_0.5	DiP, OT
46	Yes	11	11-A	1000	McF_6	Inf	unif	singleC	DiP, DiP-A
47	Yes	11	11-A	1000	McF_6	Inf	unif	wholeT_0.01	DiP, DiP-A
48	Yes	11	11-A	1000	McF_6	Inf	unif	wholeT_0.5	DiP, DiP-A
49	Yes	11	11-A	200	Bozic	0	last	singleC	DiP, DiP-A, OT, OT-A
50	Yes	11	11-A	200	Bozic	0	last	wholeT_0.01	DiP
51	Yes	11	11-A	200	Bozic	0	last	wholeT_0.5	DiP, DiP-A, OT, OT-A
52	Yes	11	11-A	200	Bozic	0	unif	singleC	DiP, DiP-A, OT, OT-A
53	Yes	11	11-A	200	Bozic	0	unif	wholeT_0.01	DiP, DiP-A
54	Yes	11	11-A	200	Bozic	0	unif	wholeT_0.5	DiP, DiP-A, OT, OT-A
55	Yes	11	11-A	200	Bozic	Inf	last	singleC	DiP, DiP-A, OT, OT-A
56	Yes	11	11-A	200	Bozic	Inf	last	wholeT_0.01	DiP, OT, OT-A
57	Yes	11	11-A	200	Bozic	Inf	last	wholeT_0.5	DiP, DiP-A, OT, OT-A
58	Yes	11	11-A	200	Bozic	Inf	unif	singleC	DiP, DiP-A
59	Yes	11	11-A	200	Bozic	Inf	unif	wholeT_0.01	DiP, DiP-A, OT, OT-A
60	Yes	11	11-A	200	Bozic	Inf	unif	wholeT_0.5	DiP, DiP-A
61	Yes	11	11-A	200	exp	0	last	singleC	DiP, DiP-A, OT, OT-A
62	Yes	11	11-A	200	exp	0	last	wholeT_0.01	DiP, DiP-A, OT, OT-A
63	Yes	11	11-A	200	exp	0	last	wholeT_0.5	DiP, DiP-A, OT, OT-A
64	Yes	11	11-A	200	exp	0	unif	singleC	DiP, DiP-A, OT, OT-A
65	Yes	11	11-A	200	exp	0	unif	wholeT_0.01	DiP, DiP-A, OT, OT-A
66	Yes	11	11-A	200	exp	0	unif	wholeT_0.5	DiP, DiP-A, OT, OT-A
67	Yes	11	11-A	200	exp	Inf	last	singleC	DiP, DiP-A, OT, OT-A
68	Yes	11	11-A	200	exp	Inf	last	wholeT_0.01	DiP, DiP-A, OT, OT-A
69	Yes	11	11-A	200	exp	Inf	last	wholeT_0.5	DiP, DiP-A, OT, OT-A
70	Yes	11	11-A	200	exp	Inf	unif	singleC	DiP, DiP-A, OT, OT-A
71	Yes	11	11-A	200	exp	Inf	unif	wholeT_0.01	DiP, DiP-A
72	Yes	11	11-A	200	exp	Inf	unif	wholeT_0.5	DiP, DiP-A
73	Yes	11	11-A	200	McF_4	0	last	singleC	DiP, DiP-A, OT, OT-A
74	Yes	11	11-A	200	McF_4	0	last	wholeT_0.01	DiP, DiP-A, OT, OT-A
75	Yes	11	11-A	200	McF_4	0	last	wholeT_0.5	DiP, DiP-A, OT, OT-A
76	Yes	11	11-A	200	McF_4	0	unif	singleC	DiP, DiP-A, OT, OT-A
77	Yes	11	11-A	200	McF_4	0	unif	wholeT_0.01	DiP, DiP-A

Table 4: *(continued)*

	Conjunction	Drivers	Tree	S.Size	Model	sh	S.Time	S.Type	Best method(s)
78	Yes	11	11-A	200	McF_4	0	unif	wholeT_0.5	DiP, DiP-A, OT, OT-A
79	Yes	11	11-A	200	McF_4	Inf	last	singleC	DiP, DiP-A, OT, OT-A
80	Yes	11	11-A	200	McF_4	Inf	last	wholeT_0.01	OT, OT-A
81	Yes	11	11-A	200	McF_4	Inf	last	wholeT_0.5	DiP, DiP-A, OT, OT-A
82	Yes	11	11-A	200	McF_4	Inf	unif	singleC	DiP, DiP-A, OT, OT-A
83	Yes	11	11-A	200	McF_4	Inf	unif	wholeT_0.01	DiP, DiP-A, OT, OT-A
84	Yes	11	11-A	200	McF_4	Inf	unif	wholeT_0.5	DiP, DiP-A, OT, OT-A
85	Yes	11	11-A	200	McF_6	0	last	singleC	DiP, OT
86	Yes	11	11-A	200	McF_6	0	last	wholeT_0.01	DiP, OT
87	Yes	11	11-A	200	McF_6	0	last	wholeT_0.5	DiP, OT
88	Yes	11	11-A	200	McF_6	0	unif	singleC	DiP, DiP-A, OT, OT-A
89	Yes	11	11-A	200	McF_6	0	unif	wholeT_0.01	DiP, DiP-A, OT, OT-A
90	Yes	11	11-A	200	McF_6	0	unif	wholeT_0.5	DiP, OT, OT-A
91	Yes	11	11-A	200	McF_6	Inf	last	singleC	DiP, OT
92	Yes	11	11-A	200	McF_6	Inf	last	wholeT_0.01	DiP, OT
93	Yes	11	11-A	200	McF_6	Inf	last	wholeT_0.5	DiP, OT
94	Yes	11	11-A	200	McF_6	Inf	unif	singleC	OT, OT-A
95	Yes	11	11-A	200	McF_6	Inf	unif	wholeT_0.01	OT, OT-A
96	Yes	11	11-A	200	McF_6	Inf	unif	wholeT_0.5	OT, OT-A
97	Yes	11	11-A	100	Bozic	0	last	singleC	DiP, DiP-A, OT, OT-A
98	Yes	11	11-A	100	Bozic	0	last	wholeT_0.01	DiP, DiP-A
99	Yes	11	11-A	100	Bozic	0	last	wholeT_0.5	DiP, DiP-A, OT, OT-A
100	Yes	11	11-A	100	Bozic	0	unif	singleC	DiP, DiP-A, OT, OT-A
101	Yes	11	11-A	100	Bozic	0	unif	wholeT_0.01	DiP, DiP-A, OT, OT-A
102	Yes	11	11-A	100	Bozic	0	unif	wholeT_0.5	DiP, DiP-A, OT, OT-A
103	Yes	11	11-A	100	Bozic	Inf	last	singleC	DiP, DiP-A, OT, OT-A
104	Yes	11	11-A	100	Bozic	Inf	last	wholeT_0.01	DiP, DiP-A
105	Yes	11	11-A	100	Bozic	Inf	last	wholeT_0.5	DiP, DiP-A, OT, OT-A
106	Yes	11	11-A	100	Bozic	Inf	unif	singleC	DiP, DiP-A
107	Yes	11	11-A	100	Bozic	Inf	unif	wholeT_0.01	DiP, DiP-A
108	Yes	11	11-A	100	Bozic	Inf	unif	wholeT_0.5	DiP, DiP-A
109	Yes	11	11-A	100	exp	0	last	singleC	DiP, DiP-A, OT, OT-A
110	Yes	11	11-A	100	exp	0	last	wholeT_0.01	DiP, DiP-A, OT, OT-A
111	Yes	11	11-A	100	exp	0	last	wholeT_0.5	DiP, DiP-A, OT, OT-A
112	Yes	11	11-A	100	exp	0	unif	singleC	DiP, DiP-A, OT, OT-A
113	Yes	11	11-A	100	exp	0	unif	wholeT_0.01	DiP, DiP-A
114	Yes	11	11-A	100	exp	0	unif	wholeT_0.5	DiP, DiP-A, OT, OT-A
115	Yes	11	11-A	100	exp	Inf	last	singleC	DiP, DiP-A, OT, OT-A
116	Yes	11	11-A	100	exp	Inf	last	wholeT_0.01	DiP, DiP-A, OT, OT-A
117	Yes	11	11-A	100	exp	Inf	last	wholeT_0.5	DiP, DiP-A, OT, OT-A

Table 4: *(continued)*

	Conjunction	Drivers	Tree	S.Size	Model	sh	S.Time	S.Type	Best method(s)
118	Yes	11	11-A	100	exp	Inf	unif	singleC	DiP, DiP-A, OT, OT-A
119	Yes	11	11-A	100	exp	Inf	unif	wholeT_0.01	DiP, DiP-A
120	Yes	11	11-A	100	exp	Inf	unif	wholeT_0.5	DiP, DiP-A, OT, OT-A
121	Yes	11	11-A	100	McF_4	0	last	singleC	DiP, DiP-A, OT, OT-A
122	Yes	11	11-A	100	McF_4	0	last	wholeT_0.01	DiP, DiP-A
123	Yes	11	11-A	100	McF_4	0	last	wholeT_0.5	DiP, DiP-A
124	Yes	11	11-A	100	McF_4	0	unif	singleC	DiP, DiP-A, OT, OT-A
125	Yes	11	11-A	100	McF_4	0	unif	wholeT_0.01	DiP, DiP-A
126	Yes	11	11-A	100	McF_4	0	unif	wholeT_0.5	DiP, DiP-A, OT, OT-A
127	Yes	11	11-A	100	McF_4	Inf	last	singleC	DiP, DiP-A, OT, OT-A
128	Yes	11	11-A	100	McF_4	Inf	last	wholeT_0.01	DiP, DiP-A, OT
129	Yes	11	11-A	100	McF_4	Inf	last	wholeT_0.5	DiP, DiP-A, OT, OT-A
130	Yes	11	11-A	100	McF_4	Inf	unif	singleC	DiP, DiP-A, OT, OT-A
131	Yes	11	11-A	100	McF_4	Inf	unif	wholeT_0.01	DiP, DiP-A
132	Yes	11	11-A	100	McF_4	Inf	unif	wholeT_0.5	DiP, DiP-A, OT, OT-A
133	Yes	11	11-A	100	McF_6	0	last	singleC	DiP, OT
134	Yes	11	11-A	100	McF_6	0	last	wholeT_0.01	DiP, OT
135	Yes	11	11-A	100	McF_6	0	last	wholeT_0.5	DiP, OT
136	Yes	11	11-A	100	McF_6	0	unif	singleC	DiP, DiP-A, OT, OT-A
137	Yes	11	11-A	100	McF_6	0	unif	wholeT_0.01	DiP, DiP-A
138	Yes	11	11-A	100	McF_6	0	unif	wholeT_0.5	DiP, DiP-A, OT, OT-A
139	Yes	11	11-A	100	McF_6	Inf	last	singleC	DiP, OT
140	Yes	11	11-A	100	McF_6	Inf	last	wholeT_0.01	DiP
141	Yes	11	11-A	100	McF_6	Inf	last	wholeT_0.5	DiP, OT
142	Yes	11	11-A	100	McF_6	Inf	unif	singleC	DiP, DiP-A, OT, OT-A
143	Yes	11	11-A	100	McF_6	Inf	unif	wholeT_0.01	DiP, OT, OT-A
144	Yes	11	11-A	100	McF_6	Inf	unif	wholeT_0.5	DiP, DiP-A, OT, OT-A
145	Yes	9	9-A	1000	Bozic	0	last	singleC	DiP, DiP-A, OT, OT-A
146	Yes	9	9-A	1000	Bozic	0	last	wholeT_0.01	DiP, DiP-A
147	Yes	9	9-A	1000	Bozic	0	last	wholeT_0.5	DiP, DiP-A, OT, OT-A
148	Yes	9	9-A	1000	Bozic	0	unif	singleC	DiP, DiP-A, OT, OT-A
149	Yes	9	9-A	1000	Bozic	0	unif	wholeT_0.01	DiP, DiP-A
150	Yes	9	9-A	1000	Bozic	0	unif	wholeT_0.5	DiP, DiP-A, OT, OT-A
151	Yes	9	9-A	1000	Bozic	Inf	last	singleC	OT, OT-A
152	Yes	9	9-A	1000	Bozic	Inf	last	wholeT_0.01	DiP, DiP-A, OT, OT-A
153	Yes	9	9-A	1000	Bozic	Inf	last	wholeT_0.5	OT, OT-A
154	Yes	9	9-A	1000	Bozic	Inf	unif	singleC	DiP, DiP-A
155	Yes	9	9-A	1000	Bozic	Inf	unif	wholeT_0.01	DiP, DiP-A
156	Yes	9	9-A	1000	Bozic	Inf	unif	wholeT_0.5	DiP, DiP-A, OT, OT-A
157	Yes	9	9-A	1000	exp	0	last	singleC	DiP, DiP-A, OT, OT-A

Table 4: *(continued)*

	Conjunction	Drivers	Tree	S.Size	Model	sh	S.Time	S.Type	Best method(s)
158	Yes	9	9-A	1000	exp	0	last	wholeT_0.01	DiP-A, OT, OT-A
159	Yes	9	9-A	1000	exp	0	last	wholeT_0.5	DiP, DiP-A, OT, OT-A
160	Yes	9	9-A	1000	exp	0	unif	singleC	DiP, DiP-A, OT, OT-A
161	Yes	9	9-A	1000	exp	0	unif	wholeT_0.01	DiP, DiP-A, OT, OT-A
162	Yes	9	9-A	1000	exp	0	unif	wholeT_0.5	DiP, DiP-A, OT, OT-A
163	Yes	9	9-A	1000	exp	Inf	last	singleC	OT, OT-A
164	Yes	9	9-A	1000	exp	Inf	last	wholeT_0.01	DiP, DiP-A, OT, OT-A
165	Yes	9	9-A	1000	exp	Inf	last	wholeT_0.5	OT, OT-A
166	Yes	9	9-A	1000	exp	Inf	unif	singleC	DiP, DiP-A, OT, OT-A
167	Yes	9	9-A	1000	exp	Inf	unif	wholeT_0.01	OT, OT-A
168	Yes	9	9-A	1000	exp	Inf	unif	wholeT_0.5	DiP, DiP-A, OT, OT-A
169	Yes	9	9-A	1000	McF_4	0	last	singleC	DiP, DiP-A, OT, OT-A
170	Yes	9	9-A	1000	McF_4	0	last	wholeT_0.01	DiP, DiP-A, OT, OT-A
171	Yes	9	9-A	1000	McF_4	0	last	wholeT_0.5	DiP, DiP-A, OT, OT-A
172	Yes	9	9-A	1000	McF_4	0	unif	singleC	DiP, DiP-A, OT, OT-A
173	Yes	9	9-A	1000	McF_4	0	unif	wholeT_0.01	DiP, DiP-A
174	Yes	9	9-A	1000	McF_4	0	unif	wholeT_0.5	DiP, DiP-A, OT, OT-A
175	Yes	9	9-A	1000	McF_4	Inf	last	singleC	OT, OT-A
176	Yes	9	9-A	1000	McF_4	Inf	last	wholeT_0.01	DiP, OT
177	Yes	9	9-A	1000	McF_4	Inf	last	wholeT_0.5	DiP, DiP-A, OT, OT-A
178	Yes	9	9-A	1000	McF_4	Inf	unif	singleC	DiP, DiP-A, OT, OT-A
179	Yes	9	9-A	1000	McF_4	Inf	unif	wholeT_0.01	OT, OT-A
180	Yes	9	9-A	1000	McF_4	Inf	unif	wholeT_0.5	DiP, DiP-A, OT, OT-A
181	Yes	9	9-A	1000	McF_6	0	last	singleC	OT
182	Yes	9	9-A	1000	McF_6	0	last	wholeT_0.01	DiP, OT
183	Yes	9	9-A	1000	McF_6	0	last	wholeT_0.5	DiP, OT
184	Yes	9	9-A	1000	McF_6	0	unif	singleC	DiP, DiP-A, OT, OT-A
185	Yes	9	9-A	1000	McF_6	0	unif	wholeT_0.01	DiP, DiP-A, OT, OT-A
186	Yes	9	9-A	1000	McF_6	0	unif	wholeT_0.5	DiP, DiP-A, OT, OT-A
187	Yes	9	9-A	1000	McF_6	Inf	last	singleC	DiP, OT
188	Yes	9	9-A	1000	McF_6	Inf	last	wholeT_0.01	OT
189	Yes	9	9-A	1000	McF_6	Inf	last	wholeT_0.5	DiP, OT
190	Yes	9	9-A	1000	McF_6	Inf	unif	singleC	DiP, DiP-A, OT, OT-A
191	Yes	9	9-A	1000	McF_6	Inf	unif	wholeT_0.01	DiP, DiP-A, OT, OT-A
192	Yes	9	9-A	1000	McF_6	Inf	unif	wholeT_0.5	DiP, DiP-A, OT, OT-A
193	Yes	9	9-A	200	Bozic	0	last	singleC	DiP, DiP-A, OT, OT-A
194	Yes	9	9-A	200	Bozic	0	last	wholeT_0.01	DiP, DiP-A
195	Yes	9	9-A	200	Bozic	0	last	wholeT_0.5	DiP, DiP-A, OT, OT-A
196	Yes	9	9-A	200	Bozic	0	unif	singleC	DiP, DiP-A, OT, OT-A
197	Yes	9	9-A	200	Bozic	0	unif	wholeT_0.01	DiP, DiP-A, OT, OT-A

Table 4: *(continued)*

	Conjunction	Drivers	Tree	S.Size	Model	sh	S.Time	S.Type	Best method(s)
198	Yes	9	9-A	200	Bozic	0	unif	wholeT_0.5	DiP, DiP-A, OT, OT-A
199	Yes	9	9-A	200	Bozic	Inf	last	singleC	OT, OT-A
200	Yes	9	9-A	200	Bozic	Inf	last	wholeT_0.01	DiP, DiP-A
201	Yes	9	9-A	200	Bozic	Inf	last	wholeT_0.5	OT, OT-A
202	Yes	9	9-A	200	Bozic	Inf	unif	singleC	DiP, DiP-A, OT, OT-A
203	Yes	9	9-A	200	Bozic	Inf	unif	wholeT_0.01	DiP, DiP-A, OT, OT-A
204	Yes	9	9-A	200	Bozic	Inf	unif	wholeT_0.5	DiP, DiP-A, OT, OT-A
205	Yes	9	9-A	200	exp	0	last	singleC	DiP, DiP-A, OT, OT-A
206	Yes	9	9-A	200	exp	0	last	wholeT_0.01	DiP, DiP-A, OT, OT-A
207	Yes	9	9-A	200	exp	0	last	wholeT_0.5	DiP, DiP-A, OT, OT-A
208	Yes	9	9-A	200	exp	0	unif	singleC	DiP, DiP-A, OT, OT-A
209	Yes	9	9-A	200	exp	0	unif	wholeT_0.01	DiP, DiP-A, OT, OT-A
210	Yes	9	9-A	200	exp	0	unif	wholeT_0.5	DiP, DiP-A, OT, OT-A
211	Yes	9	9-A	200	exp	Inf	last	singleC	DiP, DiP-A, OT, OT-A
212	Yes	9	9-A	200	exp	Inf	last	wholeT_0.01	DiP, DiP-A
213	Yes	9	9-A	200	exp	Inf	last	wholeT_0.5	DiP, DiP-A, OT, OT-A
214	Yes	9	9-A	200	exp	Inf	unif	singleC	DiP, DiP-A, OT, OT-A
215	Yes	9	9-A	200	exp	Inf	unif	wholeT_0.01	DiP, DiP-A
216	Yes	9	9-A	200	exp	Inf	unif	wholeT_0.5	DiP, DiP-A, OT, OT-A
217	Yes	9	9-A	200	McF_4	0	last	singleC	DiP, DiP-A, OT, OT-A
218	Yes	9	9-A	200	McF_4	0	last	wholeT_0.01	DiP, DiP-A
219	Yes	9	9-A	200	McF_4	0	last	wholeT_0.5	DiP, DiP-A, OT, OT-A
220	Yes	9	9-A	200	McF_4	0	unif	singleC	DiP, DiP-A, OT, OT-A
221	Yes	9	9-A	200	McF_4	0	unif	wholeT_0.01	DiP, DiP-A
222	Yes	9	9-A	200	McF_4	0	unif	wholeT_0.5	DiP, DiP-A, OT, OT-A
223	Yes	9	9-A	200	McF_4	Inf	last	singleC	OT, OT-A
224	Yes	9	9-A	200	McF_4	Inf	last	wholeT_0.01	DiP, OT
225	Yes	9	9-A	200	McF_4	Inf	last	wholeT_0.5	DiP, DiP-A, OT, OT-A
226	Yes	9	9-A	200	McF_4	Inf	unif	singleC	DiP, DiP-A, OT, OT-A
227	Yes	9	9-A	200	McF_4	Inf	unif	wholeT_0.01	DiP, DiP-A, OT, OT-A
228	Yes	9	9-A	200	McF_4	Inf	unif	wholeT_0.5	DiP, DiP-A, OT, OT-A
229	Yes	9	9-A	200	McF_6	0	last	singleC	DiP, OT
230	Yes	9	9-A	200	McF_6	0	last	wholeT_0.01	DiP, OT
231	Yes	9	9-A	200	McF_6	0	last	wholeT_0.5	DiP, OT
232	Yes	9	9-A	200	McF_6	0	unif	singleC	DiP, DiP-A, OT, OT-A
233	Yes	9	9-A	200	McF_6	0	unif	wholeT_0.01	DiP, DiP-A, OT, OT-A
234	Yes	9	9-A	200	McF_6	0	unif	wholeT_0.5	DiP, DiP-A, OT, OT-A
235	Yes	9	9-A	200	McF_6	Inf	last	singleC	DiP, OT
236	Yes	9	9-A	200	McF_6	Inf	last	wholeT_0.01	OT
237	Yes	9	9-A	200	McF_6	Inf	last	wholeT_0.5	DiP, OT

Table 4: *(continued)*

	Conjunction	Drivers	Tree	S.Size	Model	sh	S.Time	S.Type	Best method(s)
238	Yes	9	9-A	200	McF_6	Inf	unif	singleC	DiP, DiP-A, OT, OT-A
239	Yes	9	9-A	200	McF_6	Inf	unif	wholeT_0.01	DiP, DiP-A, OT, OT-A
240	Yes	9	9-A	200	McF_6	Inf	unif	wholeT_0.5	DiP, DiP-A, OT, OT-A
241	Yes	9	9-A	100	Bozic	0	last	singleC	DiP, DiP-A, OT, OT-A
242	Yes	9	9-A	100	Bozic	0	last	wholeT_0.01	DiP, DiP-A, OT, OT-A
243	Yes	9	9-A	100	Bozic	0	last	wholeT_0.5	DiP, DiP-A, OT, OT-A
244	Yes	9	9-A	100	Bozic	0	unif	singleC	DiP, DiP-A, OT, OT-A
245	Yes	9	9-A	100	Bozic	0	unif	wholeT_0.01	DiP, DiP-A
246	Yes	9	9-A	100	Bozic	0	unif	wholeT_0.5	DiP, DiP-A, OT, OT-A
247	Yes	9	9-A	100	Bozic	Inf	last	singleC	DiP, DiP-A, OT, OT-A
248	Yes	9	9-A	100	Bozic	Inf	last	wholeT_0.01	DiP, DiP-A
249	Yes	9	9-A	100	Bozic	Inf	last	wholeT_0.5	DiP, DiP-A, OT, OT-A
250	Yes	9	9-A	100	Bozic	Inf	unif	singleC	DiP, DiP-A, OT, OT-A
251	Yes	9	9-A	100	Bozic	Inf	unif	wholeT_0.01	DiP, DiP-A, OT, OT-A
252	Yes	9	9-A	100	Bozic	Inf	unif	wholeT_0.5	DiP, DiP-A, OT, OT-A
253	Yes	9	9-A	100	exp	0	last	singleC	DiP, DiP-A, OT, OT-A
254	Yes	9	9-A	100	exp	0	last	wholeT_0.01	DiP, DiP-A, OT, OT-A
255	Yes	9	9-A	100	exp	0	last	wholeT_0.5	DiP, DiP-A, OT, OT-A
256	Yes	9	9-A	100	exp	0	unif	singleC	DiP, DiP-A, OT, OT-A
257	Yes	9	9-A	100	exp	0	unif	wholeT_0.01	DiP, DiP-A, OT, OT-A
258	Yes	9	9-A	100	exp	0	unif	wholeT_0.5	DiP, DiP-A, OT, OT-A
259	Yes	9	9-A	100	exp	Inf	last	singleC	DiP, DiP-A, OT, OT-A
260	Yes	9	9-A	100	exp	Inf	last	wholeT_0.01	DiP, DiP-A
261	Yes	9	9-A	100	exp	Inf	last	wholeT_0.5	DiP, DiP-A, OT, OT-A
262	Yes	9	9-A	100	exp	Inf	unif	singleC	DiP, DiP-A, OT, OT-A
263	Yes	9	9-A	100	exp	Inf	unif	wholeT_0.01	DiP, DiP-A
264	Yes	9	9-A	100	exp	Inf	unif	wholeT_0.5	DiP, DiP-A, OT, OT-A
265	Yes	9	9-A	100	McF_4	0	last	singleC	DiP, DiP-A, OT, OT-A
266	Yes	9	9-A	100	McF_4	0	last	wholeT_0.01	DiP, DiP-A
267	Yes	9	9-A	100	McF_4	0	last	wholeT_0.5	DiP, DiP-A, OT, OT-A
268	Yes	9	9-A	100	McF_4	0	unif	singleC	DiP, DiP-A, OT, OT-A
269	Yes	9	9-A	100	McF_4	0	unif	wholeT_0.01	DiP, DiP-A
270	Yes	9	9-A	100	McF_4	0	unif	wholeT_0.5	DiP, DiP-A, OT, OT-A
271	Yes	9	9-A	100	McF_4	Inf	last	singleC	DiP, DiP-A, OT, OT-A
272	Yes	9	9-A	100	McF_4	Inf	last	wholeT_0.01	DiP, OT
273	Yes	9	9-A	100	McF_4	Inf	last	wholeT_0.5	DiP, DiP-A, OT, OT-A
274	Yes	9	9-A	100	McF_4	Inf	unif	singleC	DiP, DiP-A, OT, OT-A
275	Yes	9	9-A	100	McF_4	Inf	unif	wholeT_0.01	DiP, DiP-A, OT, OT-A
276	Yes	9	9-A	100	McF_4	Inf	unif	wholeT_0.5	DiP, DiP-A, OT, OT-A
277	Yes	9	9-A	100	McF_6	0	last	singleC	DiP, OT

Table 4: *(continued)*

	Conjunction	Drivers	Tree	S.Size	Model	sh	S.Time	S.Type	Best method(s)
278	Yes	9	9-A	100	McF_6	0	last	wholeT_0.01	DiP, OT
279	Yes	9	9-A	100	McF_6	0	last	wholeT_0.5	DiP, OT
280	Yes	9	9-A	100	McF_6	0	unif	singleC	DiP, DiP-A, OT
281	Yes	9	9-A	100	McF_6	0	unif	wholeT_0.01	DiP, DiP-A, OT, OT-A
282	Yes	9	9-A	100	McF_6	0	unif	wholeT_0.5	DiP, DiP-A, OT, OT-A
283	Yes	9	9-A	100	McF_6	Inf	last	singleC	DiP, OT
284	Yes	9	9-A	100	McF_6	Inf	last	wholeT_0.01	DiP, OT
285	Yes	9	9-A	100	McF_6	Inf	last	wholeT_0.5	DiP, OT
286	Yes	9	9-A	100	McF_6	Inf	unif	singleC	DiP, DiP-A, OT, OT-A
287	Yes	9	9-A	100	McF_6	Inf	unif	wholeT_0.01	DiP, OT, OT-A
288	Yes	9	9-A	100	McF_6	Inf	unif	wholeT_0.5	DiP, DiP-A, OT, OT-A
289	Yes	7	7-A	1000	Bozic	0	last	singleC	DiP, DiP-A, OT, OT-A
290	Yes	7	7-A	1000	Bozic	0	last	wholeT_0.01	DiP, DiP-A
291	Yes	7	7-A	1000	Bozic	0	last	wholeT_0.5	DiP, DiP-A, OT, OT-A
292	Yes	7	7-A	1000	Bozic	0	unif	singleC	DiP, DiP-A, OT, OT-A
293	Yes	7	7-A	1000	Bozic	0	unif	wholeT_0.01	CBN, CBN-A, DiP, DiP-A, OT, OT-A
294	Yes	7	7-A	1000	Bozic	0	unif	wholeT_0.5	DiP, DiP-A, OT, OT-A
295	Yes	7	7-A	1000	Bozic	Inf	last	singleC	DiP, DiP-A, OT, OT-A
296	Yes	7	7-A	1000	Bozic	Inf	last	wholeT_0.01	DiP, DiP-A, OT, OT-A
297	Yes	7	7-A	1000	Bozic	Inf	last	wholeT_0.5	DiP, DiP-A, OT, OT-A
298	Yes	7	7-A	1000	Bozic	Inf	unif	singleC	CBN, CBN-A
299	Yes	7	7-A	1000	Bozic	Inf	unif	wholeT_0.01	DiP, DiP-A
300	Yes	7	7-A	1000	Bozic	Inf	unif	wholeT_0.5	CBN, CBN-A
301	Yes	7	7-A	1000	exp	0	last	singleC	DiP, DiP-A, OT, OT-A
302	Yes	7	7-A	1000	exp	0	last	wholeT_0.01	DiP, DiP-A, OT, OT-A
303	Yes	7	7-A	1000	exp	0	last	wholeT_0.5	DiP, DiP-A, OT, OT-A
304	Yes	7	7-A	1000	exp	0	unif	singleC	DiP, DiP-A, OT, OT-A
305	Yes	7	7-A	1000	exp	0	unif	wholeT_0.01	DiP, DiP-A
306	Yes	7	7-A	1000	exp	0	unif	wholeT_0.5	DiP, DiP-A, OT, OT-A
307	Yes	7	7-A	1000	exp	Inf	last	singleC	DiP, DiP-A, OT, OT-A
308	Yes	7	7-A	1000	exp	Inf	last	wholeT_0.01	DiP, DiP-A, OT, OT-A
309	Yes	7	7-A	1000	exp	Inf	last	wholeT_0.5	DiP, DiP-A, OT, OT-A
310	Yes	7	7-A	1000	exp	Inf	unif	singleC	DiP, DiP-A
311	Yes	7	7-A	1000	exp	Inf	unif	wholeT_0.01	DiP, DiP-A, OT, OT-A
312	Yes	7	7-A	1000	exp	Inf	unif	wholeT_0.5	DiP, DiP-A
313	Yes	7	7-A	1000	McF_4	0	last	singleC	DiP, DiP-A, OT, OT-A
314	Yes	7	7-A	1000	McF_4	0	last	wholeT_0.01	OT, OT-A
315	Yes	7	7-A	1000	McF_4	0	last	wholeT_0.5	DiP, DiP-A, OT, OT-A
316	Yes	7	7-A	1000	McF_4	0	unif	singleC	DiP, DiP-A
317	Yes	7	7-A	1000	McF_4	0	unif	wholeT_0.01	DiP, DiP-A, OT

Table 4: *(continued)*

	Conjunction	Drivers	Tree	S.Size	Model	sh	S.Time	S.Type	Best method(s)
318	Yes	7	7-A	1000	McF_4	0	unif	wholeT_0.5	DiP, DiP-A
319	Yes	7	7-A	1000	McF_4	Inf	last	singleC	DiP, DiP-A, OT, OT-A
320	Yes	7	7-A	1000	McF_4	Inf	last	wholeT_0.01	OT, OT-A
321	Yes	7	7-A	1000	McF_4	Inf	last	wholeT_0.5	DiP, DiP-A, OT, OT-A
322	Yes	7	7-A	1000	McF_4	Inf	unif	singleC	DiP, DiP-A, OT, OT-A
323	Yes	7	7-A	1000	McF_4	Inf	unif	wholeT_0.01	DiP, DiP-A, OT, OT-A
324	Yes	7	7-A	1000	McF_4	Inf	unif	wholeT_0.5	DiP, DiP-A, OT, OT-A
325	Yes	7	7-A	1000	McF_6	0	last	singleC	DiP, OT, OT-A
326	Yes	7	7-A	1000	McF_6	0	last	wholeT_0.01	DiP, OT, OT-A
327	Yes	7	7-A	1000	McF_6	0	last	wholeT_0.5	DiP, OT, OT-A
328	Yes	7	7-A	1000	McF_6	0	unif	singleC	CBN, CBN-A, DiP, DiP-A, OT, OT-A
329	Yes	7	7-A	1000	McF_6	0	unif	wholeT_0.01	CBN, CBN-A, DiP, DiP-A, OT, OT-A
330	Yes	7	7-A	1000	McF_6	0	unif	wholeT_0.5	CBN, CBN-A, DiP, DiP-A, OT, OT-A
331	Yes	7	7-A	1000	McF_6	Inf	last	singleC	DiP, DiP-A, OT, OT-A
332	Yes	7	7-A	1000	McF_6	Inf	last	wholeT_0.01	DiP, OT, OT-A
333	Yes	7	7-A	1000	McF_6	Inf	last	wholeT_0.5	DiP, OT, OT-A
334	Yes	7	7-A	1000	McF_6	Inf	unif	singleC	CBN, CBN-A, DiP, DiP-A, OT, OT-A
335	Yes	7	7-A	1000	McF_6	Inf	unif	wholeT_0.01	CBN, CBN-A, OT, OT-A
336	Yes	7	7-A	1000	McF_6	Inf	unif	wholeT_0.5	CBN, CBN-A, DiP, DiP-A, OT, OT-A
337	Yes	7	7-A	200	Bozic	0	last	singleC	DiP, DiP-A, OT, OT-A
338	Yes	7	7-A	200	Bozic	0	last	wholeT_0.01	DiP, DiP-A, OT, OT-A
339	Yes	7	7-A	200	Bozic	0	last	wholeT_0.5	DiP, DiP-A, OT, OT-A
340	Yes	7	7-A	200	Bozic	0	unif	singleC	DiP, DiP-A, OT, OT-A
341	Yes	7	7-A	200	Bozic	0	unif	wholeT_0.01	CBN, CBN-A, DiP, DiP-A, OT, OT-A
342	Yes	7	7-A	200	Bozic	0	unif	wholeT_0.5	DiP, DiP-A, OT, OT-A
343	Yes	7	7-A	200	Bozic	Inf	last	singleC	DiP, DiP-A, OT, OT-A
344	Yes	7	7-A	200	Bozic	Inf	last	wholeT_0.01	DiP, DiP-A
345	Yes	7	7-A	200	Bozic	Inf	last	wholeT_0.5	DiP, DiP-A, OT, OT-A
346	Yes	7	7-A	200	Bozic	Inf	unif	singleC	DiP, DiP-A
347	Yes	7	7-A	200	Bozic	Inf	unif	wholeT_0.01	DiP, DiP-A, OT, OT-A
348	Yes	7	7-A	200	Bozic	Inf	unif	wholeT_0.5	DiP, DiP-A
349	Yes	7	7-A	200	exp	0	last	singleC	DiP, DiP-A, OT, OT-A
350	Yes	7	7-A	200	exp	0	last	wholeT_0.01	DiP, DiP-A
351	Yes	7	7-A	200	exp	0	last	wholeT_0.5	DiP, DiP-A, OT, OT-A
352	Yes	7	7-A	200	exp	0	unif	singleC	DiP, DiP-A, OT, OT-A
353	Yes	7	7-A	200	exp	0	unif	wholeT_0.01	DiP, DiP-A
354	Yes	7	7-A	200	exp	0	unif	wholeT_0.5	DiP, DiP-A, OT, OT-A
355	Yes	7	7-A	200	exp	Inf	last	singleC	DiP, DiP-A, OT, OT-A
356	Yes	7	7-A	200	exp	Inf	last	wholeT_0.01	DiP, DiP-A
357	Yes	7	7-A	200	exp	Inf	last	wholeT_0.5	DiP, DiP-A, OT, OT-A

Table 4: *(continued)*

	Conjunction	Drivers	Tree	S.Size	Model	sh	S.Time	S.Type	Best method(s)
358	Yes	7	7-A	200	exp	Inf	unif	singleC	DiP, DiP-A
359	Yes	7	7-A	200	exp	Inf	unif	wholeT_0.01	DiP, DiP-A, OT, OT-A
360	Yes	7	7-A	200	exp	Inf	unif	wholeT_0.5	DiP, DiP-A
361	Yes	7	7-A	200	McF_4	0	last	singleC	DiP, DiP-A, OT, OT-A
362	Yes	7	7-A	200	McF_4	0	last	wholeT_0.01	DiP, DiP-A, OT, OT-A
363	Yes	7	7-A	200	McF_4	0	last	wholeT_0.5	DiP, DiP-A
364	Yes	7	7-A	200	McF_4	0	unif	singleC	DiP, DiP-A, OT, OT-A
365	Yes	7	7-A	200	McF_4	0	unif	wholeT_0.01	DiP, DiP-A, OT, OT-A
366	Yes	7	7-A	200	McF_4	0	unif	wholeT_0.5	DiP, DiP-A, OT, OT-A
367	Yes	7	7-A	200	McF_4	Inf	last	singleC	DiP, DiP-A, OT, OT-A
368	Yes	7	7-A	200	McF_4	Inf	last	wholeT_0.01	DiP, DiP-A, OT, OT-A
369	Yes	7	7-A	200	McF_4	Inf	last	wholeT_0.5	DiP, DiP-A, OT, OT-A
370	Yes	7	7-A	200	McF_4	Inf	unif	singleC	DiP, DiP-A, OT, OT-A
371	Yes	7	7-A	200	McF_4	Inf	unif	wholeT_0.01	DiP, DiP-A, OT, OT-A
372	Yes	7	7-A	200	McF_4	Inf	unif	wholeT_0.5	DiP, DiP-A, OT, OT-A
373	Yes	7	7-A	200	McF_6	0	last	singleC	DiP, OT, OT-A
374	Yes	7	7-A	200	McF_6	0	last	wholeT_0.01	DiP, OT, OT-A
375	Yes	7	7-A	200	McF_6	0	last	wholeT_0.5	DiP, OT, OT-A
376	Yes	7	7-A	200	McF_6	0	unif	singleC	CBN, CBN-A, DiP, DiP-A
377	Yes	7	7-A	200	McF_6	0	unif	wholeT_0.01	CBN, CBN-A, DiP, DiP-A, OT, OT-A
378	Yes	7	7-A	200	McF_6	0	unif	wholeT_0.5	CBN, CBN-A, DiP, DiP-A
379	Yes	7	7-A	200	McF_6	Inf	last	singleC	DiP, DiP-A, OT, OT-A
380	Yes	7	7-A	200	McF_6	Inf	last	wholeT_0.01	DiP, OT, OT-A
381	Yes	7	7-A	200	McF_6	Inf	last	wholeT_0.5	DiP, DiP-A, OT, OT-A
382	Yes	7	7-A	200	McF_6	Inf	unif	singleC	CBN, CBN-A, DiP, DiP-A
383	Yes	7	7-A	200	McF_6	Inf	unif	wholeT_0.01	CBN, CBN-A, DiP, DiP-A
384	Yes	7	7-A	200	McF_6	Inf	unif	wholeT_0.5	CBN, CBN-A, DiP, DiP-A
385	Yes	7	7-A	100	Bozic	0	last	singleC	DiP, DiP-A, OT, OT-A
386	Yes	7	7-A	100	Bozic	0	last	wholeT_0.01	DiP, DiP-A, OT, OT-A
387	Yes	7	7-A	100	Bozic	0	last	wholeT_0.5	DiP, DiP-A, OT, OT-A
388	Yes	7	7-A	100	Bozic	0	unif	singleC	DiP, DiP-A, OT, OT-A
389	Yes	7	7-A	100	Bozic	0	unif	wholeT_0.01	CBN, DiP, DiP-A, OT, OT-A
390	Yes	7	7-A	100	Bozic	0	unif	wholeT_0.5	DiP, DiP-A, OT, OT-A
391	Yes	7	7-A	100	Bozic	Inf	last	singleC	DiP, DiP-A, OT, OT-A
392	Yes	7	7-A	100	Bozic	Inf	last	wholeT_0.01	DiP, DiP-A, OT, OT-A
393	Yes	7	7-A	100	Bozic	Inf	last	wholeT_0.5	DiP, DiP-A, OT, OT-A
394	Yes	7	7-A	100	Bozic	Inf	unif	singleC	DiP, DiP-A
395	Yes	7	7-A	100	Bozic	Inf	unif	wholeT_0.01	DiP-A, OT, OT-A
396	Yes	7	7-A	100	Bozic	Inf	unif	wholeT_0.5	DiP, DiP-A
397	Yes	7	7-A	100	exp	0	last	singleC	DiP, DiP-A, OT, OT-A

Table 4: *(continued)*

	Conjunction	Drivers	Tree	S.Size	Model	sh	S.Time	S.Type	Best method(s)
398	Yes	7	7-A	100	exp	0	last	wholeT_0.01	DiP, DiP-A
399	Yes	7	7-A	100	exp	0	last	wholeT_0.5	DiP, DiP-A, OT, OT-A
400	Yes	7	7-A	100	exp	0	unif	singleC	DiP, DiP-A, OT, OT-A
401	Yes	7	7-A	100	exp	0	unif	wholeT_0.01	DiP, DiP-A
402	Yes	7	7-A	100	exp	0	unif	wholeT_0.5	DiP, DiP-A, OT, OT-A
403	Yes	7	7-A	100	exp	Inf	last	singleC	DiP, DiP-A, OT, OT-A
404	Yes	7	7-A	100	exp	Inf	last	wholeT_0.01	DiP, DiP-A
405	Yes	7	7-A	100	exp	Inf	last	wholeT_0.5	DiP, DiP-A, OT, OT-A
406	Yes	7	7-A	100	exp	Inf	unif	singleC	DiP, DiP-A, OT, OT-A
407	Yes	7	7-A	100	exp	Inf	unif	wholeT_0.01	DiP, DiP-A, OT, OT-A
408	Yes	7	7-A	100	exp	Inf	unif	wholeT_0.5	DiP, DiP-A
409	Yes	7	7-A	100	McF_4	0	last	singleC	DiP, DiP-A, OT, OT-A
410	Yes	7	7-A	100	McF_4	0	last	wholeT_0.01	DiP, DiP-A
411	Yes	7	7-A	100	McF_4	0	last	wholeT_0.5	DiP, DiP-A, OT, OT-A
412	Yes	7	7-A	100	McF_4	0	unif	singleC	DiP, DiP-A
413	Yes	7	7-A	100	McF_4	0	unif	wholeT_0.01	DiP, DiP-A
414	Yes	7	7-A	100	McF_4	0	unif	wholeT_0.5	DiP, DiP-A, OT, OT-A
415	Yes	7	7-A	100	McF_4	Inf	last	singleC	DiP, DiP-A, OT, OT-A
416	Yes	7	7-A	100	McF_4	Inf	last	wholeT_0.01	DiP, DiP-A, OT, OT-A
417	Yes	7	7-A	100	McF_4	Inf	last	wholeT_0.5	DiP, DiP-A, OT, OT-A
418	Yes	7	7-A	100	McF_4	Inf	unif	singleC	DiP, DiP-A, OT, OT-A
419	Yes	7	7-A	100	McF_4	Inf	unif	wholeT_0.01	DiP, DiP-A
420	Yes	7	7-A	100	McF_4	Inf	unif	wholeT_0.5	DiP, DiP-A, OT, OT-A
421	Yes	7	7-A	100	McF_6	0	last	singleC	DiP, DiP-A, OT, OT-A
422	Yes	7	7-A	100	McF_6	0	last	wholeT_0.01	DiP, OT, OT-A
423	Yes	7	7-A	100	McF_6	0	last	wholeT_0.5	DiP, OT, OT-A
424	Yes	7	7-A	100	McF_6	0	unif	singleC	CBN, CBN-A, DiP, DiP-A
425	Yes	7	7-A	100	McF_6	0	unif	wholeT_0.01	CBN, CBN-A, DiP, DiP-A
426	Yes	7	7-A	100	McF_6	0	unif	wholeT_0.5	CBN, CBN-A, DiP, DiP-A
427	Yes	7	7-A	100	McF_6	Inf	last	singleC	DiP, DiP-A, OT, OT-A
428	Yes	7	7-A	100	McF_6	Inf	last	wholeT_0.01	DiP, OT, OT-A
429	Yes	7	7-A	100	McF_6	Inf	last	wholeT_0.5	DiP, DiP-A, OT, OT-A
430	Yes	7	7-A	100	McF_6	Inf	unif	singleC	CBN, CBN-A, DiP, DiP-A
431	Yes	7	7-A	100	McF_6	Inf	unif	wholeT_0.01	CBN, CBN-A, DiP, DiP-A
432	Yes	7	7-A	100	McF_6	Inf	unif	wholeT_0.5	CBN, CBN-A, DiP, DiP-A
433	No	11	11-B	1000	Bozic	0	last	singleC	OT, OT-A
434	No	11	11-B	1000	Bozic	0	last	wholeT_0.01	DiP, DiP-A
435	No	11	11-B	1000	Bozic	0	last	wholeT_0.5	DiP, DiP-A, OT, OT-A
436	No	11	11-B	1000	Bozic	0	unif	singleC	DiP, DiP-A, OT, OT-A
437	No	11	11-B	1000	Bozic	0	unif	wholeT_0.01	DiP, DiP-A

Table 4: *(continued)*

	Conjunction	Drivers	Tree	S.Size	Model	sh	S.Time	S.Type	Best method(s)
438	No	11	11-B	1000	Bozic	0	unif	wholeT_0.5	DiP, DiP-A
439	No	11	11-B	1000	Bozic	Inf	last	singleC	OT, OT-A
440	No	11	11-B	1000	Bozic	Inf	last	wholeT_0.01	DiP-A
441	No	11	11-B	1000	Bozic	Inf	last	wholeT_0.5	OT, OT-A
442	No	11	11-B	1000	Bozic	Inf	unif	singleC	DiP, DiP-A
443	No	11	11-B	1000	Bozic	Inf	unif	wholeT_0.01	OT, OT-A
444	No	11	11-B	1000	Bozic	Inf	unif	wholeT_0.5	DiP, DiP-A
445	No	11	11-B	1000	exp	0	last	singleC	DiP, DiP-A, OT, OT-A
446	No	11	11-B	1000	exp	0	last	wholeT_0.01	DiP-A, OT, OT-A
447	No	11	11-B	1000	exp	0	last	wholeT_0.5	DiP, DiP-A, OT, OT-A
448	No	11	11-B	1000	exp	0	unif	singleC	DiP, DiP-A, OT, OT-A
449	No	11	11-B	1000	exp	0	unif	wholeT_0.01	DiP, DiP-A
450	No	11	11-B	1000	exp	0	unif	wholeT_0.5	DiP, DiP-A, OT, OT-A
451	No	11	11-B	1000	exp	Inf	last	singleC	OT, OT-A
452	No	11	11-B	1000	exp	Inf	last	wholeT_0.01	OT, OT-A
453	No	11	11-B	1000	exp	Inf	last	wholeT_0.5	OT, OT-A
454	No	11	11-B	1000	exp	Inf	unif	singleC	DiP-A, OT, OT-A
455	No	11	11-B	1000	exp	Inf	unif	wholeT_0.01	DiP, DiP-A, OT, OT-A
456	No	11	11-B	1000	exp	Inf	unif	wholeT_0.5	DiP, DiP-A, OT, OT-A
457	No	11	11-B	1000	McF_4	0	last	singleC	DiP, DiP-A, OT, OT-A
458	No	11	11-B	1000	McF_4	0	last	wholeT_0.01	DiP, DiP-A, OT, OT-A
459	No	11	11-B	1000	McF_4	0	last	wholeT_0.5	DiP-A, OT, OT-A
460	No	11	11-B	1000	McF_4	0	unif	singleC	DiP, DiP-A
461	No	11	11-B	1000	McF_4	0	unif	wholeT_0.01	DiP-A, OT, OT-A
462	No	11	11-B	1000	McF_4	0	unif	wholeT_0.5	DiP, DiP-A, OT, OT-A
463	No	11	11-B	1000	McF_4	Inf	last	singleC	OT, OT-A
464	No	11	11-B	1000	McF_4	Inf	last	wholeT_0.01	DiP, DiP-A, OT, OT-A
465	No	11	11-B	1000	McF_4	Inf	last	wholeT_0.5	DiP-A, OT, OT-A
466	No	11	11-B	1000	McF_4	Inf	unif	singleC	DiP, DiP-A, OT, OT-A
467	No	11	11-B	1000	McF_4	Inf	unif	wholeT_0.01	OT, OT-A
468	No	11	11-B	1000	McF_4	Inf	unif	wholeT_0.5	DiP, DiP-A, OT, OT-A
469	No	11	11-B	1000	McF_6	0	last	singleC	DiP, OT
470	No	11	11-B	1000	McF_6	0	last	wholeT_0.01	DiP, OT
471	No	11	11-B	1000	McF_6	0	last	wholeT_0.5	DiP, OT
472	No	11	11-B	1000	McF_6	0	unif	singleC	DiP, DiP-A, OT, OT-A
473	No	11	11-B	1000	McF_6	0	unif	wholeT_0.01	DiP, DiP-A, OT, OT-A
474	No	11	11-B	1000	McF_6	0	unif	wholeT_0.5	DiP, DiP-A
475	No	11	11-B	1000	McF_6	Inf	last	singleC	DiP, OT
476	No	11	11-B	1000	McF_6	Inf	last	wholeT_0.01	DiP, OT
477	No	11	11-B	1000	McF_6	Inf	last	wholeT_0.5	DiP, OT

Table 4: *(continued)*

	Conjunction	Drivers	Tree	S.Size	Model	sh	S.Time	S.Type	Best method(s)
478	No	11	11-B	1000	McF_6	Inf	unif	singleC	DiP, DiP-A
479	No	11	11-B	1000	McF_6	Inf	unif	wholeT_0.01	DiP, DiP-A
480	No	11	11-B	1000	McF_6	Inf	unif	wholeT_0.5	DiP, DiP-A
481	No	11	11-B	200	Bozic	0	last	singleC	DiP, DiP-A, OT, OT-A
482	No	11	11-B	200	Bozic	0	last	wholeT_0.01	DiP
483	No	11	11-B	200	Bozic	0	last	wholeT_0.5	DiP, DiP-A, OT, OT-A
484	No	11	11-B	200	Bozic	0	unif	singleC	DiP, DiP-A, OT, OT-A
485	No	11	11-B	200	Bozic	0	unif	wholeT_0.01	DiP, DiP-A
486	No	11	11-B	200	Bozic	0	unif	wholeT_0.5	DiP, DiP-A, OT, OT-A
487	No	11	11-B	200	Bozic	Inf	last	singleC	DiP, DiP-A, OT, OT-A
488	No	11	11-B	200	Bozic	Inf	last	wholeT_0.01	DiP, DiP-A, OT, OT-A
489	No	11	11-B	200	Bozic	Inf	last	wholeT_0.5	DiP, DiP-A, OT, OT-A
490	No	11	11-B	200	Bozic	Inf	unif	singleC	DiP, DiP-A
491	No	11	11-B	200	Bozic	Inf	unif	wholeT_0.01	DiP, DiP-A
492	No	11	11-B	200	Bozic	Inf	unif	wholeT_0.5	DiP, DiP-A
493	No	11	11-B	200	exp	0	last	singleC	DiP, DiP-A, OT, OT-A
494	No	11	11-B	200	exp	0	last	wholeT_0.01	DiP, DiP-A, OT, OT-A
495	No	11	11-B	200	exp	0	last	wholeT_0.5	DiP, DiP-A, OT, OT-A
496	No	11	11-B	200	exp	0	unif	singleC	DiP, DiP-A, OT, OT-A
497	No	11	11-B	200	exp	0	unif	wholeT_0.01	DiP, DiP-A, OT, OT-A
498	No	11	11-B	200	exp	0	unif	wholeT_0.5	DiP, DiP-A, OT, OT-A
499	No	11	11-B	200	exp	Inf	last	singleC	DiP, DiP-A, OT, OT-A
500	No	11	11-B	200	exp	Inf	last	wholeT_0.01	DiP, OT, OT-A
501	No	11	11-B	200	exp	Inf	last	wholeT_0.5	DiP, DiP-A, OT, OT-A
502	No	11	11-B	200	exp	Inf	unif	singleC	DiP, DiP-A, OT, OT-A
503	No	11	11-B	200	exp	Inf	unif	wholeT_0.01	DiP, DiP-A
504	No	11	11-B	200	exp	Inf	unif	wholeT_0.5	DiP, DiP-A, OT, OT-A
505	No	11	11-B	200	McF_4	0	last	singleC	DiP, DiP-A, OT, OT-A
506	No	11	11-B	200	McF_4	0	last	wholeT_0.01	DiP, DiP-A, OT, OT-A
507	No	11	11-B	200	McF_4	0	last	wholeT_0.5	DiP-A, OT, OT-A
508	No	11	11-B	200	McF_4	0	unif	singleC	DiP, DiP-A, OT, OT-A
509	No	11	11-B	200	McF_4	0	unif	wholeT_0.01	DiP, DiP-A
510	No	11	11-B	200	McF_4	0	unif	wholeT_0.5	DiP, DiP-A, OT
511	No	11	11-B	200	McF_4	Inf	last	singleC	OT, OT-A
512	No	11	11-B	200	McF_4	Inf	last	wholeT_0.01	DiP, DiP-A, OT, OT-A
513	No	11	11-B	200	McF_4	Inf	last	wholeT_0.5	DiP, DiP-A, OT, OT-A
514	No	11	11-B	200	McF_4	Inf	unif	singleC	DiP, DiP-A, OT, OT-A
515	No	11	11-B	200	McF_4	Inf	unif	wholeT_0.01	DiP, DiP-A, OT, OT-A
516	No	11	11-B	200	McF_4	Inf	unif	wholeT_0.5	DiP, DiP-A, OT, OT-A
517	No	11	11-B	200	McF_6	0	last	singleC	DiP, OT

Table 4: *(continued)*

	Conjunction	Drivers	Tree	S.Size	Model	sh	S.Time	S.Type	Best method(s)
518	No	11	11-B	200	McF_6	0	last	wholeT_0.01	DiP, OT
519	No	11	11-B	200	McF_6	0	last	wholeT_0.5	DiP, OT
520	No	11	11-B	200	McF_6	0	unif	singleC	DiP, DiP-A, OT, OT-A
521	No	11	11-B	200	McF_6	0	unif	wholeT_0.01	DiP, DiP-A, OT, OT-A
522	No	11	11-B	200	McF_6	0	unif	wholeT_0.5	OT, OT-A
523	No	11	11-B	200	McF_6	Inf	last	singleC	DiP, OT
524	No	11	11-B	200	McF_6	Inf	last	wholeT_0.01	DiP, OT
525	No	11	11-B	200	McF_6	Inf	last	wholeT_0.5	DiP, OT
526	No	11	11-B	200	McF_6	Inf	unif	singleC	OT, OT-A
527	No	11	11-B	200	McF_6	Inf	unif	wholeT_0.01	OT, OT-A
528	No	11	11-B	200	McF_6	Inf	unif	wholeT_0.5	DiP, OT, OT-A
529	No	11	11-B	100	Bozic	0	last	singleC	DiP, DiP-A, OT, OT-A
530	No	11	11-B	100	Bozic	0	last	wholeT_0.01	DiP, DiP-A
531	No	11	11-B	100	Bozic	0	last	wholeT_0.5	DiP, DiP-A, OT, OT-A
532	No	11	11-B	100	Bozic	0	unif	singleC	DiP, DiP-A, OT, OT-A
533	No	11	11-B	100	Bozic	0	unif	wholeT_0.01	DiP, DiP-A
534	No	11	11-B	100	Bozic	0	unif	wholeT_0.5	DiP, DiP-A, OT, OT-A
535	No	11	11-B	100	Bozic	Inf	last	singleC	DiP, DiP-A, OT, OT-A
536	No	11	11-B	100	Bozic	Inf	last	wholeT_0.01	DiP, DiP-A, OT, OT-A
537	No	11	11-B	100	Bozic	Inf	last	wholeT_0.5	DiP, DiP-A, OT, OT-A
538	No	11	11-B	100	Bozic	Inf	unif	singleC	DiP, DiP-A
539	No	11	11-B	100	Bozic	Inf	unif	wholeT_0.01	DiP, DiP-A
540	No	11	11-B	100	Bozic	Inf	unif	wholeT_0.5	DiP, DiP-A, OT, OT-A
541	No	11	11-B	100	exp	0	last	singleC	DiP, DiP-A, OT, OT-A
542	No	11	11-B	100	exp	0	last	wholeT_0.01	DiP, DiP-A
543	No	11	11-B	100	exp	0	last	wholeT_0.5	DiP, DiP-A, OT, OT-A
544	No	11	11-B	100	exp	0	unif	singleC	DiP, DiP-A, OT, OT-A
545	No	11	11-B	100	exp	0	unif	wholeT_0.01	DiP, DiP-A, OT, OT-A
546	No	11	11-B	100	exp	0	unif	wholeT_0.5	DiP, DiP-A, OT, OT-A
547	No	11	11-B	100	exp	Inf	last	singleC	DiP, DiP-A, OT, OT-A
548	No	11	11-B	100	exp	Inf	last	wholeT_0.01	DiP, DiP-A
549	No	11	11-B	100	exp	Inf	last	wholeT_0.5	DiP, DiP-A, OT, OT-A
550	No	11	11-B	100	exp	Inf	unif	singleC	DiP, DiP-A, OT, OT-A
551	No	11	11-B	100	exp	Inf	unif	wholeT_0.01	DiP, DiP-A
552	No	11	11-B	100	exp	Inf	unif	wholeT_0.5	DiP, DiP-A, OT, OT-A
553	No	11	11-B	100	McF_4	0	last	singleC	DiP, DiP-A
554	No	11	11-B	100	McF_4	0	last	wholeT_0.01	DiP, DiP-A, OT, OT-A
555	No	11	11-B	100	McF_4	0	last	wholeT_0.5	DiP, DiP-A, OT, OT-A
556	No	11	11-B	100	McF_4	0	unif	singleC	DiP, DiP-A
557	No	11	11-B	100	McF_4	0	unif	wholeT_0.01	DiP, DiP-A

Table 4: *(continued)*

	Conjunction	Drivers	Tree	S.Size	Model	sh	S.Time	S.Type	Best method(s)
558	No	11	11-B	100	McF_4	0	unif	wholeT_0.5	DiP, DiP-A, OT, OT-A
559	No	11	11-B	100	McF_4	Inf	last	singleC	DiP, DiP-A, OT, OT-A
560	No	11	11-B	100	McF_4	Inf	last	wholeT_0.01	DiP, DiP-A, OT, OT-A
561	No	11	11-B	100	McF_4	Inf	last	wholeT_0.5	DiP, DiP-A, OT, OT-A
562	No	11	11-B	100	McF_4	Inf	unif	singleC	DiP, DiP-A, OT, OT-A
563	No	11	11-B	100	McF_4	Inf	unif	wholeT_0.01	DiP, DiP-A, OT, OT-A
564	No	11	11-B	100	McF_4	Inf	unif	wholeT_0.5	DiP, DiP-A, OT, OT-A
565	No	11	11-B	100	McF_6	0	last	singleC	DiP, OT
566	No	11	11-B	100	McF_6	0	last	wholeT_0.01	DiP, OT
567	No	11	11-B	100	McF_6	0	last	wholeT_0.5	DiP, OT
568	No	11	11-B	100	McF_6	0	unif	singleC	DiP, DiP-A
569	No	11	11-B	100	McF_6	0	unif	wholeT_0.01	DiP, DiP-A, OT, OT-A
570	No	11	11-B	100	McF_6	0	unif	wholeT_0.5	DiP, DiP-A, OT, OT-A
571	No	11	11-B	100	McF_6	Inf	last	singleC	DiP, OT
572	No	11	11-B	100	McF_6	Inf	last	wholeT_0.01	DiP, OT
573	No	11	11-B	100	McF_6	Inf	last	wholeT_0.5	DiP, OT
574	No	11	11-B	100	McF_6	Inf	unif	singleC	DiP, DiP-A, OT, OT-A
575	No	11	11-B	100	McF_6	Inf	unif	wholeT_0.01	DiP, DiP-A, OT, OT-A
576	No	11	11-B	100	McF_6	Inf	unif	wholeT_0.5	DiP, DiP-A, OT, OT-A
577	No	9	9-B	1000	Bozic	0	last	singleC	DiP-A, OT, OT-A
578	No	9	9-B	1000	Bozic	0	last	wholeT_0.01	DiP, DiP-A
579	No	9	9-B	1000	Bozic	0	last	wholeT_0.5	DiP, DiP-A, OT, OT-A
580	No	9	9-B	1000	Bozic	0	unif	singleC	DiP, DiP-A, OT, OT-A
581	No	9	9-B	1000	Bozic	0	unif	wholeT_0.01	DiP, DiP-A, OT, OT-A
582	No	9	9-B	1000	Bozic	0	unif	wholeT_0.5	DiP, DiP-A
583	No	9	9-B	1000	Bozic	Inf	last	singleC	OT, OT-A
584	No	9	9-B	1000	Bozic	Inf	last	wholeT_0.01	DiP, DiP-A
585	No	9	9-B	1000	Bozic	Inf	last	wholeT_0.5	OT, OT-A
586	No	9	9-B	1000	Bozic	Inf	unif	singleC	DiP, DiP-A
587	No	9	9-B	1000	Bozic	Inf	unif	wholeT_0.01	DiP, DiP-A, OT, OT-A
588	No	9	9-B	1000	Bozic	Inf	unif	wholeT_0.5	DiP, DiP-A, OT, OT-A
589	No	9	9-B	1000	exp	0	last	singleC	DiP, DiP-A, OT, OT-A
590	No	9	9-B	1000	exp	0	last	wholeT_0.01	DiP-A, OT, OT-A
591	No	9	9-B	1000	exp	0	last	wholeT_0.5	DiP, DiP-A, OT, OT-A
592	No	9	9-B	1000	exp	0	unif	singleC	DiP, DiP-A, OT, OT-A
593	No	9	9-B	1000	exp	0	unif	wholeT_0.01	DiP, DiP-A
594	No	9	9-B	1000	exp	0	unif	wholeT_0.5	DiP, DiP-A, OT, OT-A
595	No	9	9-B	1000	exp	Inf	last	singleC	DiP, DiP-A, OT, OT-A
596	No	9	9-B	1000	exp	Inf	last	wholeT_0.01	CBN-A, DiP, DiP-A, OT, OT-A
597	No	9	9-B	1000	exp	Inf	last	wholeT_0.5	DiP, DiP-A, OT, OT-A

Table 4: *(continued)*

	Conjunction	Drivers	Tree	S.Size	Model	sh	S.Time	S.Type	Best method(s)
598	No	9	9-B	1000	exp	Inf	unif	singleC	DiP, DiP-A, OT, OT-A
599	No	9	9-B	1000	exp	Inf	unif	wholeT_0.01	DiP-A, OT, OT-A
600	No	9	9-B	1000	exp	Inf	unif	wholeT_0.5	DiP, DiP-A, OT, OT-A
601	No	9	9-B	1000	McF_4	0	last	singleC	DiP-A, OT, OT-A
602	No	9	9-B	1000	McF_4	0	last	wholeT_0.01	DiP, DiP-A, OT, OT-A
603	No	9	9-B	1000	McF_4	0	last	wholeT_0.5	OT, OT-A
604	No	9	9-B	1000	McF_4	0	unif	singleC	DiP, DiP-A, OT, OT-A
605	No	9	9-B	1000	McF_4	0	unif	wholeT_0.01	DiP, DiP-A, OT, OT-A
606	No	9	9-B	1000	McF_4	0	unif	wholeT_0.5	DiP, DiP-A, OT, OT-A
607	No	9	9-B	1000	McF_4	Inf	last	singleC	OT, OT-A
608	No	9	9-B	1000	McF_4	Inf	last	wholeT_0.01	DiP, DiP-A, OT, OT-A
609	No	9	9-B	1000	McF_4	Inf	last	wholeT_0.5	DiP, DiP-A, OT, OT-A
610	No	9	9-B	1000	McF_4	Inf	unif	singleC	DiP, DiP-A, OT, OT-A
611	No	9	9-B	1000	McF_4	Inf	unif	wholeT_0.01	DiP-A, OT, OT-A
612	No	9	9-B	1000	McF_4	Inf	unif	wholeT_0.5	DiP, DiP-A, OT, OT-A
613	No	9	9-B	1000	McF_6	0	last	singleC	DiP, OT
614	No	9	9-B	1000	McF_6	0	last	wholeT_0.01	DiP, OT
615	No	9	9-B	1000	McF_6	0	last	wholeT_0.5	DiP, OT
616	No	9	9-B	1000	McF_6	0	unif	singleC	DiP, DiP-A, OT, OT-A
617	No	9	9-B	1000	McF_6	0	unif	wholeT_0.01	DiP, DiP-A, OT, OT-A
618	No	9	9-B	1000	McF_6	0	unif	wholeT_0.5	DiP, DiP-A, OT, OT-A
619	No	9	9-B	1000	McF_6	Inf	last	singleC	DiP, OT
620	No	9	9-B	1000	McF_6	Inf	last	wholeT_0.01	DiP, OT
621	No	9	9-B	1000	McF_6	Inf	last	wholeT_0.5	DiP, OT
622	No	9	9-B	1000	McF_6	Inf	unif	singleC	DiP, DiP-A, OT, OT-A
623	No	9	9-B	1000	McF_6	Inf	unif	wholeT_0.01	DiP, DiP-A, OT, OT-A
624	No	9	9-B	1000	McF_6	Inf	unif	wholeT_0.5	DiP, DiP-A, OT, OT-A
625	No	9	9-B	200	Bozic	0	last	singleC	DiP, DiP-A, OT, OT-A
626	No	9	9-B	200	Bozic	0	last	wholeT_0.01	DiP, DiP-A
627	No	9	9-B	200	Bozic	0	last	wholeT_0.5	DiP, DiP-A, OT, OT-A
628	No	9	9-B	200	Bozic	0	unif	singleC	DiP, DiP-A, OT, OT-A
629	No	9	9-B	200	Bozic	0	unif	wholeT_0.01	DiP, DiP-A
630	No	9	9-B	200	Bozic	0	unif	wholeT_0.5	DiP, DiP-A, OT, OT-A
631	No	9	9-B	200	Bozic	Inf	last	singleC	DiP, DiP-A, OT, OT-A
632	No	9	9-B	200	Bozic	Inf	last	wholeT_0.01	DiP, DiP-A, OT, OT-A
633	No	9	9-B	200	Bozic	Inf	last	wholeT_0.5	DiP, DiP-A, OT, OT-A
634	No	9	9-B	200	Bozic	Inf	unif	singleC	DiP, DiP-A, OT, OT-A
635	No	9	9-B	200	Bozic	Inf	unif	wholeT_0.01	DiP, DiP-A, OT, OT-A
636	No	9	9-B	200	Bozic	Inf	unif	wholeT_0.5	DiP, DiP-A
637	No	9	9-B	200	exp	0	last	singleC	DiP, DiP-A, OT, OT-A

Table 4: *(continued)*

	Conjunction	Drivers	Tree	S.Size	Model	sh	S.Time	S.Type	Best method(s)
638	No	9	9-B	200	exp	0	last	wholeT_0.01	DiP, DiP-A
639	No	9	9-B	200	exp	0	last	wholeT_0.5	DiP, DiP-A, OT, OT-A
640	No	9	9-B	200	exp	0	unif	singleC	DiP, DiP-A, OT, OT-A
641	No	9	9-B	200	exp	0	unif	wholeT_0.01	DiP, DiP-A, OT, OT-A
642	No	9	9-B	200	exp	0	unif	wholeT_0.5	DiP, DiP-A, OT, OT-A
643	No	9	9-B	200	exp	Inf	last	singleC	DiP, DiP-A, OT, OT-A
644	No	9	9-B	200	exp	Inf	last	wholeT_0.01	DiP, DiP-A
645	No	9	9-B	200	exp	Inf	last	wholeT_0.5	DiP, DiP-A, OT, OT-A
646	No	9	9-B	200	exp	Inf	unif	singleC	DiP, DiP-A, OT, OT-A
647	No	9	9-B	200	exp	Inf	unif	wholeT_0.01	DiP, DiP-A, OT, OT-A
648	No	9	9-B	200	exp	Inf	unif	wholeT_0.5	DiP, DiP-A, OT, OT-A
649	No	9	9-B	200	McF_4	0	last	singleC	OT, OT-A
650	No	9	9-B	200	McF_4	0	last	wholeT_0.01	DiP, DiP-A, OT, OT-A
651	No	9	9-B	200	McF_4	0	last	wholeT_0.5	DiP-A, OT, OT-A
652	No	9	9-B	200	McF_4	0	unif	singleC	DiP, DiP-A, OT, OT-A
653	No	9	9-B	200	McF_4	0	unif	wholeT_0.01	DiP, DiP-A
654	No	9	9-B	200	McF_4	0	unif	wholeT_0.5	DiP, DiP-A, OT, OT-A
655	No	9	9-B	200	McF_4	Inf	last	singleC	OT, OT-A
656	No	9	9-B	200	McF_4	Inf	last	wholeT_0.01	OT, OT-A
657	No	9	9-B	200	McF_4	Inf	last	wholeT_0.5	DiP, DiP-A, OT, OT-A
658	No	9	9-B	200	McF_4	Inf	unif	singleC	DiP, DiP-A, OT, OT-A
659	No	9	9-B	200	McF_4	Inf	unif	wholeT_0.01	DiP, DiP-A, OT, OT-A
660	No	9	9-B	200	McF_4	Inf	unif	wholeT_0.5	DiP, DiP-A, OT, OT-A
661	No	9	9-B	200	McF_6	0	last	singleC	DiP, OT
662	No	9	9-B	200	McF_6	0	last	wholeT_0.01	DiP, OT
663	No	9	9-B	200	McF_6	0	last	wholeT_0.5	DiP, OT
664	No	9	9-B	200	McF_6	0	unif	singleC	DiP, DiP-A, OT, OT-A
665	No	9	9-B	200	McF_6	0	unif	wholeT_0.01	DiP, DiP-A, OT, OT-A
666	No	9	9-B	200	McF_6	0	unif	wholeT_0.5	DiP, OT, OT-A
667	No	9	9-B	200	McF_6	Inf	last	singleC	DiP, OT
668	No	9	9-B	200	McF_6	Inf	last	wholeT_0.01	DiP, OT
669	No	9	9-B	200	McF_6	Inf	last	wholeT_0.5	DiP, OT
670	No	9	9-B	200	McF_6	Inf	unif	singleC	DiP, OT, OT-A
671	No	9	9-B	200	McF_6	Inf	unif	wholeT_0.01	OT, OT-A
672	No	9	9-B	200	McF_6	Inf	unif	wholeT_0.5	OT, OT-A
673	No	9	9-B	100	Bozic	0	last	singleC	DiP, DiP-A, OT, OT-A
674	No	9	9-B	100	Bozic	0	last	wholeT_0.01	DiP, DiP-A
675	No	9	9-B	100	Bozic	0	last	wholeT_0.5	DiP, DiP-A, OT, OT-A
676	No	9	9-B	100	Bozic	0	unif	singleC	DiP, DiP-A, OT, OT-A
677	No	9	9-B	100	Bozic	0	unif	wholeT_0.01	DiP, DiP-A

Table 4: *(continued)*

	Conjunction	Drivers	Tree	S.Size	Model	sh	S.Time	S.Type	Best method(s)
678	No	9	9-B	100	Bozic	0	unif	wholeT_0.5	DiP, DiP-A, OT, OT-A
679	No	9	9-B	100	Bozic	Inf	last	singleC	DiP, DiP-A, OT, OT-A
680	No	9	9-B	100	Bozic	Inf	last	wholeT_0.01	DiP, DiP-A
681	No	9	9-B	100	Bozic	Inf	last	wholeT_0.5	DiP, DiP-A, OT, OT-A
682	No	9	9-B	100	Bozic	Inf	unif	singleC	DiP, DiP-A, OT, OT-A
683	No	9	9-B	100	Bozic	Inf	unif	wholeT_0.01	DiP, DiP-A, OT, OT-A
684	No	9	9-B	100	Bozic	Inf	unif	wholeT_0.5	DiP, DiP-A
685	No	9	9-B	100	exp	0	last	singleC	DiP, DiP-A, OT, OT-A
686	No	9	9-B	100	exp	0	last	wholeT_0.01	DiP, DiP-A, OT, OT-A
687	No	9	9-B	100	exp	0	last	wholeT_0.5	DiP, DiP-A, OT, OT-A
688	No	9	9-B	100	exp	0	unif	singleC	DiP, DiP-A, OT, OT-A
689	No	9	9-B	100	exp	0	unif	wholeT_0.01	DiP, DiP-A, OT, OT-A
690	No	9	9-B	100	exp	0	unif	wholeT_0.5	DiP, DiP-A, OT, OT-A
691	No	9	9-B	100	exp	Inf	last	singleC	DiP, DiP-A, OT, OT-A
692	No	9	9-B	100	exp	Inf	last	wholeT_0.01	DiP, DiP-A
693	No	9	9-B	100	exp	Inf	last	wholeT_0.5	DiP, DiP-A, OT, OT-A
694	No	9	9-B	100	exp	Inf	unif	singleC	DiP, DiP-A
695	No	9	9-B	100	exp	Inf	unif	wholeT_0.01	DiP, DiP-A, OT, OT-A
696	No	9	9-B	100	exp	Inf	unif	wholeT_0.5	DiP, DiP-A, OT, OT-A
697	No	9	9-B	100	McF_4	0	last	singleC	DiP, DiP-A, OT, OT-A
698	No	9	9-B	100	McF_4	0	last	wholeT_0.01	DiP, DiP-A, OT, OT-A
699	No	9	9-B	100	McF_4	0	last	wholeT_0.5	DiP, DiP-A, OT, OT-A
700	No	9	9-B	100	McF_4	0	unif	singleC	DiP, DiP-A, OT, OT-A
701	No	9	9-B	100	McF_4	0	unif	wholeT_0.01	DiP, DiP-A
702	No	9	9-B	100	McF_4	0	unif	wholeT_0.5	DiP, DiP-A, OT, OT-A
703	No	9	9-B	100	McF_4	Inf	last	singleC	DiP, DiP-A, OT, OT-A
704	No	9	9-B	100	McF_4	Inf	last	wholeT_0.01	DiP, DiP-A, OT, OT-A
705	No	9	9-B	100	McF_4	Inf	last	wholeT_0.5	DiP, DiP-A, OT, OT-A
706	No	9	9-B	100	McF_4	Inf	unif	singleC	DiP, DiP-A, OT, OT-A
707	No	9	9-B	100	McF_4	Inf	unif	wholeT_0.01	DiP, DiP-A, OT, OT-A
708	No	9	9-B	100	McF_4	Inf	unif	wholeT_0.5	DiP, DiP-A, OT, OT-A
709	No	9	9-B	100	McF_6	0	last	singleC	DiP, DiP-A, OT
710	No	9	9-B	100	McF_6	0	last	wholeT_0.01	DiP, OT
711	No	9	9-B	100	McF_6	0	last	wholeT_0.5	DiP, OT
712	No	9	9-B	100	McF_6	0	unif	singleC	DiP, DiP-A, OT, OT-A
713	No	9	9-B	100	McF_6	0	unif	wholeT_0.01	DiP, DiP-A, OT, OT-A
714	No	9	9-B	100	McF_6	0	unif	wholeT_0.5	DiP, DiP-A, OT, OT-A
715	No	9	9-B	100	McF_6	Inf	last	singleC	DiP, DiP-A, OT
716	No	9	9-B	100	McF_6	Inf	last	wholeT_0.01	DiP, OT
717	No	9	9-B	100	McF_6	Inf	last	wholeT_0.5	DiP, DiP-A, OT

Table 4: *(continued)*

	Conjunction	Drivers	Tree	S.Size	Model	sh	S.Time	S.Type	Best method(s)
718	No	9	9-B	100	McF_6	Inf	unif	singleC	DiP, DiP-A, OT, OT-A
719	No	9	9-B	100	McF_6	Inf	unif	wholeT_0.01	DiP, DiP-A, OT, OT-A
720	No	9	9-B	100	McF_6	Inf	unif	wholeT_0.5	DiP, DiP-A, OT, OT-A
721	No	7	7-B	1000	Bozic	0	last	singleC	DiP, DiP-A, OT, OT-A
722	No	7	7-B	1000	Bozic	0	last	wholeT_0.01	DiP, DiP-A, OT, OT-A
723	No	7	7-B	1000	Bozic	0	last	wholeT_0.5	DiP, DiP-A, OT, OT-A
724	No	7	7-B	1000	Bozic	0	unif	singleC	DiP, DiP-A, OT, OT-A
725	No	7	7-B	1000	Bozic	0	unif	wholeT_0.01	CBN-A, DiP, DiP-A, OT, OT-A
726	No	7	7-B	1000	Bozic	0	unif	wholeT_0.5	DiP, DiP-A, OT, OT-A
727	No	7	7-B	1000	Bozic	Inf	last	singleC	DiP, DiP-A, OT, OT-A
728	No	7	7-B	1000	Bozic	Inf	last	wholeT_0.01	CBN, CBN-A, DiP, DiP-A, OT, OT-A
729	No	7	7-B	1000	Bozic	Inf	last	wholeT_0.5	DiP-A, OT, OT-A
730	No	7	7-B	1000	Bozic	Inf	unif	singleC	CBN, CBN-A, DiP
731	No	7	7-B	1000	Bozic	Inf	unif	wholeT_0.01	CBN, CBN-A, OT, OT-A
732	No	7	7-B	1000	Bozic	Inf	unif	wholeT_0.5	CBN, CBN-A, DiP, DiP-A
733	No	7	7-B	1000	exp	0	last	singleC	DiP, DiP-A, OT, OT-A
734	No	7	7-B	1000	exp	0	last	wholeT_0.01	DiP, DiP-A, OT, OT-A
735	No	7	7-B	1000	exp	0	last	wholeT_0.5	DiP, DiP-A, OT, OT-A
736	No	7	7-B	1000	exp	0	unif	singleC	DiP, DiP-A, OT, OT-A
737	No	7	7-B	1000	exp	0	unif	wholeT_0.01	DiP, DiP-A
738	No	7	7-B	1000	exp	0	unif	wholeT_0.5	DiP, DiP-A, OT, OT-A
739	No	7	7-B	1000	exp	Inf	last	singleC	DiP, DiP-A, OT, OT-A
740	No	7	7-B	1000	exp	Inf	last	wholeT_0.01	CBN, CBN-A, OT, OT-A
741	No	7	7-B	1000	exp	Inf	last	wholeT_0.5	DiP, DiP-A, OT, OT-A
742	No	7	7-B	1000	exp	Inf	unif	singleC	DiP, DiP-A
743	No	7	7-B	1000	exp	Inf	unif	wholeT_0.01	CBN, CBN-A, DiP, DiP-A, OT, OT-A
744	No	7	7-B	1000	exp	Inf	unif	wholeT_0.5	DiP, DiP-A
745	No	7	7-B	1000	McF_4	0	last	singleC	DiP, DiP-A, OT, OT-A
746	No	7	7-B	1000	McF_4	0	last	wholeT_0.01	DiP, DiP-A, OT, OT-A
747	No	7	7-B	1000	McF_4	0	last	wholeT_0.5	DiP, DiP-A, OT, OT-A
748	No	7	7-B	1000	McF_4	0	unif	singleC	DiP, DiP-A, OT, OT-A
749	No	7	7-B	1000	McF_4	0	unif	wholeT_0.01	DiP, DiP-A, OT, OT-A
750	No	7	7-B	1000	McF_4	0	unif	wholeT_0.5	DiP, DiP-A, OT, OT-A
751	No	7	7-B	1000	McF_4	Inf	last	singleC	DiP, DiP-A, OT, OT-A
752	No	7	7-B	1000	McF_4	Inf	last	wholeT_0.01	DiP, DiP-A, OT, OT-A
753	No	7	7-B	1000	McF_4	Inf	last	wholeT_0.5	DiP, DiP-A, OT, OT-A
754	No	7	7-B	1000	McF_4	Inf	unif	singleC	DiP, DiP-A, OT, OT-A
755	No	7	7-B	1000	McF_4	Inf	unif	wholeT_0.01	DiP, DiP-A, OT, OT-A
756	No	7	7-B	1000	McF_4	Inf	unif	wholeT_0.5	DiP, DiP-A, OT, OT-A
757	No	7	7-B	1000	McF_6	0	last	singleC	DiP, DiP-A, OT, OT-A

Table 4: *(continued)*

	Conjunction	Drivers	Tree	S.Size	Model	sh	S.Time	S.Type	Best method(s)
758	No	7	7-B	1000	McF_6	0	last	wholeT_0.01	DiP, DiP-A, OT, OT-A
759	No	7	7-B	1000	McF_6	0	last	wholeT_0.5	DiP, DiP-A, OT, OT-A
760	No	7	7-B	1000	McF_6	0	unif	singleC	CBN, CBN-A, DiP, DiP-A, OT, OT-A
761	No	7	7-B	1000	McF_6	0	unif	wholeT_0.01	CBN, CBN-A, DiP, DiP-A, OT, OT-A
762	No	7	7-B	1000	McF_6	0	unif	wholeT_0.5	CBN, CBN-A, DiP, DiP-A, OT, OT-A
763	No	7	7-B	1000	McF_6	Inf	last	singleC	DiP, DiP-A, OT, OT-A
764	No	7	7-B	1000	McF_6	Inf	last	wholeT_0.01	DiP, DiP-A, OT, OT-A
765	No	7	7-B	1000	McF_6	Inf	last	wholeT_0.5	DiP, DiP-A, OT, OT-A
766	No	7	7-B	1000	McF_6	Inf	unif	singleC	CBN, CBN-A, DiP, DiP-A, OT, OT-A
767	No	7	7-B	1000	McF_6	Inf	unif	wholeT_0.01	CBN, CBN-A, DiP, DiP-A, OT, OT-A
768	No	7	7-B	1000	McF_6	Inf	unif	wholeT_0.5	CBN, CBN-A, DiP, DiP-A, OT, OT-A
769	No	7	7-B	200	Bozic	0	last	singleC	DiP, DiP-A, OT, OT-A
770	No	7	7-B	200	Bozic	0	last	wholeT_0.01	DiP, DiP-A, OT, OT-A
771	No	7	7-B	200	Bozic	0	last	wholeT_0.5	DiP, DiP-A, OT, OT-A
772	No	7	7-B	200	Bozic	0	unif	singleC	DiP, DiP-A, OT, OT-A
773	No	7	7-B	200	Bozic	0	unif	wholeT_0.01	DiP, DiP-A
774	No	7	7-B	200	Bozic	0	unif	wholeT_0.5	DiP, DiP-A, OT, OT-A
775	No	7	7-B	200	Bozic	Inf	last	singleC	DiP, DiP-A, OT, OT-A
776	No	7	7-B	200	Bozic	Inf	last	wholeT_0.01	CBN, CBN-A, OT, OT-A
777	No	7	7-B	200	Bozic	Inf	last	wholeT_0.5	DiP, DiP-A, OT, OT-A
778	No	7	7-B	200	Bozic	Inf	unif	singleC	DiP, DiP-A
779	No	7	7-B	200	Bozic	Inf	unif	wholeT_0.01	DiP, DiP-A, OT, OT-A
780	No	7	7-B	200	Bozic	Inf	unif	wholeT_0.5	DiP, DiP-A
781	No	7	7-B	200	exp	0	last	singleC	DiP, DiP-A, OT, OT-A
782	No	7	7-B	200	exp	0	last	wholeT_0.01	DiP, DiP-A
783	No	7	7-B	200	exp	0	last	wholeT_0.5	DiP, DiP-A, OT, OT-A
784	No	7	7-B	200	exp	0	unif	singleC	DiP, DiP-A, OT, OT-A
785	No	7	7-B	200	exp	0	unif	wholeT_0.01	DiP, DiP-A
786	No	7	7-B	200	exp	0	unif	wholeT_0.5	DiP, DiP-A, OT, OT-A
787	No	7	7-B	200	exp	Inf	last	singleC	DiP, DiP-A, OT, OT-A
788	No	7	7-B	200	exp	Inf	last	wholeT_0.01	DiP
789	No	7	7-B	200	exp	Inf	last	wholeT_0.5	DiP, DiP-A, OT, OT-A
790	No	7	7-B	200	exp	Inf	unif	singleC	DiP, DiP-A
791	No	7	7-B	200	exp	Inf	unif	wholeT_0.01	DiP, DiP-A
792	No	7	7-B	200	exp	Inf	unif	wholeT_0.5	DiP, DiP-A
793	No	7	7-B	200	McF_4	0	last	singleC	DiP, DiP-A, OT, OT-A
794	No	7	7-B	200	McF_4	0	last	wholeT_0.01	DiP, DiP-A, OT, OT-A
795	No	7	7-B	200	McF_4	0	last	wholeT_0.5	DiP, DiP-A, OT, OT-A
796	No	7	7-B	200	McF_4	0	unif	singleC	DiP, DiP-A, OT, OT-A
797	No	7	7-B	200	McF_4	0	unif	wholeT_0.01	DiP, DiP-A, OT, OT-A

Table 4: *(continued)*

	Conjunction	Drivers	Tree	S.Size	Model	sh	S.Time	S.Type	Best method(s)
798	No	7	7-B	200	McF_4	0	unif	wholeT_0.5	DiP, DiP-A, OT, OT-A
799	No	7	7-B	200	McF_4	Inf	last	singleC	DiP, DiP-A, OT, OT-A
800	No	7	7-B	200	McF_4	Inf	last	wholeT_0.01	DiP, DiP-A, OT, OT-A
801	No	7	7-B	200	McF_4	Inf	last	wholeT_0.5	DiP, DiP-A, OT, OT-A
802	No	7	7-B	200	McF_4	Inf	unif	singleC	DiP, DiP-A, OT, OT-A
803	No	7	7-B	200	McF_4	Inf	unif	wholeT_0.01	DiP, DiP-A, OT, OT-A
804	No	7	7-B	200	McF_4	Inf	unif	wholeT_0.5	DiP, DiP-A, OT, OT-A
805	No	7	7-B	200	McF_6	0	last	singleC	DiP, DiP-A, OT, OT-A
806	No	7	7-B	200	McF_6	0	last	wholeT_0.01	DiP, DiP-A, OT, OT-A
807	No	7	7-B	200	McF_6	0	last	wholeT_0.5	DiP, DiP-A, OT, OT-A
808	No	7	7-B	200	McF_6	0	unif	singleC	CBN, CBN-A, DiP, DiP-A, OT, OT-A
809	No	7	7-B	200	McF_6	0	unif	wholeT_0.01	CBN, CBN-A, DiP, DiP-A, OT, OT-A
810	No	7	7-B	200	McF_6	0	unif	wholeT_0.5	CBN, CBN-A, DiP, OT, OT-A
811	No	7	7-B	200	McF_6	Inf	last	singleC	DiP, DiP-A, OT, OT-A
812	No	7	7-B	200	McF_6	Inf	last	wholeT_0.01	DiP, OT, OT-A
813	No	7	7-B	200	McF_6	Inf	last	wholeT_0.5	DiP, DiP-A, OT, OT-A
814	No	7	7-B	200	McF_6	Inf	unif	singleC	CBN, CBN-A
815	No	7	7-B	200	McF_6	Inf	unif	wholeT_0.01	CBN, CBN-A, DiP, DiP-A
816	No	7	7-B	200	McF_6	Inf	unif	wholeT_0.5	CBN, CBN-A, DiP
817	No	7	7-B	100	Bozic	0	last	singleC	DiP, DiP-A, OT, OT-A
818	No	7	7-B	100	Bozic	0	last	wholeT_0.01	DiP, DiP-A, OT, OT-A
819	No	7	7-B	100	Bozic	0	last	wholeT_0.5	DiP, DiP-A, OT, OT-A
820	No	7	7-B	100	Bozic	0	unif	singleC	DiP, DiP-A, OT, OT-A
821	No	7	7-B	100	Bozic	0	unif	wholeT_0.01	DiP, DiP-A
822	No	7	7-B	100	Bozic	0	unif	wholeT_0.5	DiP, DiP-A, OT, OT-A
823	No	7	7-B	100	Bozic	Inf	last	singleC	DiP, DiP-A, OT, OT-A
824	No	7	7-B	100	Bozic	Inf	last	wholeT_0.01	CBN, CBN-A, DiP
825	No	7	7-B	100	Bozic	Inf	last	wholeT_0.5	DiP, DiP-A, OT, OT-A
826	No	7	7-B	100	Bozic	Inf	unif	singleC	DiP, DiP-A
827	No	7	7-B	100	Bozic	Inf	unif	wholeT_0.01	DiP, DiP-A
828	No	7	7-B	100	Bozic	Inf	unif	wholeT_0.5	DiP, DiP-A
829	No	7	7-B	100	exp	0	last	singleC	DiP, DiP-A, OT, OT-A
830	No	7	7-B	100	exp	0	last	wholeT_0.01	DiP, DiP-A
831	No	7	7-B	100	exp	0	last	wholeT_0.5	DiP, DiP-A, OT, OT-A
832	No	7	7-B	100	exp	0	unif	singleC	DiP, DiP-A, OT, OT-A
833	No	7	7-B	100	exp	0	unif	wholeT_0.01	DiP, DiP-A, OT, OT-A
834	No	7	7-B	100	exp	0	unif	wholeT_0.5	DiP, DiP-A, OT, OT-A
835	No	7	7-B	100	exp	Inf	last	singleC	DiP, DiP-A, OT, OT-A
836	No	7	7-B	100	exp	Inf	last	wholeT_0.01	DiP, DiP-A
837	No	7	7-B	100	exp	Inf	last	wholeT_0.5	DiP, DiP-A, OT, OT-A

Table 4: *(continued)*

	Conjunction	Drivers	Tree	S.Size	Model	sh	S.Time	S.Type	Best method(s)
838	No	7	7-B	100	exp	Inf	unif	singleC	DiP, DiP-A
839	No	7	7-B	100	exp	Inf	unif	wholeT_0.01	DiP, DiP-A, OT, OT-A
840	No	7	7-B	100	exp	Inf	unif	wholeT_0.5	DiP, DiP-A
841	No	7	7-B	100	McF_4	0	last	singleC	DiP, DiP-A, OT, OT-A
842	No	7	7-B	100	McF_4	0	last	wholeT_0.01	DiP, DiP-A, OT, OT-A
843	No	7	7-B	100	McF_4	0	last	wholeT_0.5	DiP, DiP-A, OT, OT-A
844	No	7	7-B	100	McF_4	0	unif	singleC	DiP, DiP-A
845	No	7	7-B	100	McF_4	0	unif	wholeT_0.01	DiP, DiP-A
846	No	7	7-B	100	McF_4	0	unif	wholeT_0.5	DiP, DiP-A, OT, OT-A
847	No	7	7-B	100	McF_4	Inf	last	singleC	DiP, DiP-A, OT, OT-A
848	No	7	7-B	100	McF_4	Inf	last	wholeT_0.01	DiP, DiP-A, OT, OT-A
849	No	7	7-B	100	McF_4	Inf	last	wholeT_0.5	DiP, DiP-A, OT, OT-A
850	No	7	7-B	100	McF_4	Inf	unif	singleC	DiP, DiP-A, OT, OT-A
851	No	7	7-B	100	McF_4	Inf	unif	wholeT_0.01	DiP, DiP-A
852	No	7	7-B	100	McF_4	Inf	unif	wholeT_0.5	DiP, DiP-A
853	No	7	7-B	100	McF_6	0	last	singleC	DiP, DiP-A, OT, OT-A
854	No	7	7-B	100	McF_6	0	last	wholeT_0.01	DiP, OT, OT-A
855	No	7	7-B	100	McF_6	0	last	wholeT_0.5	DiP, DiP-A, OT, OT-A
856	No	7	7-B	100	McF_6	0	unif	singleC	CBN, CBN-A, DiP, OT, OT-A
857	No	7	7-B	100	McF_6	0	unif	wholeT_0.01	CBN, CBN-A, DiP, DiP-A
858	No	7	7-B	100	McF_6	0	unif	wholeT_0.5	CBN, CBN-A
859	No	7	7-B	100	McF_6	Inf	last	singleC	DiP, DiP-A, OT, OT-A
860	No	7	7-B	100	McF_6	Inf	last	wholeT_0.01	DiP, OT, OT-A
861	No	7	7-B	100	McF_6	Inf	last	wholeT_0.5	DiP, DiP-A, OT, OT-A
862	No	7	7-B	100	McF_6	Inf	unif	singleC	CBN, CBN-A
863	No	7	7-B	100	McF_6	Inf	unif	wholeT_0.01	CBN, CBN-A
864	No	7	7-B	100	McF_6	Inf	unif	wholeT_0.5	CBN, CBN-A

3 Drivers Unknown (MCB-2)

3.1 Confidence sets (MCB-2), Diff, Drivers Unknown

Table 5: Confidence sets (method MCB-2) when Drivers are Unknown for measure Diff.

	Conjunction	Drivers	Tree	S.Size	Model	sh	S.Time	S.Type	Best method(s)
1	Yes	11	11-A	1000	Bozic	0	last	singleC	S1:OT, S1:OT-A
2	Yes	11	11-A	1000	Bozic	0	last	wholeT_0.01	S1:DiP, S1:DiP-A, S5:DiP, S5:DiP-A
3	Yes	11	11-A	1000	Bozic	0	last	wholeT_0.5	S1:OT, S1:OT-A
4	Yes	11	11-A	1000	Bozic	0	unif	singleC	S1:DiP, S1:DiP-A, S1:OT, S1:OT-A
5	Yes	11	11-A	1000	Bozic	0	unif	wholeT_0.01	J1:CBN-A, J1:DiP, J1:DiP-A, S5:CBN, S5:CBN-A, S5:DiP, S5:DiP-A, S5:OT, S5:OT-A
6	Yes	11	11-A	1000	Bozic	0	unif	wholeT_0.5	S1:DiP, S1:DiP-A, S1:OT, S1:OT-A, S5:DiP, S5:DiP-A, S5:OT, S5:OT-A
7	Yes	11	11-A	1000	Bozic	Inf	last	singleC	S5:DiP, S5:DiP-A, S5:OT, S5:OT-A
8	Yes	11	11-A	1000	Bozic	Inf	last	wholeT_0.01	J1:DiP, J1:DiP-A, J1:OT, J1:OT-A, J5:DiP, J5:DiP-A, J5:OT, J5:OT-A
9	Yes	11	11-A	1000	Bozic	Inf	last	wholeT_0.5	S5:DiP, S5:DiP-A, S5:OT, S5:OT-A
10	Yes	11	11-A	1000	Bozic	Inf	unif	singleC	S1:CBN, S1:CBN-A, S1:DiP, S1:DiP-A, S1:OT, S1:OT-A
11	Yes	11	11-A	1000	Bozic	Inf	unif	wholeT_0.01	J1:CBN-A, S5:CBN-A
12	Yes	11	11-A	1000	Bozic	Inf	unif	wholeT_0.5	J1:DiP, J1:DiP-A, J1:OT, J1:OT-A, S1:CBN, S1:CBN-A, S1:OT, S1:OT-A, S5:DiP, S5:DiP-A, S5:OT, S5:OT-A
13	Yes	11	11-A	1000	exp	0	last	singleC	S1:OT, S1:OT-A
14	Yes	11	11-A	1000	exp	0	last	wholeT_0.01	S1:DiP-A, S1:OT, S1:OT-A, S5:DiP-A, S5:OT, S5:OT-A
15	Yes	11	11-A	1000	exp	0	last	wholeT_0.5	S1:OT, S1:OT-A
16	Yes	11	11-A	1000	exp	0	unif	singleC	S1:DiP, S1:DiP-A, S1:OT, S1:OT-A, S5:DiP, S5:DiP-A, S5:OT, S5:OT-A
17	Yes	11	11-A	1000	exp	0	unif	wholeT_0.01	S1:CBN-A, S1:DiP, S1:DiP-A, S1:OT, S1:OT-A
18	Yes	11	11-A	1000	exp	0	unif	wholeT_0.5	S1:DiP, S1:DiP-A, S1:OT, S1:OT-A, S5:DiP, S5:DiP-A, S5:OT, S5:OT-A
19	Yes	11	11-A	1000	exp	Inf	last	singleC	S5:DiP, S5:DiP-A, S5:OT, S5:OT-A
20	Yes	11	11-A	1000	exp	Inf	last	wholeT_0.01	S5:DiP, S5:DiP-A, S5:OT, S5:OT-A
21	Yes	11	11-A	1000	exp	Inf	last	wholeT_0.5	S5:DiP, S5:DiP-A, S5:OT, S5:OT-A
22	Yes	11	11-A	1000	exp	Inf	unif	singleC	S1:CBN, S1:CBN-A, S1:OT, S1:OT-A
23	Yes	11	11-A	1000	exp	Inf	unif	wholeT_0.01	J1:CBN-A, J1:OT, J1:OT-A, S5:DiP, S5:DiP-A, S5:OT, S5:OT-A
24	Yes	11	11-A	1000	exp	Inf	unif	wholeT_0.5	S1:CBN, S1:CBN-A, S1:OT, S1:OT-A
25	Yes	11	11-A	1000	McF_4	0	last	singleC	S1:DiP-A, S1:OT, S1:OT-A

Table 5: *(continued)*

	Conjunction	Drivers	Tree	S.Size	Model	sh	S.Time	S.Type	Best method(s)
26	Yes	11	11-A	1000	McF_4	0	last	wholeT_0.01	S1:DiP-A, S1:OT, S1:OT-A, S5:DiP, S5:DiP-A, S5:OT, S5:OT-A
27	Yes	11	11-A	1000	McF_4	0	last	wholeT_0.5	S1:DiP-A, S1:OT, S1:OT-A
28	Yes	11	11-A	1000	McF_4	0	unif	singleC	S1:DiP, S1:DiP-A, S1:OT, S1:OT-A
29	Yes	11	11-A	1000	McF_4	0	unif	wholeT_0.01	S1:OT, S1:OT-A, S5:DiP, S5:DiP-A, S5:OT, S5:OT-A
30	Yes	11	11-A	1000	McF_4	0	unif	wholeT_0.5	S1:DiP, S1:DiP-A, S1:OT, S1:OT-A, S5:DiP, S5:DiP-A, S5:OT, S5:OT-A
31	Yes	11	11-A	1000	McF_4	Inf	last	singleC	S1:OT, S1:OT-A
32	Yes	11	11-A	1000	McF_4	Inf	last	wholeT_0.01	S5:DiP, S5:DiP-A, S5:OT, S5:OT-A
33	Yes	11	11-A	1000	McF_4	Inf	last	wholeT_0.5	S1:DiP-A, S1:OT, S1:OT-A
34	Yes	11	11-A	1000	McF_4	Inf	unif	singleC	S1:OT, S1:OT-A
35	Yes	11	11-A	1000	McF_4	Inf	unif	wholeT_0.01	S5:DiP, S5:DiP-A, S5:OT, S5:OT-A
36	Yes	11	11-A	1000	McF_4	Inf	unif	wholeT_0.5	S1:OT, S1:OT-A
37	Yes	11	11-A	1000	McF_6	0	last	singleC	S1:DiP, S1:OT, S5:DiP
38	Yes	11	11-A	1000	McF_6	0	last	wholeT_0.01	S5:DiP
39	Yes	11	11-A	1000	McF_6	0	last	wholeT_0.5	S1:DiP, S5:DiP
40	Yes	11	11-A	1000	McF_6	0	unif	singleC	S1:DiP, S1:DiP-A, S1:OT, S1:OT-A
41	Yes	11	11-A	1000	McF_6	0	unif	wholeT_0.01	S1:DiP, S1:DiP-A, S1:OT, S1:OT-A
42	Yes	11	11-A	1000	McF_6	0	unif	wholeT_0.5	S1:DiP, S1:DiP-A
43	Yes	11	11-A	1000	McF_6	Inf	last	singleC	S1:OT
44	Yes	11	11-A	1000	McF_6	Inf	last	wholeT_0.01	S5:OT
45	Yes	11	11-A	1000	McF_6	Inf	last	wholeT_0.5	S1:DiP, S1:OT, S5:OT
46	Yes	11	11-A	1000	McF_6	Inf	unif	singleC	S1:DiP, S1:DiP-A
47	Yes	11	11-A	1000	McF_6	Inf	unif	wholeT_0.01	S1:DiP, S1:DiP-A, S5:DiP, S5:DiP-A, S5:OT, S5:OT-A
48	Yes	11	11-A	1000	McF_6	Inf	unif	wholeT_0.5	S1:DiP, S1:DiP-A
49	Yes	11	11-A	200	Bozic	0	last	singleC	S1:OT, S1:OT-A
50	Yes	11	11-A	200	Bozic	0	last	wholeT_0.01	J1:OT, J1:OT-A, J5:OT, J5:OT-A, S5:OT, S5:OT-A
51	Yes	11	11-A	200	Bozic	0	last	wholeT_0.5	S1:OT, S1:OT-A
52	Yes	11	11-A	200	Bozic	0	unif	singleC	S1:OT, S1:OT-A
53	Yes	11	11-A	200	Bozic	0	unif	wholeT_0.01	J1:CBN, J1:CBN-A, J1:DiP, J1:DiP-A, J1:OT, J1:OT-A, S5:CBN, S5:CBN-A, S5:DiP, S5:DiP-A, S5:OT, S5:OT-A
54	Yes	11	11-A	200	Bozic	0	unif	wholeT_0.5	S1:OT, S1:OT-A, S5:DiP, S5:DiP-A, S5:OT, S5:OT-A
55	Yes	11	11-A	200	Bozic	Inf	last	singleC	S5:OT, S5:OT-A
56	Yes	11	11-A	200	Bozic	Inf	last	wholeT_0.01	J5:OT, J5:OT-A
57	Yes	11	11-A	200	Bozic	Inf	last	wholeT_0.5	S5:OT, S5:OT-A

Table 5: *(continued)*

	Conjunction	Drivers	Tree	S.Size	Model	sh	S.Time	S.Type	Best method(s)
58	Yes	11	11-A	200	Bozic	Inf	unif	singleC	J1:CBN, J1:CBN-A, J1:OT, J1:OT-A, S5:OT, S5:OT-A
59	Yes	11	11-A	200	Bozic	Inf	unif	wholeT_0.01	J5:OT, J5:OT-A, S5:CBN, S5:CBN-A, S5:OT, S5:OT-A
60	Yes	11	11-A	200	Bozic	Inf	unif	wholeT_0.5	J1:CBN-A, J1:OT, J1:OT-A
61	Yes	11	11-A	200	exp	0	last	singleC	S1:OT, S1:OT-A
62	Yes	11	11-A	200	exp	0	last	wholeT_0.01	S1:OT, S1:OT-A, S5:OT, S5:OT-A
63	Yes	11	11-A	200	exp	0	last	wholeT_0.5	S1:OT, S1:OT-A
64	Yes	11	11-A	200	exp	0	unif	singleC	S1:DiP, S1:DiP-A, S1:OT, S1:OT-A, S5:DiP, S5:DiP-A, S5:OT, S5:OT-A
65	Yes	11	11-A	200	exp	0	unif	wholeT_0.01	S1:OT, S1:OT-A
66	Yes	11	11-A	200	exp	0	unif	wholeT_0.5	S1:DiP, S1:DiP-A, S1:OT, S1:OT-A, S5:DiP, S5:DiP-A, S5:OT, S5:OT-A
67	Yes	11	11-A	200	exp	Inf	last	singleC	S5:OT, S5:OT-A
68	Yes	11	11-A	200	exp	Inf	last	wholeT_0.01	J5:CBN-A, J5:OT, J5:OT-A
69	Yes	11	11-A	200	exp	Inf	last	wholeT_0.5	S5:OT, S5:OT-A
70	Yes	11	11-A	200	exp	Inf	unif	singleC	S1:OT, S1:OT-A, S5:DiP, S5:DiP-A, S5:OT, S5:OT-A
71	Yes	11	11-A	200	exp	Inf	unif	wholeT_0.01	S5:CBN-A, S5:OT, S5:OT-A
72	Yes	11	11-A	200	exp	Inf	unif	wholeT_0.5	S1:OT, S1:OT-A
73	Yes	11	11-A	200	McF_4	0	last	singleC	S1:OT, S1:OT-A, S5:OT, S5:OT-A
74	Yes	11	11-A	200	McF_4	0	last	wholeT_0.01	S1:OT, S1:OT-A, S5:OT, S5:OT-A
75	Yes	11	11-A	200	McF_4	0	last	wholeT_0.5	S1:OT, S1:OT-A, S5:OT, S5:OT-A
76	Yes	11	11-A	200	McF_4	0	unif	singleC	S5:CBN-A, S5:OT, S5:OT-A
77	Yes	11	11-A	200	McF_4	0	unif	wholeT_0.01	S5:CBN, S5:OT, S5:OT-A
78	Yes	11	11-A	200	McF_4	0	unif	wholeT_0.5	S5:OT, S5:OT-A
79	Yes	11	11-A	200	McF_4	Inf	last	singleC	S1:OT, S1:OT-A, S5:OT, S5:OT-A
80	Yes	11	11-A	200	McF_4	Inf	last	wholeT_0.01	S5:OT, S5:OT-A
81	Yes	11	11-A	200	McF_4	Inf	last	wholeT_0.5	S1:OT, S1:OT-A, S5:OT, S5:OT-A
82	Yes	11	11-A	200	McF_4	Inf	unif	singleC	S5:CBN-A, S5:OT, S5:OT-A
83	Yes	11	11-A	200	McF_4	Inf	unif	wholeT_0.01	S5:CBN-A, S5:OT, S5:OT-A
84	Yes	11	11-A	200	McF_4	Inf	unif	wholeT_0.5	S1:OT, S1:OT-A, S5:CBN-A, S5:OT, S5:OT-A
85	Yes	11	11-A	200	McF_6	0	last	singleC	S5:OT
86	Yes	11	11-A	200	McF_6	0	last	wholeT_0.01	S5:OT
87	Yes	11	11-A	200	McF_6	0	last	wholeT_0.5	S1:OT, S5:OT
88	Yes	11	11-A	200	McF_6	0	unif	singleC	J1:CBN, J1:CBN-A, S1:OT, S1:OT-A, S5:CBN, S5:CBN-A, S5:OT, S5:OT-A
89	Yes	11	11-A	200	McF_6	0	unif	wholeT_0.01	S1:OT, S1:OT-A, S5:CBN, S5:CBN-A, S5:OT, S5:OT-A

Table 5: *(continued)*

	Conjunction	Drivers	Tree	S.Size	Model	sh	S.Time	S.Type	Best method(s)
90	Yes	11	11-A	200	McF_6	0	unif	wholeT_0.5	J1:CBN, J1:CBN-A, S1:OT, S1:OT-A, S5:CBN, S5:CBN-A, S5:OT, S5:OT-A
91	Yes	11	11-A	200	McF_6	Inf	last	singleC	S1:OT, S5:OT
92	Yes	11	11-A	200	McF_6	Inf	last	wholeT_0.01	S5:OT, S5:OT-A
93	Yes	11	11-A	200	McF_6	Inf	last	wholeT_0.5	S1:OT, S5:OT
94	Yes	11	11-A	200	McF_6	Inf	unif	singleC	J1:CBN-A, S1:OT, S1:OT-A, S5:CBN, S5:CBN-A, S5:OT, S5:OT-A
95	Yes	11	11-A	200	McF_6	Inf	unif	wholeT_0.01	J1:CBN-A, S5:CBN, S5:CBN-A, S5:OT, S5:OT-A
96	Yes	11	11-A	200	McF_6	Inf	unif	wholeT_0.5	J1:CBN-A, J1:OT, J1:OT-A, S1:CBN-A, S1:OT, S1:OT-A, S5:CBN, S5:CBN-A, S5:OT, S5:OT-A
97	Yes	11	11-A	100	Bozic	0	last	singleC	S5:OT, S5:OT-A
98	Yes	11	11-A	100	Bozic	0	last	wholeT_0.01	J5:OT, J5:OT-A, S5:OT, S5:OT-A
99	Yes	11	11-A	100	Bozic	0	last	wholeT_0.5	S1:OT, S1:OT-A, S5:OT, S5:OT-A
100	Yes	11	11-A	100	Bozic	0	unif	singleC	J1:OT, J1:OT-A, S5:DiP, S5:DiP-A, S5:OT, S5:OT-A
101	Yes	11	11-A	100	Bozic	0	unif	wholeT_0.01	J1:OT, J1:OT-A, S5:OT, S5:OT-A
102	Yes	11	11-A	100	Bozic	0	unif	wholeT_0.5	S5:DiP, S5:DiP-A, S5:OT, S5:OT-A
103	Yes	11	11-A	100	Bozic	Inf	last	singleC	J1:OT, J1:OT-A, J5:OT, J5:OT-A, S5:OT, S5:OT-A
104	Yes	11	11-A	100	Bozic	Inf	last	wholeT_0.01	J5:CBN-A, J5:OT, J5:OT-A
105	Yes	11	11-A	100	Bozic	Inf	last	wholeT_0.5	J1:OT, J1:OT-A, J5:OT, J5:OT-A, S5:OT, S5:OT-A
106	Yes	11	11-A	100	Bozic	Inf	unif	singleC	S5:OT, S5:OT-A
107	Yes	11	11-A	100	Bozic	Inf	unif	wholeT_0.01	J5:OT, J5:OT-A, S5:OT, S5:OT-A
108	Yes	11	11-A	100	Bozic	Inf	unif	wholeT_0.5	S5:DiP, S5:DiP-A, S5:OT, S5:OT-A
109	Yes	11	11-A	100	exp	0	last	singleC	S1:OT, S1:OT-A
110	Yes	11	11-A	100	exp	0	last	wholeT_0.01	S1:OT, S1:OT-A, S5:OT, S5:OT-A
111	Yes	11	11-A	100	exp	0	last	wholeT_0.5	S1:OT, S1:OT-A
112	Yes	11	11-A	100	exp	0	unif	singleC	S1:OT, S1:OT-A, S5:DiP, S5:DiP-A, S5:OT, S5:OT-A
113	Yes	11	11-A	100	exp	0	unif	wholeT_0.01	J1:OT, J1:OT-A, S1:OT, S1:OT-A, S5:DiP, S5:DiP-A, S5:OT, S5:OT-A
114	Yes	11	11-A	100	exp	0	unif	wholeT_0.5	S1:OT, S1:OT-A, S5:DiP, S5:DiP-A, S5:OT, S5:OT-A
115	Yes	11	11-A	100	exp	Inf	last	singleC	S5:OT, S5:OT-A
116	Yes	11	11-A	100	exp	Inf	last	wholeT_0.01	J5:CBN-A, J5:OT, J5:OT-A
117	Yes	11	11-A	100	exp	Inf	last	wholeT_0.5	S5:OT, S5:OT-A

Table 5: *(continued)*

	Conjunction	Drivers	Tree	S.Size	Model	sh	S.Time	S.Type	Best method(s)
118	Yes	11	11-A	100	exp	Inf	unif	singleC	J1:CBN, J1:CBN-A, J1:OT, J1:OT-A, S5:DiP, S5:DiP-A, S5:OT, S5:OT-A
119	Yes	11	11-A	100	exp	Inf	unif	wholeT_0.01	J5:OT, J5:OT-A, S5:OT, S5:OT-A
120	Yes	11	11-A	100	exp	Inf	unif	wholeT_0.5	J1:OT, J1:OT-A, S5:DiP, S5:DiP-A, S5:OT, S5:OT-A
121	Yes	11	11-A	100	McF_4	0	last	singleC	S5:OT, S5:OT-A
122	Yes	11	11-A	100	McF_4	0	last	wholeT_0.01	S5:OT, S5:OT-A
123	Yes	11	11-A	100	McF_4	0	last	wholeT_0.5	S5:OT, S5:OT-A
124	Yes	11	11-A	100	McF_4	0	unif	singleC	S5:CBN-A, S5:OT, S5:OT-A
125	Yes	11	11-A	100	McF_4	0	unif	wholeT_0.01	S5:CBN-A, S5:OT, S5:OT-A
126	Yes	11	11-A	100	McF_4	0	unif	wholeT_0.5	S5:CBN, S5:CBN-A, S5:OT, S5:OT-A
127	Yes	11	11-A	100	McF_4	Inf	last	singleC	S1:OT, S1:OT-A, S5:OT, S5:OT-A
128	Yes	11	11-A	100	McF_4	Inf	last	wholeT_0.01	S5:OT, S5:OT-A
129	Yes	11	11-A	100	McF_4	Inf	last	wholeT_0.5	S5:OT, S5:OT-A
130	Yes	11	11-A	100	McF_4	Inf	unif	singleC	S5:CBN-A, S5:OT, S5:OT-A
131	Yes	11	11-A	100	McF_4	Inf	unif	wholeT_0.01	S5:CBN-A, S5:OT, S5:OT-A
132	Yes	11	11-A	100	McF_4	Inf	unif	wholeT_0.5	S5:CBN, S5:CBN-A, S5:OT, S5:OT-A
133	Yes	11	11-A	100	McF_6	0	last	singleC	S1:OT, S5:OT
134	Yes	11	11-A	100	McF_6	0	last	wholeT_0.01	S5:OT, S5:OT-A
135	Yes	11	11-A	100	McF_6	0	last	wholeT_0.5	S1:OT, S5:OT
136	Yes	11	11-A	100	McF_6	0	unif	singleC	J1:CBN-A, S5:CBN, S5:CBN-A, S5:OT, S5:OT-A
137	Yes	11	11-A	100	McF_6	0	unif	wholeT_0.01	S5:CBN, S5:OT, S5:OT-A
138	Yes	11	11-A	100	McF_6	0	unif	wholeT_0.5	J1:CBN, J1:CBN-A, S1:OT, S1:OT-A, S5:CBN, S5:CBN-A, S5:OT, S5:OT-A
139	Yes	11	11-A	100	McF_6	Inf	last	singleC	S5:OT
140	Yes	11	11-A	100	McF_6	Inf	last	wholeT_0.01	S5:OT, S5:OT-A
141	Yes	11	11-A	100	McF_6	Inf	last	wholeT_0.5	S5:OT
142	Yes	11	11-A	100	McF_6	Inf	unif	singleC	J1:CBN, J1:CBN-A, S1:OT, S1:OT-A, S5:CBN, S5:CBN-A, S5:OT, S5:OT-A
143	Yes	11	11-A	100	McF_6	Inf	unif	wholeT_0.01	S5:CBN, S5:CBN-A, S5:OT, S5:OT-A
144	Yes	11	11-A	100	McF_6	Inf	unif	wholeT_0.5	J1:CBN-A, S1:OT, S1:OT-A, S5:CBN, S5:CBN-A, S5:OT, S5:OT-A
145	Yes	9	9-A	1000	Bozic	0	last	singleC	S1:OT, S1:OT-A
146	Yes	9	9-A	1000	Bozic	0	last	wholeT_0.01	S1:DiP, S1:DiP-A, S1:OT, S1:OT-A, S5:DiP, S5:DiP-A, S5:OT, S5:OT-A
147	Yes	9	9-A	1000	Bozic	0	last	wholeT_0.5	S1:OT, S1:OT-A
148	Yes	9	9-A	1000	Bozic	0	unif	singleC	S1:DiP, S1:DiP-A, S1:OT, S1:OT-A, S5:DiP, S5:DiP-A, S5:OT, S5:OT-A

Table 5: *(continued)*

	Conjunction	Drivers	Tree	S.Size	Model	sh	S.Time	S.Type	Best method(s)
149	Yes	9	9-A	1000	Bozic	0	unif	wholeT_0.01	J1:DiP, J1:DiP-A, J1:OT, J1:OT-A, S1:DiP, S1:DiP-A, S1:OT, S1:OT-A
150	Yes	9	9-A	1000	Bozic	0	unif	wholeT_0.5	S1:DiP, S1:DiP-A, S1:OT, S1:OT-A, S5:DiP, S5:DiP-A, S5:OT, S5:OT-A
151	Yes	9	9-A	1000	Bozic	Inf	last	singleC	S5:OT, S5:OT-A
152	Yes	9	9-A	1000	Bozic	Inf	last	wholeT_0.01	J5:DiP, J5:DiP-A, J5:OT, J5:OT-A
153	Yes	9	9-A	1000	Bozic	Inf	last	wholeT_0.5	S5:DiP, S5:DiP-A, S5:OT, S5:OT-A
154	Yes	9	9-A	1000	Bozic	Inf	unif	singleC	S1:OT, S1:OT-A
155	Yes	9	9-A	1000	Bozic	Inf	unif	wholeT_0.01	S5:DiP, S5:DiP-A, S5:OT, S5:OT-A
156	Yes	9	9-A	1000	Bozic	Inf	unif	wholeT_0.5	S1:CBN, S1:OT, S1:OT-A
157	Yes	9	9-A	1000	exp	0	last	singleC	S1:DiP, S1:DiP-A, S1:OT, S1:OT-A
158	Yes	9	9-A	1000	exp	0	last	wholeT_0.01	S1:DiP-A, S1:OT, S1:OT-A
159	Yes	9	9-A	1000	exp	0	last	wholeT_0.5	S1:DiP, S1:DiP-A, S1:OT, S1:OT-A, S5:DiP, S5:DiP-A, S5:OT, S5:OT-A
160	Yes	9	9-A	1000	exp	0	unif	singleC	S1:DiP, S1:DiP-A, S1:OT, S1:OT-A, S5:DiP, S5:DiP-A, S5:OT, S5:OT-A
161	Yes	9	9-A	1000	exp	0	unif	wholeT_0.01	S1:DiP, S1:DiP-A, S1:OT, S1:OT-A
162	Yes	9	9-A	1000	exp	0	unif	wholeT_0.5	S1:DiP, S1:DiP-A, S1:OT, S1:OT-A, S5:DiP, S5:DiP-A, S5:OT, S5:OT-A
163	Yes	9	9-A	1000	exp	Inf	last	singleC	S5:DiP, S5:DiP-A, S5:OT, S5:OT-A
164	Yes	9	9-A	1000	exp	Inf	last	wholeT_0.01	J1:OT, J1:OT-A, J5:DiP, J5:DiP-A, J5:OT, J5:OT-A, S5:CBN-A
165	Yes	9	9-A	1000	exp	Inf	last	wholeT_0.5	S5:DiP, S5:DiP-A, S5:OT, S5:OT-A
166	Yes	9	9-A	1000	exp	Inf	unif	singleC	S1:OT, S1:OT-A
167	Yes	9	9-A	1000	exp	Inf	unif	wholeT_0.01	J1:DiP, J1:DiP-A, J1:OT, J1:OT-A
168	Yes	9	9-A	1000	exp	Inf	unif	wholeT_0.5	S1:OT, S1:OT-A
169	Yes	9	9-A	1000	McF_4	0	last	singleC	S1:DiP, S1:DiP-A, S1:OT, S1:OT-A, S5:DiP
170	Yes	9	9-A	1000	McF_4	0	last	wholeT_0.01	S5:DiP, S5:DiP-A
171	Yes	9	9-A	1000	McF_4	0	last	wholeT_0.5	S1:DiP, S1:DiP-A, S1:OT, S1:OT-A
172	Yes	9	9-A	1000	McF_4	0	unif	singleC	S1:DiP, S1:DiP-A, S1:OT, S1:OT-A
173	Yes	9	9-A	1000	McF_4	0	unif	wholeT_0.01	S5:CBN-A, S5:DiP, S5:DiP-A, S5:OT, S5:OT-A
174	Yes	9	9-A	1000	McF_4	0	unif	wholeT_0.5	S1:DiP, S1:DiP-A, S1:OT, S1:OT-A
175	Yes	9	9-A	1000	McF_4	Inf	last	singleC	S1:OT, S1:OT-A
176	Yes	9	9-A	1000	McF_4	Inf	last	wholeT_0.01	S5:OT
177	Yes	9	9-A	1000	McF_4	Inf	last	wholeT_0.5	S1:DiP, S1:DiP-A, S1:OT, S1:OT-A
178	Yes	9	9-A	1000	McF_4	Inf	unif	singleC	S1:OT, S1:OT-A
179	Yes	9	9-A	1000	McF_4	Inf	unif	wholeT_0.01	S5:CBN-A, S5:DiP, S5:DiP-A, S5:OT, S5:OT-A

Table 5: *(continued)*

	Conjunction	Drivers	Tree	S.Size	Model	sh	S.Time	S.Type	Best method(s)
180	Yes	9	9-A	1000	McF_4	Inf	unif	wholeT_0.5	S1:OT, S1:OT-A
181	Yes	9	9-A	1000	McF_6	0	last	singleC	S1:OT, S5:OT
182	Yes	9	9-A	1000	McF_6	0	last	wholeT_0.01	S5:DiP, S5:OT
183	Yes	9	9-A	1000	McF_6	0	last	wholeT_0.5	S1:OT, S5:OT
184	Yes	9	9-A	1000	McF_6	0	unif	singleC	S1:DiP, S1:DiP-A
185	Yes	9	9-A	1000	McF_6	0	unif	wholeT_0.01	S5:CBN, S5:CBN-A, S5:DiP, S5:DiP-A
186	Yes	9	9-A	1000	McF_6	0	unif	wholeT_0.5	S1:CBN-A, S1:DiP, S1:DiP-A
187	Yes	9	9-A	1000	McF_6	Inf	last	singleC	S5:DiP
188	Yes	9	9-A	1000	McF_6	Inf	last	wholeT_0.01	S5:OT
189	Yes	9	9-A	1000	McF_6	Inf	last	wholeT_0.5	S5:DiP
190	Yes	9	9-A	1000	McF_6	Inf	unif	singleC	S1:CBN, S1:CBN-A, S1:DiP, S1:DiP-A, S1:OT, S1:OT-A, S5:CBN, S5:CBN-A
191	Yes	9	9-A	1000	McF_6	Inf	unif	wholeT_0.01	J1:CBN, J1:CBN-A, S5:CBN, S5:CBN-A
192	Yes	9	9-A	1000	McF_6	Inf	unif	wholeT_0.5	S1:CBN, S1:CBN-A, S1:DiP, S1:DiP-A, S1:OT, S1:OT-A, S5:CBN, S5:CBN-A
193	Yes	9	9-A	200	Bozic	0	last	singleC	S1:OT, S1:OT-A
194	Yes	9	9-A	200	Bozic	0	last	wholeT_0.01	S5:OT, S5:OT-A
195	Yes	9	9-A	200	Bozic	0	last	wholeT_0.5	S1:OT, S1:OT-A
196	Yes	9	9-A	200	Bozic	0	unif	singleC	S1:OT, S1:OT-A, S5:DiP, S5:DiP-A, S5:OT, S5:OT-A
197	Yes	9	9-A	200	Bozic	0	unif	wholeT_0.01	J1:DiP, J1:DiP-A, J1:OT, J1:OT-A, S5:DiP, S5:DiP-A, S5:OT, S5:OT-A
198	Yes	9	9-A	200	Bozic	0	unif	wholeT_0.5	S1:DiP, S1:DiP-A, S1:OT, S1:OT-A, S5:DiP, S5:DiP-A, S5:OT, S5:OT-A
199	Yes	9	9-A	200	Bozic	Inf	last	singleC	S5:OT, S5:OT-A
200	Yes	9	9-A	200	Bozic	Inf	last	wholeT_0.01	J5:OT, J5:OT-A
201	Yes	9	9-A	200	Bozic	Inf	last	wholeT_0.5	S5:OT, S5:OT-A
202	Yes	9	9-A	200	Bozic	Inf	unif	singleC	J1:DiP, J1:DiP-A, J1:OT, J1:OT-A, S5:DiP, S5:DiP-A, S5:OT, S5:OT-A
203	Yes	9	9-A	200	Bozic	Inf	unif	wholeT_0.01	S5:OT, S5:OT-A
204	Yes	9	9-A	200	Bozic	Inf	unif	wholeT_0.5	J1:DiP, J1:DiP-A, J1:OT, J1:OT-A, S5:DiP, S5:DiP-A, S5:OT, S5:OT-A
205	Yes	9	9-A	200	exp	0	last	singleC	S1:OT, S1:OT-A
206	Yes	9	9-A	200	exp	0	last	wholeT_0.01	S1:OT, S1:OT-A
207	Yes	9	9-A	200	exp	0	last	wholeT_0.5	S1:OT, S1:OT-A
208	Yes	9	9-A	200	exp	0	unif	singleC	S1:DiP, S1:DiP-A, S1:OT, S1:OT-A, S5:DiP, S5:DiP-A, S5:OT, S5:OT-A
209	Yes	9	9-A	200	exp	0	unif	wholeT_0.01	S1:OT, S1:OT-A, S5:DiP, S5:DiP-A, S5:OT, S5:OT-A

Table 5: *(continued)*

	Conjunction	Drivers	Tree	S.Size	Model	sh	S.Time	S.Type	Best method(s)
210	Yes	9	9-A	200	exp	0	unif	wholeT_0.5	S1:DiP, S1:DiP-A, S1:OT, S1:OT-A, S5:DiP, S5:DiP-A, S5:OT, S5:OT-A
211	Yes	9	9-A	200	exp	Inf	last	singleC	S5:OT, S5:OT-A
212	Yes	9	9-A	200	exp	Inf	last	wholeT_0.01	J5:OT, J5:OT-A
213	Yes	9	9-A	200	exp	Inf	last	wholeT_0.5	S5:OT, S5:OT-A
214	Yes	9	9-A	200	exp	Inf	unif	singleC	J1:DiP, J1:DiP-A, J1:OT, J1:OT-A, S1:OT, S1:OT-A, S5:DiP, S5:DiP-A, S5:OT, S5:OT-A
215	Yes	9	9-A	200	exp	Inf	unif	wholeT_0.01	S5:OT, S5:OT-A
216	Yes	9	9-A	200	exp	Inf	unif	wholeT_0.5	S1:OT, S1:OT-A
217	Yes	9	9-A	200	McF_4	0	last	singleC	S5:OT, S5:OT-A
218	Yes	9	9-A	200	McF_4	0	last	wholeT_0.01	S5:OT, S5:OT-A
219	Yes	9	9-A	200	McF_4	0	last	wholeT_0.5	S5:OT, S5:OT-A
220	Yes	9	9-A	200	McF_4	0	unif	singleC	S1:OT, S1:OT-A, S5:CBN-A, S5:OT, S5:OT-A
221	Yes	9	9-A	200	McF_4	0	unif	wholeT_0.01	S5:CBN-A, S5:OT, S5:OT-A
222	Yes	9	9-A	200	McF_4	0	unif	wholeT_0.5	S1:OT, S1:OT-A, S5:OT, S5:OT-A
223	Yes	9	9-A	200	McF_4	Inf	last	singleC	S5:OT, S5:OT-A
224	Yes	9	9-A	200	McF_4	Inf	last	wholeT_0.01	S5:OT
225	Yes	9	9-A	200	McF_4	Inf	last	wholeT_0.5	S5:OT, S5:OT-A
226	Yes	9	9-A	200	McF_4	Inf	unif	singleC	S5:OT, S5:OT-A
227	Yes	9	9-A	200	McF_4	Inf	unif	wholeT_0.01	S5:OT, S5:OT-A
228	Yes	9	9-A	200	McF_4	Inf	unif	wholeT_0.5	S1:OT, S1:OT-A, S5:CBN-A, S5:OT, S5:OT-A
229	Yes	9	9-A	200	McF_6	0	last	singleC	S5:OT
230	Yes	9	9-A	200	McF_6	0	last	wholeT_0.01	S5:OT
231	Yes	9	9-A	200	McF_6	0	last	wholeT_0.5	S5:OT
232	Yes	9	9-A	200	McF_6	0	unif	singleC	S5:CBN, S5:CBN-A
233	Yes	9	9-A	200	McF_6	0	unif	wholeT_0.01	J1:CBN, J1:CBN-A, S5:CBN, S5:CBN-A, S5:OT, S5:OT-A
234	Yes	9	9-A	200	McF_6	0	unif	wholeT_0.5	J1:CBN, J1:CBN-A, S5:CBN, S5:CBN-A, S5:OT, S5:OT-A
235	Yes	9	9-A	200	McF_6	Inf	last	singleC	S5:OT
236	Yes	9	9-A	200	McF_6	Inf	last	wholeT_0.01	S5:OT
237	Yes	9	9-A	200	McF_6	Inf	last	wholeT_0.5	S5:OT
238	Yes	9	9-A	200	McF_6	Inf	unif	singleC	J1:CBN, J1:CBN-A, S5:CBN, S5:CBN-A
239	Yes	9	9-A	200	McF_6	Inf	unif	wholeT_0.01	J1:CBN, J1:CBN-A, S5:CBN, S5:CBN-A
240	Yes	9	9-A	200	McF_6	Inf	unif	wholeT_0.5	S5:CBN, S5:CBN-A
241	Yes	9	9-A	100	Bozic	0	last	singleC	J1:DiP, J1:DiP-A, J1:OT, J1:OT-A, S5:DiP, S5:DiP-A, S5:OT, S5:OT-A

Table 5: *(continued)*

	Conjunction	Drivers	Tree	S.Size	Model	sh	S.Time	S.Type	Best method(s)
242	Yes	9	9-A	100	Bozic	0	last	wholeT_0.01	S5:OT, S5:OT-A
243	Yes	9	9-A	100	Bozic	0	last	wholeT_0.5	J1:DiP, J1:DiP-A, J1:OT, J1:OT-A, S1:OT, S1:OT-A, S5:DiP, S5:DiP-A, S5:OT, S5:OT-A
244	Yes	9	9-A	100	Bozic	0	unif	singleC	S5:DiP, S5:DiP-A, S5:OT, S5:OT-A
245	Yes	9	9-A	100	Bozic	0	unif	wholeT_0.01	J1:OT, J1:OT-A, S5:DiP, S5:DiP-A, S5:OT, S5:OT-A
246	Yes	9	9-A	100	Bozic	0	unif	wholeT_0.5	S5:DiP, S5:DiP-A, S5:OT, S5:OT-A
247	Yes	9	9-A	100	Bozic	Inf	last	singleC	J5:OT, J5:OT-A, S5:OT, S5:OT-A
248	Yes	9	9-A	100	Bozic	Inf	last	wholeT_0.01	J5:OT, J5:OT-A
249	Yes	9	9-A	100	Bozic	Inf	last	wholeT_0.5	J5:OT, J5:OT-A, S5:OT, S5:OT-A
250	Yes	9	9-A	100	Bozic	Inf	unif	singleC	S5:DiP, S5:DiP-A, S5:OT, S5:OT-A
251	Yes	9	9-A	100	Bozic	Inf	unif	wholeT_0.01	J5:DiP, J5:DiP-A, J5:OT, J5:OT-A, S5:OT, S5:OT-A
252	Yes	9	9-A	100	Bozic	Inf	unif	wholeT_0.5	J1:OT, J1:OT-A, S5:DiP, S5:DiP-A, S5:OT, S5:OT-A
253	Yes	9	9-A	100	exp	0	last	singleC	S1:OT, S1:OT-A, S5:DiP, S5:DiP-A, S5:OT, S5:OT-A
254	Yes	9	9-A	100	exp	0	last	wholeT_0.01	J1:OT, J1:OT-A, S1:OT, S1:OT-A, S5:OT, S5:OT-A
255	Yes	9	9-A	100	exp	0	last	wholeT_0.5	S1:OT, S1:OT-A, S5:DiP, S5:DiP-A, S5:OT, S5:OT-A
256	Yes	9	9-A	100	exp	0	unif	singleC	S1:OT, S1:OT-A, S5:DiP, S5:DiP-A, S5:OT, S5:OT-A
257	Yes	9	9-A	100	exp	0	unif	wholeT_0.01	S5:DiP, S5:DiP-A, S5:OT, S5:OT-A
258	Yes	9	9-A	100	exp	0	unif	wholeT_0.5	S1:OT, S1:OT-A, S5:DiP, S5:DiP-A, S5:OT, S5:OT-A
259	Yes	9	9-A	100	exp	Inf	last	singleC	S5:OT, S5:OT-A
260	Yes	9	9-A	100	exp	Inf	last	wholeT_0.01	J5:CBN-A, J5:OT, J5:OT-A
261	Yes	9	9-A	100	exp	Inf	last	wholeT_0.5	S5:OT, S5:OT-A
262	Yes	9	9-A	100	exp	Inf	unif	singleC	J1:OT, J1:OT-A, S5:DiP, S5:DiP-A, S5:OT, S5:OT-A
263	Yes	9	9-A	100	exp	Inf	unif	wholeT_0.01	J5:OT, J5:OT-A, S5:OT, S5:OT-A
264	Yes	9	9-A	100	exp	Inf	unif	wholeT_0.5	J1:OT, J1:OT-A, S5:DiP, S5:DiP-A, S5:OT, S5:OT-A
265	Yes	9	9-A	100	McF_4	0	last	singleC	S5:OT, S5:OT-A
266	Yes	9	9-A	100	McF_4	0	last	wholeT_0.01	S5:OT, S5:OT-A
267	Yes	9	9-A	100	McF_4	0	last	wholeT_0.5	S5:OT, S5:OT-A
268	Yes	9	9-A	100	McF_4	0	unif	singleC	S5:CBN-A, S5:OT, S5:OT-A
269	Yes	9	9-A	100	McF_4	0	unif	wholeT_0.01	S5:OT, S5:OT-A

Table 5: *(continued)*

	Conjunction	Drivers	Tree	S.Size	Model	sh	S.Time	S.Type	Best method(s)
270	Yes	9	9-A	100	McF_4	0	unif	wholeT_0.5	S5:CBN-A, S5:OT, S5:OT-A
271	Yes	9	9-A	100	McF_4	Inf	last	singleC	S5:OT, S5:OT-A
272	Yes	9	9-A	100	McF_4	Inf	last	wholeT_0.01	S5:OT
273	Yes	9	9-A	100	McF_4	Inf	last	wholeT_0.5	S5:OT, S5:OT-A
274	Yes	9	9-A	100	McF_4	Inf	unif	singleC	S5:CBN-A, S5:OT, S5:OT-A
275	Yes	9	9-A	100	McF_4	Inf	unif	wholeT_0.01	S5:OT, S5:OT-A
276	Yes	9	9-A	100	McF_4	Inf	unif	wholeT_0.5	S5:OT, S5:OT-A
277	Yes	9	9-A	100	McF_6	0	last	singleC	S5:OT
278	Yes	9	9-A	100	McF_6	0	last	wholeT_0.01	S5:OT
279	Yes	9	9-A	100	McF_6	0	last	wholeT_0.5	S5:OT
280	Yes	9	9-A	100	McF_6	0	unif	singleC	J1:CBN, J1:CBN-A, S5:CBN, S5:CBN-A, S5:OT, S5:OT-A
281	Yes	9	9-A	100	McF_6	0	unif	wholeT_0.01	S5:CBN, S5:CBN-A, S5:OT, S5:OT-A
282	Yes	9	9-A	100	McF_6	0	unif	wholeT_0.5	J1:CBN, J1:CBN-A, S5:CBN, S5:CBN-A, S5:OT, S5:OT-A
283	Yes	9	9-A	100	McF_6	Inf	last	singleC	S5:OT
284	Yes	9	9-A	100	McF_6	Inf	last	wholeT_0.01	S5:OT, S5:OT-A
285	Yes	9	9-A	100	McF_6	Inf	last	wholeT_0.5	S5:OT
286	Yes	9	9-A	100	McF_6	Inf	unif	singleC	J1:CBN-A, S5:CBN, S5:CBN-A, S5:OT, S5:OT-A
287	Yes	9	9-A	100	McF_6	Inf	unif	wholeT_0.01	J1:CBN, S5:CBN, S5:CBN-A, S5:OT, S5:OT-A
288	Yes	9	9-A	100	McF_6	Inf	unif	wholeT_0.5	J1:CBN-A, S5:CBN, S5:CBN-A, S5:OT, S5:OT-A
289	Yes	7	7-A	1000	Bozic	0	last	singleC	S1:OT-A, S5:OT-A
290	Yes	7	7-A	1000	Bozic	0	last	wholeT_0.01	J1:DiP-A, J1:OT-A, J5:DiP-A, J5:OT-A, S1:DiP-A, S1:OT-A, S5:DiP-A, S5:OT-A
291	Yes	7	7-A	1000	Bozic	0	last	wholeT_0.5	S1:OT-A
292	Yes	7	7-A	1000	Bozic	0	unif	singleC	S1:OT, S1:OT-A
293	Yes	7	7-A	1000	Bozic	0	unif	wholeT_0.01	J5:CBN, J5:CBN-A, J5:DiP, J5:DiP-A, J5:OT, J5:OT-A
294	Yes	7	7-A	1000	Bozic	0	unif	wholeT_0.5	J1:CBN, J1:CBN-A, J1:DiP, J1:DiP-A, J1:OT, J1:OT-A, J5:DiP, J5:DiP-A, S1:CBN, S1:CBN-A, S1:DiP, S1:DiP-A, S1:OT, S1:OT-A, S5:DiP, S5:DiP-A
295	Yes	7	7-A	1000	Bozic	Inf	last	singleC	J1:DiP-A, J1:OT-A, J5:DiP-A, J5:OT-A, S5:DiP-A, S5:OT-A
296	Yes	7	7-A	1000	Bozic	Inf	last	wholeT_0.01	J5:DiP-A, J5:OT-A
297	Yes	7	7-A	1000	Bozic	Inf	last	wholeT_0.5	J1:DiP-A, J1:OT-A, J5:DiP-A, J5:OT-A, S5:DiP-A, S5:OT-A

Table 5: *(continued)*

	Conjunction	Drivers	Tree	S.Size	Model	sh	S.Time	S.Type	Best method(s)
298	Yes	7	7-A	1000	Bozic	Inf	unif	singleC	S1:CBN, S1:CBN-A
299	Yes	7	7-A	1000	Bozic	Inf	unif	wholeT_0.01	J5:CBN, J5:CBN-A, J5:DiP-A, J5:OT, J5:OT-A, S5:CBN, S5:CBN-A, S5:DiP-A, S5:OT, S5:OT-A
300	Yes	7	7-A	1000	Bozic	Inf	unif	wholeT_0.5	S1:CBN, S1:CBN-A
301	Yes	7	7-A	1000	exp	0	last	singleC	S1:OT-A
302	Yes	7	7-A	1000	exp	0	last	wholeT_0.01	J1:OT-A, S1:OT-A, S5:OT-A
303	Yes	7	7-A	1000	exp	0	last	wholeT_0.5	S1:OT-A
304	Yes	7	7-A	1000	exp	0	unif	singleC	J1:DiP, J1:DiP-A, J5:DiP, J5:DiP-A, S1:DiP, S1:DiP-A, S5:DiP, S5:DiP-A
305	Yes	7	7-A	1000	exp	0	unif	wholeT_0.01	J1:CBN, J1:CBN-A, J1:OT, J1:OT-A, S1:CBN-A
306	Yes	7	7-A	1000	exp	0	unif	wholeT_0.5	J1:DiP, J1:DiP-A, J5:DiP, J5:DiP-A, S1:DiP, S1:DiP-A, S5:DiP, S5:DiP-A
307	Yes	7	7-A	1000	exp	Inf	last	singleC	J1:OT-A, S5:OT-A
308	Yes	7	7-A	1000	exp	Inf	last	wholeT_0.01	J5:DiP-A, J5:OT-A, S5:DiP-A, S5:OT-A
309	Yes	7	7-A	1000	exp	Inf	last	wholeT_0.5	J1:OT-A, S1:OT-A, S5:OT-A
310	Yes	7	7-A	1000	exp	Inf	unif	singleC	S1:CBN, S1:CBN-A
311	Yes	7	7-A	1000	exp	Inf	unif	wholeT_0.01	J1:CBN, J1:CBN-A, J1:OT, J1:OT-A, J5:CBN, J5:CBN-A, J5:OT, J5:OT-A, S5:CBN, S5:CBN-A, S5:OT, S5:OT-A
312	Yes	7	7-A	1000	exp	Inf	unif	wholeT_0.5	S1:CBN, S1:CBN-A
313	Yes	7	7-A	1000	McF_4	0	last	singleC	J1:DiP-A, J1:OT-A, J5:DiP-A, J5:OT-A, S1:DiP-A, S1:OT-A, S5:DiP-A, S5:OT-A
314	Yes	7	7-A	1000	McF_4	0	last	wholeT_0.01	S5:DiP-A, S5:OT-A
315	Yes	7	7-A	1000	McF_4	0	last	wholeT_0.5	J1:DiP-A, J1:OT-A, J5:DiP-A, J5:OT-A, S1:DiP-A, S1:OT-A, S5:DiP-A, S5:OT-A
316	Yes	7	7-A	1000	McF_4	0	unif	singleC	J1:DiP-A, J1:OT, J1:OT-A, S1:DiP-A, S1:OT, S1:OT-A, S5:DiP-A, S5:OT, S5:OT-A
317	Yes	7	7-A	1000	McF_4	0	unif	wholeT_0.01	J1:DiP, J1:DiP-A, J1:OT, J1:OT-A, S5:DiP, S5:DiP-A, S5:OT, S5:OT-A
318	Yes	7	7-A	1000	McF_4	0	unif	wholeT_0.5	J1:DiP-A, J1:OT, J1:OT-A, S1:DiP-A, S1:OT, S1:OT-A, S5:DiP-A, S5:OT, S5:OT-A
319	Yes	7	7-A	1000	McF_4	Inf	last	singleC	J1:DiP-A, J1:OT-A, J5:DiP-A, J5:OT-A, S1:DiP-A, S1:OT-A, S5:DiP-A, S5:OT-A
320	Yes	7	7-A	1000	McF_4	Inf	last	wholeT_0.01	J5:DiP-A, J5:OT-A, S5:DiP-A, S5:OT-A
321	Yes	7	7-A	1000	McF_4	Inf	last	wholeT_0.5	J1:DiP-A, J1:OT-A, J5:DiP-A, J5:OT-A, S1:DiP-A, S1:OT-A, S5:DiP-A, S5:OT-A

Table 5: *(continued)*

	Conjunction	Drivers	Tree	S.Size	Model	sh	S.Time	S.Type	Best method(s)
322	Yes	7	7-A	1000	McF_4	Inf	unif	singleC	J1:DiP-A, J1:OT, J1:OT-A, S1:DiP-A, S1:OT, S1:OT-A, S5:DiP-A, S5:OT, S5:OT-A
323	Yes	7	7-A	1000	McF_4	Inf	unif	wholeT_0.01	J1:DiP-A, J1:OT, J1:OT-A, J5:OT, J5:OT-A, S5:DiP-A, S5:OT, S5:OT-A
324	Yes	7	7-A	1000	McF_4	Inf	unif	wholeT_0.5	J1:DiP-A, J1:OT, J1:OT-A, S1:DiP-A, S1:OT, S1:OT-A, S5:DiP-A, S5:OT, S5:OT-A
325	Yes	7	7-A	1000	McF_6	0	last	singleC	S5:OT-A
326	Yes	7	7-A	1000	McF_6	0	last	wholeT_0.01	J1:OT-A, S5:DiP-A, S5:OT-A
327	Yes	7	7-A	1000	McF_6	0	last	wholeT_0.5	S5:OT-A
328	Yes	7	7-A	1000	McF_6	0	unif	singleC	S1:CBN-A
329	Yes	7	7-A	1000	McF_6	0	unif	wholeT_0.01	J1:DiP, J1:DiP-A, S5:DiP, S5:DiP-A
330	Yes	7	7-A	1000	McF_6	0	unif	wholeT_0.5	S1:CBN-A
331	Yes	7	7-A	1000	McF_6	Inf	last	singleC	S5:DiP-A, S5:OT-A
332	Yes	7	7-A	1000	McF_6	Inf	last	wholeT_0.01	S5:DiP-A, S5:OT-A
333	Yes	7	7-A	1000	McF_6	Inf	last	wholeT_0.5	J1:DiP-A, J5:DiP-A, S5:DiP-A, S5:OT-A
334	Yes	7	7-A	1000	McF_6	Inf	unif	singleC	S1:CBN-A
335	Yes	7	7-A	1000	McF_6	Inf	unif	wholeT_0.01	J1:CBN-A, S5:CBN-A
336	Yes	7	7-A	1000	McF_6	Inf	unif	wholeT_0.5	S1:CBN-A
337	Yes	7	7-A	200	Bozic	0	last	singleC	S1:OT-A, S5:OT-A
338	Yes	7	7-A	200	Bozic	0	last	wholeT_0.01	J1:OT-A, J5:OT-A, S5:OT-A
339	Yes	7	7-A	200	Bozic	0	last	wholeT_0.5	S1:OT-A, S5:OT-A
340	Yes	7	7-A	200	Bozic	0	unif	singleC	J1:CBN-A, J1:OT, J1:OT-A, S1:CBN, S1:OT, S1:OT-A
341	Yes	7	7-A	200	Bozic	0	unif	wholeT_0.01	J5:CBN, J5:OT, J5:OT-A
342	Yes	7	7-A	200	Bozic	0	unif	wholeT_0.5	J1:CBN, J1:CBN-A, J1:OT, J1:OT-A, S1:CBN, S1:CBN-A, S1:OT, S1:OT-A
343	Yes	7	7-A	200	Bozic	Inf	last	singleC	J5:OT-A, S5:OT-A
344	Yes	7	7-A	200	Bozic	Inf	last	wholeT_0.01	J5:OT-A
345	Yes	7	7-A	200	Bozic	Inf	last	wholeT_0.5	J5:OT-A, S5:OT-A
346	Yes	7	7-A	200	Bozic	Inf	unif	singleC	J1:CBN, J1:CBN-A, S5:CBN, S5:CBN-A
347	Yes	7	7-A	200	Bozic	Inf	unif	wholeT_0.01	J5:CBN, J5:CBN-A, J5:OT, J5:OT-A, S5:CBN, S5:CBN-A, S5:OT, S5:OT-A
348	Yes	7	7-A	200	Bozic	Inf	unif	wholeT_0.5	J1:CBN, J1:CBN-A, S1:CBN
349	Yes	7	7-A	200	exp	0	last	singleC	S1:OT-A
350	Yes	7	7-A	200	exp	0	last	wholeT_0.01	J1:CBN-A, J1:OT-A, S1:OT-A, S5:OT-A
351	Yes	7	7-A	200	exp	0	last	wholeT_0.5	S1:OT-A

Table 5: *(continued)*

	Conjunction	Drivers	Tree	S.Size	Model	sh	S.Time	S.Type	Best method(s)
352	Yes	7	7-A	200	exp	0	unif	singleC	J1:CBN, J1:CBN-A, J1:DiP, J1:DiP-A, J1:OT, J1:OT-A, J5:DiP, J5:DiP-A, S1:CBN, S1:CBN-A, S1:DiP, S1:DiP-A, S1:OT, S1:OT-A, S5:DiP, S5:DiP-A
353	Yes	7	7-A	200	exp	0	unif	wholeT_0.01	J1:CBN, J1:CBN-A, J1:OT, J1:OT-A, S1:CBN, S1:CBN-A, S1:OT, S1:OT-A
354	Yes	7	7-A	200	exp	0	unif	wholeT_0.5	J1:DiP, J1:DiP-A, J5:DiP, J5:DiP-A, S1:DiP, S1:DiP-A, S5:DiP, S5:DiP-A
355	Yes	7	7-A	200	exp	Inf	last	singleC	J1:OT-A, S5:OT-A
356	Yes	7	7-A	200	exp	Inf	last	wholeT_0.01	J5:CBN-A, J5:OT-A
357	Yes	7	7-A	200	exp	Inf	last	wholeT_0.5	S5:OT-A
358	Yes	7	7-A	200	exp	Inf	unif	singleC	J1:CBN, J1:CBN-A, J1:OT, J1:OT-A, S1:CBN, S1:CBN-A, S1:OT, S1:OT-A
359	Yes	7	7-A	200	exp	Inf	unif	wholeT_0.01	J5:CBN, J5:CBN-A, J5:OT, J5:OT-A, S5:CBN, S5:CBN-A, S5:OT, S5:OT-A
360	Yes	7	7-A	200	exp	Inf	unif	wholeT_0.5	J1:CBN, J1:CBN-A, J1:OT, J1:OT-A, S1:CBN, S1:CBN-A, S1:OT, S1:OT-A
361	Yes	7	7-A	200	McF_4	0	last	singleC	J1:OT-A, J5:OT-A, S5:OT-A
362	Yes	7	7-A	200	McF_4	0	last	wholeT_0.01	J1:OT-A, S5:OT-A
363	Yes	7	7-A	200	McF_4	0	last	wholeT_0.5	J1:OT-A, J5:OT-A, S5:OT-A
364	Yes	7	7-A	200	McF_4	0	unif	singleC	J1:OT, J1:OT-A, S5:OT, S5:OT-A
365	Yes	7	7-A	200	McF_4	0	unif	wholeT_0.01	J1:OT, J1:OT-A, S5:OT, S5:OT-A
366	Yes	7	7-A	200	McF_4	0	unif	wholeT_0.5	J1:OT, J1:OT-A, S5:OT, S5:OT-A
367	Yes	7	7-A	200	McF_4	Inf	last	singleC	J1:OT-A, J5:OT-A, S5:OT-A
368	Yes	7	7-A	200	McF_4	Inf	last	wholeT_0.01	J5:OT-A, S5:OT-A
369	Yes	7	7-A	200	McF_4	Inf	last	wholeT_0.5	J1:OT-A, J5:OT-A, S5:OT-A
370	Yes	7	7-A	200	McF_4	Inf	unif	singleC	J1:OT, J1:OT-A, S5:OT, S5:OT-A
371	Yes	7	7-A	200	McF_4	Inf	unif	wholeT_0.01	J1:OT, J1:OT-A, S5:OT, S5:OT-A
372	Yes	7	7-A	200	McF_4	Inf	unif	wholeT_0.5	J1:OT, J1:OT-A, S5:OT, S5:OT-A
373	Yes	7	7-A	200	McF_6	0	last	singleC	S5:OT-A
374	Yes	7	7-A	200	McF_6	0	last	wholeT_0.01	S5:OT-A
375	Yes	7	7-A	200	McF_6	0	last	wholeT_0.5	S5:OT-A
376	Yes	7	7-A	200	McF_6	0	unif	singleC	J1:CBN-A, J1:OT, J1:OT-A, S5:CBN-A, S5:OT, S5:OT-A
377	Yes	7	7-A	200	McF_6	0	unif	wholeT_0.01	J1:CBN-A, J1:OT, J1:OT-A, S5:CBN-A, S5:OT, S5:OT-A
378	Yes	7	7-A	200	McF_6	0	unif	wholeT_0.5	J1:CBN-A, S5:CBN-A
379	Yes	7	7-A	200	McF_6	Inf	last	singleC	S5:OT-A
380	Yes	7	7-A	200	McF_6	Inf	last	wholeT_0.01	S5:OT-A
381	Yes	7	7-A	200	McF_6	Inf	last	wholeT_0.5	S5:OT-A

Table 5: *(continued)*

	Conjunction	Drivers	Tree	S.Size	Model	sh	S.Time	S.Type	Best method(s)
382	Yes	7	7-A	200	McF_6	Inf	unif	singleC	J1:CBN-A, J1:OT, J1:OT-A, S5:CBN-A, S5:OT, S5:OT-A
383	Yes	7	7-A	200	McF_6	Inf	unif	wholeT_0.01	J1:CBN-A, S5:CBN-A, S5:OT-A
384	Yes	7	7-A	200	McF_6	Inf	unif	wholeT_0.5	J1:CBN-A, J1:OT, J1:OT-A, S5:CBN-A
385	Yes	7	7-A	100	Bozic	0	last	singleC	S5:OT-A
386	Yes	7	7-A	100	Bozic	0	last	wholeT_0.01	J5:OT-A, S5:OT-A
387	Yes	7	7-A	100	Bozic	0	last	wholeT_0.5	S5:OT-A
388	Yes	7	7-A	100	Bozic	0	unif	singleC	J1:CBN, J1:CBN-A, J1:OT, J1:OT-A, J5:DiP, J5:DiP-A, S1:OT, S1:OT-A, S5:DiP, S5:DiP-A
389	Yes	7	7-A	100	Bozic	0	unif	wholeT_0.01	J5:CBN, J5:CBN-A, J5:OT, J5:OT-A, S5:CBN-A
390	Yes	7	7-A	100	Bozic	0	unif	wholeT_0.5	J1:CBN, J1:CBN-A, J1:OT, J1:OT-A, J5:DiP, J5:DiP-A, S1:OT, S1:OT-A, S5:DiP, S5:DiP-A
391	Yes	7	7-A	100	Bozic	Inf	last	singleC	J5:OT-A
392	Yes	7	7-A	100	Bozic	Inf	last	wholeT_0.01	J5:OT-A
393	Yes	7	7-A	100	Bozic	Inf	last	wholeT_0.5	J5:OT-A, S5:OT-A
394	Yes	7	7-A	100	Bozic	Inf	unif	singleC	J1:CBN, J1:CBN-A, S5:CBN, S5:CBN-A
395	Yes	7	7-A	100	Bozic	Inf	unif	wholeT_0.01	J5:CBN, J5:CBN-A, J5:OT, J5:OT-A, S5:CBN-A, S5:OT, S5:OT-A
396	Yes	7	7-A	100	Bozic	Inf	unif	wholeT_0.5	J1:CBN, J1:CBN-A, S5:CBN, S5:CBN-A
397	Yes	7	7-A	100	exp	0	last	singleC	S1:OT-A
398	Yes	7	7-A	100	exp	0	last	wholeT_0.01	J1:CBN-A, J1:OT-A, J5:OT-A, S1:OT-A, S5:OT-A
399	Yes	7	7-A	100	exp	0	last	wholeT_0.5	S1:OT-A
400	Yes	7	7-A	100	exp	0	unif	singleC	J1:CBN, J1:CBN-A, J1:OT, J1:OT-A, J5:DiP, J5:DiP-A, S1:CBN, S1:CBN-A, S1:OT, S1:OT-A, S5:DiP, S5:DiP-A
401	Yes	7	7-A	100	exp	0	unif	wholeT_0.01	J1:CBN, J1:CBN-A, J1:OT, J1:OT-A, J5:CBN, J5:CBN-A, J5:DiP, J5:DiP-A, J5:OT, J5:OT-A, S1:OT, S1:OT-A, S5:CBN, S5:CBN-A, S5:DiP, S5:DiP-A, S5:OT, S5:OT-A
402	Yes	7	7-A	100	exp	0	unif	wholeT_0.5	J1:CBN, J1:CBN-A, J1:OT, J1:OT-A, J5:DiP, J5:DiP-A, S1:CBN, S1:CBN-A, S1:OT, S1:OT-A, S5:DiP, S5:DiP-A
403	Yes	7	7-A	100	exp	Inf	last	singleC	S5:OT-A
404	Yes	7	7-A	100	exp	Inf	last	wholeT_0.01	J5:OT-A
405	Yes	7	7-A	100	exp	Inf	last	wholeT_0.5	S5:OT-A

Table 5: *(continued)*

	Conjunction	Drivers	Tree	S.Size	Model	sh	S.Time	S.Type	Best method(s)
406	Yes	7	7-A	100	exp	Inf	unif	singleC	J1:CBN, J1:CBN-A, J1:OT, J1:OT-A, J5:CBN, J5:CBN-A, J5:OT, J5:OT-A, S1:OT, S1:OT-A, S5:CBN, S5:CBN-A, S5:OT, S5:OT-A
407	Yes	7	7-A	100	exp	Inf	unif	wholeT_0.01	J5:CBN, J5:CBN-A, J5:OT, J5:OT-A, S5:OT, S5:OT-A
408	Yes	7	7-A	100	exp	Inf	unif	wholeT_0.5	J1:CBN, J1:CBN-A, J1:OT, J1:OT-A, S1:CBN-A, S1:OT, S1:OT-A
409	Yes	7	7-A	100	McF_4	0	last	singleC	J1:OT-A, J5:OT-A, S5:OT-A
410	Yes	7	7-A	100	McF_4	0	last	wholeT_0.01	S5:OT-A
411	Yes	7	7-A	100	McF_4	0	last	wholeT_0.5	J1:OT-A, J5:OT-A, S5:OT-A
412	Yes	7	7-A	100	McF_4	0	unif	singleC	J1:OT, J1:OT-A, S5:OT, S5:OT-A
413	Yes	7	7-A	100	McF_4	0	unif	wholeT_0.01	J1:OT-A, S5:OT, S5:OT-A
414	Yes	7	7-A	100	McF_4	0	unif	wholeT_0.5	J1:OT, J1:OT-A, S5:OT, S5:OT-A
415	Yes	7	7-A	100	McF_4	Inf	last	singleC	J1:OT-A, J5:OT-A, S5:OT-A
416	Yes	7	7-A	100	McF_4	Inf	last	wholeT_0.01	J5:OT-A, S5:OT-A
417	Yes	7	7-A	100	McF_4	Inf	last	wholeT_0.5	J5:OT-A, S5:OT-A
418	Yes	7	7-A	100	McF_4	Inf	unif	singleC	J1:OT, J1:OT-A, S5:OT, S5:OT-A
419	Yes	7	7-A	100	McF_4	Inf	unif	wholeT_0.01	J1:OT, J1:OT-A, J5:OT-A, S5:OT, S5:OT-A
420	Yes	7	7-A	100	McF_4	Inf	unif	wholeT_0.5	J1:OT, J1:OT-A, J5:OT, J5:OT-A, S5:OT, S5:OT-A
421	Yes	7	7-A	100	McF_6	0	last	singleC	S5:OT-A
422	Yes	7	7-A	100	McF_6	0	last	wholeT_0.01	S5:OT-A
423	Yes	7	7-A	100	McF_6	0	last	wholeT_0.5	S5:OT-A
424	Yes	7	7-A	100	McF_6	0	unif	singleC	J1:CBN-A, S5:CBN, S5:CBN-A, S5:OT-A
425	Yes	7	7-A	100	McF_6	0	unif	wholeT_0.01	J5:CBN-A, S5:CBN, S5:CBN-A, S5:OT, S5:OT-A
426	Yes	7	7-A	100	McF_6	0	unif	wholeT_0.5	J1:CBN-A, S5:CBN-A
427	Yes	7	7-A	100	McF_6	Inf	last	singleC	S5:OT-A
428	Yes	7	7-A	100	McF_6	Inf	last	wholeT_0.01	S5:OT-A
429	Yes	7	7-A	100	McF_6	Inf	last	wholeT_0.5	S5:OT-A
430	Yes	7	7-A	100	McF_6	Inf	unif	singleC	S5:CBN-A
431	Yes	7	7-A	100	McF_6	Inf	unif	wholeT_0.01	S5:CBN-A
432	Yes	7	7-A	100	McF_6	Inf	unif	wholeT_0.5	J1:CBN-A, J1:OT-A, J5:CBN-A, S5:CBN-A, S5:OT-A
433	No	11	11-B	1000	Bozic	0	last	singleC	S1:OT, S1:OT-A
434	No	11	11-B	1000	Bozic	0	last	wholeT_0.01	S1:DiP, S1:DiP-A, S5:DiP, S5:DiP-A
435	No	11	11-B	1000	Bozic	0	last	wholeT_0.5	S1:OT, S1:OT-A

Table 5: *(continued)*

	Conjunction	Drivers	Tree	S.Size	Model	sh	S.Time	S.Type	Best method(s)
436	No	11	11-B	1000	Bozic	0	unif	singleC	S1:DiP, S1:DiP-A, S1:OT, S1:OT-A, S5:DiP, S5:DiP-A, S5:OT, S5:OT-A
437	No	11	11-B	1000	Bozic	0	unif	wholeT_0.01	J1:CBN-A, S1:OT, S1:OT-A, S5:DiP, S5:DiP-A, S5:OT, S5:OT-A
438	No	11	11-B	1000	Bozic	0	unif	wholeT_0.5	S1:DiP, S1:DiP-A, S1:OT, S1:OT-A, S5:DiP, S5:DiP-A, S5:OT, S5:OT-A
439	No	11	11-B	1000	Bozic	Inf	last	singleC	S5:DiP, S5:DiP-A, S5:OT, S5:OT-A
440	No	11	11-B	1000	Bozic	Inf	last	wholeT_0.01	J1:DiP, J1:DiP-A, J1:OT, J1:OT-A, J5:DiP, J5:DiP-A, J5:OT, J5:OT-A
441	No	11	11-B	1000	Bozic	Inf	last	wholeT_0.5	S5:DiP, S5:DiP-A, S5:OT, S5:OT-A
442	No	11	11-B	1000	Bozic	Inf	unif	singleC	S1:CBN, S1:CBN-A, S1:DiP-A, S1:OT, S1:OT-A
443	No	11	11-B	1000	Bozic	Inf	unif	wholeT_0.01	J1:CBN-A, J1:DiP, J1:DiP-A, J1:OT, J1:OT-A, J5:DiP, J5:DiP-A, J5:OT, J5:OT-A, S5:CBN, S5:CBN-A, S5:DiP, S5:DiP-A, S5:OT, S5:OT-A
444	No	11	11-B	1000	Bozic	Inf	unif	wholeT_0.5	S1:CBN, S1:CBN-A, S1:OT, S1:OT-A
445	No	11	11-B	1000	exp	0	last	singleC	S1:OT, S1:OT-A
446	No	11	11-B	1000	exp	0	last	wholeT_0.01	S1:DiP-A, S1:OT, S1:OT-A, S5:DiP-A, S5:OT, S5:OT-A
447	No	11	11-B	1000	exp	0	last	wholeT_0.5	S1:OT, S1:OT-A
448	No	11	11-B	1000	exp	0	unif	singleC	S1:DiP, S1:DiP-A, S1:OT, S1:OT-A, S5:DiP, S5:DiP-A, S5:OT, S5:OT-A
449	No	11	11-B	1000	exp	0	unif	wholeT_0.01	S1:CBN-A, S1:DiP-A, S1:OT, S1:OT-A
450	No	11	11-B	1000	exp	0	unif	wholeT_0.5	S1:DiP, S1:DiP-A, S1:OT, S1:OT-A, S5:DiP, S5:DiP-A, S5:OT, S5:OT-A
451	No	11	11-B	1000	exp	Inf	last	singleC	S5:DiP, S5:DiP-A, S5:OT, S5:OT-A
452	No	11	11-B	1000	exp	Inf	last	wholeT_0.01	S5:DiP, S5:DiP-A, S5:OT, S5:OT-A
453	No	11	11-B	1000	exp	Inf	last	wholeT_0.5	S5:DiP, S5:DiP-A, S5:OT, S5:OT-A
454	No	11	11-B	1000	exp	Inf	unif	singleC	S1:CBN, S1:CBN-A, S1:OT, S1:OT-A
455	No	11	11-B	1000	exp	Inf	unif	wholeT_0.01	S5:DiP, S5:DiP-A, S5:OT, S5:OT-A
456	No	11	11-B	1000	exp	Inf	unif	wholeT_0.5	S1:CBN, S1:CBN-A, S1:OT, S1:OT-A
457	No	11	11-B	1000	McF_4	0	last	singleC	S1:DiP-A, S1:OT, S1:OT-A
458	No	11	11-B	1000	McF_4	0	last	wholeT_0.01	S1:DiP, S1:DiP-A, S1:OT, S1:OT-A, S5:DiP, S5:DiP-A, S5:OT, S5:OT-A
459	No	11	11-B	1000	McF_4	0	last	wholeT_0.5	S1:DiP-A, S1:OT, S1:OT-A
460	No	11	11-B	1000	McF_4	0	unif	singleC	S1:DiP, S1:DiP-A, S1:OT, S1:OT-A
461	No	11	11-B	1000	McF_4	0	unif	wholeT_0.01	S1:OT, S1:OT-A
462	No	11	11-B	1000	McF_4	0	unif	wholeT_0.5	S1:DiP, S1:DiP-A, S1:OT, S1:OT-A
463	No	11	11-B	1000	McF_4	Inf	last	singleC	S1:OT, S1:OT-A

Table 5: *(continued)*

	Conjunction	Drivers	Tree	S.Size	Model	sh	S.Time	S.Type	Best method(s)
464	No	11	11-B	1000	McF_4	Inf	last	wholeT_0.01	S5:DiP, S5:DiP-A, S5:OT, S5:OT-A
465	No	11	11-B	1000	McF_4	Inf	last	wholeT_0.5	S1:DiP-A, S1:OT, S1:OT-A
466	No	11	11-B	1000	McF_4	Inf	unif	singleC	S1:OT, S1:OT-A
467	No	11	11-B	1000	McF_4	Inf	unif	wholeT_0.01	S5:DiP, S5:DiP-A, S5:OT, S5:OT-A
468	No	11	11-B	1000	McF_4	Inf	unif	wholeT_0.5	S1:OT, S1:OT-A
469	No	11	11-B	1000	McF_6	0	last	singleC	S5:DiP, S5:OT
470	No	11	11-B	1000	McF_6	0	last	wholeT_0.01	S5:DiP, S5:OT
471	No	11	11-B	1000	McF_6	0	last	wholeT_0.5	S5:DiP, S5:OT
472	No	11	11-B	1000	McF_6	0	unif	singleC	S1:DiP, S1:DiP-A, S1:OT, S1:OT-A
473	No	11	11-B	1000	McF_6	0	unif	wholeT_0.01	S1:DiP, S1:DiP-A, S1:OT, S1:OT-A
474	No	11	11-B	1000	McF_6	0	unif	wholeT_0.5	S1:DiP, S1:DiP-A, S1:OT, S1:OT-A
475	No	11	11-B	1000	McF_6	Inf	last	singleC	S5:DiP, S5:OT
476	No	11	11-B	1000	McF_6	Inf	last	wholeT_0.01	S5:OT
477	No	11	11-B	1000	McF_6	Inf	last	wholeT_0.5	S1:DiP, S1:OT, S5:DiP, S5:OT
478	No	11	11-B	1000	McF_6	Inf	unif	singleC	S1:DiP, S1:DiP-A
479	No	11	11-B	1000	McF_6	Inf	unif	wholeT_0.01	S1:DiP, S1:DiP-A
480	No	11	11-B	1000	McF_6	Inf	unif	wholeT_0.5	S1:DiP, S1:DiP-A, S1:OT, S1:OT-A
481	No	11	11-B	200	Bozic	0	last	singleC	S1:OT, S1:OT-A
482	No	11	11-B	200	Bozic	0	last	wholeT_0.01	J1:OT, J1:OT-A, J5:OT, J5:OT-A, S5:OT, S5:OT-A
483	No	11	11-B	200	Bozic	0	last	wholeT_0.5	S1:OT, S1:OT-A
484	No	11	11-B	200	Bozic	0	unif	singleC	S1:OT, S1:OT-A
485	No	11	11-B	200	Bozic	0	unif	wholeT_0.01	J1:CBN-A, S5:CBN-A, S5:DiP, S5:DiP-A, S5:OT, S5:OT-A
486	No	11	11-B	200	Bozic	0	unif	wholeT_0.5	S1:OT, S1:OT-A, S5:DiP, S5:DiP-A, S5:OT, S5:OT-A
487	No	11	11-B	200	Bozic	Inf	last	singleC	S5:OT, S5:OT-A
488	No	11	11-B	200	Bozic	Inf	last	wholeT_0.01	J5:OT, J5:OT-A
489	No	11	11-B	200	Bozic	Inf	last	wholeT_0.5	S5:OT, S5:OT-A
490	No	11	11-B	200	Bozic	Inf	unif	singleC	J1:CBN, J1:CBN-A, J1:OT, J1:OT-A, S5:DiP, S5:DiP-A, S5:OT, S5:OT-A
491	No	11	11-B	200	Bozic	Inf	unif	wholeT_0.01	J5:CBN-A, J5:OT, J5:OT-A, S5:CBN, S5:CBN-A, S5:OT, S5:OT-A
492	No	11	11-B	200	Bozic	Inf	unif	wholeT_0.5	J1:CBN, J1:CBN-A, J1:OT, J1:OT-A, S5:OT, S5:OT-A
493	No	11	11-B	200	exp	0	last	singleC	S1:OT, S1:OT-A
494	No	11	11-B	200	exp	0	last	wholeT_0.01	S1:OT, S1:OT-A, S5:OT, S5:OT-A
495	No	11	11-B	200	exp	0	last	wholeT_0.5	S1:OT, S1:OT-A
496	No	11	11-B	200	exp	0	unif	singleC	S1:DiP, S1:DiP-A, S1:OT, S1:OT-A, S5:DiP, S5:DiP-A, S5:OT, S5:OT-A

Table 5: *(continued)*

	Conjunction	Drivers	Tree	S.Size	Model	sh	S.Time	S.Type	Best method(s)
497	No	11	11-B	200	exp	0	unif	wholeT_0.01	S1:OT, S1:OT-A
498	No	11	11-B	200	exp	0	unif	wholeT_0.5	S1:DiP, S1:DiP-A, S1:OT, S1:OT-A, S5:DiP, S5:DiP-A, S5:OT, S5:OT-A
499	No	11	11-B	200	exp	Inf	last	singleC	S5:OT, S5:OT-A
500	No	11	11-B	200	exp	Inf	last	wholeT_0.01	J5:OT, J5:OT-A
501	No	11	11-B	200	exp	Inf	last	wholeT_0.5	S5:OT, S5:OT-A
502	No	11	11-B	200	exp	Inf	unif	singleC	J1:OT, J1:OT-A, S1:OT, S1:OT-A
503	No	11	11-B	200	exp	Inf	unif	wholeT_0.01	S5:CBN-A, S5:OT, S5:OT-A
504	No	11	11-B	200	exp	Inf	unif	wholeT_0.5	S1:OT, S1:OT-A
505	No	11	11-B	200	McF_4	0	last	singleC	S1:OT, S1:OT-A, S5:OT, S5:OT-A
506	No	11	11-B	200	McF_4	0	last	wholeT_0.01	S5:OT, S5:OT-A
507	No	11	11-B	200	McF_4	0	last	wholeT_0.5	S1:OT, S1:OT-A, S5:OT, S5:OT-A
508	No	11	11-B	200	McF_4	0	unif	singleC	S1:OT, S1:OT-A, S5:OT, S5:OT-A
509	No	11	11-B	200	McF_4	0	unif	wholeT_0.01	S5:OT, S5:OT-A
510	No	11	11-B	200	McF_4	0	unif	wholeT_0.5	S1:OT, S1:OT-A, S5:CBN-A, S5:OT, S5:OT-A
511	No	11	11-B	200	McF_4	Inf	last	singleC	S1:OT, S1:OT-A, S5:OT, S5:OT-A
512	No	11	11-B	200	McF_4	Inf	last	wholeT_0.01	S5:OT, S5:OT-A
513	No	11	11-B	200	McF_4	Inf	last	wholeT_0.5	S1:OT, S1:OT-A
514	No	11	11-B	200	McF_4	Inf	unif	singleC	S1:OT, S1:OT-A, S5:CBN-A, S5:OT, S5:OT-A
515	No	11	11-B	200	McF_4	Inf	unif	wholeT_0.01	S5:CBN-A, S5:OT, S5:OT-A
516	No	11	11-B	200	McF_4	Inf	unif	wholeT_0.5	S1:OT, S1:OT-A, S5:OT, S5:OT-A
517	No	11	11-B	200	McF_6	0	last	singleC	S5:OT
518	No	11	11-B	200	McF_6	0	last	wholeT_0.01	S5:OT
519	No	11	11-B	200	McF_6	0	last	wholeT_0.5	S5:OT
520	No	11	11-B	200	McF_6	0	unif	singleC	J1:CBN-A, S1:OT, S1:OT-A, S5:CBN-A, S5:OT, S5:OT-A
521	No	11	11-B	200	McF_6	0	unif	wholeT_0.01	S1:OT, S1:OT-A, S5:CBN, S5:OT, S5:OT-A
522	No	11	11-B	200	McF_6	0	unif	wholeT_0.5	S1:OT, S1:OT-A, S5:CBN, S5:OT, S5:OT-A
523	No	11	11-B	200	McF_6	Inf	last	singleC	S5:OT
524	No	11	11-B	200	McF_6	Inf	last	wholeT_0.01	S5:OT
525	No	11	11-B	200	McF_6	Inf	last	wholeT_0.5	S5:OT
526	No	11	11-B	200	McF_6	Inf	unif	singleC	J1:CBN-A, S1:OT, S1:OT-A, S5:CBN, S5:CBN-A, S5:OT, S5:OT-A
527	No	11	11-B	200	McF_6	Inf	unif	wholeT_0.01	S5:CBN, S5:CBN-A, S5:OT, S5:OT-A
528	No	11	11-B	200	McF_6	Inf	unif	wholeT_0.5	J1:CBN-A, S1:OT, S1:OT-A, S5:CBN, S5:CBN-A, S5:OT, S5:OT-A

Table 5: *(continued)*

	Conjunction	Drivers	Tree	S.Size	Model	sh	S.Time	S.Type	Best method(s)
529	No	11	11-B	100	Bozic	0	last	singleC	S5:OT, S5:OT-A
530	No	11	11-B	100	Bozic	0	last	wholeT_0.01	J1:OT, J1:OT-A, J5:CBN-A, J5:OT, J5:OT-A, S5:OT, S5:OT-A
531	No	11	11-B	100	Bozic	0	last	wholeT_0.5	S1:OT, S1:OT-A, S5:DiP, S5:DiP-A, S5:OT, S5:OT-A
532	No	11	11-B	100	Bozic	0	unif	singleC	J1:OT, J1:OT-A, S5:DiP, S5:DiP-A, S5:OT, S5:OT-A
533	No	11	11-B	100	Bozic	0	unif	wholeT_0.01	S5:CBN, S5:OT, S5:OT-A
534	No	11	11-B	100	Bozic	0	unif	wholeT_0.5	S5:DiP, S5:DiP-A, S5:OT, S5:OT-A
535	No	11	11-B	100	Bozic	Inf	last	singleC	S5:OT, S5:OT-A
536	No	11	11-B	100	Bozic	Inf	last	wholeT_0.01	J5:OT, J5:OT-A
537	No	11	11-B	100	Bozic	Inf	last	wholeT_0.5	J5:OT, J5:OT-A, S5:OT, S5:OT-A
538	No	11	11-B	100	Bozic	Inf	unif	singleC	J1:OT, J1:OT-A, S5:CBN-A, S5:OT, S5:OT-A
539	No	11	11-B	100	Bozic	Inf	unif	wholeT_0.01	J5:CBN, J5:CBN-A, J5:OT, J5:OT-A, S5:CBN-A, S5:OT, S5:OT-A
540	No	11	11-B	100	Bozic	Inf	unif	wholeT_0.5	S5:OT, S5:OT-A
541	No	11	11-B	100	exp	0	last	singleC	S1:OT, S1:OT-A
542	No	11	11-B	100	exp	0	last	wholeT_0.01	S1:OT, S1:OT-A, S5:OT, S5:OT-A
543	No	11	11-B	100	exp	0	last	wholeT_0.5	S1:OT, S1:OT-A
544	No	11	11-B	100	exp	0	unif	singleC	S1:CBN-A, S1:OT, S1:OT-A, S5:DiP, S5:DiP-A, S5:OT, S5:OT-A
545	No	11	11-B	100	exp	0	unif	wholeT_0.01	J1:OT, J1:OT-A, S1:OT, S1:OT-A, S5:DiP, S5:DiP-A, S5:OT, S5:OT-A
546	No	11	11-B	100	exp	0	unif	wholeT_0.5	S1:OT, S1:OT-A, S5:DiP, S5:DiP-A, S5:OT, S5:OT-A
547	No	11	11-B	100	exp	Inf	last	singleC	S5:OT, S5:OT-A
548	No	11	11-B	100	exp	Inf	last	wholeT_0.01	J5:CBN-A, J5:OT, J5:OT-A
549	No	11	11-B	100	exp	Inf	last	wholeT_0.5	S5:OT, S5:OT-A
550	No	11	11-B	100	exp	Inf	unif	singleC	J1:OT, J1:OT-A, S5:DiP, S5:DiP-A, S5:OT, S5:OT-A
551	No	11	11-B	100	exp	Inf	unif	wholeT_0.01	S5:CBN, S5:CBN-A, S5:OT, S5:OT-A
552	No	11	11-B	100	exp	Inf	unif	wholeT_0.5	J1:OT, J1:OT-A, S5:DiP, S5:DiP-A, S5:OT, S5:OT-A
553	No	11	11-B	100	McF_4	0	last	singleC	S1:OT, S1:OT-A, S5:OT, S5:OT-A
554	No	11	11-B	100	McF_4	0	last	wholeT_0.01	S5:OT, S5:OT-A
555	No	11	11-B	100	McF_4	0	last	wholeT_0.5	S1:OT, S1:OT-A, S5:OT, S5:OT-A
556	No	11	11-B	100	McF_4	0	unif	singleC	S5:CBN-A, S5:OT, S5:OT-A
557	No	11	11-B	100	McF_4	0	unif	wholeT_0.01	S5:CBN, S5:CBN-A, S5:OT, S5:OT-A
558	No	11	11-B	100	McF_4	0	unif	wholeT_0.5	S5:OT, S5:OT-A

Table 5: *(continued)*

	Conjunction	Drivers	Tree	S.Size	Model	sh	S.Time	S.Type	Best method(s)
559	No	11	11-B	100	McF_4	Inf	last	singleC	S1:OT, S1:OT-A, S5:OT, S5:OT-A
560	No	11	11-B	100	McF_4	Inf	last	wholeT_0.01	S5:OT, S5:OT-A
561	No	11	11-B	100	McF_4	Inf	last	wholeT_0.5	S1:OT, S1:OT-A, S5:OT, S5:OT-A
562	No	11	11-B	100	McF_4	Inf	unif	singleC	S5:OT, S5:OT-A
563	No	11	11-B	100	McF_4	Inf	unif	wholeT_0.01	S5:CBN-A, S5:OT, S5:OT-A
564	No	11	11-B	100	McF_4	Inf	unif	wholeT_0.5	S1:OT, S1:OT-A, S5:CBN-A, S5:OT, S5:OT-A
565	No	11	11-B	100	McF_6	0	last	singleC	S5:OT
566	No	11	11-B	100	McF_6	0	last	wholeT_0.01	S5:OT
567	No	11	11-B	100	McF_6	0	last	wholeT_0.5	S5:OT
568	No	11	11-B	100	McF_6	0	unif	singleC	S1:OT, S1:OT-A, S5:CBN, S5:CBN-A, S5:OT, S5:OT-A
569	No	11	11-B	100	McF_6	0	unif	wholeT_0.01	S5:CBN-A, S5:OT, S5:OT-A
570	No	11	11-B	100	McF_6	0	unif	wholeT_0.5	J1:CBN, J1:CBN-A, S1:OT, S1:OT-A, S5:CBN, S5:CBN-A, S5:OT, S5:OT-A
571	No	11	11-B	100	McF_6	Inf	last	singleC	S5:OT
572	No	11	11-B	100	McF_6	Inf	last	wholeT_0.01	S5:OT, S5:OT-A
573	No	11	11-B	100	McF_6	Inf	last	wholeT_0.5	S5:OT
574	No	11	11-B	100	McF_6	Inf	unif	singleC	S1:OT, S1:OT-A, S5:CBN, S5:CBN-A, S5:OT, S5:OT-A
575	No	11	11-B	100	McF_6	Inf	unif	wholeT_0.01	S5:CBN, S5:CBN-A, S5:OT, S5:OT-A
576	No	11	11-B	100	McF_6	Inf	unif	wholeT_0.5	S5:CBN-A, S5:OT, S5:OT-A
577	No	9	9-B	1000	Bozic	0	last	singleC	S1:OT, S1:OT-A
578	No	9	9-B	1000	Bozic	0	last	wholeT_0.01	S1:CBN-A, S1:DiP, S1:DiP-A, S1:OT, S1:OT-A, S5:CBN-A, S5:DiP, S5:DiP-A, S5:OT, S5:OT-A
579	No	9	9-B	1000	Bozic	0	last	wholeT_0.5	S1:OT, S1:OT-A
580	No	9	9-B	1000	Bozic	0	unif	singleC	S1:DiP, S1:DiP-A, S1:OT, S1:OT-A, S5:DiP, S5:DiP-A, S5:OT, S5:OT-A
581	No	9	9-B	1000	Bozic	0	unif	wholeT_0.01	J1:DiP, J1:DiP-A, J1:OT, J1:OT-A, S1:DiP, S1:DiP-A, S1:OT, S1:OT-A, S5:DiP, S5:DiP-A, S5:OT, S5:OT-A
582	No	9	9-B	1000	Bozic	0	unif	wholeT_0.5	S1:DiP, S1:DiP-A, S1:OT, S1:OT-A, S5:DiP, S5:DiP-A, S5:OT, S5:OT-A
583	No	9	9-B	1000	Bozic	Inf	last	singleC	S5:DiP, S5:DiP-A, S5:OT, S5:OT-A
584	No	9	9-B	1000	Bozic	Inf	last	wholeT_0.01	J5:DiP, J5:DiP-A
585	No	9	9-B	1000	Bozic	Inf	last	wholeT_0.5	S5:DiP, S5:DiP-A, S5:OT, S5:OT-A
586	No	9	9-B	1000	Bozic	Inf	unif	singleC	S1:CBN, S1:CBN-A, S1:OT, S1:OT-A
587	No	9	9-B	1000	Bozic	Inf	unif	wholeT_0.01	J1:CBN-A, S5:CBN, S5:CBN-A, S5:DiP, S5:DiP-A, S5:OT, S5:OT-A

Table 5: *(continued)*

	Conjunction	Drivers	Tree	S.Size	Model	sh	S.Time	S.Type	Best method(s)
588	No	9	9-B	1000	Bozic	Inf	unif	wholeT_0.5	S1:CBN, S1:CBN-A, S1:OT, S1:OT-A
589	No	9	9-B	1000	exp	0	last	singleC	S1:OT, S1:OT-A
590	No	9	9-B	1000	exp	0	last	wholeT_0.01	S1:DiP-A, S1:OT, S1:OT-A
591	No	9	9-B	1000	exp	0	last	wholeT_0.5	S1:DiP, S1:DiP-A, S1:OT, S1:OT-A, S5:DiP, S5:DiP-A, S5:OT, S5:OT-A
592	No	9	9-B	1000	exp	0	unif	singleC	S1:DiP, S1:DiP-A, S1:OT, S1:OT-A, S5:DiP, S5:DiP-A, S5:OT, S5:OT-A
593	No	9	9-B	1000	exp	0	unif	wholeT_0.01	S1:DiP, S1:DiP-A, S1:OT, S1:OT-A, S5:DiP, S5:DiP-A, S5:OT, S5:OT-A
594	No	9	9-B	1000	exp	0	unif	wholeT_0.5	S1:DiP, S1:DiP-A, S1:OT, S1:OT-A, S5:DiP, S5:DiP-A, S5:OT, S5:OT-A
595	No	9	9-B	1000	exp	Inf	last	singleC	S5:DiP, S5:DiP-A, S5:OT, S5:OT-A
596	No	9	9-B	1000	exp	Inf	last	wholeT_0.01	J1:DiP, J1:DiP-A, J5:DiP, J5:DiP-A, S5:DiP, S5:DiP-A
597	No	9	9-B	1000	exp	Inf	last	wholeT_0.5	S5:DiP, S5:DiP-A, S5:OT, S5:OT-A
598	No	9	9-B	1000	exp	Inf	unif	singleC	S1:CBN, S1:CBN-A, S1:OT, S1:OT-A
599	No	9	9-B	1000	exp	Inf	unif	wholeT_0.01	J1:OT, J1:OT-A, J5:DiP, J5:DiP-A, J5:OT, J5:OT-A, S5:DiP, S5:DiP-A, S5:OT, S5:OT-A
600	No	9	9-B	1000	exp	Inf	unif	wholeT_0.5	S1:OT, S1:OT-A
601	No	9	9-B	1000	McF_4	0	last	singleC	S1:DiP, S1:DiP-A, S1:OT, S1:OT-A
602	No	9	9-B	1000	McF_4	0	last	wholeT_0.01	S5:DiP, S5:DiP-A, S5:OT, S5:OT-A
603	No	9	9-B	1000	McF_4	0	last	wholeT_0.5	S1:DiP-A, S1:OT, S1:OT-A
604	No	9	9-B	1000	McF_4	0	unif	singleC	S1:DiP, S1:DiP-A, S1:OT, S1:OT-A
605	No	9	9-B	1000	McF_4	0	unif	wholeT_0.01	S1:OT, S1:OT-A, S5:DiP, S5:DiP-A, S5:OT, S5:OT-A
606	No	9	9-B	1000	McF_4	0	unif	wholeT_0.5	S1:DiP, S1:DiP-A, S1:OT, S1:OT-A
607	No	9	9-B	1000	McF_4	Inf	last	singleC	S1:OT, S1:OT-A
608	No	9	9-B	1000	McF_4	Inf	last	wholeT_0.01	S5:DiP, S5:DiP-A, S5:OT, S5:OT-A
609	No	9	9-B	1000	McF_4	Inf	last	wholeT_0.5	S1:OT, S1:OT-A
610	No	9	9-B	1000	McF_4	Inf	unif	singleC	S1:DiP, S1:DiP-A, S1:OT, S1:OT-A
611	No	9	9-B	1000	McF_4	Inf	unif	wholeT_0.01	S5:DiP, S5:DiP-A, S5:OT, S5:OT-A
612	No	9	9-B	1000	McF_4	Inf	unif	wholeT_0.5	S1:DiP, S1:DiP-A, S1:OT, S1:OT-A
613	No	9	9-B	1000	McF_6	0	last	singleC	S5:OT
614	No	9	9-B	1000	McF_6	0	last	wholeT_0.01	J1:OT, S5:OT
615	No	9	9-B	1000	McF_6	0	last	wholeT_0.5	S1:OT, S5:OT
616	No	9	9-B	1000	McF_6	0	unif	singleC	S1:DiP, S1:DiP-A, S1:OT, S1:OT-A
617	No	9	9-B	1000	McF_6	0	unif	wholeT_0.01	S5:DiP, S5:DiP-A, S5:OT, S5:OT-A
618	No	9	9-B	1000	McF_6	0	unif	wholeT_0.5	S1:DiP, S1:DiP-A, S1:OT, S1:OT-A
619	No	9	9-B	1000	McF_6	Inf	last	singleC	S5:DiP, S5:DiP-A, S5:OT, S5:OT-A

Table 5: *(continued)*

	Conjunction	Drivers	Tree	S.Size	Model	sh	S.Time	S.Type	Best method(s)
620	No	9	9-B	1000	McF_6	Inf	last	wholeT_0.01	S5:DiP, S5:DiP-A, S5:OT-A
621	No	9	9-B	1000	McF_6	Inf	last	wholeT_0.5	S1:DiP, S1:DiP-A, S1:OT-A, S5:DiP, S5:DiP-A, S5:OT-A
622	No	9	9-B	1000	McF_6	Inf	unif	singleC	S1:DiP, S1:DiP-A, S1:OT, S1:OT-A
623	No	9	9-B	1000	McF_6	Inf	unif	wholeT_0.01	S5:CBN, S5:DiP, S5:DiP-A, S5:OT, S5:OT-A
624	No	9	9-B	1000	McF_6	Inf	unif	wholeT_0.5	S1:DiP, S1:DiP-A, S1:OT, S1:OT-A
625	No	9	9-B	200	Bozic	0	last	singleC	S1:OT, S1:OT-A
626	No	9	9-B	200	Bozic	0	last	wholeT_0.01	S5:OT, S5:OT-A
627	No	9	9-B	200	Bozic	0	last	wholeT_0.5	S1:OT, S1:OT-A
628	No	9	9-B	200	Bozic	0	unif	singleC	S1:OT, S1:OT-A, S5:DiP, S5:DiP-A, S5:OT, S5:OT-A
629	No	9	9-B	200	Bozic	0	unif	wholeT_0.01	J1:DiP, J1:DiP-A, J1:OT, J1:OT-A, S5:DiP, S5:DiP-A, S5:OT, S5:OT-A
630	No	9	9-B	200	Bozic	0	unif	wholeT_0.5	S1:DiP, S1:DiP-A, S1:OT, S1:OT-A, S5:DiP, S5:DiP-A, S5:OT, S5:OT-A
631	No	9	9-B	200	Bozic	Inf	last	singleC	S5:OT, S5:OT-A
632	No	9	9-B	200	Bozic	Inf	last	wholeT_0.01	J5:CBN-A, J5:OT, J5:OT-A
633	No	9	9-B	200	Bozic	Inf	last	wholeT_0.5	S5:OT, S5:OT-A
634	No	9	9-B	200	Bozic	Inf	unif	singleC	J1:DiP, J1:DiP-A, J1:OT, J1:OT-A, S5:DiP, S5:DiP-A, S5:OT, S5:OT-A
635	No	9	9-B	200	Bozic	Inf	unif	wholeT_0.01	J5:OT, J5:OT-A, S5:CBN-A, S5:OT, S5:OT-A
636	No	9	9-B	200	Bozic	Inf	unif	wholeT_0.5	J1:DiP, J1:DiP-A, J1:OT, J1:OT-A, S5:DiP, S5:DiP-A, S5:OT, S5:OT-A
637	No	9	9-B	200	exp	0	last	singleC	S1:OT, S1:OT-A
638	No	9	9-B	200	exp	0	last	wholeT_0.01	S1:OT, S1:OT-A
639	No	9	9-B	200	exp	0	last	wholeT_0.5	S1:OT, S1:OT-A
640	No	9	9-B	200	exp	0	unif	singleC	S1:DiP, S1:DiP-A, S1:OT, S1:OT-A, S5:DiP, S5:DiP-A, S5:OT, S5:OT-A
641	No	9	9-B	200	exp	0	unif	wholeT_0.01	S1:OT, S1:OT-A
642	No	9	9-B	200	exp	0	unif	wholeT_0.5	S1:DiP, S1:DiP-A, S1:OT, S1:OT-A, S5:DiP, S5:DiP-A, S5:OT, S5:OT-A
643	No	9	9-B	200	exp	Inf	last	singleC	S5:OT, S5:OT-A
644	No	9	9-B	200	exp	Inf	last	wholeT_0.01	J5:CBN-A, J5:OT, J5:OT-A
645	No	9	9-B	200	exp	Inf	last	wholeT_0.5	S5:OT, S5:OT-A
646	No	9	9-B	200	exp	Inf	unif	singleC	J1:OT, J1:OT-A, S1:OT, S1:OT-A, S5:DiP, S5:DiP-A, S5:OT, S5:OT-A
647	No	9	9-B	200	exp	Inf	unif	wholeT_0.01	S5:OT, S5:OT-A
648	No	9	9-B	200	exp	Inf	unif	wholeT_0.5	S1:OT, S1:OT-A

Table 5: *(continued)*

	Conjunction	Drivers	Tree	S.Size	Model	sh	S.Time	S.Type	Best method(s)
649	No	9	9-B	200	McF_4	0	last	singleC	S1:OT, S1:OT-A, S5:OT, S5:OT-A
650	No	9	9-B	200	McF_4	0	last	wholeT_0.01	S5:OT, S5:OT-A
651	No	9	9-B	200	McF_4	0	last	wholeT_0.5	S1:OT, S1:OT-A, S5:OT, S5:OT-A
652	No	9	9-B	200	McF_4	0	unif	singleC	S5:OT, S5:OT-A
653	No	9	9-B	200	McF_4	0	unif	wholeT_0.01	S5:OT, S5:OT-A
654	No	9	9-B	200	McF_4	0	unif	wholeT_0.5	S1:OT, S1:OT-A, S5:OT, S5:OT-A
655	No	9	9-B	200	McF_4	Inf	last	singleC	S1:OT, S1:OT-A, S5:OT, S5:OT-A
656	No	9	9-B	200	McF_4	Inf	last	wholeT_0.01	S5:OT, S5:OT-A
657	No	9	9-B	200	McF_4	Inf	last	wholeT_0.5	S1:OT, S1:OT-A, S5:OT, S5:OT-A
658	No	9	9-B	200	McF_4	Inf	unif	singleC	S1:OT, S1:OT-A, S5:OT, S5:OT-A
659	No	9	9-B	200	McF_4	Inf	unif	wholeT_0.01	S5:OT, S5:OT-A
660	No	9	9-B	200	McF_4	Inf	unif	wholeT_0.5	S1:OT, S1:OT-A, S5:OT, S5:OT-A
661	No	9	9-B	200	McF_6	0	last	singleC	S5:OT, S5:OT-A
662	No	9	9-B	200	McF_6	0	last	wholeT_0.01	J1:OT, J1:OT-A, S5:OT, S5:OT-A
663	No	9	9-B	200	McF_6	0	last	wholeT_0.5	S5:OT, S5:OT-A
664	No	9	9-B	200	McF_6	0	unif	singleC	S1:OT, S1:OT-A, S5:CBN, S5:CBN-A, S5:OT, S5:OT-A
665	No	9	9-B	200	McF_6	0	unif	wholeT_0.01	S5:OT, S5:OT-A
666	No	9	9-B	200	McF_6	0	unif	wholeT_0.5	S5:CBN-A, S5:OT, S5:OT-A
667	No	9	9-B	200	McF_6	Inf	last	singleC	S5:OT-A
668	No	9	9-B	200	McF_6	Inf	last	wholeT_0.01	S5:OT-A
669	No	9	9-B	200	McF_6	Inf	last	wholeT_0.5	S5:OT-A
670	No	9	9-B	200	McF_6	Inf	unif	singleC	S5:CBN, S5:OT, S5:OT-A
671	No	9	9-B	200	McF_6	Inf	unif	wholeT_0.01	S5:OT, S5:OT-A
672	No	9	9-B	200	McF_6	Inf	unif	wholeT_0.5	S1:OT, S1:OT-A, S5:CBN, S5:CBN-A, S5:OT, S5:OT-A
673	No	9	9-B	100	Bozic	0	last	singleC	S1:OT, S1:OT-A, S5:OT, S5:OT-A
674	No	9	9-B	100	Bozic	0	last	wholeT_0.01	S5:OT, S5:OT-A
675	No	9	9-B	100	Bozic	0	last	wholeT_0.5	J1:DiP, J1:DiP-A, J1:OT, J1:OT-A, S1:OT, S1:OT-A, S5:DiP, S5:DiP-A, S5:OT, S5:OT-A
676	No	9	9-B	100	Bozic	0	unif	singleC	S5:DiP, S5:DiP-A, S5:OT, S5:OT-A
677	No	9	9-B	100	Bozic	0	unif	wholeT_0.01	J1:OT, J1:OT-A, S5:DiP, S5:DiP-A, S5:OT, S5:OT-A
678	No	9	9-B	100	Bozic	0	unif	wholeT_0.5	S5:DiP, S5:DiP-A, S5:OT, S5:OT-A
679	No	9	9-B	100	Bozic	Inf	last	singleC	J1:OT, J1:OT-A, J5:OT, J5:OT-A, S5:OT, S5:OT-A
680	No	9	9-B	100	Bozic	Inf	last	wholeT_0.01	J5:OT, J5:OT-A
681	No	9	9-B	100	Bozic	Inf	last	wholeT_0.5	J1:OT, J1:OT-A, J5:OT, J5:OT-A, S5:OT, S5:OT-A

Table 5: *(continued)*

	Conjunction	Drivers	Tree	S.Size	Model	sh	S.Time	S.Type	Best method(s)
682	No	9	9-B	100	Bozic	Inf	unif	singleC	S5:DiP, S5:DiP-A, S5:OT, S5:OT-A
683	No	9	9-B	100	Bozic	Inf	unif	wholeT_0.01	J5:OT, J5:OT-A
684	No	9	9-B	100	Bozic	Inf	unif	wholeT_0.5	J1:OT, J1:OT-A, S5:DiP, S5:DiP-A, S5:OT, S5:OT-A
685	No	9	9-B	100	exp	0	last	singleC	S1:OT, S1:OT-A
686	No	9	9-B	100	exp	0	last	wholeT_0.01	S1:OT, S1:OT-A, S5:OT, S5:OT-A
687	No	9	9-B	100	exp	0	last	wholeT_0.5	S1:OT, S1:OT-A
688	No	9	9-B	100	exp	0	unif	singleC	S1:DiP, S1:DiP-A, S1:OT, S1:OT-A, S5:DiP, S5:DiP-A, S5:OT, S5:OT-A
689	No	9	9-B	100	exp	0	unif	wholeT_0.01	J1:OT, J1:OT-A, S1:OT, S1:OT-A, S5:DiP, S5:DiP-A, S5:OT, S5:OT-A
690	No	9	9-B	100	exp	0	unif	wholeT_0.5	S1:DiP, S1:DiP-A, S1:OT, S1:OT-A, S5:DiP, S5:DiP-A, S5:OT, S5:OT-A
691	No	9	9-B	100	exp	Inf	last	singleC	S5:OT, S5:OT-A
692	No	9	9-B	100	exp	Inf	last	wholeT_0.01	J5:CBN-A, J5:OT, J5:OT-A
693	No	9	9-B	100	exp	Inf	last	wholeT_0.5	S5:OT, S5:OT-A
694	No	9	9-B	100	exp	Inf	unif	singleC	J1:OT, J1:OT-A, S5:DiP, S5:DiP-A, S5:OT, S5:OT-A
695	No	9	9-B	100	exp	Inf	unif	wholeT_0.01	S5:OT, S5:OT-A
696	No	9	9-B	100	exp	Inf	unif	wholeT_0.5	J1:OT, J1:OT-A, S5:DiP, S5:DiP-A, S5:OT, S5:OT-A
697	No	9	9-B	100	McF_4	0	last	singleC	S5:OT, S5:OT-A
698	No	9	9-B	100	McF_4	0	last	wholeT_0.01	S5:OT, S5:OT-A
699	No	9	9-B	100	McF_4	0	last	wholeT_0.5	S5:OT, S5:OT-A
700	No	9	9-B	100	McF_4	0	unif	singleC	S5:OT, S5:OT-A
701	No	9	9-B	100	McF_4	0	unif	wholeT_0.01	S5:OT, S5:OT-A
702	No	9	9-B	100	McF_4	0	unif	wholeT_0.5	S5:OT, S5:OT-A
703	No	9	9-B	100	McF_4	Inf	last	singleC	S5:OT, S5:OT-A
704	No	9	9-B	100	McF_4	Inf	last	wholeT_0.01	S5:OT, S5:OT-A
705	No	9	9-B	100	McF_4	Inf	last	wholeT_0.5	S5:OT, S5:OT-A
706	No	9	9-B	100	McF_4	Inf	unif	singleC	S5:OT, S5:OT-A
707	No	9	9-B	100	McF_4	Inf	unif	wholeT_0.01	S5:OT, S5:OT-A
708	No	9	9-B	100	McF_4	Inf	unif	wholeT_0.5	S5:OT, S5:OT-A
709	No	9	9-B	100	McF_6	0	last	singleC	S5:OT, S5:OT-A
710	No	9	9-B	100	McF_6	0	last	wholeT_0.01	S5:OT, S5:OT-A
711	No	9	9-B	100	McF_6	0	last	wholeT_0.5	S5:OT, S5:OT-A
712	No	9	9-B	100	McF_6	0	unif	singleC	S5:CBN-A, S5:OT, S5:OT-A
713	No	9	9-B	100	McF_6	0	unif	wholeT_0.01	S5:CBN, S5:OT, S5:OT-A
714	No	9	9-B	100	McF_6	0	unif	wholeT_0.5	S5:CBN, S5:CBN-A, S5:OT, S5:OT-A
715	No	9	9-B	100	McF_6	Inf	last	singleC	S5:OT-A

Table 5: *(continued)*

	Conjunction	Drivers	Tree	S.Size	Model	sh	S.Time	S.Type	Best method(s)
716	No	9	9-B	100	McF_6	Inf	last	wholeT_0.01	S5:OT-A
717	No	9	9-B	100	McF_6	Inf	last	wholeT_0.5	S5:OT-A
718	No	9	9-B	100	McF_6	Inf	unif	singleC	S5:OT, S5:OT-A
719	No	9	9-B	100	McF_6	Inf	unif	wholeT_0.01	S5:CBN, S5:CBN-A, S5:OT, S5:OT-A
720	No	9	9-B	100	McF_6	Inf	unif	wholeT_0.5	S5:CBN, S5:CBN-A, S5:OT, S5:OT-A
721	No	7	7-B	1000	Bozic	0	last	singleC	S1:OT-A, S5:OT-A
722	No	7	7-B	1000	Bozic	0	last	wholeT_0.01	J1:DiP-A, J1:OT-A, J5:DiP-A, J5:OT-A, S1:DiP-A, S1:OT-A, S5:DiP-A, S5:OT-A
723	No	7	7-B	1000	Bozic	0	last	wholeT_0.5	S1:OT-A
724	No	7	7-B	1000	Bozic	0	unif	singleC	S1:CBN-A, S1:OT, S1:OT-A
725	No	7	7-B	1000	Bozic	0	unif	wholeT_0.01	J5:CBN, J5:CBN-A, J5:DiP-A, J5:OT, J5:OT-A
726	No	7	7-B	1000	Bozic	0	unif	wholeT_0.5	J1:CBN, J1:CBN-A, J1:DiP, J1:DiP-A, J1:OT, J1:OT-A, J5:DiP, J5:DiP-A, S1:CBN, S1:CBN-A, S1:OT, S1:OT-A, S5:DiP, S5:DiP-A
727	No	7	7-B	1000	Bozic	Inf	last	singleC	J1:DiP-A, J1:OT-A, J5:DiP-A, J5:OT-A, S5:DiP-A, S5:OT-A
728	No	7	7-B	1000	Bozic	Inf	last	wholeT_0.01	J5:CBN-A, J5:DiP-A, J5:OT-A
729	No	7	7-B	1000	Bozic	Inf	last	wholeT_0.5	J1:DiP-A, J1:OT-A, S5:DiP-A, S5:OT-A
730	No	7	7-B	1000	Bozic	Inf	unif	singleC	S1:CBN, S1:CBN-A
731	No	7	7-B	1000	Bozic	Inf	unif	wholeT_0.01	J1:OT, J1:OT-A, J5:CBN, J5:CBN-A, J5:DiP-A, J5:OT, J5:OT-A, S5:CBN, S5:CBN-A, S5:DiP-A, S5:OT, S5:OT-A
732	No	7	7-B	1000	Bozic	Inf	unif	wholeT_0.5	S1:CBN, S1:CBN-A
733	No	7	7-B	1000	exp	0	last	singleC	S1:OT-A
734	No	7	7-B	1000	exp	0	last	wholeT_0.01	J1:OT-A, S1:OT-A, S5:OT-A
735	No	7	7-B	1000	exp	0	last	wholeT_0.5	S1:OT-A
736	No	7	7-B	1000	exp	0	unif	singleC	J1:DiP, J1:DiP-A, J5:DiP, J5:DiP-A, S1:DiP, S1:DiP-A, S5:DiP, S5:DiP-A
737	No	7	7-B	1000	exp	0	unif	wholeT_0.01	J1:CBN, J1:CBN-A, J1:OT, J1:OT-A, S1:CBN-A
738	No	7	7-B	1000	exp	0	unif	wholeT_0.5	J1:DiP, J1:DiP-A, J5:DiP, J5:DiP-A, S1:DiP, S1:DiP-A, S5:DiP, S5:DiP-A
739	No	7	7-B	1000	exp	Inf	last	singleC	J1:OT-A, S1:OT-A, S5:OT-A
740	No	7	7-B	1000	exp	Inf	last	wholeT_0.01	S5:OT-A
741	No	7	7-B	1000	exp	Inf	last	wholeT_0.5	S1:OT-A, S5:OT-A
742	No	7	7-B	1000	exp	Inf	unif	singleC	S1:CBN, S1:CBN-A

Table 5: *(continued)*

	Conjunction	Drivers	Tree	S.Size	Model	sh	S.Time	S.Type	Best method(s)
743	No	7	7-B	1000	exp	Inf	unif	wholeT_0.01	J1:CBN, J1:CBN-A, J1:OT, J1:OT-A, J5:CBN, J5:CBN-A, J5:OT, J5:OT-A, S5:CBN, S5:CBN-A, S5:OT, S5:OT-A
744	No	7	7-B	1000	exp	Inf	unif	wholeT_0.5	S1:CBN, S1:CBN-A, S1:OT, S1:OT-A
745	No	7	7-B	1000	McF_4	0	last	singleC	S1:DiP-A, S1:OT-A, S5:DiP-A, S5:OT-A
746	No	7	7-B	1000	McF_4	0	last	wholeT_0.01	S5:DiP-A, S5:OT-A
747	No	7	7-B	1000	McF_4	0	last	wholeT_0.5	S1:DiP-A, S1:OT-A, S5:DiP-A, S5:OT-A
748	No	7	7-B	1000	McF_4	0	unif	singleC	S1:DiP, S1:DiP-A, S1:OT, S1:OT-A
749	No	7	7-B	1000	McF_4	0	unif	wholeT_0.01	J1:DiP-A, J1:OT, J1:OT-A, S5:DiP-A, S5:OT, S5:OT-A
750	No	7	7-B	1000	McF_4	0	unif	wholeT_0.5	S1:DiP-A, S1:OT, S1:OT-A
751	No	7	7-B	1000	McF_4	Inf	last	singleC	S1:DiP-A, S1:OT-A, S5:DiP-A, S5:OT-A
752	No	7	7-B	1000	McF_4	Inf	last	wholeT_0.01	S5:DiP-A, S5:OT-A
753	No	7	7-B	1000	McF_4	Inf	last	wholeT_0.5	S1:DiP-A, S1:OT-A, S5:DiP-A, S5:OT-A
754	No	7	7-B	1000	McF_4	Inf	unif	singleC	S1:OT, S1:OT-A
755	No	7	7-B	1000	McF_4	Inf	unif	wholeT_0.01	J1:OT, J1:OT-A, S5:OT, S5:OT-A
756	No	7	7-B	1000	McF_4	Inf	unif	wholeT_0.5	S1:OT, S1:OT-A
757	No	7	7-B	1000	McF_6	0	last	singleC	J1:DiP-A, J1:OT-A, S5:DiP-A, S5:OT-A
758	No	7	7-B	1000	McF_6	0	last	wholeT_0.01	J5:DiP-A, J5:OT-A, S5:DiP-A, S5:OT-A
759	No	7	7-B	1000	McF_6	0	last	wholeT_0.5	J1:DiP-A, J1:OT-A, S1:DiP-A, S1:OT-A, S5:DiP-A, S5:OT-A
760	No	7	7-B	1000	McF_6	0	unif	singleC	S1:DiP, S1:DiP-A, S1:OT, S1:OT-A, S5:DiP, S5:DiP-A, S5:OT, S5:OT-A
761	No	7	7-B	1000	McF_6	0	unif	wholeT_0.01	S5:DiP, S5:DiP-A, S5:OT, S5:OT-A
762	No	7	7-B	1000	McF_6	0	unif	wholeT_0.5	S1:DiP, S1:DiP-A, S1:OT, S1:OT-A, S5:DiP, S5:DiP-A, S5:OT, S5:OT-A
763	No	7	7-B	1000	McF_6	Inf	last	singleC	J1:DiP-A, J1:OT-A, S5:DiP-A, S5:OT-A
764	No	7	7-B	1000	McF_6	Inf	last	wholeT_0.01	J5:DiP-A, J5:OT-A, S5:DiP-A, S5:OT-A
765	No	7	7-B	1000	McF_6	Inf	last	wholeT_0.5	J1:DiP-A, J1:OT-A, S5:DiP-A, S5:OT-A
766	No	7	7-B	1000	McF_6	Inf	unif	singleC	S1:DiP-A, S1:OT, S1:OT-A, S5:DiP-A, S5:OT, S5:OT-A
767	No	7	7-B	1000	McF_6	Inf	unif	wholeT_0.01	S5:DiP-A, S5:OT, S5:OT-A
768	No	7	7-B	1000	McF_6	Inf	unif	wholeT_0.5	S1:DiP-A, S1:OT, S1:OT-A, S5:DiP-A, S5:OT, S5:OT-A
769	No	7	7-B	200	Bozic	0	last	singleC	S1:OT-A, S5:OT-A
770	No	7	7-B	200	Bozic	0	last	wholeT_0.01	J1:OT-A, J5:OT-A, S5:OT-A
771	No	7	7-B	200	Bozic	0	last	wholeT_0.5	S1:OT-A
772	No	7	7-B	200	Bozic	0	unif	singleC	J1:CBN, J1:CBN-A, J1:OT, J1:OT-A, S1:CBN, S1:OT, S1:OT-A
773	No	7	7-B	200	Bozic	0	unif	wholeT_0.01	J5:CBN, J5:CBN-A, J5:OT, J5:OT-A

Table 5: *(continued)*

	Conjunction	Drivers	Tree	S.Size	Model	sh	S.Time	S.Type	Best method(s)
774	No	7	7-B	200	Bozic	0	unif	wholeT_0.5	J1:CBN, J1:CBN-A, J1:OT, J1:OT-A, J5:DiP, J5:DiP-A, S1:CBN, S1:CBN-A, S1:OT, S1:OT-A, S5:DiP, S5:DiP-A
775	No	7	7-B	200	Bozic	Inf	last	singleC	S5:OT-A
776	No	7	7-B	200	Bozic	Inf	last	wholeT_0.01	J5:CBN-A, J5:OT-A
777	No	7	7-B	200	Bozic	Inf	last	wholeT_0.5	S5:OT-A
778	No	7	7-B	200	Bozic	Inf	unif	singleC	J1:CBN, J1:CBN-A, S5:CBN, S5:CBN-A, S5:OT, S5:OT-A
779	No	7	7-B	200	Bozic	Inf	unif	wholeT_0.01	J5:CBN, J5:CBN-A, J5:OT, J5:OT-A, S5:CBN, S5:CBN-A, S5:OT, S5:OT-A
780	No	7	7-B	200	Bozic	Inf	unif	wholeT_0.5	J1:CBN, J1:CBN-A
781	No	7	7-B	200	exp	0	last	singleC	S1:OT-A
782	No	7	7-B	200	exp	0	last	wholeT_0.01	J1:OT-A, S1:OT-A, S5:OT-A
783	No	7	7-B	200	exp	0	last	wholeT_0.5	S1:OT-A
784	No	7	7-B	200	exp	0	unif	singleC	J1:CBN, J1:CBN-A, J1:OT, J1:OT-A, J5:DiP, J5:DiP-A, S1:CBN, S1:CBN-A, S1:OT, S1:OT-A, S5:DiP, S5:DiP-A
785	No	7	7-B	200	exp	0	unif	wholeT_0.01	J1:CBN, J1:CBN-A, J1:OT, J1:OT-A, S1:CBN-A, S1:OT, S1:OT-A
786	No	7	7-B	200	exp	0	unif	wholeT_0.5	J1:CBN, J1:CBN-A, J1:DiP, J1:DiP-A, J1:OT, J1:OT-A, J5:DiP, J5:DiP-A, S1:CBN, S1:CBN-A, S1:DiP, S1:DiP-A, S1:OT, S1:OT-A, S5:DiP, S5:DiP-A
787	No	7	7-B	200	exp	Inf	last	singleC	J1:OT-A, S5:OT-A
788	No	7	7-B	200	exp	Inf	last	wholeT_0.01	J5:OT-A, S5:OT-A
789	No	7	7-B	200	exp	Inf	last	wholeT_0.5	S5:OT-A
790	No	7	7-B	200	exp	Inf	unif	singleC	J1:CBN, J1:CBN-A, J1:OT, J1:OT-A, S1:CBN, S1:CBN-A, S1:OT, S1:OT-A
791	No	7	7-B	200	exp	Inf	unif	wholeT_0.01	J5:CBN, J5:CBN-A, J5:OT, J5:OT-A, S5:OT, S5:OT-A
792	No	7	7-B	200	exp	Inf	unif	wholeT_0.5	S1:CBN, S1:CBN-A, S1:OT, S1:OT-A
793	No	7	7-B	200	McF_4	0	last	singleC	S5:OT-A
794	No	7	7-B	200	McF_4	0	last	wholeT_0.01	S5:OT-A
795	No	7	7-B	200	McF_4	0	last	wholeT_0.5	S5:OT-A
796	No	7	7-B	200	McF_4	0	unif	singleC	J1:OT, J1:OT-A, S1:OT-A, S5:OT, S5:OT-A
797	No	7	7-B	200	McF_4	0	unif	wholeT_0.01	J1:OT, J1:OT-A, S5:OT, S5:OT-A
798	No	7	7-B	200	McF_4	0	unif	wholeT_0.5	J1:OT, J1:OT-A, S1:OT, S1:OT-A, S5:OT, S5:OT-A
799	No	7	7-B	200	McF_4	Inf	last	singleC	S5:OT-A

Table 5: *(continued)*

	Conjunction	Drivers	Tree	S.Size	Model	sh	S.Time	S.Type	Best method(s)
800	No	7	7-B	200	McF_4	Inf	last	wholeT_0.01	S5:OT-A
801	No	7	7-B	200	McF_4	Inf	last	wholeT_0.5	S5:OT-A
802	No	7	7-B	200	McF_4	Inf	unif	singleC	J1:OT, J1:OT-A, S5:OT, S5:OT-A
803	No	7	7-B	200	McF_4	Inf	unif	wholeT_0.01	J1:OT, J1:OT-A, S5:OT, S5:OT-A
804	No	7	7-B	200	McF_4	Inf	unif	wholeT_0.5	J1:OT, J1:OT-A, S1:OT, S1:OT-A, S5:OT, S5:OT-A
805	No	7	7-B	200	McF_6	0	last	singleC	J1:OT-A, J5:OT-A, S5:OT-A
806	No	7	7-B	200	McF_6	0	last	wholeT_0.01	J5:OT-A, S5:OT-A
807	No	7	7-B	200	McF_6	0	last	wholeT_0.5	J1:OT-A, S5:OT-A
808	No	7	7-B	200	McF_6	0	unif	singleC	S5:OT, S5:OT-A
809	No	7	7-B	200	McF_6	0	unif	wholeT_0.01	S5:OT, S5:OT-A
810	No	7	7-B	200	McF_6	0	unif	wholeT_0.5	S5:CBN-A, S5:OT, S5:OT-A
811	No	7	7-B	200	McF_6	Inf	last	singleC	J1:OT-A, J5:OT-A, S5:OT-A
812	No	7	7-B	200	McF_6	Inf	last	wholeT_0.01	J5:OT-A, S5:OT-A
813	No	7	7-B	200	McF_6	Inf	last	wholeT_0.5	J1:OT-A, S5:OT-A
814	No	7	7-B	200	McF_6	Inf	unif	singleC	S5:CBN-A, S5:OT, S5:OT-A
815	No	7	7-B	200	McF_6	Inf	unif	wholeT_0.01	S5:CBN-A, S5:OT, S5:OT-A
816	No	7	7-B	200	McF_6	Inf	unif	wholeT_0.5	J1:CBN-A, J1:OT-A, J5:CBN-A, S5:CBN-A, S5:OT, S5:OT-A
817	No	7	7-B	100	Bozic	0	last	singleC	S5:OT-A
818	No	7	7-B	100	Bozic	0	last	wholeT_0.01	J1:OT-A, J5:OT-A, S5:OT-A
819	No	7	7-B	100	Bozic	0	last	wholeT_0.5	S1:OT-A, S5:OT-A
820	No	7	7-B	100	Bozic	0	unif	singleC	J1:CBN, J1:CBN-A, J1:OT, J1:OT-A, S1:OT, S1:OT-A
821	No	7	7-B	100	Bozic	0	unif	wholeT_0.01	J5:CBN, J5:CBN-A, J5:OT, J5:OT-A
822	No	7	7-B	100	Bozic	0	unif	wholeT_0.5	J1:CBN, J1:CBN-A, J1:OT, J1:OT-A, J5:DiP, J5:DiP-A, S1:OT, S1:OT-A, S5:DiP, S5:DiP-A
823	No	7	7-B	100	Bozic	Inf	last	singleC	J1:OT-A, S5:OT-A
824	No	7	7-B	100	Bozic	Inf	last	wholeT_0.01	J5:CBN-A, J5:OT-A
825	No	7	7-B	100	Bozic	Inf	last	wholeT_0.5	S5:OT-A
826	No	7	7-B	100	Bozic	Inf	unif	singleC	J1:CBN-A, S5:CBN, S5:CBN-A
827	No	7	7-B	100	Bozic	Inf	unif	wholeT_0.01	J5:CBN, J5:CBN-A, J5:OT, J5:OT-A
828	No	7	7-B	100	Bozic	Inf	unif	wholeT_0.5	J1:CBN, J1:CBN-A, S5:CBN, S5:CBN-A, S5:OT, S5:OT-A
829	No	7	7-B	100	exp	0	last	singleC	S1:OT-A
830	No	7	7-B	100	exp	0	last	wholeT_0.01	J1:CBN, J1:CBN-A, J1:OT-A, J5:CBN-A, J5:OT-A, S1:OT-A, S5:OT-A
831	No	7	7-B	100	exp	0	last	wholeT_0.5	S1:OT-A

Table 5: *(continued)*

	Conjunction	Drivers	Tree	S.Size	Model	sh	S.Time	S.Type	Best method(s)
832	No	7	7-B	100	exp	0	unif	singleC	J1:CBN, J1:CBN-A, J1:OT, J1:OT-A, J5:DiP, J5:DiP-A, S1:CBN, S1:CBN-A, S1:OT, S1:OT-A, S5:DiP, S5:DiP-A
833	No	7	7-B	100	exp	0	unif	wholeT_0.01	J1:CBN, J1:CBN-A, J1:OT, J1:OT-A, J5:DiP, J5:DiP-A, S1:OT, S1:OT-A, S5:CBN, S5:CBN-A, S5:DiP, S5:DiP-A
834	No	7	7-B	100	exp	0	unif	wholeT_0.5	J1:CBN, J1:CBN-A, J1:OT, J1:OT-A, J5:DiP, J5:DiP-A, S1:CBN, S1:CBN-A, S1:OT, S1:OT-A, S5:DiP, S5:DiP-A
835	No	7	7-B	100	exp	Inf	last	singleC	S5:OT-A
836	No	7	7-B	100	exp	Inf	last	wholeT_0.01	J5:OT-A
837	No	7	7-B	100	exp	Inf	last	wholeT_0.5	S5:OT-A
838	No	7	7-B	100	exp	Inf	unif	singleC	J1:CBN, J1:CBN-A, J1:OT, J1:OT-A, J5:CBN, J5:CBN-A, J5:OT, J5:OT-A, S1:OT, S1:OT-A, S5:CBN, S5:CBN-A, S5:OT, S5:OT-A
839	No	7	7-B	100	exp	Inf	unif	wholeT_0.01	J5:CBN, J5:CBN-A, J5:OT, J5:OT-A, S5:OT, S5:OT-A
840	No	7	7-B	100	exp	Inf	unif	wholeT_0.5	J1:CBN, J1:CBN-A, S1:OT, S1:OT-A
841	No	7	7-B	100	McF_4	0	last	singleC	S5:OT-A
842	No	7	7-B	100	McF_4	0	last	wholeT_0.01	S5:OT-A
843	No	7	7-B	100	McF_4	0	last	wholeT_0.5	S5:OT-A
844	No	7	7-B	100	McF_4	0	unif	singleC	J1:OT, J1:OT-A, S5:OT, S5:OT-A
845	No	7	7-B	100	McF_4	0	unif	wholeT_0.01	J1:OT-A, S5:OT, S5:OT-A
846	No	7	7-B	100	McF_4	0	unif	wholeT_0.5	J1:OT, J1:OT-A, S5:OT, S5:OT-A
847	No	7	7-B	100	McF_4	Inf	last	singleC	S5:OT-A
848	No	7	7-B	100	McF_4	Inf	last	wholeT_0.01	S5:OT-A
849	No	7	7-B	100	McF_4	Inf	last	wholeT_0.5	S5:OT-A
850	No	7	7-B	100	McF_4	Inf	unif	singleC	J1:OT, J1:OT-A, S5:OT, S5:OT-A
851	No	7	7-B	100	McF_4	Inf	unif	wholeT_0.01	J1:OT-A, S5:OT, S5:OT-A
852	No	7	7-B	100	McF_4	Inf	unif	wholeT_0.5	J1:OT, J1:OT-A, J5:CBN-A, S5:OT, S5:OT-A
853	No	7	7-B	100	McF_6	0	last	singleC	J5:OT-A, S5:OT-A
854	No	7	7-B	100	McF_6	0	last	wholeT_0.01	J5:OT-A, S5:OT-A
855	No	7	7-B	100	McF_6	0	last	wholeT_0.5	S5:OT-A
856	No	7	7-B	100	McF_6	0	unif	singleC	J1:CBN-A, J1:OT, J1:OT-A, J5:CBN-A, S5:CBN-A, S5:OT, S5:OT-A
857	No	7	7-B	100	McF_6	0	unif	wholeT_0.01	S5:CBN-A, S5:OT, S5:OT-A
858	No	7	7-B	100	McF_6	0	unif	wholeT_0.5	J1:CBN-A, S5:CBN-A, S5:OT, S5:OT-A
859	No	7	7-B	100	McF_6	Inf	last	singleC	S5:OT-A

Table 5: *(continued)*

	Conjunction	Drivers	Tree	S.Size	Model	sh	S.Time	S.Type	Best method(s)
860	No	7	7-B	100	McF_6	Inf	last	wholeT_0.01	J5:OT-A, S5:OT-A
861	No	7	7-B	100	McF_6	Inf	last	wholeT_0.5	J1:OT-A, S5:OT-A
862	No	7	7-B	100	McF_6	Inf	unif	singleC	J1:CBN-A, J5:CBN-A, S5:CBN-A, S5:OT, S5:OT-A
863	No	7	7-B	100	McF_6	Inf	unif	wholeT_0.01	J1:CBN-A, J5:CBN-A, S5:CBN-A, S5:OT-A
864	No	7	7-B	100	McF_6	Inf	unif	wholeT_0.5	J1:CBN-A, J5:CBN-A, S5:CBN-A, S5:OT, S5:OT-A

3.2 Confidence sets (MCB-2), PFD, Drivers Unknown

Table 6: Confidence sets (method MCB-2) when Drivers are Unknown for measure PFD.

	Conjunction	Drivers	Tree	S.Size	Model	sh	S.Time	S.Type	Best method(s)
1	Yes	11	11-A	1000	Bozic	0	last	singleC	S1:OT, S1:OT-A
2	Yes	11	11-A	1000	Bozic	0	last	wholeT_0.01	J1:DiP, J1:DiP-A, J5:DiP, J5:DiP-A, S1:DiP, S1:DiP-A, S5:DiP, S5:DiP-A
3	Yes	11	11-A	1000	Bozic	0	last	wholeT_0.5	S1:OT, S1:OT-A
4	Yes	11	11-A	1000	Bozic	0	unif	singleC	S1:CBN, S1:CBN-A
5	Yes	11	11-A	1000	Bozic	0	unif	wholeT_0.01	J1:CBN-A, J1:DiP-A, S5:CBN, S5:CBN-A, S5:DiP, S5:DiP-A, S5:OT, S5:OT-A
6	Yes	11	11-A	1000	Bozic	0	unif	wholeT_0.5	S1:CBN, S1:CBN-A, S5:CBN, S5:CBN-A
7	Yes	11	11-A	1000	Bozic	Inf	last	singleC	J1:DiP, J1:DiP-A, J1:OT, J1:OT-A, J5:DiP, J5:DiP-A, J5:OT, J5:OT-A, S1:DiP-A, S5:DiP, S5:DiP-A, S5:OT, S5:OT-A
8	Yes	11	11-A	1000	Bozic	Inf	last	wholeT_0.01	J5:DiP, J5:DiP-A, J5:OT, J5:OT-A
9	Yes	11	11-A	1000	Bozic	Inf	last	wholeT_0.5	J1:DiP, J1:DiP-A, J1:OT, J1:OT-A, J5:DiP, J5:DiP-A, J5:OT, J5:OT-A, S5:DiP, S5:DiP-A, S5:OT, S5:OT-A
10	Yes	11	11-A	1000	Bozic	Inf	unif	singleC	J1:CBN, J1:CBN-A, S1:CBN, S1:CBN-A, S1:DiP, S1:DiP-A, S1:OT, S1:OT-A
11	Yes	11	11-A	1000	Bozic	Inf	unif	wholeT_0.01	J1:CBN, J1:CBN-A, S5:CBN-A, S5:OT, S5:OT-A
12	Yes	11	11-A	1000	Bozic	Inf	unif	wholeT_0.5	S1:CBN, S1:CBN-A, S1:DiP-A, S1:OT, S1:OT-A
13	Yes	11	11-A	1000	exp	0	last	singleC	S1:OT, S1:OT-A
14	Yes	11	11-A	1000	exp	0	last	wholeT_0.01	J1:DiP, J1:DiP-A, J1:OT, J1:OT-A, S1:DiP-A, S1:OT, S1:OT-A, S5:DiP-A, S5:OT, S5:OT-A
15	Yes	11	11-A	1000	exp	0	last	wholeT_0.5	S1:OT, S1:OT-A
16	Yes	11	11-A	1000	exp	0	unif	singleC	S1:CBN, S1:CBN-A, S5:CBN, S5:CBN-A
17	Yes	11	11-A	1000	exp	0	unif	wholeT_0.01	S1:DiP, S1:DiP-A, S1:OT, S1:OT-A
18	Yes	11	11-A	1000	exp	0	unif	wholeT_0.5	S1:CBN, S1:CBN-A, S5:CBN, S5:CBN-A
19	Yes	11	11-A	1000	exp	Inf	last	singleC	J1:DiP, J1:DiP-A, J1:OT, J1:OT-A, S5:DiP, S5:DiP-A, S5:OT, S5:OT-A
20	Yes	11	11-A	1000	exp	Inf	last	wholeT_0.01	J5:DiP, J5:DiP-A, J5:OT, J5:OT-A, S5:DiP, S5:DiP-A, S5:OT, S5:OT-A
21	Yes	11	11-A	1000	exp	Inf	last	wholeT_0.5	J1:DiP, J1:DiP-A, J1:OT, J1:OT-A, S1:DiP-A, S5:DiP, S5:DiP-A, S5:OT, S5:OT-A

Table 6: *(continued)*

	Conjunction	Drivers	Tree	S.Size	Model	sh	S.Time	S.Type	Best method(s)
22	Yes	11	11-A	1000	exp	Inf	unif	singleC	S1:CBN, S1:CBN-A, S1:OT, S1:OT-A
23	Yes	11	11-A	1000	exp	Inf	unif	wholeT_0.01	J1:CBN-A, J1:DiP, J1:DiP-A, J1:OT, J1:OT-A
24	Yes	11	11-A	1000	exp	Inf	unif	wholeT_0.5	S1:CBN, S1:CBN-A, S1:OT, S1:OT-A
25	Yes	11	11-A	1000	McF_4	0	last	singleC	J1:CBN, J1:DiP, J1:DiP-A, J1:OT, J1:OT-A, J5:CBN, J5:DiP, J5:DiP-A, J5:OT, J5:OT-A, S1:DiP-A, S1:OT, S1:OT-A, S5:DiP, S5:DiP-A, S5:OT, S5:OT-A
26	Yes	11	11-A	1000	McF_4	0	last	wholeT_0.01	J1:DiP, J1:DiP-A, J1:OT, J1:OT-A, J5:CBN, J5:DiP, J5:DiP-A, J5:OT, J5:OT-A, S1:DiP-A, S5:DiP, S5:DiP-A, S5:OT, S5:OT-A
27	Yes	11	11-A	1000	McF_4	0	last	wholeT_0.5	J1:DiP, J1:DiP-A, J1:OT, J1:OT-A, J5:CBN, J5:DiP, J5:DiP-A, J5:OT, J5:OT-A, S1:DiP-A, S1:OT, S1:OT-A, S5:DiP, S5:DiP-A, S5:OT, S5:OT-A
28	Yes	11	11-A	1000	McF_4	0	unif	singleC	J1:CBN, J1:CBN-A, J1:DiP, J1:DiP-A, J1:OT, J1:OT-A, S1:DiP, S1:DiP-A, S1:OT, S1:OT-A, S5:DiP, S5:DiP-A, S5:OT, S5:OT-A
29	Yes	11	11-A	1000	McF_4	0	unif	wholeT_0.01	J1:CBN-A, J1:DiP, J1:DiP-A, J1:OT, J1:OT-A, S1:DiP-A, S5:CBN-A, S5:DiP, S5:DiP-A, S5:OT, S5:OT-A
30	Yes	11	11-A	1000	McF_4	0	unif	wholeT_0.5	J1:CBN, J1:CBN-A, J1:DiP, J1:DiP-A, J1:OT, J1:OT-A, S1:DiP, S1:DiP-A, S1:OT, S1:OT-A, S5:CBN-A, S5:DiP, S5:DiP-A, S5:OT, S5:OT-A
31	Yes	11	11-A	1000	McF_4	Inf	last	singleC	J1:DiP, J1:DiP-A, J1:OT, J1:OT-A, J5:DiP, J5:DiP-A, J5:OT, J5:OT-A, S1:OT, S1:OT-A, S5:DiP, S5:DiP-A, S5:OT, S5:OT-A
32	Yes	11	11-A	1000	McF_4	Inf	last	wholeT_0.01	J1:DiP, J1:DiP-A, J1:OT, J1:OT-A, J5:DiP, J5:DiP-A, J5:OT, J5:OT-A, S5:DiP, S5:DiP-A, S5:OT, S5:OT-A
33	Yes	11	11-A	1000	McF_4	Inf	last	wholeT_0.5	J1:DiP, J1:DiP-A, J1:OT, J1:OT-A, J5:DiP, J5:DiP-A, J5:OT, J5:OT-A, S1:DiP-A, S1:OT, S1:OT-A, S5:DiP, S5:DiP-A, S5:OT, S5:OT-A

Table 6: *(continued)*

	Conjunction	Drivers	Tree	S.Size	Model	sh	S.Time	S.Type	Best method(s)
34	Yes	11	11-A	1000	McF_4	Inf	unif	singleC	J1:CBN-A, J1:DiP, J1:DiP-A, J1:OT, J1:OT-A, S1:DiP, S1:DiP-A, S1:OT, S1:OT-A, S5:CBN-A, S5:DiP, S5:DiP-A, S5:OT, S5:OT-A
35	Yes	11	11-A	1000	McF_4	Inf	unif	wholeT_0.01	J1:CBN-A, J1:DiP, J1:DiP-A, J1:OT, J1:OT-A, J5:DiP, J5:DiP-A, J5:OT, J5:OT-A, S5:CBN-A, S5:DiP, S5:DiP-A, S5:OT, S5:OT-A
36	Yes	11	11-A	1000	McF_4	Inf	unif	wholeT_0.5	J1:CBN-A, J1:DiP, J1:DiP-A, J1:OT, J1:OT-A, S1:DiP, S1:DiP-A, S1:OT, S1:OT-A, S5:CBN-A, S5:DiP, S5:DiP-A, S5:OT, S5:OT-A
37	Yes	11	11-A	1000	McF_6	0	last	singleC	J1:DiP, J1:OT, J5:DiP, J5:OT, S1:DiP, S1:OT, S5:DiP, S5:OT
38	Yes	11	11-A	1000	McF_6	0	last	wholeT_0.01	J1:DiP, J1:OT, J5:DiP, J5:OT, S1:DiP, S5:DiP, S5:OT
39	Yes	11	11-A	1000	McF_6	0	last	wholeT_0.5	J1:DiP, J1:OT, J5:DiP, J5:OT, S1:DiP, S1:OT, S5:DiP, S5:OT
40	Yes	11	11-A	1000	McF_6	0	unif	singleC	J1:CBN, J1:CBN-A, J1:DiP, J1:DiP-A, J1:OT, J1:OT-A, J5:CBN, J5:CBN-A, J5:DiP, J5:DiP-A, J5:OT, J5:OT-A, S1:DiP, S1:DiP-A, S1:OT, S1:OT-A, S5:CBN, S5:CBN-A, S5:DiP, S5:DiP-A, S5:OT, S5:OT-A
41	Yes	11	11-A	1000	McF_6	0	unif	wholeT_0.01	J1:CBN, J1:CBN-A, J1:DiP, J1:DiP-A, J1:OT, J1:OT-A, J5:CBN, J5:CBN-A, J5:DiP, J5:DiP-A, J5:OT, J5:OT-A, S1:DiP, S1:DiP-A, S5:CBN, S5:CBN-A, S5:DiP, S5:DiP-A, S5:OT, S5:OT-A
42	Yes	11	11-A	1000	McF_6	0	unif	wholeT_0.5	J1:CBN, J1:CBN-A, J1:DiP, J1:DiP-A, J1:OT, J1:OT-A, J5:CBN, J5:CBN-A, J5:DiP, J5:DiP-A, J5:OT, J5:OT-A, S1:DiP, S1:DiP-A, S1:OT, S1:OT-A, S5:CBN, S5:CBN-A, S5:DiP, S5:DiP-A, S5:OT, S5:OT-A
43	Yes	11	11-A	1000	McF_6	Inf	last	singleC	J1:DiP, J1:OT, J5:DiP, J5:OT, S1:DiP, S5:DiP, S5:OT
44	Yes	11	11-A	1000	McF_6	Inf	last	wholeT_0.01	J1:DiP, J1:OT, J5:DiP, J5:OT, S1:DiP, S5:DiP, S5:OT

Table 6: *(continued)*

	Conjunction	Drivers	Tree	S.Size	Model	sh	S.Time	S.Type	Best method(s)
45	Yes	11	11-A	1000	McF_6	Inf	last	wholeT_0.5	J1:DiP, J1:OT, J5:DiP, J5:OT, S1:DiP, S5:DiP, S5:OT
46	Yes	11	11-A	1000	McF_6	Inf	unif	singleC	J1:CBN, J1:CBN-A, J1:DiP, J1:DiP-A, J1:OT, J1:OT-A, J5:CBN-A, J5:DiP, J5:DiP-A, J5:OT, J5:OT-A, S1:DiP, S1:DiP-A, S5:CBN-A, S5:DiP, S5:DiP-A, S5:OT, S5:OT-A
47	Yes	11	11-A	1000	McF_6	Inf	unif	wholeT_0.01	J1:CBN, J1:CBN-A, J1:DiP, J1:DiP-A, J1:OT, J1:OT-A, J5:CBN-A, J5:DiP, J5:DiP-A, J5:OT, J5:OT-A, S1:DiP, S1:DiP-A, S5:CBN, S5:CBN-A, S5:DiP, S5:DiP-A, S5:OT, S5:OT-A
48	Yes	11	11-A	1000	McF_6	Inf	unif	wholeT_0.5	J1:CBN, J1:CBN-A, J1:DiP, J1:DiP-A, J5:CBN-A, J5:DiP, J5:DiP-A, J5:OT, J5:OT-A, S1:DiP, S1:DiP-A, S5:CBN-A, S5:DiP, S5:DiP-A, S5:OT, S5:OT-A
49	Yes	11	11-A	200	Bozic	0	last	singleC	S5:OT, S5:OT-A
50	Yes	11	11-A	200	Bozic	0	last	wholeT_0.01	J1:OT, J1:OT-A, J5:OT, J5:OT-A, S5:OT, S5:OT-A
51	Yes	11	11-A	200	Bozic	0	last	wholeT_0.5	S1:OT, S1:OT-A
52	Yes	11	11-A	200	Bozic	0	unif	singleC	S1:OT, S1:OT-A
53	Yes	11	11-A	200	Bozic	0	unif	wholeT_0.01	J1:CBN-A, J1:DiP, J1:DiP-A, J1:OT, J1:OT-A, S1:DiP-A, S5:CBN, S5:CBN-A, S5:DiP, S5:DiP-A, S5:OT, S5:OT-A
54	Yes	11	11-A	200	Bozic	0	unif	wholeT_0.5	J1:CBN, J1:CBN-A, S1:CBN, S1:CBN-A
55	Yes	11	11-A	200	Bozic	Inf	last	singleC	J5:OT, J5:OT-A, S5:OT, S5:OT-A
56	Yes	11	11-A	200	Bozic	Inf	last	wholeT_0.01	J5:OT, J5:OT-A
57	Yes	11	11-A	200	Bozic	Inf	last	wholeT_0.5	J5:OT, J5:OT-A, S5:OT, S5:OT-A
58	Yes	11	11-A	200	Bozic	Inf	unif	singleC	J1:CBN, J1:CBN-A, J1:OT, J1:OT-A
59	Yes	11	11-A	200	Bozic	Inf	unif	wholeT_0.01	S5:CBN, S5:CBN-A, S5:OT, S5:OT-A
60	Yes	11	11-A	200	Bozic	Inf	unif	wholeT_0.5	J1:CBN, J1:CBN-A, J1:OT, J1:OT-A
61	Yes	11	11-A	200	exp	0	last	singleC	S1:OT, S1:OT-A
62	Yes	11	11-A	200	exp	0	last	wholeT_0.01	J1:OT, J1:OT-A, S1:OT, S1:OT-A, S5:OT, S5:OT-A
63	Yes	11	11-A	200	exp	0	last	wholeT_0.5	S1:OT, S1:OT-A
64	Yes	11	11-A	200	exp	0	unif	singleC	S1:CBN, S1:CBN-A, S5:CBN, S5:CBN-A
65	Yes	11	11-A	200	exp	0	unif	wholeT_0.01	S1:OT, S1:OT-A
66	Yes	11	11-A	200	exp	0	unif	wholeT_0.5	S1:CBN, S1:CBN-A, S5:CBN, S5:CBN-A
67	Yes	11	11-A	200	exp	Inf	last	singleC	J1:OT, J1:OT-A, S5:OT, S5:OT-A
68	Yes	11	11-A	200	exp	Inf	last	wholeT_0.01	J5:CBN-A, J5:OT, J5:OT-A

Table 6: *(continued)*

	Conjunction	Drivers	Tree	S.Size	Model	sh	S.Time	S.Type	Best method(s)
69	Yes	11	11-A	200	exp	Inf	last	wholeT_0.5	J1:OT, J1:OT-A, S5:OT, S5:OT-A
70	Yes	11	11-A	200	exp	Inf	unif	singleC	S1:OT, S1:OT-A
71	Yes	11	11-A	200	exp	Inf	unif	wholeT_0.01	S5:CBN-A, S5:OT, S5:OT-A
72	Yes	11	11-A	200	exp	Inf	unif	wholeT_0.5	S1:OT, S1:OT-A
73	Yes	11	11-A	200	McF_4	0	last	singleC	J1:DiP-A, J1:OT, J1:OT-A, J5:DiP-A, J5:OT, J5:OT-A, S5:DiP, S5:DiP-A, S5:OT, S5:OT-A
74	Yes	11	11-A	200	McF_4	0	last	wholeT_0.01	J1:DiP, J1:DiP-A, J1:OT, J1:OT-A, J5:DiP-A, J5:OT, J5:OT-A, S1:DiP-A, S5:DiP, S5:DiP-A, S5:OT, S5:OT-A
75	Yes	11	11-A	200	McF_4	0	last	wholeT_0.5	J1:OT, J1:OT-A, J5:OT, J5:OT-A, S1:DiP-A, S5:DiP-A, S5:OT, S5:OT-A
76	Yes	11	11-A	200	McF_4	0	unif	singleC	J1:CBN-A, J1:OT, J1:OT-A, S5:OT, S5:OT-A
77	Yes	11	11-A	200	McF_4	0	unif	wholeT_0.01	J1:CBN-A, J1:DiP, J1:DiP-A, J1:OT, J1:OT-A, S1:DiP, S1:DiP-A, S5:CBN-A, S5:DiP, S5:DiP-A, S5:OT, S5:OT-A
78	Yes	11	11-A	200	McF_4	0	unif	wholeT_0.5	J1:CBN-A, J1:OT, J1:OT-A, S1:DiP, S1:DiP-A, S5:CBN, S5:CBN-A, S5:OT, S5:OT-A
79	Yes	11	11-A	200	McF_4	Inf	last	singleC	J1:OT, J1:OT-A, J5:OT, J5:OT-A, S1:DiP-A, S5:OT, S5:OT-A
80	Yes	11	11-A	200	McF_4	Inf	last	wholeT_0.01	J1:DiP-A, J1:OT, J1:OT-A, J5:DiP-A, J5:OT, J5:OT-A, S5:OT
81	Yes	11	11-A	200	McF_4	Inf	last	wholeT_0.5	J1:OT, J1:OT-A, J5:OT, J5:OT-A, S5:OT, S5:OT-A
82	Yes	11	11-A	200	McF_4	Inf	unif	singleC	J1:CBN-A, J1:OT, J1:OT-A, J5:OT, J5:OT-A, S5:CBN-A, S5:OT, S5:OT-A
83	Yes	11	11-A	200	McF_4	Inf	unif	wholeT_0.01	J1:OT, J1:OT-A, J5:OT, J5:OT-A, S5:CBN-A, S5:OT, S5:OT-A
84	Yes	11	11-A	200	McF_4	Inf	unif	wholeT_0.5	J1:CBN-A, J1:OT, J1:OT-A, J5:OT, J5:OT-A, S5:OT, S5:OT-A
85	Yes	11	11-A	200	McF_6	0	last	singleC	J1:OT, J5:OT, S1:DiP, S5:DiP, S5:OT
86	Yes	11	11-A	200	McF_6	0	last	wholeT_0.01	J1:DiP, J1:OT, J5:DiP, J5:OT, S1:DiP, S5:DiP, S5:OT
87	Yes	11	11-A	200	McF_6	0	last	wholeT_0.5	J1:OT, J5:OT, S1:DiP, S5:DiP, S5:OT
88	Yes	11	11-A	200	McF_6	0	unif	singleC	J1:CBN, J1:CBN-A, J1:OT, J1:OT-A, J5:CBN, J5:CBN-A, J5:OT, J5:OT-A, S5:CBN, S5:CBN-A, S5:OT, S5:OT-A

Table 6: *(continued)*

	Conjunction	Drivers	Tree	S.Size	Model	sh	S.Time	S.Type	Best method(s)
89	Yes	11	11-A	200	McF_6	0	unif	wholeT_0.01	J1:CBN, J1:CBN-A, J1:DiP, J1:DiP-A, J1:OT, J1:OT-A, J5:CBN, J5:CBN-A, J5:DiP, J5:DiP-A, J5:OT, J5:OT-A, S5:CBN, S5:CBN-A, S5:DiP, S5:DiP-A, S5:OT, S5:OT-A
90	Yes	11	11-A	200	McF_6	0	unif	wholeT_0.5	J1:CBN, J1:CBN-A, J1:DiP, J5:CBN, J5:CBN-A, J5:DiP-A, J5:OT, J5:OT-A, S5:CBN, S5:CBN-A, S5:DiP, S5:OT, S5:OT-A
91	Yes	11	11-A	200	McF_6	Inf	last	singleC	J1:OT, J5:OT, S5:OT
92	Yes	11	11-A	200	McF_6	Inf	last	wholeT_0.01	J1:OT, J5:OT, S5:OT
93	Yes	11	11-A	200	McF_6	Inf	last	wholeT_0.5	J1:OT, J5:OT, S5:OT
94	Yes	11	11-A	200	McF_6	Inf	unif	singleC	J1:CBN-A, J1:OT, J1:OT-A, J5:CBN-A, J5:OT, J5:OT-A, S5:CBN, S5:CBN-A, S5:OT, S5:OT-A
95	Yes	11	11-A	200	McF_6	Inf	unif	wholeT_0.01	J1:CBN-A, J1:OT, J1:OT-A, J5:CBN-A, J5:OT, J5:OT-A, S5:CBN, S5:CBN-A, S5:OT, S5:OT-A
96	Yes	11	11-A	200	McF_6	Inf	unif	wholeT_0.5	J1:CBN-A, J1:OT, J1:OT-A, J5:CBN-A, J5:OT, J5:OT-A, S5:CBN, S5:CBN-A, S5:OT, S5:OT-A
97	Yes	11	11-A	100	Bozic	0	last	singleC	S5:OT, S5:OT-A
98	Yes	11	11-A	100	Bozic	0	last	wholeT_0.01	J5:OT, J5:OT-A, S5:OT, S5:OT-A
99	Yes	11	11-A	100	Bozic	0	last	wholeT_0.5	J1:CBN, J1:CBN-A, J1:OT, J1:OT-A, S1:OT, S1:OT-A
100	Yes	11	11-A	100	Bozic	0	unif	singleC	S1:OT, S1:OT-A
101	Yes	11	11-A	100	Bozic	0	unif	wholeT_0.01	S5:OT, S5:OT-A
102	Yes	11	11-A	100	Bozic	0	unif	wholeT_0.5	S1:OT, S1:OT-A
103	Yes	11	11-A	100	Bozic	Inf	last	singleC	J5:OT, J5:OT-A
104	Yes	11	11-A	100	Bozic	Inf	last	wholeT_0.01	J5:CBN-A, J5:OT, J5:OT-A
105	Yes	11	11-A	100	Bozic	Inf	last	wholeT_0.5	J5:OT, J5:OT-A
106	Yes	11	11-A	100	Bozic	Inf	unif	singleC	J1:CBN, J1:CBN-A, J1:OT, J1:OT-A, S1:OT, S1:OT-A, S5:CBN, S5:CBN-A
107	Yes	11	11-A	100	Bozic	Inf	unif	wholeT_0.01	J5:OT, J5:OT-A
108	Yes	11	11-A	100	Bozic	Inf	unif	wholeT_0.5	J1:CBN, J1:CBN-A, J1:OT, J1:OT-A, S1:OT, S1:OT-A, S5:CBN, S5:CBN-A
109	Yes	11	11-A	100	exp	0	last	singleC	S1:OT, S1:OT-A
110	Yes	11	11-A	100	exp	0	last	wholeT_0.01	J1:OT, J1:OT-A, S5:OT, S5:OT-A
111	Yes	11	11-A	100	exp	0	last	wholeT_0.5	S1:OT, S1:OT-A
112	Yes	11	11-A	100	exp	0	unif	singleC	S1:CBN, S1:CBN-A

Table 6: *(continued)*

	Conjunction	Drivers	Tree	S.Size	Model	sh	S.Time	S.Type	Best method(s)
113	Yes	11	11-A	100	exp	0	unif	wholeT_0.01	S1:OT, S1:OT-A
114	Yes	11	11-A	100	exp	0	unif	wholeT_0.5	S1:CBN, S1:CBN-A, S5:CBN, S5:CBN-A
115	Yes	11	11-A	100	exp	Inf	last	singleC	S5:OT, S5:OT-A
116	Yes	11	11-A	100	exp	Inf	last	wholeT_0.01	J5:CBN-A, J5:OT, J5:OT-A
117	Yes	11	11-A	100	exp	Inf	last	wholeT_0.5	S5:OT, S5:OT-A
118	Yes	11	11-A	100	exp	Inf	unif	singleC	J1:CBN-A, J1:OT, J1:OT-A, S1:OT, S1:OT-A
119	Yes	11	11-A	100	exp	Inf	unif	wholeT_0.01	S5:OT, S5:OT-A
120	Yes	11	11-A	100	exp	Inf	unif	wholeT_0.5	J1:CBN, J1:CBN-A, J1:OT, J1:OT-A, S1:OT, S1:OT-A
121	Yes	11	11-A	100	McF_4	0	last	singleC	J5:OT, J5:OT-A, S5:OT, S5:OT-A
122	Yes	11	11-A	100	McF_4	0	last	wholeT_0.01	J1:DiP-A, J5:OT, J5:OT-A, S1:DiP, S1:DiP-A, S5:DiP, S5:DiP-A, S5:OT
123	Yes	11	11-A	100	McF_4	0	last	wholeT_0.5	J5:OT, J5:OT-A, S5:OT, S5:OT-A
124	Yes	11	11-A	100	McF_4	0	unif	singleC	J1:CBN-A, J1:OT, J1:OT-A, S5:CBN-A, S5:OT, S5:OT-A
125	Yes	11	11-A	100	McF_4	0	unif	wholeT_0.01	S5:CBN-A, S5:DiP-A, S5:OT, S5:OT-A
126	Yes	11	11-A	100	McF_4	0	unif	wholeT_0.5	J1:CBN-A, J1:OT, J1:OT-A, S5:CBN, S5:CBN-A, S5:OT, S5:OT-A
127	Yes	11	11-A	100	McF_4	Inf	last	singleC	J5:OT, J5:OT-A, S5:OT, S5:OT-A
128	Yes	11	11-A	100	McF_4	Inf	last	wholeT_0.01	J5:OT, J5:OT-A
129	Yes	11	11-A	100	McF_4	Inf	last	wholeT_0.5	J5:OT, J5:OT-A, S5:OT, S5:OT-A
130	Yes	11	11-A	100	McF_4	Inf	unif	singleC	J1:OT, J1:OT-A, J5:OT, J5:OT-A, S5:CBN-A, S5:OT, S5:OT-A
131	Yes	11	11-A	100	McF_4	Inf	unif	wholeT_0.01	J5:OT, J5:OT-A
132	Yes	11	11-A	100	McF_4	Inf	unif	wholeT_0.5	J1:CBN-A, J5:OT, J5:OT-A, S5:CBN-A, S5:OT, S5:OT-A
133	Yes	11	11-A	100	McF_6	0	last	singleC	J1:OT, J5:OT, S1:DiP, S5:DiP, S5:OT
134	Yes	11	11-A	100	McF_6	0	last	wholeT_0.01	J1:DiP, J5:OT, S1:DiP, S5:DiP, S5:OT
135	Yes	11	11-A	100	McF_6	0	last	wholeT_0.5	J1:OT, J5:OT, S5:OT
136	Yes	11	11-A	100	McF_6	0	unif	singleC	J1:CBN-A, J5:CBN, J5:CBN-A, J5:OT, J5:OT-A
137	Yes	11	11-A	100	McF_6	0	unif	wholeT_0.01	J1:DiP, J1:DiP-A, J5:CBN, J5:CBN-A, J5:DiP, J5:DiP-A, J5:OT, J5:OT-A, S1:DiP, S5:DiP, S5:DiP-A, S5:OT, S5:OT-A
138	Yes	11	11-A	100	McF_6	0	unif	wholeT_0.5	J1:CBN, J1:CBN-A, J5:CBN-A, S5:CBN, S5:CBN-A
139	Yes	11	11-A	100	McF_6	Inf	last	singleC	J1:OT, J5:OT, S5:OT
140	Yes	11	11-A	100	McF_6	Inf	last	wholeT_0.01	J5:OT, S5:OT

Table 6: *(continued)*

	Conjunction	Drivers	Tree	S.Size	Model	sh	S.Time	S.Type	Best method(s)
141	Yes	11	11-A	100	McF_6	Inf	last	wholeT_0.5	J1:OT, J5:OT, S5:OT
142	Yes	11	11-A	100	McF_6	Inf	unif	singleC	J1:CBN, J1:CBN-A, J5:CBN-A, J5:OT, J5:OT-A
143	Yes	11	11-A	100	McF_6	Inf	unif	wholeT_0.01	J5:CBN-A, J5:OT, J5:OT-A, S5:CBN, S5:CBN-A, S5:OT, S5:OT-A
144	Yes	11	11-A	100	McF_6	Inf	unif	wholeT_0.5	J1:CBN-A, J1:OT, J1:OT-A, J5:CBN-A, J5:OT, J5:OT-A, S5:CBN, S5:CBN-A, S5:OT, S5:OT-A
145	Yes	9	9-A	1000	Bozic	0	last	singleC	S1:OT, S1:OT-A
146	Yes	9	9-A	1000	Bozic	0	last	wholeT_0.01	J1:DiP, J1:DiP-A, J1:OT, J1:OT-A, J5:DiP, J5:DiP-A, J5:OT, J5:OT-A, S1:DiP, S1:DiP-A, S1:OT, S1:OT-A, S5:DiP, S5:DiP-A, S5:OT, S5:OT-A
147	Yes	9	9-A	1000	Bozic	0	last	wholeT_0.5	S1:OT, S1:OT-A
148	Yes	9	9-A	1000	Bozic	0	unif	singleC	S1:CBN, S1:CBN-A, S5:CBN, S5:CBN-A
149	Yes	9	9-A	1000	Bozic	0	unif	wholeT_0.01	J1:DiP, J1:DiP-A, J1:OT, J1:OT-A, S1:DiP, S1:DiP-A, S1:OT, S1:OT-A
150	Yes	9	9-A	1000	Bozic	0	unif	wholeT_0.5	S1:CBN, S1:CBN-A, S5:CBN, S5:CBN-A
151	Yes	9	9-A	1000	Bozic	Inf	last	singleC	J1:DiP, J1:DiP-A, J1:OT, J1:OT-A, J5:DiP, J5:DiP-A, J5:OT, J5:OT-A, S5:OT, S5:OT-A
152	Yes	9	9-A	1000	Bozic	Inf	last	wholeT_0.01	J5:DiP, J5:DiP-A, J5:OT, J5:OT-A
153	Yes	9	9-A	1000	Bozic	Inf	last	wholeT_0.5	J1:DiP, J1:DiP-A, J1:OT, J1:OT-A, J5:DiP, J5:DiP-A, J5:OT, J5:OT-A, S5:DiP, S5:DiP-A, S5:OT, S5:OT-A
154	Yes	9	9-A	1000	Bozic	Inf	unif	singleC	S1:CBN, S1:DiP, S1:DiP-A, S1:OT, S1:OT-A
155	Yes	9	9-A	1000	Bozic	Inf	unif	wholeT_0.01	S5:DiP, S5:DiP-A, S5:OT, S5:OT-A
156	Yes	9	9-A	1000	Bozic	Inf	unif	wholeT_0.5	S1:CBN, S1:DiP-A, S1:OT, S1:OT-A
157	Yes	9	9-A	1000	exp	0	last	singleC	J1:CBN, J1:CBN-A, S1:CBN, S1:CBN-A, S5:CBN, S5:CBN-A
158	Yes	9	9-A	1000	exp	0	last	wholeT_0.01	S1:DiP-A, S1:OT, S1:OT-A
159	Yes	9	9-A	1000	exp	0	last	wholeT_0.5	J1:CBN, J1:CBN-A, S1:CBN, S1:CBN-A, S5:CBN, S5:CBN-A
160	Yes	9	9-A	1000	exp	0	unif	singleC	S1:CBN, S1:CBN-A, S5:CBN, S5:CBN-A
161	Yes	9	9-A	1000	exp	0	unif	wholeT_0.01	J1:CBN, J1:CBN-A, S1:CBN, S1:CBN-A, S5:CBN, S5:CBN-A
162	Yes	9	9-A	1000	exp	0	unif	wholeT_0.5	S1:CBN, S1:CBN-A, S5:CBN, S5:CBN-A
163	Yes	9	9-A	1000	exp	Inf	last	singleC	J1:DiP, J1:DiP-A, J1:OT, J1:OT-A, S5:DiP, S5:DiP-A, S5:OT, S5:OT-A

Table 6: *(continued)*

	Conjunction	Drivers	Tree	S.Size	Model	sh	S.Time	S.Type	Best method(s)
164	Yes	9	9-A	1000	exp	Inf	last	wholeT_0.01	J5:DiP, J5:DiP-A, J5:OT, J5:OT-A
165	Yes	9	9-A	1000	exp	Inf	last	wholeT_0.5	J1:DiP, J1:DiP-A, J1:OT, J1:OT-A, S5:DiP, S5:DiP-A, S5:OT, S5:OT-A
166	Yes	9	9-A	1000	exp	Inf	unif	singleC	S1:OT, S1:OT-A
167	Yes	9	9-A	1000	exp	Inf	unif	wholeT_0.01	J1:DiP, J1:DiP-A, J1:OT, J1:OT-A
168	Yes	9	9-A	1000	exp	Inf	unif	wholeT_0.5	S1:OT, S1:OT-A
169	Yes	9	9-A	1000	McF_4	0	last	singleC	J1:DiP, J1:DiP-A, J1:OT, J1:OT-A, J5:DiP, J5:DiP-A, J5:OT, J5:OT-A, S1:DiP, S1:DiP-A, S1:OT, S1:OT-A, S5:DiP, S5:DiP-A, S5:OT, S5:OT-A
170	Yes	9	9-A	1000	McF_4	0	last	wholeT_0.01	J1:DiP, J1:DiP-A, J1:OT, J1:OT-A, J5:CBN, J5:DiP, J5:DiP-A, J5:OT, J5:OT-A, S1:DiP-A, S5:DiP, S5:DiP-A, S5:OT, S5:OT-A
171	Yes	9	9-A	1000	McF_4	0	last	wholeT_0.5	J1:DiP, J1:DiP-A, J1:OT, J1:OT-A, J5:CBN, J5:DiP, J5:DiP-A, J5:OT, J5:OT-A, S1:DiP, S1:DiP-A, S1:OT, S1:OT-A, S5:DiP, S5:DiP-A, S5:OT, S5:OT-A
172	Yes	9	9-A	1000	McF_4	0	unif	singleC	J1:CBN-A, J1:DiP, J1:DiP-A, J1:OT, J1:OT-A, S1:DiP, S1:DiP-A, S1:OT, S1:OT-A, S5:DiP, S5:DiP-A, S5:OT, S5:OT-A
173	Yes	9	9-A	1000	McF_4	0	unif	wholeT_0.01	J1:CBN-A, J1:DiP, J1:DiP-A, J1:OT, J1:OT-A, S1:DiP-A, S5:CBN-A, S5:DiP, S5:DiP-A, S5:OT, S5:OT-A
174	Yes	9	9-A	1000	McF_4	0	unif	wholeT_0.5	J1:DiP, J1:DiP-A, J1:OT, J1:OT-A, S1:DiP, S1:DiP-A, S1:OT, S1:OT-A, S5:DiP, S5:DiP-A, S5:OT, S5:OT-A
175	Yes	9	9-A	1000	McF_4	Inf	last	singleC	J1:DiP, J1:DiP-A, J1:OT, J1:OT-A, J5:DiP, J5:DiP-A, J5:OT, J5:OT-A, S1:OT, S1:OT-A, S5:DiP, S5:DiP-A, S5:OT, S5:OT-A
176	Yes	9	9-A	1000	McF_4	Inf	last	wholeT_0.01	J1:DiP, J1:OT, J5:DiP, J5:OT, S1:DiP, S5:DiP, S5:OT
177	Yes	9	9-A	1000	McF_4	Inf	last	wholeT_0.5	J1:DiP, J1:DiP-A, J1:OT, J1:OT-A, J5:DiP, J5:DiP-A, J5:OT, J5:OT-A, S1:DiP, S1:DiP-A, S1:OT, S1:OT-A, S5:DiP, S5:DiP-A, S5:OT, S5:OT-A

Table 6: *(continued)*

	Conjunction	Drivers	Tree	S.Size	Model	sh	S.Time	S.Type	Best method(s)
178	Yes	9	9-A	1000	McF_4	Inf	unif	singleC	J1:DiP, J1:DiP-A, J1:OT, J1:OT-A, S1:DiP, S1:DiP-A, S1:OT, S1:OT-A, S5:CBN-A, S5:DiP, S5:DiP-A, S5:OT, S5:OT-A
179	Yes	9	9-A	1000	McF_4	Inf	unif	wholeT_0.01	J1:DiP, J1:DiP-A, J1:OT, J1:OT-A, S5:CBN-A, S5:DiP, S5:DiP-A, S5:OT, S5:OT-A
180	Yes	9	9-A	1000	McF_4	Inf	unif	wholeT_0.5	J1:DiP, J1:DiP-A, J1:OT, J1:OT-A, S1:DiP, S1:DiP-A, S1:OT, S1:OT-A, S5:CBN-A, S5:DiP, S5:DiP-A, S5:OT, S5:OT-A
181	Yes	9	9-A	1000	McF_6	0	last	singleC	J1:DiP, J1:OT, J5:DiP, J5:OT, S1:DiP, S5:DiP, S5:OT
182	Yes	9	9-A	1000	McF_6	0	last	wholeT_0.01	J1:DiP, J1:OT, J5:DiP, J5:OT, S1:DiP, S5:DiP, S5:OT
183	Yes	9	9-A	1000	McF_6	0	last	wholeT_0.5	J1:DiP, J1:OT, J5:DiP, J5:OT, S1:DiP, S1:OT, S5:DiP, S5:OT
184	Yes	9	9-A	1000	McF_6	0	unif	singleC	J1:CBN, J1:CBN-A, J1:DiP, J1:DiP-A, J1:OT, J1:OT-A, J5:CBN, J5:CBN-A, J5:DiP, J5:DiP-A, J5:OT, J5:OT-A, S1:DiP, S1:DiP-A, S1:OT, S1:OT-A, S5:CBN, S5:CBN-A, S5:DiP, S5:DiP-A, S5:OT, S5:OT-A
185	Yes	9	9-A	1000	McF_6	0	unif	wholeT_0.01	J1:CBN, J1:CBN-A, J1:DiP, J1:DiP-A, J1:OT, J1:OT-A, J5:CBN, J5:CBN-A, J5:DiP, J5:DiP-A, J5:OT, J5:OT-A, S1:DiP, S5:CBN, S5:CBN-A, S5:DiP, S5:DiP-A, S5:OT, S5:OT-A
186	Yes	9	9-A	1000	McF_6	0	unif	wholeT_0.5	J1:CBN, J1:CBN-A, J1:DiP, J1:DiP-A, J1:OT, J1:OT-A, J5:CBN, J5:CBN-A, J5:DiP, J5:DiP-A, J5:OT, J5:OT-A, S1:CBN-A, S1:DiP, S1:DiP-A, S1:OT, S1:OT-A, S5:CBN, S5:CBN-A, S5:DiP, S5:DiP-A, S5:OT, S5:OT-A
187	Yes	9	9-A	1000	McF_6	Inf	last	singleC	J1:DiP, J1:OT, J5:DiP, J5:OT, S1:DiP, S5:DiP, S5:OT
188	Yes	9	9-A	1000	McF_6	Inf	last	wholeT_0.01	J1:DiP, J1:OT, J5:DiP, J5:OT, S5:OT
189	Yes	9	9-A	1000	McF_6	Inf	last	wholeT_0.5	J1:DiP, J1:OT, J5:DiP, J5:OT, S1:DiP, S5:DiP, S5:OT

Table 6: *(continued)*

	Conjunction	Drivers	Tree	S.Size	Model	sh	S.Time	S.Type	Best method(s)
190	Yes	9	9-A	1000	McF_6	Inf	unif	singleC	J1:CBN, J1:CBN-A, J1:DiP, J1:DiP-A, J1:OT, J1:OT-A, J5:CBN, J5:CBN-A, J5:DiP, J5:DiP-A, J5:OT, J5:OT-A, S1:DiP, S1:DiP-A, S1:OT, S1:OT-A, S5:CBN, S5:CBN-A, S5:DiP, S5:DiP-A, S5:OT, S5:OT-A
191	Yes	9	9-A	1000	McF_6	Inf	unif	wholeT_0.01	J1:CBN, J1:CBN-A, J1:DiP, J1:DiP-A, J1:OT, J1:OT-A, J5:CBN, J5:CBN-A, J5:DiP, J5:DiP-A, J5:OT, J5:OT-A, S1:DiP, S1:DiP-A, S5:CBN, S5:CBN-A, S5:DiP, S5:DiP-A, S5:OT, S5:OT-A
192	Yes	9	9-A	1000	McF_6	Inf	unif	wholeT_0.5	J1:CBN, J1:CBN-A, J1:DiP, J1:DiP-A, J1:OT, J1:OT-A, J5:CBN, J5:CBN-A, J5:DiP, J5:DiP-A, J5:OT, J5:OT-A, S1:DiP, S1:DiP-A, S1:OT, S1:OT-A, S5:CBN, S5:CBN-A, S5:DiP, S5:DiP-A, S5:OT, S5:OT-A
193	Yes	9	9-A	200	Bozic	0	last	singleC	S1:OT, S1:OT-A
194	Yes	9	9-A	200	Bozic	0	last	wholeT_0.01	J1:OT, J1:OT-A, J5:OT, J5:OT-A, S5:OT, S5:OT-A
195	Yes	9	9-A	200	Bozic	0	last	wholeT_0.5	S1:OT, S1:OT-A
196	Yes	9	9-A	200	Bozic	0	unif	singleC	J1:CBN, J1:CBN-A, S1:CBN, S1:CBN-A
197	Yes	9	9-A	200	Bozic	0	unif	wholeT_0.01	S1:DiP, S1:DiP-A
198	Yes	9	9-A	200	Bozic	0	unif	wholeT_0.5	J1:CBN, J1:CBN-A, S1:CBN, S1:CBN-A, S5:CBN, S5:CBN-A
199	Yes	9	9-A	200	Bozic	Inf	last	singleC	J5:OT, J5:OT-A, S5:OT, S5:OT-A
200	Yes	9	9-A	200	Bozic	Inf	last	wholeT_0.01	J5:OT, J5:OT-A
201	Yes	9	9-A	200	Bozic	Inf	last	wholeT_0.5	J5:OT, J5:OT-A, S5:OT, S5:OT-A
202	Yes	9	9-A	200	Bozic	Inf	unif	singleC	S1:OT, S1:OT-A
203	Yes	9	9-A	200	Bozic	Inf	unif	wholeT_0.01	S5:OT, S5:OT-A
204	Yes	9	9-A	200	Bozic	Inf	unif	wholeT_0.5	S1:OT, S1:OT-A
205	Yes	9	9-A	200	exp	0	last	singleC	J1:CBN, J1:CBN-A, S1:CBN, S1:CBN-A, S5:CBN, S5:CBN-A
206	Yes	9	9-A	200	exp	0	last	wholeT_0.01	S1:OT, S1:OT-A
207	Yes	9	9-A	200	exp	0	last	wholeT_0.5	J1:CBN, J1:CBN-A, S1:CBN, S1:CBN-A, S5:CBN, S5:CBN-A
208	Yes	9	9-A	200	exp	0	unif	singleC	S1:CBN, S1:CBN-A, S5:CBN, S5:CBN-A
209	Yes	9	9-A	200	exp	0	unif	wholeT_0.01	S1:OT, S1:OT-A
210	Yes	9	9-A	200	exp	0	unif	wholeT_0.5	S1:CBN, S1:CBN-A, S5:CBN, S5:CBN-A
211	Yes	9	9-A	200	exp	Inf	last	singleC	J1:OT, J1:OT-A, S5:OT, S5:OT-A

Table 6: *(continued)*

	Conjunction	Drivers	Tree	S.Size	Model	sh	S.Time	S.Type	Best method(s)
212	Yes	9	9-A	200	exp	Inf	last	wholeT_0.01	J5:OT, J5:OT-A
213	Yes	9	9-A	200	exp	Inf	last	wholeT_0.5	J1:OT, J1:OT-A, S5:OT, S5:OT-A
214	Yes	9	9-A	200	exp	Inf	unif	singleC	S1:OT, S1:OT-A
215	Yes	9	9-A	200	exp	Inf	unif	wholeT_0.01	J1:CBN, J1:OT, J1:OT-A, S5:CBN, S5:CBN-A
216	Yes	9	9-A	200	exp	Inf	unif	wholeT_0.5	S1:OT, S1:OT-A
217	Yes	9	9-A	200	McF_4	0	last	singleC	J1:DiP-A, J1:OT, J1:OT-A, J5:DiP-A, J5:OT, J5:OT-A, S5:DiP-A, S5:OT, S5:OT-A
218	Yes	9	9-A	200	McF_4	0	last	wholeT_0.01	J1:DiP, J1:DiP-A, J1:OT, J1:OT-A, J5:DiP, J5:DiP-A, J5:OT, J5:OT-A, S1:DiP, S1:DiP-A, S5:DiP, S5:DiP-A, S5:OT, S5:OT-A
219	Yes	9	9-A	200	McF_4	0	last	wholeT_0.5	J1:OT, J1:OT-A, J5:OT, J5:OT-A, S1:DiP-A, S5:DiP-A, S5:OT, S5:OT-A
220	Yes	9	9-A	200	McF_4	0	unif	singleC	J1:OT, J1:OT-A, S5:CBN-A, S5:OT, S5:OT-A
221	Yes	9	9-A	200	McF_4	0	unif	wholeT_0.01	J1:OT, J1:OT-A, S1:DiP, S1:DiP-A, S5:CBN-A, S5:DiP, S5:DiP-A, S5:OT, S5:OT-A
222	Yes	9	9-A	200	McF_4	0	unif	wholeT_0.5	J1:OT, J1:OT-A, S5:OT, S5:OT-A
223	Yes	9	9-A	200	McF_4	Inf	last	singleC	J5:OT, J5:OT-A, S5:OT, S5:OT-A
224	Yes	9	9-A	200	McF_4	Inf	last	wholeT_0.01	J1:DiP-A, J5:DiP-A, J5:OT, S5:DiP-A, S5:OT
225	Yes	9	9-A	200	McF_4	Inf	last	wholeT_0.5	J1:OT, J1:OT-A, J5:OT, J5:OT-A, S5:OT, S5:OT-A
226	Yes	9	9-A	200	McF_4	Inf	unif	singleC	J1:OT, J1:OT-A, S5:OT, S5:OT-A
227	Yes	9	9-A	200	McF_4	Inf	unif	wholeT_0.01	J1:OT, J1:OT-A, S5:OT, S5:OT-A
228	Yes	9	9-A	200	McF_4	Inf	unif	wholeT_0.5	J1:OT, J1:OT-A, S5:CBN-A, S5:OT, S5:OT-A
229	Yes	9	9-A	200	McF_6	0	last	singleC	J1:OT, J5:OT, S1:DiP, S5:DiP, S5:OT
230	Yes	9	9-A	200	McF_6	0	last	wholeT_0.01	J1:DiP, J5:DiP, J5:OT, S1:DiP, S5:DiP, S5:OT
231	Yes	9	9-A	200	McF_6	0	last	wholeT_0.5	J1:OT, J5:OT, S5:OT
232	Yes	9	9-A	200	McF_6	0	unif	singleC	J1:CBN, J1:CBN-A, J1:DiP, J1:DiP-A, J1:OT, J1:OT-A, J5:CBN, J5:CBN-A, J5:DiP, J5:DiP-A, J5:OT, J5:OT-A, S1:DiP, S1:DiP-A, S5:CBN, S5:CBN-A, S5:DiP, S5:DiP-A, S5:OT, S5:OT-A

Table 6: *(continued)*

	Conjunction	Drivers	Tree	S.Size	Model	sh	S.Time	S.Type	Best method(s)
233	Yes	9	9-A	200	McF_6	0	unif	wholeT_0.01	J1:CBN, J1:CBN-A, J1:DiP, J1:DiP-A, J1:OT, J1:OT-A, J5:CBN, J5:CBN-A, J5:DiP, J5:DiP-A, J5:OT, J5:OT-A, S1:DiP, S1:DiP-A, S5:CBN, S5:CBN-A, S5:DiP, S5:DiP-A, S5:OT, S5:OT-A
234	Yes	9	9-A	200	McF_6	0	unif	wholeT_0.5	J1:CBN, J1:CBN-A, J1:DiP, J1:DiP-A, J1:OT, J1:OT-A, J5:CBN, J5:CBN-A, J5:DiP, J5:DiP-A, J5:OT, J5:OT-A, S1:DiP, S1:DiP-A, S5:CBN, S5:CBN-A, S5:DiP, S5:DiP-A, S5:OT, S5:OT-A
235	Yes	9	9-A	200	McF_6	Inf	last	singleC	J1:OT, J5:OT, S5:OT
236	Yes	9	9-A	200	McF_6	Inf	last	wholeT_0.01	J5:OT, S5:OT
237	Yes	9	9-A	200	McF_6	Inf	last	wholeT_0.5	J1:OT, J5:OT, S5:OT
238	Yes	9	9-A	200	McF_6	Inf	unif	singleC	J1:CBN, J1:CBN-A, J1:DiP, J1:DiP-A, J1:OT, J1:OT-A, J5:CBN, J5:CBN-A, J5:DiP-A, J5:OT, J5:OT-A, S1:DiP, S1:DiP-A, S5:CBN, S5:CBN-A, S5:DiP, S5:DiP-A, S5:OT, S5:OT-A
239	Yes	9	9-A	200	McF_6	Inf	unif	wholeT_0.01	J1:CBN, J1:CBN-A, J1:DiP-A, J1:OT, J1:OT-A, J5:CBN, J5:CBN-A, J5:DiP-A, J5:OT, J5:OT-A, S1:DiP, S5:CBN, S5:CBN-A, S5:DiP, S5:DiP-A, S5:OT, S5:OT-A
240	Yes	9	9-A	200	McF_6	Inf	unif	wholeT_0.5	J1:CBN, J1:CBN-A, J1:DiP-A, J1:OT, J1:OT-A, J5:CBN, J5:CBN-A, J5:DiP-A, J5:OT, J5:OT-A, S1:DiP, S1:DiP-A, S5:CBN, S5:CBN-A, S5:DiP, S5:DiP-A, S5:OT, S5:OT-A
241	Yes	9	9-A	100	Bozic	0	last	singleC	S1:OT, S1:OT-A
242	Yes	9	9-A	100	Bozic	0	last	wholeT_0.01	J5:OT, J5:OT-A, S5:OT, S5:OT-A
243	Yes	9	9-A	100	Bozic	0	last	wholeT_0.5	S1:OT, S1:OT-A
244	Yes	9	9-A	100	Bozic	0	unif	singleC	S1:CBN, S1:OT, S1:OT-A
245	Yes	9	9-A	100	Bozic	0	unif	wholeT_0.01	J1:CBN, J1:CBN-A, J1:OT, J1:OT-A, S1:OT, S1:OT-A, S5:CBN
246	Yes	9	9-A	100	Bozic	0	unif	wholeT_0.5	S1:CBN, S1:CBN-A, S1:OT, S1:OT-A
247	Yes	9	9-A	100	Bozic	Inf	last	singleC	S5:OT, S5:OT-A
248	Yes	9	9-A	100	Bozic	Inf	last	wholeT_0.01	J5:OT, J5:OT-A
249	Yes	9	9-A	100	Bozic	Inf	last	wholeT_0.5	J5:OT, J5:OT-A
250	Yes	9	9-A	100	Bozic	Inf	unif	singleC	J1:CBN, J1:CBN-A, J1:OT, J1:OT-A, S1:OT, S1:OT-A

Table 6: *(continued)*

	Conjunction	Drivers	Tree	S.Size	Model	sh	S.Time	S.Type	Best method(s)
251	Yes	9	9-A	100	Bozic	Inf	unif	wholeT_0.01	S5:OT, S5:OT-A
252	Yes	9	9-A	100	Bozic	Inf	unif	wholeT_0.5	J1:CBN, J1:CBN-A, J1:OT, J1:OT-A, S1:OT, S1:OT-A
253	Yes	9	9-A	100	exp	0	last	singleC	S1:OT, S1:OT-A
254	Yes	9	9-A	100	exp	0	last	wholeT_0.01	S5:OT, S5:OT-A
255	Yes	9	9-A	100	exp	0	last	wholeT_0.5	S1:OT, S1:OT-A
256	Yes	9	9-A	100	exp	0	unif	singleC	S1:CBN, S1:CBN-A, S5:CBN, S5:CBN-A
257	Yes	9	9-A	100	exp	0	unif	wholeT_0.01	J1:CBN, J1:CBN-A, S1:OT, S1:OT-A
258	Yes	9	9-A	100	exp	0	unif	wholeT_0.5	J1:CBN, J1:CBN-A, S1:CBN, S1:CBN-A, S5:CBN, S5:CBN-A
259	Yes	9	9-A	100	exp	Inf	last	singleC	S5:OT, S5:OT-A
260	Yes	9	9-A	100	exp	Inf	last	wholeT_0.01	J5:OT, J5:OT-A
261	Yes	9	9-A	100	exp	Inf	last	wholeT_0.5	S5:OT, S5:OT-A
262	Yes	9	9-A	100	exp	Inf	unif	singleC	J1:CBN, J1:CBN-A, S1:OT, S1:OT-A
263	Yes	9	9-A	100	exp	Inf	unif	wholeT_0.01	J1:OT, J1:OT-A, S1:OT, S1:OT-A, S5:CBN, S5:CBN-A
264	Yes	9	9-A	100	exp	Inf	unif	wholeT_0.5	J1:CBN-A, S1:OT, S1:OT-A
265	Yes	9	9-A	100	McF_4	0	last	singleC	J5:OT, J5:OT-A, S5:OT, S5:OT-A
266	Yes	9	9-A	100	McF_4	0	last	wholeT_0.01	J1:DiP-A, J5:DiP, J5:DiP-A, J5:OT, J5:OT-A, S1:DiP, S1:DiP-A, S5:DiP, S5:DiP-A, S5:OT, S5:OT-A
267	Yes	9	9-A	100	McF_4	0	last	wholeT_0.5	J5:OT, J5:OT-A, S5:OT, S5:OT-A
268	Yes	9	9-A	100	McF_4	0	unif	singleC	S5:OT, S5:OT-A
269	Yes	9	9-A	100	McF_4	0	unif	wholeT_0.01	S5:OT, S5:OT-A
270	Yes	9	9-A	100	McF_4	0	unif	wholeT_0.5	J1:OT, J1:OT-A, S5:OT, S5:OT-A
271	Yes	9	9-A	100	McF_4	Inf	last	singleC	J5:OT, J5:OT-A, S5:OT, S5:OT-A
272	Yes	9	9-A	100	McF_4	Inf	last	wholeT_0.01	J5:OT, S5:OT
273	Yes	9	9-A	100	McF_4	Inf	last	wholeT_0.5	J5:OT, J5:OT-A, S5:OT, S5:OT-A
274	Yes	9	9-A	100	McF_4	Inf	unif	singleC	J1:OT, J1:OT-A, S5:CBN-A, S5:OT, S5:OT-A
275	Yes	9	9-A	100	McF_4	Inf	unif	wholeT_0.01	S5:OT, S5:OT-A
276	Yes	9	9-A	100	McF_4	Inf	unif	wholeT_0.5	J1:OT, J1:OT-A, S5:OT, S5:OT-A
277	Yes	9	9-A	100	McF_6	0	last	singleC	J5:OT, S5:OT
278	Yes	9	9-A	100	McF_6	0	last	wholeT_0.01	J5:OT, S5:OT
279	Yes	9	9-A	100	McF_6	0	last	wholeT_0.5	J5:OT, S5:OT
280	Yes	9	9-A	100	McF_6	0	unif	singleC	J1:CBN, J1:CBN-A, J1:OT, J5:CBN, J5:CBN-A, J5:OT, J5:OT-A, S5:CBN, S5:CBN-A, S5:OT

Table 6: *(continued)*

	Conjunction	Drivers	Tree	S.Size	Model	sh	S.Time	S.Type	Best method(s)
281	Yes	9	9-A	100	McF_6	0	unif	wholeT_0.01	J1:DiP, J1:DiP-A, J5:CBN, J5:CBN-A, J5:OT, J5:OT-A, S5:CBN, S5:DiP, S5:DiP-A, S5:OT, S5:OT-A
282	Yes	9	9-A	100	McF_6	0	unif	wholeT_0.5	J1:CBN, J1:CBN-A, J1:OT, J1:OT-A, J5:CBN, J5:CBN-A, J5:OT, J5:OT-A, S5:CBN-A, S5:OT, S5:OT-A
283	Yes	9	9-A	100	McF_6	Inf	last	singleC	J1:OT, J5:OT, S5:OT
284	Yes	9	9-A	100	McF_6	Inf	last	wholeT_0.01	J5:OT, S5:OT
285	Yes	9	9-A	100	McF_6	Inf	last	wholeT_0.5	J5:OT, S5:OT
286	Yes	9	9-A	100	McF_6	Inf	unif	singleC	J1:CBN-A, J5:CBN, J5:CBN-A, J5:OT, J5:OT-A, S5:CBN, S5:CBN-A, S5:OT, S5:OT-A
287	Yes	9	9-A	100	McF_6	Inf	unif	wholeT_0.01	J5:CBN-A, J5:OT, J5:OT-A, S5:CBN, S5:OT, S5:OT-A
288	Yes	9	9-A	100	McF_6	Inf	unif	wholeT_0.5	J1:CBN, J1:CBN-A, J1:OT, J1:OT-A, J5:CBN, J5:CBN-A, J5:OT, J5:OT-A, S5:CBN-A, S5:OT, S5:OT-A
289	Yes	7	7-A	1000	Bozic	0	last	singleC	J1:CBN-A, J1:OT-A, J5:CBN-A, J5:OT-A, S1:OT-A, S5:OT-A
290	Yes	7	7-A	1000	Bozic	0	last	wholeT_0.01	J1:DiP-A, J1:OT-A, J5:DiP-A, J5:OT-A, S1:DiP-A, S1:OT-A, S5:DiP-A, S5:OT-A
291	Yes	7	7-A	1000	Bozic	0	last	wholeT_0.5	J1:CBN-A, J1:OT-A, J5:CBN-A, J5:OT-A, S1:OT-A, S5:CBN-A, S5:OT-A
292	Yes	7	7-A	1000	Bozic	0	unif	singleC	J1:CBN, J1:CBN-A, J1:OT, J1:OT-A, S1:CBN, S1:CBN-A, S1:OT, S1:OT-A
293	Yes	7	7-A	1000	Bozic	0	unif	wholeT_0.01	J5:CBN, J5:CBN-A, J5:DiP-A, J5:OT, J5:OT-A
294	Yes	7	7-A	1000	Bozic	0	unif	wholeT_0.5	NA
295	Yes	7	7-A	1000	Bozic	Inf	last	singleC	J1:DiP-A, J1:OT-A, J5:DiP-A, J5:OT-A, S1:DiP-A, S5:DiP-A, S5:OT-A
296	Yes	7	7-A	1000	Bozic	Inf	last	wholeT_0.01	J5:CBN-A
297	Yes	7	7-A	1000	Bozic	Inf	last	wholeT_0.5	J1:DiP-A, J1:OT-A, J5:DiP-A, J5:OT-A, S1:DiP-A, S5:DiP-A, S5:OT-A
298	Yes	7	7-A	1000	Bozic	Inf	unif	singleC	J1:CBN, J1:CBN-A, J5:CBN, J5:CBN-A, J5:OT, J5:OT-A, S1:CBN, S1:CBN-A, S5:CBN, S5:CBN-A
299	Yes	7	7-A	1000	Bozic	Inf	unif	wholeT_0.01	J5:CBN, J5:CBN-A, S5:CBN, S5:CBN-A
300	Yes	7	7-A	1000	Bozic	Inf	unif	wholeT_0.5	J1:CBN, J1:CBN-A, S1:CBN, S1:CBN-A
301	Yes	7	7-A	1000	exp	0	last	singleC	J1:CBN-A, J1:OT-A, S1:OT-A

Table 6: *(continued)*

	Conjunction	Drivers	Tree	S.Size	Model	sh	S.Time	S.Type	Best method(s)
302	Yes	7	7-A	1000	exp	0	last	wholeT_0.01	J1:CBN, J1:CBN-A, J1:OT-A, J5:CBN-A, J5:OT-A, S1:CBN, S1:CBN-A, S1:OT-A, S5:CBN, S5:OT-A
303	Yes	7	7-A	1000	exp	0	last	wholeT_0.5	J1:CBN-A, J1:OT-A, S1:OT-A
304	Yes	7	7-A	1000	exp	0	unif	singleC	NA
305	Yes	7	7-A	1000	exp	0	unif	wholeT_0.01	J1:CBN, J1:OT-A
306	Yes	7	7-A	1000	exp	0	unif	wholeT_0.5	NA
307	Yes	7	7-A	1000	exp	Inf	last	singleC	J1:OT-A, J5:CBN-A, J5:OT-A, S5:OT-A
308	Yes	7	7-A	1000	exp	Inf	last	wholeT_0.01	J1:CBN-A, J5:CBN-A, J5:DiP-A, J5:OT-A, S5:CBN-A, S5:DiP-A, S5:OT-A
309	Yes	7	7-A	1000	exp	Inf	last	wholeT_0.5	J1:OT-A, J5:CBN-A, J5:OT-A, S1:OT-A, S5:OT-A
310	Yes	7	7-A	1000	exp	Inf	unif	singleC	J1:CBN, J1:CBN-A, J1:OT, J1:OT-A, S1:CBN, S1:CBN-A
311	Yes	7	7-A	1000	exp	Inf	unif	wholeT_0.01	J1:CBN, J1:CBN-A, J5:CBN, J5:CBN-A, S5:CBN, S5:CBN-A
312	Yes	7	7-A	1000	exp	Inf	unif	wholeT_0.5	J1:CBN, J1:CBN-A, J1:OT, J1:OT-A, S1:CBN, S1:CBN-A
313	Yes	7	7-A	1000	McF_4	0	last	singleC	J1:DiP-A, J1:OT-A, J5:DiP-A, J5:OT-A, S1:DiP-A, S1:OT-A, S5:DiP-A, S5:OT-A
314	Yes	7	7-A	1000	McF_4	0	last	wholeT_0.01	J1:DiP-A, J1:OT-A, J5:DiP-A, J5:OT-A, S5:OT, S5:OT-A
315	Yes	7	7-A	1000	McF_4	0	last	wholeT_0.5	J1:DiP-A, J1:OT-A, J5:DiP-A, J5:OT-A, S1:DiP-A, S1:OT-A, S5:DiP-A, S5:OT-A
316	Yes	7	7-A	1000	McF_4	0	unif	singleC	J1:CBN-A, J1:DiP, J1:DiP-A, J1:OT, J1:OT-A, J5:CBN-A, J5:DiP, J5:DiP-A, J5:OT, J5:OT-A, S1:CBN-A, S1:DiP, S1:DiP-A, S1:OT, S1:OT-A, S5:CBN-A, S5:DiP, S5:DiP-A, S5:OT, S5:OT-A
317	Yes	7	7-A	1000	McF_4	0	unif	wholeT_0.01	J1:CBN, J1:DiP, J1:DiP-A, J1:OT, J1:OT-A, J5:CBN, J5:DiP, J5:DiP-A, J5:OT, J5:OT-A, S1:DiP, S1:DiP-A, S5:CBN, S5:DiP, S5:DiP-A, S5:OT, S5:OT-A
318	Yes	7	7-A	1000	McF_4	0	unif	wholeT_0.5	J1:CBN-A, J1:DiP, J1:DiP-A, J1:OT, J1:OT-A, J5:CBN-A, J5:DiP, J5:DiP-A, J5:OT, J5:OT-A, S1:CBN-A, S1:DiP, S1:DiP-A, S1:OT, S1:OT-A, S5:CBN-A, S5:DiP, S5:DiP-A, S5:OT, S5:OT-A

Table 6: *(continued)*

	Conjunction	Drivers	Tree	S.Size	Model	sh	S.Time	S.Type	Best method(s)
319	Yes	7	7-A	1000	McF_4	Inf	last	singleC	J1:DiP-A, J1:OT-A, J5:DiP-A, J5:OT-A, S1:DiP-A, S1:OT-A, S5:DiP-A, S5:OT-A
320	Yes	7	7-A	1000	McF_4	Inf	last	wholeT_0.01	J1:DiP-A, J5:DiP-A, J5:OT-A, S5:DiP-A, S5:OT-A
321	Yes	7	7-A	1000	McF_4	Inf	last	wholeT_0.5	J1:DiP-A, J1:OT-A, J5:DiP-A, J5:OT-A, S1:DiP-A, S1:OT-A, S5:DiP-A, S5:OT-A
322	Yes	7	7-A	1000	McF_4	Inf	unif	singleC	J1:DiP-A, J1:OT, J1:OT-A, J5:DiP-A, J5:OT, J5:OT-A, S1:DiP-A, S1:OT, S1:OT-A, S5:DiP-A, S5:OT, S5:OT-A
323	Yes	7	7-A	1000	McF_4	Inf	unif	wholeT_0.01	J1:DiP-A, J1:OT, J1:OT-A, J5:DiP-A, J5:OT, J5:OT-A, S5:DiP-A, S5:OT, S5:OT-A
324	Yes	7	7-A	1000	McF_4	Inf	unif	wholeT_0.5	J1:DiP-A, J1:OT, J1:OT-A, J5:DiP-A, J5:OT, J5:OT-A, S1:DiP-A, S1:OT, S1:OT-A, S5:DiP-A, S5:OT, S5:OT-A
325	Yes	7	7-A	1000	McF_6	0	last	singleC	J1:DiP, J1:OT, J1:OT-A, J5:DiP, J5:OT, J5:OT-A, S1:DiP, S5:DiP, S5:OT, S5:OT-A
326	Yes	7	7-A	1000	McF_6	0	last	wholeT_0.01	J1:DiP, J1:OT, J1:OT-A, J5:DiP, J5:OT, J5:OT-A, S1:DiP, S5:DiP, S5:OT, S5:OT-A
327	Yes	7	7-A	1000	McF_6	0	last	wholeT_0.5	J1:DiP, J1:OT, J1:OT-A, J5:DiP, J5:OT, J5:OT-A, S1:DiP, S5:DiP, S5:OT, S5:OT-A
328	Yes	7	7-A	1000	McF_6	0	unif	singleC	none
329	Yes	7	7-A	1000	McF_6	0	unif	wholeT_0.01	J1:CBN, J1:CBN-A, J1:DiP, J1:DiP-A, J1:OT, J1:OT-A, J5:CBN-A, J5:DiP, J5:DiP-A, J5:OT, J5:OT-A, S5:CBN, S5:CBN-A, S5:DiP, S5:DiP-A, S5:OT, S5:OT-A
330	Yes	7	7-A	1000	McF_6	0	unif	wholeT_0.5	none
331	Yes	7	7-A	1000	McF_6	Inf	last	singleC	J1:DiP, J1:DiP-A, J1:OT, J1:OT-A, J5:DiP, J5:DiP-A, J5:OT, J5:OT-A, S1:DiP, S1:DiP-A, S5:DiP, S5:DiP-A, S5:OT, S5:OT-A
332	Yes	7	7-A	1000	McF_6	Inf	last	wholeT_0.01	J5:OT, J5:OT-A, S5:OT, S5:OT-A
333	Yes	7	7-A	1000	McF_6	Inf	last	wholeT_0.5	J1:DiP, J1:DiP-A, J1:OT, J1:OT-A, J5:DiP, J5:DiP-A, J5:OT, J5:OT-A, S1:DiP, S1:DiP-A, S5:DiP, S5:DiP-A, S5:OT, S5:OT-A

Table 6: *(continued)*

	Conjunction	Drivers	Tree	S.Size	Model	sh	S.Time	S.Type	Best method(s)
334	Yes	7	7-A	1000	McF_6	Inf	unif	singleC	J1:CBN, J1:CBN-A, J1:DiP, J1:DiP-A, J1:OT, J1:OT-A, J5:CBN-A, J5:DiP-A, J5:OT, J5:OT-A, S1:CBN, S1:CBN-A, S1:DiP, S1:DiP-A, S1:OT, S1:OT-A, S5:CBN, S5:CBN-A, S5:DiP, S5:DiP-A, S5:OT, S5:OT-A
335	Yes	7	7-A	1000	McF_6	Inf	unif	wholeT_0.01	J1:CBN, J1:CBN-A, J1:DiP, J1:DiP-A, J1:OT, J1:OT-A, J5:CBN-A, J5:DiP-A, J5:OT, J5:OT-A, S5:CBN, S5:CBN-A, S5:DiP, S5:DiP-A, S5:OT, S5:OT-A
336	Yes	7	7-A	1000	McF_6	Inf	unif	wholeT_0.5	J1:CBN, J1:CBN-A, J1:DiP, J1:DiP-A, J1:OT, J1:OT-A, J5:CBN-A, J5:DiP-A, J5:OT, J5:OT-A, S1:CBN, S1:CBN-A, S1:DiP, S1:DiP-A, S1:OT, S1:OT-A, S5:CBN, S5:CBN-A, S5:DiP, S5:DiP-A, S5:OT, S5:OT-A
337	Yes	7	7-A	200	Bozic	0	last	singleC	J1:CBN-A, J1:OT-A, J5:CBN-A, J5:OT-A, S1:OT-A, S5:CBN, S5:OT-A
338	Yes	7	7-A	200	Bozic	0	last	wholeT_0.01	J1:OT-A, J5:OT-A, S5:OT-A
339	Yes	7	7-A	200	Bozic	0	last	wholeT_0.5	J1:CBN-A, J1:OT-A, J5:CBN-A, J5:OT-A, S5:CBN-A, S5:OT-A
340	Yes	7	7-A	200	Bozic	0	unif	singleC	J1:CBN, J1:CBN-A, J1:OT, J1:OT-A, S1:OT, S1:OT-A
341	Yes	7	7-A	200	Bozic	0	unif	wholeT_0.01	J5:CBN, J5:OT, J5:OT-A
342	Yes	7	7-A	200	Bozic	0	unif	wholeT_0.5	J1:CBN, J1:CBN-A, J1:OT, J1:OT-A, S1:CBN, S1:CBN-A, S1:OT, S1:OT-A
343	Yes	7	7-A	200	Bozic	Inf	last	singleC	J5:OT-A, S5:OT-A
344	Yes	7	7-A	200	Bozic	Inf	last	wholeT_0.01	J5:CBN-A, J5:OT-A
345	Yes	7	7-A	200	Bozic	Inf	last	wholeT_0.5	J5:OT-A, S5:OT-A
346	Yes	7	7-A	200	Bozic	Inf	unif	singleC	J1:CBN, J1:CBN-A, J5:CBN, J5:CBN-A, J5:OT, J5:OT-A, S5:CBN, S5:CBN-A
347	Yes	7	7-A	200	Bozic	Inf	unif	wholeT_0.01	J5:CBN, J5:CBN-A, S5:CBN, S5:CBN-A
348	Yes	7	7-A	200	Bozic	Inf	unif	wholeT_0.5	J1:CBN, J1:CBN-A
349	Yes	7	7-A	200	exp	0	last	singleC	J1:CBN-A, J1:OT-A, S1:OT-A
350	Yes	7	7-A	200	exp	0	last	wholeT_0.01	J1:CBN, J1:CBN-A, J5:CBN-A, J5:OT-A, S1:CBN, S1:CBN-A, S5:CBN
351	Yes	7	7-A	200	exp	0	last	wholeT_0.5	J1:OT-A, S1:OT-A
352	Yes	7	7-A	200	exp	0	unif	singleC	NA
353	Yes	7	7-A	200	exp	0	unif	wholeT_0.01	J1:CBN, J1:CBN-A, J1:OT, J1:OT-A
354	Yes	7	7-A	200	exp	0	unif	wholeT_0.5	NA

Table 6: *(continued)*

	Conjunction	Drivers	Tree	S.Size	Model	sh	S.Time	S.Type	Best method(s)
355	Yes	7	7-A	200	exp	Inf	last	singleC	J1:OT-A, J5:CBN-A, J5:OT-A, S5:OT-A
356	Yes	7	7-A	200	exp	Inf	last	wholeT_0.01	J5:CBN-A, J5:OT-A, S5:CBN-A
357	Yes	7	7-A	200	exp	Inf	last	wholeT_0.5	J1:OT-A, J5:CBN-A, J5:OT-A, S5:OT-A
358	Yes	7	7-A	200	exp	Inf	unif	singleC	J1:CBN, J1:CBN-A, J1:OT, J1:OT-A
359	Yes	7	7-A	200	exp	Inf	unif	wholeT_0.01	J5:CBN, J5:CBN-A, J5:OT, J5:OT-A, S5:CBN, S5:CBN-A
360	Yes	7	7-A	200	exp	Inf	unif	wholeT_0.5	J1:CBN, J1:CBN-A, J1:OT, J1:OT-A, S1:CBN-A
361	Yes	7	7-A	200	McF_4	0	last	singleC	J1:OT-A, J5:OT-A, S5:OT-A
362	Yes	7	7-A	200	McF_4	0	last	wholeT_0.01	J5:OT-A, S5:OT-A
363	Yes	7	7-A	200	McF_4	0	last	wholeT_0.5	J1:OT-A, J5:OT-A, S5:OT-A
364	Yes	7	7-A	200	McF_4	0	unif	singleC	J1:CBN, J1:CBN-A, J1:OT, J1:OT-A, J5:CBN, J5:CBN-A, J5:OT, J5:OT-A, S5:CBN, S5:CBN-A, S5:OT, S5:OT-A
365	Yes	7	7-A	200	McF_4	0	unif	wholeT_0.01	J1:CBN, J1:CBN-A, J1:DiP-A, J1:OT, J1:OT-A, J5:CBN, J5:DiP-A, J5:OT, J5:OT-A, S1:DiP-A, S5:CBN, S5:CBN-A, S5:DiP-A, S5:OT, S5:OT-A
366	Yes	7	7-A	200	McF_4	0	unif	wholeT_0.5	J1:CBN-A, J1:OT, J1:OT-A, J5:CBN, J5:CBN-A, J5:OT, J5:OT-A, S5:CBN, S5:CBN-A, S5:OT, S5:OT-A
367	Yes	7	7-A	200	McF_4	Inf	last	singleC	J1:OT-A, J5:OT-A, S5:OT-A
368	Yes	7	7-A	200	McF_4	Inf	last	wholeT_0.01	J5:OT-A, S5:OT-A
369	Yes	7	7-A	200	McF_4	Inf	last	wholeT_0.5	J1:OT-A, J5:OT-A, S5:OT-A
370	Yes	7	7-A	200	McF_4	Inf	unif	singleC	J1:OT, J1:OT-A, J5:CBN-A, J5:OT, J5:OT-A, S5:OT, S5:OT-A
371	Yes	7	7-A	200	McF_4	Inf	unif	wholeT_0.01	J1:OT, J1:OT-A, J5:OT, J5:OT-A, S5:OT, S5:OT-A
372	Yes	7	7-A	200	McF_4	Inf	unif	wholeT_0.5	J1:OT, J1:OT-A, J5:OT, J5:OT-A, S5:OT, S5:OT-A
373	Yes	7	7-A	200	McF_6	0	last	singleC	J1:OT, J1:OT-A, J5:OT, J5:OT-A, S5:OT, S5:OT-A
374	Yes	7	7-A	200	McF_6	0	last	wholeT_0.01	J5:OT, J5:OT-A, S5:OT, S5:OT-A
375	Yes	7	7-A	200	McF_6	0	last	wholeT_0.5	J1:OT, J1:OT-A, J5:OT, J5:OT-A, S5:OT, S5:OT-A
376	Yes	7	7-A	200	McF_6	0	unif	singleC	J1:CBN, J1:CBN-A, J1:DiP-A, J5:CBN, J5:CBN-A, S1:DiP-A, S5:CBN, S5:CBN-A, S5:DiP-A

Table 6: *(continued)*

	Conjunction	Drivers	Tree	S.Size	Model	sh	S.Time	S.Type	Best method(s)
377	Yes	7	7-A	200	McF_6	0	unif	wholeT_0.01	J1:CBN, J1:CBN-A, J1:DiP-A, J1:OT, J1:OT-A, J5:CBN, J5:CBN-A, J5:DiP-A, J5:OT, J5:OT-A, S1:DiP, S1:DiP-A, S5:CBN, S5:CBN-A, S5:DiP-A, S5:OT, S5:OT-A
378	Yes	7	7-A	200	McF_6	0	unif	wholeT_0.5	J1:CBN, J1:CBN-A, J5:CBN, J5:CBN-A, S5:CBN, S5:CBN-A
379	Yes	7	7-A	200	McF_6	Inf	last	singleC	J1:DiP-A, J5:DiP-A, J5:OT, J5:OT-A, S5:DiP-A, S5:OT, S5:OT-A
380	Yes	7	7-A	200	McF_6	Inf	last	wholeT_0.01	J5:OT, J5:OT-A, S5:OT, S5:OT-A
381	Yes	7	7-A	200	McF_6	Inf	last	wholeT_0.5	J1:DiP-A, J1:OT, J1:OT-A, J5:DiP-A, J5:OT, J5:OT-A, S5:DiP-A, S5:OT, S5:OT-A
382	Yes	7	7-A	200	McF_6	Inf	unif	singleC	J1:CBN, J1:CBN-A, J5:CBN-A, S5:CBN, S5:CBN-A
383	Yes	7	7-A	200	McF_6	Inf	unif	wholeT_0.01	J1:CBN, J1:CBN-A, J5:CBN-A, S5:CBN, S5:CBN-A
384	Yes	7	7-A	200	McF_6	Inf	unif	wholeT_0.5	J1:CBN, J1:CBN-A, J5:CBN-A, S5:CBN, S5:CBN-A
385	Yes	7	7-A	100	Bozic	0	last	singleC	J5:CBN-A, J5:OT-A, S5:CBN, S5:OT-A
386	Yes	7	7-A	100	Bozic	0	last	wholeT_0.01	J5:OT-A, S5:OT-A
387	Yes	7	7-A	100	Bozic	0	last	wholeT_0.5	J5:OT-A, S5:CBN-A, S5:OT-A
388	Yes	7	7-A	100	Bozic	0	unif	singleC	J1:CBN, J1:CBN-A, J1:OT, J1:OT-A, S1:OT, S1:OT-A
389	Yes	7	7-A	100	Bozic	0	unif	wholeT_0.01	J5:CBN, J5:CBN-A, J5:OT, J5:OT-A, S5:CBN-A
390	Yes	7	7-A	100	Bozic	0	unif	wholeT_0.5	J1:CBN, J1:CBN-A, J1:OT, J1:OT-A, S1:OT, S1:OT-A
391	Yes	7	7-A	100	Bozic	Inf	last	singleC	J5:OT-A
392	Yes	7	7-A	100	Bozic	Inf	last	wholeT_0.01	J5:CBN-A, J5:OT-A
393	Yes	7	7-A	100	Bozic	Inf	last	wholeT_0.5	J5:OT-A
394	Yes	7	7-A	100	Bozic	Inf	unif	singleC	J5:CBN, J5:CBN-A, J5:OT, J5:OT-A, S5:CBN, S5:CBN-A
395	Yes	7	7-A	100	Bozic	Inf	unif	wholeT_0.01	J5:CBN, J5:CBN-A, J5:OT, J5:OT-A, S5:CBN-A, S5:OT, S5:OT-A
396	Yes	7	7-A	100	Bozic	Inf	unif	wholeT_0.5	J5:CBN, J5:CBN-A, J5:OT, J5:OT-A, S5:CBN, S5:CBN-A
397	Yes	7	7-A	100	exp	0	last	singleC	J1:OT-A, S1:OT-A
398	Yes	7	7-A	100	exp	0	last	wholeT_0.01	J1:CBN, J1:CBN-A, J5:CBN-A, J5:OT-A, S5:CBN

Table 6: *(continued)*

	Conjunction	Drivers	Tree	S.Size	Model	sh	S.Time	S.Type	Best method(s)
399	Yes	7	7-A	100	exp	0	last	wholeT_0.5	J1:OT-A, S1:OT-A
400	Yes	7	7-A	100	exp	0	unif	singleC	J1:OT, J1:OT-A, S1:CBN, S1:CBN-A, S1:OT, S1:OT-A
401	Yes	7	7-A	100	exp	0	unif	wholeT_0.01	J1:CBN, J1:CBN-A, J1:OT, J1:OT-A, S1:OT, S1:OT-A
402	Yes	7	7-A	100	exp	0	unif	wholeT_0.5	NA
403	Yes	7	7-A	100	exp	Inf	last	singleC	J1:OT-A, J5:OT-A, S5:OT-A
404	Yes	7	7-A	100	exp	Inf	last	wholeT_0.01	J5:CBN-A, J5:OT-A
405	Yes	7	7-A	100	exp	Inf	last	wholeT_0.5	J1:CBN, J1:OT-A, J5:CBN-A, J5:OT-A, S5:OT-A
406	Yes	7	7-A	100	exp	Inf	unif	singleC	J1:CBN, J1:CBN-A, J1:OT, J1:OT-A
407	Yes	7	7-A	100	exp	Inf	unif	wholeT_0.01	J5:CBN, J5:CBN-A, J5:OT, J5:OT-A, S5:CBN, S5:CBN-A, S5:OT, S5:OT-A
408	Yes	7	7-A	100	exp	Inf	unif	wholeT_0.5	J1:CBN, J1:CBN-A, J1:OT, J1:OT-A, S1:OT, S1:OT-A
409	Yes	7	7-A	100	McF_4	0	last	singleC	J1:OT-A, J5:OT-A, S5:OT-A
410	Yes	7	7-A	100	McF_4	0	last	wholeT_0.01	J5:OT-A, S5:OT-A
411	Yes	7	7-A	100	McF_4	0	last	wholeT_0.5	J1:OT-A, J5:OT-A, S5:OT-A
412	Yes	7	7-A	100	McF_4	0	unif	singleC	J1:CBN, J1:CBN-A, J1:OT, J1:OT-A, J5:CBN, J5:CBN-A, J5:OT, J5:OT-A, S5:CBN-A, S5:OT, S5:OT-A
413	Yes	7	7-A	100	McF_4	0	unif	wholeT_0.01	J1:CBN-A, J5:CBN, J5:CBN-A, J5:OT, J5:OT-A, S5:CBN, S5:CBN-A, S5:OT, S5:OT-A
414	Yes	7	7-A	100	McF_4	0	unif	wholeT_0.5	J1:CBN-A, J1:OT, J1:OT-A, J5:CBN, J5:CBN-A, J5:OT, J5:OT-A, S5:CBN, S5:CBN-A, S5:OT, S5:OT-A
415	Yes	7	7-A	100	McF_4	Inf	last	singleC	J1:OT-A, J5:OT-A, S5:OT-A
416	Yes	7	7-A	100	McF_4	Inf	last	wholeT_0.01	J5:OT-A, S5:OT-A
417	Yes	7	7-A	100	McF_4	Inf	last	wholeT_0.5	J5:OT-A, S5:OT-A
418	Yes	7	7-A	100	McF_4	Inf	unif	singleC	J1:OT, J1:OT-A, J5:OT, J5:OT-A, S5:CBN-A, S5:OT, S5:OT-A
419	Yes	7	7-A	100	McF_4	Inf	unif	wholeT_0.01	J1:CBN-A, J5:CBN-A, J5:OT-A, S5:CBN-A, S5:OT, S5:OT-A
420	Yes	7	7-A	100	McF_4	Inf	unif	wholeT_0.5	J1:OT, J1:OT-A, J5:CBN-A, J5:OT, J5:OT-A, S5:OT, S5:OT-A
421	Yes	7	7-A	100	McF_6	0	last	singleC	J5:OT, J5:OT-A, S5:OT, S5:OT-A
422	Yes	7	7-A	100	McF_6	0	last	wholeT_0.01	J5:OT, J5:OT-A, S5:OT, S5:OT-A
423	Yes	7	7-A	100	McF_6	0	last	wholeT_0.5	J5:OT, J5:OT-A, S5:OT, S5:OT-A

Table 6: *(continued)*

	Conjunction	Drivers	Tree	S.Size	Model	sh	S.Time	S.Type	Best method(s)
424	Yes	7	7-A	100	McF_6	0	unif	singleC	J1:CBN, J1:CBN-A, J5:CBN, J5:CBN-A, S5:CBN, S5:CBN-A
425	Yes	7	7-A	100	McF_6	0	unif	wholeT_0.01	J1:DiP-A, J5:CBN, J5:CBN-A, J5:DiP-A, S5:CBN, S5:CBN-A, S5:DiP-A
426	Yes	7	7-A	100	McF_6	0	unif	wholeT_0.5	J1:CBN, J1:CBN-A, J5:CBN, J5:CBN-A, S5:CBN, S5:CBN-A
427	Yes	7	7-A	100	McF_6	Inf	last	singleC	J5:OT, J5:OT-A, S5:OT, S5:OT-A
428	Yes	7	7-A	100	McF_6	Inf	last	wholeT_0.01	J5:OT, J5:OT-A, S5:OT, S5:OT-A
429	Yes	7	7-A	100	McF_6	Inf	last	wholeT_0.5	J5:OT, J5:OT-A, S5:OT, S5:OT-A
430	Yes	7	7-A	100	McF_6	Inf	unif	singleC	J5:CBN, J5:CBN-A, S5:CBN, S5:CBN-A
431	Yes	7	7-A	100	McF_6	Inf	unif	wholeT_0.01	J5:CBN, J5:CBN-A, S5:CBN, S5:CBN-A
432	Yes	7	7-A	100	McF_6	Inf	unif	wholeT_0.5	J1:CBN, J1:CBN-A, J5:CBN, J5:CBN-A, S5:CBN, S5:CBN-A
433	No	11	11-B	1000	Bozic	0	last	singleC	S1:OT, S1:OT-A, S5:DiP, S5:DiP-A, S5:OT, S5:OT-A
434	No	11	11-B	1000	Bozic	0	last	wholeT_0.01	J1:DiP, J1:DiP-A, J5:DiP, J5:DiP-A, S1:DiP, S1:DiP-A, S5:DiP, S5:DiP-A
435	No	11	11-B	1000	Bozic	0	last	wholeT_0.5	S1:OT, S1:OT-A
436	No	11	11-B	1000	Bozic	0	unif	singleC	S1:CBN, S1:CBN-A
437	No	11	11-B	1000	Bozic	0	unif	wholeT_0.01	S5:CBN-A, S5:DiP, S5:DiP-A, S5:OT, S5:OT-A
438	No	11	11-B	1000	Bozic	0	unif	wholeT_0.5	S1:CBN, S1:CBN-A, S5:CBN, S5:CBN-A
439	No	11	11-B	1000	Bozic	Inf	last	singleC	J1:DiP, J1:DiP-A, J1:OT, J1:OT-A, J5:DiP, J5:DiP-A, J5:OT, J5:OT-A, S5:DiP, S5:DiP-A, S5:OT, S5:OT-A
440	No	11	11-B	1000	Bozic	Inf	last	wholeT_0.01	J5:DiP, J5:DiP-A, J5:OT, J5:OT-A
441	No	11	11-B	1000	Bozic	Inf	last	wholeT_0.5	J1:DiP, J1:DiP-A, J1:OT, J1:OT-A, J5:DiP, J5:DiP-A, J5:OT, J5:OT-A, S5:DiP, S5:DiP-A, S5:OT, S5:OT-A
442	No	11	11-B	1000	Bozic	Inf	unif	singleC	S1:CBN, S1:CBN-A, S1:DiP, S1:DiP-A, S1:OT, S1:OT-A
443	No	11	11-B	1000	Bozic	Inf	unif	wholeT_0.01	J1:CBN-A, J1:DiP, J1:DiP-A, J1:OT, J1:OT-A, S5:CBN, S5:CBN-A, S5:DiP, S5:DiP-A, S5:OT, S5:OT-A
444	No	11	11-B	1000	Bozic	Inf	unif	wholeT_0.5	J1:CBN, J1:CBN-A, S1:CBN, S1:CBN-A, S1:DiP-A, S1:OT, S1:OT-A
445	No	11	11-B	1000	exp	0	last	singleC	S1:OT, S1:OT-A
446	No	11	11-B	1000	exp	0	last	wholeT_0.01	J1:DiP, J1:DiP-A, J1:OT, J1:OT-A, S1:DiP-A, S1:OT, S1:OT-A, S5:DiP, S5:DiP-A, S5:OT, S5:OT-A

Table 6: *(continued)*

	Conjunction	Drivers	Tree	S.Size	Model	sh	S.Time	S.Type	Best method(s)
447	No	11	11-B	1000	exp	0	last	wholeT_0.5	S1:OT, S1:OT-A
448	No	11	11-B	1000	exp	0	unif	singleC	S1:CBN, S1:CBN-A, S5:CBN, S5:CBN-A
449	No	11	11-B	1000	exp	0	unif	wholeT_0.01	S1:DiP, S1:DiP-A, S1:OT, S1:OT-A
450	No	11	11-B	1000	exp	0	unif	wholeT_0.5	S1:CBN, S1:CBN-A, S5:CBN, S5:CBN-A
451	No	11	11-B	1000	exp	Inf	last	singleC	J1:DiP, J1:DiP-A, J1:OT, J1:OT-A, S1:DiP-A, S5:DiP, S5:DiP-A, S5:OT, S5:OT-A
452	No	11	11-B	1000	exp	Inf	last	wholeT_0.01	J5:DiP, J5:DiP-A, J5:OT, J5:OT-A, S5:DiP, S5:DiP-A, S5:OT, S5:OT-A
453	No	11	11-B	1000	exp	Inf	last	wholeT_0.5	J1:DiP, J1:DiP-A, J1:OT, J1:OT-A, S5:DiP, S5:DiP-A, S5:OT, S5:OT-A
454	No	11	11-B	1000	exp	Inf	unif	singleC	S1:CBN-A, S1:OT, S1:OT-A
455	No	11	11-B	1000	exp	Inf	unif	wholeT_0.01	J1:DiP, J1:DiP-A, J1:OT, J1:OT-A
456	No	11	11-B	1000	exp	Inf	unif	wholeT_0.5	S1:CBN-A, S1:OT, S1:OT-A
457	No	11	11-B	1000	McF_4	0	last	singleC	J1:CBN, J1:DiP, J1:DiP-A, J1:OT, J1:OT-A, J5:CBN, J5:DiP, J5:DiP-A, J5:OT, J5:OT-A, S1:DiP-A, S1:OT, S1:OT-A, S5:DiP, S5:DiP-A, S5:OT, S5:OT-A
458	No	11	11-B	1000	McF_4	0	last	wholeT_0.01	J1:DiP, J1:DiP-A, J1:OT, J1:OT-A, J5:CBN, J5:DiP, J5:DiP-A, J5:OT, J5:OT-A, S1:DiP, S1:DiP-A, S5:DiP, S5:DiP-A, S5:OT, S5:OT-A
459	No	11	11-B	1000	McF_4	0	last	wholeT_0.5	J1:CBN, J1:DiP, J1:DiP-A, J1:OT, J1:OT-A, J5:CBN, J5:DiP, J5:DiP-A, J5:OT, J5:OT-A, S1:DiP-A, S1:OT, S1:OT-A, S5:DiP, S5:DiP-A, S5:OT, S5:OT-A
460	No	11	11-B	1000	McF_4	0	unif	singleC	J1:CBN, J1:CBN-A, J1:DiP, J1:DiP-A, J1:OT, J1:OT-A, S1:DiP, S1:DiP-A, S1:OT, S1:OT-A, S5:CBN-A, S5:DiP, S5:DiP-A, S5:OT, S5:OT-A
461	No	11	11-B	1000	McF_4	0	unif	wholeT_0.01	J1:CBN-A, J1:DiP, J1:DiP-A, J1:OT, J1:OT-A, S5:DiP, S5:DiP-A, S5:OT, S5:OT-A
462	No	11	11-B	1000	McF_4	0	unif	wholeT_0.5	J1:CBN-A, J1:DiP, J1:DiP-A, J1:OT, J1:OT-A, S1:DiP, S1:DiP-A, S1:OT, S1:OT-A, S5:CBN, S5:DiP, S5:DiP-A, S5:OT, S5:OT-A

Table 6: *(continued)*

	Conjunction	Drivers	Tree	S.Size	Model	sh	S.Time	S.Type	Best method(s)
463	No	11	11-B	1000	McF_4	Inf	last	singleC	J1:DiP, J1:DiP-A, J1:OT, J1:OT-A, J5:DiP, J5:DiP-A, J5:OT, J5:OT-A, S1:OT, S1:OT-A, S5:DiP, S5:DiP-A, S5:OT, S5:OT-A
464	No	11	11-B	1000	McF_4	Inf	last	wholeT_0.01	J1:DiP, J1:DiP-A, J1:OT, J1:OT-A, J5:DiP, J5:DiP-A, J5:OT, J5:OT-A, S1:DiP, S1:DiP-A, S5:DiP, S5:DiP-A, S5:OT, S5:OT-A
465	No	11	11-B	1000	McF_4	Inf	last	wholeT_0.5	J1:DiP, J1:DiP-A, J1:OT, J1:OT-A, J5:DiP, J5:DiP-A, J5:OT, J5:OT-A, S1:DiP-A, S1:OT, S1:OT-A, S5:DiP, S5:DiP-A, S5:OT, S5:OT-A
466	No	11	11-B	1000	McF_4	Inf	unif	singleC	J1:CBN-A, J1:DiP, J1:DiP-A, J1:OT, J1:OT-A, S1:DiP, S1:DiP-A, S1:OT, S1:OT-A, S5:DiP, S5:DiP-A, S5:OT, S5:OT-A
467	No	11	11-B	1000	McF_4	Inf	unif	wholeT_0.01	J1:CBN-A, J1:DiP, J1:DiP-A, J1:OT, J1:OT-A, S5:DiP, S5:DiP-A, S5:OT, S5:OT-A
468	No	11	11-B	1000	McF_4	Inf	unif	wholeT_0.5	J1:CBN-A, J1:DiP, J1:DiP-A, J1:OT, J1:OT-A, S1:DiP, S1:DiP-A, S1:OT, S1:OT-A, S5:CBN-A, S5:DiP, S5:DiP-A, S5:OT, S5:OT-A
469	No	11	11-B	1000	McF_6	0	last	singleC	J1:DiP, J1:OT, J5:DiP, J5:OT, S1:DiP, S5:DiP, S5:OT
470	No	11	11-B	1000	McF_6	0	last	wholeT_0.01	J1:DiP, J1:OT, J5:DiP, J5:OT, S1:DiP, S5:DiP, S5:OT
471	No	11	11-B	1000	McF_6	0	last	wholeT_0.5	J1:DiP, J1:OT, J5:DiP, J5:OT, S1:DiP, S5:DiP, S5:OT
472	No	11	11-B	1000	McF_6	0	unif	singleC	J1:CBN, J1:CBN-A, J1:DiP, J1:DiP-A, J1:OT, J1:OT-A, J5:CBN, J5:CBN-A, J5:DiP, J5:DiP-A, J5:OT, J5:OT-A, S1:DiP, S1:DiP-A, S1:OT, S1:OT-A, S5:CBN, S5:CBN-A, S5:DiP, S5:DiP-A, S5:OT, S5:OT-A
473	No	11	11-B	1000	McF_6	0	unif	wholeT_0.01	J1:CBN, J1:CBN-A, J1:DiP, J1:DiP-A, J1:OT, J1:OT-A, J5:CBN, J5:CBN-A, J5:DiP, J5:DiP-A, J5:OT, J5:OT-A, S1:DiP, S1:DiP-A, S5:CBN, S5:DiP, S5:DiP-A, S5:OT, S5:OT-A

Table 6: *(continued)*

	Conjunction	Drivers	Tree	S.Size	Model	sh	S.Time	S.Type	Best method(s)
474	No	11	11-B	1000	McF_6	0	unif	wholeT_0.5	J1:CBN, J1:CBN-A, J1:DiP, J1:DiP-A, J1:OT, J1:OT-A, J5:CBN, J5:CBN-A, J5:DiP, J5:DiP-A, J5:OT, J5:OT-A, S1:DiP, S1:DiP-A, S1:OT, S1:OT-A, S5:CBN, S5:CBN-A, S5:DiP, S5:DiP-A, S5:OT, S5:OT-A
475	No	11	11-B	1000	McF_6	Inf	last	singleC	J1:DiP, J1:OT, J5:DiP, J5:OT, S1:DiP, S5:DiP, S5:OT
476	No	11	11-B	1000	McF_6	Inf	last	wholeT_0.01	J1:DiP, J1:OT, J5:DiP, J5:OT, S1:DiP, S5:DiP, S5:OT
477	No	11	11-B	1000	McF_6	Inf	last	wholeT_0.5	J1:DiP, J1:OT, J5:DiP, J5:OT, S1:DiP, S1:OT, S5:DiP, S5:OT
478	No	11	11-B	1000	McF_6	Inf	unif	singleC	J1:CBN-A, J1:DiP, J1:DiP-A, J1:OT, J1:OT-A, J5:CBN-A, J5:DiP, J5:DiP-A, J5:OT, J5:OT-A, S1:DiP, S1:DiP-A, S5:CBN, S5:CBN-A, S5:DiP, S5:DiP-A, S5:OT, S5:OT-A
479	No	11	11-B	1000	McF_6	Inf	unif	wholeT_0.01	J1:CBN-A, J1:DiP, J1:DiP-A, J1:OT, J1:OT-A, J5:CBN-A, J5:DiP, J5:DiP-A, J5:OT, J5:OT-A, S1:DiP, S1:DiP-A, S5:CBN, S5:DiP, S5:DiP-A, S5:OT, S5:OT-A
480	No	11	11-B	1000	McF_6	Inf	unif	wholeT_0.5	J1:CBN, J1:CBN-A, J1:DiP, J1:DiP-A, J1:OT, J1:OT-A, J5:CBN-A, J5:DiP, J5:DiP-A, J5:OT, J5:OT-A, S1:DiP, S1:DiP-A, S5:CBN, S5:CBN-A, S5:DiP, S5:DiP-A, S5:OT, S5:OT-A
481	No	11	11-B	200	Bozic	0	last	singleC	S5:OT, S5:OT-A
482	No	11	11-B	200	Bozic	0	last	wholeT_0.01	J1:OT, J1:OT-A, J5:OT, J5:OT-A, S5:OT, S5:OT-A
483	No	11	11-B	200	Bozic	0	last	wholeT_0.5	S1:OT, S1:OT-A
484	No	11	11-B	200	Bozic	0	unif	singleC	S1:OT, S1:OT-A
485	No	11	11-B	200	Bozic	0	unif	wholeT_0.01	J1:CBN, J1:CBN-A, S5:DiP-A, S5:OT, S5:OT-A
486	No	11	11-B	200	Bozic	0	unif	wholeT_0.5	S1:CBN, S1:CBN-A
487	No	11	11-B	200	Bozic	Inf	last	singleC	J5:OT, J5:OT-A, S5:OT, S5:OT-A
488	No	11	11-B	200	Bozic	Inf	last	wholeT_0.01	J5:OT, J5:OT-A
489	No	11	11-B	200	Bozic	Inf	last	wholeT_0.5	J5:OT, J5:OT-A, S5:OT, S5:OT-A
490	No	11	11-B	200	Bozic	Inf	unif	singleC	J1:CBN, J1:CBN-A, J1:OT, J1:OT-A

Table 6: *(continued)*

	Conjunction	Drivers	Tree	S.Size	Model	sh	S.Time	S.Type	Best method(s)
491	No	11	11-B	200	Bozic	Inf	unif	wholeT_0.01	J5:CBN, J5:CBN-A, S5:CBN, S5:CBN-A, S5:OT, S5:OT-A
492	No	11	11-B	200	Bozic	Inf	unif	wholeT_0.5	J1:CBN, J1:CBN-A, J1:OT, J1:OT-A
493	No	11	11-B	200	exp	0	last	singleC	S1:OT, S1:OT-A
494	No	11	11-B	200	exp	0	last	wholeT_0.01	J1:OT, J1:OT-A, S1:OT, S1:OT-A, S5:OT, S5:OT-A
495	No	11	11-B	200	exp	0	last	wholeT_0.5	S1:OT, S1:OT-A
496	No	11	11-B	200	exp	0	unif	singleC	S1:CBN, S1:CBN-A, S5:CBN, S5:CBN-A
497	No	11	11-B	200	exp	0	unif	wholeT_0.01	S1:OT, S1:OT-A
498	No	11	11-B	200	exp	0	unif	wholeT_0.5	S1:CBN, S1:CBN-A, S5:CBN, S5:CBN-A
499	No	11	11-B	200	exp	Inf	last	singleC	J1:OT, J1:OT-A, S5:OT, S5:OT-A
500	No	11	11-B	200	exp	Inf	last	wholeT_0.01	J5:OT, J5:OT-A
501	No	11	11-B	200	exp	Inf	last	wholeT_0.5	J1:OT, J1:OT-A, S5:OT, S5:OT-A
502	No	11	11-B	200	exp	Inf	unif	singleC	S1:OT, S1:OT-A
503	No	11	11-B	200	exp	Inf	unif	wholeT_0.01	S5:OT, S5:OT-A
504	No	11	11-B	200	exp	Inf	unif	wholeT_0.5	J1:CBN-A, S1:OT, S1:OT-A
505	No	11	11-B	200	McF_4	0	last	singleC	J1:DiP-A, J1:OT, J1:OT-A, J5:DiP-A, J5:OT, J5:OT-A, S1:DiP-A, S5:DiP, S5:DiP-A, S5:OT, S5:OT-A
506	No	11	11-B	200	McF_4	0	last	wholeT_0.01	J1:DiP, J1:DiP-A, J1:OT, J1:OT-A, J5:CBN, J5:DiP, J5:DiP-A, J5:OT, J5:OT-A, S1:DiP, S1:DiP-A, S5:DiP, S5:DiP-A, S5:OT, S5:OT-A
507	No	11	11-B	200	McF_4	0	last	wholeT_0.5	J1:DiP-A, J1:OT, J1:OT-A, J5:DiP-A, J5:OT, J5:OT-A, S5:DiP, S5:DiP-A, S5:OT, S5:OT-A
508	No	11	11-B	200	McF_4	0	unif	singleC	J1:CBN, J1:CBN-A, J1:OT, J1:OT-A, S5:DiP, S5:DiP-A, S5:OT, S5:OT-A
509	No	11	11-B	200	McF_4	0	unif	wholeT_0.01	J1:CBN-A, J1:DiP, J1:DiP-A, J1:OT, J1:OT-A, S1:DiP, S1:DiP-A, S5:DiP, S5:DiP-A, S5:OT, S5:OT-A
510	No	11	11-B	200	McF_4	0	unif	wholeT_0.5	J1:CBN-A, J1:OT, J1:OT-A, S5:CBN-A, S5:OT, S5:OT-A
511	No	11	11-B	200	McF_4	Inf	last	singleC	J1:OT, J1:OT-A, J5:OT, J5:OT-A, S5:OT, S5:OT-A
512	No	11	11-B	200	McF_4	Inf	last	wholeT_0.01	J1:DiP-A, J1:OT, J1:OT-A, J5:OT, J5:OT-A, S1:DiP-A, S5:DiP-A, S5:OT, S5:OT-A
513	No	11	11-B	200	McF_4	Inf	last	wholeT_0.5	J1:OT, J1:OT-A, J5:OT, J5:OT-A, S5:OT, S5:OT-A

Table 6: *(continued)*

	Conjunction	Drivers	Tree	S.Size	Model	sh	S.Time	S.Type	Best method(s)
514	No	11	11-B	200	McF_4	Inf	unif	singleC	J1:CBN-A, J1:OT, J1:OT-A, S5:CBN-A, S5:OT, S5:OT-A
515	No	11	11-B	200	McF_4	Inf	unif	wholeT_0.01	J1:OT, J1:OT-A, J5:OT, J5:OT-A, S5:CBN-A, S5:OT, S5:OT-A
516	No	11	11-B	200	McF_4	Inf	unif	wholeT_0.5	J1:CBN-A, J1:OT, J1:OT-A, S5:OT, S5:OT-A
517	No	11	11-B	200	McF_6	0	last	singleC	J1:DiP, J1:OT, J5:DiP, J5:OT, S1:DiP, S5:DiP, S5:OT
518	No	11	11-B	200	McF_6	0	last	wholeT_0.01	J1:DiP, J1:OT, J5:DiP, J5:OT, S1:DiP, S5:DiP, S5:OT
519	No	11	11-B	200	McF_6	0	last	wholeT_0.5	J1:DiP, J1:OT, J5:OT, S1:DiP, S5:DiP, S5:OT
520	No	11	11-B	200	McF_6	0	unif	singleC	J1:CBN, J1:CBN-A, J1:DiP, J1:OT, J1:OT-A, J5:CBN, J5:CBN-A, J5:DiP, J5:DiP-A, J5:OT, J5:OT-A, S5:CBN, S5:CBN-A, S5:OT, S5:OT-A
521	No	11	11-B	200	McF_6	0	unif	wholeT_0.01	J1:CBN, J1:CBN-A, J1:DiP, J1:DiP-A, J1:OT, J1:OT-A, J5:CBN, J5:CBN-A, J5:DiP, J5:DiP-A, J5:OT, J5:OT-A, S5:CBN-A, S5:DiP, S5:DiP-A, S5:OT, S5:OT-A
522	No	11	11-B	200	McF_6	0	unif	wholeT_0.5	J1:CBN, J1:CBN-A, J1:DiP, J1:OT, J1:OT-A, J5:CBN, J5:CBN-A, J5:DiP-A, J5:OT, J5:OT-A, S5:OT, S5:OT-A
523	No	11	11-B	200	McF_6	Inf	last	singleC	J1:OT, J5:OT, S5:OT
524	No	11	11-B	200	McF_6	Inf	last	wholeT_0.01	J5:OT, S1:DiP, S5:DiP, S5:OT
525	No	11	11-B	200	McF_6	Inf	last	wholeT_0.5	J1:OT, J5:OT, S1:DiP, S5:DiP, S5:OT
526	No	11	11-B	200	McF_6	Inf	unif	singleC	J1:CBN, J1:CBN-A, J1:OT, J1:OT-A, J5:CBN-A, J5:DiP-A, J5:OT, J5:OT-A, S5:CBN-A, S5:OT, S5:OT-A
527	No	11	11-B	200	McF_6	Inf	unif	wholeT_0.01	J1:CBN-A, J1:OT, J1:OT-A, J5:CBN-A, J5:DiP-A, J5:OT, J5:OT-A, S5:CBN, S5:OT, S5:OT-A
528	No	11	11-B	200	McF_6	Inf	unif	wholeT_0.5	J1:CBN-A, J5:CBN-A, J5:OT, J5:OT-A, S5:CBN-A
529	No	11	11-B	100	Bozic	0	last	singleC	S5:OT, S5:OT-A
530	No	11	11-B	100	Bozic	0	last	wholeT_0.01	J5:CBN-A, J5:OT, J5:OT-A, S5:OT, S5:OT-A
531	No	11	11-B	100	Bozic	0	last	wholeT_0.5	J1:OT, J1:OT-A, S1:OT, S1:OT-A
532	No	11	11-B	100	Bozic	0	unif	singleC	S1:OT, S1:OT-A

Table 6: *(continued)*

	Conjunction	Drivers	Tree	S.Size	Model	sh	S.Time	S.Type	Best method(s)
533	No	11	11-B	100	Bozic	0	unif	wholeT_0.01	J1:CBN-A, J1:OT, J1:OT-A, S5:OT, S5:OT-A
534	No	11	11-B	100	Bozic	0	unif	wholeT_0.5	J1:CBN, S1:OT, S1:OT-A
535	No	11	11-B	100	Bozic	Inf	last	singleC	J5:OT, J5:OT-A
536	No	11	11-B	100	Bozic	Inf	last	wholeT_0.01	J5:OT, J5:OT-A
537	No	11	11-B	100	Bozic	Inf	last	wholeT_0.5	J5:OT, J5:OT-A
538	No	11	11-B	100	Bozic	Inf	unif	singleC	J1:OT, J1:OT-A, J5:CBN, J5:CBN-A, S1:OT, S1:OT-A, S5:CBN, S5:CBN-A
539	No	11	11-B	100	Bozic	Inf	unif	wholeT_0.01	J5:CBN, J5:CBN-A, S5:CBN, S5:CBN-A, S5:OT, S5:OT-A
540	No	11	11-B	100	Bozic	Inf	unif	wholeT_0.5	J1:CBN, J1:CBN-A, J1:OT, J1:OT-A, S1:OT, S1:OT-A, S5:CBN, S5:CBN-A
541	No	11	11-B	100	exp	0	last	singleC	S1:OT, S1:OT-A
542	No	11	11-B	100	exp	0	last	wholeT_0.01	J1:OT, J1:OT-A, S5:OT, S5:OT-A
543	No	11	11-B	100	exp	0	last	wholeT_0.5	S1:OT, S1:OT-A
544	No	11	11-B	100	exp	0	unif	singleC	J1:CBN, J1:CBN-A, S1:CBN-A, S1:OT, S1:OT-A
545	No	11	11-B	100	exp	0	unif	wholeT_0.01	S1:OT, S1:OT-A
546	No	11	11-B	100	exp	0	unif	wholeT_0.5	J1:CBN, J1:CBN-A, S1:OT, S1:OT-A
547	No	11	11-B	100	exp	Inf	last	singleC	J5:OT, J5:OT-A, S5:OT, S5:OT-A
548	No	11	11-B	100	exp	Inf	last	wholeT_0.01	J5:CBN-A, J5:OT, J5:OT-A
549	No	11	11-B	100	exp	Inf	last	wholeT_0.5	S5:OT, S5:OT-A
550	No	11	11-B	100	exp	Inf	unif	singleC	J1:CBN, J1:OT, J1:OT-A, S1:OT, S1:OT-A, S5:CBN, S5:CBN-A
551	No	11	11-B	100	exp	Inf	unif	wholeT_0.01	S5:CBN-A, S5:OT, S5:OT-A
552	No	11	11-B	100	exp	Inf	unif	wholeT_0.5	J1:CBN-A, J1:OT, J1:OT-A, S1:OT, S1:OT-A
553	No	11	11-B	100	McF_4	0	last	singleC	J5:OT, J5:OT-A, S5:OT, S5:OT-A
554	No	11	11-B	100	McF_4	0	last	wholeT_0.01	J1:DiP, J1:DiP-A, J5:DiP-A, J5:OT, J5:OT-A, S1:DiP, S1:DiP-A, S5:DiP, S5:DiP-A, S5:OT, S5:OT-A
555	No	11	11-B	100	McF_4	0	last	wholeT_0.5	J5:OT, J5:OT-A, S5:OT, S5:OT-A
556	No	11	11-B	100	McF_4	0	unif	singleC	J1:CBN-A, J1:OT, J1:OT-A, S5:CBN-A, S5:OT, S5:OT-A
557	No	11	11-B	100	McF_4	0	unif	wholeT_0.01	J1:DiP, J1:DiP-A, S1:DiP, S1:DiP-A, S5:CBN, S5:CBN-A, S5:DiP, S5:DiP-A, S5:OT, S5:OT-A
558	No	11	11-B	100	McF_4	0	unif	wholeT_0.5	J1:CBN-A, J1:OT, J1:OT-A, S5:CBN, S5:OT, S5:OT-A
559	No	11	11-B	100	McF_4	Inf	last	singleC	J5:OT, J5:OT-A, S5:OT, S5:OT-A

Table 6: *(continued)*

	Conjunction	Drivers	Tree	S.Size	Model	sh	S.Time	S.Type	Best method(s)
560	No	11	11-B	100	McF_4	Inf	last	wholeT_0.01	J5:OT, J5:OT-A, S5:OT, S5:OT-A
561	No	11	11-B	100	McF_4	Inf	last	wholeT_0.5	J5:OT, J5:OT-A, S5:OT, S5:OT-A
562	No	11	11-B	100	McF_4	Inf	unif	singleC	J1:OT, J1:OT-A, S5:CBN-A, S5:OT, S5:OT-A
563	No	11	11-B	100	McF_4	Inf	unif	wholeT_0.01	J5:OT, J5:OT-A, S5:OT, S5:OT-A
564	No	11	11-B	100	McF_4	Inf	unif	wholeT_0.5	J5:OT, J5:OT-A, S5:OT, S5:OT-A
565	No	11	11-B	100	McF_6	0	last	singleC	J1:OT, J5:OT, S5:OT
566	No	11	11-B	100	McF_6	0	last	wholeT_0.01	J1:DiP, J5:DiP, J5:OT, S1:DiP, S5:DiP, S5:OT
567	No	11	11-B	100	McF_6	0	last	wholeT_0.5	J1:OT, J5:OT, S5:OT
568	No	11	11-B	100	McF_6	0	unif	singleC	J1:CBN, J1:CBN-A, J5:CBN, J5:CBN-A, J5:OT, J5:OT-A
569	No	11	11-B	100	McF_6	0	unif	wholeT_0.01	J1:DiP, J1:DiP-A, J5:CBN-A, J5:DiP, J5:DiP-A, J5:OT, J5:OT-A, S1:DiP, S5:DiP, S5:DiP-A, S5:OT, S5:OT-A
570	No	11	11-B	100	McF_6	0	unif	wholeT_0.5	J1:CBN, J1:CBN-A, J1:OT, J1:OT-A, J5:CBN, J5:CBN-A, J5:OT, J5:OT-A, S5:CBN, S5:CBN-A, S5:OT, S5:OT-A
571	No	11	11-B	100	McF_6	Inf	last	singleC	J5:OT, S5:OT
572	No	11	11-B	100	McF_6	Inf	last	wholeT_0.01	J5:OT, S5:OT
573	No	11	11-B	100	McF_6	Inf	last	wholeT_0.5	J1:OT, J5:OT, S5:OT
574	No	11	11-B	100	McF_6	Inf	unif	singleC	J5:CBN-A, J5:OT, J5:OT-A, S5:CBN
575	No	11	11-B	100	McF_6	Inf	unif	wholeT_0.01	J5:CBN-A, J5:OT, J5:OT-A, S5:CBN, S5:CBN-A, S5:OT, S5:OT-A
576	No	11	11-B	100	McF_6	Inf	unif	wholeT_0.5	J1:CBN-A, J5:CBN-A, J5:OT, J5:OT-A, S5:OT
577	No	9	9-B	1000	Bozic	0	last	singleC	S1:OT, S1:OT-A
578	No	9	9-B	1000	Bozic	0	last	wholeT_0.01	S1:CBN-A, S1:DiP, S1:DiP-A, S1:OT, S1:OT-A, S5:CBN-A, S5:DiP, S5:DiP-A, S5:OT, S5:OT-A
579	No	9	9-B	1000	Bozic	0	last	wholeT_0.5	S1:OT, S1:OT-A
580	No	9	9-B	1000	Bozic	0	unif	singleC	S1:CBN, S1:CBN-A, S5:CBN, S5:CBN-A
581	No	9	9-B	1000	Bozic	0	unif	wholeT_0.01	J1:DiP, J1:DiP-A, J1:OT, J1:OT-A, S1:DiP, S1:DiP-A, S1:OT, S1:OT-A
582	No	9	9-B	1000	Bozic	0	unif	wholeT_0.5	S1:CBN, S1:CBN-A, S5:CBN, S5:CBN-A
583	No	9	9-B	1000	Bozic	Inf	last	singleC	J1:DiP, J1:DiP-A, J1:OT, J1:OT-A, J5:DiP, J5:DiP-A, J5:OT, J5:OT-A, S1:DiP, S1:DiP-A, S5:DiP, S5:DiP-A, S5:OT, S5:OT-A
584	No	9	9-B	1000	Bozic	Inf	last	wholeT_0.01	J5:DiP, J5:DiP-A

Table 6: *(continued)*

	Conjunction	Drivers	Tree	S.Size	Model	sh	S.Time	S.Type	Best method(s)
585	No	9	9-B	1000	Bozic	Inf	last	wholeT_0.5	J1:DiP, J1:DiP-A, J1:OT, J1:OT-A, J5:DiP, J5:DiP-A, J5:OT, J5:OT-A, S1:DiP, S1:DiP-A, S5:DiP, S5:DiP-A, S5:OT, S5:OT-A
586	No	9	9-B	1000	Bozic	Inf	unif	singleC	S1:CBN, S1:CBN-A, S1:DiP, S1:DiP-A, S1:OT, S1:OT-A
587	No	9	9-B	1000	Bozic	Inf	unif	wholeT_0.01	J1:CBN-A, S5:CBN, S5:CBN-A, S5:DiP, S5:DiP-A, S5:OT, S5:OT-A
588	No	9	9-B	1000	Bozic	Inf	unif	wholeT_0.5	S1:CBN, S1:CBN-A, S1:DiP-A, S1:OT, S1:OT-A
589	No	9	9-B	1000	exp	0	last	singleC	J1:CBN, J1:CBN-A, S1:CBN, S1:CBN-A, S5:CBN, S5:CBN-A
590	No	9	9-B	1000	exp	0	last	wholeT_0.01	S1:DiP-A, S1:OT, S1:OT-A
591	No	9	9-B	1000	exp	0	last	wholeT_0.5	J1:CBN, J1:CBN-A, S1:CBN, S1:CBN-A, S5:CBN, S5:CBN-A
592	No	9	9-B	1000	exp	0	unif	singleC	S1:CBN, S1:CBN-A, S5:CBN, S5:CBN-A
593	No	9	9-B	1000	exp	0	unif	wholeT_0.01	J1:CBN, J1:CBN-A, S1:CBN, S1:CBN-A, S5:CBN, S5:CBN-A
594	No	9	9-B	1000	exp	0	unif	wholeT_0.5	S1:CBN, S1:CBN-A, S5:CBN, S5:CBN-A
595	No	9	9-B	1000	exp	Inf	last	singleC	J1:DiP, J1:DiP-A, J1:OT, J1:OT-A, S1:DiP-A, S5:DiP, S5:DiP-A, S5:OT, S5:OT-A
596	No	9	9-B	1000	exp	Inf	last	wholeT_0.01	J5:DiP, J5:DiP-A
597	No	9	9-B	1000	exp	Inf	last	wholeT_0.5	J1:DiP, J1:DiP-A, J1:OT, J1:OT-A, S1:DiP, S1:DiP-A, S5:DiP, S5:DiP-A, S5:OT, S5:OT-A
598	No	9	9-B	1000	exp	Inf	unif	singleC	S1:OT, S1:OT-A
599	No	9	9-B	1000	exp	Inf	unif	wholeT_0.01	J1:OT, J1:OT-A
600	No	9	9-B	1000	exp	Inf	unif	wholeT_0.5	S1:OT, S1:OT-A
601	No	9	9-B	1000	McF_4	0	last	singleC	J1:DiP, J1:DiP-A, J1:OT, J1:OT-A, J5:CBN, J5:DiP, J5:DiP-A, J5:OT, J5:OT-A, S1:DiP, S1:DiP-A, S1:OT, S1:OT-A, S5:DiP, S5:DiP-A, S5:OT, S5:OT-A
602	No	9	9-B	1000	McF_4	0	last	wholeT_0.01	J1:DiP, J1:DiP-A, J1:OT, J1:OT-A, J5:CBN, J5:DiP, J5:DiP-A, J5:OT, J5:OT-A, S1:DiP, S1:DiP-A, S5:DiP, S5:DiP-A, S5:OT, S5:OT-A

Table 6: *(continued)*

	Conjunction	Drivers	Tree	S.Size	Model	sh	S.Time	S.Type	Best method(s)
603	No	9	9-B	1000	McF_4	0	last	wholeT_0.5	J1:DiP, J1:DiP-A, J1:OT, J1:OT-A, J5:CBN, J5:DiP, J5:DiP-A, J5:OT, J5:OT-A, S1:DiP-A, S1:OT, S1:OT-A, S5:DiP, S5:DiP-A, S5:OT, S5:OT-A
604	No	9	9-B	1000	McF_4	0	unif	singleC	J1:DiP, J1:DiP-A, J1:OT, J1:OT-A, S1:DiP, S1:DiP-A, S1:OT, S1:OT-A, S5:DiP, S5:DiP-A, S5:OT, S5:OT-A
605	No	9	9-B	1000	McF_4	0	unif	wholeT_0.01	J1:DiP, J1:DiP-A, J1:OT, J1:OT-A, S1:DiP-A, S5:DiP, S5:DiP-A, S5:OT, S5:OT-A
606	No	9	9-B	1000	McF_4	0	unif	wholeT_0.5	J1:DiP, J1:DiP-A, J1:OT, J1:OT-A, S1:DiP, S1:DiP-A, S1:OT, S1:OT-A, S5:DiP, S5:DiP-A, S5:OT, S5:OT-A
607	No	9	9-B	1000	McF_4	Inf	last	singleC	J1:DiP, J1:DiP-A, J1:OT, J1:OT-A, J5:DiP, J5:DiP-A, J5:OT, J5:OT-A, S1:OT, S1:OT-A, S5:DiP, S5:DiP-A, S5:OT, S5:OT-A
608	No	9	9-B	1000	McF_4	Inf	last	wholeT_0.01	J1:DiP, J1:DiP-A, J1:OT, J1:OT-A, J5:DiP, J5:DiP-A, J5:OT, J5:OT-A, S1:DiP, S1:DiP-A, S5:DiP, S5:DiP-A, S5:OT, S5:OT-A
609	No	9	9-B	1000	McF_4	Inf	last	wholeT_0.5	J1:DiP, J1:DiP-A, J1:OT, J1:OT-A, J5:DiP, J5:DiP-A, J5:OT, J5:OT-A, S1:DiP, S1:DiP-A, S1:OT, S1:OT-A, S5:DiP, S5:DiP-A, S5:OT, S5:OT-A
610	No	9	9-B	1000	McF_4	Inf	unif	singleC	J1:DiP, J1:DiP-A, J1:OT, J1:OT-A, S1:DiP, S1:DiP-A, S1:OT, S1:OT-A, S5:DiP, S5:DiP-A, S5:OT, S5:OT-A
611	No	9	9-B	1000	McF_4	Inf	unif	wholeT_0.01	J1:DiP, J1:DiP-A, J1:OT, J1:OT-A, S1:DiP-A, S5:DiP, S5:DiP-A, S5:OT, S5:OT-A
612	No	9	9-B	1000	McF_4	Inf	unif	wholeT_0.5	J1:DiP, J1:DiP-A, J1:OT, J1:OT-A, S1:DiP, S1:DiP-A, S1:OT, S1:OT-A, S5:DiP, S5:DiP-A, S5:OT, S5:OT-A
613	No	9	9-B	1000	McF_6	0	last	singleC	J1:DiP, J1:OT, J5:DiP, J5:OT, S1:DiP, S5:DiP, S5:OT
614	No	9	9-B	1000	McF_6	0	last	wholeT_0.01	J1:DiP, J1:OT, J5:DiP, J5:OT, S1:DiP, S5:DiP, S5:OT
615	No	9	9-B	1000	McF_6	0	last	wholeT_0.5	J1:DiP, J1:OT, J5:DiP, J5:OT, S1:DiP, S1:OT, S5:DiP, S5:OT

Table 6: *(continued)*

	Conjunction	Drivers	Tree	S.Size	Model	sh	S.Time	S.Type	Best method(s)
616	No	9	9-B	1000	McF_6	0	unif	singleC	J1:CBN, J1:CBN-A, J1:DiP, J1:DiP-A, J1:OT, J1:OT-A, J5:CBN, J5:CBN-A, J5:DiP, J5:DiP-A, J5:OT, J5:OT-A, S1:DiP, S1:DiP-A, S1:OT, S1:OT-A, S5:CBN, S5:CBN-A, S5:DiP, S5:DiP-A, S5:OT, S5:OT-A
617	No	9	9-B	1000	McF_6	0	unif	wholeT_0.01	J1:CBN, J1:CBN-A, J1:DiP, J1:DiP-A, J1:OT, J1:OT-A, J5:CBN, J5:CBN-A, J5:DiP, J5:DiP-A, J5:OT, J5:OT-A, S1:DiP, S1:DiP-A, S5:CBN, S5:DiP, S5:DiP-A, S5:OT, S5:OT-A
618	No	9	9-B	1000	McF_6	0	unif	wholeT_0.5	J1:CBN, J1:CBN-A, J1:DiP, J1:DiP-A, J1:OT, J1:OT-A, J5:CBN, J5:CBN-A, J5:DiP, J5:DiP-A, J5:OT, J5:OT-A, S1:DiP, S1:DiP-A, S1:OT, S1:OT-A, S5:CBN, S5:CBN-A, S5:DiP, S5:DiP-A, S5:OT, S5:OT-A
619	No	9	9-B	1000	McF_6	Inf	last	singleC	J1:DiP, J1:OT, J5:DiP, J5:OT, S1:DiP, S5:DiP, S5:OT
620	No	9	9-B	1000	McF_6	Inf	last	wholeT_0.01	J1:DiP, J1:OT, J5:DiP, J5:OT, S1:DiP, S5:DiP, S5:OT
621	No	9	9-B	1000	McF_6	Inf	last	wholeT_0.5	J1:DiP, J1:OT, J5:DiP, J5:OT, S1:DiP, S1:OT, S5:DiP, S5:OT
622	No	9	9-B	1000	McF_6	Inf	unif	singleC	J1:CBN, J1:CBN-A, J1:DiP, J1:DiP-A, J1:OT, J1:OT-A, J5:CBN-A, J5:DiP, J5:DiP-A, J5:OT, J5:OT-A, S1:DiP, S1:DiP-A, S1:OT, S1:OT-A, S5:CBN, S5:CBN-A, S5:DiP, S5:DiP-A, S5:OT, S5:OT-A
623	No	9	9-B	1000	McF_6	Inf	unif	wholeT_0.01	J1:CBN, J1:CBN-A, J1:DiP, J1:DiP-A, J1:OT, J1:OT-A, J5:CBN-A, J5:DiP, J5:DiP-A, J5:OT, J5:OT-A, S1:DiP, S5:CBN, S5:DiP, S5:DiP-A, S5:OT, S5:OT-A
624	No	9	9-B	1000	McF_6	Inf	unif	wholeT_0.5	J1:CBN, J1:CBN-A, J1:DiP, J1:DiP-A, J1:OT, J1:OT-A, J5:CBN-A, J5:DiP, J5:DiP-A, J5:OT, J5:OT-A, S1:DiP, S1:DiP-A, S1:OT, S1:OT-A, S5:CBN, S5:CBN-A, S5:DiP, S5:DiP-A, S5:OT, S5:OT-A

Table 6: *(continued)*

	Conjunction	Drivers	Tree	S.Size	Model	sh	S.Time	S.Type	Best method(s)
625	No	9	9-B	200	Bozic	0	last	singleC	S1:OT, S1:OT-A
626	No	9	9-B	200	Bozic	0	last	wholeT_0.01	S5:OT, S5:OT-A
627	No	9	9-B	200	Bozic	0	last	wholeT_0.5	S1:OT, S1:OT-A
628	No	9	9-B	200	Bozic	0	unif	singleC	S1:CBN, S1:CBN-A
629	No	9	9-B	200	Bozic	0	unif	wholeT_0.01	S1:DiP, S1:DiP-A
630	No	9	9-B	200	Bozic	0	unif	wholeT_0.5	S1:CBN, S1:CBN-A, S5:CBN, S5:CBN-A
631	No	9	9-B	200	Bozic	Inf	last	singleC	J1:OT, J1:OT-A, S5:OT, S5:OT-A
632	No	9	9-B	200	Bozic	Inf	last	wholeT_0.01	J5:CBN-A, J5:OT, J5:OT-A
633	No	9	9-B	200	Bozic	Inf	last	wholeT_0.5	J1:OT, J1:OT-A, J5:OT, J5:OT-A, S5:OT, S5:OT-A
634	No	9	9-B	200	Bozic	Inf	unif	singleC	J1:CBN, J1:CBN-A, S1:OT, S1:OT-A
635	No	9	9-B	200	Bozic	Inf	unif	wholeT_0.01	S5:CBN-A, S5:OT, S5:OT-A
636	No	9	9-B	200	Bozic	Inf	unif	wholeT_0.5	S1:OT, S1:OT-A
637	No	9	9-B	200	exp	0	last	singleC	S1:OT, S1:OT-A
638	No	9	9-B	200	exp	0	last	wholeT_0.01	S1:OT, S1:OT-A
639	No	9	9-B	200	exp	0	last	wholeT_0.5	S1:CBN, S1:CBN-A
640	No	9	9-B	200	exp	0	unif	singleC	S1:CBN, S1:CBN-A, S5:CBN, S5:CBN-A
641	No	9	9-B	200	exp	0	unif	wholeT_0.01	S1:CBN, S1:CBN-A
642	No	9	9-B	200	exp	0	unif	wholeT_0.5	S1:CBN, S1:CBN-A, S5:CBN, S5:CBN-A
643	No	9	9-B	200	exp	Inf	last	singleC	J1:OT, J1:OT-A, S5:OT, S5:OT-A
644	No	9	9-B	200	exp	Inf	last	wholeT_0.01	J5:CBN-A, J5:OT, J5:OT-A
645	No	9	9-B	200	exp	Inf	last	wholeT_0.5	J1:OT, J1:OT-A, S5:OT, S5:OT-A
646	No	9	9-B	200	exp	Inf	unif	singleC	S1:OT, S1:OT-A
647	No	9	9-B	200	exp	Inf	unif	wholeT_0.01	J1:CBN-A, J1:OT, J1:OT-A, S5:CBN, S5:CBN-A
648	No	9	9-B	200	exp	Inf	unif	wholeT_0.5	S1:OT, S1:OT-A
649	No	9	9-B	200	McF_4	0	last	singleC	J1:OT, J1:OT-A, J5:OT, J5:OT-A, S5:DiP-A, S5:OT, S5:OT-A
650	No	9	9-B	200	McF_4	0	last	wholeT_0.01	J1:DiP, J1:DiP-A, J1:OT, J1:OT-A, J5:DiP, J5:DiP-A, J5:OT, J5:OT-A, S1:DiP, S1:DiP-A, S5:DiP, S5:DiP-A, S5:OT, S5:OT-A
651	No	9	9-B	200	McF_4	0	last	wholeT_0.5	J1:OT, J1:OT-A, J5:DiP-A, J5:OT, J5:OT-A, S1:DiP-A, S5:DiP, S5:DiP-A, S5:OT, S5:OT-A
652	No	9	9-B	200	McF_4	0	unif	singleC	J1:OT, J1:OT-A, S5:OT, S5:OT-A
653	No	9	9-B	200	McF_4	0	unif	wholeT_0.01	J1:DiP, J1:DiP-A, J1:OT, J1:OT-A, S1:DiP, S1:DiP-A, S5:DiP, S5:DiP-A, S5:OT, S5:OT-A
654	No	9	9-B	200	McF_4	0	unif	wholeT_0.5	J1:OT, J1:OT-A, S5:OT, S5:OT-A

Table 6: *(continued)*

	Conjunction	Drivers	Tree	S.Size	Model	sh	S.Time	S.Type	Best method(s)
655	No	9	9-B	200	McF_4	Inf	last	singleC	J1:OT, J1:OT-A, J5:OT, J5:OT-A, S5:OT, S5:OT-A
656	No	9	9-B	200	McF_4	Inf	last	wholeT_0.01	J1:DiP-A, J1:OT, J1:OT-A, J5:OT, J5:OT-A, S5:DiP-A, S5:OT, S5:OT-A
657	No	9	9-B	200	McF_4	Inf	last	wholeT_0.5	J1:OT, J1:OT-A, J5:OT, J5:OT-A, S1:DiP-A, S5:DiP-A, S5:OT, S5:OT-A
658	No	9	9-B	200	McF_4	Inf	unif	singleC	J1:OT, J1:OT-A, S5:OT, S5:OT-A
659	No	9	9-B	200	McF_4	Inf	unif	wholeT_0.01	J1:OT, J1:OT-A, S5:OT, S5:OT-A
660	No	9	9-B	200	McF_4	Inf	unif	wholeT_0.5	J1:OT, J1:OT-A, S5:OT, S5:OT-A
661	No	9	9-B	200	McF_6	0	last	singleC	J1:DiP, J1:OT, J5:OT, S1:DiP, S5:DiP, S5:OT
662	No	9	9-B	200	McF_6	0	last	wholeT_0.01	J1:DiP, J1:OT, J5:DiP, J5:OT, S1:DiP, S5:DiP, S5:OT
663	No	9	9-B	200	McF_6	0	last	wholeT_0.5	J1:OT, J5:OT, S1:DiP, S5:DiP, S5:OT
664	No	9	9-B	200	McF_6	0	unif	singleC	J1:CBN, J1:CBN-A, J1:DiP-A, J1:OT, J1:OT-A, J5:CBN, J5:CBN-A, J5:OT, J5:OT-A, S1:DiP, S5:CBN, S5:CBN-A, S5:DiP, S5:DiP-A, S5:OT, S5:OT-A
665	No	9	9-B	200	McF_6	0	unif	wholeT_0.01	J1:CBN, J1:CBN-A, J1:DiP, J1:DiP-A, J1:OT, J1:OT-A, J5:CBN, J5:CBN-A, J5:DiP, J5:DiP-A, J5:OT, J5:OT-A, S1:DiP, S1:DiP-A, S5:CBN-A, S5:DiP, S5:DiP-A, S5:OT, S5:OT-A
666	No	9	9-B	200	McF_6	0	unif	wholeT_0.5	J1:CBN, J1:CBN-A, J1:DiP-A, J1:OT, J1:OT-A, J5:CBN-A, J5:DiP-A, J5:OT, J5:OT-A, S1:DiP, S5:CBN-A, S5:DiP, S5:DiP-A, S5:OT, S5:OT-A
667	No	9	9-B	200	McF_6	Inf	last	singleC	J1:OT, J5:OT, S1:DiP, S5:DiP, S5:OT
668	No	9	9-B	200	McF_6	Inf	last	wholeT_0.01	J1:OT, J5:OT, S1:DiP, S5:DiP, S5:OT
669	No	9	9-B	200	McF_6	Inf	last	wholeT_0.5	J1:OT, J5:OT, S1:DiP, S5:DiP, S5:OT
670	No	9	9-B	200	McF_6	Inf	unif	singleC	J1:CBN, J1:CBN-A, J1:OT, J1:OT-A, J5:CBN-A, J5:OT, J5:OT-A, S5:CBN, S5:OT, S5:OT-A
671	No	9	9-B	200	McF_6	Inf	unif	wholeT_0.01	J1:CBN, J1:CBN-A, J1:OT, J1:OT-A, J5:CBN-A, J5:OT, J5:OT-A, S5:CBN, S5:OT, S5:OT-A
672	No	9	9-B	200	McF_6	Inf	unif	wholeT_0.5	J1:CBN, J1:CBN-A, J1:DiP-A, J1:OT, J1:OT-A, J5:CBN-A, J5:OT, J5:OT-A, S5:CBN, S5:CBN-A, S5:DiP-A, S5:OT, S5:OT-A

Table 6: *(continued)*

	Conjunction	Drivers	Tree	S.Size	Model	sh	S.Time	S.Type	Best method(s)
673	No	9	9-B	100	Bozic	0	last	singleC	S1:OT, S1:OT-A
674	No	9	9-B	100	Bozic	0	last	wholeT_0.01	S5:OT, S5:OT-A
675	No	9	9-B	100	Bozic	0	last	wholeT_0.5	S1:OT, S1:OT-A
676	No	9	9-B	100	Bozic	0	unif	singleC	S1:OT, S1:OT-A
677	No	9	9-B	100	Bozic	0	unif	wholeT_0.01	J1:OT, J1:OT-A
678	No	9	9-B	100	Bozic	0	unif	wholeT_0.5	J1:CBN, J1:CBN-A, S1:CBN, S1:CBN-A
679	No	9	9-B	100	Bozic	Inf	last	singleC	J5:OT, J5:OT-A
680	No	9	9-B	100	Bozic	Inf	last	wholeT_0.01	J5:OT, J5:OT-A
681	No	9	9-B	100	Bozic	Inf	last	wholeT_0.5	J5:OT, J5:OT-A
682	No	9	9-B	100	Bozic	Inf	unif	singleC	J1:CBN, J1:CBN-A, J1:OT, J1:OT-A, S1:OT, S1:OT-A, S5:CBN, S5:CBN-A
683	No	9	9-B	100	Bozic	Inf	unif	wholeT_0.01	J5:CBN, J5:CBN-A, S5:CBN, S5:OT, S5:OT-A
684	No	9	9-B	100	Bozic	Inf	unif	wholeT_0.5	J1:CBN, J1:CBN-A, J1:OT, J1:OT-A, S1:OT, S1:OT-A, S5:CBN-A
685	No	9	9-B	100	exp	0	last	singleC	S1:OT, S1:OT-A
686	No	9	9-B	100	exp	0	last	wholeT_0.01	S5:OT, S5:OT-A
687	No	9	9-B	100	exp	0	last	wholeT_0.5	S1:OT, S1:OT-A
688	No	9	9-B	100	exp	0	unif	singleC	J1:CBN, J1:CBN-A, S1:CBN, S1:CBN-A, S5:CBN, S5:CBN-A
689	No	9	9-B	100	exp	0	unif	wholeT_0.01	S1:OT, S1:OT-A
690	No	9	9-B	100	exp	0	unif	wholeT_0.5	S1:CBN, S1:CBN-A, S5:CBN, S5:CBN-A
691	No	9	9-B	100	exp	Inf	last	singleC	S5:OT, S5:OT-A
692	No	9	9-B	100	exp	Inf	last	wholeT_0.01	J5:CBN-A, J5:OT, J5:OT-A
693	No	9	9-B	100	exp	Inf	last	wholeT_0.5	S5:OT, S5:OT-A
694	No	9	9-B	100	exp	Inf	unif	singleC	S1:OT, S1:OT-A
695	No	9	9-B	100	exp	Inf	unif	wholeT_0.01	J1:OT, J1:OT-A, S1:OT, S1:OT-A, S5:CBN, S5:CBN-A
696	No	9	9-B	100	exp	Inf	unif	wholeT_0.5	S1:OT, S1:OT-A
697	No	9	9-B	100	McF_4	0	last	singleC	J1:OT, J1:OT-A, J5:OT, J5:OT-A, S5:OT, S5:OT-A
698	No	9	9-B	100	McF_4	0	last	wholeT_0.01	J1:DiP, J1:DiP-A, J5:OT, J5:OT-A, S1:DiP, S1:DiP-A, S5:DiP, S5:DiP-A, S5:OT, S5:OT-A
699	No	9	9-B	100	McF_4	0	last	wholeT_0.5	J1:OT, J1:OT-A, J5:OT, J5:OT-A, S5:OT, S5:OT-A
700	No	9	9-B	100	McF_4	0	unif	singleC	J1:OT, J1:OT-A, S5:OT, S5:OT-A
701	No	9	9-B	100	McF_4	0	unif	wholeT_0.01	S1:DiP, S1:DiP-A, S5:DiP-A, S5:OT, S5:OT-A
702	No	9	9-B	100	McF_4	0	unif	wholeT_0.5	J1:OT, J1:OT-A, S5:OT, S5:OT-A

Table 6: *(continued)*

	Conjunction	Drivers	Tree	S.Size	Model	sh	S.Time	S.Type	Best method(s)
703	No	9	9-B	100	McF_4	Inf	last	singleC	J1:OT, J1:OT-A, J5:OT, J5:OT-A, S5:OT, S5:OT-A
704	No	9	9-B	100	McF_4	Inf	last	wholeT_0.01	J5:OT, J5:OT-A, S5:OT, S5:OT-A
705	No	9	9-B	100	McF_4	Inf	last	wholeT_0.5	J1:OT, J1:OT-A, J5:OT, J5:OT-A, S5:OT, S5:OT-A
706	No	9	9-B	100	McF_4	Inf	unif	singleC	J1:OT, J1:OT-A, S5:OT, S5:OT-A
707	No	9	9-B	100	McF_4	Inf	unif	wholeT_0.01	S5:OT, S5:OT-A
708	No	9	9-B	100	McF_4	Inf	unif	wholeT_0.5	J1:OT, J1:OT-A, S5:OT, S5:OT-A
709	No	9	9-B	100	McF_6	0	last	singleC	J1:OT, J5:OT, S5:OT
710	No	9	9-B	100	McF_6	0	last	wholeT_0.01	J1:DiP, J5:OT, S1:DiP, S5:OT
711	No	9	9-B	100	McF_6	0	last	wholeT_0.5	J1:OT, J5:OT, S5:OT
712	No	9	9-B	100	McF_6	0	unif	singleC	J1:CBN, J1:CBN-A, J1:OT, J1:OT-A, J5:CBN-A, J5:OT, J5:OT-A, S5:CBN-A, S5:OT, S5:OT-A
713	No	9	9-B	100	McF_6	0	unif	wholeT_0.01	J1:CBN, J1:CBN-A, J1:DiP, J1:DiP-A, J1:OT, J5:CBN-A, J5:OT, J5:OT-A, S1:DiP, S1:DiP-A, S5:CBN, S5:DiP, S5:DiP-A, S5:OT, S5:OT-A
714	No	9	9-B	100	McF_6	0	unif	wholeT_0.5	J1:CBN, J1:CBN-A, J1:OT, J1:OT-A, J5:CBN-A, J5:OT, J5:OT-A, S5:CBN, S5:CBN-A, S5:OT, S5:OT-A
715	No	9	9-B	100	McF_6	Inf	last	singleC	J1:OT, J5:OT, S5:OT
716	No	9	9-B	100	McF_6	Inf	last	wholeT_0.01	J5:OT, S5:OT
717	No	9	9-B	100	McF_6	Inf	last	wholeT_0.5	J1:OT, J5:OT, S5:OT
718	No	9	9-B	100	McF_6	Inf	unif	singleC	J1:CBN, J1:CBN-A, J1:OT, J1:OT-A, J5:CBN-A, J5:OT, J5:OT-A, S5:CBN-A, S5:OT, S5:OT-A
719	No	9	9-B	100	McF_6	Inf	unif	wholeT_0.01	J5:CBN-A, J5:OT, J5:OT-A, S5:CBN, S5:CBN-A, S5:OT, S5:OT-A
720	No	9	9-B	100	McF_6	Inf	unif	wholeT_0.5	J1:CBN, J1:CBN-A, J1:OT, J5:CBN-A, J5:OT, J5:OT-A, S5:CBN, S5:CBN-A, S5:OT, S5:OT-A
721	No	7	7-B	1000	Bozic	0	last	singleC	J1:CBN-A, J1:OT-A, J5:CBN-A, J5:OT-A, S1:CBN, S1:OT-A, S5:OT-A
722	No	7	7-B	1000	Bozic	0	last	wholeT_0.01	J1:DiP-A, J1:OT-A, J5:DiP-A, J5:OT-A, S1:DiP-A, S1:OT-A, S5:DiP-A, S5:OT-A
723	No	7	7-B	1000	Bozic	0	last	wholeT_0.5	J1:CBN-A, J1:OT-A, J5:CBN-A, J5:OT-A, S1:OT-A, S5:CBN, S5:CBN-A, S5:OT-A

Table 6: *(continued)*

	Conjunction	Drivers	Tree	S.Size	Model	sh	S.Time	S.Type	Best method(s)
724	No	7	7-B	1000	Bozic	0	unif	singleC	J1:CBN, J1:CBN-A, J1:OT, J1:OT-A, S1:CBN, S1:CBN-A, S1:OT, S1:OT-A
725	No	7	7-B	1000	Bozic	0	unif	wholeT_0.01	J5:CBN, J5:CBN-A, J5:DiP-A, J5:OT, J5:OT-A
726	No	7	7-B	1000	Bozic	0	unif	wholeT_0.5	NA
727	No	7	7-B	1000	Bozic	Inf	last	singleC	J1:DiP-A, J1:OT-A, J5:DiP-A, J5:OT-A, S1:DiP-A, S5:DiP-A, S5:OT-A
728	No	7	7-B	1000	Bozic	Inf	last	wholeT_0.01	J5:CBN, J5:CBN-A, J5:DiP, J5:DiP-A, J5:OT, J5:OT-A
729	No	7	7-B	1000	Bozic	Inf	last	wholeT_0.5	J1:DiP-A, J1:OT-A, J5:CBN, J5:DiP-A, J5:OT-A, S5:DiP-A, S5:OT-A
730	No	7	7-B	1000	Bozic	Inf	unif	singleC	J1:CBN, J1:CBN-A, J5:CBN, J5:CBN-A, J5:OT, J5:OT-A, S1:CBN, S1:CBN-A, S5:CBN, S5:CBN-A, S5:OT, S5:OT-A
731	No	7	7-B	1000	Bozic	Inf	unif	wholeT_0.01	J1:CBN-A, J1:OT, J1:OT-A, J5:CBN, J5:CBN-A, S5:CBN, S5:CBN-A
732	No	7	7-B	1000	Bozic	Inf	unif	wholeT_0.5	J1:CBN, J1:CBN-A, J1:OT, J1:OT-A, S1:CBN, S1:CBN-A
733	No	7	7-B	1000	exp	0	last	singleC	J1:CBN-A, J1:OT-A, S1:OT-A
734	No	7	7-B	1000	exp	0	last	wholeT_0.01	J1:CBN, J1:CBN-A, J1:OT-A, J5:CBN-A, J5:OT-A, S1:CBN, S1:CBN-A, S1:OT-A, S5:CBN, S5:CBN-A, S5:OT-A
735	No	7	7-B	1000	exp	0	last	wholeT_0.5	J1:CBN-A, J1:OT-A, S1:OT-A
736	No	7	7-B	1000	exp	0	unif	singleC	NA
737	No	7	7-B	1000	exp	0	unif	wholeT_0.01	J1:CBN, J1:CBN-A, J1:OT-A
738	No	7	7-B	1000	exp	0	unif	wholeT_0.5	NA
739	No	7	7-B	1000	exp	Inf	last	singleC	J1:OT-A, J5:CBN-A, J5:OT-A, S1:OT-A, S5:OT-A
740	No	7	7-B	1000	exp	Inf	last	wholeT_0.01	J1:CBN, J1:CBN-A, J1:DiP-A, J1:OT, J1:OT-A, J5:CBN-A, J5:DiP-A, J5:OT-A, S5:CBN, S5:CBN-A, S5:OT, S5:OT-A
741	No	7	7-B	1000	exp	Inf	last	wholeT_0.5	J1:OT-A, J5:CBN-A, J5:OT-A, S1:OT-A, S5:OT-A
742	No	7	7-B	1000	exp	Inf	unif	singleC	J1:CBN, J1:CBN-A, J1:OT, J1:OT-A, S1:CBN, S1:CBN-A
743	No	7	7-B	1000	exp	Inf	unif	wholeT_0.01	J1:CBN, J1:CBN-A, J1:OT, J1:OT-A, J5:CBN, J5:CBN-A, J5:OT, J5:OT-A, S5:CBN, S5:CBN-A, S5:OT, S5:OT-A
744	No	7	7-B	1000	exp	Inf	unif	wholeT_0.5	J1:CBN, J1:CBN-A, J1:OT, J1:OT-A, S1:CBN, S1:CBN-A

Table 6: *(continued)*

	Conjunction	Drivers	Tree	S.Size	Model	sh	S.Time	S.Type	Best method(s)
745	No	7	7-B	1000	McF_4	0	last	singleC	J1:DiP-A, J1:OT-A, J5:DiP-A, J5:OT-A, S1:DiP, S1:DiP-A, S1:OT, S1:OT-A, S5:DiP, S5:DiP-A, S5:OT, S5:OT-A
746	No	7	7-B	1000	McF_4	0	last	wholeT_0.01	J1:CBN-A, J1:DiP, J1:DiP-A, J1:OT, J1:OT-A, J5:DiP-A, J5:OT-A, S1:DiP, S1:DiP-A, S5:DiP, S5:DiP-A, S5:OT, S5:OT-A
747	No	7	7-B	1000	McF_4	0	last	wholeT_0.5	J1:DiP-A, J1:OT-A, J5:DiP-A, J5:OT-A, S1:DiP, S1:DiP-A, S1:OT, S1:OT-A, S5:DiP, S5:DiP-A, S5:OT, S5:OT-A
748	No	7	7-B	1000	McF_4	0	unif	singleC	J1:CBN-A, J1:DiP, J1:DiP-A, J1:OT, J1:OT-A, J5:CBN, J5:CBN-A, J5:DiP, J5:DiP-A, J5:OT, J5:OT-A, S1:DiP, S1:DiP-A, S1:OT, S1:OT-A, S5:CBN-A, S5:DiP, S5:DiP-A, S5:OT, S5:OT-A
749	No	7	7-B	1000	McF_4	0	unif	wholeT_0.01	J1:CBN, J1:DiP, J1:DiP-A, J1:OT, J1:OT-A, J5:CBN, J5:DiP, J5:DiP-A, J5:OT, J5:OT-A, S1:DiP, S1:DiP-A, S5:CBN, S5:CBN-A, S5:DiP, S5:DiP-A, S5:OT, S5:OT-A
750	No	7	7-B	1000	McF_4	0	unif	wholeT_0.5	J1:CBN-A, J1:DiP, J1:DiP-A, J1:OT, J1:OT-A, J5:CBN, J5:CBN-A, J5:DiP, J5:DiP-A, J5:OT, J5:OT-A, S1:DiP, S1:DiP-A, S1:OT, S1:OT-A, S5:CBN-A, S5:DiP, S5:DiP-A, S5:OT, S5:OT-A
751	No	7	7-B	1000	McF_4	Inf	last	singleC	J1:DiP-A, J1:OT-A, J5:DiP-A, J5:OT-A, S1:DiP, S1:DiP-A, S1:OT, S1:OT-A, S5:DiP, S5:DiP-A, S5:OT, S5:OT-A
752	No	7	7-B	1000	McF_4	Inf	last	wholeT_0.01	J1:DiP, J1:DiP-A, J1:OT, J1:OT-A, J5:DiP-A, J5:OT-A, S1:DiP, S1:DiP-A, S5:DiP, S5:DiP-A, S5:OT, S5:OT-A
753	No	7	7-B	1000	McF_4	Inf	last	wholeT_0.5	J1:DiP-A, J1:OT-A, J5:DiP-A, J5:OT-A, S1:DiP, S1:DiP-A, S1:OT, S1:OT-A, S5:DiP, S5:DiP-A, S5:OT, S5:OT-A
754	No	7	7-B	1000	McF_4	Inf	unif	singleC	J1:CBN-A, J1:DiP-A, J1:OT, J1:OT-A, J5:CBN-A, J5:DiP-A, J5:OT, J5:OT-A, S1:DiP, S1:DiP-A, S1:OT, S1:OT-A, S5:CBN-A, S5:DiP-A, S5:OT, S5:OT-A

Table 6: *(continued)*

	Conjunction	Drivers	Tree	S.Size	Model	sh	S.Time	S.Type	Best method(s)
755	No	7	7-B	1000	McF_4	Inf	unif	wholeT_0.01	J1:DiP-A, J1:OT, J1:OT-A, J5:CBN-A, J5:DiP-A, J5:OT, J5:OT-A, S1:DiP, S1:DiP-A, S5:DiP-A, S5:OT, S5:OT-A
756	No	7	7-B	1000	McF_4	Inf	unif	wholeT_0.5	J1:DiP-A, J1:OT, J1:OT-A, J5:CBN-A, J5:DiP-A, J5:OT, J5:OT-A, S1:DiP, S1:DiP-A, S1:OT, S1:OT-A, S5:DiP-A, S5:OT, S5:OT-A
757	No	7	7-B	1000	McF_6	0	last	singleC	J1:DiP, J1:DiP-A, J1:OT, J1:OT-A, J5:DiP, J5:DiP-A, J5:OT, J5:OT-A, S1:DiP, S1:DiP-A, S5:DiP, S5:DiP-A, S5:OT, S5:OT-A
758	No	7	7-B	1000	McF_6	0	last	wholeT_0.01	J1:DiP, J1:DiP-A, J5:DiP, J5:DiP-A, J5:OT, J5:OT-A, S1:DiP, S1:DiP-A, S5:DiP, S5:DiP-A, S5:OT, S5:OT-A
759	No	7	7-B	1000	McF_6	0	last	wholeT_0.5	J1:DiP, J1:DiP-A, J1:OT, J1:OT-A, J5:DiP, J5:DiP-A, J5:OT, J5:OT-A, S1:DiP, S1:DiP-A, S1:OT, S1:OT-A, S5:DiP, S5:DiP-A, S5:OT, S5:OT-A
760	No	7	7-B	1000	McF_6	0	unif	singleC	none
761	No	7	7-B	1000	McF_6	0	unif	wholeT_0.01	J1:CBN, J1:CBN-A, J1:DiP, J1:DiP-A, J1:OT, J1:OT-A, J5:CBN, J5:CBN-A, J5:DiP, J5:DiP-A, J5:OT, J5:OT-A, S1:DiP, S5:CBN, S5:CBN-A, S5:DiP, S5:DiP-A, S5:OT, S5:OT-A
762	No	7	7-B	1000	McF_6	0	unif	wholeT_0.5	none
763	No	7	7-B	1000	McF_6	Inf	last	singleC	J1:DiP, J1:DiP-A, J1:OT, J1:OT-A, J5:DiP, J5:DiP-A, J5:OT, J5:OT-A, S1:DiP, S1:DiP-A, S5:DiP, S5:DiP-A, S5:OT, S5:OT-A
764	No	7	7-B	1000	McF_6	Inf	last	wholeT_0.01	J1:DiP-A, J5:DiP-A, J5:OT, J5:OT-A, S1:DiP-A, S5:DiP-A, S5:OT, S5:OT-A
765	No	7	7-B	1000	McF_6	Inf	last	wholeT_0.5	J1:DiP, J1:DiP-A, J1:OT, J1:OT-A, J5:DiP, J5:DiP-A, J5:OT, J5:OT-A, S1:DiP, S1:DiP-A, S5:DiP, S5:DiP-A, S5:OT, S5:OT-A

Table 6: *(continued)*

	Conjunction	Drivers	Tree	S.Size	Model	sh	S.Time	S.Type	Best method(s)
766	No	7	7-B	1000	McF_6	Inf	unif	singleC	J1:CBN, J1:CBN-A, J1:DiP, J1:DiP-A, J1:OT, J1:OT-A, J5:CBN-A, J5:DiP-A, J5:OT, J5:OT-A, S1:CBN, S1:CBN-A, S1:DiP, S1:DiP-A, S1:OT, S1:OT-A, S5:CBN, S5:CBN-A, S5:DiP, S5:DiP-A, S5:OT, S5:OT-A
767	No	7	7-B	1000	McF_6	Inf	unif	wholeT_0.01	J1:CBN, J1:CBN-A, J1:DiP, J1:DiP-A, J1:OT, J1:OT-A, J5:CBN, J5:CBN-A, J5:DiP, J5:DiP-A, J5:OT, J5:OT-A, S5:CBN, S5:CBN-A, S5:DiP, S5:DiP-A, S5:OT, S5:OT-A
768	No	7	7-B	1000	McF_6	Inf	unif	wholeT_0.5	none
769	No	7	7-B	200	Bozic	0	last	singleC	J1:CBN-A, J1:OT-A, J5:CBN-A, J5:OT-A, S5:CBN, S5:OT-A
770	No	7	7-B	200	Bozic	0	last	wholeT_0.01	J1:OT-A, J5:OT-A, S5:OT-A
771	No	7	7-B	200	Bozic	0	last	wholeT_0.5	J1:CBN-A, J1:OT-A, J5:CBN-A, J5:OT-A, S5:CBN, S5:CBN-A, S5:OT-A
772	No	7	7-B	200	Bozic	0	unif	singleC	J1:CBN, J1:CBN-A, J1:OT, J1:OT-A, S1:OT, S1:OT-A
773	No	7	7-B	200	Bozic	0	unif	wholeT_0.01	J5:CBN, J5:CBN-A, J5:OT, J5:OT-A
774	No	7	7-B	200	Bozic	0	unif	wholeT_0.5	J1:OT, J1:OT-A, S1:CBN, S1:CBN-A, S1:OT, S1:OT-A
775	No	7	7-B	200	Bozic	Inf	last	singleC	J5:CBN, J5:OT-A, S5:OT-A
776	No	7	7-B	200	Bozic	Inf	last	wholeT_0.01	J5:CBN, J5:CBN-A, J5:OT, J5:OT-A
777	No	7	7-B	200	Bozic	Inf	last	wholeT_0.5	J5:CBN, J5:OT-A, S5:OT-A
778	No	7	7-B	200	Bozic	Inf	unif	singleC	J1:CBN, J1:CBN-A, J5:CBN, J5:CBN-A, J5:OT, J5:OT-A, S5:CBN, S5:CBN-A, S5:OT, S5:OT-A
779	No	7	7-B	200	Bozic	Inf	unif	wholeT_0.01	J5:CBN, J5:CBN-A, J5:OT, J5:OT-A, S5:CBN, S5:CBN-A, S5:OT, S5:OT-A
780	No	7	7-B	200	Bozic	Inf	unif	wholeT_0.5	J1:CBN, J1:CBN-A
781	No	7	7-B	200	exp	0	last	singleC	J1:OT-A, S1:OT-A
782	No	7	7-B	200	exp	0	last	wholeT_0.01	J1:CBN, J1:CBN-A, J1:OT-A, J5:CBN-A, J5:OT-A, S1:CBN, S1:CBN-A, S5:CBN, S5:CBN-A
783	No	7	7-B	200	exp	0	last	wholeT_0.5	J1:CBN-A, J1:OT-A, S1:OT-A
784	No	7	7-B	200	exp	0	unif	singleC	NA
785	No	7	7-B	200	exp	0	unif	wholeT_0.01	J1:CBN-A, J1:OT-A
786	No	7	7-B	200	exp	0	unif	wholeT_0.5	NA
787	No	7	7-B	200	exp	Inf	last	singleC	J1:OT-A, J5:CBN-A, J5:OT-A, S5:OT-A

Table 6: *(continued)*

	Conjunction	Drivers	Tree	S.Size	Model	sh	S.Time	S.Type	Best method(s)
788	No	7	7-B	200	exp	Inf	last	wholeT_0.01	J5:CBN, J5:CBN-A, J5:OT-A
789	No	7	7-B	200	exp	Inf	last	wholeT_0.5	J1:CBN, J1:OT-A, J5:CBN-A, J5:OT-A, S5:OT-A
790	No	7	7-B	200	exp	Inf	unif	singleC	J1:CBN, J1:CBN-A, J1:OT, J1:OT-A
791	No	7	7-B	200	exp	Inf	unif	wholeT_0.01	J5:CBN, J5:CBN-A, J5:OT, J5:OT-A, S5:CBN, S5:CBN-A, S5:OT, S5:OT-A
792	No	7	7-B	200	exp	Inf	unif	wholeT_0.5	J1:CBN, J1:CBN-A, J1:OT, J1:OT-A, S1:CBN, S1:CBN-A
793	No	7	7-B	200	McF_4	0	last	singleC	J1:OT-A, J5:OT-A, S5:OT, S5:OT-A
794	No	7	7-B	200	McF_4	0	last	wholeT_0.01	J1:DiP, J1:DiP-A, J1:OT, J1:OT-A, J5:OT-A, S1:DiP, S1:DiP-A, S5:DiP, S5:DiP-A, S5:OT, S5:OT-A
795	No	7	7-B	200	McF_4	0	last	wholeT_0.5	J1:OT-A, J5:OT-A, S5:OT, S5:OT-A
796	No	7	7-B	200	McF_4	0	unif	singleC	J1:CBN, J1:CBN-A, J1:DiP-A, J1:OT, J1:OT-A, J5:CBN, J5:CBN-A, J5:OT, J5:OT-A, S1:DiP-A, S5:CBN, S5:CBN-A, S5:DiP-A, S5:OT, S5:OT-A
797	No	7	7-B	200	McF_4	0	unif	wholeT_0.01	J1:CBN, J1:CBN-A, J1:DiP-A, J1:OT, J1:OT-A, J5:CBN, J5:CBN-A, J5:DiP-A, J5:OT, J5:OT-A, S5:CBN, S5:CBN-A, S5:DiP-A, S5:OT, S5:OT-A
798	No	7	7-B	200	McF_4	0	unif	wholeT_0.5	J1:CBN, J1:CBN-A, J1:OT, J1:OT-A, J5:CBN, J5:CBN-A, J5:OT, J5:OT-A, S5:CBN, S5:CBN-A, S5:OT, S5:OT-A
799	No	7	7-B	200	McF_4	Inf	last	singleC	J1:OT-A, J5:OT-A, S5:OT, S5:OT-A
800	No	7	7-B	200	McF_4	Inf	last	wholeT_0.01	J1:OT, J1:OT-A, J5:OT-A, S5:OT, S5:OT-A
801	No	7	7-B	200	McF_4	Inf	last	wholeT_0.5	J1:OT-A, J5:OT-A, S5:OT, S5:OT-A
802	No	7	7-B	200	McF_4	Inf	unif	singleC	J1:OT, J1:OT-A, J5:CBN-A, J5:OT, J5:OT-A, S5:OT, S5:OT-A
803	No	7	7-B	200	McF_4	Inf	unif	wholeT_0.01	J1:OT, J1:OT-A, J5:CBN-A, J5:OT, J5:OT-A, S5:OT, S5:OT-A
804	No	7	7-B	200	McF_4	Inf	unif	wholeT_0.5	J1:CBN-A, J1:OT, J1:OT-A, J5:CBN-A, J5:OT, J5:OT-A, S5:CBN-A, S5:OT, S5:OT-A
805	No	7	7-B	200	McF_6	0	last	singleC	J1:DiP-A, J1:OT, J1:OT-A, J5:DiP-A, J5:OT, J5:OT-A, S1:DiP-A, S5:DiP-A, S5:OT, S5:OT-A

Table 6: *(continued)*

	Conjunction	Drivers	Tree	S.Size	Model	sh	S.Time	S.Type	Best method(s)
806	No	7	7-B	200	McF_6	0	last	wholeT_0.01	J1:DiP, J1:DiP-A, J5:DiP, J5:DiP-A, J5:OT, J5:OT-A, S1:DiP, S1:DiP-A, S5:DiP, S5:DiP-A, S5:OT, S5:OT-A
807	No	7	7-B	200	McF_6	0	last	wholeT_0.5	J1:DiP-A, J1:OT, J1:OT-A, J5:DiP-A, J5:OT, J5:OT-A, S1:DiP-A, S5:DiP-A, S5:OT, S5:OT-A
808	No	7	7-B	200	McF_6	0	unif	singleC	J1:CBN, J1:CBN-A, J1:DiP, J1:DiP-A, J1:OT, J1:OT-A, J5:CBN, J5:CBN-A, J5:DiP-A, J5:OT, J5:OT-A, S1:DiP, S1:DiP-A, S5:CBN, S5:CBN-A, S5:DiP, S5:DiP-A, S5:OT, S5:OT-A
809	No	7	7-B	200	McF_6	0	unif	wholeT_0.01	J1:CBN, J1:CBN-A, J1:DiP, J1:DiP-A, J1:OT, J1:OT-A, J5:CBN, J5:CBN-A, J5:DiP-A, J5:OT, J5:OT-A, S1:DiP, S1:DiP-A, S5:CBN, S5:CBN-A, S5:DiP, S5:DiP-A, S5:OT, S5:OT-A
810	No	7	7-B	200	McF_6	0	unif	wholeT_0.5	J1:CBN, J1:CBN-A, J1:DiP, J1:DiP-A, J1:OT, J1:OT-A, J5:CBN, J5:CBN-A, J5:DiP-A, J5:OT, J5:OT-A, S1:DiP, S5:CBN, S5:CBN-A, S5:DiP, S5:OT, S5:OT-A
811	No	7	7-B	200	McF_6	Inf	last	singleC	J1:DiP-A, J1:OT, J1:OT-A, J5:DiP-A, J5:OT, J5:OT-A, S1:DiP-A, S5:DiP-A, S5:OT, S5:OT-A
812	No	7	7-B	200	McF_6	Inf	last	wholeT_0.01	J5:OT, J5:OT-A, S5:OT, S5:OT-A
813	No	7	7-B	200	McF_6	Inf	last	wholeT_0.5	J1:DiP-A, J1:OT, J1:OT-A, J5:DiP-A, J5:OT, J5:OT-A, S1:DiP-A, S5:DiP-A, S5:OT, S5:OT-A
814	No	7	7-B	200	McF_6	Inf	unif	singleC	J1:CBN, J1:CBN-A, J1:DiP-A, J1:OT, J1:OT-A, J5:CBN, J5:CBN-A, J5:OT, J5:OT-A, S5:CBN, S5:CBN-A, S5:OT, S5:OT-A
815	No	7	7-B	200	McF_6	Inf	unif	wholeT_0.01	J1:CBN, J1:CBN-A, J1:DiP-A, J5:CBN, J5:CBN-A, S1:DiP, S5:CBN, S5:CBN-A, S5:DiP-A
816	No	7	7-B	200	McF_6	Inf	unif	wholeT_0.5	J1:CBN, J1:CBN-A, J1:DiP-A, J5:CBN, J5:CBN-A, S1:DiP, S5:CBN, S5:CBN-A, S5:DiP, S5:DiP-A
817	No	7	7-B	100	Bozic	0	last	singleC	J5:CBN-A, J5:OT-A, S5:OT-A
818	No	7	7-B	100	Bozic	0	last	wholeT_0.01	J5:OT-A, S5:CBN, S5:OT-A

Table 6: *(continued)*

	Conjunction	Drivers	Tree	S.Size	Model	sh	S.Time	S.Type	Best method(s)
819	No	7	7-B	100	Bozic	0	last	wholeT_0.5	J5:OT-A, S5:CBN, S5:CBN-A, S5:OT-A
820	No	7	7-B	100	Bozic	0	unif	singleC	J1:CBN, J1:CBN-A, J1:OT, J1:OT-A
821	No	7	7-B	100	Bozic	0	unif	wholeT_0.01	J5:CBN, J5:CBN-A, J5:OT, J5:OT-A
822	No	7	7-B	100	Bozic	0	unif	wholeT_0.5	J1:CBN, J1:CBN-A, J1:OT, J1:OT-A, S1:OT, S1:OT-A
823	No	7	7-B	100	Bozic	Inf	last	singleC	J5:CBN, J5:OT-A
824	No	7	7-B	100	Bozic	Inf	last	wholeT_0.01	J5:CBN, J5:CBN-A, J5:OT-A
825	No	7	7-B	100	Bozic	Inf	last	wholeT_0.5	J5:CBN, J5:OT-A, S5:OT-A
826	No	7	7-B	100	Bozic	Inf	unif	singleC	J5:CBN, J5:CBN-A, J5:OT, J5:OT-A, S5:CBN, S5:CBN-A
827	No	7	7-B	100	Bozic	Inf	unif	wholeT_0.01	J5:CBN, J5:CBN-A, J5:OT, J5:OT-A, S5:CBN-A
828	No	7	7-B	100	Bozic	Inf	unif	wholeT_0.5	J5:CBN, J5:CBN-A, J5:OT, J5:OT-A, S5:CBN, S5:CBN-A, S5:OT, S5:OT-A
829	No	7	7-B	100	exp	0	last	singleC	J1:OT-A, S1:OT-A
830	No	7	7-B	100	exp	0	last	wholeT_0.01	J1:CBN, J1:CBN-A, J5:CBN-A, J5:OT-A, S5:CBN, S5:CBN-A
831	No	7	7-B	100	exp	0	last	wholeT_0.5	J1:OT-A, S1:OT-A
832	No	7	7-B	100	exp	0	unif	singleC	J1:OT, J1:OT-A, S1:CBN, S1:CBN-A, S1:OT, S1:OT-A
833	No	7	7-B	100	exp	0	unif	wholeT_0.01	J1:CBN, J1:CBN-A, J1:OT, J1:OT-A, S1:OT, S1:OT-A
834	No	7	7-B	100	exp	0	unif	wholeT_0.5	NA
835	No	7	7-B	100	exp	Inf	last	singleC	J1:OT-A, J5:CBN-A, J5:OT-A, S5:OT-A
836	No	7	7-B	100	exp	Inf	last	wholeT_0.01	J5:CBN, J5:CBN-A, J5:OT-A
837	No	7	7-B	100	exp	Inf	last	wholeT_0.5	J1:OT-A, J5:CBN-A, J5:OT-A, S5:OT-A
838	No	7	7-B	100	exp	Inf	unif	singleC	J1:CBN, J1:CBN-A, J1:OT, J1:OT-A
839	No	7	7-B	100	exp	Inf	unif	wholeT_0.01	J5:CBN, J5:CBN-A, J5:OT, J5:OT-A, S5:CBN, S5:OT, S5:OT-A
840	No	7	7-B	100	exp	Inf	unif	wholeT_0.5	J1:CBN, J1:CBN-A, J1:OT, J1:OT-A, S1:OT, S1:OT-A
841	No	7	7-B	100	McF_4	0	last	singleC	J1:OT-A, J5:OT-A, S5:OT, S5:OT-A
842	No	7	7-B	100	McF_4	0	last	wholeT_0.01	J5:OT-A, S1:DiP, S1:DiP-A, S5:DiP-A, S5:OT, S5:OT-A
843	No	7	7-B	100	McF_4	0	last	wholeT_0.5	J5:OT-A, S5:OT, S5:OT-A
844	No	7	7-B	100	McF_4	0	unif	singleC	J1:CBN, J1:CBN-A, J1:OT, J1:OT-A, J5:CBN, J5:CBN-A, J5:OT, J5:OT-A, S5:CBN, S5:CBN-A, S5:OT, S5:OT-A

Table 6: *(continued)*

	Conjunction	Drivers	Tree	S.Size	Model	sh	S.Time	S.Type	Best method(s)
845	No	7	7-B	100	McF_4	0	unif	wholeT_0.01	J1:CBN-A, J1:OT, J1:OT-A, J5:CBN, J5:CBN-A, J5:OT, J5:OT-A, S1:DiP-A, S5:CBN, S5:CBN-A, S5:OT, S5:OT-A
846	No	7	7-B	100	McF_4	0	unif	wholeT_0.5	J1:CBN, J1:CBN-A, J1:OT, J1:OT-A, J5:CBN, J5:CBN-A, J5:OT, J5:OT-A, S5:CBN-A, S5:OT, S5:OT-A
847	No	7	7-B	100	McF_4	Inf	last	singleC	J1:OT-A, J5:OT-A, S5:OT, S5:OT-A
848	No	7	7-B	100	McF_4	Inf	last	wholeT_0.01	J5:OT-A, S5:OT, S5:OT-A
849	No	7	7-B	100	McF_4	Inf	last	wholeT_0.5	J1:OT-A, J5:OT-A, S5:OT, S5:OT-A
850	No	7	7-B	100	McF_4	Inf	unif	singleC	J1:CBN-A, J1:OT, J1:OT-A, J5:CBN-A, J5:OT, J5:OT-A, S5:OT, S5:OT-A
851	No	7	7-B	100	McF_4	Inf	unif	wholeT_0.01	J5:CBN-A, J5:OT, J5:OT-A, S5:CBN-A, S5:OT, S5:OT-A
852	No	7	7-B	100	McF_4	Inf	unif	wholeT_0.5	J1:CBN-A, J5:CBN-A, S5:CBN-A
853	No	7	7-B	100	McF_6	0	last	singleC	J5:OT, J5:OT-A, S5:OT, S5:OT-A
854	No	7	7-B	100	McF_6	0	last	wholeT_0.01	J5:OT, J5:OT-A, S5:OT, S5:OT-A
855	No	7	7-B	100	McF_6	0	last	wholeT_0.5	J5:OT, J5:OT-A, S5:OT, S5:OT-A
856	No	7	7-B	100	McF_6	0	unif	singleC	J1:CBN, J1:CBN-A, J5:CBN, J5:CBN-A, S5:CBN, S5:CBN-A
857	No	7	7-B	100	McF_6	0	unif	wholeT_0.01	J1:DiP-A, J5:CBN, J5:CBN-A, J5:DiP-A, S5:CBN, S5:CBN-A, S5:DiP, S5:DiP-A
858	No	7	7-B	100	McF_6	0	unif	wholeT_0.5	J1:CBN, J1:CBN-A, J5:CBN, J5:CBN-A, S5:CBN, S5:CBN-A
859	No	7	7-B	100	McF_6	Inf	last	singleC	J5:OT, J5:OT-A, S5:OT, S5:OT-A
860	No	7	7-B	100	McF_6	Inf	last	wholeT_0.01	J5:OT, J5:OT-A, S5:OT, S5:OT-A
861	No	7	7-B	100	McF_6	Inf	last	wholeT_0.5	J1:OT, J1:OT-A, J5:OT, J5:OT-A, S5:OT, S5:OT-A
862	No	7	7-B	100	McF_6	Inf	unif	singleC	J1:CBN, J1:CBN-A, J5:CBN, J5:CBN-A, S5:CBN, S5:CBN-A
863	No	7	7-B	100	McF_6	Inf	unif	wholeT_0.01	J5:CBN, J5:CBN-A, S5:CBN, S5:CBN-A
864	No	7	7-B	100	McF_6	Inf	unif	wholeT_0.5	J1:CBN, J1:CBN-A, J5:CBN, J5:CBN-A, S5:CBN, S5:CBN-A

3.3 Confidence sets (MCB-2), PND, Drivers Unknown

Table 7: Confidence sets (method MCB-2) when Drivers are Unknown for measure PND.

	Conjunction	Drivers	Tree	S.Size	Model	sh	S.Time	S.Type	Best method(s)
1	Yes	11	11-A	1000	Bozic	0	last	singleC	S1:OT, S1:OT-A
2	Yes	11	11-A	1000	Bozic	0	last	wholeT_0.01	S1:DiP, S1:DiP-A, S1:OT, S1:OT-A, S5:DiP, S5:DiP-A, S5:OT, S5:OT-A
3	Yes	11	11-A	1000	Bozic	0	last	wholeT_0.5	S1:OT, S1:OT-A
4	Yes	11	11-A	1000	Bozic	0	unif	singleC	S1:CBN, S1:CBN-A, S1:DiP, S1:DiP-A, S1:OT, S1:OT-A
5	Yes	11	11-A	1000	Bozic	0	unif	wholeT_0.01	S1:CBN, S1:CBN-A
6	Yes	11	11-A	1000	Bozic	0	unif	wholeT_0.5	none
7	Yes	11	11-A	1000	Bozic	Inf	last	singleC	S1:DiP, S1:DiP-A, S1:OT, S1:OT-A, S5:DiP, S5:DiP-A, S5:OT, S5:OT-A
8	Yes	11	11-A	1000	Bozic	Inf	last	wholeT_0.01	J1:DiP, J1:DiP-A, J1:OT, J1:OT-A, S1:DiP, S1:DiP-A, S1:OT, S1:OT-A, S5:DiP, S5:DiP-A, S5:OT, S5:OT-A
9	Yes	11	11-A	1000	Bozic	Inf	last	wholeT_0.5	S1:CBN, S1:OT, S1:OT-A
10	Yes	11	11-A	1000	Bozic	Inf	unif	singleC	S1:CBN-A, S1:DiP-A, S1:OT, S1:OT-A
11	Yes	11	11-A	1000	Bozic	Inf	unif	wholeT_0.01	S1:CBN, S1:CBN-A, S1:DiP, S1:DiP-A, S1:OT, S1:OT-A, S5:CBN, S5:CBN-A, S5:DiP, S5:DiP-A, S5:OT, S5:OT-A
12	Yes	11	11-A	1000	Bozic	Inf	unif	wholeT_0.5	S1:CBN, S1:CBN-A, S1:OT, S1:OT-A
13	Yes	11	11-A	1000	exp	0	last	singleC	S1:OT, S1:OT-A
14	Yes	11	11-A	1000	exp	0	last	wholeT_0.01	S1:DiP-A, S1:OT, S1:OT-A, S5:DiP-A, S5:OT, S5:OT-A
15	Yes	11	11-A	1000	exp	0	last	wholeT_0.5	S1:OT, S1:OT-A
16	Yes	11	11-A	1000	exp	0	unif	singleC	none
17	Yes	11	11-A	1000	exp	0	unif	wholeT_0.01	S1:CBN, S1:CBN-A, S1:OT, S1:OT-A
18	Yes	11	11-A	1000	exp	0	unif	wholeT_0.5	none
19	Yes	11	11-A	1000	exp	Inf	last	singleC	S1:OT, S1:OT-A
20	Yes	11	11-A	1000	exp	Inf	last	wholeT_0.01	J1:CBN-A, J1:DiP, J1:DiP-A, J1:OT, J1:OT-A, S1:DiP, S1:DiP-A, S1:OT, S1:OT-A, S5:CBN-A, S5:DiP, S5:DiP-A, S5:OT, S5:OT-A
21	Yes	11	11-A	1000	exp	Inf	last	wholeT_0.5	S1:CBN, S1:DiP, S1:DiP-A, S1:OT, S1:OT-A, S5:DiP, S5:DiP-A, S5:OT, S5:OT-A
22	Yes	11	11-A	1000	exp	Inf	unif	singleC	S1:CBN-A, S1:OT, S1:OT-A
23	Yes	11	11-A	1000	exp	Inf	unif	wholeT_0.01	S1:CBN, S1:CBN-A, S1:DiP-A, S1:OT, S1:OT-A

Table 7: *(continued)*

	Conjunction	Drivers	Tree	S.Size	Model	sh	S.Time	S.Type	Best method(s)
24	Yes	11	11-A	1000	exp	Inf	unif	wholeT_0.5	S1:OT, S1:OT-A
25	Yes	11	11-A	1000	McF_4	0	last	singleC	S1:DiP-A, S1:OT, S1:OT-A
26	Yes	11	11-A	1000	McF_4	0	last	wholeT_0.01	S1:DiP-A, S1:OT, S1:OT-A
27	Yes	11	11-A	1000	McF_4	0	last	wholeT_0.5	S1:DiP-A, S1:OT, S1:OT-A
28	Yes	11	11-A	1000	McF_4	0	unif	singleC	S1:DiP, S1:DiP-A, S1:OT, S1:OT-A
29	Yes	11	11-A	1000	McF_4	0	unif	wholeT_0.01	S1:CBN, S1:CBN-A, S1:OT, S1:OT-A
30	Yes	11	11-A	1000	McF_4	0	unif	wholeT_0.5	S1:CBN, S1:DiP, S1:DiP-A, S1:OT, S1:OT-A, S5:DiP, S5:DiP-A, S5:OT, S5:OT-A
31	Yes	11	11-A	1000	McF_4	Inf	last	singleC	S1:OT, S1:OT-A
32	Yes	11	11-A	1000	McF_4	Inf	last	wholeT_0.01	S1:OT, S1:OT-A, S5:OT, S5:OT-A
33	Yes	11	11-A	1000	McF_4	Inf	last	wholeT_0.5	S1:DiP-A, S1:OT, S1:OT-A
34	Yes	11	11-A	1000	McF_4	Inf	unif	singleC	S1:OT, S1:OT-A
35	Yes	11	11-A	1000	McF_4	Inf	unif	wholeT_0.01	S1:OT, S1:OT-A
36	Yes	11	11-A	1000	McF_4	Inf	unif	wholeT_0.5	S1:CBN, S1:CBN-A, S1:OT, S1:OT-A
37	Yes	11	11-A	1000	McF_6	0	last	singleC	S1:DiP, S1:DiP-A
38	Yes	11	11-A	1000	McF_6	0	last	wholeT_0.01	S1:DiP, S1:DiP-A, S5:DiP, S5:DiP-A
39	Yes	11	11-A	1000	McF_6	0	last	wholeT_0.5	S1:DiP, S1:DiP-A
40	Yes	11	11-A	1000	McF_6	0	unif	singleC	S1:CBN, S1:CBN-A, S1:DiP, S1:DiP-A, S1:OT, S1:OT-A
41	Yes	11	11-A	1000	McF_6	0	unif	wholeT_0.01	S1:CBN, S1:CBN-A, S1:DiP, S1:DiP-A, S1:OT, S1:OT-A
42	Yes	11	11-A	1000	McF_6	0	unif	wholeT_0.5	S1:CBN, S1:CBN-A, S1:DiP, S1:DiP-A, S1:OT, S1:OT-A
43	Yes	11	11-A	1000	McF_6	Inf	last	singleC	S1:DiP-A, S1:OT, S1:OT-A
44	Yes	11	11-A	1000	McF_6	Inf	last	wholeT_0.01	S1:DiP-A, S5:DiP-A
45	Yes	11	11-A	1000	McF_6	Inf	last	wholeT_0.5	S1:DiP, S1:DiP-A, S1:OT, S1:OT-A
46	Yes	11	11-A	1000	McF_6	Inf	unif	singleC	S1:CBN, S1:CBN-A, S1:DiP, S1:DiP-A, S1:OT, S1:OT-A
47	Yes	11	11-A	1000	McF_6	Inf	unif	wholeT_0.01	S1:CBN, S1:CBN-A, S1:DiP, S1:DiP-A, S1:OT, S1:OT-A
48	Yes	11	11-A	1000	McF_6	Inf	unif	wholeT_0.5	S1:CBN, S1:CBN-A, S1:DiP, S1:DiP-A, S1:OT, S1:OT-A
49	Yes	11	11-A	200	Bozic	0	last	singleC	S1:CBN, S1:CBN-A, S1:OT, S1:OT-A
50	Yes	11	11-A	200	Bozic	0	last	wholeT_0.01	S1:CBN, S1:CBN-A, S1:OT, S1:OT-A
51	Yes	11	11-A	200	Bozic	0	last	wholeT_0.5	S1:CBN-A, S1:OT, S1:OT-A
52	Yes	11	11-A	200	Bozic	0	unif	singleC	S1:CBN, S1:CBN-A, S1:OT, S1:OT-A
53	Yes	11	11-A	200	Bozic	0	unif	wholeT_0.01	S1:CBN, S1:CBN-A
54	Yes	11	11-A	200	Bozic	0	unif	wholeT_0.5	J1:CBN, J1:CBN-A, J1:OT, J1:OT-A, S1:CBN, S1:CBN-A, S1:OT, S1:OT-A

Table 7: *(continued)*

	Conjunction	Drivers	Tree	S.Size	Model	sh	S.Time	S.Type	Best method(s)
55	Yes	11	11-A	200	Bozic	Inf	last	singleC	S1:OT, S1:OT-A
56	Yes	11	11-A	200	Bozic	Inf	last	wholeT_0.01	J1:OT, J1:OT-A, S1:CBN-A, S1:OT, S1:OT-A, S5:CBN-A, S5:OT, S5:OT-A
57	Yes	11	11-A	200	Bozic	Inf	last	wholeT_0.5	S1:OT, S1:OT-A
58	Yes	11	11-A	200	Bozic	Inf	unif	singleC	S1:CBN, S1:CBN-A, S1:OT, S1:OT-A
59	Yes	11	11-A	200	Bozic	Inf	unif	wholeT_0.01	S1:CBN, S1:CBN-A, S1:OT, S1:OT-A, S5:OT, S5:OT-A
60	Yes	11	11-A	200	Bozic	Inf	unif	wholeT_0.5	S1:CBN-A, S1:OT, S1:OT-A
61	Yes	11	11-A	200	exp	0	last	singleC	S1:OT, S1:OT-A
62	Yes	11	11-A	200	exp	0	last	wholeT_0.01	S1:CBN-A, S1:OT, S1:OT-A, S5:OT, S5:OT-A
63	Yes	11	11-A	200	exp	0	last	wholeT_0.5	S1:OT, S1:OT-A
64	Yes	11	11-A	200	exp	0	unif	singleC	none
65	Yes	11	11-A	200	exp	0	unif	wholeT_0.01	S1:CBN, S1:CBN-A, S1:OT, S1:OT-A
66	Yes	11	11-A	200	exp	0	unif	wholeT_0.5	none
67	Yes	11	11-A	200	exp	Inf	last	singleC	S1:OT, S1:OT-A, S5:OT, S5:OT-A
68	Yes	11	11-A	200	exp	Inf	last	wholeT_0.01	J1:OT, J1:OT-A, S1:CBN-A, S1:OT, S1:OT-A, S5:OT, S5:OT-A
69	Yes	11	11-A	200	exp	Inf	last	wholeT_0.5	S1:CBN, S1:CBN-A, S1:OT, S1:OT-A
70	Yes	11	11-A	200	exp	Inf	unif	singleC	S1:OT, S1:OT-A
71	Yes	11	11-A	200	exp	Inf	unif	wholeT_0.01	S1:CBN, S1:CBN-A, S1:OT, S1:OT-A
72	Yes	11	11-A	200	exp	Inf	unif	wholeT_0.5	S1:CBN, S1:CBN-A, S1:OT, S1:OT-A
73	Yes	11	11-A	200	McF_4	0	last	singleC	S1:OT, S1:OT-A
74	Yes	11	11-A	200	McF_4	0	last	wholeT_0.01	S1:OT, S1:OT-A
75	Yes	11	11-A	200	McF_4	0	last	wholeT_0.5	S1:OT, S1:OT-A
76	Yes	11	11-A	200	McF_4	0	unif	singleC	S1:CBN, S1:CBN-A, S1:OT, S1:OT-A
77	Yes	11	11-A	200	McF_4	0	unif	wholeT_0.01	S1:CBN, S1:CBN-A, S1:OT, S1:OT-A
78	Yes	11	11-A	200	McF_4	0	unif	wholeT_0.5	S1:OT, S1:OT-A
79	Yes	11	11-A	200	McF_4	Inf	last	singleC	S1:OT, S1:OT-A
80	Yes	11	11-A	200	McF_4	Inf	last	wholeT_0.01	S1:OT, S1:OT-A, S5:OT, S5:OT-A
81	Yes	11	11-A	200	McF_4	Inf	last	wholeT_0.5	S1:OT, S1:OT-A
82	Yes	11	11-A	200	McF_4	Inf	unif	singleC	S1:OT, S1:OT-A
83	Yes	11	11-A	200	McF_4	Inf	unif	wholeT_0.01	S1:CBN-A, S1:OT, S1:OT-A
84	Yes	11	11-A	200	McF_4	Inf	unif	wholeT_0.5	S1:CBN, S1:OT, S1:OT-A
85	Yes	11	11-A	200	McF_6	0	last	singleC	S1:OT, S1:OT-A
86	Yes	11	11-A	200	McF_6	0	last	wholeT_0.01	S1:OT, S1:OT-A, S5:OT, S5:OT-A
87	Yes	11	11-A	200	McF_6	0	last	wholeT_0.5	S1:OT, S1:OT-A
88	Yes	11	11-A	200	McF_6	0	unif	singleC	S1:CBN, S1:CBN-A, S1:OT, S1:OT-A
89	Yes	11	11-A	200	McF_6	0	unif	wholeT_0.01	S1:CBN, S1:CBN-A, S1:OT, S1:OT-A
90	Yes	11	11-A	200	McF_6	0	unif	wholeT_0.5	S1:CBN, S1:CBN-A, S1:OT, S1:OT-A

Table 7: *(continued)*

	Conjunction	Drivers	Tree	S.Size	Model	sh	S.Time	S.Type	Best method(s)
91	Yes	11	11-A	200	McF_6	Inf	last	singleC	S1:OT, S1:OT-A
92	Yes	11	11-A	200	McF_6	Inf	last	wholeT_0.01	S1:OT, S1:OT-A, S5:OT, S5:OT-A
93	Yes	11	11-A	200	McF_6	Inf	last	wholeT_0.5	S1:OT, S1:OT-A
94	Yes	11	11-A	200	McF_6	Inf	unif	singleC	S1:CBN, S1:CBN-A, S1:OT, S1:OT-A
95	Yes	11	11-A	200	McF_6	Inf	unif	wholeT_0.01	S1:OT, S1:OT-A
96	Yes	11	11-A	200	McF_6	Inf	unif	wholeT_0.5	S1:CBN-A, S1:OT, S1:OT-A
97	Yes	11	11-A	100	Bozic	0	last	singleC	S1:CBN, S1:CBN-A, S1:OT, S1:OT-A
98	Yes	11	11-A	100	Bozic	0	last	wholeT_0.01	S1:CBN, S1:OT, S1:OT-A
99	Yes	11	11-A	100	Bozic	0	last	wholeT_0.5	S1:CBN-A, S1:OT, S1:OT-A
100	Yes	11	11-A	100	Bozic	0	unif	singleC	S1:CBN, S1:CBN-A, S1:OT, S1:OT-A
101	Yes	11	11-A	100	Bozic	0	unif	wholeT_0.01	S1:CBN, S1:CBN-A
102	Yes	11	11-A	100	Bozic	0	unif	wholeT_0.5	S1:CBN, S1:CBN-A, S1:OT, S1:OT-A
103	Yes	11	11-A	100	Bozic	Inf	last	singleC	S1:OT, S1:OT-A
104	Yes	11	11-A	100	Bozic	Inf	last	wholeT_0.01	S1:OT, S1:OT-A, S5:CBN-A, S5:OT, S5:OT-A
105	Yes	11	11-A	100	Bozic	Inf	last	wholeT_0.5	S1:CBN, S1:CBN-A, S1:OT, S1:OT-A
106	Yes	11	11-A	100	Bozic	Inf	unif	singleC	S1:CBN, S1:CBN-A, S1:OT, S1:OT-A
107	Yes	11	11-A	100	Bozic	Inf	unif	wholeT_0.01	J1:CBN, J1:CBN-A, J1:OT, J1:OT-A, S1:CBN, S1:CBN-A, S1:OT, S1:OT-A, S5:CBN, S5:CBN-A, S5:OT, S5:OT-A
108	Yes	11	11-A	100	Bozic	Inf	unif	wholeT_0.5	S1:CBN, S1:CBN-A, S1:OT, S1:OT-A
109	Yes	11	11-A	100	exp	0	last	singleC	S1:OT, S1:OT-A
110	Yes	11	11-A	100	exp	0	last	wholeT_0.01	S1:CBN, S1:CBN-A, S1:OT, S1:OT-A
111	Yes	11	11-A	100	exp	0	last	wholeT_0.5	S1:CBN-A, S1:OT, S1:OT-A
112	Yes	11	11-A	100	exp	0	unif	singleC	none
113	Yes	11	11-A	100	exp	0	unif	wholeT_0.01	S1:CBN, S1:CBN-A, S1:OT, S1:OT-A
114	Yes	11	11-A	100	exp	0	unif	wholeT_0.5	none
115	Yes	11	11-A	100	exp	Inf	last	singleC	S1:OT, S1:OT-A
116	Yes	11	11-A	100	exp	Inf	last	wholeT_0.01	J1:CBN, J1:OT, J1:OT-A, S1:OT, S1:OT-A, S5:CBN-A, S5:OT, S5:OT-A
117	Yes	11	11-A	100	exp	Inf	last	wholeT_0.5	S1:CBN, S1:OT, S1:OT-A
118	Yes	11	11-A	100	exp	Inf	unif	singleC	S1:CBN, S1:OT, S1:OT-A
119	Yes	11	11-A	100	exp	Inf	unif	wholeT_0.01	J1:CBN, J1:CBN-A, S1:CBN, S1:CBN-A, S1:OT, S1:OT-A
120	Yes	11	11-A	100	exp	Inf	unif	wholeT_0.5	S1:CBN, S1:CBN-A, S1:OT, S1:OT-A
121	Yes	11	11-A	100	McF_4	0	last	singleC	S1:CBN, S1:OT, S1:OT-A
122	Yes	11	11-A	100	McF_4	0	last	wholeT_0.01	S1:OT, S1:OT-A
123	Yes	11	11-A	100	McF_4	0	last	wholeT_0.5	S1:OT, S1:OT-A
124	Yes	11	11-A	100	McF_4	0	unif	singleC	S1:CBN, S1:CBN-A, S1:OT, S1:OT-A
125	Yes	11	11-A	100	McF_4	0	unif	wholeT_0.01	S1:CBN, S1:CBN-A, S1:OT, S1:OT-A

Table 7: *(continued)*

	Conjunction	Drivers	Tree	S.Size	Model	sh	S.Time	S.Type	Best method(s)
126	Yes	11	11-A	100	McF_4	0	unif	wholeT_0.5	S1:CBN, S1:CBN-A, S1:OT, S1:OT-A
127	Yes	11	11-A	100	McF_4	Inf	last	singleC	S1:OT, S1:OT-A
128	Yes	11	11-A	100	McF_4	Inf	last	wholeT_0.01	S1:OT, S1:OT-A, S5:OT, S5:OT-A
129	Yes	11	11-A	100	McF_4	Inf	last	wholeT_0.5	S1:OT, S1:OT-A
130	Yes	11	11-A	100	McF_4	Inf	unif	singleC	S1:OT, S1:OT-A
131	Yes	11	11-A	100	McF_4	Inf	unif	wholeT_0.01	S1:OT, S1:OT-A
132	Yes	11	11-A	100	McF_4	Inf	unif	wholeT_0.5	S1:OT, S1:OT-A
133	Yes	11	11-A	100	McF_6	0	last	singleC	S1:OT, S1:OT-A
134	Yes	11	11-A	100	McF_6	0	last	wholeT_0.01	S1:OT, S1:OT-A, S5:OT, S5:OT-A
135	Yes	11	11-A	100	McF_6	0	last	wholeT_0.5	S1:OT, S1:OT-A
136	Yes	11	11-A	100	McF_6	0	unif	singleC	S1:CBN, S1:CBN-A, S1:OT, S1:OT-A
137	Yes	11	11-A	100	McF_6	0	unif	wholeT_0.01	S1:CBN, S1:CBN-A, S1:OT, S1:OT-A
138	Yes	11	11-A	100	McF_6	0	unif	wholeT_0.5	S1:CBN, S1:OT, S1:OT-A
139	Yes	11	11-A	100	McF_6	Inf	last	singleC	S1:OT, S1:OT-A, S5:OT, S5:OT-A
140	Yes	11	11-A	100	McF_6	Inf	last	wholeT_0.01	S1:OT, S1:OT-A, S5:OT, S5:OT-A
141	Yes	11	11-A	100	McF_6	Inf	last	wholeT_0.5	S1:OT, S1:OT-A
142	Yes	11	11-A	100	McF_6	Inf	unif	singleC	S1:CBN, S1:OT, S1:OT-A
143	Yes	11	11-A	100	McF_6	Inf	unif	wholeT_0.01	S1:CBN, S1:CBN-A, S1:OT, S1:OT-A
144	Yes	11	11-A	100	McF_6	Inf	unif	wholeT_0.5	S1:CBN, S1:CBN-A, S1:OT, S1:OT-A
145	Yes	9	9-A	1000	Bozic	0	last	singleC	S1:OT, S1:OT-A
146	Yes	9	9-A	1000	Bozic	0	last	wholeT_0.01	S1:DiP, S1:DiP-A, S1:OT, S1:OT-A, S5:DiP, S5:DiP-A, S5:OT, S5:OT-A
147	Yes	9	9-A	1000	Bozic	0	last	wholeT_0.5	S1:OT, S1:OT-A
148	Yes	9	9-A	1000	Bozic	0	unif	singleC	none
149	Yes	9	9-A	1000	Bozic	0	unif	wholeT_0.01	S1:CBN, S1:CBN-A
150	Yes	9	9-A	1000	Bozic	0	unif	wholeT_0.5	none
151	Yes	9	9-A	1000	Bozic	Inf	last	singleC	S1:CBN, S1:CBN-A, S1:OT, S1:OT-A, S5:CBN-A
152	Yes	9	9-A	1000	Bozic	Inf	last	wholeT_0.01	J1:CBN-A, S1:CBN, S1:CBN-A, S1:DiP-A, S1:OT, S1:OT-A, S5:CBN, S5:CBN-A, S5:DiP-A, S5:OT, S5:OT-A
153	Yes	9	9-A	1000	Bozic	Inf	last	wholeT_0.5	S1:CBN, S1:CBN-A, S1:OT, S1:OT-A
154	Yes	9	9-A	1000	Bozic	Inf	unif	singleC	S1:CBN, S1:CBN-A, S1:OT, S1:OT-A
155	Yes	9	9-A	1000	Bozic	Inf	unif	wholeT_0.01	S1:CBN, S1:CBN-A, S1:DiP, S1:DiP-A, S1:OT, S1:OT-A, S5:CBN, S5:CBN-A, S5:DiP, S5:DiP-A, S5:OT, S5:OT-A
156	Yes	9	9-A	1000	Bozic	Inf	unif	wholeT_0.5	S1:CBN, S1:CBN-A, S1:OT, S1:OT-A
157	Yes	9	9-A	1000	exp	0	last	singleC	S1:CBN, S1:CBN-A, S1:DiP, S1:DiP-A, S1:OT, S1:OT-A
158	Yes	9	9-A	1000	exp	0	last	wholeT_0.01	S1:CBN-A, S1:DiP-A, S1:OT, S1:OT-A

Table 7: *(continued)*

	Conjunction	Drivers	Tree	S.Size	Model	sh	S.Time	S.Type	Best method(s)
159	Yes	9	9-A	1000	exp	0	last	wholeT_0.5	none
160	Yes	9	9-A	1000	exp	0	unif	singleC	none
161	Yes	9	9-A	1000	exp	0	unif	wholeT_0.01	S1:CBN, S1:CBN-A, S1:DiP, S1:DiP-A, S1:OT, S1:OT-A
162	Yes	9	9-A	1000	exp	0	unif	wholeT_0.5	none
163	Yes	9	9-A	1000	exp	Inf	last	singleC	S1:CBN, S1:CBN-A, S1:OT, S1:OT-A
164	Yes	9	9-A	1000	exp	Inf	last	wholeT_0.01	S1:CBN, S1:CBN-A, S1:DiP, S1:DiP-A, S1:OT, S1:OT-A, S5:CBN, S5:CBN-A, S5:DiP, S5:DiP-A, S5:OT, S5:OT-A
165	Yes	9	9-A	1000	exp	Inf	last	wholeT_0.5	S1:CBN, S1:CBN-A, S1:OT, S1:OT-A
166	Yes	9	9-A	1000	exp	Inf	unif	singleC	S1:CBN, S1:CBN-A, S1:OT, S1:OT-A
167	Yes	9	9-A	1000	exp	Inf	unif	wholeT_0.01	S1:CBN, S1:CBN-A, S1:OT, S1:OT-A
168	Yes	9	9-A	1000	exp	Inf	unif	wholeT_0.5	S1:CBN-A, S1:OT, S1:OT-A
169	Yes	9	9-A	1000	McF_4	0	last	singleC	S1:CBN, S1:DiP, S1:DiP-A, S1:OT, S1:OT-A
170	Yes	9	9-A	1000	McF_4	0	last	wholeT_0.01	S1:CBN, S1:CBN-A, S1:DiP, S1:DiP-A, S1:OT, S1:OT-A
171	Yes	9	9-A	1000	McF_4	0	last	wholeT_0.5	S1:DiP, S1:DiP-A, S1:OT, S1:OT-A
172	Yes	9	9-A	1000	McF_4	0	unif	singleC	S1:CBN, S1:CBN-A, S1:DiP, S1:DiP-A, S1:OT, S1:OT-A
173	Yes	9	9-A	1000	McF_4	0	unif	wholeT_0.01	S1:CBN, S1:CBN-A, S1:DiP-A, S1:OT-A
174	Yes	9	9-A	1000	McF_4	0	unif	wholeT_0.5	S1:CBN, S1:CBN-A, S1:DiP, S1:DiP-A, S1:OT, S1:OT-A
175	Yes	9	9-A	1000	McF_4	Inf	last	singleC	S1:OT, S1:OT-A
176	Yes	9	9-A	1000	McF_4	Inf	last	wholeT_0.01	S1:OT-A
177	Yes	9	9-A	1000	McF_4	Inf	last	wholeT_0.5	S1:CBN, S1:DiP, S1:DiP-A, S1:OT, S1:OT-A
178	Yes	9	9-A	1000	McF_4	Inf	unif	singleC	S1:CBN, S1:CBN-A, S1:OT, S1:OT-A
179	Yes	9	9-A	1000	McF_4	Inf	unif	wholeT_0.01	S1:CBN, S1:CBN-A, S1:OT, S1:OT-A
180	Yes	9	9-A	1000	McF_4	Inf	unif	wholeT_0.5	S1:CBN, S1:CBN-A, S1:OT, S1:OT-A
181	Yes	9	9-A	1000	McF_6	0	last	singleC	S1:OT-A
182	Yes	9	9-A	1000	McF_6	0	last	wholeT_0.01	S1:DiP-A, S5:DiP-A
183	Yes	9	9-A	1000	McF_6	0	last	wholeT_0.5	S1:DiP-A, S1:OT-A, S5:DiP-A, S5:OT-A
184	Yes	9	9-A	1000	McF_6	0	unif	singleC	S1:CBN, S1:CBN-A, S1:DiP, S1:DiP-A
185	Yes	9	9-A	1000	McF_6	0	unif	wholeT_0.01	S1:CBN, S1:CBN-A, S1:DiP, S1:DiP-A
186	Yes	9	9-A	1000	McF_6	0	unif	wholeT_0.5	S1:CBN, S1:CBN-A, S1:DiP, S1:DiP-A
187	Yes	9	9-A	1000	McF_6	Inf	last	singleC	S1:DiP-A, S1:OT-A, S5:DiP-A, S5:OT-A
188	Yes	9	9-A	1000	McF_6	Inf	last	wholeT_0.01	S1:OT-A, S5:OT-A
189	Yes	9	9-A	1000	McF_6	Inf	last	wholeT_0.5	S1:DiP-A, S1:OT-A, S5:DiP-A, S5:OT-A
190	Yes	9	9-A	1000	McF_6	Inf	unif	singleC	S1:CBN, S1:CBN-A

Table 7: *(continued)*

	Conjunction	Drivers	Tree	S.Size	Model	sh	S.Time	S.Type	Best method(s)
191	Yes	9	9-A	1000	McF_6	Inf	unif	wholeT_0.01	S1:CBN, S1:CBN-A, S1:DiP, S1:DiP-A, S1:OT, S1:OT-A
192	Yes	9	9-A	1000	McF_6	Inf	unif	wholeT_0.5	S1:CBN, S1:CBN-A
193	Yes	9	9-A	200	Bozic	0	last	singleC	S1:CBN, S1:CBN-A, S1:OT, S1:OT-A
194	Yes	9	9-A	200	Bozic	0	last	wholeT_0.01	S1:CBN, S1:CBN-A, S1:OT, S1:OT-A
195	Yes	9	9-A	200	Bozic	0	last	wholeT_0.5	S1:OT, S1:OT-A
196	Yes	9	9-A	200	Bozic	0	unif	singleC	S1:CBN, S1:CBN-A, S1:OT, S1:OT-A
197	Yes	9	9-A	200	Bozic	0	unif	wholeT_0.01	S1:CBN, S1:CBN-A, S1:OT-A
198	Yes	9	9-A	200	Bozic	0	unif	wholeT_0.5	none
199	Yes	9	9-A	200	Bozic	Inf	last	singleC	S1:CBN, S1:CBN-A, S1:OT, S1:OT-A
200	Yes	9	9-A	200	Bozic	Inf	last	wholeT_0.01	S1:CBN, S1:CBN-A, S5:CBN, S5:CBN-A
201	Yes	9	9-A	200	Bozic	Inf	last	wholeT_0.5	S1:CBN, S1:CBN-A, S1:OT, S1:OT-A
202	Yes	9	9-A	200	Bozic	Inf	unif	singleC	S1:CBN, S1:CBN-A, S1:OT, S1:OT-A
203	Yes	9	9-A	200	Bozic	Inf	unif	wholeT_0.01	J1:CBN, J1:CBN-A, S1:CBN, S1:CBN-A, S1:OT, S1:OT-A, S5:CBN, S5:CBN-A, S5:OT, S5:OT-A
204	Yes	9	9-A	200	Bozic	Inf	unif	wholeT_0.5	S1:CBN, S1:CBN-A, S1:OT, S1:OT-A
205	Yes	9	9-A	200	exp	0	last	singleC	S1:OT, S1:OT-A
206	Yes	9	9-A	200	exp	0	last	wholeT_0.01	S1:CBN-A, S1:OT, S1:OT-A
207	Yes	9	9-A	200	exp	0	last	wholeT_0.5	S1:CBN, S1:OT, S1:OT-A
208	Yes	9	9-A	200	exp	0	unif	singleC	none
209	Yes	9	9-A	200	exp	0	unif	wholeT_0.01	S1:CBN-A, S1:OT, S1:OT-A
210	Yes	9	9-A	200	exp	0	unif	wholeT_0.5	none
211	Yes	9	9-A	200	exp	Inf	last	singleC	S1:CBN, S1:CBN-A, S1:OT, S1:OT-A
212	Yes	9	9-A	200	exp	Inf	last	wholeT_0.01	S1:CBN, S1:CBN-A, S5:CBN, S5:CBN-A
213	Yes	9	9-A	200	exp	Inf	last	wholeT_0.5	S1:CBN, S1:CBN-A, S1:OT, S1:OT-A
214	Yes	9	9-A	200	exp	Inf	unif	singleC	S1:CBN, S1:CBN-A, S1:OT, S1:OT-A
215	Yes	9	9-A	200	exp	Inf	unif	wholeT_0.01	S1:CBN, S1:CBN-A
216	Yes	9	9-A	200	exp	Inf	unif	wholeT_0.5	S1:CBN, S1:CBN-A, S1:OT, S1:OT-A
217	Yes	9	9-A	200	McF_4	0	last	singleC	S1:CBN, S1:CBN-A, S1:OT, S1:OT-A
218	Yes	9	9-A	200	McF_4	0	last	wholeT_0.01	S1:CBN, S1:CBN-A, S1:OT, S1:OT-A
219	Yes	9	9-A	200	McF_4	0	last	wholeT_0.5	S1:CBN, S1:CBN-A, S1:OT, S1:OT-A
220	Yes	9	9-A	200	McF_4	0	unif	singleC	S1:CBN, S1:CBN-A, S1:OT, S1:OT-A
221	Yes	9	9-A	200	McF_4	0	unif	wholeT_0.01	S1:CBN, S1:CBN-A
222	Yes	9	9-A	200	McF_4	0	unif	wholeT_0.5	S1:CBN, S1:CBN-A, S1:OT, S1:OT-A
223	Yes	9	9-A	200	McF_4	Inf	last	singleC	S1:CBN-A, S1:OT, S1:OT-A
224	Yes	9	9-A	200	McF_4	Inf	last	wholeT_0.01	S1:OT-A
225	Yes	9	9-A	200	McF_4	Inf	last	wholeT_0.5	S1:CBN, S1:CBN-A, S1:OT, S1:OT-A
226	Yes	9	9-A	200	McF_4	Inf	unif	singleC	S1:CBN, S1:CBN-A, S1:OT, S1:OT-A
227	Yes	9	9-A	200	McF_4	Inf	unif	wholeT_0.01	S1:CBN, S1:CBN-A, S1:OT, S1:OT-A

Table 7: *(continued)*

	Conjunction	Drivers	Tree	S.Size	Model	sh	S.Time	S.Type	Best method(s)
228	Yes	9	9-A	200	McF_4	Inf	unif	wholeT_0.5	S1:CBN, S1:CBN-A, S1:OT, S1:OT-A
229	Yes	9	9-A	200	McF_6	0	last	singleC	S1:OT-A, S5:OT-A
230	Yes	9	9-A	200	McF_6	0	last	wholeT_0.01	S1:OT-A, S5:OT-A
231	Yes	9	9-A	200	McF_6	0	last	wholeT_0.5	S1:OT-A, S5:OT-A
232	Yes	9	9-A	200	McF_6	0	unif	singleC	S1:CBN, S1:CBN-A, S1:OT, S1:OT-A
233	Yes	9	9-A	200	McF_6	0	unif	wholeT_0.01	S1:CBN, S1:CBN-A
234	Yes	9	9-A	200	McF_6	0	unif	wholeT_0.5	S1:CBN, S1:CBN-A
235	Yes	9	9-A	200	McF_6	Inf	last	singleC	S1:OT-A, S5:OT-A
236	Yes	9	9-A	200	McF_6	Inf	last	wholeT_0.01	S1:OT-A, S5:OT-A
237	Yes	9	9-A	200	McF_6	Inf	last	wholeT_0.5	S1:OT-A, S5:OT-A
238	Yes	9	9-A	200	McF_6	Inf	unif	singleC	S1:CBN, S1:CBN-A
239	Yes	9	9-A	200	McF_6	Inf	unif	wholeT_0.01	S1:CBN, S1:CBN-A, S1:OT, S1:OT-A, S5:CBN-A
240	Yes	9	9-A	200	McF_6	Inf	unif	wholeT_0.5	S1:CBN, S1:CBN-A, S1:OT, S1:OT-A
241	Yes	9	9-A	100	Bozic	0	last	singleC	S1:CBN, S1:CBN-A, S1:OT, S1:OT-A
242	Yes	9	9-A	100	Bozic	0	last	wholeT_0.01	S1:CBN, S1:CBN-A, S1:OT, S1:OT-A
243	Yes	9	9-A	100	Bozic	0	last	wholeT_0.5	S1:CBN, S1:CBN-A, S1:OT, S1:OT-A
244	Yes	9	9-A	100	Bozic	0	unif	singleC	S1:CBN, S1:CBN-A, S1:OT, S1:OT-A
245	Yes	9	9-A	100	Bozic	0	unif	wholeT_0.01	S1:CBN, S1:CBN-A
246	Yes	9	9-A	100	Bozic	0	unif	wholeT_0.5	S1:CBN, S1:CBN-A, S1:OT, S1:OT-A
247	Yes	9	9-A	100	Bozic	Inf	last	singleC	S1:CBN, S1:CBN-A, S1:OT, S1:OT-A
248	Yes	9	9-A	100	Bozic	Inf	last	wholeT_0.01	J1:CBN, J1:CBN-A, S1:CBN, S1:CBN-A, S1:OT, S1:OT-A, S5:CBN, S5:CBN-A, S5:OT, S5:OT-A
249	Yes	9	9-A	100	Bozic	Inf	last	wholeT_0.5	S1:CBN, S1:CBN-A, S1:OT, S1:OT-A, S5:CBN-A, S5:OT, S5:OT-A
250	Yes	9	9-A	100	Bozic	Inf	unif	singleC	S1:CBN, S1:CBN-A, S1:OT, S1:OT-A
251	Yes	9	9-A	100	Bozic	Inf	unif	wholeT_0.01	S1:CBN, S1:CBN-A, S1:OT, S1:OT-A, S5:CBN-A
252	Yes	9	9-A	100	Bozic	Inf	unif	wholeT_0.5	S1:CBN, S1:CBN-A, S1:OT, S1:OT-A
253	Yes	9	9-A	100	exp	0	last	singleC	S1:CBN-A, S1:OT, S1:OT-A
254	Yes	9	9-A	100	exp	0	last	wholeT_0.01	S1:CBN-A, S1:OT, S1:OT-A
255	Yes	9	9-A	100	exp	0	last	wholeT_0.5	S1:OT, S1:OT-A
256	Yes	9	9-A	100	exp	0	unif	singleC	none
257	Yes	9	9-A	100	exp	0	unif	wholeT_0.01	S1:CBN, S1:CBN-A, S1:OT, S1:OT-A
258	Yes	9	9-A	100	exp	0	unif	wholeT_0.5	none
259	Yes	9	9-A	100	exp	Inf	last	singleC	S1:CBN, S1:CBN-A, S1:OT, S1:OT-A
260	Yes	9	9-A	100	exp	Inf	last	wholeT_0.01	S1:CBN, S1:CBN-A, S1:OT, S1:OT-A, S5:CBN, S5:CBN-A, S5:OT, S5:OT-A
261	Yes	9	9-A	100	exp	Inf	last	wholeT_0.5	S1:CBN, S1:CBN-A

Table 7: *(continued)*

	Conjunction	Drivers	Tree	S.Size	Model	sh	S.Time	S.Type	Best method(s)
262	Yes	9	9-A	100	exp	Inf	unif	singleC	S1:CBN, S1:CBN-A, S1:OT, S1:OT-A
263	Yes	9	9-A	100	exp	Inf	unif	wholeT_0.01	S1:CBN, S1:CBN-A
264	Yes	9	9-A	100	exp	Inf	unif	wholeT_0.5	S1:CBN, S1:CBN-A, S1:OT, S1:OT-A
265	Yes	9	9-A	100	McF_4	0	last	singleC	S1:CBN, S1:CBN-A, S1:OT, S1:OT-A
266	Yes	9	9-A	100	McF_4	0	last	wholeT_0.01	S1:CBN, S1:CBN-A, S1:OT, S1:OT-A
267	Yes	9	9-A	100	McF_4	0	last	wholeT_0.5	S1:CBN, S1:CBN-A, S1:OT, S1:OT-A
268	Yes	9	9-A	100	McF_4	0	unif	singleC	S1:CBN, S1:CBN-A, S1:OT, S1:OT-A
269	Yes	9	9-A	100	McF_4	0	unif	wholeT_0.01	S1:CBN, S1:CBN-A
270	Yes	9	9-A	100	McF_4	0	unif	wholeT_0.5	S1:CBN, S1:CBN-A, S1:OT, S1:OT-A
271	Yes	9	9-A	100	McF_4	Inf	last	singleC	S1:CBN, S1:CBN-A, S1:OT, S1:OT-A
272	Yes	9	9-A	100	McF_4	Inf	last	wholeT_0.01	S1:OT-A
273	Yes	9	9-A	100	McF_4	Inf	last	wholeT_0.5	S1:CBN, S1:CBN-A, S1:OT, S1:OT-A
274	Yes	9	9-A	100	McF_4	Inf	unif	singleC	S1:CBN, S1:CBN-A, S1:OT, S1:OT-A
275	Yes	9	9-A	100	McF_4	Inf	unif	wholeT_0.01	S1:CBN, S1:CBN-A, S1:OT, S1:OT-A
276	Yes	9	9-A	100	McF_4	Inf	unif	wholeT_0.5	S1:CBN, S1:CBN-A, S1:OT, S1:OT-A
277	Yes	9	9-A	100	McF_6	0	last	singleC	S1:OT-A
278	Yes	9	9-A	100	McF_6	0	last	wholeT_0.01	S1:OT-A, S5:OT-A
279	Yes	9	9-A	100	McF_6	0	last	wholeT_0.5	S1:OT-A
280	Yes	9	9-A	100	McF_6	0	unif	singleC	S1:CBN, S1:CBN-A
281	Yes	9	9-A	100	McF_6	0	unif	wholeT_0.01	S1:CBN, S1:CBN-A
282	Yes	9	9-A	100	McF_6	0	unif	wholeT_0.5	S1:CBN, S1:CBN-A
283	Yes	9	9-A	100	McF_6	Inf	last	singleC	S1:OT-A, S5:OT-A
284	Yes	9	9-A	100	McF_6	Inf	last	wholeT_0.01	S1:OT-A, S5:OT-A
285	Yes	9	9-A	100	McF_6	Inf	last	wholeT_0.5	S1:OT-A, S5:OT-A
286	Yes	9	9-A	100	McF_6	Inf	unif	singleC	S1:CBN, S1:CBN-A
287	Yes	9	9-A	100	McF_6	Inf	unif	wholeT_0.01	S1:CBN, S1:CBN-A
288	Yes	9	9-A	100	McF_6	Inf	unif	wholeT_0.5	S1:CBN, S1:CBN-A
289	Yes	7	7-A	1000	Bozic	0	last	singleC	S1:OT-A, S5:OT-A
290	Yes	7	7-A	1000	Bozic	0	last	wholeT_0.01	J1:DiP-A, J1:OT-A, J5:DiP-A, J5:OT-A, S1:DiP-A, S1:OT-A, S5:DiP-A, S5:OT-A
291	Yes	7	7-A	1000	Bozic	0	last	wholeT_0.5	S1:OT-A
292	Yes	7	7-A	1000	Bozic	0	unif	singleC	S1:CBN-A, S1:OT, S1:OT-A
293	Yes	7	7-A	1000	Bozic	0	unif	wholeT_0.01	S1:CBN, S1:CBN-A, S1:DiP, S1:DiP-A
294	Yes	7	7-A	1000	Bozic	0	unif	wholeT_0.5	J1:CBN, J1:CBN-A, J1:DiP-A, J1:OT, J1:OT-A, S1:CBN, S1:CBN-A, S1:DiP-A, S1:OT, S1:OT-A
295	Yes	7	7-A	1000	Bozic	Inf	last	singleC	J1:DiP-A, J1:OT-A, J5:DiP-A, J5:OT-A, S1:DiP-A, S1:OT-A, S5:DiP-A, S5:OT-A
296	Yes	7	7-A	1000	Bozic	Inf	last	wholeT_0.01	J1:DiP-A, J1:OT-A, J5:DiP-A, J5:OT-A, S1:DiP-A, S1:OT-A, S5:DiP-A, S5:OT-A

Table 7: *(continued)*

	Conjunction	Drivers	Tree	S.Size	Model	sh	S.Time	S.Type	Best method(s)
297	Yes	7	7-A	1000	Bozic	Inf	last	wholeT_0.5	J1:DiP-A, J1:OT-A, J5:DiP-A, J5:OT-A, S1:DiP-A, S1:OT-A, S5:DiP-A, S5:OT-A
298	Yes	7	7-A	1000	Bozic	Inf	unif	singleC	S1:CBN, S1:CBN-A, S1:OT, S1:OT-A
299	Yes	7	7-A	1000	Bozic	Inf	unif	wholeT_0.01	J1:CBN, J1:CBN-A, J1:DiP-A, J1:OT, J1:OT-A, J5:CBN, J5:CBN-A, J5:DiP-A, J5:OT, J5:OT-A, S1:CBN, S1:CBN-A, S1:DiP-A, S1:OT, S1:OT-A, S5:CBN, S5:CBN-A, S5:DiP-A, S5:OT, S5:OT-A
300	Yes	7	7-A	1000	Bozic	Inf	unif	wholeT_0.5	S1:CBN, S1:CBN-A, S1:OT, S1:OT-A
301	Yes	7	7-A	1000	exp	0	last	singleC	S1:OT-A
302	Yes	7	7-A	1000	exp	0	last	wholeT_0.01	J1:OT-A, S1:OT-A, S5:OT-A
303	Yes	7	7-A	1000	exp	0	last	wholeT_0.5	S1:OT-A
304	Yes	7	7-A	1000	exp	0	unif	singleC	none
305	Yes	7	7-A	1000	exp	0	unif	wholeT_0.01	S1:CBN-A, S1:OT, S1:OT-A
306	Yes	7	7-A	1000	exp	0	unif	wholeT_0.5	none
307	Yes	7	7-A	1000	exp	Inf	last	singleC	J1:OT-A, S1:OT-A, S5:OT-A
308	Yes	7	7-A	1000	exp	Inf	last	wholeT_0.01	J1:DiP-A, J1:OT-A, J5:DiP-A, J5:OT-A, S1:DiP-A, S1:OT-A, S5:DiP-A, S5:OT-A
309	Yes	7	7-A	1000	exp	Inf	last	wholeT_0.5	J1:OT-A, S1:OT-A, S5:OT-A
310	Yes	7	7-A	1000	exp	Inf	unif	singleC	S1:CBN, S1:CBN-A, S1:OT, S1:OT-A
311	Yes	7	7-A	1000	exp	Inf	unif	wholeT_0.01	J1:CBN, J1:CBN-A, J1:OT, J1:OT-A, J5:CBN, J5:CBN-A, J5:OT, J5:OT-A, S1:CBN, S1:CBN-A, S1:OT, S1:OT-A, S5:CBN, S5:CBN-A, S5:OT, S5:OT-A
312	Yes	7	7-A	1000	exp	Inf	unif	wholeT_0.5	S1:CBN-A, S1:OT, S1:OT-A
313	Yes	7	7-A	1000	McF_4	0	last	singleC	J1:DiP-A, J1:OT-A, J5:DiP-A, J5:OT-A, S1:DiP-A, S1:OT-A, S5:DiP-A, S5:OT-A
314	Yes	7	7-A	1000	McF_4	0	last	wholeT_0.01	S1:OT-A
315	Yes	7	7-A	1000	McF_4	0	last	wholeT_0.5	J1:DiP-A, J1:OT-A, J5:DiP-A, J5:OT-A, S1:DiP-A, S1:OT-A, S5:DiP-A, S5:OT-A
316	Yes	7	7-A	1000	McF_4	0	unif	singleC	J1:DiP-A, J1:OT, J1:OT-A, S1:DiP-A, S1:OT, S1:OT-A, S5:DiP-A, S5:OT, S5:OT-A
317	Yes	7	7-A	1000	McF_4	0	unif	wholeT_0.01	S1:CBN, S1:CBN-A, S1:DiP, S1:DiP-A, S1:OT, S1:OT-A
318	Yes	7	7-A	1000	McF_4	0	unif	wholeT_0.5	J1:DiP-A, J1:OT, J1:OT-A, S1:DiP-A, S1:OT, S1:OT-A, S5:DiP-A, S5:OT, S5:OT-A
319	Yes	7	7-A	1000	McF_4	Inf	last	singleC	J1:DiP-A, J1:OT-A, J5:DiP-A, J5:OT-A, S1:DiP-A, S1:OT-A, S5:DiP-A, S5:OT-A

Table 7: *(continued)*

	Conjunction	Drivers	Tree	S.Size	Model	sh	S.Time	S.Type	Best method(s)
320	Yes	7	7-A	1000	McF_4	Inf	last	wholeT_0.01	S1:OT-A
321	Yes	7	7-A	1000	McF_4	Inf	last	wholeT_0.5	J1:DiP-A, J1:OT-A, J5:DiP-A, J5:OT-A, S1:DiP-A, S1:OT-A, S5:DiP-A, S5:OT-A
322	Yes	7	7-A	1000	McF_4	Inf	unif	singleC	J1:DiP-A, J1:OT, J1:OT-A, S1:DiP-A, S1:OT, S1:OT-A, S5:DiP-A, S5:OT, S5:OT-A
323	Yes	7	7-A	1000	McF_4	Inf	unif	wholeT_0.01	J1:DiP-A, J1:OT, J1:OT-A, J5:OT, J5:OT-A, S1:DiP-A, S1:OT, S1:OT-A, S5:DiP-A, S5:OT, S5:OT-A
324	Yes	7	7-A	1000	McF_4	Inf	unif	wholeT_0.5	J1:DiP-A, J1:OT, J1:OT-A, S1:DiP-A, S1:OT, S1:OT-A, S5:DiP-A, S5:OT, S5:OT-A
325	Yes	7	7-A	1000	McF_6	0	last	singleC	S1:DiP-A, S5:DiP-A
326	Yes	7	7-A	1000	McF_6	0	last	wholeT_0.01	J1:DiP-A, J1:OT-A, S1:OT-A, S5:DiP-A, S5:OT-A
327	Yes	7	7-A	1000	McF_6	0	last	wholeT_0.5	S1:DiP-A, S5:DiP-A
328	Yes	7	7-A	1000	McF_6	0	unif	singleC	S1:CBN-A
329	Yes	7	7-A	1000	McF_6	0	unif	wholeT_0.01	S1:CBN-A, S1:DiP, S1:DiP-A
330	Yes	7	7-A	1000	McF_6	0	unif	wholeT_0.5	S1:CBN-A
331	Yes	7	7-A	1000	McF_6	Inf	last	singleC	S1:CBN-A, S1:DiP-A, S1:OT-A, S5:CBN-A, S5:DiP-A, S5:OT-A
332	Yes	7	7-A	1000	McF_6	Inf	last	wholeT_0.01	S5:DiP-A
333	Yes	7	7-A	1000	McF_6	Inf	last	wholeT_0.5	S1:CBN-A, S1:DiP-A, S5:CBN-A, S5:DiP-A
334	Yes	7	7-A	1000	McF_6	Inf	unif	singleC	S1:CBN-A
335	Yes	7	7-A	1000	McF_6	Inf	unif	wholeT_0.01	S1:CBN-A
336	Yes	7	7-A	1000	McF_6	Inf	unif	wholeT_0.5	S1:CBN-A
337	Yes	7	7-A	200	Bozic	0	last	singleC	S1:OT-A, S5:OT-A
338	Yes	7	7-A	200	Bozic	0	last	wholeT_0.01	J1:OT-A, J5:OT-A, S1:OT-A, S5:OT-A
339	Yes	7	7-A	200	Bozic	0	last	wholeT_0.5	S1:OT-A
340	Yes	7	7-A	200	Bozic	0	unif	singleC	S1:CBN, S1:CBN-A, S1:OT, S1:OT-A
341	Yes	7	7-A	200	Bozic	0	unif	wholeT_0.01	J1:OT, J1:OT-A, S1:CBN, S1:CBN-A, S1:OT, S1:OT-A, S5:OT, S5:OT-A
342	Yes	7	7-A	200	Bozic	0	unif	wholeT_0.5	S1:CBN, S1:CBN-A, S1:OT, S1:OT-A
343	Yes	7	7-A	200	Bozic	Inf	last	singleC	J1:OT-A, J5:OT-A, S1:OT-A, S5:OT-A
344	Yes	7	7-A	200	Bozic	Inf	last	wholeT_0.01	J1:OT-A, J5:OT-A, S1:OT-A, S5:OT-A
345	Yes	7	7-A	200	Bozic	Inf	last	wholeT_0.5	J1:OT-A, J5:OT-A, S1:OT-A, S5:OT-A
346	Yes	7	7-A	200	Bozic	Inf	unif	singleC	S1:CBN, S1:CBN-A, S1:OT, S1:OT-A

Table 7: *(continued)*

	Conjunction	Drivers	Tree	S.Size	Model	sh	S.Time	S.Type	Best method(s)
347	Yes	7	7-A	200	Bozic	Inf	unif	wholeT_0.01	J1:CBN, J1:CBN-A, J1:OT, J1:OT-A, J5:CBN, J5:CBN-A, J5:OT, J5:OT-A, S1:CBN, S1:CBN-A, S1:OT, S1:OT-A, S5:CBN, S5:CBN-A, S5:OT, S5:OT-A
348	Yes	7	7-A	200	Bozic	Inf	unif	wholeT_0.5	S1:CBN, S1:CBN-A, S1:OT, S1:OT-A
349	Yes	7	7-A	200	exp	0	last	singleC	S1:OT-A
350	Yes	7	7-A	200	exp	0	last	wholeT_0.01	J1:OT-A, S1:OT-A, S5:OT-A
351	Yes	7	7-A	200	exp	0	last	wholeT_0.5	S1:OT-A
352	Yes	7	7-A	200	exp	0	unif	singleC	none
353	Yes	7	7-A	200	exp	0	unif	wholeT_0.01	S1:CBN, S1:CBN-A, S1:OT, S1:OT-A
354	Yes	7	7-A	200	exp	0	unif	wholeT_0.5	none
355	Yes	7	7-A	200	exp	Inf	last	singleC	J1:OT-A, S1:OT-A, S5:OT-A
356	Yes	7	7-A	200	exp	Inf	last	wholeT_0.01	J1:OT-A, J5:OT-A, S1:OT-A, S5:OT-A
357	Yes	7	7-A	200	exp	Inf	last	wholeT_0.5	S1:OT-A, S5:OT-A
358	Yes	7	7-A	200	exp	Inf	unif	singleC	S1:CBN, S1:CBN-A, S1:OT, S1:OT-A
359	Yes	7	7-A	200	exp	Inf	unif	wholeT_0.01	J1:CBN, J1:CBN-A, J1:OT, J1:OT-A, J5:CBN, J5:CBN-A, J5:OT, J5:OT-A, S1:CBN, S1:CBN-A, S1:OT, S1:OT-A, S5:CBN, S5:CBN-A, S5:OT, S5:OT-A
360	Yes	7	7-A	200	exp	Inf	unif	wholeT_0.5	S1:OT, S1:OT-A
361	Yes	7	7-A	200	McF_4	0	last	singleC	J1:OT-A, J5:OT-A, S1:OT-A, S5:OT-A
362	Yes	7	7-A	200	McF_4	0	last	wholeT_0.01	S1:OT-A
363	Yes	7	7-A	200	McF_4	0	last	wholeT_0.5	J1:OT-A, J5:OT-A, S1:OT-A, S5:OT-A
364	Yes	7	7-A	200	McF_4	0	unif	singleC	J1:OT, J1:OT-A, S1:CBN-A, S1:OT, S1:OT-A, S5:OT, S5:OT-A
365	Yes	7	7-A	200	McF_4	0	unif	wholeT_0.01	S1:OT, S1:OT-A
366	Yes	7	7-A	200	McF_4	0	unif	wholeT_0.5	J1:OT, J1:OT-A, S1:OT, S1:OT-A, S5:OT, S5:OT-A
367	Yes	7	7-A	200	McF_4	Inf	last	singleC	J1:OT-A, J5:OT-A, S1:OT-A, S5:OT-A
368	Yes	7	7-A	200	McF_4	Inf	last	wholeT_0.01	S1:OT-A
369	Yes	7	7-A	200	McF_4	Inf	last	wholeT_0.5	J1:OT-A, J5:OT-A, S1:OT-A, S5:OT-A
370	Yes	7	7-A	200	McF_4	Inf	unif	singleC	J1:OT, J1:OT-A, S1:OT, S1:OT-A, S5:OT, S5:OT-A
371	Yes	7	7-A	200	McF_4	Inf	unif	wholeT_0.01	J1:OT, J1:OT-A, S1:OT, S1:OT-A, S5:OT, S5:OT-A
372	Yes	7	7-A	200	McF_4	Inf	unif	wholeT_0.5	J1:OT, J1:OT-A, S1:OT, S1:OT-A, S5:OT, S5:OT-A
373	Yes	7	7-A	200	McF_6	0	last	singleC	S1:CBN, S1:OT-A, S5:CBN, S5:CBN-A, S5:OT-A

Table 7: *(continued)*

	Conjunction	Drivers	Tree	S.Size	Model	sh	S.Time	S.Type	Best method(s)
374	Yes	7	7-A	200	McF_6	0	last	wholeT_0.01	J1:OT-A, J5:CBN-A, S1:DiP-A, S1:OT-A, S5:OT-A
375	Yes	7	7-A	200	McF_6	0	last	wholeT_0.5	S1:CBN, S1:CBN-A, S1:OT-A, S5:CBN, S5:CBN-A, S5:OT-A
376	Yes	7	7-A	200	McF_6	0	unif	singleC	S1:CBN-A
377	Yes	7	7-A	200	McF_6	0	unif	wholeT_0.01	S1:CBN-A, S1:OT, S1:OT-A
378	Yes	7	7-A	200	McF_6	0	unif	wholeT_0.5	S1:CBN, S1:CBN-A, S1:OT, S1:OT-A
379	Yes	7	7-A	200	McF_6	Inf	last	singleC	J1:CBN-A, S1:CBN, S1:CBN-A, S1:OT-A, S5:CBN-A, S5:OT-A
380	Yes	7	7-A	200	McF_6	Inf	last	wholeT_0.01	J5:CBN-A, S1:OT-A, S5:OT-A
381	Yes	7	7-A	200	McF_6	Inf	last	wholeT_0.5	S1:CBN, S1:CBN-A, S1:OT-A, S5:CBN-A, S5:OT-A
382	Yes	7	7-A	200	McF_6	Inf	unif	singleC	S1:CBN-A
383	Yes	7	7-A	200	McF_6	Inf	unif	wholeT_0.01	S1:CBN-A
384	Yes	7	7-A	200	McF_6	Inf	unif	wholeT_0.5	S1:CBN, S1:CBN-A, S1:OT, S1:OT-A
385	Yes	7	7-A	100	Bozic	0	last	singleC	S1:OT-A
386	Yes	7	7-A	100	Bozic	0	last	wholeT_0.01	J1:OT-A, J5:OT-A, S1:OT-A, S5:OT-A
387	Yes	7	7-A	100	Bozic	0	last	wholeT_0.5	S1:OT-A
388	Yes	7	7-A	100	Bozic	0	unif	singleC	S1:CBN, S1:CBN-A, S1:OT, S1:OT-A
389	Yes	7	7-A	100	Bozic	0	unif	wholeT_0.01	S1:CBN, S1:CBN-A
390	Yes	7	7-A	100	Bozic	0	unif	wholeT_0.5	S1:CBN, S1:CBN-A, S1:OT, S1:OT-A
391	Yes	7	7-A	100	Bozic	Inf	last	singleC	J1:OT-A, S1:OT-A, S5:OT-A
392	Yes	7	7-A	100	Bozic	Inf	last	wholeT_0.01	J1:OT-A, J5:OT-A, S1:OT-A, S5:OT-A
393	Yes	7	7-A	100	Bozic	Inf	last	wholeT_0.5	J1:OT-A, J5:OT-A, S1:OT-A, S5:OT-A
394	Yes	7	7-A	100	Bozic	Inf	unif	singleC	S1:CBN, S1:CBN-A, S1:OT, S1:OT-A
395	Yes	7	7-A	100	Bozic	Inf	unif	wholeT_0.01	J1:CBN, J1:CBN-A, J1:OT, J1:OT-A, J5:CBN, J5:CBN-A, J5:OT, J5:OT-A, S1:CBN, S1:CBN-A, S1:OT, S1:OT-A, S5:CBN, S5:CBN-A, S5:OT, S5:OT-A
396	Yes	7	7-A	100	Bozic	Inf	unif	wholeT_0.5	S1:CBN, S1:CBN-A, S1:OT, S1:OT-A
397	Yes	7	7-A	100	exp	0	last	singleC	S1:OT-A
398	Yes	7	7-A	100	exp	0	last	wholeT_0.01	S1:OT-A, S5:OT-A
399	Yes	7	7-A	100	exp	0	last	wholeT_0.5	S1:OT-A
400	Yes	7	7-A	100	exp	0	unif	singleC	J1:OT, J1:OT-A, S1:CBN, S1:CBN-A, S1:OT, S1:OT-A
401	Yes	7	7-A	100	exp	0	unif	wholeT_0.01	S1:CBN, S1:CBN-A, S1:OT, S1:OT-A
402	Yes	7	7-A	100	exp	0	unif	wholeT_0.5	J1:CBN, J1:CBN-A, J1:OT, J1:OT-A, S1:CBN, S1:CBN-A, S1:OT, S1:OT-A
403	Yes	7	7-A	100	exp	Inf	last	singleC	S1:OT-A, S5:OT-A
404	Yes	7	7-A	100	exp	Inf	last	wholeT_0.01	J1:OT-A, J5:OT-A, S1:OT-A, S5:OT-A

Table 7: *(continued)*

	Conjunction	Drivers	Tree	S.Size	Model	sh	S.Time	S.Type	Best method(s)
405	Yes	7	7-A	100	exp	Inf	last	wholeT_0.5	S1:OT-A, S5:OT-A
406	Yes	7	7-A	100	exp	Inf	unif	singleC	S1:CBN, S1:OT, S1:OT-A
407	Yes	7	7-A	100	exp	Inf	unif	wholeT_0.01	J1:CBN, J1:CBN-A, J1:OT, J1:OT-A, J5:CBN, J5:CBN-A, J5:OT, J5:OT-A, S1:CBN, S1:CBN-A, S1:OT, S1:OT-A, S5:CBN, S5:CBN-A, S5:OT, S5:OT-A
408	Yes	7	7-A	100	exp	Inf	unif	wholeT_0.5	S1:CBN, S1:CBN-A, S1:OT, S1:OT-A
409	Yes	7	7-A	100	McF_4	0	last	singleC	S1:OT-A
410	Yes	7	7-A	100	McF_4	0	last	wholeT_0.01	S1:OT-A
411	Yes	7	7-A	100	McF_4	0	last	wholeT_0.5	S1:OT-A
412	Yes	7	7-A	100	McF_4	0	unif	singleC	J1:OT, J1:OT-A, S1:CBN-A, S1:OT, S1:OT-A, S5:OT, S5:OT-A
413	Yes	7	7-A	100	McF_4	0	unif	wholeT_0.01	S1:CBN-A, S1:OT, S1:OT-A
414	Yes	7	7-A	100	McF_4	0	unif	wholeT_0.5	J1:OT, J1:OT-A, S1:OT, S1:OT-A, S5:OT, S5:OT-A
415	Yes	7	7-A	100	McF_4	Inf	last	singleC	J1:OT-A, J5:OT-A, S1:OT-A, S5:OT-A
416	Yes	7	7-A	100	McF_4	Inf	last	wholeT_0.01	S1:OT-A
417	Yes	7	7-A	100	McF_4	Inf	last	wholeT_0.5	J1:OT-A, J5:OT-A, S1:OT-A, S5:OT-A
418	Yes	7	7-A	100	McF_4	Inf	unif	singleC	J1:OT, J1:OT-A, S1:OT, S1:OT-A, S5:OT, S5:OT-A
419	Yes	7	7-A	100	McF_4	Inf	unif	wholeT_0.01	J1:OT, J1:OT-A, S1:OT, S1:OT-A, S5:OT, S5:OT-A
420	Yes	7	7-A	100	McF_4	Inf	unif	wholeT_0.5	J1:OT, J1:OT-A, S1:OT, S1:OT-A, S5:OT, S5:OT-A
421	Yes	7	7-A	100	McF_6	0	last	singleC	S1:CBN, S1:CBN-A, S1:OT-A, S5:CBN, S5:CBN-A, S5:OT-A
422	Yes	7	7-A	100	McF_6	0	last	wholeT_0.01	S1:OT-A, S5:OT-A
423	Yes	7	7-A	100	McF_6	0	last	wholeT_0.5	S1:CBN, S1:CBN-A, S1:OT-A, S5:CBN, S5:CBN-A, S5:OT-A
424	Yes	7	7-A	100	McF_6	0	unif	singleC	S1:CBN, S1:CBN-A, S1:OT, S1:OT-A
425	Yes	7	7-A	100	McF_6	0	unif	wholeT_0.01	S1:CBN, S1:CBN-A, S1:OT, S1:OT-A, S5:CBN-A
426	Yes	7	7-A	100	McF_6	0	unif	wholeT_0.5	S1:CBN-A, S1:OT, S1:OT-A
427	Yes	7	7-A	100	McF_6	Inf	last	singleC	J1:CBN-A, S1:CBN, S1:CBN-A, S1:OT-A, S5:CBN-A, S5:OT-A
428	Yes	7	7-A	100	McF_6	Inf	last	wholeT_0.01	J1:CBN-A, J5:CBN-A, S1:OT-A, S5:OT-A
429	Yes	7	7-A	100	McF_6	Inf	last	wholeT_0.5	S1:CBN, S1:CBN-A, S1:OT-A, S5:CBN, S5:CBN-A, S5:OT-A
430	Yes	7	7-A	100	McF_6	Inf	unif	singleC	S1:CBN-A, S1:OT, S1:OT-A

Table 7: *(continued)*

	Conjunction	Drivers	Tree	S.Size	Model	sh	S.Time	S.Type	Best method(s)
431	Yes	7	7-A	100	McF_6	Inf	unif	wholeT_0.01	S1:CBN-A, S1:OT-A
432	Yes	7	7-A	100	McF_6	Inf	unif	wholeT_0.5	S1:CBN-A, S1:OT-A
433	No	11	11-B	1000	Bozic	0	last	singleC	S1:OT, S1:OT-A
434	No	11	11-B	1000	Bozic	0	last	wholeT_0.01	S1:DiP, S1:DiP-A, S1:OT, S1:OT-A, S5:DiP, S5:DiP-A, S5:OT, S5:OT-A
435	No	11	11-B	1000	Bozic	0	last	wholeT_0.5	S1:OT, S1:OT-A
436	No	11	11-B	1000	Bozic	0	unif	singleC	none
437	No	11	11-B	1000	Bozic	0	unif	wholeT_0.01	S1:CBN, S1:CBN-A
438	No	11	11-B	1000	Bozic	0	unif	wholeT_0.5	none
439	No	11	11-B	1000	Bozic	Inf	last	singleC	S1:OT, S1:OT-A
440	No	11	11-B	1000	Bozic	Inf	last	wholeT_0.01	J1:DiP, J1:DiP-A, J1:OT, J1:OT-A, S1:CBN-A, S1:DiP, S1:DiP-A, S1:OT, S1:OT-A, S5:DiP, S5:DiP-A, S5:OT, S5:OT-A
441	No	11	11-B	1000	Bozic	Inf	last	wholeT_0.5	S1:OT, S1:OT-A
442	No	11	11-B	1000	Bozic	Inf	unif	singleC	S1:CBN-A, S1:DiP-A, S1:OT, S1:OT-A
443	No	11	11-B	1000	Bozic	Inf	unif	wholeT_0.01	S1:CBN, S1:CBN-A, S1:DiP, S1:DiP-A, S1:OT, S1:OT-A, S5:CBN, S5:CBN-A, S5:DiP, S5:DiP-A, S5:OT, S5:OT-A
444	No	11	11-B	1000	Bozic	Inf	unif	wholeT_0.5	S1:CBN, S1:CBN-A, S1:OT, S1:OT-A
445	No	11	11-B	1000	exp	0	last	singleC	S1:CBN-A, S1:OT, S1:OT-A
446	No	11	11-B	1000	exp	0	last	wholeT_0.01	S1:DiP-A, S1:OT, S1:OT-A, S5:DiP-A, S5:OT, S5:OT-A
447	No	11	11-B	1000	exp	0	last	wholeT_0.5	S1:OT, S1:OT-A
448	No	11	11-B	1000	exp	0	unif	singleC	none
449	No	11	11-B	1000	exp	0	unif	wholeT_0.01	S1:CBN, S1:CBN-A, S1:OT, S1:OT-A
450	No	11	11-B	1000	exp	0	unif	wholeT_0.5	none
451	No	11	11-B	1000	exp	Inf	last	singleC	S1:CBN-A, S1:DiP, S1:DiP-A, S1:OT, S1:OT-A, S5:DiP, S5:DiP-A, S5:OT, S5:OT-A
452	No	11	11-B	1000	exp	Inf	last	wholeT_0.01	J1:DiP, J1:DiP-A, J1:OT, J1:OT-A, S1:DiP, S1:DiP-A, S1:OT, S1:OT-A, S5:DiP, S5:DiP-A, S5:OT, S5:OT-A
453	No	11	11-B	1000	exp	Inf	last	wholeT_0.5	S1:OT, S1:OT-A
454	No	11	11-B	1000	exp	Inf	unif	singleC	S1:CBN-A, S1:OT, S1:OT-A
455	No	11	11-B	1000	exp	Inf	unif	wholeT_0.01	S1:CBN, S1:CBN-A, S1:DiP-A, S1:OT, S1:OT-A
456	No	11	11-B	1000	exp	Inf	unif	wholeT_0.5	S1:CBN, S1:OT, S1:OT-A
457	No	11	11-B	1000	McF_4	0	last	singleC	S1:DiP-A, S1:OT, S1:OT-A
458	No	11	11-B	1000	McF_4	0	last	wholeT_0.01	S1:DiP, S1:DiP-A, S1:OT, S1:OT-A

Table 7: *(continued)*

	Conjunction	Drivers	Tree	S.Size	Model	sh	S.Time	S.Type	Best method(s)
459	No	11	11-B	1000	McF_4	0	last	wholeT_0.5	S1:DiP-A, S1:OT, S1:OT-A
460	No	11	11-B	1000	McF_4	0	unif	singleC	S1:DiP, S1:DiP-A, S1:OT, S1:OT-A
461	No	11	11-B	1000	McF_4	0	unif	wholeT_0.01	S1:CBN, S1:OT, S1:OT-A
462	No	11	11-B	1000	McF_4	0	unif	wholeT_0.5	S1:DiP, S1:DiP-A, S1:OT, S1:OT-A
463	No	11	11-B	1000	McF_4	Inf	last	singleC	S1:OT, S1:OT-A
464	No	11	11-B	1000	McF_4	Inf	last	wholeT_0.01	S1:DiP, S1:DiP-A, S1:OT, S1:OT-A, S5:DiP, S5:DiP-A, S5:OT, S5:OT-A
465	No	11	11-B	1000	McF_4	Inf	last	wholeT_0.5	S1:DiP-A, S1:OT, S1:OT-A
466	No	11	11-B	1000	McF_4	Inf	unif	singleC	S1:CBN-A, S1:OT, S1:OT-A
467	No	11	11-B	1000	McF_4	Inf	unif	wholeT_0.01	S1:OT, S1:OT-A
468	No	11	11-B	1000	McF_4	Inf	unif	wholeT_0.5	S1:CBN, S1:OT, S1:OT-A
469	No	11	11-B	1000	McF_6	0	last	singleC	S1:DiP, S1:DiP-A, S1:OT, S1:OT-A, S5:DiP, S5:DiP-A, S5:OT, S5:OT-A
470	No	11	11-B	1000	McF_6	0	last	wholeT_0.01	S1:DiP, S1:DiP-A, S1:OT, S1:OT-A, S5:DiP, S5:DiP-A, S5:OT, S5:OT-A
471	No	11	11-B	1000	McF_6	0	last	wholeT_0.5	S1:DiP, S1:DiP-A, S1:OT, S1:OT-A, S5:DiP, S5:DiP-A, S5:OT, S5:OT-A
472	No	11	11-B	1000	McF_6	0	unif	singleC	S1:DiP, S1:DiP-A, S1:OT, S1:OT-A
473	No	11	11-B	1000	McF_6	0	unif	wholeT_0.01	S1:DiP, S1:DiP-A, S1:OT, S1:OT-A
474	No	11	11-B	1000	McF_6	0	unif	wholeT_0.5	S1:DiP, S1:DiP-A, S1:OT, S1:OT-A
475	No	11	11-B	1000	McF_6	Inf	last	singleC	S1:DiP, S1:DiP-A, S1:OT, S1:OT-A, S5:DiP, S5:DiP-A, S5:OT, S5:OT-A
476	No	11	11-B	1000	McF_6	Inf	last	wholeT_0.01	S1:DiP-A, S1:OT, S1:OT-A, S5:DiP-A, S5:OT, S5:OT-A
477	No	11	11-B	1000	McF_6	Inf	last	wholeT_0.5	S1:DiP, S1:DiP-A, S1:OT, S1:OT-A, S5:DiP, S5:DiP-A, S5:OT, S5:OT-A
478	No	11	11-B	1000	McF_6	Inf	unif	singleC	S1:DiP, S1:DiP-A, S1:OT, S1:OT-A
479	No	11	11-B	1000	McF_6	Inf	unif	wholeT_0.01	S1:DiP, S1:DiP-A, S1:OT, S1:OT-A
480	No	11	11-B	1000	McF_6	Inf	unif	wholeT_0.5	S1:DiP, S1:DiP-A, S1:OT, S1:OT-A
481	No	11	11-B	200	Bozic	0	last	singleC	S1:OT, S1:OT-A
482	No	11	11-B	200	Bozic	0	last	wholeT_0.01	S1:OT, S1:OT-A
483	No	11	11-B	200	Bozic	0	last	wholeT_0.5	S1:CBN, S1:CBN-A, S1:OT, S1:OT-A
484	No	11	11-B	200	Bozic	0	unif	singleC	S1:CBN, S1:CBN-A, S1:OT, S1:OT-A
485	No	11	11-B	200	Bozic	0	unif	wholeT_0.01	S1:CBN, S1:CBN-A
486	No	11	11-B	200	Bozic	0	unif	wholeT_0.5	S1:CBN, S1:CBN-A, S1:OT, S1:OT-A
487	No	11	11-B	200	Bozic	Inf	last	singleC	S1:CBN, S1:OT, S1:OT-A
488	No	11	11-B	200	Bozic	Inf	last	wholeT_0.01	J1:OT, J1:OT-A, S1:OT, S1:OT-A, S5:OT, S5:OT-A
489	No	11	11-B	200	Bozic	Inf	last	wholeT_0.5	S1:OT, S1:OT-A
490	No	11	11-B	200	Bozic	Inf	unif	singleC	S1:CBN, S1:CBN-A, S1:OT, S1:OT-A

Table 7: *(continued)*

	Conjunction	Drivers	Tree	S.Size	Model	sh	S.Time	S.Type	Best method(s)
491	No	11	11-B	200	Bozic	Inf	unif	wholeT_0.01	S1:CBN, S1:CBN-A, S1:OT, S1:OT-A, S5:CBN, S5:CBN-A, S5:OT, S5:OT-A
492	No	11	11-B	200	Bozic	Inf	unif	wholeT_0.5	S1:CBN, S1:CBN-A, S1:OT, S1:OT-A
493	No	11	11-B	200	exp	0	last	singleC	S1:CBN-A, S1:OT, S1:OT-A
494	No	11	11-B	200	exp	0	last	wholeT_0.01	S1:CBN, S1:CBN-A, S1:OT, S1:OT-A
495	No	11	11-B	200	exp	0	last	wholeT_0.5	S1:CBN, S1:OT, S1:OT-A
496	No	11	11-B	200	exp	0	unif	singleC	none
497	No	11	11-B	200	exp	0	unif	wholeT_0.01	S1:CBN, S1:CBN-A, S1:OT, S1:OT-A
498	No	11	11-B	200	exp	0	unif	wholeT_0.5	none
499	No	11	11-B	200	exp	Inf	last	singleC	S1:OT, S1:OT-A
500	No	11	11-B	200	exp	Inf	last	wholeT_0.01	J1:OT, J1:OT-A, S1:CBN, S1:OT, S1:OT-A, S5:CBN-A, S5:OT, S5:OT-A
501	No	11	11-B	200	exp	Inf	last	wholeT_0.5	S1:OT, S1:OT-A
502	No	11	11-B	200	exp	Inf	unif	singleC	S1:CBN, S1:CBN-A, S1:OT, S1:OT-A
503	No	11	11-B	200	exp	Inf	unif	wholeT_0.01	S1:CBN, S1:CBN-A, S1:OT, S1:OT-A
504	No	11	11-B	200	exp	Inf	unif	wholeT_0.5	S1:CBN, S1:CBN-A, S1:OT, S1:OT-A
505	No	11	11-B	200	McF_4	0	last	singleC	S1:OT, S1:OT-A
506	No	11	11-B	200	McF_4	0	last	wholeT_0.01	S1:OT, S1:OT-A
507	No	11	11-B	200	McF_4	0	last	wholeT_0.5	S1:OT, S1:OT-A
508	No	11	11-B	200	McF_4	0	unif	singleC	S1:CBN-A, S1:OT, S1:OT-A
509	No	11	11-B	200	McF_4	0	unif	wholeT_0.01	S1:CBN, S1:CBN-A, S1:OT, S1:OT-A
510	No	11	11-B	200	McF_4	0	unif	wholeT_0.5	S1:OT, S1:OT-A
511	No	11	11-B	200	McF_4	Inf	last	singleC	S1:OT, S1:OT-A
512	No	11	11-B	200	McF_4	Inf	last	wholeT_0.01	S1:OT, S1:OT-A, S5:OT, S5:OT-A
513	No	11	11-B	200	McF_4	Inf	last	wholeT_0.5	S1:OT, S1:OT-A
514	No	11	11-B	200	McF_4	Inf	unif	singleC	S1:OT, S1:OT-A
515	No	11	11-B	200	McF_4	Inf	unif	wholeT_0.01	S1:OT, S1:OT-A
516	No	11	11-B	200	McF_4	Inf	unif	wholeT_0.5	S1:OT, S1:OT-A
517	No	11	11-B	200	McF_6	0	last	singleC	S1:OT, S1:OT-A, S5:OT, S5:OT-A
518	No	11	11-B	200	McF_6	0	last	wholeT_0.01	S1:OT, S1:OT-A, S5:OT, S5:OT-A
519	No	11	11-B	200	McF_6	0	last	wholeT_0.5	S1:OT, S1:OT-A, S5:OT, S5:OT-A
520	No	11	11-B	200	McF_6	0	unif	singleC	S1:OT, S1:OT-A
521	No	11	11-B	200	McF_6	0	unif	wholeT_0.01	S1:OT, S1:OT-A
522	No	11	11-B	200	McF_6	0	unif	wholeT_0.5	S1:OT, S1:OT-A
523	No	11	11-B	200	McF_6	Inf	last	singleC	S1:OT, S1:OT-A, S5:OT, S5:OT-A
524	No	11	11-B	200	McF_6	Inf	last	wholeT_0.01	S1:OT, S1:OT-A, S5:OT, S5:OT-A
525	No	11	11-B	200	McF_6	Inf	last	wholeT_0.5	S1:OT, S1:OT-A, S5:OT, S5:OT-A
526	No	11	11-B	200	McF_6	Inf	unif	singleC	S1:OT, S1:OT-A
527	No	11	11-B	200	McF_6	Inf	unif	wholeT_0.01	S1:OT, S1:OT-A
528	No	11	11-B	200	McF_6	Inf	unif	wholeT_0.5	S1:OT, S1:OT-A

Table 7: *(continued)*

	Conjunction	Drivers	Tree	S.Size	Model	sh	S.Time	S.Type	Best method(s)
529	No	11	11-B	100	Bozic	0	last	singleC	S1:CBN, S1:CBN-A, S1:OT, S1:OT-A
530	No	11	11-B	100	Bozic	0	last	wholeT_0.01	S1:CBN, S1:CBN-A, S1:OT, S1:OT-A
531	No	11	11-B	100	Bozic	0	last	wholeT_0.5	S1:CBN, S1:OT, S1:OT-A
532	No	11	11-B	100	Bozic	0	unif	singleC	S1:CBN, S1:CBN-A, S1:OT, S1:OT-A
533	No	11	11-B	100	Bozic	0	unif	wholeT_0.01	S1:CBN, S1:CBN-A
534	No	11	11-B	100	Bozic	0	unif	wholeT_0.5	S1:CBN, S1:CBN-A, S1:OT, S1:OT-A
535	No	11	11-B	100	Bozic	Inf	last	singleC	S1:CBN-A, S1:OT, S1:OT-A
536	No	11	11-B	100	Bozic	Inf	last	wholeT_0.01	J1:OT, J1:OT-A, S1:CBN-A, S1:OT, S1:OT-A, S5:OT, S5:OT-A
537	No	11	11-B	100	Bozic	Inf	last	wholeT_0.5	S1:OT, S1:OT-A
538	No	11	11-B	100	Bozic	Inf	unif	singleC	S1:CBN, S1:CBN-A, S1:OT, S1:OT-A
539	No	11	11-B	100	Bozic	Inf	unif	wholeT_0.01	J1:CBN, J1:CBN-A, J1:OT, J1:OT-A, S1:CBN, S1:CBN-A, S1:OT, S1:OT-A, S5:CBN, S5:OT, S5:OT-A
540	No	11	11-B	100	Bozic	Inf	unif	wholeT_0.5	S1:CBN, S1:CBN-A, S1:OT, S1:OT-A
541	No	11	11-B	100	exp	0	last	singleC	S1:OT, S1:OT-A
542	No	11	11-B	100	exp	0	last	wholeT_0.01	S1:CBN, S1:OT, S1:OT-A
543	No	11	11-B	100	exp	0	last	wholeT_0.5	S1:CBN-A, S1:OT, S1:OT-A
544	No	11	11-B	100	exp	0	unif	singleC	J1:CBN, J1:CBN-A, J1:OT, J1:OT-A, S1:CBN, S1:CBN-A, S1:OT, S1:OT-A
545	No	11	11-B	100	exp	0	unif	wholeT_0.01	S1:CBN, S1:CBN-A, S1:OT, S1:OT-A
546	No	11	11-B	100	exp	0	unif	wholeT_0.5	J1:CBN, J1:CBN-A, J1:OT, J1:OT-A, S1:CBN, S1:CBN-A, S1:OT, S1:OT-A
547	No	11	11-B	100	exp	Inf	last	singleC	S1:CBN, S1:OT, S1:OT-A
548	No	11	11-B	100	exp	Inf	last	wholeT_0.01	S1:OT, S1:OT-A, S5:CBN-A, S5:OT, S5:OT-A
549	No	11	11-B	100	exp	Inf	last	wholeT_0.5	S1:OT, S1:OT-A
550	No	11	11-B	100	exp	Inf	unif	singleC	S1:CBN, S1:OT, S1:OT-A
551	No	11	11-B	100	exp	Inf	unif	wholeT_0.01	S1:CBN, S1:CBN-A, S1:OT, S1:OT-A
552	No	11	11-B	100	exp	Inf	unif	wholeT_0.5	S1:CBN-A, S1:OT, S1:OT-A
553	No	11	11-B	100	McF_4	0	last	singleC	S1:OT, S1:OT-A
554	No	11	11-B	100	McF_4	0	last	wholeT_0.01	S1:OT, S1:OT-A, S5:OT, S5:OT-A
555	No	11	11-B	100	McF_4	0	last	wholeT_0.5	S1:OT, S1:OT-A
556	No	11	11-B	100	McF_4	0	unif	singleC	S1:CBN, S1:CBN-A, S1:OT, S1:OT-A
557	No	11	11-B	100	McF_4	0	unif	wholeT_0.01	S1:CBN, S1:CBN-A, S1:OT, S1:OT-A
558	No	11	11-B	100	McF_4	0	unif	wholeT_0.5	S1:CBN, S1:OT, S1:OT-A
559	No	11	11-B	100	McF_4	Inf	last	singleC	S1:OT, S1:OT-A
560	No	11	11-B	100	McF_4	Inf	last	wholeT_0.01	S1:OT, S1:OT-A, S5:OT, S5:OT-A
561	No	11	11-B	100	McF_4	Inf	last	wholeT_0.5	S1:OT, S1:OT-A
562	No	11	11-B	100	McF_4	Inf	unif	singleC	S1:OT, S1:OT-A

Table 7: *(continued)*

	Conjunction	Drivers	Tree	S.Size	Model	sh	S.Time	S.Type	Best method(s)
563	No	11	11-B	100	McF_4	Inf	unif	wholeT_0.01	S1:OT, S1:OT-A
564	No	11	11-B	100	McF_4	Inf	unif	wholeT_0.5	S1:OT, S1:OT-A
565	No	11	11-B	100	McF_6	0	last	singleC	S1:OT, S1:OT-A, S5:OT, S5:OT-A
566	No	11	11-B	100	McF_6	0	last	wholeT_0.01	S1:OT, S1:OT-A, S5:OT, S5:OT-A
567	No	11	11-B	100	McF_6	0	last	wholeT_0.5	S1:OT, S1:OT-A, S5:OT, S5:OT-A
568	No	11	11-B	100	McF_6	0	unif	singleC	S1:OT, S1:OT-A
569	No	11	11-B	100	McF_6	0	unif	wholeT_0.01	S1:OT, S1:OT-A
570	No	11	11-B	100	McF_6	0	unif	wholeT_0.5	S1:OT, S1:OT-A
571	No	11	11-B	100	McF_6	Inf	last	singleC	S1:OT, S1:OT-A, S5:OT, S5:OT-A
572	No	11	11-B	100	McF_6	Inf	last	wholeT_0.01	S1:OT, S1:OT-A, S5:OT, S5:OT-A
573	No	11	11-B	100	McF_6	Inf	last	wholeT_0.5	S1:OT, S1:OT-A, S5:OT, S5:OT-A
574	No	11	11-B	100	McF_6	Inf	unif	singleC	S1:OT, S1:OT-A
575	No	11	11-B	100	McF_6	Inf	unif	wholeT_0.01	S1:OT, S1:OT-A
576	No	11	11-B	100	McF_6	Inf	unif	wholeT_0.5	S1:OT, S1:OT-A
577	No	9	9-B	1000	Bozic	0	last	singleC	S1:CBN-A, S1:OT, S1:OT-A
578	No	9	9-B	1000	Bozic	0	last	wholeT_0.01	S1:CBN-A, S1:DiP, S1:DiP-A, S1:OT, S1:OT-A, S5:CBN-A, S5:DiP, S5:DiP-A, S5:OT, S5:OT-A
579	No	9	9-B	1000	Bozic	0	last	wholeT_0.5	S1:OT, S1:OT-A
580	No	9	9-B	1000	Bozic	0	unif	singleC	none
581	No	9	9-B	1000	Bozic	0	unif	wholeT_0.01	S1:CBN, S1:CBN-A, S1:OT, S1:OT-A
582	No	9	9-B	1000	Bozic	0	unif	wholeT_0.5	none
583	No	9	9-B	1000	Bozic	Inf	last	singleC	S1:CBN, S1:CBN-A, S1:DiP, S1:DiP-A, S1:OT, S1:OT-A, S5:DiP, S5:DiP-A, S5:OT, S5:OT-A
584	No	9	9-B	1000	Bozic	Inf	last	wholeT_0.01	J1:CBN, J1:CBN-A, J1:DiP, J1:DiP-A, J1:OT, J1:OT-A, S1:CBN, S1:CBN-A, S1:DiP, S1:DiP-A, S1:OT, S1:OT-A, S5:CBN-A, S5:DiP, S5:DiP-A, S5:OT, S5:OT-A
585	No	9	9-B	1000	Bozic	Inf	last	wholeT_0.5	S1:CBN, S1:CBN-A, S1:DiP, S1:DiP-A, S1:OT, S1:OT-A, S5:DiP, S5:DiP-A, S5:OT, S5:OT-A
586	No	9	9-B	1000	Bozic	Inf	unif	singleC	S1:CBN, S1:CBN-A, S1:OT, S1:OT-A
587	No	9	9-B	1000	Bozic	Inf	unif	wholeT_0.01	S1:CBN, S1:CBN-A, S1:DiP, S1:DiP-A, S1:OT, S1:OT-A, S5:CBN, S5:CBN-A, S5:DiP, S5:DiP-A, S5:OT, S5:OT-A
588	No	9	9-B	1000	Bozic	Inf	unif	wholeT_0.5	S1:CBN, S1:OT, S1:OT-A
589	No	9	9-B	1000	exp	0	last	singleC	S1:OT, S1:OT-A
590	No	9	9-B	1000	exp	0	last	wholeT_0.01	S1:CBN-A, S1:DiP-A, S1:OT, S1:OT-A

Table 7: *(continued)*

	Conjunction	Drivers	Tree	S.Size	Model	sh	S.Time	S.Type	Best method(s)
591	No	9	9-B	1000	exp	0	last	wholeT_0.5	none
592	No	9	9-B	1000	exp	0	unif	singleC	none
593	No	9	9-B	1000	exp	0	unif	wholeT_0.01	none
594	No	9	9-B	1000	exp	0	unif	wholeT_0.5	none
595	No	9	9-B	1000	exp	Inf	last	singleC	S1:DiP, S1:DiP-A, S1:OT, S1:OT-A, S5:DiP, S5:DiP-A, S5:OT, S5:OT-A
596	No	9	9-B	1000	exp	Inf	last	wholeT_0.01	J1:CBN-A, J1:DiP, J1:DiP-A, J1:OT, J1:OT-A, S1:CBN, S1:CBN-A, S1:DiP, S1:DiP-A, S1:OT, S1:OT-A, S5:CBN, S5:CBN-A, S5:DiP, S5:DiP-A, S5:OT, S5:OT-A
597	No	9	9-B	1000	exp	Inf	last	wholeT_0.5	S1:CBN, S1:DiP, S1:DiP-A, S1:OT, S1:OT-A, S5:DiP, S5:DiP-A, S5:OT, S5:OT-A
598	No	9	9-B	1000	exp	Inf	unif	singleC	S1:CBN, S1:CBN-A, S1:OT, S1:OT-A
599	No	9	9-B	1000	exp	Inf	unif	wholeT_0.01	S1:CBN, S1:CBN-A, S1:OT, S1:OT-A
600	No	9	9-B	1000	exp	Inf	unif	wholeT_0.5	S1:CBN, S1:CBN-A, S1:OT, S1:OT-A
601	No	9	9-B	1000	McF_4	0	last	singleC	S1:DiP, S1:DiP-A, S1:OT, S1:OT-A
602	No	9	9-B	1000	McF_4	0	last	wholeT_0.01	S1:DiP, S1:DiP-A, S1:OT, S1:OT-A, S5:DiP, S5:DiP-A, S5:OT, S5:OT-A
603	No	9	9-B	1000	McF_4	0	last	wholeT_0.5	S1:DiP-A, S1:OT, S1:OT-A
604	No	9	9-B	1000	McF_4	0	unif	singleC	S1:DiP, S1:DiP-A, S1:OT, S1:OT-A
605	No	9	9-B	1000	McF_4	0	unif	wholeT_0.01	S1:DiP-A, S1:OT, S1:OT-A
606	No	9	9-B	1000	McF_4	0	unif	wholeT_0.5	S1:DiP, S1:DiP-A, S1:OT, S1:OT-A
607	No	9	9-B	1000	McF_4	Inf	last	singleC	S1:OT, S1:OT-A
608	No	9	9-B	1000	McF_4	Inf	last	wholeT_0.01	S1:DiP, S1:DiP-A, S1:OT, S1:OT-A, S5:DiP, S5:DiP-A, S5:OT, S5:OT-A
609	No	9	9-B	1000	McF_4	Inf	last	wholeT_0.5	S1:OT, S1:OT-A
610	No	9	9-B	1000	McF_4	Inf	unif	singleC	S1:CBN-A, S1:DiP, S1:DiP-A, S1:OT, S1:OT-A
611	No	9	9-B	1000	McF_4	Inf	unif	wholeT_0.01	S1:OT, S1:OT-A
612	No	9	9-B	1000	McF_4	Inf	unif	wholeT_0.5	S1:CBN, S1:DiP, S1:DiP-A, S1:OT, S1:OT-A
613	No	9	9-B	1000	McF_6	0	last	singleC	S1:DiP-A, S1:OT-A, S5:DiP-A, S5:OT-A
614	No	9	9-B	1000	McF_6	0	last	wholeT_0.01	J1:DiP-A, J1:OT-A, S1:DiP-A, S1:OT-A, S5:DiP-A, S5:OT-A
615	No	9	9-B	1000	McF_6	0	last	wholeT_0.5	S1:DiP-A, S1:OT-A, S5:DiP-A, S5:OT-A
616	No	9	9-B	1000	McF_6	0	unif	singleC	S1:CBN, S1:DiP, S1:DiP-A, S1:OT, S1:OT-A

Table 7: *(continued)*

	Conjunction	Drivers	Tree	S.Size	Model	sh	S.Time	S.Type	Best method(s)
617	No	9	9-B	1000	McF_6	0	unif	wholeT_0.01	S1:DiP, S1:DiP-A, S1:OT, S1:OT-A, S5:DiP, S5:DiP-A, S5:OT, S5:OT-A
618	No	9	9-B	1000	McF_6	0	unif	wholeT_0.5	S1:DiP, S1:DiP-A, S1:OT, S1:OT-A
619	No	9	9-B	1000	McF_6	Inf	last	singleC	S1:DiP-A, S1:OT-A, S5:DiP-A, S5:OT-A
620	No	9	9-B	1000	McF_6	Inf	last	wholeT_0.01	S1:DiP-A, S1:OT-A, S5:DiP-A, S5:OT-A
621	No	9	9-B	1000	McF_6	Inf	last	wholeT_0.5	S1:DiP-A, S1:OT-A, S5:DiP-A, S5:OT-A
622	No	9	9-B	1000	McF_6	Inf	unif	singleC	S1:CBN, S1:CBN-A, S1:DiP, S1:DiP-A, S1:OT, S1:OT-A
623	No	9	9-B	1000	McF_6	Inf	unif	wholeT_0.01	S1:DiP, S1:DiP-A, S1:OT, S1:OT-A
624	No	9	9-B	1000	McF_6	Inf	unif	wholeT_0.5	S1:DiP, S1:DiP-A, S1:OT, S1:OT-A
625	No	9	9-B	200	Bozic	0	last	singleC	S1:CBN, S1:CBN-A, S1:OT, S1:OT-A
626	No	9	9-B	200	Bozic	0	last	wholeT_0.01	S1:CBN, S1:CBN-A, S1:OT, S1:OT-A, S5:OT, S5:OT-A
627	No	9	9-B	200	Bozic	0	last	wholeT_0.5	S1:OT, S1:OT-A
628	No	9	9-B	200	Bozic	0	unif	singleC	S1:CBN, S1:CBN-A, S1:OT, S1:OT-A
629	No	9	9-B	200	Bozic	0	unif	wholeT_0.01	S1:CBN, S1:CBN-A
630	No	9	9-B	200	Bozic	0	unif	wholeT_0.5	none
631	No	9	9-B	200	Bozic	Inf	last	singleC	S1:OT, S1:OT-A, S5:OT, S5:OT-A
632	No	9	9-B	200	Bozic	Inf	last	wholeT_0.01	J1:CBN-A, J1:OT, J1:OT-A, S1:CBN-A, S1:OT, S1:OT-A, S5:CBN-A, S5:OT, S5:OT-A
633	No	9	9-B	200	Bozic	Inf	last	wholeT_0.5	S1:CBN, S1:CBN-A, S1:OT, S1:OT-A, S5:OT, S5:OT-A
634	No	9	9-B	200	Bozic	Inf	unif	singleC	S1:CBN, S1:CBN-A, S1:OT, S1:OT-A
635	No	9	9-B	200	Bozic	Inf	unif	wholeT_0.01	S1:CBN, S1:CBN-A, S1:OT, S1:OT-A, S5:CBN, S5:CBN-A, S5:OT, S5:OT-A
636	No	9	9-B	200	Bozic	Inf	unif	wholeT_0.5	S1:CBN, S1:CBN-A, S1:OT, S1:OT-A
637	No	9	9-B	200	exp	0	last	singleC	S1:OT, S1:OT-A
638	No	9	9-B	200	exp	0	last	wholeT_0.01	S1:CBN, S1:OT, S1:OT-A
639	No	9	9-B	200	exp	0	last	wholeT_0.5	S1:CBN, S1:CBN-A, S1:OT, S1:OT-A
640	No	9	9-B	200	exp	0	unif	singleC	none
641	No	9	9-B	200	exp	0	unif	wholeT_0.01	S1:CBN, S1:CBN-A, S1:OT, S1:OT-A
642	No	9	9-B	200	exp	0	unif	wholeT_0.5	none
643	No	9	9-B	200	exp	Inf	last	singleC	S1:CBN, S1:CBN-A, S1:OT, S1:OT-A, S5:OT, S5:OT-A
644	No	9	9-B	200	exp	Inf	last	wholeT_0.01	J1:CBN, J1:CBN-A, J1:OT, J1:OT-A, S1:CBN, S1:CBN-A, S1:OT, S1:OT-A, S5:CBN-A, S5:OT, S5:OT-A
645	No	9	9-B	200	exp	Inf	last	wholeT_0.5	S1:OT, S1:OT-A, S5:OT, S5:OT-A
646	No	9	9-B	200	exp	Inf	unif	singleC	S1:CBN, S1:OT, S1:OT-A

Table 7: *(continued)*

	Conjunction	Drivers	Tree	S.Size	Model	sh	S.Time	S.Type	Best method(s)
647	No	9	9-B	200	exp	Inf	unif	wholeT_0.01	S1:CBN, S1:CBN-A, S1:OT, S1:OT-A
648	No	9	9-B	200	exp	Inf	unif	wholeT_0.5	S1:CBN, S1:CBN-A, S1:OT, S1:OT-A
649	No	9	9-B	200	McF_4	0	last	singleC	S1:OT, S1:OT-A
650	No	9	9-B	200	McF_4	0	last	wholeT_0.01	S1:OT, S1:OT-A, S5:OT, S5:OT-A
651	No	9	9-B	200	McF_4	0	last	wholeT_0.5	S1:OT, S1:OT-A
652	No	9	9-B	200	McF_4	0	unif	singleC	S1:CBN-A, S1:OT, S1:OT-A
653	No	9	9-B	200	McF_4	0	unif	wholeT_0.01	S1:CBN, S1:CBN-A, S1:OT, S1:OT-A
654	No	9	9-B	200	McF_4	0	unif	wholeT_0.5	S1:OT, S1:OT-A
655	No	9	9-B	200	McF_4	Inf	last	singleC	S1:OT, S1:OT-A
656	No	9	9-B	200	McF_4	Inf	last	wholeT_0.01	S1:OT, S1:OT-A
657	No	9	9-B	200	McF_4	Inf	last	wholeT_0.5	S1:OT, S1:OT-A
658	No	9	9-B	200	McF_4	Inf	unif	singleC	S1:CBN, S1:OT, S1:OT-A
659	No	9	9-B	200	McF_4	Inf	unif	wholeT_0.01	S1:CBN-A, S1:OT, S1:OT-A
660	No	9	9-B	200	McF_4	Inf	unif	wholeT_0.5	S1:OT, S1:OT-A
661	No	9	9-B	200	McF_6	0	last	singleC	S1:OT-A, S5:OT-A
662	No	9	9-B	200	McF_6	0	last	wholeT_0.01	J1:OT-A, S1:OT-A, S5:OT-A
663	No	9	9-B	200	McF_6	0	last	wholeT_0.5	S1:OT-A, S5:OT-A
664	No	9	9-B	200	McF_6	0	unif	singleC	S1:OT, S1:OT-A
665	No	9	9-B	200	McF_6	0	unif	wholeT_0.01	S1:OT, S1:OT-A
666	No	9	9-B	200	McF_6	0	unif	wholeT_0.5	S1:OT, S1:OT-A
667	No	9	9-B	200	McF_6	Inf	last	singleC	S1:OT-A, S5:OT-A
668	No	9	9-B	200	McF_6	Inf	last	wholeT_0.01	S1:OT-A, S5:OT-A
669	No	9	9-B	200	McF_6	Inf	last	wholeT_0.5	S1:OT-A, S5:OT-A
670	No	9	9-B	200	McF_6	Inf	unif	singleC	S1:OT, S1:OT-A
671	No	9	9-B	200	McF_6	Inf	unif	wholeT_0.01	S1:OT, S1:OT-A
672	No	9	9-B	200	McF_6	Inf	unif	wholeT_0.5	S1:OT, S1:OT-A
673	No	9	9-B	100	Bozic	0	last	singleC	S1:CBN, S1:CBN-A, S1:OT, S1:OT-A
674	No	9	9-B	100	Bozic	0	last	wholeT_0.01	S1:CBN-A, S1:OT, S1:OT-A
675	No	9	9-B	100	Bozic	0	last	wholeT_0.5	S1:CBN, S1:OT, S1:OT-A
676	No	9	9-B	100	Bozic	0	unif	singleC	S1:CBN, S1:CBN-A, S1:OT, S1:OT-A
677	No	9	9-B	100	Bozic	0	unif	wholeT_0.01	S1:CBN, S1:CBN-A
678	No	9	9-B	100	Bozic	0	unif	wholeT_0.5	J1:CBN, J1:CBN-A, J1:OT, J1:OT-A, S1:CBN, S1:CBN-A, S1:OT, S1:OT-A
679	No	9	9-B	100	Bozic	Inf	last	singleC	S1:CBN, S1:CBN-A, S1:OT, S1:OT-A, S5:OT, S5:OT-A
680	No	9	9-B	100	Bozic	Inf	last	wholeT_0.01	J1:OT, J1:OT-A, S1:OT, S1:OT-A, S5:OT, S5:OT-A
681	No	9	9-B	100	Bozic	Inf	last	wholeT_0.5	S1:OT, S1:OT-A
682	No	9	9-B	100	Bozic	Inf	unif	singleC	S1:CBN, S1:CBN-A, S1:OT, S1:OT-A

Table 7: *(continued)*

	Conjunction	Drivers	Tree	S.Size	Model	sh	S.Time	S.Type	Best method(s)
683	No	9	9-B	100	Bozic	Inf	unif	wholeT_0.01	S1:CBN, S1:CBN-A, S1:OT, S1:OT-A, S5:CBN
684	No	9	9-B	100	Bozic	Inf	unif	wholeT_0.5	S1:CBN, S1:CBN-A, S1:OT, S1:OT-A
685	No	9	9-B	100	exp	0	last	singleC	S1:OT, S1:OT-A
686	No	9	9-B	100	exp	0	last	wholeT_0.01	S1:CBN-A, S1:OT, S1:OT-A
687	No	9	9-B	100	exp	0	last	wholeT_0.5	S1:OT, S1:OT-A
688	No	9	9-B	100	exp	0	unif	singleC	none
689	No	9	9-B	100	exp	0	unif	wholeT_0.01	S1:CBN, S1:CBN-A, S1:OT, S1:OT-A
690	No	9	9-B	100	exp	0	unif	wholeT_0.5	none
691	No	9	9-B	100	exp	Inf	last	singleC	S1:CBN, S1:OT, S1:OT-A, S5:OT, S5:OT-A
692	No	9	9-B	100	exp	Inf	last	wholeT_0.01	J1:CBN-A, J1:OT, J1:OT-A, S1:CBN, S1:CBN-A, S1:OT, S1:OT-A, S5:CBN-A, S5:OT, S5:OT-A
693	No	9	9-B	100	exp	Inf	last	wholeT_0.5	S1:OT, S1:OT-A, S5:OT, S5:OT-A
694	No	9	9-B	100	exp	Inf	unif	singleC	S1:CBN, S1:CBN-A, S1:OT, S1:OT-A
695	No	9	9-B	100	exp	Inf	unif	wholeT_0.01	J1:CBN, J1:CBN-A, S1:CBN, S1:CBN-A, S1:OT, S1:OT-A
696	No	9	9-B	100	exp	Inf	unif	wholeT_0.5	S1:CBN, S1:CBN-A, S1:OT, S1:OT-A
697	No	9	9-B	100	McF_4	0	last	singleC	S1:OT, S1:OT-A
698	No	9	9-B	100	McF_4	0	last	wholeT_0.01	S1:OT, S1:OT-A, S5:OT, S5:OT-A
699	No	9	9-B	100	McF_4	0	last	wholeT_0.5	S1:OT, S1:OT-A
700	No	9	9-B	100	McF_4	0	unif	singleC	S1:CBN, S1:CBN-A, S1:OT, S1:OT-A
701	No	9	9-B	100	McF_4	0	unif	wholeT_0.01	S1:CBN, S1:CBN-A, S1:OT, S1:OT-A
702	No	9	9-B	100	McF_4	0	unif	wholeT_0.5	S1:OT, S1:OT-A
703	No	9	9-B	100	McF_4	Inf	last	singleC	S1:OT, S1:OT-A
704	No	9	9-B	100	McF_4	Inf	last	wholeT_0.01	S1:OT, S1:OT-A, S5:OT, S5:OT-A
705	No	9	9-B	100	McF_4	Inf	last	wholeT_0.5	S1:OT, S1:OT-A
706	No	9	9-B	100	McF_4	Inf	unif	singleC	S1:CBN-A, S1:OT, S1:OT-A
707	No	9	9-B	100	McF_4	Inf	unif	wholeT_0.01	S1:OT, S1:OT-A
708	No	9	9-B	100	McF_4	Inf	unif	wholeT_0.5	S1:OT, S1:OT-A
709	No	9	9-B	100	McF_6	0	last	singleC	S1:OT-A, S5:OT-A
710	No	9	9-B	100	McF_6	0	last	wholeT_0.01	J1:OT-A, S1:OT-A, S5:OT-A
711	No	9	9-B	100	McF_6	0	last	wholeT_0.5	S1:OT-A, S5:OT-A
712	No	9	9-B	100	McF_6	0	unif	singleC	S1:OT, S1:OT-A
713	No	9	9-B	100	McF_6	0	unif	wholeT_0.01	S1:OT, S1:OT-A
714	No	9	9-B	100	McF_6	0	unif	wholeT_0.5	S1:OT, S1:OT-A
715	No	9	9-B	100	McF_6	Inf	last	singleC	S1:OT-A, S5:OT-A
716	No	9	9-B	100	McF_6	Inf	last	wholeT_0.01	S1:OT-A, S5:OT-A
717	No	9	9-B	100	McF_6	Inf	last	wholeT_0.5	S1:OT-A, S5:OT-A

Table 7: *(continued)*

	Conjunction	Drivers	Tree	S.Size	Model	sh	S.Time	S.Type	Best method(s)
718	No	9	9-B	100	McF_6	Inf	unif	singleC	S1:OT, S1:OT-A
719	No	9	9-B	100	McF_6	Inf	unif	wholeT_0.01	S1:CBN, S1:OT, S1:OT-A
720	No	9	9-B	100	McF_6	Inf	unif	wholeT_0.5	S1:OT, S1:OT-A
721	No	7	7-B	1000	Bozic	0	last	singleC	S1:OT-A, S5:OT-A
722	No	7	7-B	1000	Bozic	0	last	wholeT_0.01	J1:DiP-A, J1:OT-A, J5:DiP-A, J5:OT-A, S1:DiP-A, S1:OT-A, S5:DiP-A, S5:OT-A
723	No	7	7-B	1000	Bozic	0	last	wholeT_0.5	S1:OT-A
724	No	7	7-B	1000	Bozic	0	unif	singleC	S1:OT, S1:OT-A
725	No	7	7-B	1000	Bozic	0	unif	wholeT_0.01	S1:CBN, S1:CBN-A, S1:DiP, S1:DiP-A, S1:OT, S1:OT-A
726	No	7	7-B	1000	Bozic	0	unif	wholeT_0.5	J1:CBN, J1:CBN-A, J1:OT, J1:OT-A, S1:CBN, S1:CBN-A, S1:OT, S1:OT-A
727	No	7	7-B	1000	Bozic	Inf	last	singleC	S1:DiP-A, S1:OT-A
728	No	7	7-B	1000	Bozic	Inf	last	wholeT_0.01	J1:DiP-A, J1:OT-A, S1:DiP-A, S1:OT-A, S5:DiP-A, S5:OT-A
729	No	7	7-B	1000	Bozic	Inf	last	wholeT_0.5	S1:DiP-A, S1:OT-A
730	No	7	7-B	1000	Bozic	Inf	unif	singleC	S1:CBN, S1:CBN-A, S1:OT, S1:OT-A
731	No	7	7-B	1000	Bozic	Inf	unif	wholeT_0.01	J1:CBN, J1:CBN-A, J1:OT, J1:OT-A, S1:CBN, S1:CBN-A, S1:OT, S1:OT-A
732	No	7	7-B	1000	Bozic	Inf	unif	wholeT_0.5	S1:CBN, S1:CBN-A, S1:OT, S1:OT-A
733	No	7	7-B	1000	exp	0	last	singleC	S1:OT-A
734	No	7	7-B	1000	exp	0	last	wholeT_0.01	J1:OT-A, S1:OT-A, S5:OT-A
735	No	7	7-B	1000	exp	0	last	wholeT_0.5	S1:CBN-A, S1:OT-A
736	No	7	7-B	1000	exp	0	unif	singleC	none
737	No	7	7-B	1000	exp	0	unif	wholeT_0.01	S1:CBN-A, S1:OT, S1:OT-A
738	No	7	7-B	1000	exp	0	unif	wholeT_0.5	none
739	No	7	7-B	1000	exp	Inf	last	singleC	S1:OT-A
740	No	7	7-B	1000	exp	Inf	last	wholeT_0.01	S1:OT-A, S5:OT-A
741	No	7	7-B	1000	exp	Inf	last	wholeT_0.5	S1:OT-A, S5:OT-A
742	No	7	7-B	1000	exp	Inf	unif	singleC	S1:CBN, S1:OT, S1:OT-A
743	No	7	7-B	1000	exp	Inf	unif	wholeT_0.01	J1:CBN, J1:CBN-A, J1:OT, J1:OT-A, J5:CBN, J5:CBN-A, J5:OT, J5:OT-A, S1:CBN, S1:CBN-A, S1:OT, S1:OT-A, S5:CBN, S5:CBN-A, S5:OT, S5:OT-A
744	No	7	7-B	1000	exp	Inf	unif	wholeT_0.5	S1:CBN, S1:CBN-A, S1:OT, S1:OT-A
745	No	7	7-B	1000	McF_4	0	last	singleC	S1:DiP-A, S1:OT-A, S5:DiP-A, S5:OT-A
746	No	7	7-B	1000	McF_4	0	last	wholeT_0.01	S1:DiP-A, S1:OT-A, S5:DiP-A, S5:OT-A
747	No	7	7-B	1000	McF_4	0	last	wholeT_0.5	S1:DiP-A, S1:OT-A, S5:DiP-A, S5:OT-A
748	No	7	7-B	1000	McF_4	0	unif	singleC	S1:DiP, S1:DiP-A, S1:OT, S1:OT-A
749	No	7	7-B	1000	McF_4	0	unif	wholeT_0.01	S1:DiP-A, S1:OT, S1:OT-A

Table 7: *(continued)*

	Conjunction	Drivers	Tree	S.Size	Model	sh	S.Time	S.Type	Best method(s)
750	No	7	7-B	1000	McF_4	0	unif	wholeT_0.5	S1:DiP-A, S1:OT, S1:OT-A
751	No	7	7-B	1000	McF_4	Inf	last	singleC	S1:DiP-A, S1:OT-A, S5:DiP-A, S5:OT-A
752	No	7	7-B	1000	McF_4	Inf	last	wholeT_0.01	S1:DiP-A, S1:OT-A, S5:DiP-A, S5:OT-A
753	No	7	7-B	1000	McF_4	Inf	last	wholeT_0.5	S1:DiP-A, S1:OT-A, S5:DiP-A, S5:OT-A
754	No	7	7-B	1000	McF_4	Inf	unif	singleC	S1:OT, S1:OT-A
755	No	7	7-B	1000	McF_4	Inf	unif	wholeT_0.01	S1:OT, S1:OT-A
756	No	7	7-B	1000	McF_4	Inf	unif	wholeT_0.5	S1:OT, S1:OT-A
757	No	7	7-B	1000	McF_6	0	last	singleC	J1:DiP-A, J1:OT-A, S1:DiP-A, S1:OT-A, S5:DiP-A, S5:OT-A
758	No	7	7-B	1000	McF_6	0	last	wholeT_0.01	J1:DiP-A, J1:OT-A, J5:DiP-A, J5:OT-A, S1:DiP-A, S1:OT-A, S5:DiP-A, S5:OT-A
759	No	7	7-B	1000	McF_6	0	last	wholeT_0.5	J1:DiP-A, J1:OT-A, S1:DiP-A, S1:OT-A, S5:DiP-A, S5:OT-A
760	No	7	7-B	1000	McF_6	0	unif	singleC	S1:DiP, S1:DiP-A, S1:OT, S1:OT-A, S5:DiP, S5:DiP-A, S5:OT, S5:OT-A
761	No	7	7-B	1000	McF_6	0	unif	wholeT_0.01	S1:DiP, S1:DiP-A, S1:OT, S1:OT-A, S5:DiP, S5:DiP-A, S5:OT, S5:OT-A
762	No	7	7-B	1000	McF_6	0	unif	wholeT_0.5	S1:DiP, S1:DiP-A, S1:OT, S1:OT-A, S5:DiP, S5:DiP-A, S5:OT, S5:OT-A
763	No	7	7-B	1000	McF_6	Inf	last	singleC	J1:DiP-A, J1:OT-A, S1:DiP-A, S1:OT-A, S5:DiP-A, S5:OT-A
764	No	7	7-B	1000	McF_6	Inf	last	wholeT_0.01	J1:DiP-A, J1:OT-A, J5:DiP-A, J5:OT-A, S1:DiP-A, S1:OT-A, S5:DiP-A, S5:OT-A
765	No	7	7-B	1000	McF_6	Inf	last	wholeT_0.5	J1:DiP-A, J1:OT-A, S1:DiP-A, S1:OT-A, S5:DiP-A, S5:OT-A
766	No	7	7-B	1000	McF_6	Inf	unif	singleC	S1:DiP-A, S1:OT, S1:OT-A, S5:DiP-A, S5:OT, S5:OT-A
767	No	7	7-B	1000	McF_6	Inf	unif	wholeT_0.01	S1:DiP-A, S1:OT, S1:OT-A, S5:DiP-A, S5:OT, S5:OT-A
768	No	7	7-B	1000	McF_6	Inf	unif	wholeT_0.5	S1:DiP-A, S1:OT, S1:OT-A, S5:DiP-A, S5:OT, S5:OT-A
769	No	7	7-B	200	Bozic	0	last	singleC	S1:OT-A, S5:OT-A
770	No	7	7-B	200	Bozic	0	last	wholeT_0.01	S1:OT-A
771	No	7	7-B	200	Bozic	0	last	wholeT_0.5	S1:OT-A
772	No	7	7-B	200	Bozic	0	unif	singleC	S1:CBN, S1:CBN-A, S1:OT, S1:OT-A
773	No	7	7-B	200	Bozic	0	unif	wholeT_0.01	J1:CBN, J1:CBN-A, J1:OT-A, S1:CBN, S1:CBN-A, S1:OT, S1:OT-A, S5:CBN, S5:CBN-A, S5:OT-A
774	No	7	7-B	200	Bozic	0	unif	wholeT_0.5	S1:CBN, S1:CBN-A, S1:OT, S1:OT-A
775	No	7	7-B	200	Bozic	Inf	last	singleC	S1:OT-A

Table 7: *(continued)*

	Conjunction	Drivers	Tree	S.Size	Model	sh	S.Time	S.Type	Best method(s)
776	No	7	7-B	200	Bozic	Inf	last	wholeT_0.01	J1:OT-A, S1:OT-A, S5:OT-A
777	No	7	7-B	200	Bozic	Inf	last	wholeT_0.5	S1:OT-A
778	No	7	7-B	200	Bozic	Inf	unif	singleC	S1:OT, S1:OT-A
779	No	7	7-B	200	Bozic	Inf	unif	wholeT_0.01	S1:CBN, S1:CBN-A, S1:OT, S1:OT-A
780	No	7	7-B	200	Bozic	Inf	unif	wholeT_0.5	S1:CBN, S1:OT, S1:OT-A
781	No	7	7-B	200	exp	0	last	singleC	S1:OT-A
782	No	7	7-B	200	exp	0	last	wholeT_0.01	S1:OT-A, S5:OT-A
783	No	7	7-B	200	exp	0	last	wholeT_0.5	S1:OT-A
784	No	7	7-B	200	exp	0	unif	singleC	J1:CBN, J1:CBN-A, J1:OT, J1:OT-A, S1:CBN, S1:CBN-A, S1:OT, S1:OT-A
785	No	7	7-B	200	exp	0	unif	wholeT_0.01	S1:CBN, S1:CBN-A, S1:OT, S1:OT-A
786	No	7	7-B	200	exp	0	unif	wholeT_0.5	none
787	No	7	7-B	200	exp	Inf	last	singleC	S1:OT-A
788	No	7	7-B	200	exp	Inf	last	wholeT_0.01	S1:OT-A, S5:OT-A
789	No	7	7-B	200	exp	Inf	last	wholeT_0.5	S1:OT-A
790	No	7	7-B	200	exp	Inf	unif	singleC	S1:CBN, S1:CBN-A, S1:OT, S1:OT-A
791	No	7	7-B	200	exp	Inf	unif	wholeT_0.01	J1:OT, J1:OT-A, S1:CBN, S1:CBN-A, S1:OT, S1:OT-A
792	No	7	7-B	200	exp	Inf	unif	wholeT_0.5	S1:CBN, S1:OT, S1:OT-A
793	No	7	7-B	200	McF_4	0	last	singleC	S1:OT-A, S5:OT-A
794	No	7	7-B	200	McF_4	0	last	wholeT_0.01	S1:OT-A, S5:OT-A
795	No	7	7-B	200	McF_4	0	last	wholeT_0.5	S1:OT-A, S5:OT-A
796	No	7	7-B	200	McF_4	0	unif	singleC	S1:OT, S1:OT-A
797	No	7	7-B	200	McF_4	0	unif	wholeT_0.01	S1:OT, S1:OT-A
798	No	7	7-B	200	McF_4	0	unif	wholeT_0.5	S1:OT, S1:OT-A
799	No	7	7-B	200	McF_4	Inf	last	singleC	S1:OT-A, S5:OT-A
800	No	7	7-B	200	McF_4	Inf	last	wholeT_0.01	S1:OT-A, S5:OT-A
801	No	7	7-B	200	McF_4	Inf	last	wholeT_0.5	S1:OT-A, S5:OT-A
802	No	7	7-B	200	McF_4	Inf	unif	singleC	S1:OT, S1:OT-A
803	No	7	7-B	200	McF_4	Inf	unif	wholeT_0.01	S1:OT, S1:OT-A
804	No	7	7-B	200	McF_4	Inf	unif	wholeT_0.5	S1:OT, S1:OT-A
805	No	7	7-B	200	McF_6	0	last	singleC	J1:OT-A, J5:OT-A, S1:OT-A, S5:OT-A
806	No	7	7-B	200	McF_6	0	last	wholeT_0.01	J1:DiP-A, J1:OT-A, J5:DiP-A, J5:OT-A, S1:DiP-A, S1:OT-A, S5:DiP-A, S5:OT-A
807	No	7	7-B	200	McF_6	0	last	wholeT_0.5	J1:OT-A, S1:OT-A, S5:OT-A
808	No	7	7-B	200	McF_6	0	unif	singleC	S1:OT, S1:OT-A, S5:OT, S5:OT-A
809	No	7	7-B	200	McF_6	0	unif	wholeT_0.01	J1:OT-A, S1:OT, S1:OT-A, S5:OT, S5:OT-A
810	No	7	7-B	200	McF_6	0	unif	wholeT_0.5	S1:OT, S1:OT-A, S5:OT, S5:OT-A
811	No	7	7-B	200	McF_6	Inf	last	singleC	J1:OT-A, J5:OT-A, S1:OT-A, S5:OT-A

Table 7: *(continued)*

	Conjunction	Drivers	Tree	S.Size	Model	sh	S.Time	S.Type	Best method(s)
812	No	7	7-B	200	McF_6	Inf	last	wholeT_0.01	J1:OT-A, J5:OT-A, S1:OT-A, S5:OT-A
813	No	7	7-B	200	McF_6	Inf	last	wholeT_0.5	J1:OT-A, S1:OT-A, S5:OT-A
814	No	7	7-B	200	McF_6	Inf	unif	singleC	S1:OT, S1:OT-A, S5:OT, S5:OT-A
815	No	7	7-B	200	McF_6	Inf	unif	wholeT_0.01	S1:OT, S1:OT-A, S5:OT, S5:OT-A
816	No	7	7-B	200	McF_6	Inf	unif	wholeT_0.5	J1:OT-A, S1:OT, S1:OT-A, S5:OT, S5:OT-A
817	No	7	7-B	100	Bozic	0	last	singleC	S1:OT-A
818	No	7	7-B	100	Bozic	0	last	wholeT_0.01	S1:OT-A
819	No	7	7-B	100	Bozic	0	last	wholeT_0.5	S1:OT-A
820	No	7	7-B	100	Bozic	0	unif	singleC	S1:CBN, S1:CBN-A, S1:OT, S1:OT-A
821	No	7	7-B	100	Bozic	0	unif	wholeT_0.01	S1:CBN, S1:CBN-A, S1:OT, S1:OT-A
822	No	7	7-B	100	Bozic	0	unif	wholeT_0.5	S1:CBN, S1:CBN-A, S1:OT, S1:OT-A
823	No	7	7-B	100	Bozic	Inf	last	singleC	S1:OT-A
824	No	7	7-B	100	Bozic	Inf	last	wholeT_0.01	S1:CBN-A, S1:OT-A, S5:OT-A
825	No	7	7-B	100	Bozic	Inf	last	wholeT_0.5	S1:OT-A
826	No	7	7-B	100	Bozic	Inf	unif	singleC	S1:CBN-A, S1:OT, S1:OT-A
827	No	7	7-B	100	Bozic	Inf	unif	wholeT_0.01	J1:CBN, J1:CBN-A, J1:OT, J1:OT-A, S1:CBN, S1:CBN-A, S1:OT, S1:OT-A
828	No	7	7-B	100	Bozic	Inf	unif	wholeT_0.5	S1:CBN-A, S1:OT, S1:OT-A
829	No	7	7-B	100	exp	0	last	singleC	S1:OT-A
830	No	7	7-B	100	exp	0	last	wholeT_0.01	S1:OT-A, S5:OT-A
831	No	7	7-B	100	exp	0	last	wholeT_0.5	S1:OT-A
832	No	7	7-B	100	exp	0	unif	singleC	J1:CBN, J1:CBN-A, J1:OT, J1:OT-A, S1:CBN, S1:CBN-A, S1:OT, S1:OT-A
833	No	7	7-B	100	exp	0	unif	wholeT_0.01	S1:CBN, S1:CBN-A, S1:OT, S1:OT-A
834	No	7	7-B	100	exp	0	unif	wholeT_0.5	J1:CBN, J1:CBN-A, J1:OT, J1:OT-A, S1:CBN, S1:CBN-A, S1:OT, S1:OT-A
835	No	7	7-B	100	exp	Inf	last	singleC	S1:OT-A
836	No	7	7-B	100	exp	Inf	last	wholeT_0.01	S1:OT-A, S5:OT-A
837	No	7	7-B	100	exp	Inf	last	wholeT_0.5	S1:OT-A
838	No	7	7-B	100	exp	Inf	unif	singleC	S1:CBN-A, S1:OT, S1:OT-A
839	No	7	7-B	100	exp	Inf	unif	wholeT_0.01	S1:CBN, S1:CBN-A, S1:OT, S1:OT-A
840	No	7	7-B	100	exp	Inf	unif	wholeT_0.5	S1:CBN, S1:CBN-A, S1:OT, S1:OT-A
841	No	7	7-B	100	McF_4	0	last	singleC	S1:OT-A, S5:OT-A
842	No	7	7-B	100	McF_4	0	last	wholeT_0.01	S1:OT-A, S5:OT-A
843	No	7	7-B	100	McF_4	0	last	wholeT_0.5	S1:OT-A
844	No	7	7-B	100	McF_4	0	unif	singleC	S1:OT, S1:OT-A
845	No	7	7-B	100	McF_4	0	unif	wholeT_0.01	S1:OT, S1:OT-A
846	No	7	7-B	100	McF_4	0	unif	wholeT_0.5	S1:OT, S1:OT-A
847	No	7	7-B	100	McF_4	Inf	last	singleC	S1:OT-A

Table 7: *(continued)*

	Conjunction	Drivers	Tree	S.Size	Model	sh	S.Time	S.Type	Best method(s)
848	No	7	7-B	100	McF_4	Inf	last	wholeT_0.01	S1:OT-A, S5:OT-A
849	No	7	7-B	100	McF_4	Inf	last	wholeT_0.5	S1:OT-A, S5:OT-A
850	No	7	7-B	100	McF_4	Inf	unif	singleC	S1:OT, S1:OT-A
851	No	7	7-B	100	McF_4	Inf	unif	wholeT_0.01	S1:OT, S1:OT-A
852	No	7	7-B	100	McF_4	Inf	unif	wholeT_0.5	S1:OT, S1:OT-A
853	No	7	7-B	100	McF_6	0	last	singleC	J1:OT-A, J5:OT-A, S1:OT-A, S5:OT-A
854	No	7	7-B	100	McF_6	0	last	wholeT_0.01	J1:OT-A, J5:OT-A, S1:OT-A, S5:OT-A
855	No	7	7-B	100	McF_6	0	last	wholeT_0.5	J1:OT-A, S1:OT-A, S5:OT-A
856	No	7	7-B	100	McF_6	0	unif	singleC	S1:OT, S1:OT-A, S5:OT, S5:OT-A
857	No	7	7-B	100	McF_6	0	unif	wholeT_0.01	J1:OT-A, S1:OT, S1:OT-A, S5:OT, S5:OT-A
858	No	7	7-B	100	McF_6	0	unif	wholeT_0.5	S1:OT, S1:OT-A, S5:OT, S5:OT-A
859	No	7	7-B	100	McF_6	Inf	last	singleC	J1:OT-A, S1:OT-A, S5:OT-A
860	No	7	7-B	100	McF_6	Inf	last	wholeT_0.01	J1:OT-A, J5:OT-A, S1:OT-A, S5:OT-A
861	No	7	7-B	100	McF_6	Inf	last	wholeT_0.5	J1:OT-A, S1:OT-A, S5:OT-A
862	No	7	7-B	100	McF_6	Inf	unif	singleC	S1:OT, S1:OT-A, S5:OT, S5:OT-A
863	No	7	7-B	100	McF_6	Inf	unif	wholeT_0.01	S1:OT-A, S5:OT-A
864	No	7	7-B	100	McF_6	Inf	unif	wholeT_0.5	S1:OT, S1:OT-A, S5:OT, S5:OT-A

3.4 Confidence sets (MCB-2), FPF, Drivers Unknown

Table 8: Confidence sets (method MCB-2) when Drivers are Unknown for measure FPF.

	Conjunction	Drivers	Tree	S.Size	Model	sh	S.Time	S.Type	Best method(s)
1	Yes	11	11-A	1000	Bozic	0	last	singleC	J1:DiP, J1:DiP-A, J1:OT, J1:OT-A, J5:DiP, J5:DiP-A, J5:OT, J5:OT-A, S1:OT, S1:OT-A, S5:OT, S5:OT-A
2	Yes	11	11-A	1000	Bozic	0	last	wholeT_0.01	J1:DiP, J1:DiP-A, J5:DiP, J5:DiP-A, S1:DiP, S1:DiP-A, S5:DiP, S5:DiP-A
3	Yes	11	11-A	1000	Bozic	0	last	wholeT_0.5	J1:DiP, J1:DiP-A, J1:OT, J1:OT-A, J5:DiP, J5:DiP-A, J5:OT, J5:OT-A, S1:OT, S1:OT-A, S5:DiP, S5:DiP-A, S5:OT, S5:OT-A
4	Yes	11	11-A	1000	Bozic	0	unif	singleC	J1:DiP, J1:DiP-A, J1:OT, J1:OT-A, J5:CBN, J5:CBN-A, J5:DiP, J5:DiP-A, J5:OT, J5:OT-A, S1:DiP, S1:DiP-A, S1:OT, S1:OT-A, S5:DiP, S5:DiP-A, S5:OT, S5:OT-A
5	Yes	11	11-A	1000	Bozic	0	unif	wholeT_0.01	J5:DiP, J5:DiP-A, J5:OT, J5:OT-A
6	Yes	11	11-A	1000	Bozic	0	unif	wholeT_0.5	J1:CBN, J1:CBN-A, J1:DiP, J1:DiP-A, J1:OT, J1:OT-A, J5:CBN, J5:CBN-A, J5:DiP, J5:DiP-A, J5:OT, J5:OT-A, S1:DiP, S1:DiP-A, S1:OT, S1:OT-A, S5:DiP, S5:DiP-A, S5:OT, S5:OT-A
7	Yes	11	11-A	1000	Bozic	Inf	last	singleC	J1:DiP, J1:DiP-A, J1:OT, J1:OT-A, J5:DiP, J5:DiP-A, J5:OT, J5:OT-A, S1:DiP-A, S5:DiP, S5:DiP-A, S5:OT, S5:OT-A
8	Yes	11	11-A	1000	Bozic	Inf	last	wholeT_0.01	J5:DiP, J5:DiP-A, J5:OT, J5:OT-A
9	Yes	11	11-A	1000	Bozic	Inf	last	wholeT_0.5	J1:DiP, J1:DiP-A, J1:OT, J1:OT-A, J5:DiP, J5:DiP-A, J5:OT, J5:OT-A, S5:DiP, S5:DiP-A, S5:OT, S5:OT-A
10	Yes	11	11-A	1000	Bozic	Inf	unif	singleC	J1:DiP, J1:DiP-A, J1:OT, J1:OT-A, J5:DiP, J5:DiP-A, J5:OT, J5:OT-A, S5:DiP, S5:DiP-A, S5:OT, S5:OT-A
11	Yes	11	11-A	1000	Bozic	Inf	unif	wholeT_0.01	J5:DiP, J5:DiP-A, J5:OT, J5:OT-A
12	Yes	11	11-A	1000	Bozic	Inf	unif	wholeT_0.5	J1:DiP, J1:DiP-A, J1:OT, J1:OT-A, J5:DiP, J5:DiP-A, J5:OT, J5:OT-A, S1:CBN, S5:DiP, S5:DiP-A, S5:OT, S5:OT-A

Table 8: *(continued)*

	Conjunction	Drivers	Tree	S.Size	Model	sh	S.Time	S.Type	Best method(s)
13	Yes	11	11-A	1000	exp	0	last	singleC	J1:DiP, J1:DiP-A, J1:OT, J1:OT-A, J5:CBN, J5:CBN-A, J5:DiP, J5:DiP-A, J5:OT, J5:OT-A, S1:DiP, S1:DiP-A, S1:OT, S1:OT-A, S5:DiP, S5:DiP-A, S5:OT, S5:OT-A
14	Yes	11	11-A	1000	exp	0	last	wholeT_0.01	J1:DiP, J1:DiP-A, J1:OT, J1:OT-A, J5:DiP, J5:DiP-A, J5:OT, J5:OT-A, S1:DiP-A, S1:OT, S1:OT-A, S5:DiP-A, S5:OT, S5:OT-A
15	Yes	11	11-A	1000	exp	0	last	wholeT_0.5	J1:DiP, J1:DiP-A, J1:OT, J1:OT-A, J5:CBN, J5:CBN-A, J5:DiP, J5:DiP-A, J5:OT, J5:OT-A, S1:DiP, S1:DiP-A, S1:OT, S1:OT-A, S5:DiP, S5:DiP-A, S5:OT, S5:OT-A
16	Yes	11	11-A	1000	exp	0	unif	singleC	J1:CBN, J1:CBN-A, J1:DiP, J1:DiP-A, J1:OT, J1:OT-A, J5:CBN, J5:CBN-A, J5:DiP, J5:DiP-A, J5:OT, J5:OT-A, S1:DiP, S1:DiP-A, S1:OT, S1:OT-A, S5:DiP, S5:DiP-A, S5:OT, S5:OT-A
17	Yes	11	11-A	1000	exp	0	unif	wholeT_0.01	J1:DiP, J1:DiP-A, J1:OT, J1:OT-A, J5:CBN, J5:CBN-A, J5:DiP, J5:DiP-A, J5:OT, J5:OT-A, S1:DiP, S1:DiP-A, S5:DiP, S5:DiP-A, S5:OT, S5:OT-A
18	Yes	11	11-A	1000	exp	0	unif	wholeT_0.5	J1:CBN, J1:CBN-A, J1:DiP, J1:DiP-A, J1:OT, J1:OT-A, J5:CBN, J5:CBN-A, J5:DiP, J5:DiP-A, J5:OT, J5:OT-A, S1:DiP, S1:DiP-A, S1:OT, S1:OT-A, S5:DiP, S5:DiP-A, S5:OT, S5:OT-A
19	Yes	11	11-A	1000	exp	Inf	last	singleC	J1:DiP, J1:DiP-A, J1:OT, J1:OT-A, J5:DiP, J5:DiP-A, J5:OT, J5:OT-A, S5:DiP, S5:DiP-A, S5:OT, S5:OT-A
20	Yes	11	11-A	1000	exp	Inf	last	wholeT_0.01	J5:DiP, J5:DiP-A, J5:OT, J5:OT-A, S5:DiP, S5:DiP-A, S5:OT, S5:OT-A
21	Yes	11	11-A	1000	exp	Inf	last	wholeT_0.5	J1:DiP, J1:DiP-A, J1:OT, J1:OT-A, J5:DiP, J5:DiP-A, J5:OT, J5:OT-A, S1:DiP-A, S5:DiP, S5:DiP-A, S5:OT, S5:OT-A

Table 8: *(continued)*

	Conjunction	Drivers	Tree	S.Size	Model	sh	S.Time	S.Type	Best method(s)
22	Yes	11	11-A	1000	exp	Inf	unif	singleC	J1:DiP, J1:DiP-A, J1:OT, J1:OT-A, J5:CBN, J5:CBN-A, J5:DiP, J5:DiP-A, J5:OT, J5:OT-A, S5:DiP, S5:DiP-A, S5:OT, S5:OT-A
23	Yes	11	11-A	1000	exp	Inf	unif	wholeT_0.01	J5:DiP, J5:DiP-A, J5:OT, J5:OT-A, S5:DiP, S5:DiP-A, S5:OT, S5:OT-A
24	Yes	11	11-A	1000	exp	Inf	unif	wholeT_0.5	J1:DiP, J1:DiP-A, J1:OT, J1:OT-A, J5:CBN, J5:CBN-A, J5:DiP, J5:DiP-A, J5:OT, J5:OT-A, S5:DiP, S5:DiP-A, S5:OT, S5:OT-A
25	Yes	11	11-A	1000	McF_4	0	last	singleC	J1:CBN, J1:DiP, J1:DiP-A, J1:OT, J1:OT-A, J5:CBN, J5:DiP, J5:DiP-A, J5:OT, J5:OT-A, S1:DiP-A, S1:OT, S1:OT-A, S5:DiP, S5:DiP-A, S5:OT, S5:OT-A
26	Yes	11	11-A	1000	McF_4	0	last	wholeT_0.01	J1:DiP, J1:DiP-A, J1:OT, J1:OT-A, J5:CBN, J5:DiP, J5:DiP-A, J5:OT, J5:OT-A, S1:DiP-A, S5:DiP, S5:DiP-A, S5:OT, S5:OT-A
27	Yes	11	11-A	1000	McF_4	0	last	wholeT_0.5	J1:DiP, J1:DiP-A, J1:OT, J1:OT-A, J5:CBN, J5:DiP, J5:DiP-A, J5:OT, J5:OT-A, S1:DiP-A, S1:OT, S1:OT-A, S5:DiP, S5:DiP-A, S5:OT, S5:OT-A
28	Yes	11	11-A	1000	McF_4	0	unif	singleC	J1:CBN, J1:CBN-A, J1:DiP, J1:DiP-A, J1:OT, J1:OT-A, J5:DiP, J5:DiP-A, J5:OT, J5:OT-A, S1:DiP, S1:DiP-A, S1:OT, S1:OT-A, S5:DiP, S5:DiP-A, S5:OT, S5:OT-A
29	Yes	11	11-A	1000	McF_4	0	unif	wholeT_0.01	J1:CBN-A, J1:DiP, J1:DiP-A, J1:OT, J1:OT-A, J5:DiP, J5:DiP-A, J5:OT, J5:OT-A, S1:DiP-A, S5:CBN-A, S5:DiP, S5:DiP-A, S5:OT, S5:OT-A
30	Yes	11	11-A	1000	McF_4	0	unif	wholeT_0.5	J1:CBN, J1:CBN-A, J1:DiP, J1:DiP-A, J1:OT, J1:OT-A, J5:DiP, J5:DiP-A, J5:OT, J5:OT-A, S1:DiP, S1:DiP-A, S1:OT, S1:OT-A, S5:CBN-A, S5:DiP, S5:DiP-A, S5:OT, S5:OT-A

Table 8: *(continued)*

	Conjunction	Drivers	Tree	S.Size	Model	sh	S.Time	S.Type	Best method(s)
31	Yes	11	11-A	1000	McF_4	Inf	last	singleC	J1:DiP, J1:DiP-A, J1:OT, J1:OT-A, J5:DiP, J5:DiP-A, J5:OT, J5:OT-A, S1:OT, S1:OT-A, S5:DiP, S5:DiP-A, S5:OT, S5:OT-A
32	Yes	11	11-A	1000	McF_4	Inf	last	wholeT_0.01	J1:DiP, J1:DiP-A, J1:OT, J1:OT-A, J5:DiP, J5:DiP-A, J5:OT, J5:OT-A, S5:DiP, S5:DiP-A, S5:OT, S5:OT-A
33	Yes	11	11-A	1000	McF_4	Inf	last	wholeT_0.5	J1:DiP, J1:DiP-A, J1:OT, J1:OT-A, J5:DiP, J5:DiP-A, J5:OT, J5:OT-A, S1:DiP-A, S1:OT, S1:OT-A, S5:DiP, S5:DiP-A, S5:OT, S5:OT-A
34	Yes	11	11-A	1000	McF_4	Inf	unif	singleC	J1:CBN-A, J1:DiP, J1:DiP-A, J1:OT, J1:OT-A, J5:DiP, J5:DiP-A, J5:OT, J5:OT-A, S1:DiP, S1:DiP-A, S1:OT, S1:OT-A, S5:CBN-A, S5:DiP, S5:DiP-A, S5:OT, S5:OT-A
35	Yes	11	11-A	1000	McF_4	Inf	unif	wholeT_0.01	J1:CBN-A, J1:DiP, J1:DiP-A, J1:OT, J1:OT-A, J5:DiP, J5:DiP-A, J5:OT, J5:OT-A, S5:CBN-A, S5:DiP, S5:DiP-A, S5:OT, S5:OT-A
36	Yes	11	11-A	1000	McF_4	Inf	unif	wholeT_0.5	J1:CBN-A, J1:DiP, J1:DiP-A, J1:OT, J1:OT-A, J5:DiP, J5:DiP-A, J5:OT, J5:OT-A, S1:DiP, S1:DiP-A, S1:OT, S1:OT-A, S5:CBN-A, S5:DiP, S5:DiP-A, S5:OT, S5:OT-A
37	Yes	11	11-A	1000	McF_6	0	last	singleC	J1:DiP, J1:OT, J5:DiP, J5:OT, S1:DiP, S1:OT, S5:DiP, S5:OT
38	Yes	11	11-A	1000	McF_6	0	last	wholeT_0.01	J1:DiP, J1:OT, J5:DiP, J5:OT, S1:DiP, S5:DiP, S5:OT
39	Yes	11	11-A	1000	McF_6	0	last	wholeT_0.5	J1:DiP, J1:OT, J5:DiP, J5:OT, S1:DiP, S1:OT, S5:DiP, S5:OT
40	Yes	11	11-A	1000	McF_6	0	unif	singleC	J1:CBN, J1:CBN-A, J1:DiP, J1:DiP-A, J1:OT, J1:OT-A, J5:CBN, J5:CBN-A, J5:DiP, J5:DiP-A, J5:OT, J5:OT-A, S1:DiP, S1:DiP-A, S1:OT, S1:OT-A, S5:CBN, S5:CBN-A, S5:DiP, S5:DiP-A, S5:OT, S5:OT-A

Table 8: *(continued)*

	Conjunction	Drivers	Tree	S.Size	Model	sh	S.Time	S.Type	Best method(s)
41	Yes	11	11-A	1000	McF_6	0	unif	wholeT_0.01	J1:CBN, J1:CBN-A, J1:DiP, J1:DiP-A, J1:OT, J1:OT-A, J5:CBN, J5:CBN-A, J5:DiP, J5:DiP-A, J5:OT, J5:OT-A, S1:DiP, S1:DiP-A, S5:CBN, S5:CBN-A, S5:DiP, S5:DiP-A, S5:OT, S5:OT-A
42	Yes	11	11-A	1000	McF_6	0	unif	wholeT_0.5	J1:CBN, J1:CBN-A, J1:DiP, J1:DiP-A, J1:OT, J1:OT-A, J5:CBN, J5:CBN-A, J5:DiP, J5:DiP-A, J5:OT, J5:OT-A, S1:DiP, S1:DiP-A, S1:OT, S1:OT-A, S5:CBN, S5:CBN-A, S5:DiP, S5:DiP-A, S5:OT, S5:OT-A
43	Yes	11	11-A	1000	McF_6	Inf	last	singleC	J1:DiP, J1:OT, J5:DiP, J5:OT, S1:DiP, S5:DiP, S5:OT
44	Yes	11	11-A	1000	McF_6	Inf	last	wholeT_0.01	J1:DiP, J1:OT, J5:DiP, J5:OT, S1:DiP, S5:DiP, S5:OT
45	Yes	11	11-A	1000	McF_6	Inf	last	wholeT_0.5	J1:DiP, J1:OT, J5:DiP, J5:OT, S1:DiP, S5:DiP, S5:OT
46	Yes	11	11-A	1000	McF_6	Inf	unif	singleC	J1:CBN, J1:CBN-A, J1:DiP, J1:DiP-A, J1:OT, J1:OT-A, J5:CBN-A, J5:DiP, J5:DiP-A, J5:OT, J5:OT-A, S1:DiP, S1:DiP-A, S5:CBN-A, S5:DiP, S5:DiP-A, S5:OT, S5:OT-A
47	Yes	11	11-A	1000	McF_6	Inf	unif	wholeT_0.01	J1:CBN, J1:CBN-A, J1:DiP, J1:DiP-A, J1:OT, J1:OT-A, J5:CBN-A, J5:DiP, J5:DiP-A, J5:OT, J5:OT-A, S1:DiP, S1:DiP-A, S5:CBN, S5:CBN-A, S5:DiP, S5:DiP-A, S5:OT, S5:OT-A
48	Yes	11	11-A	1000	McF_6	Inf	unif	wholeT_0.5	J1:CBN, J1:CBN-A, J1:DiP, J1:DiP-A, J5:CBN-A, J5:DiP, J5:DiP-A, J5:OT, J5:OT-A, S1:DiP, S1:DiP-A, S5:CBN-A, S5:DiP, S5:DiP-A, S5:OT, S5:OT-A
49	Yes	11	11-A	200	Bozic	0	last	singleC	J1:DiP, J1:DiP-A, J1:OT, J1:OT-A, J5:DiP, J5:DiP-A, J5:OT, J5:OT-A, S1:DiP, S1:DiP-A, S5:DiP, S5:DiP-A, S5:OT, S5:OT-A
50	Yes	11	11-A	200	Bozic	0	last	wholeT_0.01	J1:DiP, J1:DiP-A, J1:OT, J1:OT-A, J5:DiP, J5:DiP-A, J5:OT, J5:OT-A, S5:DiP

Table 8: *(continued)*

	Conjunction	Drivers	Tree	S.Size	Model	sh	S.Time	S.Type	Best method(s)
51	Yes	11	11-A	200	Bozic	0	last	wholeT_0.5	J1:DiP, J1:DiP-A, J1:OT, J1:OT-A, J5:DiP, J5:DiP-A, J5:OT, J5:OT-A, S1:DiP, S1:DiP-A, S5:DiP, S5:DiP-A, S5:OT, S5:OT-A
52	Yes	11	11-A	200	Bozic	0	unif	singleC	J1:DiP, J1:DiP-A, J1:OT, J1:OT-A, J5:CBN, J5:CBN-A, J5:DiP, J5:DiP-A, J5:OT, J5:OT-A, S1:DiP, S1:DiP-A, S1:OT, S1:OT-A, S5:DiP, S5:DiP-A, S5:OT, S5:OT-A
53	Yes	11	11-A	200	Bozic	0	unif	wholeT_0.01	J5:DiP, J5:DiP-A, J5:OT, J5:OT-A
54	Yes	11	11-A	200	Bozic	0	unif	wholeT_0.5	J1:DiP, J1:DiP-A, J1:OT, J1:OT-A, J5:CBN, J5:CBN-A, J5:DiP, J5:DiP-A, J5:OT, J5:OT-A, S1:DiP, S1:DiP-A, S1:OT, S1:OT-A, S5:DiP, S5:DiP-A, S5:OT, S5:OT-A
55	Yes	11	11-A	200	Bozic	Inf	last	singleC	J1:DiP, J1:DiP-A, J5:DiP, J5:DiP-A, J5:OT, J5:OT-A, S5:DiP, S5:DiP-A, S5:OT, S5:OT-A
56	Yes	11	11-A	200	Bozic	Inf	last	wholeT_0.01	J5:DiP, J5:DiP-A, J5:OT, J5:OT-A
57	Yes	11	11-A	200	Bozic	Inf	last	wholeT_0.5	J1:DiP, J1:DiP-A, J5:DiP, J5:DiP-A, J5:OT, J5:OT-A, S5:DiP, S5:DiP-A, S5:OT, S5:OT-A
58	Yes	11	11-A	200	Bozic	Inf	unif	singleC	J1:DiP, J1:DiP-A, J1:OT, J1:OT-A, J5:DiP, J5:DiP-A, J5:OT, J5:OT-A, S5:DiP, S5:DiP-A, S5:OT, S5:OT-A
59	Yes	11	11-A	200	Bozic	Inf	unif	wholeT_0.01	J5:DiP, J5:DiP-A, J5:OT, J5:OT-A
60	Yes	11	11-A	200	Bozic	Inf	unif	wholeT_0.5	J1:CBN-A, J1:DiP, J1:DiP-A, J1:OT, J1:OT-A, J5:DiP, J5:DiP-A, J5:OT, J5:OT-A, S5:DiP, S5:DiP-A, S5:OT, S5:OT-A
61	Yes	11	11-A	200	exp	0	last	singleC	J1:DiP, J1:DiP-A, J1:OT, J1:OT-A, J5:CBN, J5:CBN-A, J5:DiP, J5:DiP-A, J5:OT, J5:OT-A, S1:DiP, S1:DiP-A, S1:OT, S1:OT-A, S5:DiP, S5:DiP-A, S5:OT, S5:OT-A
62	Yes	11	11-A	200	exp	0	last	wholeT_0.01	J1:DiP, J1:DiP-A, J1:OT, J1:OT-A, J5:DiP, J5:DiP-A, J5:OT, J5:OT-A, S5:DiP, S5:OT, S5:OT-A

Table 8: *(continued)*

	Conjunction	Drivers	Tree	S.Size	Model	sh	S.Time	S.Type	Best method(s)
63	Yes	11	11-A	200	exp	0	last	wholeT_0.5	J1:DiP, J1:DiP-A, J1:OT, J1:OT-A, J5:CBN, J5:CBN-A, J5:DiP, J5:DiP-A, J5:OT, J5:OT-A, S1:DiP, S1:DiP-A, S1:OT, S1:OT-A, S5:DiP, S5:DiP-A, S5:OT, S5:OT-A
64	Yes	11	11-A	200	exp	0	unif	singleC	J1:CBN, J1:CBN-A, J1:DiP, J1:DiP-A, J1:OT, J1:OT-A, J5:CBN, J5:CBN-A, J5:DiP, J5:DiP-A, J5:OT, J5:OT-A, S1:DiP, S1:DiP-A, S1:OT, S1:OT-A, S5:DiP, S5:DiP-A, S5:OT, S5:OT-A
65	Yes	11	11-A	200	exp	0	unif	wholeT_0.01	J1:DiP, J1:DiP-A, J1:OT, J1:OT-A, J5:CBN, J5:CBN-A, J5:DiP, J5:DiP-A, J5:OT, J5:OT-A, S1:DiP, S1:DiP-A, S1:OT, S1:OT-A, S5:DiP, S5:DiP-A, S5:OT, S5:OT-A
66	Yes	11	11-A	200	exp	0	unif	wholeT_0.5	J1:CBN, J1:CBN-A, J1:DiP, J1:DiP-A, J1:OT, J1:OT-A, J5:CBN, J5:CBN-A, J5:DiP, J5:DiP-A, J5:OT, J5:OT-A, S1:DiP, S1:DiP-A, S1:OT, S1:OT-A, S5:DiP, S5:DiP-A, S5:OT, S5:OT-A
67	Yes	11	11-A	200	exp	Inf	last	singleC	J1:DiP, J1:DiP-A, J1:OT, J1:OT-A, J5:DiP, J5:DiP-A, J5:OT, J5:OT-A, S1:DiP, S1:DiP-A, S5:DiP, S5:DiP-A, S5:OT, S5:OT-A
68	Yes	11	11-A	200	exp	Inf	last	wholeT_0.01	J5:DiP, J5:DiP-A, J5:OT, J5:OT-A
69	Yes	11	11-A	200	exp	Inf	last	wholeT_0.5	J1:DiP, J1:DiP-A, J1:OT, J1:OT-A, J5:DiP, J5:DiP-A, J5:OT, J5:OT-A, S1:DiP, S1:DiP-A, S5:DiP, S5:DiP-A, S5:OT, S5:OT-A
70	Yes	11	11-A	200	exp	Inf	unif	singleC	J1:DiP, J1:DiP-A, J1:OT, J1:OT-A, J5:CBN, J5:CBN-A, J5:DiP, J5:DiP-A, J5:OT, J5:OT-A, S1:DiP, S1:DiP-A, S5:DiP, S5:DiP-A, S5:OT, S5:OT-A
71	Yes	11	11-A	200	exp	Inf	unif	wholeT_0.01	J5:DiP, J5:DiP-A, J5:OT, J5:OT-A, S5:OT, S5:OT-A
72	Yes	11	11-A	200	exp	Inf	unif	wholeT_0.5	J1:DiP, J1:DiP-A, J1:OT, J1:OT-A, J5:CBN, J5:CBN-A, J5:DiP, J5:DiP-A, J5:OT, J5:OT-A, S1:DiP, S1:DiP-A, S5:DiP, S5:DiP-A, S5:OT, S5:OT-A

Table 8: *(continued)*

	Conjunction	Drivers	Tree	S.Size	Model	sh	S.Time	S.Type	Best method(s)
73	Yes	11	11-A	200	McF_4	0	last	singleC	J1:DiP, J1:DiP-A, J1:OT, J1:OT-A, J5:DiP, J5:DiP-A, J5:OT, J5:OT-A, S5:DiP, S5:DiP-A, S5:OT, S5:OT-A
74	Yes	11	11-A	200	McF_4	0	last	wholeT_0.01	J1:DiP, J1:DiP-A, J1:OT, J1:OT-A, J5:DiP, J5:DiP-A, J5:OT, J5:OT-A, S1:DiP-A, S5:DiP, S5:DiP-A, S5:OT, S5:OT-A
75	Yes	11	11-A	200	McF_4	0	last	wholeT_0.5	J1:DiP, J1:DiP-A, J1:OT, J1:OT-A, J5:DiP, J5:DiP-A, J5:OT, J5:OT-A, S1:DiP-A, S5:DiP, S5:DiP-A, S5:OT, S5:OT-A
76	Yes	11	11-A	200	McF_4	0	unif	singleC	J1:CBN-A, J1:DiP, J1:DiP-A, J1:OT, J1:OT-A, J5:DiP, J5:DiP-A, J5:OT, J5:OT-A, S1:DiP, S5:CBN-A, S5:DiP, S5:DiP-A, S5:OT, S5:OT-A
77	Yes	11	11-A	200	McF_4	0	unif	wholeT_0.01	J1:CBN-A, J1:DiP, J1:DiP-A, J1:OT, J1:OT-A, J5:DiP, J5:DiP-A, J5:OT, J5:OT-A, S1:DiP, S1:DiP-A, S5:CBN-A, S5:DiP, S5:DiP-A, S5:OT, S5:OT-A
78	Yes	11	11-A	200	McF_4	0	unif	wholeT_0.5	J1:CBN-A, J1:DiP, J1:DiP-A, J1:OT, J1:OT-A, J5:DiP, J5:DiP-A, J5:OT, J5:OT-A, S1:DiP, S1:DiP-A, S5:CBN, S5:CBN-A, S5:DiP, S5:DiP-A, S5:OT, S5:OT-A
79	Yes	11	11-A	200	McF_4	Inf	last	singleC	J1:DiP, J1:DiP-A, J1:OT, J1:OT-A, J5:DiP, J5:DiP-A, J5:OT, J5:OT-A, S1:DiP, S1:DiP-A, S5:DiP, S5:DiP-A, S5:OT, S5:OT-A
80	Yes	11	11-A	200	McF_4	Inf	last	wholeT_0.01	J1:DiP, J1:DiP-A, J1:OT, J1:OT-A, J5:DiP, J5:DiP-A, J5:OT, J5:OT-A, S5:OT
81	Yes	11	11-A	200	McF_4	Inf	last	wholeT_0.5	J1:DiP, J1:DiP-A, J1:OT, J1:OT-A, J5:DiP, J5:DiP-A, J5:OT, J5:OT-A, S1:DiP, S1:DiP-A, S5:DiP, S5:DiP-A, S5:OT, S5:OT-A
82	Yes	11	11-A	200	McF_4	Inf	unif	singleC	J1:CBN-A, J1:DiP, J1:DiP-A, J1:OT, J1:OT-A, J5:CBN-A, J5:DiP, J5:DiP-A, J5:OT, J5:OT-A, S5:CBN-A, S5:DiP, S5:DiP-A, S5:OT, S5:OT-A

Table 8: *(continued)*

	Conjunction	Drivers	Tree	S.Size	Model	sh	S.Time	S.Type	Best method(s)
83	Yes	11	11-A	200	McF_4	Inf	unif	wholeT_0.01	J1:CBN-A, J1:DiP, J1:DiP-A, J1:OT, J1:OT-A, J5:DiP, J5:DiP-A, J5:OT, J5:OT-A, S1:DiP, S5:CBN-A, S5:DiP, S5:DiP-A, S5:OT, S5:OT-A
84	Yes	11	11-A	200	McF_4	Inf	unif	wholeT_0.5	J1:CBN-A, J1:DiP, J1:DiP-A, J1:OT, J1:OT-A, J5:DiP, J5:DiP-A, J5:OT, J5:OT-A, S1:DiP, S5:DiP, S5:DiP-A, S5:OT, S5:OT-A
85	Yes	11	11-A	200	McF_6	0	last	singleC	J1:DiP, J1:OT, J5:DiP, J5:OT, S1:DiP, S5:DiP, S5:OT
86	Yes	11	11-A	200	McF_6	0	last	wholeT_0.01	J1:DiP, J1:OT, J5:DiP, J5:OT, S1:DiP, S5:DiP, S5:OT
87	Yes	11	11-A	200	McF_6	0	last	wholeT_0.5	J1:DiP, J1:OT, J5:DiP, J5:OT, S1:DiP, S5:DiP, S5:OT
88	Yes	11	11-A	200	McF_6	0	unif	singleC	J1:CBN, J1:CBN-A, J1:OT, J1:OT-A, J5:CBN, J5:CBN-A, J5:DiP, J5:OT, J5:OT-A, S5:CBN, S5:CBN-A, S5:OT, S5:OT-A
89	Yes	11	11-A	200	McF_6	0	unif	wholeT_0.01	J1:CBN, J1:CBN-A, J1:DiP, J1:DiP-A, J1:OT, J1:OT-A, J5:CBN, J5:CBN-A, J5:DiP, J5:DiP-A, J5:OT, J5:OT-A, S5:CBN, S5:CBN-A, S5:DiP, S5:DiP-A, S5:OT, S5:OT-A
90	Yes	11	11-A	200	McF_6	0	unif	wholeT_0.5	J1:CBN, J1:CBN-A, J1:DiP, J5:CBN, J5:CBN-A, J5:DiP, J5:DiP-A, J5:OT, J5:OT-A, S5:CBN, S5:CBN-A, S5:DiP, S5:OT, S5:OT-A
91	Yes	11	11-A	200	McF_6	Inf	last	singleC	J1:DiP, J1:OT, J5:DiP, J5:OT, S1:DiP, S5:DiP, S5:OT
92	Yes	11	11-A	200	McF_6	Inf	last	wholeT_0.01	J1:DiP, J1:OT, J5:DiP, J5:OT, S1:DiP, S5:DiP, S5:OT
93	Yes	11	11-A	200	McF_6	Inf	last	wholeT_0.5	J1:DiP, J1:OT, J5:DiP, J5:OT, S1:DiP, S5:DiP, S5:OT
94	Yes	11	11-A	200	McF_6	Inf	unif	singleC	J1:CBN-A, J1:OT, J1:OT-A, J5:CBN, J5:CBN-A, J5:OT, J5:OT-A, S5:CBN, S5:CBN-A
95	Yes	11	11-A	200	McF_6	Inf	unif	wholeT_0.01	J1:CBN-A, J1:OT, J1:OT-A, J5:CBN-A, J5:DiP, J5:DiP-A, J5:OT, J5:OT-A, S5:CBN, S5:CBN-A, S5:OT, S5:OT-A

Table 8: *(continued)*

	Conjunction	Drivers	Tree	S.Size	Model	sh	S.Time	S.Type	Best method(s)
96	Yes	11	11-A	200	McF_6	Inf	unif	wholeT_0.5	J1:CBN-A, J1:OT, J1:OT-A, J5:CBN, J5:CBN-A, J5:DiP, J5:DiP-A, J5:OT, J5:OT-A, S5:CBN, S5:CBN-A, S5:OT, S5:OT-A
97	Yes	11	11-A	100	Bozic	0	last	singleC	J1:DiP, J1:DiP-A, J5:DiP, J5:DiP-A, J5:OT, J5:OT-A, S1:DiP, S1:DiP-A, S5:DiP, S5:DiP-A, S5:OT, S5:OT-A
98	Yes	11	11-A	100	Bozic	0	last	wholeT_0.01	J1:DiP, J1:DiP-A, J5:DiP, J5:DiP-A, J5:OT, J5:OT-A
99	Yes	11	11-A	100	Bozic	0	last	wholeT_0.5	J1:DiP, J1:DiP-A, J5:DiP, J5:DiP-A, J5:OT, J5:OT-A, S1:DiP, S1:DiP-A, S5:DiP, S5:DiP-A, S5:OT, S5:OT-A
100	Yes	11	11-A	100	Bozic	0	unif	singleC	J1:DiP, J1:DiP-A, J1:OT, J1:OT-A, J5:CBN, J5:CBN-A, J5:DiP, J5:DiP-A, J5:OT, J5:OT-A, S1:DiP, S1:DiP-A, S5:DiP, S5:DiP-A, S5:OT, S5:OT-A
101	Yes	11	11-A	100	Bozic	0	unif	wholeT_0.01	J5:DiP, J5:DiP-A, J5:OT, J5:OT-A, S5:DiP, S5:DiP-A, S5:OT, S5:OT-A
102	Yes	11	11-A	100	Bozic	0	unif	wholeT_0.5	J1:DiP, J1:DiP-A, J5:CBN, J5:CBN-A, J5:DiP, J5:DiP-A, J5:OT, J5:OT-A, S1:DiP, S1:DiP-A, S5:DiP, S5:DiP-A, S5:OT, S5:OT-A
103	Yes	11	11-A	100	Bozic	Inf	last	singleC	J1:DiP, J1:DiP-A, J5:DiP, J5:DiP-A, J5:OT, J5:OT-A, S1:DiP, S5:DiP, S5:DiP-A
104	Yes	11	11-A	100	Bozic	Inf	last	wholeT_0.01	J5:DiP, J5:DiP-A, J5:OT, J5:OT-A
105	Yes	11	11-A	100	Bozic	Inf	last	wholeT_0.5	J1:DiP, J1:DiP-A, J5:DiP, J5:DiP-A, J5:OT, J5:OT-A, S1:DiP, S1:DiP-A, S5:DiP, S5:DiP-A
106	Yes	11	11-A	100	Bozic	Inf	unif	singleC	J1:DiP, J1:DiP-A, J5:DiP, J5:DiP-A, J5:OT, J5:OT-A, S5:DiP, S5:DiP-A, S5:OT, S5:OT-A
107	Yes	11	11-A	100	Bozic	Inf	unif	wholeT_0.01	J5:DiP, J5:DiP-A, J5:OT, J5:OT-A
108	Yes	11	11-A	100	Bozic	Inf	unif	wholeT_0.5	J1:DiP, J1:DiP-A, J5:CBN, J5:CBN-A, J5:DiP, J5:DiP-A, J5:OT, J5:OT-A, S1:DiP, S1:DiP-A, S5:DiP, S5:DiP-A, S5:OT, S5:OT-A

Table 8: *(continued)*

	Conjunction	Drivers	Tree	S.Size	Model	sh	S.Time	S.Type	Best method(s)
109	Yes	11	11-A	100	exp	0	last	singleC	J1:DiP, J1:DiP-A, J1:OT, J1:OT-A, J5:CBN, J5:CBN-A, J5:DiP, J5:DiP-A, J5:OT, J5:OT-A, S1:DiP, S1:DiP-A, S5:DiP, S5:DiP-A, S5:OT, S5:OT-A
110	Yes	11	11-A	100	exp	0	last	wholeT_0.01	J1:DiP, J1:DiP-A, J1:OT, J1:OT-A, J5:DiP, J5:DiP-A, J5:OT, J5:OT-A, S1:DiP, S1:DiP-A, S5:DiP, S5:DiP-A, S5:OT, S5:OT-A
111	Yes	11	11-A	100	exp	0	last	wholeT_0.5	J1:DiP, J1:DiP-A, J1:OT, J1:OT-A, J5:CBN, J5:CBN-A, J5:DiP, J5:DiP-A, J5:OT, J5:OT-A, S1:DiP, S1:DiP-A, S1:OT, S1:OT-A, S5:DiP, S5:DiP-A, S5:OT, S5:OT-A
112	Yes	11	11-A	100	exp	0	unif	singleC	J1:CBN, J1:CBN-A, J1:DiP, J1:DiP-A, J1:OT, J1:OT-A, J5:CBN, J5:CBN-A, J5:DiP, J5:DiP-A, J5:OT, J5:OT-A, S1:DiP, S1:DiP-A, S1:OT, S1:OT-A, S5:DiP, S5:DiP-A, S5:OT, S5:OT-A
113	Yes	11	11-A	100	exp	0	unif	wholeT_0.01	J1:DiP, J1:DiP-A, J1:OT, J1:OT-A, J5:CBN, J5:CBN-A, J5:DiP, J5:DiP-A, J5:OT, J5:OT-A, S1:DiP, S1:DiP-A, S5:DiP, S5:DiP-A, S5:OT, S5:OT-A
114	Yes	11	11-A	100	exp	0	unif	wholeT_0.5	J1:DiP, J1:DiP-A, J1:OT, J1:OT-A, J5:CBN, J5:CBN-A, J5:DiP, J5:DiP-A, J5:OT, J5:OT-A, S1:DiP, S1:DiP-A, S5:DiP, S5:DiP-A, S5:OT, S5:OT-A
115	Yes	11	11-A	100	exp	Inf	last	singleC	J1:DiP, J1:DiP-A, J5:DiP, J5:DiP-A, J5:OT, J5:OT-A, S1:DiP, S1:DiP-A, S5:DiP, S5:DiP-A, S5:OT, S5:OT-A
116	Yes	11	11-A	100	exp	Inf	last	wholeT_0.01	J5:DiP, J5:DiP-A, J5:OT, J5:OT-A
117	Yes	11	11-A	100	exp	Inf	last	wholeT_0.5	J1:DiP, J1:DiP-A, J5:DiP, J5:DiP-A, J5:OT, J5:OT-A, S1:DiP, S1:DiP-A, S5:DiP, S5:DiP-A, S5:OT, S5:OT-A
118	Yes	11	11-A	100	exp	Inf	unif	singleC	J1:DiP, J1:DiP-A, J5:CBN, J5:CBN-A, J5:DiP, J5:DiP-A, J5:OT, J5:OT-A, S1:DiP, S1:DiP-A, S5:DiP, S5:DiP-A, S5:OT, S5:OT-A
119	Yes	11	11-A	100	exp	Inf	unif	wholeT_0.01	J5:DiP, J5:DiP-A, J5:OT, J5:OT-A

Table 8: *(continued)*

	Conjunction	Drivers	Tree	S.Size	Model	sh	S.Time	S.Type	Best method(s)
120	Yes	11	11-A	100	exp	Inf	unif	wholeT_0.5	J1:DiP, J1:DiP-A, J5:CBN, J5:CBN-A, J5:DiP, J5:DiP-A, J5:OT, J5:OT-A, S1:DiP, S1:DiP-A, S5:DiP, S5:DiP-A, S5:OT, S5:OT-A
121	Yes	11	11-A	100	McF_4	0	last	singleC	J1:DiP, J1:DiP-A, J5:DiP, J5:DiP-A, J5:OT, J5:OT-A, S1:DiP, S1:DiP-A, S5:DiP, S5:DiP-A, S5:OT, S5:OT-A
122	Yes	11	11-A	100	McF_4	0	last	wholeT_0.01	J1:DiP, J1:DiP-A, J5:DiP, J5:DiP-A, J5:OT, J5:OT-A, S1:DiP, S1:DiP-A, S5:DiP, S5:DiP-A, S5:OT
123	Yes	11	11-A	100	McF_4	0	last	wholeT_0.5	J1:DiP, J1:DiP-A, J5:DiP, J5:DiP-A, J5:OT, J5:OT-A, S1:DiP, S1:DiP-A, S5:DiP, S5:DiP-A, S5:OT, S5:OT-A
124	Yes	11	11-A	100	McF_4	0	unif	singleC	J1:CBN, J1:CBN-A, J1:DiP, J1:DiP-A, J1:OT, J1:OT-A, J5:DiP, J5:DiP-A, J5:OT, J5:OT-A, S1:DiP, S1:DiP-A, S5:DiP, S5:DiP-A, S5:OT, S5:OT-A
125	Yes	11	11-A	100	McF_4	0	unif	wholeT_0.01	J1:DiP, J1:DiP-A, J5:DiP, J5:DiP-A, J5:OT, J5:OT-A, S5:DiP, S5:DiP-A
126	Yes	11	11-A	100	McF_4	0	unif	wholeT_0.5	J1:CBN-A, J1:DiP, J1:DiP-A, J1:OT, J1:OT-A, J5:CBN-A, J5:DiP, J5:DiP-A, J5:OT, J5:OT-A, S1:DiP, S1:DiP-A, S5:CBN-A, S5:DiP, S5:DiP-A, S5:OT, S5:OT-A
127	Yes	11	11-A	100	McF_4	Inf	last	singleC	J1:DiP, J1:DiP-A, J5:DiP, J5:DiP-A, J5:OT, J5:OT-A, S1:DiP, S1:DiP-A, S5:DiP, S5:DiP-A, S5:OT, S5:OT-A
128	Yes	11	11-A	100	McF_4	Inf	last	wholeT_0.01	J1:DiP, J1:DiP-A, J5:DiP, J5:DiP-A, J5:OT, J5:OT-A, S1:DiP, S5:DiP, S5:DiP-A
129	Yes	11	11-A	100	McF_4	Inf	last	wholeT_0.5	J1:DiP, J1:DiP-A, J5:DiP, J5:DiP-A, J5:OT, J5:OT-A, S1:DiP, S1:DiP-A, S5:DiP, S5:DiP-A, S5:OT, S5:OT-A
130	Yes	11	11-A	100	McF_4	Inf	unif	singleC	J1:DiP, J1:DiP-A, J1:OT, J1:OT-A, J5:DiP, J5:DiP-A, J5:OT, J5:OT-A, S1:DiP, S1:DiP-A, S5:CBN-A, S5:DiP, S5:DiP-A, S5:OT, S5:OT-A
131	Yes	11	11-A	100	McF_4	Inf	unif	wholeT_0.01	J1:DiP, J1:DiP-A, J5:DiP, J5:DiP-A, J5:OT, J5:OT-A, S5:DiP, S5:DiP-A

Table 8: *(continued)*

	Conjunction	Drivers	Tree	S.Size	Model	sh	S.Time	S.Type	Best method(s)
132	Yes	11	11-A	100	McF_4	Inf	unif	wholeT_0.5	J1:CBN-A, J1:DiP, J1:DiP-A, J5:CBN-A, J5:DiP, J5:DiP-A, J5:OT, J5:OT-A, S1:DiP, S5:CBN-A, S5:DiP
133	Yes	11	11-A	100	McF_6	0	last	singleC	J1:DiP, J1:OT, J5:DiP, J5:OT, S1:DiP, S5:DiP
134	Yes	11	11-A	100	McF_6	0	last	wholeT_0.01	J1:DiP, J5:DiP, J5:OT, S1:DiP, S5:DiP, S5:OT
135	Yes	11	11-A	100	McF_6	0	last	wholeT_0.5	J1:DiP, J5:DiP, J5:OT, S1:DiP, S5:DiP, S5:OT
136	Yes	11	11-A	100	McF_6	0	unif	singleC	J1:CBN-A, J5:CBN, J5:CBN-A, J5:DiP, J5:DiP-A, J5:OT, J5:OT-A
137	Yes	11	11-A	100	McF_6	0	unif	wholeT_0.01	J1:DiP, J1:DiP-A, J5:CBN, J5:CBN-A, J5:DiP, J5:DiP-A, J5:OT, J5:OT-A, S5:DiP, S5:OT
138	Yes	11	11-A	100	McF_6	0	unif	wholeT_0.5	J1:CBN, J1:CBN-A, J5:CBN-A, J5:DiP, J5:DiP-A, S5:CBN, S5:CBN-A
139	Yes	11	11-A	100	McF_6	Inf	last	singleC	J1:DiP, J1:OT, J5:DiP, J5:OT, S1:DiP, S5:DiP
140	Yes	11	11-A	100	McF_6	Inf	last	wholeT_0.01	J1:DiP, J5:DiP, J5:OT, S1:DiP, S5:DiP
141	Yes	11	11-A	100	McF_6	Inf	last	wholeT_0.5	J1:DiP, J5:DiP, J5:OT, S1:DiP, S5:DiP
142	Yes	11	11-A	100	McF_6	Inf	unif	singleC	J1:CBN, J1:CBN-A, J5:CBN-A, J5:DiP, J5:DiP-A, J5:OT, J5:OT-A
143	Yes	11	11-A	100	McF_6	Inf	unif	wholeT_0.01	J1:DiP, J5:CBN-A, J5:DiP, J5:DiP-A, J5:OT, J5:OT-A, S5:CBN, S5:CBN-A
144	Yes	11	11-A	100	McF_6	Inf	unif	wholeT_0.5	J1:CBN-A, J1:DiP, J1:DiP-A, J1:OT, J1:OT-A, J5:CBN-A, J5:DiP, J5:DiP-A, J5:OT, J5:OT-A, S5:CBN, S5:CBN-A, S5:OT, S5:OT-A
145	Yes	9	9-A	1000	Bozic	0	last	singleC	J1:DiP, J1:DiP-A, J1:OT, J1:OT-A, J5:DiP, J5:DiP-A, J5:OT, J5:OT-A, S1:OT, S1:OT-A, S5:DiP, S5:DiP-A, S5:OT, S5:OT-A
146	Yes	9	9-A	1000	Bozic	0	last	wholeT_0.01	J1:DiP, J1:DiP-A, J1:OT, J1:OT-A, J5:DiP, J5:DiP-A, J5:OT, J5:OT-A, S1:DiP, S1:DiP-A, S1:OT, S1:OT-A, S5:DiP, S5:DiP-A, S5:OT, S5:OT-A
147	Yes	9	9-A	1000	Bozic	0	last	wholeT_0.5	J1:DiP, J1:DiP-A, J1:OT, J1:OT-A, J5:CBN, J5:CBN-A, J5:DiP, J5:DiP-A, J5:OT, J5:OT-A, S1:OT, S1:OT-A, S5:DiP, S5:DiP-A, S5:OT, S5:OT-A

Table 8: *(continued)*

	Conjunction	Drivers	Tree	S.Size	Model	sh	S.Time	S.Type	Best method(s)
148	Yes	9	9-A	1000	Bozic	0	unif	singleC	J1:DiP, J1:DiP-A, J1:OT, J1:OT-A, J5:CBN, J5:CBN-A, J5:DiP, J5:DiP-A, J5:OT, J5:OT-A, S1:DiP, S1:DiP-A, S1:OT, S1:OT-A, S5:DiP, S5:DiP-A, S5:OT, S5:OT-A
149	Yes	9	9-A	1000	Bozic	0	unif	wholeT_0.01	J1:DiP, J1:DiP-A, J1:OT, J1:OT-A, J5:DiP, J5:DiP-A, J5:OT, J5:OT-A, S1:DiP, S1:DiP-A, S1:OT, S1:OT-A, S5:DiP, S5:DiP-A, S5:OT, S5:OT-A
150	Yes	9	9-A	1000	Bozic	0	unif	wholeT_0.5	J1:CBN, J1:CBN-A, J1:DiP, J1:DiP-A, J1:OT, J1:OT-A, J5:CBN, J5:CBN-A, J5:DiP, J5:DiP-A, J5:OT, J5:OT-A, S1:DiP, S1:DiP-A, S1:OT, S1:OT-A, S5:DiP, S5:DiP-A, S5:OT, S5:OT-A
151	Yes	9	9-A	1000	Bozic	Inf	last	singleC	J1:DiP, J1:DiP-A, J1:OT, J1:OT-A, J5:DiP, J5:DiP-A, J5:OT, J5:OT-A, S5:OT, S5:OT-A
152	Yes	9	9-A	1000	Bozic	Inf	last	wholeT_0.01	J5:DiP, J5:DiP-A, J5:OT, J5:OT-A
153	Yes	9	9-A	1000	Bozic	Inf	last	wholeT_0.5	J1:DiP, J1:DiP-A, J1:OT, J1:OT-A, J5:DiP, J5:DiP-A, J5:OT, J5:OT-A, S5:DiP, S5:DiP-A, S5:OT, S5:OT-A
154	Yes	9	9-A	1000	Bozic	Inf	unif	singleC	J1:DiP, J1:DiP-A, J1:OT, J1:OT-A, J5:CBN, J5:CBN-A, J5:DiP, J5:DiP-A, J5:OT, J5:OT-A, S1:DiP, S1:DiP-A, S1:OT, S1:OT-A, S5:DiP, S5:DiP-A, S5:OT, S5:OT-A
155	Yes	9	9-A	1000	Bozic	Inf	unif	wholeT_0.01	J5:DiP, J5:DiP-A, J5:OT, J5:OT-A, S5:DiP, S5:DiP-A, S5:OT, S5:OT-A
156	Yes	9	9-A	1000	Bozic	Inf	unif	wholeT_0.5	J1:DiP, J1:DiP-A, J1:OT, J1:OT-A, J5:CBN, J5:CBN-A, J5:DiP, J5:DiP-A, J5:OT, J5:OT-A, S1:CBN, S1:DiP, S1:DiP-A, S1:OT, S1:OT-A, S5:DiP, S5:DiP-A, S5:OT, S5:OT-A
157	Yes	9	9-A	1000	exp	0	last	singleC	J1:DiP, J1:DiP-A, J1:OT, J1:OT-A, J5:CBN, J5:CBN-A, J5:DiP, J5:DiP-A, J5:OT, J5:OT-A, S1:DiP, S1:DiP-A, S1:OT, S1:OT-A, S5:DiP, S5:DiP-A, S5:OT, S5:OT-A

Table 8: *(continued)*

	Conjunction	Drivers	Tree	S.Size	Model	sh	S.Time	S.Type	Best method(s)
158	Yes	9	9-A	1000	exp	0	last	wholeT_0.01	J1:DiP, J1:DiP-A, J1:OT, J1:OT-A, J5:DiP, J5:DiP-A, J5:OT, J5:OT-A, S1:DiP-A, S1:OT, S1:OT-A, S5:DiP, S5:DiP-A, S5:OT, S5:OT-A
159	Yes	9	9-A	1000	exp	0	last	wholeT_0.5	J1:DiP, J1:DiP-A, J1:OT, J1:OT-A, J5:CBN, J5:CBN-A, J5:DiP, J5:DiP-A, J5:OT, J5:OT-A, S1:DiP, S1:DiP-A, S1:OT, S1:OT-A, S5:DiP, S5:DiP-A, S5:OT, S5:OT-A
160	Yes	9	9-A	1000	exp	0	unif	singleC	J1:CBN, J1:CBN-A, J1:DiP, J1:DiP-A, J1:OT, J1:OT-A, J5:CBN, J5:CBN-A, J5:DiP, J5:DiP-A, J5:OT, J5:OT-A, S1:DiP, S1:DiP-A, S1:OT, S1:OT-A, S5:DiP, S5:DiP-A, S5:OT, S5:OT-A
161	Yes	9	9-A	1000	exp	0	unif	wholeT_0.01	J1:DiP, J1:DiP-A, J1:OT, J1:OT-A, J5:CBN, J5:CBN-A, J5:DiP, J5:DiP-A, J5:OT, J5:OT-A, S1:DiP, S1:DiP-A, S1:OT, S1:OT-A, S5:DiP, S5:DiP-A, S5:OT, S5:OT-A
162	Yes	9	9-A	1000	exp	0	unif	wholeT_0.5	J1:CBN, J1:CBN-A, J1:DiP, J1:DiP-A, J1:OT, J1:OT-A, J5:CBN, J5:CBN-A, J5:DiP, J5:DiP-A, J5:OT, J5:OT-A, S1:DiP, S1:DiP-A, S1:OT, S1:OT-A, S5:DiP, S5:DiP-A, S5:OT, S5:OT-A
163	Yes	9	9-A	1000	exp	Inf	last	singleC	J1:DiP, J1:DiP-A, J1:OT, J1:OT-A, J5:DiP, J5:DiP-A, J5:OT, J5:OT-A, S5:DiP, S5:DiP-A, S5:OT, S5:OT-A
164	Yes	9	9-A	1000	exp	Inf	last	wholeT_0.01	J5:DiP, J5:DiP-A, J5:OT, J5:OT-A
165	Yes	9	9-A	1000	exp	Inf	last	wholeT_0.5	J1:DiP, J1:DiP-A, J1:OT, J1:OT-A, J5:DiP, J5:DiP-A, J5:OT, J5:OT-A, S5:DiP, S5:DiP-A, S5:OT, S5:OT-A
166	Yes	9	9-A	1000	exp	Inf	unif	singleC	J1:DiP, J1:DiP-A, J1:OT, J1:OT-A, J5:CBN, J5:CBN-A, J5:DiP, J5:DiP-A, J5:OT, J5:OT-A, S1:DiP, S1:DiP-A, S1:OT, S1:OT-A, S5:DiP, S5:DiP-A, S5:OT, S5:OT-A
167	Yes	9	9-A	1000	exp	Inf	unif	wholeT_0.01	J1:DiP, J1:DiP-A, J1:OT, J1:OT-A, J5:DiP, J5:DiP-A, J5:OT, J5:OT-A, S5:DiP, S5:DiP-A, S5:OT, S5:OT-A

Table 8: *(continued)*

	Conjunction	Drivers	Tree	S.Size	Model	sh	S.Time	S.Type	Best method(s)
168	Yes	9	9-A	1000	exp	Inf	unif	wholeT_0.5	J1:DiP, J1:DiP-A, J1:OT, J1:OT-A, J5:CBN, J5:CBN-A, J5:DiP, J5:DiP-A, J5:OT, J5:OT-A, S1:DiP, S1:DiP-A, S1:OT, S1:OT-A, S5:DiP, S5:DiP-A, S5:OT, S5:OT-A
169	Yes	9	9-A	1000	McF_4	0	last	singleC	J1:DiP, J1:DiP-A, J1:OT, J1:OT-A, J5:DiP, J5:DiP-A, J5:OT, J5:OT-A, S1:DiP, S1:DiP-A, S1:OT, S1:OT-A, S5:DiP, S5:DiP-A, S5:OT, S5:OT-A
170	Yes	9	9-A	1000	McF_4	0	last	wholeT_0.01	J1:DiP, J1:DiP-A, J1:OT, J1:OT-A, J5:CBN, J5:DiP, J5:DiP-A, J5:OT, J5:OT-A, S1:DiP-A, S5:DiP, S5:DiP-A, S5:OT, S5:OT-A
171	Yes	9	9-A	1000	McF_4	0	last	wholeT_0.5	J1:DiP, J1:DiP-A, J1:OT, J1:OT-A, J5:CBN, J5:DiP, J5:DiP-A, J5:OT, J5:OT-A, S1:DiP, S1:DiP-A, S1:OT, S1:OT-A, S5:DiP, S5:DiP-A, S5:OT, S5:OT-A
172	Yes	9	9-A	1000	McF_4	0	unif	singleC	J1:CBN-A, J1:DiP, J1:DiP-A, J1:OT, J1:OT-A, J5:DiP, J5:DiP-A, J5:OT, J5:OT-A, S1:DiP, S1:DiP-A, S1:OT, S1:OT-A, S5:DiP, S5:DiP-A, S5:OT, S5:OT-A
173	Yes	9	9-A	1000	McF_4	0	unif	wholeT_0.01	J1:CBN-A, J1:DiP, J1:DiP-A, J1:OT, J1:OT-A, J5:DiP, J5:DiP-A, J5:OT, J5:OT-A, S1:DiP-A, S5:CBN-A, S5:DiP, S5:DiP-A, S5:OT, S5:OT-A
174	Yes	9	9-A	1000	McF_4	0	unif	wholeT_0.5	J1:DiP, J1:DiP-A, J1:OT, J1:OT-A, J5:DiP, J5:DiP-A, J5:OT, J5:OT-A, S1:DiP, S1:DiP-A, S1:OT, S1:OT-A, S5:DiP, S5:DiP-A, S5:OT, S5:OT-A
175	Yes	9	9-A	1000	McF_4	Inf	last	singleC	J1:DiP, J1:DiP-A, J1:OT, J1:OT-A, J5:DiP, J5:DiP-A, J5:OT, J5:OT-A, S1:OT, S1:OT-A, S5:DiP, S5:DiP-A, S5:OT, S5:OT-A
176	Yes	9	9-A	1000	McF_4	Inf	last	wholeT_0.01	J1:DiP, J1:OT, J5:DiP, J5:OT, S1:DiP, S5:DiP, S5:OT

Table 8: *(continued)*

	Conjunction	Drivers	Tree	S.Size	Model	sh	S.Time	S.Type	Best method(s)
177	Yes	9	9-A	1000	McF_4	Inf	last	wholeT_0.5	J1:DiP, J1:DiP-A, J1:OT, J1:OT-A, J5:DiP, J5:DiP-A, J5:OT, J5:OT-A, S1:DiP, S1:DiP-A, S1:OT, S1:OT-A, S5:DiP, S5:DiP-A, S5:OT, S5:OT-A
178	Yes	9	9-A	1000	McF_4	Inf	unif	singleC	J1:DiP, J1:DiP-A, J1:OT, J1:OT-A, J5:DiP, J5:DiP-A, J5:OT, J5:OT-A, S1:DiP, S1:DiP-A, S1:OT, S1:OT-A, S5:CBN-A, S5:DiP, S5:DiP-A, S5:OT, S5:OT-A
179	Yes	9	9-A	1000	McF_4	Inf	unif	wholeT_0.01	J1:DiP, J1:DiP-A, J1:OT, J1:OT-A, J5:DiP, J5:DiP-A, J5:OT, J5:OT-A, S5:CBN-A, S5:DiP, S5:DiP-A, S5:OT, S5:OT-A
180	Yes	9	9-A	1000	McF_4	Inf	unif	wholeT_0.5	J1:DiP, J1:DiP-A, J1:OT, J1:OT-A, J5:DiP, J5:DiP-A, J5:OT, J5:OT-A, S1:DiP, S1:DiP-A, S1:OT, S1:OT-A, S5:CBN-A, S5:DiP, S5:DiP-A, S5:OT, S5:OT-A
181	Yes	9	9-A	1000	McF_6	0	last	singleC	J1:DiP, J1:OT, J5:DiP, J5:OT, S1:DiP, S5:DiP, S5:OT
182	Yes	9	9-A	1000	McF_6	0	last	wholeT_0.01	J1:DiP, J1:OT, J5:DiP, J5:OT, S1:DiP, S5:DiP, S5:OT
183	Yes	9	9-A	1000	McF_6	0	last	wholeT_0.5	J1:DiP, J1:OT, J5:DiP, J5:OT, S1:DiP, S1:OT, S5:DiP, S5:OT
184	Yes	9	9-A	1000	McF_6	0	unif	singleC	J1:CBN, J1:CBN-A, J1:DiP, J1:DiP-A, J1:OT, J1:OT-A, J5:CBN, J5:CBN-A, J5:DiP, J5:DiP-A, J5:OT, J5:OT-A, S1:DiP, S1:DiP-A, S1:OT, S1:OT-A, S5:CBN, S5:CBN-A, S5:DiP, S5:DiP-A, S5:OT, S5:OT-A
185	Yes	9	9-A	1000	McF_6	0	unif	wholeT_0.01	J1:CBN, J1:CBN-A, J1:DiP, J1:DiP-A, J1:OT, J1:OT-A, J5:CBN, J5:CBN-A, J5:DiP, J5:DiP-A, J5:OT, J5:OT-A, S1:DiP, S5:CBN, S5:CBN-A, S5:DiP, S5:DiP-A, S5:OT, S5:OT-A

Table 8: *(continued)*

	Conjunction	Drivers	Tree	S.Size	Model	sh	S.Time	S.Type	Best method(s)
186	Yes	9	9-A	1000	McF_6	0	unif	wholeT_0.5	J1:CBN, J1:CBN-A, J1:DiP, J1:DiP-A, J1:OT, J1:OT-A, J5:CBN, J5:CBN-A, J5:DiP, J5:DiP-A, J5:OT, J5:OT-A, S1:CBN-A, S1:DiP, S1:DiP-A, S1:OT, S1:OT-A, S5:CBN, S5:CBN-A, S5:DiP, S5:DiP-A, S5:OT, S5:OT-A
187	Yes	9	9-A	1000	McF_6	Inf	last	singleC	J1:DiP, J1:OT, J5:DiP, J5:OT, S1:DiP, S5:DiP, S5:OT
188	Yes	9	9-A	1000	McF_6	Inf	last	wholeT_0.01	J1:DiP, J1:OT, J5:DiP, J5:OT, S5:OT
189	Yes	9	9-A	1000	McF_6	Inf	last	wholeT_0.5	J1:DiP, J1:OT, J5:DiP, J5:OT, S1:DiP, S5:DiP, S5:OT
190	Yes	9	9-A	1000	McF_6	Inf	unif	singleC	J1:CBN, J1:CBN-A, J1:DiP, J1:DiP-A, J1:OT, J1:OT-A, J5:CBN, J5:CBN-A, J5:DiP, J5:DiP-A, J5:OT, J5:OT-A, S1:DiP, S1:DiP-A, S1:OT, S1:OT-A, S5:CBN, S5:CBN-A, S5:DiP, S5:DiP-A, S5:OT, S5:OT-A
191	Yes	9	9-A	1000	McF_6	Inf	unif	wholeT_0.01	J1:CBN, J1:CBN-A, J1:DiP, J1:DiP-A, J1:OT, J1:OT-A, J5:CBN, J5:CBN-A, J5:DiP, J5:DiP-A, J5:OT, J5:OT-A, S1:DiP, S1:DiP-A, S5:CBN, S5:CBN-A, S5:DiP, S5:DiP-A, S5:OT, S5:OT-A
192	Yes	9	9-A	1000	McF_6	Inf	unif	wholeT_0.5	J1:CBN, J1:CBN-A, J1:DiP, J1:DiP-A, J1:OT, J1:OT-A, J5:CBN, J5:CBN-A, J5:DiP, J5:DiP-A, J5:OT, J5:OT-A, S1:DiP, S1:DiP-A, S1:OT, S1:OT-A, S5:CBN, S5:CBN-A, S5:DiP, S5:DiP-A, S5:OT, S5:OT-A
193	Yes	9	9-A	200	Bozic	0	last	singleC	J1:DiP, J1:DiP-A, J1:OT, J1:OT-A, J5:DiP, J5:DiP-A, J5:OT, J5:OT-A, S1:DiP, S1:DiP-A, S1:OT, S1:OT-A, S5:DiP, S5:DiP-A, S5:OT, S5:OT-A
194	Yes	9	9-A	200	Bozic	0	last	wholeT_0.01	J1:DiP, J1:DiP-A, J1:OT, J1:OT-A, J5:DiP, J5:DiP-A, J5:OT, J5:OT-A, S1:DiP, S1:DiP-A, S5:DiP, S5:DiP-A, S5:OT, S5:OT-A
195	Yes	9	9-A	200	Bozic	0	last	wholeT_0.5	J1:DiP, J1:DiP-A, J1:OT, J1:OT-A, J5:DiP, J5:DiP-A, J5:OT, J5:OT-A, S1:DiP, S1:DiP-A, S5:DiP, S5:DiP-A, S5:OT, S5:OT-A

Table 8: *(continued)*

	Conjunction	Drivers	Tree	S.Size	Model	sh	S.Time	S.Type	Best method(s)
196	Yes	9	9-A	200	Bozic	0	unif	singleC	J1:DiP, J1:DiP-A, J1:OT, J1:OT-A, J5:CBN, J5:CBN-A, J5:DiP, J5:DiP-A, J5:OT, J5:OT-A, S1:DiP, S1:DiP-A, S1:OT, S1:OT-A, S5:DiP, S5:DiP-A, S5:OT, S5:OT-A
197	Yes	9	9-A	200	Bozic	0	unif	wholeT_0.01	J1:DiP, J1:DiP-A, J1:OT, J1:OT-A, J5:DiP, J5:DiP-A, J5:OT, J5:OT-A, S5:DiP, S5:DiP-A, S5:OT, S5:OT-A
198	Yes	9	9-A	200	Bozic	0	unif	wholeT_0.5	J1:DiP, J1:DiP-A, J1:OT, J1:OT-A, J5:CBN, J5:CBN-A, J5:DiP, J5:DiP-A, J5:OT, J5:OT-A, S1:DiP, S1:DiP-A, S1:OT, S1:OT-A, S5:DiP, S5:DiP-A, S5:OT, S5:OT-A
199	Yes	9	9-A	200	Bozic	Inf	last	singleC	J1:DiP, J1:DiP-A, J5:DiP, J5:DiP-A, J5:OT, J5:OT-A, S5:DiP, S5:DiP-A, S5:OT, S5:OT-A
200	Yes	9	9-A	200	Bozic	Inf	last	wholeT_0.01	J5:DiP, J5:DiP-A, J5:OT, J5:OT-A
201	Yes	9	9-A	200	Bozic	Inf	last	wholeT_0.5	J1:DiP, J1:DiP-A, J5:DiP, J5:DiP-A, J5:OT, J5:OT-A, S5:DiP, S5:DiP-A, S5:OT, S5:OT-A
202	Yes	9	9-A	200	Bozic	Inf	unif	singleC	J1:DiP, J1:DiP-A, J1:OT, J1:OT-A, J5:DiP, J5:DiP-A, J5:OT, J5:OT-A, S5:DiP, S5:DiP-A, S5:OT, S5:OT-A
203	Yes	9	9-A	200	Bozic	Inf	unif	wholeT_0.01	J5:DiP, J5:DiP-A, J5:OT, J5:OT-A, S5:OT, S5:OT-A
204	Yes	9	9-A	200	Bozic	Inf	unif	wholeT_0.5	J1:DiP, J1:DiP-A, J1:OT, J1:OT-A, J5:DiP, J5:DiP-A, J5:OT, J5:OT-A, S5:DiP, S5:DiP-A, S5:OT, S5:OT-A
205	Yes	9	9-A	200	exp	0	last	singleC	J1:DiP, J1:DiP-A, J1:OT, J1:OT-A, J5:CBN, J5:CBN-A, J5:DiP, J5:DiP-A, J5:OT, J5:OT-A, S1:DiP, S1:DiP-A, S1:OT, S1:OT-A, S5:DiP, S5:DiP-A, S5:OT, S5:OT-A
206	Yes	9	9-A	200	exp	0	last	wholeT_0.01	J1:DiP, J1:DiP-A, J1:OT, J1:OT-A, J5:DiP, J5:DiP-A, J5:OT, J5:OT-A, S1:DiP, S1:DiP-A, S1:OT, S1:OT-A, S5:DiP, S5:DiP-A, S5:OT, S5:OT-A

Table 8: *(continued)*

	Conjunction	Drivers	Tree	S.Size	Model	sh	S.Time	S.Type	Best method(s)
207	Yes	9	9-A	200	exp	0	last	wholeT_0.5	J1:DiP, J1:DiP-A, J1:OT, J1:OT-A, J5:CBN, J5:CBN-A, J5:DiP, J5:DiP-A, J5:OT, J5:OT-A, S1:DiP, S1:DiP-A, S1:OT, S1:OT-A, S5:DiP, S5:DiP-A, S5:OT, S5:OT-A
208	Yes	9	9-A	200	exp	0	unif	singleC	J1:CBN, J1:CBN-A, J1:DiP, J1:DiP-A, J1:OT, J1:OT-A, J5:CBN, J5:CBN-A, J5:DiP, J5:DiP-A, J5:OT, J5:OT-A, S1:DiP, S1:DiP-A, S1:OT, S1:OT-A, S5:DiP, S5:DiP-A, S5:OT, S5:OT-A
209	Yes	9	9-A	200	exp	0	unif	wholeT_0.01	J1:DiP, J1:DiP-A, J1:OT, J1:OT-A, J5:CBN, J5:CBN-A, J5:DiP, J5:DiP-A, J5:OT, J5:OT-A, S1:DiP, S1:DiP-A, S1:OT, S1:OT-A, S5:DiP, S5:DiP-A, S5:OT, S5:OT-A
210	Yes	9	9-A	200	exp	0	unif	wholeT_0.5	J1:CBN, J1:CBN-A, J1:DiP, J1:DiP-A, J1:OT, J1:OT-A, J5:CBN, J5:CBN-A, J5:DiP, J5:DiP-A, J5:OT, J5:OT-A, S1:DiP, S1:DiP-A, S1:OT, S1:OT-A, S5:DiP, S5:DiP-A, S5:OT, S5:OT-A
211	Yes	9	9-A	200	exp	Inf	last	singleC	J1:DiP, J1:DiP-A, J1:OT, J1:OT-A, J5:DiP, J5:DiP-A, J5:OT, J5:OT-A, S1:DiP, S1:DiP-A, S5:DiP, S5:DiP-A, S5:OT, S5:OT-A
212	Yes	9	9-A	200	exp	Inf	last	wholeT_0.01	J5:DiP, J5:DiP-A, J5:OT, J5:OT-A
213	Yes	9	9-A	200	exp	Inf	last	wholeT_0.5	J1:DiP, J1:DiP-A, J1:OT, J1:OT-A, J5:DiP, J5:DiP-A, J5:OT, J5:OT-A, S1:DiP, S1:DiP-A, S5:DiP, S5:DiP-A, S5:OT, S5:OT-A
214	Yes	9	9-A	200	exp	Inf	unif	singleC	J1:DiP, J1:DiP-A, J1:OT, J1:OT-A, J5:CBN, J5:CBN-A, J5:DiP, J5:DiP-A, J5:OT, J5:OT-A, S1:DiP, S1:DiP-A, S5:DiP, S5:DiP-A, S5:OT, S5:OT-A
215	Yes	9	9-A	200	exp	Inf	unif	wholeT_0.01	J5:DiP, J5:DiP-A, J5:OT, J5:OT-A, S5:DiP, S5:DiP-A, S5:OT, S5:OT-A
216	Yes	9	9-A	200	exp	Inf	unif	wholeT_0.5	J1:DiP, J1:DiP-A, J1:OT, J1:OT-A, J5:CBN, J5:CBN-A, J5:DiP, J5:DiP-A, J5:OT, J5:OT-A, S1:DiP, S1:DiP-A, S1:OT, S1:OT-A, S5:DiP, S5:DiP-A, S5:OT, S5:OT-A

Table 8: *(continued)*

	Conjunction	Drivers	Tree	S.Size	Model	sh	S.Time	S.Type	Best method(s)
217	Yes	9	9-A	200	McF_4	0	last	singleC	J1:DiP, J1:DiP-A, J1:OT, J1:OT-A, J5:DiP, J5:DiP-A, J5:OT, J5:OT-A, S5:DiP, S5:DiP-A, S5:OT, S5:OT-A
218	Yes	9	9-A	200	McF_4	0	last	wholeT_0.01	J1:DiP, J1:DiP-A, J1:OT, J1:OT-A, J5:DiP, J5:DiP-A, J5:OT, J5:OT-A, S1:DiP, S1:DiP-A, S5:DiP, S5:DiP-A, S5:OT, S5:OT-A
219	Yes	9	9-A	200	McF_4	0	last	wholeT_0.5	J1:DiP, J1:DiP-A, J1:OT, J1:OT-A, J5:DiP, J5:DiP-A, J5:OT, J5:OT-A, S1:DiP, S1:DiP-A, S5:DiP, S5:DiP-A, S5:OT, S5:OT-A
220	Yes	9	9-A	200	McF_4	0	unif	singleC	J1:DiP, J1:DiP-A, J1:OT, J1:OT-A, J5:DiP, J5:DiP-A, J5:OT, J5:OT-A, S1:DiP, S1:DiP-A, S5:CBN-A, S5:DiP, S5:DiP-A, S5:OT, S5:OT-A
221	Yes	9	9-A	200	McF_4	0	unif	wholeT_0.01	J1:DiP, J1:DiP-A, J1:OT, J1:OT-A, J5:DiP, J5:DiP-A, J5:OT, J5:OT-A, S1:DiP, S1:DiP-A, S5:CBN-A, S5:DiP, S5:DiP-A, S5:OT, S5:OT-A
222	Yes	9	9-A	200	McF_4	0	unif	wholeT_0.5	J1:DiP, J1:DiP-A, J1:OT, J1:OT-A, J5:DiP, J5:DiP-A, J5:OT, J5:OT-A, S1:DiP, S1:DiP-A, S5:DiP, S5:DiP-A, S5:OT, S5:OT-A
223	Yes	9	9-A	200	McF_4	Inf	last	singleC	J1:DiP, J1:DiP-A, J5:DiP, J5:DiP-A, J5:OT, J5:OT-A, S5:DiP, S5:DiP-A, S5:OT, S5:OT-A
224	Yes	9	9-A	200	McF_4	Inf	last	wholeT_0.01	J1:DiP, J1:DiP-A, J5:DiP, J5:DiP-A, J5:OT, S1:DiP, S5:DiP, S5:DiP-A, S5:OT
225	Yes	9	9-A	200	McF_4	Inf	last	wholeT_0.5	J1:DiP, J1:DiP-A, J1:OT, J1:OT-A, J5:DiP, J5:DiP-A, J5:OT, J5:OT-A, S1:DiP, S1:DiP-A, S5:DiP, S5:DiP-A, S5:OT, S5:OT-A
226	Yes	9	9-A	200	McF_4	Inf	unif	singleC	J1:DiP, J1:DiP-A, J1:OT, J1:OT-A, J5:DiP, J5:DiP-A, J5:OT, J5:OT-A, S1:DiP, S1:DiP-A, S5:DiP, S5:DiP-A, S5:OT, S5:OT-A
227	Yes	9	9-A	200	McF_4	Inf	unif	wholeT_0.01	J1:DiP, J1:DiP-A, J1:OT, J1:OT-A, J5:DiP, J5:DiP-A, J5:OT, J5:OT-A, S1:DiP, S1:DiP-A, S5:DiP, S5:DiP-A, S5:OT, S5:OT-A

Table 8: *(continued)*

	Conjunction	Drivers	Tree	S.Size	Model	sh	S.Time	S.Type	Best method(s)
228	Yes	9	9-A	200	McF_4	Inf	unif	wholeT_0.5	J1:DiP, J1:DiP-A, J1:OT, J1:OT-A, J5:DiP, J5:DiP-A, J5:OT, J5:OT-A, S1:DiP, S1:DiP-A, S5:CBN-A, S5:DiP, S5:DiP-A, S5:OT, S5:OT-A
229	Yes	9	9-A	200	McF_6	0	last	singleC	J1:DiP, J1:OT, J5:DiP, J5:OT, S1:DiP, S5:DiP, S5:OT
230	Yes	9	9-A	200	McF_6	0	last	wholeT_0.01	J1:DiP, J5:DiP, J5:OT, S1:DiP, S5:DiP, S5:OT
231	Yes	9	9-A	200	McF_6	0	last	wholeT_0.5	J1:DiP, J1:OT, J5:DiP, J5:OT, S1:DiP, S5:DiP, S5:OT
232	Yes	9	9-A	200	McF_6	0	unif	singleC	J1:CBN, J1:CBN-A, J1:DiP, J1:DiP-A, J1:OT, J1:OT-A, J5:CBN, J5:CBN-A, J5:DiP, J5:DiP-A, J5:OT, J5:OT-A, S1:DiP, S1:DiP-A, S5:CBN, S5:CBN-A, S5:DiP, S5:DiP-A, S5:OT, S5:OT-A
233	Yes	9	9-A	200	McF_6	0	unif	wholeT_0.01	J1:CBN, J1:CBN-A, J1:DiP, J1:DiP-A, J1:OT, J1:OT-A, J5:CBN, J5:CBN-A, J5:DiP, J5:DiP-A, J5:OT, J5:OT-A, S1:DiP, S1:DiP-A, S5:CBN, S5:CBN-A, S5:DiP, S5:DiP-A, S5:OT, S5:OT-A
234	Yes	9	9-A	200	McF_6	0	unif	wholeT_0.5	J1:CBN, J1:CBN-A, J1:DiP, J1:DiP-A, J1:OT, J1:OT-A, J5:CBN, J5:CBN-A, J5:DiP, J5:DiP-A, J5:OT, J5:OT-A, S1:DiP, S1:DiP-A, S5:CBN, S5:CBN-A, S5:DiP, S5:DiP-A, S5:OT, S5:OT-A
235	Yes	9	9-A	200	McF_6	Inf	last	singleC	J1:DiP, J1:OT, J5:DiP, J5:OT, S1:DiP, S5:DiP, S5:OT
236	Yes	9	9-A	200	McF_6	Inf	last	wholeT_0.01	J1:DiP, J5:DiP, J5:OT, S1:DiP, S5:DiP, S5:OT
237	Yes	9	9-A	200	McF_6	Inf	last	wholeT_0.5	J1:DiP, J1:OT, J5:DiP, J5:OT, S1:DiP, S5:DiP, S5:OT
238	Yes	9	9-A	200	McF_6	Inf	unif	singleC	J1:CBN, J1:CBN-A, J1:DiP, J1:DiP-A, J1:OT, J1:OT-A, J5:CBN, J5:CBN-A, J5:DiP, J5:DiP-A, J5:OT, J5:OT-A, S1:DiP, S1:DiP-A, S5:CBN, S5:CBN-A, S5:DiP, S5:DiP-A, S5:OT, S5:OT-A

Table 8: *(continued)*

	Conjunction	Drivers	Tree	S.Size	Model	sh	S.Time	S.Type	Best method(s)
239	Yes	9	9-A	200	McF_6	Inf	unif	wholeT_0.01	J1:CBN, J1:CBN-A, J1:DiP, J1:DiP-A, J1:OT, J1:OT-A, J5:CBN, J5:CBN-A, J5:DiP, J5:DiP-A, J5:OT, J5:OT-A, S1:DiP, S5:CBN, S5:CBN-A, S5:DiP, S5:DiP-A, S5:OT, S5:OT-A
240	Yes	9	9-A	200	McF_6	Inf	unif	wholeT_0.5	J1:CBN, J1:CBN-A, J1:DiP, J1:DiP-A, J1:OT, J1:OT-A, J5:CBN, J5:CBN-A, J5:DiP, J5:DiP-A, J5:OT, J5:OT-A, S1:DiP, S1:DiP-A, S5:CBN, S5:CBN-A, S5:DiP, S5:DiP-A, S5:OT, S5:OT-A
241	Yes	9	9-A	100	Bozic	0	last	singleC	J1:DiP, J1:DiP-A, J1:OT, J1:OT-A, J5:DiP, J5:DiP-A, J5:OT, J5:OT-A, S1:DiP, S1:DiP-A, S5:DiP, S5:DiP-A, S5:OT, S5:OT-A
242	Yes	9	9-A	100	Bozic	0	last	wholeT_0.01	J1:DiP, J1:DiP-A, J5:DiP, J5:DiP-A, J5:OT, J5:OT-A, S1:DiP, S1:DiP-A, S5:DiP, S5:DiP-A, S5:OT, S5:OT-A
243	Yes	9	9-A	100	Bozic	0	last	wholeT_0.5	J1:DiP, J1:DiP-A, J1:OT, J1:OT-A, J5:DiP, J5:DiP-A, J5:OT, J5:OT-A, S1:DiP, S1:DiP-A, S5:DiP, S5:DiP-A, S5:OT, S5:OT-A
244	Yes	9	9-A	100	Bozic	0	unif	singleC	J1:DiP, J1:DiP-A, J1:OT, J1:OT-A, J5:CBN, J5:CBN-A, J5:DiP, J5:DiP-A, J5:OT, J5:OT-A, S1:DiP, S1:DiP-A, S5:DiP, S5:DiP-A, S5:OT, S5:OT-A
245	Yes	9	9-A	100	Bozic	0	unif	wholeT_0.01	J1:DiP, J1:DiP-A, J5:DiP, J5:DiP-A, J5:OT, J5:OT-A, S1:DiP, S1:DiP-A, S5:DiP, S5:DiP-A, S5:OT, S5:OT-A
246	Yes	9	9-A	100	Bozic	0	unif	wholeT_0.5	J1:DiP, J1:DiP-A, J1:OT, J1:OT-A, J5:CBN, J5:CBN-A, J5:DiP, J5:DiP-A, J5:OT, J5:OT-A, S1:DiP, S1:DiP-A, S5:DiP, S5:DiP-A, S5:OT, S5:OT-A
247	Yes	9	9-A	100	Bozic	Inf	last	singleC	J1:DiP, J1:DiP-A, J5:DiP, J5:DiP-A, J5:OT, J5:OT-A, S1:DiP, S1:DiP-A, S5:DiP, S5:DiP-A
248	Yes	9	9-A	100	Bozic	Inf	last	wholeT_0.01	J5:DiP, J5:DiP-A
249	Yes	9	9-A	100	Bozic	Inf	last	wholeT_0.5	J1:DiP, J1:DiP-A, J5:DiP, J5:DiP-A, J5:OT, J5:OT-A, S1:DiP, S1:DiP-A, S5:DiP, S5:DiP-A

Table 8: *(continued)*

	Conjunction	Drivers	Tree	S.Size	Model	sh	S.Time	S.Type	Best method(s)
250	Yes	9	9-A	100	Bozic	Inf	unif	singleC	J1:DiP, J1:DiP-A, J5:DiP, J5:DiP-A, J5:OT, J5:OT-A, S1:DiP, S1:DiP-A, S5:DiP, S5:DiP-A, S5:OT, S5:OT-A
251	Yes	9	9-A	100	Bozic	Inf	unif	wholeT_0.01	J5:DiP, J5:DiP-A, J5:OT, J5:OT-A
252	Yes	9	9-A	100	Bozic	Inf	unif	wholeT_0.5	J1:DiP, J1:DiP-A, J5:DiP, J5:DiP-A, J5:OT, J5:OT-A, S5:DiP, S5:DiP-A, S5:OT, S5:OT-A
253	Yes	9	9-A	100	exp	0	last	singleC	J1:DiP, J1:DiP-A, J1:OT, J1:OT-A, J5:CBN, J5:CBN-A, J5:DiP, J5:DiP-A, J5:OT, J5:OT-A, S1:DiP, S1:DiP-A, S5:DiP, S5:DiP-A, S5:OT, S5:OT-A
254	Yes	9	9-A	100	exp	0	last	wholeT_0.01	J1:DiP, J1:DiP-A, J1:OT, J1:OT-A, J5:DiP, J5:DiP-A, J5:OT, J5:OT-A, S1:DiP, S1:DiP-A, S5:DiP, S5:DiP-A, S5:OT, S5:OT-A
255	Yes	9	9-A	100	exp	0	last	wholeT_0.5	J1:DiP, J1:DiP-A, J1:OT, J1:OT-A, J5:CBN, J5:CBN-A, J5:DiP, J5:DiP-A, J5:OT, J5:OT-A, S1:DiP, S1:DiP-A, S1:OT, S1:OT-A, S5:DiP, S5:DiP-A, S5:OT, S5:OT-A
256	Yes	9	9-A	100	exp	0	unif	singleC	J1:CBN, J1:CBN-A, J1:DiP, J1:DiP-A, J1:OT, J1:OT-A, J5:CBN, J5:CBN-A, J5:DiP, J5:DiP-A, J5:OT, J5:OT-A, S1:DiP, S1:DiP-A, S1:OT, S1:OT-A, S5:DiP, S5:DiP-A, S5:OT, S5:OT-A
257	Yes	9	9-A	100	exp	0	unif	wholeT_0.01	J1:DiP, J1:DiP-A, J1:OT, J1:OT-A, J5:CBN, J5:CBN-A, J5:DiP, J5:DiP-A, J5:OT, J5:OT-A, S1:DiP, S1:DiP-A, S5:DiP, S5:DiP-A, S5:OT, S5:OT-A
258	Yes	9	9-A	100	exp	0	unif	wholeT_0.5	J1:DiP, J1:DiP-A, J1:OT, J1:OT-A, J5:CBN, J5:CBN-A, J5:DiP, J5:DiP-A, J5:OT, J5:OT-A, S1:DiP, S1:DiP-A, S1:OT, S1:OT-A, S5:DiP, S5:DiP-A, S5:OT, S5:OT-A
259	Yes	9	9-A	100	exp	Inf	last	singleC	J1:DiP, J1:DiP-A, J5:DiP, J5:DiP-A, J5:OT, J5:OT-A, S1:DiP, S1:DiP-A, S5:DiP, S5:DiP-A, S5:OT, S5:OT-A
260	Yes	9	9-A	100	exp	Inf	last	wholeT_0.01	J5:DiP, J5:DiP-A, J5:OT, J5:OT-A

Table 8: *(continued)*

	Conjunction	Drivers	Tree	S.Size	Model	sh	S.Time	S.Type	Best method(s)
261	Yes	9	9-A	100	exp	Inf	last	wholeT_0.5	J1:DiP, J1:DiP-A, J5:DiP, J5:DiP-A, J5:OT, J5:OT-A, S1:DiP, S1:DiP-A, S5:DiP, S5:DiP-A, S5:OT, S5:OT-A
262	Yes	9	9-A	100	exp	Inf	unif	singleC	J1:DiP, J1:DiP-A, J5:CBN, J5:CBN-A, J5:DiP, J5:DiP-A, J5:OT, J5:OT-A, S1:DiP, S1:DiP-A, S5:DiP, S5:DiP-A, S5:OT, S5:OT-A
263	Yes	9	9-A	100	exp	Inf	unif	wholeT_0.01	J5:DiP, J5:DiP-A, J5:OT, J5:OT-A, S5:DiP, S5:DiP-A, S5:OT, S5:OT-A
264	Yes	9	9-A	100	exp	Inf	unif	wholeT_0.5	J1:DiP, J1:DiP-A, J1:OT, J1:OT-A, J5:CBN, J5:CBN-A, J5:DiP, J5:DiP-A, J5:OT, J5:OT-A, S1:DiP, S1:DiP-A, S5:DiP, S5:DiP-A, S5:OT, S5:OT-A
265	Yes	9	9-A	100	McF_4	0	last	singleC	J1:DiP, J1:DiP-A, J5:DiP, J5:DiP-A, J5:OT, J5:OT-A, S1:DiP, S1:DiP-A, S5:DiP, S5:DiP-A, S5:OT, S5:OT-A
266	Yes	9	9-A	100	McF_4	0	last	wholeT_0.01	J1:DiP, J1:DiP-A, J5:DiP, J5:DiP-A, J5:OT, J5:OT-A, S1:DiP, S1:DiP-A, S5:DiP, S5:DiP-A, S5:OT, S5:OT-A
267	Yes	9	9-A	100	McF_4	0	last	wholeT_0.5	J1:DiP, J1:DiP-A, J5:DiP, J5:DiP-A, J5:OT, J5:OT-A, S1:DiP, S1:DiP-A, S5:DiP, S5:DiP-A, S5:OT, S5:OT-A
268	Yes	9	9-A	100	McF_4	0	unif	singleC	J1:DiP, J1:DiP-A, J5:DiP, J5:DiP-A, J5:OT, J5:OT-A, S1:DiP, S1:DiP-A, S5:CBN-A, S5:DiP, S5:DiP-A, S5:OT, S5:OT-A
269	Yes	9	9-A	100	McF_4	0	unif	wholeT_0.01	J1:DiP, J1:DiP-A, J5:DiP, J5:DiP-A, J5:OT, J5:OT-A, S5:DiP, S5:DiP-A, S5:OT, S5:OT-A
270	Yes	9	9-A	100	McF_4	0	unif	wholeT_0.5	J1:DiP, J1:DiP-A, J1:OT, J1:OT-A, J5:DiP, J5:DiP-A, J5:OT, J5:OT-A, S1:DiP, S1:DiP-A, S5:CBN-A, S5:DiP, S5:DiP-A, S5:OT, S5:OT-A
271	Yes	9	9-A	100	McF_4	Inf	last	singleC	J1:DiP, J1:DiP-A, J5:DiP, J5:DiP-A, J5:OT, J5:OT-A, S1:DiP, S1:DiP-A, S5:DiP, S5:DiP-A, S5:OT, S5:OT-A
272	Yes	9	9-A	100	McF_4	Inf	last	wholeT_0.01	J1:DiP, J5:DiP, J5:OT, S1:DiP, S5:DiP, S5:OT

Table 8: *(continued)*

	Conjunction	Drivers	Tree	S.Size	Model	sh	S.Time	S.Type	Best method(s)
273	Yes	9	9-A	100	McF_4	Inf	last	wholeT_0.5	J1:DiP, J1:DiP-A, J5:DiP, J5:DiP-A, J5:OT, J5:OT-A, S1:DiP, S1:DiP-A, S5:DiP, S5:DiP-A, S5:OT, S5:OT-A
274	Yes	9	9-A	100	McF_4	Inf	unif	singleC	J1:DiP, J1:DiP-A, J1:OT, J1:OT-A, J5:DiP, J5:DiP-A, J5:OT, J5:OT-A, S1:DiP, S1:DiP-A, S5:CBN-A, S5:DiP, S5:DiP-A, S5:OT, S5:OT-A
275	Yes	9	9-A	100	McF_4	Inf	unif	wholeT_0.01	J1:DiP, J1:DiP-A, J5:DiP, J5:DiP-A, J5:OT, J5:OT-A, S5:DiP, S5:DiP-A, S5:OT, S5:OT-A
276	Yes	9	9-A	100	McF_4	Inf	unif	wholeT_0.5	J1:DiP, J1:DiP-A, J1:OT, J1:OT-A, J5:DiP, J5:DiP-A, J5:OT, J5:OT-A, S1:DiP, S1:DiP-A, S5:DiP, S5:DiP-A, S5:OT, S5:OT-A
277	Yes	9	9-A	100	McF_6	0	last	singleC	J1:DiP, J5:DiP, J5:OT, S1:DiP, S5:DiP, S5:OT
278	Yes	9	9-A	100	McF_6	0	last	wholeT_0.01	J1:DiP, J5:DiP, J5:OT, S1:DiP, S5:DiP, S5:OT
279	Yes	9	9-A	100	McF_6	0	last	wholeT_0.5	J1:DiP, J5:DiP, J5:OT, S1:DiP, S5:DiP, S5:OT
280	Yes	9	9-A	100	McF_6	0	unif	singleC	J1:CBN, J1:CBN-A, J1:DiP, J1:DiP-A, J1:OT, J5:CBN, J5:CBN-A, J5:DiP, J5:DiP-A, J5:OT, J5:OT-A, S1:DiP, S1:DiP-A, S5:CBN, S5:CBN-A, S5:DiP, S5:DiP-A, S5:OT
281	Yes	9	9-A	100	McF_6	0	unif	wholeT_0.01	J1:DiP, J1:DiP-A, J5:CBN, J5:CBN-A, J5:DiP, J5:DiP-A, J5:OT, J5:OT-A, S5:CBN, S5:DiP, S5:DiP-A, S5:OT, S5:OT-A
282	Yes	9	9-A	100	McF_6	0	unif	wholeT_0.5	J1:CBN, J1:CBN-A, J1:DiP, J1:DiP-A, J1:OT, J5:CBN, J5:CBN-A, J5:DiP, J5:DiP-A, J5:OT, J5:OT-A, S1:DiP, S1:DiP-A, S5:CBN-A, S5:DiP, S5:DiP-A, S5:OT, S5:OT-A
283	Yes	9	9-A	100	McF_6	Inf	last	singleC	J1:DiP, J1:DiP-A, J1:OT, J5:DiP, J5:DiP-A, J5:OT, S1:DiP, S1:DiP-A, S5:DiP, S5:DiP-A, S5:OT
284	Yes	9	9-A	100	McF_6	Inf	last	wholeT_0.01	J1:DiP, J5:DiP, J5:OT, S1:DiP, S5:DiP, S5:OT

Table 8: *(continued)*

	Conjunction	Drivers	Tree	S.Size	Model	sh	S.Time	S.Type	Best method(s)
285	Yes	9	9-A	100	McF_6	Inf	last	wholeT_0.5	J1:DiP, J1:DiP-A, J5:DiP, J5:DiP-A, J5:OT, S1:DiP, S5:DiP, S5:DiP-A, S5:OT
286	Yes	9	9-A	100	McF_6	Inf	unif	singleC	J1:CBN-A, J1:DiP, J1:DiP-A, J5:CBN, J5:CBN-A, J5:DiP, J5:DiP-A, J5:OT, J5:OT-A, S1:DiP, S1:DiP-A, S5:CBN, S5:CBN-A, S5:DiP, S5:DiP-A, S5:OT, S5:OT-A
287	Yes	9	9-A	100	McF_6	Inf	unif	wholeT_0.01	J1:DiP, J5:CBN-A, J5:DiP, J5:DiP-A, J5:OT, J5:OT-A, S1:DiP, S5:CBN, S5:DiP, S5:OT, S5:OT-A
288	Yes	9	9-A	100	McF_6	Inf	unif	wholeT_0.5	J1:CBN, J1:CBN-A, J1:DiP, J1:DiP-A, J1:OT, J1:OT-A, J5:CBN, J5:CBN-A, J5:DiP, J5:DiP-A, J5:OT, J5:OT-A, S1:DiP, S1:DiP-A, S5:CBN-A, S5:DiP, S5:DiP-A, S5:OT, S5:OT-A
289	Yes	7	7-A	1000	Bozic	0	last	singleC	J1:CBN, J1:CBN-A, J1:DiP, J1:DiP-A, J1:OT, J1:OT-A, J5:CBN, J5:CBN-A, J5:DiP, J5:DiP-A, J5:OT, J5:OT-A, S1:DiP, S1:DiP-A, S1:OT, S1:OT-A, S5:DiP, S5:DiP-A, S5:OT, S5:OT-A
290	Yes	7	7-A	1000	Bozic	0	last	wholeT_0.01	none
291	Yes	7	7-A	1000	Bozic	0	last	wholeT_0.5	J1:CBN, J1:CBN-A, J1:DiP, J1:DiP-A, J1:OT, J1:OT-A, J5:CBN, J5:CBN-A, J5:DiP, J5:DiP-A, J5:OT, J5:OT-A, S1:DiP, S1:DiP-A, S1:OT, S1:OT-A, S5:CBN, S5:CBN-A, S5:DiP, S5:DiP-A, S5:OT, S5:OT-A
292	Yes	7	7-A	1000	Bozic	0	unif	singleC	none
293	Yes	7	7-A	1000	Bozic	0	unif	wholeT_0.01	J5:CBN, J5:CBN-A, J5:DiP, J5:DiP-A, J5:OT, J5:OT-A
294	Yes	7	7-A	1000	Bozic	0	unif	wholeT_0.5	none
295	Yes	7	7-A	1000	Bozic	Inf	last	singleC	J1:DiP, J1:DiP-A, J1:OT, J1:OT-A, J5:DiP, J5:DiP-A, J5:OT, J5:OT-A, S1:DiP, S1:DiP-A, S5:DiP, S5:DiP-A, S5:OT, S5:OT-A
296	Yes	7	7-A	1000	Bozic	Inf	last	wholeT_0.01	J5:CBN, J5:CBN-A, J5:DiP, J5:OT
297	Yes	7	7-A	1000	Bozic	Inf	last	wholeT_0.5	J1:DiP, J1:DiP-A, J1:OT, J1:OT-A, J5:DiP, J5:DiP-A, J5:OT, J5:OT-A, S1:DiP, S1:DiP-A, S5:DiP, S5:DiP-A, S5:OT, S5:OT-A

Table 8: *(continued)*

	Conjunction	Drivers	Tree	S.Size	Model	sh	S.Time	S.Type	Best method(s)
298	Yes	7	7-A	1000	Bozic	Inf	unif	singleC	J1:CBN, J1:CBN-A, J5:CBN, J5:CBN-A, J5:DiP, J5:DiP-A, J5:OT, J5:OT-A, S1:CBN, S1:CBN-A, S5:CBN, S5:CBN-A
299	Yes	7	7-A	1000	Bozic	Inf	unif	wholeT_0.01	J5:CBN, J5:CBN-A, S5:CBN, S5:CBN-A
300	Yes	7	7-A	1000	Bozic	Inf	unif	wholeT_0.5	J1:CBN, J1:CBN-A, J5:CBN, J5:CBN-A, J5:DiP, J5:DiP-A, J5:OT, J5:OT-A, S1:CBN, S1:CBN-A, S5:CBN, S5:CBN-A, S5:DiP, S5:DiP-A, S5:OT, S5:OT-A
301	Yes	7	7-A	1000	exp	0	last	singleC	J1:CBN, J1:CBN-A, J1:DiP, J1:DiP-A, J1:OT, J1:OT-A, J5:CBN, J5:CBN-A, J5:DiP, J5:DiP-A, J5:OT, J5:OT-A, S1:DiP, S1:DiP-A, S1:OT, S1:OT-A, S5:CBN, S5:CBN-A, S5:DiP, S5:DiP-A, S5:OT, S5:OT-A
302	Yes	7	7-A	1000	exp	0	last	wholeT_0.01	none
303	Yes	7	7-A	1000	exp	0	last	wholeT_0.5	J1:CBN, J1:CBN-A, J1:DiP, J1:DiP-A, J1:OT, J1:OT-A, J5:CBN, J5:CBN-A, J5:DiP, J5:DiP-A, J5:OT, J5:OT-A, S1:DiP, S1:DiP-A, S1:OT, S1:OT-A, S5:CBN, S5:CBN-A, S5:DiP, S5:DiP-A, S5:OT, S5:OT-A
304	Yes	7	7-A	1000	exp	0	unif	singleC	none
305	Yes	7	7-A	1000	exp	0	unif	wholeT_0.01	J1:CBN, J1:CBN-A, J1:DiP, J1:DiP-A, J1:OT, J1:OT-A, J5:CBN, J5:CBN-A, J5:DiP, J5:DiP-A, J5:OT, J5:OT-A, S5:CBN, S5:CBN-A, S5:DiP, S5:DiP-A, S5:OT, S5:OT-A
306	Yes	7	7-A	1000	exp	0	unif	wholeT_0.5	none
307	Yes	7	7-A	1000	exp	Inf	last	singleC	J1:DiP, J1:DiP-A, J1:OT, J1:OT-A, J5:CBN, J5:CBN-A, J5:DiP, J5:DiP-A, J5:OT, J5:OT-A, S1:DiP, S1:DiP-A, S5:DiP, S5:DiP-A, S5:OT, S5:OT-A
308	Yes	7	7-A	1000	exp	Inf	last	wholeT_0.01	J1:CBN-A, J5:CBN, J5:CBN-A, J5:DiP, J5:DiP-A, J5:OT, J5:OT-A, S5:CBN, S5:CBN-A, S5:DiP, S5:DiP-A, S5:OT, S5:OT-A

Table 8: *(continued)*

	Conjunction	Drivers	Tree	S.Size	Model	sh	S.Time	S.Type	Best method(s)
309	Yes	7	7-A	1000	exp	Inf	last	wholeT_0.5	J1:DiP, J1:DiP-A, J1:OT, J1:OT-A, J5:CBN, J5:CBN-A, J5:DiP, J5:DiP-A, J5:OT, J5:OT-A, S1:DiP, S1:DiP-A, S1:OT, S1:OT-A, S5:DiP, S5:DiP-A, S5:OT, S5:OT-A
310	Yes	7	7-A	1000	exp	Inf	unif	singleC	J1:CBN, J1:CBN-A, J1:DiP, J1:DiP-A, J1:OT, J1:OT-A, J5:CBN, J5:CBN-A, J5:DiP, J5:DiP-A, J5:OT, J5:OT-A, S1:CBN, S1:CBN-A, S1:DiP, S1:DiP-A, S5:CBN, S5:CBN-A, S5:DiP, S5:DiP-A, S5:OT, S5:OT-A
311	Yes	7	7-A	1000	exp	Inf	unif	wholeT_0.01	J1:CBN, J1:CBN-A, J5:CBN, J5:CBN-A, S5:CBN, S5:CBN-A
312	Yes	7	7-A	1000	exp	Inf	unif	wholeT_0.5	J1:CBN, J1:CBN-A, J1:DiP, J1:DiP-A, J1:OT, J1:OT-A, J5:CBN, J5:CBN-A, J5:DiP, J5:DiP-A, J5:OT, J5:OT-A, S1:CBN, S1:CBN-A, S5:CBN, S5:CBN-A, S5:DiP, S5:DiP-A, S5:OT, S5:OT-A
313	Yes	7	7-A	1000	McF_4	0	last	singleC	J1:DiP, J1:DiP-A, J1:OT, J1:OT-A, J5:DiP, J5:DiP-A, J5:OT, J5:OT-A, S1:DiP, S1:DiP-A, S1:OT, S1:OT-A, S5:DiP, S5:DiP-A, S5:OT, S5:OT-A
314	Yes	7	7-A	1000	McF_4	0	last	wholeT_0.01	J1:DiP-A, J1:OT, J1:OT-A, J5:DiP, J5:DiP-A, J5:OT, J5:OT-A, S5:OT, S5:OT-A
315	Yes	7	7-A	1000	McF_4	0	last	wholeT_0.5	J1:DiP, J1:DiP-A, J1:OT, J1:OT-A, J5:DiP, J5:DiP-A, J5:OT, J5:OT-A, S1:DiP, S1:DiP-A, S1:OT, S1:OT-A, S5:DiP, S5:DiP-A, S5:OT, S5:OT-A
316	Yes	7	7-A	1000	McF_4	0	unif	singleC	none
317	Yes	7	7-A	1000	McF_4	0	unif	wholeT_0.01	J1:CBN, J1:CBN-A, J1:DiP, J1:DiP-A, J1:OT, J1:OT-A, J5:CBN, J5:CBN-A, J5:DiP, J5:DiP-A, J5:OT, J5:OT-A, S1:DiP, S1:DiP-A, S5:CBN, S5:CBN-A, S5:DiP, S5:DiP-A, S5:OT, S5:OT-A
318	Yes	7	7-A	1000	McF_4	0	unif	wholeT_0.5	none
319	Yes	7	7-A	1000	McF_4	Inf	last	singleC	J1:DiP, J1:DiP-A, J1:OT, J1:OT-A, J5:DiP, J5:DiP-A, J5:OT, J5:OT-A, S1:DiP, S1:DiP-A, S1:OT, S1:OT-A, S5:DiP, S5:DiP-A, S5:OT, S5:OT-A

Table 8: *(continued)*

	Conjunction	Drivers	Tree	S.Size	Model	sh	S.Time	S.Type	Best method(s)
320	Yes	7	7-A	1000	McF_4	Inf	last	wholeT_0.01	J1:DiP, J1:DiP-A, J5:DiP, J5:DiP-A, J5:OT, J5:OT-A, S5:DiP, S5:DiP-A, S5:OT, S5:OT-A
321	Yes	7	7-A	1000	McF_4	Inf	last	wholeT_0.5	J1:DiP, J1:DiP-A, J1:OT, J1:OT-A, J5:DiP, J5:DiP-A, J5:OT, J5:OT-A, S1:DiP, S1:DiP-A, S1:OT, S1:OT-A, S5:DiP, S5:DiP-A, S5:OT, S5:OT-A
322	Yes	7	7-A	1000	McF_4	Inf	unif	singleC	none
323	Yes	7	7-A	1000	McF_4	Inf	unif	wholeT_0.01	J1:CBN, J1:CBN-A, J1:DiP, J1:DiP-A, J1:OT, J1:OT-A, J5:CBN, J5:CBN-A, J5:DiP, J5:DiP-A, J5:OT, J5:OT-A, S1:DiP, S1:DiP-A, S5:CBN, S5:CBN-A, S5:DiP, S5:DiP-A, S5:OT, S5:OT-A
324	Yes	7	7-A	1000	McF_4	Inf	unif	wholeT_0.5	none
325	Yes	7	7-A	1000	McF_6	0	last	singleC	J1:DiP, J1:OT, J1:OT-A, J5:DiP, J5:OT, J5:OT-A, S1:DiP, S5:DiP, S5:OT, S5:OT-A
326	Yes	7	7-A	1000	McF_6	0	last	wholeT_0.01	J1:DiP, J1:OT, J1:OT-A, J5:DiP, J5:OT, J5:OT-A, S1:DiP, S5:DiP, S5:OT, S5:OT-A
327	Yes	7	7-A	1000	McF_6	0	last	wholeT_0.5	J1:DiP, J1:OT, J1:OT-A, J5:DiP, J5:OT, J5:OT-A, S1:DiP, S5:DiP, S5:OT, S5:OT-A
328	Yes	7	7-A	1000	McF_6	0	unif	singleC	none
329	Yes	7	7-A	1000	McF_6	0	unif	wholeT_0.01	J1:CBN, J1:CBN-A, J1:DiP, J1:DiP-A, J1:OT, J1:OT-A, J5:CBN, J5:CBN-A, J5:DiP, J5:DiP-A, J5:OT, J5:OT-A, S5:CBN, S5:CBN-A, S5:DiP, S5:DiP-A, S5:OT, S5:OT-A
330	Yes	7	7-A	1000	McF_6	0	unif	wholeT_0.5	none
331	Yes	7	7-A	1000	McF_6	Inf	last	singleC	J1:DiP, J1:DiP-A, J1:OT, J1:OT-A, J5:DiP, J5:DiP-A, J5:OT, J5:OT-A, S1:DiP, S1:DiP-A, S5:DiP, S5:DiP-A, S5:OT, S5:OT-A
332	Yes	7	7-A	1000	McF_6	Inf	last	wholeT_0.01	J1:DiP, J5:DiP, J5:OT, J5:OT-A, S1:DiP, S5:DiP, S5:OT, S5:OT-A
333	Yes	7	7-A	1000	McF_6	Inf	last	wholeT_0.5	J1:DiP, J1:DiP-A, J1:OT, J1:OT-A, J5:DiP, J5:DiP-A, J5:OT, J5:OT-A, S1:DiP, S1:DiP-A, S5:DiP, S5:DiP-A, S5:OT, S5:OT-A

Table 8: *(continued)*

	Conjunction	Drivers	Tree	S.Size	Model	sh	S.Time	S.Type	Best method(s)
334	Yes	7	7-A	1000	McF_6	Inf	unif	singleC	none
335	Yes	7	7-A	1000	McF_6	Inf	unif	wholeT_0.01	J1:CBN, J1:CBN-A, J1:DiP, J1:DiP-A, J1:OT, J1:OT-A, J5:CBN, J5:CBN-A, J5:DiP, J5:DiP-A, J5:OT, J5:OT-A, S5:CBN, S5:CBN-A, S5:DiP, S5:DiP-A, S5:OT, S5:OT-A
336	Yes	7	7-A	1000	McF_6	Inf	unif	wholeT_0.5	none
337	Yes	7	7-A	200	Bozic	0	last	singleC	J1:CBN, J1:CBN-A, J1:DiP, J1:DiP-A, J1:OT, J1:OT-A, J5:CBN, J5:CBN-A, J5:DiP, J5:DiP-A, J5:OT, J5:OT-A, S1:DiP, S1:DiP-A, S1:OT, S1:OT-A, S5:CBN, S5:DiP, S5:DiP-A, S5:OT, S5:OT-A
338	Yes	7	7-A	200	Bozic	0	last	wholeT_0.01	J1:CBN, J1:CBN-A, J1:DiP, J1:DiP-A, J1:OT, J1:OT-A, J5:CBN, J5:CBN-A, J5:DiP, J5:DiP-A, J5:OT, J5:OT-A, S1:DiP, S1:DiP-A, S5:CBN, S5:CBN-A, S5:DiP, S5:DiP-A, S5:OT, S5:OT-A
339	Yes	7	7-A	200	Bozic	0	last	wholeT_0.5	J1:CBN, J1:CBN-A, J1:DiP, J1:DiP-A, J1:OT, J1:OT-A, J5:CBN, J5:CBN-A, J5:DiP, J5:DiP-A, J5:OT, J5:OT-A, S1:DiP, S1:DiP-A, S5:CBN, S5:CBN-A, S5:DiP, S5:DiP-A, S5:OT, S5:OT-A
340	Yes	7	7-A	200	Bozic	0	unif	singleC	J1:CBN, J1:CBN-A, J1:DiP, J1:DiP-A, J1:OT, J1:OT-A, J5:CBN, J5:CBN-A, J5:DiP, J5:DiP-A, J5:OT, J5:OT-A, S1:DiP, S1:DiP-A, S1:OT, S1:OT-A, S5:CBN, S5:CBN-A, S5:DiP, S5:DiP-A, S5:OT, S5:OT-A
341	Yes	7	7-A	200	Bozic	0	unif	wholeT_0.01	J5:CBN, J5:CBN-A, J5:DiP, J5:DiP-A, J5:OT, J5:OT-A
342	Yes	7	7-A	200	Bozic	0	unif	wholeT_0.5	none
343	Yes	7	7-A	200	Bozic	Inf	last	singleC	J1:DiP, J1:DiP-A, J5:DiP, J5:DiP-A, J5:OT, J5:OT-A, S5:DiP, S5:DiP-A, S5:OT, S5:OT-A
344	Yes	7	7-A	200	Bozic	Inf	last	wholeT_0.01	J5:CBN-A, J5:DiP, J5:DiP-A, J5:OT
345	Yes	7	7-A	200	Bozic	Inf	last	wholeT_0.5	J1:DiP, J1:DiP-A, J5:DiP, J5:DiP-A, J5:OT, J5:OT-A, S1:DiP, S1:DiP-A, S5:DiP, S5:DiP-A, S5:OT, S5:OT-A

Table 8: *(continued)*

	Conjunction	Drivers	Tree	S.Size	Model	sh	S.Time	S.Type	Best method(s)
346	Yes	7	7-A	200	Bozic	Inf	unif	singleC	J1:CBN, J1:CBN-A, J5:CBN, J5:CBN-A, J5:DiP, J5:DiP-A, J5:OT, J5:OT-A, S5:CBN, S5:CBN-A, S5:DiP, S5:DiP-A
347	Yes	7	7-A	200	Bozic	Inf	unif	wholeT_0.01	J5:CBN, J5:CBN-A, S5:CBN, S5:CBN-A
348	Yes	7	7-A	200	Bozic	Inf	unif	wholeT_0.5	J1:CBN, J1:CBN-A, J5:CBN, J5:CBN-A, J5:DiP, J5:DiP-A, J5:OT, J5:OT-A, S5:CBN, S5:CBN-A, S5:DiP, S5:DiP-A, S5:OT, S5:OT-A
349	Yes	7	7-A	200	exp	0	last	singleC	J1:CBN, J1:CBN-A, J1:DiP, J1:DiP-A, J1:OT, J1:OT-A, J5:CBN, J5:CBN-A, J5:DiP, J5:DiP-A, J5:OT, J5:OT-A, S1:DiP, S1:DiP-A, S1:OT, S1:OT-A, S5:CBN, S5:CBN-A, S5:DiP, S5:DiP-A, S5:OT, S5:OT-A
350	Yes	7	7-A	200	exp	0	last	wholeT_0.01	J1:CBN, J1:CBN-A, J1:DiP, J1:DiP-A, J1:OT, J1:OT-A, J5:CBN, J5:CBN-A, J5:DiP, J5:DiP-A, J5:OT, J5:OT-A, S1:CBN, S1:CBN-A, S1:DiP, S1:DiP-A, S5:CBN, S5:DiP, S5:DiP-A
351	Yes	7	7-A	200	exp	0	last	wholeT_0.5	J1:CBN, J1:CBN-A, J1:DiP, J1:DiP-A, J1:OT, J1:OT-A, J5:CBN, J5:CBN-A, J5:DiP, J5:DiP-A, J5:OT, J5:OT-A, S1:CBN, S1:DiP, S1:DiP-A, S1:OT, S1:OT-A, S5:CBN, S5:CBN-A, S5:DiP, S5:DiP-A, S5:OT, S5:OT-A
352	Yes	7	7-A	200	exp	0	unif	singleC	none
353	Yes	7	7-A	200	exp	0	unif	wholeT_0.01	J1:CBN, J1:CBN-A, J1:DiP, J1:DiP-A, J1:OT, J1:OT-A, J5:CBN, J5:CBN-A, J5:DiP, J5:DiP-A, J5:OT, J5:OT-A, S1:DiP, S1:DiP-A, S5:CBN, S5:CBN-A, S5:DiP, S5:DiP-A, S5:OT, S5:OT-A
354	Yes	7	7-A	200	exp	0	unif	wholeT_0.5	none
355	Yes	7	7-A	200	exp	Inf	last	singleC	J1:DiP, J1:DiP-A, J1:OT, J1:OT-A, J5:CBN, J5:CBN-A, J5:DiP, J5:DiP-A, J5:OT, J5:OT-A, S1:DiP, S1:DiP-A, S5:DiP, S5:DiP-A, S5:OT, S5:OT-A
356	Yes	7	7-A	200	exp	Inf	last	wholeT_0.01	J1:DiP, J5:CBN, J5:CBN-A, J5:DiP, J5:DiP-A, J5:OT, J5:OT-A, S1:DiP, S5:CBN-A, S5:DiP, S5:DiP-A

Table 8: *(continued)*

	Conjunction	Drivers	Tree	S.Size	Model	sh	S.Time	S.Type	Best method(s)
357	Yes	7	7-A	200	exp	Inf	last	wholeT_0.5	J1:DiP, J1:DiP-A, J1:OT, J1:OT-A, J5:CBN, J5:CBN-A, J5:DiP, J5:DiP-A, J5:OT, J5:OT-A, S1:DiP, S1:DiP-A, S5:DiP, S5:DiP-A, S5:OT, S5:OT-A
358	Yes	7	7-A	200	exp	Inf	unif	singleC	J1:CBN, J1:CBN-A, J1:DiP, J1:DiP-A, J1:OT, J1:OT-A, J5:CBN, J5:CBN-A, J5:DiP, J5:DiP-A, J5:OT, J5:OT-A, S1:CBN-A, S1:DiP, S1:DiP-A, S5:CBN, S5:CBN-A, S5:DiP, S5:DiP-A, S5:OT, S5:OT-A
359	Yes	7	7-A	200	exp	Inf	unif	wholeT_0.01	J5:CBN, J5:CBN-A, J5:DiP-A, J5:OT, J5:OT-A, S5:CBN, S5:CBN-A
360	Yes	7	7-A	200	exp	Inf	unif	wholeT_0.5	J1:CBN, J1:CBN-A, J1:DiP, J1:DiP-A, J1:OT, J1:OT-A, J5:CBN, J5:CBN-A, J5:DiP, J5:DiP-A, J5:OT, J5:OT-A, S1:CBN-A, S1:DiP, S1:DiP-A, S5:CBN, S5:CBN-A, S5:DiP, S5:DiP-A, S5:OT, S5:OT-A
361	Yes	7	7-A	200	McF_4	0	last	singleC	J1:DiP, J1:DiP-A, J1:OT, J1:OT-A, J5:DiP, J5:DiP-A, J5:OT, J5:OT-A, S5:DiP, S5:DiP-A, S5:OT, S5:OT-A
362	Yes	7	7-A	200	McF_4	0	last	wholeT_0.01	J1:DiP, J1:DiP-A, J5:DiP, J5:DiP-A, J5:OT, J5:OT-A, S1:DiP, S1:DiP-A, S5:DiP, S5:DiP-A, S5:OT, S5:OT-A
363	Yes	7	7-A	200	McF_4	0	last	wholeT_0.5	J1:DiP, J1:DiP-A, J1:OT, J1:OT-A, J5:DiP, J5:DiP-A, J5:OT, J5:OT-A, S1:DiP, S1:DiP-A, S5:DiP, S5:DiP-A, S5:OT, S5:OT-A
364	Yes	7	7-A	200	McF_4	0	unif	singleC	J1:CBN, J1:CBN-A, J1:DiP, J1:DiP-A, J1:OT, J1:OT-A, J5:CBN, J5:CBN-A, J5:DiP, J5:DiP-A, J5:OT, J5:OT-A, S1:DiP, S1:DiP-A, S5:CBN, S5:CBN-A, S5:DiP, S5:DiP-A, S5:OT, S5:OT-A
365	Yes	7	7-A	200	McF_4	0	unif	wholeT_0.01	J1:CBN, J1:CBN-A, J1:DiP, J1:DiP-A, J1:OT, J1:OT-A, J5:CBN, J5:CBN-A, J5:DiP, J5:DiP-A, J5:OT, J5:OT-A, S1:DiP, S1:DiP-A, S5:CBN, S5:CBN-A, S5:DiP, S5:DiP-A, S5:OT, S5:OT-A

Table 8: *(continued)*

	Conjunction	Drivers	Tree	S.Size	Model	sh	S.Time	S.Type	Best method(s)
366	Yes	7	7-A	200	McF_4	0	unif	wholeT_0.5	J1:CBN, J1:CBN-A, J1:DiP, J1:DiP-A, J1:OT, J1:OT-A, J5:CBN, J5:CBN-A, J5:DiP, J5:DiP-A, J5:OT, J5:OT-A, S1:DiP, S1:DiP-A, S5:CBN, S5:CBN-A, S5:DiP, S5:DiP-A, S5:OT, S5:OT-A
367	Yes	7	7-A	200	McF_4	Inf	last	singleC	J1:DiP, J1:DiP-A, J1:OT, J1:OT-A, J5:DiP, J5:DiP-A, J5:OT, J5:OT-A, S1:DiP, S1:DiP-A, S5:DiP, S5:DiP-A, S5:OT, S5:OT-A
368	Yes	7	7-A	200	McF_4	Inf	last	wholeT_0.01	J1:DiP, J1:DiP-A, J5:DiP, J5:DiP-A, J5:OT, J5:OT-A, S1:DiP, S1:DiP-A, S5:DiP, S5:DiP-A, S5:OT, S5:OT-A
369	Yes	7	7-A	200	McF_4	Inf	last	wholeT_0.5	J1:DiP, J1:DiP-A, J1:OT, J1:OT-A, J5:DiP, J5:DiP-A, J5:OT, J5:OT-A, S1:DiP, S1:DiP-A, S5:DiP, S5:DiP-A, S5:OT, S5:OT-A
370	Yes	7	7-A	200	McF_4	Inf	unif	singleC	J1:CBN, J1:CBN-A, J1:DiP, J1:DiP-A, J1:OT, J1:OT-A, J5:CBN, J5:CBN-A, J5:DiP, J5:DiP-A, J5:OT, J5:OT-A, S1:DiP, S1:DiP-A, S5:CBN, S5:CBN-A, S5:DiP, S5:DiP-A, S5:OT, S5:OT-A
371	Yes	7	7-A	200	McF_4	Inf	unif	wholeT_0.01	J1:CBN, J1:CBN-A, J1:DiP, J1:DiP-A, J1:OT, J1:OT-A, J5:CBN, J5:CBN-A, J5:DiP, J5:DiP-A, J5:OT, J5:OT-A, S1:DiP, S1:DiP-A, S5:CBN, S5:CBN-A, S5:DiP, S5:DiP-A, S5:OT, S5:OT-A
372	Yes	7	7-A	200	McF_4	Inf	unif	wholeT_0.5	J1:CBN, J1:CBN-A, J1:DiP, J1:DiP-A, J1:OT, J1:OT-A, J5:CBN, J5:CBN-A, J5:DiP, J5:DiP-A, J5:OT, J5:OT-A, S1:DiP, S1:DiP-A, S5:CBN, S5:CBN-A, S5:DiP, S5:DiP-A, S5:OT, S5:OT-A
373	Yes	7	7-A	200	McF_6	0	last	singleC	J1:DiP, J1:OT, J1:OT-A, J5:DiP, J5:OT, J5:OT-A, S1:DiP, S5:DiP, S5:OT, S5:OT-A
374	Yes	7	7-A	200	McF_6	0	last	wholeT_0.01	J1:DiP, J5:DiP, J5:OT, J5:OT-A, S1:DiP, S5:DiP, S5:OT, S5:OT-A
375	Yes	7	7-A	200	McF_6	0	last	wholeT_0.5	J1:DiP, J1:OT, J1:OT-A, J5:DiP, J5:OT, J5:OT-A, S1:DiP, S5:DiP, S5:OT, S5:OT-A

Table 8: *(continued)*

	Conjunction	Drivers	Tree	S.Size	Model	sh	S.Time	S.Type	Best method(s)
376	Yes	7	7-A	200	McF_6	0	unif	singleC	J1:CBN, J1:CBN-A, J1:DiP, J1:DiP-A, J5:CBN, J5:CBN-A, J5:DiP, J5:DiP-A, S1:DiP, S1:DiP-A, S5:CBN, S5:CBN-A, S5:DiP, S5:DiP-A
377	Yes	7	7-A	200	McF_6	0	unif	wholeT_0.01	J1:CBN, J1:CBN-A, J1:DiP, J1:DiP-A, J1:OT, J1:OT-A, J5:CBN, J5:CBN-A, J5:DiP, J5:DiP-A, J5:OT, J5:OT-A, S1:DiP, S1:DiP-A, S5:CBN, S5:CBN-A, S5:DiP, S5:DiP-A, S5:OT, S5:OT-A
378	Yes	7	7-A	200	McF_6	0	unif	wholeT_0.5	J1:CBN, J1:CBN-A, J1:DiP, J1:DiP-A, J5:CBN, J5:CBN-A, J5:DiP, J5:DiP-A, S1:DiP, S1:DiP-A, S5:CBN, S5:CBN-A, S5:DiP, S5:DiP-A
379	Yes	7	7-A	200	McF_6	Inf	last	singleC	J1:DiP, J1:DiP-A, J5:DiP, J5:DiP-A, J5:OT, J5:OT-A, S1:DiP, S5:DiP, S5:DiP-A, S5:OT, S5:OT-A
380	Yes	7	7-A	200	McF_6	Inf	last	wholeT_0.01	J1:DiP, J5:DiP, J5:OT, J5:OT-A, S1:DiP, S5:DiP, S5:OT, S5:OT-A
381	Yes	7	7-A	200	McF_6	Inf	last	wholeT_0.5	J1:DiP, J1:DiP-A, J1:OT, J1:OT-A, J5:DiP, J5:DiP-A, J5:OT, J5:OT-A, S1:DiP, S5:DiP, S5:DiP-A, S5:OT, S5:OT-A
382	Yes	7	7-A	200	McF_6	Inf	unif	singleC	J1:CBN, J1:CBN-A, J1:DiP, J1:DiP-A, J5:CBN, J5:CBN-A, J5:DiP, J5:DiP-A, S1:DiP, S1:DiP-A, S5:CBN, S5:CBN-A, S5:DiP, S5:DiP-A
383	Yes	7	7-A	200	McF_6	Inf	unif	wholeT_0.01	J1:CBN, J1:CBN-A, J1:DiP, J1:DiP-A, J5:CBN, J5:CBN-A, J5:DiP, J5:DiP-A, S1:DiP, S1:DiP-A, S5:CBN, S5:CBN-A, S5:DiP, S5:DiP-A
384	Yes	7	7-A	200	McF_6	Inf	unif	wholeT_0.5	J1:CBN, J1:CBN-A, J1:DiP, J1:DiP-A, J5:CBN, J5:CBN-A, J5:DiP, J5:DiP-A, S1:DiP, S1:DiP-A, S5:CBN, S5:CBN-A, S5:DiP, S5:DiP-A
385	Yes	7	7-A	100	Bozic	0	last	singleC	J1:DiP, J1:DiP-A, J5:CBN, J5:CBN-A, J5:DiP, J5:DiP-A, J5:OT, J5:OT-A, S1:DiP, S1:DiP-A, S5:CBN, S5:DiP, S5:DiP-A, S5:OT, S5:OT-A

Table 8: *(continued)*

	Conjunction	Drivers	Tree	S.Size	Model	sh	S.Time	S.Type	Best method(s)
386	Yes	7	7-A	100	Bozic	0	last	wholeT_0.01	J1:DiP, J1:DiP-A, J5:CBN, J5:CBN-A, J5:DiP, J5:DiP-A, J5:OT, J5:OT-A, S1:DiP, S1:DiP-A, S5:CBN, S5:CBN-A, S5:DiP, S5:DiP-A, S5:OT, S5:OT-A
387	Yes	7	7-A	100	Bozic	0	last	wholeT_0.5	J1:CBN, J1:CBN-A, J1:DiP, J1:DiP-A, J5:CBN, J5:CBN-A, J5:DiP, J5:DiP-A, J5:OT, J5:OT-A, S1:DiP, S1:DiP-A, S5:CBN, S5:CBN-A, S5:DiP, S5:DiP-A, S5:OT, S5:OT-A
388	Yes	7	7-A	100	Bozic	0	unif	singleC	J1:CBN, J1:CBN-A, J1:DiP, J1:DiP-A, J1:OT, J1:OT-A, J5:CBN, J5:CBN-A, J5:DiP, J5:DiP-A, J5:OT, J5:OT-A, S1:DiP, S1:DiP-A, S5:CBN, S5:CBN-A, S5:DiP, S5:DiP-A, S5:OT, S5:OT-A
389	Yes	7	7-A	100	Bozic	0	unif	wholeT_0.01	J5:CBN, J5:CBN-A, J5:DiP, J5:DiP-A, J5:OT, J5:OT-A
390	Yes	7	7-A	100	Bozic	0	unif	wholeT_0.5	J1:CBN, J1:CBN-A, J1:DiP, J1:DiP-A, J1:OT, J1:OT-A, J5:CBN, J5:CBN-A, J5:DiP, J5:DiP-A, J5:OT, J5:OT-A, S1:DiP, S1:DiP-A, S5:CBN, S5:CBN-A, S5:DiP, S5:DiP-A, S5:OT, S5:OT-A
391	Yes	7	7-A	100	Bozic	Inf	last	singleC	J1:DiP, J1:DiP-A, J5:DiP, J5:DiP-A, J5:OT, J5:OT-A, S1:DiP, S1:DiP-A, S5:DiP, S5:DiP-A
392	Yes	7	7-A	100	Bozic	Inf	last	wholeT_0.01	J5:CBN, J5:DiP, J5:DiP-A
393	Yes	7	7-A	100	Bozic	Inf	last	wholeT_0.5	J1:DiP, J1:DiP-A, J5:DiP, J5:DiP-A, J5:OT, J5:OT-A, S1:DiP, S1:DiP-A, S5:DiP, S5:DiP-A
394	Yes	7	7-A	100	Bozic	Inf	unif	singleC	J5:CBN, J5:CBN-A, J5:DiP, J5:DiP-A, J5:OT, J5:OT-A, S5:CBN, S5:CBN-A, S5:DiP, S5:DiP-A
395	Yes	7	7-A	100	Bozic	Inf	unif	wholeT_0.01	J5:CBN, J5:CBN-A, J5:OT, J5:OT-A, S5:CBN, S5:CBN-A
396	Yes	7	7-A	100	Bozic	Inf	unif	wholeT_0.5	J5:CBN, J5:CBN-A, J5:DiP, J5:DiP-A, J5:OT, J5:OT-A, S5:CBN, S5:CBN-A, S5:DiP, S5:DiP-A

Table 8: *(continued)*

	Conjunction	Drivers	Tree	S.Size	Model	sh	S.Time	S.Type	Best method(s)
397	Yes	7	7-A	100	exp	0	last	singleC	J1:CBN, J1:CBN-A, J1:DiP, J1:DiP-A, J1:OT, J1:OT-A, J5:CBN, J5:CBN-A, J5:DiP, J5:DiP-A, J5:OT, J5:OT-A, S1:DiP, S1:DiP-A, S1:OT, S1:OT-A, S5:CBN, S5:CBN-A, S5:DiP, S5:DiP-A, S5:OT, S5:OT-A
398	Yes	7	7-A	100	exp	0	last	wholeT_0.01	J1:CBN, J1:CBN-A, J1:DiP, J1:DiP-A, J5:CBN, J5:CBN-A, J5:DiP, J5:DiP-A, J5:OT, J5:OT-A, S1:DiP, S1:DiP-A, S5:CBN, S5:DiP, S5:DiP-A
399	Yes	7	7-A	100	exp	0	last	wholeT_0.5	J1:CBN, J1:CBN-A, J1:DiP, J1:DiP-A, J1:OT, J1:OT-A, J5:CBN, J5:CBN-A, J5:DiP, J5:DiP-A, J5:OT, J5:OT-A, S1:DiP, S1:DiP-A, S1:OT, S1:OT-A, S5:CBN, S5:CBN-A, S5:DiP, S5:DiP-A, S5:OT, S5:OT-A
400	Yes	7	7-A	100	exp	0	unif	singleC	none
401	Yes	7	7-A	100	exp	0	unif	wholeT_0.01	J1:DiP, J1:DiP-A, J5:CBN, J5:CBN-A, J5:DiP, J5:DiP-A, J5:OT, J5:OT-A, S1:DiP, S1:DiP-A, S5:CBN, S5:CBN-A, S5:DiP, S5:DiP-A, S5:OT, S5:OT-A
402	Yes	7	7-A	100	exp	0	unif	wholeT_0.5	none
403	Yes	7	7-A	100	exp	Inf	last	singleC	J1:DiP, J1:DiP-A, J1:OT, J1:OT-A, J5:CBN, J5:CBN-A, J5:DiP, J5:DiP-A, J5:OT, J5:OT-A, S1:DiP, S1:DiP-A, S5:DiP, S5:DiP-A, S5:OT, S5:OT-A
404	Yes	7	7-A	100	exp	Inf	last	wholeT_0.01	J5:DiP, J5:DiP-A, S5:DiP
405	Yes	7	7-A	100	exp	Inf	last	wholeT_0.5	J1:CBN, J1:DiP, J1:DiP-A, J1:OT, J1:OT-A, J5:CBN, J5:CBN-A, J5:DiP, J5:DiP-A, J5:OT, J5:OT-A, S1:DiP, S1:DiP-A, S5:DiP, S5:DiP-A, S5:OT, S5:OT-A
406	Yes	7	7-A	100	exp	Inf	unif	singleC	J1:DiP, J1:DiP-A, J5:CBN, J5:CBN-A, J5:DiP, J5:DiP-A, J5:OT, J5:OT-A, S1:DiP, S1:DiP-A, S5:CBN, S5:CBN-A, S5:DiP, S5:DiP-A, S5:OT, S5:OT-A
407	Yes	7	7-A	100	exp	Inf	unif	wholeT_0.01	J5:CBN, J5:CBN-A, J5:OT, J5:OT-A, S5:CBN, S5:CBN-A, S5:OT, S5:OT-A

Table 8: *(continued)*

	Conjunction	Drivers	Tree	S.Size	Model	sh	S.Time	S.Type	Best method(s)
408	Yes	7	7-A	100	exp	Inf	unif	wholeT_0.5	J1:CBN-A, J1:DiP, J1:DiP-A, J5:CBN, J5:CBN-A, J5:DiP, J5:DiP-A, J5:OT, J5:OT-A, S1:DiP, S1:DiP-A, S5:CBN, S5:CBN-A, S5:DiP, S5:DiP-A, S5:OT, S5:OT-A
409	Yes	7	7-A	100	McF_4	0	last	singleC	J1:DiP, J1:DiP-A, J1:OT, J1:OT-A, J5:DiP, J5:DiP-A, J5:OT, J5:OT-A, S1:DiP, S1:DiP-A, S5:DiP, S5:DiP-A, S5:OT, S5:OT-A
410	Yes	7	7-A	100	McF_4	0	last	wholeT_0.01	J1:DiP, J1:DiP-A, J5:DiP, J5:DiP-A, J5:OT, J5:OT-A, S5:DiP, S5:DiP-A, S5:OT, S5:OT-A
411	Yes	7	7-A	100	McF_4	0	last	wholeT_0.5	J1:DiP, J1:DiP-A, J1:OT, J1:OT-A, J5:DiP, J5:DiP-A, J5:OT, J5:OT-A, S1:DiP, S1:DiP-A, S5:DiP, S5:DiP-A, S5:OT, S5:OT-A
412	Yes	7	7-A	100	McF_4	0	unif	singleC	J1:CBN, J1:CBN-A, J1:DiP, J1:DiP-A, J1:OT, J1:OT-A, J5:CBN, J5:CBN-A, J5:DiP, J5:DiP-A, J5:OT, J5:OT-A, S1:DiP, S1:DiP-A, S5:CBN, S5:CBN-A, S5:DiP, S5:DiP-A, S5:OT, S5:OT-A
413	Yes	7	7-A	100	McF_4	0	unif	wholeT_0.01	J1:DiP, J1:DiP-A, J5:CBN, J5:CBN-A, J5:DiP, J5:DiP-A, J5:OT, J5:OT-A, S5:CBN, S5:CBN-A, S5:DiP, S5:DiP-A, S5:OT, S5:OT-A
414	Yes	7	7-A	100	McF_4	0	unif	wholeT_0.5	J1:CBN, J1:CBN-A, J1:DiP, J1:DiP-A, J1:OT, J1:OT-A, J5:CBN, J5:CBN-A, J5:DiP, J5:DiP-A, J5:OT, J5:OT-A, S1:DiP, S1:DiP-A, S5:CBN, S5:CBN-A, S5:DiP, S5:DiP-A, S5:OT, S5:OT-A
415	Yes	7	7-A	100	McF_4	Inf	last	singleC	J1:DiP, J1:DiP-A, J1:OT, J1:OT-A, J5:DiP, J5:DiP-A, J5:OT, J5:OT-A, S1:DiP, S1:DiP-A, S5:DiP, S5:DiP-A, S5:OT, S5:OT-A
416	Yes	7	7-A	100	McF_4	Inf	last	wholeT_0.01	J1:DiP, J1:DiP-A, J5:DiP, J5:DiP-A, J5:OT, J5:OT-A, S1:DiP, S1:DiP-A, S5:DiP, S5:DiP-A, S5:OT, S5:OT-A
417	Yes	7	7-A	100	McF_4	Inf	last	wholeT_0.5	J1:DiP, J1:DiP-A, J5:DiP, J5:DiP-A, J5:OT, J5:OT-A, S1:DiP, S1:DiP-A, S5:DiP, S5:DiP-A, S5:OT, S5:OT-A

Table 8: *(continued)*

	Conjunction	Drivers	Tree	S.Size	Model	sh	S.Time	S.Type	Best method(s)
418	Yes	7	7-A	100	McF_4	Inf	unif	singleC	J1:CBN, J1:CBN-A, J1:DiP, J1:DiP-A, J1:OT, J1:OT-A, J5:CBN, J5:CBN-A, J5:DiP, J5:DiP-A, J5:OT, J5:OT-A, S1:DiP, S1:DiP-A, S5:CBN, S5:CBN-A, S5:DiP, S5:DiP-A, S5:OT, S5:OT-A
419	Yes	7	7-A	100	McF_4	Inf	unif	wholeT_0.01	J1:CBN, J1:CBN-A, J1:DiP, J1:DiP-A, J5:CBN, J5:CBN-A, J5:DiP, J5:DiP-A, J5:OT, J5:OT-A, S5:CBN, S5:CBN-A, S5:DiP, S5:DiP-A, S5:OT, S5:OT-A
420	Yes	7	7-A	100	McF_4	Inf	unif	wholeT_0.5	J1:CBN, J1:CBN-A, J1:DiP, J1:DiP-A, J1:OT, J1:OT-A, J5:CBN, J5:CBN-A, J5:DiP, J5:DiP-A, J5:OT, J5:OT-A, S1:DiP, S1:DiP-A, S5:CBN, S5:CBN-A, S5:DiP, S5:DiP-A, S5:OT, S5:OT-A
421	Yes	7	7-A	100	McF_6	0	last	singleC	J1:DiP, J1:DiP-A, J5:DiP, J5:DiP-A, J5:OT, J5:OT-A, S1:DiP, S1:DiP-A, S5:DiP, S5:DiP-A, S5:OT, S5:OT-A
422	Yes	7	7-A	100	McF_6	0	last	wholeT_0.01	J1:DiP, J5:DiP, J5:OT, J5:OT-A, S1:DiP, S5:DiP, S5:OT, S5:OT-A
423	Yes	7	7-A	100	McF_6	0	last	wholeT_0.5	J1:DiP, J5:DiP, J5:DiP-A, J5:OT, J5:OT-A, S1:DiP, S5:DiP, S5:DiP-A, S5:OT, S5:OT-A
424	Yes	7	7-A	100	McF_6	0	unif	singleC	J1:CBN, J1:CBN-A, J1:DiP, J1:DiP-A, J5:CBN, J5:CBN-A, J5:DiP, J5:DiP-A, S1:DiP, S1:DiP-A, S5:CBN, S5:CBN-A, S5:DiP, S5:DiP-A
425	Yes	7	7-A	100	McF_6	0	unif	wholeT_0.01	J1:DiP, J5:CBN, J5:CBN-A, J5:DiP, J5:DiP-A, S5:CBN, S5:CBN-A, S5:DiP, S5:DiP-A
426	Yes	7	7-A	100	McF_6	0	unif	wholeT_0.5	J1:CBN, J1:CBN-A, J1:DiP, J1:DiP-A, J5:CBN, J5:CBN-A, J5:DiP, J5:DiP-A, S1:DiP, S1:DiP-A, S5:CBN, S5:CBN-A, S5:DiP, S5:DiP-A
427	Yes	7	7-A	100	McF_6	Inf	last	singleC	J1:DiP, J1:DiP-A, J5:DiP, J5:DiP-A, J5:OT, J5:OT-A, S1:DiP, S1:DiP-A, S5:DiP, S5:DiP-A, S5:OT, S5:OT-A
428	Yes	7	7-A	100	McF_6	Inf	last	wholeT_0.01	J5:DiP, J5:OT, J5:OT-A, S1:DiP, S5:DiP, S5:OT, S5:OT-A

Table 8: *(continued)*

	Conjunction	Drivers	Tree	S.Size	Model	sh	S.Time	S.Type	Best method(s)
429	Yes	7	7-A	100	McF_6	Inf	last	wholeT_0.5	J1:DiP, J1:DiP-A, J5:DiP, J5:DiP-A, J5:OT, J5:OT-A, S1:DiP, S1:DiP-A, S5:DiP, S5:DiP-A, S5:OT, S5:OT-A
430	Yes	7	7-A	100	McF_6	Inf	unif	singleC	J1:DiP, J1:DiP-A, J5:CBN, J5:CBN-A, J5:DiP, J5:DiP-A, S1:DiP, S1:DiP-A, S5:CBN, S5:CBN-A, S5:DiP, S5:DiP-A
431	Yes	7	7-A	100	McF_6	Inf	unif	wholeT_0.01	J1:DiP, J1:DiP-A, J5:CBN, J5:CBN-A, J5:DiP, J5:DiP-A, S5:CBN, S5:CBN-A, S5:DiP, S5:DiP-A
432	Yes	7	7-A	100	McF_6	Inf	unif	wholeT_0.5	J1:CBN, J1:CBN-A, J1:DiP, J1:DiP-A, J5:CBN, J5:CBN-A, J5:DiP, J5:DiP-A, S1:DiP, S1:DiP-A, S5:CBN, S5:CBN-A, S5:DiP, S5:DiP-A
433	No	11	11-B	1000	Bozic	0	last	singleC	J1:DiP, J1:DiP-A, J1:OT, J1:OT-A, J5:DiP, J5:DiP-A, J5:OT, J5:OT-A, S1:OT, S1:OT-A, S5:DiP, S5:DiP-A, S5:OT, S5:OT-A
434	No	11	11-B	1000	Bozic	0	last	wholeT_0.01	J1:DiP, J1:DiP-A, J5:DiP, J5:DiP-A, S1:DiP, S1:DiP-A, S5:DiP, S5:DiP-A
435	No	11	11-B	1000	Bozic	0	last	wholeT_0.5	J1:DiP, J1:DiP-A, J1:OT, J1:OT-A, J5:DiP, J5:DiP-A, J5:OT, J5:OT-A, S1:OT, S1:OT-A, S5:DiP, S5:DiP-A, S5:OT, S5:OT-A
436	No	11	11-B	1000	Bozic	0	unif	singleC	J1:DiP, J1:DiP-A, J1:OT, J1:OT-A, J5:CBN, J5:CBN-A, J5:DiP, J5:DiP-A, J5:OT, J5:OT-A, S1:DiP, S1:DiP-A, S1:OT, S1:OT-A, S5:DiP, S5:DiP-A, S5:OT, S5:OT-A
437	No	11	11-B	1000	Bozic	0	unif	wholeT_0.01	J5:DiP, J5:DiP-A, J5:OT, J5:OT-A, S5:OT, S5:OT-A
438	No	11	11-B	1000	Bozic	0	unif	wholeT_0.5	J1:CBN, J1:CBN-A, J1:DiP, J1:DiP-A, J1:OT, J1:OT-A, J5:CBN, J5:CBN-A, J5:DiP, J5:DiP-A, J5:OT, J5:OT-A, S1:DiP, S1:DiP-A, S1:OT, S1:OT-A, S5:DiP, S5:DiP-A, S5:OT, S5:OT-A
439	No	11	11-B	1000	Bozic	Inf	last	singleC	J1:DiP, J1:DiP-A, J1:OT, J1:OT-A, J5:DiP, J5:DiP-A, J5:OT, J5:OT-A, S5:DiP, S5:DiP-A, S5:OT, S5:OT-A
440	No	11	11-B	1000	Bozic	Inf	last	wholeT_0.01	J5:DiP, J5:DiP-A, J5:OT, J5:OT-A

Table 8: *(continued)*

	Conjunction	Drivers	Tree	S.Size	Model	sh	S.Time	S.Type	Best method(s)
441	No	11	11-B	1000	Bozic	Inf	last	wholeT_0.5	J1:DiP, J1:DiP-A, J1:OT, J1:OT-A, J5:DiP, J5:DiP-A, J5:OT, J5:OT-A, S5:DiP, S5:DiP-A, S5:OT, S5:OT-A
442	No	11	11-B	1000	Bozic	Inf	unif	singleC	J1:DiP, J1:DiP-A, J1:OT, J1:OT-A, J5:DiP, J5:DiP-A, J5:OT, J5:OT-A, S5:DiP, S5:DiP-A, S5:OT, S5:OT-A
443	No	11	11-B	1000	Bozic	Inf	unif	wholeT_0.01	J5:DiP, J5:DiP-A, J5:OT, J5:OT-A
444	No	11	11-B	1000	Bozic	Inf	unif	wholeT_0.5	J1:DiP, J1:DiP-A, J1:OT, J1:OT-A, J5:DiP, J5:DiP-A, J5:OT, J5:OT-A, S5:DiP, S5:DiP-A, S5:OT, S5:OT-A
445	No	11	11-B	1000	exp	0	last	singleC	J1:DiP, J1:DiP-A, J1:OT, J1:OT-A, J5:CBN, J5:CBN-A, J5:DiP, J5:DiP-A, J5:OT, J5:OT-A, S1:DiP, S1:DiP-A, S1:OT, S1:OT-A, S5:DiP, S5:DiP-A, S5:OT, S5:OT-A
446	No	11	11-B	1000	exp	0	last	wholeT_0.01	J1:DiP, J1:DiP-A, J1:OT, J1:OT-A, J5:DiP, J5:DiP-A, J5:OT, J5:OT-A, S1:DiP-A, S1:OT, S1:OT-A, S5:DiP, S5:DiP-A, S5:OT, S5:OT-A
447	No	11	11-B	1000	exp	0	last	wholeT_0.5	J1:DiP, J1:DiP-A, J1:OT, J1:OT-A, J5:CBN, J5:CBN-A, J5:DiP, J5:DiP-A, J5:OT, J5:OT-A, S1:DiP, S1:DiP-A, S1:OT, S1:OT-A, S5:DiP, S5:DiP-A, S5:OT, S5:OT-A
448	No	11	11-B	1000	exp	0	unif	singleC	J1:CBN, J1:CBN-A, J1:DiP, J1:DiP-A, J1:OT, J1:OT-A, J5:CBN, J5:CBN-A, J5:DiP, J5:DiP-A, J5:OT, J5:OT-A, S1:DiP, S1:DiP-A, S1:OT, S1:OT-A, S5:DiP, S5:DiP-A, S5:OT, S5:OT-A
449	No	11	11-B	1000	exp	0	unif	wholeT_0.01	J1:DiP, J1:DiP-A, J1:OT, J1:OT-A, J5:CBN, J5:CBN-A, J5:DiP, J5:DiP-A, J5:OT, J5:OT-A, S1:DiP, S1:DiP-A, S1:OT, S1:OT-A, S5:DiP, S5:DiP-A, S5:OT, S5:OT-A
450	No	11	11-B	1000	exp	0	unif	wholeT_0.5	J1:CBN, J1:CBN-A, J1:DiP, J1:DiP-A, J1:OT, J1:OT-A, J5:CBN, J5:CBN-A, J5:DiP, J5:DiP-A, J5:OT, J5:OT-A, S1:DiP, S1:DiP-A, S1:OT, S1:OT-A, S5:DiP, S5:DiP-A, S5:OT, S5:OT-A

Table 8: *(continued)*

	Conjunction	Drivers	Tree	S.Size	Model	sh	S.Time	S.Type	Best method(s)
451	No	11	11-B	1000	exp	Inf	last	singleC	J1:DiP, J1:DiP-A, J1:OT, J1:OT-A, J5:DiP, J5:DiP-A, J5:OT, J5:OT-A, S1:DiP-A, S5:DiP, S5:DiP-A, S5:OT, S5:OT-A
452	No	11	11-B	1000	exp	Inf	last	wholeT_0.01	J5:DiP, J5:DiP-A, J5:OT, J5:OT-A, S5:DiP, S5:DiP-A, S5:OT, S5:OT-A
453	No	11	11-B	1000	exp	Inf	last	wholeT_0.5	J1:DiP, J1:DiP-A, J1:OT, J1:OT-A, J5:DiP, J5:DiP-A, J5:OT, J5:OT-A, S5:DiP, S5:DiP-A, S5:OT, S5:OT-A
454	No	11	11-B	1000	exp	Inf	unif	singleC	J1:DiP, J1:DiP-A, J1:OT, J1:OT-A, J5:CBN, J5:CBN-A, J5:DiP, J5:DiP-A, J5:OT, J5:OT-A, S5:DiP, S5:DiP-A, S5:OT, S5:OT-A
455	No	11	11-B	1000	exp	Inf	unif	wholeT_0.01	J5:DiP, J5:DiP-A, J5:OT, J5:OT-A, S5:DiP, S5:DiP-A, S5:OT, S5:OT-A
456	No	11	11-B	1000	exp	Inf	unif	wholeT_0.5	J1:DiP, J1:DiP-A, J1:OT, J1:OT-A, J5:CBN, J5:CBN-A, J5:DiP, J5:DiP-A, J5:OT, J5:OT-A, S5:DiP, S5:DiP-A, S5:OT, S5:OT-A
457	No	11	11-B	1000	McF_4	0	last	singleC	J1:CBN, J1:DiP, J1:DiP-A, J1:OT, J1:OT-A, J5:CBN, J5:DiP, J5:DiP-A, J5:OT, J5:OT-A, S1:DiP-A, S1:OT, S1:OT-A, S5:DiP, S5:DiP-A, S5:OT, S5:OT-A
458	No	11	11-B	1000	McF_4	0	last	wholeT_0.01	J1:DiP, J1:DiP-A, J1:OT, J1:OT-A, J5:CBN, J5:DiP, J5:DiP-A, J5:OT, J5:OT-A, S1:DiP, S1:DiP-A, S5:DiP, S5:DiP-A, S5:OT, S5:OT-A
459	No	11	11-B	1000	McF_4	0	last	wholeT_0.5	J1:CBN, J1:DiP, J1:DiP-A, J1:OT, J1:OT-A, J5:CBN, J5:DiP, J5:DiP-A, J5:OT, J5:OT-A, S1:DiP-A, S1:OT, S1:OT-A, S5:DiP, S5:DiP-A, S5:OT, S5:OT-A
460	No	11	11-B	1000	McF_4	0	unif	singleC	J1:CBN, J1:CBN-A, J1:DiP, J1:DiP-A, J1:OT, J1:OT-A, J5:DiP, J5:DiP-A, J5:OT, J5:OT-A, S1:DiP, S1:DiP-A, S1:OT, S1:OT-A, S5:CBN-A, S5:DiP, S5:DiP-A, S5:OT, S5:OT-A

Table 8: *(continued)*

	Conjunction	Drivers	Tree	S.Size	Model	sh	S.Time	S.Type	Best method(s)
461	No	11	11-B	1000	McF_4	0	unif	wholeT_0.01	J1:CBN-A, J1:DiP, J1:DiP-A, J1:OT, J1:OT-A, J5:DiP, J5:DiP-A, J5:OT, J5:OT-A, S5:DiP, S5:DiP-A, S5:OT, S5:OT-A
462	No	11	11-B	1000	McF_4	0	unif	wholeT_0.5	J1:CBN-A, J1:DiP, J1:DiP-A, J1:OT, J1:OT-A, J5:DiP, J5:DiP-A, J5:OT, J5:OT-A, S1:DiP, S1:DiP-A, S1:OT, S1:OT-A, S5:CBN, S5:DiP, S5:DiP-A, S5:OT, S5:OT-A
463	No	11	11-B	1000	McF_4	Inf	last	singleC	J1:DiP, J1:DiP-A, J1:OT, J1:OT-A, J5:DiP, J5:DiP-A, J5:OT, J5:OT-A, S1:OT, S1:OT-A, S5:DiP, S5:DiP-A, S5:OT, S5:OT-A
464	No	11	11-B	1000	McF_4	Inf	last	wholeT_0.01	J1:DiP, J1:DiP-A, J1:OT, J1:OT-A, J5:DiP, J5:DiP-A, J5:OT, J5:OT-A, S1:DiP, S1:DiP-A, S5:DiP, S5:DiP-A, S5:OT, S5:OT-A
465	No	11	11-B	1000	McF_4	Inf	last	wholeT_0.5	J1:DiP, J1:DiP-A, J1:OT, J1:OT-A, J5:DiP, J5:DiP-A, J5:OT, J5:OT-A, S1:DiP-A, S1:OT, S1:OT-A, S5:DiP, S5:DiP-A, S5:OT, S5:OT-A
466	No	11	11-B	1000	McF_4	Inf	unif	singleC	J1:CBN-A, J1:DiP, J1:DiP-A, J1:OT, J1:OT-A, J5:DiP, J5:DiP-A, J5:OT, J5:OT-A, S1:DiP, S1:DiP-A, S1:OT, S1:OT-A, S5:DiP, S5:DiP-A, S5:OT, S5:OT-A
467	No	11	11-B	1000	McF_4	Inf	unif	wholeT_0.01	J1:CBN-A, J1:DiP, J1:DiP-A, J1:OT, J1:OT-A, J5:DiP, J5:DiP-A, J5:OT, J5:OT-A, S5:DiP, S5:DiP-A, S5:OT, S5:OT-A
468	No	11	11-B	1000	McF_4	Inf	unif	wholeT_0.5	J1:CBN-A, J1:DiP, J1:DiP-A, J1:OT, J1:OT-A, J5:DiP, J5:DiP-A, J5:OT, J5:OT-A, S1:DiP, S1:DiP-A, S1:OT, S1:OT-A, S5:CBN-A, S5:DiP, S5:DiP-A, S5:OT, S5:OT-A
469	No	11	11-B	1000	McF_6	0	last	singleC	J1:DiP, J1:OT, J5:DiP, J5:OT, S1:DiP, S5:DiP, S5:OT
470	No	11	11-B	1000	McF_6	0	last	wholeT_0.01	J1:DiP, J1:OT, J5:DiP, J5:OT, S1:DiP, S5:DiP, S5:OT

Table 8: *(continued)*

	Conjunction	Drivers	Tree	S.Size	Model	sh	S.Time	S.Type	Best method(s)
471	No	11	11-B	1000	McF_6	0	last	wholeT_0.5	J1:DiP, J1:OT, J5:DiP, J5:OT, S1:DiP, S5:DiP, S5:OT
472	No	11	11-B	1000	McF_6	0	unif	singleC	J1:CBN, J1:CBN-A, J1:DiP, J1:DiP-A, J1:OT, J1:OT-A, J5:CBN, J5:CBN-A, J5:DiP, J5:DiP-A, J5:OT, J5:OT-A, S1:DiP, S1:DiP-A, S1:OT, S1:OT-A, S5:CBN, S5:CBN-A, S5:DiP, S5:DiP-A, S5:OT, S5:OT-A
473	No	11	11-B	1000	McF_6	0	unif	wholeT_0.01	J1:CBN, J1:CBN-A, J1:DiP, J1:DiP-A, J1:OT, J1:OT-A, J5:CBN, J5:CBN-A, J5:DiP, J5:DiP-A, J5:OT, J5:OT-A, S1:DiP, S1:DiP-A, S5:CBN, S5:DiP, S5:DiP-A, S5:OT, S5:OT-A
474	No	11	11-B	1000	McF_6	0	unif	wholeT_0.5	J1:CBN, J1:CBN-A, J1:DiP, J1:DiP-A, J1:OT, J1:OT-A, J5:CBN, J5:CBN-A, J5:DiP, J5:DiP-A, J5:OT, J5:OT-A, S1:DiP, S1:DiP-A, S1:OT, S1:OT-A, S5:CBN, S5:CBN-A, S5:DiP, S5:DiP-A, S5:OT, S5:OT-A
475	No	11	11-B	1000	McF_6	Inf	last	singleC	J1:DiP, J1:OT, J5:DiP, J5:OT, S1:DiP, S5:DiP, S5:OT
476	No	11	11-B	1000	McF_6	Inf	last	wholeT_0.01	J1:DiP, J1:OT, J5:DiP, J5:OT, S1:DiP, S5:DiP, S5:OT
477	No	11	11-B	1000	McF_6	Inf	last	wholeT_0.5	J1:DiP, J1:OT, J5:DiP, J5:OT, S1:DiP, S1:OT, S5:DiP, S5:OT
478	No	11	11-B	1000	McF_6	Inf	unif	singleC	J1:CBN-A, J1:DiP, J1:DiP-A, J1:OT, J1:OT-A, J5:CBN-A, J5:DiP, J5:DiP-A, J5:OT, J5:OT-A, S1:DiP, S1:DiP-A, S5:CBN, S5:CBN-A, S5:DiP, S5:DiP-A, S5:OT, S5:OT-A
479	No	11	11-B	1000	McF_6	Inf	unif	wholeT_0.01	J1:CBN-A, J1:DiP, J1:DiP-A, J1:OT, J1:OT-A, J5:CBN-A, J5:DiP, J5:DiP-A, J5:OT, J5:OT-A, S1:DiP, S1:DiP-A, S5:CBN, S5:DiP, S5:DiP-A, S5:OT, S5:OT-A
480	No	11	11-B	1000	McF_6	Inf	unif	wholeT_0.5	J1:CBN, J1:CBN-A, J1:DiP, J1:DiP-A, J1:OT, J1:OT-A, J5:CBN-A, J5:DiP, J5:DiP-A, J5:OT, J5:OT-A, S1:DiP, S1:DiP-A, S5:CBN, S5:CBN-A, S5:DiP, S5:DiP-A, S5:OT, S5:OT-A

Table 8: *(continued)*

	Conjunction	Drivers	Tree	S.Size	Model	sh	S.Time	S.Type	Best method(s)
481	No	11	11-B	200	Bozic	0	last	singleC	J1:DiP, J1:DiP-A, J1:OT, J1:OT-A, J5:DiP, J5:DiP-A, J5:OT, J5:OT-A, S1:DiP, S1:DiP-A, S5:DiP, S5:DiP-A, S5:OT, S5:OT-A
482	No	11	11-B	200	Bozic	0	last	wholeT_0.01	J1:DiP, J5:DiP, J5:OT, J5:OT-A
483	No	11	11-B	200	Bozic	0	last	wholeT_0.5	J1:DiP, J1:DiP-A, J1:OT, J1:OT-A, J5:DiP, J5:DiP-A, J5:OT, J5:OT-A, S1:DiP, S1:DiP-A, S5:DiP, S5:DiP-A, S5:OT, S5:OT-A
484	No	11	11-B	200	Bozic	0	unif	singleC	J1:DiP, J1:DiP-A, J1:OT, J1:OT-A, J5:CBN, J5:CBN-A, J5:DiP, J5:DiP-A, J5:OT, J5:OT-A, S1:DiP, S1:DiP-A, S1:OT, S1:OT-A, S5:DiP, S5:DiP-A, S5:OT, S5:OT-A
485	No	11	11-B	200	Bozic	0	unif	wholeT_0.01	J5:DiP, J5:DiP-A, J5:OT, J5:OT-A, S5:DiP, S5:DiP-A
486	No	11	11-B	200	Bozic	0	unif	wholeT_0.5	J1:CBN, J1:CBN-A, J1:DiP, J1:DiP-A, J1:OT, J1:OT-A, J5:CBN, J5:CBN-A, J5:DiP, J5:DiP-A, J5:OT, J5:OT-A, S1:DiP, S1:DiP-A, S1:OT, S1:OT-A, S5:DiP, S5:DiP-A, S5:OT, S5:OT-A
487	No	11	11-B	200	Bozic	Inf	last	singleC	J1:DiP, J1:DiP-A, J5:DiP, J5:DiP-A, J5:OT, J5:OT-A, S5:DiP, S5:DiP-A, S5:OT, S5:OT-A
488	No	11	11-B	200	Bozic	Inf	last	wholeT_0.01	J5:DiP, J5:DiP-A, J5:OT, J5:OT-A
489	No	11	11-B	200	Bozic	Inf	last	wholeT_0.5	J1:DiP, J1:DiP-A, J5:DiP, J5:DiP-A, J5:OT, J5:OT-A, S5:DiP, S5:DiP-A, S5:OT, S5:OT-A
490	No	11	11-B	200	Bozic	Inf	unif	singleC	J1:CBN, J1:CBN-A, J1:DiP, J1:DiP-A, J1:OT, J1:OT-A, J5:DiP, J5:DiP-A, J5:OT, J5:OT-A, S5:DiP, S5:DiP-A, S5:OT, S5:OT-A
491	No	11	11-B	200	Bozic	Inf	unif	wholeT_0.01	J5:CBN-A, J5:DiP, J5:DiP-A, J5:OT, J5:OT-A
492	No	11	11-B	200	Bozic	Inf	unif	wholeT_0.5	J1:DiP, J1:DiP-A, J1:OT, J1:OT-A, J5:DiP, J5:DiP-A, J5:OT, J5:OT-A, S5:DiP, S5:DiP-A, S5:OT, S5:OT-A

Table 8: *(continued)*

	Conjunction	Drivers	Tree	S.Size	Model	sh	S.Time	S.Type	Best method(s)
493	No	11	11-B	200	exp	0	last	singleC	J1:DiP, J1:DiP-A, J1:OT, J1:OT-A, J5:CBN, J5:CBN-A, J5:DiP, J5:DiP-A, J5:OT, J5:OT-A, S1:DiP, S1:DiP-A, S1:OT, S1:OT-A, S5:DiP, S5:DiP-A, S5:OT, S5:OT-A
494	No	11	11-B	200	exp	0	last	wholeT_0.01	J1:DiP, J1:DiP-A, J1:OT, J1:OT-A, J5:DiP, J5:DiP-A, J5:OT, J5:OT-A, S1:OT, S1:OT-A, S5:DiP, S5:DiP-A, S5:OT, S5:OT-A
495	No	11	11-B	200	exp	0	last	wholeT_0.5	J1:DiP, J1:DiP-A, J1:OT, J1:OT-A, J5:CBN, J5:CBN-A, J5:DiP, J5:DiP-A, J5:OT, J5:OT-A, S1:DiP, S1:DiP-A, S1:OT, S1:OT-A, S5:DiP, S5:DiP-A, S5:OT, S5:OT-A
496	No	11	11-B	200	exp	0	unif	singleC	J1:CBN, J1:CBN-A, J1:DiP, J1:DiP-A, J1:OT, J1:OT-A, J5:CBN, J5:CBN-A, J5:DiP, J5:DiP-A, J5:OT, J5:OT-A, S1:DiP, S1:DiP-A, S1:OT, S1:OT-A, S5:DiP, S5:DiP-A, S5:OT, S5:OT-A
497	No	11	11-B	200	exp	0	unif	wholeT_0.01	J1:DiP, J1:DiP-A, J1:OT, J1:OT-A, J5:CBN, J5:CBN-A, J5:DiP, J5:DiP-A, J5:OT, J5:OT-A, S1:DiP, S1:DiP-A, S1:OT, S1:OT-A, S5:DiP, S5:DiP-A, S5:OT, S5:OT-A
498	No	11	11-B	200	exp	0	unif	wholeT_0.5	J1:CBN, J1:CBN-A, J1:DiP, J1:DiP-A, J1:OT, J1:OT-A, J5:CBN, J5:CBN-A, J5:DiP, J5:DiP-A, J5:OT, J5:OT-A, S1:DiP, S1:DiP-A, S1:OT, S1:OT-A, S5:DiP, S5:DiP-A, S5:OT, S5:OT-A
499	No	11	11-B	200	exp	Inf	last	singleC	J1:DiP, J1:DiP-A, J1:OT, J1:OT-A, J5:DiP, J5:DiP-A, J5:OT, J5:OT-A, S1:DiP, S1:DiP-A, S5:DiP, S5:DiP-A, S5:OT, S5:OT-A
500	No	11	11-B	200	exp	Inf	last	wholeT_0.01	J5:DiP, J5:DiP-A, J5:OT, J5:OT-A
501	No	11	11-B	200	exp	Inf	last	wholeT_0.5	J1:DiP, J1:DiP-A, J1:OT, J1:OT-A, J5:DiP, J5:DiP-A, J5:OT, J5:OT-A, S1:DiP, S1:DiP-A, S5:DiP, S5:DiP-A, S5:OT, S5:OT-A

Table 8: *(continued)*

	Conjunction	Drivers	Tree	S.Size	Model	sh	S.Time	S.Type	Best method(s)
502	No	11	11-B	200	exp	Inf	unif	singleC	J1:DiP, J1:DiP-A, J1:OT, J1:OT-A, J5:CBN, J5:CBN-A, J5:DiP, J5:DiP-A, J5:OT, J5:OT-A, S5:DiP, S5:DiP-A, S5:OT, S5:OT-A
503	No	11	11-B	200	exp	Inf	unif	wholeT_0.01	J5:DiP, J5:DiP-A, J5:OT, J5:OT-A, S5:OT, S5:OT-A
504	No	11	11-B	200	exp	Inf	unif	wholeT_0.5	J1:DiP, J1:DiP-A, J1:OT, J1:OT-A, J5:CBN, J5:CBN-A, J5:DiP, J5:DiP-A, J5:OT, J5:OT-A, S1:DiP, S1:DiP-A, S5:DiP, S5:DiP-A, S5:OT, S5:OT-A
505	No	11	11-B	200	McF_4	0	last	singleC	J1:DiP, J1:DiP-A, J1:OT, J1:OT-A, J5:DiP, J5:DiP-A, J5:OT, J5:OT-A, S1:DiP-A, S5:DiP, S5:DiP-A, S5:OT, S5:OT-A
506	No	11	11-B	200	McF_4	0	last	wholeT_0.01	J1:DiP, J1:DiP-A, J1:OT, J1:OT-A, J5:CBN, J5:DiP, J5:DiP-A, J5:OT, J5:OT-A, S1:DiP, S1:DiP-A, S5:DiP, S5:DiP-A, S5:OT, S5:OT-A
507	No	11	11-B	200	McF_4	0	last	wholeT_0.5	J1:DiP, J1:DiP-A, J1:OT, J1:OT-A, J5:DiP, J5:DiP-A, J5:OT, J5:OT-A, S5:DiP, S5:DiP-A, S5:OT, S5:OT-A
508	No	11	11-B	200	McF_4	0	unif	singleC	J1:CBN, J1:CBN-A, J1:DiP, J1:DiP-A, J1:OT, J1:OT-A, J5:DiP, J5:DiP-A, J5:OT, J5:OT-A, S5:DiP, S5:DiP-A, S5:OT, S5:OT-A
509	No	11	11-B	200	McF_4	0	unif	wholeT_0.01	J1:CBN-A, J1:DiP, J1:DiP-A, J1:OT, J1:OT-A, J5:DiP, J5:DiP-A, J5:OT, J5:OT-A, S1:DiP, S1:DiP-A, S5:DiP, S5:DiP-A, S5:OT, S5:OT-A
510	No	11	11-B	200	McF_4	0	unif	wholeT_0.5	J1:CBN-A, J1:DiP, J1:DiP-A, J1:OT, J1:OT-A, J5:DiP, J5:DiP-A, J5:OT, J5:OT-A, S1:DiP, S1:DiP-A, S5:CBN-A, S5:DiP, S5:DiP-A, S5:OT, S5:OT-A
511	No	11	11-B	200	McF_4	Inf	last	singleC	J1:DiP, J1:DiP-A, J1:OT, J1:OT-A, J5:DiP, J5:DiP-A, J5:OT, J5:OT-A, S5:OT, S5:OT-A
512	No	11	11-B	200	McF_4	Inf	last	wholeT_0.01	J1:DiP, J1:DiP-A, J1:OT, J1:OT-A, J5:DiP, J5:DiP-A, J5:OT, J5:OT-A, S1:DiP, S1:DiP-A, S5:DiP, S5:DiP-A, S5:OT, S5:OT-A

Table 8: *(continued)*

	Conjunction	Drivers	Tree	S.Size	Model	sh	S.Time	S.Type	Best method(s)
513	No	11	11-B	200	McF_4	Inf	last	wholeT_0.5	J1:DiP, J1:DiP-A, J1:OT, J1:OT-A, J5:DiP, J5:DiP-A, J5:OT, J5:OT-A, S1:DiP, S1:DiP-A, S5:DiP, S5:DiP-A, S5:OT, S5:OT-A
514	No	11	11-B	200	McF_4	Inf	unif	singleC	J1:CBN-A, J1:DiP, J1:DiP-A, J1:OT, J1:OT-A, J5:DiP, J5:DiP-A, J5:OT, J5:OT-A, S1:DiP, S5:CBN-A, S5:DiP, S5:DiP-A, S5:OT, S5:OT-A
515	No	11	11-B	200	McF_4	Inf	unif	wholeT_0.01	J1:DiP, J1:DiP-A, J1:OT, J1:OT-A, J5:DiP, J5:DiP-A, J5:OT, J5:OT-A, S1:DiP, S1:DiP-A, S5:DiP, S5:DiP-A, S5:OT, S5:OT-A
516	No	11	11-B	200	McF_4	Inf	unif	wholeT_0.5	J1:CBN-A, J1:DiP, J1:DiP-A, J1:OT, J1:OT-A, J5:DiP, J5:DiP-A, J5:OT, J5:OT-A, S1:DiP, S1:DiP-A, S5:DiP, S5:DiP-A, S5:OT, S5:OT-A
517	No	11	11-B	200	McF_6	0	last	singleC	J1:DiP, J1:OT, J5:DiP, J5:OT, S1:DiP, S5:DiP, S5:OT
518	No	11	11-B	200	McF_6	0	last	wholeT_0.01	J1:DiP, J1:OT, J5:DiP, J5:OT, S1:DiP, S5:DiP, S5:OT
519	No	11	11-B	200	McF_6	0	last	wholeT_0.5	J1:DiP, J1:OT, J5:DiP, J5:OT, S1:DiP, S5:DiP, S5:OT
520	No	11	11-B	200	McF_6	0	unif	singleC	J1:CBN, J1:CBN-A, J1:DiP, J1:OT, J1:OT-A, J5:CBN, J5:CBN-A, J5:DiP, J5:DiP-A, J5:OT, J5:OT-A, S5:CBN, S5:CBN-A, S5:OT, S5:OT-A
521	No	11	11-B	200	McF_6	0	unif	wholeT_0.01	J1:CBN, J1:CBN-A, J1:DiP, J1:DiP-A, J1:OT, J1:OT-A, J5:CBN, J5:CBN-A, J5:DiP, J5:DiP-A, J5:OT, J5:OT-A, S5:CBN-A, S5:DiP, S5:DiP-A, S5:OT, S5:OT-A
522	No	11	11-B	200	McF_6	0	unif	wholeT_0.5	J1:CBN, J1:CBN-A, J1:DiP, J1:OT, J1:OT-A, J5:CBN, J5:CBN-A, J5:DiP, J5:DiP-A, J5:OT, J5:OT-A, S5:OT, S5:OT-A
523	No	11	11-B	200	McF_6	Inf	last	singleC	J1:DiP, J1:OT, J5:DiP, J5:OT, S1:DiP, S5:DiP, S5:OT
524	No	11	11-B	200	McF_6	Inf	last	wholeT_0.01	J1:DiP, J1:OT, J5:DiP, J5:OT, S1:DiP, S5:DiP, S5:OT

Table 8: *(continued)*

	Conjunction	Drivers	Tree	S.Size	Model	sh	S.Time	S.Type	Best method(s)
525	No	11	11-B	200	McF_6	Inf	last	wholeT_0.5	J1:DiP, J1:OT, J5:DiP, J5:OT, S1:DiP, S5:DiP, S5:OT
526	No	11	11-B	200	McF_6	Inf	unif	singleC	J1:CBN, J1:CBN-A, J1:OT, J1:OT-A, J5:CBN-A, J5:DiP, J5:DiP-A, J5:OT, J5:OT-A, S5:CBN-A
527	No	11	11-B	200	McF_6	Inf	unif	wholeT_0.01	J1:CBN-A, J1:DiP, J1:OT, J1:OT-A, J5:CBN-A, J5:DiP, J5:DiP-A, J5:OT, J5:OT-A, S5:CBN, S5:OT, S5:OT-A
528	No	11	11-B	200	McF_6	Inf	unif	wholeT_0.5	J1:CBN-A, J5:CBN-A, J5:DiP, J5:DiP-A, J5:OT, J5:OT-A, S5:CBN-A
529	No	11	11-B	100	Bozic	0	last	singleC	J1:DiP, J1:DiP-A, J5:DiP, J5:DiP-A, J5:OT, J5:OT-A, S1:DiP, S1:DiP-A, S5:DiP, S5:DiP-A, S5:OT, S5:OT-A
530	No	11	11-B	100	Bozic	0	last	wholeT_0.01	J1:DiP, J1:DiP-A, J5:DiP, J5:DiP-A, J5:OT, J5:OT-A
531	No	11	11-B	100	Bozic	0	last	wholeT_0.5	J1:DiP, J1:DiP-A, J5:DiP, J5:DiP-A, J5:OT, J5:OT-A, S1:DiP, S1:DiP-A, S5:DiP, S5:DiP-A, S5:OT, S5:OT-A
532	No	11	11-B	100	Bozic	0	unif	singleC	J1:DiP, J1:DiP-A, J1:OT, J1:OT-A, J5:CBN, J5:CBN-A, J5:DiP, J5:DiP-A, J5:OT, J5:OT-A, S1:DiP, S1:DiP-A, S5:DiP, S5:DiP-A, S5:OT, S5:OT-A
533	No	11	11-B	100	Bozic	0	unif	wholeT_0.01	J1:DiP, J1:DiP-A, J5:DiP, J5:DiP-A, J5:OT, J5:OT-A, S5:DiP, S5:DiP-A, S5:OT, S5:OT-A
534	No	11	11-B	100	Bozic	0	unif	wholeT_0.5	J1:DiP, J1:DiP-A, J1:OT, J1:OT-A, J5:CBN, J5:CBN-A, J5:DiP, J5:DiP-A, J5:OT, J5:OT-A, S1:DiP, S1:DiP-A, S5:DiP, S5:DiP-A, S5:OT, S5:OT-A
535	No	11	11-B	100	Bozic	Inf	last	singleC	J1:DiP, J1:DiP-A, J5:DiP, J5:DiP-A, J5:OT, J5:OT-A, S5:DiP, S5:DiP-A
536	No	11	11-B	100	Bozic	Inf	last	wholeT_0.01	J5:DiP, J5:DiP-A, J5:OT, J5:OT-A
537	No	11	11-B	100	Bozic	Inf	last	wholeT_0.5	J1:DiP, J1:DiP-A, J5:DiP, J5:DiP-A, J5:OT, J5:OT-A, S1:DiP, S1:DiP-A, S5:DiP, S5:DiP-A
538	No	11	11-B	100	Bozic	Inf	unif	singleC	J1:DiP, J1:DiP-A, J5:DiP, J5:DiP-A, J5:OT, J5:OT-A, S5:DiP, S5:DiP-A, S5:OT, S5:OT-A
539	No	11	11-B	100	Bozic	Inf	unif	wholeT_0.01	J5:DiP, J5:DiP-A, J5:OT, J5:OT-A

Table 8: *(continued)*

	Conjunction	Drivers	Tree	S.Size	Model	sh	S.Time	S.Type	Best method(s)
540	No	11	11-B	100	Bozic	Inf	unif	wholeT_0.5	J1:DiP, J1:DiP-A, J5:DiP, J5:DiP-A, J5:OT, J5:OT-A, S5:DiP, S5:DiP-A, S5:OT, S5:OT-A
541	No	11	11-B	100	exp	0	last	singleC	J1:DiP, J1:DiP-A, J1:OT, J1:OT-A, J5:CBN, J5:CBN-A, J5:DiP, J5:DiP-A, J5:OT, J5:OT-A, S1:DiP, S1:DiP-A, S1:OT, S1:OT-A, S5:DiP, S5:DiP-A, S5:OT, S5:OT-A
542	No	11	11-B	100	exp	0	last	wholeT_0.01	J1:DiP, J1:DiP-A, J1:OT, J1:OT-A, J5:DiP, J5:DiP-A, J5:OT, J5:OT-A, S1:DiP, S1:DiP-A, S5:DiP, S5:DiP-A, S5:OT, S5:OT-A
543	No	11	11-B	100	exp	0	last	wholeT_0.5	J1:DiP, J1:DiP-A, J1:OT, J1:OT-A, J5:CBN, J5:CBN-A, J5:DiP, J5:DiP-A, J5:OT, J5:OT-A, S1:DiP, S1:DiP-A, S1:OT, S1:OT-A, S5:DiP, S5:DiP-A, S5:OT, S5:OT-A
544	No	11	11-B	100	exp	0	unif	singleC	J1:DiP, J1:DiP-A, J1:OT, J1:OT-A, J5:CBN, J5:CBN-A, J5:DiP, J5:DiP-A, J5:OT, J5:OT-A, S1:DiP, S1:DiP-A, S1:OT, S1:OT-A, S5:DiP, S5:DiP-A, S5:OT, S5:OT-A
545	No	11	11-B	100	exp	0	unif	wholeT_0.01	J1:DiP, J1:DiP-A, J1:OT, J1:OT-A, J5:CBN, J5:CBN-A, J5:DiP, J5:DiP-A, J5:OT, J5:OT-A, S1:DiP, S1:DiP-A, S5:DiP, S5:DiP-A, S5:OT, S5:OT-A
546	No	11	11-B	100	exp	0	unif	wholeT_0.5	J1:CBN, J1:CBN-A, J1:DiP, J1:DiP-A, J1:OT, J1:OT-A, J5:CBN, J5:CBN-A, J5:DiP, J5:DiP-A, J5:OT, J5:OT-A, S1:DiP, S1:DiP-A, S1:OT, S1:OT-A, S5:DiP, S5:DiP-A, S5:OT, S5:OT-A
547	No	11	11-B	100	exp	Inf	last	singleC	J1:DiP, J1:DiP-A, J5:DiP, J5:DiP-A, J5:OT, J5:OT-A, S1:DiP, S1:DiP-A, S5:DiP, S5:DiP-A, S5:OT, S5:OT-A
548	No	11	11-B	100	exp	Inf	last	wholeT_0.01	J5:CBN-A, J5:DiP, J5:DiP-A, J5:OT, J5:OT-A
549	No	11	11-B	100	exp	Inf	last	wholeT_0.5	J1:DiP, J1:DiP-A, J5:DiP, J5:DiP-A, J5:OT, J5:OT-A, S1:DiP, S1:DiP-A, S5:DiP, S5:DiP-A, S5:OT, S5:OT-A

Table 8: *(continued)*

	Conjunction	Drivers	Tree	S.Size	Model	sh	S.Time	S.Type	Best method(s)
550	No	11	11-B	100	exp	Inf	unif	singleC	J1:DiP, J1:DiP-A, J5:DiP, J5:DiP-A, J5:OT, J5:OT-A, S1:DiP, S1:DiP-A, S5:DiP, S5:DiP-A, S5:OT, S5:OT-A
551	No	11	11-B	100	exp	Inf	unif	wholeT_0.01	J5:DiP, J5:DiP-A, J5:OT, J5:OT-A, S5:DiP, S5:DiP-A, S5:OT, S5:OT-A
552	No	11	11-B	100	exp	Inf	unif	wholeT_0.5	J1:DiP, J1:DiP-A, J5:CBN, J5:CBN-A, J5:DiP, J5:DiP-A, J5:OT, J5:OT-A, S1:DiP, S1:DiP-A, S5:DiP, S5:DiP-A, S5:OT, S5:OT-A
553	No	11	11-B	100	McF_4	0	last	singleC	J1:DiP, J1:DiP-A, J5:DiP, J5:DiP-A, J5:OT, J5:OT-A, S1:DiP, S1:DiP-A, S5:DiP, S5:DiP-A, S5:OT, S5:OT-A
554	No	11	11-B	100	McF_4	0	last	wholeT_0.01	J1:DiP, J1:DiP-A, J5:DiP, J5:DiP-A, J5:OT, J5:OT-A, S1:DiP, S1:DiP-A, S5:DiP, S5:DiP-A, S5:OT, S5:OT-A
555	No	11	11-B	100	McF_4	0	last	wholeT_0.5	J1:DiP, J1:DiP-A, J5:DiP, J5:DiP-A, J5:OT, J5:OT-A, S1:DiP, S1:DiP-A, S5:DiP, S5:DiP-A, S5:OT, S5:OT-A
556	No	11	11-B	100	McF_4	0	unif	singleC	J1:CBN-A, J1:DiP, J1:DiP-A, J1:OT, J1:OT-A, J5:DiP, J5:DiP-A, J5:OT, J5:OT-A, S1:DiP, S1:DiP-A, S5:DiP, S5:DiP-A, S5:OT, S5:OT-A
557	No	11	11-B	100	McF_4	0	unif	wholeT_0.01	J1:DiP, J1:DiP-A, J5:DiP, J5:DiP-A, J5:OT, J5:OT-A, S5:DiP, S5:DiP-A, S5:OT, S5:OT-A
558	No	11	11-B	100	McF_4	0	unif	wholeT_0.5	J1:CBN-A, J1:DiP, J1:DiP-A, J1:OT, J1:OT-A, J5:DiP, J5:DiP-A, J5:OT, J5:OT-A, S1:DiP, S1:DiP-A, S5:DiP, S5:DiP-A, S5:OT, S5:OT-A
559	No	11	11-B	100	McF_4	Inf	last	singleC	J1:DiP, J1:DiP-A, J5:DiP, J5:DiP-A, J5:OT, J5:OT-A, S1:DiP, S1:DiP-A, S5:DiP, S5:DiP-A, S5:OT, S5:OT-A
560	No	11	11-B	100	McF_4	Inf	last	wholeT_0.01	J1:DiP, J1:DiP-A, J5:DiP, J5:DiP-A, J5:OT, J5:OT-A, S1:DiP, S5:DiP, S5:DiP-A, S5:OT, S5:OT-A
561	No	11	11-B	100	McF_4	Inf	last	wholeT_0.5	J1:DiP, J1:DiP-A, J5:DiP, J5:DiP-A, J5:OT, J5:OT-A, S1:DiP, S1:DiP-A, S5:DiP, S5:DiP-A, S5:OT, S5:OT-A

Table 8: *(continued)*

	Conjunction	Drivers	Tree	S.Size	Model	sh	S.Time	S.Type	Best method(s)
562	No	11	11-B	100	McF_4	Inf	unif	singleC	J1:DiP, J1:DiP-A, J1:OT, J1:OT-A, J5:DiP, J5:DiP-A, J5:OT, J5:OT-A, S1:DiP, S1:DiP-A, S5:DiP, S5:DiP-A, S5:OT, S5:OT-A
563	No	11	11-B	100	McF_4	Inf	unif	wholeT_0.01	J1:DiP, J1:DiP-A, J5:DiP, J5:DiP-A, J5:OT, J5:OT-A, S5:DiP, S5:DiP-A, S5:OT, S5:OT-A
564	No	11	11-B	100	McF_4	Inf	unif	wholeT_0.5	J1:DiP, J1:DiP-A, J5:DiP, J5:DiP-A, J5:OT, J5:OT-A, S1:DiP, S1:DiP-A, S5:DiP, S5:DiP-A, S5:OT, S5:OT-A
565	No	11	11-B	100	McF_6	0	last	singleC	J1:DiP, J1:OT, J5:DiP, J5:OT, S1:DiP, S5:DiP, S5:OT
566	No	11	11-B	100	McF_6	0	last	wholeT_0.01	J1:DiP, J5:DiP, J5:OT, S1:DiP, S5:DiP, S5:OT
567	No	11	11-B	100	McF_6	0	last	wholeT_0.5	J1:DiP, J1:OT, J5:DiP, J5:OT, S1:DiP, S5:DiP, S5:OT
568	No	11	11-B	100	McF_6	0	unif	singleC	J1:CBN, J1:CBN-A, J5:CBN, J5:CBN-A, J5:DiP, J5:OT, J5:OT-A
569	No	11	11-B	100	McF_6	0	unif	wholeT_0.01	J1:DiP, J5:CBN-A, J5:DiP, J5:DiP-A, J5:OT, J5:OT-A
570	No	11	11-B	100	McF_6	0	unif	wholeT_0.5	J1:CBN, J1:CBN-A, J1:DiP, J5:CBN, J5:CBN-A, J5:DiP, J5:DiP-A, J5:OT, J5:OT-A, S5:CBN-A
571	No	11	11-B	100	McF_6	Inf	last	singleC	J1:DiP, J5:DiP, J5:OT, S1:DiP, S5:DiP, S5:OT
572	No	11	11-B	100	McF_6	Inf	last	wholeT_0.01	J1:DiP, J5:DiP, J5:OT, S1:DiP, S5:DiP
573	No	11	11-B	100	McF_6	Inf	last	wholeT_0.5	J1:DiP, J5:DiP, J5:OT
574	No	11	11-B	100	McF_6	Inf	unif	singleC	J5:CBN-A, J5:DiP, J5:OT, J5:OT-A, S5:CBN
575	No	11	11-B	100	McF_6	Inf	unif	wholeT_0.01	J5:CBN-A, J5:DiP, J5:DiP-A, J5:OT, J5:OT-A
576	No	11	11-B	100	McF_6	Inf	unif	wholeT_0.5	J1:CBN-A, J1:DiP, J5:CBN-A, J5:DiP, J5:DiP-A, J5:OT, J5:OT-A
577	No	9	9-B	1000	Bozic	0	last	singleC	J1:DiP, J1:DiP-A, J1:OT, J1:OT-A, J5:DiP, J5:DiP-A, J5:OT, J5:OT-A, S1:OT, S1:OT-A, S5:DiP, S5:DiP-A, S5:OT, S5:OT-A

Table 8: *(continued)*

	Conjunction	Drivers	Tree	S.Size	Model	sh	S.Time	S.Type	Best method(s)
578	No	9	9-B	1000	Bozic	0	last	wholeT_0.01	J1:CBN-A, J1:DiP, J1:DiP-A, J1:OT, J1:OT-A, J5:CBN-A, J5:DiP, J5:DiP-A, J5:OT, J5:OT-A, S1:CBN-A, S1:DiP, S1:DiP-A, S1:OT, S1:OT-A, S5:CBN-A, S5:DiP, S5:DiP-A, S5:OT, S5:OT-A
579	No	9	9-B	1000	Bozic	0	last	wholeT_0.5	J1:DiP, J1:DiP-A, J1:OT, J1:OT-A, J5:CBN, J5:CBN-A, J5:DiP, J5:DiP-A, J5:OT, J5:OT-A, S1:OT, S1:OT-A, S5:DiP, S5:DiP-A, S5:OT, S5:OT-A
580	No	9	9-B	1000	Bozic	0	unif	singleC	J1:DiP, J1:DiP-A, J1:OT, J1:OT-A, J5:CBN, J5:CBN-A, J5:DiP, J5:DiP-A, J5:OT, J5:OT-A, S1:DiP, S1:DiP-A, S1:OT, S1:OT-A, S5:DiP, S5:DiP-A, S5:OT, S5:OT-A
581	No	9	9-B	1000	Bozic	0	unif	wholeT_0.01	J1:DiP, J1:DiP-A, J1:OT, J1:OT-A, J5:DiP, J5:DiP-A, J5:OT, J5:OT-A, S5:DiP, S5:DiP-A, S5:OT, S5:OT-A
582	No	9	9-B	1000	Bozic	0	unif	wholeT_0.5	J1:CBN, J1:CBN-A, J1:DiP, J1:DiP-A, J1:OT, J1:OT-A, J5:CBN, J5:CBN-A, J5:DiP, J5:DiP-A, J5:OT, J5:OT-A, S1:DiP, S1:DiP-A, S1:OT, S1:OT-A, S5:DiP, S5:DiP-A, S5:OT, S5:OT-A
583	No	9	9-B	1000	Bozic	Inf	last	singleC	J1:DiP, J1:DiP-A, J1:OT, J1:OT-A, J5:DiP, J5:DiP-A, J5:OT, J5:OT-A, S1:DiP, S1:DiP-A, S5:DiP, S5:DiP-A, S5:OT, S5:OT-A
584	No	9	9-B	1000	Bozic	Inf	last	wholeT_0.01	J5:DiP, J5:DiP-A
585	No	9	9-B	1000	Bozic	Inf	last	wholeT_0.5	J1:DiP, J1:DiP-A, J1:OT, J1:OT-A, J5:DiP, J5:DiP-A, J5:OT, J5:OT-A, S1:DiP, S1:DiP-A, S5:DiP, S5:DiP-A, S5:OT, S5:OT-A
586	No	9	9-B	1000	Bozic	Inf	unif	singleC	J1:DiP, J1:DiP-A, J1:OT, J1:OT-A, J5:CBN, J5:CBN-A, J5:DiP, J5:DiP-A, J5:OT, J5:OT-A, S5:DiP, S5:DiP-A, S5:OT, S5:OT-A
587	No	9	9-B	1000	Bozic	Inf	unif	wholeT_0.01	J1:CBN-A, J5:DiP, J5:DiP-A, J5:OT, J5:OT-A

Table 8: *(continued)*

	Conjunction	Drivers	Tree	S.Size	Model	sh	S.Time	S.Type	Best method(s)
588	No	9	9-B	1000	Bozic	Inf	unif	wholeT_0.5	J1:DiP, J1:DiP-A, J1:OT, J1:OT-A, J5:CBN, J5:CBN-A, J5:DiP, J5:DiP-A, J5:OT, J5:OT-A, S5:DiP, S5:DiP-A, S5:OT, S5:OT-A
589	No	9	9-B	1000	exp	0	last	singleC	J1:DiP, J1:DiP-A, J1:OT, J1:OT-A, J5:CBN, J5:CBN-A, J5:DiP, J5:DiP-A, J5:OT, J5:OT-A, S1:DiP, S1:DiP-A, S1:OT, S1:OT-A, S5:DiP, S5:DiP-A, S5:OT, S5:OT-A
590	No	9	9-B	1000	exp	0	last	wholeT_0.01	J1:DiP, J1:DiP-A, J1:OT, J1:OT-A, J5:DiP, J5:DiP-A, J5:OT, J5:OT-A, S1:DiP-A, S1:OT, S1:OT-A, S5:DiP, S5:DiP-A, S5:OT, S5:OT-A
591	No	9	9-B	1000	exp	0	last	wholeT_0.5	J1:DiP, J1:DiP-A, J1:OT, J1:OT-A, J5:CBN, J5:CBN-A, J5:DiP, J5:DiP-A, J5:OT, J5:OT-A, S1:DiP, S1:DiP-A, S1:OT, S1:OT-A, S5:DiP, S5:DiP-A, S5:OT, S5:OT-A
592	No	9	9-B	1000	exp	0	unif	singleC	J1:CBN, J1:CBN-A, J1:DiP, J1:DiP-A, J1:OT, J1:OT-A, J5:CBN, J5:CBN-A, J5:DiP, J5:DiP-A, J5:OT, J5:OT-A, S1:DiP, S1:DiP-A, S1:OT, S1:OT-A, S5:DiP, S5:DiP-A, S5:OT, S5:OT-A
593	No	9	9-B	1000	exp	0	unif	wholeT_0.01	J1:DiP, J1:DiP-A, J1:OT, J1:OT-A, J5:CBN, J5:CBN-A, J5:DiP, J5:DiP-A, J5:OT, J5:OT-A, S1:DiP, S1:DiP-A, S1:OT, S1:OT-A, S5:DiP, S5:DiP-A, S5:OT, S5:OT-A
594	No	9	9-B	1000	exp	0	unif	wholeT_0.5	J1:CBN, J1:CBN-A, J1:DiP, J1:DiP-A, J1:OT, J1:OT-A, J5:CBN, J5:CBN-A, J5:DiP, J5:DiP-A, J5:OT, J5:OT-A, S1:DiP, S1:DiP-A, S1:OT, S1:OT-A, S5:DiP, S5:DiP-A, S5:OT, S5:OT-A
595	No	9	9-B	1000	exp	Inf	last	singleC	J1:DiP, J1:DiP-A, J1:OT, J1:OT-A, J5:DiP, J5:DiP-A, J5:OT, J5:OT-A, S1:DiP-A, S5:DiP, S5:DiP-A, S5:OT, S5:OT-A
596	No	9	9-B	1000	exp	Inf	last	wholeT_0.01	J5:DiP, J5:DiP-A

Table 8: *(continued)*

	Conjunction	Drivers	Tree	S.Size	Model	sh	S.Time	S.Type	Best method(s)
597	No	9	9-B	1000	exp	Inf	last	wholeT_0.5	J1:DiP, J1:DiP-A, J1:OT, J1:OT-A, J5:DiP, J5:DiP-A, J5:OT, J5:OT-A, S1:DiP, S1:DiP-A, S5:DiP, S5:DiP-A, S5:OT, S5:OT-A
598	No	9	9-B	1000	exp	Inf	unif	singleC	J1:DiP, J1:DiP-A, J1:OT, J1:OT-A, J5:CBN, J5:CBN-A, J5:DiP, J5:DiP-A, J5:OT, J5:OT-A, S1:DiP, S1:DiP-A, S1:OT, S1:OT-A, S5:DiP, S5:DiP-A, S5:OT, S5:OT-A
599	No	9	9-B	1000	exp	Inf	unif	wholeT_0.01	J5:DiP, J5:DiP-A, J5:OT, J5:OT-A, S5:DiP, S5:DiP-A, S5:OT, S5:OT-A
600	No	9	9-B	1000	exp	Inf	unif	wholeT_0.5	J1:DiP, J1:DiP-A, J1:OT, J1:OT-A, J5:CBN, J5:CBN-A, J5:DiP, J5:DiP-A, J5:OT, J5:OT-A, S1:DiP, S1:DiP-A, S1:OT, S1:OT-A, S5:DiP, S5:DiP-A, S5:OT, S5:OT-A
601	No	9	9-B	1000	McF_4	0	last	singleC	J1:DiP, J1:DiP-A, J1:OT, J1:OT-A, J5:CBN, J5:DiP, J5:DiP-A, J5:OT, J5:OT-A, S1:DiP, S1:DiP-A, S1:OT, S1:OT-A, S5:DiP, S5:DiP-A, S5:OT, S5:OT-A
602	No	9	9-B	1000	McF_4	0	last	wholeT_0.01	J1:DiP, J1:DiP-A, J1:OT, J1:OT-A, J5:CBN, J5:DiP, J5:DiP-A, J5:OT, J5:OT-A, S1:DiP, S1:DiP-A, S5:DiP, S5:DiP-A, S5:OT, S5:OT-A
603	No	9	9-B	1000	McF_4	0	last	wholeT_0.5	J1:DiP, J1:DiP-A, J1:OT, J1:OT-A, J5:CBN, J5:DiP, J5:DiP-A, J5:OT, J5:OT-A, S1:DiP-A, S1:OT, S1:OT-A, S5:DiP, S5:DiP-A, S5:OT, S5:OT-A
604	No	9	9-B	1000	McF_4	0	unif	singleC	J1:DiP, J1:DiP-A, J1:OT, J1:OT-A, J5:DiP, J5:DiP-A, J5:OT, J5:OT-A, S1:DiP, S1:DiP-A, S1:OT, S1:OT-A, S5:DiP, S5:DiP-A, S5:OT, S5:OT-A
605	No	9	9-B	1000	McF_4	0	unif	wholeT_0.01	J1:DiP, J1:DiP-A, J1:OT, J1:OT-A, J5:DiP, J5:DiP-A, J5:OT, J5:OT-A, S1:DiP-A, S5:DiP, S5:DiP-A, S5:OT, S5:OT-A

Table 8: *(continued)*

	Conjunction	Drivers	Tree	S.Size	Model	sh	S.Time	S.Type	Best method(s)
606	No	9	9-B	1000	McF_4	0	unif	wholeT_0.5	J1:DiP, J1:DiP-A, J1:OT, J1:OT-A, J5:DiP, J5:DiP-A, J5:OT, J5:OT-A, S1:DiP, S1:DiP-A, S1:OT, S1:OT-A, S5:DiP, S5:DiP-A, S5:OT, S5:OT-A
607	No	9	9-B	1000	McF_4	Inf	last	singleC	J1:DiP, J1:DiP-A, J1:OT, J1:OT-A, J5:DiP, J5:DiP-A, J5:OT, J5:OT-A, S1:OT, S1:OT-A, S5:DiP, S5:DiP-A, S5:OT, S5:OT-A
608	No	9	9-B	1000	McF_4	Inf	last	wholeT_0.01	J1:DiP, J1:DiP-A, J1:OT, J1:OT-A, J5:DiP, J5:DiP-A, J5:OT, J5:OT-A, S1:DiP, S1:DiP-A, S5:DiP, S5:DiP-A, S5:OT, S5:OT-A
609	No	9	9-B	1000	McF_4	Inf	last	wholeT_0.5	J1:DiP, J1:DiP-A, J1:OT, J1:OT-A, J5:DiP, J5:DiP-A, J5:OT, J5:OT-A, S1:DiP, S1:DiP-A, S1:OT, S1:OT-A, S5:DiP, S5:DiP-A, S5:OT, S5:OT-A
610	No	9	9-B	1000	McF_4	Inf	unif	singleC	J1:DiP, J1:DiP-A, J1:OT, J1:OT-A, J5:DiP, J5:DiP-A, J5:OT, J5:OT-A, S1:DiP, S1:DiP-A, S1:OT, S1:OT-A, S5:DiP, S5:DiP-A, S5:OT, S5:OT-A
611	No	9	9-B	1000	McF_4	Inf	unif	wholeT_0.01	J1:DiP, J1:DiP-A, J1:OT, J1:OT-A, J5:DiP, J5:DiP-A, J5:OT, J5:OT-A, S1:DiP-A, S5:DiP, S5:DiP-A, S5:OT, S5:OT-A
612	No	9	9-B	1000	McF_4	Inf	unif	wholeT_0.5	J1:DiP, J1:DiP-A, J1:OT, J1:OT-A, J5:DiP, J5:DiP-A, J5:OT, J5:OT-A, S1:DiP, S1:DiP-A, S1:OT, S1:OT-A, S5:DiP, S5:DiP-A, S5:OT, S5:OT-A
613	No	9	9-B	1000	McF_6	0	last	singleC	J1:DiP, J1:OT, J5:DiP, J5:OT, S1:DiP, S5:DiP, S5:OT
614	No	9	9-B	1000	McF_6	0	last	wholeT_0.01	J1:DiP, J1:OT, J5:DiP, J5:OT, S1:DiP, S5:DiP, S5:OT
615	No	9	9-B	1000	McF_6	0	last	wholeT_0.5	J1:DiP, J1:OT, J5:DiP, J5:OT, S1:DiP, S1:OT, S5:DiP, S5:OT
616	No	9	9-B	1000	McF_6	0	unif	singleC	J1:CBN, J1:CBN-A, J1:DiP, J1:DiP-A, J1:OT, J1:OT-A, J5:CBN, J5:CBN-A, J5:DiP, J5:DiP-A, J5:OT, J5:OT-A, S1:DiP, S1:DiP-A, S1:OT, S1:OT-A, S5:CBN, S5:CBN-A, S5:DiP, S5:DiP-A, S5:OT, S5:OT-A

Table 8: *(continued)*

	Conjunction	Drivers	Tree	S.Size	Model	sh	S.Time	S.Type	Best method(s)
617	No	9	9-B	1000	McF_6	0	unif	wholeT_0.01	J1:CBN, J1:CBN-A, J1:DiP, J1:DiP-A, J1:OT, J1:OT-A, J5:CBN, J5:CBN-A, J5:DiP, J5:DiP-A, J5:OT, J5:OT-A, S1:DiP, S1:DiP-A, S5:CBN, S5:DiP, S5:DiP-A, S5:OT, S5:OT-A
618	No	9	9-B	1000	McF_6	0	unif	wholeT_0.5	J1:CBN, J1:CBN-A, J1:DiP, J1:DiP-A, J1:OT, J1:OT-A, J5:CBN, J5:CBN-A, J5:DiP, J5:DiP-A, J5:OT, J5:OT-A, S1:DiP, S1:DiP-A, S1:OT, S1:OT-A, S5:CBN, S5:CBN-A, S5:DiP, S5:DiP-A, S5:OT, S5:OT-A
619	No	9	9-B	1000	McF_6	Inf	last	singleC	J1:DiP, J1:OT, J5:DiP, J5:OT, S1:DiP, S5:DiP, S5:OT
620	No	9	9-B	1000	McF_6	Inf	last	wholeT_0.01	J1:DiP, J1:OT, J5:DiP, J5:OT, S1:DiP, S5:DiP, S5:OT
621	No	9	9-B	1000	McF_6	Inf	last	wholeT_0.5	J1:DiP, J1:OT, J5:DiP, J5:OT, S1:DiP, S1:OT, S5:DiP, S5:OT
622	No	9	9-B	1000	McF_6	Inf	unif	singleC	J1:CBN, J1:CBN-A, J1:DiP, J1:DiP-A, J1:OT, J1:OT-A, J5:CBN-A, J5:DiP, J5:DiP-A, J5:OT, J5:OT-A, S1:DiP, S1:DiP-A, S1:OT, S1:OT-A, S5:CBN, S5:CBN-A, S5:DiP, S5:DiP-A, S5:OT, S5:OT-A
623	No	9	9-B	1000	McF_6	Inf	unif	wholeT_0.01	J1:CBN, J1:CBN-A, J1:DiP, J1:DiP-A, J1:OT, J1:OT-A, J5:CBN-A, J5:DiP, J5:DiP-A, J5:OT, J5:OT-A, S1:DiP, S5:CBN, S5:DiP, S5:DiP-A, S5:OT, S5:OT-A
624	No	9	9-B	1000	McF_6	Inf	unif	wholeT_0.5	J1:CBN, J1:CBN-A, J1:DiP, J1:DiP-A, J1:OT, J1:OT-A, J5:CBN-A, J5:DiP, J5:DiP-A, J5:OT, J5:OT-A, S1:DiP, S1:DiP-A, S1:OT, S1:OT-A, S5:CBN, S5:CBN-A, S5:DiP, S5:DiP-A, S5:OT, S5:OT-A
625	No	9	9-B	200	Bozic	0	last	singleC	J1:DiP, J1:DiP-A, J1:OT, J1:OT-A, J5:DiP, J5:DiP-A, J5:OT, J5:OT-A, S1:DiP, S1:DiP-A, S5:DiP, S5:DiP-A, S5:OT, S5:OT-A

Table 8: *(continued)*

	Conjunction	Drivers	Tree	S.Size	Model	sh	S.Time	S.Type	Best method(s)
626	No	9	9-B	200	Bozic	0	last	wholeT_0.01	J1:CBN-A, J1:DiP, J1:DiP-A, J1:OT, J1:OT-A, J5:CBN-A, J5:DiP, J5:DiP-A, J5:OT, J5:OT-A, S1:DiP, S1:DiP-A, S5:CBN-A, S5:DiP, S5:DiP-A, S5:OT, S5:OT-A
627	No	9	9-B	200	Bozic	0	last	wholeT_0.5	J1:DiP, J1:DiP-A, J1:OT, J1:OT-A, J5:CBN, J5:CBN-A, J5:DiP, J5:DiP-A, J5:OT, J5:OT-A, S1:DiP, S1:DiP-A, S5:DiP, S5:DiP-A, S5:OT, S5:OT-A
628	No	9	9-B	200	Bozic	0	unif	singleC	J1:DiP, J1:DiP-A, J1:OT, J1:OT-A, J5:CBN, J5:CBN-A, J5:DiP, J5:DiP-A, J5:OT, J5:OT-A, S1:DiP, S1:DiP-A, S5:DiP, S5:DiP-A, S5:OT, S5:OT-A
629	No	9	9-B	200	Bozic	0	unif	wholeT_0.01	J1:DiP, J1:DiP-A, J1:OT, J1:OT-A, J5:DiP, J5:DiP-A, J5:OT, J5:OT-A, S5:DiP, S5:DiP-A, S5:OT, S5:OT-A
630	No	9	9-B	200	Bozic	0	unif	wholeT_0.5	J1:DiP, J1:DiP-A, J1:OT, J1:OT-A, J5:CBN, J5:CBN-A, J5:DiP, J5:DiP-A, J5:OT, J5:OT-A, S1:DiP, S1:DiP-A, S1:OT, S1:OT-A, S5:DiP, S5:DiP-A, S5:OT, S5:OT-A
631	No	9	9-B	200	Bozic	Inf	last	singleC	J1:DiP, J1:DiP-A, J5:DiP, J5:DiP-A, J5:OT, J5:OT-A, S1:DiP, S1:DiP-A, S5:DiP, S5:DiP-A, S5:OT, S5:OT-A
632	No	9	9-B	200	Bozic	Inf	last	wholeT_0.01	J5:CBN-A, J5:DiP, J5:DiP-A, J5:OT, J5:OT-A
633	No	9	9-B	200	Bozic	Inf	last	wholeT_0.5	J1:DiP, J1:DiP-A, J1:OT, J1:OT-A, J5:DiP, J5:DiP-A, J5:OT, J5:OT-A, S1:DiP-A, S5:DiP, S5:DiP-A, S5:OT, S5:OT-A
634	No	9	9-B	200	Bozic	Inf	unif	singleC	J1:DiP, J1:DiP-A, J1:OT, J1:OT-A, J5:DiP, J5:DiP-A, J5:OT, J5:OT-A, S5:DiP, S5:DiP-A, S5:OT, S5:OT-A
635	No	9	9-B	200	Bozic	Inf	unif	wholeT_0.01	J5:DiP, J5:DiP-A, J5:OT, J5:OT-A
636	No	9	9-B	200	Bozic	Inf	unif	wholeT_0.5	J1:DiP, J1:DiP-A, J1:OT, J1:OT-A, J5:CBN, J5:DiP, J5:DiP-A, J5:OT, J5:OT-A, S5:DiP, S5:DiP-A, S5:OT, S5:OT-A

Table 8: *(continued)*

	Conjunction	Drivers	Tree	S.Size	Model	sh	S.Time	S.Type	Best method(s)
637	No	9	9-B	200	exp	0	last	singleC	J1:DiP, J1:DiP-A, J1:OT, J1:OT-A, J5:CBN, J5:CBN-A, J5:DiP, J5:DiP-A, J5:OT, J5:OT-A, S1:DiP, S1:DiP-A, S1:OT, S1:OT-A, S5:DiP, S5:DiP-A, S5:OT, S5:OT-A
638	No	9	9-B	200	exp	0	last	wholeT_0.01	J1:DiP, J1:DiP-A, J1:OT, J1:OT-A, J5:DiP, J5:DiP-A, J5:OT, J5:OT-A, S1:DiP, S1:DiP-A, S5:DiP, S5:DiP-A, S5:OT, S5:OT-A
639	No	9	9-B	200	exp	0	last	wholeT_0.5	J1:DiP, J1:DiP-A, J1:OT, J1:OT-A, J5:CBN, J5:CBN-A, J5:DiP, J5:DiP-A, J5:OT, J5:OT-A, S1:DiP, S1:DiP-A, S1:OT, S1:OT-A, S5:DiP, S5:DiP-A, S5:OT, S5:OT-A
640	No	9	9-B	200	exp	0	unif	singleC	J1:CBN, J1:CBN-A, J1:DiP, J1:DiP-A, J1:OT, J1:OT-A, J5:CBN, J5:CBN-A, J5:DiP, J5:DiP-A, J5:OT, J5:OT-A, S1:DiP, S1:DiP-A, S1:OT, S1:OT-A, S5:DiP, S5:DiP-A, S5:OT, S5:OT-A
641	No	9	9-B	200	exp	0	unif	wholeT_0.01	J1:DiP, J1:DiP-A, J1:OT, J1:OT-A, J5:CBN, J5:CBN-A, J5:DiP, J5:DiP-A, J5:OT, J5:OT-A, S1:DiP, S1:DiP-A, S1:OT, S1:OT-A, S5:DiP, S5:DiP-A, S5:OT, S5:OT-A
642	No	9	9-B	200	exp	0	unif	wholeT_0.5	J1:CBN, J1:CBN-A, J1:DiP, J1:DiP-A, J1:OT, J1:OT-A, J5:CBN, J5:CBN-A, J5:DiP, J5:DiP-A, J5:OT, J5:OT-A, S1:DiP, S1:DiP-A, S1:OT, S1:OT-A, S5:DiP, S5:DiP-A, S5:OT, S5:OT-A
643	No	9	9-B	200	exp	Inf	last	singleC	J1:DiP, J1:DiP-A, J1:OT, J1:OT-A, J5:DiP, J5:DiP-A, J5:OT, J5:OT-A, S1:DiP, S1:DiP-A, S5:DiP, S5:DiP-A, S5:OT, S5:OT-A
644	No	9	9-B	200	exp	Inf	last	wholeT_0.01	J5:CBN-A, J5:DiP, J5:DiP-A, J5:OT, J5:OT-A
645	No	9	9-B	200	exp	Inf	last	wholeT_0.5	J1:DiP, J1:DiP-A, J1:OT, J1:OT-A, J5:DiP, J5:DiP-A, J5:OT, J5:OT-A, S1:DiP, S1:DiP-A, S5:DiP, S5:DiP-A, S5:OT, S5:OT-A

Table 8: *(continued)*

	Conjunction	Drivers	Tree	S.Size	Model	sh	S.Time	S.Type	Best method(s)
646	No	9	9-B	200	exp	Inf	unif	singleC	J1:DiP, J1:DiP-A, J1:OT, J1:OT-A, J5:CBN, J5:CBN-A, J5:DiP, J5:DiP-A, J5:OT, J5:OT-A, S1:DiP, S1:DiP-A, S5:DiP, S5:DiP-A, S5:OT, S5:OT-A
647	No	9	9-B	200	exp	Inf	unif	wholeT_0.01	J5:DiP, J5:DiP-A, J5:OT, J5:OT-A, S5:DiP, S5:OT, S5:OT-A
648	No	9	9-B	200	exp	Inf	unif	wholeT_0.5	J1:DiP, J1:DiP-A, J1:OT, J1:OT-A, J5:CBN, J5:CBN-A, J5:DiP, J5:DiP-A, J5:OT, J5:OT-A, S1:DiP, S1:DiP-A, S1:OT, S1:OT-A, S5:DiP, S5:DiP-A, S5:OT, S5:OT-A
649	No	9	9-B	200	McF_4	0	last	singleC	J1:DiP, J1:DiP-A, J1:OT, J1:OT-A, J5:DiP, J5:DiP-A, J5:OT, J5:OT-A, S5:DiP, S5:DiP-A, S5:OT, S5:OT-A
650	No	9	9-B	200	McF_4	0	last	wholeT_0.01	J1:DiP, J1:DiP-A, J1:OT, J1:OT-A, J5:DiP, J5:DiP-A, J5:OT, J5:OT-A, S1:DiP, S1:DiP-A, S5:DiP, S5:DiP-A, S5:OT, S5:OT-A
651	No	9	9-B	200	McF_4	0	last	wholeT_0.5	J1:DiP, J1:DiP-A, J1:OT, J1:OT-A, J5:DiP, J5:DiP-A, J5:OT, J5:OT-A, S1:DiP-A, S5:DiP, S5:DiP-A, S5:OT, S5:OT-A
652	No	9	9-B	200	McF_4	0	unif	singleC	J1:DiP, J1:DiP-A, J1:OT, J1:OT-A, J5:DiP, J5:DiP-A, J5:OT, J5:OT-A, S1:DiP, S1:DiP-A, S5:DiP, S5:DiP-A, S5:OT, S5:OT-A
653	No	9	9-B	200	McF_4	0	unif	wholeT_0.01	J1:DiP, J1:DiP-A, J1:OT, J1:OT-A, J5:DiP, J5:DiP-A, J5:OT, J5:OT-A, S1:DiP, S1:DiP-A, S5:DiP, S5:DiP-A, S5:OT, S5:OT-A
654	No	9	9-B	200	McF_4	0	unif	wholeT_0.5	J1:DiP, J1:DiP-A, J1:OT, J1:OT-A, J5:DiP, J5:DiP-A, J5:OT, J5:OT-A, S1:DiP, S1:DiP-A, S5:DiP, S5:DiP-A, S5:OT, S5:OT-A
655	No	9	9-B	200	McF_4	Inf	last	singleC	J1:DiP, J1:DiP-A, J1:OT, J1:OT-A, J5:DiP, J5:DiP-A, J5:OT, J5:OT-A, S5:DiP, S5:DiP-A, S5:OT, S5:OT-A
656	No	9	9-B	200	McF_4	Inf	last	wholeT_0.01	J1:DiP, J1:DiP-A, J1:OT, J1:OT-A, J5:DiP, J5:DiP-A, J5:OT, J5:OT-A, S5:DiP, S5:DiP-A, S5:OT, S5:OT-A

Table 8: *(continued)*

	Conjunction	Drivers	Tree	S.Size	Model	sh	S.Time	S.Type	Best method(s)
657	No	9	9-B	200	McF_4	Inf	last	wholeT_0.5	J1:DiP, J1:DiP-A, J1:OT, J1:OT-A, J5:DiP, J5:DiP-A, J5:OT, J5:OT-A, S1:DiP, S1:DiP-A, S5:DiP, S5:DiP-A, S5:OT, S5:OT-A
658	No	9	9-B	200	McF_4	Inf	unif	singleC	J1:DiP, J1:DiP-A, J1:OT, J1:OT-A, J5:DiP, J5:DiP-A, J5:OT, J5:OT-A, S1:DiP, S1:DiP-A, S5:DiP, S5:DiP-A, S5:OT, S5:OT-A
659	No	9	9-B	200	McF_4	Inf	unif	wholeT_0.01	J1:DiP, J1:DiP-A, J1:OT, J1:OT-A, J5:DiP, J5:DiP-A, J5:OT, J5:OT-A, S5:DiP, S5:DiP-A, S5:OT, S5:OT-A
660	No	9	9-B	200	McF_4	Inf	unif	wholeT_0.5	J1:DiP, J1:DiP-A, J1:OT, J1:OT-A, J5:DiP, J5:DiP-A, J5:OT, J5:OT-A, S1:DiP, S1:DiP-A, S5:DiP, S5:DiP-A, S5:OT, S5:OT-A
661	No	9	9-B	200	McF_6	0	last	singleC	J1:DiP, J1:OT, J5:DiP, J5:OT, S1:DiP, S5:DiP, S5:OT
662	No	9	9-B	200	McF_6	0	last	wholeT_0.01	J1:DiP, J1:OT, J5:DiP, J5:OT, S1:DiP, S5:DiP, S5:OT
663	No	9	9-B	200	McF_6	0	last	wholeT_0.5	J1:DiP, J1:OT, J5:DiP, J5:OT, S1:DiP, S5:DiP, S5:OT
664	No	9	9-B	200	McF_6	0	unif	singleC	J1:CBN, J1:CBN-A, J1:DiP, J1:DiP-A, J1:OT, J1:OT-A, J5:CBN, J5:CBN-A, J5:DiP, J5:DiP-A, J5:OT, J5:OT-A, S1:DiP, S5:CBN, S5:CBN-A, S5:DiP, S5:DiP-A, S5:OT, S5:OT-A
665	No	9	9-B	200	McF_6	0	unif	wholeT_0.01	J1:CBN, J1:CBN-A, J1:DiP, J1:DiP-A, J1:OT, J1:OT-A, J5:CBN, J5:CBN-A, J5:DiP, J5:DiP-A, J5:OT, J5:OT-A, S1:DiP, S1:DiP-A, S5:CBN-A, S5:DiP, S5:DiP-A, S5:OT, S5:OT-A
666	No	9	9-B	200	McF_6	0	unif	wholeT_0.5	J1:CBN, J1:CBN-A, J1:DiP, J1:DiP-A, J1:OT, J1:OT-A, J5:CBN-A, J5:DiP, J5:DiP-A, J5:OT, J5:OT-A, S1:DiP, S5:CBN-A, S5:DiP, S5:DiP-A, S5:OT, S5:OT-A
667	No	9	9-B	200	McF_6	Inf	last	singleC	J1:DiP, J1:OT, J5:DiP, J5:OT, S1:DiP, S5:DiP, S5:OT
668	No	9	9-B	200	McF_6	Inf	last	wholeT_0.01	J1:DiP, J1:OT, J5:DiP, J5:OT, S1:DiP, S5:DiP, S5:OT

Table 8: *(continued)*

	Conjunction	Drivers	Tree	S.Size	Model	sh	S.Time	S.Type	Best method(s)
669	No	9	9-B	200	McF_6	Inf	last	wholeT_0.5	J1:DiP, J1:OT, J5:DiP, J5:OT, S1:DiP, S5:DiP, S5:OT
670	No	9	9-B	200	McF_6	Inf	unif	singleC	J1:CBN, J1:CBN-A, J1:DiP, J1:DiP-A, J1:OT, J1:OT-A, J5:CBN-A, J5:DiP, J5:DiP-A, J5:OT, J5:OT-A, S1:DiP, S5:CBN, S5:DiP, S5:OT, S5:OT-A
671	No	9	9-B	200	McF_6	Inf	unif	wholeT_0.01	J1:CBN, J1:CBN-A, J1:DiP, J1:OT, J1:OT-A, J5:CBN-A, J5:DiP, J5:DiP-A, J5:OT, J5:OT-A, S5:CBN, S5:OT, S5:OT-A
672	No	9	9-B	200	McF_6	Inf	unif	wholeT_0.5	J1:CBN, J1:CBN-A, J1:DiP, J1:DiP-A, J1:OT, J1:OT-A, J5:CBN-A, J5:DiP, J5:DiP-A, J5:OT, J5:OT-A, S5:CBN, S5:CBN-A, S5:DiP, S5:DiP-A, S5:OT, S5:OT-A
673	No	9	9-B	100	Bozic	0	last	singleC	J1:DiP, J1:DiP-A, J1:OT, J1:OT-A, J5:DiP, J5:DiP-A, J5:OT, J5:OT-A, S1:DiP, S1:DiP-A, S5:DiP, S5:DiP-A, S5:OT, S5:OT-A
674	No	9	9-B	100	Bozic	0	last	wholeT_0.01	J1:DiP, J1:DiP-A, J1:OT, J1:OT-A, J5:CBN-A, J5:DiP, J5:DiP-A, J5:OT, J5:OT-A, S1:DiP, S1:DiP-A, S5:DiP, S5:DiP-A, S5:OT, S5:OT-A
675	No	9	9-B	100	Bozic	0	last	wholeT_0.5	J1:DiP, J1:DiP-A, J1:OT, J1:OT-A, J5:DiP, J5:DiP-A, J5:OT, J5:OT-A, S1:DiP, S1:DiP-A, S5:DiP, S5:DiP-A, S5:OT, S5:OT-A
676	No	9	9-B	100	Bozic	0	unif	singleC	J1:DiP, J1:DiP-A, J1:OT, J1:OT-A, J5:CBN, J5:CBN-A, J5:DiP, J5:DiP-A, J5:OT, J5:OT-A, S1:DiP, S1:DiP-A, S5:DiP, S5:DiP-A, S5:OT, S5:OT-A
677	No	9	9-B	100	Bozic	0	unif	wholeT_0.01	J1:DiP, J1:DiP-A, J5:DiP, J5:DiP-A, J5:OT, J5:OT-A, S1:DiP, S1:DiP-A, S5:DiP, S5:DiP-A, S5:OT, S5:OT-A
678	No	9	9-B	100	Bozic	0	unif	wholeT_0.5	J1:DiP, J1:DiP-A, J1:OT, J1:OT-A, J5:CBN, J5:CBN-A, J5:DiP, J5:DiP-A, J5:OT, J5:OT-A, S1:DiP, S1:DiP-A, S5:DiP, S5:DiP-A, S5:OT, S5:OT-A

Table 8: *(continued)*

	Conjunction	Drivers	Tree	S.Size	Model	sh	S.Time	S.Type	Best method(s)
679	No	9	9-B	100	Bozic	Inf	last	singleC	J1:DiP, J1:DiP-A, J5:DiP, J5:DiP-A, J5:OT, J5:OT-A, S1:DiP, S1:DiP-A, S5:DiP, S5:DiP-A
680	No	9	9-B	100	Bozic	Inf	last	wholeT_0.01	J5:DiP, J5:DiP-A
681	No	9	9-B	100	Bozic	Inf	last	wholeT_0.5	J1:DiP, J1:DiP-A, J5:DiP, J5:DiP-A, J5:OT, J5:OT-A, S1:DiP, S1:DiP-A, S5:DiP, S5:DiP-A
682	No	9	9-B	100	Bozic	Inf	unif	singleC	J1:DiP, J1:DiP-A, J5:DiP, J5:DiP-A, J5:OT, J5:OT-A, S1:DiP, S1:DiP-A, S5:DiP, S5:DiP-A, S5:OT, S5:OT-A
683	No	9	9-B	100	Bozic	Inf	unif	wholeT_0.01	J5:OT, J5:OT-A
684	No	9	9-B	100	Bozic	Inf	unif	wholeT_0.5	J1:DiP, J1:DiP-A, J5:DiP, J5:DiP-A, J5:OT, J5:OT-A, S1:DiP, S1:DiP-A, S5:DiP, S5:DiP-A, S5:OT, S5:OT-A
685	No	9	9-B	100	exp	0	last	singleC	J1:DiP, J1:DiP-A, J1:OT, J1:OT-A, J5:CBN, J5:CBN-A, J5:DiP, J5:DiP-A, J5:OT, J5:OT-A, S1:DiP, S1:DiP-A, S1:OT, S1:OT-A, S5:DiP, S5:DiP-A, S5:OT, S5:OT-A
686	No	9	9-B	100	exp	0	last	wholeT_0.01	J1:DiP, J1:DiP-A, J1:OT, J1:OT-A, J5:DiP, J5:DiP-A, J5:OT, J5:OT-A, S1:DiP, S1:DiP-A, S5:DiP, S5:DiP-A, S5:OT, S5:OT-A
687	No	9	9-B	100	exp	0	last	wholeT_0.5	J1:DiP, J1:DiP-A, J1:OT, J1:OT-A, J5:CBN, J5:CBN-A, J5:DiP, J5:DiP-A, J5:OT, J5:OT-A, S1:DiP, S1:DiP-A, S1:OT, S1:OT-A, S5:DiP, S5:DiP-A, S5:OT, S5:OT-A
688	No	9	9-B	100	exp	0	unif	singleC	J1:DiP, J1:DiP-A, J1:OT, J1:OT-A, J5:CBN, J5:CBN-A, J5:DiP, J5:DiP-A, J5:OT, J5:OT-A, S1:DiP, S1:DiP-A, S1:OT, S1:OT-A, S5:DiP, S5:DiP-A, S5:OT, S5:OT-A
689	No	9	9-B	100	exp	0	unif	wholeT_0.01	J1:DiP, J1:DiP-A, J1:OT, J1:OT-A, J5:CBN, J5:CBN-A, J5:DiP, J5:DiP-A, J5:OT, J5:OT-A, S1:DiP, S1:DiP-A, S5:DiP, S5:DiP-A, S5:OT, S5:OT-A

Table 8: *(continued)*

	Conjunction	Drivers	Tree	S.Size	Model	sh	S.Time	S.Type	Best method(s)
690	No	9	9-B	100	exp	0	unif	wholeT_0.5	J1:DiP, J1:DiP-A, J1:OT, J1:OT-A, J5:CBN, J5:CBN-A, J5:DiP, J5:DiP-A, J5:OT, J5:OT-A, S1:DiP, S1:DiP-A, S1:OT, S1:OT-A, S5:DiP, S5:DiP-A, S5:OT, S5:OT-A
691	No	9	9-B	100	exp	Inf	last	singleC	J1:DiP, J1:DiP-A, J5:DiP, J5:DiP-A, J5:OT, J5:OT-A, S1:DiP, S1:DiP-A, S5:DiP, S5:DiP-A, S5:OT, S5:OT-A
692	No	9	9-B	100	exp	Inf	last	wholeT_0.01	J5:DiP, J5:DiP-A, J5:OT, J5:OT-A
693	No	9	9-B	100	exp	Inf	last	wholeT_0.5	J1:DiP, J1:DiP-A, J5:DiP, J5:DiP-A, J5:OT, J5:OT-A, S1:DiP, S1:DiP-A, S5:DiP, S5:DiP-A, S5:OT, S5:OT-A
694	No	9	9-B	100	exp	Inf	unif	singleC	J1:DiP, J1:DiP-A, J1:OT, J1:OT-A, J5:CBN, J5:CBN-A, J5:DiP, J5:DiP-A, J5:OT, J5:OT-A, S1:DiP, S1:DiP-A, S5:DiP, S5:DiP-A, S5:OT, S5:OT-A
695	No	9	9-B	100	exp	Inf	unif	wholeT_0.01	J5:DiP, J5:DiP-A, J5:OT, J5:OT-A, S5:DiP, S5:DiP-A, S5:OT, S5:OT-A
696	No	9	9-B	100	exp	Inf	unif	wholeT_0.5	J1:DiP, J1:DiP-A, J1:OT, J1:OT-A, J5:CBN, J5:CBN-A, J5:DiP, J5:DiP-A, J5:OT, J5:OT-A, S1:DiP, S1:DiP-A, S5:DiP, S5:DiP-A, S5:OT, S5:OT-A
697	No	9	9-B	100	McF_4	0	last	singleC	J1:DiP, J1:DiP-A, J1:OT, J1:OT-A, J5:DiP, J5:DiP-A, J5:OT, J5:OT-A, S1:DiP, S1:DiP-A, S5:DiP, S5:DiP-A, S5:OT, S5:OT-A
698	No	9	9-B	100	McF_4	0	last	wholeT_0.01	J1:DiP, J1:DiP-A, J5:DiP, J5:DiP-A, J5:OT, J5:OT-A, S1:DiP, S1:DiP-A, S5:DiP, S5:DiP-A, S5:OT, S5:OT-A
699	No	9	9-B	100	McF_4	0	last	wholeT_0.5	J1:DiP, J1:DiP-A, J1:OT, J1:OT-A, J5:DiP, J5:DiP-A, J5:OT, J5:OT-A, S1:DiP, S1:DiP-A, S5:DiP, S5:DiP-A, S5:OT, S5:OT-A
700	No	9	9-B	100	McF_4	0	unif	singleC	J1:DiP, J1:DiP-A, J1:OT, J1:OT-A, J5:DiP, J5:DiP-A, J5:OT, J5:OT-A, S1:DiP, S1:DiP-A, S5:DiP, S5:DiP-A, S5:OT, S5:OT-A
701	No	9	9-B	100	McF_4	0	unif	wholeT_0.01	J1:DiP, J1:DiP-A, J5:DiP, J5:DiP-A, J5:OT, J5:OT-A, S1:DiP, S1:DiP-A, S5:DiP, S5:DiP-A, S5:OT, S5:OT-A

Table 8: *(continued)*

	Conjunction	Drivers	Tree	S.Size	Model	sh	S.Time	S.Type	Best method(s)
702	No	9	9-B	100	McF_4	0	unif	wholeT_0.5	J1:DiP, J1:DiP-A, J1:OT, J1:OT-A, J5:DiP, J5:DiP-A, J5:OT, J5:OT-A, S1:DiP, S1:DiP-A, S5:DiP, S5:DiP-A, S5:OT, S5:OT-A
703	No	9	9-B	100	McF_4	Inf	last	singleC	J1:DiP, J1:DiP-A, J1:OT, J1:OT-A, J5:DiP, J5:DiP-A, J5:OT, J5:OT-A, S1:DiP, S1:DiP-A, S5:DiP, S5:DiP-A, S5:OT, S5:OT-A
704	No	9	9-B	100	McF_4	Inf	last	wholeT_0.01	J1:DiP, J1:DiP-A, J5:DiP, J5:DiP-A, J5:OT, J5:OT-A, S1:DiP, S1:DiP-A, S5:DiP, S5:DiP-A, S5:OT, S5:OT-A
705	No	9	9-B	100	McF_4	Inf	last	wholeT_0.5	J1:DiP, J1:DiP-A, J1:OT, J1:OT-A, J5:DiP, J5:DiP-A, J5:OT, J5:OT-A, S1:DiP, S1:DiP-A, S5:DiP, S5:DiP-A, S5:OT, S5:OT-A
706	No	9	9-B	100	McF_4	Inf	unif	singleC	J1:DiP, J1:DiP-A, J1:OT, J1:OT-A, J5:DiP, J5:DiP-A, J5:OT, J5:OT-A, S1:DiP, S1:DiP-A, S5:DiP, S5:DiP-A, S5:OT, S5:OT-A
707	No	9	9-B	100	McF_4	Inf	unif	wholeT_0.01	J1:DiP, J1:DiP-A, J5:DiP, J5:DiP-A, J5:OT, J5:OT-A, S5:DiP, S5:DiP-A, S5:OT, S5:OT-A
708	No	9	9-B	100	McF_4	Inf	unif	wholeT_0.5	J1:DiP, J1:DiP-A, J1:OT, J1:OT-A, J5:DiP, J5:DiP-A, J5:OT, J5:OT-A, S1:DiP, S1:DiP-A, S5:DiP, S5:DiP-A, S5:OT, S5:OT-A
709	No	9	9-B	100	McF_6	0	last	singleC	J1:DiP, J1:DiP-A, J1:OT, J5:DiP, J5:DiP-A, J5:OT, S1:DiP, S1:DiP-A, S5:DiP, S5:DiP-A, S5:OT
710	No	9	9-B	100	McF_6	0	last	wholeT_0.01	J1:DiP, J5:DiP, J5:OT, S1:DiP, S5:DiP, S5:OT
711	No	9	9-B	100	McF_6	0	last	wholeT_0.5	J1:DiP, J1:DiP-A, J1:OT, J5:DiP, J5:DiP-A, J5:OT, S1:DiP, S5:DiP, S5:OT
712	No	9	9-B	100	McF_6	0	unif	singleC	J1:CBN, J1:CBN-A, J1:DiP, J1:DiP-A, J1:OT, J1:OT-A, J5:CBN-A, J5:DiP, J5:DiP-A, J5:OT, J5:OT-A, S1:DiP, S1:DiP-A, S5:CBN-A, S5:DiP, S5:DiP-A, S5:OT, S5:OT-A

Table 8: *(continued)*

	Conjunction	Drivers	Tree	S.Size	Model	sh	S.Time	S.Type	Best method(s)
713	No	9	9-B	100	McF_6	0	unif	wholeT_0.01	J1:CBN, J1:CBN-A, J1:DiP, J1:DiP-A, J1:OT, J5:CBN-A, J5:DiP, J5:DiP-A, J5:OT, J5:OT-A, S1:DiP, S5:CBN, S5:DiP, S5:DiP-A, S5:OT, S5:OT-A
714	No	9	9-B	100	McF_6	0	unif	wholeT_0.5	J1:CBN, J1:CBN-A, J1:DiP, J1:DiP-A, J1:OT, J1:OT-A, J5:CBN-A, J5:DiP, J5:DiP-A, J5:OT, J5:OT-A, S1:DiP, S5:CBN, S5:CBN-A, S5:DiP, S5:OT, S5:OT-A
715	No	9	9-B	100	McF_6	Inf	last	singleC	J1:DiP, J1:DiP-A, J1:OT, J5:DiP, J5:DiP-A, J5:OT, S1:DiP, S1:DiP-A, S5:DiP, S5:DiP-A, S5:OT
716	No	9	9-B	100	McF_6	Inf	last	wholeT_0.01	J1:DiP, J5:DiP, J5:OT, S1:DiP, S5:DiP, S5:OT
717	No	9	9-B	100	McF_6	Inf	last	wholeT_0.5	J1:DiP, J1:DiP-A, J1:OT, J5:DiP, J5:DiP-A, J5:OT, S1:DiP, S1:DiP-A, S5:DiP, S5:DiP-A, S5:OT
718	No	9	9-B	100	McF_6	Inf	unif	singleC	J1:CBN, J1:CBN-A, J1:DiP, J1:DiP-A, J1:OT, J1:OT-A, J5:CBN-A, J5:DiP, J5:DiP-A, J5:OT, J5:OT-A, S1:DiP, S1:DiP-A, S5:CBN-A, S5:DiP, S5:DiP-A, S5:OT, S5:OT-A
719	No	9	9-B	100	McF_6	Inf	unif	wholeT_0.01	J1:DiP, J5:CBN, J5:CBN-A, J5:DiP, J5:DiP-A, J5:OT, J5:OT-A, S1:DiP, S5:CBN, S5:CBN-A, S5:DiP, S5:DiP-A, S5:OT, S5:OT-A
720	No	9	9-B	100	McF_6	Inf	unif	wholeT_0.5	J1:CBN, J1:CBN-A, J1:DiP, J1:DiP-A, J1:OT, J1:OT-A, J5:CBN-A, J5:DiP, J5:DiP-A, J5:OT, J5:OT-A, S1:DiP, S5:CBN, S5:CBN-A, S5:DiP, S5:DiP-A, S5:OT, S5:OT-A
721	No	7	7-B	1000	Bozic	0	last	singleC	J1:CBN, J1:CBN-A, J1:DiP, J1:DiP-A, J1:OT, J1:OT-A, J5:CBN, J5:CBN-A, J5:DiP, J5:DiP-A, J5:OT, J5:OT-A, S1:CBN, S1:DiP, S1:DiP-A, S1:OT, S1:OT-A, S5:CBN, S5:DiP, S5:DiP-A, S5:OT, S5:OT-A
722	No	7	7-B	1000	Bozic	0	last	wholeT_0.01	none

Table 8: *(continued)*

	Conjunction	Drivers	Tree	S.Size	Model	sh	S.Time	S.Type	Best method(s)
723	No	7	7-B	1000	Bozic	0	last	wholeT_0.5	J1:CBN, J1:CBN-A, J1:DiP, J1:DiP-A, J1:OT, J1:OT-A, J5:CBN, J5:CBN-A, J5:DiP, J5:DiP-A, J5:OT, J5:OT-A, S1:DiP, S1:DiP-A, S1:OT, S1:OT-A, S5:CBN, S5:CBN-A, S5:DiP, S5:DiP-A, S5:OT, S5:OT-A
724	No	7	7-B	1000	Bozic	0	unif	singleC	none
725	No	7	7-B	1000	Bozic	0	unif	wholeT_0.01	J5:CBN, J5:CBN-A, J5:DiP, J5:DiP-A, J5:OT, J5:OT-A
726	No	7	7-B	1000	Bozic	0	unif	wholeT_0.5	none
727	No	7	7-B	1000	Bozic	Inf	last	singleC	J1:DiP, J1:DiP-A, J1:OT, J1:OT-A, J5:DiP, J5:DiP-A, J5:OT, J5:OT-A, S1:DiP-A, S5:DiP, S5:DiP-A, S5:OT, S5:OT-A
728	No	7	7-B	1000	Bozic	Inf	last	wholeT_0.01	J5:CBN, J5:CBN-A, J5:DiP, J5:DiP-A, J5:OT, J5:OT-A
729	No	7	7-B	1000	Bozic	Inf	last	wholeT_0.5	J1:DiP, J1:DiP-A, J1:OT, J1:OT-A, J5:CBN, J5:CBN-A, J5:DiP, J5:DiP-A, J5:OT, J5:OT-A, S5:DiP, S5:DiP-A, S5:OT, S5:OT-A
730	No	7	7-B	1000	Bozic	Inf	unif	singleC	J1:CBN, J1:CBN-A, J5:CBN, J5:CBN-A, J5:DiP, J5:DiP-A, J5:OT, J5:OT-A, S1:CBN, S1:CBN-A, S5:CBN, S5:CBN-A, S5:DiP, S5:DiP-A, S5:OT, S5:OT-A
731	No	7	7-B	1000	Bozic	Inf	unif	wholeT_0.01	J5:CBN, J5:CBN-A, S5:CBN, S5:CBN-A
732	No	7	7-B	1000	Bozic	Inf	unif	wholeT_0.5	J1:CBN, J1:CBN-A, J1:DiP, J1:DiP-A, J1:OT, J1:OT-A, J5:CBN, J5:CBN-A, J5:DiP, J5:DiP-A, J5:OT, J5:OT-A, S1:CBN, S1:CBN-A, S5:CBN, S5:CBN-A, S5:DiP, S5:DiP-A, S5:OT, S5:OT-A
733	No	7	7-B	1000	exp	0	last	singleC	J1:CBN, J1:CBN-A, J1:DiP, J1:DiP-A, J1:OT, J1:OT-A, J5:CBN, J5:CBN-A, J5:DiP, J5:DiP-A, J5:OT, J5:OT-A, S1:DiP, S1:DiP-A, S1:OT, S1:OT-A, S5:CBN, S5:CBN-A, S5:DiP, S5:DiP-A, S5:OT, S5:OT-A
734	No	7	7-B	1000	exp	0	last	wholeT_0.01	none

Table 8: *(continued)*

	Conjunction	Drivers	Tree	S.Size	Model	sh	S.Time	S.Type	Best method(s)
735	No	7	7-B	1000	exp	0	last	wholeT_0.5	J1:CBN, J1:CBN-A, J1:DiP, J1:DiP-A, J1:OT, J1:OT-A, J5:CBN, J5:CBN-A, J5:DiP, J5:DiP-A, J5:OT, J5:OT-A, S1:DiP, S1:DiP-A, S1:OT, S1:OT-A, S5:CBN, S5:CBN-A, S5:DiP, S5:DiP-A, S5:OT, S5:OT-A
736	No	7	7-B	1000	exp	0	unif	singleC	none
737	No	7	7-B	1000	exp	0	unif	wholeT_0.01	J1:CBN, J1:CBN-A, J1:DiP, J1:DiP-A, J1:OT, J1:OT-A, J5:CBN, J5:CBN-A, J5:DiP, J5:DiP-A, J5:OT, J5:OT-A, S5:CBN, S5:CBN-A, S5:DiP, S5:DiP-A, S5:OT, S5:OT-A
738	No	7	7-B	1000	exp	0	unif	wholeT_0.5	none
739	No	7	7-B	1000	exp	Inf	last	singleC	J1:DiP, J1:DiP-A, J1:OT, J1:OT-A, J5:CBN, J5:CBN-A, J5:DiP, J5:DiP-A, J5:OT, J5:OT-A, S1:DiP, S1:DiP-A, S1:OT, S1:OT-A, S5:DiP, S5:DiP-A, S5:OT, S5:OT-A
740	No	7	7-B	1000	exp	Inf	last	wholeT_0.01	J1:CBN, J1:CBN-A, J1:DiP-A, J1:OT, J1:OT-A, J5:CBN, J5:CBN-A, J5:DiP, J5:DiP-A, J5:OT, J5:OT-A, S5:CBN, S5:CBN-A, S5:OT, S5:OT-A
741	No	7	7-B	1000	exp	Inf	last	wholeT_0.5	J1:CBN, J1:DiP, J1:DiP-A, J1:OT, J1:OT-A, J5:CBN, J5:CBN-A, J5:DiP, J5:DiP-A, J5:OT, J5:OT-A, S1:DiP, S1:DiP-A, S1:OT, S1:OT-A, S5:DiP, S5:DiP-A, S5:OT, S5:OT-A
742	No	7	7-B	1000	exp	Inf	unif	singleC	J1:CBN, J1:CBN-A, J1:DiP, J1:DiP-A, J1:OT, J1:OT-A, J5:CBN, J5:CBN-A, J5:DiP, J5:DiP-A, J5:OT, J5:OT-A, S1:CBN, S1:CBN-A, S1:DiP, S1:DiP-A, S5:CBN, S5:CBN-A, S5:DiP, S5:DiP-A, S5:OT, S5:OT-A
743	No	7	7-B	1000	exp	Inf	unif	wholeT_0.01	J1:CBN, J1:CBN-A, J5:CBN, J5:CBN-A, J5:DiP-A, S5:CBN, S5:CBN-A

Table 8: *(continued)*

	Conjunction	Drivers	Tree	S.Size	Model	sh	S.Time	S.Type	Best method(s)
744	No	7	7-B	1000	exp	Inf	unif	wholeT_0.5	J1:CBN, J1:CBN-A, J1:DiP, J1:DiP-A, J1:OT, J1:OT-A, J5:CBN, J5:CBN-A, J5:DiP, J5:DiP-A, J5:OT, J5:OT-A, S1:CBN, S1:CBN-A, S1:DiP, S1:DiP-A, S5:CBN, S5:CBN-A, S5:DiP, S5:DiP-A, S5:OT, S5:OT-A
745	No	7	7-B	1000	McF_4	0	last	singleC	J1:DiP, J1:DiP-A, J1:OT, J1:OT-A, J5:DiP, J5:DiP-A, J5:OT, J5:OT-A, S1:DiP, S1:DiP-A, S1:OT, S1:OT-A, S5:DiP, S5:DiP-A, S5:OT, S5:OT-A
746	No	7	7-B	1000	McF_4	0	last	wholeT_0.01	J1:CBN-A, J1:DiP, J1:DiP-A, J1:OT, J1:OT-A, J5:CBN-A, J5:DiP, J5:DiP-A, J5:OT, J5:OT-A, S1:DiP, S1:DiP-A, S5:DiP, S5:DiP-A, S5:OT, S5:OT-A
747	No	7	7-B	1000	McF_4	0	last	wholeT_0.5	J1:DiP, J1:DiP-A, J1:OT, J1:OT-A, J5:DiP, J5:DiP-A, J5:OT, J5:OT-A, S1:DiP, S1:DiP-A, S1:OT, S1:OT-A, S5:DiP, S5:DiP-A, S5:OT, S5:OT-A
748	No	7	7-B	1000	McF_4	0	unif	singleC	J1:CBN, J1:CBN-A, J1:DiP, J1:DiP-A, J1:OT, J1:OT-A, J5:CBN, J5:CBN-A, J5:DiP, J5:DiP-A, J5:OT, J5:OT-A, S1:DiP, S1:DiP-A, S1:OT, S1:OT-A, S5:CBN, S5:CBN-A, S5:DiP, S5:DiP-A, S5:OT, S5:OT-A
749	No	7	7-B	1000	McF_4	0	unif	wholeT_0.01	J1:CBN, J1:CBN-A, J1:DiP, J1:DiP-A, J1:OT, J1:OT-A, J5:CBN, J5:CBN-A, J5:DiP, J5:DiP-A, J5:OT, J5:OT-A, S1:DiP, S1:DiP-A, S5:CBN, S5:CBN-A, S5:DiP, S5:DiP-A, S5:OT, S5:OT-A
750	No	7	7-B	1000	McF_4	0	unif	wholeT_0.5	J1:CBN, J1:CBN-A, J1:DiP, J1:DiP-A, J1:OT, J1:OT-A, J5:CBN, J5:CBN-A, J5:DiP, J5:DiP-A, J5:OT, J5:OT-A, S1:DiP, S1:DiP-A, S1:OT, S1:OT-A, S5:CBN, S5:CBN-A, S5:DiP, S5:DiP-A, S5:OT, S5:OT-A
751	No	7	7-B	1000	McF_4	Inf	last	singleC	J1:DiP, J1:DiP-A, J1:OT, J1:OT-A, J5:DiP, J5:DiP-A, J5:OT, J5:OT-A, S1:DiP, S1:DiP-A, S1:OT, S1:OT-A, S5:DiP, S5:DiP-A, S5:OT, S5:OT-A

Table 8: *(continued)*

	Conjunction	Drivers	Tree	S.Size	Model	sh	S.Time	S.Type	Best method(s)
752	No	7	7-B	1000	McF_4	Inf	last	wholeT_0.01	J1:DiP, J1:DiP-A, J1:OT, J1:OT-A, J5:DiP, J5:DiP-A, J5:OT, J5:OT-A, S1:DiP, S1:DiP-A, S5:DiP, S5:DiP-A, S5:OT, S5:OT-A
753	No	7	7-B	1000	McF_4	Inf	last	wholeT_0.5	J1:DiP, J1:DiP-A, J1:OT, J1:OT-A, J5:DiP, J5:DiP-A, J5:OT, J5:OT-A, S1:DiP, S1:DiP-A, S1:OT, S1:OT-A, S5:DiP, S5:DiP-A, S5:OT, S5:OT-A
754	No	7	7-B	1000	McF_4	Inf	unif	singleC	J1:CBN, J1:CBN-A, J1:DiP, J1:DiP-A, J1:OT, J1:OT-A, J5:CBN, J5:CBN-A, J5:DiP, J5:DiP-A, J5:OT, J5:OT-A, S1:DiP, S1:DiP-A, S1:OT, S1:OT-A, S5:CBN, S5:CBN-A, S5:DiP, S5:DiP-A, S5:OT, S5:OT-A
755	No	7	7-B	1000	McF_4	Inf	unif	wholeT_0.01	J1:CBN, J1:CBN-A, J1:DiP, J1:DiP-A, J1:OT, J1:OT-A, J5:CBN, J5:CBN-A, J5:DiP, J5:DiP-A, J5:OT, J5:OT-A, S1:DiP, S1:DiP-A, S5:CBN, S5:CBN-A, S5:DiP, S5:DiP-A, S5:OT, S5:OT-A
756	No	7	7-B	1000	McF_4	Inf	unif	wholeT_0.5	J1:CBN, J1:CBN-A, J1:DiP, J1:DiP-A, J1:OT, J1:OT-A, J5:CBN, J5:CBN-A, J5:DiP, J5:DiP-A, J5:OT, J5:OT-A, S1:DiP, S1:DiP-A, S1:OT, S1:OT-A, S5:CBN, S5:CBN-A, S5:DiP, S5:DiP-A, S5:OT, S5:OT-A
757	No	7	7-B	1000	McF_6	0	last	singleC	J1:DiP, J1:DiP-A, J1:OT, J1:OT-A, J5:DiP, J5:DiP-A, J5:OT, J5:OT-A, S1:DiP, S1:DiP-A, S5:DiP, S5:DiP-A, S5:OT, S5:OT-A
758	No	7	7-B	1000	McF_6	0	last	wholeT_0.01	J1:DiP, J1:DiP-A, J5:DiP, J5:DiP-A, J5:OT, J5:OT-A, S1:DiP, S1:DiP-A, S5:DiP, S5:DiP-A, S5:OT, S5:OT-A
759	No	7	7-B	1000	McF_6	0	last	wholeT_0.5	J1:DiP, J1:DiP-A, J1:OT, J1:OT-A, J5:DiP, J5:DiP-A, J5:OT, J5:OT-A, S1:DiP, S1:DiP-A, S1:OT, S1:OT-A, S5:DiP, S5:DiP-A, S5:OT, S5:OT-A
760	No	7	7-B	1000	McF_6	0	unif	singleC	none

Table 8: *(continued)*

	Conjunction	Drivers	Tree	S.Size	Model	sh	S.Time	S.Type	Best method(s)
761	No	7	7-B	1000	McF_6	0	unif	wholeT_0.01	J1:CBN, J1:CBN-A, J1:DiP, J1:DiP-A, J1:OT, J1:OT-A, J5:CBN, J5:CBN-A, J5:DiP, J5:DiP-A, J5:OT, J5:OT-A, S1:DiP, S5:CBN, S5:CBN-A, S5:DiP, S5:DiP-A, S5:OT, S5:OT-A
762	No	7	7-B	1000	McF_6	0	unif	wholeT_0.5	none
763	No	7	7-B	1000	McF_6	Inf	last	singleC	J1:DiP, J1:DiP-A, J1:OT, J1:OT-A, J5:DiP, J5:DiP-A, J5:OT, J5:OT-A, S1:DiP, S1:DiP-A, S5:DiP, S5:DiP-A, S5:OT, S5:OT-A
764	No	7	7-B	1000	McF_6	Inf	last	wholeT_0.01	J1:DiP, J1:DiP-A, J5:DiP, J5:DiP-A, J5:OT, J5:OT-A, S1:DiP, S1:DiP-A, S5:DiP, S5:DiP-A, S5:OT, S5:OT-A
765	No	7	7-B	1000	McF_6	Inf	last	wholeT_0.5	J1:DiP, J1:DiP-A, J1:OT, J1:OT-A, J5:DiP, J5:DiP-A, J5:OT, J5:OT-A, S1:DiP, S1:DiP-A, S5:DiP, S5:DiP-A, S5:OT, S5:OT-A
766	No	7	7-B	1000	McF_6	Inf	unif	singleC	none
767	No	7	7-B	1000	McF_6	Inf	unif	wholeT_0.01	J1:CBN, J1:CBN-A, J1:DiP, J1:DiP-A, J1:OT, J1:OT-A, J5:CBN, J5:CBN-A, J5:DiP, J5:DiP-A, J5:OT, J5:OT-A, S5:CBN, S5:CBN-A, S5:DiP, S5:DiP-A, S5:OT, S5:OT-A
768	No	7	7-B	1000	McF_6	Inf	unif	wholeT_0.5	none
769	No	7	7-B	200	Bozic	0	last	singleC	J1:CBN, J1:CBN-A, J1:DiP, J1:DiP-A, J1:OT, J1:OT-A, J5:CBN, J5:CBN-A, J5:DiP, J5:DiP-A, J5:OT, J5:OT-A, S1:DiP, S1:DiP-A, S5:CBN, S5:DiP, S5:DiP-A, S5:OT, S5:OT-A
770	No	7	7-B	200	Bozic	0	last	wholeT_0.01	J1:CBN, J1:CBN-A, J1:DiP, J1:DiP-A, J1:OT, J1:OT-A, J5:CBN, J5:CBN-A, J5:DiP, J5:DiP-A, J5:OT, J5:OT-A, S1:DiP, S1:DiP-A, S5:CBN, S5:CBN-A, S5:DiP, S5:DiP-A, S5:OT, S5:OT-A
771	No	7	7-B	200	Bozic	0	last	wholeT_0.5	J1:CBN, J1:CBN-A, J1:DiP, J1:DiP-A, J1:OT, J1:OT-A, J5:CBN, J5:CBN-A, J5:DiP, J5:DiP-A, J5:OT, J5:OT-A, S1:DiP, S1:DiP-A, S1:OT, S5:CBN, S5:CBN-A, S5:DiP, S5:DiP-A, S5:OT, S5:OT-A

Table 8: *(continued)*

	Conjunction	Drivers	Tree	S.Size	Model	sh	S.Time	S.Type	Best method(s)
772	No	7	7-B	200	Bozic	0	unif	singleC	J1:CBN, J1:CBN-A, J1:DiP, J1:DiP-A, J1:OT, J1:OT-A, J5:CBN, J5:CBN-A, J5:DiP, J5:DiP-A, J5:OT, J5:OT-A, S1:DiP, S1:DiP-A, S1:OT, S1:OT-A, S5:CBN, S5:CBN-A, S5:DiP, S5:DiP-A, S5:OT, S5:OT-A
773	No	7	7-B	200	Bozic	0	unif	wholeT_0.01	J5:CBN, J5:CBN-A, J5:DiP, J5:DiP-A, J5:OT, J5:OT-A
774	No	7	7-B	200	Bozic	0	unif	wholeT_0.5	none
775	No	7	7-B	200	Bozic	Inf	last	singleC	J1:DiP, J1:DiP-A, J5:CBN, J5:CBN-A, J5:DiP, J5:DiP-A, J5:OT, J5:OT-A, S1:DiP, S5:DiP, S5:DiP-A, S5:OT, S5:OT-A
776	No	7	7-B	200	Bozic	Inf	last	wholeT_0.01	J5:CBN, J5:CBN-A, J5:OT, J5:OT-A
777	No	7	7-B	200	Bozic	Inf	last	wholeT_0.5	J1:DiP, J1:DiP-A, J1:OT, J1:OT-A, J5:CBN, J5:CBN-A, J5:DiP, J5:DiP-A, J5:OT, J5:OT-A, S1:DiP, S5:DiP, S5:DiP-A, S5:OT, S5:OT-A
778	No	7	7-B	200	Bozic	Inf	unif	singleC	J1:CBN, J1:CBN-A, J5:CBN, J5:CBN-A, J5:DiP, J5:DiP-A, J5:OT, J5:OT-A, S5:CBN, S5:CBN-A, S5:DiP, S5:DiP-A, S5:OT, S5:OT-A
779	No	7	7-B	200	Bozic	Inf	unif	wholeT_0.01	J5:CBN, J5:CBN-A, J5:OT, J5:OT-A, S5:CBN, S5:CBN-A, S5:OT, S5:OT-A
780	No	7	7-B	200	Bozic	Inf	unif	wholeT_0.5	J1:CBN, J1:CBN-A, J1:DiP, J1:DiP-A, J5:CBN, J5:CBN-A, J5:DiP, J5:DiP-A, J5:OT, J5:OT-A, S5:CBN, S5:CBN-A, S5:DiP, S5:DiP-A, S5:OT, S5:OT-A
781	No	7	7-B	200	exp	0	last	singleC	J1:CBN, J1:CBN-A, J1:DiP, J1:DiP-A, J1:OT, J1:OT-A, J5:CBN, J5:CBN-A, J5:DiP, J5:DiP-A, J5:OT, J5:OT-A, S1:DiP, S1:DiP-A, S1:OT, S1:OT-A, S5:CBN, S5:CBN-A, S5:DiP, S5:DiP-A, S5:OT, S5:OT-A
782	No	7	7-B	200	exp	0	last	wholeT_0.01	J1:CBN, J1:CBN-A, J1:DiP, J1:DiP-A, J1:OT, J1:OT-A, J5:CBN, J5:CBN-A, J5:DiP, J5:DiP-A, J5:OT, J5:OT-A, S1:CBN, S1:CBN-A, S1:DiP, S1:DiP-A, S5:CBN, S5:CBN-A, S5:DiP, S5:DiP-A
783	No	7	7-B	200	exp	0	last	wholeT_0.5	none

Table 8: *(continued)*

	Conjunction	Drivers	Tree	S.Size	Model	sh	S.Time	S.Type	Best method(s)
784	No	7	7-B	200	exp	0	unif	singleC	none
785	No	7	7-B	200	exp	0	unif	wholeT_0.01	J1:CBN, J1:CBN-A, J1:DiP, J1:DiP-A, J1:OT, J1:OT-A, J5:CBN, J5:CBN-A, J5:DiP, J5:DiP-A, J5:OT, J5:OT-A, S1:DiP, S1:DiP-A, S5:CBN, S5:CBN-A, S5:DiP, S5:DiP-A, S5:OT, S5:OT-A
786	No	7	7-B	200	exp	0	unif	wholeT_0.5	none
787	No	7	7-B	200	exp	Inf	last	singleC	J1:DiP, J1:DiP-A, J1:OT, J1:OT-A, J5:CBN, J5:CBN-A, J5:DiP, J5:DiP-A, J5:OT, J5:OT-A, S1:DiP, S1:DiP-A, S5:DiP, S5:DiP-A, S5:OT, S5:OT-A
788	No	7	7-B	200	exp	Inf	last	wholeT_0.01	J1:DiP, J5:CBN, J5:CBN-A, J5:DiP, J5:DiP-A, J5:OT, J5:OT-A, S5:DiP
789	No	7	7-B	200	exp	Inf	last	wholeT_0.5	J1:CBN, J1:DiP, J1:DiP-A, J1:OT, J1:OT-A, J5:CBN, J5:CBN-A, J5:DiP, J5:DiP-A, J5:OT, J5:OT-A, S1:DiP, S1:DiP-A, S5:DiP, S5:DiP-A, S5:OT, S5:OT-A
790	No	7	7-B	200	exp	Inf	unif	singleC	J1:CBN, J1:CBN-A, J1:DiP, J1:DiP-A, J1:OT, J1:OT-A, J5:CBN, J5:CBN-A, J5:DiP, J5:DiP-A, J5:OT, J5:OT-A, S1:DiP, S1:DiP-A, S5:CBN, S5:CBN-A, S5:DiP, S5:DiP-A, S5:OT, S5:OT-A
791	No	7	7-B	200	exp	Inf	unif	wholeT_0.01	J5:CBN, J5:CBN-A, J5:DiP, J5:DiP-A, J5:OT, J5:OT-A, S5:CBN, S5:CBN-A, S5:OT, S5:OT-A
792	No	7	7-B	200	exp	Inf	unif	wholeT_0.5	J1:CBN, J1:CBN-A, J1:DiP, J1:DiP-A, J1:OT, J1:OT-A, J5:CBN, J5:CBN-A, J5:DiP, J5:DiP-A, J5:OT, J5:OT-A, S1:CBN, S1:CBN-A, S1:DiP, S1:DiP-A, S5:CBN, S5:CBN-A, S5:DiP, S5:DiP-A, S5:OT, S5:OT-A
793	No	7	7-B	200	McF_4	0	last	singleC	J1:DiP, J1:DiP-A, J1:OT, J1:OT-A, J5:DiP, J5:DiP-A, J5:OT, J5:OT-A, S1:DiP, S1:DiP-A, S5:DiP, S5:DiP-A, S5:OT, S5:OT-A
794	No	7	7-B	200	McF_4	0	last	wholeT_0.01	J1:DiP, J1:DiP-A, J1:OT, J1:OT-A, J5:CBN-A, J5:DiP, J5:DiP-A, J5:OT, J5:OT-A, S1:DiP, S1:DiP-A, S5:DiP, S5:DiP-A, S5:OT, S5:OT-A

Table 8: *(continued)*

	Conjunction	Drivers	Tree	S.Size	Model	sh	S.Time	S.Type	Best method(s)
795	No	7	7-B	200	McF_4	0	last	wholeT_0.5	J1:DiP, J1:DiP-A, J1:OT, J1:OT-A, J5:DiP, J5:DiP-A, J5:OT, J5:OT-A, S1:DiP, S1:DiP-A, S5:DiP, S5:DiP-A, S5:OT, S5:OT-A
796	No	7	7-B	200	McF_4	0	unif	singleC	J1:CBN, J1:CBN-A, J1:DiP, J1:DiP-A, J1:OT, J1:OT-A, J5:CBN, J5:CBN-A, J5:DiP, J5:DiP-A, J5:OT, J5:OT-A, S1:DiP, S1:DiP-A, S5:CBN, S5:CBN-A, S5:DiP, S5:DiP-A, S5:OT, S5:OT-A
797	No	7	7-B	200	McF_4	0	unif	wholeT_0.01	J1:CBN, J1:CBN-A, J1:DiP, J1:DiP-A, J1:OT, J1:OT-A, J5:CBN, J5:CBN-A, J5:DiP, J5:DiP-A, J5:OT, J5:OT-A, S5:CBN, S5:CBN-A, S5:DiP, S5:DiP-A, S5:OT, S5:OT-A
798	No	7	7-B	200	McF_4	0	unif	wholeT_0.5	J1:CBN, J1:CBN-A, J1:DiP, J1:DiP-A, J1:OT, J1:OT-A, J5:CBN, J5:CBN-A, J5:DiP, J5:DiP-A, J5:OT, J5:OT-A, S1:DiP, S1:DiP-A, S5:CBN, S5:CBN-A, S5:DiP, S5:DiP-A, S5:OT, S5:OT-A
799	No	7	7-B	200	McF_4	Inf	last	singleC	J1:DiP, J1:DiP-A, J1:OT, J1:OT-A, J5:DiP, J5:DiP-A, J5:OT, J5:OT-A, S1:DiP, S1:DiP-A, S5:DiP, S5:DiP-A, S5:OT, S5:OT-A
800	No	7	7-B	200	McF_4	Inf	last	wholeT_0.01	J1:DiP, J1:DiP-A, J1:OT, J1:OT-A, J5:DiP, J5:DiP-A, J5:OT, J5:OT-A, S1:DiP, S1:DiP-A, S5:DiP, S5:DiP-A, S5:OT, S5:OT-A
801	No	7	7-B	200	McF_4	Inf	last	wholeT_0.5	J1:DiP, J1:DiP-A, J1:OT, J1:OT-A, J5:DiP, J5:DiP-A, J5:OT, J5:OT-A, S1:DiP, S1:DiP-A, S5:DiP, S5:DiP-A, S5:OT, S5:OT-A
802	No	7	7-B	200	McF_4	Inf	unif	singleC	J1:CBN, J1:CBN-A, J1:DiP, J1:DiP-A, J1:OT, J1:OT-A, J5:CBN, J5:CBN-A, J5:DiP, J5:DiP-A, J5:OT, J5:OT-A, S1:DiP, S1:DiP-A, S5:CBN, S5:CBN-A, S5:DiP, S5:DiP-A, S5:OT, S5:OT-A

Table 8: *(continued)*

	Conjunction	Drivers	Tree	S.Size	Model	sh	S.Time	S.Type	Best method(s)
803	No	7	7-B	200	McF_4	Inf	unif	wholeT_0.01	J1:CBN, J1:CBN-A, J1:DiP, J1:DiP-A, J1:OT, J1:OT-A, J5:CBN, J5:CBN-A, J5:DiP, J5:DiP-A, J5:OT, J5:OT-A, S1:DiP, S1:DiP-A, S5:CBN, S5:CBN-A, S5:DiP, S5:DiP-A, S5:OT, S5:OT-A
804	No	7	7-B	200	McF_4	Inf	unif	wholeT_0.5	J1:CBN, J1:CBN-A, J1:DiP, J1:DiP-A, J1:OT, J1:OT-A, J5:CBN, J5:CBN-A, J5:DiP, J5:DiP-A, J5:OT, J5:OT-A, S1:DiP, S1:DiP-A, S5:CBN, S5:CBN-A, S5:DiP, S5:DiP-A, S5:OT, S5:OT-A
805	No	7	7-B	200	McF_6	0	last	singleC	J1:DiP, J1:DiP-A, J1:OT, J1:OT-A, J5:DiP, J5:DiP-A, J5:OT, J5:OT-A, S1:DiP, S1:DiP-A, S5:DiP, S5:DiP-A, S5:OT, S5:OT-A
806	No	7	7-B	200	McF_6	0	last	wholeT_0.01	J1:DiP, J1:DiP-A, J5:DiP, J5:DiP-A, J5:OT, J5:OT-A, S1:DiP, S1:DiP-A, S5:DiP, S5:DiP-A, S5:OT, S5:OT-A
807	No	7	7-B	200	McF_6	0	last	wholeT_0.5	J1:DiP, J1:DiP-A, J1:OT, J1:OT-A, J5:DiP, J5:DiP-A, J5:OT, J5:OT-A, S1:DiP, S1:DiP-A, S5:DiP, S5:DiP-A, S5:OT, S5:OT-A
808	No	7	7-B	200	McF_6	0	unif	singleC	J1:CBN, J1:CBN-A, J1:DiP, J1:DiP-A, J1:OT, J1:OT-A, J5:CBN, J5:CBN-A, J5:DiP, J5:DiP-A, J5:OT, J5:OT-A, S1:DiP, S1:DiP-A, S5:CBN, S5:CBN-A, S5:DiP, S5:DiP-A, S5:OT, S5:OT-A
809	No	7	7-B	200	McF_6	0	unif	wholeT_0.01	J1:CBN, J1:CBN-A, J1:DiP, J1:DiP-A, J1:OT, J1:OT-A, J5:CBN, J5:CBN-A, J5:DiP, J5:DiP-A, J5:OT, J5:OT-A, S1:DiP, S1:DiP-A, S5:CBN, S5:CBN-A, S5:DiP, S5:DiP-A, S5:OT, S5:OT-A
810	No	7	7-B	200	McF_6	0	unif	wholeT_0.5	J1:CBN, J1:CBN-A, J1:DiP, J1:DiP-A, J1:OT, J1:OT-A, J5:CBN, J5:CBN-A, J5:DiP, J5:DiP-A, J5:OT, J5:OT-A, S1:DiP, S5:CBN, S5:CBN-A, S5:DiP, S5:OT, S5:OT-A
811	No	7	7-B	200	McF_6	Inf	last	singleC	J1:DiP, J1:DiP-A, J1:OT, J1:OT-A, J5:DiP, J5:DiP-A, J5:OT, J5:OT-A, S1:DiP, S1:DiP-A, S5:DiP, S5:DiP-A, S5:OT, S5:OT-A

Table 8: *(continued)*

	Conjunction	Drivers	Tree	S.Size	Model	sh	S.Time	S.Type	Best method(s)
812	No	7	7-B	200	McF_6	Inf	last	wholeT_0.01	J1:DiP, J5:DiP, J5:OT, J5:OT-A, S1:DiP, S5:DiP, S5:OT, S5:OT-A
813	No	7	7-B	200	McF_6	Inf	last	wholeT_0.5	J1:DiP, J1:DiP-A, J1:OT, J1:OT-A, J5:DiP, J5:DiP-A, J5:OT, J5:OT-A, S1:DiP, S1:DiP-A, S5:DiP, S5:DiP-A, S5:OT, S5:OT-A
814	No	7	7-B	200	McF_6	Inf	unif	singleC	J1:CBN, J1:CBN-A, J1:DiP, J1:DiP-A, J1:OT, J1:OT-A, J5:CBN, J5:CBN-A, J5:DiP, J5:DiP-A, J5:OT, J5:OT-A, S5:CBN, S5:CBN-A, S5:DiP, S5:OT, S5:OT-A
815	No	7	7-B	200	McF_6	Inf	unif	wholeT_0.01	J1:CBN, J1:CBN-A, J1:DiP, J1:DiP-A, J5:CBN, J5:CBN-A, J5:DiP, J5:DiP-A, S1:DiP, S5:CBN, S5:CBN-A, S5:DiP, S5:DiP-A
816	No	7	7-B	200	McF_6	Inf	unif	wholeT_0.5	J1:CBN, J1:CBN-A, J1:DiP, J1:DiP-A, J5:CBN, J5:CBN-A, J5:DiP, J5:DiP-A, S1:DiP, S5:CBN, S5:CBN-A, S5:DiP, S5:DiP-A
817	No	7	7-B	100	Bozic	0	last	singleC	J1:DiP, J1:DiP-A, J5:CBN, J5:CBN-A, J5:DiP, J5:DiP-A, J5:OT, J5:OT-A, S1:DiP, S1:DiP-A, S5:CBN, S5:DiP, S5:DiP-A, S5:OT, S5:OT-A
818	No	7	7-B	100	Bozic	0	last	wholeT_0.01	J1:DiP, J1:DiP-A, J1:OT, J5:CBN, J5:CBN-A, J5:DiP, J5:DiP-A, J5:OT, J5:OT-A, S1:DiP, S1:DiP-A, S5:CBN, S5:CBN-A, S5:DiP, S5:DiP-A, S5:OT, S5:OT-A
819	No	7	7-B	100	Bozic	0	last	wholeT_0.5	J1:CBN, J1:DiP, J1:DiP-A, J5:CBN, J5:CBN-A, J5:DiP, J5:DiP-A, J5:OT, J5:OT-A, S1:DiP, S1:DiP-A, S5:CBN, S5:CBN-A, S5:DiP, S5:DiP-A, S5:OT, S5:OT-A
820	No	7	7-B	100	Bozic	0	unif	singleC	J1:CBN, J1:CBN-A, J1:DiP, J1:DiP-A, J1:OT, J1:OT-A, J5:CBN, J5:CBN-A, J5:DiP, J5:DiP-A, J5:OT, J5:OT-A, S1:DiP, S1:DiP-A, S5:CBN, S5:CBN-A, S5:DiP, S5:DiP-A, S5:OT, S5:OT-A
821	No	7	7-B	100	Bozic	0	unif	wholeT_0.01	J5:CBN, J5:CBN-A, J5:DiP, J5:DiP-A, J5:OT, J5:OT-A

Table 8: *(continued)*

	Conjunction	Drivers	Tree	S.Size	Model	sh	S.Time	S.Type	Best method(s)
822	No	7	7-B	100	Bozic	0	unif	wholeT_0.5	J1:CBN, J1:CBN-A, J1:DiP, J1:DiP-A, J1:OT, J1:OT-A, J5:CBN, J5:CBN-A, J5:DiP, J5:DiP-A, J5:OT, J5:OT-A, S1:DiP, S1:DiP-A, S5:CBN, S5:CBN-A, S5:DiP, S5:DiP-A, S5:OT, S5:OT-A
823	No	7	7-B	100	Bozic	Inf	last	singleC	J1:DiP, J1:DiP-A, J5:CBN, J5:CBN-A, J5:DiP, J5:DiP-A, J5:OT, J5:OT-A, S1:DiP, S1:DiP-A, S5:DiP, S5:DiP-A
824	No	7	7-B	100	Bozic	Inf	last	wholeT_0.01	J5:CBN, J5:CBN-A, J5:DiP
825	No	7	7-B	100	Bozic	Inf	last	wholeT_0.5	J1:DiP, J1:DiP-A, J5:CBN, J5:CBN-A, J5:DiP, J5:DiP-A, J5:OT, J5:OT-A, S1:DiP, S1:DiP-A, S5:DiP, S5:DiP-A, S5:OT, S5:OT-A
826	No	7	7-B	100	Bozic	Inf	unif	singleC	J5:CBN, J5:CBN-A, J5:DiP, J5:DiP-A, J5:OT, J5:OT-A, S5:CBN, S5:CBN-A, S5:DiP, S5:DiP-A
827	No	7	7-B	100	Bozic	Inf	unif	wholeT_0.01	J5:CBN, J5:CBN-A, J5:OT, J5:OT-A, S5:CBN
828	No	7	7-B	100	Bozic	Inf	unif	wholeT_0.5	J5:CBN, J5:CBN-A, J5:DiP, J5:DiP-A, J5:OT, J5:OT-A, S5:CBN, S5:CBN-A, S5:DiP, S5:DiP-A, S5:OT, S5:OT-A
829	No	7	7-B	100	exp	0	last	singleC	J1:CBN, J1:CBN-A, J1:DiP, J1:DiP-A, J1:OT, J1:OT-A, J5:CBN, J5:CBN-A, J5:DiP, J5:DiP-A, J5:OT, J5:OT-A, S1:DiP, S1:DiP-A, S1:OT, S1:OT-A, S5:CBN, S5:CBN-A, S5:DiP, S5:DiP-A, S5:OT, S5:OT-A
830	No	7	7-B	100	exp	0	last	wholeT_0.01	J1:CBN, J1:CBN-A, J1:DiP, J1:DiP-A, J5:CBN, J5:CBN-A, J5:DiP, J5:DiP-A, J5:OT, J5:OT-A, S1:DiP, S1:DiP-A, S5:CBN, S5:CBN-A, S5:DiP, S5:DiP-A
831	No	7	7-B	100	exp	0	last	wholeT_0.5	J1:CBN, J1:CBN-A, J1:DiP, J1:DiP-A, J1:OT, J1:OT-A, J5:CBN, J5:CBN-A, J5:DiP, J5:DiP-A, J5:OT, J5:OT-A, S1:DiP, S1:DiP-A, S1:OT, S1:OT-A, S5:CBN, S5:CBN-A, S5:DiP, S5:DiP-A, S5:OT, S5:OT-A
832	No	7	7-B	100	exp	0	unif	singleC	none

Table 8: *(continued)*

	Conjunction	Drivers	Tree	S.Size	Model	sh	S.Time	S.Type	Best method(s)
833	No	7	7-B	100	exp	0	unif	wholeT_0.01	J1:DiP, J1:DiP-A, J5:CBN, J5:CBN-A, J5:DiP, J5:DiP-A, J5:OT, J5:OT-A, S1:DiP, S1:DiP-A, S5:CBN, S5:CBN-A, S5:DiP, S5:DiP-A, S5:OT, S5:OT-A
834	No	7	7-B	100	exp	0	unif	wholeT_0.5	none
835	No	7	7-B	100	exp	Inf	last	singleC	J1:DiP, J1:DiP-A, J1:OT, J1:OT-A, J5:CBN, J5:CBN-A, J5:DiP, J5:DiP-A, J5:OT, J5:OT-A, S1:DiP, S1:DiP-A, S5:DiP, S5:DiP-A, S5:OT, S5:OT-A
836	No	7	7-B	100	exp	Inf	last	wholeT_0.01	J5:DiP, J5:DiP-A, S5:DiP
837	No	7	7-B	100	exp	Inf	last	wholeT_0.5	J1:DiP, J1:DiP-A, J1:OT, J1:OT-A, J5:CBN, J5:CBN-A, J5:DiP, J5:DiP-A, J5:OT, J5:OT-A, S1:DiP, S1:DiP-A, S5:DiP, S5:DiP-A, S5:OT, S5:OT-A
838	No	7	7-B	100	exp	Inf	unif	singleC	J1:DiP, J1:DiP-A, J5:CBN, J5:CBN-A, J5:DiP, J5:DiP-A, J5:OT, J5:OT-A, S1:DiP, S1:DiP-A, S5:CBN, S5:CBN-A, S5:DiP, S5:DiP-A, S5:OT, S5:OT-A
839	No	7	7-B	100	exp	Inf	unif	wholeT_0.01	J5:CBN, J5:CBN-A, J5:DiP, J5:DiP-A, J5:OT, J5:OT-A
840	No	7	7-B	100	exp	Inf	unif	wholeT_0.5	J1:CBN, J1:DiP, J1:DiP-A, J5:CBN, J5:CBN-A, J5:DiP, J5:DiP-A, J5:OT, J5:OT-A, S1:DiP, S1:DiP-A, S5:CBN, S5:CBN-A, S5:DiP, S5:DiP-A, S5:OT, S5:OT-A
841	No	7	7-B	100	McF_4	0	last	singleC	J1:DiP, J1:DiP-A, J1:OT, J1:OT-A, J5:DiP, J5:DiP-A, J5:OT, J5:OT-A, S1:DiP, S1:DiP-A, S5:DiP, S5:DiP-A, S5:OT, S5:OT-A
842	No	7	7-B	100	McF_4	0	last	wholeT_0.01	J1:DiP, J1:DiP-A, J5:DiP, J5:DiP-A, J5:OT, J5:OT-A, S1:DiP, S1:DiP-A, S5:DiP, S5:DiP-A, S5:OT, S5:OT-A
843	No	7	7-B	100	McF_4	0	last	wholeT_0.5	J1:DiP, J1:DiP-A, J1:OT, J1:OT-A, J5:DiP, J5:DiP-A, J5:OT, J5:OT-A, S1:DiP, S1:DiP-A, S5:DiP, S5:DiP-A, S5:OT, S5:OT-A

Table 8: *(continued)*

	Conjunction	Drivers	Tree	S.Size	Model	sh	S.Time	S.Type	Best method(s)
844	No	7	7-B	100	McF_4	0	unif	singleC	J1:CBN, J1:CBN-A, J1:DiP, J1:DiP-A, J1:OT, J1:OT-A, J5:CBN, J5:CBN-A, J5:DiP, J5:DiP-A, J5:OT, J5:OT-A, S1:DiP, S1:DiP-A, S5:CBN, S5:CBN-A, S5:DiP, S5:DiP-A, S5:OT, S5:OT-A
845	No	7	7-B	100	McF_4	0	unif	wholeT_0.01	J1:CBN-A, J1:DiP, J1:DiP-A, J1:OT, J1:OT-A, J5:CBN, J5:CBN-A, J5:DiP, J5:DiP-A, J5:OT, J5:OT-A, S1:DiP, S1:DiP-A, S5:CBN, S5:CBN-A, S5:DiP, S5:DiP-A, S5:OT, S5:OT-A
846	No	7	7-B	100	McF_4	0	unif	wholeT_0.5	J1:CBN, J1:CBN-A, J1:DiP, J1:DiP-A, J1:OT, J1:OT-A, J5:CBN, J5:CBN-A, J5:DiP, J5:DiP-A, J5:OT, J5:OT-A, S1:DiP, S1:DiP-A, S5:CBN, S5:CBN-A, S5:DiP, S5:DiP-A, S5:OT, S5:OT-A
847	No	7	7-B	100	McF_4	Inf	last	singleC	J1:DiP, J1:DiP-A, J1:OT, J1:OT-A, J5:DiP, J5:DiP-A, J5:OT, J5:OT-A, S1:DiP, S1:DiP-A, S5:DiP, S5:DiP-A, S5:OT, S5:OT-A
848	No	7	7-B	100	McF_4	Inf	last	wholeT_0.01	J1:DiP, J1:DiP-A, J5:DiP, J5:DiP-A, J5:OT, J5:OT-A, S1:DiP, S1:DiP-A, S5:DiP, S5:DiP-A, S5:OT, S5:OT-A
849	No	7	7-B	100	McF_4	Inf	last	wholeT_0.5	J1:DiP, J1:DiP-A, J1:OT, J1:OT-A, J5:DiP, J5:DiP-A, J5:OT, J5:OT-A, S1:DiP, S1:DiP-A, S5:DiP, S5:DiP-A, S5:OT, S5:OT-A
850	No	7	7-B	100	McF_4	Inf	unif	singleC	J1:CBN, J1:CBN-A, J1:DiP, J1:DiP-A, J1:OT, J1:OT-A, J5:CBN, J5:CBN-A, J5:DiP, J5:DiP-A, J5:OT, J5:OT-A, S1:DiP, S1:DiP-A, S5:CBN, S5:CBN-A, S5:DiP, S5:DiP-A, S5:OT, S5:OT-A
851	No	7	7-B	100	McF_4	Inf	unif	wholeT_0.01	J1:CBN, J1:CBN-A, J1:DiP, J1:DiP-A, J5:CBN, J5:CBN-A, J5:DiP, J5:DiP-A, J5:OT, J5:OT-A, S1:DiP, S1:DiP-A, S5:CBN, S5:CBN-A, S5:DiP, S5:DiP-A, S5:OT, S5:OT-A
852	No	7	7-B	100	McF_4	Inf	unif	wholeT_0.5	J1:CBN, J1:CBN-A, J1:DiP, J1:DiP-A, J5:CBN, J5:CBN-A, J5:DiP, J5:DiP-A, S1:DiP, S1:DiP-A, S5:CBN, S5:CBN-A, S5:DiP, S5:DiP-A

Table 8: *(continued)*

	Conjunction	Drivers	Tree	S.Size	Model	sh	S.Time	S.Type	Best method(s)
853	No	7	7-B	100	McF_6	0	last	singleC	J1:DiP, J1:DiP-A, J5:DiP, J5:DiP-A, J5:OT, J5:OT-A, S1:DiP, S1:DiP-A, S5:DiP, S5:DiP-A, S5:OT, S5:OT-A
854	No	7	7-B	100	McF_6	0	last	wholeT_0.01	J1:DiP, J5:DiP, J5:OT, J5:OT-A, S1:DiP, S5:DiP, S5:OT, S5:OT-A
855	No	7	7-B	100	McF_6	0	last	wholeT_0.5	J1:DiP, J1:DiP-A, J5:DiP, J5:DiP-A, J5:OT, J5:OT-A, S1:DiP, S1:DiP-A, S5:DiP, S5:DiP-A, S5:OT, S5:OT-A
856	No	7	7-B	100	McF_6	0	unif	singleC	J1:CBN, J1:CBN-A, J1:DiP, J5:CBN, J5:CBN-A, J5:DiP, J5:DiP-A, S1:DiP, S5:CBN, S5:CBN-A, S5:DiP
857	No	7	7-B	100	McF_6	0	unif	wholeT_0.01	J1:DiP, J1:DiP-A, J5:CBN, J5:CBN-A, J5:DiP, J5:DiP-A, S5:CBN, S5:CBN-A, S5:DiP, S5:DiP-A
858	No	7	7-B	100	McF_6	0	unif	wholeT_0.5	J1:CBN, J1:CBN-A, J5:CBN, J5:CBN-A, J5:DiP, J5:DiP-A, S5:CBN, S5:CBN-A
859	No	7	7-B	100	McF_6	Inf	last	singleC	J1:DiP, J1:DiP-A, J5:DiP, J5:DiP-A, J5:OT, J5:OT-A, S1:DiP, S1:DiP-A, S5:DiP, S5:DiP-A, S5:OT, S5:OT-A
860	No	7	7-B	100	McF_6	Inf	last	wholeT_0.01	J5:DiP, J5:OT, J5:OT-A, S1:DiP, S5:DiP, S5:OT, S5:OT-A
861	No	7	7-B	100	McF_6	Inf	last	wholeT_0.5	J1:DiP, J1:DiP-A, J1:OT, J1:OT-A, J5:DiP, J5:DiP-A, J5:OT, J5:OT-A, S1:DiP, S1:DiP-A, S5:DiP, S5:DiP-A, S5:OT, S5:OT-A
862	No	7	7-B	100	McF_6	Inf	unif	singleC	J1:CBN, J1:CBN-A, J1:DiP, J5:CBN, J5:CBN-A, J5:DiP, J5:DiP-A, S5:CBN, S5:CBN-A
863	No	7	7-B	100	McF_6	Inf	unif	wholeT_0.01	J1:DiP, J5:CBN, J5:CBN-A, J5:DiP, J5:DiP-A, S5:CBN, S5:CBN-A, S5:DiP
864	No	7	7-B	100	McF_6	Inf	unif	wholeT_0.5	J1:CBN, J1:CBN-A, J1:DiP, J5:CBN, J5:CBN-A, J5:DiP, J5:DiP-A, S5:CBN, S5:CBN-A

4 Drivers Known (MCB)

4.1 Confidence sets (MCB), Diff, Drivers Known

Table 9: Confidence sets (method MCB) when Drivers are Known for measure Diff.

	Conjunction	Drivers	Tree	S.Size	Model	sh	S.Time	S.Type	Best method(s)
1	Yes	11	11-A	1000	Bozic	0	last	singleC	OT, OT-A
2	Yes	11	11-A	1000	Bozic	0	last	wholeT_0.01	CBN-A, DiP, DiP-A, OT, OT-A
3	Yes	11	11-A	1000	Bozic	0	last	wholeT_0.5	OT, OT-A
4	Yes	11	11-A	1000	Bozic	0	unif	singleC	OT, OT-A
5	Yes	11	11-A	1000	Bozic	0	unif	wholeT_0.01	CBN-A, OT, OT-A
6	Yes	11	11-A	1000	Bozic	0	unif	wholeT_0.5	OT, OT-A
7	Yes	11	11-A	1000	Bozic	Inf	last	singleC	OT, OT-A
8	Yes	11	11-A	1000	Bozic	Inf	last	wholeT_0.01	CBN-A, DiP, DiP-A, OT, OT-A
9	Yes	11	11-A	1000	Bozic	Inf	last	wholeT_0.5	OT, OT-A
10	Yes	11	11-A	1000	Bozic	Inf	unif	singleC	OT, OT-A
11	Yes	11	11-A	1000	Bozic	Inf	unif	wholeT_0.01	CBN-A, OT, OT-A
12	Yes	11	11-A	1000	Bozic	Inf	unif	wholeT_0.5	OT, OT-A
13	Yes	11	11-A	1000	exp	0	last	singleC	OT, OT-A
14	Yes	11	11-A	1000	exp	0	last	wholeT_0.01	OT, OT-A
15	Yes	11	11-A	1000	exp	0	last	wholeT_0.5	OT-A
16	Yes	11	11-A	1000	exp	0	unif	singleC	OT, OT-A
17	Yes	11	11-A	1000	exp	0	unif	wholeT_0.01	OT, OT-A
18	Yes	11	11-A	1000	exp	0	unif	wholeT_0.5	OT, OT-A
19	Yes	11	11-A	1000	exp	Inf	last	singleC	OT, OT-A
20	Yes	11	11-A	1000	exp	Inf	last	wholeT_0.01	OT, OT-A
21	Yes	11	11-A	1000	exp	Inf	last	wholeT_0.5	OT, OT-A
22	Yes	11	11-A	1000	exp	Inf	unif	singleC	OT, OT-A
23	Yes	11	11-A	1000	exp	Inf	unif	wholeT_0.01	CBN, OT, OT-A
24	Yes	11	11-A	1000	exp	Inf	unif	wholeT_0.5	OT, OT-A
25	Yes	11	11-A	1000	McF_4	0	last	singleC	OT, OT-A
26	Yes	11	11-A	1000	McF_4	0	last	wholeT_0.01	OT, OT-A
27	Yes	11	11-A	1000	McF_4	0	last	wholeT_0.5	OT, OT-A
28	Yes	11	11-A	1000	McF_4	0	unif	singleC	OT, OT-A
29	Yes	11	11-A	1000	McF_4	0	unif	wholeT_0.01	OT, OT-A
30	Yes	11	11-A	1000	McF_4	0	unif	wholeT_0.5	OT, OT-A
31	Yes	11	11-A	1000	McF_4	Inf	last	singleC	OT, OT-A
32	Yes	11	11-A	1000	McF_4	Inf	last	wholeT_0.01	OT, OT-A
33	Yes	11	11-A	1000	McF_4	Inf	last	wholeT_0.5	OT, OT-A
34	Yes	11	11-A	1000	McF_4	Inf	unif	singleC	OT, OT-A
35	Yes	11	11-A	1000	McF_4	Inf	unif	wholeT_0.01	OT, OT-A
36	Yes	11	11-A	1000	McF_4	Inf	unif	wholeT_0.5	OT, OT-A
37	Yes	11	11-A	1000	McF_6	0	last	singleC	DiP

Table 9: *(continued)*

	Conjunction	Drivers	Tree	S.Size	Model	sh	S.Time	S.Type	Best method(s)
38	Yes	11	11-A	1000	McF_6	0	last	wholeT_0.01	DiP
39	Yes	11	11-A	1000	McF_6	0	last	wholeT_0.5	DiP
40	Yes	11	11-A	1000	McF_6	0	unif	singleC	DiP, DiP-A, OT
41	Yes	11	11-A	1000	McF_6	0	unif	wholeT_0.01	DiP, DiP-A
42	Yes	11	11-A	1000	McF_6	0	unif	wholeT_0.5	DiP, DiP-A, OT, OT-A
43	Yes	11	11-A	1000	McF_6	Inf	last	singleC	OT
44	Yes	11	11-A	1000	McF_6	Inf	last	wholeT_0.01	OT
45	Yes	11	11-A	1000	McF_6	Inf	last	wholeT_0.5	DiP, OT
46	Yes	11	11-A	1000	McF_6	Inf	unif	singleC	DiP, DiP-A, OT
47	Yes	11	11-A	1000	McF_6	Inf	unif	wholeT_0.01	DiP, DiP-A, OT, OT-A
48	Yes	11	11-A	1000	McF_6	Inf	unif	wholeT_0.5	DiP, DiP-A, OT-A
49	Yes	11	11-A	200	Bozic	0	last	singleC	OT, OT-A
50	Yes	11	11-A	200	Bozic	0	last	wholeT_0.01	OT, OT-A
51	Yes	11	11-A	200	Bozic	0	last	wholeT_0.5	OT, OT-A
52	Yes	11	11-A	200	Bozic	0	unif	singleC	OT, OT-A
53	Yes	11	11-A	200	Bozic	0	unif	wholeT_0.01	OT, OT-A
54	Yes	11	11-A	200	Bozic	0	unif	wholeT_0.5	OT, OT-A
55	Yes	11	11-A	200	Bozic	Inf	last	singleC	OT, OT-A
56	Yes	11	11-A	200	Bozic	Inf	last	wholeT_0.01	OT, OT-A
57	Yes	11	11-A	200	Bozic	Inf	last	wholeT_0.5	OT, OT-A
58	Yes	11	11-A	200	Bozic	Inf	unif	singleC	OT, OT-A
59	Yes	11	11-A	200	Bozic	Inf	unif	wholeT_0.01	OT, OT-A
60	Yes	11	11-A	200	Bozic	Inf	unif	wholeT_0.5	OT, OT-A
61	Yes	11	11-A	200	exp	0	last	singleC	OT, OT-A
62	Yes	11	11-A	200	exp	0	last	wholeT_0.01	OT, OT-A
63	Yes	11	11-A	200	exp	0	last	wholeT_0.5	OT, OT-A
64	Yes	11	11-A	200	exp	0	unif	singleC	OT, OT-A
65	Yes	11	11-A	200	exp	0	unif	wholeT_0.01	OT, OT-A
66	Yes	11	11-A	200	exp	0	unif	wholeT_0.5	OT, OT-A
67	Yes	11	11-A	200	exp	Inf	last	singleC	OT, OT-A
68	Yes	11	11-A	200	exp	Inf	last	wholeT_0.01	CBN-A, OT, OT-A
69	Yes	11	11-A	200	exp	Inf	last	wholeT_0.5	OT, OT-A
70	Yes	11	11-A	200	exp	Inf	unif	singleC	OT, OT-A
71	Yes	11	11-A	200	exp	Inf	unif	wholeT_0.01	OT, OT-A
72	Yes	11	11-A	200	exp	Inf	unif	wholeT_0.5	OT, OT-A
73	Yes	11	11-A	200	McF_4	0	last	singleC	OT, OT-A
74	Yes	11	11-A	200	McF_4	0	last	wholeT_0.01	OT, OT-A
75	Yes	11	11-A	200	McF_4	0	last	wholeT_0.5	OT, OT-A
76	Yes	11	11-A	200	McF_4	0	unif	singleC	OT, OT-A
77	Yes	11	11-A	200	McF_4	0	unif	wholeT_0.01	OT, OT-A

Table 9: *(continued)*

	Conjunction	Drivers	Tree	S.Size	Model	sh	S.Time	S.Type	Best method(s)
78	Yes	11	11-A	200	McF_4	0	unif	wholeT_0.5	OT, OT-A
79	Yes	11	11-A	200	McF_4	Inf	last	singleC	OT, OT-A
80	Yes	11	11-A	200	McF_4	Inf	last	wholeT_0.01	OT, OT-A
81	Yes	11	11-A	200	McF_4	Inf	last	wholeT_0.5	OT, OT-A
82	Yes	11	11-A	200	McF_4	Inf	unif	singleC	OT, OT-A
83	Yes	11	11-A	200	McF_4	Inf	unif	wholeT_0.01	OT, OT-A
84	Yes	11	11-A	200	McF_4	Inf	unif	wholeT_0.5	OT, OT-A
85	Yes	11	11-A	200	McF_6	0	last	singleC	OT
86	Yes	11	11-A	200	McF_6	0	last	wholeT_0.01	OT
87	Yes	11	11-A	200	McF_6	0	last	wholeT_0.5	OT
88	Yes	11	11-A	200	McF_6	0	unif	singleC	OT, OT-A
89	Yes	11	11-A	200	McF_6	0	unif	wholeT_0.01	OT, OT-A
90	Yes	11	11-A	200	McF_6	0	unif	wholeT_0.5	OT, OT-A
91	Yes	11	11-A	200	McF_6	Inf	last	singleC	OT
92	Yes	11	11-A	200	McF_6	Inf	last	wholeT_0.01	OT, OT-A
93	Yes	11	11-A	200	McF_6	Inf	last	wholeT_0.5	OT
94	Yes	11	11-A	200	McF_6	Inf	unif	singleC	CBN, CBN-A, OT, OT-A
95	Yes	11	11-A	200	McF_6	Inf	unif	wholeT_0.01	CBN-A, OT, OT-A
96	Yes	11	11-A	200	McF_6	Inf	unif	wholeT_0.5	OT, OT-A
97	Yes	11	11-A	100	Bozic	0	last	singleC	OT, OT-A
98	Yes	11	11-A	100	Bozic	0	last	wholeT_0.01	OT, OT-A
99	Yes	11	11-A	100	Bozic	0	last	wholeT_0.5	OT, OT-A
100	Yes	11	11-A	100	Bozic	0	unif	singleC	OT, OT-A
101	Yes	11	11-A	100	Bozic	0	unif	wholeT_0.01	OT, OT-A
102	Yes	11	11-A	100	Bozic	0	unif	wholeT_0.5	OT, OT-A
103	Yes	11	11-A	100	Bozic	Inf	last	singleC	OT, OT-A
104	Yes	11	11-A	100	Bozic	Inf	last	wholeT_0.01	OT
105	Yes	11	11-A	100	Bozic	Inf	last	wholeT_0.5	OT, OT-A
106	Yes	11	11-A	100	Bozic	Inf	unif	singleC	OT, OT-A
107	Yes	11	11-A	100	Bozic	Inf	unif	wholeT_0.01	OT, OT-A
108	Yes	11	11-A	100	Bozic	Inf	unif	wholeT_0.5	OT, OT-A
109	Yes	11	11-A	100	exp	0	last	singleC	OT, OT-A
110	Yes	11	11-A	100	exp	0	last	wholeT_0.01	OT, OT-A
111	Yes	11	11-A	100	exp	0	last	wholeT_0.5	OT
112	Yes	11	11-A	100	exp	0	unif	singleC	OT, OT-A
113	Yes	11	11-A	100	exp	0	unif	wholeT_0.01	OT, OT-A
114	Yes	11	11-A	100	exp	0	unif	wholeT_0.5	OT, OT-A
115	Yes	11	11-A	100	exp	Inf	last	singleC	OT, OT-A
116	Yes	11	11-A	100	exp	Inf	last	wholeT_0.01	OT, OT-A
117	Yes	11	11-A	100	exp	Inf	last	wholeT_0.5	OT, OT-A

Table 9: *(continued)*

	Conjunction	Drivers	Tree	S.Size	Model	sh	S.Time	S.Type	Best method(s)
118	Yes	11	11-A	100	exp	Inf	unif	singleC	OT, OT-A
119	Yes	11	11-A	100	exp	Inf	unif	wholeT_0.01	OT, OT-A
120	Yes	11	11-A	100	exp	Inf	unif	wholeT_0.5	OT, OT-A
121	Yes	11	11-A	100	McF_4	0	last	singleC	OT, OT-A
122	Yes	11	11-A	100	McF_4	0	last	wholeT_0.01	OT
123	Yes	11	11-A	100	McF_4	0	last	wholeT_0.5	OT, OT-A
124	Yes	11	11-A	100	McF_4	0	unif	singleC	OT, OT-A
125	Yes	11	11-A	100	McF_4	0	unif	wholeT_0.01	OT, OT-A
126	Yes	11	11-A	100	McF_4	0	unif	wholeT_0.5	OT, OT-A
127	Yes	11	11-A	100	McF_4	Inf	last	singleC	OT, OT-A
128	Yes	11	11-A	100	McF_4	Inf	last	wholeT_0.01	OT, OT-A
129	Yes	11	11-A	100	McF_4	Inf	last	wholeT_0.5	OT, OT-A
130	Yes	11	11-A	100	McF_4	Inf	unif	singleC	OT, OT-A
131	Yes	11	11-A	100	McF_4	Inf	unif	wholeT_0.01	OT, OT-A
132	Yes	11	11-A	100	McF_4	Inf	unif	wholeT_0.5	OT, OT-A
133	Yes	11	11-A	100	McF_6	0	last	singleC	OT
134	Yes	11	11-A	100	McF_6	0	last	wholeT_0.01	OT, OT-A
135	Yes	11	11-A	100	McF_6	0	last	wholeT_0.5	OT
136	Yes	11	11-A	100	McF_6	0	unif	singleC	OT, OT-A
137	Yes	11	11-A	100	McF_6	0	unif	wholeT_0.01	OT, OT-A
138	Yes	11	11-A	100	McF_6	0	unif	wholeT_0.5	OT, OT-A
139	Yes	11	11-A	100	McF_6	Inf	last	singleC	OT
140	Yes	11	11-A	100	McF_6	Inf	last	wholeT_0.01	OT, OT-A
141	Yes	11	11-A	100	McF_6	Inf	last	wholeT_0.5	OT
142	Yes	11	11-A	100	McF_6	Inf	unif	singleC	OT, OT-A
143	Yes	11	11-A	100	McF_6	Inf	unif	wholeT_0.01	CBN-A, OT, OT-A
144	Yes	11	11-A	100	McF_6	Inf	unif	wholeT_0.5	OT, OT-A
145	Yes	9	9-A	1000	Bozic	0	last	singleC	OT, OT-A
146	Yes	9	9-A	1000	Bozic	0	last	wholeT_0.01	OT, OT-A
147	Yes	9	9-A	1000	Bozic	0	last	wholeT_0.5	OT, OT-A
148	Yes	9	9-A	1000	Bozic	0	unif	singleC	OT, OT-A
149	Yes	9	9-A	1000	Bozic	0	unif	wholeT_0.01	OT, OT-A
150	Yes	9	9-A	1000	Bozic	0	unif	wholeT_0.5	OT, OT-A
151	Yes	9	9-A	1000	Bozic	Inf	last	singleC	OT, OT-A
152	Yes	9	9-A	1000	Bozic	Inf	last	wholeT_0.01	OT, OT-A
153	Yes	9	9-A	1000	Bozic	Inf	last	wholeT_0.5	OT, OT-A
154	Yes	9	9-A	1000	Bozic	Inf	unif	singleC	OT, OT-A
155	Yes	9	9-A	1000	Bozic	Inf	unif	wholeT_0.01	CBN-A, OT, OT-A
156	Yes	9	9-A	1000	Bozic	Inf	unif	wholeT_0.5	OT, OT-A
157	Yes	9	9-A	1000	exp	0	last	singleC	OT, OT-A

Table 9: *(continued)*

	Conjunction	Drivers	Tree	S.Size	Model	sh	S.Time	S.Type	Best method(s)
158	Yes	9	9-A	1000	exp	0	last	wholeT_0.01	OT, OT-A
159	Yes	9	9-A	1000	exp	0	last	wholeT_0.5	OT, OT-A
160	Yes	9	9-A	1000	exp	0	unif	singleC	OT, OT-A
161	Yes	9	9-A	1000	exp	0	unif	wholeT_0.01	OT, OT-A
162	Yes	9	9-A	1000	exp	0	unif	wholeT_0.5	OT, OT-A
163	Yes	9	9-A	1000	exp	Inf	last	singleC	OT, OT-A
164	Yes	9	9-A	1000	exp	Inf	last	wholeT_0.01	CBN, OT, OT-A
165	Yes	9	9-A	1000	exp	Inf	last	wholeT_0.5	OT, OT-A
166	Yes	9	9-A	1000	exp	Inf	unif	singleC	OT, OT-A
167	Yes	9	9-A	1000	exp	Inf	unif	wholeT_0.01	OT, OT-A
168	Yes	9	9-A	1000	exp	Inf	unif	wholeT_0.5	OT, OT-A
169	Yes	9	9-A	1000	McF_4	0	last	singleC	OT, OT-A
170	Yes	9	9-A	1000	McF_4	0	last	wholeT_0.01	DiP, DiP-A, OT, OT-A
171	Yes	9	9-A	1000	McF_4	0	last	wholeT_0.5	OT, OT-A
172	Yes	9	9-A	1000	McF_4	0	unif	singleC	OT, OT-A
173	Yes	9	9-A	1000	McF_4	0	unif	wholeT_0.01	DiP-A, OT, OT-A
174	Yes	9	9-A	1000	McF_4	0	unif	wholeT_0.5	OT, OT-A
175	Yes	9	9-A	1000	McF_4	Inf	last	singleC	OT, OT-A
176	Yes	9	9-A	1000	McF_4	Inf	last	wholeT_0.01	OT
177	Yes	9	9-A	1000	McF_4	Inf	last	wholeT_0.5	OT, OT-A
178	Yes	9	9-A	1000	McF_4	Inf	unif	singleC	OT, OT-A
179	Yes	9	9-A	1000	McF_4	Inf	unif	wholeT_0.01	OT, OT-A
180	Yes	9	9-A	1000	McF_4	Inf	unif	wholeT_0.5	OT, OT-A
181	Yes	9	9-A	1000	McF_6	0	last	singleC	OT
182	Yes	9	9-A	1000	McF_6	0	last	wholeT_0.01	DiP, OT
183	Yes	9	9-A	1000	McF_6	0	last	wholeT_0.5	OT
184	Yes	9	9-A	1000	McF_6	0	unif	singleC	DiP, DiP-A, OT, OT-A
185	Yes	9	9-A	1000	McF_6	0	unif	wholeT_0.01	DiP, DiP-A, OT, OT-A
186	Yes	9	9-A	1000	McF_6	0	unif	wholeT_0.5	DiP, DiP-A, OT, OT-A
187	Yes	9	9-A	1000	McF_6	Inf	last	singleC	OT
188	Yes	9	9-A	1000	McF_6	Inf	last	wholeT_0.01	OT
189	Yes	9	9-A	1000	McF_6	Inf	last	wholeT_0.5	DiP, OT
190	Yes	9	9-A	1000	McF_6	Inf	unif	singleC	CBN, OT, OT-A
191	Yes	9	9-A	1000	McF_6	Inf	unif	wholeT_0.01	CBN-A, OT, OT-A
192	Yes	9	9-A	1000	McF_6	Inf	unif	wholeT_0.5	OT, OT-A
193	Yes	9	9-A	200	Bozic	0	last	singleC	OT, OT-A
194	Yes	9	9-A	200	Bozic	0	last	wholeT_0.01	OT, OT-A
195	Yes	9	9-A	200	Bozic	0	last	wholeT_0.5	OT, OT-A
196	Yes	9	9-A	200	Bozic	0	unif	singleC	OT, OT-A
197	Yes	9	9-A	200	Bozic	0	unif	wholeT_0.01	OT, OT-A

Table 9: *(continued)*

	Conjunction	Drivers	Tree	S.Size	Model	sh	S.Time	S.Type	Best method(s)
198	Yes	9	9-A	200	Bozic	0	unif	wholeT_0.5	OT, OT-A
199	Yes	9	9-A	200	Bozic	Inf	last	singleC	OT, OT-A
200	Yes	9	9-A	200	Bozic	Inf	last	wholeT_0.01	OT, OT-A
201	Yes	9	9-A	200	Bozic	Inf	last	wholeT_0.5	OT, OT-A
202	Yes	9	9-A	200	Bozic	Inf	unif	singleC	OT, OT-A
203	Yes	9	9-A	200	Bozic	Inf	unif	wholeT_0.01	OT, OT-A
204	Yes	9	9-A	200	Bozic	Inf	unif	wholeT_0.5	OT, OT-A
205	Yes	9	9-A	200	exp	0	last	singleC	OT, OT-A
206	Yes	9	9-A	200	exp	0	last	wholeT_0.01	OT, OT-A
207	Yes	9	9-A	200	exp	0	last	wholeT_0.5	OT, OT-A
208	Yes	9	9-A	200	exp	0	unif	singleC	OT, OT-A
209	Yes	9	9-A	200	exp	0	unif	wholeT_0.01	OT, OT-A
210	Yes	9	9-A	200	exp	0	unif	wholeT_0.5	OT, OT-A
211	Yes	9	9-A	200	exp	Inf	last	singleC	OT, OT-A
212	Yes	9	9-A	200	exp	Inf	last	wholeT_0.01	OT, OT-A
213	Yes	9	9-A	200	exp	Inf	last	wholeT_0.5	OT, OT-A
214	Yes	9	9-A	200	exp	Inf	unif	singleC	OT, OT-A
215	Yes	9	9-A	200	exp	Inf	unif	wholeT_0.01	OT, OT-A
216	Yes	9	9-A	200	exp	Inf	unif	wholeT_0.5	OT
217	Yes	9	9-A	200	McF_4	0	last	singleC	OT, OT-A
218	Yes	9	9-A	200	McF_4	0	last	wholeT_0.01	OT, OT-A
219	Yes	9	9-A	200	McF_4	0	last	wholeT_0.5	OT, OT-A
220	Yes	9	9-A	200	McF_4	0	unif	singleC	OT, OT-A
221	Yes	9	9-A	200	McF_4	0	unif	wholeT_0.01	OT, OT-A
222	Yes	9	9-A	200	McF_4	0	unif	wholeT_0.5	OT, OT-A
223	Yes	9	9-A	200	McF_4	Inf	last	singleC	OT, OT-A
224	Yes	9	9-A	200	McF_4	Inf	last	wholeT_0.01	OT
225	Yes	9	9-A	200	McF_4	Inf	last	wholeT_0.5	OT, OT-A
226	Yes	9	9-A	200	McF_4	Inf	unif	singleC	OT, OT-A
227	Yes	9	9-A	200	McF_4	Inf	unif	wholeT_0.01	OT, OT-A
228	Yes	9	9-A	200	McF_4	Inf	unif	wholeT_0.5	OT, OT-A
229	Yes	9	9-A	200	McF_6	0	last	singleC	OT
230	Yes	9	9-A	200	McF_6	0	last	wholeT_0.01	OT
231	Yes	9	9-A	200	McF_6	0	last	wholeT_0.5	OT
232	Yes	9	9-A	200	McF_6	0	unif	singleC	CBN, OT, OT-A
233	Yes	9	9-A	200	McF_6	0	unif	wholeT_0.01	OT, OT-A
234	Yes	9	9-A	200	McF_6	0	unif	wholeT_0.5	OT, OT-A
235	Yes	9	9-A	200	McF_6	Inf	last	singleC	OT
236	Yes	9	9-A	200	McF_6	Inf	last	wholeT_0.01	OT
237	Yes	9	9-A	200	McF_6	Inf	last	wholeT_0.5	OT

Table 9: *(continued)*

	Conjunction	Drivers	Tree	S.Size	Model	sh	S.Time	S.Type	Best method(s)
238	Yes	9	9-A	200	McF_6	Inf	unif	singleC	OT, OT-A
239	Yes	9	9-A	200	McF_6	Inf	unif	wholeT_0.01	OT, OT-A
240	Yes	9	9-A	200	McF_6	Inf	unif	wholeT_0.5	OT, OT-A
241	Yes	9	9-A	100	Bozic	0	last	singleC	OT, OT-A
242	Yes	9	9-A	100	Bozic	0	last	wholeT_0.01	OT, OT-A
243	Yes	9	9-A	100	Bozic	0	last	wholeT_0.5	OT, OT-A
244	Yes	9	9-A	100	Bozic	0	unif	singleC	OT, OT-A
245	Yes	9	9-A	100	Bozic	0	unif	wholeT_0.01	OT, OT-A
246	Yes	9	9-A	100	Bozic	0	unif	wholeT_0.5	OT, OT-A
247	Yes	9	9-A	100	Bozic	Inf	last	singleC	OT, OT-A
248	Yes	9	9-A	100	Bozic	Inf	last	wholeT_0.01	OT, OT-A
249	Yes	9	9-A	100	Bozic	Inf	last	wholeT_0.5	OT, OT-A
250	Yes	9	9-A	100	Bozic	Inf	unif	singleC	OT, OT-A
251	Yes	9	9-A	100	Bozic	Inf	unif	wholeT_0.01	OT, OT-A
252	Yes	9	9-A	100	Bozic	Inf	unif	wholeT_0.5	OT, OT-A
253	Yes	9	9-A	100	exp	0	last	singleC	OT, OT-A
254	Yes	9	9-A	100	exp	0	last	wholeT_0.01	OT, OT-A
255	Yes	9	9-A	100	exp	0	last	wholeT_0.5	OT, OT-A
256	Yes	9	9-A	100	exp	0	unif	singleC	OT, OT-A
257	Yes	9	9-A	100	exp	0	unif	wholeT_0.01	OT, OT-A
258	Yes	9	9-A	100	exp	0	unif	wholeT_0.5	OT, OT-A
259	Yes	9	9-A	100	exp	Inf	last	singleC	OT, OT-A
260	Yes	9	9-A	100	exp	Inf	last	wholeT_0.01	OT, OT-A
261	Yes	9	9-A	100	exp	Inf	last	wholeT_0.5	OT, OT-A
262	Yes	9	9-A	100	exp	Inf	unif	singleC	OT, OT-A
263	Yes	9	9-A	100	exp	Inf	unif	wholeT_0.01	OT, OT-A
264	Yes	9	9-A	100	exp	Inf	unif	wholeT_0.5	OT, OT-A
265	Yes	9	9-A	100	McF_4	0	last	singleC	OT, OT-A
266	Yes	9	9-A	100	McF_4	0	last	wholeT_0.01	OT, OT-A
267	Yes	9	9-A	100	McF_4	0	last	wholeT_0.5	OT, OT-A
268	Yes	9	9-A	100	McF_4	0	unif	singleC	OT-A
269	Yes	9	9-A	100	McF_4	0	unif	wholeT_0.01	OT, OT-A
270	Yes	9	9-A	100	McF_4	0	unif	wholeT_0.5	OT, OT-A
271	Yes	9	9-A	100	McF_4	Inf	last	singleC	OT, OT-A
272	Yes	9	9-A	100	McF_4	Inf	last	wholeT_0.01	OT
273	Yes	9	9-A	100	McF_4	Inf	last	wholeT_0.5	OT, OT-A
274	Yes	9	9-A	100	McF_4	Inf	unif	singleC	OT, OT-A
275	Yes	9	9-A	100	McF_4	Inf	unif	wholeT_0.01	OT, OT-A
276	Yes	9	9-A	100	McF_4	Inf	unif	wholeT_0.5	OT, OT-A
277	Yes	9	9-A	100	McF_6	0	last	singleC	OT

Table 9: *(continued)*

	Conjunction	Drivers	Tree	S.Size	Model	sh	S.Time	S.Type	Best method(s)
278	Yes	9	9-A	100	McF_6	0	last	wholeT_0.01	OT
279	Yes	9	9-A	100	McF_6	0	last	wholeT_0.5	OT
280	Yes	9	9-A	100	McF_6	0	unif	singleC	OT
281	Yes	9	9-A	100	McF_6	0	unif	wholeT_0.01	OT, OT-A
282	Yes	9	9-A	100	McF_6	0	unif	wholeT_0.5	OT, OT-A
283	Yes	9	9-A	100	McF_6	Inf	last	singleC	OT
284	Yes	9	9-A	100	McF_6	Inf	last	wholeT_0.01	OT
285	Yes	9	9-A	100	McF_6	Inf	last	wholeT_0.5	OT
286	Yes	9	9-A	100	McF_6	Inf	unif	singleC	OT, OT-A
287	Yes	9	9-A	100	McF_6	Inf	unif	wholeT_0.01	OT, OT-A
288	Yes	9	9-A	100	McF_6	Inf	unif	wholeT_0.5	OT, OT-A
289	Yes	7	7-A	1000	Bozic	0	last	singleC	OT-A
290	Yes	7	7-A	1000	Bozic	0	last	wholeT_0.01	OT-A
291	Yes	7	7-A	1000	Bozic	0	last	wholeT_0.5	OT-A
292	Yes	7	7-A	1000	Bozic	0	unif	singleC	OT, OT-A
293	Yes	7	7-A	1000	Bozic	0	unif	wholeT_0.01	CBN, CBN-A
294	Yes	7	7-A	1000	Bozic	0	unif	wholeT_0.5	OT, OT-A
295	Yes	7	7-A	1000	Bozic	Inf	last	singleC	OT-A
296	Yes	7	7-A	1000	Bozic	Inf	last	wholeT_0.01	OT-A
297	Yes	7	7-A	1000	Bozic	Inf	last	wholeT_0.5	OT-A
298	Yes	7	7-A	1000	Bozic	Inf	unif	singleC	CBN, CBN-A
299	Yes	7	7-A	1000	Bozic	Inf	unif	wholeT_0.01	OT, OT-A
300	Yes	7	7-A	1000	Bozic	Inf	unif	wholeT_0.5	CBN, CBN-A
301	Yes	7	7-A	1000	exp	0	last	singleC	OT-A
302	Yes	7	7-A	1000	exp	0	last	wholeT_0.01	OT-A
303	Yes	7	7-A	1000	exp	0	last	wholeT_0.5	OT-A
304	Yes	7	7-A	1000	exp	0	unif	singleC	OT, OT-A
305	Yes	7	7-A	1000	exp	0	unif	wholeT_0.01	CBN, CBN-A, OT, OT-A
306	Yes	7	7-A	1000	exp	0	unif	wholeT_0.5	OT, OT-A
307	Yes	7	7-A	1000	exp	Inf	last	singleC	OT-A
308	Yes	7	7-A	1000	exp	Inf	last	wholeT_0.01	OT-A
309	Yes	7	7-A	1000	exp	Inf	last	wholeT_0.5	OT-A
310	Yes	7	7-A	1000	exp	Inf	unif	singleC	CBN, CBN-A, OT
311	Yes	7	7-A	1000	exp	Inf	unif	wholeT_0.01	CBN, OT, OT-A
312	Yes	7	7-A	1000	exp	Inf	unif	wholeT_0.5	CBN, CBN-A
313	Yes	7	7-A	1000	McF_4	0	last	singleC	OT-A
314	Yes	7	7-A	1000	McF_4	0	last	wholeT_0.01	OT-A
315	Yes	7	7-A	1000	McF_4	0	last	wholeT_0.5	OT-A
316	Yes	7	7-A	1000	McF_4	0	unif	singleC	OT, OT-A
317	Yes	7	7-A	1000	McF_4	0	unif	wholeT_0.01	DiP-A, OT, OT-A

Table 9: *(continued)*

	Conjunction	Drivers	Tree	S.Size	Model	sh	S.Time	S.Type	Best method(s)
318	Yes	7	7-A	1000	McF_4	0	unif	wholeT_0.5	OT, OT-A
319	Yes	7	7-A	1000	McF_4	Inf	last	singleC	OT-A
320	Yes	7	7-A	1000	McF_4	Inf	last	wholeT_0.01	OT-A
321	Yes	7	7-A	1000	McF_4	Inf	last	wholeT_0.5	OT-A
322	Yes	7	7-A	1000	McF_4	Inf	unif	singleC	OT, OT-A
323	Yes	7	7-A	1000	McF_4	Inf	unif	wholeT_0.01	OT, OT-A
324	Yes	7	7-A	1000	McF_4	Inf	unif	wholeT_0.5	OT, OT-A
325	Yes	7	7-A	1000	McF_6	0	last	singleC	OT-A
326	Yes	7	7-A	1000	McF_6	0	last	wholeT_0.01	OT-A
327	Yes	7	7-A	1000	McF_6	0	last	wholeT_0.5	OT-A
328	Yes	7	7-A	1000	McF_6	0	unif	singleC	CBN-A, DiP-A
329	Yes	7	7-A	1000	McF_6	0	unif	wholeT_0.01	CBN-A, DiP, DiP-A
330	Yes	7	7-A	1000	McF_6	0	unif	wholeT_0.5	CBN-A
331	Yes	7	7-A	1000	McF_6	Inf	last	singleC	DiP-A, OT-A
332	Yes	7	7-A	1000	McF_6	Inf	last	wholeT_0.01	DiP-A, OT-A
333	Yes	7	7-A	1000	McF_6	Inf	last	wholeT_0.5	DiP-A, OT-A
334	Yes	7	7-A	1000	McF_6	Inf	unif	singleC	CBN-A
335	Yes	7	7-A	1000	McF_6	Inf	unif	wholeT_0.01	CBN-A
336	Yes	7	7-A	1000	McF_6	Inf	unif	wholeT_0.5	CBN-A
337	Yes	7	7-A	200	Bozic	0	last	singleC	OT-A
338	Yes	7	7-A	200	Bozic	0	last	wholeT_0.01	OT-A
339	Yes	7	7-A	200	Bozic	0	last	wholeT_0.5	OT-A
340	Yes	7	7-A	200	Bozic	0	unif	singleC	OT, OT-A
341	Yes	7	7-A	200	Bozic	0	unif	wholeT_0.01	CBN, CBN-A, OT-A
342	Yes	7	7-A	200	Bozic	0	unif	wholeT_0.5	OT, OT-A
343	Yes	7	7-A	200	Bozic	Inf	last	singleC	OT-A
344	Yes	7	7-A	200	Bozic	Inf	last	wholeT_0.01	OT-A
345	Yes	7	7-A	200	Bozic	Inf	last	wholeT_0.5	OT-A
346	Yes	7	7-A	200	Bozic	Inf	unif	singleC	CBN, CBN-A
347	Yes	7	7-A	200	Bozic	Inf	unif	wholeT_0.01	OT, OT-A
348	Yes	7	7-A	200	Bozic	Inf	unif	wholeT_0.5	CBN, CBN-A
349	Yes	7	7-A	200	exp	0	last	singleC	OT-A
350	Yes	7	7-A	200	exp	0	last	wholeT_0.01	OT-A
351	Yes	7	7-A	200	exp	0	last	wholeT_0.5	OT-A
352	Yes	7	7-A	200	exp	0	unif	singleC	OT, OT-A
353	Yes	7	7-A	200	exp	0	unif	wholeT_0.01	CBN, CBN-A, OT, OT-A
354	Yes	7	7-A	200	exp	0	unif	wholeT_0.5	OT, OT-A
355	Yes	7	7-A	200	exp	Inf	last	singleC	OT-A
356	Yes	7	7-A	200	exp	Inf	last	wholeT_0.01	OT-A
357	Yes	7	7-A	200	exp	Inf	last	wholeT_0.5	OT-A

Table 9: *(continued)*

	Conjunction	Drivers	Tree	S.Size	Model	sh	S.Time	S.Type	Best method(s)
358	Yes	7	7-A	200	exp	Inf	unif	singleC	CBN, CBN-A, OT, OT-A
359	Yes	7	7-A	200	exp	Inf	unif	wholeT_0.01	OT, OT-A
360	Yes	7	7-A	200	exp	Inf	unif	wholeT_0.5	CBN, CBN-A, OT, OT-A
361	Yes	7	7-A	200	McF_4	0	last	singleC	OT-A
362	Yes	7	7-A	200	McF_4	0	last	wholeT_0.01	OT-A
363	Yes	7	7-A	200	McF_4	0	last	wholeT_0.5	OT-A
364	Yes	7	7-A	200	McF_4	0	unif	singleC	OT, OT-A
365	Yes	7	7-A	200	McF_4	0	unif	wholeT_0.01	OT, OT-A
366	Yes	7	7-A	200	McF_4	0	unif	wholeT_0.5	OT, OT-A
367	Yes	7	7-A	200	McF_4	Inf	last	singleC	OT-A
368	Yes	7	7-A	200	McF_4	Inf	last	wholeT_0.01	OT-A
369	Yes	7	7-A	200	McF_4	Inf	last	wholeT_0.5	OT-A
370	Yes	7	7-A	200	McF_4	Inf	unif	singleC	OT, OT-A
371	Yes	7	7-A	200	McF_4	Inf	unif	wholeT_0.01	OT, OT-A
372	Yes	7	7-A	200	McF_4	Inf	unif	wholeT_0.5	OT, OT-A
373	Yes	7	7-A	200	McF_6	0	last	singleC	OT-A
374	Yes	7	7-A	200	McF_6	0	last	wholeT_0.01	OT-A
375	Yes	7	7-A	200	McF_6	0	last	wholeT_0.5	OT-A
376	Yes	7	7-A	200	McF_6	0	unif	singleC	CBN-A
377	Yes	7	7-A	200	McF_6	0	unif	wholeT_0.01	CBN-A, OT, OT-A
378	Yes	7	7-A	200	McF_6	0	unif	wholeT_0.5	CBN-A
379	Yes	7	7-A	200	McF_6	Inf	last	singleC	OT-A
380	Yes	7	7-A	200	McF_6	Inf	last	wholeT_0.01	OT-A
381	Yes	7	7-A	200	McF_6	Inf	last	wholeT_0.5	OT-A
382	Yes	7	7-A	200	McF_6	Inf	unif	singleC	CBN-A
383	Yes	7	7-A	200	McF_6	Inf	unif	wholeT_0.01	CBN-A
384	Yes	7	7-A	200	McF_6	Inf	unif	wholeT_0.5	CBN-A
385	Yes	7	7-A	100	Bozic	0	last	singleC	OT-A
386	Yes	7	7-A	100	Bozic	0	last	wholeT_0.01	OT-A
387	Yes	7	7-A	100	Bozic	0	last	wholeT_0.5	OT-A
388	Yes	7	7-A	100	Bozic	0	unif	singleC	OT, OT-A
389	Yes	7	7-A	100	Bozic	0	unif	wholeT_0.01	CBN, CBN-A, OT, OT-A
390	Yes	7	7-A	100	Bozic	0	unif	wholeT_0.5	OT, OT-A
391	Yes	7	7-A	100	Bozic	Inf	last	singleC	OT-A
392	Yes	7	7-A	100	Bozic	Inf	last	wholeT_0.01	OT-A
393	Yes	7	7-A	100	Bozic	Inf	last	wholeT_0.5	OT-A
394	Yes	7	7-A	100	Bozic	Inf	unif	singleC	CBN, CBN-A
395	Yes	7	7-A	100	Bozic	Inf	unif	wholeT_0.01	CBN, CBN-A, OT, OT-A
396	Yes	7	7-A	100	Bozic	Inf	unif	wholeT_0.5	CBN, CBN-A
397	Yes	7	7-A	100	exp	0	last	singleC	OT-A

Table 9: *(continued)*

	Conjunction	Drivers	Tree	S.Size	Model	sh	S.Time	S.Type	Best method(s)
398	Yes	7	7-A	100	exp	0	last	wholeT_0.01	OT-A
399	Yes	7	7-A	100	exp	0	last	wholeT_0.5	OT-A
400	Yes	7	7-A	100	exp	0	unif	singleC	OT, OT-A
401	Yes	7	7-A	100	exp	0	unif	wholeT_0.01	CBN, OT, OT-A
402	Yes	7	7-A	100	exp	0	unif	wholeT_0.5	OT, OT-A
403	Yes	7	7-A	100	exp	Inf	last	singleC	OT-A
404	Yes	7	7-A	100	exp	Inf	last	wholeT_0.01	OT-A
405	Yes	7	7-A	100	exp	Inf	last	wholeT_0.5	OT-A
406	Yes	7	7-A	100	exp	Inf	unif	singleC	CBN-A, OT, OT-A
407	Yes	7	7-A	100	exp	Inf	unif	wholeT_0.01	OT, OT-A
408	Yes	7	7-A	100	exp	Inf	unif	wholeT_0.5	CBN, OT, OT-A
409	Yes	7	7-A	100	McF_4	0	last	singleC	OT-A
410	Yes	7	7-A	100	McF_4	0	last	wholeT_0.01	OT-A
411	Yes	7	7-A	100	McF_4	0	last	wholeT_0.5	OT-A
412	Yes	7	7-A	100	McF_4	0	unif	singleC	OT, OT-A
413	Yes	7	7-A	100	McF_4	0	unif	wholeT_0.01	OT, OT-A
414	Yes	7	7-A	100	McF_4	0	unif	wholeT_0.5	OT, OT-A
415	Yes	7	7-A	100	McF_4	Inf	last	singleC	OT-A
416	Yes	7	7-A	100	McF_4	Inf	last	wholeT_0.01	OT-A
417	Yes	7	7-A	100	McF_4	Inf	last	wholeT_0.5	OT-A
418	Yes	7	7-A	100	McF_4	Inf	unif	singleC	OT, OT-A
419	Yes	7	7-A	100	McF_4	Inf	unif	wholeT_0.01	OT-A
420	Yes	7	7-A	100	McF_4	Inf	unif	wholeT_0.5	OT, OT-A
421	Yes	7	7-A	100	McF_6	0	last	singleC	OT-A
422	Yes	7	7-A	100	McF_6	0	last	wholeT_0.01	OT-A
423	Yes	7	7-A	100	McF_6	0	last	wholeT_0.5	OT-A
424	Yes	7	7-A	100	McF_6	0	unif	singleC	CBN, CBN-A
425	Yes	7	7-A	100	McF_6	0	unif	wholeT_0.01	CBN, CBN-A, OT, OT-A
426	Yes	7	7-A	100	McF_6	0	unif	wholeT_0.5	CBN-A
427	Yes	7	7-A	100	McF_6	Inf	last	singleC	OT-A
428	Yes	7	7-A	100	McF_6	Inf	last	wholeT_0.01	OT-A
429	Yes	7	7-A	100	McF_6	Inf	last	wholeT_0.5	OT-A
430	Yes	7	7-A	100	McF_6	Inf	unif	singleC	CBN-A
431	Yes	7	7-A	100	McF_6	Inf	unif	wholeT_0.01	CBN-A
432	Yes	7	7-A	100	McF_6	Inf	unif	wholeT_0.5	CBN-A
433	No	11	11-B	1000	Bozic	0	last	singleC	OT, OT-A
434	No	11	11-B	1000	Bozic	0	last	wholeT_0.01	OT, OT-A
435	No	11	11-B	1000	Bozic	0	last	wholeT_0.5	OT, OT-A
436	No	11	11-B	1000	Bozic	0	unif	singleC	OT, OT-A
437	No	11	11-B	1000	Bozic	0	unif	wholeT_0.01	OT, OT-A

Table 9: *(continued)*

	Conjunction	Drivers	Tree	S.Size	Model	sh	S.Time	S.Type	Best method(s)
438	No	11	11-B	1000	Bozic	0	unif	wholeT_0.5	OT, OT-A
439	No	11	11-B	1000	Bozic	Inf	last	singleC	OT, OT-A
440	No	11	11-B	1000	Bozic	Inf	last	wholeT_0.01	DiP-A
441	No	11	11-B	1000	Bozic	Inf	last	wholeT_0.5	OT, OT-A
442	No	11	11-B	1000	Bozic	Inf	unif	singleC	OT, OT-A
443	No	11	11-B	1000	Bozic	Inf	unif	wholeT_0.01	OT, OT-A
444	No	11	11-B	1000	Bozic	Inf	unif	wholeT_0.5	OT, OT-A
445	No	11	11-B	1000	exp	0	last	singleC	OT, OT-A
446	No	11	11-B	1000	exp	0	last	wholeT_0.01	OT, OT-A
447	No	11	11-B	1000	exp	0	last	wholeT_0.5	OT, OT-A
448	No	11	11-B	1000	exp	0	unif	singleC	OT, OT-A
449	No	11	11-B	1000	exp	0	unif	wholeT_0.01	OT, OT-A
450	No	11	11-B	1000	exp	0	unif	wholeT_0.5	OT, OT-A
451	No	11	11-B	1000	exp	Inf	last	singleC	OT, OT-A
452	No	11	11-B	1000	exp	Inf	last	wholeT_0.01	OT, OT-A
453	No	11	11-B	1000	exp	Inf	last	wholeT_0.5	OT, OT-A
454	No	11	11-B	1000	exp	Inf	unif	singleC	OT, OT-A
455	No	11	11-B	1000	exp	Inf	unif	wholeT_0.01	OT, OT-A
456	No	11	11-B	1000	exp	Inf	unif	wholeT_0.5	OT, OT-A
457	No	11	11-B	1000	McF_4	0	last	singleC	OT, OT-A
458	No	11	11-B	1000	McF_4	0	last	wholeT_0.01	DiP, DiP-A, OT, OT-A
459	No	11	11-B	1000	McF_4	0	last	wholeT_0.5	OT, OT-A
460	No	11	11-B	1000	McF_4	0	unif	singleC	OT, OT-A
461	No	11	11-B	1000	McF_4	0	unif	wholeT_0.01	OT, OT-A
462	No	11	11-B	1000	McF_4	0	unif	wholeT_0.5	OT, OT-A
463	No	11	11-B	1000	McF_4	Inf	last	singleC	OT, OT-A
464	No	11	11-B	1000	McF_4	Inf	last	wholeT_0.01	OT, OT-A
465	No	11	11-B	1000	McF_4	Inf	last	wholeT_0.5	OT, OT-A
466	No	11	11-B	1000	McF_4	Inf	unif	singleC	OT, OT-A
467	No	11	11-B	1000	McF_4	Inf	unif	wholeT_0.01	OT, OT-A
468	No	11	11-B	1000	McF_4	Inf	unif	wholeT_0.5	OT, OT-A
469	No	11	11-B	1000	McF_6	0	last	singleC	DiP, OT
470	No	11	11-B	1000	McF_6	0	last	wholeT_0.01	DiP, OT
471	No	11	11-B	1000	McF_6	0	last	wholeT_0.5	DiP, OT
472	No	11	11-B	1000	McF_6	0	unif	singleC	DiP, DiP-A, OT, OT-A
473	No	11	11-B	1000	McF_6	0	unif	wholeT_0.01	DiP, DiP-A, OT
474	No	11	11-B	1000	McF_6	0	unif	wholeT_0.5	DiP, DiP-A
475	No	11	11-B	1000	McF_6	Inf	last	singleC	DiP, OT
476	No	11	11-B	1000	McF_6	Inf	last	wholeT_0.01	OT
477	No	11	11-B	1000	McF_6	Inf	last	wholeT_0.5	DiP, OT

Table 9: *(continued)*

	Conjunction	Drivers	Tree	S.Size	Model	sh	S.Time	S.Type	Best method(s)
478	No	11	11-B	1000	McF_6	Inf	unif	singleC	DiP, DiP-A
479	No	11	11-B	1000	McF_6	Inf	unif	wholeT_0.01	DiP, DiP-A
480	No	11	11-B	1000	McF_6	Inf	unif	wholeT_0.5	DiP, DiP-A, OT-A
481	No	11	11-B	200	Bozic	0	last	singleC	OT, OT-A
482	No	11	11-B	200	Bozic	0	last	wholeT_0.01	OT, OT-A
483	No	11	11-B	200	Bozic	0	last	wholeT_0.5	OT, OT-A
484	No	11	11-B	200	Bozic	0	unif	singleC	OT, OT-A
485	No	11	11-B	200	Bozic	0	unif	wholeT_0.01	OT, OT-A
486	No	11	11-B	200	Bozic	0	unif	wholeT_0.5	OT, OT-A
487	No	11	11-B	200	Bozic	Inf	last	singleC	OT, OT-A
488	No	11	11-B	200	Bozic	Inf	last	wholeT_0.01	OT, OT-A
489	No	11	11-B	200	Bozic	Inf	last	wholeT_0.5	OT, OT-A
490	No	11	11-B	200	Bozic	Inf	unif	singleC	OT, OT-A
491	No	11	11-B	200	Bozic	Inf	unif	wholeT_0.01	OT, OT-A
492	No	11	11-B	200	Bozic	Inf	unif	wholeT_0.5	OT, OT-A
493	No	11	11-B	200	exp	0	last	singleC	OT, OT-A
494	No	11	11-B	200	exp	0	last	wholeT_0.01	OT, OT-A
495	No	11	11-B	200	exp	0	last	wholeT_0.5	OT, OT-A
496	No	11	11-B	200	exp	0	unif	singleC	OT, OT-A
497	No	11	11-B	200	exp	0	unif	wholeT_0.01	OT, OT-A
498	No	11	11-B	200	exp	0	unif	wholeT_0.5	OT, OT-A
499	No	11	11-B	200	exp	Inf	last	singleC	OT, OT-A
500	No	11	11-B	200	exp	Inf	last	wholeT_0.01	OT, OT-A
501	No	11	11-B	200	exp	Inf	last	wholeT_0.5	OT, OT-A
502	No	11	11-B	200	exp	Inf	unif	singleC	OT, OT-A
503	No	11	11-B	200	exp	Inf	unif	wholeT_0.01	OT
504	No	11	11-B	200	exp	Inf	unif	wholeT_0.5	OT, OT-A
505	No	11	11-B	200	McF_4	0	last	singleC	OT, OT-A
506	No	11	11-B	200	McF_4	0	last	wholeT_0.01	OT, OT-A
507	No	11	11-B	200	McF_4	0	last	wholeT_0.5	OT, OT-A
508	No	11	11-B	200	McF_4	0	unif	singleC	OT, OT-A
509	No	11	11-B	200	McF_4	0	unif	wholeT_0.01	OT, OT-A
510	No	11	11-B	200	McF_4	0	unif	wholeT_0.5	OT, OT-A
511	No	11	11-B	200	McF_4	Inf	last	singleC	OT, OT-A
512	No	11	11-B	200	McF_4	Inf	last	wholeT_0.01	OT, OT-A
513	No	11	11-B	200	McF_4	Inf	last	wholeT_0.5	OT, OT-A
514	No	11	11-B	200	McF_4	Inf	unif	singleC	OT, OT-A
515	No	11	11-B	200	McF_4	Inf	unif	wholeT_0.01	OT, OT-A
516	No	11	11-B	200	McF_4	Inf	unif	wholeT_0.5	OT, OT-A
517	No	11	11-B	200	McF_6	0	last	singleC	OT

Table 9: *(continued)*

	Conjunction	Drivers	Tree	S.Size	Model	sh	S.Time	S.Type	Best method(s)
518	No	11	11-B	200	McF_6	0	last	wholeT_0.01	OT
519	No	11	11-B	200	McF_6	0	last	wholeT_0.5	OT
520	No	11	11-B	200	McF_6	0	unif	singleC	OT, OT-A
521	No	11	11-B	200	McF_6	0	unif	wholeT_0.01	OT, OT-A
522	No	11	11-B	200	McF_6	0	unif	wholeT_0.5	OT, OT-A
523	No	11	11-B	200	McF_6	Inf	last	singleC	OT
524	No	11	11-B	200	McF_6	Inf	last	wholeT_0.01	OT
525	No	11	11-B	200	McF_6	Inf	last	wholeT_0.5	OT
526	No	11	11-B	200	McF_6	Inf	unif	singleC	OT, OT-A
527	No	11	11-B	200	McF_6	Inf	unif	wholeT_0.01	OT, OT-A
528	No	11	11-B	200	McF_6	Inf	unif	wholeT_0.5	OT, OT-A
529	No	11	11-B	100	Bozic	0	last	singleC	OT, OT-A
530	No	11	11-B	100	Bozic	0	last	wholeT_0.01	OT, OT-A
531	No	11	11-B	100	Bozic	0	last	wholeT_0.5	OT, OT-A
532	No	11	11-B	100	Bozic	0	unif	singleC	OT, OT-A
533	No	11	11-B	100	Bozic	0	unif	wholeT_0.01	OT, OT-A
534	No	11	11-B	100	Bozic	0	unif	wholeT_0.5	OT, OT-A
535	No	11	11-B	100	Bozic	Inf	last	singleC	OT, OT-A
536	No	11	11-B	100	Bozic	Inf	last	wholeT_0.01	OT, OT-A
537	No	11	11-B	100	Bozic	Inf	last	wholeT_0.5	OT, OT-A
538	No	11	11-B	100	Bozic	Inf	unif	singleC	OT, OT-A
539	No	11	11-B	100	Bozic	Inf	unif	wholeT_0.01	OT, OT-A
540	No	11	11-B	100	Bozic	Inf	unif	wholeT_0.5	OT, OT-A
541	No	11	11-B	100	exp	0	last	singleC	OT, OT-A
542	No	11	11-B	100	exp	0	last	wholeT_0.01	OT, OT-A
543	No	11	11-B	100	exp	0	last	wholeT_0.5	OT, OT-A
544	No	11	11-B	100	exp	0	unif	singleC	OT, OT-A
545	No	11	11-B	100	exp	0	unif	wholeT_0.01	OT, OT-A
546	No	11	11-B	100	exp	0	unif	wholeT_0.5	OT, OT-A
547	No	11	11-B	100	exp	Inf	last	singleC	OT, OT-A
548	No	11	11-B	100	exp	Inf	last	wholeT_0.01	OT, OT-A
549	No	11	11-B	100	exp	Inf	last	wholeT_0.5	OT, OT-A
550	No	11	11-B	100	exp	Inf	unif	singleC	OT, OT-A
551	No	11	11-B	100	exp	Inf	unif	wholeT_0.01	OT, OT-A
552	No	11	11-B	100	exp	Inf	unif	wholeT_0.5	OT, OT-A
553	No	11	11-B	100	McF_4	0	last	singleC	OT, OT-A
554	No	11	11-B	100	McF_4	0	last	wholeT_0.01	OT, OT-A
555	No	11	11-B	100	McF_4	0	last	wholeT_0.5	OT, OT-A
556	No	11	11-B	100	McF_4	0	unif	singleC	OT, OT-A
557	No	11	11-B	100	McF_4	0	unif	wholeT_0.01	OT, OT-A

Table 9: *(continued)*

	Conjunction	Drivers	Tree	S.Size	Model	sh	S.Time	S.Type	Best method(s)
558	No	11	11-B	100	McF_4	0	unif	wholeT_0.5	OT, OT-A
559	No	11	11-B	100	McF_4	Inf	last	singleC	OT, OT-A
560	No	11	11-B	100	McF_4	Inf	last	wholeT_0.01	OT, OT-A
561	No	11	11-B	100	McF_4	Inf	last	wholeT_0.5	OT, OT-A
562	No	11	11-B	100	McF_4	Inf	unif	singleC	OT, OT-A
563	No	11	11-B	100	McF_4	Inf	unif	wholeT_0.01	OT, OT-A
564	No	11	11-B	100	McF_4	Inf	unif	wholeT_0.5	OT, OT-A
565	No	11	11-B	100	McF_6	0	last	singleC	OT
566	No	11	11-B	100	McF_6	0	last	wholeT_0.01	OT
567	No	11	11-B	100	McF_6	0	last	wholeT_0.5	OT
568	No	11	11-B	100	McF_6	0	unif	singleC	OT, OT-A
569	No	11	11-B	100	McF_6	0	unif	wholeT_0.01	OT, OT-A
570	No	11	11-B	100	McF_6	0	unif	wholeT_0.5	OT, OT-A
571	No	11	11-B	100	McF_6	Inf	last	singleC	OT
572	No	11	11-B	100	McF_6	Inf	last	wholeT_0.01	OT, OT-A
573	No	11	11-B	100	McF_6	Inf	last	wholeT_0.5	OT
574	No	11	11-B	100	McF_6	Inf	unif	singleC	OT, OT-A
575	No	11	11-B	100	McF_6	Inf	unif	wholeT_0.01	OT, OT-A
576	No	11	11-B	100	McF_6	Inf	unif	wholeT_0.5	OT, OT-A
577	No	9	9-B	1000	Bozic	0	last	singleC	OT, OT-A
578	No	9	9-B	1000	Bozic	0	last	wholeT_0.01	OT, OT-A
579	No	9	9-B	1000	Bozic	0	last	wholeT_0.5	OT-A
580	No	9	9-B	1000	Bozic	0	unif	singleC	OT, OT-A
581	No	9	9-B	1000	Bozic	0	unif	wholeT_0.01	OT, OT-A
582	No	9	9-B	1000	Bozic	0	unif	wholeT_0.5	OT, OT-A
583	No	9	9-B	1000	Bozic	Inf	last	singleC	OT, OT-A
584	No	9	9-B	1000	Bozic	Inf	last	wholeT_0.01	DiP, DiP-A
585	No	9	9-B	1000	Bozic	Inf	last	wholeT_0.5	OT, OT-A
586	No	9	9-B	1000	Bozic	Inf	unif	singleC	OT, OT-A
587	No	9	9-B	1000	Bozic	Inf	unif	wholeT_0.01	OT, OT-A
588	No	9	9-B	1000	Bozic	Inf	unif	wholeT_0.5	OT, OT-A
589	No	9	9-B	1000	exp	0	last	singleC	OT, OT-A
590	No	9	9-B	1000	exp	0	last	wholeT_0.01	OT, OT-A
591	No	9	9-B	1000	exp	0	last	wholeT_0.5	OT, OT-A
592	No	9	9-B	1000	exp	0	unif	singleC	OT
593	No	9	9-B	1000	exp	0	unif	wholeT_0.01	OT, OT-A
594	No	9	9-B	1000	exp	0	unif	wholeT_0.5	OT, OT-A
595	No	9	9-B	1000	exp	Inf	last	singleC	OT, OT-A
596	No	9	9-B	1000	exp	Inf	last	wholeT_0.01	CBN-A, DiP-A, OT, OT-A
597	No	9	9-B	1000	exp	Inf	last	wholeT_0.5	OT, OT-A

Table 9: *(continued)*

	Conjunction	Drivers	Tree	S.Size	Model	sh	S.Time	S.Type	Best method(s)
598	No	9	9-B	1000	exp	Inf	unif	singleC	OT, OT-A
599	No	9	9-B	1000	exp	Inf	unif	wholeT_0.01	OT, OT-A
600	No	9	9-B	1000	exp	Inf	unif	wholeT_0.5	OT, OT-A
601	No	9	9-B	1000	McF_4	0	last	singleC	OT, OT-A
602	No	9	9-B	1000	McF_4	0	last	wholeT_0.01	DiP, DiP-A, OT, OT-A
603	No	9	9-B	1000	McF_4	0	last	wholeT_0.5	OT, OT-A
604	No	9	9-B	1000	McF_4	0	unif	singleC	OT, OT-A
605	No	9	9-B	1000	McF_4	0	unif	wholeT_0.01	OT, OT-A
606	No	9	9-B	1000	McF_4	0	unif	wholeT_0.5	OT, OT-A
607	No	9	9-B	1000	McF_4	Inf	last	singleC	OT, OT-A
608	No	9	9-B	1000	McF_4	Inf	last	wholeT_0.01	DiP, DiP-A, OT, OT-A
609	No	9	9-B	1000	McF_4	Inf	last	wholeT_0.5	OT, OT-A
610	No	9	9-B	1000	McF_4	Inf	unif	singleC	OT-A
611	No	9	9-B	1000	McF_4	Inf	unif	wholeT_0.01	OT, OT-A
612	No	9	9-B	1000	McF_4	Inf	unif	wholeT_0.5	OT, OT-A
613	No	9	9-B	1000	McF_6	0	last	singleC	OT
614	No	9	9-B	1000	McF_6	0	last	wholeT_0.01	OT
615	No	9	9-B	1000	McF_6	0	last	wholeT_0.5	OT
616	No	9	9-B	1000	McF_6	0	unif	singleC	DiP, DiP-A, OT, OT-A
617	No	9	9-B	1000	McF_6	0	unif	wholeT_0.01	DiP, DiP-A, OT, OT-A
618	No	9	9-B	1000	McF_6	0	unif	wholeT_0.5	DiP, DiP-A, OT, OT-A
619	No	9	9-B	1000	McF_6	Inf	last	singleC	DiP, DiP-A, OT-A
620	No	9	9-B	1000	McF_6	Inf	last	wholeT_0.01	DiP, DiP-A, OT-A
621	No	9	9-B	1000	McF_6	Inf	last	wholeT_0.5	DiP, DiP-A, OT-A
622	No	9	9-B	1000	McF_6	Inf	unif	singleC	DiP, DiP-A, OT, OT-A
623	No	9	9-B	1000	McF_6	Inf	unif	wholeT_0.01	DiP, DiP-A, OT, OT-A
624	No	9	9-B	1000	McF_6	Inf	unif	wholeT_0.5	DiP, DiP-A, OT, OT-A
625	No	9	9-B	200	Bozic	0	last	singleC	OT, OT-A
626	No	9	9-B	200	Bozic	0	last	wholeT_0.01	OT, OT-A
627	No	9	9-B	200	Bozic	0	last	wholeT_0.5	OT, OT-A
628	No	9	9-B	200	Bozic	0	unif	singleC	OT, OT-A
629	No	9	9-B	200	Bozic	0	unif	wholeT_0.01	OT, OT-A
630	No	9	9-B	200	Bozic	0	unif	wholeT_0.5	OT, OT-A
631	No	9	9-B	200	Bozic	Inf	last	singleC	OT, OT-A
632	No	9	9-B	200	Bozic	Inf	last	wholeT_0.01	OT, OT-A
633	No	9	9-B	200	Bozic	Inf	last	wholeT_0.5	OT, OT-A
634	No	9	9-B	200	Bozic	Inf	unif	singleC	OT, OT-A
635	No	9	9-B	200	Bozic	Inf	unif	wholeT_0.01	OT, OT-A
636	No	9	9-B	200	Bozic	Inf	unif	wholeT_0.5	OT, OT-A
637	No	9	9-B	200	exp	0	last	singleC	OT, OT-A

Table 9: *(continued)*

	Conjunction	Drivers	Tree	S.Size	Model	sh	S.Time	S.Type	Best method(s)
638	No	9	9-B	200	exp	0	last	wholeT_0.01	OT, OT-A
639	No	9	9-B	200	exp	0	last	wholeT_0.5	OT, OT-A
640	No	9	9-B	200	exp	0	unif	singleC	OT, OT-A
641	No	9	9-B	200	exp	0	unif	wholeT_0.01	OT, OT-A
642	No	9	9-B	200	exp	0	unif	wholeT_0.5	OT, OT-A
643	No	9	9-B	200	exp	Inf	last	singleC	OT, OT-A
644	No	9	9-B	200	exp	Inf	last	wholeT_0.01	OT, OT-A
645	No	9	9-B	200	exp	Inf	last	wholeT_0.5	OT, OT-A
646	No	9	9-B	200	exp	Inf	unif	singleC	OT, OT-A
647	No	9	9-B	200	exp	Inf	unif	wholeT_0.01	OT, OT-A
648	No	9	9-B	200	exp	Inf	unif	wholeT_0.5	OT, OT-A
649	No	9	9-B	200	McF_4	0	last	singleC	OT, OT-A
650	No	9	9-B	200	McF_4	0	last	wholeT_0.01	OT, OT-A
651	No	9	9-B	200	McF_4	0	last	wholeT_0.5	OT, OT-A
652	No	9	9-B	200	McF_4	0	unif	singleC	OT, OT-A
653	No	9	9-B	200	McF_4	0	unif	wholeT_0.01	OT, OT-A
654	No	9	9-B	200	McF_4	0	unif	wholeT_0.5	OT, OT-A
655	No	9	9-B	200	McF_4	Inf	last	singleC	OT, OT-A
656	No	9	9-B	200	McF_4	Inf	last	wholeT_0.01	OT, OT-A
657	No	9	9-B	200	McF_4	Inf	last	wholeT_0.5	OT, OT-A
658	No	9	9-B	200	McF_4	Inf	unif	singleC	OT, OT-A
659	No	9	9-B	200	McF_4	Inf	unif	wholeT_0.01	OT, OT-A
660	No	9	9-B	200	McF_4	Inf	unif	wholeT_0.5	OT, OT-A
661	No	9	9-B	200	McF_6	0	last	singleC	OT, OT-A
662	No	9	9-B	200	McF_6	0	last	wholeT_0.01	OT, OT-A
663	No	9	9-B	200	McF_6	0	last	wholeT_0.5	OT, OT-A
664	No	9	9-B	200	McF_6	0	unif	singleC	OT, OT-A
665	No	9	9-B	200	McF_6	0	unif	wholeT_0.01	OT, OT-A
666	No	9	9-B	200	McF_6	0	unif	wholeT_0.5	OT, OT-A
667	No	9	9-B	200	McF_6	Inf	last	singleC	OT-A
668	No	9	9-B	200	McF_6	Inf	last	wholeT_0.01	OT-A
669	No	9	9-B	200	McF_6	Inf	last	wholeT_0.5	OT-A
670	No	9	9-B	200	McF_6	Inf	unif	singleC	OT, OT-A
671	No	9	9-B	200	McF_6	Inf	unif	wholeT_0.01	OT, OT-A
672	No	9	9-B	200	McF_6	Inf	unif	wholeT_0.5	OT, OT-A
673	No	9	9-B	100	Bozic	0	last	singleC	OT, OT-A
674	No	9	9-B	100	Bozic	0	last	wholeT_0.01	OT, OT-A
675	No	9	9-B	100	Bozic	0	last	wholeT_0.5	OT, OT-A
676	No	9	9-B	100	Bozic	0	unif	singleC	OT, OT-A
677	No	9	9-B	100	Bozic	0	unif	wholeT_0.01	OT, OT-A

Table 9: *(continued)*

	Conjunction	Drivers	Tree	S.Size	Model	sh	S.Time	S.Type	Best method(s)
678	No	9	9-B	100	Bozic	0	unif	wholeT_0.5	OT, OT-A
679	No	9	9-B	100	Bozic	Inf	last	singleC	OT, OT-A
680	No	9	9-B	100	Bozic	Inf	last	wholeT_0.01	OT, OT-A
681	No	9	9-B	100	Bozic	Inf	last	wholeT_0.5	OT, OT-A
682	No	9	9-B	100	Bozic	Inf	unif	singleC	OT, OT-A
683	No	9	9-B	100	Bozic	Inf	unif	wholeT_0.01	OT, OT-A
684	No	9	9-B	100	Bozic	Inf	unif	wholeT_0.5	OT, OT-A
685	No	9	9-B	100	exp	0	last	singleC	OT, OT-A
686	No	9	9-B	100	exp	0	last	wholeT_0.01	OT, OT-A
687	No	9	9-B	100	exp	0	last	wholeT_0.5	OT, OT-A
688	No	9	9-B	100	exp	0	unif	singleC	OT, OT-A
689	No	9	9-B	100	exp	0	unif	wholeT_0.01	OT, OT-A
690	No	9	9-B	100	exp	0	unif	wholeT_0.5	OT, OT-A
691	No	9	9-B	100	exp	Inf	last	singleC	OT, OT-A
692	No	9	9-B	100	exp	Inf	last	wholeT_0.01	OT, OT-A
693	No	9	9-B	100	exp	Inf	last	wholeT_0.5	OT, OT-A
694	No	9	9-B	100	exp	Inf	unif	singleC	OT, OT-A
695	No	9	9-B	100	exp	Inf	unif	wholeT_0.01	OT, OT-A
696	No	9	9-B	100	exp	Inf	unif	wholeT_0.5	OT, OT-A
697	No	9	9-B	100	McF_4	0	last	singleC	OT, OT-A
698	No	9	9-B	100	McF_4	0	last	wholeT_0.01	OT, OT-A
699	No	9	9-B	100	McF_4	0	last	wholeT_0.5	OT, OT-A
700	No	9	9-B	100	McF_4	0	unif	singleC	OT, OT-A
701	No	9	9-B	100	McF_4	0	unif	wholeT_0.01	OT, OT-A
702	No	9	9-B	100	McF_4	0	unif	wholeT_0.5	OT, OT-A
703	No	9	9-B	100	McF_4	Inf	last	singleC	OT, OT-A
704	No	9	9-B	100	McF_4	Inf	last	wholeT_0.01	OT
705	No	9	9-B	100	McF_4	Inf	last	wholeT_0.5	OT, OT-A
706	No	9	9-B	100	McF_4	Inf	unif	singleC	OT, OT-A
707	No	9	9-B	100	McF_4	Inf	unif	wholeT_0.01	OT, OT-A
708	No	9	9-B	100	McF_4	Inf	unif	wholeT_0.5	OT, OT-A
709	No	9	9-B	100	McF_6	0	last	singleC	OT, OT-A
710	No	9	9-B	100	McF_6	0	last	wholeT_0.01	OT, OT-A
711	No	9	9-B	100	McF_6	0	last	wholeT_0.5	OT, OT-A
712	No	9	9-B	100	McF_6	0	unif	singleC	OT, OT-A
713	No	9	9-B	100	McF_6	0	unif	wholeT_0.01	OT, OT-A
714	No	9	9-B	100	McF_6	0	unif	wholeT_0.5	OT, OT-A
715	No	9	9-B	100	McF_6	Inf	last	singleC	OT-A
716	No	9	9-B	100	McF_6	Inf	last	wholeT_0.01	OT-A
717	No	9	9-B	100	McF_6	Inf	last	wholeT_0.5	OT-A

Table 9: *(continued)*

	Conjunction	Drivers	Tree	S.Size	Model	sh	S.Time	S.Type	Best method(s)
718	No	9	9-B	100	McF_6	Inf	unif	singleC	OT, OT-A
719	No	9	9-B	100	McF_6	Inf	unif	wholeT_0.01	OT, OT-A
720	No	9	9-B	100	McF_6	Inf	unif	wholeT_0.5	OT, OT-A
721	No	7	7-B	1000	Bozic	0	last	singleC	OT-A
722	No	7	7-B	1000	Bozic	0	last	wholeT_0.01	OT-A
723	No	7	7-B	1000	Bozic	0	last	wholeT_0.5	OT-A
724	No	7	7-B	1000	Bozic	0	unif	singleC	OT, OT-A
725	No	7	7-B	1000	Bozic	0	unif	wholeT_0.01	CBN-A, OT, OT-A
726	No	7	7-B	1000	Bozic	0	unif	wholeT_0.5	OT
727	No	7	7-B	1000	Bozic	Inf	last	singleC	DiP-A, OT-A
728	No	7	7-B	1000	Bozic	Inf	last	wholeT_0.01	DiP-A, OT-A
729	No	7	7-B	1000	Bozic	Inf	last	wholeT_0.5	DiP-A, OT-A
730	No	7	7-B	1000	Bozic	Inf	unif	singleC	CBN, CBN-A, OT, OT-A
731	No	7	7-B	1000	Bozic	Inf	unif	wholeT_0.01	OT, OT-A
732	No	7	7-B	1000	Bozic	Inf	unif	wholeT_0.5	CBN, CBN-A
733	No	7	7-B	1000	exp	0	last	singleC	OT-A
734	No	7	7-B	1000	exp	0	last	wholeT_0.01	OT-A
735	No	7	7-B	1000	exp	0	last	wholeT_0.5	OT-A
736	No	7	7-B	1000	exp	0	unif	singleC	OT, OT-A
737	No	7	7-B	1000	exp	0	unif	wholeT_0.01	CBN, CBN-A, OT, OT-A
738	No	7	7-B	1000	exp	0	unif	wholeT_0.5	OT, OT-A
739	No	7	7-B	1000	exp	Inf	last	singleC	OT-A
740	No	7	7-B	1000	exp	Inf	last	wholeT_0.01	OT-A
741	No	7	7-B	1000	exp	Inf	last	wholeT_0.5	OT-A
742	No	7	7-B	1000	exp	Inf	unif	singleC	CBN, OT, OT-A
743	No	7	7-B	1000	exp	Inf	unif	wholeT_0.01	OT, OT-A
744	No	7	7-B	1000	exp	Inf	unif	wholeT_0.5	OT, OT-A
745	No	7	7-B	1000	McF_4	0	last	singleC	DiP-A, OT-A
746	No	7	7-B	1000	McF_4	0	last	wholeT_0.01	DiP-A, OT-A
747	No	7	7-B	1000	McF_4	0	last	wholeT_0.5	DiP-A, OT-A
748	No	7	7-B	1000	McF_4	0	unif	singleC	DiP-A, OT, OT-A
749	No	7	7-B	1000	McF_4	0	unif	wholeT_0.01	OT, OT-A
750	No	7	7-B	1000	McF_4	0	unif	wholeT_0.5	DiP-A, OT, OT-A
751	No	7	7-B	1000	McF_4	Inf	last	singleC	DiP-A, OT-A
752	No	7	7-B	1000	McF_4	Inf	last	wholeT_0.01	DiP-A, OT-A
753	No	7	7-B	1000	McF_4	Inf	last	wholeT_0.5	DiP-A, OT-A
754	No	7	7-B	1000	McF_4	Inf	unif	singleC	OT, OT-A
755	No	7	7-B	1000	McF_4	Inf	unif	wholeT_0.01	OT, OT-A
756	No	7	7-B	1000	McF_4	Inf	unif	wholeT_0.5	OT, OT-A
757	No	7	7-B	1000	McF_6	0	last	singleC	DiP-A, OT-A

Table 9: *(continued)*

	Conjunction	Drivers	Tree	S.Size	Model	sh	S.Time	S.Type	Best method(s)
758	No	7	7-B	1000	McF_6	0	last	wholeT_0.01	DiP-A, OT-A
759	No	7	7-B	1000	McF_6	0	last	wholeT_0.5	DiP-A, OT-A
760	No	7	7-B	1000	McF_6	0	unif	singleC	DiP, DiP-A, OT, OT-A
761	No	7	7-B	1000	McF_6	0	unif	wholeT_0.01	DiP, DiP-A, OT, OT-A
762	No	7	7-B	1000	McF_6	0	unif	wholeT_0.5	DiP, DiP-A, OT, OT-A
763	No	7	7-B	1000	McF_6	Inf	last	singleC	DiP-A, OT-A
764	No	7	7-B	1000	McF_6	Inf	last	wholeT_0.01	DiP-A, OT-A
765	No	7	7-B	1000	McF_6	Inf	last	wholeT_0.5	DiP-A, OT-A
766	No	7	7-B	1000	McF_6	Inf	unif	singleC	DiP-A, OT, OT-A
767	No	7	7-B	1000	McF_6	Inf	unif	wholeT_0.01	DiP-A, OT, OT-A
768	No	7	7-B	1000	McF_6	Inf	unif	wholeT_0.5	DiP-A, OT, OT-A
769	No	7	7-B	200	Bozic	0	last	singleC	OT-A
770	No	7	7-B	200	Bozic	0	last	wholeT_0.01	OT-A
771	No	7	7-B	200	Bozic	0	last	wholeT_0.5	OT-A
772	No	7	7-B	200	Bozic	0	unif	singleC	OT, OT-A
773	No	7	7-B	200	Bozic	0	unif	wholeT_0.01	CBN, CBN-A, OT, OT-A
774	No	7	7-B	200	Bozic	0	unif	wholeT_0.5	OT, OT-A
775	No	7	7-B	200	Bozic	Inf	last	singleC	OT-A
776	No	7	7-B	200	Bozic	Inf	last	wholeT_0.01	OT-A
777	No	7	7-B	200	Bozic	Inf	last	wholeT_0.5	OT-A
778	No	7	7-B	200	Bozic	Inf	unif	singleC	CBN, CBN-A, OT, OT-A
779	No	7	7-B	200	Bozic	Inf	unif	wholeT_0.01	OT, OT-A
780	No	7	7-B	200	Bozic	Inf	unif	wholeT_0.5	CBN, CBN-A, OT, OT-A
781	No	7	7-B	200	exp	0	last	singleC	OT-A
782	No	7	7-B	200	exp	0	last	wholeT_0.01	OT-A
783	No	7	7-B	200	exp	0	last	wholeT_0.5	OT-A
784	No	7	7-B	200	exp	0	unif	singleC	OT, OT-A
785	No	7	7-B	200	exp	0	unif	wholeT_0.01	OT, OT-A
786	No	7	7-B	200	exp	0	unif	wholeT_0.5	OT, OT-A
787	No	7	7-B	200	exp	Inf	last	singleC	OT-A
788	No	7	7-B	200	exp	Inf	last	wholeT_0.01	OT-A
789	No	7	7-B	200	exp	Inf	last	wholeT_0.5	OT-A
790	No	7	7-B	200	exp	Inf	unif	singleC	CBN, CBN-A, OT, OT-A
791	No	7	7-B	200	exp	Inf	unif	wholeT_0.01	OT, OT-A
792	No	7	7-B	200	exp	Inf	unif	wholeT_0.5	OT, OT-A
793	No	7	7-B	200	McF_4	0	last	singleC	OT-A
794	No	7	7-B	200	McF_4	0	last	wholeT_0.01	OT-A
795	No	7	7-B	200	McF_4	0	last	wholeT_0.5	OT-A
796	No	7	7-B	200	McF_4	0	unif	singleC	OT, OT-A
797	No	7	7-B	200	McF_4	0	unif	wholeT_0.01	OT-A

Table 9: *(continued)*

	Conjunction	Drivers	Tree	S.Size	Model	sh	S.Time	S.Type	Best method(s)
798	No	7	7-B	200	McF_4	0	unif	wholeT_0.5	OT, OT-A
799	No	7	7-B	200	McF_4	Inf	last	singleC	OT-A
800	No	7	7-B	200	McF_4	Inf	last	wholeT_0.01	OT-A
801	No	7	7-B	200	McF_4	Inf	last	wholeT_0.5	OT-A
802	No	7	7-B	200	McF_4	Inf	unif	singleC	OT, OT-A
803	No	7	7-B	200	McF_4	Inf	unif	wholeT_0.01	OT, OT-A
804	No	7	7-B	200	McF_4	Inf	unif	wholeT_0.5	OT, OT-A
805	No	7	7-B	200	McF_6	0	last	singleC	OT-A
806	No	7	7-B	200	McF_6	0	last	wholeT_0.01	DiP-A, OT-A
807	No	7	7-B	200	McF_6	0	last	wholeT_0.5	OT-A
808	No	7	7-B	200	McF_6	0	unif	singleC	CBN-A, OT, OT-A
809	No	7	7-B	200	McF_6	0	unif	wholeT_0.01	OT, OT-A
810	No	7	7-B	200	McF_6	0	unif	wholeT_0.5	CBN-A, OT, OT-A
811	No	7	7-B	200	McF_6	Inf	last	singleC	OT-A
812	No	7	7-B	200	McF_6	Inf	last	wholeT_0.01	OT-A
813	No	7	7-B	200	McF_6	Inf	last	wholeT_0.5	OT-A
814	No	7	7-B	200	McF_6	Inf	unif	singleC	OT, OT-A
815	No	7	7-B	200	McF_6	Inf	unif	wholeT_0.01	CBN-A, OT, OT-A
816	No	7	7-B	200	McF_6	Inf	unif	wholeT_0.5	CBN-A, OT, OT-A
817	No	7	7-B	100	Bozic	0	last	singleC	OT-A
818	No	7	7-B	100	Bozic	0	last	wholeT_0.01	OT-A
819	No	7	7-B	100	Bozic	0	last	wholeT_0.5	OT-A
820	No	7	7-B	100	Bozic	0	unif	singleC	OT, OT-A
821	No	7	7-B	100	Bozic	0	unif	wholeT_0.01	CBN, CBN-A, OT, OT-A
822	No	7	7-B	100	Bozic	0	unif	wholeT_0.5	OT, OT-A
823	No	7	7-B	100	Bozic	Inf	last	singleC	OT-A
824	No	7	7-B	100	Bozic	Inf	last	wholeT_0.01	OT-A
825	No	7	7-B	100	Bozic	Inf	last	wholeT_0.5	OT-A
826	No	7	7-B	100	Bozic	Inf	unif	singleC	CBN, OT, OT-A
827	No	7	7-B	100	Bozic	Inf	unif	wholeT_0.01	OT, OT-A
828	No	7	7-B	100	Bozic	Inf	unif	wholeT_0.5	CBN-A, OT, OT-A
829	No	7	7-B	100	exp	0	last	singleC	OT-A
830	No	7	7-B	100	exp	0	last	wholeT_0.01	OT-A
831	No	7	7-B	100	exp	0	last	wholeT_0.5	OT-A
832	No	7	7-B	100	exp	0	unif	singleC	OT, OT-A
833	No	7	7-B	100	exp	0	unif	wholeT_0.01	OT, OT-A
834	No	7	7-B	100	exp	0	unif	wholeT_0.5	OT, OT-A
835	No	7	7-B	100	exp	Inf	last	singleC	OT-A
836	No	7	7-B	100	exp	Inf	last	wholeT_0.01	OT-A
837	No	7	7-B	100	exp	Inf	last	wholeT_0.5	OT-A

Table 9: *(continued)*

	Conjunction	Drivers	Tree	S.Size	Model	sh	S.Time	S.Type	Best method(s)
838	No	7	7-B	100	exp	Inf	unif	singleC	OT, OT-A
839	No	7	7-B	100	exp	Inf	unif	wholeT_0.01	CBN, OT, OT-A
840	No	7	7-B	100	exp	Inf	unif	wholeT_0.5	OT-A
841	No	7	7-B	100	McF_4	0	last	singleC	OT-A
842	No	7	7-B	100	McF_4	0	last	wholeT_0.01	OT-A
843	No	7	7-B	100	McF_4	0	last	wholeT_0.5	OT-A
844	No	7	7-B	100	McF_4	0	unif	singleC	OT, OT-A
845	No	7	7-B	100	McF_4	0	unif	wholeT_0.01	OT, OT-A
846	No	7	7-B	100	McF_4	0	unif	wholeT_0.5	OT, OT-A
847	No	7	7-B	100	McF_4	Inf	last	singleC	OT-A
848	No	7	7-B	100	McF_4	Inf	last	wholeT_0.01	OT-A
849	No	7	7-B	100	McF_4	Inf	last	wholeT_0.5	OT-A
850	No	7	7-B	100	McF_4	Inf	unif	singleC	OT, OT-A
851	No	7	7-B	100	McF_4	Inf	unif	wholeT_0.01	OT, OT-A
852	No	7	7-B	100	McF_4	Inf	unif	wholeT_0.5	OT, OT-A
853	No	7	7-B	100	McF_6	0	last	singleC	OT-A
854	No	7	7-B	100	McF_6	0	last	wholeT_0.01	OT-A
855	No	7	7-B	100	McF_6	0	last	wholeT_0.5	OT-A
856	No	7	7-B	100	McF_6	0	unif	singleC	CBN-A, OT, OT-A
857	No	7	7-B	100	McF_6	0	unif	wholeT_0.01	CBN-A, OT, OT-A
858	No	7	7-B	100	McF_6	0	unif	wholeT_0.5	CBN-A, OT, OT-A
859	No	7	7-B	100	McF_6	Inf	last	singleC	OT-A
860	No	7	7-B	100	McF_6	Inf	last	wholeT_0.01	OT-A
861	No	7	7-B	100	McF_6	Inf	last	wholeT_0.5	OT-A
862	No	7	7-B	100	McF_6	Inf	unif	singleC	CBN-A, OT, OT-A
863	No	7	7-B	100	McF_6	Inf	unif	wholeT_0.01	CBN-A, OT-A
864	No	7	7-B	100	McF_6	Inf	unif	wholeT_0.5	CBN-A, OT, OT-A

4.2 Confidence sets (MCB), PFD, Drivers Known

Table 10: Confidence sets (method MCB) when Drivers are Known for measure PFD.

	Conjunction	Drivers	Tree	S.Size	Model	sh	S.Time	S.Type	Best method(s)
1	Yes	11	11-A	1000	Bozic	0	last	singleC	OT, OT-A
2	Yes	11	11-A	1000	Bozic	0	last	wholeT_0.01	DiP, DiP-A
3	Yes	11	11-A	1000	Bozic	0	last	wholeT_0.5	OT, OT-A
4	Yes	11	11-A	1000	Bozic	0	unif	singleC	DiP, DiP-A
5	Yes	11	11-A	1000	Bozic	0	unif	wholeT_0.01	DiP, DiP-A, OT, OT-A
6	Yes	11	11-A	1000	Bozic	0	unif	wholeT_0.5	OT, OT-A
7	Yes	11	11-A	1000	Bozic	Inf	last	singleC	OT, OT-A
8	Yes	11	11-A	1000	Bozic	Inf	last	wholeT_0.01	DiP, DiP-A
9	Yes	11	11-A	1000	Bozic	Inf	last	wholeT_0.5	OT
10	Yes	11	11-A	1000	Bozic	Inf	unif	singleC	OT, OT-A
11	Yes	11	11-A	1000	Bozic	Inf	unif	wholeT_0.01	OT, OT-A
12	Yes	11	11-A	1000	Bozic	Inf	unif	wholeT_0.5	OT, OT-A
13	Yes	11	11-A	1000	exp	0	last	singleC	OT, OT-A
14	Yes	11	11-A	1000	exp	0	last	wholeT_0.01	DiP-A, OT, OT-A
15	Yes	11	11-A	1000	exp	0	last	wholeT_0.5	OT, OT-A
16	Yes	11	11-A	1000	exp	0	unif	singleC	OT, OT-A
17	Yes	11	11-A	1000	exp	0	unif	wholeT_0.01	DiP-A
18	Yes	11	11-A	1000	exp	0	unif	wholeT_0.5	OT, OT-A
19	Yes	11	11-A	1000	exp	Inf	last	singleC	OT, OT-A
20	Yes	11	11-A	1000	exp	Inf	last	wholeT_0.01	OT, OT-A
21	Yes	11	11-A	1000	exp	Inf	last	wholeT_0.5	OT, OT-A
22	Yes	11	11-A	1000	exp	Inf	unif	singleC	OT, OT-A
23	Yes	11	11-A	1000	exp	Inf	unif	wholeT_0.01	OT, OT-A
24	Yes	11	11-A	1000	exp	Inf	unif	wholeT_0.5	OT, OT-A
25	Yes	11	11-A	1000	McF_4	0	last	singleC	DiP-A, OT, OT-A
26	Yes	11	11-A	1000	McF_4	0	last	wholeT_0.01	OT, OT-A
27	Yes	11	11-A	1000	McF_4	0	last	wholeT_0.5	DiP-A, OT, OT-A
28	Yes	11	11-A	1000	McF_4	0	unif	singleC	DiP, DiP-A, OT, OT-A
29	Yes	11	11-A	1000	McF_4	0	unif	wholeT_0.01	DiP, DiP-A
30	Yes	11	11-A	1000	McF_4	0	unif	wholeT_0.5	DiP, DiP-A
31	Yes	11	11-A	1000	McF_4	Inf	last	singleC	OT, OT-A
32	Yes	11	11-A	1000	McF_4	Inf	last	wholeT_0.01	OT, OT-A
33	Yes	11	11-A	1000	McF_4	Inf	last	wholeT_0.5	DiP-A, OT, OT-A
34	Yes	11	11-A	1000	McF_4	Inf	unif	singleC	DiP, DiP-A, OT, OT-A
35	Yes	11	11-A	1000	McF_4	Inf	unif	wholeT_0.01	OT, OT-A
36	Yes	11	11-A	1000	McF_4	Inf	unif	wholeT_0.5	DiP, DiP-A, OT, OT-A
37	Yes	11	11-A	1000	McF_6	0	last	singleC	DiP, OT

Table 10: *(continued)*

	Conjunction	Drivers	Tree	S.Size	Model	sh	S.Time	S.Type	Best method(s)
38	Yes	11	11-A	1000	McF_6	0	last	wholeT_0.01	DiP, OT
39	Yes	11	11-A	1000	McF_6	0	last	wholeT_0.5	DiP, OT
40	Yes	11	11-A	1000	McF_6	0	unif	singleC	DiP, DiP-A, OT, OT-A
41	Yes	11	11-A	1000	McF_6	0	unif	wholeT_0.01	DiP, DiP-A, OT, OT-A
42	Yes	11	11-A	1000	McF_6	0	unif	wholeT_0.5	DiP, DiP-A
43	Yes	11	11-A	1000	McF_6	Inf	last	singleC	DiP, OT
44	Yes	11	11-A	1000	McF_6	Inf	last	wholeT_0.01	DiP, OT
45	Yes	11	11-A	1000	McF_6	Inf	last	wholeT_0.5	DiP, OT
46	Yes	11	11-A	1000	McF_6	Inf	unif	singleC	DiP, DiP-A
47	Yes	11	11-A	1000	McF_6	Inf	unif	wholeT_0.01	DiP, DiP-A
48	Yes	11	11-A	1000	McF_6	Inf	unif	wholeT_0.5	DiP, DiP-A
49	Yes	11	11-A	200	Bozic	0	last	singleC	OT, OT-A
50	Yes	11	11-A	200	Bozic	0	last	wholeT_0.01	OT, OT-A
51	Yes	11	11-A	200	Bozic	0	last	wholeT_0.5	OT, OT-A
52	Yes	11	11-A	200	Bozic	0	unif	singleC	OT, OT-A
53	Yes	11	11-A	200	Bozic	0	unif	wholeT_0.01	DiP, DiP-A, OT, OT-A
54	Yes	11	11-A	200	Bozic	0	unif	wholeT_0.5	OT, OT-A
55	Yes	11	11-A	200	Bozic	Inf	last	singleC	OT, OT-A
56	Yes	11	11-A	200	Bozic	Inf	last	wholeT_0.01	OT, OT-A
57	Yes	11	11-A	200	Bozic	Inf	last	wholeT_0.5	OT, OT-A
58	Yes	11	11-A	200	Bozic	Inf	unif	singleC	OT, OT-A
59	Yes	11	11-A	200	Bozic	Inf	unif	wholeT_0.01	OT, OT-A
60	Yes	11	11-A	200	Bozic	Inf	unif	wholeT_0.5	OT, OT-A
61	Yes	11	11-A	200	exp	0	last	singleC	OT, OT-A
62	Yes	11	11-A	200	exp	0	last	wholeT_0.01	OT, OT-A
63	Yes	11	11-A	200	exp	0	last	wholeT_0.5	OT, OT-A
64	Yes	11	11-A	200	exp	0	unif	singleC	OT, OT-A
65	Yes	11	11-A	200	exp	0	unif	wholeT_0.01	OT, OT-A
66	Yes	11	11-A	200	exp	0	unif	wholeT_0.5	CBN, CBN-A
67	Yes	11	11-A	200	exp	Inf	last	singleC	OT, OT-A
68	Yes	11	11-A	200	exp	Inf	last	wholeT_0.01	OT, OT-A
69	Yes	11	11-A	200	exp	Inf	last	wholeT_0.5	OT, OT-A
70	Yes	11	11-A	200	exp	Inf	unif	singleC	OT, OT-A
71	Yes	11	11-A	200	exp	Inf	unif	wholeT_0.01	OT, OT-A
72	Yes	11	11-A	200	exp	Inf	unif	wholeT_0.5	OT, OT-A
73	Yes	11	11-A	200	McF_4	0	last	singleC	OT, OT-A
74	Yes	11	11-A	200	McF_4	0	last	wholeT_0.01	DiP-A, OT, OT-A
75	Yes	11	11-A	200	McF_4	0	last	wholeT_0.5	DiP-A, OT, OT-A
76	Yes	11	11-A	200	McF_4	0	unif	singleC	OT, OT-A
77	Yes	11	11-A	200	McF_4	0	unif	wholeT_0.01	DiP, DiP-A, OT, OT-A

Table 10: *(continued)*

	Conjunction	Drivers	Tree	S.Size	Model	sh	S.Time	S.Type	Best method(s)
78	Yes	11	11-A	200	McF_4	0	unif	wholeT_0.5	OT, OT-A
79	Yes	11	11-A	200	McF_4	Inf	last	singleC	OT, OT-A
80	Yes	11	11-A	200	McF_4	Inf	last	wholeT_0.01	OT, OT-A
81	Yes	11	11-A	200	McF_4	Inf	last	wholeT_0.5	OT, OT-A
82	Yes	11	11-A	200	McF_4	Inf	unif	singleC	OT, OT-A
83	Yes	11	11-A	200	McF_4	Inf	unif	wholeT_0.01	OT, OT-A
84	Yes	11	11-A	200	McF_4	Inf	unif	wholeT_0.5	OT, OT-A
85	Yes	11	11-A	200	McF_6	0	last	singleC	DiP, OT
86	Yes	11	11-A	200	McF_6	0	last	wholeT_0.01	DiP, OT
87	Yes	11	11-A	200	McF_6	0	last	wholeT_0.5	DiP, OT
88	Yes	11	11-A	200	McF_6	0	unif	singleC	OT, OT-A
89	Yes	11	11-A	200	McF_6	0	unif	wholeT_0.01	DiP, DiP-A, OT, OT-A
90	Yes	11	11-A	200	McF_6	0	unif	wholeT_0.5	OT, OT-A
91	Yes	11	11-A	200	McF_6	Inf	last	singleC	OT
92	Yes	11	11-A	200	McF_6	Inf	last	wholeT_0.01	OT
93	Yes	11	11-A	200	McF_6	Inf	last	wholeT_0.5	OT
94	Yes	11	11-A	200	McF_6	Inf	unif	singleC	CBN-A, OT, OT-A
95	Yes	11	11-A	200	McF_6	Inf	unif	wholeT_0.01	OT, OT-A
96	Yes	11	11-A	200	McF_6	Inf	unif	wholeT_0.5	OT, OT-A
97	Yes	11	11-A	100	Bozic	0	last	singleC	OT, OT-A
98	Yes	11	11-A	100	Bozic	0	last	wholeT_0.01	OT, OT-A
99	Yes	11	11-A	100	Bozic	0	last	wholeT_0.5	OT, OT-A
100	Yes	11	11-A	100	Bozic	0	unif	singleC	OT, OT-A
101	Yes	11	11-A	100	Bozic	0	unif	wholeT_0.01	OT, OT-A
102	Yes	11	11-A	100	Bozic	0	unif	wholeT_0.5	CBN, CBN-A
103	Yes	11	11-A	100	Bozic	Inf	last	singleC	OT, OT-A
104	Yes	11	11-A	100	Bozic	Inf	last	wholeT_0.01	OT, OT-A
105	Yes	11	11-A	100	Bozic	Inf	last	wholeT_0.5	OT, OT-A
106	Yes	11	11-A	100	Bozic	Inf	unif	singleC	OT, OT-A
107	Yes	11	11-A	100	Bozic	Inf	unif	wholeT_0.01	OT, OT-A
108	Yes	11	11-A	100	Bozic	Inf	unif	wholeT_0.5	OT, OT-A
109	Yes	11	11-A	100	exp	0	last	singleC	OT, OT-A
110	Yes	11	11-A	100	exp	0	last	wholeT_0.01	OT, OT-A
111	Yes	11	11-A	100	exp	0	last	wholeT_0.5	OT, OT-A
112	Yes	11	11-A	100	exp	0	unif	singleC	CBN, CBN-A
113	Yes	11	11-A	100	exp	0	unif	wholeT_0.01	OT, OT-A
114	Yes	11	11-A	100	exp	0	unif	wholeT_0.5	CBN, CBN-A
115	Yes	11	11-A	100	exp	Inf	last	singleC	OT, OT-A
116	Yes	11	11-A	100	exp	Inf	last	wholeT_0.01	OT, OT-A
117	Yes	11	11-A	100	exp	Inf	last	wholeT_0.5	OT, OT-A

Table 10: *(continued)*

	Conjunction	Drivers	Tree	S.Size	Model	sh	S.Time	S.Type	Best method(s)
118	Yes	11	11-A	100	exp	Inf	unif	singleC	OT, OT-A
119	Yes	11	11-A	100	exp	Inf	unif	wholeT_0.01	OT, OT-A
120	Yes	11	11-A	100	exp	Inf	unif	wholeT_0.5	OT, OT-A
121	Yes	11	11-A	100	McF_4	0	last	singleC	OT, OT-A
122	Yes	11	11-A	100	McF_4	0	last	wholeT_0.01	DiP, DiP-A, OT, OT-A
123	Yes	11	11-A	100	McF_4	0	last	wholeT_0.5	OT, OT-A
124	Yes	11	11-A	100	McF_4	0	unif	singleC	OT, OT-A
125	Yes	11	11-A	100	McF_4	0	unif	wholeT_0.01	DiP, DiP-A, OT, OT-A
126	Yes	11	11-A	100	McF_4	0	unif	wholeT_0.5	OT, OT-A
127	Yes	11	11-A	100	McF_4	Inf	last	singleC	OT, OT-A
128	Yes	11	11-A	100	McF_4	Inf	last	wholeT_0.01	OT, OT-A
129	Yes	11	11-A	100	McF_4	Inf	last	wholeT_0.5	OT, OT-A
130	Yes	11	11-A	100	McF_4	Inf	unif	singleC	OT, OT-A
131	Yes	11	11-A	100	McF_4	Inf	unif	wholeT_0.01	OT, OT-A
132	Yes	11	11-A	100	McF_4	Inf	unif	wholeT_0.5	OT, OT-A
133	Yes	11	11-A	100	McF_6	0	last	singleC	DiP, OT
134	Yes	11	11-A	100	McF_6	0	last	wholeT_0.01	DiP, OT
135	Yes	11	11-A	100	McF_6	0	last	wholeT_0.5	OT
136	Yes	11	11-A	100	McF_6	0	unif	singleC	OT, OT-A
137	Yes	11	11-A	100	McF_6	0	unif	wholeT_0.01	DiP, DiP-A, OT
138	Yes	11	11-A	100	McF_6	0	unif	wholeT_0.5	OT, OT-A
139	Yes	11	11-A	100	McF_6	Inf	last	singleC	OT
140	Yes	11	11-A	100	McF_6	Inf	last	wholeT_0.01	OT
141	Yes	11	11-A	100	McF_6	Inf	last	wholeT_0.5	OT
142	Yes	11	11-A	100	McF_6	Inf	unif	singleC	OT, OT-A
143	Yes	11	11-A	100	McF_6	Inf	unif	wholeT_0.01	OT, OT-A
144	Yes	11	11-A	100	McF_6	Inf	unif	wholeT_0.5	OT, OT-A
145	Yes	9	9-A	1000	Bozic	0	last	singleC	OT, OT-A
146	Yes	9	9-A	1000	Bozic	0	last	wholeT_0.01	DiP, DiP-A
147	Yes	9	9-A	1000	Bozic	0	last	wholeT_0.5	OT, OT-A
148	Yes	9	9-A	1000	Bozic	0	unif	singleC	OT, OT-A
149	Yes	9	9-A	1000	Bozic	0	unif	wholeT_0.01	DiP, DiP-A, OT
150	Yes	9	9-A	1000	Bozic	0	unif	wholeT_0.5	OT, OT-A
151	Yes	9	9-A	1000	Bozic	Inf	last	singleC	OT, OT-A
152	Yes	9	9-A	1000	Bozic	Inf	last	wholeT_0.01	DiP, DiP-A, OT, OT-A
153	Yes	9	9-A	1000	Bozic	Inf	last	wholeT_0.5	OT, OT-A
154	Yes	9	9-A	1000	Bozic	Inf	unif	singleC	DiP, DiP-A, OT
155	Yes	9	9-A	1000	Bozic	Inf	unif	wholeT_0.01	DiP, DiP-A, OT, OT-A
156	Yes	9	9-A	1000	Bozic	Inf	unif	wholeT_0.5	DiP-A, OT, OT-A
157	Yes	9	9-A	1000	exp	0	last	singleC	OT, OT-A

Table 10: *(continued)*

	Conjunction	Drivers	Tree	S.Size	Model	sh	S.Time	S.Type	Best method(s)
158	Yes	9	9-A	1000	exp	0	last	wholeT_0.01	DiP-A, OT, OT-A
159	Yes	9	9-A	1000	exp	0	last	wholeT_0.5	OT, OT-A
160	Yes	9	9-A	1000	exp	0	unif	singleC	OT, OT-A
161	Yes	9	9-A	1000	exp	0	unif	wholeT_0.01	OT
162	Yes	9	9-A	1000	exp	0	unif	wholeT_0.5	CBN, CBN-A
163	Yes	9	9-A	1000	exp	Inf	last	singleC	OT, OT-A
164	Yes	9	9-A	1000	exp	Inf	last	wholeT_0.01	CBN, DiP, DiP-A, OT, OT-A
165	Yes	9	9-A	1000	exp	Inf	last	wholeT_0.5	OT, OT-A
166	Yes	9	9-A	1000	exp	Inf	unif	singleC	OT, OT-A
167	Yes	9	9-A	1000	exp	Inf	unif	wholeT_0.01	OT, OT-A
168	Yes	9	9-A	1000	exp	Inf	unif	wholeT_0.5	OT, OT-A
169	Yes	9	9-A	1000	McF_4	0	last	singleC	DiP, DiP-A, OT, OT-A
170	Yes	9	9-A	1000	McF_4	0	last	wholeT_0.01	DiP, DiP-A, OT, OT-A
171	Yes	9	9-A	1000	McF_4	0	last	wholeT_0.5	DiP, DiP-A, OT, OT-A
172	Yes	9	9-A	1000	McF_4	0	unif	singleC	DiP, DiP-A, OT, OT-A
173	Yes	9	9-A	1000	McF_4	0	unif	wholeT_0.01	DiP, DiP-A
174	Yes	9	9-A	1000	McF_4	0	unif	wholeT_0.5	DiP, DiP-A, OT-A
175	Yes	9	9-A	1000	McF_4	Inf	last	singleC	OT, OT-A
176	Yes	9	9-A	1000	McF_4	Inf	last	wholeT_0.01	DiP, OT
177	Yes	9	9-A	1000	McF_4	Inf	last	wholeT_0.5	DiP, DiP-A, OT, OT-A
178	Yes	9	9-A	1000	McF_4	Inf	unif	singleC	DiP, DiP-A, OT, OT-A
179	Yes	9	9-A	1000	McF_4	Inf	unif	wholeT_0.01	OT, OT-A
180	Yes	9	9-A	1000	McF_4	Inf	unif	wholeT_0.5	DiP, DiP-A, OT, OT-A
181	Yes	9	9-A	1000	McF_6	0	last	singleC	OT
182	Yes	9	9-A	1000	McF_6	0	last	wholeT_0.01	DiP, OT
183	Yes	9	9-A	1000	McF_6	0	last	wholeT_0.5	DiP, OT
184	Yes	9	9-A	1000	McF_6	0	unif	singleC	DiP, DiP-A, OT, OT-A
185	Yes	9	9-A	1000	McF_6	0	unif	wholeT_0.01	DiP, DiP-A
186	Yes	9	9-A	1000	McF_6	0	unif	wholeT_0.5	DiP, DiP-A, OT
187	Yes	9	9-A	1000	McF_6	Inf	last	singleC	DiP, OT
188	Yes	9	9-A	1000	McF_6	Inf	last	wholeT_0.01	OT
189	Yes	9	9-A	1000	McF_6	Inf	last	wholeT_0.5	DiP, OT
190	Yes	9	9-A	1000	McF_6	Inf	unif	singleC	DiP, DiP-A, OT, OT-A
191	Yes	9	9-A	1000	McF_6	Inf	unif	wholeT_0.01	DiP, DiP-A, OT, OT-A
192	Yes	9	9-A	1000	McF_6	Inf	unif	wholeT_0.5	DiP, DiP-A, OT, OT-A
193	Yes	9	9-A	200	Bozic	0	last	singleC	OT, OT-A
194	Yes	9	9-A	200	Bozic	0	last	wholeT_0.01	OT, OT-A
195	Yes	9	9-A	200	Bozic	0	last	wholeT_0.5	OT, OT-A
196	Yes	9	9-A	200	Bozic	0	unif	singleC	CBN
197	Yes	9	9-A	200	Bozic	0	unif	wholeT_0.01	DiP-A, OT, OT-A

Table 10: *(continued)*

	Conjunction	Drivers	Tree	S.Size	Model	sh	S.Time	S.Type	Best method(s)
198	Yes	9	9-A	200	Bozic	0	unif	wholeT_0.5	CBN, CBN-A
199	Yes	9	9-A	200	Bozic	Inf	last	singleC	OT, OT-A
200	Yes	9	9-A	200	Bozic	Inf	last	wholeT_0.01	OT, OT-A
201	Yes	9	9-A	200	Bozic	Inf	last	wholeT_0.5	OT, OT-A
202	Yes	9	9-A	200	Bozic	Inf	unif	singleC	OT, OT-A
203	Yes	9	9-A	200	Bozic	Inf	unif	wholeT_0.01	OT, OT-A
204	Yes	9	9-A	200	Bozic	Inf	unif	wholeT_0.5	OT, OT-A
205	Yes	9	9-A	200	exp	0	last	singleC	OT, OT-A
206	Yes	9	9-A	200	exp	0	last	wholeT_0.01	OT, OT-A
207	Yes	9	9-A	200	exp	0	last	wholeT_0.5	OT, OT-A
208	Yes	9	9-A	200	exp	0	unif	singleC	CBN-A
209	Yes	9	9-A	200	exp	0	unif	wholeT_0.01	OT, OT-A
210	Yes	9	9-A	200	exp	0	unif	wholeT_0.5	CBN, CBN-A
211	Yes	9	9-A	200	exp	Inf	last	singleC	OT, OT-A
212	Yes	9	9-A	200	exp	Inf	last	wholeT_0.01	OT, OT-A
213	Yes	9	9-A	200	exp	Inf	last	wholeT_0.5	OT, OT-A
214	Yes	9	9-A	200	exp	Inf	unif	singleC	OT, OT-A
215	Yes	9	9-A	200	exp	Inf	unif	wholeT_0.01	CBN-A, OT, OT-A
216	Yes	9	9-A	200	exp	Inf	unif	wholeT_0.5	OT, OT-A
217	Yes	9	9-A	200	McF_4	0	last	singleC	DiP-A, OT, OT-A
218	Yes	9	9-A	200	McF_4	0	last	wholeT_0.01	DiP, DiP-A
219	Yes	9	9-A	200	McF_4	0	last	wholeT_0.5	DiP-A, OT, OT-A
220	Yes	9	9-A	200	McF_4	0	unif	singleC	OT, OT-A
221	Yes	9	9-A	200	McF_4	0	unif	wholeT_0.01	DiP, DiP-A
222	Yes	9	9-A	200	McF_4	0	unif	wholeT_0.5	OT, OT-A
223	Yes	9	9-A	200	McF_4	Inf	last	singleC	OT, OT-A
224	Yes	9	9-A	200	McF_4	Inf	last	wholeT_0.01	DiP-A, OT
225	Yes	9	9-A	200	McF_4	Inf	last	wholeT_0.5	OT, OT-A
226	Yes	9	9-A	200	McF_4	Inf	unif	singleC	OT, OT-A
227	Yes	9	9-A	200	McF_4	Inf	unif	wholeT_0.01	OT, OT-A
228	Yes	9	9-A	200	McF_4	Inf	unif	wholeT_0.5	OT, OT-A
229	Yes	9	9-A	200	McF_6	0	last	singleC	DiP, OT
230	Yes	9	9-A	200	McF_6	0	last	wholeT_0.01	DiP, OT
231	Yes	9	9-A	200	McF_6	0	last	wholeT_0.5	OT
232	Yes	9	9-A	200	McF_6	0	unif	singleC	DiP, DiP-A, OT, OT-A
233	Yes	9	9-A	200	McF_6	0	unif	wholeT_0.01	DiP, DiP-A, OT-A
234	Yes	9	9-A	200	McF_6	0	unif	wholeT_0.5	DiP, DiP-A, OT, OT-A
235	Yes	9	9-A	200	McF_6	Inf	last	singleC	OT
236	Yes	9	9-A	200	McF_6	Inf	last	wholeT_0.01	OT
237	Yes	9	9-A	200	McF_6	Inf	last	wholeT_0.5	OT

Table 10: *(continued)*

	Conjunction	Drivers	Tree	S.Size	Model	sh	S.Time	S.Type	Best method(s)
238	Yes	9	9-A	200	McF_6	Inf	unif	singleC	OT, OT-A
239	Yes	9	9-A	200	McF_6	Inf	unif	wholeT_0.01	CBN, DiP-A, OT, OT-A
240	Yes	9	9-A	200	McF_6	Inf	unif	wholeT_0.5	DiP, DiP-A, OT, OT-A
241	Yes	9	9-A	100	Bozic	0	last	singleC	OT, OT-A
242	Yes	9	9-A	100	Bozic	0	last	wholeT_0.01	OT, OT-A
243	Yes	9	9-A	100	Bozic	0	last	wholeT_0.5	OT, OT-A
244	Yes	9	9-A	100	Bozic	0	unif	singleC	CBN, CBN-A
245	Yes	9	9-A	100	Bozic	0	unif	wholeT_0.01	OT, OT-A
246	Yes	9	9-A	100	Bozic	0	unif	wholeT_0.5	CBN, CBN-A
247	Yes	9	9-A	100	Bozic	Inf	last	singleC	OT, OT-A
248	Yes	9	9-A	100	Bozic	Inf	last	wholeT_0.01	OT, OT-A
249	Yes	9	9-A	100	Bozic	Inf	last	wholeT_0.5	OT, OT-A
250	Yes	9	9-A	100	Bozic	Inf	unif	singleC	OT, OT-A
251	Yes	9	9-A	100	Bozic	Inf	unif	wholeT_0.01	OT, OT-A
252	Yes	9	9-A	100	Bozic	Inf	unif	wholeT_0.5	OT, OT-A
253	Yes	9	9-A	100	exp	0	last	singleC	OT, OT-A
254	Yes	9	9-A	100	exp	0	last	wholeT_0.01	OT, OT-A
255	Yes	9	9-A	100	exp	0	last	wholeT_0.5	OT, OT-A
256	Yes	9	9-A	100	exp	0	unif	singleC	CBN, CBN-A
257	Yes	9	9-A	100	exp	0	unif	wholeT_0.01	OT, OT-A
258	Yes	9	9-A	100	exp	0	unif	wholeT_0.5	CBN, CBN-A
259	Yes	9	9-A	100	exp	Inf	last	singleC	OT, OT-A
260	Yes	9	9-A	100	exp	Inf	last	wholeT_0.01	OT, OT-A
261	Yes	9	9-A	100	exp	Inf	last	wholeT_0.5	OT, OT-A
262	Yes	9	9-A	100	exp	Inf	unif	singleC	OT, OT-A
263	Yes	9	9-A	100	exp	Inf	unif	wholeT_0.01	OT, OT-A
264	Yes	9	9-A	100	exp	Inf	unif	wholeT_0.5	OT, OT-A
265	Yes	9	9-A	100	McF_4	0	last	singleC	OT, OT-A
266	Yes	9	9-A	100	McF_4	0	last	wholeT_0.01	DiP, DiP-A
267	Yes	9	9-A	100	McF_4	0	last	wholeT_0.5	OT, OT-A
268	Yes	9	9-A	100	McF_4	0	unif	singleC	OT, OT-A
269	Yes	9	9-A	100	McF_4	0	unif	wholeT_0.01	OT, OT-A
270	Yes	9	9-A	100	McF_4	0	unif	wholeT_0.5	OT, OT-A
271	Yes	9	9-A	100	McF_4	Inf	last	singleC	OT, OT-A
272	Yes	9	9-A	100	McF_4	Inf	last	wholeT_0.01	OT
273	Yes	9	9-A	100	McF_4	Inf	last	wholeT_0.5	OT, OT-A
274	Yes	9	9-A	100	McF_4	Inf	unif	singleC	OT, OT-A
275	Yes	9	9-A	100	McF_4	Inf	unif	wholeT_0.01	OT, OT-A
276	Yes	9	9-A	100	McF_4	Inf	unif	wholeT_0.5	OT, OT-A
277	Yes	9	9-A	100	McF_6	0	last	singleC	OT

Table 10: *(continued)*

	Conjunction	Drivers	Tree	S.Size	Model	sh	S.Time	S.Type	Best method(s)
278	Yes	9	9-A	100	McF_6	0	last	wholeT_0.01	OT
279	Yes	9	9-A	100	McF_6	0	last	wholeT_0.5	OT
280	Yes	9	9-A	100	McF_6	0	unif	singleC	OT
281	Yes	9	9-A	100	McF_6	0	unif	wholeT_0.01	DiP, DiP-A, OT, OT-A
282	Yes	9	9-A	100	McF_6	0	unif	wholeT_0.5	OT, OT-A
283	Yes	9	9-A	100	McF_6	Inf	last	singleC	OT
284	Yes	9	9-A	100	McF_6	Inf	last	wholeT_0.01	OT
285	Yes	9	9-A	100	McF_6	Inf	last	wholeT_0.5	OT
286	Yes	9	9-A	100	McF_6	Inf	unif	singleC	OT, OT-A
287	Yes	9	9-A	100	McF_6	Inf	unif	wholeT_0.01	OT, OT-A
288	Yes	9	9-A	100	McF_6	Inf	unif	wholeT_0.5	OT, OT-A
289	Yes	7	7-A	1000	Bozic	0	last	singleC	OT-A
290	Yes	7	7-A	1000	Bozic	0	last	wholeT_0.01	DiP-A
291	Yes	7	7-A	1000	Bozic	0	last	wholeT_0.5	OT-A
292	Yes	7	7-A	1000	Bozic	0	unif	singleC	OT, OT-A
293	Yes	7	7-A	1000	Bozic	0	unif	wholeT_0.01	CBN, CBN-A
294	Yes	7	7-A	1000	Bozic	0	unif	wholeT_0.5	OT, OT-A
295	Yes	7	7-A	1000	Bozic	Inf	last	singleC	DiP-A, OT, OT-A
296	Yes	7	7-A	1000	Bozic	Inf	last	wholeT_0.01	DiP-A, OT, OT-A
297	Yes	7	7-A	1000	Bozic	Inf	last	wholeT_0.5	DiP-A, OT, OT-A
298	Yes	7	7-A	1000	Bozic	Inf	unif	singleC	CBN, CBN-A
299	Yes	7	7-A	1000	Bozic	Inf	unif	wholeT_0.01	CBN, CBN-A, OT, OT-A
300	Yes	7	7-A	1000	Bozic	Inf	unif	wholeT_0.5	CBN, CBN-A
301	Yes	7	7-A	1000	exp	0	last	singleC	OT-A
302	Yes	7	7-A	1000	exp	0	last	wholeT_0.01	OT-A
303	Yes	7	7-A	1000	exp	0	last	wholeT_0.5	OT-A
304	Yes	7	7-A	1000	exp	0	unif	singleC	OT, OT-A
305	Yes	7	7-A	1000	exp	0	unif	wholeT_0.01	CBN, CBN-A
306	Yes	7	7-A	1000	exp	0	unif	wholeT_0.5	OT, OT-A
307	Yes	7	7-A	1000	exp	Inf	last	singleC	OT-A
308	Yes	7	7-A	1000	exp	Inf	last	wholeT_0.01	DiP-A, OT-A
309	Yes	7	7-A	1000	exp	Inf	last	wholeT_0.5	OT-A
310	Yes	7	7-A	1000	exp	Inf	unif	singleC	CBN, CBN-A, OT, OT-A
311	Yes	7	7-A	1000	exp	Inf	unif	wholeT_0.01	CBN, CBN-A
312	Yes	7	7-A	1000	exp	Inf	unif	wholeT_0.5	CBN, CBN-A
313	Yes	7	7-A	1000	McF_4	0	last	singleC	DiP-A, OT, OT-A
314	Yes	7	7-A	1000	McF_4	0	last	wholeT_0.01	OT, OT-A
315	Yes	7	7-A	1000	McF_4	0	last	wholeT_0.5	DiP-A, OT-A
316	Yes	7	7-A	1000	McF_4	0	unif	singleC	DiP, DiP-A
317	Yes	7	7-A	1000	McF_4	0	unif	wholeT_0.01	DiP, DiP-A, OT

Table 10: *(continued)*

	Conjunction	Drivers	Tree	S.Size	Model	sh	S.Time	S.Type	Best method(s)
318	Yes	7	7-A	1000	McF_4	0	unif	wholeT_0.5	DiP, DiP-A, OT
319	Yes	7	7-A	1000	McF_4	Inf	last	singleC	DiP-A, OT-A
320	Yes	7	7-A	1000	McF_4	Inf	last	wholeT_0.01	OT, OT-A
321	Yes	7	7-A	1000	McF_4	Inf	last	wholeT_0.5	DiP-A, OT-A
322	Yes	7	7-A	1000	McF_4	Inf	unif	singleC	OT, OT-A
323	Yes	7	7-A	1000	McF_4	Inf	unif	wholeT_0.01	DiP-A, OT, OT-A
324	Yes	7	7-A	1000	McF_4	Inf	unif	wholeT_0.5	DiP-A, OT, OT-A
325	Yes	7	7-A	1000	McF_6	0	last	singleC	DiP, OT, OT-A
326	Yes	7	7-A	1000	McF_6	0	last	wholeT_0.01	DiP, OT, OT-A
327	Yes	7	7-A	1000	McF_6	0	last	wholeT_0.5	DiP, OT, OT-A
328	Yes	7	7-A	1000	McF_6	0	unif	singleC	none
329	Yes	7	7-A	1000	McF_6	0	unif	wholeT_0.01	none
330	Yes	7	7-A	1000	McF_6	0	unif	wholeT_0.5	none
331	Yes	7	7-A	1000	McF_6	Inf	last	singleC	DiP, DiP-A, OT, OT-A
332	Yes	7	7-A	1000	McF_6	Inf	last	wholeT_0.01	OT, OT-A
333	Yes	7	7-A	1000	McF_6	Inf	last	wholeT_0.5	DiP, OT, OT-A
334	Yes	7	7-A	1000	McF_6	Inf	unif	singleC	none
335	Yes	7	7-A	1000	McF_6	Inf	unif	wholeT_0.01	CBN, CBN-A, OT, OT-A
336	Yes	7	7-A	1000	McF_6	Inf	unif	wholeT_0.5	none
337	Yes	7	7-A	200	Bozic	0	last	singleC	OT-A
338	Yes	7	7-A	200	Bozic	0	last	wholeT_0.01	OT-A
339	Yes	7	7-A	200	Bozic	0	last	wholeT_0.5	OT-A
340	Yes	7	7-A	200	Bozic	0	unif	singleC	OT, OT-A
341	Yes	7	7-A	200	Bozic	0	unif	wholeT_0.01	CBN, CBN-A
342	Yes	7	7-A	200	Bozic	0	unif	wholeT_0.5	OT, OT-A
343	Yes	7	7-A	200	Bozic	Inf	last	singleC	OT-A
344	Yes	7	7-A	200	Bozic	Inf	last	wholeT_0.01	OT-A
345	Yes	7	7-A	200	Bozic	Inf	last	wholeT_0.5	OT-A
346	Yes	7	7-A	200	Bozic	Inf	unif	singleC	CBN, CBN-A, OT, OT-A
347	Yes	7	7-A	200	Bozic	Inf	unif	wholeT_0.01	CBN, CBN-A
348	Yes	7	7-A	200	Bozic	Inf	unif	wholeT_0.5	CBN, CBN-A, OT, OT-A
349	Yes	7	7-A	200	exp	0	last	singleC	OT-A
350	Yes	7	7-A	200	exp	0	last	wholeT_0.01	OT-A
351	Yes	7	7-A	200	exp	0	last	wholeT_0.5	OT-A
352	Yes	7	7-A	200	exp	0	unif	singleC	CBN, CBN-A
353	Yes	7	7-A	200	exp	0	unif	wholeT_0.01	CBN, CBN-A, OT, OT-A
354	Yes	7	7-A	200	exp	0	unif	wholeT_0.5	CBN, CBN-A
355	Yes	7	7-A	200	exp	Inf	last	singleC	OT-A
356	Yes	7	7-A	200	exp	Inf	last	wholeT_0.01	OT-A
357	Yes	7	7-A	200	exp	Inf	last	wholeT_0.5	OT-A

Table 10: *(continued)*

	Conjunction	Drivers	Tree	S.Size	Model	sh	S.Time	S.Type	Best method(s)
358	Yes	7	7-A	200	exp	Inf	unif	singleC	CBN, CBN-A, OT, OT-A
359	Yes	7	7-A	200	exp	Inf	unif	wholeT_0.01	CBN, CBN-A
360	Yes	7	7-A	200	exp	Inf	unif	wholeT_0.5	CBN, CBN-A, OT, OT-A
361	Yes	7	7-A	200	McF_4	0	last	singleC	OT, OT-A
362	Yes	7	7-A	200	McF_4	0	last	wholeT_0.01	OT-A
363	Yes	7	7-A	200	McF_4	0	last	wholeT_0.5	OT-A
364	Yes	7	7-A	200	McF_4	0	unif	singleC	OT, OT-A
365	Yes	7	7-A	200	McF_4	0	unif	wholeT_0.01	DiP-A, OT-A
366	Yes	7	7-A	200	McF_4	0	unif	wholeT_0.5	OT, OT-A
367	Yes	7	7-A	200	McF_4	Inf	last	singleC	OT-A
368	Yes	7	7-A	200	McF_4	Inf	last	wholeT_0.01	OT, OT-A
369	Yes	7	7-A	200	McF_4	Inf	last	wholeT_0.5	OT-A
370	Yes	7	7-A	200	McF_4	Inf	unif	singleC	OT, OT-A
371	Yes	7	7-A	200	McF_4	Inf	unif	wholeT_0.01	OT, OT-A
372	Yes	7	7-A	200	McF_4	Inf	unif	wholeT_0.5	OT, OT-A
373	Yes	7	7-A	200	McF_6	0	last	singleC	OT, OT-A
374	Yes	7	7-A	200	McF_6	0	last	wholeT_0.01	OT, OT-A
375	Yes	7	7-A	200	McF_6	0	last	wholeT_0.5	OT, OT-A
376	Yes	7	7-A	200	McF_6	0	unif	singleC	CBN, CBN-A, DiP-A
377	Yes	7	7-A	200	McF_6	0	unif	wholeT_0.01	none
378	Yes	7	7-A	200	McF_6	0	unif	wholeT_0.5	CBN, CBN-A
379	Yes	7	7-A	200	McF_6	Inf	last	singleC	DiP-A, OT, OT-A
380	Yes	7	7-A	200	McF_6	Inf	last	wholeT_0.01	OT, OT-A
381	Yes	7	7-A	200	McF_6	Inf	last	wholeT_0.5	DiP-A, OT, OT-A
382	Yes	7	7-A	200	McF_6	Inf	unif	singleC	CBN, CBN-A
383	Yes	7	7-A	200	McF_6	Inf	unif	wholeT_0.01	CBN, CBN-A
384	Yes	7	7-A	200	McF_6	Inf	unif	wholeT_0.5	CBN, CBN-A
385	Yes	7	7-A	100	Bozic	0	last	singleC	OT-A
386	Yes	7	7-A	100	Bozic	0	last	wholeT_0.01	OT-A
387	Yes	7	7-A	100	Bozic	0	last	wholeT_0.5	OT-A
388	Yes	7	7-A	100	Bozic	0	unif	singleC	OT, OT-A
389	Yes	7	7-A	100	Bozic	0	unif	wholeT_0.01	CBN, CBN-A, OT-A
390	Yes	7	7-A	100	Bozic	0	unif	wholeT_0.5	OT, OT-A
391	Yes	7	7-A	100	Bozic	Inf	last	singleC	OT-A
392	Yes	7	7-A	100	Bozic	Inf	last	wholeT_0.01	OT-A
393	Yes	7	7-A	100	Bozic	Inf	last	wholeT_0.5	OT-A
394	Yes	7	7-A	100	Bozic	Inf	unif	singleC	CBN, CBN-A
395	Yes	7	7-A	100	Bozic	Inf	unif	wholeT_0.01	CBN, CBN-A
396	Yes	7	7-A	100	Bozic	Inf	unif	wholeT_0.5	CBN, CBN-A, OT, OT-A
397	Yes	7	7-A	100	exp	0	last	singleC	OT-A

Table 10: *(continued)*

	Conjunction	Drivers	Tree	S.Size	Model	sh	S.Time	S.Type	Best method(s)
398	Yes	7	7-A	100	exp	0	last	wholeT_0.01	OT-A
399	Yes	7	7-A	100	exp	0	last	wholeT_0.5	OT-A
400	Yes	7	7-A	100	exp	0	unif	singleC	OT, OT-A
401	Yes	7	7-A	100	exp	0	unif	wholeT_0.01	OT, OT-A
402	Yes	7	7-A	100	exp	0	unif	wholeT_0.5	CBN, CBN-A
403	Yes	7	7-A	100	exp	Inf	last	singleC	OT-A
404	Yes	7	7-A	100	exp	Inf	last	wholeT_0.01	OT-A
405	Yes	7	7-A	100	exp	Inf	last	wholeT_0.5	OT-A
406	Yes	7	7-A	100	exp	Inf	unif	singleC	OT, OT-A
407	Yes	7	7-A	100	exp	Inf	unif	wholeT_0.01	CBN, CBN-A, OT, OT-A
408	Yes	7	7-A	100	exp	Inf	unif	wholeT_0.5	OT, OT-A
409	Yes	7	7-A	100	McF_4	0	last	singleC	OT-A
410	Yes	7	7-A	100	McF_4	0	last	wholeT_0.01	OT, OT-A
411	Yes	7	7-A	100	McF_4	0	last	wholeT_0.5	OT-A
412	Yes	7	7-A	100	McF_4	0	unif	singleC	OT, OT-A
413	Yes	7	7-A	100	McF_4	0	unif	wholeT_0.01	OT, OT-A
414	Yes	7	7-A	100	McF_4	0	unif	wholeT_0.5	OT, OT-A
415	Yes	7	7-A	100	McF_4	Inf	last	singleC	OT-A
416	Yes	7	7-A	100	McF_4	Inf	last	wholeT_0.01	OT, OT-A
417	Yes	7	7-A	100	McF_4	Inf	last	wholeT_0.5	OT-A
418	Yes	7	7-A	100	McF_4	Inf	unif	singleC	OT, OT-A
419	Yes	7	7-A	100	McF_4	Inf	unif	wholeT_0.01	OT, OT-A
420	Yes	7	7-A	100	McF_4	Inf	unif	wholeT_0.5	OT, OT-A
421	Yes	7	7-A	100	McF_6	0	last	singleC	OT, OT-A
422	Yes	7	7-A	100	McF_6	0	last	wholeT_0.01	OT, OT-A
423	Yes	7	7-A	100	McF_6	0	last	wholeT_0.5	OT, OT-A
424	Yes	7	7-A	100	McF_6	0	unif	singleC	CBN
425	Yes	7	7-A	100	McF_6	0	unif	wholeT_0.01	CBN, CBN-A, DiP-A
426	Yes	7	7-A	100	McF_6	0	unif	wholeT_0.5	CBN, CBN-A
427	Yes	7	7-A	100	McF_6	Inf	last	singleC	OT, OT-A
428	Yes	7	7-A	100	McF_6	Inf	last	wholeT_0.01	OT, OT-A
429	Yes	7	7-A	100	McF_6	Inf	last	wholeT_0.5	OT, OT-A
430	Yes	7	7-A	100	McF_6	Inf	unif	singleC	CBN, CBN-A
431	Yes	7	7-A	100	McF_6	Inf	unif	wholeT_0.01	CBN, CBN-A
432	Yes	7	7-A	100	McF_6	Inf	unif	wholeT_0.5	CBN, CBN-A
433	No	11	11-B	1000	Bozic	0	last	singleC	OT, OT-A
434	No	11	11-B	1000	Bozic	0	last	wholeT_0.01	DiP, DiP-A, OT, OT-A
435	No	11	11-B	1000	Bozic	0	last	wholeT_0.5	OT, OT-A
436	No	11	11-B	1000	Bozic	0	unif	singleC	DiP, DiP-A, OT, OT-A
437	No	11	11-B	1000	Bozic	0	unif	wholeT_0.01	DiP, DiP-A, OT, OT-A

Table 10: *(continued)*

	Conjunction	Drivers	Tree	S.Size	Model	sh	S.Time	S.Type	Best method(s)
438	No	11	11-B	1000	Bozic	0	unif	wholeT_0.5	OT, OT-A
439	No	11	11-B	1000	Bozic	Inf	last	singleC	OT, OT-A
440	No	11	11-B	1000	Bozic	Inf	last	wholeT_0.01	DiP-A
441	No	11	11-B	1000	Bozic	Inf	last	wholeT_0.5	OT, OT-A
442	No	11	11-B	1000	Bozic	Inf	unif	singleC	DiP-A, OT, OT-A
443	No	11	11-B	1000	Bozic	Inf	unif	wholeT_0.01	OT, OT-A
444	No	11	11-B	1000	Bozic	Inf	unif	wholeT_0.5	OT, OT-A
445	No	11	11-B	1000	exp	0	last	singleC	OT, OT-A
446	No	11	11-B	1000	exp	0	last	wholeT_0.01	DiP-A, OT, OT-A
447	No	11	11-B	1000	exp	0	last	wholeT_0.5	OT, OT-A
448	No	11	11-B	1000	exp	0	unif	singleC	OT, OT-A
449	No	11	11-B	1000	exp	0	unif	wholeT_0.01	DiP-A, OT, OT-A
450	No	11	11-B	1000	exp	0	unif	wholeT_0.5	OT, OT-A
451	No	11	11-B	1000	exp	Inf	last	singleC	OT, OT-A
452	No	11	11-B	1000	exp	Inf	last	wholeT_0.01	OT, OT-A
453	No	11	11-B	1000	exp	Inf	last	wholeT_0.5	OT, OT-A
454	No	11	11-B	1000	exp	Inf	unif	singleC	OT, OT-A
455	No	11	11-B	1000	exp	Inf	unif	wholeT_0.01	OT, OT-A
456	No	11	11-B	1000	exp	Inf	unif	wholeT_0.5	OT, OT-A
457	No	11	11-B	1000	McF_4	0	last	singleC	DiP, DiP-A, OT, OT-A
458	No	11	11-B	1000	McF_4	0	last	wholeT_0.01	DiP-A, OT, OT-A
459	No	11	11-B	1000	McF_4	0	last	wholeT_0.5	DiP-A, OT, OT-A
460	No	11	11-B	1000	McF_4	0	unif	singleC	DiP, DiP-A
461	No	11	11-B	1000	McF_4	0	unif	wholeT_0.01	DiP, DiP-A, OT, OT-A
462	No	11	11-B	1000	McF_4	0	unif	wholeT_0.5	DiP, DiP-A, OT
463	No	11	11-B	1000	McF_4	Inf	last	singleC	OT, OT-A
464	No	11	11-B	1000	McF_4	Inf	last	wholeT_0.01	DiP, OT, OT-A
465	No	11	11-B	1000	McF_4	Inf	last	wholeT_0.5	DiP-A, OT, OT-A
466	No	11	11-B	1000	McF_4	Inf	unif	singleC	DiP, DiP-A, OT, OT-A
467	No	11	11-B	1000	McF_4	Inf	unif	wholeT_0.01	OT, OT-A
468	No	11	11-B	1000	McF_4	Inf	unif	wholeT_0.5	DiP, DiP-A, OT, OT-A
469	No	11	11-B	1000	McF_6	0	last	singleC	DiP, OT
470	No	11	11-B	1000	McF_6	0	last	wholeT_0.01	DiP, OT
471	No	11	11-B	1000	McF_6	0	last	wholeT_0.5	DiP, OT
472	No	11	11-B	1000	McF_6	0	unif	singleC	DiP, DiP-A, OT, OT-A
473	No	11	11-B	1000	McF_6	0	unif	wholeT_0.01	DiP, DiP-A, OT, OT-A
474	No	11	11-B	1000	McF_6	0	unif	wholeT_0.5	DiP, DiP-A
475	No	11	11-B	1000	McF_6	Inf	last	singleC	DiP, OT
476	No	11	11-B	1000	McF_6	Inf	last	wholeT_0.01	DiP, OT
477	No	11	11-B	1000	McF_6	Inf	last	wholeT_0.5	DiP, OT

Table 10: *(continued)*

	Conjunction	Drivers	Tree	S.Size	Model	sh	S.Time	S.Type	Best method(s)
478	No	11	11-B	1000	McF_6	Inf	unif	singleC	DiP, DiP-A
479	No	11	11-B	1000	McF_6	Inf	unif	wholeT_0.01	DiP, DiP-A
480	No	11	11-B	1000	McF_6	Inf	unif	wholeT_0.5	DiP, DiP-A, OT, OT-A
481	No	11	11-B	200	Bozic	0	last	singleC	OT, OT-A
482	No	11	11-B	200	Bozic	0	last	wholeT_0.01	OT, OT-A
483	No	11	11-B	200	Bozic	0	last	wholeT_0.5	OT, OT-A
484	No	11	11-B	200	Bozic	0	unif	singleC	OT, OT-A
485	No	11	11-B	200	Bozic	0	unif	wholeT_0.01	DiP, DiP-A, OT, OT-A
486	No	11	11-B	200	Bozic	0	unif	wholeT_0.5	OT, OT-A
487	No	11	11-B	200	Bozic	Inf	last	singleC	OT
488	No	11	11-B	200	Bozic	Inf	last	wholeT_0.01	OT, OT-A
489	No	11	11-B	200	Bozic	Inf	last	wholeT_0.5	OT, OT-A
490	No	11	11-B	200	Bozic	Inf	unif	singleC	OT, OT-A
491	No	11	11-B	200	Bozic	Inf	unif	wholeT_0.01	OT, OT-A
492	No	11	11-B	200	Bozic	Inf	unif	wholeT_0.5	OT, OT-A
493	No	11	11-B	200	exp	0	last	singleC	OT, OT-A
494	No	11	11-B	200	exp	0	last	wholeT_0.01	OT, OT-A
495	No	11	11-B	200	exp	0	last	wholeT_0.5	OT, OT-A
496	No	11	11-B	200	exp	0	unif	singleC	CBN, CBN-A
497	No	11	11-B	200	exp	0	unif	wholeT_0.01	OT, OT-A
498	No	11	11-B	200	exp	0	unif	wholeT_0.5	CBN-A
499	No	11	11-B	200	exp	Inf	last	singleC	OT, OT-A
500	No	11	11-B	200	exp	Inf	last	wholeT_0.01	OT, OT-A
501	No	11	11-B	200	exp	Inf	last	wholeT_0.5	OT, OT-A
502	No	11	11-B	200	exp	Inf	unif	singleC	OT, OT-A
503	No	11	11-B	200	exp	Inf	unif	wholeT_0.01	OT, OT-A
504	No	11	11-B	200	exp	Inf	unif	wholeT_0.5	OT, OT-A
505	No	11	11-B	200	McF_4	0	last	singleC	DiP, OT, OT-A
506	No	11	11-B	200	McF_4	0	last	wholeT_0.01	DiP, DiP-A, OT, OT-A
507	No	11	11-B	200	McF_4	0	last	wholeT_0.5	DiP-A, OT, OT-A
508	No	11	11-B	200	McF_4	0	unif	singleC	DiP, DiP-A, OT, OT-A
509	No	11	11-B	200	McF_4	0	unif	wholeT_0.01	DiP, DiP-A
510	No	11	11-B	200	McF_4	0	unif	wholeT_0.5	OT, OT-A
511	No	11	11-B	200	McF_4	Inf	last	singleC	OT, OT-A
512	No	11	11-B	200	McF_4	Inf	last	wholeT_0.01	DiP-A, OT, OT-A
513	No	11	11-B	200	McF_4	Inf	last	wholeT_0.5	OT, OT-A
514	No	11	11-B	200	McF_4	Inf	unif	singleC	OT, OT-A
515	No	11	11-B	200	McF_4	Inf	unif	wholeT_0.01	OT, OT-A
516	No	11	11-B	200	McF_4	Inf	unif	wholeT_0.5	OT, OT-A
517	No	11	11-B	200	McF_6	0	last	singleC	DiP, OT

Table 10: *(continued)*

	Conjunction	Drivers	Tree	S.Size	Model	sh	S.Time	S.Type	Best method(s)
518	No	11	11-B	200	McF_6	0	last	wholeT_0.01	DiP, OT
519	No	11	11-B	200	McF_6	0	last	wholeT_0.5	DiP, OT
520	No	11	11-B	200	McF_6	0	unif	singleC	OT, OT-A
521	No	11	11-B	200	McF_6	0	unif	wholeT_0.01	DiP, DiP-A, OT, OT-A
522	No	11	11-B	200	McF_6	0	unif	wholeT_0.5	OT, OT-A
523	No	11	11-B	200	McF_6	Inf	last	singleC	OT
524	No	11	11-B	200	McF_6	Inf	last	wholeT_0.01	DiP, OT
525	No	11	11-B	200	McF_6	Inf	last	wholeT_0.5	OT
526	No	11	11-B	200	McF_6	Inf	unif	singleC	OT, OT-A
527	No	11	11-B	200	McF_6	Inf	unif	wholeT_0.01	OT, OT-A
528	No	11	11-B	200	McF_6	Inf	unif	wholeT_0.5	OT, OT-A
529	No	11	11-B	100	Bozic	0	last	singleC	OT, OT-A
530	No	11	11-B	100	Bozic	0	last	wholeT_0.01	OT, OT-A
531	No	11	11-B	100	Bozic	0	last	wholeT_0.5	OT, OT-A
532	No	11	11-B	100	Bozic	0	unif	singleC	OT, OT-A
533	No	11	11-B	100	Bozic	0	unif	wholeT_0.01	DiP, DiP-A, OT, OT-A
534	No	11	11-B	100	Bozic	0	unif	wholeT_0.5	OT, OT-A
535	No	11	11-B	100	Bozic	Inf	last	singleC	OT, OT-A
536	No	11	11-B	100	Bozic	Inf	last	wholeT_0.01	OT, OT-A
537	No	11	11-B	100	Bozic	Inf	last	wholeT_0.5	OT, OT-A
538	No	11	11-B	100	Bozic	Inf	unif	singleC	OT, OT-A
539	No	11	11-B	100	Bozic	Inf	unif	wholeT_0.01	OT, OT-A
540	No	11	11-B	100	Bozic	Inf	unif	wholeT_0.5	OT-A
541	No	11	11-B	100	exp	0	last	singleC	OT, OT-A
542	No	11	11-B	100	exp	0	last	wholeT_0.01	OT-A
543	No	11	11-B	100	exp	0	last	wholeT_0.5	OT, OT-A
544	No	11	11-B	100	exp	0	unif	singleC	CBN, CBN-A
545	No	11	11-B	100	exp	0	unif	wholeT_0.01	OT, OT-A
546	No	11	11-B	100	exp	0	unif	wholeT_0.5	CBN, CBN-A
547	No	11	11-B	100	exp	Inf	last	singleC	OT, OT-A
548	No	11	11-B	100	exp	Inf	last	wholeT_0.01	OT, OT-A
549	No	11	11-B	100	exp	Inf	last	wholeT_0.5	OT, OT-A
550	No	11	11-B	100	exp	Inf	unif	singleC	OT-A
551	No	11	11-B	100	exp	Inf	unif	wholeT_0.01	OT, OT-A
552	No	11	11-B	100	exp	Inf	unif	wholeT_0.5	OT, OT-A
553	No	11	11-B	100	McF_4	0	last	singleC	OT, OT-A
554	No	11	11-B	100	McF_4	0	last	wholeT_0.01	DiP, DiP-A, OT, OT-A
555	No	11	11-B	100	McF_4	0	last	wholeT_0.5	OT, OT-A
556	No	11	11-B	100	McF_4	0	unif	singleC	OT, OT-A
557	No	11	11-B	100	McF_4	0	unif	wholeT_0.01	DiP, DiP-A

Table 10: *(continued)*

	Conjunction	Drivers	Tree	S.Size	Model	sh	S.Time	S.Type	Best method(s)
558	No	11	11-B	100	McF_4	0	unif	wholeT_0.5	OT, OT-A
559	No	11	11-B	100	McF_4	Inf	last	singleC	OT, OT-A
560	No	11	11-B	100	McF_4	Inf	last	wholeT_0.01	OT, OT-A
561	No	11	11-B	100	McF_4	Inf	last	wholeT_0.5	OT, OT-A
562	No	11	11-B	100	McF_4	Inf	unif	singleC	OT, OT-A
563	No	11	11-B	100	McF_4	Inf	unif	wholeT_0.01	OT, OT-A
564	No	11	11-B	100	McF_4	Inf	unif	wholeT_0.5	OT, OT-A
565	No	11	11-B	100	McF_6	0	last	singleC	OT
566	No	11	11-B	100	McF_6	0	last	wholeT_0.01	DiP, OT
567	No	11	11-B	100	McF_6	0	last	wholeT_0.5	OT
568	No	11	11-B	100	McF_6	0	unif	singleC	OT, OT-A
569	No	11	11-B	100	McF_6	0	unif	wholeT_0.01	DiP, DiP-A, OT, OT-A
570	No	11	11-B	100	McF_6	0	unif	wholeT_0.5	OT, OT-A
571	No	11	11-B	100	McF_6	Inf	last	singleC	OT
572	No	11	11-B	100	McF_6	Inf	last	wholeT_0.01	OT
573	No	11	11-B	100	McF_6	Inf	last	wholeT_0.5	OT
574	No	11	11-B	100	McF_6	Inf	unif	singleC	OT, OT-A
575	No	11	11-B	100	McF_6	Inf	unif	wholeT_0.01	OT, OT-A
576	No	11	11-B	100	McF_6	Inf	unif	wholeT_0.5	OT, OT-A
577	No	9	9-B	1000	Bozic	0	last	singleC	OT, OT-A
578	No	9	9-B	1000	Bozic	0	last	wholeT_0.01	DiP, DiP-A, OT, OT-A
579	No	9	9-B	1000	Bozic	0	last	wholeT_0.5	OT, OT-A
580	No	9	9-B	1000	Bozic	0	unif	singleC	OT, OT-A
581	No	9	9-B	1000	Bozic	0	unif	wholeT_0.01	DiP, DiP-A, OT, OT-A
582	No	9	9-B	1000	Bozic	0	unif	wholeT_0.5	OT, OT-A
583	No	9	9-B	1000	Bozic	Inf	last	singleC	OT, OT-A
584	No	9	9-B	1000	Bozic	Inf	last	wholeT_0.01	DiP-A
585	No	9	9-B	1000	Bozic	Inf	last	wholeT_0.5	OT, OT-A
586	No	9	9-B	1000	Bozic	Inf	unif	singleC	DiP, DiP-A, OT, OT-A
587	No	9	9-B	1000	Bozic	Inf	unif	wholeT_0.01	OT, OT-A
588	No	9	9-B	1000	Bozic	Inf	unif	wholeT_0.5	DiP-A, OT, OT-A
589	No	9	9-B	1000	exp	0	last	singleC	OT, OT-A
590	No	9	9-B	1000	exp	0	last	wholeT_0.01	DiP-A, OT, OT-A
591	No	9	9-B	1000	exp	0	last	wholeT_0.5	OT, OT-A
592	No	9	9-B	1000	exp	0	unif	singleC	OT, OT-A
593	No	9	9-B	1000	exp	0	unif	wholeT_0.01	DiP, DiP-A, OT, OT-A
594	No	9	9-B	1000	exp	0	unif	wholeT_0.5	OT, OT-A
595	No	9	9-B	1000	exp	Inf	last	singleC	DiP, DiP-A, OT, OT-A
596	No	9	9-B	1000	exp	Inf	last	wholeT_0.01	CBN-A, DiP, DiP-A, OT, OT-A
597	No	9	9-B	1000	exp	Inf	last	wholeT_0.5	DiP-A, OT, OT-A

Table 10: *(continued)*

	Conjunction	Drivers	Tree	S.Size	Model	sh	S.Time	S.Type	Best method(s)
598	No	9	9-B	1000	exp	Inf	unif	singleC	OT, OT-A
599	No	9	9-B	1000	exp	Inf	unif	wholeT_0.01	OT, OT-A
600	No	9	9-B	1000	exp	Inf	unif	wholeT_0.5	OT, OT-A
601	No	9	9-B	1000	McF_4	0	last	singleC	OT, OT-A
602	No	9	9-B	1000	McF_4	0	last	wholeT_0.01	DiP, DiP-A, OT, OT-A
603	No	9	9-B	1000	McF_4	0	last	wholeT_0.5	DiP-A, OT, OT-A
604	No	9	9-B	1000	McF_4	0	unif	singleC	DiP, DiP-A, OT, OT-A
605	No	9	9-B	1000	McF_4	0	unif	wholeT_0.01	DiP, DiP-A, OT, OT-A
606	No	9	9-B	1000	McF_4	0	unif	wholeT_0.5	DiP, DiP-A, OT, OT-A
607	No	9	9-B	1000	McF_4	Inf	last	singleC	OT, OT-A
608	No	9	9-B	1000	McF_4	Inf	last	wholeT_0.01	DiP, DiP-A, OT, OT-A
609	No	9	9-B	1000	McF_4	Inf	last	wholeT_0.5	DiP, OT, OT-A
610	No	9	9-B	1000	McF_4	Inf	unif	singleC	DiP, DiP-A, OT, OT-A
611	No	9	9-B	1000	McF_4	Inf	unif	wholeT_0.01	DiP-A, OT, OT-A
612	No	9	9-B	1000	McF_4	Inf	unif	wholeT_0.5	DiP, DiP-A, OT, OT-A
613	No	9	9-B	1000	McF_6	0	last	singleC	DiP, OT
614	No	9	9-B	1000	McF_6	0	last	wholeT_0.01	DiP, OT
615	No	9	9-B	1000	McF_6	0	last	wholeT_0.5	DiP, OT
616	No	9	9-B	1000	McF_6	0	unif	singleC	DiP, DiP-A, OT, OT-A
617	No	9	9-B	1000	McF_6	0	unif	wholeT_0.01	DiP, DiP-A, OT, OT-A
618	No	9	9-B	1000	McF_6	0	unif	wholeT_0.5	DiP, DiP-A, OT, OT-A
619	No	9	9-B	1000	McF_6	Inf	last	singleC	DiP, OT
620	No	9	9-B	1000	McF_6	Inf	last	wholeT_0.01	DiP, OT
621	No	9	9-B	1000	McF_6	Inf	last	wholeT_0.5	DiP, OT
622	No	9	9-B	1000	McF_6	Inf	unif	singleC	DiP, DiP-A, OT
623	No	9	9-B	1000	McF_6	Inf	unif	wholeT_0.01	DiP, DiP-A, OT, OT-A
624	No	9	9-B	1000	McF_6	Inf	unif	wholeT_0.5	DiP, DiP-A, OT, OT-A
625	No	9	9-B	200	Bozic	0	last	singleC	OT, OT-A
626	No	9	9-B	200	Bozic	0	last	wholeT_0.01	OT, OT-A
627	No	9	9-B	200	Bozic	0	last	wholeT_0.5	OT, OT-A
628	No	9	9-B	200	Bozic	0	unif	singleC	OT, OT-A
629	No	9	9-B	200	Bozic	0	unif	wholeT_0.01	DiP, DiP-A, OT, OT-A
630	No	9	9-B	200	Bozic	0	unif	wholeT_0.5	CBN, CBN-A
631	No	9	9-B	200	Bozic	Inf	last	singleC	OT, OT-A
632	No	9	9-B	200	Bozic	Inf	last	wholeT_0.01	OT, OT-A
633	No	9	9-B	200	Bozic	Inf	last	wholeT_0.5	OT, OT-A
634	No	9	9-B	200	Bozic	Inf	unif	singleC	OT, OT-A
635	No	9	9-B	200	Bozic	Inf	unif	wholeT_0.01	OT, OT-A
636	No	9	9-B	200	Bozic	Inf	unif	wholeT_0.5	OT, OT-A
637	No	9	9-B	200	exp	0	last	singleC	OT, OT-A

Table 10: *(continued)*

	Conjunction	Drivers	Tree	S.Size	Model	sh	S.Time	S.Type	Best method(s)
638	No	9	9-B	200	exp	0	last	wholeT_0.01	OT, OT-A
639	No	9	9-B	200	exp	0	last	wholeT_0.5	OT, OT-A
640	No	9	9-B	200	exp	0	unif	singleC	CBN, CBN-A
641	No	9	9-B	200	exp	0	unif	wholeT_0.01	OT, OT-A
642	No	9	9-B	200	exp	0	unif	wholeT_0.5	CBN, CBN-A
643	No	9	9-B	200	exp	Inf	last	singleC	OT, OT-A
644	No	9	9-B	200	exp	Inf	last	wholeT_0.01	OT, OT-A
645	No	9	9-B	200	exp	Inf	last	wholeT_0.5	OT, OT-A
646	No	9	9-B	200	exp	Inf	unif	singleC	OT, OT-A
647	No	9	9-B	200	exp	Inf	unif	wholeT_0.01	OT, OT-A
648	No	9	9-B	200	exp	Inf	unif	wholeT_0.5	OT, OT-A
649	No	9	9-B	200	McF_4	0	last	singleC	OT, OT-A
650	No	9	9-B	200	McF_4	0	last	wholeT_0.01	DiP, DiP-A, OT, OT-A
651	No	9	9-B	200	McF_4	0	last	wholeT_0.5	DiP-A, OT, OT-A
652	No	9	9-B	200	McF_4	0	unif	singleC	OT, OT-A
653	No	9	9-B	200	McF_4	0	unif	wholeT_0.01	DiP, DiP-A, OT, OT-A
654	No	9	9-B	200	McF_4	0	unif	wholeT_0.5	OT, OT-A
655	No	9	9-B	200	McF_4	Inf	last	singleC	OT, OT-A
656	No	9	9-B	200	McF_4	Inf	last	wholeT_0.01	OT, OT-A
657	No	9	9-B	200	McF_4	Inf	last	wholeT_0.5	DiP-A, OT, OT-A
658	No	9	9-B	200	McF_4	Inf	unif	singleC	OT, OT-A
659	No	9	9-B	200	McF_4	Inf	unif	wholeT_0.01	OT, OT-A
660	No	9	9-B	200	McF_4	Inf	unif	wholeT_0.5	OT, OT-A
661	No	9	9-B	200	McF_6	0	last	singleC	DiP, OT
662	No	9	9-B	200	McF_6	0	last	wholeT_0.01	DiP, OT
663	No	9	9-B	200	McF_6	0	last	wholeT_0.5	DiP, OT
664	No	9	9-B	200	McF_6	0	unif	singleC	DiP, DiP-A, OT, OT-A
665	No	9	9-B	200	McF_6	0	unif	wholeT_0.01	DiP, OT
666	No	9	9-B	200	McF_6	0	unif	wholeT_0.5	DiP, OT, OT-A
667	No	9	9-B	200	McF_6	Inf	last	singleC	DiP, OT
668	No	9	9-B	200	McF_6	Inf	last	wholeT_0.01	DiP, OT
669	No	9	9-B	200	McF_6	Inf	last	wholeT_0.5	DiP, OT
670	No	9	9-B	200	McF_6	Inf	unif	singleC	OT, OT-A
671	No	9	9-B	200	McF_6	Inf	unif	wholeT_0.01	OT, OT-A
672	No	9	9-B	200	McF_6	Inf	unif	wholeT_0.5	OT, OT-A
673	No	9	9-B	100	Bozic	0	last	singleC	OT, OT-A
674	No	9	9-B	100	Bozic	0	last	wholeT_0.01	OT, OT-A
675	No	9	9-B	100	Bozic	0	last	wholeT_0.5	OT, OT-A
676	No	9	9-B	100	Bozic	0	unif	singleC	CBN, CBN-A
677	No	9	9-B	100	Bozic	0	unif	wholeT_0.01	OT, OT-A

Table 10: *(continued)*

	Conjunction	Drivers	Tree	S.Size	Model	sh	S.Time	S.Type	Best method(s)
678	No	9	9-B	100	Bozic	0	unif	wholeT_0.5	CBN, CBN-A
679	No	9	9-B	100	Bozic	Inf	last	singleC	OT, OT-A
680	No	9	9-B	100	Bozic	Inf	last	wholeT_0.01	OT, OT-A
681	No	9	9-B	100	Bozic	Inf	last	wholeT_0.5	OT, OT-A
682	No	9	9-B	100	Bozic	Inf	unif	singleC	OT, OT-A
683	No	9	9-B	100	Bozic	Inf	unif	wholeT_0.01	OT, OT-A
684	No	9	9-B	100	Bozic	Inf	unif	wholeT_0.5	OT, OT-A
685	No	9	9-B	100	exp	0	last	singleC	OT, OT-A
686	No	9	9-B	100	exp	0	last	wholeT_0.01	OT, OT-A
687	No	9	9-B	100	exp	0	last	wholeT_0.5	OT, OT-A
688	No	9	9-B	100	exp	0	unif	singleC	CBN, CBN-A
689	No	9	9-B	100	exp	0	unif	wholeT_0.01	OT, OT-A
690	No	9	9-B	100	exp	0	unif	wholeT_0.5	CBN, CBN-A
691	No	9	9-B	100	exp	Inf	last	singleC	OT, OT-A
692	No	9	9-B	100	exp	Inf	last	wholeT_0.01	OT, OT-A
693	No	9	9-B	100	exp	Inf	last	wholeT_0.5	OT, OT-A
694	No	9	9-B	100	exp	Inf	unif	singleC	OT, OT-A
695	No	9	9-B	100	exp	Inf	unif	wholeT_0.01	OT, OT-A
696	No	9	9-B	100	exp	Inf	unif	wholeT_0.5	OT, OT-A
697	No	9	9-B	100	McF_4	0	last	singleC	OT, OT-A
698	No	9	9-B	100	McF_4	0	last	wholeT_0.01	DiP, DiP-A, OT, OT-A
699	No	9	9-B	100	McF_4	0	last	wholeT_0.5	OT, OT-A
700	No	9	9-B	100	McF_4	0	unif	singleC	OT, OT-A
701	No	9	9-B	100	McF_4	0	unif	wholeT_0.01	DiP, DiP-A, OT, OT-A
702	No	9	9-B	100	McF_4	0	unif	wholeT_0.5	OT, OT-A
703	No	9	9-B	100	McF_4	Inf	last	singleC	OT, OT-A
704	No	9	9-B	100	McF_4	Inf	last	wholeT_0.01	OT, OT-A
705	No	9	9-B	100	McF_4	Inf	last	wholeT_0.5	OT, OT-A
706	No	9	9-B	100	McF_4	Inf	unif	singleC	OT, OT-A
707	No	9	9-B	100	McF_4	Inf	unif	wholeT_0.01	OT, OT-A
708	No	9	9-B	100	McF_4	Inf	unif	wholeT_0.5	OT, OT-A
709	No	9	9-B	100	McF_6	0	last	singleC	OT
710	No	9	9-B	100	McF_6	0	last	wholeT_0.01	OT
711	No	9	9-B	100	McF_6	0	last	wholeT_0.5	OT
712	No	9	9-B	100	McF_6	0	unif	singleC	OT, OT-A
713	No	9	9-B	100	McF_6	0	unif	wholeT_0.01	DiP, DiP-A, OT, OT-A
714	No	9	9-B	100	McF_6	0	unif	wholeT_0.5	OT, OT-A
715	No	9	9-B	100	McF_6	Inf	last	singleC	OT
716	No	9	9-B	100	McF_6	Inf	last	wholeT_0.01	OT
717	No	9	9-B	100	McF_6	Inf	last	wholeT_0.5	OT

Table 10: *(continued)*

	Conjunction	Drivers	Tree	S.Size	Model	sh	S.Time	S.Type	Best method(s)
718	No	9	9-B	100	McF_6	Inf	unif	singleC	OT, OT-A
719	No	9	9-B	100	McF_6	Inf	unif	wholeT_0.01	OT, OT-A
720	No	9	9-B	100	McF_6	Inf	unif	wholeT_0.5	OT, OT-A
721	No	7	7-B	1000	Bozic	0	last	singleC	DiP-A, OT, OT-A
722	No	7	7-B	1000	Bozic	0	last	wholeT_0.01	DiP-A, OT, OT-A
723	No	7	7-B	1000	Bozic	0	last	wholeT_0.5	OT, OT-A
724	No	7	7-B	1000	Bozic	0	unif	singleC	DiP, DiP-A, OT, OT-A
725	No	7	7-B	1000	Bozic	0	unif	wholeT_0.01	CBN-A, DiP, DiP-A, OT, OT-A
726	No	7	7-B	1000	Bozic	0	unif	wholeT_0.5	OT, OT-A
727	No	7	7-B	1000	Bozic	Inf	last	singleC	DiP-A, OT, OT-A
728	No	7	7-B	1000	Bozic	Inf	last	wholeT_0.01	none
729	No	7	7-B	1000	Bozic	Inf	last	wholeT_0.5	DiP-A, OT, OT-A
730	No	7	7-B	1000	Bozic	Inf	unif	singleC	CBN, CBN-A
731	No	7	7-B	1000	Bozic	Inf	unif	wholeT_0.01	CBN, CBN-A, OT, OT-A
732	No	7	7-B	1000	Bozic	Inf	unif	wholeT_0.5	CBN, CBN-A
733	No	7	7-B	1000	exp	0	last	singleC	OT-A
734	No	7	7-B	1000	exp	0	last	wholeT_0.01	OT-A
735	No	7	7-B	1000	exp	0	last	wholeT_0.5	OT-A
736	No	7	7-B	1000	exp	0	unif	singleC	OT, OT-A
737	No	7	7-B	1000	exp	0	unif	wholeT_0.01	CBN, CBN-A, OT, OT-A
738	No	7	7-B	1000	exp	0	unif	wholeT_0.5	OT, OT-A
739	No	7	7-B	1000	exp	Inf	last	singleC	OT, OT-A
740	No	7	7-B	1000	exp	Inf	last	wholeT_0.01	CBN-A, OT, OT-A
741	No	7	7-B	1000	exp	Inf	last	wholeT_0.5	OT, OT-A
742	No	7	7-B	1000	exp	Inf	unif	singleC	OT
743	No	7	7-B	1000	exp	Inf	unif	wholeT_0.01	CBN-A, OT, OT-A
744	No	7	7-B	1000	exp	Inf	unif	wholeT_0.5	OT, OT-A
745	No	7	7-B	1000	McF_4	0	last	singleC	DiP, DiP-A, OT, OT-A
746	No	7	7-B	1000	McF_4	0	last	wholeT_0.01	DiP, DiP-A, OT, OT-A
747	No	7	7-B	1000	McF_4	0	last	wholeT_0.5	DiP, DiP-A, OT, OT-A
748	No	7	7-B	1000	McF_4	0	unif	singleC	DiP, DiP-A, OT, OT-A
749	No	7	7-B	1000	McF_4	0	unif	wholeT_0.01	DiP, DiP-A, OT, OT-A
750	No	7	7-B	1000	McF_4	0	unif	wholeT_0.5	DiP, DiP-A, OT, OT-A
751	No	7	7-B	1000	McF_4	Inf	last	singleC	DiP, DiP-A, OT, OT-A
752	No	7	7-B	1000	McF_4	Inf	last	wholeT_0.01	DiP, DiP-A, OT, OT-A
753	No	7	7-B	1000	McF_4	Inf	last	wholeT_0.5	DiP, DiP-A, OT, OT-A
754	No	7	7-B	1000	McF_4	Inf	unif	singleC	DiP-A, OT, OT-A
755	No	7	7-B	1000	McF_4	Inf	unif	wholeT_0.01	DiP, DiP-A, OT, OT-A
756	No	7	7-B	1000	McF_4	Inf	unif	wholeT_0.5	DiP, DiP-A, OT, OT-A
757	No	7	7-B	1000	McF_6	0	last	singleC	DiP, DiP-A, OT, OT-A

Table 10: *(continued)*

	Conjunction	Drivers	Tree	S.Size	Model	sh	S.Time	S.Type	Best method(s)
758	No	7	7-B	1000	McF_6	0	last	wholeT_0.01	DiP, DiP-A, OT, OT-A
759	No	7	7-B	1000	McF_6	0	last	wholeT_0.5	DiP, DiP-A, OT, OT-A
760	No	7	7-B	1000	McF_6	0	unif	singleC	none
761	No	7	7-B	1000	McF_6	0	unif	wholeT_0.01	none
762	No	7	7-B	1000	McF_6	0	unif	wholeT_0.5	CBN, DiP, DiP-A, OT-A
763	No	7	7-B	1000	McF_6	Inf	last	singleC	DiP, DiP-A, OT, OT-A
764	No	7	7-B	1000	McF_6	Inf	last	wholeT_0.01	DiP-A, OT, OT-A
765	No	7	7-B	1000	McF_6	Inf	last	wholeT_0.5	DiP, DiP-A, OT, OT-A
766	No	7	7-B	1000	McF_6	Inf	unif	singleC	CBN, CBN-A, DiP, DiP-A, OT
767	No	7	7-B	1000	McF_6	Inf	unif	wholeT_0.01	none
768	No	7	7-B	1000	McF_6	Inf	unif	wholeT_0.5	none
769	No	7	7-B	200	Bozic	0	last	singleC	OT-A
770	No	7	7-B	200	Bozic	0	last	wholeT_0.01	OT-A
771	No	7	7-B	200	Bozic	0	last	wholeT_0.5	OT-A
772	No	7	7-B	200	Bozic	0	unif	singleC	OT, OT-A
773	No	7	7-B	200	Bozic	0	unif	wholeT_0.01	CBN, CBN-A, OT, OT-A
774	No	7	7-B	200	Bozic	0	unif	wholeT_0.5	OT, OT-A
775	No	7	7-B	200	Bozic	Inf	last	singleC	OT, OT-A
776	No	7	7-B	200	Bozic	Inf	last	wholeT_0.01	CBN, CBN-A, OT-A
777	No	7	7-B	200	Bozic	Inf	last	wholeT_0.5	OT, OT-A
778	No	7	7-B	200	Bozic	Inf	unif	singleC	CBN, CBN-A, OT, OT-A
779	No	7	7-B	200	Bozic	Inf	unif	wholeT_0.01	CBN, CBN-A, OT, OT-A
780	No	7	7-B	200	Bozic	Inf	unif	wholeT_0.5	CBN, CBN-A, OT, OT-A
781	No	7	7-B	200	exp	0	last	singleC	OT-A
782	No	7	7-B	200	exp	0	last	wholeT_0.01	OT-A
783	No	7	7-B	200	exp	0	last	wholeT_0.5	OT-A
784	No	7	7-B	200	exp	0	unif	singleC	OT, OT-A
785	No	7	7-B	200	exp	0	unif	wholeT_0.01	OT, OT-A
786	No	7	7-B	200	exp	0	unif	wholeT_0.5	OT, OT-A
787	No	7	7-B	200	exp	Inf	last	singleC	OT, OT-A
788	No	7	7-B	200	exp	Inf	last	wholeT_0.01	CBN-A, OT-A
789	No	7	7-B	200	exp	Inf	last	wholeT_0.5	OT, OT-A
790	No	7	7-B	200	exp	Inf	unif	singleC	OT, OT-A
791	No	7	7-B	200	exp	Inf	unif	wholeT_0.01	CBN, CBN-A, OT, OT-A
792	No	7	7-B	200	exp	Inf	unif	wholeT_0.5	OT, OT-A
793	No	7	7-B	200	McF_4	0	last	singleC	OT, OT-A
794	No	7	7-B	200	McF_4	0	last	wholeT_0.01	DiP, DiP-A, OT, OT-A
795	No	7	7-B	200	McF_4	0	last	wholeT_0.5	OT, OT-A
796	No	7	7-B	200	McF_4	0	unif	singleC	DiP-A, OT, OT-A
797	No	7	7-B	200	McF_4	0	unif	wholeT_0.01	DiP, DiP-A, OT, OT-A

Table 10: *(continued)*

	Conjunction	Drivers	Tree	S.Size	Model	sh	S.Time	S.Type	Best method(s)
798	No	7	7-B	200	McF_4	0	unif	wholeT_0.5	OT, OT-A
799	No	7	7-B	200	McF_4	Inf	last	singleC	OT, OT-A
800	No	7	7-B	200	McF_4	Inf	last	wholeT_0.01	OT, OT-A
801	No	7	7-B	200	McF_4	Inf	last	wholeT_0.5	OT, OT-A
802	No	7	7-B	200	McF_4	Inf	unif	singleC	OT, OT-A
803	No	7	7-B	200	McF_4	Inf	unif	wholeT_0.01	OT, OT-A
804	No	7	7-B	200	McF_4	Inf	unif	wholeT_0.5	OT, OT-A
805	No	7	7-B	200	McF_6	0	last	singleC	DiP-A, OT, OT-A
806	No	7	7-B	200	McF_6	0	last	wholeT_0.01	DiP, DiP-A, OT, OT-A
807	No	7	7-B	200	McF_6	0	last	wholeT_0.5	DiP-A, OT, OT-A
808	No	7	7-B	200	McF_6	0	unif	singleC	none
809	No	7	7-B	200	McF_6	0	unif	wholeT_0.01	CBN-A, DiP, DiP-A, OT-A
810	No	7	7-B	200	McF_6	0	unif	wholeT_0.5	CBN, CBN-A, DiP, OT, OT-A
811	No	7	7-B	200	McF_6	Inf	last	singleC	DiP-A, OT-A
812	No	7	7-B	200	McF_6	Inf	last	wholeT_0.01	OT, OT-A
813	No	7	7-B	200	McF_6	Inf	last	wholeT_0.5	DiP-A, OT, OT-A
814	No	7	7-B	200	McF_6	Inf	unif	singleC	CBN, CBN-A, OT-A
815	No	7	7-B	200	McF_6	Inf	unif	wholeT_0.01	CBN, CBN-A, OT-A
816	No	7	7-B	200	McF_6	Inf	unif	wholeT_0.5	none
817	No	7	7-B	100	Bozic	0	last	singleC	OT-A
818	No	7	7-B	100	Bozic	0	last	wholeT_0.01	OT-A
819	No	7	7-B	100	Bozic	0	last	wholeT_0.5	OT-A
820	No	7	7-B	100	Bozic	0	unif	singleC	OT, OT-A
821	No	7	7-B	100	Bozic	0	unif	wholeT_0.01	CBN-A, OT, OT-A
822	No	7	7-B	100	Bozic	0	unif	wholeT_0.5	OT, OT-A
823	No	7	7-B	100	Bozic	Inf	last	singleC	OT, OT-A
824	No	7	7-B	100	Bozic	Inf	last	wholeT_0.01	CBN-A, OT-A
825	No	7	7-B	100	Bozic	Inf	last	wholeT_0.5	OT, OT-A
826	No	7	7-B	100	Bozic	Inf	unif	singleC	OT, OT-A
827	No	7	7-B	100	Bozic	Inf	unif	wholeT_0.01	CBN-A, OT, OT-A
828	No	7	7-B	100	Bozic	Inf	unif	wholeT_0.5	CBN-A, OT, OT-A
829	No	7	7-B	100	exp	0	last	singleC	OT-A
830	No	7	7-B	100	exp	0	last	wholeT_0.01	OT-A
831	No	7	7-B	100	exp	0	last	wholeT_0.5	OT-A
832	No	7	7-B	100	exp	0	unif	singleC	OT, OT-A
833	No	7	7-B	100	exp	0	unif	wholeT_0.01	OT, OT-A
834	No	7	7-B	100	exp	0	unif	wholeT_0.5	CBN, CBN-A
835	No	7	7-B	100	exp	Inf	last	singleC	OT, OT-A
836	No	7	7-B	100	exp	Inf	last	wholeT_0.01	OT-A
837	No	7	7-B	100	exp	Inf	last	wholeT_0.5	OT-A

Table 10: *(continued)*

	Conjunction	Drivers	Tree	S.Size	Model	sh	S.Time	S.Type	Best method(s)
838	No	7	7-B	100	exp	Inf	unif	singleC	OT, OT-A
839	No	7	7-B	100	exp	Inf	unif	wholeT_0.01	CBN, CBN-A, OT, OT-A
840	No	7	7-B	100	exp	Inf	unif	wholeT_0.5	OT, OT-A
841	No	7	7-B	100	McF_4	0	last	singleC	OT, OT-A
842	No	7	7-B	100	McF_4	0	last	wholeT_0.01	OT, OT-A
843	No	7	7-B	100	McF_4	0	last	wholeT_0.5	OT, OT-A
844	No	7	7-B	100	McF_4	0	unif	singleC	OT, OT-A
845	No	7	7-B	100	McF_4	0	unif	wholeT_0.01	OT, OT-A
846	No	7	7-B	100	McF_4	0	unif	wholeT_0.5	OT
847	No	7	7-B	100	McF_4	Inf	last	singleC	OT, OT-A
848	No	7	7-B	100	McF_4	Inf	last	wholeT_0.01	OT, OT-A
849	No	7	7-B	100	McF_4	Inf	last	wholeT_0.5	OT, OT-A
850	No	7	7-B	100	McF_4	Inf	unif	singleC	OT, OT-A
851	No	7	7-B	100	McF_4	Inf	unif	wholeT_0.01	OT, OT-A
852	No	7	7-B	100	McF_4	Inf	unif	wholeT_0.5	OT, OT-A
853	No	7	7-B	100	McF_6	0	last	singleC	OT, OT-A
854	No	7	7-B	100	McF_6	0	last	wholeT_0.01	OT, OT-A
855	No	7	7-B	100	McF_6	0	last	wholeT_0.5	OT, OT-A
856	No	7	7-B	100	McF_6	0	unif	singleC	CBN, CBN-A, OT, OT-A
857	No	7	7-B	100	McF_6	0	unif	wholeT_0.01	CBN, CBN-A, DiP, DiP-A
858	No	7	7-B	100	McF_6	0	unif	wholeT_0.5	CBN, CBN-A, OT, OT-A
859	No	7	7-B	100	McF_6	Inf	last	singleC	OT, OT-A
860	No	7	7-B	100	McF_6	Inf	last	wholeT_0.01	OT, OT-A
861	No	7	7-B	100	McF_6	Inf	last	wholeT_0.5	OT, OT-A
862	No	7	7-B	100	McF_6	Inf	unif	singleC	CBN, CBN-A
863	No	7	7-B	100	McF_6	Inf	unif	wholeT_0.01	CBN, CBN-A
864	No	7	7-B	100	McF_6	Inf	unif	wholeT_0.5	CBN, CBN-A

4.3 Confidence sets (MCB), PND, Drivers Known

Table 11: Confidence sets (method MCB) when Drivers are Known for measure PND.

	Conjunction	Drivers	Tree	S.Size	Model	sh	S.Time	S.Type	Best method(s)
1	Yes	11	11-A	1000	Bozic	0	last	singleC	CBN, CBN-A
2	Yes	11	11-A	1000	Bozic	0	last	wholeT_0.01	CBN, CBN-A, OT, OT-A
3	Yes	11	11-A	1000	Bozic	0	last	wholeT_0.5	CBN, CBN-A, OT, OT-A
4	Yes	11	11-A	1000	Bozic	0	unif	singleC	CBN, CBN-A
5	Yes	11	11-A	1000	Bozic	0	unif	wholeT_0.01	CBN, CBN-A
6	Yes	11	11-A	1000	Bozic	0	unif	wholeT_0.5	CBN, CBN-A
7	Yes	11	11-A	1000	Bozic	Inf	last	singleC	CBN, CBN-A
8	Yes	11	11-A	1000	Bozic	Inf	last	wholeT_0.01	CBN, CBN-A
9	Yes	11	11-A	1000	Bozic	Inf	last	wholeT_0.5	CBN, CBN-A
10	Yes	11	11-A	1000	Bozic	Inf	unif	singleC	CBN, CBN-A
11	Yes	11	11-A	1000	Bozic	Inf	unif	wholeT_0.01	CBN, CBN-A
12	Yes	11	11-A	1000	Bozic	Inf	unif	wholeT_0.5	CBN, CBN-A
13	Yes	11	11-A	1000	exp	0	last	singleC	CBN, CBN-A
14	Yes	11	11-A	1000	exp	0	last	wholeT_0.01	CBN, CBN-A
15	Yes	11	11-A	1000	exp	0	last	wholeT_0.5	CBN, CBN-A
16	Yes	11	11-A	1000	exp	0	unif	singleC	CBN, CBN-A
17	Yes	11	11-A	1000	exp	0	unif	wholeT_0.01	CBN, CBN-A
18	Yes	11	11-A	1000	exp	0	unif	wholeT_0.5	CBN, CBN-A
19	Yes	11	11-A	1000	exp	Inf	last	singleC	CBN, CBN-A
20	Yes	11	11-A	1000	exp	Inf	last	wholeT_0.01	CBN, CBN-A
21	Yes	11	11-A	1000	exp	Inf	last	wholeT_0.5	CBN, CBN-A
22	Yes	11	11-A	1000	exp	Inf	unif	singleC	CBN, CBN-A
23	Yes	11	11-A	1000	exp	Inf	unif	wholeT_0.01	CBN
24	Yes	11	11-A	1000	exp	Inf	unif	wholeT_0.5	CBN, CBN-A
25	Yes	11	11-A	1000	McF_4	0	last	singleC	CBN-A, OT, OT-A
26	Yes	11	11-A	1000	McF_4	0	last	wholeT_0.01	OT, OT-A
27	Yes	11	11-A	1000	McF_4	0	last	wholeT_0.5	CBN, CBN-A, OT, OT-A
28	Yes	11	11-A	1000	McF_4	0	unif	singleC	CBN, CBN-A
29	Yes	11	11-A	1000	McF_4	0	unif	wholeT_0.01	CBN, CBN-A
30	Yes	11	11-A	1000	McF_4	0	unif	wholeT_0.5	CBN, CBN-A
31	Yes	11	11-A	1000	McF_4	Inf	last	singleC	CBN, CBN-A, OT, OT-A
32	Yes	11	11-A	1000	McF_4	Inf	last	wholeT_0.01	CBN, CBN-A, DiP-A, OT, OT-A
33	Yes	11	11-A	1000	McF_4	Inf	last	wholeT_0.5	CBN, CBN-A
34	Yes	11	11-A	1000	McF_4	Inf	unif	singleC	CBN, CBN-A
35	Yes	11	11-A	1000	McF_4	Inf	unif	wholeT_0.01	CBN, CBN-A
36	Yes	11	11-A	1000	McF_4	Inf	unif	wholeT_0.5	CBN, CBN-A
37	Yes	11	11-A	1000	McF_6	0	last	singleC	DiP, DiP-A

Table 11: *(continued)*

	Conjunction	Drivers	Tree	S.Size	Model	sh	S.Time	S.Type	Best method(s)
38	Yes	11	11-A	1000	McF_6	0	last	wholeT_0.01	DiP, DiP-A
39	Yes	11	11-A	1000	McF_6	0	last	wholeT_0.5	DiP, DiP-A
40	Yes	11	11-A	1000	McF_6	0	unif	singleC	none
41	Yes	11	11-A	1000	McF_6	0	unif	wholeT_0.01	CBN, CBN-A, DiP, DiP-A, OT-A
42	Yes	11	11-A	1000	McF_6	0	unif	wholeT_0.5	none
43	Yes	11	11-A	1000	McF_6	Inf	last	singleC	DiP-A, OT, OT-A
44	Yes	11	11-A	1000	McF_6	Inf	last	wholeT_0.01	DiP-A
45	Yes	11	11-A	1000	McF_6	Inf	last	wholeT_0.5	DiP-A, OT, OT-A
46	Yes	11	11-A	1000	McF_6	Inf	unif	singleC	CBN, OT, OT-A
47	Yes	11	11-A	1000	McF_6	Inf	unif	wholeT_0.01	CBN, CBN-A, OT, OT-A
48	Yes	11	11-A	1000	McF_6	Inf	unif	wholeT_0.5	CBN, CBN-A, OT, OT-A
49	Yes	11	11-A	200	Bozic	0	last	singleC	CBN, CBN-A
50	Yes	11	11-A	200	Bozic	0	last	wholeT_0.01	CBN, CBN-A, OT, OT-A
51	Yes	11	11-A	200	Bozic	0	last	wholeT_0.5	CBN, CBN-A
52	Yes	11	11-A	200	Bozic	0	unif	singleC	CBN, CBN-A
53	Yes	11	11-A	200	Bozic	0	unif	wholeT_0.01	CBN, CBN-A
54	Yes	11	11-A	200	Bozic	0	unif	wholeT_0.5	CBN, CBN-A
55	Yes	11	11-A	200	Bozic	Inf	last	singleC	CBN, CBN-A
56	Yes	11	11-A	200	Bozic	Inf	last	wholeT_0.01	CBN, CBN-A
57	Yes	11	11-A	200	Bozic	Inf	last	wholeT_0.5	CBN, CBN-A
58	Yes	11	11-A	200	Bozic	Inf	unif	singleC	CBN, CBN-A
59	Yes	11	11-A	200	Bozic	Inf	unif	wholeT_0.01	CBN, CBN-A
60	Yes	11	11-A	200	Bozic	Inf	unif	wholeT_0.5	CBN, CBN-A
61	Yes	11	11-A	200	exp	0	last	singleC	CBN, CBN-A
62	Yes	11	11-A	200	exp	0	last	wholeT_0.01	CBN, CBN-A
63	Yes	11	11-A	200	exp	0	last	wholeT_0.5	CBN, CBN-A
64	Yes	11	11-A	200	exp	0	unif	singleC	CBN, CBN-A
65	Yes	11	11-A	200	exp	0	unif	wholeT_0.01	CBN, CBN-A
66	Yes	11	11-A	200	exp	0	unif	wholeT_0.5	CBN, CBN-A
67	Yes	11	11-A	200	exp	Inf	last	singleC	CBN, CBN-A
68	Yes	11	11-A	200	exp	Inf	last	wholeT_0.01	CBN-A
69	Yes	11	11-A	200	exp	Inf	last	wholeT_0.5	CBN, CBN-A
70	Yes	11	11-A	200	exp	Inf	unif	singleC	CBN, CBN-A
71	Yes	11	11-A	200	exp	Inf	unif	wholeT_0.01	CBN, CBN-A
72	Yes	11	11-A	200	exp	Inf	unif	wholeT_0.5	CBN, CBN-A
73	Yes	11	11-A	200	McF_4	0	last	singleC	CBN, CBN-A, OT, OT-A
74	Yes	11	11-A	200	McF_4	0	last	wholeT_0.01	CBN-A, OT, OT-A
75	Yes	11	11-A	200	McF_4	0	last	wholeT_0.5	CBN, CBN-A
76	Yes	11	11-A	200	McF_4	0	unif	singleC	CBN, CBN-A
77	Yes	11	11-A	200	McF_4	0	unif	wholeT_0.01	CBN, CBN-A

Table 11: *(continued)*

	Conjunction	Drivers	Tree	S.Size	Model	sh	S.Time	S.Type	Best method(s)
78	Yes	11	11-A	200	McF_4	0	unif	wholeT_0.5	CBN, CBN-A
79	Yes	11	11-A	200	McF_4	Inf	last	singleC	CBN, CBN-A
80	Yes	11	11-A	200	McF_4	Inf	last	wholeT_0.01	CBN, CBN-A, OT, OT-A
81	Yes	11	11-A	200	McF_4	Inf	last	wholeT_0.5	CBN, CBN-A, OT, OT-A
82	Yes	11	11-A	200	McF_4	Inf	unif	singleC	CBN, CBN-A
83	Yes	11	11-A	200	McF_4	Inf	unif	wholeT_0.01	CBN, CBN-A
84	Yes	11	11-A	200	McF_4	Inf	unif	wholeT_0.5	CBN, CBN-A
85	Yes	11	11-A	200	McF_6	0	last	singleC	OT, OT-A
86	Yes	11	11-A	200	McF_6	0	last	wholeT_0.01	OT, OT-A
87	Yes	11	11-A	200	McF_6	0	last	wholeT_0.5	OT, OT-A
88	Yes	11	11-A	200	McF_6	0	unif	singleC	CBN-A, OT, OT-A
89	Yes	11	11-A	200	McF_6	0	unif	wholeT_0.01	CBN, CBN-A, OT, OT-A
90	Yes	11	11-A	200	McF_6	0	unif	wholeT_0.5	CBN, CBN-A, OT, OT-A
91	Yes	11	11-A	200	McF_6	Inf	last	singleC	OT, OT-A
92	Yes	11	11-A	200	McF_6	Inf	last	wholeT_0.01	OT, OT-A
93	Yes	11	11-A	200	McF_6	Inf	last	wholeT_0.5	OT, OT-A
94	Yes	11	11-A	200	McF_6	Inf	unif	singleC	CBN, CBN-A, OT, OT-A
95	Yes	11	11-A	200	McF_6	Inf	unif	wholeT_0.01	CBN, CBN-A, OT, OT-A
96	Yes	11	11-A	200	McF_6	Inf	unif	wholeT_0.5	CBN, CBN-A, OT, OT-A
97	Yes	11	11-A	100	Bozic	0	last	singleC	CBN, CBN-A
98	Yes	11	11-A	100	Bozic	0	last	wholeT_0.01	CBN, CBN-A
99	Yes	11	11-A	100	Bozic	0	last	wholeT_0.5	CBN, CBN-A
100	Yes	11	11-A	100	Bozic	0	unif	singleC	CBN, CBN-A
101	Yes	11	11-A	100	Bozic	0	unif	wholeT_0.01	CBN, CBN-A
102	Yes	11	11-A	100	Bozic	0	unif	wholeT_0.5	CBN, CBN-A
103	Yes	11	11-A	100	Bozic	Inf	last	singleC	CBN, CBN-A
104	Yes	11	11-A	100	Bozic	Inf	last	wholeT_0.01	CBN, CBN-A
105	Yes	11	11-A	100	Bozic	Inf	last	wholeT_0.5	CBN, CBN-A
106	Yes	11	11-A	100	Bozic	Inf	unif	singleC	CBN, CBN-A
107	Yes	11	11-A	100	Bozic	Inf	unif	wholeT_0.01	CBN, CBN-A
108	Yes	11	11-A	100	Bozic	Inf	unif	wholeT_0.5	CBN, CBN-A
109	Yes	11	11-A	100	exp	0	last	singleC	CBN, CBN-A
110	Yes	11	11-A	100	exp	0	last	wholeT_0.01	CBN, CBN-A
111	Yes	11	11-A	100	exp	0	last	wholeT_0.5	CBN, CBN-A
112	Yes	11	11-A	100	exp	0	unif	singleC	CBN, CBN-A
113	Yes	11	11-A	100	exp	0	unif	wholeT_0.01	CBN, CBN-A
114	Yes	11	11-A	100	exp	0	unif	wholeT_0.5	CBN, CBN-A
115	Yes	11	11-A	100	exp	Inf	last	singleC	CBN, CBN-A
116	Yes	11	11-A	100	exp	Inf	last	wholeT_0.01	CBN, CBN-A
117	Yes	11	11-A	100	exp	Inf	last	wholeT_0.5	CBN, CBN-A

Table 11: *(continued)*

	Conjunction	Drivers	Tree	S.Size	Model	sh	S.Time	S.Type	Best method(s)
118	Yes	11	11-A	100	exp	Inf	unif	singleC	CBN, CBN-A
119	Yes	11	11-A	100	exp	Inf	unif	wholeT_0.01	CBN, CBN-A
120	Yes	11	11-A	100	exp	Inf	unif	wholeT_0.5	CBN, CBN-A
121	Yes	11	11-A	100	McF_4	0	last	singleC	CBN, CBN-A
122	Yes	11	11-A	100	McF_4	0	last	wholeT_0.01	OT, OT-A
123	Yes	11	11-A	100	McF_4	0	last	wholeT_0.5	CBN, CBN-A, OT
124	Yes	11	11-A	100	McF_4	0	unif	singleC	CBN, CBN-A
125	Yes	11	11-A	100	McF_4	0	unif	wholeT_0.01	CBN
126	Yes	11	11-A	100	McF_4	0	unif	wholeT_0.5	CBN, CBN-A
127	Yes	11	11-A	100	McF_4	Inf	last	singleC	CBN, CBN-A, OT, OT-A
128	Yes	11	11-A	100	McF_4	Inf	last	wholeT_0.01	CBN, CBN-A, OT, OT-A
129	Yes	11	11-A	100	McF_4	Inf	last	wholeT_0.5	CBN, CBN-A
130	Yes	11	11-A	100	McF_4	Inf	unif	singleC	CBN, CBN-A
131	Yes	11	11-A	100	McF_4	Inf	unif	wholeT_0.01	CBN, CBN-A
132	Yes	11	11-A	100	McF_4	Inf	unif	wholeT_0.5	CBN, CBN-A
133	Yes	11	11-A	100	McF_6	0	last	singleC	OT, OT-A
134	Yes	11	11-A	100	McF_6	0	last	wholeT_0.01	OT, OT-A
135	Yes	11	11-A	100	McF_6	0	last	wholeT_0.5	OT, OT-A
136	Yes	11	11-A	100	McF_6	0	unif	singleC	CBN, CBN-A, OT, OT-A
137	Yes	11	11-A	100	McF_6	0	unif	wholeT_0.01	CBN, CBN-A, OT, OT-A
138	Yes	11	11-A	100	McF_6	0	unif	wholeT_0.5	CBN, CBN-A, OT, OT-A
139	Yes	11	11-A	100	McF_6	Inf	last	singleC	OT, OT-A
140	Yes	11	11-A	100	McF_6	Inf	last	wholeT_0.01	OT, OT-A
141	Yes	11	11-A	100	McF_6	Inf	last	wholeT_0.5	OT, OT-A
142	Yes	11	11-A	100	McF_6	Inf	unif	singleC	CBN, CBN-A
143	Yes	11	11-A	100	McF_6	Inf	unif	wholeT_0.01	CBN, CBN-A, OT, OT-A
144	Yes	11	11-A	100	McF_6	Inf	unif	wholeT_0.5	CBN-A, OT, OT-A
145	Yes	9	9-A	1000	Bozic	0	last	singleC	CBN, CBN-A
146	Yes	9	9-A	1000	Bozic	0	last	wholeT_0.01	CBN, CBN-A
147	Yes	9	9-A	1000	Bozic	0	last	wholeT_0.5	CBN, CBN-A
148	Yes	9	9-A	1000	Bozic	0	unif	singleC	CBN, CBN-A
149	Yes	9	9-A	1000	Bozic	0	unif	wholeT_0.01	CBN, CBN-A
150	Yes	9	9-A	1000	Bozic	0	unif	wholeT_0.5	CBN, CBN-A
151	Yes	9	9-A	1000	Bozic	Inf	last	singleC	CBN, CBN-A
152	Yes	9	9-A	1000	Bozic	Inf	last	wholeT_0.01	CBN, CBN-A
153	Yes	9	9-A	1000	Bozic	Inf	last	wholeT_0.5	CBN, CBN-A
154	Yes	9	9-A	1000	Bozic	Inf	unif	singleC	CBN, CBN-A
155	Yes	9	9-A	1000	Bozic	Inf	unif	wholeT_0.01	CBN, CBN-A
156	Yes	9	9-A	1000	Bozic	Inf	unif	wholeT_0.5	CBN
157	Yes	9	9-A	1000	exp	0	last	singleC	CBN, CBN-A

Table 11: *(continued)*

	Conjunction	Drivers	Tree	S.Size	Model	sh	S.Time	S.Type	Best method(s)
158	Yes	9	9-A	1000	exp	0	last	wholeT_0.01	CBN, CBN-A
159	Yes	9	9-A	1000	exp	0	last	wholeT_0.5	CBN, CBN-A
160	Yes	9	9-A	1000	exp	0	unif	singleC	CBN, CBN-A
161	Yes	9	9-A	1000	exp	0	unif	wholeT_0.01	CBN, CBN-A
162	Yes	9	9-A	1000	exp	0	unif	wholeT_0.5	CBN, CBN-A
163	Yes	9	9-A	1000	exp	Inf	last	singleC	CBN, CBN-A
164	Yes	9	9-A	1000	exp	Inf	last	wholeT_0.01	CBN, CBN-A
165	Yes	9	9-A	1000	exp	Inf	last	wholeT_0.5	CBN, CBN-A
166	Yes	9	9-A	1000	exp	Inf	unif	singleC	CBN, CBN-A
167	Yes	9	9-A	1000	exp	Inf	unif	wholeT_0.01	CBN, CBN-A
168	Yes	9	9-A	1000	exp	Inf	unif	wholeT_0.5	CBN, CBN-A
169	Yes	9	9-A	1000	McF_4	0	last	singleC	CBN, CBN-A, OT-A
170	Yes	9	9-A	1000	McF_4	0	last	wholeT_0.01	CBN
171	Yes	9	9-A	1000	McF_4	0	last	wholeT_0.5	CBN, CBN-A, OT-A
172	Yes	9	9-A	1000	McF_4	0	unif	singleC	CBN, CBN-A
173	Yes	9	9-A	1000	McF_4	0	unif	wholeT_0.01	CBN, CBN-A
174	Yes	9	9-A	1000	McF_4	0	unif	wholeT_0.5	CBN, CBN-A
175	Yes	9	9-A	1000	McF_4	Inf	last	singleC	CBN, CBN-A
176	Yes	9	9-A	1000	McF_4	Inf	last	wholeT_0.01	CBN, CBN-A, OT-A
177	Yes	9	9-A	1000	McF_4	Inf	last	wholeT_0.5	CBN, CBN-A
178	Yes	9	9-A	1000	McF_4	Inf	unif	singleC	CBN, CBN-A
179	Yes	9	9-A	1000	McF_4	Inf	unif	wholeT_0.01	CBN, CBN-A
180	Yes	9	9-A	1000	McF_4	Inf	unif	wholeT_0.5	CBN, CBN-A
181	Yes	9	9-A	1000	McF_6	0	last	singleC	OT-A
182	Yes	9	9-A	1000	McF_6	0	last	wholeT_0.01	DiP-A
183	Yes	9	9-A	1000	McF_6	0	last	wholeT_0.5	OT-A
184	Yes	9	9-A	1000	McF_6	0	unif	singleC	CBN, CBN-A
185	Yes	9	9-A	1000	McF_6	0	unif	wholeT_0.01	CBN, CBN-A
186	Yes	9	9-A	1000	McF_6	0	unif	wholeT_0.5	CBN, CBN-A
187	Yes	9	9-A	1000	McF_6	Inf	last	singleC	OT-A
188	Yes	9	9-A	1000	McF_6	Inf	last	wholeT_0.01	OT-A
189	Yes	9	9-A	1000	McF_6	Inf	last	wholeT_0.5	DiP-A, OT-A
190	Yes	9	9-A	1000	McF_6	Inf	unif	singleC	CBN, CBN-A
191	Yes	9	9-A	1000	McF_6	Inf	unif	wholeT_0.01	CBN, CBN-A
192	Yes	9	9-A	1000	McF_6	Inf	unif	wholeT_0.5	CBN, CBN-A
193	Yes	9	9-A	200	Bozic	0	last	singleC	CBN, CBN-A
194	Yes	9	9-A	200	Bozic	0	last	wholeT_0.01	CBN, CBN-A
195	Yes	9	9-A	200	Bozic	0	last	wholeT_0.5	CBN, CBN-A
196	Yes	9	9-A	200	Bozic	0	unif	singleC	CBN, CBN-A
197	Yes	9	9-A	200	Bozic	0	unif	wholeT_0.01	CBN, CBN-A

Table 11: *(continued)*

	Conjunction	Drivers	Tree	S.Size	Model	sh	S.Time	S.Type	Best method(s)
198	Yes	9	9-A	200	Bozic	0	unif	wholeT_0.5	CBN, CBN-A
199	Yes	9	9-A	200	Bozic	Inf	last	singleC	CBN, CBN-A
200	Yes	9	9-A	200	Bozic	Inf	last	wholeT_0.01	CBN, CBN-A
201	Yes	9	9-A	200	Bozic	Inf	last	wholeT_0.5	CBN, CBN-A
202	Yes	9	9-A	200	Bozic	Inf	unif	singleC	CBN, CBN-A
203	Yes	9	9-A	200	Bozic	Inf	unif	wholeT_0.01	CBN, CBN-A
204	Yes	9	9-A	200	Bozic	Inf	unif	wholeT_0.5	CBN, CBN-A
205	Yes	9	9-A	200	exp	0	last	singleC	CBN, CBN-A
206	Yes	9	9-A	200	exp	0	last	wholeT_0.01	CBN, CBN-A
207	Yes	9	9-A	200	exp	0	last	wholeT_0.5	CBN, CBN-A
208	Yes	9	9-A	200	exp	0	unif	singleC	CBN, CBN-A
209	Yes	9	9-A	200	exp	0	unif	wholeT_0.01	CBN, CBN-A
210	Yes	9	9-A	200	exp	0	unif	wholeT_0.5	CBN, CBN-A
211	Yes	9	9-A	200	exp	Inf	last	singleC	CBN, CBN-A
212	Yes	9	9-A	200	exp	Inf	last	wholeT_0.01	CBN, CBN-A
213	Yes	9	9-A	200	exp	Inf	last	wholeT_0.5	CBN, CBN-A
214	Yes	9	9-A	200	exp	Inf	unif	singleC	CBN, CBN-A
215	Yes	9	9-A	200	exp	Inf	unif	wholeT_0.01	CBN, CBN-A
216	Yes	9	9-A	200	exp	Inf	unif	wholeT_0.5	CBN, CBN-A
217	Yes	9	9-A	200	McF_4	0	last	singleC	CBN, CBN-A, OT-A
218	Yes	9	9-A	200	McF_4	0	last	wholeT_0.01	CBN, CBN-A, OT, OT-A
219	Yes	9	9-A	200	McF_4	0	last	wholeT_0.5	CBN, CBN-A
220	Yes	9	9-A	200	McF_4	0	unif	singleC	CBN, CBN-A
221	Yes	9	9-A	200	McF_4	0	unif	wholeT_0.01	CBN, CBN-A
222	Yes	9	9-A	200	McF_4	0	unif	wholeT_0.5	CBN, CBN-A
223	Yes	9	9-A	200	McF_4	Inf	last	singleC	CBN, CBN-A
224	Yes	9	9-A	200	McF_4	Inf	last	wholeT_0.01	CBN, OT-A
225	Yes	9	9-A	200	McF_4	Inf	last	wholeT_0.5	CBN, CBN-A
226	Yes	9	9-A	200	McF_4	Inf	unif	singleC	CBN, CBN-A
227	Yes	9	9-A	200	McF_4	Inf	unif	wholeT_0.01	CBN, CBN-A
228	Yes	9	9-A	200	McF_4	Inf	unif	wholeT_0.5	CBN, CBN-A
229	Yes	9	9-A	200	McF_6	0	last	singleC	OT-A
230	Yes	9	9-A	200	McF_6	0	last	wholeT_0.01	OT-A
231	Yes	9	9-A	200	McF_6	0	last	wholeT_0.5	OT-A
232	Yes	9	9-A	200	McF_6	0	unif	singleC	CBN, CBN-A
233	Yes	9	9-A	200	McF_6	0	unif	wholeT_0.01	CBN, CBN-A
234	Yes	9	9-A	200	McF_6	0	unif	wholeT_0.5	CBN, CBN-A
235	Yes	9	9-A	200	McF_6	Inf	last	singleC	OT-A
236	Yes	9	9-A	200	McF_6	Inf	last	wholeT_0.01	OT-A
237	Yes	9	9-A	200	McF_6	Inf	last	wholeT_0.5	OT-A

Table 11: *(continued)*

	Conjunction	Drivers	Tree	S.Size	Model	sh	S.Time	S.Type	Best method(s)
238	Yes	9	9-A	200	McF_6	Inf	unif	singleC	CBN, CBN-A
239	Yes	9	9-A	200	McF_6	Inf	unif	wholeT_0.01	CBN, CBN-A
240	Yes	9	9-A	200	McF_6	Inf	unif	wholeT_0.5	CBN, CBN-A
241	Yes	9	9-A	100	Bozic	0	last	singleC	CBN, CBN-A
242	Yes	9	9-A	100	Bozic	0	last	wholeT_0.01	CBN, CBN-A
243	Yes	9	9-A	100	Bozic	0	last	wholeT_0.5	CBN, CBN-A
244	Yes	9	9-A	100	Bozic	0	unif	singleC	CBN, CBN-A
245	Yes	9	9-A	100	Bozic	0	unif	wholeT_0.01	CBN, CBN-A
246	Yes	9	9-A	100	Bozic	0	unif	wholeT_0.5	CBN, CBN-A
247	Yes	9	9-A	100	Bozic	Inf	last	singleC	CBN, CBN-A
248	Yes	9	9-A	100	Bozic	Inf	last	wholeT_0.01	CBN, CBN-A
249	Yes	9	9-A	100	Bozic	Inf	last	wholeT_0.5	CBN, CBN-A
250	Yes	9	9-A	100	Bozic	Inf	unif	singleC	CBN, CBN-A
251	Yes	9	9-A	100	Bozic	Inf	unif	wholeT_0.01	CBN, CBN-A
252	Yes	9	9-A	100	Bozic	Inf	unif	wholeT_0.5	CBN, CBN-A
253	Yes	9	9-A	100	exp	0	last	singleC	CBN
254	Yes	9	9-A	100	exp	0	last	wholeT_0.01	CBN, CBN-A
255	Yes	9	9-A	100	exp	0	last	wholeT_0.5	CBN, CBN-A
256	Yes	9	9-A	100	exp	0	unif	singleC	CBN, CBN-A
257	Yes	9	9-A	100	exp	0	unif	wholeT_0.01	CBN, CBN-A
258	Yes	9	9-A	100	exp	0	unif	wholeT_0.5	CBN, CBN-A
259	Yes	9	9-A	100	exp	Inf	last	singleC	CBN, CBN-A
260	Yes	9	9-A	100	exp	Inf	last	wholeT_0.01	CBN, CBN-A
261	Yes	9	9-A	100	exp	Inf	last	wholeT_0.5	CBN, CBN-A
262	Yes	9	9-A	100	exp	Inf	unif	singleC	CBN, CBN-A
263	Yes	9	9-A	100	exp	Inf	unif	wholeT_0.01	CBN, CBN-A
264	Yes	9	9-A	100	exp	Inf	unif	wholeT_0.5	CBN, CBN-A
265	Yes	9	9-A	100	McF_4	0	last	singleC	CBN, CBN-A
266	Yes	9	9-A	100	McF_4	0	last	wholeT_0.01	CBN, CBN-A
267	Yes	9	9-A	100	McF_4	0	last	wholeT_0.5	CBN, CBN-A
268	Yes	9	9-A	100	McF_4	0	unif	singleC	CBN
269	Yes	9	9-A	100	McF_4	0	unif	wholeT_0.01	CBN, CBN-A
270	Yes	9	9-A	100	McF_4	0	unif	wholeT_0.5	CBN, CBN-A
271	Yes	9	9-A	100	McF_4	Inf	last	singleC	CBN, CBN-A
272	Yes	9	9-A	100	McF_4	Inf	last	wholeT_0.01	CBN
273	Yes	9	9-A	100	McF_4	Inf	last	wholeT_0.5	CBN, CBN-A
274	Yes	9	9-A	100	McF_4	Inf	unif	singleC	CBN, CBN-A
275	Yes	9	9-A	100	McF_4	Inf	unif	wholeT_0.01	CBN, CBN-A
276	Yes	9	9-A	100	McF_4	Inf	unif	wholeT_0.5	CBN, CBN-A
277	Yes	9	9-A	100	McF_6	0	last	singleC	OT-A

Table 11: *(continued)*

	Conjunction	Drivers	Tree	S.Size	Model	sh	S.Time	S.Type	Best method(s)
278	Yes	9	9-A	100	McF_6	0	last	wholeT_0.01	OT-A
279	Yes	9	9-A	100	McF_6	0	last	wholeT_0.5	OT-A
280	Yes	9	9-A	100	McF_6	0	unif	singleC	CBN, CBN-A
281	Yes	9	9-A	100	McF_6	0	unif	wholeT_0.01	CBN, CBN-A
282	Yes	9	9-A	100	McF_6	0	unif	wholeT_0.5	CBN, CBN-A
283	Yes	9	9-A	100	McF_6	Inf	last	singleC	OT-A
284	Yes	9	9-A	100	McF_6	Inf	last	wholeT_0.01	OT-A
285	Yes	9	9-A	100	McF_6	Inf	last	wholeT_0.5	CBN, OT-A
286	Yes	9	9-A	100	McF_6	Inf	unif	singleC	CBN, CBN-A
287	Yes	9	9-A	100	McF_6	Inf	unif	wholeT_0.01	CBN-A
288	Yes	9	9-A	100	McF_6	Inf	unif	wholeT_0.5	CBN, CBN-A
289	Yes	7	7-A	1000	Bozic	0	last	singleC	CBN, CBN-A, OT-A
290	Yes	7	7-A	1000	Bozic	0	last	wholeT_0.01	OT-A
291	Yes	7	7-A	1000	Bozic	0	last	wholeT_0.5	CBN, CBN-A, OT-A
292	Yes	7	7-A	1000	Bozic	0	unif	singleC	CBN, CBN-A
293	Yes	7	7-A	1000	Bozic	0	unif	wholeT_0.01	CBN, CBN-A
294	Yes	7	7-A	1000	Bozic	0	unif	wholeT_0.5	CBN, CBN-A
295	Yes	7	7-A	1000	Bozic	Inf	last	singleC	CBN, OT-A
296	Yes	7	7-A	1000	Bozic	Inf	last	wholeT_0.01	CBN-A
297	Yes	7	7-A	1000	Bozic	Inf	last	wholeT_0.5	CBN, OT-A
298	Yes	7	7-A	1000	Bozic	Inf	unif	singleC	CBN, CBN-A
299	Yes	7	7-A	1000	Bozic	Inf	unif	wholeT_0.01	CBN, CBN-A
300	Yes	7	7-A	1000	Bozic	Inf	unif	wholeT_0.5	CBN, CBN-A
301	Yes	7	7-A	1000	exp	0	last	singleC	CBN-A
302	Yes	7	7-A	1000	exp	0	last	wholeT_0.01	CBN, CBN-A, OT-A
303	Yes	7	7-A	1000	exp	0	last	wholeT_0.5	CBN, CBN-A
304	Yes	7	7-A	1000	exp	0	unif	singleC	CBN, CBN-A
305	Yes	7	7-A	1000	exp	0	unif	wholeT_0.01	CBN, CBN-A
306	Yes	7	7-A	1000	exp	0	unif	wholeT_0.5	CBN, CBN-A
307	Yes	7	7-A	1000	exp	Inf	last	singleC	CBN, CBN-A, OT-A
308	Yes	7	7-A	1000	exp	Inf	last	wholeT_0.01	CBN-A
309	Yes	7	7-A	1000	exp	Inf	last	wholeT_0.5	CBN, CBN-A
310	Yes	7	7-A	1000	exp	Inf	unif	singleC	CBN, CBN-A
311	Yes	7	7-A	1000	exp	Inf	unif	wholeT_0.01	CBN, CBN-A
312	Yes	7	7-A	1000	exp	Inf	unif	wholeT_0.5	CBN, CBN-A
313	Yes	7	7-A	1000	McF_4	0	last	singleC	CBN, OT-A
314	Yes	7	7-A	1000	McF_4	0	last	wholeT_0.01	OT-A
315	Yes	7	7-A	1000	McF_4	0	last	wholeT_0.5	CBN, OT-A
316	Yes	7	7-A	1000	McF_4	0	unif	singleC	CBN, CBN-A, OT-A
317	Yes	7	7-A	1000	McF_4	0	unif	wholeT_0.01	CBN-A, DiP, DiP-A, OT, OT-A

Table 11: *(continued)*

	Conjunction	Drivers	Tree	S.Size	Model	sh	S.Time	S.Type	Best method(s)
318	Yes	7	7-A	1000	McF_4	0	unif	wholeT_0.5	CBN, CBN-A, OT, OT-A
319	Yes	7	7-A	1000	McF_4	Inf	last	singleC	CBN, OT-A
320	Yes	7	7-A	1000	McF_4	Inf	last	wholeT_0.01	CBN, OT-A
321	Yes	7	7-A	1000	McF_4	Inf	last	wholeT_0.5	CBN
322	Yes	7	7-A	1000	McF_4	Inf	unif	singleC	CBN, CBN-A
323	Yes	7	7-A	1000	McF_4	Inf	unif	wholeT_0.01	CBN, CBN-A, OT, OT-A
324	Yes	7	7-A	1000	McF_4	Inf	unif	wholeT_0.5	CBN, CBN-A
325	Yes	7	7-A	1000	McF_6	0	last	singleC	DiP-A
326	Yes	7	7-A	1000	McF_6	0	last	wholeT_0.01	DiP-A, OT-A
327	Yes	7	7-A	1000	McF_6	0	last	wholeT_0.5	DiP-A
328	Yes	7	7-A	1000	McF_6	0	unif	singleC	CBN-A
329	Yes	7	7-A	1000	McF_6	0	unif	wholeT_0.01	CBN-A, DiP, DiP-A
330	Yes	7	7-A	1000	McF_6	0	unif	wholeT_0.5	CBN-A
331	Yes	7	7-A	1000	McF_6	Inf	last	singleC	CBN-A, DiP-A
332	Yes	7	7-A	1000	McF_6	Inf	last	wholeT_0.01	DiP-A
333	Yes	7	7-A	1000	McF_6	Inf	last	wholeT_0.5	CBN-A, DiP-A
334	Yes	7	7-A	1000	McF_6	Inf	unif	singleC	CBN-A
335	Yes	7	7-A	1000	McF_6	Inf	unif	wholeT_0.01	CBN-A
336	Yes	7	7-A	1000	McF_6	Inf	unif	wholeT_0.5	CBN-A
337	Yes	7	7-A	200	Bozic	0	last	singleC	CBN
338	Yes	7	7-A	200	Bozic	0	last	wholeT_0.01	CBN, OT-A
339	Yes	7	7-A	200	Bozic	0	last	wholeT_0.5	CBN, CBN-A
340	Yes	7	7-A	200	Bozic	0	unif	singleC	CBN, CBN-A
341	Yes	7	7-A	200	Bozic	0	unif	wholeT_0.01	CBN, CBN-A
342	Yes	7	7-A	200	Bozic	0	unif	wholeT_0.5	CBN, CBN-A
343	Yes	7	7-A	200	Bozic	Inf	last	singleC	CBN, CBN-A, OT-A
344	Yes	7	7-A	200	Bozic	Inf	last	wholeT_0.01	CBN-A
345	Yes	7	7-A	200	Bozic	Inf	last	wholeT_0.5	CBN, OT-A
346	Yes	7	7-A	200	Bozic	Inf	unif	singleC	CBN, CBN-A
347	Yes	7	7-A	200	Bozic	Inf	unif	wholeT_0.01	CBN, CBN-A
348	Yes	7	7-A	200	Bozic	Inf	unif	wholeT_0.5	CBN, CBN-A
349	Yes	7	7-A	200	exp	0	last	singleC	CBN, CBN-A
350	Yes	7	7-A	200	exp	0	last	wholeT_0.01	CBN, CBN-A
351	Yes	7	7-A	200	exp	0	last	wholeT_0.5	CBN, CBN-A
352	Yes	7	7-A	200	exp	0	unif	singleC	CBN, CBN-A
353	Yes	7	7-A	200	exp	0	unif	wholeT_0.01	CBN, CBN-A
354	Yes	7	7-A	200	exp	0	unif	wholeT_0.5	CBN, CBN-A
355	Yes	7	7-A	200	exp	Inf	last	singleC	CBN
356	Yes	7	7-A	200	exp	Inf	last	wholeT_0.01	CBN-A
357	Yes	7	7-A	200	exp	Inf	last	wholeT_0.5	CBN

Table 11: *(continued)*

	Conjunction	Drivers	Tree	S.Size	Model	sh	S.Time	S.Type	Best method(s)
358	Yes	7	7-A	200	exp	Inf	unif	singleC	CBN, CBN-A
359	Yes	7	7-A	200	exp	Inf	unif	wholeT_0.01	CBN-A
360	Yes	7	7-A	200	exp	Inf	unif	wholeT_0.5	CBN, CBN-A
361	Yes	7	7-A	200	McF_4	0	last	singleC	CBN, OT-A
362	Yes	7	7-A	200	McF_4	0	last	wholeT_0.01	OT-A
363	Yes	7	7-A	200	McF_4	0	last	wholeT_0.5	CBN, OT-A
364	Yes	7	7-A	200	McF_4	0	unif	singleC	CBN, CBN-A
365	Yes	7	7-A	200	McF_4	0	unif	wholeT_0.01	CBN, CBN-A, OT, OT-A
366	Yes	7	7-A	200	McF_4	0	unif	wholeT_0.5	CBN, CBN-A
367	Yes	7	7-A	200	McF_4	Inf	last	singleC	CBN
368	Yes	7	7-A	200	McF_4	Inf	last	wholeT_0.01	CBN, OT-A
369	Yes	7	7-A	200	McF_4	Inf	last	wholeT_0.5	CBN, CBN-A
370	Yes	7	7-A	200	McF_4	Inf	unif	singleC	CBN, CBN-A
371	Yes	7	7-A	200	McF_4	Inf	unif	wholeT_0.01	CBN, CBN-A
372	Yes	7	7-A	200	McF_4	Inf	unif	wholeT_0.5	CBN, CBN-A
373	Yes	7	7-A	200	McF_6	0	last	singleC	CBN-A, OT-A
374	Yes	7	7-A	200	McF_6	0	last	wholeT_0.01	OT-A
375	Yes	7	7-A	200	McF_6	0	last	wholeT_0.5	CBN-A, OT-A
376	Yes	7	7-A	200	McF_6	0	unif	singleC	CBN-A
377	Yes	7	7-A	200	McF_6	0	unif	wholeT_0.01	CBN-A, OT, OT-A
378	Yes	7	7-A	200	McF_6	0	unif	wholeT_0.5	CBN-A
379	Yes	7	7-A	200	McF_6	Inf	last	singleC	CBN, CBN-A, OT-A
380	Yes	7	7-A	200	McF_6	Inf	last	wholeT_0.01	OT-A
381	Yes	7	7-A	200	McF_6	Inf	last	wholeT_0.5	CBN, CBN-A, OT-A
382	Yes	7	7-A	200	McF_6	Inf	unif	singleC	CBN-A
383	Yes	7	7-A	200	McF_6	Inf	unif	wholeT_0.01	CBN-A
384	Yes	7	7-A	200	McF_6	Inf	unif	wholeT_0.5	CBN-A
385	Yes	7	7-A	100	Bozic	0	last	singleC	CBN, CBN-A
386	Yes	7	7-A	100	Bozic	0	last	wholeT_0.01	CBN, OT-A
387	Yes	7	7-A	100	Bozic	0	last	wholeT_0.5	CBN, CBN-A
388	Yes	7	7-A	100	Bozic	0	unif	singleC	CBN, CBN-A
389	Yes	7	7-A	100	Bozic	0	unif	wholeT_0.01	CBN, CBN-A
390	Yes	7	7-A	100	Bozic	0	unif	wholeT_0.5	CBN, CBN-A
391	Yes	7	7-A	100	Bozic	Inf	last	singleC	CBN, CBN-A, OT-A
392	Yes	7	7-A	100	Bozic	Inf	last	wholeT_0.01	CBN-A
393	Yes	7	7-A	100	Bozic	Inf	last	wholeT_0.5	CBN, CBN-A, OT-A
394	Yes	7	7-A	100	Bozic	Inf	unif	singleC	CBN, CBN-A
395	Yes	7	7-A	100	Bozic	Inf	unif	wholeT_0.01	CBN, CBN-A
396	Yes	7	7-A	100	Bozic	Inf	unif	wholeT_0.5	CBN, CBN-A
397	Yes	7	7-A	100	exp	0	last	singleC	CBN, CBN-A

Table 11: *(continued)*

	Conjunction	Drivers	Tree	S.Size	Model	sh	S.Time	S.Type	Best method(s)
398	Yes	7	7-A	100	exp	0	last	wholeT_0.01	CBN, CBN-A
399	Yes	7	7-A	100	exp	0	last	wholeT_0.5	CBN-A
400	Yes	7	7-A	100	exp	0	unif	singleC	CBN, CBN-A
401	Yes	7	7-A	100	exp	0	unif	wholeT_0.01	CBN, CBN-A
402	Yes	7	7-A	100	exp	0	unif	wholeT_0.5	CBN, CBN-A
403	Yes	7	7-A	100	exp	Inf	last	singleC	CBN, CBN-A
404	Yes	7	7-A	100	exp	Inf	last	wholeT_0.01	CBN-A
405	Yes	7	7-A	100	exp	Inf	last	wholeT_0.5	CBN
406	Yes	7	7-A	100	exp	Inf	unif	singleC	CBN, CBN-A
407	Yes	7	7-A	100	exp	Inf	unif	wholeT_0.01	CBN, CBN-A
408	Yes	7	7-A	100	exp	Inf	unif	wholeT_0.5	CBN, CBN-A
409	Yes	7	7-A	100	McF_4	0	last	singleC	CBN, CBN-A, OT-A
410	Yes	7	7-A	100	McF_4	0	last	wholeT_0.01	OT-A
411	Yes	7	7-A	100	McF_4	0	last	wholeT_0.5	CBN, OT-A
412	Yes	7	7-A	100	McF_4	0	unif	singleC	CBN-A
413	Yes	7	7-A	100	McF_4	0	unif	wholeT_0.01	CBN, CBN-A, OT-A
414	Yes	7	7-A	100	McF_4	0	unif	wholeT_0.5	CBN-A
415	Yes	7	7-A	100	McF_4	Inf	last	singleC	CBN, CBN-A
416	Yes	7	7-A	100	McF_4	Inf	last	wholeT_0.01	CBN, CBN-A, OT-A
417	Yes	7	7-A	100	McF_4	Inf	last	wholeT_0.5	CBN, CBN-A
418	Yes	7	7-A	100	McF_4	Inf	unif	singleC	CBN, CBN-A
419	Yes	7	7-A	100	McF_4	Inf	unif	wholeT_0.01	CBN, CBN-A
420	Yes	7	7-A	100	McF_4	Inf	unif	wholeT_0.5	CBN, CBN-A
421	Yes	7	7-A	100	McF_6	0	last	singleC	CBN, CBN-A, OT-A
422	Yes	7	7-A	100	McF_6	0	last	wholeT_0.01	OT-A
423	Yes	7	7-A	100	McF_6	0	last	wholeT_0.5	CBN-A, OT-A
424	Yes	7	7-A	100	McF_6	0	unif	singleC	CBN, CBN-A
425	Yes	7	7-A	100	McF_6	0	unif	wholeT_0.01	CBN, CBN-A, OT, OT-A
426	Yes	7	7-A	100	McF_6	0	unif	wholeT_0.5	CBN-A
427	Yes	7	7-A	100	McF_6	Inf	last	singleC	CBN, CBN-A, OT-A
428	Yes	7	7-A	100	McF_6	Inf	last	wholeT_0.01	OT-A
429	Yes	7	7-A	100	McF_6	Inf	last	wholeT_0.5	CBN-A, OT-A
430	Yes	7	7-A	100	McF_6	Inf	unif	singleC	CBN-A
431	Yes	7	7-A	100	McF_6	Inf	unif	wholeT_0.01	CBN-A
432	Yes	7	7-A	100	McF_6	Inf	unif	wholeT_0.5	CBN-A
433	No	11	11-B	1000	Bozic	0	last	singleC	CBN, CBN-A, OT, OT-A
434	No	11	11-B	1000	Bozic	0	last	wholeT_0.01	OT, OT-A
435	No	11	11-B	1000	Bozic	0	last	wholeT_0.5	CBN, CBN-A
436	No	11	11-B	1000	Bozic	0	unif	singleC	CBN, CBN-A
437	No	11	11-B	1000	Bozic	0	unif	wholeT_0.01	CBN, CBN-A

Table 11: *(continued)*

	Conjunction	Drivers	Tree	S.Size	Model	sh	S.Time	S.Type	Best method(s)
438	No	11	11-B	1000	Bozic	0	unif	wholeT_0.5	CBN, CBN-A
439	No	11	11-B	1000	Bozic	Inf	last	singleC	OT, OT-A
440	No	11	11-B	1000	Bozic	Inf	last	wholeT_0.01	CBN, CBN-A, OT, OT-A
441	No	11	11-B	1000	Bozic	Inf	last	wholeT_0.5	CBN, OT, OT-A
442	No	11	11-B	1000	Bozic	Inf	unif	singleC	CBN, CBN-A
443	No	11	11-B	1000	Bozic	Inf	unif	wholeT_0.01	CBN, CBN-A, OT, OT-A
444	No	11	11-B	1000	Bozic	Inf	unif	wholeT_0.5	CBN, CBN-A, OT, OT-A
445	No	11	11-B	1000	exp	0	last	singleC	CBN, CBN-A
446	No	11	11-B	1000	exp	0	last	wholeT_0.01	CBN, CBN-A
447	No	11	11-B	1000	exp	0	last	wholeT_0.5	CBN, CBN-A
448	No	11	11-B	1000	exp	0	unif	singleC	CBN, CBN-A
449	No	11	11-B	1000	exp	0	unif	wholeT_0.01	CBN, CBN-A
450	No	11	11-B	1000	exp	0	unif	wholeT_0.5	CBN, CBN-A
451	No	11	11-B	1000	exp	Inf	last	singleC	CBN, CBN-A, OT, OT-A
452	No	11	11-B	1000	exp	Inf	last	wholeT_0.01	CBN, CBN-A, OT, OT-A
453	No	11	11-B	1000	exp	Inf	last	wholeT_0.5	CBN-A, OT, OT-A
454	No	11	11-B	1000	exp	Inf	unif	singleC	CBN, CBN-A
455	No	11	11-B	1000	exp	Inf	unif	wholeT_0.01	CBN, CBN-A
456	No	11	11-B	1000	exp	Inf	unif	wholeT_0.5	CBN, CBN-A
457	No	11	11-B	1000	McF_4	0	last	singleC	OT, OT-A
458	No	11	11-B	1000	McF_4	0	last	wholeT_0.01	DiP, DiP-A, OT, OT-A
459	No	11	11-B	1000	McF_4	0	last	wholeT_0.5	OT, OT-A
460	No	11	11-B	1000	McF_4	0	unif	singleC	OT, OT-A
461	No	11	11-B	1000	McF_4	0	unif	wholeT_0.01	CBN-A, OT, OT-A
462	No	11	11-B	1000	McF_4	0	unif	wholeT_0.5	CBN, OT, OT-A
463	No	11	11-B	1000	McF_4	Inf	last	singleC	OT, OT-A
464	No	11	11-B	1000	McF_4	Inf	last	wholeT_0.01	OT, OT-A
465	No	11	11-B	1000	McF_4	Inf	last	wholeT_0.5	CBN-A, OT, OT-A
466	No	11	11-B	1000	McF_4	Inf	unif	singleC	CBN, CBN-A, OT, OT-A
467	No	11	11-B	1000	McF_4	Inf	unif	wholeT_0.01	CBN, CBN-A, OT, OT-A
468	No	11	11-B	1000	McF_4	Inf	unif	wholeT_0.5	CBN, CBN-A, OT, OT-A
469	No	11	11-B	1000	McF_6	0	last	singleC	DiP, DiP-A, OT, OT-A
470	No	11	11-B	1000	McF_6	0	last	wholeT_0.01	DiP, DiP-A, OT, OT-A
471	No	11	11-B	1000	McF_6	0	last	wholeT_0.5	DiP, DiP-A, OT, OT-A
472	No	11	11-B	1000	McF_6	0	unif	singleC	DiP, DiP-A, OT, OT-A
473	No	11	11-B	1000	McF_6	0	unif	wholeT_0.01	DiP, DiP-A, OT, OT-A
474	No	11	11-B	1000	McF_6	0	unif	wholeT_0.5	DiP, DiP-A, OT, OT-A
475	No	11	11-B	1000	McF_6	Inf	last	singleC	DiP, DiP-A, OT, OT-A
476	No	11	11-B	1000	McF_6	Inf	last	wholeT_0.01	DiP-A, OT, OT-A
477	No	11	11-B	1000	McF_6	Inf	last	wholeT_0.5	DiP, DiP-A, OT, OT-A

Table 11: *(continued)*

	Conjunction	Drivers	Tree	S.Size	Model	sh	S.Time	S.Type	Best method(s)
478	No	11	11-B	1000	McF_6	Inf	unif	singleC	DiP, DiP-A, OT
479	No	11	11-B	1000	McF_6	Inf	unif	wholeT_0.01	DiP, DiP-A, OT, OT-A
480	No	11	11-B	1000	McF_6	Inf	unif	wholeT_0.5	DiP, DiP-A, OT, OT-A
481	No	11	11-B	200	Bozic	0	last	singleC	CBN, CBN-A
482	No	11	11-B	200	Bozic	0	last	wholeT_0.01	CBN, CBN-A
483	No	11	11-B	200	Bozic	0	last	wholeT_0.5	CBN, CBN-A
484	No	11	11-B	200	Bozic	0	unif	singleC	CBN, CBN-A
485	No	11	11-B	200	Bozic	0	unif	wholeT_0.01	CBN, CBN-A
486	No	11	11-B	200	Bozic	0	unif	wholeT_0.5	CBN, CBN-A
487	No	11	11-B	200	Bozic	Inf	last	singleC	CBN, CBN-A, OT, OT-A
488	No	11	11-B	200	Bozic	Inf	last	wholeT_0.01	CBN, CBN-A, OT, OT-A
489	No	11	11-B	200	Bozic	Inf	last	wholeT_0.5	CBN, CBN-A, OT, OT-A
490	No	11	11-B	200	Bozic	Inf	unif	singleC	CBN, CBN-A
491	No	11	11-B	200	Bozic	Inf	unif	wholeT_0.01	CBN, CBN-A
492	No	11	11-B	200	Bozic	Inf	unif	wholeT_0.5	CBN, CBN-A
493	No	11	11-B	200	exp	0	last	singleC	CBN, CBN-A
494	No	11	11-B	200	exp	0	last	wholeT_0.01	CBN, CBN-A
495	No	11	11-B	200	exp	0	last	wholeT_0.5	CBN, CBN-A
496	No	11	11-B	200	exp	0	unif	singleC	CBN, CBN-A
497	No	11	11-B	200	exp	0	unif	wholeT_0.01	CBN, CBN-A
498	No	11	11-B	200	exp	0	unif	wholeT_0.5	CBN, CBN-A
499	No	11	11-B	200	exp	Inf	last	singleC	CBN, CBN-A, OT, OT-A
500	No	11	11-B	200	exp	Inf	last	wholeT_0.01	CBN, CBN-A
501	No	11	11-B	200	exp	Inf	last	wholeT_0.5	CBN, CBN-A, OT, OT-A
502	No	11	11-B	200	exp	Inf	unif	singleC	CBN, CBN-A
503	No	11	11-B	200	exp	Inf	unif	wholeT_0.01	CBN, CBN-A
504	No	11	11-B	200	exp	Inf	unif	wholeT_0.5	CBN, CBN-A
505	No	11	11-B	200	McF_4	0	last	singleC	OT, OT-A
506	No	11	11-B	200	McF_4	0	last	wholeT_0.01	OT, OT-A
507	No	11	11-B	200	McF_4	0	last	wholeT_0.5	OT, OT-A
508	No	11	11-B	200	McF_4	0	unif	singleC	CBN, CBN-A
509	No	11	11-B	200	McF_4	0	unif	wholeT_0.01	CBN, CBN-A, OT, OT-A
510	No	11	11-B	200	McF_4	0	unif	wholeT_0.5	CBN, CBN-A
511	No	11	11-B	200	McF_4	Inf	last	singleC	CBN, CBN-A, OT, OT-A
512	No	11	11-B	200	McF_4	Inf	last	wholeT_0.01	OT, OT-A
513	No	11	11-B	200	McF_4	Inf	last	wholeT_0.5	CBN, OT, OT-A
514	No	11	11-B	200	McF_4	Inf	unif	singleC	CBN, CBN-A, OT-A
515	No	11	11-B	200	McF_4	Inf	unif	wholeT_0.01	CBN, CBN-A, OT, OT-A
516	No	11	11-B	200	McF_4	Inf	unif	wholeT_0.5	CBN, CBN-A
517	No	11	11-B	200	McF_6	0	last	singleC	OT, OT-A

Table 11: *(continued)*

	Conjunction	Drivers	Tree	S.Size	Model	sh	S.Time	S.Type	Best method(s)
518	No	11	11-B	200	McF_6	0	last	wholeT_0.01	OT, OT-A
519	No	11	11-B	200	McF_6	0	last	wholeT_0.5	OT, OT-A
520	No	11	11-B	200	McF_6	0	unif	singleC	OT, OT-A
521	No	11	11-B	200	McF_6	0	unif	wholeT_0.01	CBN-A, OT, OT-A
522	No	11	11-B	200	McF_6	0	unif	wholeT_0.5	OT, OT-A
523	No	11	11-B	200	McF_6	Inf	last	singleC	OT, OT-A
524	No	11	11-B	200	McF_6	Inf	last	wholeT_0.01	OT, OT-A
525	No	11	11-B	200	McF_6	Inf	last	wholeT_0.5	OT, OT-A
526	No	11	11-B	200	McF_6	Inf	unif	singleC	OT, OT-A
527	No	11	11-B	200	McF_6	Inf	unif	wholeT_0.01	OT, OT-A
528	No	11	11-B	200	McF_6	Inf	unif	wholeT_0.5	OT, OT-A
529	No	11	11-B	100	Bozic	0	last	singleC	CBN, CBN-A
530	No	11	11-B	100	Bozic	0	last	wholeT_0.01	CBN, CBN-A
531	No	11	11-B	100	Bozic	0	last	wholeT_0.5	CBN, CBN-A
532	No	11	11-B	100	Bozic	0	unif	singleC	CBN, CBN-A
533	No	11	11-B	100	Bozic	0	unif	wholeT_0.01	CBN, CBN-A
534	No	11	11-B	100	Bozic	0	unif	wholeT_0.5	CBN, CBN-A
535	No	11	11-B	100	Bozic	Inf	last	singleC	CBN, CBN-A, OT, OT-A
536	No	11	11-B	100	Bozic	Inf	last	wholeT_0.01	CBN, CBN-A, OT
537	No	11	11-B	100	Bozic	Inf	last	wholeT_0.5	CBN, CBN-A, OT, OT-A
538	No	11	11-B	100	Bozic	Inf	unif	singleC	CBN, CBN-A
539	No	11	11-B	100	Bozic	Inf	unif	wholeT_0.01	CBN, CBN-A
540	No	11	11-B	100	Bozic	Inf	unif	wholeT_0.5	CBN, CBN-A
541	No	11	11-B	100	exp	0	last	singleC	CBN, CBN-A
542	No	11	11-B	100	exp	0	last	wholeT_0.01	CBN, CBN-A
543	No	11	11-B	100	exp	0	last	wholeT_0.5	CBN, CBN-A
544	No	11	11-B	100	exp	0	unif	singleC	CBN, CBN-A
545	No	11	11-B	100	exp	0	unif	wholeT_0.01	CBN, CBN-A
546	No	11	11-B	100	exp	0	unif	wholeT_0.5	CBN, CBN-A
547	No	11	11-B	100	exp	Inf	last	singleC	CBN, CBN-A
548	No	11	11-B	100	exp	Inf	last	wholeT_0.01	CBN, CBN-A
549	No	11	11-B	100	exp	Inf	last	wholeT_0.5	CBN, CBN-A
550	No	11	11-B	100	exp	Inf	unif	singleC	CBN, CBN-A
551	No	11	11-B	100	exp	Inf	unif	wholeT_0.01	CBN, CBN-A
552	No	11	11-B	100	exp	Inf	unif	wholeT_0.5	CBN, CBN-A
553	No	11	11-B	100	McF_4	0	last	singleC	CBN-A, OT-A
554	No	11	11-B	100	McF_4	0	last	wholeT_0.01	OT, OT-A
555	No	11	11-B	100	McF_4	0	last	wholeT_0.5	CBN-A, OT, OT-A
556	No	11	11-B	100	McF_4	0	unif	singleC	CBN, CBN-A
557	No	11	11-B	100	McF_4	0	unif	wholeT_0.01	CBN, CBN-A

Table 11: *(continued)*

	Conjunction	Drivers	Tree	S.Size	Model	sh	S.Time	S.Type	Best method(s)
558	No	11	11-B	100	McF_4	0	unif	wholeT_0.5	CBN, CBN-A
559	No	11	11-B	100	McF_4	Inf	last	singleC	CBN, CBN-A, OT, OT-A
560	No	11	11-B	100	McF_4	Inf	last	wholeT_0.01	OT, OT-A
561	No	11	11-B	100	McF_4	Inf	last	wholeT_0.5	CBN-A, OT, OT-A
562	No	11	11-B	100	McF_4	Inf	unif	singleC	CBN, CBN-A
563	No	11	11-B	100	McF_4	Inf	unif	wholeT_0.01	CBN, CBN-A
564	No	11	11-B	100	McF_4	Inf	unif	wholeT_0.5	CBN, CBN-A
565	No	11	11-B	100	McF_6	0	last	singleC	OT, OT-A
566	No	11	11-B	100	McF_6	0	last	wholeT_0.01	OT, OT-A
567	No	11	11-B	100	McF_6	0	last	wholeT_0.5	OT, OT-A
568	No	11	11-B	100	McF_6	0	unif	singleC	CBN-A, OT, OT-A
569	No	11	11-B	100	McF_6	0	unif	wholeT_0.01	OT, OT-A
570	No	11	11-B	100	McF_6	0	unif	wholeT_0.5	OT, OT-A
571	No	11	11-B	100	McF_6	Inf	last	singleC	OT, OT-A
572	No	11	11-B	100	McF_6	Inf	last	wholeT_0.01	OT, OT-A
573	No	11	11-B	100	McF_6	Inf	last	wholeT_0.5	OT, OT-A
574	No	11	11-B	100	McF_6	Inf	unif	singleC	CBN, CBN-A, OT, OT-A
575	No	11	11-B	100	McF_6	Inf	unif	wholeT_0.01	CBN, CBN-A, OT, OT-A
576	No	11	11-B	100	McF_6	Inf	unif	wholeT_0.5	CBN, CBN-A, OT, OT-A
577	No	9	9-B	1000	Bozic	0	last	singleC	CBN, CBN-A, OT, OT-A
578	No	9	9-B	1000	Bozic	0	last	wholeT_0.01	OT, OT-A
579	No	9	9-B	1000	Bozic	0	last	wholeT_0.5	CBN, CBN-A, OT, OT-A
580	No	9	9-B	1000	Bozic	0	unif	singleC	CBN, CBN-A
581	No	9	9-B	1000	Bozic	0	unif	wholeT_0.01	CBN, CBN-A
582	No	9	9-B	1000	Bozic	0	unif	wholeT_0.5	CBN, CBN-A
583	No	9	9-B	1000	Bozic	Inf	last	singleC	OT, OT-A
584	No	9	9-B	1000	Bozic	Inf	last	wholeT_0.01	DiP, DiP-A, OT, OT-A
585	No	9	9-B	1000	Bozic	Inf	last	wholeT_0.5	OT, OT-A
586	No	9	9-B	1000	Bozic	Inf	unif	singleC	CBN, OT, OT-A
587	No	9	9-B	1000	Bozic	Inf	unif	wholeT_0.01	CBN-A, OT, OT-A
588	No	9	9-B	1000	Bozic	Inf	unif	wholeT_0.5	CBN, CBN-A, OT, OT-A
589	No	9	9-B	1000	exp	0	last	singleC	CBN, CBN-A
590	No	9	9-B	1000	exp	0	last	wholeT_0.01	CBN, CBN-A
591	No	9	9-B	1000	exp	0	last	wholeT_0.5	CBN, CBN-A
592	No	9	9-B	1000	exp	0	unif	singleC	CBN, CBN-A
593	No	9	9-B	1000	exp	0	unif	wholeT_0.01	CBN, CBN-A
594	No	9	9-B	1000	exp	0	unif	wholeT_0.5	CBN, CBN-A
595	No	9	9-B	1000	exp	Inf	last	singleC	OT, OT-A
596	No	9	9-B	1000	exp	Inf	last	wholeT_0.01	OT, OT-A
597	No	9	9-B	1000	exp	Inf	last	wholeT_0.5	OT, OT-A

Table 11: *(continued)*

	Conjunction	Drivers	Tree	S.Size	Model	sh	S.Time	S.Type	Best method(s)
598	No	9	9-B	1000	exp	Inf	unif	singleC	CBN, CBN-A, OT, OT-A
599	No	9	9-B	1000	exp	Inf	unif	wholeT_0.01	CBN, CBN-A, OT, OT-A
600	No	9	9-B	1000	exp	Inf	unif	wholeT_0.5	CBN, CBN-A
601	No	9	9-B	1000	McF_4	0	last	singleC	DiP-A, OT, OT-A
602	No	9	9-B	1000	McF_4	0	last	wholeT_0.01	DiP, DiP-A, OT, OT-A
603	No	9	9-B	1000	McF_4	0	last	wholeT_0.5	OT, OT-A
604	No	9	9-B	1000	McF_4	0	unif	singleC	OT, OT-A
605	No	9	9-B	1000	McF_4	0	unif	wholeT_0.01	OT, OT-A
606	No	9	9-B	1000	McF_4	0	unif	wholeT_0.5	OT, OT-A
607	No	9	9-B	1000	McF_4	Inf	last	singleC	OT, OT-A
608	No	9	9-B	1000	McF_4	Inf	last	wholeT_0.01	DiP, DiP-A, OT, OT-A
609	No	9	9-B	1000	McF_4	Inf	last	wholeT_0.5	OT, OT-A
610	No	9	9-B	1000	McF_4	Inf	unif	singleC	OT, OT-A
611	No	9	9-B	1000	McF_4	Inf	unif	wholeT_0.01	OT, OT-A
612	No	9	9-B	1000	McF_4	Inf	unif	wholeT_0.5	OT, OT-A
613	No	9	9-B	1000	McF_6	0	last	singleC	DiP-A, OT-A
614	No	9	9-B	1000	McF_6	0	last	wholeT_0.01	DiP-A, OT-A
615	No	9	9-B	1000	McF_6	0	last	wholeT_0.5	DiP-A, OT-A
616	No	9	9-B	1000	McF_6	0	unif	singleC	DiP, DiP-A, OT, OT-A
617	No	9	9-B	1000	McF_6	0	unif	wholeT_0.01	DiP, DiP-A, OT, OT-A
618	No	9	9-B	1000	McF_6	0	unif	wholeT_0.5	none
619	No	9	9-B	1000	McF_6	Inf	last	singleC	DiP-A, OT-A
620	No	9	9-B	1000	McF_6	Inf	last	wholeT_0.01	DiP-A, OT-A
621	No	9	9-B	1000	McF_6	Inf	last	wholeT_0.5	DiP-A, OT-A
622	No	9	9-B	1000	McF_6	Inf	unif	singleC	DiP, DiP-A, OT, OT-A
623	No	9	9-B	1000	McF_6	Inf	unif	wholeT_0.01	DiP, DiP-A, OT, OT-A
624	No	9	9-B	1000	McF_6	Inf	unif	wholeT_0.5	DiP, DiP-A, OT, OT-A
625	No	9	9-B	200	Bozic	0	last	singleC	CBN, CBN-A, OT, OT-A
626	No	9	9-B	200	Bozic	0	last	wholeT_0.01	CBN, CBN-A, OT
627	No	9	9-B	200	Bozic	0	last	wholeT_0.5	CBN, CBN-A
628	No	9	9-B	200	Bozic	0	unif	singleC	CBN, CBN-A
629	No	9	9-B	200	Bozic	0	unif	wholeT_0.01	CBN, CBN-A
630	No	9	9-B	200	Bozic	0	unif	wholeT_0.5	CBN, CBN-A
631	No	9	9-B	200	Bozic	Inf	last	singleC	OT, OT-A
632	No	9	9-B	200	Bozic	Inf	last	wholeT_0.01	CBN, OT, OT-A
633	No	9	9-B	200	Bozic	Inf	last	wholeT_0.5	OT, OT-A
634	No	9	9-B	200	Bozic	Inf	unif	singleC	CBN, CBN-A
635	No	9	9-B	200	Bozic	Inf	unif	wholeT_0.01	CBN-A
636	No	9	9-B	200	Bozic	Inf	unif	wholeT_0.5	CBN, CBN-A
637	No	9	9-B	200	exp	0	last	singleC	CBN, CBN-A

Table 11: *(continued)*

	Conjunction	Drivers	Tree	S.Size	Model	sh	S.Time	S.Type	Best method(s)
638	No	9	9-B	200	exp	0	last	wholeT_0.01	CBN, CBN-A
639	No	9	9-B	200	exp	0	last	wholeT_0.5	CBN, CBN-A
640	No	9	9-B	200	exp	0	unif	singleC	CBN, CBN-A
641	No	9	9-B	200	exp	0	unif	wholeT_0.01	CBN, CBN-A
642	No	9	9-B	200	exp	0	unif	wholeT_0.5	CBN, CBN-A
643	No	9	9-B	200	exp	Inf	last	singleC	CBN, CBN-A, OT, OT-A
644	No	9	9-B	200	exp	Inf	last	wholeT_0.01	OT, OT-A
645	No	9	9-B	200	exp	Inf	last	wholeT_0.5	CBN, CBN-A, OT, OT-A
646	No	9	9-B	200	exp	Inf	unif	singleC	CBN, CBN-A
647	No	9	9-B	200	exp	Inf	unif	wholeT_0.01	CBN, CBN-A
648	No	9	9-B	200	exp	Inf	unif	wholeT_0.5	CBN, CBN-A
649	No	9	9-B	200	McF_4	0	last	singleC	OT, OT-A
650	No	9	9-B	200	McF_4	0	last	wholeT_0.01	OT, OT-A
651	No	9	9-B	200	McF_4	0	last	wholeT_0.5	OT, OT-A
652	No	9	9-B	200	McF_4	0	unif	singleC	CBN, CBN-A, OT, OT-A
653	No	9	9-B	200	McF_4	0	unif	wholeT_0.01	CBN, OT, OT-A
654	No	9	9-B	200	McF_4	0	unif	wholeT_0.5	CBN, CBN-A, OT, OT-A
655	No	9	9-B	200	McF_4	Inf	last	singleC	OT, OT-A
656	No	9	9-B	200	McF_4	Inf	last	wholeT_0.01	OT, OT-A
657	No	9	9-B	200	McF_4	Inf	last	wholeT_0.5	OT, OT-A
658	No	9	9-B	200	McF_4	Inf	unif	singleC	CBN, CBN-A, OT, OT-A
659	No	9	9-B	200	McF_4	Inf	unif	wholeT_0.01	CBN, CBN-A, OT, OT-A
660	No	9	9-B	200	McF_4	Inf	unif	wholeT_0.5	CBN, CBN-A, OT, OT-A
661	No	9	9-B	200	McF_6	0	last	singleC	OT-A
662	No	9	9-B	200	McF_6	0	last	wholeT_0.01	OT-A
663	No	9	9-B	200	McF_6	0	last	wholeT_0.5	OT-A
664	No	9	9-B	200	McF_6	0	unif	singleC	CBN-A, OT, OT-A
665	No	9	9-B	200	McF_6	0	unif	wholeT_0.01	OT, OT-A
666	No	9	9-B	200	McF_6	0	unif	wholeT_0.5	OT, OT-A
667	No	9	9-B	200	McF_6	Inf	last	singleC	OT-A
668	No	9	9-B	200	McF_6	Inf	last	wholeT_0.01	OT-A
669	No	9	9-B	200	McF_6	Inf	last	wholeT_0.5	OT-A
670	No	9	9-B	200	McF_6	Inf	unif	singleC	OT, OT-A
671	No	9	9-B	200	McF_6	Inf	unif	wholeT_0.01	OT, OT-A
672	No	9	9-B	200	McF_6	Inf	unif	wholeT_0.5	OT, OT-A
673	No	9	9-B	100	Bozic	0	last	singleC	CBN, CBN-A
674	No	9	9-B	100	Bozic	0	last	wholeT_0.01	CBN, CBN-A
675	No	9	9-B	100	Bozic	0	last	wholeT_0.5	CBN, CBN-A
676	No	9	9-B	100	Bozic	0	unif	singleC	CBN, CBN-A
677	No	9	9-B	100	Bozic	0	unif	wholeT_0.01	CBN, CBN-A

Table 11: *(continued)*

	Conjunction	Drivers	Tree	S.Size	Model	sh	S.Time	S.Type	Best method(s)
678	No	9	9-B	100	Bozic	0	unif	wholeT_0.5	CBN, CBN-A
679	No	9	9-B	100	Bozic	Inf	last	singleC	CBN-A, OT, OT-A
680	No	9	9-B	100	Bozic	Inf	last	wholeT_0.01	OT, OT-A
681	No	9	9-B	100	Bozic	Inf	last	wholeT_0.5	CBN, CBN-A, OT, OT-A
682	No	9	9-B	100	Bozic	Inf	unif	singleC	CBN, CBN-A
683	No	9	9-B	100	Bozic	Inf	unif	wholeT_0.01	CBN, CBN-A
684	No	9	9-B	100	Bozic	Inf	unif	wholeT_0.5	CBN, CBN-A
685	No	9	9-B	100	exp	0	last	singleC	CBN, CBN-A
686	No	9	9-B	100	exp	0	last	wholeT_0.01	CBN, CBN-A
687	No	9	9-B	100	exp	0	last	wholeT_0.5	CBN, CBN-A
688	No	9	9-B	100	exp	0	unif	singleC	CBN, CBN-A
689	No	9	9-B	100	exp	0	unif	wholeT_0.01	CBN, CBN-A
690	No	9	9-B	100	exp	0	unif	wholeT_0.5	CBN, CBN-A
691	No	9	9-B	100	exp	Inf	last	singleC	CBN, CBN-A
692	No	9	9-B	100	exp	Inf	last	wholeT_0.01	CBN, CBN-A, OT, OT-A
693	No	9	9-B	100	exp	Inf	last	wholeT_0.5	CBN, CBN-A
694	No	9	9-B	100	exp	Inf	unif	singleC	CBN, CBN-A
695	No	9	9-B	100	exp	Inf	unif	wholeT_0.01	CBN, CBN-A
696	No	9	9-B	100	exp	Inf	unif	wholeT_0.5	CBN, CBN-A
697	No	9	9-B	100	McF_4	0	last	singleC	OT, OT-A
698	No	9	9-B	100	McF_4	0	last	wholeT_0.01	OT, OT-A
699	No	9	9-B	100	McF_4	0	last	wholeT_0.5	OT, OT-A
700	No	9	9-B	100	McF_4	0	unif	singleC	CBN, CBN-A, OT, OT-A
701	No	9	9-B	100	McF_4	0	unif	wholeT_0.01	CBN, CBN-A, OT, OT-A
702	No	9	9-B	100	McF_4	0	unif	wholeT_0.5	CBN, CBN-A
703	No	9	9-B	100	McF_4	Inf	last	singleC	OT, OT-A
704	No	9	9-B	100	McF_4	Inf	last	wholeT_0.01	OT, OT-A
705	No	9	9-B	100	McF_4	Inf	last	wholeT_0.5	OT, OT-A
706	No	9	9-B	100	McF_4	Inf	unif	singleC	CBN, CBN-A, OT, OT-A
707	No	9	9-B	100	McF_4	Inf	unif	wholeT_0.01	CBN, CBN-A, OT, OT-A
708	No	9	9-B	100	McF_4	Inf	unif	wholeT_0.5	CBN, CBN-A, OT
709	No	9	9-B	100	McF_6	0	last	singleC	OT-A
710	No	9	9-B	100	McF_6	0	last	wholeT_0.01	OT-A
711	No	9	9-B	100	McF_6	0	last	wholeT_0.5	OT-A
712	No	9	9-B	100	McF_6	0	unif	singleC	OT, OT-A
713	No	9	9-B	100	McF_6	0	unif	wholeT_0.01	CBN, OT, OT-A
714	No	9	9-B	100	McF_6	0	unif	wholeT_0.5	OT, OT-A
715	No	9	9-B	100	McF_6	Inf	last	singleC	OT-A
716	No	9	9-B	100	McF_6	Inf	last	wholeT_0.01	OT-A
717	No	9	9-B	100	McF_6	Inf	last	wholeT_0.5	OT-A

Table 11: *(continued)*

	Conjunction	Drivers	Tree	S.Size	Model	sh	S.Time	S.Type	Best method(s)
718	No	9	9-B	100	McF_6	Inf	unif	singleC	OT, OT-A
719	No	9	9-B	100	McF_6	Inf	unif	wholeT_0.01	OT, OT-A
720	No	9	9-B	100	McF_6	Inf	unif	wholeT_0.5	OT, OT-A
721	No	7	7-B	1000	Bozic	0	last	singleC	OT-A
722	No	7	7-B	1000	Bozic	0	last	wholeT_0.01	OT-A
723	No	7	7-B	1000	Bozic	0	last	wholeT_0.5	OT-A
724	No	7	7-B	1000	Bozic	0	unif	singleC	CBN, CBN-A, OT, OT-A
725	No	7	7-B	1000	Bozic	0	unif	wholeT_0.01	CBN, CBN-A, DiP-A, OT, OT-A
726	No	7	7-B	1000	Bozic	0	unif	wholeT_0.5	CBN, CBN-A, OT, OT-A
727	No	7	7-B	1000	Bozic	Inf	last	singleC	DiP-A, OT-A
728	No	7	7-B	1000	Bozic	Inf	last	wholeT_0.01	DiP-A, OT-A
729	No	7	7-B	1000	Bozic	Inf	last	wholeT_0.5	DiP-A, OT-A
730	No	7	7-B	1000	Bozic	Inf	unif	singleC	OT, OT-A
731	No	7	7-B	1000	Bozic	Inf	unif	wholeT_0.01	CBN, CBN-A, OT, OT-A
732	No	7	7-B	1000	Bozic	Inf	unif	wholeT_0.5	OT, OT-A
733	No	7	7-B	1000	exp	0	last	singleC	CBN-A
734	No	7	7-B	1000	exp	0	last	wholeT_0.01	CBN, CBN-A, OT-A
735	No	7	7-B	1000	exp	0	last	wholeT_0.5	CBN-A
736	No	7	7-B	1000	exp	0	unif	singleC	CBN, CBN-A
737	No	7	7-B	1000	exp	0	unif	wholeT_0.01	CBN, CBN-A, OT-A
738	No	7	7-B	1000	exp	0	unif	wholeT_0.5	CBN, CBN-A
739	No	7	7-B	1000	exp	Inf	last	singleC	OT-A
740	No	7	7-B	1000	exp	Inf	last	wholeT_0.01	OT-A
741	No	7	7-B	1000	exp	Inf	last	wholeT_0.5	OT-A
742	No	7	7-B	1000	exp	Inf	unif	singleC	CBN, CBN-A, OT, OT-A
743	No	7	7-B	1000	exp	Inf	unif	wholeT_0.01	CBN-A, OT, OT-A
744	No	7	7-B	1000	exp	Inf	unif	wholeT_0.5	CBN, CBN-A
745	No	7	7-B	1000	McF_4	0	last	singleC	DiP-A, OT-A
746	No	7	7-B	1000	McF_4	0	last	wholeT_0.01	DiP-A, OT-A
747	No	7	7-B	1000	McF_4	0	last	wholeT_0.5	DiP-A
748	No	7	7-B	1000	McF_4	0	unif	singleC	DiP, DiP-A, OT, OT-A
749	No	7	7-B	1000	McF_4	0	unif	wholeT_0.01	DiP-A, OT, OT-A
750	No	7	7-B	1000	McF_4	0	unif	wholeT_0.5	DiP-A, OT, OT-A
751	No	7	7-B	1000	McF_4	Inf	last	singleC	DiP-A, OT-A
752	No	7	7-B	1000	McF_4	Inf	last	wholeT_0.01	DiP-A, OT-A
753	No	7	7-B	1000	McF_4	Inf	last	wholeT_0.5	DiP-A, OT-A
754	No	7	7-B	1000	McF_4	Inf	unif	singleC	OT, OT-A
755	No	7	7-B	1000	McF_4	Inf	unif	wholeT_0.01	OT, OT-A
756	No	7	7-B	1000	McF_4	Inf	unif	wholeT_0.5	OT, OT-A
757	No	7	7-B	1000	McF_6	0	last	singleC	DiP-A, OT-A

Table 11: *(continued)*

	Conjunction	Drivers	Tree	S.Size	Model	sh	S.Time	S.Type	Best method(s)
758	No	7	7-B	1000	McF_6	0	last	wholeT_0.01	DiP-A, OT-A
759	No	7	7-B	1000	McF_6	0	last	wholeT_0.5	DiP-A, OT-A
760	No	7	7-B	1000	McF_6	0	unif	singleC	DiP, DiP-A, OT, OT-A
761	No	7	7-B	1000	McF_6	0	unif	wholeT_0.01	DiP-A, OT, OT-A
762	No	7	7-B	1000	McF_6	0	unif	wholeT_0.5	DiP, DiP-A, OT, OT-A
763	No	7	7-B	1000	McF_6	Inf	last	singleC	DiP-A, OT-A
764	No	7	7-B	1000	McF_6	Inf	last	wholeT_0.01	DiP-A, OT-A
765	No	7	7-B	1000	McF_6	Inf	last	wholeT_0.5	DiP-A, OT-A
766	No	7	7-B	1000	McF_6	Inf	unif	singleC	DiP-A, OT, OT-A
767	No	7	7-B	1000	McF_6	Inf	unif	wholeT_0.01	DiP-A, OT, OT-A
768	No	7	7-B	1000	McF_6	Inf	unif	wholeT_0.5	DiP-A, OT, OT-A
769	No	7	7-B	200	Bozic	0	last	singleC	CBN, OT-A
770	No	7	7-B	200	Bozic	0	last	wholeT_0.01	OT-A
771	No	7	7-B	200	Bozic	0	last	wholeT_0.5	CBN, CBN-A, OT-A
772	No	7	7-B	200	Bozic	0	unif	singleC	CBN, CBN-A
773	No	7	7-B	200	Bozic	0	unif	wholeT_0.01	CBN, CBN-A, OT, OT-A
774	No	7	7-B	200	Bozic	0	unif	wholeT_0.5	CBN, CBN-A
775	No	7	7-B	200	Bozic	Inf	last	singleC	OT-A
776	No	7	7-B	200	Bozic	Inf	last	wholeT_0.01	OT-A
777	No	7	7-B	200	Bozic	Inf	last	wholeT_0.5	OT-A
778	No	7	7-B	200	Bozic	Inf	unif	singleC	CBN, CBN-A, OT, OT-A
779	No	7	7-B	200	Bozic	Inf	unif	wholeT_0.01	CBN, CBN-A, OT, OT-A
780	No	7	7-B	200	Bozic	Inf	unif	wholeT_0.5	CBN, CBN-A
781	No	7	7-B	200	exp	0	last	singleC	CBN-A
782	No	7	7-B	200	exp	0	last	wholeT_0.01	CBN, CBN-A, OT-A
783	No	7	7-B	200	exp	0	last	wholeT_0.5	CBN, CBN-A
784	No	7	7-B	200	exp	0	unif	singleC	CBN, CBN-A
785	No	7	7-B	200	exp	0	unif	wholeT_0.01	CBN, CBN-A
786	No	7	7-B	200	exp	0	unif	wholeT_0.5	CBN
787	No	7	7-B	200	exp	Inf	last	singleC	OT-A
788	No	7	7-B	200	exp	Inf	last	wholeT_0.01	OT-A
789	No	7	7-B	200	exp	Inf	last	wholeT_0.5	OT-A
790	No	7	7-B	200	exp	Inf	unif	singleC	CBN, CBN-A
791	No	7	7-B	200	exp	Inf	unif	wholeT_0.01	CBN, CBN-A
792	No	7	7-B	200	exp	Inf	unif	wholeT_0.5	CBN, CBN-A
793	No	7	7-B	200	McF_4	0	last	singleC	OT-A
794	No	7	7-B	200	McF_4	0	last	wholeT_0.01	OT-A
795	No	7	7-B	200	McF_4	0	last	wholeT_0.5	OT-A
796	No	7	7-B	200	McF_4	0	unif	singleC	OT, OT-A
797	No	7	7-B	200	McF_4	0	unif	wholeT_0.01	OT, OT-A

Table 11: *(continued)*

	Conjunction	Drivers	Tree	S.Size	Model	sh	S.Time	S.Type	Best method(s)
798	No	7	7-B	200	McF_4	0	unif	wholeT_0.5	OT, OT-A
799	No	7	7-B	200	McF_4	Inf	last	singleC	OT-A
800	No	7	7-B	200	McF_4	Inf	last	wholeT_0.01	OT-A
801	No	7	7-B	200	McF_4	Inf	last	wholeT_0.5	OT-A
802	No	7	7-B	200	McF_4	Inf	unif	singleC	OT, OT-A
803	No	7	7-B	200	McF_4	Inf	unif	wholeT_0.01	OT, OT-A
804	No	7	7-B	200	McF_4	Inf	unif	wholeT_0.5	OT, OT-A
805	No	7	7-B	200	McF_6	0	last	singleC	OT-A
806	No	7	7-B	200	McF_6	0	last	wholeT_0.01	OT-A
807	No	7	7-B	200	McF_6	0	last	wholeT_0.5	OT-A
808	No	7	7-B	200	McF_6	0	unif	singleC	OT, OT-A
809	No	7	7-B	200	McF_6	0	unif	wholeT_0.01	OT, OT-A
810	No	7	7-B	200	McF_6	0	unif	wholeT_0.5	OT, OT-A
811	No	7	7-B	200	McF_6	Inf	last	singleC	OT-A
812	No	7	7-B	200	McF_6	Inf	last	wholeT_0.01	OT-A
813	No	7	7-B	200	McF_6	Inf	last	wholeT_0.5	OT-A
814	No	7	7-B	200	McF_6	Inf	unif	singleC	OT, OT-A
815	No	7	7-B	200	McF_6	Inf	unif	wholeT_0.01	OT, OT-A
816	No	7	7-B	200	McF_6	Inf	unif	wholeT_0.5	OT, OT-A
817	No	7	7-B	100	Bozic	0	last	singleC	CBN, CBN-A, OT-A
818	No	7	7-B	100	Bozic	0	last	wholeT_0.01	OT-A
819	No	7	7-B	100	Bozic	0	last	wholeT_0.5	CBN, CBN-A, OT-A
820	No	7	7-B	100	Bozic	0	unif	singleC	CBN, CBN-A
821	No	7	7-B	100	Bozic	0	unif	wholeT_0.01	CBN, CBN-A, OT-A
822	No	7	7-B	100	Bozic	0	unif	wholeT_0.5	CBN, CBN-A
823	No	7	7-B	100	Bozic	Inf	last	singleC	OT-A
824	No	7	7-B	100	Bozic	Inf	last	wholeT_0.01	OT-A
825	No	7	7-B	100	Bozic	Inf	last	wholeT_0.5	OT-A
826	No	7	7-B	100	Bozic	Inf	unif	singleC	CBN, CBN-A
827	No	7	7-B	100	Bozic	Inf	unif	wholeT_0.01	CBN, CBN-A, OT-A
828	No	7	7-B	100	Bozic	Inf	unif	wholeT_0.5	CBN, CBN-A
829	No	7	7-B	100	exp	0	last	singleC	CBN-A
830	No	7	7-B	100	exp	0	last	wholeT_0.01	CBN, CBN-A
831	No	7	7-B	100	exp	0	last	wholeT_0.5	CBN, CBN-A
832	No	7	7-B	100	exp	0	unif	singleC	CBN, CBN-A
833	No	7	7-B	100	exp	0	unif	wholeT_0.01	CBN, CBN-A
834	No	7	7-B	100	exp	0	unif	wholeT_0.5	CBN, CBN-A
835	No	7	7-B	100	exp	Inf	last	singleC	OT-A
836	No	7	7-B	100	exp	Inf	last	wholeT_0.01	OT-A
837	No	7	7-B	100	exp	Inf	last	wholeT_0.5	CBN, OT-A

Table 11: *(continued)*

	Conjunction	Drivers	Tree	S.Size	Model	sh	S.Time	S.Type	Best method(s)
838	No	7	7-B	100	exp	Inf	unif	singleC	CBN, CBN-A
839	No	7	7-B	100	exp	Inf	unif	wholeT_0.01	CBN, CBN-A
840	No	7	7-B	100	exp	Inf	unif	wholeT_0.5	CBN, CBN-A
841	No	7	7-B	100	McF_4	0	last	singleC	OT-A
842	No	7	7-B	100	McF_4	0	last	wholeT_0.01	OT-A
843	No	7	7-B	100	McF_4	0	last	wholeT_0.5	OT-A
844	No	7	7-B	100	McF_4	0	unif	singleC	OT-A
845	No	7	7-B	100	McF_4	0	unif	wholeT_0.01	OT, OT-A
846	No	7	7-B	100	McF_4	0	unif	wholeT_0.5	OT, OT-A
847	No	7	7-B	100	McF_4	Inf	last	singleC	OT-A
848	No	7	7-B	100	McF_4	Inf	last	wholeT_0.01	OT-A
849	No	7	7-B	100	McF_4	Inf	last	wholeT_0.5	OT-A
850	No	7	7-B	100	McF_4	Inf	unif	singleC	OT, OT-A
851	No	7	7-B	100	McF_4	Inf	unif	wholeT_0.01	OT, OT-A
852	No	7	7-B	100	McF_4	Inf	unif	wholeT_0.5	OT, OT-A
853	No	7	7-B	100	McF_6	0	last	singleC	OT-A
854	No	7	7-B	100	McF_6	0	last	wholeT_0.01	OT-A
855	No	7	7-B	100	McF_6	0	last	wholeT_0.5	OT-A
856	No	7	7-B	100	McF_6	0	unif	singleC	OT, OT-A
857	No	7	7-B	100	McF_6	0	unif	wholeT_0.01	OT, OT-A
858	No	7	7-B	100	McF_6	0	unif	wholeT_0.5	OT-A
859	No	7	7-B	100	McF_6	Inf	last	singleC	OT-A
860	No	7	7-B	100	McF_6	Inf	last	wholeT_0.01	OT-A
861	No	7	7-B	100	McF_6	Inf	last	wholeT_0.5	OT-A
862	No	7	7-B	100	McF_6	Inf	unif	singleC	OT-A
863	No	7	7-B	100	McF_6	Inf	unif	wholeT_0.01	OT-A
864	No	7	7-B	100	McF_6	Inf	unif	wholeT_0.5	OT, OT-A

4.4 Confidence sets (MCB), FPF, Drivers Known

Table 12: Confidence sets (method MCB) when Drivers are Known for measure FPF.

	Conjunction	Drivers	Tree	S.Size	Model	sh	S.Time	S.Type	Best method(s)
1	Yes	11	11-A	1000	Bozic	0	last	singleC	DiP-A, OT, OT-A
2	Yes	11	11-A	1000	Bozic	0	last	wholeT_0.01	DiP, DiP-A
3	Yes	11	11-A	1000	Bozic	0	last	wholeT_0.5	DiP, DiP-A, OT, OT-A
4	Yes	11	11-A	1000	Bozic	0	unif	singleC	DiP, DiP-A
5	Yes	11	11-A	1000	Bozic	0	unif	wholeT_0.01	DiP-A
6	Yes	11	11-A	1000	Bozic	0	unif	wholeT_0.5	DiP, DiP-A
7	Yes	11	11-A	1000	Bozic	Inf	last	singleC	OT, OT-A
8	Yes	11	11-A	1000	Bozic	Inf	last	wholeT_0.01	DiP, DiP-A
9	Yes	11	11-A	1000	Bozic	Inf	last	wholeT_0.5	OT, OT-A
10	Yes	11	11-A	1000	Bozic	Inf	unif	singleC	DiP, DiP-A, OT, OT-A
11	Yes	11	11-A	1000	Bozic	Inf	unif	wholeT_0.01	DiP, OT, OT-A
12	Yes	11	11-A	1000	Bozic	Inf	unif	wholeT_0.5	DiP, DiP-A, OT, OT-A
13	Yes	11	11-A	1000	exp	0	last	singleC	DiP, DiP-A, OT, OT-A
14	Yes	11	11-A	1000	exp	0	last	wholeT_0.01	DiP-A, OT, OT-A
15	Yes	11	11-A	1000	exp	0	last	wholeT_0.5	DiP, DiP-A, OT, OT-A
16	Yes	11	11-A	1000	exp	0	unif	singleC	DiP, DiP-A, OT, OT-A
17	Yes	11	11-A	1000	exp	0	unif	wholeT_0.01	DiP, DiP-A
18	Yes	11	11-A	1000	exp	0	unif	wholeT_0.5	DiP, DiP-A, OT, OT-A
19	Yes	11	11-A	1000	exp	Inf	last	singleC	DiP, DiP-A, OT, OT-A
20	Yes	11	11-A	1000	exp	Inf	last	wholeT_0.01	OT, OT-A
21	Yes	11	11-A	1000	exp	Inf	last	wholeT_0.5	DiP, DiP-A, OT, OT-A
22	Yes	11	11-A	1000	exp	Inf	unif	singleC	DiP, DiP-A, OT, OT-A
23	Yes	11	11-A	1000	exp	Inf	unif	wholeT_0.01	DiP, DiP-A, OT, OT-A
24	Yes	11	11-A	1000	exp	Inf	unif	wholeT_0.5	DiP, DiP-A, OT, OT-A
25	Yes	11	11-A	1000	McF_4	0	last	singleC	DiP, DiP-A, OT, OT-A
26	Yes	11	11-A	1000	McF_4	0	last	wholeT_0.01	DiP-A, OT, OT-A
27	Yes	11	11-A	1000	McF_4	0	last	wholeT_0.5	DiP-A, OT, OT-A
28	Yes	11	11-A	1000	McF_4	0	unif	singleC	DiP, DiP-A
29	Yes	11	11-A	1000	McF_4	0	unif	wholeT_0.01	DiP-A
30	Yes	11	11-A	1000	McF_4	0	unif	wholeT_0.5	DiP, DiP-A
31	Yes	11	11-A	1000	McF_4	Inf	last	singleC	OT, OT-A
32	Yes	11	11-A	1000	McF_4	Inf	last	wholeT_0.01	OT, OT-A
33	Yes	11	11-A	1000	McF_4	Inf	last	wholeT_0.5	DiP-A, OT, OT-A
34	Yes	11	11-A	1000	McF_4	Inf	unif	singleC	DiP, DiP-A, OT, OT-A
35	Yes	11	11-A	1000	McF_4	Inf	unif	wholeT_0.01	DiP-A, OT, OT-A
36	Yes	11	11-A	1000	McF_4	Inf	unif	wholeT_0.5	DiP, DiP-A, OT, OT-A
37	Yes	11	11-A	1000	McF_6	0	last	singleC	DiP, OT

Table 12: *(continued)*

	Conjunction	Drivers	Tree	S.Size	Model	sh	S.Time	S.Type	Best method(s)
38	Yes	11	11-A	1000	McF_6	0	last	wholeT_0.01	DiP, OT
39	Yes	11	11-A	1000	McF_6	0	last	wholeT_0.5	DiP, OT
40	Yes	11	11-A	1000	McF_6	0	unif	singleC	DiP, DiP-A, OT-A
41	Yes	11	11-A	1000	McF_6	0	unif	wholeT_0.01	DiP, DiP-A, OT, OT-A
42	Yes	11	11-A	1000	McF_6	0	unif	wholeT_0.5	DiP, DiP-A, OT
43	Yes	11	11-A	1000	McF_6	Inf	last	singleC	DiP, OT
44	Yes	11	11-A	1000	McF_6	Inf	last	wholeT_0.01	DiP, OT
45	Yes	11	11-A	1000	McF_6	Inf	last	wholeT_0.5	DiP, OT
46	Yes	11	11-A	1000	McF_6	Inf	unif	singleC	DiP, DiP-A
47	Yes	11	11-A	1000	McF_6	Inf	unif	wholeT_0.01	DiP, DiP-A
48	Yes	11	11-A	1000	McF_6	Inf	unif	wholeT_0.5	DiP, DiP-A
49	Yes	11	11-A	200	Bozic	0	last	singleC	DiP, DiP-A, OT, OT-A
50	Yes	11	11-A	200	Bozic	0	last	wholeT_0.01	DiP, DiP-A
51	Yes	11	11-A	200	Bozic	0	last	wholeT_0.5	DiP, DiP-A, OT, OT-A
52	Yes	11	11-A	200	Bozic	0	unif	singleC	DiP, DiP-A
53	Yes	11	11-A	200	Bozic	0	unif	wholeT_0.01	DiP, DiP-A
54	Yes	11	11-A	200	Bozic	0	unif	wholeT_0.5	DiP, DiP-A, OT, OT-A
55	Yes	11	11-A	200	Bozic	Inf	last	singleC	DiP, DiP-A, OT, OT-A
56	Yes	11	11-A	200	Bozic	Inf	last	wholeT_0.01	DiP, DiP-A, OT, OT-A
57	Yes	11	11-A	200	Bozic	Inf	last	wholeT_0.5	DiP, DiP-A, OT, OT-A
58	Yes	11	11-A	200	Bozic	Inf	unif	singleC	DiP, DiP-A
59	Yes	11	11-A	200	Bozic	Inf	unif	wholeT_0.01	DiP, DiP-A, OT, OT-A
60	Yes	11	11-A	200	Bozic	Inf	unif	wholeT_0.5	DiP, DiP-A
61	Yes	11	11-A	200	exp	0	last	singleC	DiP, DiP-A, OT, OT-A
62	Yes	11	11-A	200	exp	0	last	wholeT_0.01	DiP, DiP-A, OT, OT-A
63	Yes	11	11-A	200	exp	0	last	wholeT_0.5	DiP, DiP-A, OT, OT-A
64	Yes	11	11-A	200	exp	0	unif	singleC	DiP, DiP-A, OT, OT-A
65	Yes	11	11-A	200	exp	0	unif	wholeT_0.01	DiP, DiP-A
66	Yes	11	11-A	200	exp	0	unif	wholeT_0.5	DiP, DiP-A, OT, OT-A
67	Yes	11	11-A	200	exp	Inf	last	singleC	DiP, DiP-A, OT, OT-A
68	Yes	11	11-A	200	exp	Inf	last	wholeT_0.01	DiP, DiP-A, OT, OT-A
69	Yes	11	11-A	200	exp	Inf	last	wholeT_0.5	DiP, DiP-A
70	Yes	11	11-A	200	exp	Inf	unif	singleC	DiP, DiP-A, OT, OT-A
71	Yes	11	11-A	200	exp	Inf	unif	wholeT_0.01	DiP, DiP-A
72	Yes	11	11-A	200	exp	Inf	unif	wholeT_0.5	DiP, DiP-A, OT-A
73	Yes	11	11-A	200	McF_4	0	last	singleC	DiP, DiP-A, OT, OT-A
74	Yes	11	11-A	200	McF_4	0	last	wholeT_0.01	DiP-A, OT, OT-A
75	Yes	11	11-A	200	McF_4	0	last	wholeT_0.5	DiP, DiP-A, OT, OT-A
76	Yes	11	11-A	200	McF_4	0	unif	singleC	DiP, DiP-A, OT, OT-A
77	Yes	11	11-A	200	McF_4	0	unif	wholeT_0.01	DiP, DiP-A

Table 12: *(continued)*

	Conjunction	Drivers	Tree	S.Size	Model	sh	S.Time	S.Type	Best method(s)
78	Yes	11	11-A	200	McF_4	0	unif	wholeT_0.5	DiP, DiP-A
79	Yes	11	11-A	200	McF_4	Inf	last	singleC	DiP, DiP-A, OT, OT-A
80	Yes	11	11-A	200	McF_4	Inf	last	wholeT_0.01	OT, OT-A
81	Yes	11	11-A	200	McF_4	Inf	last	wholeT_0.5	DiP, DiP-A, OT, OT-A
82	Yes	11	11-A	200	McF_4	Inf	unif	singleC	DiP, DiP-A, OT, OT-A
83	Yes	11	11-A	200	McF_4	Inf	unif	wholeT_0.01	DiP, DiP-A, OT, OT-A
84	Yes	11	11-A	200	McF_4	Inf	unif	wholeT_0.5	DiP, DiP-A, OT, OT-A
85	Yes	11	11-A	200	McF_6	0	last	singleC	DiP, OT
86	Yes	11	11-A	200	McF_6	0	last	wholeT_0.01	DiP, OT
87	Yes	11	11-A	200	McF_6	0	last	wholeT_0.5	DiP, OT
88	Yes	11	11-A	200	McF_6	0	unif	singleC	DiP, OT, OT-A
89	Yes	11	11-A	200	McF_6	0	unif	wholeT_0.01	DiP, DiP-A, OT, OT-A
90	Yes	11	11-A	200	McF_6	0	unif	wholeT_0.5	DiP, OT, OT-A
91	Yes	11	11-A	200	McF_6	Inf	last	singleC	DiP, OT
92	Yes	11	11-A	200	McF_6	Inf	last	wholeT_0.01	DiP, OT
93	Yes	11	11-A	200	McF_6	Inf	last	wholeT_0.5	DiP, OT
94	Yes	11	11-A	200	McF_6	Inf	unif	singleC	OT, OT-A
95	Yes	11	11-A	200	McF_6	Inf	unif	wholeT_0.01	OT, OT-A
96	Yes	11	11-A	200	McF_6	Inf	unif	wholeT_0.5	OT, OT-A
97	Yes	11	11-A	100	Bozic	0	last	singleC	DiP, DiP-A, OT
98	Yes	11	11-A	100	Bozic	0	last	wholeT_0.01	DiP, DiP-A
99	Yes	11	11-A	100	Bozic	0	last	wholeT_0.5	DiP, DiP-A, OT, OT-A
100	Yes	11	11-A	100	Bozic	0	unif	singleC	DiP, DiP-A, OT
101	Yes	11	11-A	100	Bozic	0	unif	wholeT_0.01	DiP, DiP-A, OT, OT-A
102	Yes	11	11-A	100	Bozic	0	unif	wholeT_0.5	DiP, DiP-A, OT, OT-A
103	Yes	11	11-A	100	Bozic	Inf	last	singleC	DiP, DiP-A, OT, OT-A
104	Yes	11	11-A	100	Bozic	Inf	last	wholeT_0.01	DiP, DiP-A
105	Yes	11	11-A	100	Bozic	Inf	last	wholeT_0.5	DiP, DiP-A, OT-A
106	Yes	11	11-A	100	Bozic	Inf	unif	singleC	DiP, DiP-A, OT, OT-A
107	Yes	11	11-A	100	Bozic	Inf	unif	wholeT_0.01	DiP, DiP-A
108	Yes	11	11-A	100	Bozic	Inf	unif	wholeT_0.5	DiP, DiP-A
109	Yes	11	11-A	100	exp	0	last	singleC	DiP, DiP-A, OT-A
110	Yes	11	11-A	100	exp	0	last	wholeT_0.01	DiP, DiP-A, OT, OT-A
111	Yes	11	11-A	100	exp	0	last	wholeT_0.5	DiP, DiP-A, OT, OT-A
112	Yes	11	11-A	100	exp	0	unif	singleC	DiP, DiP-A, OT, OT-A
113	Yes	11	11-A	100	exp	0	unif	wholeT_0.01	DiP, DiP-A
114	Yes	11	11-A	100	exp	0	unif	wholeT_0.5	DiP, DiP-A, OT, OT-A
115	Yes	11	11-A	100	exp	Inf	last	singleC	DiP, DiP-A, OT
116	Yes	11	11-A	100	exp	Inf	last	wholeT_0.01	DiP, DiP-A, OT, OT-A
117	Yes	11	11-A	100	exp	Inf	last	wholeT_0.5	DiP, DiP-A, OT, OT-A

Table 12: *(continued)*

	Conjunction	Drivers	Tree	S.Size	Model	sh	S.Time	S.Type	Best method(s)
118	Yes	11	11-A	100	exp	Inf	unif	singleC	DiP, DiP-A, OT, OT-A
119	Yes	11	11-A	100	exp	Inf	unif	wholeT_0.01	DiP, DiP-A
120	Yes	11	11-A	100	exp	Inf	unif	wholeT_0.5	DiP, DiP-A, OT, OT-A
121	Yes	11	11-A	100	McF_4	0	last	singleC	DiP, DiP-A
122	Yes	11	11-A	100	McF_4	0	last	wholeT_0.01	DiP, DiP-A
123	Yes	11	11-A	100	McF_4	0	last	wholeT_0.5	DiP, DiP-A, OT, OT-A
124	Yes	11	11-A	100	McF_4	0	unif	singleC	DiP, DiP-A, OT-A
125	Yes	11	11-A	100	McF_4	0	unif	wholeT_0.01	DiP, DiP-A
126	Yes	11	11-A	100	McF_4	0	unif	wholeT_0.5	DiP, DiP-A, OT, OT-A
127	Yes	11	11-A	100	McF_4	Inf	last	singleC	DiP, DiP-A, OT, OT-A
128	Yes	11	11-A	100	McF_4	Inf	last	wholeT_0.01	DiP, DiP-A
129	Yes	11	11-A	100	McF_4	Inf	last	wholeT_0.5	DiP, DiP-A, OT, OT-A
130	Yes	11	11-A	100	McF_4	Inf	unif	singleC	DiP, DiP-A, OT, OT-A
131	Yes	11	11-A	100	McF_4	Inf	unif	wholeT_0.01	DiP
132	Yes	11	11-A	100	McF_4	Inf	unif	wholeT_0.5	DiP, DiP-A, OT, OT-A
133	Yes	11	11-A	100	McF_6	0	last	singleC	DiP
134	Yes	11	11-A	100	McF_6	0	last	wholeT_0.01	DiP, OT
135	Yes	11	11-A	100	McF_6	0	last	wholeT_0.5	DiP, OT
136	Yes	11	11-A	100	McF_6	0	unif	singleC	DiP, DiP-A, OT, OT-A
137	Yes	11	11-A	100	McF_6	0	unif	wholeT_0.01	DiP, DiP-A
138	Yes	11	11-A	100	McF_6	0	unif	wholeT_0.5	DiP, DiP-A, OT, OT-A
139	Yes	11	11-A	100	McF_6	Inf	last	singleC	DiP, OT
140	Yes	11	11-A	100	McF_6	Inf	last	wholeT_0.01	DiP
141	Yes	11	11-A	100	McF_6	Inf	last	wholeT_0.5	DiP, OT
142	Yes	11	11-A	100	McF_6	Inf	unif	singleC	DiP, DiP-A, OT, OT-A
143	Yes	11	11-A	100	McF_6	Inf	unif	wholeT_0.01	DiP, OT, OT-A
144	Yes	11	11-A	100	McF_6	Inf	unif	wholeT_0.5	DiP, DiP-A, OT, OT-A
145	Yes	9	9-A	1000	Bozic	0	last	singleC	DiP, DiP-A, OT, OT-A
146	Yes	9	9-A	1000	Bozic	0	last	wholeT_0.01	DiP, DiP-A
147	Yes	9	9-A	1000	Bozic	0	last	wholeT_0.5	DiP, DiP-A, OT, OT-A
148	Yes	9	9-A	1000	Bozic	0	unif	singleC	DiP, DiP-A, OT
149	Yes	9	9-A	1000	Bozic	0	unif	wholeT_0.01	DiP, DiP-A, OT-A
150	Yes	9	9-A	1000	Bozic	0	unif	wholeT_0.5	DiP, DiP-A, OT-A
151	Yes	9	9-A	1000	Bozic	Inf	last	singleC	OT, OT-A
152	Yes	9	9-A	1000	Bozic	Inf	last	wholeT_0.01	DiP, DiP-A, OT-A
153	Yes	9	9-A	1000	Bozic	Inf	last	wholeT_0.5	OT, OT-A
154	Yes	9	9-A	1000	Bozic	Inf	unif	singleC	DiP, DiP-A, OT-A
155	Yes	9	9-A	1000	Bozic	Inf	unif	wholeT_0.01	DiP, DiP-A
156	Yes	9	9-A	1000	Bozic	Inf	unif	wholeT_0.5	DiP, DiP-A, OT, OT-A
157	Yes	9	9-A	1000	exp	0	last	singleC	DiP, DiP-A, OT, OT-A

Table 12: *(continued)*

	Conjunction	Drivers	Tree	S.Size	Model	sh	S.Time	S.Type	Best method(s)
158	Yes	9	9-A	1000	exp	0	last	wholeT_0.01	DiP-A, OT, OT-A
159	Yes	9	9-A	1000	exp	0	last	wholeT_0.5	DiP, DiP-A, OT, OT-A
160	Yes	9	9-A	1000	exp	0	unif	singleC	DiP, DiP-A, OT, OT-A
161	Yes	9	9-A	1000	exp	0	unif	wholeT_0.01	DiP, DiP-A, OT
162	Yes	9	9-A	1000	exp	0	unif	wholeT_0.5	DiP, DiP-A, OT, OT-A
163	Yes	9	9-A	1000	exp	Inf	last	singleC	OT, OT-A
164	Yes	9	9-A	1000	exp	Inf	last	wholeT_0.01	DiP, DiP-A, OT, OT-A
165	Yes	9	9-A	1000	exp	Inf	last	wholeT_0.5	OT, OT-A
166	Yes	9	9-A	1000	exp	Inf	unif	singleC	DiP, DiP-A, OT, OT-A
167	Yes	9	9-A	1000	exp	Inf	unif	wholeT_0.01	OT, OT-A
168	Yes	9	9-A	1000	exp	Inf	unif	wholeT_0.5	DiP, DiP-A, OT, OT-A
169	Yes	9	9-A	1000	McF_4	0	last	singleC	DiP, DiP-A, OT, OT-A
170	Yes	9	9-A	1000	McF_4	0	last	wholeT_0.01	DiP, DiP-A, OT, OT-A
171	Yes	9	9-A	1000	McF_4	0	last	wholeT_0.5	DiP, DiP-A, OT, OT-A
172	Yes	9	9-A	1000	McF_4	0	unif	singleC	DiP-A, OT, OT-A
173	Yes	9	9-A	1000	McF_4	0	unif	wholeT_0.01	DiP, DiP-A
174	Yes	9	9-A	1000	McF_4	0	unif	wholeT_0.5	DiP, DiP-A, OT, OT-A
175	Yes	9	9-A	1000	McF_4	Inf	last	singleC	OT, OT-A
176	Yes	9	9-A	1000	McF_4	Inf	last	wholeT_0.01	DiP, OT
177	Yes	9	9-A	1000	McF_4	Inf	last	wholeT_0.5	DiP, OT, OT-A
178	Yes	9	9-A	1000	McF_4	Inf	unif	singleC	DiP, DiP-A, OT, OT-A
179	Yes	9	9-A	1000	McF_4	Inf	unif	wholeT_0.01	OT, OT-A
180	Yes	9	9-A	1000	McF_4	Inf	unif	wholeT_0.5	DiP, DiP-A, OT, OT-A
181	Yes	9	9-A	1000	McF_6	0	last	singleC	OT
182	Yes	9	9-A	1000	McF_6	0	last	wholeT_0.01	DiP, OT
183	Yes	9	9-A	1000	McF_6	0	last	wholeT_0.5	DiP, OT
184	Yes	9	9-A	1000	McF_6	0	unif	singleC	DiP, DiP-A, OT, OT-A
185	Yes	9	9-A	1000	McF_6	0	unif	wholeT_0.01	DiP, DiP-A, OT, OT-A
186	Yes	9	9-A	1000	McF_6	0	unif	wholeT_0.5	DiP, DiP-A
187	Yes	9	9-A	1000	McF_6	Inf	last	singleC	DiP, OT
188	Yes	9	9-A	1000	McF_6	Inf	last	wholeT_0.01	OT
189	Yes	9	9-A	1000	McF_6	Inf	last	wholeT_0.5	DiP, OT
190	Yes	9	9-A	1000	McF_6	Inf	unif	singleC	DiP, DiP-A, OT, OT-A
191	Yes	9	9-A	1000	McF_6	Inf	unif	wholeT_0.01	DiP, DiP-A, OT, OT-A
192	Yes	9	9-A	1000	McF_6	Inf	unif	wholeT_0.5	DiP, DiP-A, OT, OT-A
193	Yes	9	9-A	200	Bozic	0	last	singleC	DiP, DiP-A, OT, OT-A
194	Yes	9	9-A	200	Bozic	0	last	wholeT_0.01	DiP, DiP-A
195	Yes	9	9-A	200	Bozic	0	last	wholeT_0.5	DiP, DiP-A
196	Yes	9	9-A	200	Bozic	0	unif	singleC	DiP, DiP-A, OT, OT-A
197	Yes	9	9-A	200	Bozic	0	unif	wholeT_0.01	DiP-A, OT, OT-A

Table 12: *(continued)*

	Conjunction	Drivers	Tree	S.Size	Model	sh	S.Time	S.Type	Best method(s)
198	Yes	9	9-A	200	Bozic	0	unif	wholeT_0.5	DiP, DiP-A, OT, OT-A
199	Yes	9	9-A	200	Bozic	Inf	last	singleC	OT, OT-A
200	Yes	9	9-A	200	Bozic	Inf	last	wholeT_0.01	DiP, DiP-A
201	Yes	9	9-A	200	Bozic	Inf	last	wholeT_0.5	OT, OT-A
202	Yes	9	9-A	200	Bozic	Inf	unif	singleC	DiP, DiP-A, OT, OT-A
203	Yes	9	9-A	200	Bozic	Inf	unif	wholeT_0.01	DiP, DiP-A, OT, OT-A
204	Yes	9	9-A	200	Bozic	Inf	unif	wholeT_0.5	DiP, DiP-A, OT, OT-A
205	Yes	9	9-A	200	exp	0	last	singleC	DiP, DiP-A, OT, OT-A
206	Yes	9	9-A	200	exp	0	last	wholeT_0.01	DiP, DiP-A, OT, OT-A
207	Yes	9	9-A	200	exp	0	last	wholeT_0.5	DiP, DiP-A, OT, OT-A
208	Yes	9	9-A	200	exp	0	unif	singleC	DiP, DiP-A, OT, OT-A
209	Yes	9	9-A	200	exp	0	unif	wholeT_0.01	DiP, DiP-A, OT, OT-A
210	Yes	9	9-A	200	exp	0	unif	wholeT_0.5	DiP, DiP-A, OT, OT-A
211	Yes	9	9-A	200	exp	Inf	last	singleC	DiP, DiP-A, OT, OT-A
212	Yes	9	9-A	200	exp	Inf	last	wholeT_0.01	DiP, DiP-A
213	Yes	9	9-A	200	exp	Inf	last	wholeT_0.5	DiP, DiP-A, OT, OT-A
214	Yes	9	9-A	200	exp	Inf	unif	singleC	DiP, DiP-A, OT, OT-A
215	Yes	9	9-A	200	exp	Inf	unif	wholeT_0.01	DiP, DiP-A
216	Yes	9	9-A	200	exp	Inf	unif	wholeT_0.5	DiP, DiP-A, OT, OT-A
217	Yes	9	9-A	200	McF_4	0	last	singleC	DiP, DiP-A, OT, OT-A
218	Yes	9	9-A	200	McF_4	0	last	wholeT_0.01	DiP, DiP-A
219	Yes	9	9-A	200	McF_4	0	last	wholeT_0.5	DiP, DiP-A, OT, OT-A
220	Yes	9	9-A	200	McF_4	0	unif	singleC	DiP, DiP-A, OT, OT-A
221	Yes	9	9-A	200	McF_4	0	unif	wholeT_0.01	DiP, DiP-A
222	Yes	9	9-A	200	McF_4	0	unif	wholeT_0.5	DiP, DiP-A, OT, OT-A
223	Yes	9	9-A	200	McF_4	Inf	last	singleC	OT, OT-A
224	Yes	9	9-A	200	McF_4	Inf	last	wholeT_0.01	DiP, OT
225	Yes	9	9-A	200	McF_4	Inf	last	wholeT_0.5	DiP, DiP-A, OT, OT-A
226	Yes	9	9-A	200	McF_4	Inf	unif	singleC	DiP, DiP-A, OT, OT-A
227	Yes	9	9-A	200	McF_4	Inf	unif	wholeT_0.01	DiP, DiP-A, OT, OT-A
228	Yes	9	9-A	200	McF_4	Inf	unif	wholeT_0.5	DiP, DiP-A, OT, OT-A
229	Yes	9	9-A	200	McF_6	0	last	singleC	DiP, OT
230	Yes	9	9-A	200	McF_6	0	last	wholeT_0.01	DiP, OT
231	Yes	9	9-A	200	McF_6	0	last	wholeT_0.5	DiP, OT
232	Yes	9	9-A	200	McF_6	0	unif	singleC	DiP, DiP-A, OT, OT-A
233	Yes	9	9-A	200	McF_6	0	unif	wholeT_0.01	DiP, DiP-A, OT, OT-A
234	Yes	9	9-A	200	McF_6	0	unif	wholeT_0.5	DiP, DiP-A, OT, OT-A
235	Yes	9	9-A	200	McF_6	Inf	last	singleC	DiP, OT
236	Yes	9	9-A	200	McF_6	Inf	last	wholeT_0.01	OT
237	Yes	9	9-A	200	McF_6	Inf	last	wholeT_0.5	DiP, OT

Table 12: *(continued)*

	Conjunction	Drivers	Tree	S.Size	Model	sh	S.Time	S.Type	Best method(s)
238	Yes	9	9-A	200	McF_6	Inf	unif	singleC	DiP, DiP-A, OT
239	Yes	9	9-A	200	McF_6	Inf	unif	wholeT_0.01	DiP, DiP-A, OT, OT-A
240	Yes	9	9-A	200	McF_6	Inf	unif	wholeT_0.5	DiP, DiP-A, OT, OT-A
241	Yes	9	9-A	100	Bozic	0	last	singleC	DiP, DiP-A, OT, OT-A
242	Yes	9	9-A	100	Bozic	0	last	wholeT_0.01	DiP, DiP-A, OT, OT-A
243	Yes	9	9-A	100	Bozic	0	last	wholeT_0.5	DiP, DiP-A, OT, OT-A
244	Yes	9	9-A	100	Bozic	0	unif	singleC	DiP, DiP-A, OT, OT-A
245	Yes	9	9-A	100	Bozic	0	unif	wholeT_0.01	DiP, DiP-A
246	Yes	9	9-A	100	Bozic	0	unif	wholeT_0.5	DiP, DiP-A, OT, OT-A
247	Yes	9	9-A	100	Bozic	Inf	last	singleC	DiP, DiP-A, OT, OT-A
248	Yes	9	9-A	100	Bozic	Inf	last	wholeT_0.01	DiP, DiP-A, OT
249	Yes	9	9-A	100	Bozic	Inf	last	wholeT_0.5	DiP, DiP-A, OT, OT-A
250	Yes	9	9-A	100	Bozic	Inf	unif	singleC	DiP, DiP-A, OT, OT-A
251	Yes	9	9-A	100	Bozic	Inf	unif	wholeT_0.01	DiP, DiP-A, OT, OT-A
252	Yes	9	9-A	100	Bozic	Inf	unif	wholeT_0.5	DiP, DiP-A, OT
253	Yes	9	9-A	100	exp	0	last	singleC	DiP, DiP-A, OT, OT-A
254	Yes	9	9-A	100	exp	0	last	wholeT_0.01	DiP, DiP-A, OT, OT-A
255	Yes	9	9-A	100	exp	0	last	wholeT_0.5	DiP, DiP-A, OT, OT-A
256	Yes	9	9-A	100	exp	0	unif	singleC	DiP, DiP-A, OT, OT-A
257	Yes	9	9-A	100	exp	0	unif	wholeT_0.01	DiP, DiP-A, OT, OT-A
258	Yes	9	9-A	100	exp	0	unif	wholeT_0.5	DiP, DiP-A, OT, OT-A
259	Yes	9	9-A	100	exp	Inf	last	singleC	DiP, DiP-A, OT-A
260	Yes	9	9-A	100	exp	Inf	last	wholeT_0.01	DiP, DiP-A
261	Yes	9	9-A	100	exp	Inf	last	wholeT_0.5	DiP, DiP-A, OT, OT-A
262	Yes	9	9-A	100	exp	Inf	unif	singleC	DiP, DiP-A, OT, OT-A
263	Yes	9	9-A	100	exp	Inf	unif	wholeT_0.01	DiP, DiP-A
264	Yes	9	9-A	100	exp	Inf	unif	wholeT_0.5	DiP, DiP-A, OT, OT-A
265	Yes	9	9-A	100	McF_4	0	last	singleC	DiP, DiP-A, OT, OT-A
266	Yes	9	9-A	100	McF_4	0	last	wholeT_0.01	DiP, DiP-A
267	Yes	9	9-A	100	McF_4	0	last	wholeT_0.5	DiP, DiP-A, OT, OT-A
268	Yes	9	9-A	100	McF_4	0	unif	singleC	DiP, OT-A
269	Yes	9	9-A	100	McF_4	0	unif	wholeT_0.01	DiP, DiP-A
270	Yes	9	9-A	100	McF_4	0	unif	wholeT_0.5	DiP, DiP-A, OT, OT-A
271	Yes	9	9-A	100	McF_4	Inf	last	singleC	DiP-A, OT, OT-A
272	Yes	9	9-A	100	McF_4	Inf	last	wholeT_0.01	DiP, OT
273	Yes	9	9-A	100	McF_4	Inf	last	wholeT_0.5	DiP-A, OT, OT-A
274	Yes	9	9-A	100	McF_4	Inf	unif	singleC	DiP, DiP-A, OT, OT-A
275	Yes	9	9-A	100	McF_4	Inf	unif	wholeT_0.01	DiP, DiP-A, OT, OT-A
276	Yes	9	9-A	100	McF_4	Inf	unif	wholeT_0.5	DiP, DiP-A, OT-A
277	Yes	9	9-A	100	McF_6	0	last	singleC	DiP, OT

Table 12: *(continued)*

	Conjunction	Drivers	Tree	S.Size	Model	sh	S.Time	S.Type	Best method(s)
278	Yes	9	9-A	100	McF_6	0	last	wholeT_0.01	DiP, OT
279	Yes	9	9-A	100	McF_6	0	last	wholeT_0.5	DiP, OT
280	Yes	9	9-A	100	McF_6	0	unif	singleC	DiP, DiP-A, OT
281	Yes	9	9-A	100	McF_6	0	unif	wholeT_0.01	DiP, DiP-A, OT, OT-A
282	Yes	9	9-A	100	McF_6	0	unif	wholeT_0.5	DiP, OT, OT-A
283	Yes	9	9-A	100	McF_6	Inf	last	singleC	DiP, DiP-A, OT
284	Yes	9	9-A	100	McF_6	Inf	last	wholeT_0.01	DiP, OT
285	Yes	9	9-A	100	McF_6	Inf	last	wholeT_0.5	DiP, OT
286	Yes	9	9-A	100	McF_6	Inf	unif	singleC	DiP, DiP-A, OT
287	Yes	9	9-A	100	McF_6	Inf	unif	wholeT_0.01	DiP, DiP-A, OT, OT-A
288	Yes	9	9-A	100	McF_6	Inf	unif	wholeT_0.5	DiP, DiP-A, OT, OT-A
289	Yes	7	7-A	1000	Bozic	0	last	singleC	DiP, DiP-A, OT, OT-A
290	Yes	7	7-A	1000	Bozic	0	last	wholeT_0.01	DiP, DiP-A
291	Yes	7	7-A	1000	Bozic	0	last	wholeT_0.5	DiP, DiP-A, OT, OT-A
292	Yes	7	7-A	1000	Bozic	0	unif	singleC	DiP, DiP-A, OT, OT-A
293	Yes	7	7-A	1000	Bozic	0	unif	wholeT_0.01	none
294	Yes	7	7-A	1000	Bozic	0	unif	wholeT_0.5	DiP, DiP-A, OT, OT-A
295	Yes	7	7-A	1000	Bozic	Inf	last	singleC	DiP, DiP-A, OT
296	Yes	7	7-A	1000	Bozic	Inf	last	wholeT_0.01	DiP, DiP-A, OT
297	Yes	7	7-A	1000	Bozic	Inf	last	wholeT_0.5	DiP, DiP-A, OT, OT-A
298	Yes	7	7-A	1000	Bozic	Inf	unif	singleC	CBN, CBN-A, OT
299	Yes	7	7-A	1000	Bozic	Inf	unif	wholeT_0.01	DiP, DiP-A
300	Yes	7	7-A	1000	Bozic	Inf	unif	wholeT_0.5	CBN, CBN-A
301	Yes	7	7-A	1000	exp	0	last	singleC	DiP, DiP-A, OT, OT-A
302	Yes	7	7-A	1000	exp	0	last	wholeT_0.01	DiP, DiP-A, OT, OT-A
303	Yes	7	7-A	1000	exp	0	last	wholeT_0.5	DiP, DiP-A, OT, OT-A
304	Yes	7	7-A	1000	exp	0	unif	singleC	DiP, DiP-A, OT, OT-A
305	Yes	7	7-A	1000	exp	0	unif	wholeT_0.01	DiP, DiP-A
306	Yes	7	7-A	1000	exp	0	unif	wholeT_0.5	DiP, DiP-A, OT, OT-A
307	Yes	7	7-A	1000	exp	Inf	last	singleC	DiP, DiP-A, OT, OT-A
308	Yes	7	7-A	1000	exp	Inf	last	wholeT_0.01	DiP, DiP-A, OT, OT-A
309	Yes	7	7-A	1000	exp	Inf	last	wholeT_0.5	DiP, DiP-A, OT, OT-A
310	Yes	7	7-A	1000	exp	Inf	unif	singleC	DiP, DiP-A
311	Yes	7	7-A	1000	exp	Inf	unif	wholeT_0.01	DiP-A, OT, OT-A
312	Yes	7	7-A	1000	exp	Inf	unif	wholeT_0.5	DiP, DiP-A
313	Yes	7	7-A	1000	McF_4	0	last	singleC	DiP, DiP-A, OT, OT-A
314	Yes	7	7-A	1000	McF_4	0	last	wholeT_0.01	OT, OT-A
315	Yes	7	7-A	1000	McF_4	0	last	wholeT_0.5	DiP, DiP-A, OT
316	Yes	7	7-A	1000	McF_4	0	unif	singleC	DiP, DiP-A
317	Yes	7	7-A	1000	McF_4	0	unif	wholeT_0.01	DiP, DiP-A

Table 12: *(continued)*

	Conjunction	Drivers	Tree	S.Size	Model	sh	S.Time	S.Type	Best method(s)
318	Yes	7	7-A	1000	McF_4	0	unif	wholeT_0.5	DiP, DiP-A
319	Yes	7	7-A	1000	McF_4	Inf	last	singleC	DiP, DiP-A, OT, OT-A
320	Yes	7	7-A	1000	McF_4	Inf	last	wholeT_0.01	OT, OT-A
321	Yes	7	7-A	1000	McF_4	Inf	last	wholeT_0.5	DiP, DiP-A, OT, OT-A
322	Yes	7	7-A	1000	McF_4	Inf	unif	singleC	DiP, DiP-A, OT, OT-A
323	Yes	7	7-A	1000	McF_4	Inf	unif	wholeT_0.01	DiP, DiP-A, OT, OT-A
324	Yes	7	7-A	1000	McF_4	Inf	unif	wholeT_0.5	DiP, OT, OT-A
325	Yes	7	7-A	1000	McF_6	0	last	singleC	DiP, OT, OT-A
326	Yes	7	7-A	1000	McF_6	0	last	wholeT_0.01	DiP, OT, OT-A
327	Yes	7	7-A	1000	McF_6	0	last	wholeT_0.5	DiP, OT, OT-A
328	Yes	7	7-A	1000	McF_6	0	unif	singleC	none
329	Yes	7	7-A	1000	McF_6	0	unif	wholeT_0.01	none
330	Yes	7	7-A	1000	McF_6	0	unif	wholeT_0.5	none
331	Yes	7	7-A	1000	McF_6	Inf	last	singleC	DiP, DiP-A, OT, OT-A
332	Yes	7	7-A	1000	McF_6	Inf	last	wholeT_0.01	DiP, OT, OT-A
333	Yes	7	7-A	1000	McF_6	Inf	last	wholeT_0.5	DiP, OT, OT-A
334	Yes	7	7-A	1000	McF_6	Inf	unif	singleC	none
335	Yes	7	7-A	1000	McF_6	Inf	unif	wholeT_0.01	CBN, CBN-A, OT, OT-A
336	Yes	7	7-A	1000	McF_6	Inf	unif	wholeT_0.5	none
337	Yes	7	7-A	200	Bozic	0	last	singleC	DiP, DiP-A, OT, OT-A
338	Yes	7	7-A	200	Bozic	0	last	wholeT_0.01	DiP, DiP-A, OT, OT-A
339	Yes	7	7-A	200	Bozic	0	last	wholeT_0.5	DiP, DiP-A, OT, OT-A
340	Yes	7	7-A	200	Bozic	0	unif	singleC	DiP, DiP-A, OT, OT-A
341	Yes	7	7-A	200	Bozic	0	unif	wholeT_0.01	none
342	Yes	7	7-A	200	Bozic	0	unif	wholeT_0.5	DiP, DiP-A, OT, OT-A
343	Yes	7	7-A	200	Bozic	Inf	last	singleC	DiP, DiP-A, OT, OT-A
344	Yes	7	7-A	200	Bozic	Inf	last	wholeT_0.01	DiP, DiP-A, OT, OT-A
345	Yes	7	7-A	200	Bozic	Inf	last	wholeT_0.5	DiP, DiP-A, OT, OT-A
346	Yes	7	7-A	200	Bozic	Inf	unif	singleC	DiP, DiP-A
347	Yes	7	7-A	200	Bozic	Inf	unif	wholeT_0.01	DiP-A, OT, OT-A
348	Yes	7	7-A	200	Bozic	Inf	unif	wholeT_0.5	DiP, DiP-A
349	Yes	7	7-A	200	exp	0	last	singleC	DiP, DiP-A, OT, OT-A
350	Yes	7	7-A	200	exp	0	last	wholeT_0.01	DiP, DiP-A
351	Yes	7	7-A	200	exp	0	last	wholeT_0.5	DiP, DiP-A, OT, OT-A
352	Yes	7	7-A	200	exp	0	unif	singleC	DiP, DiP-A, OT, OT-A
353	Yes	7	7-A	200	exp	0	unif	wholeT_0.01	DiP, DiP-A
354	Yes	7	7-A	200	exp	0	unif	wholeT_0.5	DiP, DiP-A, OT, OT-A
355	Yes	7	7-A	200	exp	Inf	last	singleC	DiP, OT, OT-A
356	Yes	7	7-A	200	exp	Inf	last	wholeT_0.01	DiP, DiP-A, OT
357	Yes	7	7-A	200	exp	Inf	last	wholeT_0.5	DiP, DiP-A, OT, OT-A

Table 12: *(continued)*

	Conjunction	Drivers	Tree	S.Size	Model	sh	S.Time	S.Type	Best method(s)
358	Yes	7	7-A	200	exp	Inf	unif	singleC	DiP, DiP-A
359	Yes	7	7-A	200	exp	Inf	unif	wholeT_0.01	DiP, DiP-A, OT, OT-A
360	Yes	7	7-A	200	exp	Inf	unif	wholeT_0.5	DiP, DiP-A
361	Yes	7	7-A	200	McF_4	0	last	singleC	DiP, DiP-A, OT, OT-A
362	Yes	7	7-A	200	McF_4	0	last	wholeT_0.01	DiP, DiP-A, OT, OT-A
363	Yes	7	7-A	200	McF_4	0	last	wholeT_0.5	DiP, DiP-A
364	Yes	7	7-A	200	McF_4	0	unif	singleC	DiP, DiP-A, OT, OT-A
365	Yes	7	7-A	200	McF_4	0	unif	wholeT_0.01	DiP, DiP-A
366	Yes	7	7-A	200	McF_4	0	unif	wholeT_0.5	DiP, DiP-A, OT
367	Yes	7	7-A	200	McF_4	Inf	last	singleC	DiP, DiP-A, OT, OT-A
368	Yes	7	7-A	200	McF_4	Inf	last	wholeT_0.01	DiP, DiP-A, OT, OT-A
369	Yes	7	7-A	200	McF_4	Inf	last	wholeT_0.5	DiP, DiP-A, OT, OT-A
370	Yes	7	7-A	200	McF_4	Inf	unif	singleC	DiP, DiP-A, OT, OT-A
371	Yes	7	7-A	200	McF_4	Inf	unif	wholeT_0.01	DiP, DiP-A, OT, OT-A
372	Yes	7	7-A	200	McF_4	Inf	unif	wholeT_0.5	DiP, DiP-A, OT, OT-A
373	Yes	7	7-A	200	McF_6	0	last	singleC	DiP, OT, OT-A
374	Yes	7	7-A	200	McF_6	0	last	wholeT_0.01	DiP, OT, OT-A
375	Yes	7	7-A	200	McF_6	0	last	wholeT_0.5	DiP, OT, OT-A
376	Yes	7	7-A	200	McF_6	0	unif	singleC	CBN, CBN-A, DiP, DiP-A
377	Yes	7	7-A	200	McF_6	0	unif	wholeT_0.01	CBN, CBN-A, DiP, DiP-A, OT
378	Yes	7	7-A	200	McF_6	0	unif	wholeT_0.5	CBN, CBN-A, DiP, DiP-A
379	Yes	7	7-A	200	McF_6	Inf	last	singleC	DiP, DiP-A, OT, OT-A
380	Yes	7	7-A	200	McF_6	Inf	last	wholeT_0.01	OT, OT-A
381	Yes	7	7-A	200	McF_6	Inf	last	wholeT_0.5	DiP, OT, OT-A
382	Yes	7	7-A	200	McF_6	Inf	unif	singleC	CBN, CBN-A, DiP, DiP-A
383	Yes	7	7-A	200	McF_6	Inf	unif	wholeT_0.01	CBN, CBN-A, DiP, DiP-A
384	Yes	7	7-A	200	McF_6	Inf	unif	wholeT_0.5	CBN, CBN-A, DiP, DiP-A
385	Yes	7	7-A	100	Bozic	0	last	singleC	DiP-A, OT, OT-A
386	Yes	7	7-A	100	Bozic	0	last	wholeT_0.01	DiP, DiP-A, OT, OT-A
387	Yes	7	7-A	100	Bozic	0	last	wholeT_0.5	DiP, DiP-A, OT-A
388	Yes	7	7-A	100	Bozic	0	unif	singleC	DiP, DiP-A, OT, OT-A
389	Yes	7	7-A	100	Bozic	0	unif	wholeT_0.01	CBN, DiP, DiP-A, OT, OT-A
390	Yes	7	7-A	100	Bozic	0	unif	wholeT_0.5	DiP, DiP-A, OT, OT-A
391	Yes	7	7-A	100	Bozic	Inf	last	singleC	DiP, DiP-A, OT, OT-A
392	Yes	7	7-A	100	Bozic	Inf	last	wholeT_0.01	DiP, DiP-A, OT
393	Yes	7	7-A	100	Bozic	Inf	last	wholeT_0.5	DiP, DiP-A, OT, OT-A
394	Yes	7	7-A	100	Bozic	Inf	unif	singleC	DiP, DiP-A
395	Yes	7	7-A	100	Bozic	Inf	unif	wholeT_0.01	OT, OT-A
396	Yes	7	7-A	100	Bozic	Inf	unif	wholeT_0.5	DiP, DiP-A
397	Yes	7	7-A	100	exp	0	last	singleC	DiP-A, OT, OT-A

Table 12: *(continued)*

	Conjunction	Drivers	Tree	S.Size	Model	sh	S.Time	S.Type	Best method(s)
398	Yes	7	7-A	100	exp	0	last	wholeT_0.01	DiP, DiP-A
399	Yes	7	7-A	100	exp	0	last	wholeT_0.5	DiP, DiP-A, OT, OT-A
400	Yes	7	7-A	100	exp	0	unif	singleC	DiP, DiP-A, OT, OT-A
401	Yes	7	7-A	100	exp	0	unif	wholeT_0.01	DiP, DiP-A, OT-A
402	Yes	7	7-A	100	exp	0	unif	wholeT_0.5	DiP, DiP-A, OT, OT-A
403	Yes	7	7-A	100	exp	Inf	last	singleC	DiP, DiP-A, OT-A
404	Yes	7	7-A	100	exp	Inf	last	wholeT_0.01	DiP, DiP-A
405	Yes	7	7-A	100	exp	Inf	last	wholeT_0.5	DiP, DiP-A, OT, OT-A
406	Yes	7	7-A	100	exp	Inf	unif	singleC	DiP, DiP-A, OT-A
407	Yes	7	7-A	100	exp	Inf	unif	wholeT_0.01	DiP, OT, OT-A
408	Yes	7	7-A	100	exp	Inf	unif	wholeT_0.5	DiP, DiP-A
409	Yes	7	7-A	100	McF_4	0	last	singleC	DiP, DiP-A, OT, OT-A
410	Yes	7	7-A	100	McF_4	0	last	wholeT_0.01	DiP, DiP-A, OT-A
411	Yes	7	7-A	100	McF_4	0	last	wholeT_0.5	DiP, DiP-A, OT, OT-A
412	Yes	7	7-A	100	McF_4	0	unif	singleC	DiP, DiP-A
413	Yes	7	7-A	100	McF_4	0	unif	wholeT_0.01	DiP, DiP-A
414	Yes	7	7-A	100	McF_4	0	unif	wholeT_0.5	DiP, DiP-A, OT
415	Yes	7	7-A	100	McF_4	Inf	last	singleC	DiP, DiP-A, OT, OT-A
416	Yes	7	7-A	100	McF_4	Inf	last	wholeT_0.01	DiP, DiP-A, OT, OT-A
417	Yes	7	7-A	100	McF_4	Inf	last	wholeT_0.5	DiP, DiP-A, OT, OT-A
418	Yes	7	7-A	100	McF_4	Inf	unif	singleC	DiP, DiP-A, OT
419	Yes	7	7-A	100	McF_4	Inf	unif	wholeT_0.01	DiP, DiP-A, OT, OT-A
420	Yes	7	7-A	100	McF_4	Inf	unif	wholeT_0.5	DiP, DiP-A, OT
421	Yes	7	7-A	100	McF_6	0	last	singleC	DiP, DiP-A, OT, OT-A
422	Yes	7	7-A	100	McF_6	0	last	wholeT_0.01	DiP, OT, OT-A
423	Yes	7	7-A	100	McF_6	0	last	wholeT_0.5	DiP, OT, OT-A
424	Yes	7	7-A	100	McF_6	0	unif	singleC	CBN, CBN-A, DiP, DiP-A
425	Yes	7	7-A	100	McF_6	0	unif	wholeT_0.01	CBN, CBN-A, DiP, DiP-A
426	Yes	7	7-A	100	McF_6	0	unif	wholeT_0.5	CBN, CBN-A, DiP, DiP-A
427	Yes	7	7-A	100	McF_6	Inf	last	singleC	DiP, DiP-A, OT, OT-A
428	Yes	7	7-A	100	McF_6	Inf	last	wholeT_0.01	DiP, OT, OT-A
429	Yes	7	7-A	100	McF_6	Inf	last	wholeT_0.5	DiP, DiP-A, OT, OT-A
430	Yes	7	7-A	100	McF_6	Inf	unif	singleC	CBN, CBN-A, DiP, DiP-A
431	Yes	7	7-A	100	McF_6	Inf	unif	wholeT_0.01	CBN, CBN-A, DiP, DiP-A
432	Yes	7	7-A	100	McF_6	Inf	unif	wholeT_0.5	CBN, CBN-A, DiP, DiP-A
433	No	11	11-B	1000	Bozic	0	last	singleC	OT, OT-A
434	No	11	11-B	1000	Bozic	0	last	wholeT_0.01	DiP, DiP-A
435	No	11	11-B	1000	Bozic	0	last	wholeT_0.5	DiP, DiP-A, OT, OT-A
436	No	11	11-B	1000	Bozic	0	unif	singleC	DiP, DiP-A, OT, OT-A
437	No	11	11-B	1000	Bozic	0	unif	wholeT_0.01	DiP-A

Table 12: *(continued)*

	Conjunction	Drivers	Tree	S.Size	Model	sh	S.Time	S.Type	Best method(s)
438	No	11	11-B	1000	Bozic	0	unif	wholeT_0.5	DiP, DiP-A, OT, OT-A
439	No	11	11-B	1000	Bozic	Inf	last	singleC	OT, OT-A
440	No	11	11-B	1000	Bozic	Inf	last	wholeT_0.01	DiP-A
441	No	11	11-B	1000	Bozic	Inf	last	wholeT_0.5	OT, OT-A
442	No	11	11-B	1000	Bozic	Inf	unif	singleC	DiP, DiP-A
443	No	11	11-B	1000	Bozic	Inf	unif	wholeT_0.01	OT, OT-A
444	No	11	11-B	1000	Bozic	Inf	unif	wholeT_0.5	DiP, DiP-A
445	No	11	11-B	1000	exp	0	last	singleC	DiP, DiP-A, OT, OT-A
446	No	11	11-B	1000	exp	0	last	wholeT_0.01	DiP, DiP-A, OT, OT-A
447	No	11	11-B	1000	exp	0	last	wholeT_0.5	DiP-A, OT, OT-A
448	No	11	11-B	1000	exp	0	unif	singleC	DiP, DiP-A, OT, OT-A
449	No	11	11-B	1000	exp	0	unif	wholeT_0.01	DiP, DiP-A
450	No	11	11-B	1000	exp	0	unif	wholeT_0.5	DiP, DiP-A, OT, OT-A
451	No	11	11-B	1000	exp	Inf	last	singleC	OT, OT-A
452	No	11	11-B	1000	exp	Inf	last	wholeT_0.01	OT, OT-A
453	No	11	11-B	1000	exp	Inf	last	wholeT_0.5	OT, OT-A
454	No	11	11-B	1000	exp	Inf	unif	singleC	DiP, DiP-A, OT, OT-A
455	No	11	11-B	1000	exp	Inf	unif	wholeT_0.01	DiP, DiP-A, OT, OT-A
456	No	11	11-B	1000	exp	Inf	unif	wholeT_0.5	DiP, DiP-A, OT, OT-A
457	No	11	11-B	1000	McF_4	0	last	singleC	DiP, DiP-A, OT, OT-A
458	No	11	11-B	1000	McF_4	0	last	wholeT_0.01	DiP, DiP-A, OT, OT-A
459	No	11	11-B	1000	McF_4	0	last	wholeT_0.5	DiP-A, OT, OT-A
460	No	11	11-B	1000	McF_4	0	unif	singleC	DiP, DiP-A
461	No	11	11-B	1000	McF_4	0	unif	wholeT_0.01	DiP-A, OT, OT-A
462	No	11	11-B	1000	McF_4	0	unif	wholeT_0.5	DiP, DiP-A, OT, OT-A
463	No	11	11-B	1000	McF_4	Inf	last	singleC	OT, OT-A
464	No	11	11-B	1000	McF_4	Inf	last	wholeT_0.01	DiP, DiP-A, OT, OT-A
465	No	11	11-B	1000	McF_4	Inf	last	wholeT_0.5	DiP-A, OT, OT-A
466	No	11	11-B	1000	McF_4	Inf	unif	singleC	DiP, DiP-A, OT, OT-A
467	No	11	11-B	1000	McF_4	Inf	unif	wholeT_0.01	OT, OT-A
468	No	11	11-B	1000	McF_4	Inf	unif	wholeT_0.5	DiP, DiP-A, OT, OT-A
469	No	11	11-B	1000	McF_6	0	last	singleC	DiP, OT
470	No	11	11-B	1000	McF_6	0	last	wholeT_0.01	DiP, OT
471	No	11	11-B	1000	McF_6	0	last	wholeT_0.5	DiP, OT
472	No	11	11-B	1000	McF_6	0	unif	singleC	DiP, DiP-A, OT
473	No	11	11-B	1000	McF_6	0	unif	wholeT_0.01	DiP, DiP-A, OT, OT-A
474	No	11	11-B	1000	McF_6	0	unif	wholeT_0.5	DiP, DiP-A
475	No	11	11-B	1000	McF_6	Inf	last	singleC	DiP, OT
476	No	11	11-B	1000	McF_6	Inf	last	wholeT_0.01	DiP, OT
477	No	11	11-B	1000	McF_6	Inf	last	wholeT_0.5	DiP, OT

Table 12: *(continued)*

	Conjunction	Drivers	Tree	S.Size	Model	sh	S.Time	S.Type	Best method(s)
478	No	11	11-B	1000	McF_6	Inf	unif	singleC	DiP, DiP-A
479	No	11	11-B	1000	McF_6	Inf	unif	wholeT_0.01	DiP, DiP-A, OT-A
480	No	11	11-B	1000	McF_6	Inf	unif	wholeT_0.5	DiP, DiP-A
481	No	11	11-B	200	Bozic	0	last	singleC	DiP, DiP-A, OT, OT-A
482	No	11	11-B	200	Bozic	0	last	wholeT_0.01	DiP
483	No	11	11-B	200	Bozic	0	last	wholeT_0.5	DiP, DiP-A, OT, OT-A
484	No	11	11-B	200	Bozic	0	unif	singleC	DiP, DiP-A, OT, OT-A
485	No	11	11-B	200	Bozic	0	unif	wholeT_0.01	DiP, DiP-A
486	No	11	11-B	200	Bozic	0	unif	wholeT_0.5	DiP, DiP-A, OT, OT-A
487	No	11	11-B	200	Bozic	Inf	last	singleC	DiP, DiP-A, OT, OT-A
488	No	11	11-B	200	Bozic	Inf	last	wholeT_0.01	DiP-A, OT, OT-A
489	No	11	11-B	200	Bozic	Inf	last	wholeT_0.5	DiP, DiP-A, OT, OT-A
490	No	11	11-B	200	Bozic	Inf	unif	singleC	DiP, DiP-A
491	No	11	11-B	200	Bozic	Inf	unif	wholeT_0.01	DiP, DiP-A
492	No	11	11-B	200	Bozic	Inf	unif	wholeT_0.5	DiP, DiP-A
493	No	11	11-B	200	exp	0	last	singleC	DiP, DiP-A, OT, OT-A
494	No	11	11-B	200	exp	0	last	wholeT_0.01	DiP, DiP-A, OT, OT-A
495	No	11	11-B	200	exp	0	last	wholeT_0.5	DiP, DiP-A, OT, OT-A
496	No	11	11-B	200	exp	0	unif	singleC	DiP, DiP-A, OT, OT-A
497	No	11	11-B	200	exp	0	unif	wholeT_0.01	DiP, DiP-A, OT
498	No	11	11-B	200	exp	0	unif	wholeT_0.5	DiP, DiP-A, OT, OT-A
499	No	11	11-B	200	exp	Inf	last	singleC	DiP, DiP-A, OT, OT-A
500	No	11	11-B	200	exp	Inf	last	wholeT_0.01	DiP, OT, OT-A
501	No	11	11-B	200	exp	Inf	last	wholeT_0.5	DiP, DiP-A, OT, OT-A
502	No	11	11-B	200	exp	Inf	unif	singleC	DiP, DiP-A, OT, OT-A
503	No	11	11-B	200	exp	Inf	unif	wholeT_0.01	DiP, DiP-A
504	No	11	11-B	200	exp	Inf	unif	wholeT_0.5	DiP, DiP-A, OT, OT-A
505	No	11	11-B	200	McF_4	0	last	singleC	DiP, DiP-A, OT, OT-A
506	No	11	11-B	200	McF_4	0	last	wholeT_0.01	DiP, DiP-A, OT, OT-A
507	No	11	11-B	200	McF_4	0	last	wholeT_0.5	DiP-A, OT, OT-A
508	No	11	11-B	200	McF_4	0	unif	singleC	DiP, DiP-A, OT, OT-A
509	No	11	11-B	200	McF_4	0	unif	wholeT_0.01	DiP, DiP-A
510	No	11	11-B	200	McF_4	0	unif	wholeT_0.5	DiP, DiP-A, OT, OT-A
511	No	11	11-B	200	McF_4	Inf	last	singleC	OT, OT-A
512	No	11	11-B	200	McF_4	Inf	last	wholeT_0.01	DiP, OT, OT-A
513	No	11	11-B	200	McF_4	Inf	last	wholeT_0.5	DiP, DiP-A, OT
514	No	11	11-B	200	McF_4	Inf	unif	singleC	DiP, DiP-A, OT, OT-A
515	No	11	11-B	200	McF_4	Inf	unif	wholeT_0.01	DiP, DiP-A, OT, OT-A
516	No	11	11-B	200	McF_4	Inf	unif	wholeT_0.5	DiP, DiP-A, OT, OT-A
517	No	11	11-B	200	McF_6	0	last	singleC	DiP, OT

Table 12: *(continued)*

	Conjunction	Drivers	Tree	S.Size	Model	sh	S.Time	S.Type	Best method(s)
518	No	11	11-B	200	McF_6	0	last	wholeT_0.01	DiP, OT
519	No	11	11-B	200	McF_6	0	last	wholeT_0.5	DiP, OT
520	No	11	11-B	200	McF_6	0	unif	singleC	DiP, DiP-A, OT, OT-A
521	No	11	11-B	200	McF_6	0	unif	wholeT_0.01	DiP, DiP-A, OT, OT-A
522	No	11	11-B	200	McF_6	0	unif	wholeT_0.5	OT, OT-A
523	No	11	11-B	200	McF_6	Inf	last	singleC	DiP, OT
524	No	11	11-B	200	McF_6	Inf	last	wholeT_0.01	DiP, OT
525	No	11	11-B	200	McF_6	Inf	last	wholeT_0.5	DiP, OT
526	No	11	11-B	200	McF_6	Inf	unif	singleC	OT, OT-A
527	No	11	11-B	200	McF_6	Inf	unif	wholeT_0.01	OT, OT-A
528	No	11	11-B	200	McF_6	Inf	unif	wholeT_0.5	CBN-A, DiP, DiP-A, OT, OT-A
529	No	11	11-B	100	Bozic	0	last	singleC	DiP, DiP-A, OT, OT-A
530	No	11	11-B	100	Bozic	0	last	wholeT_0.01	DiP, DiP-A
531	No	11	11-B	100	Bozic	0	last	wholeT_0.5	DiP, DiP-A, OT
532	No	11	11-B	100	Bozic	0	unif	singleC	DiP, DiP-A, OT, OT-A
533	No	11	11-B	100	Bozic	0	unif	wholeT_0.01	DiP, DiP-A
534	No	11	11-B	100	Bozic	0	unif	wholeT_0.5	DiP, DiP-A, OT, OT-A
535	No	11	11-B	100	Bozic	Inf	last	singleC	DiP, DiP-A, OT-A
536	No	11	11-B	100	Bozic	Inf	last	wholeT_0.01	DiP, DiP-A, OT
537	No	11	11-B	100	Bozic	Inf	last	wholeT_0.5	DiP, DiP-A, OT
538	No	11	11-B	100	Bozic	Inf	unif	singleC	DiP, DiP-A
539	No	11	11-B	100	Bozic	Inf	unif	wholeT_0.01	DiP, DiP-A
540	No	11	11-B	100	Bozic	Inf	unif	wholeT_0.5	DiP, DiP-A, OT, OT-A
541	No	11	11-B	100	exp	0	last	singleC	DiP, DiP-A, OT, OT-A
542	No	11	11-B	100	exp	0	last	wholeT_0.01	DiP, DiP-A
543	No	11	11-B	100	exp	0	last	wholeT_0.5	DiP, DiP-A, OT, OT-A
544	No	11	11-B	100	exp	0	unif	singleC	DiP, DiP-A, OT, OT-A
545	No	11	11-B	100	exp	0	unif	wholeT_0.01	DiP, DiP-A, OT, OT-A
546	No	11	11-B	100	exp	0	unif	wholeT_0.5	DiP, DiP-A, OT, OT-A
547	No	11	11-B	100	exp	Inf	last	singleC	DiP, DiP-A, OT
548	No	11	11-B	100	exp	Inf	last	wholeT_0.01	DiP, DiP-A, OT, OT-A
549	No	11	11-B	100	exp	Inf	last	wholeT_0.5	DiP, DiP-A, OT, OT-A
550	No	11	11-B	100	exp	Inf	unif	singleC	DiP, DiP-A, OT, OT-A
551	No	11	11-B	100	exp	Inf	unif	wholeT_0.01	DiP, DiP-A
552	No	11	11-B	100	exp	Inf	unif	wholeT_0.5	DiP, DiP-A, OT
553	No	11	11-B	100	McF_4	0	last	singleC	DiP, DiP-A, OT
554	No	11	11-B	100	McF_4	0	last	wholeT_0.01	DiP, DiP-A, OT
555	No	11	11-B	100	McF_4	0	last	wholeT_0.5	DiP, DiP-A, OT, OT-A
556	No	11	11-B	100	McF_4	0	unif	singleC	DiP, DiP-A
557	No	11	11-B	100	McF_4	0	unif	wholeT_0.01	DiP, DiP-A

Table 12: *(continued)*

	Conjunction	Drivers	Tree	S.Size	Model	sh	S.Time	S.Type	Best method(s)
558	No	11	11-B	100	McF_4	0	unif	wholeT_0.5	DiP, DiP-A, OT-A
559	No	11	11-B	100	McF_4	Inf	last	singleC	DiP, DiP-A, OT, OT-A
560	No	11	11-B	100	McF_4	Inf	last	wholeT_0.01	DiP, DiP-A, OT, OT-A
561	No	11	11-B	100	McF_4	Inf	last	wholeT_0.5	DiP, DiP-A, OT, OT-A
562	No	11	11-B	100	McF_4	Inf	unif	singleC	DiP, DiP-A, OT, OT-A
563	No	11	11-B	100	McF_4	Inf	unif	wholeT_0.01	DiP, DiP-A, OT, OT-A
564	No	11	11-B	100	McF_4	Inf	unif	wholeT_0.5	DiP, DiP-A
565	No	11	11-B	100	McF_6	0	last	singleC	DiP, OT
566	No	11	11-B	100	McF_6	0	last	wholeT_0.01	DiP, OT
567	No	11	11-B	100	McF_6	0	last	wholeT_0.5	DiP, OT
568	No	11	11-B	100	McF_6	0	unif	singleC	DiP, DiP-A
569	No	11	11-B	100	McF_6	0	unif	wholeT_0.01	DiP, DiP-A, OT, OT-A
570	No	11	11-B	100	McF_6	0	unif	wholeT_0.5	DiP, DiP-A, OT, OT-A
571	No	11	11-B	100	McF_6	Inf	last	singleC	DiP, OT
572	No	11	11-B	100	McF_6	Inf	last	wholeT_0.01	DiP, OT
573	No	11	11-B	100	McF_6	Inf	last	wholeT_0.5	DiP, OT
574	No	11	11-B	100	McF_6	Inf	unif	singleC	DiP, DiP-A, OT, OT-A
575	No	11	11-B	100	McF_6	Inf	unif	wholeT_0.01	DiP, DiP-A, OT, OT-A
576	No	11	11-B	100	McF_6	Inf	unif	wholeT_0.5	DiP, DiP-A, OT, OT-A
577	No	9	9-B	1000	Bozic	0	last	singleC	DiP-A, OT, OT-A
578	No	9	9-B	1000	Bozic	0	last	wholeT_0.01	DiP, DiP-A
579	No	9	9-B	1000	Bozic	0	last	wholeT_0.5	DiP, DiP-A, OT-A
580	No	9	9-B	1000	Bozic	0	unif	singleC	DiP, DiP-A, OT, OT-A
581	No	9	9-B	1000	Bozic	0	unif	wholeT_0.01	DiP, DiP-A
582	No	9	9-B	1000	Bozic	0	unif	wholeT_0.5	DiP, DiP-A
583	No	9	9-B	1000	Bozic	Inf	last	singleC	OT, OT-A
584	No	9	9-B	1000	Bozic	Inf	last	wholeT_0.01	DiP, DiP-A
585	No	9	9-B	1000	Bozic	Inf	last	wholeT_0.5	OT, OT-A
586	No	9	9-B	1000	Bozic	Inf	unif	singleC	DiP, DiP-A
587	No	9	9-B	1000	Bozic	Inf	unif	wholeT_0.01	DiP, DiP-A, OT-A
588	No	9	9-B	1000	Bozic	Inf	unif	wholeT_0.5	DiP, DiP-A, OT-A
589	No	9	9-B	1000	exp	0	last	singleC	DiP-A, OT, OT-A
590	No	9	9-B	1000	exp	0	last	wholeT_0.01	DiP-A, OT, OT-A
591	No	9	9-B	1000	exp	0	last	wholeT_0.5	DiP, DiP-A, OT, OT-A
592	No	9	9-B	1000	exp	0	unif	singleC	DiP, DiP-A, OT, OT-A
593	No	9	9-B	1000	exp	0	unif	wholeT_0.01	DiP, DiP-A
594	No	9	9-B	1000	exp	0	unif	wholeT_0.5	DiP, DiP-A, OT, OT-A
595	No	9	9-B	1000	exp	Inf	last	singleC	DiP, OT, OT-A
596	No	9	9-B	1000	exp	Inf	last	wholeT_0.01	CBN-A, DiP, DiP-A, OT, OT-A
597	No	9	9-B	1000	exp	Inf	last	wholeT_0.5	DiP, DiP-A, OT, OT-A

Table 12: *(continued)*

	Conjunction	Drivers	Tree	S.Size	Model	sh	S.Time	S.Type	Best method(s)
598	No	9	9-B	1000	exp	Inf	unif	singleC	DiP, DiP-A, OT, OT-A
599	No	9	9-B	1000	exp	Inf	unif	wholeT_0.01	DiP-A, OT, OT-A
600	No	9	9-B	1000	exp	Inf	unif	wholeT_0.5	DiP, DiP-A, OT, OT-A
601	No	9	9-B	1000	McF_4	0	last	singleC	OT, OT-A
602	No	9	9-B	1000	McF_4	0	last	wholeT_0.01	DiP, DiP-A, OT, OT-A
603	No	9	9-B	1000	McF_4	0	last	wholeT_0.5	DiP-A, OT, OT-A
604	No	9	9-B	1000	McF_4	0	unif	singleC	DiP, DiP-A, OT, OT-A
605	No	9	9-B	1000	McF_4	0	unif	wholeT_0.01	DiP-A, OT, OT-A
606	No	9	9-B	1000	McF_4	0	unif	wholeT_0.5	DiP, DiP-A, OT, OT-A
607	No	9	9-B	1000	McF_4	Inf	last	singleC	OT, OT-A
608	No	9	9-B	1000	McF_4	Inf	last	wholeT_0.01	DiP, DiP-A, OT, OT-A
609	No	9	9-B	1000	McF_4	Inf	last	wholeT_0.5	DiP, DiP-A, OT, OT-A
610	No	9	9-B	1000	McF_4	Inf	unif	singleC	DiP, DiP-A, OT, OT-A
611	No	9	9-B	1000	McF_4	Inf	unif	wholeT_0.01	DiP-A, OT, OT-A
612	No	9	9-B	1000	McF_4	Inf	unif	wholeT_0.5	DiP, DiP-A, OT, OT-A
613	No	9	9-B	1000	McF_6	0	last	singleC	DiP, OT
614	No	9	9-B	1000	McF_6	0	last	wholeT_0.01	DiP, OT
615	No	9	9-B	1000	McF_6	0	last	wholeT_0.5	DiP, OT
616	No	9	9-B	1000	McF_6	0	unif	singleC	DiP, DiP-A, OT, OT-A
617	No	9	9-B	1000	McF_6	0	unif	wholeT_0.01	DiP, DiP-A, OT, OT-A
618	No	9	9-B	1000	McF_6	0	unif	wholeT_0.5	DiP, DiP-A, OT, OT-A
619	No	9	9-B	1000	McF_6	Inf	last	singleC	DiP, OT
620	No	9	9-B	1000	McF_6	Inf	last	wholeT_0.01	DiP, OT
621	No	9	9-B	1000	McF_6	Inf	last	wholeT_0.5	DiP, OT
622	No	9	9-B	1000	McF_6	Inf	unif	singleC	DiP, DiP-A, OT, OT-A
623	No	9	9-B	1000	McF_6	Inf	unif	wholeT_0.01	DiP, DiP-A, OT, OT-A
624	No	9	9-B	1000	McF_6	Inf	unif	wholeT_0.5	DiP, DiP-A, OT, OT-A
625	No	9	9-B	200	Bozic	0	last	singleC	DiP, DiP-A, OT
626	No	9	9-B	200	Bozic	0	last	wholeT_0.01	DiP, DiP-A
627	No	9	9-B	200	Bozic	0	last	wholeT_0.5	DiP, DiP-A, OT, OT-A
628	No	9	9-B	200	Bozic	0	unif	singleC	DiP, DiP-A, OT, OT-A
629	No	9	9-B	200	Bozic	0	unif	wholeT_0.01	DiP, DiP-A
630	No	9	9-B	200	Bozic	0	unif	wholeT_0.5	DiP, DiP-A, OT-A
631	No	9	9-B	200	Bozic	Inf	last	singleC	DiP, DiP-A, OT, OT-A
632	No	9	9-B	200	Bozic	Inf	last	wholeT_0.01	DiP, DiP-A, OT, OT-A
633	No	9	9-B	200	Bozic	Inf	last	wholeT_0.5	DiP, DiP-A, OT, OT-A
634	No	9	9-B	200	Bozic	Inf	unif	singleC	DiP, DiP-A, OT, OT-A
635	No	9	9-B	200	Bozic	Inf	unif	wholeT_0.01	DiP, OT, OT-A
636	No	9	9-B	200	Bozic	Inf	unif	wholeT_0.5	DiP, DiP-A
637	No	9	9-B	200	exp	0	last	singleC	DiP, DiP-A, OT, OT-A

Table 12: *(continued)*

	Conjunction	Drivers	Tree	S.Size	Model	sh	S.Time	S.Type	Best method(s)
638	No	9	9-B	200	exp	0	last	wholeT_0.01	DiP, DiP-A
639	No	9	9-B	200	exp	0	last	wholeT_0.5	DiP, DiP-A, OT, OT-A
640	No	9	9-B	200	exp	0	unif	singleC	DiP, DiP-A, OT, OT-A
641	No	9	9-B	200	exp	0	unif	wholeT_0.01	DiP, DiP-A, OT
642	No	9	9-B	200	exp	0	unif	wholeT_0.5	DiP, DiP-A, OT, OT-A
643	No	9	9-B	200	exp	Inf	last	singleC	DiP, DiP-A, OT, OT-A
644	No	9	9-B	200	exp	Inf	last	wholeT_0.01	DiP, DiP-A
645	No	9	9-B	200	exp	Inf	last	wholeT_0.5	DiP, DiP-A, OT, OT-A
646	No	9	9-B	200	exp	Inf	unif	singleC	DiP, DiP-A, OT, OT-A
647	No	9	9-B	200	exp	Inf	unif	wholeT_0.01	DiP, DiP-A, OT, OT-A
648	No	9	9-B	200	exp	Inf	unif	wholeT_0.5	DiP, DiP-A, OT, OT-A
649	No	9	9-B	200	McF_4	0	last	singleC	OT, OT-A
650	No	9	9-B	200	McF_4	0	last	wholeT_0.01	DiP, DiP-A, OT, OT-A
651	No	9	9-B	200	McF_4	0	last	wholeT_0.5	DiP, DiP-A, OT, OT-A
652	No	9	9-B	200	McF_4	0	unif	singleC	DiP, DiP-A, OT, OT-A
653	No	9	9-B	200	McF_4	0	unif	wholeT_0.01	DiP, DiP-A, OT-A
654	No	9	9-B	200	McF_4	0	unif	wholeT_0.5	DiP, DiP-A, OT, OT-A
655	No	9	9-B	200	McF_4	Inf	last	singleC	OT, OT-A
656	No	9	9-B	200	McF_4	Inf	last	wholeT_0.01	OT, OT-A
657	No	9	9-B	200	McF_4	Inf	last	wholeT_0.5	DiP, DiP-A, OT, OT-A
658	No	9	9-B	200	McF_4	Inf	unif	singleC	DiP, DiP-A, OT, OT-A
659	No	9	9-B	200	McF_4	Inf	unif	wholeT_0.01	DiP, DiP-A, OT, OT-A
660	No	9	9-B	200	McF_4	Inf	unif	wholeT_0.5	DiP, DiP-A, OT, OT-A
661	No	9	9-B	200	McF_6	0	last	singleC	DiP, OT
662	No	9	9-B	200	McF_6	0	last	wholeT_0.01	DiP, OT
663	No	9	9-B	200	McF_6	0	last	wholeT_0.5	DiP, OT
664	No	9	9-B	200	McF_6	0	unif	singleC	DiP, OT, OT-A
665	No	9	9-B	200	McF_6	0	unif	wholeT_0.01	DiP, DiP-A, OT, OT-A
666	No	9	9-B	200	McF_6	0	unif	wholeT_0.5	OT, OT-A
667	No	9	9-B	200	McF_6	Inf	last	singleC	DiP, OT
668	No	9	9-B	200	McF_6	Inf	last	wholeT_0.01	DiP, OT
669	No	9	9-B	200	McF_6	Inf	last	wholeT_0.5	DiP, OT
670	No	9	9-B	200	McF_6	Inf	unif	singleC	DiP, OT, OT-A
671	No	9	9-B	200	McF_6	Inf	unif	wholeT_0.01	OT, OT-A
672	No	9	9-B	200	McF_6	Inf	unif	wholeT_0.5	OT, OT-A
673	No	9	9-B	100	Bozic	0	last	singleC	DiP, DiP-A, OT-A
674	No	9	9-B	100	Bozic	0	last	wholeT_0.01	DiP, DiP-A, OT
675	No	9	9-B	100	Bozic	0	last	wholeT_0.5	DiP, DiP-A, OT, OT-A
676	No	9	9-B	100	Bozic	0	unif	singleC	DiP, DiP-A, OT, OT-A
677	No	9	9-B	100	Bozic	0	unif	wholeT_0.01	DiP, DiP-A, OT

Table 12: *(continued)*

	Conjunction	Drivers	Tree	S.Size	Model	sh	S.Time	S.Type	Best method(s)
678	No	9	9-B	100	Bozic	0	unif	wholeT_0.5	DiP, DiP-A, OT, OT-A
679	No	9	9-B	100	Bozic	Inf	last	singleC	DiP, DiP-A, OT, OT-A
680	No	9	9-B	100	Bozic	Inf	last	wholeT_0.01	DiP
681	No	9	9-B	100	Bozic	Inf	last	wholeT_0.5	DiP, DiP-A, OT, OT-A
682	No	9	9-B	100	Bozic	Inf	unif	singleC	DiP, DiP-A, OT
683	No	9	9-B	100	Bozic	Inf	unif	wholeT_0.01	DiP, DiP-A, OT, OT-A
684	No	9	9-B	100	Bozic	Inf	unif	wholeT_0.5	DiP, DiP-A, OT-A
685	No	9	9-B	100	exp	0	last	singleC	DiP, DiP-A, OT, OT-A
686	No	9	9-B	100	exp	0	last	wholeT_0.01	DiP, DiP-A, OT, OT-A
687	No	9	9-B	100	exp	0	last	wholeT_0.5	DiP, DiP-A, OT, OT-A
688	No	9	9-B	100	exp	0	unif	singleC	DiP, DiP-A, OT, OT-A
689	No	9	9-B	100	exp	0	unif	wholeT_0.01	DiP, DiP-A, OT
690	No	9	9-B	100	exp	0	unif	wholeT_0.5	DiP, DiP-A, OT, OT-A
691	No	9	9-B	100	exp	Inf	last	singleC	DiP, DiP-A, OT, OT-A
692	No	9	9-B	100	exp	Inf	last	wholeT_0.01	DiP, DiP-A
693	No	9	9-B	100	exp	Inf	last	wholeT_0.5	DiP, DiP-A, OT, OT-A
694	No	9	9-B	100	exp	Inf	unif	singleC	DiP, DiP-A, OT
695	No	9	9-B	100	exp	Inf	unif	wholeT_0.01	DiP, DiP-A, OT, OT-A
696	No	9	9-B	100	exp	Inf	unif	wholeT_0.5	DiP, DiP-A, OT
697	No	9	9-B	100	McF_4	0	last	singleC	DiP, DiP-A, OT, OT-A
698	No	9	9-B	100	McF_4	0	last	wholeT_0.01	DiP, DiP-A, OT, OT-A
699	No	9	9-B	100	McF_4	0	last	wholeT_0.5	DiP, DiP-A, OT, OT-A
700	No	9	9-B	100	McF_4	0	unif	singleC	DiP, DiP-A, OT-A
701	No	9	9-B	100	McF_4	0	unif	wholeT_0.01	DiP, DiP-A
702	No	9	9-B	100	McF_4	0	unif	wholeT_0.5	DiP, DiP-A, OT, OT-A
703	No	9	9-B	100	McF_4	Inf	last	singleC	DiP, DiP-A, OT, OT-A
704	No	9	9-B	100	McF_4	Inf	last	wholeT_0.01	DiP, DiP-A, OT, OT-A
705	No	9	9-B	100	McF_4	Inf	last	wholeT_0.5	DiP, DiP-A, OT, OT-A
706	No	9	9-B	100	McF_4	Inf	unif	singleC	DiP, DiP-A, OT, OT-A
707	No	9	9-B	100	McF_4	Inf	unif	wholeT_0.01	DiP, DiP-A, OT, OT-A
708	No	9	9-B	100	McF_4	Inf	unif	wholeT_0.5	DiP, DiP-A, OT, OT-A
709	No	9	9-B	100	McF_6	0	last	singleC	DiP, DiP-A, OT
710	No	9	9-B	100	McF_6	0	last	wholeT_0.01	DiP, OT
711	No	9	9-B	100	McF_6	0	last	wholeT_0.5	DiP, OT
712	No	9	9-B	100	McF_6	0	unif	singleC	DiP, DiP-A, OT, OT-A
713	No	9	9-B	100	McF_6	0	unif	wholeT_0.01	DiP, DiP-A, OT, OT-A
714	No	9	9-B	100	McF_6	0	unif	wholeT_0.5	DiP, DiP-A, OT, OT-A
715	No	9	9-B	100	McF_6	Inf	last	singleC	DiP-A, OT
716	No	9	9-B	100	McF_6	Inf	last	wholeT_0.01	DiP, OT
717	No	9	9-B	100	McF_6	Inf	last	wholeT_0.5	DiP, DiP-A, OT

Table 12: *(continued)*

	Conjunction	Drivers	Tree	S.Size	Model	sh	S.Time	S.Type	Best method(s)
718	No	9	9-B	100	McF_6	Inf	unif	singleC	DiP, DiP-A, OT, OT-A
719	No	9	9-B	100	McF_6	Inf	unif	wholeT_0.01	DiP, OT
720	No	9	9-B	100	McF_6	Inf	unif	wholeT_0.5	DiP, DiP-A, OT, OT-A
721	No	7	7-B	1000	Bozic	0	last	singleC	DiP, DiP-A, OT, OT-A
722	No	7	7-B	1000	Bozic	0	last	wholeT_0.01	DiP, DiP-A, OT, OT-A
723	No	7	7-B	1000	Bozic	0	last	wholeT_0.5	DiP, DiP-A, OT, OT-A
724	No	7	7-B	1000	Bozic	0	unif	singleC	DiP, DiP-A, OT, OT-A
725	No	7	7-B	1000	Bozic	0	unif	wholeT_0.01	CBN-A, DiP, DiP-A, OT, OT-A
726	No	7	7-B	1000	Bozic	0	unif	wholeT_0.5	DiP, DiP-A, OT, OT-A
727	No	7	7-B	1000	Bozic	Inf	last	singleC	DiP-A, OT, OT-A
728	No	7	7-B	1000	Bozic	Inf	last	wholeT_0.01	none
729	No	7	7-B	1000	Bozic	Inf	last	wholeT_0.5	DiP-A, OT, OT-A
730	No	7	7-B	1000	Bozic	Inf	unif	singleC	CBN, CBN-A, DiP
731	No	7	7-B	1000	Bozic	Inf	unif	wholeT_0.01	CBN, CBN-A, OT, OT-A
732	No	7	7-B	1000	Bozic	Inf	unif	wholeT_0.5	CBN, CBN-A, DiP, DiP-A
733	No	7	7-B	1000	exp	0	last	singleC	DiP, DiP-A, OT
734	No	7	7-B	1000	exp	0	last	wholeT_0.01	DiP, DiP-A, OT, OT-A
735	No	7	7-B	1000	exp	0	last	wholeT_0.5	DiP, DiP-A, OT, OT-A
736	No	7	7-B	1000	exp	0	unif	singleC	DiP, DiP-A, OT, OT-A
737	No	7	7-B	1000	exp	0	unif	wholeT_0.01	DiP, DiP-A
738	No	7	7-B	1000	exp	0	unif	wholeT_0.5	DiP, DiP-A, OT, OT-A
739	No	7	7-B	1000	exp	Inf	last	singleC	DiP, DiP-A, OT, OT-A
740	No	7	7-B	1000	exp	Inf	last	wholeT_0.01	CBN, CBN-A, OT, OT-A
741	No	7	7-B	1000	exp	Inf	last	wholeT_0.5	DiP, DiP-A, OT, OT-A
742	No	7	7-B	1000	exp	Inf	unif	singleC	DiP, DiP-A
743	No	7	7-B	1000	exp	Inf	unif	wholeT_0.01	CBN, CBN-A, DiP, DiP-A, OT
744	No	7	7-B	1000	exp	Inf	unif	wholeT_0.5	DiP, DiP-A
745	No	7	7-B	1000	McF_4	0	last	singleC	DiP, DiP-A, OT, OT-A
746	No	7	7-B	1000	McF_4	0	last	wholeT_0.01	DiP, DiP-A, OT, OT-A
747	No	7	7-B	1000	McF_4	0	last	wholeT_0.5	DiP, DiP-A, OT, OT-A
748	No	7	7-B	1000	McF_4	0	unif	singleC	DiP, DiP-A, OT, OT-A
749	No	7	7-B	1000	McF_4	0	unif	wholeT_0.01	DiP, DiP-A, OT, OT-A
750	No	7	7-B	1000	McF_4	0	unif	wholeT_0.5	DiP, DiP-A, OT, OT-A
751	No	7	7-B	1000	McF_4	Inf	last	singleC	DiP, OT, OT-A
752	No	7	7-B	1000	McF_4	Inf	last	wholeT_0.01	DiP, DiP-A, OT, OT-A
753	No	7	7-B	1000	McF_4	Inf	last	wholeT_0.5	DiP, DiP-A, OT, OT-A
754	No	7	7-B	1000	McF_4	Inf	unif	singleC	DiP, DiP-A, OT, OT-A
755	No	7	7-B	1000	McF_4	Inf	unif	wholeT_0.01	DiP, DiP-A, OT, OT-A
756	No	7	7-B	1000	McF_4	Inf	unif	wholeT_0.5	DiP, DiP-A, OT, OT-A
757	No	7	7-B	1000	McF_6	0	last	singleC	DiP, DiP-A, OT, OT-A

Table 12: *(continued)*

	Conjunction	Drivers	Tree	S.Size	Model	sh	S.Time	S.Type	Best method(s)
758	No	7	7-B	1000	McF_6	0	last	wholeT_0.01	DiP, DiP-A, OT, OT-A
759	No	7	7-B	1000	McF_6	0	last	wholeT_0.5	DiP, DiP-A, OT, OT-A
760	No	7	7-B	1000	McF_6	0	unif	singleC	none
761	No	7	7-B	1000	McF_6	0	unif	wholeT_0.01	none
762	No	7	7-B	1000	McF_6	0	unif	wholeT_0.5	none
763	No	7	7-B	1000	McF_6	Inf	last	singleC	DiP, DiP-A, OT, OT-A
764	No	7	7-B	1000	McF_6	Inf	last	wholeT_0.01	DiP, DiP-A, OT, OT-A
765	No	7	7-B	1000	McF_6	Inf	last	wholeT_0.5	DiP-A, OT, OT-A
766	No	7	7-B	1000	McF_6	Inf	unif	singleC	none
767	No	7	7-B	1000	McF_6	Inf	unif	wholeT_0.01	none
768	No	7	7-B	1000	McF_6	Inf	unif	wholeT_0.5	none
769	No	7	7-B	200	Bozic	0	last	singleC	DiP, DiP-A, OT, OT-A
770	No	7	7-B	200	Bozic	0	last	wholeT_0.01	DiP, DiP-A, OT-A
771	No	7	7-B	200	Bozic	0	last	wholeT_0.5	DiP, DiP-A, OT-A
772	No	7	7-B	200	Bozic	0	unif	singleC	DiP, DiP-A, OT, OT-A
773	No	7	7-B	200	Bozic	0	unif	wholeT_0.01	DiP, DiP-A
774	No	7	7-B	200	Bozic	0	unif	wholeT_0.5	DiP, DiP-A, OT, OT-A
775	No	7	7-B	200	Bozic	Inf	last	singleC	DiP, DiP-A, OT, OT-A
776	No	7	7-B	200	Bozic	Inf	last	wholeT_0.01	CBN, CBN-A, OT, OT-A
777	No	7	7-B	200	Bozic	Inf	last	wholeT_0.5	DiP, DiP-A, OT, OT-A
778	No	7	7-B	200	Bozic	Inf	unif	singleC	DiP
779	No	7	7-B	200	Bozic	Inf	unif	wholeT_0.01	DiP-A, OT, OT-A
780	No	7	7-B	200	Bozic	Inf	unif	wholeT_0.5	DiP, DiP-A
781	No	7	7-B	200	exp	0	last	singleC	DiP, DiP-A, OT, OT-A
782	No	7	7-B	200	exp	0	last	wholeT_0.01	DiP, DiP-A
783	No	7	7-B	200	exp	0	last	wholeT_0.5	DiP, DiP-A, OT, OT-A
784	No	7	7-B	200	exp	0	unif	singleC	DiP, DiP-A, OT, OT-A
785	No	7	7-B	200	exp	0	unif	wholeT_0.01	DiP, DiP-A
786	No	7	7-B	200	exp	0	unif	wholeT_0.5	DiP, DiP-A, OT, OT-A
787	No	7	7-B	200	exp	Inf	last	singleC	DiP, DiP-A, OT, OT-A
788	No	7	7-B	200	exp	Inf	last	wholeT_0.01	DiP, OT
789	No	7	7-B	200	exp	Inf	last	wholeT_0.5	DiP, DiP-A, OT, OT-A
790	No	7	7-B	200	exp	Inf	unif	singleC	DiP, DiP-A
791	No	7	7-B	200	exp	Inf	unif	wholeT_0.01	DiP, DiP-A
792	No	7	7-B	200	exp	Inf	unif	wholeT_0.5	DiP, DiP-A
793	No	7	7-B	200	McF_4	0	last	singleC	DiP, DiP-A, OT, OT-A
794	No	7	7-B	200	McF_4	0	last	wholeT_0.01	DiP, DiP-A, OT, OT-A
795	No	7	7-B	200	McF_4	0	last	wholeT_0.5	DiP, DiP-A, OT, OT-A
796	No	7	7-B	200	McF_4	0	unif	singleC	DiP, DiP-A, OT, OT-A
797	No	7	7-B	200	McF_4	0	unif	wholeT_0.01	DiP, DiP-A, OT, OT-A

Table 12: *(continued)*

	Conjunction	Drivers	Tree	S.Size	Model	sh	S.Time	S.Type	Best method(s)
798	No	7	7-B	200	McF_4	0	unif	wholeT_0.5	DiP, DiP-A, OT, OT-A
799	No	7	7-B	200	McF_4	Inf	last	singleC	DiP, DiP-A, OT, OT-A
800	No	7	7-B	200	McF_4	Inf	last	wholeT_0.01	DiP, DiP-A, OT, OT-A
801	No	7	7-B	200	McF_4	Inf	last	wholeT_0.5	DiP, DiP-A, OT, OT-A
802	No	7	7-B	200	McF_4	Inf	unif	singleC	DiP, DiP-A, OT, OT-A
803	No	7	7-B	200	McF_4	Inf	unif	wholeT_0.01	DiP, DiP-A, OT, OT-A
804	No	7	7-B	200	McF_4	Inf	unif	wholeT_0.5	DiP, DiP-A, OT, OT-A
805	No	7	7-B	200	McF_6	0	last	singleC	DiP, DiP-A, OT, OT-A
806	No	7	7-B	200	McF_6	0	last	wholeT_0.01	DiP, OT, OT-A
807	No	7	7-B	200	McF_6	0	last	wholeT_0.5	DiP-A, OT
808	No	7	7-B	200	McF_6	0	unif	singleC	none
809	No	7	7-B	200	McF_6	0	unif	wholeT_0.01	none
810	No	7	7-B	200	McF_6	0	unif	wholeT_0.5	CBN, CBN-A, OT, OT-A
811	No	7	7-B	200	McF_6	Inf	last	singleC	DiP, DiP-A, OT, OT-A
812	No	7	7-B	200	McF_6	Inf	last	wholeT_0.01	DiP, OT, OT-A
813	No	7	7-B	200	McF_6	Inf	last	wholeT_0.5	DiP, DiP-A, OT, OT-A
814	No	7	7-B	200	McF_6	Inf	unif	singleC	CBN, CBN-A, OT-A
815	No	7	7-B	200	McF_6	Inf	unif	wholeT_0.01	CBN, CBN-A, DiP, DiP-A
816	No	7	7-B	200	McF_6	Inf	unif	wholeT_0.5	CBN, CBN-A, DiP, DiP-A
817	No	7	7-B	100	Bozic	0	last	singleC	DiP, DiP-A, OT
818	No	7	7-B	100	Bozic	0	last	wholeT_0.01	DiP, DiP-A, OT-A
819	No	7	7-B	100	Bozic	0	last	wholeT_0.5	DiP, DiP-A, OT, OT-A
820	No	7	7-B	100	Bozic	0	unif	singleC	DiP, DiP-A, OT, OT-A
821	No	7	7-B	100	Bozic	0	unif	wholeT_0.01	DiP, DiP-A
822	No	7	7-B	100	Bozic	0	unif	wholeT_0.5	DiP, DiP-A, OT, OT-A
823	No	7	7-B	100	Bozic	Inf	last	singleC	DiP, DiP-A, OT, OT-A
824	No	7	7-B	100	Bozic	Inf	last	wholeT_0.01	CBN, CBN-A, DiP
825	No	7	7-B	100	Bozic	Inf	last	wholeT_0.5	DiP, DiP-A, OT, OT-A
826	No	7	7-B	100	Bozic	Inf	unif	singleC	DiP, DiP-A
827	No	7	7-B	100	Bozic	Inf	unif	wholeT_0.01	DiP, DiP-A
828	No	7	7-B	100	Bozic	Inf	unif	wholeT_0.5	DiP, DiP-A
829	No	7	7-B	100	exp	0	last	singleC	DiP, DiP-A, OT, OT-A
830	No	7	7-B	100	exp	0	last	wholeT_0.01	DiP, DiP-A
831	No	7	7-B	100	exp	0	last	wholeT_0.5	DiP, DiP-A, OT, OT-A
832	No	7	7-B	100	exp	0	unif	singleC	DiP, DiP-A, OT, OT-A
833	No	7	7-B	100	exp	0	unif	wholeT_0.01	DiP, DiP-A, OT, OT-A
834	No	7	7-B	100	exp	0	unif	wholeT_0.5	DiP, DiP-A, OT, OT-A
835	No	7	7-B	100	exp	Inf	last	singleC	DiP, DiP-A, OT, OT-A
836	No	7	7-B	100	exp	Inf	last	wholeT_0.01	DiP, DiP-A
837	No	7	7-B	100	exp	Inf	last	wholeT_0.5	DiP, DiP-A, OT, OT-A

Table 12: *(continued)*

	Conjunction	Drivers	Tree	S.Size	Model	sh	S.Time	S.Type	Best method(s)
838	No	7	7-B	100	exp	Inf	unif	singleC	DiP, DiP-A
839	No	7	7-B	100	exp	Inf	unif	wholeT_0.01	DiP, DiP-A, OT, OT-A
840	No	7	7-B	100	exp	Inf	unif	wholeT_0.5	DiP, DiP-A
841	No	7	7-B	100	McF_4	0	last	singleC	DiP, DiP-A, OT, OT-A
842	No	7	7-B	100	McF_4	0	last	wholeT_0.01	DiP, DiP-A, OT, OT-A
843	No	7	7-B	100	McF_4	0	last	wholeT_0.5	DiP, DiP-A, OT, OT-A
844	No	7	7-B	100	McF_4	0	unif	singleC	DiP, DiP-A
845	No	7	7-B	100	McF_4	0	unif	wholeT_0.01	DiP, DiP-A, OT
846	No	7	7-B	100	McF_4	0	unif	wholeT_0.5	DiP, DiP-A, OT, OT-A
847	No	7	7-B	100	McF_4	Inf	last	singleC	DiP, DiP-A, OT, OT-A
848	No	7	7-B	100	McF_4	Inf	last	wholeT_0.01	DiP, DiP-A, OT, OT-A
849	No	7	7-B	100	McF_4	Inf	last	wholeT_0.5	DiP, DiP-A, OT, OT-A
850	No	7	7-B	100	McF_4	Inf	unif	singleC	DiP, DiP-A, OT, OT-A
851	No	7	7-B	100	McF_4	Inf	unif	wholeT_0.01	DiP, DiP-A
852	No	7	7-B	100	McF_4	Inf	unif	wholeT_0.5	DiP, DiP-A
853	No	7	7-B	100	McF_6	0	last	singleC	DiP, DiP-A, OT
854	No	7	7-B	100	McF_6	0	last	wholeT_0.01	DiP, OT, OT-A
855	No	7	7-B	100	McF_6	0	last	wholeT_0.5	DiP, DiP-A, OT, OT-A
856	No	7	7-B	100	McF_6	0	unif	singleC	CBN, CBN-A, DiP, OT
857	No	7	7-B	100	McF_6	0	unif	wholeT_0.01	CBN, CBN-A, DiP-A
858	No	7	7-B	100	McF_6	0	unif	wholeT_0.5	CBN, CBN-A
859	No	7	7-B	100	McF_6	Inf	last	singleC	DiP, DiP-A, OT, OT-A
860	No	7	7-B	100	McF_6	Inf	last	wholeT_0.01	DiP, OT, OT-A
861	No	7	7-B	100	McF_6	Inf	last	wholeT_0.5	DiP, DiP-A, OT, OT-A
862	No	7	7-B	100	McF_6	Inf	unif	singleC	CBN, CBN-A
863	No	7	7-B	100	McF_6	Inf	unif	wholeT_0.01	CBN, CBN-A
864	No	7	7-B	100	McF_6	Inf	unif	wholeT_0.5	CBN, CBN-A

5 Drivers Unknown (MCB)

5.1 Confidence sets (MCB), Diff, Drivers Unknown

Table 13: Confidence sets (method MCB) when Drivers are Unknown for measure Diff.

	Conjunction	Drivers	Tree	S.Size	Model	sh	S.Time	S.Type	Best method(s)
1	Yes	11	11-A	1000	Bozic	0	last	singleC	S1:OT, S1:OT-A
2	Yes	11	11-A	1000	Bozic	0	last	wholeT_0.01	S1:DiP, S1:DiP-A, S5:DiP, S5:DiP-A
3	Yes	11	11-A	1000	Bozic	0	last	wholeT_0.5	S1:OT, S1:OT-A
4	Yes	11	11-A	1000	Bozic	0	unif	singleC	S1:DiP, S1:DiP-A, S1:OT, S1:OT-A
5	Yes	11	11-A	1000	Bozic	0	unif	wholeT_0.01	J1:CBN-A, J1:DiP, J1:DiP-A, S5:CBN, S5:CBN-A, S5:DiP, S5:DiP-A, S5:OT, S5:OT-A
6	Yes	11	11-A	1000	Bozic	0	unif	wholeT_0.5	S1:DiP, S1:DiP-A, S1:OT, S1:OT-A, S5:DiP, S5:DiP-A, S5:OT, S5:OT-A
7	Yes	11	11-A	1000	Bozic	Inf	last	singleC	S5:DiP, S5:DiP-A, S5:OT, S5:OT-A
8	Yes	11	11-A	1000	Bozic	Inf	last	wholeT_0.01	J1:DiP, J1:DiP-A, J1:OT, J1:OT-A, J5:DiP, J5:DiP-A, J5:OT, J5:OT-A
9	Yes	11	11-A	1000	Bozic	Inf	last	wholeT_0.5	S5:DiP, S5:DiP-A, S5:OT, S5:OT-A
10	Yes	11	11-A	1000	Bozic	Inf	unif	singleC	S1:CBN, S1:CBN-A, S1:DiP, S1:DiP-A, S1:OT, S1:OT-A
11	Yes	11	11-A	1000	Bozic	Inf	unif	wholeT_0.01	J1:CBN, J1:CBN-A, S5:CBN, S5:CBN-A, S5:OT, S5:OT-A
12	Yes	11	11-A	1000	Bozic	Inf	unif	wholeT_0.5	J1:DiP, J1:DiP-A, J1:OT, J1:OT-A, S1:CBN, S1:CBN-A, S1:DiP-A, S1:OT, S1:OT-A, S5:DiP
13	Yes	11	11-A	1000	exp	0	last	singleC	S1:OT, S1:OT-A
14	Yes	11	11-A	1000	exp	0	last	wholeT_0.01	S1:DiP-A, S1:OT, S1:OT-A, S5:DiP-A, S5:OT-A
15	Yes	11	11-A	1000	exp	0	last	wholeT_0.5	S1:OT, S1:OT-A
16	Yes	11	11-A	1000	exp	0	unif	singleC	S1:DiP, S1:DiP-A, S1:OT, S1:OT-A, S5:DiP, S5:DiP-A, S5:OT, S5:OT-A
17	Yes	11	11-A	1000	exp	0	unif	wholeT_0.01	S1:CBN-A, S1:DiP, S1:DiP-A, S1:OT, S1:OT-A
18	Yes	11	11-A	1000	exp	0	unif	wholeT_0.5	S1:DiP, S1:DiP-A, S1:OT, S1:OT-A, S5:DiP, S5:DiP-A, S5:OT, S5:OT-A
19	Yes	11	11-A	1000	exp	Inf	last	singleC	S5:DiP, S5:DiP-A, S5:OT, S5:OT-A
20	Yes	11	11-A	1000	exp	Inf	last	wholeT_0.01	S5:DiP, S5:DiP-A, S5:OT, S5:OT-A
21	Yes	11	11-A	1000	exp	Inf	last	wholeT_0.5	S5:DiP, S5:DiP-A, S5:OT, S5:OT-A
22	Yes	11	11-A	1000	exp	Inf	unif	singleC	S1:CBN, S1:CBN-A, S1:OT, S1:OT-A
23	Yes	11	11-A	1000	exp	Inf	unif	wholeT_0.01	J1:CBN-A, J1:OT, J1:OT-A, J5:OT-A, S5:DiP, S5:DiP-A, S5:OT, S5:OT-A
24	Yes	11	11-A	1000	exp	Inf	unif	wholeT_0.5	S1:CBN, S1:CBN-A, S1:OT, S1:OT-A

Table 13: *(continued)*

	Conjunction	Drivers	Tree	S.Size	Model	sh	S.Time	S.Type	Best method(s)
25	Yes	11	11-A	1000	McF_4	0	last	singleC	S1:DiP-A, S1:OT, S1:OT-A
26	Yes	11	11-A	1000	McF_4	0	last	wholeT_0.01	S1:OT, S1:OT-A, S5:DiP, S5:DiP-A, S5:OT, S5:OT-A
27	Yes	11	11-A	1000	McF_4	0	last	wholeT_0.5	S1:DiP-A, S1:OT, S1:OT-A
28	Yes	11	11-A	1000	McF_4	0	unif	singleC	S1:DiP, S1:DiP-A, S1:OT, S1:OT-A
29	Yes	11	11-A	1000	McF_4	0	unif	wholeT_0.01	S1:OT, S1:OT-A, S5:DiP, S5:DiP-A, S5:OT, S5:OT-A
30	Yes	11	11-A	1000	McF_4	0	unif	wholeT_0.5	S1:DiP, S1:DiP-A, S1:OT, S1:OT-A, S5:DiP, S5:DiP-A, S5:OT, S5:OT-A
31	Yes	11	11-A	1000	McF_4	Inf	last	singleC	S1:OT, S1:OT-A
32	Yes	11	11-A	1000	McF_4	Inf	last	wholeT_0.01	S5:DiP, S5:OT, S5:OT-A
33	Yes	11	11-A	1000	McF_4	Inf	last	wholeT_0.5	S1:DiP-A, S1:OT, S1:OT-A
34	Yes	11	11-A	1000	McF_4	Inf	unif	singleC	S1:OT, S1:OT-A
35	Yes	11	11-A	1000	McF_4	Inf	unif	wholeT_0.01	S5:DiP, S5:DiP-A, S5:OT, S5:OT-A
36	Yes	11	11-A	1000	McF_4	Inf	unif	wholeT_0.5	S1:OT, S1:OT-A
37	Yes	11	11-A	1000	McF_6	0	last	singleC	S1:DiP, S1:OT
38	Yes	11	11-A	1000	McF_6	0	last	wholeT_0.01	S5:DiP
39	Yes	11	11-A	1000	McF_6	0	last	wholeT_0.5	S1:DiP, S5:DiP
40	Yes	11	11-A	1000	McF_6	0	unif	singleC	S1:DiP, S1:DiP-A
41	Yes	11	11-A	1000	McF_6	0	unif	wholeT_0.01	S1:DiP, S1:DiP-A, S1:OT, S1:OT-A
42	Yes	11	11-A	1000	McF_6	0	unif	wholeT_0.5	S1:DiP, S1:DiP-A, S1:OT, S1:OT-A
43	Yes	11	11-A	1000	McF_6	Inf	last	singleC	S1:DiP, S1:OT, S5:OT
44	Yes	11	11-A	1000	McF_6	Inf	last	wholeT_0.01	S5:OT
45	Yes	11	11-A	1000	McF_6	Inf	last	wholeT_0.5	S1:DiP, S1:OT
46	Yes	11	11-A	1000	McF_6	Inf	unif	singleC	S1:DiP, S1:DiP-A
47	Yes	11	11-A	1000	McF_6	Inf	unif	wholeT_0.01	S1:DiP, S1:DiP-A, S5:DiP, S5:DiP-A, S5:OT, S5:OT-A
48	Yes	11	11-A	1000	McF_6	Inf	unif	wholeT_0.5	S1:DiP, S1:DiP-A
49	Yes	11	11-A	200	Bozic	0	last	singleC	S1:OT, S1:OT-A
50	Yes	11	11-A	200	Bozic	0	last	wholeT_0.01	J1:OT, J1:OT-A, J5:OT-A, S5:OT, S5:OT-A
51	Yes	11	11-A	200	Bozic	0	last	wholeT_0.5	S1:OT, S1:OT-A
52	Yes	11	11-A	200	Bozic	0	unif	singleC	S1:OT, S1:OT-A, S5:OT
53	Yes	11	11-A	200	Bozic	0	unif	wholeT_0.01	J1:CBN, J1:CBN-A, J1:DiP, J1:DiP-A, J1:OT, J1:OT-A, S5:CBN, S5:CBN-A, S5:DiP, S5:DiP-A, S5:OT, S5:OT-A
54	Yes	11	11-A	200	Bozic	0	unif	wholeT_0.5	S1:OT, S1:OT-A, S5:DiP, S5:DiP-A, S5:OT, S5:OT-A
55	Yes	11	11-A	200	Bozic	Inf	last	singleC	S5:OT, S5:OT-A
56	Yes	11	11-A	200	Bozic	Inf	last	wholeT_0.01	J5:OT, J5:OT-A

Table 13: *(continued)*

	Conjunction	Drivers	Tree	S.Size	Model	sh	S.Time	S.Type	Best method(s)
57	Yes	11	11-A	200	Bozic	Inf	last	wholeT_0.5	S5:OT, S5:OT-A
58	Yes	11	11-A	200	Bozic	Inf	unif	singleC	J1:CBN, J1:CBN-A, J1:OT, J1:OT-A
59	Yes	11	11-A	200	Bozic	Inf	unif	wholeT_0.01	J5:OT, J5:OT-A, S5:CBN, S5:CBN-A, S5:OT, S5:OT-A
60	Yes	11	11-A	200	Bozic	Inf	unif	wholeT_0.5	J1:CBN-A, J1:OT, J1:OT-A
61	Yes	11	11-A	200	exp	0	last	singleC	S1:OT, S1:OT-A
62	Yes	11	11-A	200	exp	0	last	wholeT_0.01	S1:OT, S1:OT-A, S5:OT, S5:OT-A
63	Yes	11	11-A	200	exp	0	last	wholeT_0.5	S1:OT, S1:OT-A
64	Yes	11	11-A	200	exp	0	unif	singleC	S1:DiP, S1:DiP-A, S1:OT, S1:OT-A, S5:DiP, S5:DiP-A, S5:OT, S5:OT-A
65	Yes	11	11-A	200	exp	0	unif	wholeT_0.01	S1:OT, S1:OT-A
66	Yes	11	11-A	200	exp	0	unif	wholeT_0.5	S1:DiP, S1:DiP-A, S1:OT, S1:OT-A, S5:DiP, S5:DiP-A, S5:OT, S5:OT-A
67	Yes	11	11-A	200	exp	Inf	last	singleC	S5:OT, S5:OT-A
68	Yes	11	11-A	200	exp	Inf	last	wholeT_0.01	J5:OT, J5:OT-A
69	Yes	11	11-A	200	exp	Inf	last	wholeT_0.5	S5:OT, S5:OT-A
70	Yes	11	11-A	200	exp	Inf	unif	singleC	S1:OT, S1:OT-A
71	Yes	11	11-A	200	exp	Inf	unif	wholeT_0.01	J5:DiP-A, S5:CBN-A, S5:OT, S5:OT-A
72	Yes	11	11-A	200	exp	Inf	unif	wholeT_0.5	S1:OT, S1:OT-A
73	Yes	11	11-A	200	McF_4	0	last	singleC	S1:OT, S1:OT-A, S5:OT, S5:OT-A
74	Yes	11	11-A	200	McF_4	0	last	wholeT_0.01	S1:OT, S1:OT-A, S5:OT, S5:OT-A
75	Yes	11	11-A	200	McF_4	0	last	wholeT_0.5	S1:OT, S1:OT-A, S5:OT, S5:OT-A
76	Yes	11	11-A	200	McF_4	0	unif	singleC	S5:CBN-A, S5:OT, S5:OT-A
77	Yes	11	11-A	200	McF_4	0	unif	wholeT_0.01	S5:OT, S5:OT-A
78	Yes	11	11-A	200	McF_4	0	unif	wholeT_0.5	S5:OT, S5:OT-A
79	Yes	11	11-A	200	McF_4	Inf	last	singleC	S1:OT, S1:OT-A, S5:OT, S5:OT-A
80	Yes	11	11-A	200	McF_4	Inf	last	wholeT_0.01	S5:OT, S5:OT-A
81	Yes	11	11-A	200	McF_4	Inf	last	wholeT_0.5	S5:OT, S5:OT-A
82	Yes	11	11-A	200	McF_4	Inf	unif	singleC	S5:CBN-A, S5:OT, S5:OT-A
83	Yes	11	11-A	200	McF_4	Inf	unif	wholeT_0.01	S5:CBN-A, S5:OT, S5:OT-A
84	Yes	11	11-A	200	McF_4	Inf	unif	wholeT_0.5	S1:OT, S1:OT-A, S5:CBN-A, S5:OT, S5:OT-A
85	Yes	11	11-A	200	McF_6	0	last	singleC	S1:OT, S5:OT
86	Yes	11	11-A	200	McF_6	0	last	wholeT_0.01	S5:OT
87	Yes	11	11-A	200	McF_6	0	last	wholeT_0.5	S1:OT, S5:OT
88	Yes	11	11-A	200	McF_6	0	unif	singleC	J1:CBN, J1:CBN-A, S1:OT, S1:OT-A, S5:CBN, S5:CBN-A, S5:OT, S5:OT-A
89	Yes	11	11-A	200	McF_6	0	unif	wholeT_0.01	S1:OT, S1:OT-A, S5:CBN, S5:CBN-A, S5:OT, S5:OT-A

Table 13: *(continued)*

	Conjunction	Drivers	Tree	S.Size	Model	sh	S.Time	S.Type	Best method(s)
90	Yes	11	11-A	200	McF_6	0	unif	wholeT_0.5	J1:CBN, J1:CBN-A, S1:OT, S1:OT-A, S5:CBN, S5:CBN-A, S5:OT, S5:OT-A
91	Yes	11	11-A	200	McF_6	Inf	last	singleC	S1:OT, S5:OT
92	Yes	11	11-A	200	McF_6	Inf	last	wholeT_0.01	S5:OT, S5:OT-A
93	Yes	11	11-A	200	McF_6	Inf	last	wholeT_0.5	S1:OT, S5:OT
94	Yes	11	11-A	200	McF_6	Inf	unif	singleC	J1:CBN-A, S1:OT, S1:OT-A, S5:CBN, S5:CBN-A, S5:OT, S5:OT-A
95	Yes	11	11-A	200	McF_6	Inf	unif	wholeT_0.01	J1:CBN-A, S5:CBN, S5:CBN-A, S5:OT, S5:OT-A
96	Yes	11	11-A	200	McF_6	Inf	unif	wholeT_0.5	J1:CBN-A, J1:OT, S1:CBN-A, S1:OT, S1:OT-A, S5:CBN, S5:CBN-A, S5:OT, S5:OT-A
97	Yes	11	11-A	100	Bozic	0	last	singleC	S5:OT, S5:OT-A
98	Yes	11	11-A	100	Bozic	0	last	wholeT_0.01	J5:OT, J5:OT-A, S5:OT, S5:OT-A
99	Yes	11	11-A	100	Bozic	0	last	wholeT_0.5	S1:OT, S1:OT-A, S5:OT, S5:OT-A
100	Yes	11	11-A	100	Bozic	0	unif	singleC	J1:OT, J1:OT-A, S5:DiP, S5:DiP-A, S5:OT, S5:OT-A
101	Yes	11	11-A	100	Bozic	0	unif	wholeT_0.01	J1:OT, J1:OT-A, S5:OT, S5:OT-A
102	Yes	11	11-A	100	Bozic	0	unif	wholeT_0.5	S5:DiP, S5:DiP-A, S5:OT, S5:OT-A
103	Yes	11	11-A	100	Bozic	Inf	last	singleC	J1:OT, J1:OT-A, J5:OT, J5:OT-A, S5:OT, S5:OT-A
104	Yes	11	11-A	100	Bozic	Inf	last	wholeT_0.01	J5:CBN-A, J5:OT, J5:OT-A
105	Yes	11	11-A	100	Bozic	Inf	last	wholeT_0.5	J1:OT, J1:OT-A, J5:OT, J5:OT-A, S5:OT, S5:OT-A
106	Yes	11	11-A	100	Bozic	Inf	unif	singleC	S5:OT, S5:OT-A
107	Yes	11	11-A	100	Bozic	Inf	unif	wholeT_0.01	J5:OT, J5:OT-A, S5:OT, S5:OT-A
108	Yes	11	11-A	100	Bozic	Inf	unif	wholeT_0.5	S5:DiP-A, S5:OT, S5:OT-A
109	Yes	11	11-A	100	exp	0	last	singleC	S1:OT, S1:OT-A
110	Yes	11	11-A	100	exp	0	last	wholeT_0.01	S1:OT, S1:OT-A, S5:OT, S5:OT-A
111	Yes	11	11-A	100	exp	0	last	wholeT_0.5	S1:OT-A
112	Yes	11	11-A	100	exp	0	unif	singleC	S1:OT, S1:OT-A, S5:DiP, S5:DiP-A, S5:OT, S5:OT-A
113	Yes	11	11-A	100	exp	0	unif	wholeT_0.01	J1:OT, J1:OT-A, S1:OT, S1:OT-A, S5:DiP, S5:DiP-A, S5:OT, S5:OT-A
114	Yes	11	11-A	100	exp	0	unif	wholeT_0.5	S1:OT, S1:OT-A, S5:DiP, S5:DiP-A, S5:OT, S5:OT-A
115	Yes	11	11-A	100	exp	Inf	last	singleC	S5:OT, S5:OT-A
116	Yes	11	11-A	100	exp	Inf	last	wholeT_0.01	J5:CBN-A, J5:OT, J5:OT-A
117	Yes	11	11-A	100	exp	Inf	last	wholeT_0.5	S5:OT, S5:OT-A

Table 13: *(continued)*

	Conjunction	Drivers	Tree	S.Size	Model	sh	S.Time	S.Type	Best method(s)
118	Yes	11	11-A	100	exp	Inf	unif	singleC	J1:CBN-A, J1:OT, J1:OT-A, S1:OT-A, S5:DiP, S5:DiP-A, S5:OT, S5:OT-A
119	Yes	11	11-A	100	exp	Inf	unif	wholeT_0.01	J5:OT, J5:OT-A, S5:OT, S5:OT-A
120	Yes	11	11-A	100	exp	Inf	unif	wholeT_0.5	J1:OT, J1:OT-A, S5:DiP, S5:DiP-A, S5:OT, S5:OT-A
121	Yes	11	11-A	100	McF_4	0	last	singleC	S5:OT, S5:OT-A
122	Yes	11	11-A	100	McF_4	0	last	wholeT_0.01	S5:OT, S5:OT-A
123	Yes	11	11-A	100	McF_4	0	last	wholeT_0.5	S5:OT, S5:OT-A
124	Yes	11	11-A	100	McF_4	0	unif	singleC	S5:CBN, S5:CBN-A, S5:OT, S5:OT-A
125	Yes	11	11-A	100	McF_4	0	unif	wholeT_0.01	S5:CBN-A, S5:OT, S5:OT-A
126	Yes	11	11-A	100	McF_4	0	unif	wholeT_0.5	S5:CBN, S5:CBN-A, S5:OT, S5:OT-A
127	Yes	11	11-A	100	McF_4	Inf	last	singleC	S1:OT, S1:OT-A, S5:OT, S5:OT-A
128	Yes	11	11-A	100	McF_4	Inf	last	wholeT_0.01	S5:OT, S5:OT-A
129	Yes	11	11-A	100	McF_4	Inf	last	wholeT_0.5	S5:OT, S5:OT-A
130	Yes	11	11-A	100	McF_4	Inf	unif	singleC	S5:CBN-A, S5:OT, S5:OT-A
131	Yes	11	11-A	100	McF_4	Inf	unif	wholeT_0.01	S5:CBN-A, S5:OT, S5:OT-A
132	Yes	11	11-A	100	McF_4	Inf	unif	wholeT_0.5	S5:CBN, S5:CBN-A, S5:OT, S5:OT-A
133	Yes	11	11-A	100	McF_6	0	last	singleC	S1:OT, S5:OT
134	Yes	11	11-A	100	McF_6	0	last	wholeT_0.01	S5:OT, S5:OT-A
135	Yes	11	11-A	100	McF_6	0	last	wholeT_0.5	S5:OT
136	Yes	11	11-A	100	McF_6	0	unif	singleC	J1:CBN-A, S5:CBN, S5:CBN-A, S5:OT, S5:OT-A
137	Yes	11	11-A	100	McF_6	0	unif	wholeT_0.01	S5:CBN, S5:OT, S5:OT-A
138	Yes	11	11-A	100	McF_6	0	unif	wholeT_0.5	J1:CBN, J1:CBN-A, S1:OT, S1:OT-A, S5:CBN, S5:CBN-A, S5:OT, S5:OT-A
139	Yes	11	11-A	100	McF_6	Inf	last	singleC	S5:OT
140	Yes	11	11-A	100	McF_6	Inf	last	wholeT_0.01	S5:OT, S5:OT-A
141	Yes	11	11-A	100	McF_6	Inf	last	wholeT_0.5	S5:OT
142	Yes	11	11-A	100	McF_6	Inf	unif	singleC	J1:CBN, J1:CBN-A, S1:OT, S1:OT-A, S5:CBN, S5:CBN-A, S5:OT, S5:OT-A
143	Yes	11	11-A	100	McF_6	Inf	unif	wholeT_0.01	S5:CBN, S5:CBN-A, S5:OT, S5:OT-A
144	Yes	11	11-A	100	McF_6	Inf	unif	wholeT_0.5	J1:CBN-A, S1:OT, S1:OT-A, S5:CBN, S5:CBN-A, S5:OT, S5:OT-A
145	Yes	9	9-A	1000	Bozic	0	last	singleC	S1:OT, S1:OT-A
146	Yes	9	9-A	1000	Bozic	0	last	wholeT_0.01	S1:DiP, S1:DiP-A, S1:OT, S1:OT-A, S5:DiP, S5:DiP-A, S5:OT, S5:OT-A
147	Yes	9	9-A	1000	Bozic	0	last	wholeT_0.5	S1:OT, S1:OT-A
148	Yes	9	9-A	1000	Bozic	0	unif	singleC	S1:DiP, S1:DiP-A, S1:OT, S1:OT-A, S5:DiP, S5:DiP-A, S5:OT, S5:OT-A

Table 13: *(continued)*

	Conjunction	Drivers	Tree	S.Size	Model	sh	S.Time	S.Type	Best method(s)
149	Yes	9	9-A	1000	Bozic	0	unif	wholeT_0.01	J1:DiP, J1:DiP-A, J1:OT, J1:OT-A, S1:DiP, S1:DiP-A, S1:OT, S1:OT-A
150	Yes	9	9-A	1000	Bozic	0	unif	wholeT_0.5	S1:DiP, S1:DiP-A, S1:OT, S1:OT-A, S5:DiP, S5:DiP-A, S5:OT, S5:OT-A
151	Yes	9	9-A	1000	Bozic	Inf	last	singleC	S5:DiP, S5:DiP-A, S5:OT, S5:OT-A
152	Yes	9	9-A	1000	Bozic	Inf	last	wholeT_0.01	J5:DiP, J5:DiP-A, J5:OT, J5:OT-A
153	Yes	9	9-A	1000	Bozic	Inf	last	wholeT_0.5	S5:DiP-A, S5:OT, S5:OT-A
154	Yes	9	9-A	1000	Bozic	Inf	unif	singleC	S1:OT, S1:OT-A
155	Yes	9	9-A	1000	Bozic	Inf	unif	wholeT_0.01	S5:DiP, S5:DiP-A, S5:OT, S5:OT-A
156	Yes	9	9-A	1000	Bozic	Inf	unif	wholeT_0.5	S1:CBN, S1:OT, S1:OT-A
157	Yes	9	9-A	1000	exp	0	last	singleC	J1:OT, S1:DiP, S1:DiP-A, S1:OT, S1:OT-A, S5:OT, S5:OT-A
158	Yes	9	9-A	1000	exp	0	last	wholeT_0.01	S1:DiP-A, S1:OT, S1:OT-A
159	Yes	9	9-A	1000	exp	0	last	wholeT_0.5	S1:DiP, S1:DiP-A, S1:OT, S1:OT-A, S5:DiP, S5:DiP-A, S5:OT
160	Yes	9	9-A	1000	exp	0	unif	singleC	S1:DiP, S1:DiP-A, S1:OT, S1:OT-A, S5:DiP, S5:DiP-A, S5:OT, S5:OT-A
161	Yes	9	9-A	1000	exp	0	unif	wholeT_0.01	S1:DiP, S1:DiP-A, S1:OT, S1:OT-A
162	Yes	9	9-A	1000	exp	0	unif	wholeT_0.5	S1:DiP, S1:DiP-A, S1:OT, S1:OT-A, S5:DiP, S5:DiP-A, S5:OT, S5:OT-A
163	Yes	9	9-A	1000	exp	Inf	last	singleC	S5:DiP, S5:DiP-A, S5:OT, S5:OT-A
164	Yes	9	9-A	1000	exp	Inf	last	wholeT_0.01	J5:DiP, J5:DiP-A, J5:OT, J5:OT-A, S5:CBN-A
165	Yes	9	9-A	1000	exp	Inf	last	wholeT_0.5	S5:DiP, S5:DiP-A, S5:OT, S5:OT-A
166	Yes	9	9-A	1000	exp	Inf	unif	singleC	S1:OT, S1:OT-A
167	Yes	9	9-A	1000	exp	Inf	unif	wholeT_0.01	J1:DiP, J1:DiP-A, J1:OT, J1:OT-A
168	Yes	9	9-A	1000	exp	Inf	unif	wholeT_0.5	S1:OT, S1:OT-A
169	Yes	9	9-A	1000	McF_4	0	last	singleC	S1:DiP, S1:DiP-A, S1:OT, S1:OT-A, S5:DiP
170	Yes	9	9-A	1000	McF_4	0	last	wholeT_0.01	S5:DiP, S5:DiP-A
171	Yes	9	9-A	1000	McF_4	0	last	wholeT_0.5	S1:DiP, S1:DiP-A, S1:OT, S1:OT-A
172	Yes	9	9-A	1000	McF_4	0	unif	singleC	S1:DiP-A, S1:OT, S1:OT-A
173	Yes	9	9-A	1000	McF_4	0	unif	wholeT_0.01	S5:CBN-A, S5:DiP, S5:DiP-A, S5:OT, S5:OT-A
174	Yes	9	9-A	1000	McF_4	0	unif	wholeT_0.5	S1:DiP-A, S1:OT, S1:OT-A
175	Yes	9	9-A	1000	McF_4	Inf	last	singleC	S1:OT, S1:OT-A
176	Yes	9	9-A	1000	McF_4	Inf	last	wholeT_0.01	S5:OT
177	Yes	9	9-A	1000	McF_4	Inf	last	wholeT_0.5	S1:DiP, S1:DiP-A, S1:OT, S1:OT-A
178	Yes	9	9-A	1000	McF_4	Inf	unif	singleC	S1:OT, S1:OT-A

Table 13: *(continued)*

	Conjunction	Drivers	Tree	S.Size	Model	sh	S.Time	S.Type	Best method(s)
179	Yes	9	9-A	1000	McF_4	Inf	unif	wholeT_0.01	S5:CBN-A, S5:DiP, S5:DiP-A, S5:OT, S5:OT-A
180	Yes	9	9-A	1000	McF_4	Inf	unif	wholeT_0.5	S1:OT, S1:OT-A
181	Yes	9	9-A	1000	McF_6	0	last	singleC	S1:OT, S5:OT
182	Yes	9	9-A	1000	McF_6	0	last	wholeT_0.01	S5:DiP, S5:OT
183	Yes	9	9-A	1000	McF_6	0	last	wholeT_0.5	S1:OT, S5:OT
184	Yes	9	9-A	1000	McF_6	0	unif	singleC	S1:DiP, S1:DiP-A
185	Yes	9	9-A	1000	McF_6	0	unif	wholeT_0.01	S5:CBN, S5:CBN-A, S5:DiP, S5:DiP-A
186	Yes	9	9-A	1000	McF_6	0	unif	wholeT_0.5	S1:CBN-A, S1:DiP, S1:DiP-A
187	Yes	9	9-A	1000	McF_6	Inf	last	singleC	S5:DiP
188	Yes	9	9-A	1000	McF_6	Inf	last	wholeT_0.01	S5:OT
189	Yes	9	9-A	1000	McF_6	Inf	last	wholeT_0.5	S5:DiP
190	Yes	9	9-A	1000	McF_6	Inf	unif	singleC	S1:CBN, S1:CBN-A, S1:DiP, S1:DiP-A, S1:OT, S1:OT-A, S5:CBN-A
191	Yes	9	9-A	1000	McF_6	Inf	unif	wholeT_0.01	J1:CBN, J1:CBN-A, S5:CBN, S5:CBN-A
192	Yes	9	9-A	1000	McF_6	Inf	unif	wholeT_0.5	S1:CBN, S1:CBN-A, S1:DiP, S1:DiP-A, S1:OT, S1:OT-A, S5:CBN, S5:CBN-A
193	Yes	9	9-A	200	Bozic	0	last	singleC	S1:OT, S1:OT-A
194	Yes	9	9-A	200	Bozic	0	last	wholeT_0.01	S5:OT, S5:OT-A
195	Yes	9	9-A	200	Bozic	0	last	wholeT_0.5	S1:OT, S1:OT-A
196	Yes	9	9-A	200	Bozic	0	unif	singleC	S1:OT, S1:OT-A, S5:DiP, S5:DiP-A, S5:OT, S5:OT-A
197	Yes	9	9-A	200	Bozic	0	unif	wholeT_0.01	J1:DiP, J1:DiP-A, J1:OT, J1:OT-A, S5:DiP-A, S5:OT, S5:OT-A
198	Yes	9	9-A	200	Bozic	0	unif	wholeT_0.5	S1:DiP-A, S1:OT, S1:OT-A, S5:DiP, S5:DiP-A, S5:OT, S5:OT-A
199	Yes	9	9-A	200	Bozic	Inf	last	singleC	S5:OT, S5:OT-A
200	Yes	9	9-A	200	Bozic	Inf	last	wholeT_0.01	J5:OT, J5:OT-A
201	Yes	9	9-A	200	Bozic	Inf	last	wholeT_0.5	S5:OT, S5:OT-A
202	Yes	9	9-A	200	Bozic	Inf	unif	singleC	J1:DiP, J1:DiP-A, J1:OT, J1:OT-A, S5:DiP, S5:DiP-A, S5:OT, S5:OT-A
203	Yes	9	9-A	200	Bozic	Inf	unif	wholeT_0.01	S5:OT, S5:OT-A
204	Yes	9	9-A	200	Bozic	Inf	unif	wholeT_0.5	J1:DiP, J1:DiP-A, J1:OT, J1:OT-A, S5:DiP, S5:DiP-A, S5:OT, S5:OT-A
205	Yes	9	9-A	200	exp	0	last	singleC	S1:OT, S1:OT-A
206	Yes	9	9-A	200	exp	0	last	wholeT_0.01	S1:OT, S1:OT-A
207	Yes	9	9-A	200	exp	0	last	wholeT_0.5	S1:OT, S1:OT-A
208	Yes	9	9-A	200	exp	0	unif	singleC	S1:DiP, S1:DiP-A, S1:OT, S1:OT-A, S5:DiP, S5:DiP-A, S5:OT, S5:OT-A

Table 13: *(continued)*

	Conjunction	Drivers	Tree	S.Size	Model	sh	S.Time	S.Type	Best method(s)
209	Yes	9	9-A	200	exp	0	unif	wholeT_0.01	S1:OT, S1:OT-A, S5:DiP, S5:DiP-A, S5:OT
210	Yes	9	9-A	200	exp	0	unif	wholeT_0.5	S1:DiP, S1:DiP-A, S1:OT, S1:OT-A, S5:DiP, S5:DiP-A, S5:OT, S5:OT-A
211	Yes	9	9-A	200	exp	Inf	last	singleC	S5:OT, S5:OT-A
212	Yes	9	9-A	200	exp	Inf	last	wholeT_0.01	J5:OT, J5:OT-A
213	Yes	9	9-A	200	exp	Inf	last	wholeT_0.5	S5:OT, S5:OT-A
214	Yes	9	9-A	200	exp	Inf	unif	singleC	J1:DiP, J1:DiP-A, J1:OT, J1:OT-A, S1:OT, S1:OT-A, S5:DiP, S5:DiP-A, S5:OT, S5:OT-A
215	Yes	9	9-A	200	exp	Inf	unif	wholeT_0.01	J1:OT-A, J5:OT, S5:OT, S5:OT-A
216	Yes	9	9-A	200	exp	Inf	unif	wholeT_0.5	S1:OT, S1:OT-A
217	Yes	9	9-A	200	McF_4	0	last	singleC	S5:OT, S5:OT-A
218	Yes	9	9-A	200	McF_4	0	last	wholeT_0.01	S5:OT, S5:OT-A
219	Yes	9	9-A	200	McF_4	0	last	wholeT_0.5	S5:OT, S5:OT-A
220	Yes	9	9-A	200	McF_4	0	unif	singleC	S1:OT, S1:OT-A, S5:CBN-A, S5:OT, S5:OT-A
221	Yes	9	9-A	200	McF_4	0	unif	wholeT_0.01	S5:OT, S5:OT-A
222	Yes	9	9-A	200	McF_4	0	unif	wholeT_0.5	S1:OT, S1:OT-A, S5:OT, S5:OT-A
223	Yes	9	9-A	200	McF_4	Inf	last	singleC	S5:OT, S5:OT-A
224	Yes	9	9-A	200	McF_4	Inf	last	wholeT_0.01	S5:OT
225	Yes	9	9-A	200	McF_4	Inf	last	wholeT_0.5	S5:OT, S5:OT-A
226	Yes	9	9-A	200	McF_4	Inf	unif	singleC	S5:OT, S5:OT-A
227	Yes	9	9-A	200	McF_4	Inf	unif	wholeT_0.01	S5:CBN-A, S5:OT, S5:OT-A
228	Yes	9	9-A	200	McF_4	Inf	unif	wholeT_0.5	S1:OT, S1:OT-A, S5:CBN-A, S5:OT, S5:OT-A
229	Yes	9	9-A	200	McF_6	0	last	singleC	S5:OT
230	Yes	9	9-A	200	McF_6	0	last	wholeT_0.01	S5:OT
231	Yes	9	9-A	200	McF_6	0	last	wholeT_0.5	S5:OT
232	Yes	9	9-A	200	McF_6	0	unif	singleC	S5:CBN, S5:CBN-A
233	Yes	9	9-A	200	McF_6	0	unif	wholeT_0.01	J1:CBN, J1:CBN-A, S5:CBN, S5:CBN-A, S5:OT, S5:OT-A
234	Yes	9	9-A	200	McF_6	0	unif	wholeT_0.5	J1:CBN, J1:CBN-A, S5:CBN, S5:CBN-A, S5:OT, S5:OT-A
235	Yes	9	9-A	200	McF_6	Inf	last	singleC	S5:OT
236	Yes	9	9-A	200	McF_6	Inf	last	wholeT_0.01	S5:OT
237	Yes	9	9-A	200	McF_6	Inf	last	wholeT_0.5	S5:OT
238	Yes	9	9-A	200	McF_6	Inf	unif	singleC	J1:CBN, J1:CBN-A, S5:CBN, S5:CBN-A
239	Yes	9	9-A	200	McF_6	Inf	unif	wholeT_0.01	J1:CBN, J1:CBN-A, S5:CBN, S5:CBN-A
240	Yes	9	9-A	200	McF_6	Inf	unif	wholeT_0.5	S5:CBN, S5:CBN-A

Table 13: *(continued)*

	Conjunction	Drivers	Tree	S.Size	Model	sh	S.Time	S.Type	Best method(s)
241	Yes	9	9-A	100	Bozic	0	last	singleC	S5:OT, S5:OT-A
242	Yes	9	9-A	100	Bozic	0	last	wholeT_0.01	S5:OT, S5:OT-A
243	Yes	9	9-A	100	Bozic	0	last	wholeT_0.5	J1:DiP, J1:DiP-A, J1:OT, J1:OT-A, S5:DiP, S5:DiP-A, S5:OT, S5:OT-A
244	Yes	9	9-A	100	Bozic	0	unif	singleC	S5:DiP, S5:DiP-A, S5:OT, S5:OT-A
245	Yes	9	9-A	100	Bozic	0	unif	wholeT_0.01	J1:OT, J1:OT-A, S5:DiP, S5:DiP-A, S5:OT, S5:OT-A
246	Yes	9	9-A	100	Bozic	0	unif	wholeT_0.5	S5:DiP, S5:DiP-A, S5:OT, S5:OT-A
247	Yes	9	9-A	100	Bozic	Inf	last	singleC	J5:OT, J5:OT-A, S5:OT, S5:OT-A
248	Yes	9	9-A	100	Bozic	Inf	last	wholeT_0.01	J5:OT, J5:OT-A
249	Yes	9	9-A	100	Bozic	Inf	last	wholeT_0.5	J5:OT, J5:OT-A, S5:OT, S5:OT-A
250	Yes	9	9-A	100	Bozic	Inf	unif	singleC	S5:DiP-A, S5:OT, S5:OT-A
251	Yes	9	9-A	100	Bozic	Inf	unif	wholeT_0.01	J5:OT, J5:OT-A, S5:OT, S5:OT-A
252	Yes	9	9-A	100	Bozic	Inf	unif	wholeT_0.5	J1:OT-A, S5:DiP, S5:DiP-A, S5:OT, S5:OT-A
253	Yes	9	9-A	100	exp	0	last	singleC	S1:OT, S1:OT-A, S5:DiP, S5:DiP-A, S5:OT, S5:OT-A
254	Yes	9	9-A	100	exp	0	last	wholeT_0.01	J1:OT, S1:OT, S1:OT-A, S5:OT, S5:OT-A
255	Yes	9	9-A	100	exp	0	last	wholeT_0.5	S1:OT, S1:OT-A, S5:DiP, S5:DiP-A, S5:OT, S5:OT-A
256	Yes	9	9-A	100	exp	0	unif	singleC	S1:OT, S1:OT-A, S5:DiP, S5:DiP-A, S5:OT, S5:OT-A
257	Yes	9	9-A	100	exp	0	unif	wholeT_0.01	S5:DiP, S5:DiP-A, S5:OT, S5:OT-A
258	Yes	9	9-A	100	exp	0	unif	wholeT_0.5	S1:DiP, S1:OT, S1:OT-A, S5:DiP, S5:DiP-A, S5:OT, S5:OT-A
259	Yes	9	9-A	100	exp	Inf	last	singleC	S5:OT, S5:OT-A
260	Yes	9	9-A	100	exp	Inf	last	wholeT_0.01	J5:CBN-A, J5:OT, J5:OT-A
261	Yes	9	9-A	100	exp	Inf	last	wholeT_0.5	S5:OT, S5:OT-A
262	Yes	9	9-A	100	exp	Inf	unif	singleC	S5:DiP, S5:DiP-A, S5:OT, S5:OT-A
263	Yes	9	9-A	100	exp	Inf	unif	wholeT_0.01	J5:OT, S5:OT, S5:OT-A
264	Yes	9	9-A	100	exp	Inf	unif	wholeT_0.5	J1:DiP, J1:DiP-A, S5:DiP, S5:DiP-A, S5:OT, S5:OT-A
265	Yes	9	9-A	100	McF_4	0	last	singleC	S5:OT, S5:OT-A
266	Yes	9	9-A	100	McF_4	0	last	wholeT_0.01	S5:OT, S5:OT-A
267	Yes	9	9-A	100	McF_4	0	last	wholeT_0.5	S5:OT, S5:OT-A
268	Yes	9	9-A	100	McF_4	0	unif	singleC	S5:CBN-A, S5:OT, S5:OT-A
269	Yes	9	9-A	100	McF_4	0	unif	wholeT_0.01	S5:OT, S5:OT-A
270	Yes	9	9-A	100	McF_4	0	unif	wholeT_0.5	S5:OT, S5:OT-A
271	Yes	9	9-A	100	McF_4	Inf	last	singleC	S5:OT, S5:OT-A

Table 13: *(continued)*

	Conjunction	Drivers	Tree	S.Size	Model	sh	S.Time	S.Type	Best method(s)
272	Yes	9	9-A	100	McF_4	Inf	last	wholeT_0.01	S5:OT
273	Yes	9	9-A	100	McF_4	Inf	last	wholeT_0.5	S5:OT, S5:OT-A
274	Yes	9	9-A	100	McF_4	Inf	unif	singleC	S5:CBN-A, S5:OT, S5:OT-A
275	Yes	9	9-A	100	McF_4	Inf	unif	wholeT_0.01	S5:CBN-A, S5:OT, S5:OT-A
276	Yes	9	9-A	100	McF_4	Inf	unif	wholeT_0.5	S5:OT, S5:OT-A
277	Yes	9	9-A	100	McF_6	0	last	singleC	S5:OT
278	Yes	9	9-A	100	McF_6	0	last	wholeT_0.01	S5:OT
279	Yes	9	9-A	100	McF_6	0	last	wholeT_0.5	S5:OT
280	Yes	9	9-A	100	McF_6	0	unif	singleC	J1:CBN, J1:CBN-A, S5:CBN, S5:CBN-A, S5:OT, S5:OT-A
281	Yes	9	9-A	100	McF_6	0	unif	wholeT_0.01	S5:CBN, S5:CBN-A, S5:OT, S5:OT-A
282	Yes	9	9-A	100	McF_6	0	unif	wholeT_0.5	J1:CBN, J1:CBN-A, S5:CBN, S5:CBN-A, S5:OT, S5:OT-A
283	Yes	9	9-A	100	McF_6	Inf	last	singleC	S5:OT
284	Yes	9	9-A	100	McF_6	Inf	last	wholeT_0.01	S5:OT, S5:OT-A
285	Yes	9	9-A	100	McF_6	Inf	last	wholeT_0.5	S5:OT
286	Yes	9	9-A	100	McF_6	Inf	unif	singleC	J1:CBN-A, S5:CBN, S5:CBN-A, S5:OT, S5:OT-A
287	Yes	9	9-A	100	McF_6	Inf	unif	wholeT_0.01	S5:CBN, S5:CBN-A, S5:OT, S5:OT-A
288	Yes	9	9-A	100	McF_6	Inf	unif	wholeT_0.5	J1:CBN-A, S5:CBN, S5:CBN-A, S5:OT, S5:OT-A
289	Yes	7	7-A	1000	Bozic	0	last	singleC	S1:OT-A, S5:OT-A
290	Yes	7	7-A	1000	Bozic	0	last	wholeT_0.01	J1:DiP-A, J1:OT-A, J5:DiP-A, J5:OT-A, S1:DiP-A, S1:OT-A, S5:DiP-A, S5:OT-A
291	Yes	7	7-A	1000	Bozic	0	last	wholeT_0.5	S1:OT-A
292	Yes	7	7-A	1000	Bozic	0	unif	singleC	S1:OT, S1:OT-A
293	Yes	7	7-A	1000	Bozic	0	unif	wholeT_0.01	J5:CBN, J5:CBN-A, J5:DiP-A, J5:OT, J5:OT-A
294	Yes	7	7-A	1000	Bozic	0	unif	wholeT_0.5	J1:DiP, J1:DiP-A, J1:OT, J5:DiP, J5:DiP-A, S1:CBN, S1:CBN-A, S1:DiP, S1:DiP-A, S1:OT, S1:OT-A, S5:DiP, S5:DiP-A
295	Yes	7	7-A	1000	Bozic	Inf	last	singleC	J1:DiP-A, J1:OT-A, J5:DiP-A, J5:OT-A, S5:DiP-A, S5:OT-A
296	Yes	7	7-A	1000	Bozic	Inf	last	wholeT_0.01	J5:DiP-A, J5:OT-A
297	Yes	7	7-A	1000	Bozic	Inf	last	wholeT_0.5	J1:DiP-A, J1:OT-A, J5:DiP-A, J5:OT-A, S5:OT-A
298	Yes	7	7-A	1000	Bozic	Inf	unif	singleC	S1:CBN, S1:CBN-A

Table 13: *(continued)*

	Conjunction	Drivers	Tree	S.Size	Model	sh	S.Time	S.Type	Best method(s)
299	Yes	7	7-A	1000	Bozic	Inf	unif	wholeT_0.01	J5:CBN, J5:CBN-A, J5:DiP-A, J5:OT, S5:CBN, S5:CBN-A, S5:DiP-A, S5:OT, S5:OT-A
300	Yes	7	7-A	1000	Bozic	Inf	unif	wholeT_0.5	S1:CBN, S1:CBN-A
301	Yes	7	7-A	1000	exp	0	last	singleC	S1:OT-A
302	Yes	7	7-A	1000	exp	0	last	wholeT_0.01	J1:OT-A, S1:OT-A, S5:OT-A
303	Yes	7	7-A	1000	exp	0	last	wholeT_0.5	S1:OT-A
304	Yes	7	7-A	1000	exp	0	unif	singleC	J1:DiP, J1:DiP-A, J5:DiP, J5:DiP-A, S1:DiP, S1:DiP-A, S5:DiP-A
305	Yes	7	7-A	1000	exp	0	unif	wholeT_0.01	J1:CBN, J1:CBN-A, J1:OT, J1:OT-A, S1:CBN-A
306	Yes	7	7-A	1000	exp	0	unif	wholeT_0.5	J1:DiP, J1:DiP-A, J5:DiP, J5:DiP-A, S1:DiP, S1:DiP-A, S5:DiP, S5:DiP-A
307	Yes	7	7-A	1000	exp	Inf	last	singleC	J1:OT-A, S1:OT-A, S5:OT-A
308	Yes	7	7-A	1000	exp	Inf	last	wholeT_0.01	J5:DiP-A, J5:OT-A, S5:DiP-A, S5:OT-A
309	Yes	7	7-A	1000	exp	Inf	last	wholeT_0.5	S1:OT-A, S5:OT-A
310	Yes	7	7-A	1000	exp	Inf	unif	singleC	S1:CBN, S1:CBN-A
311	Yes	7	7-A	1000	exp	Inf	unif	wholeT_0.01	J1:CBN, J1:CBN-A, J1:OT, J1:OT-A, J5:CBN, J5:CBN-A, J5:OT, J5:OT-A, S5:CBN, S5:CBN-A, S5:OT, S5:OT-A
312	Yes	7	7-A	1000	exp	Inf	unif	wholeT_0.5	S1:CBN, S1:CBN-A
313	Yes	7	7-A	1000	McF_4	0	last	singleC	J1:DiP-A, J1:OT-A, J5:DiP-A, J5:OT-A, S1:DiP-A, S1:OT-A, S5:DiP-A, S5:OT-A
314	Yes	7	7-A	1000	McF_4	0	last	wholeT_0.01	S5:DiP-A, S5:OT-A
315	Yes	7	7-A	1000	McF_4	0	last	wholeT_0.5	J1:DiP-A, J1:OT-A, J5:DiP-A, J5:OT-A, S1:DiP-A, S5:DiP-A, S5:OT-A
316	Yes	7	7-A	1000	McF_4	0	unif	singleC	J1:DiP-A, J1:OT, J1:OT-A, S1:DiP-A, S1:OT, S1:OT-A, S5:DiP-A, S5:OT, S5:OT-A
317	Yes	7	7-A	1000	McF_4	0	unif	wholeT_0.01	J1:DiP-A, J1:OT, J1:OT-A, S5:DiP-A, S5:OT, S5:OT-A
318	Yes	7	7-A	1000	McF_4	0	unif	wholeT_0.5	J1:DiP-A, J1:OT, J1:OT-A, S1:DiP-A, S1:OT, S1:OT-A, S5:DiP-A, S5:OT, S5:OT-A
319	Yes	7	7-A	1000	McF_4	Inf	last	singleC	J1:DiP-A, J1:OT-A, J5:DiP-A, J5:OT-A, S1:OT-A, S5:DiP-A, S5:OT-A
320	Yes	7	7-A	1000	McF_4	Inf	last	wholeT_0.01	J1:OT-A, J5:DiP-A, J5:OT-A, S5:DiP-A, S5:OT-A
321	Yes	7	7-A	1000	McF_4	Inf	last	wholeT_0.5	J1:DiP-A, J1:OT-A, J5:DiP-A, J5:OT-A, S1:DiP-A, S1:OT-A, S5:DiP-A, S5:OT-A

Table 13: *(continued)*

	Conjunction	Drivers	Tree	S.Size	Model	sh	S.Time	S.Type	Best method(s)
322	Yes	7	7-A	1000	McF_4	Inf	unif	singleC	J1:DiP-A, J1:OT, J1:OT-A, S1:DiP-A, S1:OT, S1:OT-A, S5:DiP-A, S5:OT, S5:OT-A
323	Yes	7	7-A	1000	McF_4	Inf	unif	wholeT_0.01	J1:DiP-A, J1:OT, J1:OT-A, J5:OT, S5:OT, S5:OT-A
324	Yes	7	7-A	1000	McF_4	Inf	unif	wholeT_0.5	J1:DiP-A, J1:OT, J1:OT-A, S1:DiP-A, S1:OT, S1:OT-A, S5:DiP-A, S5:OT, S5:OT-A
325	Yes	7	7-A	1000	McF_6	0	last	singleC	S5:OT-A
326	Yes	7	7-A	1000	McF_6	0	last	wholeT_0.01	J1:OT-A, S5:DiP-A, S5:OT-A
327	Yes	7	7-A	1000	McF_6	0	last	wholeT_0.5	S5:OT-A
328	Yes	7	7-A	1000	McF_6	0	unif	singleC	S1:CBN-A
329	Yes	7	7-A	1000	McF_6	0	unif	wholeT_0.01	J1:DiP, J1:DiP-A, S5:DiP, S5:DiP-A
330	Yes	7	7-A	1000	McF_6	0	unif	wholeT_0.5	S1:CBN-A
331	Yes	7	7-A	1000	McF_6	Inf	last	singleC	S5:DiP-A, S5:OT-A
332	Yes	7	7-A	1000	McF_6	Inf	last	wholeT_0.01	S5:DiP-A, S5:OT-A
333	Yes	7	7-A	1000	McF_6	Inf	last	wholeT_0.5	J1:DiP-A, J5:DiP-A, S5:DiP-A, S5:OT-A
334	Yes	7	7-A	1000	McF_6	Inf	unif	singleC	S1:CBN-A
335	Yes	7	7-A	1000	McF_6	Inf	unif	wholeT_0.01	J1:CBN-A, S5:CBN-A
336	Yes	7	7-A	1000	McF_6	Inf	unif	wholeT_0.5	S1:CBN-A
337	Yes	7	7-A	200	Bozic	0	last	singleC	S1:OT-A, S5:OT-A
338	Yes	7	7-A	200	Bozic	0	last	wholeT_0.01	J1:OT-A, J5:OT-A, S5:OT-A
339	Yes	7	7-A	200	Bozic	0	last	wholeT_0.5	S1:OT-A, S5:OT-A
340	Yes	7	7-A	200	Bozic	0	unif	singleC	J1:CBN-A, J1:OT, J1:OT-A, S1:CBN, S1:OT, S1:OT-A
341	Yes	7	7-A	200	Bozic	0	unif	wholeT_0.01	J5:CBN, J5:OT, J5:OT-A
342	Yes	7	7-A	200	Bozic	0	unif	wholeT_0.5	J1:CBN, J1:OT, J1:OT-A, S1:CBN-A, S1:OT, S1:OT-A
343	Yes	7	7-A	200	Bozic	Inf	last	singleC	J5:OT-A, S5:OT-A
344	Yes	7	7-A	200	Bozic	Inf	last	wholeT_0.01	J5:OT-A
345	Yes	7	7-A	200	Bozic	Inf	last	wholeT_0.5	J5:OT-A, S5:OT-A
346	Yes	7	7-A	200	Bozic	Inf	unif	singleC	J1:CBN, J1:CBN-A, S5:CBN, S5:CBN-A
347	Yes	7	7-A	200	Bozic	Inf	unif	wholeT_0.01	J5:CBN, J5:CBN-A, J5:OT, J5:OT-A, S5:CBN, S5:CBN-A, S5:OT, S5:OT-A
348	Yes	7	7-A	200	Bozic	Inf	unif	wholeT_0.5	J1:CBN, J1:CBN-A, S1:CBN
349	Yes	7	7-A	200	exp	0	last	singleC	S1:OT-A
350	Yes	7	7-A	200	exp	0	last	wholeT_0.01	J1:CBN-A, J1:OT-A, S1:OT-A, S5:OT-A
351	Yes	7	7-A	200	exp	0	last	wholeT_0.5	S1:OT-A

Table 13: *(continued)*

	Conjunction	Drivers	Tree	S.Size	Model	sh	S.Time	S.Type	Best method(s)
352	Yes	7	7-A	200	exp	0	unif	singleC	J1:CBN, J1:CBN-A, J1:DiP, J1:DiP-A, J1:OT, J1:OT-A, J5:DiP, J5:DiP-A, S1:CBN, S1:CBN-A, S1:DiP, S1:DiP-A, S1:OT, S1:OT-A, S5:DiP, S5:DiP-A
353	Yes	7	7-A	200	exp	0	unif	wholeT_0.01	J1:CBN, J1:CBN-A, J1:OT, J1:OT-A, S1:CBN, S1:CBN-A, S1:OT, S1:OT-A
354	Yes	7	7-A	200	exp	0	unif	wholeT_0.5	J1:DiP, J1:DiP-A, J5:DiP, J5:DiP-A, S1:DiP, S1:DiP-A, S5:DiP, S5:DiP-A
355	Yes	7	7-A	200	exp	Inf	last	singleC	S5:OT-A
356	Yes	7	7-A	200	exp	Inf	last	wholeT_0.01	J5:OT-A
357	Yes	7	7-A	200	exp	Inf	last	wholeT_0.5	S5:OT-A
358	Yes	7	7-A	200	exp	Inf	unif	singleC	J1:CBN, J1:CBN-A, J1:OT, J1:OT-A, S1:CBN, S1:CBN-A, S1:OT, S1:OT-A
359	Yes	7	7-A	200	exp	Inf	unif	wholeT_0.01	J5:CBN, J5:CBN-A, J5:OT, J5:OT-A, S5:CBN, S5:CBN-A, S5:OT, S5:OT-A
360	Yes	7	7-A	200	exp	Inf	unif	wholeT_0.5	J1:CBN, J1:CBN-A, J1:OT, J1:OT-A, S1:CBN, S1:CBN-A, S1:OT, S1:OT-A
361	Yes	7	7-A	200	McF_4	0	last	singleC	J1:OT-A, J5:OT-A, S5:OT-A
362	Yes	7	7-A	200	McF_4	0	last	wholeT_0.01	S5:OT-A
363	Yes	7	7-A	200	McF_4	0	last	wholeT_0.5	J1:OT-A, J5:OT-A, S5:OT-A
364	Yes	7	7-A	200	McF_4	0	unif	singleC	J1:OT, J1:OT-A, S5:OT, S5:OT-A
365	Yes	7	7-A	200	McF_4	0	unif	wholeT_0.01	J1:OT, J1:OT-A, S5:OT, S5:OT-A
366	Yes	7	7-A	200	McF_4	0	unif	wholeT_0.5	J1:OT, J1:OT-A, S5:OT, S5:OT-A
367	Yes	7	7-A	200	McF_4	Inf	last	singleC	J1:OT-A, J5:OT-A, S5:OT-A
368	Yes	7	7-A	200	McF_4	Inf	last	wholeT_0.01	J5:OT-A, S5:OT-A
369	Yes	7	7-A	200	McF_4	Inf	last	wholeT_0.5	J1:OT-A, J5:OT-A, S5:OT-A
370	Yes	7	7-A	200	McF_4	Inf	unif	singleC	J1:OT, J1:OT-A, S5:OT, S5:OT-A
371	Yes	7	7-A	200	McF_4	Inf	unif	wholeT_0.01	J1:OT, J1:OT-A, S5:OT, S5:OT-A
372	Yes	7	7-A	200	McF_4	Inf	unif	wholeT_0.5	J1:OT, J1:OT-A, S5:OT, S5:OT-A
373	Yes	7	7-A	200	McF_6	0	last	singleC	S5:OT-A
374	Yes	7	7-A	200	McF_6	0	last	wholeT_0.01	S5:OT-A
375	Yes	7	7-A	200	McF_6	0	last	wholeT_0.5	S5:OT-A
376	Yes	7	7-A	200	McF_6	0	unif	singleC	J1:CBN-A, J1:OT, J1:OT-A, S5:CBN-A, S5:OT, S5:OT-A
377	Yes	7	7-A	200	McF_6	0	unif	wholeT_0.01	J1:CBN-A, J1:OT, J1:OT-A, S5:CBN-A, S5:OT, S5:OT-A
378	Yes	7	7-A	200	McF_6	0	unif	wholeT_0.5	J1:CBN-A, S5:CBN-A
379	Yes	7	7-A	200	McF_6	Inf	last	singleC	S5:OT-A
380	Yes	7	7-A	200	McF_6	Inf	last	wholeT_0.01	S5:OT-A
381	Yes	7	7-A	200	McF_6	Inf	last	wholeT_0.5	S5:OT-A

Table 13: *(continued)*

	Conjunction	Drivers	Tree	S.Size	Model	sh	S.Time	S.Type	Best method(s)
382	Yes	7	7-A	200	McF_6	Inf	unif	singleC	J1:CBN-A, J1:OT, S5:CBN-A
383	Yes	7	7-A	200	McF_6	Inf	unif	wholeT_0.01	J1:CBN-A, S5:CBN-A, S5:OT-A
384	Yes	7	7-A	200	McF_6	Inf	unif	wholeT_0.5	J1:CBN-A, S5:CBN-A, S5:OT-A
385	Yes	7	7-A	100	Bozic	0	last	singleC	S5:OT-A
386	Yes	7	7-A	100	Bozic	0	last	wholeT_0.01	J5:OT-A, S5:OT-A
387	Yes	7	7-A	100	Bozic	0	last	wholeT_0.5	S5:OT-A
388	Yes	7	7-A	100	Bozic	0	unif	singleC	J1:CBN, J1:CBN-A, J1:OT, J1:OT-A, J5:DiP, J5:DiP-A, S1:OT, S1:OT-A, S5:DiP-A
389	Yes	7	7-A	100	Bozic	0	unif	wholeT_0.01	J5:CBN, J5:CBN-A, J5:OT, J5:OT-A, S5:CBN-A
390	Yes	7	7-A	100	Bozic	0	unif	wholeT_0.5	J1:CBN, J1:CBN-A, J1:OT, J1:OT-A, J5:DiP, J5:DiP-A, S1:OT, S1:OT-A, S5:DiP, S5:DiP-A
391	Yes	7	7-A	100	Bozic	Inf	last	singleC	J5:OT-A
392	Yes	7	7-A	100	Bozic	Inf	last	wholeT_0.01	J5:OT-A
393	Yes	7	7-A	100	Bozic	Inf	last	wholeT_0.5	J5:OT-A, S5:OT-A
394	Yes	7	7-A	100	Bozic	Inf	unif	singleC	J1:CBN, J1:CBN-A, S5:CBN, S5:CBN-A
395	Yes	7	7-A	100	Bozic	Inf	unif	wholeT_0.01	J5:CBN, J5:CBN-A, J5:OT, J5:OT-A, S5:CBN-A
396	Yes	7	7-A	100	Bozic	Inf	unif	wholeT_0.5	J1:CBN, J1:CBN-A, S5:CBN, S5:CBN-A
397	Yes	7	7-A	100	exp	0	last	singleC	S1:OT-A
398	Yes	7	7-A	100	exp	0	last	wholeT_0.01	J1:CBN-A, J1:OT-A, J5:CBN-A, J5:OT-A, S1:OT-A, S5:OT-A
399	Yes	7	7-A	100	exp	0	last	wholeT_0.5	S1:OT-A
400	Yes	7	7-A	100	exp	0	unif	singleC	J1:CBN, J1:CBN-A, J1:OT, J1:OT-A, J5:DiP, J5:DiP-A, S1:CBN, S1:CBN-A, S1:OT, S1:OT-A, S5:DiP, S5:DiP-A
401	Yes	7	7-A	100	exp	0	unif	wholeT_0.01	J1:CBN, J1:CBN-A, J1:OT, J1:OT-A, J5:CBN, J5:CBN-A, J5:DiP, J5:DiP-A, J5:OT, J5:OT-A, S1:OT, S1:OT-A, S5:CBN, S5:CBN-A, S5:DiP, S5:DiP-A, S5:OT, S5:OT-A
402	Yes	7	7-A	100	exp	0	unif	wholeT_0.5	J1:CBN, J1:CBN-A, J1:OT, J1:OT-A, J5:DiP, J5:DiP-A, S1:CBN, S1:CBN-A, S1:OT, S1:OT-A, S5:DiP, S5:DiP-A
403	Yes	7	7-A	100	exp	Inf	last	singleC	S5:OT-A
404	Yes	7	7-A	100	exp	Inf	last	wholeT_0.01	J5:OT-A
405	Yes	7	7-A	100	exp	Inf	last	wholeT_0.5	S5:OT-A

Table 13: *(continued)*

	Conjunction	Drivers	Tree	S.Size	Model	sh	S.Time	S.Type	Best method(s)
406	Yes	7	7-A	100	exp	Inf	unif	singleC	J1:CBN, J1:CBN-A, J1:OT, J1:OT-A, J5:CBN, J5:CBN-A, J5:OT, J5:OT-A, S1:OT, S1:OT-A, S5:CBN, S5:CBN-A, S5:OT, S5:OT-A
407	Yes	7	7-A	100	exp	Inf	unif	wholeT_0.01	J5:CBN, J5:CBN-A, J5:OT, J5:OT-A, S5:OT, S5:OT-A
408	Yes	7	7-A	100	exp	Inf	unif	wholeT_0.5	J1:CBN, J1:CBN-A, J1:OT, J1:OT-A, S1:CBN-A, S1:OT, S1:OT-A
409	Yes	7	7-A	100	McF_4	0	last	singleC	J5:OT-A, S5:OT-A
410	Yes	7	7-A	100	McF_4	0	last	wholeT_0.01	S5:OT-A
411	Yes	7	7-A	100	McF_4	0	last	wholeT_0.5	J1:OT-A, J5:OT-A, S5:OT-A
412	Yes	7	7-A	100	McF_4	0	unif	singleC	J1:OT, J1:OT-A, S5:OT, S5:OT-A
413	Yes	7	7-A	100	McF_4	0	unif	wholeT_0.01	J1:OT, J1:OT-A, S5:OT, S5:OT-A
414	Yes	7	7-A	100	McF_4	0	unif	wholeT_0.5	J1:OT, J1:OT-A, S5:OT, S5:OT-A
415	Yes	7	7-A	100	McF_4	Inf	last	singleC	J1:OT-A, J5:OT-A, S5:OT-A
416	Yes	7	7-A	100	McF_4	Inf	last	wholeT_0.01	J5:OT-A, S5:OT-A
417	Yes	7	7-A	100	McF_4	Inf	last	wholeT_0.5	J5:OT-A, S5:OT-A
418	Yes	7	7-A	100	McF_4	Inf	unif	singleC	J1:OT, J1:OT-A, S5:OT, S5:OT-A
419	Yes	7	7-A	100	McF_4	Inf	unif	wholeT_0.01	J1:OT-A, J5:OT-A, S5:OT, S5:OT-A
420	Yes	7	7-A	100	McF_4	Inf	unif	wholeT_0.5	J1:OT, J1:OT-A, J5:OT-A, S5:OT, S5:OT-A
421	Yes	7	7-A	100	McF_6	0	last	singleC	S5:OT-A
422	Yes	7	7-A	100	McF_6	0	last	wholeT_0.01	S5:OT-A
423	Yes	7	7-A	100	McF_6	0	last	wholeT_0.5	S5:OT-A
424	Yes	7	7-A	100	McF_6	0	unif	singleC	J1:CBN-A, S5:CBN-A, S5:OT-A
425	Yes	7	7-A	100	McF_6	0	unif	wholeT_0.01	J5:CBN-A, S5:CBN, S5:CBN-A, S5:OT, S5:OT-A
426	Yes	7	7-A	100	McF_6	0	unif	wholeT_0.5	J1:CBN-A, J5:CBN-A, S5:CBN-A
427	Yes	7	7-A	100	McF_6	Inf	last	singleC	S5:OT-A
428	Yes	7	7-A	100	McF_6	Inf	last	wholeT_0.01	S5:OT-A
429	Yes	7	7-A	100	McF_6	Inf	last	wholeT_0.5	S5:OT-A
430	Yes	7	7-A	100	McF_6	Inf	unif	singleC	S5:CBN-A
431	Yes	7	7-A	100	McF_6	Inf	unif	wholeT_0.01	J5:CBN-A, S5:CBN-A
432	Yes	7	7-A	100	McF_6	Inf	unif	wholeT_0.5	J1:CBN-A, J1:OT-A, J5:CBN-A, S5:CBN-A, S5:OT-A
433	No	11	11-B	1000	Bozic	0	last	singleC	S1:OT, S1:OT-A
434	No	11	11-B	1000	Bozic	0	last	wholeT_0.01	S1:DiP, S1:DiP-A, S5:DiP, S5:DiP-A
435	No	11	11-B	1000	Bozic	0	last	wholeT_0.5	S1:OT, S1:OT-A
436	No	11	11-B	1000	Bozic	0	unif	singleC	S1:DiP, S1:DiP-A, S1:OT, S1:OT-A, S5:DiP, S5:DiP-A, S5:OT, S5:OT-A

Table 13: *(continued)*

	Conjunction	Drivers	Tree	S.Size	Model	sh	S.Time	S.Type	Best method(s)
437	No	11	11-B	1000	Bozic	0	unif	wholeT_0.01	J1:CBN-A, S1:OT-A, S5:DiP-A, S5:OT, S5:OT-A
438	No	11	11-B	1000	Bozic	0	unif	wholeT_0.5	S1:DiP, S1:DiP-A, S1:OT, S1:OT-A, S5:DiP, S5:DiP-A, S5:OT, S5:OT-A
439	No	11	11-B	1000	Bozic	Inf	last	singleC	S5:DiP, S5:DiP-A, S5:OT, S5:OT-A
440	No	11	11-B	1000	Bozic	Inf	last	wholeT_0.01	J1:DiP, J1:DiP-A, J1:OT, J1:OT-A, J5:DiP, J5:DiP-A, J5:OT, J5:OT-A
441	No	11	11-B	1000	Bozic	Inf	last	wholeT_0.5	S5:DiP, S5:DiP-A, S5:OT, S5:OT-A
442	No	11	11-B	1000	Bozic	Inf	unif	singleC	S1:CBN, S1:CBN-A, S1:DiP-A, S1:OT, S1:OT-A
443	No	11	11-B	1000	Bozic	Inf	unif	wholeT_0.01	J1:CBN-A, J1:DiP, J1:DiP-A, J1:OT, J1:OT-A, J5:DiP, J5:DiP-A, J5:OT, J5:OT-A, S5:CBN, S5:CBN-A, S5:DiP, S5:DiP-A, S5:OT, S5:OT-A
444	No	11	11-B	1000	Bozic	Inf	unif	wholeT_0.5	S1:CBN, S1:CBN-A, S1:OT, S1:OT-A
445	No	11	11-B	1000	exp	0	last	singleC	S1:OT, S1:OT-A
446	No	11	11-B	1000	exp	0	last	wholeT_0.01	S1:DiP-A, S1:OT, S1:OT-A, S5:DiP, S5:DiP-A, S5:OT, S5:OT-A
447	No	11	11-B	1000	exp	0	last	wholeT_0.5	S1:OT, S1:OT-A
448	No	11	11-B	1000	exp	0	unif	singleC	S1:DiP, S1:DiP-A, S1:OT, S1:OT-A, S5:DiP, S5:DiP-A, S5:OT, S5:OT-A
449	No	11	11-B	1000	exp	0	unif	wholeT_0.01	S1:DiP, S1:DiP-A, S1:OT, S1:OT-A
450	No	11	11-B	1000	exp	0	unif	wholeT_0.5	S1:DiP, S1:DiP-A, S1:OT, S1:OT-A, S5:DiP, S5:DiP-A, S5:OT, S5:OT-A
451	No	11	11-B	1000	exp	Inf	last	singleC	S5:DiP, S5:DiP-A, S5:OT, S5:OT-A
452	No	11	11-B	1000	exp	Inf	last	wholeT_0.01	S5:DiP, S5:DiP-A, S5:OT, S5:OT-A
453	No	11	11-B	1000	exp	Inf	last	wholeT_0.5	S5:DiP, S5:DiP-A, S5:OT, S5:OT-A
454	No	11	11-B	1000	exp	Inf	unif	singleC	S1:CBN, S1:CBN-A, S1:OT
455	No	11	11-B	1000	exp	Inf	unif	wholeT_0.01	J5:OT, S5:DiP, S5:DiP-A, S5:OT, S5:OT-A
456	No	11	11-B	1000	exp	Inf	unif	wholeT_0.5	S1:CBN, S1:CBN-A, S1:OT, S1:OT-A
457	No	11	11-B	1000	McF_4	0	last	singleC	S1:DiP, S1:DiP-A, S1:OT, S1:OT-A
458	No	11	11-B	1000	McF_4	0	last	wholeT_0.01	S1:DiP, S1:DiP-A, S1:OT, S1:OT-A, S5:DiP, S5:DiP-A, S5:OT, S5:OT-A
459	No	11	11-B	1000	McF_4	0	last	wholeT_0.5	S1:DiP-A, S1:OT, S1:OT-A
460	No	11	11-B	1000	McF_4	0	unif	singleC	S1:DiP, S1:DiP-A, S1:OT, S1:OT-A
461	No	11	11-B	1000	McF_4	0	unif	wholeT_0.01	S1:OT, S1:OT-A
462	No	11	11-B	1000	McF_4	0	unif	wholeT_0.5	S1:DiP, S1:DiP-A, S1:OT, S1:OT-A
463	No	11	11-B	1000	McF_4	Inf	last	singleC	S1:OT, S1:OT-A
464	No	11	11-B	1000	McF_4	Inf	last	wholeT_0.01	S5:DiP, S5:DiP-A, S5:OT, S5:OT-A

Table 13: *(continued)*

	Conjunction	Drivers	Tree	S.Size	Model	sh	S.Time	S.Type	Best method(s)
465	No	11	11-B	1000	McF_4	Inf	last	wholeT_0.5	S1:DiP-A, S1:OT, S1:OT-A
466	No	11	11-B	1000	McF_4	Inf	unif	singleC	S1:OT, S1:OT-A
467	No	11	11-B	1000	McF_4	Inf	unif	wholeT_0.01	S5:DiP, S5:DiP-A, S5:OT, S5:OT-A
468	No	11	11-B	1000	McF_4	Inf	unif	wholeT_0.5	S1:OT, S1:OT-A
469	No	11	11-B	1000	McF_6	0	last	singleC	S1:OT, S5:DiP, S5:OT
470	No	11	11-B	1000	McF_6	0	last	wholeT_0.01	S5:DiP, S5:OT
471	No	11	11-B	1000	McF_6	0	last	wholeT_0.5	S1:OT, S5:DiP, S5:OT
472	No	11	11-B	1000	McF_6	0	unif	singleC	S1:DiP, S1:DiP-A, S1:OT, S1:OT-A
473	No	11	11-B	1000	McF_6	0	unif	wholeT_0.01	S1:DiP, S1:DiP-A, S1:OT, S1:OT-A
474	No	11	11-B	1000	McF_6	0	unif	wholeT_0.5	S1:DiP, S1:DiP-A, S1:OT-A
475	No	11	11-B	1000	McF_6	Inf	last	singleC	S1:OT, S5:DiP, S5:OT
476	No	11	11-B	1000	McF_6	Inf	last	wholeT_0.01	S5:OT
477	No	11	11-B	1000	McF_6	Inf	last	wholeT_0.5	S1:DiP, S1:OT, S5:DiP, S5:OT
478	No	11	11-B	1000	McF_6	Inf	unif	singleC	S1:DiP, S1:DiP-A, S1:OT
479	No	11	11-B	1000	McF_6	Inf	unif	wholeT_0.01	S1:DiP, S1:DiP-A
480	No	11	11-B	1000	McF_6	Inf	unif	wholeT_0.5	S1:DiP, S1:DiP-A
481	No	11	11-B	200	Bozic	0	last	singleC	S1:OT, S1:OT-A
482	No	11	11-B	200	Bozic	0	last	wholeT_0.01	J1:OT, J1:OT-A, J5:OT, J5:OT-A, S5:OT, S5:OT-A
483	No	11	11-B	200	Bozic	0	last	wholeT_0.5	S1:OT, S1:OT-A
484	No	11	11-B	200	Bozic	0	unif	singleC	S1:OT, S1:OT-A
485	No	11	11-B	200	Bozic	0	unif	wholeT_0.01	J1:CBN-A, S5:DiP-A, S5:OT, S5:OT-A
486	No	11	11-B	200	Bozic	0	unif	wholeT_0.5	S1:OT, S1:OT-A, S5:DiP, S5:DiP-A, S5:OT, S5:OT-A
487	No	11	11-B	200	Bozic	Inf	last	singleC	S5:OT, S5:OT-A
488	No	11	11-B	200	Bozic	Inf	last	wholeT_0.01	J5:OT, J5:OT-A
489	No	11	11-B	200	Bozic	Inf	last	wholeT_0.5	S5:OT, S5:OT-A
490	No	11	11-B	200	Bozic	Inf	unif	singleC	J1:CBN, J1:CBN-A, J1:OT, J1:OT-A, S5:DiP, S5:DiP-A, S5:OT, S5:OT-A
491	No	11	11-B	200	Bozic	Inf	unif	wholeT_0.01	J5:CBN-A, J5:OT, J5:OT-A, S5:CBN, S5:CBN-A, S5:OT, S5:OT-A
492	No	11	11-B	200	Bozic	Inf	unif	wholeT_0.5	J1:CBN, J1:CBN-A, J1:OT, J1:OT-A, S5:OT, S5:OT-A
493	No	11	11-B	200	exp	0	last	singleC	S1:OT, S1:OT-A
494	No	11	11-B	200	exp	0	last	wholeT_0.01	S1:OT, S1:OT-A, S5:OT, S5:OT-A
495	No	11	11-B	200	exp	0	last	wholeT_0.5	S1:OT, S1:OT-A
496	No	11	11-B	200	exp	0	unif	singleC	S1:DiP, S1:DiP-A, S1:OT, S1:OT-A, S5:DiP, S5:DiP-A, S5:OT, S5:OT-A
497	No	11	11-B	200	exp	0	unif	wholeT_0.01	S1:OT, S1:OT-A

Table 13: *(continued)*

	Conjunction	Drivers	Tree	S.Size	Model	sh	S.Time	S.Type	Best method(s)
498	No	11	11-B	200	exp	0	unif	wholeT_0.5	S1:DiP, S1:DiP-A, S1:OT, S1:OT-A, S5:DiP-A, S5:OT, S5:OT-A
499	No	11	11-B	200	exp	Inf	last	singleC	S5:OT
500	No	11	11-B	200	exp	Inf	last	wholeT_0.01	J5:OT, J5:OT-A
501	No	11	11-B	200	exp	Inf	last	wholeT_0.5	S5:OT, S5:OT-A
502	No	11	11-B	200	exp	Inf	unif	singleC	S1:OT, S1:OT-A
503	No	11	11-B	200	exp	Inf	unif	wholeT_0.01	S5:OT, S5:OT-A
504	No	11	11-B	200	exp	Inf	unif	wholeT_0.5	S1:OT, S1:OT-A
505	No	11	11-B	200	McF_4	0	last	singleC	S1:OT, S1:OT-A, S5:OT, S5:OT-A
506	No	11	11-B	200	McF_4	0	last	wholeT_0.01	S5:OT, S5:OT-A
507	No	11	11-B	200	McF_4	0	last	wholeT_0.5	S1:OT, S1:OT-A, S5:OT, S5:OT-A
508	No	11	11-B	200	McF_4	0	unif	singleC	S1:OT, S1:OT-A, S5:CBN-A, S5:OT, S5:OT-A
509	No	11	11-B	200	McF_4	0	unif	wholeT_0.01	S5:OT, S5:OT-A
510	No	11	11-B	200	McF_4	0	unif	wholeT_0.5	S1:OT, S1:OT-A, S5:OT, S5:OT-A
511	No	11	11-B	200	McF_4	Inf	last	singleC	S1:OT, S1:OT-A, S5:OT, S5:OT-A
512	No	11	11-B	200	McF_4	Inf	last	wholeT_0.01	S5:OT, S5:OT-A
513	No	11	11-B	200	McF_4	Inf	last	wholeT_0.5	S1:OT, S1:OT-A
514	No	11	11-B	200	McF_4	Inf	unif	singleC	S1:OT, S1:OT-A, S5:CBN-A, S5:OT, S5:OT-A
515	No	11	11-B	200	McF_4	Inf	unif	wholeT_0.01	S5:CBN-A, S5:OT, S5:OT-A
516	No	11	11-B	200	McF_4	Inf	unif	wholeT_0.5	S1:OT, S1:OT-A, S5:OT, S5:OT-A
517	No	11	11-B	200	McF_6	0	last	singleC	S5:OT
518	No	11	11-B	200	McF_6	0	last	wholeT_0.01	S5:OT
519	No	11	11-B	200	McF_6	0	last	wholeT_0.5	S5:OT
520	No	11	11-B	200	McF_6	0	unif	singleC	J1:CBN-A, S1:OT, S1:OT-A, S5:CBN-A, S5:OT, S5:OT-A
521	No	11	11-B	200	McF_6	0	unif	wholeT_0.01	S1:OT, S1:OT-A, S5:OT, S5:OT-A
522	No	11	11-B	200	McF_6	0	unif	wholeT_0.5	S1:OT, S1:OT-A, S5:CBN, S5:OT, S5:OT-A
523	No	11	11-B	200	McF_6	Inf	last	singleC	S5:OT
524	No	11	11-B	200	McF_6	Inf	last	wholeT_0.01	S5:OT
525	No	11	11-B	200	McF_6	Inf	last	wholeT_0.5	S5:OT
526	No	11	11-B	200	McF_6	Inf	unif	singleC	J1:CBN-A, S1:OT, S1:OT-A, S5:CBN, S5:CBN-A, S5:OT, S5:OT-A
527	No	11	11-B	200	McF_6	Inf	unif	wholeT_0.01	S1:OT, S5:CBN, S5:CBN-A, S5:OT, S5:OT-A
528	No	11	11-B	200	McF_6	Inf	unif	wholeT_0.5	J1:CBN-A, S1:OT, S1:OT-A, S5:CBN, S5:CBN-A, S5:OT, S5:OT-A
529	No	11	11-B	100	Bozic	0	last	singleC	S5:OT, S5:OT-A

Table 13: *(continued)*

	Conjunction	Drivers	Tree	S.Size	Model	sh	S.Time	S.Type	Best method(s)
530	No	11	11-B	100	Bozic	0	last	wholeT_0.01	J1:OT, J1:OT-A, J5:CBN-A, J5:OT, J5:OT-A, S5:OT, S5:OT-A
531	No	11	11-B	100	Bozic	0	last	wholeT_0.5	S1:OT, S1:OT-A, S5:DiP, S5:OT, S5:OT-A
532	No	11	11-B	100	Bozic	0	unif	singleC	S5:DiP, S5:DiP-A, S5:OT, S5:OT-A
533	No	11	11-B	100	Bozic	0	unif	wholeT_0.01	S5:CBN, S5:OT, S5:OT-A
534	No	11	11-B	100	Bozic	0	unif	wholeT_0.5	J1:OT, S5:DiP, S5:DiP-A, S5:OT, S5:OT-A
535	No	11	11-B	100	Bozic	Inf	last	singleC	S5:OT, S5:OT-A
536	No	11	11-B	100	Bozic	Inf	last	wholeT_0.01	J5:OT, J5:OT-A
537	No	11	11-B	100	Bozic	Inf	last	wholeT_0.5	J5:OT, J5:OT-A, S5:OT, S5:OT-A
538	No	11	11-B	100	Bozic	Inf	unif	singleC	J1:OT, J1:OT-A, J5:DiP, J5:OT-A, S5:CBN-A, S5:OT, S5:OT-A
539	No	11	11-B	100	Bozic	Inf	unif	wholeT_0.01	J5:CBN-A, J5:OT, J5:OT-A, S5:CBN-A, S5:OT, S5:OT-A
540	No	11	11-B	100	Bozic	Inf	unif	wholeT_0.5	S5:OT, S5:OT-A
541	No	11	11-B	100	exp	0	last	singleC	S1:OT, S1:OT-A
542	No	11	11-B	100	exp	0	last	wholeT_0.01	S1:OT, S1:OT-A, S5:OT, S5:OT-A
543	No	11	11-B	100	exp	0	last	wholeT_0.5	S1:OT, S1:OT-A
544	No	11	11-B	100	exp	0	unif	singleC	S1:CBN-A, S1:OT, S1:OT-A, S5:DiP, S5:DiP-A, S5:OT, S5:OT-A
545	No	11	11-B	100	exp	0	unif	wholeT_0.01	J1:OT, J1:OT-A, S1:OT, S1:OT-A, S5:DiP, S5:DiP-A, S5:OT, S5:OT-A
546	No	11	11-B	100	exp	0	unif	wholeT_0.5	S1:OT, S1:OT-A, S5:DiP, S5:DiP-A, S5:OT, S5:OT-A
547	No	11	11-B	100	exp	Inf	last	singleC	S5:OT, S5:OT-A
548	No	11	11-B	100	exp	Inf	last	wholeT_0.01	J5:CBN-A, J5:OT, J5:OT-A
549	No	11	11-B	100	exp	Inf	last	wholeT_0.5	S5:OT, S5:OT-A
550	No	11	11-B	100	exp	Inf	unif	singleC	J1:OT, J1:OT-A, S5:DiP, S5:DiP-A, S5:OT, S5:OT-A
551	No	11	11-B	100	exp	Inf	unif	wholeT_0.01	S5:OT, S5:OT-A
552	No	11	11-B	100	exp	Inf	unif	wholeT_0.5	J1:OT, J1:OT-A, S5:DiP, S5:DiP-A, S5:OT, S5:OT-A
553	No	11	11-B	100	McF_4	0	last	singleC	S1:OT, S1:OT-A, S5:OT, S5:OT-A
554	No	11	11-B	100	McF_4	0	last	wholeT_0.01	S5:OT, S5:OT-A
555	No	11	11-B	100	McF_4	0	last	wholeT_0.5	S1:OT, S1:OT-A, S5:OT, S5:OT-A
556	No	11	11-B	100	McF_4	0	unif	singleC	S5:CBN-A, S5:OT, S5:OT-A
557	No	11	11-B	100	McF_4	0	unif	wholeT_0.01	S5:CBN, S5:CBN-A, S5:OT, S5:OT-A
558	No	11	11-B	100	McF_4	0	unif	wholeT_0.5	S5:OT, S5:OT-A
559	No	11	11-B	100	McF_4	Inf	last	singleC	S1:OT, S1:OT-A, S5:OT, S5:OT-A

Table 13: *(continued)*

	Conjunction	Drivers	Tree	S.Size	Model	sh	S.Time	S.Type	Best method(s)
560	No	11	11-B	100	McF_4	Inf	last	wholeT_0.01	S5:OT, S5:OT-A
561	No	11	11-B	100	McF_4	Inf	last	wholeT_0.5	S1:OT, S1:OT-A, S5:OT, S5:OT-A
562	No	11	11-B	100	McF_4	Inf	unif	singleC	S1:OT, S1:OT-A, S5:CBN-A, S5:OT, S5:OT-A
563	No	11	11-B	100	McF_4	Inf	unif	wholeT_0.01	S5:CBN-A, S5:OT, S5:OT-A
564	No	11	11-B	100	McF_4	Inf	unif	wholeT_0.5	S1:OT, S1:OT-A, S5:OT, S5:OT-A
565	No	11	11-B	100	McF_6	0	last	singleC	S5:OT
566	No	11	11-B	100	McF_6	0	last	wholeT_0.01	S5:OT
567	No	11	11-B	100	McF_6	0	last	wholeT_0.5	S5:OT
568	No	11	11-B	100	McF_6	0	unif	singleC	S1:OT, S1:OT-A, S5:CBN, S5:CBN-A, S5:OT, S5:OT-A
569	No	11	11-B	100	McF_6	0	unif	wholeT_0.01	S5:CBN-A, S5:OT, S5:OT-A
570	No	11	11-B	100	McF_6	0	unif	wholeT_0.5	J1:CBN, J1:CBN-A, S1:OT, S1:OT-A, S5:CBN, S5:CBN-A, S5:OT, S5:OT-A
571	No	11	11-B	100	McF_6	Inf	last	singleC	S5:OT
572	No	11	11-B	100	McF_6	Inf	last	wholeT_0.01	S5:OT, S5:OT-A
573	No	11	11-B	100	McF_6	Inf	last	wholeT_0.5	S5:OT
574	No	11	11-B	100	McF_6	Inf	unif	singleC	S5:CBN, S5:CBN-A, S5:OT, S5:OT-A
575	No	11	11-B	100	McF_6	Inf	unif	wholeT_0.01	S5:CBN, S5:CBN-A, S5:OT, S5:OT-A
576	No	11	11-B	100	McF_6	Inf	unif	wholeT_0.5	S1:OT, S1:OT-A, S5:CBN-A, S5:OT, S5:OT-A
577	No	9	9-B	1000	Bozic	0	last	singleC	S1:OT, S1:OT-A
578	No	9	9-B	1000	Bozic	0	last	wholeT_0.01	S1:CBN-A, S1:DiP, S1:DiP-A, S1:OT, S1:OT-A, S5:CBN-A, S5:DiP, S5:DiP-A, S5:OT, S5:OT-A
579	No	9	9-B	1000	Bozic	0	last	wholeT_0.5	S1:OT, S1:OT-A
580	No	9	9-B	1000	Bozic	0	unif	singleC	S1:DiP, S1:DiP-A, S1:OT, S1:OT-A, S5:DiP, S5:DiP-A, S5:OT, S5:OT-A
581	No	9	9-B	1000	Bozic	0	unif	wholeT_0.01	J1:DiP, J1:DiP-A, J1:OT, J1:OT-A, S1:DiP, S1:DiP-A, S1:OT, S1:OT-A
582	No	9	9-B	1000	Bozic	0	unif	wholeT_0.5	S1:DiP, S1:DiP-A, S1:OT, S1:OT-A, S5:DiP, S5:DiP-A, S5:OT, S5:OT-A
583	No	9	9-B	1000	Bozic	Inf	last	singleC	S5:DiP, S5:DiP-A, S5:OT, S5:OT-A
584	No	9	9-B	1000	Bozic	Inf	last	wholeT_0.01	J5:DiP, J5:DiP-A
585	No	9	9-B	1000	Bozic	Inf	last	wholeT_0.5	S5:DiP, S5:DiP-A, S5:OT, S5:OT-A
586	No	9	9-B	1000	Bozic	Inf	unif	singleC	S1:CBN, S1:CBN-A, S1:OT, S1:OT-A
587	No	9	9-B	1000	Bozic	Inf	unif	wholeT_0.01	J1:CBN-A, S5:CBN, S5:CBN-A, S5:DiP, S5:DiP-A, S5:OT, S5:OT-A
588	No	9	9-B	1000	Bozic	Inf	unif	wholeT_0.5	S1:CBN, S1:CBN-A, S1:OT, S1:OT-A
589	No	9	9-B	1000	exp	0	last	singleC	S1:OT, S1:OT-A

Table 13: *(continued)*

	Conjunction	Drivers	Tree	S.Size	Model	sh	S.Time	S.Type	Best method(s)
590	No	9	9-B	1000	exp	0	last	wholeT_0.01	S1:DiP-A, S1:OT, S1:OT-A
591	No	9	9-B	1000	exp	0	last	wholeT_0.5	S1:DiP, S1:DiP-A, S1:OT, S1:OT-A, S5:DiP, S5:DiP-A, S5:OT, S5:OT-A
592	No	9	9-B	1000	exp	0	unif	singleC	S1:DiP, S1:DiP-A, S1:OT, S1:OT-A, S5:DiP, S5:DiP-A, S5:OT, S5:OT-A
593	No	9	9-B	1000	exp	0	unif	wholeT_0.01	S1:DiP-A, S1:OT, S1:OT-A, S5:DiP-A, S5:OT, S5:OT-A
594	No	9	9-B	1000	exp	0	unif	wholeT_0.5	S1:DiP, S1:DiP-A, S1:OT, S1:OT-A, S5:DiP, S5:DiP-A, S5:OT, S5:OT-A
595	No	9	9-B	1000	exp	Inf	last	singleC	S5:DiP, S5:DiP-A, S5:OT, S5:OT-A
596	No	9	9-B	1000	exp	Inf	last	wholeT_0.01	J1:DiP, J1:DiP-A, J5:DiP, J5:DiP-A, S5:DiP, S5:DiP-A
597	No	9	9-B	1000	exp	Inf	last	wholeT_0.5	S5:DiP, S5:DiP-A, S5:OT, S5:OT-A
598	No	9	9-B	1000	exp	Inf	unif	singleC	S1:OT, S1:OT-A
599	No	9	9-B	1000	exp	Inf	unif	wholeT_0.01	J1:OT, J1:OT-A, J5:DiP, J5:DiP-A, J5:OT, J5:OT-A, S5:DiP, S5:DiP-A, S5:OT, S5:OT-A
600	No	9	9-B	1000	exp	Inf	unif	wholeT_0.5	S1:OT, S1:OT-A
601	No	9	9-B	1000	McF_4	0	last	singleC	S1:DiP-A, S1:OT, S1:OT-A
602	No	9	9-B	1000	McF_4	0	last	wholeT_0.01	S5:DiP, S5:DiP-A, S5:OT, S5:OT-A
603	No	9	9-B	1000	McF_4	0	last	wholeT_0.5	S1:OT, S1:OT-A
604	No	9	9-B	1000	McF_4	0	unif	singleC	S1:DiP, S1:DiP-A, S1:OT, S1:OT-A
605	No	9	9-B	1000	McF_4	0	unif	wholeT_0.01	S1:OT, S1:OT-A, S5:DiP, S5:DiP-A, S5:OT, S5:OT-A
606	No	9	9-B	1000	McF_4	0	unif	wholeT_0.5	S1:DiP, S1:DiP-A, S1:OT, S1:OT-A
607	No	9	9-B	1000	McF_4	Inf	last	singleC	S1:OT, S1:OT-A
608	No	9	9-B	1000	McF_4	Inf	last	wholeT_0.01	S5:DiP, S5:DiP-A, S5:OT, S5:OT-A
609	No	9	9-B	1000	McF_4	Inf	last	wholeT_0.5	S1:OT, S1:OT-A
610	No	9	9-B	1000	McF_4	Inf	unif	singleC	S1:DiP, S1:DiP-A, S1:OT, S1:OT-A
611	No	9	9-B	1000	McF_4	Inf	unif	wholeT_0.01	S5:DiP, S5:DiP-A, S5:OT, S5:OT-A
612	No	9	9-B	1000	McF_4	Inf	unif	wholeT_0.5	S1:DiP, S1:DiP-A, S1:OT, S1:OT-A
613	No	9	9-B	1000	McF_6	0	last	singleC	S5:OT
614	No	9	9-B	1000	McF_6	0	last	wholeT_0.01	J1:OT, S5:OT
615	No	9	9-B	1000	McF_6	0	last	wholeT_0.5	S1:OT, S5:OT
616	No	9	9-B	1000	McF_6	0	unif	singleC	S1:DiP, S1:DiP-A, S1:OT, S1:OT-A
617	No	9	9-B	1000	McF_6	0	unif	wholeT_0.01	S5:CBN, S5:DiP, S5:DiP-A, S5:OT, S5:OT-A
618	No	9	9-B	1000	McF_6	0	unif	wholeT_0.5	S1:DiP, S1:DiP-A, S1:OT, S1:OT-A
619	No	9	9-B	1000	McF_6	Inf	last	singleC	S5:DiP, S5:DiP-A, S5:OT, S5:OT-A
620	No	9	9-B	1000	McF_6	Inf	last	wholeT_0.01	S5:DiP, S5:DiP-A, S5:OT-A

Table 13: *(continued)*

	Conjunction	Drivers	Tree	S.Size	Model	sh	S.Time	S.Type	Best method(s)
621	No	9	9-B	1000	McF_6	Inf	last	wholeT_0.5	S1:DiP, S1:DiP-A, S1:OT-A, S5:DiP, S5:DiP-A, S5:OT-A
622	No	9	9-B	1000	McF_6	Inf	unif	singleC	S1:DiP, S1:DiP-A, S1:OT, S1:OT-A
623	No	9	9-B	1000	McF_6	Inf	unif	wholeT_0.01	S5:DiP, S5:DiP-A, S5:OT, S5:OT-A
624	No	9	9-B	1000	McF_6	Inf	unif	wholeT_0.5	S1:DiP, S1:DiP-A, S1:OT, S1:OT-A
625	No	9	9-B	200	Bozic	0	last	singleC	S1:OT, S1:OT-A
626	No	9	9-B	200	Bozic	0	last	wholeT_0.01	S5:OT, S5:OT-A
627	No	9	9-B	200	Bozic	0	last	wholeT_0.5	S1:OT, S1:OT-A
628	No	9	9-B	200	Bozic	0	unif	singleC	S1:OT, S1:OT-A, S5:DiP, S5:DiP-A, S5:OT, S5:OT-A
629	No	9	9-B	200	Bozic	0	unif	wholeT_0.01	J1:DiP-A, J1:OT, J1:OT-A, S5:DiP, S5:DiP-A, S5:OT, S5:OT-A
630	No	9	9-B	200	Bozic	0	unif	wholeT_0.5	S1:OT, S1:OT-A, S5:DiP, S5:DiP-A, S5:OT, S5:OT-A
631	No	9	9-B	200	Bozic	Inf	last	singleC	S5:OT, S5:OT-A
632	No	9	9-B	200	Bozic	Inf	last	wholeT_0.01	J5:CBN-A, J5:OT, J5:OT-A
633	No	9	9-B	200	Bozic	Inf	last	wholeT_0.5	S5:OT, S5:OT-A
634	No	9	9-B	200	Bozic	Inf	unif	singleC	J1:DiP-A, J1:OT, J1:OT-A, S5:DiP, S5:DiP-A, S5:OT, S5:OT-A
635	No	9	9-B	200	Bozic	Inf	unif	wholeT_0.01	J5:OT, J5:OT-A, S5:CBN-A, S5:OT, S5:OT-A
636	No	9	9-B	200	Bozic	Inf	unif	wholeT_0.5	J1:DiP, J1:DiP-A, J1:OT, J1:OT-A, S5:DiP, S5:DiP-A, S5:OT, S5:OT-A
637	No	9	9-B	200	exp	0	last	singleC	S1:OT, S1:OT-A
638	No	9	9-B	200	exp	0	last	wholeT_0.01	S1:OT, S1:OT-A
639	No	9	9-B	200	exp	0	last	wholeT_0.5	S1:OT, S1:OT-A
640	No	9	9-B	200	exp	0	unif	singleC	S1:DiP, S1:DiP-A, S1:OT, S1:OT-A, S5:DiP, S5:DiP-A, S5:OT, S5:OT-A
641	No	9	9-B	200	exp	0	unif	wholeT_0.01	S1:OT, S1:OT-A
642	No	9	9-B	200	exp	0	unif	wholeT_0.5	S1:DiP, S1:DiP-A, S1:OT, S1:OT-A, S5:DiP, S5:DiP-A, S5:OT, S5:OT-A
643	No	9	9-B	200	exp	Inf	last	singleC	S5:OT, S5:OT-A
644	No	9	9-B	200	exp	Inf	last	wholeT_0.01	J5:CBN-A, J5:OT, J5:OT-A
645	No	9	9-B	200	exp	Inf	last	wholeT_0.5	S5:OT, S5:OT-A
646	No	9	9-B	200	exp	Inf	unif	singleC	J1:DiP, J1:DiP-A, J1:OT, J1:OT-A, S1:OT, S1:OT-A, S5:DiP, S5:DiP-A, S5:OT, S5:OT-A
647	No	9	9-B	200	exp	Inf	unif	wholeT_0.01	S5:OT, S5:OT-A
648	No	9	9-B	200	exp	Inf	unif	wholeT_0.5	S1:OT, S1:OT-A
649	No	9	9-B	200	McF_4	0	last	singleC	S1:OT, S1:OT-A, S5:OT, S5:OT-A

Table 13: *(continued)*

	Conjunction	Drivers	Tree	S.Size	Model	sh	S.Time	S.Type	Best method(s)
650	No	9	9-B	200	McF_4	0	last	wholeT_0.01	S5:OT, S5:OT-A
651	No	9	9-B	200	McF_4	0	last	wholeT_0.5	S1:OT, S1:OT-A, S5:OT, S5:OT-A
652	No	9	9-B	200	McF_4	0	unif	singleC	S1:OT, S5:OT, S5:OT-A
653	No	9	9-B	200	McF_4	0	unif	wholeT_0.01	S5:OT, S5:OT-A
654	No	9	9-B	200	McF_4	0	unif	wholeT_0.5	S1:OT, S1:OT-A, S5:OT, S5:OT-A
655	No	9	9-B	200	McF_4	Inf	last	singleC	S1:OT, S5:OT, S5:OT-A
656	No	9	9-B	200	McF_4	Inf	last	wholeT_0.01	S5:OT, S5:OT-A
657	No	9	9-B	200	McF_4	Inf	last	wholeT_0.5	S1:OT, S1:OT-A, S5:OT, S5:OT-A
658	No	9	9-B	200	McF_4	Inf	unif	singleC	S1:OT, S1:OT-A, S5:OT, S5:OT-A
659	No	9	9-B	200	McF_4	Inf	unif	wholeT_0.01	S5:OT, S5:OT-A
660	No	9	9-B	200	McF_4	Inf	unif	wholeT_0.5	S1:OT, S1:OT-A, S5:OT, S5:OT-A
661	No	9	9-B	200	McF_6	0	last	singleC	S5:OT, S5:OT-A
662	No	9	9-B	200	McF_6	0	last	wholeT_0.01	J1:OT, S5:OT, S5:OT-A
663	No	9	9-B	200	McF_6	0	last	wholeT_0.5	S5:OT, S5:OT-A
664	No	9	9-B	200	McF_6	0	unif	singleC	S5:CBN, S5:OT, S5:OT-A
665	No	9	9-B	200	McF_6	0	unif	wholeT_0.01	S5:CBN-A, S5:OT, S5:OT-A
666	No	9	9-B	200	McF_6	0	unif	wholeT_0.5	S1:OT-A, S5:CBN-A, S5:OT, S5:OT-A
667	No	9	9-B	200	McF_6	Inf	last	singleC	S5:OT-A
668	No	9	9-B	200	McF_6	Inf	last	wholeT_0.01	S5:OT-A
669	No	9	9-B	200	McF_6	Inf	last	wholeT_0.5	S5:OT-A
670	No	9	9-B	200	McF_6	Inf	unif	singleC	S5:CBN, S5:OT, S5:OT-A
671	No	9	9-B	200	McF_6	Inf	unif	wholeT_0.01	S5:CBN-A, S5:OT, S5:OT-A
672	No	9	9-B	200	McF_6	Inf	unif	wholeT_0.5	S1:OT, S1:OT-A, S5:CBN, S5:CBN-A, S5:OT, S5:OT-A
673	No	9	9-B	100	Bozic	0	last	singleC	S1:OT, S1:OT-A, S5:OT, S5:OT-A
674	No	9	9-B	100	Bozic	0	last	wholeT_0.01	S5:OT, S5:OT-A
675	No	9	9-B	100	Bozic	0	last	wholeT_0.5	J1:DiP, J1:DiP-A, J1:OT, J1:OT-A, S1:OT, S1:OT-A, S5:DiP, S5:DiP-A, S5:OT, S5:OT-A
676	No	9	9-B	100	Bozic	0	unif	singleC	S5:DiP, S5:DiP-A, S5:OT, S5:OT-A
677	No	9	9-B	100	Bozic	0	unif	wholeT_0.01	J1:OT, J1:OT-A, S5:DiP, S5:DiP-A, S5:OT, S5:OT-A
678	No	9	9-B	100	Bozic	0	unif	wholeT_0.5	S5:DiP, S5:DiP-A, S5:OT, S5:OT-A
679	No	9	9-B	100	Bozic	Inf	last	singleC	J1:OT, J1:OT-A, J5:OT, J5:OT-A, S5:OT, S5:OT-A
680	No	9	9-B	100	Bozic	Inf	last	wholeT_0.01	J5:OT, J5:OT-A
681	No	9	9-B	100	Bozic	Inf	last	wholeT_0.5	J1:OT, J1:OT-A, J5:OT, J5:OT-A, S5:OT, S5:OT-A
682	No	9	9-B	100	Bozic	Inf	unif	singleC	S5:DiP, S5:DiP-A, S5:OT, S5:OT-A
683	No	9	9-B	100	Bozic	Inf	unif	wholeT_0.01	J5:OT, J5:OT-A

Table 13: *(continued)*

	Conjunction	Drivers	Tree	S.Size	Model	sh	S.Time	S.Type	Best method(s)
684	No	9	9-B	100	Bozic	Inf	unif	wholeT_0.5	J1:OT, J1:OT-A, S5:DiP-A, S5:OT, S5:OT-A
685	No	9	9-B	100	exp	0	last	singleC	S1:OT, S1:OT-A
686	No	9	9-B	100	exp	0	last	wholeT_0.01	S1:OT, S1:OT-A, S5:OT, S5:OT-A
687	No	9	9-B	100	exp	0	last	wholeT_0.5	S1:OT, S1:OT-A, S5:OT
688	No	9	9-B	100	exp	0	unif	singleC	S1:DiP, S1:DiP-A, S1:OT, S1:OT-A, S5:DiP, S5:DiP-A, S5:OT, S5:OT-A
689	No	9	9-B	100	exp	0	unif	wholeT_0.01	J1:OT, J1:OT-A, S1:OT, S1:OT-A, S5:DiP, S5:DiP-A, S5:OT, S5:OT-A
690	No	9	9-B	100	exp	0	unif	wholeT_0.5	S1:OT, S1:OT-A, S5:DiP, S5:DiP-A, S5:OT, S5:OT-A
691	No	9	9-B	100	exp	Inf	last	singleC	S5:OT, S5:OT-A
692	No	9	9-B	100	exp	Inf	last	wholeT_0.01	J5:OT, J5:OT-A
693	No	9	9-B	100	exp	Inf	last	wholeT_0.5	S5:OT, S5:OT-A
694	No	9	9-B	100	exp	Inf	unif	singleC	J1:DiP, J1:DiP-A, J1:OT, J1:OT-A, S5:DiP, S5:DiP-A, S5:OT, S5:OT-A
695	No	9	9-B	100	exp	Inf	unif	wholeT_0.01	S5:OT, S5:OT-A
696	No	9	9-B	100	exp	Inf	unif	wholeT_0.5	J1:OT, S5:DiP, S5:DiP-A, S5:OT, S5:OT-A
697	No	9	9-B	100	McF_4	0	last	singleC	S5:OT, S5:OT-A
698	No	9	9-B	100	McF_4	0	last	wholeT_0.01	S5:OT, S5:OT-A
699	No	9	9-B	100	McF_4	0	last	wholeT_0.5	S5:OT, S5:OT-A
700	No	9	9-B	100	McF_4	0	unif	singleC	S5:OT, S5:OT-A
701	No	9	9-B	100	McF_4	0	unif	wholeT_0.01	S5:OT, S5:OT-A
702	No	9	9-B	100	McF_4	0	unif	wholeT_0.5	S5:OT, S5:OT-A
703	No	9	9-B	100	McF_4	Inf	last	singleC	S5:OT, S5:OT-A
704	No	9	9-B	100	McF_4	Inf	last	wholeT_0.01	S5:OT, S5:OT-A
705	No	9	9-B	100	McF_4	Inf	last	wholeT_0.5	S5:OT, S5:OT-A
706	No	9	9-B	100	McF_4	Inf	unif	singleC	S5:OT, S5:OT-A
707	No	9	9-B	100	McF_4	Inf	unif	wholeT_0.01	S5:OT, S5:OT-A
708	No	9	9-B	100	McF_4	Inf	unif	wholeT_0.5	S5:OT, S5:OT-A
709	No	9	9-B	100	McF_6	0	last	singleC	S5:OT, S5:OT-A
710	No	9	9-B	100	McF_6	0	last	wholeT_0.01	S5:OT, S5:OT-A
711	No	9	9-B	100	McF_6	0	last	wholeT_0.5	S5:OT, S5:OT-A
712	No	9	9-B	100	McF_6	0	unif	singleC	S5:CBN-A, S5:OT, S5:OT-A
713	No	9	9-B	100	McF_6	0	unif	wholeT_0.01	S5:CBN, S5:OT, S5:OT-A
714	No	9	9-B	100	McF_6	0	unif	wholeT_0.5	S5:CBN, S5:CBN-A, S5:OT, S5:OT-A
715	No	9	9-B	100	McF_6	Inf	last	singleC	S5:OT-A
716	No	9	9-B	100	McF_6	Inf	last	wholeT_0.01	S5:OT-A
717	No	9	9-B	100	McF_6	Inf	last	wholeT_0.5	S5:OT-A

Table 13: *(continued)*

	Conjunction	Drivers	Tree	S.Size	Model	sh	S.Time	S.Type	Best method(s)
718	No	9	9-B	100	McF_6	Inf	unif	singleC	S5:OT, S5:OT-A
719	No	9	9-B	100	McF_6	Inf	unif	wholeT_0.01	S5:CBN, S5:CBN-A, S5:OT, S5:OT-A
720	No	9	9-B	100	McF_6	Inf	unif	wholeT_0.5	S5:CBN, S5:CBN-A, S5:OT, S5:OT-A
721	No	7	7-B	1000	Bozic	0	last	singleC	S1:OT-A, S5:OT-A
722	No	7	7-B	1000	Bozic	0	last	wholeT_0.01	J1:DiP-A, J1:OT-A, J5:DiP-A, J5:OT-A, S1:OT-A, S5:DiP-A
723	No	7	7-B	1000	Bozic	0	last	wholeT_0.5	S1:OT-A
724	No	7	7-B	1000	Bozic	0	unif	singleC	S1:CBN-A, S1:OT, S1:OT-A
725	No	7	7-B	1000	Bozic	0	unif	wholeT_0.01	J5:CBN, J5:CBN-A, J5:DiP-A, J5:OT, J5:OT-A
726	No	7	7-B	1000	Bozic	0	unif	wholeT_0.5	J1:CBN, J1:CBN-A, J1:DiP, J1:DiP-A, J1:OT, J1:OT-A, J5:DiP, J5:DiP-A, S1:CBN, S1:CBN-A, S1:OT, S1:OT-A, S5:DiP, S5:DiP-A
727	No	7	7-B	1000	Bozic	Inf	last	singleC	J1:DiP-A, J1:OT-A, J5:DiP-A, J5:OT-A, S5:DiP-A, S5:OT-A
728	No	7	7-B	1000	Bozic	Inf	last	wholeT_0.01	J5:CBN-A, J5:DiP-A, J5:OT-A
729	No	7	7-B	1000	Bozic	Inf	last	wholeT_0.5	J1:DiP-A, J1:OT-A, S5:DiP-A, S5:OT-A
730	No	7	7-B	1000	Bozic	Inf	unif	singleC	S1:CBN, S1:CBN-A, S5:CBN
731	No	7	7-B	1000	Bozic	Inf	unif	wholeT_0.01	J1:OT, J1:OT-A, J5:CBN, J5:CBN-A, J5:DiP-A, J5:OT, J5:OT-A, S5:CBN, S5:CBN-A, S5:DiP-A, S5:OT, S5:OT-A
732	No	7	7-B	1000	Bozic	Inf	unif	wholeT_0.5	S1:CBN, S1:CBN-A
733	No	7	7-B	1000	exp	0	last	singleC	S1:OT-A
734	No	7	7-B	1000	exp	0	last	wholeT_0.01	J1:OT-A, S1:OT-A, S5:OT-A
735	No	7	7-B	1000	exp	0	last	wholeT_0.5	S1:OT-A
736	No	7	7-B	1000	exp	0	unif	singleC	J1:DiP, J1:DiP-A, J5:DiP, J5:DiP-A, S1:DiP, S1:DiP-A, S5:DiP, S5:DiP-A
737	No	7	7-B	1000	exp	0	unif	wholeT_0.01	J1:CBN, J1:CBN-A, J1:OT, J1:OT-A, S1:CBN-A
738	No	7	7-B	1000	exp	0	unif	wholeT_0.5	J1:DiP, J1:DiP-A, J5:DiP, J5:DiP-A, S1:DiP, S1:DiP-A, S5:DiP, S5:DiP-A
739	No	7	7-B	1000	exp	Inf	last	singleC	J1:OT-A, S1:OT-A, S5:OT-A
740	No	7	7-B	1000	exp	Inf	last	wholeT_0.01	S5:OT-A
741	No	7	7-B	1000	exp	Inf	last	wholeT_0.5	S1:OT-A, S5:OT-A
742	No	7	7-B	1000	exp	Inf	unif	singleC	S1:CBN, S1:CBN-A
743	No	7	7-B	1000	exp	Inf	unif	wholeT_0.01	J1:CBN, J1:CBN-A, J1:OT, J1:OT-A, J5:CBN, J5:CBN-A, J5:OT, J5:OT-A, S5:CBN, S5:CBN-A, S5:OT, S5:OT-A
744	No	7	7-B	1000	exp	Inf	unif	wholeT_0.5	S1:CBN, S1:CBN-A, S1:OT, S1:OT-A

Table 13: *(continued)*

	Conjunction	Drivers	Tree	S.Size	Model	sh	S.Time	S.Type	Best method(s)
745	No	7	7-B	1000	McF_4	0	last	singleC	S1:DiP-A, S1:OT-A, S5:DiP-A, S5:OT-A
746	No	7	7-B	1000	McF_4	0	last	wholeT_0.01	S5:DiP-A, S5:OT-A
747	No	7	7-B	1000	McF_4	0	last	wholeT_0.5	S1:DiP-A, S1:OT-A, S5:DiP-A, S5:OT-A
748	No	7	7-B	1000	McF_4	0	unif	singleC	S1:DiP-A, S1:OT, S1:OT-A
749	No	7	7-B	1000	McF_4	0	unif	wholeT_0.01	J1:DiP-A, J1:OT, J1:OT-A, S5:DiP-A, S5:OT, S5:OT-A
750	No	7	7-B	1000	McF_4	0	unif	wholeT_0.5	S1:DiP-A, S1:OT, S1:OT-A
751	No	7	7-B	1000	McF_4	Inf	last	singleC	S1:DiP-A, S1:OT-A, S5:DiP-A, S5:OT-A
752	No	7	7-B	1000	McF_4	Inf	last	wholeT_0.01	S5:DiP-A, S5:OT-A
753	No	7	7-B	1000	McF_4	Inf	last	wholeT_0.5	S1:DiP-A, S1:OT-A, S5:DiP-A, S5:OT-A
754	No	7	7-B	1000	McF_4	Inf	unif	singleC	S1:OT, S1:OT-A
755	No	7	7-B	1000	McF_4	Inf	unif	wholeT_0.01	J1:OT, J1:OT-A, S5:OT, S5:OT-A
756	No	7	7-B	1000	McF_4	Inf	unif	wholeT_0.5	S1:OT, S1:OT-A
757	No	7	7-B	1000	McF_6	0	last	singleC	J1:DiP-A, J1:OT-A, S5:DiP-A, S5:OT-A
758	No	7	7-B	1000	McF_6	0	last	wholeT_0.01	J5:DiP-A, J5:OT-A, S5:DiP-A, S5:OT-A
759	No	7	7-B	1000	McF_6	0	last	wholeT_0.5	J1:DiP-A, J1:OT-A, S1:OT-A, S5:DiP-A, S5:OT-A
760	No	7	7-B	1000	McF_6	0	unif	singleC	S1:DiP, S1:DiP-A, S1:OT, S1:OT-A, S5:DiP, S5:DiP-A, S5:OT, S5:OT-A
761	No	7	7-B	1000	McF_6	0	unif	wholeT_0.01	S5:DiP, S5:DiP-A, S5:OT, S5:OT-A
762	No	7	7-B	1000	McF_6	0	unif	wholeT_0.5	S1:DiP, S1:DiP-A, S1:OT, S1:OT-A, S5:DiP, S5:DiP-A, S5:OT, S5:OT-A
763	No	7	7-B	1000	McF_6	Inf	last	singleC	J1:DiP-A, J1:OT-A, S5:DiP-A, S5:OT-A
764	No	7	7-B	1000	McF_6	Inf	last	wholeT_0.01	J5:DiP-A, J5:OT-A, S5:DiP-A, S5:OT-A
765	No	7	7-B	1000	McF_6	Inf	last	wholeT_0.5	J1:DiP-A, J1:OT-A, S1:OT-A, S5:DiP-A, S5:OT-A
766	No	7	7-B	1000	McF_6	Inf	unif	singleC	S1:DiP-A, S1:OT, S1:OT-A, S5:DiP-A, S5:OT, S5:OT-A
767	No	7	7-B	1000	McF_6	Inf	unif	wholeT_0.01	S5:DiP-A, S5:OT, S5:OT-A
768	No	7	7-B	1000	McF_6	Inf	unif	wholeT_0.5	S1:DiP-A, S1:OT, S1:OT-A, S5:DiP-A, S5:OT, S5:OT-A
769	No	7	7-B	200	Bozic	0	last	singleC	S1:OT-A, S5:OT-A
770	No	7	7-B	200	Bozic	0	last	wholeT_0.01	J1:OT-A, J5:OT-A, S5:OT-A
771	No	7	7-B	200	Bozic	0	last	wholeT_0.5	S1:OT-A
772	No	7	7-B	200	Bozic	0	unif	singleC	J1:CBN, J1:CBN-A, J1:OT, J1:OT-A, S1:OT, S1:OT-A
773	No	7	7-B	200	Bozic	0	unif	wholeT_0.01	J5:CBN, J5:CBN-A, J5:OT, J5:OT-A
774	No	7	7-B	200	Bozic	0	unif	wholeT_0.5	J1:CBN, J1:CBN-A, J1:OT, J1:OT-A, S1:CBN-A, S1:OT, S1:OT-A
775	No	7	7-B	200	Bozic	Inf	last	singleC	S5:OT-A

Table 13: *(continued)*

	Conjunction	Drivers	Tree	S.Size	Model	sh	S.Time	S.Type	Best method(s)
776	No	7	7-B	200	Bozic	Inf	last	wholeT_0.01	J5:CBN-A, J5:OT-A
777	No	7	7-B	200	Bozic	Inf	last	wholeT_0.5	S5:OT-A
778	No	7	7-B	200	Bozic	Inf	unif	singleC	J1:CBN, J1:CBN-A, S5:CBN, S5:CBN-A, S5:OT, S5:OT-A
779	No	7	7-B	200	Bozic	Inf	unif	wholeT_0.01	J5:CBN-A, J5:OT, J5:OT-A, S5:CBN, S5:CBN-A, S5:OT, S5:OT-A
780	No	7	7-B	200	Bozic	Inf	unif	wholeT_0.5	J1:CBN, J1:CBN-A
781	No	7	7-B	200	exp	0	last	singleC	S1:OT-A
782	No	7	7-B	200	exp	0	last	wholeT_0.01	J1:OT-A, S1:OT-A, S5:OT-A
783	No	7	7-B	200	exp	0	last	wholeT_0.5	S1:OT-A
784	No	7	7-B	200	exp	0	unif	singleC	J1:CBN, J1:CBN-A, J1:OT, J5:DiP, J5:DiP-A, S1:CBN, S1:CBN-A, S1:OT, S1:OT-A, S5:DiP, S5:DiP-A
785	No	7	7-B	200	exp	0	unif	wholeT_0.01	J1:CBN, J1:CBN-A, J1:OT, J1:OT-A, S1:CBN-A, S1:OT, S1:OT-A
786	No	7	7-B	200	exp	0	unif	wholeT_0.5	J1:CBN, J1:CBN-A, J1:DiP, J1:OT, J1:OT-A, J5:DiP, J5:DiP-A, S1:CBN, S1:CBN-A, S1:DiP, S1:DiP-A, S1:OT, S1:OT-A, S5:DiP, S5:DiP-A
787	No	7	7-B	200	exp	Inf	last	singleC	J1:OT-A, S5:OT-A
788	No	7	7-B	200	exp	Inf	last	wholeT_0.01	J5:OT-A, S5:OT-A
789	No	7	7-B	200	exp	Inf	last	wholeT_0.5	S5:OT-A
790	No	7	7-B	200	exp	Inf	unif	singleC	J1:CBN, J1:CBN-A, J1:OT, J1:OT-A, S1:CBN, S1:CBN-A, S1:OT, S1:OT-A
791	No	7	7-B	200	exp	Inf	unif	wholeT_0.01	J5:CBN, J5:OT, J5:OT-A, S5:OT, S5:OT-A
792	No	7	7-B	200	exp	Inf	unif	wholeT_0.5	S1:CBN, S1:CBN-A, S1:OT, S1:OT-A
793	No	7	7-B	200	McF_4	0	last	singleC	S5:OT-A
794	No	7	7-B	200	McF_4	0	last	wholeT_0.01	S5:OT-A
795	No	7	7-B	200	McF_4	0	last	wholeT_0.5	S5:OT-A
796	No	7	7-B	200	McF_4	0	unif	singleC	J1:OT, J1:OT-A, S5:OT, S5:OT-A
797	No	7	7-B	200	McF_4	0	unif	wholeT_0.01	J1:OT, J1:OT-A, S5:OT, S5:OT-A
798	No	7	7-B	200	McF_4	0	unif	wholeT_0.5	J1:OT, J1:OT-A, S1:OT, S1:OT-A, S5:OT, S5:OT-A
799	No	7	7-B	200	McF_4	Inf	last	singleC	S5:OT-A
800	No	7	7-B	200	McF_4	Inf	last	wholeT_0.01	S5:OT-A
801	No	7	7-B	200	McF_4	Inf	last	wholeT_0.5	S5:OT-A
802	No	7	7-B	200	McF_4	Inf	unif	singleC	J1:OT, J1:OT-A, S5:OT, S5:OT-A
803	No	7	7-B	200	McF_4	Inf	unif	wholeT_0.01	J1:OT, J1:OT-A, S5:OT, S5:OT-A

Table 13: *(continued)*

	Conjunction	Drivers	Tree	S.Size	Model	sh	S.Time	S.Type	Best method(s)
804	No	7	7-B	200	McF_4	Inf	unif	wholeT_0.5	J1:OT, J1:OT-A, S1:OT, S1:OT-A, S5:OT, S5:OT-A
805	No	7	7-B	200	McF_6	0	last	singleC	J1:OT-A, J5:OT-A, S5:OT-A
806	No	7	7-B	200	McF_6	0	last	wholeT_0.01	J5:OT-A, S5:OT-A
807	No	7	7-B	200	McF_6	0	last	wholeT_0.5	J1:OT-A, S5:OT-A
808	No	7	7-B	200	McF_6	0	unif	singleC	S5:OT, S5:OT-A
809	No	7	7-B	200	McF_6	0	unif	wholeT_0.01	S5:OT, S5:OT-A
810	No	7	7-B	200	McF_6	0	unif	wholeT_0.5	S5:CBN-A, S5:OT, S5:OT-A
811	No	7	7-B	200	McF_6	Inf	last	singleC	J1:OT-A, J5:OT-A, S5:OT-A
812	No	7	7-B	200	McF_6	Inf	last	wholeT_0.01	J5:OT-A, S5:OT-A
813	No	7	7-B	200	McF_6	Inf	last	wholeT_0.5	J1:OT-A, S5:OT-A
814	No	7	7-B	200	McF_6	Inf	unif	singleC	S5:CBN-A, S5:OT, S5:OT-A
815	No	7	7-B	200	McF_6	Inf	unif	wholeT_0.01	J1:OT-A, S5:CBN-A, S5:OT, S5:OT-A
816	No	7	7-B	200	McF_6	Inf	unif	wholeT_0.5	J1:CBN-A, J1:OT, J1:OT-A, J5:CBN-A, S5:CBN-A, S5:OT, S5:OT-A
817	No	7	7-B	100	Bozic	0	last	singleC	S5:OT-A
818	No	7	7-B	100	Bozic	0	last	wholeT_0.01	J1:OT-A, J5:OT-A, S5:OT-A
819	No	7	7-B	100	Bozic	0	last	wholeT_0.5	S1:OT-A, S5:OT-A
820	No	7	7-B	100	Bozic	0	unif	singleC	J1:CBN, J1:CBN-A, J1:OT, J1:OT-A, S1:OT, S1:OT-A
821	No	7	7-B	100	Bozic	0	unif	wholeT_0.01	J5:CBN, J5:CBN-A, J5:OT, J5:OT-A
822	No	7	7-B	100	Bozic	0	unif	wholeT_0.5	J1:CBN, J1:CBN-A, J1:OT, J1:OT-A, J5:DiP, J5:DiP-A, S1:OT, S1:OT-A, S5:DiP, S5:DiP-A
823	No	7	7-B	100	Bozic	Inf	last	singleC	J1:OT-A, S5:OT-A
824	No	7	7-B	100	Bozic	Inf	last	wholeT_0.01	J5:CBN-A, J5:OT-A
825	No	7	7-B	100	Bozic	Inf	last	wholeT_0.5	S5:OT-A
826	No	7	7-B	100	Bozic	Inf	unif	singleC	S5:CBN, S5:CBN-A
827	No	7	7-B	100	Bozic	Inf	unif	wholeT_0.01	J5:CBN, J5:CBN-A, J5:OT, J5:OT-A
828	No	7	7-B	100	Bozic	Inf	unif	wholeT_0.5	J1:CBN, J1:CBN-A, S5:CBN, S5:CBN-A, S5:OT, S5:OT-A
829	No	7	7-B	100	exp	0	last	singleC	S1:OT-A
830	No	7	7-B	100	exp	0	last	wholeT_0.01	J1:CBN-A, J1:OT-A, J5:CBN-A, J5:OT-A, S1:OT-A, S5:OT-A
831	No	7	7-B	100	exp	0	last	wholeT_0.5	S1:OT-A
832	No	7	7-B	100	exp	0	unif	singleC	J1:CBN, J1:CBN-A, J1:OT, J1:OT-A, J5:DiP, J5:DiP-A, S1:CBN, S1:CBN-A, S1:OT, S1:OT-A, S5:DiP, S5:DiP-A

Table 13: *(continued)*

	Conjunction	Drivers	Tree	S.Size	Model	sh	S.Time	S.Type	Best method(s)
833	No	7	7-B	100	exp	0	unif	wholeT_0.01	J1:CBN, J1:CBN-A, J1:OT, J1:OT-A, J5:DiP, J5:DiP-A, S1:OT, S1:OT-A, S5:CBN, S5:CBN-A, S5:DiP, S5:DiP-A
834	No	7	7-B	100	exp	0	unif	wholeT_0.5	J1:CBN, J1:CBN-A, J1:OT, J1:OT-A, J5:DiP, J5:DiP-A, S1:CBN, S1:CBN-A, S1:OT, S1:OT-A, S5:DiP, S5:DiP-A
835	No	7	7-B	100	exp	Inf	last	singleC	S5:OT-A
836	No	7	7-B	100	exp	Inf	last	wholeT_0.01	J5:OT-A
837	No	7	7-B	100	exp	Inf	last	wholeT_0.5	S5:OT-A
838	No	7	7-B	100	exp	Inf	unif	singleC	J1:CBN, J1:CBN-A, J1:OT, J1:OT-A, J5:CBN, J5:OT-A, S1:OT, S1:OT-A, S5:CBN, S5:CBN-A, S5:OT, S5:OT-A
839	No	7	7-B	100	exp	Inf	unif	wholeT_0.01	J5:CBN, J5:CBN-A, J5:OT, J5:OT-A, S5:OT, S5:OT-A
840	No	7	7-B	100	exp	Inf	unif	wholeT_0.5	J1:CBN, J1:CBN-A, S1:CBN-A, S1:OT, S1:OT-A
841	No	7	7-B	100	McF_4	0	last	singleC	S5:OT-A
842	No	7	7-B	100	McF_4	0	last	wholeT_0.01	S5:OT-A
843	No	7	7-B	100	McF_4	0	last	wholeT_0.5	S5:OT-A
844	No	7	7-B	100	McF_4	0	unif	singleC	J1:OT, J1:OT-A, S5:OT, S5:OT-A
845	No	7	7-B	100	McF_4	0	unif	wholeT_0.01	J1:OT-A, S5:OT, S5:OT-A
846	No	7	7-B	100	McF_4	0	unif	wholeT_0.5	J1:OT, J1:OT-A, S5:OT, S5:OT-A
847	No	7	7-B	100	McF_4	Inf	last	singleC	S5:OT-A
848	No	7	7-B	100	McF_4	Inf	last	wholeT_0.01	S5:OT-A
849	No	7	7-B	100	McF_4	Inf	last	wholeT_0.5	S5:OT-A
850	No	7	7-B	100	McF_4	Inf	unif	singleC	J1:OT, J1:OT-A, S5:OT, S5:OT-A
851	No	7	7-B	100	McF_4	Inf	unif	wholeT_0.01	J1:OT-A, S5:OT, S5:OT-A
852	No	7	7-B	100	McF_4	Inf	unif	wholeT_0.5	J1:OT, J1:OT-A, J5:CBN-A, S5:OT, S5:OT-A
853	No	7	7-B	100	McF_6	0	last	singleC	J5:OT-A, S5:OT-A
854	No	7	7-B	100	McF_6	0	last	wholeT_0.01	J5:OT-A, S5:OT-A
855	No	7	7-B	100	McF_6	0	last	wholeT_0.5	S5:OT-A
856	No	7	7-B	100	McF_6	0	unif	singleC	J1:CBN-A, S5:CBN-A, S5:OT, S5:OT-A
857	No	7	7-B	100	McF_6	0	unif	wholeT_0.01	S5:CBN-A, S5:OT, S5:OT-A
858	No	7	7-B	100	McF_6	0	unif	wholeT_0.5	J1:CBN-A, S5:CBN-A, S5:OT, S5:OT-A
859	No	7	7-B	100	McF_6	Inf	last	singleC	S5:OT-A
860	No	7	7-B	100	McF_6	Inf	last	wholeT_0.01	J5:OT-A
861	No	7	7-B	100	McF_6	Inf	last	wholeT_0.5	J1:OT-A, S5:OT-A
862	No	7	7-B	100	McF_6	Inf	unif	singleC	J5:CBN-A, S5:CBN-A, S5:OT, S5:OT-A

Table 13: *(continued)*

	Conjunction	Drivers	Tree	S.Size	Model	sh	S.Time	S.Type	Best method(s)
863	No	7	7-B	100	McF_6	Inf	unif	wholeT_0.01	J1:CBN-A, J5:CBN-A, S5:CBN-A, S5:OT-A
864	No	7	7-B	100	McF_6	Inf	unif	wholeT_0.5	J1:CBN-A, J5:CBN-A, S5:CBN-A, S5:OT, S5:OT-A

5.2 Confidence sets (MCB), PFD, Drivers Unknown

Table 14: Confidence sets (method MCB) when Drivers are Unknown for measure PFD.

	Conjunction	Drivers	Tree	S.Size	Model	sh	S.Time	S.Type	Best method(s)
1	Yes	11	11-A	1000	Bozic	0	last	singleC	S1:OT, S1:OT-A
2	Yes	11	11-A	1000	Bozic	0	last	wholeT_0.01	J1:DiP, J1:DiP-A, J5:DiP, J5:DiP-A, S1:DiP, S1:DiP-A, S5:DiP, S5:DiP-A
3	Yes	11	11-A	1000	Bozic	0	last	wholeT_0.5	S1:OT, S1:OT-A
4	Yes	11	11-A	1000	Bozic	0	unif	singleC	S1:CBN, S1:CBN-A
5	Yes	11	11-A	1000	Bozic	0	unif	wholeT_0.01	J1:CBN-A, J1:DiP-A, S5:CBN, S5:CBN-A, S5:DiP, S5:DiP-A, S5:OT, S5:OT-A
6	Yes	11	11-A	1000	Bozic	0	unif	wholeT_0.5	S1:CBN, S1:CBN-A, S5:CBN, S5:CBN-A
7	Yes	11	11-A	1000	Bozic	Inf	last	singleC	J1:DiP, J1:DiP-A, J1:OT, J1:OT-A, J5:DiP, J5:DiP-A, J5:OT, J5:OT-A, S1:DiP-A, S5:DiP, S5:DiP-A, S5:OT, S5:OT-A
8	Yes	11	11-A	1000	Bozic	Inf	last	wholeT_0.01	J5:DiP, J5:DiP-A, J5:OT, J5:OT-A
9	Yes	11	11-A	1000	Bozic	Inf	last	wholeT_0.5	J1:DiP, J1:DiP-A, J1:OT, J1:OT-A, J5:DiP, J5:DiP-A, J5:OT, J5:OT-A, S5:DiP, S5:DiP-A, S5:OT, S5:OT-A
10	Yes	11	11-A	1000	Bozic	Inf	unif	singleC	J1:CBN-A, S1:CBN, S1:CBN-A, S1:DiP, S1:DiP-A, S1:OT, S1:OT-A
11	Yes	11	11-A	1000	Bozic	Inf	unif	wholeT_0.01	J1:CBN, J1:CBN-A, S5:CBN-A, S5:OT, S5:OT-A
12	Yes	11	11-A	1000	Bozic	Inf	unif	wholeT_0.5	S1:CBN, S1:CBN-A, S1:DiP-A, S1:OT, S1:OT-A
13	Yes	11	11-A	1000	exp	0	last	singleC	S1:OT, S1:OT-A
14	Yes	11	11-A	1000	exp	0	last	wholeT_0.01	J1:DiP, J1:DiP-A, J1:OT, J1:OT-A, S1:DiP-A, S1:OT, S5:DiP-A, S5:OT, S5:OT-A
15	Yes	11	11-A	1000	exp	0	last	wholeT_0.5	S1:OT, S1:OT-A
16	Yes	11	11-A	1000	exp	0	unif	singleC	S1:CBN, S1:CBN-A, S5:CBN, S5:CBN-A
17	Yes	11	11-A	1000	exp	0	unif	wholeT_0.01	S1:DiP, S1:DiP-A, S1:OT, S1:OT-A
18	Yes	11	11-A	1000	exp	0	unif	wholeT_0.5	S1:CBN, S1:CBN-A, S5:CBN, S5:CBN-A
19	Yes	11	11-A	1000	exp	Inf	last	singleC	J1:DiP, J1:DiP-A, J1:OT, J1:OT-A, S5:DiP, S5:DiP-A, S5:OT, S5:OT-A
20	Yes	11	11-A	1000	exp	Inf	last	wholeT_0.01	J5:DiP, J5:DiP-A, J5:OT, J5:OT-A, S5:DiP, S5:DiP-A, S5:OT, S5:OT-A
21	Yes	11	11-A	1000	exp	Inf	last	wholeT_0.5	J1:DiP, J1:DiP-A, J1:OT, J1:OT-A, S5:DiP, S5:OT, S5:OT-A
22	Yes	11	11-A	1000	exp	Inf	unif	singleC	S1:CBN, S1:CBN-A, S1:OT, S1:OT-A

Table 14: *(continued)*

	Conjunction	Drivers	Tree	S.Size	Model	sh	S.Time	S.Type	Best method(s)
23	Yes	11	11-A	1000	exp	Inf	unif	wholeT_0.01	J1:CBN-A, J1:DiP, J1:DiP-A, J1:OT, J1:OT-A
24	Yes	11	11-A	1000	exp	Inf	unif	wholeT_0.5	S1:CBN, S1:CBN-A, S1:OT, S1:OT-A
25	Yes	11	11-A	1000	McF_4	0	last	singleC	J1:DiP, J1:DiP-A, J1:OT, J1:OT-A, J5:DiP-A, J5:OT, J5:OT-A, S1:DiP-A, S1:OT, S1:OT-A, S5:DiP, S5:DiP-A, S5:OT, S5:OT-A
26	Yes	11	11-A	1000	McF_4	0	last	wholeT_0.01	J1:DiP, J1:DiP-A, J1:OT, J1:OT-A, J5:DiP, J5:DiP-A, J5:OT, J5:OT-A, S5:DiP, S5:OT, S5:OT-A
27	Yes	11	11-A	1000	McF_4	0	last	wholeT_0.5	J1:DiP, J1:DiP-A, J1:OT, J1:OT-A, J5:DiP, J5:DiP-A, J5:OT, J5:OT-A, S1:DiP-A, S1:OT, S1:OT-A, S5:DiP, S5:DiP-A, S5:OT, S5:OT-A
28	Yes	11	11-A	1000	McF_4	0	unif	singleC	J1:CBN-A, J1:DiP, J1:OT, J1:OT-A, S1:DiP-A, S1:OT, S1:OT-A, S5:DiP, S5:DiP-A, S5:OT, S5:OT-A
29	Yes	11	11-A	1000	McF_4	0	unif	wholeT_0.01	J1:CBN-A, J1:DiP, J1:DiP-A, J1:OT, J1:OT-A, S5:DiP, S5:DiP-A, S5:OT, S5:OT-A
30	Yes	11	11-A	1000	McF_4	0	unif	wholeT_0.5	J1:CBN, J1:DiP, J1:DiP-A, J1:OT, S1:DiP, S1:DiP-A, S1:OT, S1:OT-A, S5:DiP, S5:DiP-A, S5:OT, S5:OT-A
31	Yes	11	11-A	1000	McF_4	Inf	last	singleC	J1:DiP, J1:DiP-A, J1:OT, J1:OT-A, J5:DiP, J5:DiP-A, J5:OT, J5:OT-A, S1:OT-A, S5:DiP, S5:DiP-A, S5:OT-A
32	Yes	11	11-A	1000	McF_4	Inf	last	wholeT_0.01	J1:DiP, J1:DiP-A, J1:OT, J1:OT-A, J5:DiP, J5:DiP-A, J5:OT, J5:OT-A, S5:DiP, S5:DiP-A, S5:OT, S5:OT-A
33	Yes	11	11-A	1000	McF_4	Inf	last	wholeT_0.5	J1:DiP, J1:DiP-A, J1:OT, J1:OT-A, J5:DiP, J5:DiP-A, J5:OT, J5:OT-A, S1:DiP-A, S1:OT, S1:OT-A, S5:DiP, S5:DiP-A, S5:OT, S5:OT-A
34	Yes	11	11-A	1000	McF_4	Inf	unif	singleC	J1:CBN-A, J1:DiP, J1:DiP-A, J1:OT, J1:OT-A, S1:DiP, S1:DiP-A, S1:OT, S1:OT-A, S5:DiP, S5:DiP-A, S5:OT, S5:OT-A

Table 14: *(continued)*

	Conjunction	Drivers	Tree	S.Size	Model	sh	S.Time	S.Type	Best method(s)
35	Yes	11	11-A	1000	McF_4	Inf	unif	wholeT_0.01	J1:CBN-A, J1:DiP, J1:DiP-A, J1:OT, J1:OT-A, J5:DiP, J5:DiP-A, J5:OT, J5:OT-A, S1:DiP, S1:DiP-A, S5:CBN-A, S5:DiP, S5:DiP-A, S5:OT, S5:OT-A
36	Yes	11	11-A	1000	McF_4	Inf	unif	wholeT_0.5	J1:DiP, J1:DiP-A, J1:OT, J1:OT-A, S1:DiP, S1:DiP-A, S1:OT, S1:OT-A, S5:DiP-A, S5:OT, S5:OT-A
37	Yes	11	11-A	1000	McF_6	0	last	singleC	J1:DiP, J1:OT, J5:DiP, J5:OT, S1:DiP, S1:OT, S5:DiP, S5:OT
38	Yes	11	11-A	1000	McF_6	0	last	wholeT_0.01	J1:DiP, J1:OT, J5:DiP, J5:OT, S1:DiP, S5:DiP, S5:OT
39	Yes	11	11-A	1000	McF_6	0	last	wholeT_0.5	J1:DiP, J1:OT, J5:DiP, J5:OT, S1:DiP, S5:DiP, S5:OT
40	Yes	11	11-A	1000	McF_6	0	unif	singleC	J1:CBN, J1:CBN-A, J1:DiP, J1:DiP-A, J1:OT, J1:OT-A, J5:CBN, J5:CBN-A, J5:DiP, J5:DiP-A, J5:OT, J5:OT-A, S1:DiP, S1:DiP-A, S1:OT, S1:OT-A, S5:CBN, S5:CBN-A, S5:DiP, S5:DiP-A, S5:OT
41	Yes	11	11-A	1000	McF_6	0	unif	wholeT_0.01	J1:CBN-A, J1:DiP, J1:DiP-A, J1:OT, J1:OT-A, J5:CBN, J5:CBN-A, J5:DiP, J5:DiP-A, J5:OT, J5:OT-A, S1:DiP, S1:DiP-A, S5:CBN, S5:DiP, S5:DiP-A, S5:OT, S5:OT-A
42	Yes	11	11-A	1000	McF_6	0	unif	wholeT_0.5	J1:CBN, J1:DiP, J1:DiP-A, J1:OT-A, J5:CBN, J5:CBN-A, J5:DiP, J5:DiP-A, J5:OT, J5:OT-A, S1:DiP, S1:DiP-A, S1:OT, S1:OT-A, S5:CBN-A, S5:DiP, S5:DiP-A, S5:OT, S5:OT-A
43	Yes	11	11-A	1000	McF_6	Inf	last	singleC	J1:DiP, J1:OT, J5:DiP, J5:OT, S1:DiP, S5:DiP, S5:OT
44	Yes	11	11-A	1000	McF_6	Inf	last	wholeT_0.01	J1:DiP, J1:OT, J5:DiP, J5:OT, S1:DiP, S5:DiP, S5:OT
45	Yes	11	11-A	1000	McF_6	Inf	last	wholeT_0.5	J1:DiP, J1:OT, J5:DiP, J5:OT, S1:DiP, S5:DiP, S5:OT
46	Yes	11	11-A	1000	McF_6	Inf	unif	singleC	J1:CBN-A, J1:DiP, J1:DiP-A, J5:CBN-A, J5:DiP, J5:DiP-A, J5:OT, J5:OT-A, S1:DiP, S1:DiP-A, S5:CBN-A, S5:DiP, S5:DiP-A, S5:OT, S5:OT-A

Table 14: *(continued)*

	Conjunction	Drivers	Tree	S.Size	Model	sh	S.Time	S.Type	Best method(s)
47	Yes	11	11-A	1000	McF_6	Inf	unif	wholeT_0.01	J1:CBN, J1:CBN-A, J1:DiP, J1:DiP-A, J1:OT, J5:CBN-A, J5:DiP, J5:DiP-A, J5:OT, J5:OT-A, S1:DiP, S1:DiP-A, S5:CBN-A, S5:DiP, S5:DiP-A, S5:OT
48	Yes	11	11-A	1000	McF_6	Inf	unif	wholeT_0.5	J1:CBN-A, J1:DiP, J1:DiP-A, J5:CBN, J5:CBN-A, J5:DiP, J5:DiP-A, J5:OT, J5:OT-A, S1:DiP, S1:DiP-A, S5:CBN-A, S5:DiP, S5:DiP-A
49	Yes	11	11-A	200	Bozic	0	last	singleC	S1:OT, S5:OT, S5:OT-A
50	Yes	11	11-A	200	Bozic	0	last	wholeT_0.01	J1:CBN, J1:OT, J1:OT-A, J5:OT, J5:OT-A, S5:OT, S5:OT-A
51	Yes	11	11-A	200	Bozic	0	last	wholeT_0.5	S1:OT, S1:OT-A
52	Yes	11	11-A	200	Bozic	0	unif	singleC	S1:OT, S1:OT-A
53	Yes	11	11-A	200	Bozic	0	unif	wholeT_0.01	J1:DiP, J1:DiP-A, J1:OT-A, S1:DiP-A, S5:CBN, S5:CBN-A, S5:DiP, S5:DiP-A, S5:OT, S5:OT-A
54	Yes	11	11-A	200	Bozic	0	unif	wholeT_0.5	J1:CBN, J1:CBN-A, S1:CBN, S1:CBN-A
55	Yes	11	11-A	200	Bozic	Inf	last	singleC	J5:OT, J5:OT-A, S5:OT, S5:OT-A
56	Yes	11	11-A	200	Bozic	Inf	last	wholeT_0.01	J5:CBN-A, J5:OT, J5:OT-A
57	Yes	11	11-A	200	Bozic	Inf	last	wholeT_0.5	J5:OT, J5:OT-A, S5:OT, S5:OT-A
58	Yes	11	11-A	200	Bozic	Inf	unif	singleC	J1:CBN, J1:CBN-A, J1:OT, J1:OT-A
59	Yes	11	11-A	200	Bozic	Inf	unif	wholeT_0.01	S5:CBN, S5:CBN-A, S5:OT, S5:OT-A
60	Yes	11	11-A	200	Bozic	Inf	unif	wholeT_0.5	J1:CBN, J1:CBN-A, J1:OT, J1:OT-A
61	Yes	11	11-A	200	exp	0	last	singleC	S1:OT, S1:OT-A
62	Yes	11	11-A	200	exp	0	last	wholeT_0.01	J1:OT, J1:OT-A, S5:OT, S5:OT-A
63	Yes	11	11-A	200	exp	0	last	wholeT_0.5	S1:OT, S1:OT-A
64	Yes	11	11-A	200	exp	0	unif	singleC	S1:CBN, S1:CBN-A, S5:CBN, S5:CBN-A
65	Yes	11	11-A	200	exp	0	unif	wholeT_0.01	S1:OT, S1:OT-A
66	Yes	11	11-A	200	exp	0	unif	wholeT_0.5	S1:CBN, S1:CBN-A
67	Yes	11	11-A	200	exp	Inf	last	singleC	J1:OT-A, S5:OT, S5:OT-A
68	Yes	11	11-A	200	exp	Inf	last	wholeT_0.01	J5:CBN-A, J5:OT, J5:OT-A
69	Yes	11	11-A	200	exp	Inf	last	wholeT_0.5	J1:OT, J1:OT-A, S5:OT, S5:OT-A
70	Yes	11	11-A	200	exp	Inf	unif	singleC	S1:OT, S1:OT-A
71	Yes	11	11-A	200	exp	Inf	unif	wholeT_0.01	S5:CBN-A, S5:OT, S5:OT-A
72	Yes	11	11-A	200	exp	Inf	unif	wholeT_0.5	S1:OT, S1:OT-A
73	Yes	11	11-A	200	McF_4	0	last	singleC	J1:DiP-A, J1:OT, J1:OT-A, J5:DiP-A, J5:OT, J5:OT-A, S1:DiP, S5:DiP, S5:DiP-A, S5:OT, S5:OT-A

Table 14: *(continued)*

	Conjunction	Drivers	Tree	S.Size	Model	sh	S.Time	S.Type	Best method(s)
74	Yes	11	11-A	200	McF_4	0	last	wholeT_0.01	J1:DiP, J1:DiP-A, J1:OT, J1:OT-A, J5:DiP-A, J5:OT, J5:OT-A, S1:DiP-A, S5:DiP, S5:DiP-A, S5:OT, S5:OT-A
75	Yes	11	11-A	200	McF_4	0	last	wholeT_0.5	J1:OT, J1:OT-A, J5:OT, J5:OT-A, S1:DiP-A, S5:DiP-A, S5:OT, S5:OT-A
76	Yes	11	11-A	200	McF_4	0	unif	singleC	J1:CBN-A, J1:OT, J1:OT-A, S5:CBN-A, S5:OT, S5:OT-A
77	Yes	11	11-A	200	McF_4	0	unif	wholeT_0.01	J1:CBN-A, J1:DiP, J1:DiP-A, J1:OT, J1:OT-A, S1:DiP, S1:DiP-A, S5:CBN, S5:CBN-A, S5:DiP, S5:DiP-A, S5:OT, S5:OT-A
78	Yes	11	11-A	200	McF_4	0	unif	wholeT_0.5	J1:CBN, J1:CBN-A, J1:OT, J1:OT-A, S1:DiP, S5:CBN, S5:CBN-A, S5:OT, S5:OT-A
79	Yes	11	11-A	200	McF_4	Inf	last	singleC	J1:OT, J1:OT-A, J5:OT, J5:OT-A, S5:OT, S5:OT-A
80	Yes	11	11-A	200	McF_4	Inf	last	wholeT_0.01	J1:DiP-A, J1:OT, J5:DiP-A, J5:OT, J5:OT-A, S5:OT-A
81	Yes	11	11-A	200	McF_4	Inf	last	wholeT_0.5	J1:OT, J1:OT-A, J5:OT, J5:OT-A, S5:OT, S5:OT-A
82	Yes	11	11-A	200	McF_4	Inf	unif	singleC	J1:CBN-A, J1:OT, J1:OT-A, J5:CBN-A, J5:OT, J5:OT-A, S5:CBN-A, S5:OT, S5:OT-A
83	Yes	11	11-A	200	McF_4	Inf	unif	wholeT_0.01	J1:CBN-A, J1:OT, J1:OT-A, J5:OT, J5:OT-A, S5:CBN-A, S5:OT, S5:OT-A
84	Yes	11	11-A	200	McF_4	Inf	unif	wholeT_0.5	J1:CBN-A, J1:OT, J1:OT-A, J5:OT, J5:OT-A, S5:OT, S5:OT-A
85	Yes	11	11-A	200	McF_6	0	last	singleC	J1:OT, J5:OT, S1:DiP, S5:DiP, S5:OT
86	Yes	11	11-A	200	McF_6	0	last	wholeT_0.01	J1:DiP, J5:DiP, J5:OT, S1:DiP, S5:DiP
87	Yes	11	11-A	200	McF_6	0	last	wholeT_0.5	J1:OT, J5:OT, S1:DiP, S5:DiP, S5:OT
88	Yes	11	11-A	200	McF_6	0	unif	singleC	J1:CBN, J1:CBN-A, J1:OT, J1:OT-A, J5:CBN, J5:CBN-A, J5:DiP-A, J5:OT, J5:OT-A, S5:CBN, S5:CBN-A, S5:OT, S5:OT-A
89	Yes	11	11-A	200	McF_6	0	unif	wholeT_0.01	J1:CBN, J1:CBN-A, J1:DiP, J1:DiP-A, J1:OT, J1:OT-A, J5:CBN, J5:CBN-A, J5:DiP, J5:DiP-A, J5:OT, J5:OT-A, S5:CBN, S5:CBN-A, S5:DiP, S5:DiP-A, S5:OT, S5:OT-A

Table 14: *(continued)*

	Conjunction	Drivers	Tree	S.Size	Model	sh	S.Time	S.Type	Best method(s)
90	Yes	11	11-A	200	McF_6	0	unif	wholeT_0.5	J1:CBN, J1:CBN-A, J1:DiP, J1:DiP-A, J1:OT, J1:OT-A, J5:CBN, J5:CBN-A, J5:DiP-A, J5:OT, J5:OT-A, S5:CBN, S5:CBN-A, S5:DiP, S5:DiP-A, S5:OT, S5:OT-A
91	Yes	11	11-A	200	McF_6	Inf	last	singleC	J1:OT, J5:OT, S5:OT
92	Yes	11	11-A	200	McF_6	Inf	last	wholeT_0.01	J1:OT, J5:OT, S5:OT
93	Yes	11	11-A	200	McF_6	Inf	last	wholeT_0.5	J1:OT, J5:OT, S5:OT
94	Yes	11	11-A	200	McF_6	Inf	unif	singleC	J1:CBN-A, J1:OT, J1:OT-A, J5:CBN, J5:CBN-A, J5:OT, J5:OT-A, S5:CBN, S5:CBN-A, S5:OT, S5:OT-A
95	Yes	11	11-A	200	McF_6	Inf	unif	wholeT_0.01	J1:CBN, J1:CBN-A, J1:OT, J1:OT-A, J5:CBN-A, J5:OT, J5:OT-A, S5:CBN, S5:CBN-A, S5:OT, S5:OT-A
96	Yes	11	11-A	200	McF_6	Inf	unif	wholeT_0.5	J1:CBN, J1:CBN-A, J1:OT, J1:OT-A, J5:CBN, J5:CBN-A, J5:OT, J5:OT-A, S5:CBN, S5:CBN-A, S5:OT, S5:OT-A
97	Yes	11	11-A	100	Bozic	0	last	singleC	S5:OT, S5:OT-A
98	Yes	11	11-A	100	Bozic	0	last	wholeT_0.01	J5:OT, J5:OT-A, S5:OT, S5:OT-A
99	Yes	11	11-A	100	Bozic	0	last	wholeT_0.5	J1:CBN, J1:CBN-A, J1:OT, J1:OT-A, S1:OT, S1:OT-A, S5:CBN-A
100	Yes	11	11-A	100	Bozic	0	unif	singleC	J1:CBN-A, S1:OT, S1:OT-A
101	Yes	11	11-A	100	Bozic	0	unif	wholeT_0.01	S5:OT, S5:OT-A
102	Yes	11	11-A	100	Bozic	0	unif	wholeT_0.5	S1:OT, S1:OT-A
103	Yes	11	11-A	100	Bozic	Inf	last	singleC	J5:OT, J5:OT-A
104	Yes	11	11-A	100	Bozic	Inf	last	wholeT_0.01	J5:CBN-A, J5:OT, J5:OT-A
105	Yes	11	11-A	100	Bozic	Inf	last	wholeT_0.5	J5:OT, J5:OT-A
106	Yes	11	11-A	100	Bozic	Inf	unif	singleC	J1:CBN, J1:CBN-A, J1:OT, J1:OT-A, S1:OT, S1:OT-A, S5:CBN, S5:CBN-A
107	Yes	11	11-A	100	Bozic	Inf	unif	wholeT_0.01	J5:OT, J5:OT-A
108	Yes	11	11-A	100	Bozic	Inf	unif	wholeT_0.5	J1:CBN, J1:CBN-A, J1:OT, J1:OT-A, S1:OT, S1:OT-A, S5:CBN, S5:CBN-A
109	Yes	11	11-A	100	exp	0	last	singleC	S1:OT, S1:OT-A
110	Yes	11	11-A	100	exp	0	last	wholeT_0.01	J1:OT, J1:OT-A, S5:OT, S5:OT-A
111	Yes	11	11-A	100	exp	0	last	wholeT_0.5	S1:OT, S1:OT-A
112	Yes	11	11-A	100	exp	0	unif	singleC	S1:CBN, S1:CBN-A
113	Yes	11	11-A	100	exp	0	unif	wholeT_0.01	J1:CBN-A, S1:OT, S1:OT-A
114	Yes	11	11-A	100	exp	0	unif	wholeT_0.5	S1:CBN, S1:CBN-A, S5:CBN, S5:CBN-A
115	Yes	11	11-A	100	exp	Inf	last	singleC	S5:OT, S5:OT-A
116	Yes	11	11-A	100	exp	Inf	last	wholeT_0.01	J5:CBN-A, J5:OT, J5:OT-A

Table 14: *(continued)*

	Conjunction	Drivers	Tree	S.Size	Model	sh	S.Time	S.Type	Best method(s)
117	Yes	11	11-A	100	exp	Inf	last	wholeT_0.5	S5:OT, S5:OT-A
118	Yes	11	11-A	100	exp	Inf	unif	singleC	J1:CBN-A, J1:OT, J1:OT-A, S1:OT, S1:OT-A
119	Yes	11	11-A	100	exp	Inf	unif	wholeT_0.01	S5:CBN, S5:OT, S5:OT-A
120	Yes	11	11-A	100	exp	Inf	unif	wholeT_0.5	J1:CBN-A, J1:OT, J1:OT-A, S1:OT, S1:OT-A
121	Yes	11	11-A	100	McF_4	0	last	singleC	J5:OT, J5:OT-A, S5:OT, S5:OT-A
122	Yes	11	11-A	100	McF_4	0	last	wholeT_0.01	J1:DiP-A, J1:OT-A, J5:OT, J5:OT-A, S1:DiP, S1:DiP-A, S5:DiP, S5:DiP-A, S5:OT, S5:OT-A
123	Yes	11	11-A	100	McF_4	0	last	wholeT_0.5	J5:OT, J5:OT-A, S5:OT, S5:OT-A
124	Yes	11	11-A	100	McF_4	0	unif	singleC	J1:CBN, J1:OT, J1:OT-A, S5:OT, S5:OT-A
125	Yes	11	11-A	100	McF_4	0	unif	wholeT_0.01	S5:CBN, S5:CBN-A, S5:DiP-A, S5:OT, S5:OT-A
126	Yes	11	11-A	100	McF_4	0	unif	wholeT_0.5	J1:CBN-A, J1:OT, J1:OT-A, S5:CBN, S5:CBN-A, S5:OT, S5:OT-A
127	Yes	11	11-A	100	McF_4	Inf	last	singleC	J5:OT, J5:OT-A, S5:OT, S5:OT-A
128	Yes	11	11-A	100	McF_4	Inf	last	wholeT_0.01	J5:OT, J5:OT-A
129	Yes	11	11-A	100	McF_4	Inf	last	wholeT_0.5	J5:OT, J5:OT-A, S5:OT, S5:OT-A
130	Yes	11	11-A	100	McF_4	Inf	unif	singleC	J1:OT, J1:OT-A, J5:OT, J5:OT-A, S5:CBN-A, S5:OT, S5:OT-A
131	Yes	11	11-A	100	McF_4	Inf	unif	wholeT_0.01	J5:OT, J5:OT-A, S5:OT, S5:OT-A
132	Yes	11	11-A	100	McF_4	Inf	unif	wholeT_0.5	J1:CBN-A, J1:OT, J5:OT, J5:OT-A, S5:CBN-A, S5:OT, S5:OT-A
133	Yes	11	11-A	100	McF_6	0	last	singleC	J1:OT, J5:OT, S1:DiP, S5:DiP, S5:OT
134	Yes	11	11-A	100	McF_6	0	last	wholeT_0.01	J1:DiP, J5:OT, S1:DiP, S5:DiP, S5:OT
135	Yes	11	11-A	100	McF_6	0	last	wholeT_0.5	J1:OT, J5:OT, S5:OT
136	Yes	11	11-A	100	McF_6	0	unif	singleC	J1:CBN, J1:CBN-A, J5:CBN, J5:CBN-A, J5:OT, J5:OT-A, S5:CBN-A, S5:OT
137	Yes	11	11-A	100	McF_6	0	unif	wholeT_0.01	J1:CBN-A, J1:DiP, J1:DiP-A, J5:CBN, J5:CBN-A, J5:DiP, J5:DiP-A, J5:OT, J5:OT-A, S1:DiP, S1:DiP-A, S5:CBN, S5:DiP, S5:DiP-A, S5:OT, S5:OT-A
138	Yes	11	11-A	100	McF_6	0	unif	wholeT_0.5	J1:CBN, J1:CBN-A, J1:OT, J1:OT-A, J5:CBN-A, S5:CBN, S5:CBN-A
139	Yes	11	11-A	100	McF_6	Inf	last	singleC	J1:OT, J5:OT, S5:OT
140	Yes	11	11-A	100	McF_6	Inf	last	wholeT_0.01	J5:OT, S5:OT
141	Yes	11	11-A	100	McF_6	Inf	last	wholeT_0.5	J1:OT, J5:OT, S5:OT

Table 14: *(continued)*

	Conjunction	Drivers	Tree	S.Size	Model	sh	S.Time	S.Type	Best method(s)
142	Yes	11	11-A	100	McF_6	Inf	unif	singleC	J1:CBN, J1:CBN-A, J1:OT, J1:OT-A, J5:CBN-A, J5:OT, J5:OT-A, S5:CBN, S5:CBN-A, S5:OT, S5:OT-A
143	Yes	11	11-A	100	McF_6	Inf	unif	wholeT_0.01	J1:CBN-A, J5:CBN, J5:CBN-A, J5:OT, J5:OT-A, S5:CBN, S5:CBN-A, S5:OT, S5:OT-A
144	Yes	11	11-A	100	McF_6	Inf	unif	wholeT_0.5	J1:CBN-A, J1:OT, J1:OT-A, J5:CBN-A, J5:OT, J5:OT-A, S5:CBN, S5:CBN-A, S5:OT, S5:OT-A
145	Yes	9	9-A	1000	Bozic	0	last	singleC	S1:OT, S1:OT-A
146	Yes	9	9-A	1000	Bozic	0	last	wholeT_0.01	J1:DiP, J1:OT, J1:OT-A, J5:DiP, J5:DiP-A, J5:OT, J5:OT-A, S1:DiP, S1:DiP-A, S1:OT, S1:OT-A, S5:DiP, S5:DiP-A, S5:OT, S5:OT-A
147	Yes	9	9-A	1000	Bozic	0	last	wholeT_0.5	S1:OT, S1:OT-A
148	Yes	9	9-A	1000	Bozic	0	unif	singleC	S1:CBN, S1:CBN-A, S5:CBN, S5:CBN-A
149	Yes	9	9-A	1000	Bozic	0	unif	wholeT_0.01	J1:DiP, J1:DiP-A, J1:OT, J1:OT-A, S1:DiP, S1:DiP-A, S1:OT, S1:OT-A
150	Yes	9	9-A	1000	Bozic	0	unif	wholeT_0.5	S1:CBN, S1:CBN-A, S5:CBN, S5:CBN-A
151	Yes	9	9-A	1000	Bozic	Inf	last	singleC	J1:DiP, J1:DiP-A, J1:OT, J1:OT-A, J5:DiP, J5:DiP-A, J5:OT, J5:OT-A, S5:OT, S5:OT-A
152	Yes	9	9-A	1000	Bozic	Inf	last	wholeT_0.01	J5:DiP, J5:DiP-A, J5:OT, J5:OT-A
153	Yes	9	9-A	1000	Bozic	Inf	last	wholeT_0.5	J1:DiP, J1:DiP-A, J1:OT-A, J5:DiP, J5:DiP-A, J5:OT, J5:OT-A, S5:DiP, S5:DiP-A, S5:OT, S5:OT-A
154	Yes	9	9-A	1000	Bozic	Inf	unif	singleC	S1:CBN, S1:DiP, S1:DiP-A, S1:OT, S1:OT-A
155	Yes	9	9-A	1000	Bozic	Inf	unif	wholeT_0.01	S5:DiP, S5:DiP-A, S5:OT, S5:OT-A
156	Yes	9	9-A	1000	Bozic	Inf	unif	wholeT_0.5	S1:CBN, S1:DiP-A, S1:OT, S1:OT-A
157	Yes	9	9-A	1000	exp	0	last	singleC	S1:CBN, S1:CBN-A, S5:CBN, S5:CBN-A
158	Yes	9	9-A	1000	exp	0	last	wholeT_0.01	S1:DiP-A, S1:OT, S1:OT-A
159	Yes	9	9-A	1000	exp	0	last	wholeT_0.5	J1:CBN, J1:CBN-A, S1:CBN, S1:CBN-A, S5:CBN, S5:CBN-A
160	Yes	9	9-A	1000	exp	0	unif	singleC	S1:CBN, S1:CBN-A, S5:CBN, S5:CBN-A
161	Yes	9	9-A	1000	exp	0	unif	wholeT_0.01	J1:CBN, S1:CBN, S1:CBN-A, S5:CBN-A
162	Yes	9	9-A	1000	exp	0	unif	wholeT_0.5	S1:CBN, S1:CBN-A, S5:CBN, S5:CBN-A
163	Yes	9	9-A	1000	exp	Inf	last	singleC	J1:DiP, J1:DiP-A, J1:OT, S5:DiP, S5:DiP-A, S5:OT, S5:OT-A
164	Yes	9	9-A	1000	exp	Inf	last	wholeT_0.01	J5:DiP, J5:DiP-A, J5:OT, J5:OT-A

Table 14: *(continued)*

	Conjunction	Drivers	Tree	S.Size	Model	sh	S.Time	S.Type	Best method(s)
165	Yes	9	9-A	1000	exp	Inf	last	wholeT_0.5	J1:DiP, J1:DiP-A, J1:OT, J1:OT-A, S5:DiP, S5:DiP-A, S5:OT, S5:OT-A
166	Yes	9	9-A	1000	exp	Inf	unif	singleC	S1:OT, S1:OT-A
167	Yes	9	9-A	1000	exp	Inf	unif	wholeT_0.01	J1:DiP, J1:DiP-A, J1:OT, J1:OT-A
168	Yes	9	9-A	1000	exp	Inf	unif	wholeT_0.5	S1:OT, S1:OT-A
169	Yes	9	9-A	1000	McF_4	0	last	singleC	J1:DiP, J1:DiP-A, J1:OT, J1:OT-A, J5:DiP, J5:DiP-A, J5:OT, J5:OT-A, S1:DiP, S1:DiP-A, S1:OT, S5:DiP, S5:DiP-A, S5:OT, S5:OT-A
170	Yes	9	9-A	1000	McF_4	0	last	wholeT_0.01	J1:DiP, J1:DiP-A, J1:OT, J1:OT-A, J5:DiP, J5:DiP-A, J5:OT, J5:OT-A, S5:DiP, S5:DiP-A, S5:OT, S5:OT-A
171	Yes	9	9-A	1000	McF_4	0	last	wholeT_0.5	J1:DiP, J1:DiP-A, J1:OT, J1:OT-A, J5:DiP, J5:DiP-A, J5:OT, J5:OT-A, S1:DiP, S1:DiP-A, S1:OT, S1:OT-A, S5:DiP, S5:DiP-A, S5:OT, S5:OT-A
172	Yes	9	9-A	1000	McF_4	0	unif	singleC	J1:DiP, J1:DiP-A, J1:OT, J1:OT-A, S1:DiP, S1:DiP-A, S1:OT, S1:OT-A, S5:DiP, S5:DiP-A, S5:OT, S5:OT-A
173	Yes	9	9-A	1000	McF_4	0	unif	wholeT_0.01	J1:DiP, J1:DiP-A, J1:OT, J1:OT-A, S1:DiP-A, S5:DiP, S5:DiP-A, S5:OT, S5:OT-A
174	Yes	9	9-A	1000	McF_4	0	unif	wholeT_0.5	J1:DiP, J1:DiP-A, J1:OT, J1:OT-A, S1:DiP, S1:DiP-A, S1:OT, S1:OT-A, S5:DiP, S5:DiP-A, S5:OT, S5:OT-A
175	Yes	9	9-A	1000	McF_4	Inf	last	singleC	J1:DiP, J1:DiP-A, J1:OT, J1:OT-A, J5:DiP, J5:DiP-A, J5:OT, J5:OT-A, S1:OT, S1:OT-A, S5:DiP, S5:DiP-A, S5:OT, S5:OT-A
176	Yes	9	9-A	1000	McF_4	Inf	last	wholeT_0.01	J1:DiP, J1:OT, J5:DiP, J5:OT, S1:DiP, S5:DiP, S5:OT
177	Yes	9	9-A	1000	McF_4	Inf	last	wholeT_0.5	J1:DiP, J1:DiP-A, J1:OT, J1:OT-A, J5:DiP, J5:DiP-A, J5:OT, J5:OT-A, S1:DiP, S1:DiP-A, S1:OT, S1:OT-A, S5:DiP, S5:DiP-A, S5:OT, S5:OT-A
178	Yes	9	9-A	1000	McF_4	Inf	unif	singleC	J1:DiP, J1:DiP-A, J1:OT, J1:OT-A, S1:OT, S1:OT-A, S5:DiP, S5:DiP-A, S5:OT, S5:OT-A
179	Yes	9	9-A	1000	McF_4	Inf	unif	wholeT_0.01	J1:DiP, J1:DiP-A, J1:OT, J1:OT-A, S5:DiP, S5:DiP-A, S5:OT, S5:OT-A

Table 14: *(continued)*

	Conjunction	Drivers	Tree	S.Size	Model	sh	S.Time	S.Type	Best method(s)
180	Yes	9	9-A	1000	McF_4	Inf	unif	wholeT_0.5	J1:DiP, J1:DiP-A, J1:OT, J1:OT-A, S1:DiP, S1:DiP-A, S1:OT, S1:OT-A, S5:CBN-A, S5:DiP, S5:DiP-A, S5:OT, S5:OT-A
181	Yes	9	9-A	1000	McF_6	0	last	singleC	J1:DiP, J1:OT, J5:DiP, J5:OT, S5:DiP, S5:OT
182	Yes	9	9-A	1000	McF_6	0	last	wholeT_0.01	J1:DiP, J1:OT, J5:DiP, J5:OT, S5:OT
183	Yes	9	9-A	1000	McF_6	0	last	wholeT_0.5	J1:DiP, J1:OT, J5:DiP, J5:OT, S1:DiP, S5:DiP, S5:OT
184	Yes	9	9-A	1000	McF_6	0	unif	singleC	J1:CBN, J1:CBN-A, J1:DiP, J1:DiP-A, J1:OT, J1:OT-A, J5:CBN, J5:CBN-A, J5:DiP, J5:DiP-A, J5:OT, J5:OT-A, S1:DiP, S1:DiP-A, S1:OT, S1:OT-A, S5:CBN, S5:CBN-A, S5:DiP, S5:DiP-A, S5:OT, S5:OT-A
185	Yes	9	9-A	1000	McF_6	0	unif	wholeT_0.01	J1:CBN, J1:CBN-A, J1:DiP, J1:DiP-A, J1:OT, J1:OT-A, J5:CBN, J5:CBN-A, J5:DiP, J5:DiP-A, J5:OT, J5:OT-A, S1:DiP, S5:CBN-A, S5:DiP, S5:OT, S5:OT-A
186	Yes	9	9-A	1000	McF_6	0	unif	wholeT_0.5	J1:CBN, J1:CBN-A, J1:DiP, J1:DiP-A, J1:OT, J1:OT-A, J5:CBN, J5:CBN-A, J5:DiP, J5:DiP-A, J5:OT, J5:OT-A, S1:CBN-A, S1:DiP, S1:DiP-A, S1:OT, S1:OT-A, S5:CBN, S5:CBN-A, S5:DiP, S5:DiP-A, S5:OT, S5:OT-A
187	Yes	9	9-A	1000	McF_6	Inf	last	singleC	J1:DiP, J1:OT, J5:DiP, J5:OT, S1:DiP, S5:DiP, S5:OT
188	Yes	9	9-A	1000	McF_6	Inf	last	wholeT_0.01	J1:DiP, J1:OT, J5:DiP, J5:OT, S5:OT
189	Yes	9	9-A	1000	McF_6	Inf	last	wholeT_0.5	J1:DiP, J1:OT, J5:DiP, J5:OT, S1:DiP, S5:DiP, S5:OT
190	Yes	9	9-A	1000	McF_6	Inf	unif	singleC	J1:CBN, J1:CBN-A, J1:DiP, J1:DiP-A, J1:OT, J1:OT-A, J5:CBN, J5:CBN-A, J5:DiP, J5:DiP-A, J5:OT, J5:OT-A, S1:DiP, S1:DiP-A, S1:OT, S1:OT-A, S5:CBN, S5:CBN-A, S5:DiP, S5:DiP-A, S5:OT, S5:OT-A

Table 14: *(continued)*

	Conjunction	Drivers	Tree	S.Size	Model	sh	S.Time	S.Type	Best method(s)
191	Yes	9	9-A	1000	McF_6	Inf	unif	wholeT_0.01	J1:CBN, J1:CBN-A, J1:DiP, J1:DiP-A, J1:OT, J1:OT-A, J5:CBN, J5:CBN-A, J5:DiP, J5:DiP-A, J5:OT, J5:OT-A, S1:DiP, S1:DiP-A, S5:CBN, S5:CBN-A, S5:DiP, S5:DiP-A, S5:OT, S5:OT-A
192	Yes	9	9-A	1000	McF_6	Inf	unif	wholeT_0.5	J1:CBN, J1:DiP, J1:DiP-A, J1:OT, J1:OT-A, J5:CBN, J5:CBN-A, J5:DiP, J5:DiP-A, J5:OT, J5:OT-A, S1:DiP, S1:DiP-A, S1:OT, S1:OT-A, S5:CBN, S5:CBN-A, S5:DiP, S5:DiP-A, S5:OT-A
193	Yes	9	9-A	200	Bozic	0	last	singleC	S1:OT, S1:OT-A
194	Yes	9	9-A	200	Bozic	0	last	wholeT_0.01	J1:OT, J1:OT-A, J5:OT, J5:OT-A, S5:OT, S5:OT-A
195	Yes	9	9-A	200	Bozic	0	last	wholeT_0.5	S1:OT, S1:OT-A
196	Yes	9	9-A	200	Bozic	0	unif	singleC	J1:CBN, J1:CBN-A, S1:CBN, S1:CBN-A, S5:CBN, S5:CBN-A
197	Yes	9	9-A	200	Bozic	0	unif	wholeT_0.01	S1:DiP, S1:DiP-A
198	Yes	9	9-A	200	Bozic	0	unif	wholeT_0.5	J1:CBN, J1:CBN-A, S1:CBN, S1:CBN-A, S5:CBN, S5:CBN-A
199	Yes	9	9-A	200	Bozic	Inf	last	singleC	J5:OT, J5:OT-A, S5:OT
200	Yes	9	9-A	200	Bozic	Inf	last	wholeT_0.01	J5:CBN-A, J5:OT, J5:OT-A
201	Yes	9	9-A	200	Bozic	Inf	last	wholeT_0.5	J5:OT, J5:OT-A, S5:OT-A
202	Yes	9	9-A	200	Bozic	Inf	unif	singleC	S1:CBN, S1:CBN-A, S1:OT, S1:OT-A
203	Yes	9	9-A	200	Bozic	Inf	unif	wholeT_0.01	S5:OT, S5:OT-A
204	Yes	9	9-A	200	Bozic	Inf	unif	wholeT_0.5	S1:OT, S1:OT-A
205	Yes	9	9-A	200	exp	0	last	singleC	S1:CBN
206	Yes	9	9-A	200	exp	0	last	wholeT_0.01	S1:OT, S1:OT-A
207	Yes	9	9-A	200	exp	0	last	wholeT_0.5	J1:CBN, J1:CBN-A, S1:CBN, S1:CBN-A, S5:CBN, S5:CBN-A
208	Yes	9	9-A	200	exp	0	unif	singleC	S1:CBN, S1:CBN-A, S5:CBN, S5:CBN-A
209	Yes	9	9-A	200	exp	0	unif	wholeT_0.01	S1:OT, S1:OT-A
210	Yes	9	9-A	200	exp	0	unif	wholeT_0.5	S1:CBN, S1:CBN-A, S5:CBN, S5:CBN-A
211	Yes	9	9-A	200	exp	Inf	last	singleC	J1:OT, J1:OT-A, S5:OT, S5:OT-A
212	Yes	9	9-A	200	exp	Inf	last	wholeT_0.01	J5:OT, J5:OT-A
213	Yes	9	9-A	200	exp	Inf	last	wholeT_0.5	J1:OT, J1:OT-A, S5:OT, S5:OT-A
214	Yes	9	9-A	200	exp	Inf	unif	singleC	S1:OT, S1:OT-A
215	Yes	9	9-A	200	exp	Inf	unif	wholeT_0.01	J1:CBN, J1:CBN-A, J1:OT, J1:OT-A, S5:CBN, S5:CBN-A
216	Yes	9	9-A	200	exp	Inf	unif	wholeT_0.5	S1:OT, S1:OT-A

Table 14: *(continued)*

	Conjunction	Drivers	Tree	S.Size	Model	sh	S.Time	S.Type	Best method(s)
217	Yes	9	9-A	200	McF_4	0	last	singleC	J1:DiP-A, J1:OT, J1:OT-A, J5:DiP-A, J5:OT, J5:OT-A, S1:DiP-A, S5:DiP-A, S5:OT, S5:OT-A
218	Yes	9	9-A	200	McF_4	0	last	wholeT_0.01	J1:DiP, J1:DiP-A, J1:OT, J1:OT-A, J5:DiP, J5:DiP-A, J5:OT, J5:OT-A, S1:DiP, S1:DiP-A, S5:DiP, S5:DiP-A, S5:OT, S5:OT-A
219	Yes	9	9-A	200	McF_4	0	last	wholeT_0.5	J1:OT, J1:OT-A, J5:OT, J5:OT-A, S1:DiP-A, S5:DiP-A, S5:OT, S5:OT-A
220	Yes	9	9-A	200	McF_4	0	unif	singleC	J1:OT, J1:OT-A, S5:OT, S5:OT-A
221	Yes	9	9-A	200	McF_4	0	unif	wholeT_0.01	J1:OT, J1:OT-A, S1:DiP, S1:DiP-A, S5:CBN-A, S5:DiP, S5:DiP-A, S5:OT, S5:OT-A
222	Yes	9	9-A	200	McF_4	0	unif	wholeT_0.5	J1:OT, J1:OT-A, S5:OT, S5:OT-A
223	Yes	9	9-A	200	McF_4	Inf	last	singleC	J1:OT, J5:OT, J5:OT-A, S5:OT, S5:OT-A
224	Yes	9	9-A	200	McF_4	Inf	last	wholeT_0.01	J1:DiP-A, J5:OT, S5:DiP-A, S5:OT
225	Yes	9	9-A	200	McF_4	Inf	last	wholeT_0.5	J1:OT-A, J5:OT, J5:OT-A, S5:OT, S5:OT-A
226	Yes	9	9-A	200	McF_4	Inf	unif	singleC	J1:OT, J1:OT-A, S5:OT, S5:OT-A
227	Yes	9	9-A	200	McF_4	Inf	unif	wholeT_0.01	S5:OT, S5:OT-A
228	Yes	9	9-A	200	McF_4	Inf	unif	wholeT_0.5	J1:OT, J1:OT-A, S5:OT, S5:OT-A
229	Yes	9	9-A	200	McF_6	0	last	singleC	J1:OT, J5:OT, S1:DiP, S5:DiP, S5:OT
230	Yes	9	9-A	200	McF_6	0	last	wholeT_0.01	J1:DiP, J5:DiP, J5:OT, S1:DiP, S5:DiP, S5:OT
231	Yes	9	9-A	200	McF_6	0	last	wholeT_0.5	J1:OT, J5:OT, S5:OT
232	Yes	9	9-A	200	McF_6	0	unif	singleC	J1:CBN, J1:CBN-A, J1:DiP, J1:DiP-A, J1:OT, J1:OT-A, J5:CBN, J5:CBN-A, J5:DiP, J5:DiP-A, J5:OT, J5:OT-A, S1:DiP, S1:DiP-A, S5:CBN, S5:CBN-A, S5:DiP, S5:DiP-A, S5:OT, S5:OT-A
233	Yes	9	9-A	200	McF_6	0	unif	wholeT_0.01	J1:CBN, J1:CBN-A, J1:DiP, J1:DiP-A, J1:OT, J1:OT-A, J5:CBN, J5:DiP, J5:DiP-A, J5:OT, J5:OT-A, S1:DiP, S1:DiP-A, S5:DiP, S5:DiP-A, S5:OT, S5:OT-A

Table 14: *(continued)*

	Conjunction	Drivers	Tree	S.Size	Model	sh	S.Time	S.Type	Best method(s)
234	Yes	9	9-A	200	McF_6	0	unif	wholeT_0.5	J1:CBN, J1:CBN-A, J1:DiP, J1:DiP-A, J1:OT, J1:OT-A, J5:CBN, J5:CBN-A, J5:DiP, J5:DiP-A, J5:OT, J5:OT-A, S1:DiP, S1:DiP-A, S5:CBN, S5:CBN-A, S5:DiP, S5:DiP-A, S5:OT, S5:OT-A
235	Yes	9	9-A	200	McF_6	Inf	last	singleC	J1:OT, J5:OT, S5:OT
236	Yes	9	9-A	200	McF_6	Inf	last	wholeT_0.01	J5:OT
237	Yes	9	9-A	200	McF_6	Inf	last	wholeT_0.5	J1:OT, J5:OT, S5:OT
238	Yes	9	9-A	200	McF_6	Inf	unif	singleC	J1:CBN, J1:CBN-A, J1:DiP, J1:DiP-A, J1:OT, J1:OT-A, J5:CBN, J5:CBN-A, J5:DiP-A, J5:OT, J5:OT-A, S1:DiP, S1:DiP-A, S5:CBN, S5:CBN-A, S5:DiP, S5:DiP-A, S5:OT, S5:OT-A
239	Yes	9	9-A	200	McF_6	Inf	unif	wholeT_0.01	J1:CBN, J1:CBN-A, J1:DiP-A, J1:OT, J1:OT-A, J5:CBN, J5:CBN-A, J5:DiP-A, J5:OT, J5:OT-A, S1:DiP, S5:CBN, S5:CBN-A, S5:DiP, S5:DiP-A, S5:OT, S5:OT-A
240	Yes	9	9-A	200	McF_6	Inf	unif	wholeT_0.5	J1:CBN, J1:CBN-A, J1:DiP-A, J1:OT, J1:OT-A, J5:CBN, J5:CBN-A, J5:DiP-A, J5:OT, J5:OT-A, S1:DiP, S1:DiP-A, S5:CBN, S5:CBN-A, S5:DiP, S5:DiP-A, S5:OT, S5:OT-A
241	Yes	9	9-A	100	Bozic	0	last	singleC	S1:OT, S1:OT-A
242	Yes	9	9-A	100	Bozic	0	last	wholeT_0.01	J5:OT, J5:OT-A, S5:OT, S5:OT-A
243	Yes	9	9-A	100	Bozic	0	last	wholeT_0.5	S1:OT, S1:OT-A
244	Yes	9	9-A	100	Bozic	0	unif	singleC	S1:OT, S1:OT-A
245	Yes	9	9-A	100	Bozic	0	unif	wholeT_0.01	J1:CBN, J1:CBN-A, J1:OT, J1:OT-A, S1:OT, S1:OT-A, S5:CBN
246	Yes	9	9-A	100	Bozic	0	unif	wholeT_0.5	S1:CBN, S1:CBN-A, S1:OT, S1:OT-A
247	Yes	9	9-A	100	Bozic	Inf	last	singleC	S5:OT, S5:OT-A
248	Yes	9	9-A	100	Bozic	Inf	last	wholeT_0.01	J5:CBN-A, J5:OT, J5:OT-A
249	Yes	9	9-A	100	Bozic	Inf	last	wholeT_0.5	J5:OT, J5:OT-A
250	Yes	9	9-A	100	Bozic	Inf	unif	singleC	J1:CBN, J1:CBN-A, J1:OT, J1:OT-A, S1:OT, S1:OT-A
251	Yes	9	9-A	100	Bozic	Inf	unif	wholeT_0.01	S5:OT, S5:OT-A
252	Yes	9	9-A	100	Bozic	Inf	unif	wholeT_0.5	J1:CBN, J1:CBN-A, J1:OT, J1:OT-A, S1:OT, S1:OT-A
253	Yes	9	9-A	100	exp	0	last	singleC	S1:OT, S1:OT-A
254	Yes	9	9-A	100	exp	0	last	wholeT_0.01	S1:OT, S1:OT-A, S5:OT, S5:OT-A

Table 14: *(continued)*

	Conjunction	Drivers	Tree	S.Size	Model	sh	S.Time	S.Type	Best method(s)
255	Yes	9	9-A	100	exp	0	last	wholeT_0.5	S1:OT, S1:OT-A
256	Yes	9	9-A	100	exp	0	unif	singleC	S1:CBN, S1:CBN-A
257	Yes	9	9-A	100	exp	0	unif	wholeT_0.01	J1:CBN, J1:CBN-A, S1:OT, S1:OT-A
258	Yes	9	9-A	100	exp	0	unif	wholeT_0.5	J1:CBN, J1:CBN-A, S1:CBN, S1:CBN-A, S5:CBN-A
259	Yes	9	9-A	100	exp	Inf	last	singleC	S5:OT, S5:OT-A
260	Yes	9	9-A	100	exp	Inf	last	wholeT_0.01	J5:CBN-A, J5:OT, J5:OT-A
261	Yes	9	9-A	100	exp	Inf	last	wholeT_0.5	S5:OT, S5:OT-A
262	Yes	9	9-A	100	exp	Inf	unif	singleC	J1:CBN, J1:CBN-A, S1:OT, S1:OT-A
263	Yes	9	9-A	100	exp	Inf	unif	wholeT_0.01	J1:OT, J1:OT-A, S1:OT, S1:OT-A, S5:CBN, S5:CBN-A
264	Yes	9	9-A	100	exp	Inf	unif	wholeT_0.5	J1:CBN-A, S1:OT, S1:OT-A
265	Yes	9	9-A	100	McF_4	0	last	singleC	J5:OT, J5:OT-A, S5:OT, S5:OT-A
266	Yes	9	9-A	100	McF_4	0	last	wholeT_0.01	J1:DiP-A, J5:DiP, J5:DiP-A, J5:OT, J5:OT-A, S1:DiP, S1:DiP-A, S5:DiP, S5:DiP-A, S5:OT, S5:OT-A
267	Yes	9	9-A	100	McF_4	0	last	wholeT_0.5	J5:OT, J5:OT-A, S5:OT, S5:OT-A
268	Yes	9	9-A	100	McF_4	0	unif	singleC	S5:CBN-A, S5:OT, S5:OT-A
269	Yes	9	9-A	100	McF_4	0	unif	wholeT_0.01	J1:OT, S5:CBN-A, S5:OT, S5:OT-A
270	Yes	9	9-A	100	McF_4	0	unif	wholeT_0.5	J1:OT, J1:OT-A, S5:CBN-A, S5:OT, S5:OT-A
271	Yes	9	9-A	100	McF_4	Inf	last	singleC	J5:OT, J5:OT-A, S5:OT, S5:OT-A
272	Yes	9	9-A	100	McF_4	Inf	last	wholeT_0.01	J5:OT, S5:OT
273	Yes	9	9-A	100	McF_4	Inf	last	wholeT_0.5	J5:OT, J5:OT-A, S5:OT, S5:OT-A
274	Yes	9	9-A	100	McF_4	Inf	unif	singleC	J1:OT, J1:OT-A, S5:OT, S5:OT-A
275	Yes	9	9-A	100	McF_4	Inf	unif	wholeT_0.01	S5:OT, S5:OT-A
276	Yes	9	9-A	100	McF_4	Inf	unif	wholeT_0.5	J1:OT, J1:OT-A, S5:OT, S5:OT-A
277	Yes	9	9-A	100	McF_6	0	last	singleC	J5:OT, S5:OT
278	Yes	9	9-A	100	McF_6	0	last	wholeT_0.01	J5:OT, S5:OT
279	Yes	9	9-A	100	McF_6	0	last	wholeT_0.5	J5:OT, S5:OT
280	Yes	9	9-A	100	McF_6	0	unif	singleC	J1:CBN, J1:CBN-A, J1:OT, J5:CBN, J5:CBN-A, J5:OT, S5:CBN, S5:CBN-A, S5:OT, S5:OT-A
281	Yes	9	9-A	100	McF_6	0	unif	wholeT_0.01	J1:DiP, J1:DiP-A, J1:OT, J5:CBN, J5:CBN-A, J5:OT, J5:OT-A, S5:CBN, S5:CBN-A, S5:DiP, S5:DiP-A, S5:OT, S5:OT-A
282	Yes	9	9-A	100	McF_6	0	unif	wholeT_0.5	J1:CBN, J1:CBN-A, J1:OT, J1:OT-A, J5:CBN, J5:CBN-A, J5:OT, J5:OT-A, S5:CBN, S5:CBN-A, S5:OT, S5:OT-A

Table 14: *(continued)*

	Conjunction	Drivers	Tree	S.Size	Model	sh	S.Time	S.Type	Best method(s)
283	Yes	9	9-A	100	McF_6	Inf	last	singleC	J1:OT, J5:OT, S5:OT
284	Yes	9	9-A	100	McF_6	Inf	last	wholeT_0.01	J5:OT, S5:OT
285	Yes	9	9-A	100	McF_6	Inf	last	wholeT_0.5	J5:OT, S5:OT
286	Yes	9	9-A	100	McF_6	Inf	unif	singleC	J1:CBN-A, J5:CBN, J5:CBN-A, J5:OT, J5:OT-A, S5:CBN, S5:CBN-A, S5:OT
287	Yes	9	9-A	100	McF_6	Inf	unif	wholeT_0.01	J5:CBN-A, J5:OT, S5:CBN, S5:OT
288	Yes	9	9-A	100	McF_6	Inf	unif	wholeT_0.5	J1:CBN, J1:CBN-A, J1:OT, J1:OT-A, J5:CBN, J5:CBN-A, J5:OT, J5:OT-A, S5:CBN, S5:CBN-A, S5:OT, S5:OT-A
289	Yes	7	7-A	1000	Bozic	0	last	singleC	J1:CBN-A, J1:OT-A, J5:CBN-A, J5:OT-A, S1:OT-A, S5:OT-A
290	Yes	7	7-A	1000	Bozic	0	last	wholeT_0.01	J1:DiP-A, J1:OT-A, J5:DiP-A, J5:OT-A, S1:DiP-A, S1:OT-A, S5:DiP-A, S5:OT-A
291	Yes	7	7-A	1000	Bozic	0	last	wholeT_0.5	J1:CBN-A, J1:OT-A, J5:CBN-A, J5:OT-A, S1:OT-A, S5:CBN-A, S5:OT-A
292	Yes	7	7-A	1000	Bozic	0	unif	singleC	J1:CBN, J1:CBN-A, J1:OT, J1:OT-A, S1:CBN, S1:CBN-A, S1:OT, S1:OT-A
293	Yes	7	7-A	1000	Bozic	0	unif	wholeT_0.01	J5:CBN, J5:CBN-A, J5:DiP-A, J5:OT, J5:OT-A
294	Yes	7	7-A	1000	Bozic	0	unif	wholeT_0.5	NA
295	Yes	7	7-A	1000	Bozic	Inf	last	singleC	J1:DiP-A, J1:OT-A, J5:DiP-A, J5:OT-A, S1:DiP-A, S5:DiP-A, S5:OT-A
296	Yes	7	7-A	1000	Bozic	Inf	last	wholeT_0.01	J5:CBN-A
297	Yes	7	7-A	1000	Bozic	Inf	last	wholeT_0.5	J1:DiP-A, J1:OT-A, J5:DiP-A, J5:OT-A, S1:DiP-A, S5:DiP-A, S5:OT-A
298	Yes	7	7-A	1000	Bozic	Inf	unif	singleC	J1:CBN, J1:CBN-A, J5:CBN, J5:CBN-A, J5:OT, J5:OT-A, S1:CBN, S1:CBN-A, S5:CBN, S5:CBN-A, S5:OT
299	Yes	7	7-A	1000	Bozic	Inf	unif	wholeT_0.01	J5:CBN, J5:CBN-A, S5:CBN, S5:CBN-A
300	Yes	7	7-A	1000	Bozic	Inf	unif	wholeT_0.5	J1:CBN, J1:CBN-A, S1:CBN, S1:CBN-A
301	Yes	7	7-A	1000	exp	0	last	singleC	J1:CBN-A, J1:OT-A, S1:OT-A
302	Yes	7	7-A	1000	exp	0	last	wholeT_0.01	J1:CBN, J1:CBN-A, J1:OT-A, J5:CBN-A, J5:OT-A, S1:CBN, S1:CBN-A, S1:OT-A, S5:CBN, S5:OT-A
303	Yes	7	7-A	1000	exp	0	last	wholeT_0.5	J1:CBN-A, J1:OT-A, S1:OT-A
304	Yes	7	7-A	1000	exp	0	unif	singleC	NA
305	Yes	7	7-A	1000	exp	0	unif	wholeT_0.01	J1:CBN
306	Yes	7	7-A	1000	exp	0	unif	wholeT_0.5	NA
307	Yes	7	7-A	1000	exp	Inf	last	singleC	J1:OT-A, J5:CBN-A, J5:OT-A, S5:OT-A

Table 14: *(continued)*

	Conjunction	Drivers	Tree	S.Size	Model	sh	S.Time	S.Type	Best method(s)
308	Yes	7	7-A	1000	exp	Inf	last	wholeT_0.01	J1:CBN-A, J5:CBN-A, J5:DiP-A, J5:OT-A, S5:CBN-A, S5:DiP-A, S5:OT-A
309	Yes	7	7-A	1000	exp	Inf	last	wholeT_0.5	J1:OT-A, J5:CBN-A, J5:OT-A, S1:OT-A, S5:OT-A
310	Yes	7	7-A	1000	exp	Inf	unif	singleC	J1:CBN, J1:CBN-A, J1:OT, J1:OT-A, S1:CBN-A
311	Yes	7	7-A	1000	exp	Inf	unif	wholeT_0.01	J1:CBN, J1:CBN-A, J5:CBN, J5:CBN-A, S5:CBN, S5:CBN-A
312	Yes	7	7-A	1000	exp	Inf	unif	wholeT_0.5	J1:CBN, J1:CBN-A, J1:OT, J1:OT-A, S1:CBN, S1:CBN-A
313	Yes	7	7-A	1000	McF_4	0	last	singleC	J1:DiP-A, J1:OT-A, J5:DiP-A, J5:OT-A, S1:DiP-A, S1:OT-A, S5:DiP-A, S5:OT-A
314	Yes	7	7-A	1000	McF_4	0	last	wholeT_0.01	J1:DiP-A, J1:OT-A, J5:DiP-A, J5:OT-A, S5:OT, S5:OT-A
315	Yes	7	7-A	1000	McF_4	0	last	wholeT_0.5	J1:DiP-A, J1:OT-A, J5:DiP-A, J5:OT-A, S1:DiP-A, S1:OT-A, S5:DiP-A, S5:OT-A
316	Yes	7	7-A	1000	McF_4	0	unif	singleC	J1:CBN-A, J1:DiP, J1:DiP-A, J1:OT, J1:OT-A, J5:CBN-A, J5:DiP, J5:DiP-A, J5:OT, J5:OT-A, S1:CBN-A, S1:DiP, S1:DiP-A, S1:OT, S1:OT-A, S5:CBN-A, S5:DiP, S5:DiP-A, S5:OT, S5:OT-A
317	Yes	7	7-A	1000	McF_4	0	unif	wholeT_0.01	J1:CBN, J1:DiP, J1:DiP-A, J1:OT, J1:OT-A, J5:CBN, J5:DiP, J5:DiP-A, J5:OT, J5:OT-A, S1:DiP, S1:DiP-A, S5:CBN, S5:DiP, S5:DiP-A, S5:OT, S5:OT-A
318	Yes	7	7-A	1000	McF_4	0	unif	wholeT_0.5	J1:CBN-A, J1:DiP, J1:DiP-A, J1:OT, J1:OT-A, J5:CBN-A, J5:DiP, J5:DiP-A, J5:OT, J5:OT-A, S1:CBN-A, S1:DiP, S1:DiP-A, S1:OT, S1:OT-A, S5:CBN-A, S5:DiP, S5:DiP-A, S5:OT, S5:OT-A
319	Yes	7	7-A	1000	McF_4	Inf	last	singleC	J1:DiP-A, J1:OT-A, J5:DiP-A, J5:OT-A, S1:DiP-A, S5:DiP-A, S5:OT-A
320	Yes	7	7-A	1000	McF_4	Inf	last	wholeT_0.01	J1:DiP-A, J5:DiP-A, J5:OT-A, S5:DiP-A, S5:OT-A
321	Yes	7	7-A	1000	McF_4	Inf	last	wholeT_0.5	J1:DiP-A, J1:OT-A, J5:DiP-A, J5:OT-A, S1:DiP-A, S1:OT-A, S5:DiP-A, S5:OT-A
322	Yes	7	7-A	1000	McF_4	Inf	unif	singleC	J1:OT, J1:OT-A, J5:OT, J5:OT-A, S1:DiP-A, S1:OT, S1:OT-A, S5:OT, S5:OT-A

Table 14: *(continued)*

	Conjunction	Drivers	Tree	S.Size	Model	sh	S.Time	S.Type	Best method(s)
323	Yes	7	7-A	1000	McF_4	Inf	unif	wholeT_0.01	J1:DiP-A, J1:OT, J1:OT-A, J5:DiP-A, J5:OT, J5:OT-A, S1:DiP-A, S5:DiP-A, S5:OT, S5:OT-A
324	Yes	7	7-A	1000	McF_4	Inf	unif	wholeT_0.5	J1:DiP-A, J1:OT, J1:OT-A, J5:DiP-A, J5:OT, J5:OT-A, S1:DiP-A, S1:OT, S1:OT-A, S5:DiP-A, S5:OT, S5:OT-A
325	Yes	7	7-A	1000	McF_6	0	last	singleC	J1:DiP, J1:OT, J1:OT-A, J5:DiP, J5:OT, J5:OT-A, S1:DiP, S5:DiP, S5:OT, S5:OT-A
326	Yes	7	7-A	1000	McF_6	0	last	wholeT_0.01	J1:DiP, J1:OT, J1:OT-A, J5:DiP, J5:OT, J5:OT-A, S1:DiP, S5:DiP, S5:OT, S5:OT-A
327	Yes	7	7-A	1000	McF_6	0	last	wholeT_0.5	J1:DiP, J1:OT, J1:OT-A, J5:DiP, J5:OT, J5:OT-A, S1:DiP, S5:DiP, S5:OT, S5:OT-A
328	Yes	7	7-A	1000	McF_6	0	unif	singleC	none
329	Yes	7	7-A	1000	McF_6	0	unif	wholeT_0.01	J1:CBN, J1:CBN-A, J1:DiP, J1:DiP-A, J1:OT, J1:OT-A, J5:CBN-A, J5:DiP, J5:DiP-A, J5:OT, J5:OT-A, S5:CBN, S5:CBN-A, S5:DiP, S5:DiP-A, S5:OT, S5:OT-A
330	Yes	7	7-A	1000	McF_6	0	unif	wholeT_0.5	none
331	Yes	7	7-A	1000	McF_6	Inf	last	singleC	J1:DiP, J1:OT, J1:OT-A, J5:DiP, J5:OT, J5:OT-A, S1:DiP, S5:DiP, S5:DiP-A, S5:OT, S5:OT-A
332	Yes	7	7-A	1000	McF_6	Inf	last	wholeT_0.01	J5:OT, J5:OT-A, S5:OT, S5:OT-A
333	Yes	7	7-A	1000	McF_6	Inf	last	wholeT_0.5	J1:DiP, J1:OT, J1:OT-A, J5:DiP, J5:OT, J5:OT-A, S1:DiP, S5:DiP, S5:OT, S5:OT-A
334	Yes	7	7-A	1000	McF_6	Inf	unif	singleC	J1:CBN, J1:CBN-A, J1:DiP, J1:DiP-A, J1:OT, J1:OT-A, J5:CBN-A, J5:DiP-A, J5:OT, J5:OT-A, S1:CBN, S1:CBN-A, S1:DiP, S1:DiP-A, S1:OT, S1:OT-A, S5:CBN, S5:CBN-A, S5:DiP, S5:DiP-A, S5:OT, S5:OT-A
335	Yes	7	7-A	1000	McF_6	Inf	unif	wholeT_0.01	J1:CBN, J1:CBN-A, J1:DiP, J1:DiP-A, J1:OT, J1:OT-A, J5:DiP-A, J5:OT, J5:OT-A, S5:CBN, S5:CBN-A, S5:DiP, S5:DiP-A, S5:OT, S5:OT-A

Table 14: *(continued)*

	Conjunction	Drivers	Tree	S.Size	Model	sh	S.Time	S.Type	Best method(s)
336	Yes	7	7-A	1000	McF_6	Inf	unif	wholeT_0.5	J1:CBN, J1:CBN-A, J1:DiP, J1:DiP-A, J1:OT, J1:OT-A, J5:CBN-A, J5:DiP-A, J5:OT, J5:OT-A, S1:CBN-A, S1:DiP, S1:DiP-A, S1:OT, S1:OT-A, S5:CBN, S5:CBN-A, S5:DiP, S5:DiP-A, S5:OT, S5:OT-A
337	Yes	7	7-A	200	Bozic	0	last	singleC	J1:CBN-A, J1:OT-A, J5:CBN-A, J5:OT-A, S1:OT-A, S5:OT-A
338	Yes	7	7-A	200	Bozic	0	last	wholeT_0.01	J1:OT-A, J5:OT-A, S5:OT-A
339	Yes	7	7-A	200	Bozic	0	last	wholeT_0.5	J1:CBN-A, J1:OT-A, J5:CBN-A, J5:OT-A, S5:CBN-A, S5:OT-A
340	Yes	7	7-A	200	Bozic	0	unif	singleC	J1:CBN, J1:CBN-A, J1:OT, J1:OT-A, S1:CBN, S1:OT, S1:OT-A
341	Yes	7	7-A	200	Bozic	0	unif	wholeT_0.01	J5:CBN, J5:OT, J5:OT-A
342	Yes	7	7-A	200	Bozic	0	unif	wholeT_0.5	J1:CBN, J1:CBN-A, J1:OT, J1:OT-A, S1:CBN, S1:CBN-A, S1:OT, S1:OT-A
343	Yes	7	7-A	200	Bozic	Inf	last	singleC	J5:CBN, J5:OT-A, S5:OT-A
344	Yes	7	7-A	200	Bozic	Inf	last	wholeT_0.01	J5:CBN-A, J5:OT-A
345	Yes	7	7-A	200	Bozic	Inf	last	wholeT_0.5	J5:OT-A, S5:OT-A
346	Yes	7	7-A	200	Bozic	Inf	unif	singleC	J1:CBN, J1:CBN-A, J5:CBN, J5:CBN-A, J5:OT, J5:OT-A, S5:CBN, S5:CBN-A, S5:OT-A
347	Yes	7	7-A	200	Bozic	Inf	unif	wholeT_0.01	J5:CBN, J5:CBN-A, J5:OT-A, S5:CBN, S5:CBN-A, S5:OT-A
348	Yes	7	7-A	200	Bozic	Inf	unif	wholeT_0.5	J1:CBN, J1:CBN-A
349	Yes	7	7-A	200	exp	0	last	singleC	J1:CBN-A, J1:OT-A, S1:OT-A
350	Yes	7	7-A	200	exp	0	last	wholeT_0.01	J1:CBN, J1:CBN-A, J1:OT-A, J5:CBN-A, J5:OT-A, S1:CBN, S1:CBN-A, S1:OT-A, S5:CBN, S5:OT-A
351	Yes	7	7-A	200	exp	0	last	wholeT_0.5	J1:OT-A, S1:OT-A
352	Yes	7	7-A	200	exp	0	unif	singleC	NA
353	Yes	7	7-A	200	exp	0	unif	wholeT_0.01	J1:CBN, J1:CBN-A, J1:OT, J1:OT-A, S1:OT
354	Yes	7	7-A	200	exp	0	unif	wholeT_0.5	NA
355	Yes	7	7-A	200	exp	Inf	last	singleC	J1:OT-A, J5:CBN-A, J5:OT-A, S5:OT-A
356	Yes	7	7-A	200	exp	Inf	last	wholeT_0.01	J5:CBN-A, J5:OT-A, S5:CBN-A
357	Yes	7	7-A	200	exp	Inf	last	wholeT_0.5	J1:OT-A, J5:CBN-A, J5:OT-A, S5:OT-A
358	Yes	7	7-A	200	exp	Inf	unif	singleC	J1:CBN, J1:CBN-A, J1:OT, J1:OT-A
359	Yes	7	7-A	200	exp	Inf	unif	wholeT_0.01	J5:CBN, J5:CBN-A, J5:OT, J5:OT-A, S5:CBN, S5:CBN-A

Table 14: *(continued)*

	Conjunction	Drivers	Tree	S.Size	Model	sh	S.Time	S.Type	Best method(s)
360	Yes	7	7-A	200	exp	Inf	unif	wholeT_0.5	J1:CBN, J1:CBN-A, J1:OT, J1:OT-A
361	Yes	7	7-A	200	McF_4	0	last	singleC	J1:OT-A, J5:OT-A, S5:OT-A
362	Yes	7	7-A	200	McF_4	0	last	wholeT_0.01	J5:OT-A, S5:OT-A
363	Yes	7	7-A	200	McF_4	0	last	wholeT_0.5	J1:OT-A, J5:OT-A, S5:OT-A
364	Yes	7	7-A	200	McF_4	0	unif	singleC	J1:CBN, J1:CBN-A, J1:OT, J1:OT-A, J5:CBN, J5:CBN-A, J5:OT, J5:OT-A, S5:CBN, S5:CBN-A, S5:OT, S5:OT-A
365	Yes	7	7-A	200	McF_4	0	unif	wholeT_0.01	J1:CBN, J1:CBN-A, J1:DiP-A, J1:OT, J1:OT-A, J5:CBN, J5:DiP-A, J5:OT, J5:OT-A, S1:DiP-A, S5:CBN, S5:CBN-A, S5:DiP-A, S5:OT, S5:OT-A
366	Yes	7	7-A	200	McF_4	0	unif	wholeT_0.5	J1:CBN-A, J1:OT, J1:OT-A, J5:CBN, J5:CBN-A, J5:OT, J5:OT-A, S5:CBN-A, S5:OT, S5:OT-A
367	Yes	7	7-A	200	McF_4	Inf	last	singleC	J1:OT-A, J5:OT-A, S5:OT-A
368	Yes	7	7-A	200	McF_4	Inf	last	wholeT_0.01	J5:OT-A, S5:OT-A
369	Yes	7	7-A	200	McF_4	Inf	last	wholeT_0.5	J1:OT-A, J5:OT-A, S5:OT-A
370	Yes	7	7-A	200	McF_4	Inf	unif	singleC	J1:OT, J1:OT-A, J5:CBN-A, J5:OT, J5:OT-A, S5:OT, S5:OT-A
371	Yes	7	7-A	200	McF_4	Inf	unif	wholeT_0.01	J1:OT, J1:OT-A, J5:OT, J5:OT-A, S5:OT, S5:OT-A
372	Yes	7	7-A	200	McF_4	Inf	unif	wholeT_0.5	J1:OT, J1:OT-A, J5:OT, J5:OT-A, S5:OT, S5:OT-A
373	Yes	7	7-A	200	McF_6	0	last	singleC	J1:OT, J5:OT, J5:OT-A, S5:OT, S5:OT-A
374	Yes	7	7-A	200	McF_6	0	last	wholeT_0.01	J5:OT, J5:OT-A, S5:OT, S5:OT-A
375	Yes	7	7-A	200	McF_6	0	last	wholeT_0.5	J1:OT-A, J5:OT, J5:OT-A, S5:OT, S5:OT-A
376	Yes	7	7-A	200	McF_6	0	unif	singleC	J1:CBN, J1:CBN-A, J1:DiP-A, J1:OT, J1:OT-A, J5:CBN, J5:CBN-A, S1:DiP-A, S5:CBN, S5:CBN-A, S5:DiP-A, S5:OT
377	Yes	7	7-A	200	McF_6	0	unif	wholeT_0.01	J1:CBN, J1:CBN-A, J1:DiP-A, J1:OT, J1:OT-A, J5:CBN, J5:CBN-A, J5:DiP-A, J5:OT, J5:OT-A, S1:DiP, S1:DiP-A, S5:CBN, S5:CBN-A, S5:DiP-A, S5:OT, S5:OT-A
378	Yes	7	7-A	200	McF_6	0	unif	wholeT_0.5	J1:CBN, J1:CBN-A, J5:CBN, J5:CBN-A, S5:CBN, S5:CBN-A
379	Yes	7	7-A	200	McF_6	Inf	last	singleC	J1:DiP-A, J5:DiP-A, J5:OT, J5:OT-A, S5:DiP-A, S5:OT, S5:OT-A

Table 14: *(continued)*

	Conjunction	Drivers	Tree	S.Size	Model	sh	S.Time	S.Type	Best method(s)
380	Yes	7	7-A	200	McF_6	Inf	last	wholeT_0.01	J5:OT, J5:OT-A, S5:OT-A
381	Yes	7	7-A	200	McF_6	Inf	last	wholeT_0.5	J1:DiP-A, J1:OT, J5:DiP-A, J5:OT, J5:OT-A, S5:DiP-A, S5:OT, S5:OT-A
382	Yes	7	7-A	200	McF_6	Inf	unif	singleC	J1:CBN, J1:CBN-A, J1:OT, J1:OT-A, J5:CBN-A, J5:OT-A, S5:CBN, S5:CBN-A, S5:OT, S5:OT-A
383	Yes	7	7-A	200	McF_6	Inf	unif	wholeT_0.01	J1:CBN, J1:CBN-A, J5:CBN-A, J5:OT-A, S5:CBN, S5:CBN-A, S5:OT-A
384	Yes	7	7-A	200	McF_6	Inf	unif	wholeT_0.5	J1:CBN, J1:CBN-A, J5:CBN-A, S5:CBN, S5:CBN-A
385	Yes	7	7-A	100	Bozic	0	last	singleC	J1:CBN-A, J5:CBN-A, J5:OT-A, S5:CBN, S5:OT-A
386	Yes	7	7-A	100	Bozic	0	last	wholeT_0.01	J5:OT-A, S5:OT-A
387	Yes	7	7-A	100	Bozic	0	last	wholeT_0.5	J5:OT-A, S5:CBN-A, S5:OT-A
388	Yes	7	7-A	100	Bozic	0	unif	singleC	J1:CBN, J1:CBN-A, J1:OT, J1:OT-A, S1:OT, S1:OT-A
389	Yes	7	7-A	100	Bozic	0	unif	wholeT_0.01	J1:CBN-A, J5:CBN, J5:CBN-A, J5:OT, J5:OT-A, S5:CBN, S5:CBN-A
390	Yes	7	7-A	100	Bozic	0	unif	wholeT_0.5	J1:CBN, J1:CBN-A, J1:OT, J1:OT-A, S1:OT, S1:OT-A
391	Yes	7	7-A	100	Bozic	Inf	last	singleC	J5:CBN, J5:OT-A
392	Yes	7	7-A	100	Bozic	Inf	last	wholeT_0.01	J5:CBN-A, J5:OT-A
393	Yes	7	7-A	100	Bozic	Inf	last	wholeT_0.5	J5:OT-A
394	Yes	7	7-A	100	Bozic	Inf	unif	singleC	J5:CBN, J5:CBN-A, J5:OT, J5:OT-A, S5:CBN, S5:CBN-A
395	Yes	7	7-A	100	Bozic	Inf	unif	wholeT_0.01	J5:CBN, J5:CBN-A, J5:OT, J5:OT-A, S5:CBN-A, S5:OT, S5:OT-A
396	Yes	7	7-A	100	Bozic	Inf	unif	wholeT_0.5	J5:CBN, J5:CBN-A, J5:OT, J5:OT-A, S5:CBN, S5:CBN-A
397	Yes	7	7-A	100	exp	0	last	singleC	J1:OT-A, S1:OT-A
398	Yes	7	7-A	100	exp	0	last	wholeT_0.01	J1:CBN, J1:CBN-A, J5:CBN-A, J5:OT-A, S1:CBN, S5:CBN
399	Yes	7	7-A	100	exp	0	last	wholeT_0.5	J1:OT-A, S1:OT-A
400	Yes	7	7-A	100	exp	0	unif	singleC	J1:OT, J1:OT-A, S1:CBN, S1:CBN-A, S1:OT, S1:OT-A
401	Yes	7	7-A	100	exp	0	unif	wholeT_0.01	J1:CBN, J1:CBN-A, J1:OT, J1:OT-A, S1:CBN, S1:CBN-A, S1:OT, S1:OT-A
402	Yes	7	7-A	100	exp	0	unif	wholeT_0.5	NA
403	Yes	7	7-A	100	exp	Inf	last	singleC	J1:OT-A, J5:OT-A, S5:OT-A
404	Yes	7	7-A	100	exp	Inf	last	wholeT_0.01	J5:CBN, J5:CBN-A, J5:OT-A

Table 14: *(continued)*

	Conjunction	Drivers	Tree	S.Size	Model	sh	S.Time	S.Type	Best method(s)
405	Yes	7	7-A	100	exp	Inf	last	wholeT_0.5	J1:CBN, J1:OT-A, J5:CBN-A, J5:OT-A, S5:OT-A
406	Yes	7	7-A	100	exp	Inf	unif	singleC	J1:CBN, J1:CBN-A, J1:OT, J1:OT-A
407	Yes	7	7-A	100	exp	Inf	unif	wholeT_0.01	J5:CBN, J5:CBN-A, J5:OT, J5:OT-A, S5:CBN, S5:CBN-A, S5:OT, S5:OT-A
408	Yes	7	7-A	100	exp	Inf	unif	wholeT_0.5	J1:CBN, J1:CBN-A, J1:OT, J1:OT-A, S1:OT, S1:OT-A
409	Yes	7	7-A	100	McF_4	0	last	singleC	J5:OT-A, S5:OT-A
410	Yes	7	7-A	100	McF_4	0	last	wholeT_0.01	J5:OT-A, S5:OT-A
411	Yes	7	7-A	100	McF_4	0	last	wholeT_0.5	J1:OT-A, J5:OT-A, S5:OT-A
412	Yes	7	7-A	100	McF_4	0	unif	singleC	J1:CBN, J1:CBN-A, J1:OT, J1:OT-A, J5:CBN, J5:CBN-A, J5:OT, J5:OT-A, S5:CBN-A, S5:OT, S5:OT-A
413	Yes	7	7-A	100	McF_4	0	unif	wholeT_0.01	J1:CBN-A, J1:OT, J1:OT-A, J5:CBN, J5:CBN-A, J5:OT, J5:OT-A, S5:CBN-A, S5:OT, S5:OT-A
414	Yes	7	7-A	100	McF_4	0	unif	wholeT_0.5	J1:CBN-A, J1:OT, J1:OT-A, J5:CBN, J5:CBN-A, J5:OT, J5:OT-A, S5:CBN, S5:CBN-A, S5:OT, S5:OT-A
415	Yes	7	7-A	100	McF_4	Inf	last	singleC	J5:OT-A, S5:OT-A
416	Yes	7	7-A	100	McF_4	Inf	last	wholeT_0.01	J5:OT-A, S5:OT-A
417	Yes	7	7-A	100	McF_4	Inf	last	wholeT_0.5	J5:OT-A, S5:OT-A
418	Yes	7	7-A	100	McF_4	Inf	unif	singleC	J1:OT, J1:OT-A, J5:OT, J5:OT-A, S5:CBN-A, S5:OT, S5:OT-A
419	Yes	7	7-A	100	McF_4	Inf	unif	wholeT_0.01	J1:CBN-A, J1:OT, J1:OT-A, J5:CBN-A, J5:OT-A, S5:CBN-A, S5:OT, S5:OT-A
420	Yes	7	7-A	100	McF_4	Inf	unif	wholeT_0.5	J1:OT-A, J5:CBN-A, J5:OT, J5:OT-A, S5:OT
421	Yes	7	7-A	100	McF_6	0	last	singleC	J5:OT, J5:OT-A, S5:OT, S5:OT-A
422	Yes	7	7-A	100	McF_6	0	last	wholeT_0.01	J5:OT, J5:OT-A, S5:OT, S5:OT-A
423	Yes	7	7-A	100	McF_6	0	last	wholeT_0.5	J5:OT, J5:OT-A, S5:OT, S5:OT-A
424	Yes	7	7-A	100	McF_6	0	unif	singleC	J1:CBN, J1:CBN-A, J5:CBN, J5:CBN-A, S5:CBN, S5:CBN-A
425	Yes	7	7-A	100	McF_6	0	unif	wholeT_0.01	J1:DiP-A, J5:CBN, J5:CBN-A, J5:DiP-A, J5:OT, J5:OT-A, S1:DiP-A, S5:CBN, S5:CBN-A, S5:DiP-A
426	Yes	7	7-A	100	McF_6	0	unif	wholeT_0.5	J1:CBN, J1:CBN-A, J5:CBN, J5:CBN-A, S5:CBN, S5:CBN-A
427	Yes	7	7-A	100	McF_6	Inf	last	singleC	J5:OT, J5:OT-A, S5:OT, S5:OT-A
428	Yes	7	7-A	100	McF_6	Inf	last	wholeT_0.01	J5:OT, J5:OT-A, S5:OT

Table 14: *(continued)*

	Conjunction	Drivers	Tree	S.Size	Model	sh	S.Time	S.Type	Best method(s)
429	Yes	7	7-A	100	McF_6	Inf	last	wholeT_0.5	J5:OT, J5:OT-A, S5:OT, S5:OT-A
430	Yes	7	7-A	100	McF_6	Inf	unif	singleC	J1:CBN, J5:CBN, J5:CBN-A, S5:CBN, S5:CBN-A
431	Yes	7	7-A	100	McF_6	Inf	unif	wholeT_0.01	J5:CBN, J5:CBN-A, S5:CBN, S5:CBN-A
432	Yes	7	7-A	100	McF_6	Inf	unif	wholeT_0.5	J1:CBN, J1:CBN-A, J5:CBN, J5:CBN-A, S5:CBN, S5:CBN-A
433	No	11	11-B	1000	Bozic	0	last	singleC	S1:OT, S1:OT-A, S5:DiP-A, S5:OT, S5:OT-A
434	No	11	11-B	1000	Bozic	0	last	wholeT_0.01	J1:DiP, J1:DiP-A, J5:DiP, J5:DiP-A, S1:DiP, S1:DiP-A, S5:DiP, S5:DiP-A
435	No	11	11-B	1000	Bozic	0	last	wholeT_0.5	S1:OT, S1:OT-A
436	No	11	11-B	1000	Bozic	0	unif	singleC	S1:CBN, S1:CBN-A
437	No	11	11-B	1000	Bozic	0	unif	wholeT_0.01	S5:CBN-A, S5:DiP-A, S5:OT, S5:OT-A
438	No	11	11-B	1000	Bozic	0	unif	wholeT_0.5	S1:CBN, S1:CBN-A, S5:CBN, S5:CBN-A
439	No	11	11-B	1000	Bozic	Inf	last	singleC	J1:DiP, J1:DiP-A, J1:OT, J1:OT-A, J5:DiP, J5:DiP-A, J5:OT, J5:OT-A, S5:DiP, S5:DiP-A, S5:OT, S5:OT-A
440	No	11	11-B	1000	Bozic	Inf	last	wholeT_0.01	J5:DiP, J5:DiP-A, J5:OT, J5:OT-A
441	No	11	11-B	1000	Bozic	Inf	last	wholeT_0.5	J1:DiP, J1:OT, J1:OT-A, J5:DiP-A, J5:OT, J5:OT-A, S5:DiP, S5:DiP-A, S5:OT, S5:OT-A
442	No	11	11-B	1000	Bozic	Inf	unif	singleC	J1:CBN, S1:CBN, S1:CBN-A, S1:DiP, S1:DiP-A, S1:OT, S1:OT-A
443	No	11	11-B	1000	Bozic	Inf	unif	wholeT_0.01	J1:CBN-A, J1:DiP-A, J1:OT, J1:OT-A, S5:CBN, S5:CBN-A, S5:DiP, S5:DiP-A, S5:OT, S5:OT-A
444	No	11	11-B	1000	Bozic	Inf	unif	wholeT_0.5	J1:CBN, J1:CBN-A, S1:CBN, S1:CBN-A, S1:DiP-A, S1:OT, S1:OT-A
445	No	11	11-B	1000	exp	0	last	singleC	S1:OT, S1:OT-A
446	No	11	11-B	1000	exp	0	last	wholeT_0.01	J1:DiP, J1:DiP-A, J1:OT, J1:OT-A, S1:OT, S1:OT-A, S5:DiP, S5:DiP-A, S5:OT, S5:OT-A
447	No	11	11-B	1000	exp	0	last	wholeT_0.5	S1:OT, S1:OT-A
448	No	11	11-B	1000	exp	0	unif	singleC	S1:CBN, S1:CBN-A, S5:CBN, S5:CBN-A
449	No	11	11-B	1000	exp	0	unif	wholeT_0.01	S1:DiP, S1:DiP-A, S1:OT, S1:OT-A
450	No	11	11-B	1000	exp	0	unif	wholeT_0.5	S1:CBN, S1:CBN-A, S5:CBN-A
451	No	11	11-B	1000	exp	Inf	last	singleC	J1:DiP, J1:DiP-A, J1:OT, J1:OT-A, S1:DiP-A, S5:DiP, S5:DiP-A, S5:OT, S5:OT-A

Table 14: *(continued)*

	Conjunction	Drivers	Tree	S.Size	Model	sh	S.Time	S.Type	Best method(s)
452	No	11	11-B	1000	exp	Inf	last	wholeT_0.01	J5:DiP, J5:DiP-A, J5:OT, J5:OT-A, S5:DiP, S5:DiP-A, S5:OT, S5:OT-A
453	No	11	11-B	1000	exp	Inf	last	wholeT_0.5	J1:DiP, J1:DiP-A, J1:OT, J1:OT-A, S5:DiP, S5:DiP-A, S5:OT, S5:OT-A
454	No	11	11-B	1000	exp	Inf	unif	singleC	S1:CBN, S1:CBN-A, S1:OT, S1:OT-A
455	No	11	11-B	1000	exp	Inf	unif	wholeT_0.01	J1:DiP, J1:DiP-A, J1:OT, J1:OT-A
456	No	11	11-B	1000	exp	Inf	unif	wholeT_0.5	S1:CBN-A, S1:OT, S1:OT-A
457	No	11	11-B	1000	McF_4	0	last	singleC	J1:CBN, J1:DiP, J1:DiP-A, J1:OT, J5:CBN, J5:DiP, J5:DiP-A, J5:OT, J5:OT-A, S1:DiP-A, S1:OT, S1:OT-A, S5:DiP, S5:DiP-A, S5:OT, S5:OT-A
458	No	11	11-B	1000	McF_4	0	last	wholeT_0.01	J1:DiP, J1:DiP-A, J1:OT, J1:OT-A, J5:CBN, J5:DiP, J5:DiP-A, J5:OT, J5:OT-A, S1:DiP-A, S5:DiP, S5:DiP-A, S5:OT, S5:OT-A
459	No	11	11-B	1000	McF_4	0	last	wholeT_0.5	J1:DiP, J1:DiP-A, J1:OT, J1:OT-A, J5:DiP, J5:DiP-A, J5:OT, J5:OT-A, S1:OT, S1:OT-A, S5:DiP, S5:DiP-A, S5:OT, S5:OT-A
460	No	11	11-B	1000	McF_4	0	unif	singleC	J1:CBN-A, J1:DiP, J1:DiP-A, J1:OT, J1:OT-A, S1:DiP, S1:DiP-A, S1:OT, S1:OT-A, S5:CBN-A, S5:DiP, S5:DiP-A, S5:OT, S5:OT-A
461	No	11	11-B	1000	McF_4	0	unif	wholeT_0.01	J1:CBN-A, J1:DiP, J1:DiP-A, J1:OT, J1:OT-A, S1:DiP-A, S5:DiP, S5:DiP-A, S5:OT, S5:OT-A
462	No	11	11-B	1000	McF_4	0	unif	wholeT_0.5	J1:CBN-A, J1:DiP, J1:DiP-A, J1:OT, J1:OT-A, S1:DiP, S1:DiP-A, S1:OT, S1:OT-A, S5:DiP, S5:DiP-A, S5:OT, S5:OT-A
463	No	11	11-B	1000	McF_4	Inf	last	singleC	J1:DiP, J1:DiP-A, J1:OT, J1:OT-A, J5:DiP, J5:DiP-A, J5:OT, J5:OT-A, S1:OT, S1:OT-A, S5:DiP, S5:DiP-A, S5:OT, S5:OT-A
464	No	11	11-B	1000	McF_4	Inf	last	wholeT_0.01	J1:DiP, J1:DiP-A, J1:OT, J1:OT-A, J5:DiP, J5:DiP-A, J5:OT, J5:OT-A, S1:DiP, S1:DiP-A, S5:DiP, S5:DiP-A, S5:OT, S5:OT-A

Table 14: *(continued)*

	Conjunction	Drivers	Tree	S.Size	Model	sh	S.Time	S.Type	Best method(s)
465	No	11	11-B	1000	McF_4	Inf	last	wholeT_0.5	J1:DiP, J1:DiP-A, J1:OT, J1:OT-A, J5:DiP, J5:DiP-A, J5:OT, J5:OT-A, S1:DiP-A, S1:OT, S1:OT-A, S5:DiP, S5:DiP-A, S5:OT, S5:OT-A
466	No	11	11-B	1000	McF_4	Inf	unif	singleC	J1:DiP, J1:DiP-A, J1:OT, J1:OT-A, S1:DiP, S1:DiP-A, S1:OT, S1:OT-A, S5:DiP, S5:DiP-A, S5:OT, S5:OT-A
467	No	11	11-B	1000	McF_4	Inf	unif	wholeT_0.01	J1:DiP, J1:DiP-A, J1:OT, J1:OT-A, S5:DiP, S5:DiP-A, S5:OT, S5:OT-A
468	No	11	11-B	1000	McF_4	Inf	unif	wholeT_0.5	J1:CBN-A, J1:DiP, J1:DiP-A, J1:OT-A, S1:DiP, S1:DiP-A, S5:DiP, S5:DiP-A, S5:OT
469	No	11	11-B	1000	McF_6	0	last	singleC	J1:DiP, J1:OT, J5:DiP, J5:OT, S1:DiP, S5:DiP, S5:OT
470	No	11	11-B	1000	McF_6	0	last	wholeT_0.01	J1:DiP, J1:OT, J5:DiP, J5:OT, S1:DiP, S5:DiP, S5:OT
471	No	11	11-B	1000	McF_6	0	last	wholeT_0.5	J1:DiP, J1:OT, J5:DiP, J5:OT, S1:DiP, S5:DiP, S5:OT
472	No	11	11-B	1000	McF_6	0	unif	singleC	J1:CBN, J1:CBN-A, J1:DiP, J1:DiP-A, J1:OT, J1:OT-A, J5:CBN-A, J5:DiP, J5:DiP-A, J5:OT, J5:OT-A, S1:DiP, S1:DiP-A, S1:OT-A, S5:CBN, S5:DiP, S5:DiP-A, S5:OT, S5:OT-A
473	No	11	11-B	1000	McF_6	0	unif	wholeT_0.01	J1:CBN, J1:CBN-A, J1:DiP, J1:DiP-A, J1:OT, J1:OT-A, J5:CBN, J5:CBN-A, J5:DiP, J5:DiP-A, J5:OT, J5:OT-A, S1:DiP, S1:DiP-A, S5:CBN, S5:DiP, S5:DiP-A, S5:OT, S5:OT-A
474	No	11	11-B	1000	McF_6	0	unif	wholeT_0.5	J1:CBN, J1:CBN-A, J1:DiP, J1:DiP-A, J1:OT, J5:CBN, J5:CBN-A, J5:DiP, J5:DiP-A, J5:OT, J5:OT-A, S1:DiP, S1:DiP-A, S5:CBN, S5:CBN-A, S5:DiP, S5:DiP-A, S5:OT, S5:OT-A
475	No	11	11-B	1000	McF_6	Inf	last	singleC	J1:DiP, J1:OT, J5:DiP, J5:OT, S1:DiP, S5:DiP, S5:OT
476	No	11	11-B	1000	McF_6	Inf	last	wholeT_0.01	J1:DiP, J1:OT, J5:DiP, J5:OT, S1:DiP, S5:DiP, S5:OT
477	No	11	11-B	1000	McF_6	Inf	last	wholeT_0.5	J1:DiP, J1:OT, J5:DiP, J5:OT, S1:DiP, S5:DiP, S5:OT

Table 14: *(continued)*

	Conjunction	Drivers	Tree	S.Size	Model	sh	S.Time	S.Type	Best method(s)
478	No	11	11-B	1000	McF_6	Inf	unif	singleC	J1:CBN-A, J1:DiP, J1:DiP-A, J1:OT, J5:CBN-A, J5:DiP, J5:DiP-A, J5:OT, J5:OT-A, S1:DiP, S1:DiP-A, S5:CBN, S5:DiP, S5:DiP-A, S5:OT
479	No	11	11-B	1000	McF_6	Inf	unif	wholeT_0.01	J1:CBN-A, J1:DiP, J1:DiP-A, J1:OT-A, J5:CBN-A, J5:DiP, J5:DiP-A, J5:OT, J5:OT-A, S1:DiP, S1:DiP-A, S5:DiP, S5:DiP-A, S5:OT, S5:OT-A
480	No	11	11-B	1000	McF_6	Inf	unif	wholeT_0.5	J1:CBN-A, J1:DiP, J1:DiP-A, J1:OT, J1:OT-A, J5:CBN-A, J5:DiP, J5:DiP-A, J5:OT, J5:OT-A, S1:DiP, S1:DiP-A, S5:CBN, S5:CBN-A, S5:DiP, S5:DiP-A, S5:OT-A
481	No	11	11-B	200	Bozic	0	last	singleC	S5:OT, S5:OT-A
482	No	11	11-B	200	Bozic	0	last	wholeT_0.01	J1:OT, J1:OT-A, J5:CBN, J5:CBN-A, J5:OT, J5:OT-A, S5:CBN-A, S5:OT, S5:OT-A
483	No	11	11-B	200	Bozic	0	last	wholeT_0.5	S1:OT, S1:OT-A
484	No	11	11-B	200	Bozic	0	unif	singleC	S1:OT, S1:OT-A
485	No	11	11-B	200	Bozic	0	unif	wholeT_0.01	J1:CBN, J1:CBN-A, J1:DiP, J1:DiP-A, S5:CBN-A, S5:DiP-A, S5:OT, S5:OT-A
486	No	11	11-B	200	Bozic	0	unif	wholeT_0.5	S1:CBN, S1:CBN-A
487	No	11	11-B	200	Bozic	Inf	last	singleC	J5:OT, J5:OT-A, S5:OT-A
488	No	11	11-B	200	Bozic	Inf	last	wholeT_0.01	J5:OT, J5:OT-A
489	No	11	11-B	200	Bozic	Inf	last	wholeT_0.5	J5:OT, J5:OT-A, S5:OT, S5:OT-A
490	No	11	11-B	200	Bozic	Inf	unif	singleC	J1:CBN, J1:CBN-A, J1:OT, J1:OT-A
491	No	11	11-B	200	Bozic	Inf	unif	wholeT_0.01	J5:CBN, J5:CBN-A, S5:CBN, S5:CBN-A, S5:OT, S5:OT-A
492	No	11	11-B	200	Bozic	Inf	unif	wholeT_0.5	J1:CBN, J1:CBN-A, J1:OT, J1:OT-A, S5:CBN, S5:CBN-A
493	No	11	11-B	200	exp	0	last	singleC	S1:OT, S1:OT-A
494	No	11	11-B	200	exp	0	last	wholeT_0.01	J1:OT, J1:OT-A, S1:OT, S1:OT-A, S5:OT, S5:OT-A
495	No	11	11-B	200	exp	0	last	wholeT_0.5	S1:OT, S1:OT-A
496	No	11	11-B	200	exp	0	unif	singleC	S1:CBN, S1:CBN-A, S5:CBN, S5:CBN-A
497	No	11	11-B	200	exp	0	unif	wholeT_0.01	S1:OT, S1:OT-A
498	No	11	11-B	200	exp	0	unif	wholeT_0.5	S1:CBN, S1:CBN-A, S5:CBN, S5:CBN-A
499	No	11	11-B	200	exp	Inf	last	singleC	J1:OT, J1:OT-A, S5:OT, S5:OT-A
500	No	11	11-B	200	exp	Inf	last	wholeT_0.01	J5:CBN-A, J5:OT, J5:OT-A
501	No	11	11-B	200	exp	Inf	last	wholeT_0.5	J1:OT, J1:OT-A, S5:OT, S5:OT-A

Table 14: *(continued)*

	Conjunction	Drivers	Tree	S.Size	Model	sh	S.Time	S.Type	Best method(s)
502	No	11	11-B	200	exp	Inf	unif	singleC	S1:OT, S1:OT-A
503	No	11	11-B	200	exp	Inf	unif	wholeT_0.01	S5:OT, S5:OT-A
504	No	11	11-B	200	exp	Inf	unif	wholeT_0.5	J1:CBN-A, S1:OT, S1:OT-A
505	No	11	11-B	200	McF_4	0	last	singleC	J1:DiP-A, J1:OT-A, J5:DiP-A, J5:OT, J5:OT-A, S1:DiP-A, S5:DiP, S5:DiP-A, S5:OT, S5:OT-A
506	No	11	11-B	200	McF_4	0	last	wholeT_0.01	J1:DiP, J1:DiP-A, J1:OT, J1:OT-A, J5:CBN, J5:DiP-A, J5:OT, J5:OT-A, S1:DiP, S1:DiP-A, S5:DiP, S5:DiP-A, S5:OT, S5:OT-A
507	No	11	11-B	200	McF_4	0	last	wholeT_0.5	J1:DiP-A, J1:OT, J1:OT-A, J5:DiP-A, J5:OT, J5:OT-A, S1:DiP-A, S5:DiP, S5:DiP-A, S5:OT, S5:OT-A
508	No	11	11-B	200	McF_4	0	unif	singleC	J1:CBN, J1:CBN-A, J1:OT, J1:OT-A, S5:CBN-A, S5:DiP, S5:DiP-A, S5:OT, S5:OT-A
509	No	11	11-B	200	McF_4	0	unif	wholeT_0.01	J1:CBN-A, J1:DiP, J1:DiP-A, J1:OT, J1:OT-A, S1:DiP, S1:DiP-A, S5:DiP, S5:DiP-A, S5:OT, S5:OT-A
510	No	11	11-B	200	McF_4	0	unif	wholeT_0.5	J1:CBN-A, J1:OT, J1:OT-A, S5:CBN-A, S5:OT, S5:OT-A
511	No	11	11-B	200	McF_4	Inf	last	singleC	J1:OT, J1:OT-A, J5:OT, J5:OT-A, S5:OT, S5:OT-A
512	No	11	11-B	200	McF_4	Inf	last	wholeT_0.01	J1:DiP-A, J5:OT, J5:OT-A, S5:DiP-A, S5:OT, S5:OT-A
513	No	11	11-B	200	McF_4	Inf	last	wholeT_0.5	J1:OT, J1:OT-A, J5:OT, J5:OT-A, S5:OT, S5:OT-A
514	No	11	11-B	200	McF_4	Inf	unif	singleC	J1:CBN-A, J1:OT, J1:OT-A, S5:CBN-A, S5:OT, S5:OT-A
515	No	11	11-B	200	McF_4	Inf	unif	wholeT_0.01	J1:OT, J5:OT, J5:OT-A, S5:CBN-A, S5:OT, S5:OT-A
516	No	11	11-B	200	McF_4	Inf	unif	wholeT_0.5	J1:CBN-A, J1:OT, J1:OT-A, S5:OT, S5:OT-A
517	No	11	11-B	200	McF_6	0	last	singleC	J1:DiP, J1:OT, J5:DiP, J5:OT, S1:DiP, S5:DiP, S5:OT
518	No	11	11-B	200	McF_6	0	last	wholeT_0.01	J1:DiP, J1:OT, J5:DiP, J5:OT, S1:DiP, S5:DiP, S5:OT
519	No	11	11-B	200	McF_6	0	last	wholeT_0.5	J1:DiP, J1:OT, J5:OT, S1:DiP, S5:DiP, S5:OT

Table 14: *(continued)*

	Conjunction	Drivers	Tree	S.Size	Model	sh	S.Time	S.Type	Best method(s)
520	No	11	11-B	200	McF_6	0	unif	singleC	J1:CBN, J1:CBN-A, J1:DiP, J1:DiP-A, J1:OT, J1:OT-A, J5:CBN, J5:CBN-A, J5:DiP, J5:DiP-A, J5:OT, J5:OT-A, S5:CBN, S5:CBN-A, S5:DiP, S5:DiP-A, S5:OT, S5:OT-A
521	No	11	11-B	200	McF_6	0	unif	wholeT_0.01	J1:CBN, J1:CBN-A, J1:DiP, J1:DiP-A, J1:OT, J1:OT-A, J5:CBN, J5:CBN-A, J5:DiP, J5:DiP-A, J5:OT, J5:OT-A, S5:CBN-A, S5:DiP, S5:DiP-A, S5:OT, S5:OT-A
522	No	11	11-B	200	McF_6	0	unif	wholeT_0.5	J1:CBN, J1:CBN-A, J1:DiP, J1:DiP-A, J1:OT, J1:OT-A, J5:CBN, J5:CBN-A, J5:DiP-A, J5:OT, J5:OT-A, S5:CBN, S5:CBN-A, S5:OT, S5:OT-A
523	No	11	11-B	200	McF_6	Inf	last	singleC	J1:OT, J5:OT, S5:OT
524	No	11	11-B	200	McF_6	Inf	last	wholeT_0.01	J1:OT, J5:OT, S1:DiP, S5:OT
525	No	11	11-B	200	McF_6	Inf	last	wholeT_0.5	J1:OT, J5:OT, S1:DiP, S5:DiP, S5:OT
526	No	11	11-B	200	McF_6	Inf	unif	singleC	J1:CBN, J1:CBN-A, J1:OT, J1:OT-A, J5:CBN, J5:CBN-A, J5:DiP-A, J5:OT, J5:OT-A, S5:CBN, S5:CBN-A, S5:OT, S5:OT-A
527	No	11	11-B	200	McF_6	Inf	unif	wholeT_0.01	J1:CBN, J1:CBN-A, J1:OT, J1:OT-A, J5:CBN-A, J5:DiP-A, J5:OT, J5:OT-A, S5:CBN, S5:CBN-A, S5:OT, S5:OT-A
528	No	11	11-B	200	McF_6	Inf	unif	wholeT_0.5	J1:CBN, J1:CBN-A, J1:OT, J1:OT-A, J5:CBN-A, J5:OT, J5:OT-A, S5:CBN, S5:CBN-A, S5:OT, S5:OT-A
529	No	11	11-B	100	Bozic	0	last	singleC	S5:OT, S5:OT-A
530	No	11	11-B	100	Bozic	0	last	wholeT_0.01	J5:CBN-A, J5:OT, J5:OT-A, S5:OT, S5:OT-A
531	No	11	11-B	100	Bozic	0	last	wholeT_0.5	J1:CBN, J1:CBN-A, J1:OT, J1:OT-A, S1:OT, S1:OT-A
532	No	11	11-B	100	Bozic	0	unif	singleC	S1:OT, S1:OT-A
533	No	11	11-B	100	Bozic	0	unif	wholeT_0.01	J1:CBN, J1:CBN-A, J1:OT, J1:OT-A, S5:CBN, S5:OT, S5:OT-A
534	No	11	11-B	100	Bozic	0	unif	wholeT_0.5	J1:CBN-A, S1:OT, S1:OT-A
535	No	11	11-B	100	Bozic	Inf	last	singleC	J5:OT, J5:OT-A
536	No	11	11-B	100	Bozic	Inf	last	wholeT_0.01	J5:OT, J5:OT-A
537	No	11	11-B	100	Bozic	Inf	last	wholeT_0.5	J5:OT, J5:OT-A

Table 14: *(continued)*

	Conjunction	Drivers	Tree	S.Size	Model	sh	S.Time	S.Type	Best method(s)
538	No	11	11-B	100	Bozic	Inf	unif	singleC	J1:CBN, J1:OT, J1:OT-A, J5:CBN, J5:CBN-A, S1:OT, S1:OT-A, S5:CBN, S5:CBN-A
539	No	11	11-B	100	Bozic	Inf	unif	wholeT_0.01	J5:CBN, J5:CBN-A, S5:CBN, S5:CBN-A, S5:OT, S5:OT-A
540	No	11	11-B	100	Bozic	Inf	unif	wholeT_0.5	J1:CBN, J1:CBN-A, J1:OT, J1:OT-A, S1:OT, S1:OT-A, S5:CBN, S5:CBN-A
541	No	11	11-B	100	exp	0	last	singleC	S1:OT, S1:OT-A
542	No	11	11-B	100	exp	0	last	wholeT_0.01	J1:OT, J1:OT-A, S5:OT, S5:OT-A
543	No	11	11-B	100	exp	0	last	wholeT_0.5	S1:OT, S1:OT-A
544	No	11	11-B	100	exp	0	unif	singleC	J1:CBN, S1:OT, S1:OT-A
545	No	11	11-B	100	exp	0	unif	wholeT_0.01	S1:OT, S1:OT-A
546	No	11	11-B	100	exp	0	unif	wholeT_0.5	J1:CBN, J1:CBN-A, S1:OT, S1:OT-A
547	No	11	11-B	100	exp	Inf	last	singleC	J5:OT, J5:OT-A, S5:OT, S5:OT-A
548	No	11	11-B	100	exp	Inf	last	wholeT_0.01	J5:CBN-A, J5:OT, J5:OT-A
549	No	11	11-B	100	exp	Inf	last	wholeT_0.5	S5:OT, S5:OT-A
550	No	11	11-B	100	exp	Inf	unif	singleC	J1:CBN, J1:OT, J1:OT-A, S1:OT, S1:OT-A, S5:CBN, S5:CBN-A
551	No	11	11-B	100	exp	Inf	unif	wholeT_0.01	S5:CBN-A, S5:OT, S5:OT-A
552	No	11	11-B	100	exp	Inf	unif	wholeT_0.5	J1:CBN-A, J1:OT, J1:OT-A, S1:OT, S1:OT-A
553	No	11	11-B	100	McF_4	0	last	singleC	J5:OT, J5:OT-A, S5:OT, S5:OT-A
554	No	11	11-B	100	McF_4	0	last	wholeT_0.01	J1:DiP, J1:DiP-A, J1:OT, J1:OT-A, J5:DiP-A, J5:OT, J5:OT-A, S1:DiP, S1:DiP-A, S5:DiP, S5:DiP-A, S5:OT, S5:OT-A
555	No	11	11-B	100	McF_4	0	last	wholeT_0.5	J5:OT, J5:OT-A, S5:OT, S5:OT-A
556	No	11	11-B	100	McF_4	0	unif	singleC	J1:CBN-A, J1:OT, J1:OT-A, S5:CBN-A, S5:OT, S5:OT-A
557	No	11	11-B	100	McF_4	0	unif	wholeT_0.01	J1:DiP, J1:DiP-A, S1:DiP, S1:DiP-A, S5:CBN, S5:CBN-A, S5:DiP, S5:DiP-A, S5:OT, S5:OT-A
558	No	11	11-B	100	McF_4	0	unif	wholeT_0.5	J1:CBN-A, J1:OT, J1:OT-A, S5:CBN, S5:OT, S5:OT-A
559	No	11	11-B	100	McF_4	Inf	last	singleC	J5:OT, J5:OT-A, S5:OT, S5:OT-A
560	No	11	11-B	100	McF_4	Inf	last	wholeT_0.01	J5:OT, J5:OT-A, S5:OT, S5:OT-A
561	No	11	11-B	100	McF_4	Inf	last	wholeT_0.5	J5:OT, J5:OT-A, S5:OT, S5:OT-A
562	No	11	11-B	100	McF_4	Inf	unif	singleC	J1:OT, J1:OT-A, S5:CBN-A, S5:OT, S5:OT-A
563	No	11	11-B	100	McF_4	Inf	unif	wholeT_0.01	J5:OT, J5:OT-A, S5:OT

Table 14: *(continued)*

	Conjunction	Drivers	Tree	S.Size	Model	sh	S.Time	S.Type	Best method(s)
564	No	11	11-B	100	McF_4	Inf	unif	wholeT_0.5	J5:OT, J5:OT-A, S5:OT, S5:OT-A
565	No	11	11-B	100	McF_6	0	last	singleC	J1:OT, J5:OT, S5:OT
566	No	11	11-B	100	McF_6	0	last	wholeT_0.01	J1:DiP, J1:OT, J5:DiP, J5:OT, S1:DiP, S5:DiP, S5:OT
567	No	11	11-B	100	McF_6	0	last	wholeT_0.5	J1:OT, J5:OT, S5:OT
568	No	11	11-B	100	McF_6	0	unif	singleC	J1:CBN, J1:CBN-A, J1:OT, J1:OT-A, J5:CBN, J5:CBN-A, J5:OT, J5:OT-A, S5:CBN, S5:OT, S5:OT-A
569	No	11	11-B	100	McF_6	0	unif	wholeT_0.01	J1:DiP, J1:DiP-A, J5:CBN, J5:CBN-A, J5:DiP, J5:DiP-A, J5:OT, J5:OT-A, S1:DiP, S5:DiP, S5:DiP-A, S5:OT, S5:OT-A
570	No	11	11-B	100	McF_6	0	unif	wholeT_0.5	J1:CBN, J1:CBN-A, J1:OT, J1:OT-A, J5:CBN, J5:CBN-A, J5:OT, J5:OT-A, S5:CBN, S5:CBN-A, S5:OT, S5:OT-A
571	No	11	11-B	100	McF_6	Inf	last	singleC	J1:OT, J5:OT, S5:OT
572	No	11	11-B	100	McF_6	Inf	last	wholeT_0.01	J5:OT, S5:OT
573	No	11	11-B	100	McF_6	Inf	last	wholeT_0.5	J1:OT, J5:OT, S5:OT
574	No	11	11-B	100	McF_6	Inf	unif	singleC	J1:CBN, J1:CBN-A, J1:OT, J1:OT-A, J5:CBN, J5:CBN-A, J5:OT, J5:OT-A, S5:CBN, S5:CBN-A, S5:OT, S5:OT-A
575	No	11	11-B	100	McF_6	Inf	unif	wholeT_0.01	J5:CBN-A, J5:OT, J5:OT-A, S5:CBN, S5:CBN-A, S5:OT, S5:OT-A
576	No	11	11-B	100	McF_6	Inf	unif	wholeT_0.5	J1:CBN-A, J1:OT, J1:OT-A, J5:CBN, J5:CBN-A, J5:OT, J5:OT-A, S5:CBN-A, S5:OT, S5:OT-A
577	No	9	9-B	1000	Bozic	0	last	singleC	S1:OT, S1:OT-A
578	No	9	9-B	1000	Bozic	0	last	wholeT_0.01	S1:CBN-A, S1:DiP, S1:DiP-A, S1:OT, S1:OT-A, S5:CBN-A, S5:DiP-A, S5:OT, S5:OT-A
579	No	9	9-B	1000	Bozic	0	last	wholeT_0.5	S1:OT, S1:OT-A
580	No	9	9-B	1000	Bozic	0	unif	singleC	S1:CBN, S1:CBN-A, S5:CBN, S5:CBN-A
581	No	9	9-B	1000	Bozic	0	unif	wholeT_0.01	J1:DiP, J1:DiP-A, J1:OT, J1:OT-A, S1:DiP, S1:DiP-A, S1:OT, S1:OT-A
582	No	9	9-B	1000	Bozic	0	unif	wholeT_0.5	S1:CBN, S1:CBN-A, S5:CBN, S5:CBN-A
583	No	9	9-B	1000	Bozic	Inf	last	singleC	J1:DiP, J1:DiP-A, J1:OT, J1:OT-A, J5:DiP, J5:DiP-A, J5:OT, J5:OT-A, S1:DiP, S1:DiP-A, S5:DiP, S5:DiP-A, S5:OT, S5:OT-A
584	No	9	9-B	1000	Bozic	Inf	last	wholeT_0.01	J5:DiP, J5:DiP-A

Table 14: *(continued)*

	Conjunction	Drivers	Tree	S.Size	Model	sh	S.Time	S.Type	Best method(s)
585	No	9	9-B	1000	Bozic	Inf	last	wholeT_0.5	J1:DiP, J1:DiP-A, J1:OT, J1:OT-A, J5:DiP, J5:DiP-A, J5:OT, J5:OT-A, S1:DiP, S1:DiP-A, S5:DiP, S5:DiP-A, S5:OT, S5:OT-A
586	No	9	9-B	1000	Bozic	Inf	unif	singleC	S1:CBN, S1:CBN-A, S1:DiP, S1:OT, S1:OT-A
587	No	9	9-B	1000	Bozic	Inf	unif	wholeT_0.01	J1:CBN-A, S5:CBN, S5:CBN-A, S5:DiP, S5:DiP-A, S5:OT, S5:OT-A
588	No	9	9-B	1000	Bozic	Inf	unif	wholeT_0.5	S1:CBN, S1:CBN-A, S1:DiP-A, S1:OT, S1:OT-A
589	No	9	9-B	1000	exp	0	last	singleC	J1:CBN, J1:CBN-A, S1:CBN, S1:CBN-A, S5:CBN, S5:CBN-A
590	No	9	9-B	1000	exp	0	last	wholeT_0.01	S1:DiP-A, S1:OT, S1:OT-A
591	No	9	9-B	1000	exp	0	last	wholeT_0.5	J1:CBN, J1:CBN-A, S1:CBN, S1:CBN-A, S5:CBN, S5:CBN-A
592	No	9	9-B	1000	exp	0	unif	singleC	S1:CBN, S1:CBN-A, S5:CBN, S5:CBN-A
593	No	9	9-B	1000	exp	0	unif	wholeT_0.01	S1:CBN, S1:CBN-A, S5:CBN, S5:CBN-A
594	No	9	9-B	1000	exp	0	unif	wholeT_0.5	S1:CBN, S1:CBN-A, S5:CBN, S5:CBN-A
595	No	9	9-B	1000	exp	Inf	last	singleC	J1:DiP, J1:DiP-A, J1:OT, J1:OT-A, S1:DiP-A, S5:DiP, S5:DiP-A, S5:OT, S5:OT-A
596	No	9	9-B	1000	exp	Inf	last	wholeT_0.01	J5:DiP, J5:DiP-A, S5:DiP
597	No	9	9-B	1000	exp	Inf	last	wholeT_0.5	J1:DiP, J1:DiP-A, J1:OT, J1:OT-A, S5:DiP-A, S5:OT, S5:OT-A
598	No	9	9-B	1000	exp	Inf	unif	singleC	S1:OT, S1:OT-A
599	No	9	9-B	1000	exp	Inf	unif	wholeT_0.01	J1:OT, J1:OT-A
600	No	9	9-B	1000	exp	Inf	unif	wholeT_0.5	S1:OT, S1:OT-A
601	No	9	9-B	1000	McF_4	0	last	singleC	J1:DiP, J1:DiP-A, J1:OT, J1:OT-A, J5:CBN, J5:DiP, J5:DiP-A, J5:OT, J5:OT-A, S1:OT, S1:OT-A, S5:DiP, S5:DiP-A, S5:OT, S5:OT-A
602	No	9	9-B	1000	McF_4	0	last	wholeT_0.01	J1:DiP, J1:DiP-A, J1:OT, J1:OT-A, J5:DiP, J5:DiP-A, J5:OT, J5:OT-A, S5:DiP, S5:DiP-A, S5:OT-A
603	No	9	9-B	1000	McF_4	0	last	wholeT_0.5	J1:DiP, J1:DiP-A, J1:OT, J1:OT-A, J5:CBN, J5:DiP, J5:DiP-A, J5:OT, J5:OT-A, S1:OT, S1:OT-A, S5:DiP, S5:DiP-A, S5:OT, S5:OT-A

Table 14: *(continued)*

	Conjunction	Drivers	Tree	S.Size	Model	sh	S.Time	S.Type	Best method(s)
604	No	9	9-B	1000	McF_4	0	unif	singleC	J1:DiP, J1:DiP-A, J1:OT, J1:OT-A, S1:DiP, S1:DiP-A, S1:OT, S1:OT-A, S5:DiP-A, S5:OT, S5:OT-A
605	No	9	9-B	1000	McF_4	0	unif	wholeT_0.01	J1:DiP, J1:DiP-A, J1:OT, J1:OT-A, S1:DiP-A, S5:DiP, S5:DiP-A, S5:OT, S5:OT-A
606	No	9	9-B	1000	McF_4	0	unif	wholeT_0.5	J1:DiP, J1:DiP-A, J1:OT, J1:OT-A, S1:DiP, S1:DiP-A, S1:OT, S1:OT-A, S5:DiP, S5:DiP-A, S5:OT, S5:OT-A
607	No	9	9-B	1000	McF_4	Inf	last	singleC	J1:DiP, J1:DiP-A, J1:OT, J1:OT-A, J5:DiP, J5:DiP-A, J5:OT, J5:OT-A, S1:OT, S1:OT-A, S5:DiP, S5:DiP-A, S5:OT, S5:OT-A
608	No	9	9-B	1000	McF_4	Inf	last	wholeT_0.01	J1:DiP, J1:DiP-A, J1:OT, J1:OT-A, J5:DiP, J5:DiP-A, J5:OT, J5:OT-A, S1:DiP-A, S5:DiP, S5:DiP-A, S5:OT, S5:OT-A
609	No	9	9-B	1000	McF_4	Inf	last	wholeT_0.5	J1:DiP-A, J1:OT, J1:OT-A, J5:DiP, J5:DiP-A, J5:OT, J5:OT-A, S1:DiP-A, S1:OT, S1:OT-A, S5:DiP, S5:DiP-A, S5:OT, S5:OT-A
610	No	9	9-B	1000	McF_4	Inf	unif	singleC	J1:DiP, J1:DiP-A, J1:OT, J1:OT-A, S1:DiP, S1:DiP-A, S1:OT, S1:OT-A, S5:DiP, S5:DiP-A, S5:OT, S5:OT-A
611	No	9	9-B	1000	McF_4	Inf	unif	wholeT_0.01	J1:DiP, J1:DiP-A, J1:OT, J1:OT-A, S5:DiP, S5:DiP-A, S5:OT, S5:OT-A
612	No	9	9-B	1000	McF_4	Inf	unif	wholeT_0.5	J1:DiP, J1:DiP-A, J1:OT, J1:OT-A, S1:DiP, S1:DiP-A, S1:OT, S1:OT-A, S5:DiP, S5:DiP-A, S5:OT, S5:OT-A
613	No	9	9-B	1000	McF_6	0	last	singleC	J1:DiP, J1:OT, J5:DiP, J5:OT, S1:DiP, S5:DiP, S5:OT
614	No	9	9-B	1000	McF_6	0	last	wholeT_0.01	J1:DiP, J1:OT, J5:DiP, J5:OT, S1:DiP, S5:DiP, S5:OT
615	No	9	9-B	1000	McF_6	0	last	wholeT_0.5	J1:DiP, J1:OT, J5:DiP, J5:OT, S1:DiP, S1:OT, S5:DiP, S5:OT

Table 14: *(continued)*

	Conjunction	Drivers	Tree	S.Size	Model	sh	S.Time	S.Type	Best method(s)
616	No	9	9-B	1000	McF_6	0	unif	singleC	J1:CBN, J1:CBN-A, J1:DiP, J1:DiP-A, J1:OT, J1:OT-A, J5:CBN, J5:CBN-A, J5:DiP, J5:DiP-A, J5:OT, J5:OT-A, S1:DiP, S1:DiP-A, S1:OT, S1:OT-A, S5:CBN-A, S5:DiP, S5:DiP-A, S5:OT, S5:OT-A
617	No	9	9-B	1000	McF_6	0	unif	wholeT_0.01	J1:CBN, J1:CBN-A, J1:DiP, J1:DiP-A, J1:OT, J1:OT-A, J5:CBN, J5:CBN-A, J5:DiP, J5:DiP-A, J5:OT, J5:OT-A, S1:DiP, S5:DiP, S5:DiP-A, S5:OT, S5:OT-A
618	No	9	9-B	1000	McF_6	0	unif	wholeT_0.5	J1:CBN-A, J1:DiP, J1:DiP-A, J1:OT, J1:OT-A, J5:CBN, J5:CBN-A, J5:DiP, J5:DiP-A, J5:OT, J5:OT-A, S1:DiP, S1:DiP-A, S1:OT, S1:OT-A, S5:CBN, S5:DiP-A, S5:OT, S5:OT-A
619	No	9	9-B	1000	McF_6	Inf	last	singleC	J1:DiP, J1:OT, J5:DiP, J5:OT, S1:DiP, S5:DiP, S5:OT
620	No	9	9-B	1000	McF_6	Inf	last	wholeT_0.01	J1:DiP, J1:OT, J5:DiP, J5:OT, S1:DiP, S5:DiP, S5:OT
621	No	9	9-B	1000	McF_6	Inf	last	wholeT_0.5	J1:DiP, J1:OT, J5:DiP, J5:OT, S1:DiP, S5:DiP, S5:OT
622	No	9	9-B	1000	McF_6	Inf	unif	singleC	J1:CBN, J1:CBN-A, J1:DiP, J1:DiP-A, J1:OT, J1:OT-A, J5:CBN-A, J5:DiP, J5:DiP-A, J5:OT, J5:OT-A, S1:DiP, S1:DiP-A, S1:OT, S1:OT-A, S5:DiP, S5:DiP-A, S5:OT, S5:OT-A
623	No	9	9-B	1000	McF_6	Inf	unif	wholeT_0.01	J1:CBN, J1:CBN-A, J1:DiP, J1:DiP-A, J1:OT, J1:OT-A, J5:CBN-A, J5:DiP, J5:DiP-A, J5:OT, J5:OT-A, S5:DiP, S5:DiP-A, S5:OT, S5:OT-A
624	No	9	9-B	1000	McF_6	Inf	unif	wholeT_0.5	J1:CBN, J1:CBN-A, J1:DiP, J1:DiP-A, J1:OT, J1:OT-A, J5:CBN-A, J5:DiP, J5:DiP-A, J5:OT, J5:OT-A, S1:DiP, S1:DiP-A, S1:OT, S1:OT-A, S5:DiP, S5:DiP-A, S5:OT, S5:OT-A
625	No	9	9-B	200	Bozic	0	last	singleC	S1:OT, S1:OT-A
626	No	9	9-B	200	Bozic	0	last	wholeT_0.01	J1:OT, J1:OT-A, S5:OT, S5:OT-A
627	No	9	9-B	200	Bozic	0	last	wholeT_0.5	S1:OT, S1:OT-A

Table 14: *(continued)*

	Conjunction	Drivers	Tree	S.Size	Model	sh	S.Time	S.Type	Best method(s)
628	No	9	9-B	200	Bozic	0	unif	singleC	J1:CBN-A, S1:CBN, S1:CBN-A, S5:CBN, S5:CBN-A
629	No	9	9-B	200	Bozic	0	unif	wholeT_0.01	S1:DiP, S1:DiP-A
630	No	9	9-B	200	Bozic	0	unif	wholeT_0.5	S1:CBN, S1:CBN-A, S5:CBN
631	No	9	9-B	200	Bozic	Inf	last	singleC	J1:OT, J1:OT-A, S5:OT, S5:OT-A
632	No	9	9-B	200	Bozic	Inf	last	wholeT_0.01	J5:CBN-A, J5:OT, J5:OT-A
633	No	9	9-B	200	Bozic	Inf	last	wholeT_0.5	J1:OT, J1:OT-A, J5:OT, J5:OT-A
634	No	9	9-B	200	Bozic	Inf	unif	singleC	J1:CBN, J1:CBN-A, S1:OT, S1:OT-A
635	No	9	9-B	200	Bozic	Inf	unif	wholeT_0.01	S5:CBN, S5:CBN-A, S5:OT, S5:OT-A
636	No	9	9-B	200	Bozic	Inf	unif	wholeT_0.5	J1:CBN, J1:CBN-A, S1:OT, S1:OT-A
637	No	9	9-B	200	exp	0	last	singleC	S1:OT, S1:OT-A
638	No	9	9-B	200	exp	0	last	wholeT_0.01	S1:OT, S1:OT-A
639	No	9	9-B	200	exp	0	last	wholeT_0.5	S1:CBN, S1:CBN-A, S5:CBN
640	No	9	9-B	200	exp	0	unif	singleC	S1:CBN, S1:CBN-A, S5:CBN, S5:CBN-A
641	No	9	9-B	200	exp	0	unif	wholeT_0.01	J1:CBN, S1:CBN, S1:CBN-A, S5:CBN-A
642	No	9	9-B	200	exp	0	unif	wholeT_0.5	S1:CBN, S1:CBN-A, S5:CBN, S5:CBN-A
643	No	9	9-B	200	exp	Inf	last	singleC	J1:OT, J1:OT-A, S5:OT, S5:OT-A
644	No	9	9-B	200	exp	Inf	last	wholeT_0.01	J5:CBN-A, J5:OT, J5:OT-A
645	No	9	9-B	200	exp	Inf	last	wholeT_0.5	J1:OT, J1:OT-A, S5:OT, S5:OT-A
646	No	9	9-B	200	exp	Inf	unif	singleC	S1:OT, S1:OT-A
647	No	9	9-B	200	exp	Inf	unif	wholeT_0.01	J1:CBN-A, J1:OT, J1:OT-A, S5:CBN, S5:CBN-A
648	No	9	9-B	200	exp	Inf	unif	wholeT_0.5	S1:OT, S1:OT-A
649	No	9	9-B	200	McF_4	0	last	singleC	J1:OT, J1:OT-A, J5:OT, J5:OT-A, S5:DiP-A, S5:OT, S5:OT-A
650	No	9	9-B	200	McF_4	0	last	wholeT_0.01	J1:DiP, J1:DiP-A, J1:OT, J1:OT-A, J5:DiP, J5:DiP-A, J5:OT, J5:OT-A, S1:DiP, S1:DiP-A, S5:DiP, S5:DiP-A, S5:OT, S5:OT-A
651	No	9	9-B	200	McF_4	0	last	wholeT_0.5	J1:OT, J1:OT-A, J5:DiP-A, J5:OT, J5:OT-A, S1:DiP, S1:DiP-A, S5:DiP, S5:DiP-A, S5:OT, S5:OT-A
652	No	9	9-B	200	McF_4	0	unif	singleC	J1:OT, J1:OT-A, S5:OT, S5:OT-A
653	No	9	9-B	200	McF_4	0	unif	wholeT_0.01	J1:DiP, J1:DiP-A, J1:OT, J1:OT-A, S1:DiP, S1:DiP-A, S5:CBN-A, S5:DiP, S5:DiP-A, S5:OT, S5:OT-A
654	No	9	9-B	200	McF_4	0	unif	wholeT_0.5	J1:OT, J1:OT-A, S5:OT, S5:OT-A
655	No	9	9-B	200	McF_4	Inf	last	singleC	J1:OT, J1:OT-A, J5:OT, J5:OT-A, S5:OT, S5:OT-A

Table 14: *(continued)*

	Conjunction	Drivers	Tree	S.Size	Model	sh	S.Time	S.Type	Best method(s)
656	No	9	9-B	200	McF_4	Inf	last	wholeT_0.01	J1:DiP-A, J1:OT, J1:OT-A, J5:OT, J5:OT-A, S5:OT, S5:OT-A
657	No	9	9-B	200	McF_4	Inf	last	wholeT_0.5	J1:OT, J1:OT-A, J5:OT, J5:OT-A, S1:DiP-A, S5:DiP-A, S5:OT, S5:OT-A
658	No	9	9-B	200	McF_4	Inf	unif	singleC	J1:OT, J1:OT-A, S5:OT, S5:OT-A
659	No	9	9-B	200	McF_4	Inf	unif	wholeT_0.01	J1:OT, J1:OT-A, S5:OT, S5:OT-A
660	No	9	9-B	200	McF_4	Inf	unif	wholeT_0.5	J1:OT, J1:OT-A, S5:OT, S5:OT-A
661	No	9	9-B	200	McF_6	0	last	singleC	J1:DiP, J1:OT, J5:OT, S1:DiP, S5:DiP, S5:OT
662	No	9	9-B	200	McF_6	0	last	wholeT_0.01	J1:DiP, J1:OT, J5:DiP, J5:OT, S1:DiP, S5:DiP, S5:OT
663	No	9	9-B	200	McF_6	0	last	wholeT_0.5	J1:OT, J5:OT, S1:DiP, S5:DiP, S5:OT
664	No	9	9-B	200	McF_6	0	unif	singleC	J1:CBN, J1:CBN-A, J1:DiP-A, J1:OT, J1:OT-A, J5:CBN, J5:CBN-A, J5:OT, J5:OT-A, S1:DiP, S1:DiP-A, S5:CBN, S5:CBN-A, S5:DiP, S5:DiP-A, S5:OT, S5:OT-A
665	No	9	9-B	200	McF_6	0	unif	wholeT_0.01	J1:CBN-A, J1:DiP, J1:DiP-A, J1:OT, J1:OT-A, J5:CBN, J5:CBN-A, J5:DiP, J5:DiP-A, J5:OT, J5:OT-A, S1:DiP, S1:DiP-A, S5:DiP, S5:DiP-A, S5:OT, S5:OT-A
666	No	9	9-B	200	McF_6	0	unif	wholeT_0.5	J1:CBN, J1:CBN-A, J1:DiP-A, J1:OT, J1:OT-A, J5:CBN-A, J5:DiP-A, J5:OT, J5:OT-A, S1:DiP-A, S5:CBN, S5:CBN-A, S5:DiP, S5:DiP-A, S5:OT, S5:OT-A
667	No	9	9-B	200	McF_6	Inf	last	singleC	J1:OT, J5:OT, S1:DiP, S5:DiP, S5:OT
668	No	9	9-B	200	McF_6	Inf	last	wholeT_0.01	J1:OT, J5:OT, S1:DiP, S5:DiP, S5:OT
669	No	9	9-B	200	McF_6	Inf	last	wholeT_0.5	J1:OT, J5:OT, S1:DiP, S5:DiP, S5:OT
670	No	9	9-B	200	McF_6	Inf	unif	singleC	J1:CBN, J1:CBN-A, J1:OT, J1:OT-A, J5:CBN-A, J5:OT, J5:OT-A, S5:CBN, S5:OT, S5:OT-A
671	No	9	9-B	200	McF_6	Inf	unif	wholeT_0.01	J1:CBN, J1:CBN-A, J1:OT, J1:OT-A, J5:CBN-A, J5:OT, J5:OT-A, S5:CBN, S5:CBN-A, S5:OT, S5:OT-A
672	No	9	9-B	200	McF_6	Inf	unif	wholeT_0.5	J1:CBN, J1:CBN-A, J1:DiP-A, J1:OT, J1:OT-A, J5:CBN-A, J5:OT, J5:OT-A, S5:CBN, S5:CBN-A, S5:DiP-A, S5:OT, S5:OT-A
673	No	9	9-B	100	Bozic	0	last	singleC	S1:OT, S1:OT-A

Table 14: *(continued)*

	Conjunction	Drivers	Tree	S.Size	Model	sh	S.Time	S.Type	Best method(s)
674	No	9	9-B	100	Bozic	0	last	wholeT_0.01	S5:OT, S5:OT-A
675	No	9	9-B	100	Bozic	0	last	wholeT_0.5	S1:OT, S1:OT-A
676	No	9	9-B	100	Bozic	0	unif	singleC	J1:CBN, S1:OT, S1:OT-A
677	No	9	9-B	100	Bozic	0	unif	wholeT_0.01	J1:CBN-A, J1:OT, J1:OT-A
678	No	9	9-B	100	Bozic	0	unif	wholeT_0.5	J1:CBN, J1:CBN-A, S1:CBN, S1:CBN-A, S5:CBN
679	No	9	9-B	100	Bozic	Inf	last	singleC	J5:OT, J5:OT-A
680	No	9	9-B	100	Bozic	Inf	last	wholeT_0.01	J5:OT, J5:OT-A
681	No	9	9-B	100	Bozic	Inf	last	wholeT_0.5	J5:OT, J5:OT-A
682	No	9	9-B	100	Bozic	Inf	unif	singleC	J1:CBN, J1:CBN-A, J1:OT, J1:OT-A, S1:OT, S1:OT-A, S5:CBN, S5:CBN-A
683	No	9	9-B	100	Bozic	Inf	unif	wholeT_0.01	J5:CBN, J5:CBN-A, S5:CBN, S5:CBN-A, S5:OT, S5:OT-A
684	No	9	9-B	100	Bozic	Inf	unif	wholeT_0.5	J1:CBN, J1:CBN-A, J1:OT, J1:OT-A, S1:OT, S1:OT-A
685	No	9	9-B	100	exp	0	last	singleC	S1:OT, S1:OT-A
686	No	9	9-B	100	exp	0	last	wholeT_0.01	S1:OT, S5:OT, S5:OT-A
687	No	9	9-B	100	exp	0	last	wholeT_0.5	S1:OT, S1:OT-A
688	No	9	9-B	100	exp	0	unif	singleC	J1:CBN, J1:CBN-A, S1:CBN, S1:CBN-A, S5:CBN, S5:CBN-A
689	No	9	9-B	100	exp	0	unif	wholeT_0.01	S1:OT, S1:OT-A
690	No	9	9-B	100	exp	0	unif	wholeT_0.5	S1:CBN, S1:CBN-A, S5:CBN, S5:CBN-A
691	No	9	9-B	100	exp	Inf	last	singleC	S5:OT, S5:OT-A
692	No	9	9-B	100	exp	Inf	last	wholeT_0.01	J5:CBN-A, J5:OT, J5:OT-A
693	No	9	9-B	100	exp	Inf	last	wholeT_0.5	S5:OT, S5:OT-A
694	No	9	9-B	100	exp	Inf	unif	singleC	S1:OT, S1:OT-A
695	No	9	9-B	100	exp	Inf	unif	wholeT_0.01	J1:CBN, J1:CBN-A, J1:OT, J1:OT-A, J5:CBN, J5:CBN-A, S1:OT, S1:OT-A, S5:CBN, S5:CBN-A
696	No	9	9-B	100	exp	Inf	unif	wholeT_0.5	S1:OT, S1:OT-A
697	No	9	9-B	100	McF_4	0	last	singleC	J5:OT, J5:OT-A, S5:OT, S5:OT-A
698	No	9	9-B	100	McF_4	0	last	wholeT_0.01	J1:DiP, J1:DiP-A, J1:OT-A, J5:OT, J5:OT-A, S1:DiP, S1:DiP-A, S5:DiP, S5:DiP-A, S5:OT, S5:OT-A
699	No	9	9-B	100	McF_4	0	last	wholeT_0.5	J1:OT, J1:OT-A, J5:OT, J5:OT-A, S5:OT, S5:OT-A
700	No	9	9-B	100	McF_4	0	unif	singleC	J1:OT, J1:OT-A, S5:OT, S5:OT-A
701	No	9	9-B	100	McF_4	0	unif	wholeT_0.01	J1:OT, S1:DiP, S1:DiP-A, S5:DiP-A, S5:OT, S5:OT-A
702	No	9	9-B	100	McF_4	0	unif	wholeT_0.5	J1:OT, J1:OT-A, S5:OT, S5:OT-A

Table 14: *(continued)*

	Conjunction	Drivers	Tree	S.Size	Model	sh	S.Time	S.Type	Best method(s)
703	No	9	9-B	100	McF_4	Inf	last	singleC	J1:OT, J1:OT-A, J5:OT, J5:OT-A, S5:OT, S5:OT-A
704	No	9	9-B	100	McF_4	Inf	last	wholeT_0.01	J5:OT, J5:OT-A, S5:OT, S5:OT-A
705	No	9	9-B	100	McF_4	Inf	last	wholeT_0.5	J1:OT, J1:OT-A, J5:OT, J5:OT-A, S5:OT, S5:OT-A
706	No	9	9-B	100	McF_4	Inf	unif	singleC	J1:OT, J1:OT-A, S5:OT, S5:OT-A
707	No	9	9-B	100	McF_4	Inf	unif	wholeT_0.01	S5:OT, S5:OT-A
708	No	9	9-B	100	McF_4	Inf	unif	wholeT_0.5	J1:OT, J1:OT-A, S5:OT, S5:OT-A
709	No	9	9-B	100	McF_6	0	last	singleC	J1:OT, J5:OT, S5:OT
710	No	9	9-B	100	McF_6	0	last	wholeT_0.01	J1:DiP, J5:OT, S1:DiP, S5:OT
711	No	9	9-B	100	McF_6	0	last	wholeT_0.5	J1:OT, J5:OT, S5:OT
712	No	9	9-B	100	McF_6	0	unif	singleC	J1:CBN, J1:CBN-A, J1:OT, J1:OT-A, J5:CBN, J5:CBN-A, J5:OT, J5:OT-A, S5:CBN-A, S5:OT, S5:OT-A
713	No	9	9-B	100	McF_6	0	unif	wholeT_0.01	J1:CBN, J1:CBN-A, J1:DiP, J1:DiP-A, J1:OT, J5:CBN-A, J5:OT, J5:OT-A, S1:DiP, S1:DiP-A, S5:CBN, S5:DiP, S5:DiP-A, S5:OT, S5:OT-A
714	No	9	9-B	100	McF_6	0	unif	wholeT_0.5	J1:CBN, J1:CBN-A, J5:CBN-A, J5:OT, J5:OT-A, S5:CBN, S5:CBN-A, S5:OT, S5:OT-A
715	No	9	9-B	100	McF_6	Inf	last	singleC	J1:OT, J5:OT, S5:OT
716	No	9	9-B	100	McF_6	Inf	last	wholeT_0.01	J5:OT, S5:OT
717	No	9	9-B	100	McF_6	Inf	last	wholeT_0.5	J1:OT, J5:OT, S5:OT
718	No	9	9-B	100	McF_6	Inf	unif	singleC	J1:CBN, J1:CBN-A, J1:OT, J5:CBN-A, J5:OT, J5:OT-A, S5:OT, S5:OT-A
719	No	9	9-B	100	McF_6	Inf	unif	wholeT_0.01	J5:CBN-A, J5:OT, J5:OT-A, S5:CBN, S5:CBN-A, S5:OT, S5:OT-A
720	No	9	9-B	100	McF_6	Inf	unif	wholeT_0.5	J1:CBN, J1:CBN-A, J1:OT, J5:CBN-A, J5:OT, S5:CBN, S5:CBN-A, S5:OT, S5:OT-A
721	No	7	7-B	1000	Bozic	0	last	singleC	J1:CBN-A, J1:OT-A, J5:CBN-A, J5:OT-A, S1:OT-A, S5:OT-A
722	No	7	7-B	1000	Bozic	0	last	wholeT_0.01	J1:DiP-A, J1:OT-A, J5:DiP-A, J5:OT-A, S1:DiP-A, S1:OT-A, S5:DiP-A, S5:OT-A
723	No	7	7-B	1000	Bozic	0	last	wholeT_0.5	J1:CBN-A, J1:OT-A, J5:CBN-A, J5:OT-A, S1:OT-A, S5:CBN, S5:CBN-A, S5:OT-A
724	No	7	7-B	1000	Bozic	0	unif	singleC	J1:CBN, J1:CBN-A, J1:OT, J1:OT-A, S1:CBN, S1:CBN-A, S1:OT

Table 14: *(continued)*

	Conjunction	Drivers	Tree	S.Size	Model	sh	S.Time	S.Type	Best method(s)
725	No	7	7-B	1000	Bozic	0	unif	wholeT_0.01	J5:CBN, J5:CBN-A, J5:DiP-A, J5:OT, J5:OT-A
726	No	7	7-B	1000	Bozic	0	unif	wholeT_0.5	NA
727	No	7	7-B	1000	Bozic	Inf	last	singleC	J1:DiP-A, J1:OT-A, J5:DiP-A, J5:OT-A, S1:DiP-A, S5:DiP-A, S5:OT-A
728	No	7	7-B	1000	Bozic	Inf	last	wholeT_0.01	J5:CBN, J5:CBN-A, J5:DiP, J5:DiP-A, J5:OT, J5:OT-A
729	No	7	7-B	1000	Bozic	Inf	last	wholeT_0.5	J1:DiP-A, J1:OT-A, J5:CBN, J5:DiP-A, J5:OT-A, S5:DiP-A, S5:OT-A
730	No	7	7-B	1000	Bozic	Inf	unif	singleC	J1:CBN, J1:CBN-A, J5:CBN, J5:CBN-A, J5:OT, J5:OT-A, S1:CBN, S1:CBN-A, S5:CBN, S5:CBN-A, S5:OT
731	No	7	7-B	1000	Bozic	Inf	unif	wholeT_0.01	J1:CBN-A, J1:OT, J1:OT-A, J5:CBN, J5:CBN-A, S5:CBN, S5:CBN-A
732	No	7	7-B	1000	Bozic	Inf	unif	wholeT_0.5	J1:CBN, J1:CBN-A, J1:OT, J1:OT-A, S1:CBN, S1:CBN-A
733	No	7	7-B	1000	exp	0	last	singleC	J1:CBN-A, J1:OT-A, S1:OT-A
734	No	7	7-B	1000	exp	0	last	wholeT_0.01	J1:CBN, J1:CBN-A, J1:OT-A, J5:CBN-A, J5:OT-A, S1:CBN, S1:CBN-A, S1:OT-A, S5:CBN, S5:OT-A
735	No	7	7-B	1000	exp	0	last	wholeT_0.5	J1:CBN-A, J1:OT-A, S1:OT-A
736	No	7	7-B	1000	exp	0	unif	singleC	NA
737	No	7	7-B	1000	exp	0	unif	wholeT_0.01	J1:CBN, J1:CBN-A, J1:OT-A
738	No	7	7-B	1000	exp	0	unif	wholeT_0.5	NA
739	No	7	7-B	1000	exp	Inf	last	singleC	J1:OT-A, J5:CBN-A, J5:OT-A, S1:OT-A, S5:OT-A
740	No	7	7-B	1000	exp	Inf	last	wholeT_0.01	J1:CBN, J1:CBN-A, J1:OT, J1:OT-A, J5:CBN-A, J5:DiP-A, J5:OT-A, S5:CBN, S5:CBN-A, S5:OT, S5:OT-A
741	No	7	7-B	1000	exp	Inf	last	wholeT_0.5	J1:CBN, J1:OT-A, J5:CBN-A, J5:OT-A, S1:OT-A, S5:OT-A
742	No	7	7-B	1000	exp	Inf	unif	singleC	J1:CBN, J1:CBN-A, J1:OT, J1:OT-A, S1:CBN, S1:CBN-A
743	No	7	7-B	1000	exp	Inf	unif	wholeT_0.01	J1:CBN, J1:CBN-A, J1:OT, J1:OT-A, J5:CBN, J5:CBN-A, J5:OT, J5:OT-A, S5:CBN, S5:CBN-A, S5:OT, S5:OT-A
744	No	7	7-B	1000	exp	Inf	unif	wholeT_0.5	J1:CBN, J1:CBN-A, J1:OT, J1:OT-A, S1:CBN-A

Table 14: *(continued)*

	Conjunction	Drivers	Tree	S.Size	Model	sh	S.Time	S.Type	Best method(s)
745	No	7	7-B	1000	McF_4	0	last	singleC	J1:DiP-A, J1:OT-A, J5:DiP-A, J5:OT-A, S1:DiP, S1:DiP-A, S1:OT, S1:OT-A, S5:DiP, S5:DiP-A, S5:OT, S5:OT-A
746	No	7	7-B	1000	McF_4	0	last	wholeT_0.01	J1:DiP, J1:DiP-A, J1:OT, J1:OT-A, J5:DiP-A, J5:OT-A, S1:DiP, S1:DiP-A, S5:DiP, S5:DiP-A, S5:OT, S5:OT-A
747	No	7	7-B	1000	McF_4	0	last	wholeT_0.5	J1:DiP-A, J1:OT-A, J5:DiP-A, J5:OT-A, S1:DiP, S1:DiP-A, S1:OT, S1:OT-A, S5:DiP, S5:DiP-A, S5:OT, S5:OT-A
748	No	7	7-B	1000	McF_4	0	unif	singleC	J1:CBN-A, J1:DiP, J1:DiP-A, J1:OT, J1:OT-A, J5:CBN, J5:CBN-A, J5:DiP, J5:DiP-A, J5:OT, J5:OT-A, S1:DiP, S1:DiP-A, S1:OT, S1:OT-A, S5:CBN-A, S5:DiP, S5:DiP-A, S5:OT, S5:OT-A
749	No	7	7-B	1000	McF_4	0	unif	wholeT_0.01	J1:CBN, J1:DiP, J1:DiP-A, J1:OT, J1:OT-A, J5:CBN, J5:DiP, J5:DiP-A, J5:OT, J5:OT-A, S1:DiP, S1:DiP-A, S5:CBN, S5:CBN-A, S5:DiP, S5:DiP-A, S5:OT, S5:OT-A
750	No	7	7-B	1000	McF_4	0	unif	wholeT_0.5	J1:CBN-A, J1:DiP, J1:DiP-A, J1:OT, J1:OT-A, J5:CBN, J5:CBN-A, J5:DiP, J5:DiP-A, J5:OT, J5:OT-A, S1:DiP, S1:DiP-A, S1:OT, S5:CBN-A, S5:DiP, S5:DiP-A, S5:OT, S5:OT-A
751	No	7	7-B	1000	McF_4	Inf	last	singleC	J1:DiP-A, J1:OT-A, J5:DiP-A, J5:OT-A, S1:DiP, S1:OT-A, S5:DiP, S5:DiP-A, S5:OT, S5:OT-A
752	No	7	7-B	1000	McF_4	Inf	last	wholeT_0.01	J1:DiP, J1:DiP-A, J1:OT, J1:OT-A, J5:DiP-A, J5:OT-A, S1:DiP, S1:DiP-A, S5:DiP, S5:DiP-A, S5:OT, S5:OT-A
753	No	7	7-B	1000	McF_4	Inf	last	wholeT_0.5	J1:DiP-A, J1:OT-A, J5:DiP-A, J5:OT-A, S1:DiP, S1:DiP-A, S1:OT, S1:OT-A, S5:DiP, S5:DiP-A, S5:OT, S5:OT-A
754	No	7	7-B	1000	McF_4	Inf	unif	singleC	J1:CBN-A, J1:DiP-A, J1:OT, J1:OT-A, J5:CBN-A, J5:DiP-A, J5:OT, J5:OT-A, S1:DiP, S1:DiP-A, S1:OT, S1:OT-A, S5:CBN-A, S5:DiP-A, S5:OT, S5:OT-A
755	No	7	7-B	1000	McF_4	Inf	unif	wholeT_0.01	J1:DiP-A, J1:OT, J1:OT-A, J5:CBN-A, J5:DiP-A, J5:OT, J5:OT-A, S1:DiP, S1:DiP-A, S5:DiP-A, S5:OT, S5:OT-A

Table 14: *(continued)*

	Conjunction	Drivers	Tree	S.Size	Model	sh	S.Time	S.Type	Best method(s)
756	No	7	7-B	1000	McF_4	Inf	unif	wholeT_0.5	J1:DiP-A, J1:OT, J1:OT-A, J5:CBN-A, J5:DiP-A, J5:OT, J5:OT-A, S1:DiP, S1:DiP-A, S1:OT, S1:OT-A, S5:DiP-A, S5:OT, S5:OT-A
757	No	7	7-B	1000	McF_6	0	last	singleC	J1:DiP, J1:DiP-A, J1:OT, J1:OT-A, J5:DiP, J5:DiP-A, J5:OT, J5:OT-A, S1:DiP, S1:DiP-A, S5:DiP, S5:DiP-A, S5:OT, S5:OT-A
758	No	7	7-B	1000	McF_6	0	last	wholeT_0.01	J1:DiP, J1:DiP-A, J5:DiP, J5:DiP-A, J5:OT, J5:OT-A, S1:DiP, S1:DiP-A, S5:DiP, S5:DiP-A, S5:OT, S5:OT-A
759	No	7	7-B	1000	McF_6	0	last	wholeT_0.5	J1:DiP, J1:DiP-A, J1:OT, J1:OT-A, J5:DiP, J5:DiP-A, J5:OT, J5:OT-A, S1:DiP, S1:DiP-A, S1:OT, S1:OT-A, S5:DiP, S5:DiP-A, S5:OT, S5:OT-A
760	No	7	7-B	1000	McF_6	0	unif	singleC	J1:CBN, J1:DiP, J1:DiP-A, J1:OT, J1:OT-A, J5:CBN, J5:CBN-A, J5:DiP, J5:DiP-A, J5:OT, J5:OT-A, S1:CBN, S1:CBN-A, S1:DiP, S1:DiP-A, S1:OT, S1:OT-A, S5:CBN, S5:CBN-A, S5:DiP, S5:DiP-A, S5:OT, S5:OT-A
761	No	7	7-B	1000	McF_6	0	unif	wholeT_0.01	J1:CBN, J1:CBN-A, J1:DiP, J1:DiP-A, J1:OT, J1:OT-A, J5:CBN, J5:CBN-A, J5:DiP, J5:DiP-A, J5:OT, J5:OT-A, S1:DiP, S1:DiP-A, S5:CBN, S5:CBN-A, S5:DiP, S5:DiP-A, S5:OT, S5:OT-A
762	No	7	7-B	1000	McF_6	0	unif	wholeT_0.5	none
763	No	7	7-B	1000	McF_6	Inf	last	singleC	J1:DiP, J1:DiP-A, J1:OT, J1:OT-A, J5:DiP, J5:DiP-A, J5:OT, J5:OT-A, S1:DiP-A, S5:DiP, S5:DiP-A, S5:OT, S5:OT-A
764	No	7	7-B	1000	McF_6	Inf	last	wholeT_0.01	J1:DiP-A, J5:DiP-A, J5:OT, J5:OT-A, S5:DiP-A, S5:OT, S5:OT-A
765	No	7	7-B	1000	McF_6	Inf	last	wholeT_0.5	J1:DiP, J1:DiP-A, J1:OT, J1:OT-A, J5:DiP, J5:DiP-A, J5:OT, J5:OT-A, S1:DiP, S1:DiP-A, S5:DiP, S5:DiP-A, S5:OT, S5:OT-A

Table 14: *(continued)*

	Conjunction	Drivers	Tree	S.Size	Model	sh	S.Time	S.Type	Best method(s)
766	No	7	7-B	1000	McF_6	Inf	unif	singleC	J1:CBN, J1:CBN-A, J1:DiP, J1:DiP-A, J1:OT, J1:OT-A, J5:CBN-A, J5:DiP-A, J5:OT, J5:OT-A, S1:CBN, S1:CBN-A, S1:DiP, S1:DiP-A, S1:OT, S1:OT-A, S5:CBN, S5:CBN-A, S5:DiP, S5:DiP-A, S5:OT, S5:OT-A
767	No	7	7-B	1000	McF_6	Inf	unif	wholeT_0.01	J1:CBN, J1:CBN-A, J1:DiP, J1:DiP-A, J1:OT, J1:OT-A, J5:CBN, J5:CBN-A, J5:DiP, J5:DiP-A, J5:OT, J5:OT-A, S1:DiP-A, S5:CBN, S5:CBN-A, S5:DiP, S5:DiP-A, S5:OT, S5:OT-A
768	No	7	7-B	1000	McF_6	Inf	unif	wholeT_0.5	J1:CBN, J1:CBN-A, J1:DiP, J1:DiP-A, J1:OT, J1:OT-A, J5:CBN, J5:CBN-A, J5:DiP, J5:DiP-A, J5:OT, J5:OT-A, S1:CBN, S1:DiP, S1:DiP-A, S1:OT, S1:OT-A, S5:CBN, S5:CBN-A, S5:DiP, S5:DiP-A, S5:OT, S5:OT-A
769	No	7	7-B	200	Bozic	0	last	singleC	J1:CBN-A, J1:OT-A, J5:CBN-A, J5:OT-A, S5:CBN, S5:OT-A
770	No	7	7-B	200	Bozic	0	last	wholeT_0.01	J1:OT-A, J5:OT-A, S5:OT-A
771	No	7	7-B	200	Bozic	0	last	wholeT_0.5	J1:CBN-A, J1:OT-A, J5:CBN-A, J5:OT-A, S1:OT-A, S5:CBN, S5:CBN-A, S5:OT-A
772	No	7	7-B	200	Bozic	0	unif	singleC	J1:CBN, J1:CBN-A, J1:OT, J1:OT-A, S1:OT, S1:OT-A
773	No	7	7-B	200	Bozic	0	unif	wholeT_0.01	J5:CBN, J5:CBN-A, J5:OT, J5:OT-A
774	No	7	7-B	200	Bozic	0	unif	wholeT_0.5	J1:OT, J1:OT-A, S1:CBN-A, S1:OT, S1:OT-A
775	No	7	7-B	200	Bozic	Inf	last	singleC	J5:CBN, J5:OT-A, S5:OT-A
776	No	7	7-B	200	Bozic	Inf	last	wholeT_0.01	J5:CBN, J5:CBN-A, J5:OT, J5:OT-A
777	No	7	7-B	200	Bozic	Inf	last	wholeT_0.5	J5:CBN, J5:OT-A, S5:OT-A
778	No	7	7-B	200	Bozic	Inf	unif	singleC	J1:CBN, J1:CBN-A, J5:CBN, J5:CBN-A, J5:OT, J5:OT-A, S5:CBN, S5:CBN-A
779	No	7	7-B	200	Bozic	Inf	unif	wholeT_0.01	J5:CBN, J5:CBN-A, J5:OT, J5:OT-A, S5:CBN, S5:CBN-A, S5:OT, S5:OT-A
780	No	7	7-B	200	Bozic	Inf	unif	wholeT_0.5	J1:CBN, J1:CBN-A
781	No	7	7-B	200	exp	0	last	singleC	S1:OT-A
782	No	7	7-B	200	exp	0	last	wholeT_0.01	J1:CBN, J1:CBN-A, J1:OT-A, J5:CBN-A, J5:OT-A, S1:CBN, S1:CBN-A, S1:OT-A, S5:CBN, S5:CBN-A, S5:OT-A

Table 14: *(continued)*

	Conjunction	Drivers	Tree	S.Size	Model	sh	S.Time	S.Type	Best method(s)
783	No	7	7-B	200	exp	0	last	wholeT_0.5	J1:CBN-A, J1:OT-A, S1:OT-A
784	No	7	7-B	200	exp	0	unif	singleC	NA
785	No	7	7-B	200	exp	0	unif	wholeT_0.01	J1:CBN-A, J1:OT-A
786	No	7	7-B	200	exp	0	unif	wholeT_0.5	NA
787	No	7	7-B	200	exp	Inf	last	singleC	J1:OT-A, J5:CBN-A, J5:OT-A, S5:OT-A
788	No	7	7-B	200	exp	Inf	last	wholeT_0.01	J5:CBN, J5:CBN-A, J5:OT-A, S5:CBN, S5:CBN-A, S5:OT-A
789	No	7	7-B	200	exp	Inf	last	wholeT_0.5	J1:CBN, J1:OT-A, J5:CBN-A, J5:OT-A, S5:OT-A
790	No	7	7-B	200	exp	Inf	unif	singleC	J1:CBN, J1:CBN-A, J1:OT, J1:OT-A
791	No	7	7-B	200	exp	Inf	unif	wholeT_0.01	J5:CBN, J5:CBN-A, J5:OT, J5:OT-A, S5:CBN, S5:CBN-A, S5:OT, S5:OT-A
792	No	7	7-B	200	exp	Inf	unif	wholeT_0.5	J1:CBN, J1:CBN-A, J1:OT, J1:OT-A, S1:CBN, S1:CBN-A
793	No	7	7-B	200	McF_4	0	last	singleC	J1:OT-A, J5:OT-A, S5:OT, S5:OT-A
794	No	7	7-B	200	McF_4	0	last	wholeT_0.01	J1:DiP, J1:DiP-A, J1:OT, J5:OT-A, S1:DiP, S1:DiP-A, S5:DiP, S5:DiP-A, S5:OT, S5:OT-A
795	No	7	7-B	200	McF_4	0	last	wholeT_0.5	J1:OT-A, J5:OT-A, S5:OT, S5:OT-A
796	No	7	7-B	200	McF_4	0	unif	singleC	J1:CBN, J1:CBN-A, J1:DiP-A, J1:OT, J1:OT-A, J5:CBN, J5:CBN-A, J5:OT, J5:OT-A, S1:DiP-A, S5:CBN, S5:CBN-A, S5:DiP-A, S5:OT, S5:OT-A
797	No	7	7-B	200	McF_4	0	unif	wholeT_0.01	J1:CBN, J1:CBN-A, J1:DiP-A, J1:OT, J1:OT-A, J5:CBN, J5:CBN-A, J5:DiP-A, J5:OT, J5:OT-A, S1:DiP, S1:DiP-A, S5:CBN, S5:CBN-A, S5:DiP-A, S5:OT, S5:OT-A
798	No	7	7-B	200	McF_4	0	unif	wholeT_0.5	J1:CBN, J1:CBN-A, J1:OT, J1:OT-A, J5:CBN, J5:CBN-A, J5:OT, J5:OT-A, S5:CBN, S5:CBN-A, S5:OT, S5:OT-A
799	No	7	7-B	200	McF_4	Inf	last	singleC	J1:OT-A, J5:OT-A, S5:OT, S5:OT-A
800	No	7	7-B	200	McF_4	Inf	last	wholeT_0.01	J1:OT, J1:OT-A, J5:OT-A, S5:OT, S5:OT-A
801	No	7	7-B	200	McF_4	Inf	last	wholeT_0.5	J1:OT-A, S5:OT, S5:OT-A
802	No	7	7-B	200	McF_4	Inf	unif	singleC	J1:OT, J1:OT-A, J5:CBN-A, J5:OT, J5:OT-A, S5:OT, S5:OT-A
803	No	7	7-B	200	McF_4	Inf	unif	wholeT_0.01	J1:OT, J1:OT-A, J5:CBN-A, J5:OT, J5:OT-A, S5:OT, S5:OT-A

Table 14: *(continued)*

	Conjunction	Drivers	Tree	S.Size	Model	sh	S.Time	S.Type	Best method(s)
804	No	7	7-B	200	McF_4	Inf	unif	wholeT_0.5	J1:CBN-A, J1:OT, J1:OT-A, J5:CBN-A, J5:OT, J5:OT-A, S5:CBN-A, S5:OT, S5:OT-A
805	No	7	7-B	200	McF_6	0	last	singleC	J1:DiP-A, J1:OT, J1:OT-A, J5:DiP-A, J5:OT, J5:OT-A, S1:DiP-A, S5:DiP-A, S5:OT, S5:OT-A
806	No	7	7-B	200	McF_6	0	last	wholeT_0.01	J1:DiP, J1:DiP-A, J5:DiP, J5:DiP-A, J5:OT, J5:OT-A, S1:DiP, S1:DiP-A, S5:DiP, S5:DiP-A, S5:OT, S5:OT-A
807	No	7	7-B	200	McF_6	0	last	wholeT_0.5	J1:DiP-A, J1:OT, J1:OT-A, J5:DiP-A, J5:OT, J5:OT-A, S5:DiP-A, S5:OT, S5:OT-A
808	No	7	7-B	200	McF_6	0	unif	singleC	J1:CBN, J1:CBN-A, J1:DiP, J1:DiP-A, J1:OT, J1:OT-A, J5:CBN, J5:CBN-A, J5:DiP-A, J5:OT, J5:OT-A, S1:DiP, S1:DiP-A, S5:CBN, S5:CBN-A, S5:DiP, S5:DiP-A, S5:OT, S5:OT-A
809	No	7	7-B	200	McF_6	0	unif	wholeT_0.01	J1:CBN, J1:CBN-A, J1:DiP, J1:DiP-A, J1:OT, J1:OT-A, J5:CBN, J5:CBN-A, J5:OT, J5:OT-A, S1:DiP, S1:DiP-A, S5:CBN, S5:CBN-A, S5:DiP, S5:DiP-A, S5:OT, S5:OT-A
810	No	7	7-B	200	McF_6	0	unif	wholeT_0.5	J1:CBN, J1:CBN-A, J1:DiP, J1:DiP-A, J1:OT, J1:OT-A, J5:CBN, J5:CBN-A, J5:DiP-A, J5:OT, J5:OT-A, S1:DiP, S5:CBN, S5:CBN-A, S5:DiP, S5:DiP-A, S5:OT, S5:OT-A
811	No	7	7-B	200	McF_6	Inf	last	singleC	J1:DiP-A, J1:OT, J1:OT-A, J5:DiP-A, J5:OT, J5:OT-A, S1:DiP-A, S5:DiP-A, S5:OT, S5:OT-A
812	No	7	7-B	200	McF_6	Inf	last	wholeT_0.01	J5:OT, J5:OT-A, S5:OT, S5:OT-A
813	No	7	7-B	200	McF_6	Inf	last	wholeT_0.5	J1:DiP-A, J1:OT, J1:OT-A, J5:DiP-A, J5:OT, J5:OT-A, S1:DiP-A, S5:DiP-A, S5:OT, S5:OT-A
814	No	7	7-B	200	McF_6	Inf	unif	singleC	J1:CBN, J1:CBN-A, J1:OT, J1:OT-A, J5:CBN, J5:CBN-A, J5:OT, J5:OT-A, S5:CBN, S5:CBN-A, S5:OT, S5:OT-A

Table 14: *(continued)*

	Conjunction	Drivers	Tree	S.Size	Model	sh	S.Time	S.Type	Best method(s)
815	No	7	7-B	200	McF_6	Inf	unif	wholeT_0.01	J1:CBN, J1:CBN-A, J1:DiP-A, J5:CBN, J5:CBN-A, J5:OT-A, S1:DiP, S1:DiP-A, S5:CBN, S5:CBN-A, S5:DiP-A, S5:OT, S5:OT-A
816	No	7	7-B	200	McF_6	Inf	unif	wholeT_0.5	J1:CBN, J1:CBN-A, J1:DiP-A, J1:OT, J1:OT-A, J5:CBN, J5:CBN-A, J5:OT-A, S1:DiP, S1:DiP-A, S5:CBN, S5:CBN-A, S5:DiP, S5:DiP-A, S5:OT, S5:OT-A
817	No	7	7-B	100	Bozic	0	last	singleC	J1:CBN-A, J1:OT-A, J5:CBN-A, J5:OT-A, S5:CBN, S5:OT-A
818	No	7	7-B	100	Bozic	0	last	wholeT_0.01	J5:OT-A, S5:CBN, S5:OT-A
819	No	7	7-B	100	Bozic	0	last	wholeT_0.5	J5:OT-A, S5:CBN, S5:CBN-A, S5:OT-A
820	No	7	7-B	100	Bozic	0	unif	singleC	J1:CBN, J1:CBN-A, J1:OT, J1:OT-A
821	No	7	7-B	100	Bozic	0	unif	wholeT_0.01	J5:CBN, J5:CBN-A, J5:OT, J5:OT-A
822	No	7	7-B	100	Bozic	0	unif	wholeT_0.5	J1:CBN, J1:CBN-A, J1:OT, J1:OT-A, S1:OT, S1:OT-A
823	No	7	7-B	100	Bozic	Inf	last	singleC	J5:CBN, J5:OT-A, S5:OT, S5:OT-A
824	No	7	7-B	100	Bozic	Inf	last	wholeT_0.01	J5:CBN, J5:CBN-A, J5:OT-A
825	No	7	7-B	100	Bozic	Inf	last	wholeT_0.5	J5:CBN, J5:OT-A, S5:OT-A
826	No	7	7-B	100	Bozic	Inf	unif	singleC	J5:CBN, J5:CBN-A, J5:OT, J5:OT-A, S5:CBN, S5:CBN-A
827	No	7	7-B	100	Bozic	Inf	unif	wholeT_0.01	J5:CBN, J5:CBN-A, J5:OT, J5:OT-A, S5:CBN-A
828	No	7	7-B	100	Bozic	Inf	unif	wholeT_0.5	J5:CBN, J5:CBN-A, J5:OT, J5:OT-A, S5:CBN, S5:CBN-A, S5:OT, S5:OT-A
829	No	7	7-B	100	exp	0	last	singleC	J1:OT-A
830	No	7	7-B	100	exp	0	last	wholeT_0.01	J1:CBN, J1:CBN-A, J1:OT-A, J5:CBN-A, J5:OT-A, S1:CBN, S5:CBN-A
831	No	7	7-B	100	exp	0	last	wholeT_0.5	J1:OT-A, S1:OT-A
832	No	7	7-B	100	exp	0	unif	singleC	J1:OT, J1:OT-A, S1:CBN, S1:CBN-A, S1:OT, S1:OT-A
833	No	7	7-B	100	exp	0	unif	wholeT_0.01	J1:CBN, J1:CBN-A, J1:OT, J1:OT-A, S1:OT, S1:OT-A
834	No	7	7-B	100	exp	0	unif	wholeT_0.5	NA
835	No	7	7-B	100	exp	Inf	last	singleC	J1:OT-A, J5:CBN-A, J5:OT-A, S5:OT-A
836	No	7	7-B	100	exp	Inf	last	wholeT_0.01	J5:CBN, J5:CBN-A, J5:OT-A
837	No	7	7-B	100	exp	Inf	last	wholeT_0.5	J1:OT-A, J5:CBN-A, J5:OT-A, S5:OT-A
838	No	7	7-B	100	exp	Inf	unif	singleC	J1:CBN, J1:CBN-A, J1:OT, J1:OT-A
839	No	7	7-B	100	exp	Inf	unif	wholeT_0.01	J5:CBN, J5:CBN-A, J5:OT, J5:OT-A, S5:CBN, S5:OT, S5:OT-A

Table 14: *(continued)*

	Conjunction	Drivers	Tree	S.Size	Model	sh	S.Time	S.Type	Best method(s)
840	No	7	7-B	100	exp	Inf	unif	wholeT_0.5	J1:CBN, J1:CBN-A, J1:OT, S1:OT, S1:OT-A
841	No	7	7-B	100	McF_4	0	last	singleC	J1:OT-A, J5:OT-A, S5:OT, S5:OT-A
842	No	7	7-B	100	McF_4	0	last	wholeT_0.01	J5:OT-A, S1:DiP, S1:DiP-A, S5:DiP-A, S5:OT, S5:OT-A
843	No	7	7-B	100	McF_4	0	last	wholeT_0.5	J5:OT-A, S5:OT, S5:OT-A
844	No	7	7-B	100	McF_4	0	unif	singleC	J1:CBN-A, J5:CBN, J5:CBN-A, J5:OT, J5:OT-A, S5:CBN, S5:CBN-A
845	No	7	7-B	100	McF_4	0	unif	wholeT_0.01	J1:CBN, J1:CBN-A, J1:OT, J1:OT-A, J5:CBN, J5:CBN-A, J5:OT, J5:OT-A, S1:DiP-A, S5:CBN, S5:CBN-A, S5:OT, S5:OT-A
846	No	7	7-B	100	McF_4	0	unif	wholeT_0.5	J1:CBN-A, J1:OT-A, J5:CBN, J5:CBN-A, J5:OT, J5:OT-A, S5:CBN-A, S5:OT, S5:OT-A
847	No	7	7-B	100	McF_4	Inf	last	singleC	J5:OT-A, S5:OT, S5:OT-A
848	No	7	7-B	100	McF_4	Inf	last	wholeT_0.01	J5:OT-A, S5:OT, S5:OT-A
849	No	7	7-B	100	McF_4	Inf	last	wholeT_0.5	J5:OT-A, S5:OT-A
850	No	7	7-B	100	McF_4	Inf	unif	singleC	J1:CBN-A, J1:OT, J1:OT-A, J5:CBN-A, J5:OT, J5:OT-A, S5:OT, S5:OT-A
851	No	7	7-B	100	McF_4	Inf	unif	wholeT_0.01	J1:CBN-A, J1:OT-A, J5:CBN-A, J5:OT, J5:OT-A, S5:CBN-A, S5:OT, S5:OT-A
852	No	7	7-B	100	McF_4	Inf	unif	wholeT_0.5	J1:CBN-A, J5:CBN-A, J5:OT, J5:OT-A, S5:CBN-A
853	No	7	7-B	100	McF_6	0	last	singleC	J5:OT, J5:OT-A, S5:OT, S5:OT-A
854	No	7	7-B	100	McF_6	0	last	wholeT_0.01	J5:OT, J5:OT-A
855	No	7	7-B	100	McF_6	0	last	wholeT_0.5	J5:OT, J5:OT-A, S5:OT, S5:OT-A
856	No	7	7-B	100	McF_6	0	unif	singleC	J1:CBN, J1:CBN-A, J1:OT, J1:OT-A, J5:CBN, J5:CBN-A, J5:OT, J5:OT-A, S1:DiP, S5:CBN, S5:CBN-A, S5:OT, S5:OT-A
857	No	7	7-B	100	McF_6	0	unif	wholeT_0.01	J1:DiP-A, J5:CBN, J5:CBN-A, J5:DiP-A, S1:DiP, S5:CBN, S5:CBN-A, S5:DiP, S5:DiP-A
858	No	7	7-B	100	McF_6	0	unif	wholeT_0.5	J1:CBN, J1:CBN-A, J5:CBN, J5:CBN-A, S5:CBN, S5:CBN-A
859	No	7	7-B	100	McF_6	Inf	last	singleC	J5:OT, J5:OT-A, S5:OT-A
860	No	7	7-B	100	McF_6	Inf	last	wholeT_0.01	J5:OT, J5:OT-A, S5:OT, S5:OT-A
861	No	7	7-B	100	McF_6	Inf	last	wholeT_0.5	J5:OT, J5:OT-A, S5:OT, S5:OT-A

Table 14: *(continued)*

	Conjunction	Drivers	Tree	S.Size	Model	sh	S.Time	S.Type	Best method(s)
862	No	7	7-B	100	McF_6	Inf	unif	singleC	J1:CBN, J1:CBN-A, J5:CBN, J5:CBN-A, J5:OT-A, S5:CBN, S5:CBN-A, S5:OT-A
863	No	7	7-B	100	McF_6	Inf	unif	wholeT_0.01	J5:CBN, J5:CBN-A, S5:CBN, S5:CBN-A
864	No	7	7-B	100	McF_6	Inf	unif	wholeT_0.5	J1:CBN, J1:CBN-A, J5:CBN, J5:CBN-A, S5:CBN, S5:CBN-A

5.3 Confidence sets (MCB), PND, Drivers Unknown

Table 15: Confidence sets (method MCB) when Drivers are Unknown for measure PND.

	Conjunction	Drivers	Tree	S.Size	Model	sh	S.Time	S.Type	Best method(s)
1	Yes	11	11-A	1000	Bozic	0	last	singleC	S1:OT, S1:OT-A
2	Yes	11	11-A	1000	Bozic	0	last	wholeT_0.01	S1:DiP, S1:DiP-A, S1:OT, S1:OT-A, S5:DiP, S5:DiP-A, S5:OT, S5:OT-A
3	Yes	11	11-A	1000	Bozic	0	last	wholeT_0.5	S1:OT, S1:OT-A
4	Yes	11	11-A	1000	Bozic	0	unif	singleC	J1:CBN, J1:DiP, J1:OT, J5:CBN-A, S1:CBN, S1:CBN-A, S1:DiP, S1:DiP-A, S1:OT, S1:OT-A
5	Yes	11	11-A	1000	Bozic	0	unif	wholeT_0.01	S1:CBN, S1:CBN-A
6	Yes	11	11-A	1000	Bozic	0	unif	wholeT_0.5	J1:CBN, J1:CBN-A, J1:DiP, J1:DiP-A, J1:OT, J1:OT-A, J5:CBN, J5:DiP, J5:DiP-A, J5:OT, J5:OT-A, S1:CBN, S1:CBN-A, S1:DiP, S1:DiP-A, S1:OT, S1:OT-A, S5:CBN, S5:CBN-A, S5:DiP, S5:DiP-A, S5:OT, S5:OT-A
7	Yes	11	11-A	1000	Bozic	Inf	last	singleC	S1:DiP, S1:DiP-A, S1:OT, S1:OT-A, S5:DiP, S5:DiP-A, S5:OT, S5:OT-A
8	Yes	11	11-A	1000	Bozic	Inf	last	wholeT_0.01	J1:OT, J1:OT-A, S1:DiP, S1:OT, S1:OT-A, S5:DiP, S5:DiP-A, S5:OT, S5:OT-A
9	Yes	11	11-A	1000	Bozic	Inf	last	wholeT_0.5	S1:OT, S1:OT-A
10	Yes	11	11-A	1000	Bozic	Inf	unif	singleC	S1:OT, S1:OT-A
11	Yes	11	11-A	1000	Bozic	Inf	unif	wholeT_0.01	S1:CBN, S1:CBN-A, S1:DiP, S1:DiP-A, S1:OT, S1:OT-A, S5:CBN-A, S5:DiP, S5:DiP-A, S5:OT, S5:OT-A
12	Yes	11	11-A	1000	Bozic	Inf	unif	wholeT_0.5	S1:OT, S1:OT-A
13	Yes	11	11-A	1000	exp	0	last	singleC	S1:CBN, S1:OT, S1:OT-A
14	Yes	11	11-A	1000	exp	0	last	wholeT_0.01	S1:DiP-A, S1:OT, S1:OT-A, S5:OT, S5:OT-A
15	Yes	11	11-A	1000	exp	0	last	wholeT_0.5	S1:OT, S1:OT-A
16	Yes	11	11-A	1000	exp	0	unif	singleC	none
17	Yes	11	11-A	1000	exp	0	unif	wholeT_0.01	S1:CBN, S1:CBN-A, S1:OT, S1:OT-A
18	Yes	11	11-A	1000	exp	0	unif	wholeT_0.5	J1:CBN-A, J1:DiP, J1:DiP-A, J1:OT, J5:CBN, J5:CBN-A, J5:DiP, J5:DiP-A, J5:OT, J5:OT-A, S1:CBN-A, S1:DiP, S1:DiP-A, S1:OT, S1:OT-A, S5:CBN, S5:CBN-A, S5:DiP, S5:DiP-A, S5:OT, S5:OT-A
19	Yes	11	11-A	1000	exp	Inf	last	singleC	S1:CBN-A, S1:DiP-A, S1:OT, S1:OT-A

Table 15: *(continued)*

	Conjunction	Drivers	Tree	S.Size	Model	sh	S.Time	S.Type	Best method(s)
20	Yes	11	11-A	1000	exp	Inf	last	wholeT_0.01	J1:DiP, J1:DiP-A, J1:OT, J1:OT-A, S1:DiP, S1:DiP-A, S1:OT, S1:OT-A, S5:DiP, S5:DiP-A, S5:OT, S5:OT-A
21	Yes	11	11-A	1000	exp	Inf	last	wholeT_0.5	S1:CBN, S1:DiP, S1:DiP-A, S1:OT, S1:OT-A, S5:DiP, S5:DiP-A, S5:OT, S5:OT-A
22	Yes	11	11-A	1000	exp	Inf	unif	singleC	S1:CBN-A, S1:OT, S1:OT-A
23	Yes	11	11-A	1000	exp	Inf	unif	wholeT_0.01	S1:CBN, S1:CBN-A, S1:DiP-A, S1:OT, S1:OT-A
24	Yes	11	11-A	1000	exp	Inf	unif	wholeT_0.5	S1:OT, S1:OT-A
25	Yes	11	11-A	1000	McF_4	0	last	singleC	S1:DiP-A, S1:OT, S1:OT-A
26	Yes	11	11-A	1000	McF_4	0	last	wholeT_0.01	S1:DiP-A, S1:OT, S1:OT-A
27	Yes	11	11-A	1000	McF_4	0	last	wholeT_0.5	S1:DiP-A, S1:OT, S1:OT-A
28	Yes	11	11-A	1000	McF_4	0	unif	singleC	S1:DiP, S1:DiP-A, S1:OT, S1:OT-A
29	Yes	11	11-A	1000	McF_4	0	unif	wholeT_0.01	S1:CBN, S1:CBN-A, S1:DiP-A, S1:OT, S1:OT-A
30	Yes	11	11-A	1000	McF_4	0	unif	wholeT_0.5	S1:DiP, S1:DiP-A, S1:OT, S1:OT-A, S5:DiP, S5:OT, S5:OT-A
31	Yes	11	11-A	1000	McF_4	Inf	last	singleC	S1:OT, S1:OT-A
32	Yes	11	11-A	1000	McF_4	Inf	last	wholeT_0.01	S1:OT, S1:OT-A, S5:OT, S5:OT-A
33	Yes	11	11-A	1000	McF_4	Inf	last	wholeT_0.5	S1:DiP-A, S1:OT, S1:OT-A
34	Yes	11	11-A	1000	McF_4	Inf	unif	singleC	S1:OT, S1:OT-A
35	Yes	11	11-A	1000	McF_4	Inf	unif	wholeT_0.01	S1:OT, S1:OT-A
36	Yes	11	11-A	1000	McF_4	Inf	unif	wholeT_0.5	S1:CBN, S1:CBN-A, S1:OT, S1:OT-A
37	Yes	11	11-A	1000	McF_6	0	last	singleC	S1:DiP, S1:DiP-A
38	Yes	11	11-A	1000	McF_6	0	last	wholeT_0.01	S1:DiP, S1:DiP-A, S5:DiP, S5:DiP-A
39	Yes	11	11-A	1000	McF_6	0	last	wholeT_0.5	S1:DiP, S1:DiP-A
40	Yes	11	11-A	1000	McF_6	0	unif	singleC	S1:CBN, S1:CBN-A, S1:DiP, S1:DiP-A, S1:OT, S1:OT-A
41	Yes	11	11-A	1000	McF_6	0	unif	wholeT_0.01	S1:CBN, S1:CBN-A, S1:DiP, S1:DiP-A, S1:OT, S1:OT-A
42	Yes	11	11-A	1000	McF_6	0	unif	wholeT_0.5	S1:CBN, S1:CBN-A, S1:DiP, S1:DiP-A, S1:OT, S1:OT-A
43	Yes	11	11-A	1000	McF_6	Inf	last	singleC	S1:DiP-A, S1:OT, S1:OT-A
44	Yes	11	11-A	1000	McF_6	Inf	last	wholeT_0.01	S1:DiP-A, S5:DiP-A
45	Yes	11	11-A	1000	McF_6	Inf	last	wholeT_0.5	S1:DiP-A, S1:OT, S1:OT-A
46	Yes	11	11-A	1000	McF_6	Inf	unif	singleC	S1:CBN, S1:CBN-A, S1:DiP, S1:DiP-A, S1:OT, S1:OT-A
47	Yes	11	11-A	1000	McF_6	Inf	unif	wholeT_0.01	S1:CBN, S1:CBN-A, S1:DiP, S1:DiP-A, S1:OT, S1:OT-A

Table 15: *(continued)*

	Conjunction	Drivers	Tree	S.Size	Model	sh	S.Time	S.Type	Best method(s)
48	Yes	11	11-A	1000	McF_6	Inf	unif	wholeT_0.5	S1:CBN, S1:CBN-A, S1:OT, S1:OT-A
49	Yes	11	11-A	200	Bozic	0	last	singleC	S1:CBN, S1:CBN-A, S1:OT, S1:OT-A
50	Yes	11	11-A	200	Bozic	0	last	wholeT_0.01	S1:CBN, S1:CBN-A, S1:OT, S1:OT-A
51	Yes	11	11-A	200	Bozic	0	last	wholeT_0.5	S1:CBN, S1:CBN-A, S1:OT, S1:OT-A
52	Yes	11	11-A	200	Bozic	0	unif	singleC	S1:CBN, S1:CBN-A, S1:OT, S1:OT-A
53	Yes	11	11-A	200	Bozic	0	unif	wholeT_0.01	S1:CBN, S1:CBN-A
54	Yes	11	11-A	200	Bozic	0	unif	wholeT_0.5	J1:CBN, J1:CBN-A, J1:DiP, J1:DiP-A, J1:OT, J1:OT-A, J5:DiP-A, J5:OT, S1:CBN, S1:CBN-A, S1:DiP, S1:OT, S1:OT-A, S5:CBN, S5:CBN-A, S5:OT
55	Yes	11	11-A	200	Bozic	Inf	last	singleC	S1:OT, S1:OT-A
56	Yes	11	11-A	200	Bozic	Inf	last	wholeT_0.01	J1:OT, J1:OT-A, S1:OT, S1:OT-A, S5:CBN-A, S5:OT, S5:OT-A
57	Yes	11	11-A	200	Bozic	Inf	last	wholeT_0.5	S1:OT, S1:OT-A
58	Yes	11	11-A	200	Bozic	Inf	unif	singleC	S1:CBN, S1:CBN-A, S1:OT, S1:OT-A
59	Yes	11	11-A	200	Bozic	Inf	unif	wholeT_0.01	S1:CBN, S1:CBN-A, S1:OT, S1:OT-A, S5:OT, S5:OT-A
60	Yes	11	11-A	200	Bozic	Inf	unif	wholeT_0.5	S1:OT, S1:OT-A
61	Yes	11	11-A	200	exp	0	last	singleC	S1:OT, S1:OT-A
62	Yes	11	11-A	200	exp	0	last	wholeT_0.01	S1:CBN-A, S1:OT, S1:OT-A, S5:OT, S5:OT-A
63	Yes	11	11-A	200	exp	0	last	wholeT_0.5	S1:CBN-A, S1:OT, S1:OT-A
64	Yes	11	11-A	200	exp	0	unif	singleC	J1:CBN, J1:CBN-A, J1:DiP, J1:DiP-A, J1:OT, J1:OT-A, J5:CBN, J5:CBN-A, J5:DiP, J5:DiP-A, J5:OT, J5:OT-A, S1:CBN, S1:CBN-A, S1:DiP, S1:DiP-A, S1:OT, S1:OT-A, S5:CBN, S5:CBN-A, S5:DiP-A, S5:OT, S5:OT-A
65	Yes	11	11-A	200	exp	0	unif	wholeT_0.01	S1:CBN, S1:CBN-A, S1:OT, S1:OT-A
66	Yes	11	11-A	200	exp	0	unif	wholeT_0.5	J1:CBN, J1:CBN-A, J1:DiP, J1:DiP-A, J1:OT, J1:OT-A, J5:CBN, J5:CBN-A, J5:DiP, J5:OT, J5:OT-A, S1:CBN, S1:OT, S1:OT-A, S5:CBN, S5:CBN-A, S5:DiP, S5:DiP-A, S5:OT, S5:OT-A
67	Yes	11	11-A	200	exp	Inf	last	singleC	S1:OT, S1:OT-A, S5:OT, S5:OT-A
68	Yes	11	11-A	200	exp	Inf	last	wholeT_0.01	J1:CBN-A, J1:OT, J1:OT-A, S1:CBN, S1:CBN-A, S1:OT, S1:OT-A, S5:OT, S5:OT-A
69	Yes	11	11-A	200	exp	Inf	last	wholeT_0.5	S1:CBN, S1:CBN-A, S1:OT-A
70	Yes	11	11-A	200	exp	Inf	unif	singleC	S1:OT, S1:OT-A

Table 15: *(continued)*

	Conjunction	Drivers	Tree	S.Size	Model	sh	S.Time	S.Type	Best method(s)
71	Yes	11	11-A	200	exp	Inf	unif	wholeT_0.01	S1:CBN, S1:CBN-A, S1:OT, S1:OT-A
72	Yes	11	11-A	200	exp	Inf	unif	wholeT_0.5	S1:CBN, S1:CBN-A, S1:OT, S1:OT-A
73	Yes	11	11-A	200	McF_4	0	last	singleC	S1:OT, S1:OT-A
74	Yes	11	11-A	200	McF_4	0	last	wholeT_0.01	S1:CBN, S1:OT, S1:OT-A
75	Yes	11	11-A	200	McF_4	0	last	wholeT_0.5	S1:OT, S1:OT-A
76	Yes	11	11-A	200	McF_4	0	unif	singleC	S1:CBN, S1:CBN-A, S1:OT, S1:OT-A
77	Yes	11	11-A	200	McF_4	0	unif	wholeT_0.01	S1:CBN, S1:CBN-A, S1:OT, S1:OT-A
78	Yes	11	11-A	200	McF_4	0	unif	wholeT_0.5	S1:OT, S1:OT-A
79	Yes	11	11-A	200	McF_4	Inf	last	singleC	S1:OT, S1:OT-A
80	Yes	11	11-A	200	McF_4	Inf	last	wholeT_0.01	S1:OT, S1:OT-A, S5:OT, S5:OT-A
81	Yes	11	11-A	200	McF_4	Inf	last	wholeT_0.5	S1:OT, S1:OT-A
82	Yes	11	11-A	200	McF_4	Inf	unif	singleC	S1:OT, S1:OT-A
83	Yes	11	11-A	200	McF_4	Inf	unif	wholeT_0.01	S1:OT, S1:OT-A
84	Yes	11	11-A	200	McF_4	Inf	unif	wholeT_0.5	S1:OT, S1:OT-A
85	Yes	11	11-A	200	McF_6	0	last	singleC	S1:OT, S1:OT-A, S5:OT, S5:OT-A
86	Yes	11	11-A	200	McF_6	0	last	wholeT_0.01	S1:OT, S1:OT-A, S5:OT, S5:OT-A
87	Yes	11	11-A	200	McF_6	0	last	wholeT_0.5	S1:OT, S1:OT-A
88	Yes	11	11-A	200	McF_6	0	unif	singleC	S1:CBN, S1:OT, S1:OT-A
89	Yes	11	11-A	200	McF_6	0	unif	wholeT_0.01	S1:CBN, S1:CBN-A, S1:OT, S1:OT-A
90	Yes	11	11-A	200	McF_6	0	unif	wholeT_0.5	S1:CBN, S1:CBN-A, S1:OT, S1:OT-A
91	Yes	11	11-A	200	McF_6	Inf	last	singleC	S1:OT, S1:OT-A
92	Yes	11	11-A	200	McF_6	Inf	last	wholeT_0.01	S1:OT, S1:OT-A, S5:OT, S5:OT-A
93	Yes	11	11-A	200	McF_6	Inf	last	wholeT_0.5	S1:OT, S1:OT-A
94	Yes	11	11-A	200	McF_6	Inf	unif	singleC	S1:CBN, S1:OT, S1:OT-A
95	Yes	11	11-A	200	McF_6	Inf	unif	wholeT_0.01	S1:OT, S1:OT-A
96	Yes	11	11-A	200	McF_6	Inf	unif	wholeT_0.5	S1:CBN-A, S1:OT, S1:OT-A
97	Yes	11	11-A	100	Bozic	0	last	singleC	S1:CBN, S1:CBN-A, S1:OT, S1:OT-A
98	Yes	11	11-A	100	Bozic	0	last	wholeT_0.01	S1:CBN, S1:OT, S1:OT-A
99	Yes	11	11-A	100	Bozic	0	last	wholeT_0.5	S1:CBN-A, S1:OT, S1:OT-A
100	Yes	11	11-A	100	Bozic	0	unif	singleC	S1:CBN, S1:CBN-A, S1:OT, S1:OT-A
101	Yes	11	11-A	100	Bozic	0	unif	wholeT_0.01	S1:CBN, S1:CBN-A
102	Yes	11	11-A	100	Bozic	0	unif	wholeT_0.5	S1:CBN, S1:CBN-A, S1:OT, S1:OT-A
103	Yes	11	11-A	100	Bozic	Inf	last	singleC	S1:CBN-A, S1:OT, S1:OT-A
104	Yes	11	11-A	100	Bozic	Inf	last	wholeT_0.01	S1:OT, S1:OT-A, S5:CBN-A, S5:OT, S5:OT-A
105	Yes	11	11-A	100	Bozic	Inf	last	wholeT_0.5	S1:CBN, S1:CBN-A, S1:OT, S1:OT-A
106	Yes	11	11-A	100	Bozic	Inf	unif	singleC	S1:CBN, S1:CBN-A, S1:OT, S1:OT-A
107	Yes	11	11-A	100	Bozic	Inf	unif	wholeT_0.01	J1:CBN, J1:CBN-A, J1:OT, J1:OT-A, S1:CBN, S1:CBN-A, S1:OT, S1:OT-A, S5:CBN, S5:CBN-A, S5:OT, S5:OT-A

Table 15: *(continued)*

	Conjunction	Drivers	Tree	S.Size	Model	sh	S.Time	S.Type	Best method(s)
108	Yes	11	11-A	100	Bozic	Inf	unif	wholeT_0.5	S1:CBN, S1:CBN-A, S1:OT, S1:OT-A
109	Yes	11	11-A	100	exp	0	last	singleC	S1:OT, S1:OT-A
110	Yes	11	11-A	100	exp	0	last	wholeT_0.01	S1:CBN, S1:CBN-A, S1:OT, S1:OT-A, S5:OT
111	Yes	11	11-A	100	exp	0	last	wholeT_0.5	S1:OT, S1:OT-A
112	Yes	11	11-A	100	exp	0	unif	singleC	J1:CBN, J1:OT, J1:OT-A, J5:OT, S1:CBN, S1:CBN-A, S1:OT, S1:OT-A, S5:CBN
113	Yes	11	11-A	100	exp	0	unif	wholeT_0.01	S1:CBN, S1:CBN-A, S1:OT, S1:OT-A
114	Yes	11	11-A	100	exp	0	unif	wholeT_0.5	J1:CBN, J1:CBN-A, J1:DiP, J1:OT, J1:OT-A, J5:CBN, J5:CBN-A, J5:DiP, J5:DiP-A, J5:OT, S1:CBN, S1:CBN-A, S1:DiP, S1:DiP-A, S1:OT, S1:OT-A, S5:CBN, S5:CBN-A, S5:DiP, S5:DiP-A, S5:OT-A
115	Yes	11	11-A	100	exp	Inf	last	singleC	S1:OT, S1:OT-A
116	Yes	11	11-A	100	exp	Inf	last	wholeT_0.01	J1:OT, J1:OT-A, S1:CBN-A, S1:OT, S1:OT-A, S5:CBN-A, S5:OT, S5:OT-A
117	Yes	11	11-A	100	exp	Inf	last	wholeT_0.5	S1:OT, S1:OT-A
118	Yes	11	11-A	100	exp	Inf	unif	singleC	S1:CBN, S1:OT, S1:OT-A
119	Yes	11	11-A	100	exp	Inf	unif	wholeT_0.01	J1:CBN, J1:CBN-A, S1:CBN, S1:CBN-A, S1:OT, S1:OT-A
120	Yes	11	11-A	100	exp	Inf	unif	wholeT_0.5	S1:CBN-A, S1:OT, S1:OT-A
121	Yes	11	11-A	100	McF_4	0	last	singleC	S1:CBN, S1:OT, S1:OT-A
122	Yes	11	11-A	100	McF_4	0	last	wholeT_0.01	S1:OT, S1:OT-A, S5:OT-A
123	Yes	11	11-A	100	McF_4	0	last	wholeT_0.5	S1:OT, S1:OT-A
124	Yes	11	11-A	100	McF_4	0	unif	singleC	S1:CBN, S1:CBN-A, S1:OT, S1:OT-A
125	Yes	11	11-A	100	McF_4	0	unif	wholeT_0.01	S1:CBN, S1:CBN-A, S1:OT, S1:OT-A
126	Yes	11	11-A	100	McF_4	0	unif	wholeT_0.5	S1:CBN, S1:CBN-A, S1:OT, S1:OT-A
127	Yes	11	11-A	100	McF_4	Inf	last	singleC	S1:OT, S1:OT-A
128	Yes	11	11-A	100	McF_4	Inf	last	wholeT_0.01	S1:OT, S1:OT-A, S5:OT, S5:OT-A
129	Yes	11	11-A	100	McF_4	Inf	last	wholeT_0.5	S1:OT, S1:OT-A
130	Yes	11	11-A	100	McF_4	Inf	unif	singleC	S1:OT, S1:OT-A
131	Yes	11	11-A	100	McF_4	Inf	unif	wholeT_0.01	S1:CBN, S1:OT, S1:OT-A
132	Yes	11	11-A	100	McF_4	Inf	unif	wholeT_0.5	S1:CBN, S1:OT, S1:OT-A
133	Yes	11	11-A	100	McF_6	0	last	singleC	S1:OT, S1:OT-A
134	Yes	11	11-A	100	McF_6	0	last	wholeT_0.01	S1:OT, S1:OT-A, S5:OT-A
135	Yes	11	11-A	100	McF_6	0	last	wholeT_0.5	S1:OT, S1:OT-A
136	Yes	11	11-A	100	McF_6	0	unif	singleC	S1:CBN, S1:CBN-A, S1:OT, S1:OT-A
137	Yes	11	11-A	100	McF_6	0	unif	wholeT_0.01	S1:CBN, S1:CBN-A, S1:OT, S1:OT-A

Table 15: *(continued)*

	Conjunction	Drivers	Tree	S.Size	Model	sh	S.Time	S.Type	Best method(s)
138	Yes	11	11-A	100	McF_6	0	unif	wholeT_0.5	S1:CBN, S1:OT, S1:OT-A
139	Yes	11	11-A	100	McF_6	Inf	last	singleC	S1:OT, S1:OT-A, S5:OT, S5:OT-A
140	Yes	11	11-A	100	McF_6	Inf	last	wholeT_0.01	S1:OT, S1:OT-A, S5:OT, S5:OT-A
141	Yes	11	11-A	100	McF_6	Inf	last	wholeT_0.5	S1:OT, S1:OT-A
142	Yes	11	11-A	100	McF_6	Inf	unif	singleC	S1:CBN, S1:OT, S1:OT-A
143	Yes	11	11-A	100	McF_6	Inf	unif	wholeT_0.01	S1:CBN, S1:CBN-A, S1:OT, S1:OT-A
144	Yes	11	11-A	100	McF_6	Inf	unif	wholeT_0.5	S1:CBN, S1:CBN-A, S1:OT, S1:OT-A
145	Yes	9	9-A	1000	Bozic	0	last	singleC	S1:OT, S1:OT-A
146	Yes	9	9-A	1000	Bozic	0	last	wholeT_0.01	S1:DiP, S1:DiP-A, S1:OT, S1:OT-A, S5:DiP, S5:DiP-A, S5:OT, S5:OT-A
147	Yes	9	9-A	1000	Bozic	0	last	wholeT_0.5	S1:OT, S1:OT-A
148	Yes	9	9-A	1000	Bozic	0	unif	singleC	none
149	Yes	9	9-A	1000	Bozic	0	unif	wholeT_0.01	S1:CBN, S1:CBN-A
150	Yes	9	9-A	1000	Bozic	0	unif	wholeT_0.5	J1:CBN, J1:CBN-A, J1:DiP, J1:DiP-A, J1:OT, J5:CBN, J5:CBN-A, J5:DiP, J5:DiP-A, J5:OT, J5:OT-A, S1:CBN, S1:CBN-A, S1:DiP, S1:DiP-A, S1:OT, S1:OT-A, S5:CBN, S5:CBN-A, S5:DiP, S5:DiP-A, S5:OT, S5:OT-A
151	Yes	9	9-A	1000	Bozic	Inf	last	singleC	S1:CBN, S1:CBN-A, S1:OT, S1:OT-A, S5:CBN-A
152	Yes	9	9-A	1000	Bozic	Inf	last	wholeT_0.01	J1:CBN-A, S1:CBN, S1:CBN-A, S1:DiP, S1:DiP-A, S1:OT, S1:OT-A, S5:CBN, S5:CBN-A, S5:DiP, S5:DiP-A, S5:OT-A
153	Yes	9	9-A	1000	Bozic	Inf	last	wholeT_0.5	S1:CBN, S1:CBN-A, S1:OT, S1:OT-A
154	Yes	9	9-A	1000	Bozic	Inf	unif	singleC	S1:CBN, S1:CBN-A, S1:OT, S1:OT-A
155	Yes	9	9-A	1000	Bozic	Inf	unif	wholeT_0.01	S1:CBN, S1:CBN-A, S1:DiP, S1:DiP-A, S1:OT, S1:OT-A, S5:CBN, S5:CBN-A, S5:DiP, S5:DiP-A, S5:OT, S5:OT-A
156	Yes	9	9-A	1000	Bozic	Inf	unif	wholeT_0.5	S1:CBN, S1:OT, S1:OT-A
157	Yes	9	9-A	1000	exp	0	last	singleC	J1:OT-A, J5:CBN, J5:DiP, J5:OT, S1:CBN, S1:CBN-A, S1:DiP, S1:DiP-A, S1:OT, S1:OT-A, S5:DiP-A, S5:OT
158	Yes	9	9-A	1000	exp	0	last	wholeT_0.01	S1:CBN-A, S1:OT, S1:OT-A
159	Yes	9	9-A	1000	exp	0	last	wholeT_0.5	none
160	Yes	9	9-A	1000	exp	0	unif	singleC	none
161	Yes	9	9-A	1000	exp	0	unif	wholeT_0.01	J1:DiP, J5:CBN-A, S1:CBN, S1:CBN-A, S1:DiP, S1:DiP-A, S1:OT, S1:OT-A
162	Yes	9	9-A	1000	exp	0	unif	wholeT_0.5	none
163	Yes	9	9-A	1000	exp	Inf	last	singleC	S1:CBN, S1:CBN-A, S1:OT, S1:OT-A

Table 15: *(continued)*

	Conjunction	Drivers	Tree	S.Size	Model	sh	S.Time	S.Type	Best method(s)
164	Yes	9	9-A	1000	exp	Inf	last	wholeT_0.01	S1:CBN, S1:CBN-A, S1:DiP, S1:DiP-A, S1:OT, S1:OT-A, S5:CBN, S5:CBN-A, S5:DiP, S5:DiP-A, S5:OT, S5:OT-A
165	Yes	9	9-A	1000	exp	Inf	last	wholeT_0.5	S1:CBN, S1:CBN-A, S1:OT, S1:OT-A
166	Yes	9	9-A	1000	exp	Inf	unif	singleC	S1:CBN, S1:CBN-A, S1:OT, S1:OT-A
167	Yes	9	9-A	1000	exp	Inf	unif	wholeT_0.01	S1:CBN, S1:CBN-A, S1:OT, S1:OT-A
168	Yes	9	9-A	1000	exp	Inf	unif	wholeT_0.5	S1:CBN, S1:OT, S1:OT-A
169	Yes	9	9-A	1000	McF_4	0	last	singleC	S1:DiP, S1:DiP-A, S1:OT, S1:OT-A
170	Yes	9	9-A	1000	McF_4	0	last	wholeT_0.01	S1:CBN, S1:CBN-A, S1:DiP, S1:DiP-A, S1:OT, S1:OT-A
171	Yes	9	9-A	1000	McF_4	0	last	wholeT_0.5	S1:DiP, S1:DiP-A, S1:OT, S1:OT-A
172	Yes	9	9-A	1000	McF_4	0	unif	singleC	S1:CBN, S1:CBN-A, S1:DiP, S1:DiP-A, S1:OT, S1:OT-A
173	Yes	9	9-A	1000	McF_4	0	unif	wholeT_0.01	S1:CBN, S1:CBN-A, S1:DiP, S1:DiP-A, S1:OT-A
174	Yes	9	9-A	1000	McF_4	0	unif	wholeT_0.5	S1:CBN, S1:CBN-A, S1:DiP, S1:DiP-A, S1:OT, S1:OT-A
175	Yes	9	9-A	1000	McF_4	Inf	last	singleC	S1:OT, S1:OT-A
176	Yes	9	9-A	1000	McF_4	Inf	last	wholeT_0.01	S1:OT-A
177	Yes	9	9-A	1000	McF_4	Inf	last	wholeT_0.5	S1:CBN, S1:DiP, S1:DiP-A, S1:OT, S1:OT-A
178	Yes	9	9-A	1000	McF_4	Inf	unif	singleC	S1:CBN, S1:CBN-A, S1:OT, S1:OT-A
179	Yes	9	9-A	1000	McF_4	Inf	unif	wholeT_0.01	S1:CBN, S1:CBN-A, S1:OT, S1:OT-A
180	Yes	9	9-A	1000	McF_4	Inf	unif	wholeT_0.5	S1:CBN, S1:CBN-A, S1:OT, S1:OT-A
181	Yes	9	9-A	1000	McF_6	0	last	singleC	S1:DiP-A, S1:OT-A, S5:DiP-A
182	Yes	9	9-A	1000	McF_6	0	last	wholeT_0.01	S1:DiP-A, S5:DiP-A
183	Yes	9	9-A	1000	McF_6	0	last	wholeT_0.5	S1:DiP-A, S1:OT-A, S5:DiP-A, S5:OT-A
184	Yes	9	9-A	1000	McF_6	0	unif	singleC	S1:CBN, S1:CBN-A, S1:DiP, S1:DiP-A
185	Yes	9	9-A	1000	McF_6	0	unif	wholeT_0.01	S1:CBN, S1:CBN-A, S1:DiP, S1:DiP-A
186	Yes	9	9-A	1000	McF_6	0	unif	wholeT_0.5	S1:CBN, S1:CBN-A, S1:DiP, S1:DiP-A
187	Yes	9	9-A	1000	McF_6	Inf	last	singleC	S1:DiP-A, S1:OT-A, S5:DiP-A, S5:OT-A
188	Yes	9	9-A	1000	McF_6	Inf	last	wholeT_0.01	S1:OT-A, S5:OT-A
189	Yes	9	9-A	1000	McF_6	Inf	last	wholeT_0.5	S1:DiP-A, S1:OT-A, S5:DiP-A, S5:OT-A
190	Yes	9	9-A	1000	McF_6	Inf	unif	singleC	S1:CBN, S1:CBN-A
191	Yes	9	9-A	1000	McF_6	Inf	unif	wholeT_0.01	S1:CBN, S1:CBN-A, S1:DiP, S1:DiP-A, S1:OT, S1:OT-A
192	Yes	9	9-A	1000	McF_6	Inf	unif	wholeT_0.5	S1:CBN, S1:CBN-A
193	Yes	9	9-A	200	Bozic	0	last	singleC	S1:CBN, S1:OT, S1:OT-A
194	Yes	9	9-A	200	Bozic	0	last	wholeT_0.01	S1:CBN, S1:CBN-A, S1:OT, S1:OT-A
195	Yes	9	9-A	200	Bozic	0	last	wholeT_0.5	S1:OT, S1:OT-A

Table 15: *(continued)*

	Conjunction	Drivers	Tree	S.Size	Model	sh	S.Time	S.Type	Best method(s)
196	Yes	9	9-A	200	Bozic	0	unif	singleC	S1:CBN, S1:CBN-A, S1:OT, S1:OT-A
197	Yes	9	9-A	200	Bozic	0	unif	wholeT_0.01	S1:CBN, S1:CBN-A, S1:OT-A
198	Yes	9	9-A	200	Bozic	0	unif	wholeT_0.5	J1:CBN, J1:CBN-A, J1:DiP, J1:DiP-A, J1:OT, J1:OT-A, J5:CBN, J5:CBN-A, J5:DiP, J5:DiP-A, J5:OT, J5:OT-A, S1:CBN, S1:CBN-A, S1:DiP, S1:DiP-A, S1:OT, S1:OT-A, S5:CBN, S5:DiP, S5:DiP-A, S5:OT, S5:OT-A
199	Yes	9	9-A	200	Bozic	Inf	last	singleC	S1:CBN, S1:CBN-A
200	Yes	9	9-A	200	Bozic	Inf	last	wholeT_0.01	S1:CBN, S1:CBN-A, S1:OT-A, S5:CBN, S5:CBN-A, S5:OT, S5:OT-A
201	Yes	9	9-A	200	Bozic	Inf	last	wholeT_0.5	S1:CBN, S1:CBN-A, S1:OT, S1:OT-A
202	Yes	9	9-A	200	Bozic	Inf	unif	singleC	S1:CBN, S1:CBN-A, S1:OT, S1:OT-A
203	Yes	9	9-A	200	Bozic	Inf	unif	wholeT_0.01	J1:CBN, J1:CBN-A, S1:CBN, S1:CBN-A, S1:OT, S1:OT-A, S5:CBN, S5:CBN-A, S5:OT, S5:OT-A
204	Yes	9	9-A	200	Bozic	Inf	unif	wholeT_0.5	S1:CBN, S1:CBN-A, S1:OT, S1:OT-A
205	Yes	9	9-A	200	exp	0	last	singleC	S1:CBN, S1:OT, S1:OT-A
206	Yes	9	9-A	200	exp	0	last	wholeT_0.01	S1:CBN-A, S1:OT, S1:OT-A
207	Yes	9	9-A	200	exp	0	last	wholeT_0.5	S1:CBN, S1:CBN-A, S1:OT, S1:OT-A
208	Yes	9	9-A	200	exp	0	unif	singleC	none
209	Yes	9	9-A	200	exp	0	unif	wholeT_0.01	S1:CBN, S1:CBN-A, S1:OT, S1:OT-A
210	Yes	9	9-A	200	exp	0	unif	wholeT_0.5	J1:CBN-A, J1:DiP, J1:DiP-A, J1:OT-A, J5:DiP, J5:DiP-A, J5:OT, J5:OT-A, S1:CBN, S1:CBN-A, S1:DiP, S1:DiP-A, S1:OT, S1:OT-A, S5:CBN, S5:CBN-A, S5:DiP-A, S5:OT
211	Yes	9	9-A	200	exp	Inf	last	singleC	S1:CBN, S1:CBN-A, S1:OT, S1:OT-A
212	Yes	9	9-A	200	exp	Inf	last	wholeT_0.01	S1:CBN, S1:CBN-A, S1:OT-A, S5:CBN, S5:CBN-A
213	Yes	9	9-A	200	exp	Inf	last	wholeT_0.5	S1:CBN, S1:CBN-A, S1:OT, S1:OT-A
214	Yes	9	9-A	200	exp	Inf	unif	singleC	S1:CBN, S1:CBN-A, S1:OT, S1:OT-A
215	Yes	9	9-A	200	exp	Inf	unif	wholeT_0.01	S1:CBN, S1:CBN-A, S1:OT-A
216	Yes	9	9-A	200	exp	Inf	unif	wholeT_0.5	S1:CBN, S1:CBN-A, S1:OT, S1:OT-A
217	Yes	9	9-A	200	McF_4	0	last	singleC	S1:CBN, S1:CBN-A, S1:OT, S1:OT-A
218	Yes	9	9-A	200	McF_4	0	last	wholeT_0.01	S1:CBN, S1:CBN-A, S1:OT, S1:OT-A
219	Yes	9	9-A	200	McF_4	0	last	wholeT_0.5	S1:CBN, S1:CBN-A, S1:OT, S1:OT-A
220	Yes	9	9-A	200	McF_4	0	unif	singleC	S1:CBN, S1:CBN-A, S1:OT, S1:OT-A
221	Yes	9	9-A	200	McF_4	0	unif	wholeT_0.01	S1:CBN, S1:CBN-A
222	Yes	9	9-A	200	McF_4	0	unif	wholeT_0.5	S1:CBN, S1:CBN-A, S1:OT, S1:OT-A

Table 15: *(continued)*

	Conjunction	Drivers	Tree	S.Size	Model	sh	S.Time	S.Type	Best method(s)
223	Yes	9	9-A	200	McF_4	Inf	last	singleC	S1:CBN-A, S1:OT, S1:OT-A
224	Yes	9	9-A	200	McF_4	Inf	last	wholeT_0.01	S1:OT-A
225	Yes	9	9-A	200	McF_4	Inf	last	wholeT_0.5	S1:CBN-A, S1:OT, S1:OT-A
226	Yes	9	9-A	200	McF_4	Inf	unif	singleC	S1:CBN, S1:CBN-A, S1:OT, S1:OT-A
227	Yes	9	9-A	200	McF_4	Inf	unif	wholeT_0.01	S1:CBN, S1:CBN-A, S1:OT, S1:OT-A
228	Yes	9	9-A	200	McF_4	Inf	unif	wholeT_0.5	S1:CBN, S1:CBN-A, S1:OT, S1:OT-A
229	Yes	9	9-A	200	McF_6	0	last	singleC	S1:OT-A
230	Yes	9	9-A	200	McF_6	0	last	wholeT_0.01	S1:OT-A, S5:OT-A
231	Yes	9	9-A	200	McF_6	0	last	wholeT_0.5	S1:OT-A, S5:OT-A
232	Yes	9	9-A	200	McF_6	0	unif	singleC	S1:CBN, S1:CBN-A, S1:OT, S1:OT-A
233	Yes	9	9-A	200	McF_6	0	unif	wholeT_0.01	S1:CBN, S1:CBN-A
234	Yes	9	9-A	200	McF_6	0	unif	wholeT_0.5	S1:CBN, S1:CBN-A, S1:OT-A
235	Yes	9	9-A	200	McF_6	Inf	last	singleC	S1:OT-A, S5:OT-A
236	Yes	9	9-A	200	McF_6	Inf	last	wholeT_0.01	S1:OT-A, S5:OT-A
237	Yes	9	9-A	200	McF_6	Inf	last	wholeT_0.5	S1:OT-A, S5:OT-A
238	Yes	9	9-A	200	McF_6	Inf	unif	singleC	S1:CBN, S1:CBN-A
239	Yes	9	9-A	200	McF_6	Inf	unif	wholeT_0.01	S1:CBN, S1:CBN-A, S1:OT, S1:OT-A
240	Yes	9	9-A	200	McF_6	Inf	unif	wholeT_0.5	S1:CBN, S1:CBN-A, S1:OT, S1:OT-A
241	Yes	9	9-A	100	Bozic	0	last	singleC	S1:CBN, S1:CBN-A, S1:OT, S1:OT-A
242	Yes	9	9-A	100	Bozic	0	last	wholeT_0.01	S1:CBN, S1:CBN-A, S1:OT, S1:OT-A
243	Yes	9	9-A	100	Bozic	0	last	wholeT_0.5	S1:CBN, S1:CBN-A, S1:OT, S1:OT-A
244	Yes	9	9-A	100	Bozic	0	unif	singleC	S1:CBN, S1:CBN-A, S1:OT, S1:OT-A
245	Yes	9	9-A	100	Bozic	0	unif	wholeT_0.01	S1:CBN, S1:CBN-A
246	Yes	9	9-A	100	Bozic	0	unif	wholeT_0.5	J1:CBN-A, J1:DiP, J1:DiP-A, J1:OT, J5:CBN, J5:CBN-A, J5:DiP-A, J5:OT-A, S1:CBN, S1:CBN-A, S1:OT, S1:OT-A, S5:OT-A
247	Yes	9	9-A	100	Bozic	Inf	last	singleC	S1:CBN, S1:CBN-A, S1:OT, S1:OT-A
248	Yes	9	9-A	100	Bozic	Inf	last	wholeT_0.01	J1:CBN, J1:CBN-A, S1:CBN, S1:CBN-A, S1:OT, S1:OT-A, S5:CBN, S5:CBN-A, S5:OT, S5:OT-A
249	Yes	9	9-A	100	Bozic	Inf	last	wholeT_0.5	S1:CBN, S1:CBN-A, S1:OT, S1:OT-A, S5:CBN-A, S5:OT-A
250	Yes	9	9-A	100	Bozic	Inf	unif	singleC	S1:CBN, S1:CBN-A, S1:OT, S1:OT-A
251	Yes	9	9-A	100	Bozic	Inf	unif	wholeT_0.01	S1:CBN, S1:CBN-A, S1:OT, S1:OT-A
252	Yes	9	9-A	100	Bozic	Inf	unif	wholeT_0.5	S1:CBN, S1:CBN-A, S1:OT, S1:OT-A
253	Yes	9	9-A	100	exp	0	last	singleC	S1:CBN, S1:CBN-A, S1:OT, S1:OT-A
254	Yes	9	9-A	100	exp	0	last	wholeT_0.01	S1:CBN-A, S1:OT, S1:OT-A
255	Yes	9	9-A	100	exp	0	last	wholeT_0.5	S1:OT, S1:OT-A

Table 15: *(continued)*

	Conjunction	Drivers	Tree	S.Size	Model	sh	S.Time	S.Type	Best method(s)
256	Yes	9	9-A	100	exp	0	unif	singleC	J1:CBN, J1:CBN-A, J1:DiP, J1:OT, J1:OT-A, S1:CBN, S1:CBN-A, S1:OT, S1:OT-A, S5:CBN, S5:DiP, S5:OT-A
257	Yes	9	9-A	100	exp	0	unif	wholeT_0.01	S1:CBN, S1:CBN-A, S1:OT, S1:OT-A
258	Yes	9	9-A	100	exp	0	unif	wholeT_0.5	J1:CBN, J1:CBN-A, J1:DiP, J1:DiP-A, J1:OT, J1:OT-A, J5:CBN, J5:CBN-A, J5:DiP, J5:OT, J5:OT-A, S1:CBN, S1:CBN-A, S1:DiP, S1:DiP-A, S1:OT, S1:OT-A, S5:CBN, S5:CBN-A, S5:DiP, S5:DiP-A, S5:OT, S5:OT-A
259	Yes	9	9-A	100	exp	Inf	last	singleC	S1:CBN, S1:CBN-A, S1:OT, S1:OT-A
260	Yes	9	9-A	100	exp	Inf	last	wholeT_0.01	S1:CBN, S1:CBN-A, S1:OT, S1:OT-A, S5:CBN, S5:CBN-A, S5:OT, S5:OT-A
261	Yes	9	9-A	100	exp	Inf	last	wholeT_0.5	S1:CBN, S1:CBN-A, S1:OT, S1:OT-A
262	Yes	9	9-A	100	exp	Inf	unif	singleC	S1:CBN, S1:CBN-A, S1:OT, S1:OT-A
263	Yes	9	9-A	100	exp	Inf	unif	wholeT_0.01	S1:CBN, S1:CBN-A
264	Yes	9	9-A	100	exp	Inf	unif	wholeT_0.5	S1:CBN, S1:CBN-A, S1:OT, S1:OT-A
265	Yes	9	9-A	100	McF_4	0	last	singleC	S1:CBN, S1:CBN-A, S1:OT, S1:OT-A
266	Yes	9	9-A	100	McF_4	0	last	wholeT_0.01	S1:CBN, S1:CBN-A, S1:OT, S1:OT-A
267	Yes	9	9-A	100	McF_4	0	last	wholeT_0.5	S1:CBN, S1:CBN-A, S1:OT, S1:OT-A
268	Yes	9	9-A	100	McF_4	0	unif	singleC	S1:CBN, S1:CBN-A, S1:OT, S1:OT-A
269	Yes	9	9-A	100	McF_4	0	unif	wholeT_0.01	S1:CBN, S1:CBN-A
270	Yes	9	9-A	100	McF_4	0	unif	wholeT_0.5	S1:CBN, S1:CBN-A, S1:OT, S1:OT-A
271	Yes	9	9-A	100	McF_4	Inf	last	singleC	S1:CBN, S1:CBN-A, S1:OT, S1:OT-A
272	Yes	9	9-A	100	McF_4	Inf	last	wholeT_0.01	S1:OT-A, S5:OT-A
273	Yes	9	9-A	100	McF_4	Inf	last	wholeT_0.5	S1:CBN, S1:CBN-A, S1:OT, S1:OT-A
274	Yes	9	9-A	100	McF_4	Inf	unif	singleC	S1:CBN, S1:CBN-A, S1:OT, S1:OT-A
275	Yes	9	9-A	100	McF_4	Inf	unif	wholeT_0.01	S1:CBN, S1:CBN-A, S1:OT, S1:OT-A
276	Yes	9	9-A	100	McF_4	Inf	unif	wholeT_0.5	S1:CBN, S1:CBN-A, S1:OT, S1:OT-A
277	Yes	9	9-A	100	McF_6	0	last	singleC	S1:OT-A
278	Yes	9	9-A	100	McF_6	0	last	wholeT_0.01	S1:OT-A, S5:OT-A
279	Yes	9	9-A	100	McF_6	0	last	wholeT_0.5	S1:OT-A, S5:OT-A
280	Yes	9	9-A	100	McF_6	0	unif	singleC	S1:CBN, S1:CBN-A
281	Yes	9	9-A	100	McF_6	0	unif	wholeT_0.01	S1:CBN, S1:CBN-A
282	Yes	9	9-A	100	McF_6	0	unif	wholeT_0.5	S1:CBN, S1:CBN-A, S1:OT-A
283	Yes	9	9-A	100	McF_6	Inf	last	singleC	S1:OT-A
284	Yes	9	9-A	100	McF_6	Inf	last	wholeT_0.01	S1:OT-A, S5:OT-A
285	Yes	9	9-A	100	McF_6	Inf	last	wholeT_0.5	S1:OT-A, S5:OT-A
286	Yes	9	9-A	100	McF_6	Inf	unif	singleC	S1:CBN, S1:CBN-A
287	Yes	9	9-A	100	McF_6	Inf	unif	wholeT_0.01	S1:CBN, S1:CBN-A

Table 15: *(continued)*

	Conjunction	Drivers	Tree	S.Size	Model	sh	S.Time	S.Type	Best method(s)
288	Yes	9	9-A	100	McF_6	Inf	unif	wholeT_0.5	S1:CBN, S1:CBN-A
289	Yes	7	7-A	1000	Bozic	0	last	singleC	S1:OT-A, S5:OT-A
290	Yes	7	7-A	1000	Bozic	0	last	wholeT_0.01	J1:DiP-A, J1:OT-A, J5:DiP-A, J5:OT-A, S1:DiP-A, S1:OT-A, S5:DiP-A, S5:OT-A
291	Yes	7	7-A	1000	Bozic	0	last	wholeT_0.5	S1:OT-A
292	Yes	7	7-A	1000	Bozic	0	unif	singleC	S1:CBN-A, S1:OT, S1:OT-A
293	Yes	7	7-A	1000	Bozic	0	unif	wholeT_0.01	S1:CBN, S1:CBN-A, S1:DiP, S1:DiP-A, S1:OT, S1:OT-A
294	Yes	7	7-A	1000	Bozic	0	unif	wholeT_0.5	J1:CBN, J1:CBN-A, J1:OT, J1:OT-A, S1:CBN, S1:CBN-A, S1:DiP-A, S1:OT, S1:OT-A, S5:OT-A
295	Yes	7	7-A	1000	Bozic	Inf	last	singleC	J1:DiP-A, J1:OT-A, J5:DiP-A, J5:OT-A, S1:DiP-A, S1:OT-A, S5:DiP-A, S5:OT-A
296	Yes	7	7-A	1000	Bozic	Inf	last	wholeT_0.01	J1:DiP-A, J1:OT-A, J5:DiP-A, J5:OT-A, S1:DiP-A, S1:OT-A, S5:DiP-A, S5:OT-A
297	Yes	7	7-A	1000	Bozic	Inf	last	wholeT_0.5	J1:DiP-A, J1:OT-A, J5:DiP-A, J5:OT-A, S1:DiP-A, S1:OT-A, S5:DiP-A, S5:OT-A
298	Yes	7	7-A	1000	Bozic	Inf	unif	singleC	S1:DiP-A, S1:OT, S1:OT-A
299	Yes	7	7-A	1000	Bozic	Inf	unif	wholeT_0.01	J1:CBN, J1:CBN-A, J1:DiP-A, J1:OT, J1:OT-A, J5:CBN, J5:CBN-A, J5:DiP-A, J5:OT-A, S1:CBN, S1:CBN-A, S1:DiP-A, S1:OT, S1:OT-A, S5:CBN, S5:CBN-A, S5:DiP-A, S5:OT, S5:OT-A
300	Yes	7	7-A	1000	Bozic	Inf	unif	wholeT_0.5	S1:CBN, S1:CBN-A, S1:OT, S1:OT-A
301	Yes	7	7-A	1000	exp	0	last	singleC	S1:OT-A
302	Yes	7	7-A	1000	exp	0	last	wholeT_0.01	J1:OT-A, S1:OT-A, S5:OT-A
303	Yes	7	7-A	1000	exp	0	last	wholeT_0.5	S1:OT-A
304	Yes	7	7-A	1000	exp	0	unif	singleC	J1:CBN, J1:CBN-A, J1:DiP, J1:DiP-A, J1:OT, J1:OT-A, J5:CBN, J5:CBN-A, J5:DiP, J5:DiP-A, J5:OT, S1:CBN, S1:CBN-A, S1:DiP, S1:DiP-A, S1:OT, S1:OT-A, S5:CBN, S5:CBN-A, S5:DiP, S5:DiP-A, S5:OT, S5:OT-A
305	Yes	7	7-A	1000	exp	0	unif	wholeT_0.01	S1:OT-A
306	Yes	7	7-A	1000	exp	0	unif	wholeT_0.5	none
307	Yes	7	7-A	1000	exp	Inf	last	singleC	J1:OT-A, S1:OT-A, S5:OT-A
308	Yes	7	7-A	1000	exp	Inf	last	wholeT_0.01	J1:DiP-A, J1:OT-A, J5:OT-A, S1:OT-A, S5:DiP-A, S5:OT-A
309	Yes	7	7-A	1000	exp	Inf	last	wholeT_0.5	S1:OT-A, S5:OT-A
310	Yes	7	7-A	1000	exp	Inf	unif	singleC	S1:CBN, S1:CBN-A, S1:OT, S1:OT-A

Table 15: *(continued)*

	Conjunction	Drivers	Tree	S.Size	Model	sh	S.Time	S.Type	Best method(s)
311	Yes	7	7-A	1000	exp	Inf	unif	wholeT_0.01	J1:CBN, J1:CBN-A, J1:OT, J1:OT-A, J5:CBN, J5:CBN-A, J5:OT, J5:OT-A, S1:CBN, S1:CBN-A, S1:OT, S1:OT-A, S5:CBN, S5:CBN-A, S5:OT, S5:OT-A
312	Yes	7	7-A	1000	exp	Inf	unif	wholeT_0.5	S1:CBN-A, S1:OT, S1:OT-A
313	Yes	7	7-A	1000	McF_4	0	last	singleC	J1:DiP-A, J1:OT-A, J5:DiP-A, J5:OT-A, S1:DiP-A, S1:OT-A, S5:DiP-A, S5:OT-A
314	Yes	7	7-A	1000	McF_4	0	last	wholeT_0.01	S1:OT-A
315	Yes	7	7-A	1000	McF_4	0	last	wholeT_0.5	J1:DiP-A, J1:OT-A, J5:DiP-A, J5:OT-A, S1:DiP-A, S1:OT-A, S5:DiP-A, S5:OT-A
316	Yes	7	7-A	1000	McF_4	0	unif	singleC	J1:DiP-A, J1:OT, J1:OT-A, S1:DiP-A, S1:OT, S1:OT-A, S5:DiP-A, S5:OT, S5:OT-A
317	Yes	7	7-A	1000	McF_4	0	unif	wholeT_0.01	J1:OT, S1:CBN, S1:CBN-A, S1:DiP, S1:DiP-A, S1:OT, S1:OT-A, S5:OT, S5:OT-A
318	Yes	7	7-A	1000	McF_4	0	unif	wholeT_0.5	J1:DiP-A, J1:OT, J1:OT-A, S1:DiP-A, S1:OT, S1:OT-A, S5:DiP-A, S5:OT, S5:OT-A
319	Yes	7	7-A	1000	McF_4	Inf	last	singleC	J1:DiP-A, J1:OT-A, J5:DiP-A, J5:OT-A, S1:DiP-A, S1:OT-A, S5:DiP-A, S5:OT-A
320	Yes	7	7-A	1000	McF_4	Inf	last	wholeT_0.01	S1:OT-A
321	Yes	7	7-A	1000	McF_4	Inf	last	wholeT_0.5	J1:DiP-A, J1:OT-A, J5:DiP-A, J5:OT-A, S1:DiP-A, S1:OT-A, S5:DiP-A, S5:OT-A
322	Yes	7	7-A	1000	McF_4	Inf	unif	singleC	J1:DiP-A, J1:OT, J1:OT-A, S1:OT, S1:OT-A, S5:OT, S5:OT-A
323	Yes	7	7-A	1000	McF_4	Inf	unif	wholeT_0.01	J1:DiP-A, J1:OT, J1:OT-A, J5:OT-A, S1:DiP-A, S1:OT, S1:OT-A, S5:OT, S5:OT-A
324	Yes	7	7-A	1000	McF_4	Inf	unif	wholeT_0.5	J1:DiP-A, J1:OT, J1:OT-A, S1:DiP-A, S1:OT, S1:OT-A, S5:OT, S5:OT-A
325	Yes	7	7-A	1000	McF_6	0	last	singleC	S1:DiP-A, S5:DiP-A
326	Yes	7	7-A	1000	McF_6	0	last	wholeT_0.01	J1:DiP-A, J1:OT-A, S1:OT-A, S5:DiP-A, S5:OT-A
327	Yes	7	7-A	1000	McF_6	0	last	wholeT_0.5	S1:DiP-A, S5:DiP-A
328	Yes	7	7-A	1000	McF_6	0	unif	singleC	S1:CBN-A
329	Yes	7	7-A	1000	McF_6	0	unif	wholeT_0.01	S1:CBN-A, S1:DiP, S1:DiP-A
330	Yes	7	7-A	1000	McF_6	0	unif	wholeT_0.5	S1:CBN-A
331	Yes	7	7-A	1000	McF_6	Inf	last	singleC	S1:CBN-A, S1:DiP-A, S1:OT-A, S5:CBN-A, S5:DiP-A, S5:OT-A

Table 15: *(continued)*

	Conjunction	Drivers	Tree	S.Size	Model	sh	S.Time	S.Type	Best method(s)
332	Yes	7	7-A	1000	McF_6	Inf	last	wholeT_0.01	S1:DiP-A, S5:DiP-A
333	Yes	7	7-A	1000	McF_6	Inf	last	wholeT_0.5	S1:CBN-A, S1:DiP-A, S5:CBN-A, S5:DiP-A
334	Yes	7	7-A	1000	McF_6	Inf	unif	singleC	S1:CBN-A
335	Yes	7	7-A	1000	McF_6	Inf	unif	wholeT_0.01	S1:CBN-A
336	Yes	7	7-A	1000	McF_6	Inf	unif	wholeT_0.5	S1:CBN-A
337	Yes	7	7-A	200	Bozic	0	last	singleC	S1:OT-A, S5:OT-A
338	Yes	7	7-A	200	Bozic	0	last	wholeT_0.01	J1:OT-A, J5:OT-A, S1:OT-A, S5:OT-A
339	Yes	7	7-A	200	Bozic	0	last	wholeT_0.5	S1:OT-A
340	Yes	7	7-A	200	Bozic	0	unif	singleC	S1:CBN, S1:CBN-A, S1:OT, S1:OT-A
341	Yes	7	7-A	200	Bozic	0	unif	wholeT_0.01	J1:OT-A, S1:CBN, S1:CBN-A, S1:OT, S1:OT-A, S5:OT, S5:OT-A
342	Yes	7	7-A	200	Bozic	0	unif	wholeT_0.5	S1:CBN, S1:CBN-A, S1:OT, S1:OT-A
343	Yes	7	7-A	200	Bozic	Inf	last	singleC	J1:OT-A, J5:OT-A, S1:OT-A, S5:OT-A
344	Yes	7	7-A	200	Bozic	Inf	last	wholeT_0.01	J1:OT-A, J5:OT-A, S1:OT-A, S5:OT-A
345	Yes	7	7-A	200	Bozic	Inf	last	wholeT_0.5	J1:OT-A, J5:OT-A, S1:OT-A, S5:OT-A
346	Yes	7	7-A	200	Bozic	Inf	unif	singleC	S1:CBN, S1:OT, S1:OT-A
347	Yes	7	7-A	200	Bozic	Inf	unif	wholeT_0.01	J1:CBN, J1:CBN-A, J1:OT, J1:OT-A, J5:CBN, J5:CBN-A, J5:OT, J5:OT-A, S1:CBN, S1:CBN-A, S1:OT, S1:OT-A, S5:CBN, S5:CBN-A, S5:OT, S5:OT-A
348	Yes	7	7-A	200	Bozic	Inf	unif	wholeT_0.5	S1:CBN, S1:CBN-A, S1:OT, S1:OT-A
349	Yes	7	7-A	200	exp	0	last	singleC	S1:OT-A
350	Yes	7	7-A	200	exp	0	last	wholeT_0.01	J1:OT-A, S1:OT-A, S5:OT-A
351	Yes	7	7-A	200	exp	0	last	wholeT_0.5	S1:OT-A
352	Yes	7	7-A	200	exp	0	unif	singleC	J1:CBN, J1:CBN-A, J1:DiP-A, J1:OT, J1:OT-A, J5:DiP, J5:OT, J5:OT-A, S1:CBN, S1:CBN-A, S1:DiP, S1:DiP-A, S1:OT, S1:OT-A
353	Yes	7	7-A	200	exp	0	unif	wholeT_0.01	S1:CBN, S1:CBN-A, S1:OT, S1:OT-A
354	Yes	7	7-A	200	exp	0	unif	wholeT_0.5	J1:CBN, J1:CBN-A, J1:DiP, J1:DiP-A, J1:OT, J1:OT-A, J5:CBN, J5:CBN-A, J5:DiP-A, J5:OT, J5:OT-A, S1:CBN, S1:CBN-A, S1:DiP, S1:DiP-A, S1:OT, S1:OT-A, S5:CBN, S5:CBN-A, S5:DiP, S5:DiP-A, S5:OT, S5:OT-A
355	Yes	7	7-A	200	exp	Inf	last	singleC	J1:OT-A, S1:OT-A, S5:OT-A
356	Yes	7	7-A	200	exp	Inf	last	wholeT_0.01	J1:OT-A, J5:OT-A, S1:OT-A, S5:OT-A
357	Yes	7	7-A	200	exp	Inf	last	wholeT_0.5	S1:OT-A, S5:OT-A
358	Yes	7	7-A	200	exp	Inf	unif	singleC	S1:CBN, S1:CBN-A, S1:OT, S1:OT-A

Table 15: *(continued)*

	Conjunction	Drivers	Tree	S.Size	Model	sh	S.Time	S.Type	Best method(s)
359	Yes	7	7-A	200	exp	Inf	unif	wholeT_0.01	J1:CBN, J1:CBN-A, J1:OT, J1:OT-A, J5:CBN, J5:CBN-A, J5:OT, S1:CBN, S1:CBN-A, S1:OT, S1:OT-A, S5:CBN, S5:CBN-A, S5:OT, S5:OT-A
360	Yes	7	7-A	200	exp	Inf	unif	wholeT_0.5	S1:CBN-A, S1:OT, S1:OT-A
361	Yes	7	7-A	200	McF_4	0	last	singleC	J1:OT-A, J5:OT-A, S1:OT-A, S5:OT-A
362	Yes	7	7-A	200	McF_4	0	last	wholeT_0.01	S1:OT-A
363	Yes	7	7-A	200	McF_4	0	last	wholeT_0.5	J1:OT-A, J5:OT-A, S1:OT-A, S5:OT-A
364	Yes	7	7-A	200	McF_4	0	unif	singleC	J1:OT-A, S1:CBN-A, S1:OT, S1:OT-A, S5:OT, S5:OT-A
365	Yes	7	7-A	200	McF_4	0	unif	wholeT_0.01	J1:OT, J1:OT-A, S1:OT, S1:OT-A
366	Yes	7	7-A	200	McF_4	0	unif	wholeT_0.5	J1:OT, J1:OT-A, S1:OT, S1:OT-A, S5:OT, S5:OT-A
367	Yes	7	7-A	200	McF_4	Inf	last	singleC	J1:OT-A, J5:OT-A, S1:OT-A, S5:OT-A
368	Yes	7	7-A	200	McF_4	Inf	last	wholeT_0.01	S1:OT-A
369	Yes	7	7-A	200	McF_4	Inf	last	wholeT_0.5	J1:OT-A, J5:OT-A, S1:OT-A, S5:OT-A
370	Yes	7	7-A	200	McF_4	Inf	unif	singleC	J1:OT, J1:OT-A, S1:OT, S1:OT-A, S5:OT, S5:OT-A
371	Yes	7	7-A	200	McF_4	Inf	unif	wholeT_0.01	J1:OT, J1:OT-A, S1:OT, S1:OT-A, S5:OT, S5:OT-A
372	Yes	7	7-A	200	McF_4	Inf	unif	wholeT_0.5	J1:OT, J1:OT-A, S1:OT, S1:OT-A, S5:OT, S5:OT-A
373	Yes	7	7-A	200	McF_6	0	last	singleC	J1:CBN-A, S1:CBN, S1:OT-A, S5:CBN, S5:CBN-A, S5:OT-A
374	Yes	7	7-A	200	McF_6	0	last	wholeT_0.01	J1:OT-A, J5:CBN-A, S1:OT-A, S5:OT-A
375	Yes	7	7-A	200	McF_6	0	last	wholeT_0.5	S1:CBN, S1:CBN-A, S1:OT-A, S5:CBN-A, S5:OT-A
376	Yes	7	7-A	200	McF_6	0	unif	singleC	S1:CBN-A
377	Yes	7	7-A	200	McF_6	0	unif	wholeT_0.01	S1:CBN, S1:CBN-A, S1:OT, S1:OT-A
378	Yes	7	7-A	200	McF_6	0	unif	wholeT_0.5	S1:CBN, S1:CBN-A, S1:OT, S1:OT-A
379	Yes	7	7-A	200	McF_6	Inf	last	singleC	J1:CBN-A, S1:CBN, S1:CBN-A, S1:OT-A, S5:CBN-A, S5:OT-A
380	Yes	7	7-A	200	McF_6	Inf	last	wholeT_0.01	J5:CBN-A, S1:OT-A, S5:OT-A
381	Yes	7	7-A	200	McF_6	Inf	last	wholeT_0.5	S1:CBN, S1:CBN-A, S1:OT-A, S5:CBN-A, S5:OT-A
382	Yes	7	7-A	200	McF_6	Inf	unif	singleC	S1:CBN-A
383	Yes	7	7-A	200	McF_6	Inf	unif	wholeT_0.01	S1:CBN-A
384	Yes	7	7-A	200	McF_6	Inf	unif	wholeT_0.5	S1:CBN, S1:CBN-A, S1:OT, S1:OT-A
385	Yes	7	7-A	100	Bozic	0	last	singleC	S1:OT-A
386	Yes	7	7-A	100	Bozic	0	last	wholeT_0.01	J1:OT-A, J5:OT-A, S1:OT-A, S5:OT-A

Table 15: *(continued)*

	Conjunction	Drivers	Tree	S.Size	Model	sh	S.Time	S.Type	Best method(s)
387	Yes	7	7-A	100	Bozic	0	last	wholeT_0.5	S1:OT-A
388	Yes	7	7-A	100	Bozic	0	unif	singleC	S1:CBN, S1:CBN-A, S1:OT, S1:OT-A
389	Yes	7	7-A	100	Bozic	0	unif	wholeT_0.01	S1:CBN, S1:CBN-A
390	Yes	7	7-A	100	Bozic	0	unif	wholeT_0.5	S1:CBN, S1:CBN-A, S1:OT, S1:OT-A
391	Yes	7	7-A	100	Bozic	Inf	last	singleC	J1:OT-A, S1:OT-A, S5:OT-A
392	Yes	7	7-A	100	Bozic	Inf	last	wholeT_0.01	J1:OT-A, J5:OT-A, S1:OT-A, S5:OT-A
393	Yes	7	7-A	100	Bozic	Inf	last	wholeT_0.5	J1:OT-A, J5:OT-A, S1:OT-A, S5:OT-A
394	Yes	7	7-A	100	Bozic	Inf	unif	singleC	S1:CBN, S1:CBN-A, S1:OT, S1:OT-A
395	Yes	7	7-A	100	Bozic	Inf	unif	wholeT_0.01	J1:CBN, J1:CBN-A, J1:OT, J1:OT-A, J5:CBN, J5:CBN-A, J5:OT, J5:OT-A, S1:CBN, S1:CBN-A, S1:OT, S1:OT-A, S5:CBN, S5:CBN-A, S5:OT, S5:OT-A
396	Yes	7	7-A	100	Bozic	Inf	unif	wholeT_0.5	S1:CBN, S1:CBN-A, S1:OT, S1:OT-A
397	Yes	7	7-A	100	exp	0	last	singleC	S1:OT-A
398	Yes	7	7-A	100	exp	0	last	wholeT_0.01	S1:OT-A, S5:OT-A
399	Yes	7	7-A	100	exp	0	last	wholeT_0.5	S1:OT-A
400	Yes	7	7-A	100	exp	0	unif	singleC	S1:CBN, S1:CBN-A, S1:OT, S1:OT-A
401	Yes	7	7-A	100	exp	0	unif	wholeT_0.01	S1:CBN, S1:CBN-A, S1:OT, S1:OT-A
402	Yes	7	7-A	100	exp	0	unif	wholeT_0.5	J1:OT, J1:OT-A, S1:CBN, S1:CBN-A, S1:OT, S1:OT-A
403	Yes	7	7-A	100	exp	Inf	last	singleC	S1:OT-A, S5:OT-A
404	Yes	7	7-A	100	exp	Inf	last	wholeT_0.01	J1:OT-A, J5:OT-A, S1:OT-A, S5:OT-A
405	Yes	7	7-A	100	exp	Inf	last	wholeT_0.5	S1:OT-A, S5:OT-A
406	Yes	7	7-A	100	exp	Inf	unif	singleC	S1:CBN, S1:OT, S1:OT-A
407	Yes	7	7-A	100	exp	Inf	unif	wholeT_0.01	J1:OT, J1:OT-A, J5:CBN, J5:CBN-A, J5:OT, J5:OT-A, S1:CBN, S1:CBN-A, S1:OT, S1:OT-A, S5:CBN, S5:CBN-A, S5:OT, S5:OT-A
408	Yes	7	7-A	100	exp	Inf	unif	wholeT_0.5	S1:CBN, S1:CBN-A, S1:OT, S1:OT-A
409	Yes	7	7-A	100	McF_4	0	last	singleC	S1:OT-A
410	Yes	7	7-A	100	McF_4	0	last	wholeT_0.01	S1:OT-A
411	Yes	7	7-A	100	McF_4	0	last	wholeT_0.5	J1:OT-A, S1:OT-A
412	Yes	7	7-A	100	McF_4	0	unif	singleC	S1:CBN-A, S1:OT, S1:OT-A
413	Yes	7	7-A	100	McF_4	0	unif	wholeT_0.01	S1:CBN-A, S1:OT, S1:OT-A
414	Yes	7	7-A	100	McF_4	0	unif	wholeT_0.5	J1:OT, J1:OT-A, S1:OT, S1:OT-A, S5:OT, S5:OT-A
415	Yes	7	7-A	100	McF_4	Inf	last	singleC	J1:OT-A, J5:OT-A, S1:OT-A, S5:OT-A
416	Yes	7	7-A	100	McF_4	Inf	last	wholeT_0.01	S1:OT-A
417	Yes	7	7-A	100	McF_4	Inf	last	wholeT_0.5	J1:OT-A, J5:OT-A, S1:OT-A, S5:OT-A

Table 15: *(continued)*

	Conjunction	Drivers	Tree	S.Size	Model	sh	S.Time	S.Type	Best method(s)
418	Yes	7	7-A	100	McF_4	Inf	unif	singleC	J1:OT, J1:OT-A, S1:OT, S1:OT-A, S5:OT, S5:OT-A
419	Yes	7	7-A	100	McF_4	Inf	unif	wholeT_0.01	J1:OT, J1:OT-A, S1:OT, S1:OT-A, S5:OT, S5:OT-A
420	Yes	7	7-A	100	McF_4	Inf	unif	wholeT_0.5	J1:OT, J1:OT-A, S1:OT, S1:OT-A, S5:OT, S5:OT-A
421	Yes	7	7-A	100	McF_6	0	last	singleC	S1:CBN, S1:CBN-A, S1:OT-A, S5:CBN-A, S5:OT-A
422	Yes	7	7-A	100	McF_6	0	last	wholeT_0.01	J1:OT-A, S1:OT-A, S5:OT-A
423	Yes	7	7-A	100	McF_6	0	last	wholeT_0.5	S1:CBN, S1:CBN-A, S1:OT-A, S5:CBN, S5:CBN-A, S5:OT-A
424	Yes	7	7-A	100	McF_6	0	unif	singleC	S1:CBN, S1:CBN-A, S1:OT, S1:OT-A
425	Yes	7	7-A	100	McF_6	0	unif	wholeT_0.01	S1:CBN, S1:CBN-A, S1:OT, S1:OT-A, S5:CBN-A, S5:OT-A
426	Yes	7	7-A	100	McF_6	0	unif	wholeT_0.5	S1:CBN-A, S1:OT, S1:OT-A
427	Yes	7	7-A	100	McF_6	Inf	last	singleC	S1:CBN, S1:CBN-A, S1:OT-A, S5:CBN-A, S5:OT-A
428	Yes	7	7-A	100	McF_6	Inf	last	wholeT_0.01	J1:CBN-A, J5:CBN-A, S1:OT-A, S5:OT-A
429	Yes	7	7-A	100	McF_6	Inf	last	wholeT_0.5	S1:CBN, S1:CBN-A, S1:OT-A, S5:CBN, S5:CBN-A, S5:OT-A
430	Yes	7	7-A	100	McF_6	Inf	unif	singleC	S1:CBN-A, S1:OT, S1:OT-A
431	Yes	7	7-A	100	McF_6	Inf	unif	wholeT_0.01	S1:CBN-A, S1:OT-A
432	Yes	7	7-A	100	McF_6	Inf	unif	wholeT_0.5	S1:CBN-A, S1:OT-A
433	No	11	11-B	1000	Bozic	0	last	singleC	S1:OT, S1:OT-A
434	No	11	11-B	1000	Bozic	0	last	wholeT_0.01	S1:DiP, S1:DiP-A, S1:OT, S1:OT-A, S5:DiP-A, S5:OT, S5:OT-A
435	No	11	11-B	1000	Bozic	0	last	wholeT_0.5	S1:OT, S1:OT-A
436	No	11	11-B	1000	Bozic	0	unif	singleC	J1:CBN, J1:CBN-A, J1:DiP-A, J1:OT, J5:CBN, J5:CBN-A, J5:DiP, J5:DiP-A, S1:CBN, S1:CBN-A, S1:DiP, S1:OT, S1:OT-A, S5:CBN-A, S5:DiP, S5:DiP-A
437	No	11	11-B	1000	Bozic	0	unif	wholeT_0.01	S1:CBN, S1:CBN-A
438	No	11	11-B	1000	Bozic	0	unif	wholeT_0.5	none
439	No	11	11-B	1000	Bozic	Inf	last	singleC	S1:OT, S1:OT-A
440	No	11	11-B	1000	Bozic	Inf	last	wholeT_0.01	J1:CBN-A, J1:DiP, J1:DiP-A, S1:CBN-A, S1:DiP, S1:DiP-A, S1:OT, S1:OT-A, S5:DiP, S5:DiP-A, S5:OT, S5:OT-A
441	No	11	11-B	1000	Bozic	Inf	last	wholeT_0.5	S1:OT, S1:OT-A

Table 15: *(continued)*

	Conjunction	Drivers	Tree	S.Size	Model	sh	S.Time	S.Type	Best method(s)
442	No	11	11-B	1000	Bozic	Inf	unif	singleC	S1:CBN, S1:CBN-A, S1:DiP-A, S1:OT, S1:OT-A
443	No	11	11-B	1000	Bozic	Inf	unif	wholeT_0.01	S1:CBN, S1:DiP, S1:DiP-A, S1:OT, S1:OT-A, S5:CBN, S5:CBN-A, S5:DiP, S5:DiP-A, S5:OT, S5:OT-A
444	No	11	11-B	1000	Bozic	Inf	unif	wholeT_0.5	S1:CBN, S1:CBN-A, S1:OT, S1:OT-A
445	No	11	11-B	1000	exp	0	last	singleC	S1:OT, S1:OT-A
446	No	11	11-B	1000	exp	0	last	wholeT_0.01	S1:CBN, S1:DiP-A, S1:OT, S1:OT-A, S5:DiP-A, S5:OT, S5:OT-A
447	No	11	11-B	1000	exp	0	last	wholeT_0.5	S1:OT, S1:OT-A
448	No	11	11-B	1000	exp	0	unif	singleC	J1:CBN, J1:CBN-A, J1:DiP, J1:DiP-A, J1:OT, J1:OT-A, J5:CBN, J5:CBN-A, J5:DiP, J5:DiP-A, J5:OT, S1:CBN-A, S1:DiP, S1:DiP-A, S1:OT, S1:OT-A, S5:CBN, S5:CBN-A, S5:DiP, S5:DiP-A, S5:OT, S5:OT-A
449	No	11	11-B	1000	exp	0	unif	wholeT_0.01	S1:CBN, S1:CBN-A, S1:OT, S1:OT-A
450	No	11	11-B	1000	exp	0	unif	wholeT_0.5	none
451	No	11	11-B	1000	exp	Inf	last	singleC	S1:DiP, S1:DiP-A, S1:OT, S1:OT-A, S5:DiP, S5:DiP-A, S5:OT, S5:OT-A
452	No	11	11-B	1000	exp	Inf	last	wholeT_0.01	J1:DiP, J1:DiP-A, J1:OT, J1:OT-A, S1:DiP, S1:DiP-A, S1:OT, S1:OT-A, S5:DiP, S5:DiP-A, S5:OT
453	No	11	11-B	1000	exp	Inf	last	wholeT_0.5	S1:OT, S1:OT-A
454	No	11	11-B	1000	exp	Inf	unif	singleC	S1:OT, S1:OT-A
455	No	11	11-B	1000	exp	Inf	unif	wholeT_0.01	S1:CBN, S1:CBN-A, S1:DiP-A, S1:OT, S1:OT-A
456	No	11	11-B	1000	exp	Inf	unif	wholeT_0.5	S1:OT, S1:OT-A
457	No	11	11-B	1000	McF_4	0	last	singleC	S1:DiP, S1:DiP-A, S1:OT, S1:OT-A
458	No	11	11-B	1000	McF_4	0	last	wholeT_0.01	S1:DiP, S1:DiP-A, S1:OT, S1:OT-A
459	No	11	11-B	1000	McF_4	0	last	wholeT_0.5	S1:DiP-A, S1:OT, S1:OT-A
460	No	11	11-B	1000	McF_4	0	unif	singleC	S1:DiP-A, S1:OT, S1:OT-A
461	No	11	11-B	1000	McF_4	0	unif	wholeT_0.01	S1:CBN, S1:OT, S1:OT-A
462	No	11	11-B	1000	McF_4	0	unif	wholeT_0.5	S1:DiP, S1:DiP-A, S1:OT, S1:OT-A
463	No	11	11-B	1000	McF_4	Inf	last	singleC	S1:OT, S1:OT-A
464	No	11	11-B	1000	McF_4	Inf	last	wholeT_0.01	S1:DiP, S1:DiP-A, S1:OT, S1:OT-A, S5:DiP, S5:OT
465	No	11	11-B	1000	McF_4	Inf	last	wholeT_0.5	S1:DiP-A, S1:OT, S1:OT-A
466	No	11	11-B	1000	McF_4	Inf	unif	singleC	S1:OT, S1:OT-A
467	No	11	11-B	1000	McF_4	Inf	unif	wholeT_0.01	S1:OT, S1:OT-A

Table 15: *(continued)*

	Conjunction	Drivers	Tree	S.Size	Model	sh	S.Time	S.Type	Best method(s)
468	No	11	11-B	1000	McF_4	Inf	unif	wholeT_0.5	S1:CBN, S1:OT, S1:OT-A
469	No	11	11-B	1000	McF_6	0	last	singleC	S1:DiP, S1:DiP-A, S1:OT, S1:OT-A, S5:DiP, S5:DiP-A, S5:OT, S5:OT-A
470	No	11	11-B	1000	McF_6	0	last	wholeT_0.01	S1:DiP, S1:DiP-A, S1:OT, S1:OT-A, S5:DiP, S5:DiP-A, S5:OT, S5:OT-A
471	No	11	11-B	1000	McF_6	0	last	wholeT_0.5	S1:DiP, S1:DiP-A, S1:OT, S1:OT-A, S5:DiP, S5:DiP-A, S5:OT, S5:OT-A
472	No	11	11-B	1000	McF_6	0	unif	singleC	S1:DiP, S1:DiP-A, S1:OT, S1:OT-A
473	No	11	11-B	1000	McF_6	0	unif	wholeT_0.01	S1:DiP, S1:DiP-A, S1:OT, S1:OT-A
474	No	11	11-B	1000	McF_6	0	unif	wholeT_0.5	S1:DiP, S1:DiP-A, S1:OT, S1:OT-A
475	No	11	11-B	1000	McF_6	Inf	last	singleC	S1:DiP, S1:DiP-A, S1:OT, S1:OT-A, S5:DiP, S5:DiP-A, S5:OT, S5:OT-A
476	No	11	11-B	1000	McF_6	Inf	last	wholeT_0.01	S1:DiP-A, S1:OT, S1:OT-A, S5:DiP-A, S5:OT, S5:OT-A
477	No	11	11-B	1000	McF_6	Inf	last	wholeT_0.5	S1:DiP, S1:DiP-A, S1:OT, S1:OT-A, S5:DiP, S5:DiP-A, S5:OT, S5:OT-A
478	No	11	11-B	1000	McF_6	Inf	unif	singleC	S1:DiP, S1:DiP-A, S1:OT, S1:OT-A
479	No	11	11-B	1000	McF_6	Inf	unif	wholeT_0.01	S1:DiP, S1:DiP-A, S1:OT, S1:OT-A
480	No	11	11-B	1000	McF_6	Inf	unif	wholeT_0.5	S1:DiP, S1:DiP-A, S1:OT, S1:OT-A
481	No	11	11-B	200	Bozic	0	last	singleC	S1:CBN-A, S1:OT, S1:OT-A
482	No	11	11-B	200	Bozic	0	last	wholeT_0.01	S1:CBN-A, S1:OT, S1:OT-A, S5:OT-A
483	No	11	11-B	200	Bozic	0	last	wholeT_0.5	S1:CBN, S1:CBN-A, S1:OT, S1:OT-A
484	No	11	11-B	200	Bozic	0	unif	singleC	S1:CBN, S1:OT, S1:OT-A
485	No	11	11-B	200	Bozic	0	unif	wholeT_0.01	S1:CBN, S1:CBN-A
486	No	11	11-B	200	Bozic	0	unif	wholeT_0.5	S1:CBN, S1:CBN-A, S1:OT, S1:OT-A
487	No	11	11-B	200	Bozic	Inf	last	singleC	S1:CBN, S1:OT, S1:OT-A
488	No	11	11-B	200	Bozic	Inf	last	wholeT_0.01	J1:OT, J1:OT-A, S1:OT, S1:OT-A, S5:OT, S5:OT-A
489	No	11	11-B	200	Bozic	Inf	last	wholeT_0.5	S1:OT, S1:OT-A
490	No	11	11-B	200	Bozic	Inf	unif	singleC	S1:CBN, S1:CBN-A, S1:OT, S1:OT-A
491	No	11	11-B	200	Bozic	Inf	unif	wholeT_0.01	S1:CBN, S1:CBN-A, S1:OT, S1:OT-A, S5:CBN, S5:CBN-A, S5:OT, S5:OT-A
492	No	11	11-B	200	Bozic	Inf	unif	wholeT_0.5	S1:CBN, S1:CBN-A, S1:OT, S1:OT-A
493	No	11	11-B	200	exp	0	last	singleC	S1:OT, S1:OT-A
494	No	11	11-B	200	exp	0	last	wholeT_0.01	S1:CBN, S1:CBN-A, S1:OT, S1:OT-A
495	No	11	11-B	200	exp	0	last	wholeT_0.5	S1:OT, S1:OT-A

Table 15: *(continued)*

	Conjunction	Drivers	Tree	S.Size	Model	sh	S.Time	S.Type	Best method(s)
496	No	11	11-B	200	exp	0	unif	singleC	J1:CBN, J1:CBN-A, J1:DiP, J1:DiP-A, J1:OT, J1:OT-A, J5:CBN, J5:CBN-A, J5:DiP, J5:DiP-A, J5:OT, J5:OT-A, S1:CBN, S1:CBN-A, S1:DiP, S1:DiP-A, S1:OT, S1:OT-A, S5:CBN, S5:CBN-A, S5:DiP, S5:DiP-A, S5:OT
497	No	11	11-B	200	exp	0	unif	wholeT_0.01	S1:CBN, S1:CBN-A, S1:OT, S1:OT-A
498	No	11	11-B	200	exp	0	unif	wholeT_0.5	none
499	No	11	11-B	200	exp	Inf	last	singleC	S1:CBN, S1:OT, S1:OT-A
500	No	11	11-B	200	exp	Inf	last	wholeT_0.01	J1:OT, S1:OT, S1:OT-A, S5:OT, S5:OT-A
501	No	11	11-B	200	exp	Inf	last	wholeT_0.5	S1:OT, S1:OT-A
502	No	11	11-B	200	exp	Inf	unif	singleC	S1:CBN, S1:CBN-A, S1:OT, S1:OT-A
503	No	11	11-B	200	exp	Inf	unif	wholeT_0.01	S1:CBN, S1:CBN-A, S1:OT, S1:OT-A
504	No	11	11-B	200	exp	Inf	unif	wholeT_0.5	S1:CBN, S1:CBN-A, S1:OT, S1:OT-A
505	No	11	11-B	200	McF_4	0	last	singleC	S1:OT, S1:OT-A
506	No	11	11-B	200	McF_4	0	last	wholeT_0.01	S1:OT, S1:OT-A
507	No	11	11-B	200	McF_4	0	last	wholeT_0.5	S1:OT, S1:OT-A
508	No	11	11-B	200	McF_4	0	unif	singleC	S1:CBN-A, S1:OT, S1:OT-A
509	No	11	11-B	200	McF_4	0	unif	wholeT_0.01	S1:CBN, S1:CBN-A, S1:OT, S1:OT-A
510	No	11	11-B	200	McF_4	0	unif	wholeT_0.5	S1:OT, S1:OT-A
511	No	11	11-B	200	McF_4	Inf	last	singleC	S1:OT, S1:OT-A
512	No	11	11-B	200	McF_4	Inf	last	wholeT_0.01	S1:OT, S1:OT-A, S5:OT, S5:OT-A
513	No	11	11-B	200	McF_4	Inf	last	wholeT_0.5	S1:OT, S1:OT-A
514	No	11	11-B	200	McF_4	Inf	unif	singleC	S1:OT, S1:OT-A
515	No	11	11-B	200	McF_4	Inf	unif	wholeT_0.01	S1:OT, S1:OT-A
516	No	11	11-B	200	McF_4	Inf	unif	wholeT_0.5	S1:OT, S1:OT-A
517	No	11	11-B	200	McF_6	0	last	singleC	S1:OT, S1:OT-A, S5:OT, S5:OT-A
518	No	11	11-B	200	McF_6	0	last	wholeT_0.01	S1:OT, S1:OT-A, S5:OT, S5:OT-A
519	No	11	11-B	200	McF_6	0	last	wholeT_0.5	S1:OT, S1:OT-A, S5:OT, S5:OT-A
520	No	11	11-B	200	McF_6	0	unif	singleC	S1:OT, S1:OT-A
521	No	11	11-B	200	McF_6	0	unif	wholeT_0.01	S1:OT, S1:OT-A
522	No	11	11-B	200	McF_6	0	unif	wholeT_0.5	S1:OT, S1:OT-A
523	No	11	11-B	200	McF_6	Inf	last	singleC	S1:OT, S1:OT-A, S5:OT, S5:OT-A
524	No	11	11-B	200	McF_6	Inf	last	wholeT_0.01	S1:OT, S1:OT-A, S5:OT, S5:OT-A
525	No	11	11-B	200	McF_6	Inf	last	wholeT_0.5	S1:OT, S1:OT-A, S5:OT
526	No	11	11-B	200	McF_6	Inf	unif	singleC	S1:OT, S1:OT-A
527	No	11	11-B	200	McF_6	Inf	unif	wholeT_0.01	S1:OT, S1:OT-A
528	No	11	11-B	200	McF_6	Inf	unif	wholeT_0.5	S1:OT, S1:OT-A
529	No	11	11-B	100	Bozic	0	last	singleC	S1:CBN, S1:CBN-A, S1:OT, S1:OT-A

Table 15: *(continued)*

	Conjunction	Drivers	Tree	S.Size	Model	sh	S.Time	S.Type	Best method(s)
530	No	11	11-B	100	Bozic	0	last	wholeT_0.01	S1:CBN, S1:CBN-A, S1:OT, S1:OT-A
531	No	11	11-B	100	Bozic	0	last	wholeT_0.5	S1:OT, S1:OT-A
532	No	11	11-B	100	Bozic	0	unif	singleC	S1:CBN, S1:CBN-A, S1:OT, S1:OT-A
533	No	11	11-B	100	Bozic	0	unif	wholeT_0.01	S1:CBN, S1:CBN-A
534	No	11	11-B	100	Bozic	0	unif	wholeT_0.5	S1:CBN, S1:CBN-A, S1:OT, S1:OT-A
535	No	11	11-B	100	Bozic	Inf	last	singleC	S1:CBN, S1:OT, S1:OT-A
536	No	11	11-B	100	Bozic	Inf	last	wholeT_0.01	J1:OT, J1:OT-A, S1:CBN-A, S1:OT, S1:OT-A, S5:CBN-A, S5:OT, S5:OT-A
537	No	11	11-B	100	Bozic	Inf	last	wholeT_0.5	S1:OT, S1:OT-A
538	No	11	11-B	100	Bozic	Inf	unif	singleC	S1:CBN, S1:CBN-A, S1:OT, S1:OT-A
539	No	11	11-B	100	Bozic	Inf	unif	wholeT_0.01	J1:CBN, J1:CBN-A, J1:OT, J1:OT-A, S1:CBN, S1:CBN-A, S1:OT, S1:OT-A, S5:CBN, S5:OT
540	No	11	11-B	100	Bozic	Inf	unif	wholeT_0.5	S1:CBN, S1:CBN-A, S1:OT, S1:OT-A
541	No	11	11-B	100	exp	0	last	singleC	S1:OT, S1:OT-A
542	No	11	11-B	100	exp	0	last	wholeT_0.01	S1:CBN, S1:OT, S1:OT-A
543	No	11	11-B	100	exp	0	last	wholeT_0.5	S1:CBN-A, S1:OT, S1:OT-A
544	No	11	11-B	100	exp	0	unif	singleC	J1:CBN, J1:CBN-A, J1:OT, J1:OT-A, S1:CBN, S1:CBN-A, S1:OT, S1:OT-A
545	No	11	11-B	100	exp	0	unif	wholeT_0.01	S1:CBN, S1:CBN-A, S1:OT, S1:OT-A
546	No	11	11-B	100	exp	0	unif	wholeT_0.5	J1:CBN, J1:CBN-A, J1:OT, J1:OT-A, S1:CBN, S1:CBN-A, S1:OT, S1:OT-A
547	No	11	11-B	100	exp	Inf	last	singleC	S1:OT, S1:OT-A
548	No	11	11-B	100	exp	Inf	last	wholeT_0.01	J1:CBN-A, S1:OT, S1:OT-A, S5:CBN-A, S5:OT, S5:OT-A
549	No	11	11-B	100	exp	Inf	last	wholeT_0.5	S1:OT, S1:OT-A
550	No	11	11-B	100	exp	Inf	unif	singleC	S1:CBN, S1:OT, S1:OT-A
551	No	11	11-B	100	exp	Inf	unif	wholeT_0.01	S1:CBN, S1:CBN-A, S1:OT, S1:OT-A
552	No	11	11-B	100	exp	Inf	unif	wholeT_0.5	S1:OT, S1:OT-A
553	No	11	11-B	100	McF_4	0	last	singleC	S1:OT, S1:OT-A
554	No	11	11-B	100	McF_4	0	last	wholeT_0.01	S1:OT, S1:OT-A, S5:OT, S5:OT-A
555	No	11	11-B	100	McF_4	0	last	wholeT_0.5	S1:OT, S1:OT-A
556	No	11	11-B	100	McF_4	0	unif	singleC	S1:CBN, S1:CBN-A, S1:OT, S1:OT-A
557	No	11	11-B	100	McF_4	0	unif	wholeT_0.01	S1:CBN, S1:CBN-A, S1:OT, S1:OT-A
558	No	11	11-B	100	McF_4	0	unif	wholeT_0.5	S1:CBN, S1:OT, S1:OT-A
559	No	11	11-B	100	McF_4	Inf	last	singleC	S1:OT, S1:OT-A
560	No	11	11-B	100	McF_4	Inf	last	wholeT_0.01	S1:OT, S1:OT-A, S5:OT-A
561	No	11	11-B	100	McF_4	Inf	last	wholeT_0.5	S1:OT, S1:OT-A
562	No	11	11-B	100	McF_4	Inf	unif	singleC	S1:OT, S1:OT-A
563	No	11	11-B	100	McF_4	Inf	unif	wholeT_0.01	S1:OT, S1:OT-A

Table 15: *(continued)*

	Conjunction	Drivers	Tree	S.Size	Model	sh	S.Time	S.Type	Best method(s)
564	No	11	11-B	100	McF_4	Inf	unif	wholeT_0.5	S1:OT, S1:OT-A
565	No	11	11-B	100	McF_6	0	last	singleC	S1:OT, S1:OT-A, S5:OT, S5:OT-A
566	No	11	11-B	100	McF_6	0	last	wholeT_0.01	S1:OT, S1:OT-A, S5:OT, S5:OT-A
567	No	11	11-B	100	McF_6	0	last	wholeT_0.5	S1:OT, S1:OT-A, S5:OT, S5:OT-A
568	No	11	11-B	100	McF_6	0	unif	singleC	S1:OT, S1:OT-A
569	No	11	11-B	100	McF_6	0	unif	wholeT_0.01	S1:OT, S1:OT-A
570	No	11	11-B	100	McF_6	0	unif	wholeT_0.5	S1:OT, S1:OT-A
571	No	11	11-B	100	McF_6	Inf	last	singleC	S1:OT, S1:OT-A, S5:OT, S5:OT-A
572	No	11	11-B	100	McF_6	Inf	last	wholeT_0.01	S1:OT, S1:OT-A, S5:OT, S5:OT-A
573	No	11	11-B	100	McF_6	Inf	last	wholeT_0.5	S1:OT, S1:OT-A, S5:OT, S5:OT-A
574	No	11	11-B	100	McF_6	Inf	unif	singleC	S1:OT, S1:OT-A
575	No	11	11-B	100	McF_6	Inf	unif	wholeT_0.01	S1:OT, S1:OT-A
576	No	11	11-B	100	McF_6	Inf	unif	wholeT_0.5	S1:OT, S1:OT-A
577	No	9	9-B	1000	Bozic	0	last	singleC	S1:CBN-A, S1:OT, S1:OT-A
578	No	9	9-B	1000	Bozic	0	last	wholeT_0.01	S1:CBN-A, S1:DiP, S1:DiP-A, S1:OT, S1:OT-A, S5:CBN-A, S5:DiP, S5:DiP-A, S5:OT, S5:OT-A
579	No	9	9-B	1000	Bozic	0	last	wholeT_0.5	S1:OT, S1:OT-A
580	No	9	9-B	1000	Bozic	0	unif	singleC	none
581	No	9	9-B	1000	Bozic	0	unif	wholeT_0.01	S1:CBN, S1:CBN-A, S1:OT, S1:OT-A
582	No	9	9-B	1000	Bozic	0	unif	wholeT_0.5	J1:CBN, J1:CBN-A, J1:DiP-A, J1:OT, J1:OT-A, J5:CBN, J5:CBN-A, J5:DiP, J5:DiP-A, J5:OT, J5:OT-A, S1:CBN, S1:CBN-A, S1:DiP, S1:DiP-A, S1:OT, S1:OT-A, S5:CBN, S5:CBN-A, S5:DiP, S5:DiP-A, S5:OT, S5:OT-A
583	No	9	9-B	1000	Bozic	Inf	last	singleC	S1:CBN-A, S1:DiP, S1:DiP-A, S1:OT, S1:OT-A, S5:DiP, S5:DiP-A, S5:OT, S5:OT-A
584	No	9	9-B	1000	Bozic	Inf	last	wholeT_0.01	J1:DiP, J1:DiP-A, J1:OT, J1:OT-A, S1:DiP, S1:DiP-A, S1:OT, S1:OT-A, S5:DiP, S5:DiP-A, S5:OT-A
585	No	9	9-B	1000	Bozic	Inf	last	wholeT_0.5	S1:CBN, S1:CBN-A, S1:DiP, S1:DiP-A, S1:OT, S1:OT-A, S5:DiP, S5:DiP-A, S5:OT-A
586	No	9	9-B	1000	Bozic	Inf	unif	singleC	S1:CBN, S1:CBN-A, S1:OT, S1:OT-A
587	No	9	9-B	1000	Bozic	Inf	unif	wholeT_0.01	S1:CBN, S1:CBN-A, S1:DiP, S1:DiP-A, S1:OT, S1:OT-A, S5:CBN-A, S5:DiP, S5:DiP-A, S5:OT, S5:OT-A
588	No	9	9-B	1000	Bozic	Inf	unif	wholeT_0.5	S1:CBN-A, S1:OT, S1:OT-A

Table 15: *(continued)*

	Conjunction	Drivers	Tree	S.Size	Model	sh	S.Time	S.Type	Best method(s)
589	No	9	9-B	1000	exp	0	last	singleC	S1:OT, S1:OT-A
590	No	9	9-B	1000	exp	0	last	wholeT_0.01	S1:CBN, S1:CBN-A, S1:DiP-A, S1:OT, S1:OT-A
591	No	9	9-B	1000	exp	0	last	wholeT_0.5	none
592	No	9	9-B	1000	exp	0	unif	singleC	none
593	No	9	9-B	1000	exp	0	unif	wholeT_0.01	J1:CBN, J1:CBN-A, J5:CBN-A, J5:DiP-A, S1:DiP, S1:DiP-A, S1:OT, S1:OT-A, S5:DiP-A
594	No	9	9-B	1000	exp	0	unif	wholeT_0.5	J1:CBN, J1:CBN-A, J1:DiP, J1:DiP-A, J1:OT, J1:OT-A, J5:CBN, J5:CBN-A, J5:DiP, J5:DiP-A, J5:OT, J5:OT-A, S1:CBN, S1:CBN-A, S1:DiP, S1:DiP-A, S1:OT, S1:OT-A, S5:CBN-A, S5:DiP, S5:DiP-A, S5:OT, S5:OT-A
595	No	9	9-B	1000	exp	Inf	last	singleC	S1:DiP, S1:DiP-A, S1:OT, S1:OT-A, S5:DiP, S5:DiP-A, S5:OT, S5:OT-A
596	No	9	9-B	1000	exp	Inf	last	wholeT_0.01	J1:DiP, J1:DiP-A, J1:OT, J1:OT-A, S1:CBN, S1:CBN-A, S1:DiP, S1:DiP-A, S1:OT, S1:OT-A, S5:CBN, S5:CBN-A, S5:DiP, S5:DiP-A, S5:OT, S5:OT-A
597	No	9	9-B	1000	exp	Inf	last	wholeT_0.5	S1:CBN, S1:CBN-A, S1:DiP, S1:DiP-A, S1:OT, S1:OT-A, S5:DiP, S5:OT, S5:OT-A
598	No	9	9-B	1000	exp	Inf	unif	singleC	S1:CBN, S1:OT, S1:OT-A
599	No	9	9-B	1000	exp	Inf	unif	wholeT_0.01	S1:CBN, S1:CBN-A, S1:OT, S1:OT-A
600	No	9	9-B	1000	exp	Inf	unif	wholeT_0.5	S1:CBN-A, S1:OT, S1:OT-A
601	No	9	9-B	1000	McF_4	0	last	singleC	S1:DiP-A, S1:OT, S1:OT-A
602	No	9	9-B	1000	McF_4	0	last	wholeT_0.01	S1:DiP, S1:DiP-A, S1:OT, S1:OT-A, S5:DiP, S5:DiP-A, S5:OT, S5:OT-A
603	No	9	9-B	1000	McF_4	0	last	wholeT_0.5	S1:DiP-A, S1:OT, S1:OT-A
604	No	9	9-B	1000	McF_4	0	unif	singleC	S1:DiP, S1:DiP-A, S1:OT, S1:OT-A
605	No	9	9-B	1000	McF_4	0	unif	wholeT_0.01	S1:CBN, S1:DiP-A, S1:OT, S1:OT-A
606	No	9	9-B	1000	McF_4	0	unif	wholeT_0.5	S1:DiP, S1:DiP-A, S1:OT, S1:OT-A
607	No	9	9-B	1000	McF_4	Inf	last	singleC	S1:OT, S1:OT-A
608	No	9	9-B	1000	McF_4	Inf	last	wholeT_0.01	S1:DiP, S1:DiP-A, S1:OT, S1:OT-A, S5:DiP, S5:DiP-A, S5:OT, S5:OT-A
609	No	9	9-B	1000	McF_4	Inf	last	wholeT_0.5	S1:OT, S1:OT-A
610	No	9	9-B	1000	McF_4	Inf	unif	singleC	S1:DiP, S1:OT, S1:OT-A
611	No	9	9-B	1000	McF_4	Inf	unif	wholeT_0.01	S1:DiP-A, S1:OT, S1:OT-A
612	No	9	9-B	1000	McF_4	Inf	unif	wholeT_0.5	S1:CBN, S1:DiP, S1:OT, S1:OT-A

Table 15: *(continued)*

	Conjunction	Drivers	Tree	S.Size	Model	sh	S.Time	S.Type	Best method(s)
613	No	9	9-B	1000	McF_6	0	last	singleC	S1:DiP-A, S1:OT-A, S5:DiP-A, S5:OT-A
614	No	9	9-B	1000	McF_6	0	last	wholeT_0.01	J1:DiP-A, J1:OT-A, S1:DiP-A, S1:OT-A, S5:DiP-A, S5:OT-A
615	No	9	9-B	1000	McF_6	0	last	wholeT_0.5	S1:OT-A, S5:DiP-A, S5:OT-A
616	No	9	9-B	1000	McF_6	0	unif	singleC	S1:CBN, S1:DiP, S1:DiP-A, S1:OT, S1:OT-A
617	No	9	9-B	1000	McF_6	0	unif	wholeT_0.01	S1:DiP, S1:DiP-A, S1:OT, S1:OT-A, S5:DiP, S5:OT, S5:OT-A
618	No	9	9-B	1000	McF_6	0	unif	wholeT_0.5	S1:DiP, S1:DiP-A, S1:OT, S1:OT-A
619	No	9	9-B	1000	McF_6	Inf	last	singleC	S1:DiP-A, S1:OT-A, S5:DiP-A, S5:OT-A
620	No	9	9-B	1000	McF_6	Inf	last	wholeT_0.01	S1:DiP-A, S1:OT-A, S5:DiP-A, S5:OT-A
621	No	9	9-B	1000	McF_6	Inf	last	wholeT_0.5	S1:DiP-A, S1:OT-A, S5:DiP-A, S5:OT-A
622	No	9	9-B	1000	McF_6	Inf	unif	singleC	S1:CBN, S1:CBN-A, S1:DiP, S1:DiP-A, S1:OT, S1:OT-A
623	No	9	9-B	1000	McF_6	Inf	unif	wholeT_0.01	S1:DiP, S1:DiP-A, S1:OT, S1:OT-A, S5:OT
624	No	9	9-B	1000	McF_6	Inf	unif	wholeT_0.5	S1:DiP, S1:DiP-A, S1:OT
625	No	9	9-B	200	Bozic	0	last	singleC	S1:OT, S1:OT-A
626	No	9	9-B	200	Bozic	0	last	wholeT_0.01	S1:CBN, S1:CBN-A, S1:OT, S1:OT-A, S5:OT-A
627	No	9	9-B	200	Bozic	0	last	wholeT_0.5	S1:CBN-A, S1:OT, S1:OT-A
628	No	9	9-B	200	Bozic	0	unif	singleC	J1:OT-A, S1:CBN, S1:CBN-A, S1:OT, S1:OT-A
629	No	9	9-B	200	Bozic	0	unif	wholeT_0.01	S1:CBN, S1:CBN-A
630	No	9	9-B	200	Bozic	0	unif	wholeT_0.5	J1:CBN, J1:CBN-A, J1:DiP, J1:DiP-A, J1:OT, J1:OT-A, J5:CBN, J5:CBN-A, J5:DiP, J5:DiP-A, J5:OT, S1:CBN, S1:CBN-A, S1:DiP, S1:DiP-A, S1:OT, S1:OT-A, S5:DiP, S5:DiP-A, S5:OT, S5:OT-A
631	No	9	9-B	200	Bozic	Inf	last	singleC	S1:OT, S1:OT-A, S5:OT, S5:OT-A
632	No	9	9-B	200	Bozic	Inf	last	wholeT_0.01	J1:CBN-A, J1:OT, J1:OT-A, S1:CBN-A, S1:OT, S1:OT-A, S5:OT, S5:OT-A
633	No	9	9-B	200	Bozic	Inf	last	wholeT_0.5	S1:OT, S1:OT-A
634	No	9	9-B	200	Bozic	Inf	unif	singleC	S1:CBN, S1:CBN-A, S1:OT, S1:OT-A
635	No	9	9-B	200	Bozic	Inf	unif	wholeT_0.01	J1:CBN, J1:CBN-A, S1:CBN, S1:CBN-A, S1:OT, S1:OT-A, S5:CBN, S5:CBN-A, S5:OT, S5:OT-A
636	No	9	9-B	200	Bozic	Inf	unif	wholeT_0.5	S1:CBN, S1:CBN-A, S1:OT, S1:OT-A
637	No	9	9-B	200	exp	0	last	singleC	S1:OT, S1:OT-A

Table 15: *(continued)*

	Conjunction	Drivers	Tree	S.Size	Model	sh	S.Time	S.Type	Best method(s)
638	No	9	9-B	200	exp	0	last	wholeT_0.01	S1:OT, S1:OT-A
639	No	9	9-B	200	exp	0	last	wholeT_0.5	S1:OT, S1:OT-A
640	No	9	9-B	200	exp	0	unif	singleC	none
641	No	9	9-B	200	exp	0	unif	wholeT_0.01	S1:CBN, S1:CBN-A, S1:OT, S1:OT-A
642	No	9	9-B	200	exp	0	unif	wholeT_0.5	J1:CBN, J1:CBN-A, J1:DiP, J1:DiP-A, J1:OT, J1:OT-A, J5:CBN, J5:CBN-A, J5:DiP, J5:DiP-A, J5:OT, J5:OT-A, S1:CBN, S1:CBN-A, S1:DiP, S1:DiP-A, S1:OT, S1:OT-A, S5:CBN, S5:DiP, S5:DiP-A, S5:OT, S5:OT-A
643	No	9	9-B	200	exp	Inf	last	singleC	S1:CBN, S1:OT, S1:OT-A, S5:OT, S5:OT-A
644	No	9	9-B	200	exp	Inf	last	wholeT_0.01	J1:CBN-A, J1:OT, J1:OT-A, S1:CBN, S1:CBN-A, S1:OT, S1:OT-A, S5:CBN-A, S5:OT, S5:OT-A
645	No	9	9-B	200	exp	Inf	last	wholeT_0.5	S1:CBN, S1:OT, S1:OT-A, S5:OT, S5:OT-A
646	No	9	9-B	200	exp	Inf	unif	singleC	S1:CBN, S1:CBN-A, S1:OT, S1:OT-A
647	No	9	9-B	200	exp	Inf	unif	wholeT_0.01	S1:CBN, S1:CBN-A, S1:OT, S1:OT-A
648	No	9	9-B	200	exp	Inf	unif	wholeT_0.5	S1:CBN, S1:CBN-A, S1:OT, S1:OT-A
649	No	9	9-B	200	McF_4	0	last	singleC	S1:OT, S1:OT-A
650	No	9	9-B	200	McF_4	0	last	wholeT_0.01	S1:OT, S1:OT-A, S5:OT, S5:OT-A
651	No	9	9-B	200	McF_4	0	last	wholeT_0.5	S1:OT, S1:OT-A
652	No	9	9-B	200	McF_4	0	unif	singleC	S1:CBN-A, S1:OT, S1:OT-A
653	No	9	9-B	200	McF_4	0	unif	wholeT_0.01	S1:CBN, S1:CBN-A, S1:OT, S1:OT-A
654	No	9	9-B	200	McF_4	0	unif	wholeT_0.5	S1:OT, S1:OT-A
655	No	9	9-B	200	McF_4	Inf	last	singleC	S1:OT, S1:OT-A
656	No	9	9-B	200	McF_4	Inf	last	wholeT_0.01	S1:OT, S1:OT-A, S5:OT, S5:OT-A
657	No	9	9-B	200	McF_4	Inf	last	wholeT_0.5	S1:OT, S1:OT-A
658	No	9	9-B	200	McF_4	Inf	unif	singleC	S1:OT, S1:OT-A
659	No	9	9-B	200	McF_4	Inf	unif	wholeT_0.01	S1:OT, S1:OT-A
660	No	9	9-B	200	McF_4	Inf	unif	wholeT_0.5	S1:OT, S1:OT-A
661	No	9	9-B	200	McF_6	0	last	singleC	S1:OT-A, S5:OT-A
662	No	9	9-B	200	McF_6	0	last	wholeT_0.01	S1:OT-A, S5:OT-A
663	No	9	9-B	200	McF_6	0	last	wholeT_0.5	S1:OT-A, S5:OT-A
664	No	9	9-B	200	McF_6	0	unif	singleC	S1:OT, S1:OT-A
665	No	9	9-B	200	McF_6	0	unif	wholeT_0.01	S1:OT, S1:OT-A
666	No	9	9-B	200	McF_6	0	unif	wholeT_0.5	S1:OT, S1:OT-A
667	No	9	9-B	200	McF_6	Inf	last	singleC	S1:OT-A, S5:OT-A
668	No	9	9-B	200	McF_6	Inf	last	wholeT_0.01	S1:OT-A, S5:OT-A

Table 15: *(continued)*

	Conjunction	Drivers	Tree	S.Size	Model	sh	S.Time	S.Type	Best method(s)
669	No	9	9-B	200	McF_6	Inf	last	wholeT_0.5	S1:OT-A, S5:OT-A
670	No	9	9-B	200	McF_6	Inf	unif	singleC	S1:OT, S1:OT-A
671	No	9	9-B	200	McF_6	Inf	unif	wholeT_0.01	S1:OT, S1:OT-A
672	No	9	9-B	200	McF_6	Inf	unif	wholeT_0.5	S1:OT, S1:OT-A
673	No	9	9-B	100	Bozic	0	last	singleC	S1:CBN, S1:CBN-A, S1:OT, S1:OT-A
674	No	9	9-B	100	Bozic	0	last	wholeT_0.01	S1:CBN-A, S1:OT, S1:OT-A
675	No	9	9-B	100	Bozic	0	last	wholeT_0.5	S1:CBN, S1:OT, S1:OT-A
676	No	9	9-B	100	Bozic	0	unif	singleC	S1:CBN, S1:CBN-A, S1:OT, S1:OT-A
677	No	9	9-B	100	Bozic	0	unif	wholeT_0.01	S1:CBN, S1:CBN-A
678	No	9	9-B	100	Bozic	0	unif	wholeT_0.5	J1:CBN, J1:CBN-A, J1:DiP, J1:DiP-A, J1:OT, J1:OT-A, J5:CBN, J5:CBN-A, J5:DiP-A, J5:OT, S1:CBN, S1:CBN-A, S1:DiP, S1:OT, S1:OT-A, S5:CBN, S5:CBN-A, S5:DiP, S5:OT
679	No	9	9-B	100	Bozic	Inf	last	singleC	S1:CBN, S1:CBN-A, S1:OT, S1:OT-A, S5:OT-A
680	No	9	9-B	100	Bozic	Inf	last	wholeT_0.01	J1:OT, J1:OT-A, S1:OT, S1:OT-A, S5:OT, S5:OT-A
681	No	9	9-B	100	Bozic	Inf	last	wholeT_0.5	S1:OT, S1:OT-A, S5:OT-A
682	No	9	9-B	100	Bozic	Inf	unif	singleC	S1:CBN, S1:CBN-A, S1:OT, S1:OT-A
683	No	9	9-B	100	Bozic	Inf	unif	wholeT_0.01	S1:CBN, S1:CBN-A, S1:OT, S1:OT-A, S5:CBN
684	No	9	9-B	100	Bozic	Inf	unif	wholeT_0.5	S1:CBN, S1:CBN-A, S1:OT, S1:OT-A
685	No	9	9-B	100	exp	0	last	singleC	S1:OT, S1:OT-A
686	No	9	9-B	100	exp	0	last	wholeT_0.01	S1:CBN, S1:CBN-A, S1:OT, S1:OT-A
687	No	9	9-B	100	exp	0	last	wholeT_0.5	S1:OT, S1:OT-A
688	No	9	9-B	100	exp	0	unif	singleC	J1:CBN-A, J1:DiP, J1:OT, J1:OT-A, J5:CBN, J5:CBN-A, J5:DiP, J5:DiP-A, J5:OT, J5:OT-A, S1:CBN, S1:CBN-A, S1:DiP, S1:DiP-A, S1:OT, S1:OT-A, S5:CBN, S5:CBN-A, S5:DiP-A, S5:OT, S5:OT-A
689	No	9	9-B	100	exp	0	unif	wholeT_0.01	S1:CBN, S1:CBN-A, S1:OT, S1:OT-A
690	No	9	9-B	100	exp	0	unif	wholeT_0.5	J1:CBN, J1:CBN-A, J1:DiP, J1:DiP-A, J1:OT, J1:OT-A, J5:CBN, J5:CBN-A, J5:DiP-A, J5:OT, J5:OT-A, S1:CBN, S1:CBN-A, S1:DiP, S1:DiP-A, S1:OT, S1:OT-A, S5:CBN, S5:CBN-A, S5:DiP, S5:DiP-A, S5:OT, S5:OT-A
691	No	9	9-B	100	exp	Inf	last	singleC	S1:OT, S1:OT-A

Table 15: *(continued)*

	Conjunction	Drivers	Tree	S.Size	Model	sh	S.Time	S.Type	Best method(s)
692	No	9	9-B	100	exp	Inf	last	wholeT_0.01	J1:CBN-A, J1:OT, J1:OT-A, S1:CBN, S1:CBN-A, S1:OT, S1:OT-A, S5:CBN-A, S5:OT, S5:OT-A
693	No	9	9-B	100	exp	Inf	last	wholeT_0.5	S1:OT, S1:OT-A, S5:OT, S5:OT-A
694	No	9	9-B	100	exp	Inf	unif	singleC	S1:CBN, S1:CBN-A, S1:OT, S1:OT-A
695	No	9	9-B	100	exp	Inf	unif	wholeT_0.01	J1:CBN, J1:CBN-A, S1:CBN, S1:CBN-A, S1:OT, S1:OT-A
696	No	9	9-B	100	exp	Inf	unif	wholeT_0.5	S1:CBN, S1:CBN-A, S1:OT, S1:OT-A
697	No	9	9-B	100	McF_4	0	last	singleC	S1:OT, S1:OT-A
698	No	9	9-B	100	McF_4	0	last	wholeT_0.01	S1:OT, S1:OT-A, S5:OT, S5:OT-A
699	No	9	9-B	100	McF_4	0	last	wholeT_0.5	S1:OT, S1:OT-A
700	No	9	9-B	100	McF_4	0	unif	singleC	S1:CBN-A, S1:OT, S1:OT-A
701	No	9	9-B	100	McF_4	0	unif	wholeT_0.01	S1:CBN, S1:CBN-A, S1:OT, S1:OT-A
702	No	9	9-B	100	McF_4	0	unif	wholeT_0.5	S1:OT, S1:OT-A
703	No	9	9-B	100	McF_4	Inf	last	singleC	S1:OT, S1:OT-A
704	No	9	9-B	100	McF_4	Inf	last	wholeT_0.01	S1:OT, S1:OT-A, S5:OT, S5:OT-A
705	No	9	9-B	100	McF_4	Inf	last	wholeT_0.5	S1:OT, S1:OT-A
706	No	9	9-B	100	McF_4	Inf	unif	singleC	S1:CBN-A, S1:OT, S1:OT-A
707	No	9	9-B	100	McF_4	Inf	unif	wholeT_0.01	S1:OT, S1:OT-A
708	No	9	9-B	100	McF_4	Inf	unif	wholeT_0.5	S1:OT, S1:OT-A
709	No	9	9-B	100	McF_6	0	last	singleC	S1:OT-A, S5:OT-A
710	No	9	9-B	100	McF_6	0	last	wholeT_0.01	S1:OT-A, S5:OT-A
711	No	9	9-B	100	McF_6	0	last	wholeT_0.5	S1:OT-A, S5:OT-A
712	No	9	9-B	100	McF_6	0	unif	singleC	S1:OT, S1:OT-A
713	No	9	9-B	100	McF_6	0	unif	wholeT_0.01	S1:OT, S1:OT-A
714	No	9	9-B	100	McF_6	0	unif	wholeT_0.5	S1:OT, S1:OT-A
715	No	9	9-B	100	McF_6	Inf	last	singleC	S1:OT-A, S5:OT-A
716	No	9	9-B	100	McF_6	Inf	last	wholeT_0.01	S1:OT-A, S5:OT-A
717	No	9	9-B	100	McF_6	Inf	last	wholeT_0.5	S1:OT-A, S5:OT-A
718	No	9	9-B	100	McF_6	Inf	unif	singleC	S1:OT, S1:OT-A
719	No	9	9-B	100	McF_6	Inf	unif	wholeT_0.01	S1:OT, S1:OT-A
720	No	9	9-B	100	McF_6	Inf	unif	wholeT_0.5	S1:OT, S1:OT-A
721	No	7	7-B	1000	Bozic	0	last	singleC	S1:OT-A, S5:OT-A
722	No	7	7-B	1000	Bozic	0	last	wholeT_0.01	J1:DiP-A, J1:OT-A, J5:DiP-A, J5:OT-A, S1:DiP-A, S1:OT-A, S5:OT-A
723	No	7	7-B	1000	Bozic	0	last	wholeT_0.5	S1:OT-A
724	No	7	7-B	1000	Bozic	0	unif	singleC	S1:OT
725	No	7	7-B	1000	Bozic	0	unif	wholeT_0.01	S1:CBN, S1:CBN-A, S1:DiP-A, S1:OT, S1:OT-A

Table 15: *(continued)*

	Conjunction	Drivers	Tree	S.Size	Model	sh	S.Time	S.Type	Best method(s)
726	No	7	7-B	1000	Bozic	0	unif	wholeT_0.5	J1:CBN-A, J1:DiP-A, J1:OT-A, J5:CBN, J5:CBN-A, J5:DiP-A, J5:OT, J5:OT-A, S1:CBN, S1:CBN-A, S1:DiP-A, S1:OT, S1:OT-A, S5:CBN, S5:CBN-A, S5:OT-A
727	No	7	7-B	1000	Bozic	Inf	last	singleC	S1:DiP-A, S1:OT-A
728	No	7	7-B	1000	Bozic	Inf	last	wholeT_0.01	J1:DiP-A, J1:OT-A, S1:DiP-A, S1:OT-A, S5:DiP-A, S5:OT-A
729	No	7	7-B	1000	Bozic	Inf	last	wholeT_0.5	S1:DiP-A, S1:OT-A
730	No	7	7-B	1000	Bozic	Inf	unif	singleC	S1:CBN, S1:CBN-A, S1:OT, S1:OT-A
731	No	7	7-B	1000	Bozic	Inf	unif	wholeT_0.01	J1:CBN-A, J1:OT-A, S1:CBN, S1:CBN-A, S1:OT, S1:OT-A
732	No	7	7-B	1000	Bozic	Inf	unif	wholeT_0.5	S1:CBN, S1:CBN-A, S1:OT, S1:OT-A
733	No	7	7-B	1000	exp	0	last	singleC	S1:OT-A
734	No	7	7-B	1000	exp	0	last	wholeT_0.01	J1:OT-A, S1:OT-A, S5:OT-A
735	No	7	7-B	1000	exp	0	last	wholeT_0.5	S1:CBN-A, S1:OT-A
736	No	7	7-B	1000	exp	0	unif	singleC	none
737	No	7	7-B	1000	exp	0	unif	wholeT_0.01	S1:CBN-A, S1:OT, S1:OT-A
738	No	7	7-B	1000	exp	0	unif	wholeT_0.5	none
739	No	7	7-B	1000	exp	Inf	last	singleC	S1:OT-A
740	No	7	7-B	1000	exp	Inf	last	wholeT_0.01	S1:OT-A, S5:OT-A
741	No	7	7-B	1000	exp	Inf	last	wholeT_0.5	S1:OT-A, S5:OT-A
742	No	7	7-B	1000	exp	Inf	unif	singleC	S1:CBN, S1:OT, S1:OT-A
743	No	7	7-B	1000	exp	Inf	unif	wholeT_0.01	J1:CBN, J1:CBN-A, J1:OT, J1:OT-A, J5:CBN, J5:CBN-A, J5:OT, J5:OT-A, S1:CBN, S1:CBN-A, S1:OT, S1:OT-A, S5:CBN, S5:CBN-A, S5:OT, S5:OT-A
744	No	7	7-B	1000	exp	Inf	unif	wholeT_0.5	S1:OT
745	No	7	7-B	1000	McF_4	0	last	singleC	S1:DiP-A, S1:OT-A, S5:DiP-A, S5:OT-A
746	No	7	7-B	1000	McF_4	0	last	wholeT_0.01	S1:DiP-A, S1:OT-A, S5:DiP-A, S5:OT-A
747	No	7	7-B	1000	McF_4	0	last	wholeT_0.5	S1:DiP-A, S1:OT-A, S5:DiP-A, S5:OT-A
748	No	7	7-B	1000	McF_4	0	unif	singleC	S1:DiP-A, S1:OT, S1:OT-A
749	No	7	7-B	1000	McF_4	0	unif	wholeT_0.01	S1:DiP-A, S1:OT, S1:OT-A
750	No	7	7-B	1000	McF_4	0	unif	wholeT_0.5	S1:DiP-A, S1:OT, S1:OT-A
751	No	7	7-B	1000	McF_4	Inf	last	singleC	S1:DiP-A, S1:OT-A, S5:DiP-A, S5:OT-A
752	No	7	7-B	1000	McF_4	Inf	last	wholeT_0.01	S1:DiP-A, S1:OT-A, S5:DiP-A, S5:OT-A
753	No	7	7-B	1000	McF_4	Inf	last	wholeT_0.5	S1:DiP-A, S1:OT-A, S5:DiP-A, S5:OT-A
754	No	7	7-B	1000	McF_4	Inf	unif	singleC	S1:OT, S1:OT-A
755	No	7	7-B	1000	McF_4	Inf	unif	wholeT_0.01	S1:OT, S1:OT-A
756	No	7	7-B	1000	McF_4	Inf	unif	wholeT_0.5	S1:OT, S1:OT-A

Table 15: *(continued)*

	Conjunction	Drivers	Tree	S.Size	Model	sh	S.Time	S.Type	Best method(s)
757	No	7	7-B	1000	McF_6	0	last	singleC	J1:DiP-A, J1:OT-A, S1:DiP-A, S1:OT-A, S5:DiP-A, S5:OT-A
758	No	7	7-B	1000	McF_6	0	last	wholeT_0.01	J1:DiP-A, J1:OT-A, J5:DiP-A, J5:OT-A, S1:DiP-A, S1:OT-A, S5:DiP-A, S5:OT-A
759	No	7	7-B	1000	McF_6	0	last	wholeT_0.5	J1:DiP-A, J1:OT-A, S1:DiP-A, S1:OT-A, S5:DiP-A, S5:OT-A
760	No	7	7-B	1000	McF_6	0	unif	singleC	S1:DiP, S1:DiP-A, S1:OT, S1:OT-A, S5:DiP, S5:DiP-A, S5:OT, S5:OT-A
761	No	7	7-B	1000	McF_6	0	unif	wholeT_0.01	S1:DiP, S1:DiP-A, S1:OT, S1:OT-A, S5:DiP, S5:DiP-A, S5:OT, S5:OT-A
762	No	7	7-B	1000	McF_6	0	unif	wholeT_0.5	S1:DiP, S1:DiP-A, S1:OT, S1:OT-A, S5:DiP, S5:DiP-A, S5:OT, S5:OT-A
763	No	7	7-B	1000	McF_6	Inf	last	singleC	J1:DiP-A, J1:OT-A, S1:DiP-A, S1:OT-A, S5:DiP-A, S5:OT-A
764	No	7	7-B	1000	McF_6	Inf	last	wholeT_0.01	J1:DiP-A, J1:OT-A, J5:DiP-A, J5:OT-A, S1:DiP-A, S1:OT-A, S5:DiP-A, S5:OT-A
765	No	7	7-B	1000	McF_6	Inf	last	wholeT_0.5	J1:DiP-A, J1:OT-A, S1:DiP-A, S1:OT-A, S5:DiP-A, S5:OT-A
766	No	7	7-B	1000	McF_6	Inf	unif	singleC	S1:DiP-A, S1:OT, S1:OT-A, S5:DiP-A, S5:OT, S5:OT-A
767	No	7	7-B	1000	McF_6	Inf	unif	wholeT_0.01	S1:DiP-A, S1:OT, S1:OT-A, S5:DiP-A, S5:OT, S5:OT-A
768	No	7	7-B	1000	McF_6	Inf	unif	wholeT_0.5	S1:DiP-A, S1:OT, S1:OT-A, S5:DiP-A, S5:OT, S5:OT-A
769	No	7	7-B	200	Bozic	0	last	singleC	S1:OT-A, S5:OT-A
770	No	7	7-B	200	Bozic	0	last	wholeT_0.01	S1:OT-A
771	No	7	7-B	200	Bozic	0	last	wholeT_0.5	S1:OT-A
772	No	7	7-B	200	Bozic	0	unif	singleC	S1:CBN, S1:CBN-A, S1:OT, S1:OT-A
773	No	7	7-B	200	Bozic	0	unif	wholeT_0.01	J1:CBN-A, J1:OT, J1:OT-A, S1:CBN, S1:CBN-A, S1:OT, S1:OT-A, S5:CBN, S5:CBN-A, S5:OT-A
774	No	7	7-B	200	Bozic	0	unif	wholeT_0.5	S1:CBN, S1:CBN-A, S1:OT, S1:OT-A
775	No	7	7-B	200	Bozic	Inf	last	singleC	S1:OT-A
776	No	7	7-B	200	Bozic	Inf	last	wholeT_0.01	J1:OT-A, S1:OT-A, S5:OT-A
777	No	7	7-B	200	Bozic	Inf	last	wholeT_0.5	S1:OT-A
778	No	7	7-B	200	Bozic	Inf	unif	singleC	S1:OT, S1:OT-A
779	No	7	7-B	200	Bozic	Inf	unif	wholeT_0.01	S1:CBN, S1:CBN-A, S1:OT, S1:OT-A
780	No	7	7-B	200	Bozic	Inf	unif	wholeT_0.5	S1:CBN, S1:OT, S1:OT-A
781	No	7	7-B	200	exp	0	last	singleC	S1:OT-A
782	No	7	7-B	200	exp	0	last	wholeT_0.01	S1:OT-A, S5:OT-A

Table 15: *(continued)*

	Conjunction	Drivers	Tree	S.Size	Model	sh	S.Time	S.Type	Best method(s)
783	No	7	7-B	200	exp	0	last	wholeT_0.5	S1:CBN-A, S1:OT-A
784	No	7	7-B	200	exp	0	unif	singleC	J1:CBN, J1:CBN-A, J1:OT, J1:OT-A, S1:CBN, S1:CBN-A, S1:OT, S1:OT-A
785	No	7	7-B	200	exp	0	unif	wholeT_0.01	S1:CBN, S1:CBN-A, S1:OT, S1:OT-A
786	No	7	7-B	200	exp	0	unif	wholeT_0.5	J1:CBN, J1:CBN-A, J1:DiP-A, J1:OT, J1:OT-A, J5:CBN, J5:CBN-A, J5:DiP, J5:OT, J5:OT-A, S1:CBN, S1:CBN-A, S1:DiP, S1:DiP-A, S1:OT, S1:OT-A, S5:CBN, S5:CBN-A, S5:DiP, S5:DiP-A, S5:OT, S5:OT-A
787	No	7	7-B	200	exp	Inf	last	singleC	S1:OT-A
788	No	7	7-B	200	exp	Inf	last	wholeT_0.01	S1:OT-A, S5:OT-A
789	No	7	7-B	200	exp	Inf	last	wholeT_0.5	S1:OT-A, S5:OT-A
790	No	7	7-B	200	exp	Inf	unif	singleC	S1:CBN, S1:CBN-A, S1:OT, S1:OT-A
791	No	7	7-B	200	exp	Inf	unif	wholeT_0.01	J1:OT, J1:OT-A, S1:CBN, S1:CBN-A, S1:OT, S1:OT-A
792	No	7	7-B	200	exp	Inf	unif	wholeT_0.5	S1:OT, S1:OT-A
793	No	7	7-B	200	McF_4	0	last	singleC	S1:OT-A, S5:OT-A
794	No	7	7-B	200	McF_4	0	last	wholeT_0.01	S1:OT-A, S5:OT-A
795	No	7	7-B	200	McF_4	0	last	wholeT_0.5	S1:OT-A, S5:OT-A
796	No	7	7-B	200	McF_4	0	unif	singleC	S1:OT, S1:OT-A
797	No	7	7-B	200	McF_4	0	unif	wholeT_0.01	S1:OT, S1:OT-A
798	No	7	7-B	200	McF_4	0	unif	wholeT_0.5	S1:OT, S1:OT-A
799	No	7	7-B	200	McF_4	Inf	last	singleC	S1:OT-A, S5:OT-A
800	No	7	7-B	200	McF_4	Inf	last	wholeT_0.01	S1:OT-A, S5:OT-A
801	No	7	7-B	200	McF_4	Inf	last	wholeT_0.5	S1:OT-A, S5:OT-A
802	No	7	7-B	200	McF_4	Inf	unif	singleC	S1:OT, S1:OT-A
803	No	7	7-B	200	McF_4	Inf	unif	wholeT_0.01	S1:OT, S1:OT-A
804	No	7	7-B	200	McF_4	Inf	unif	wholeT_0.5	S1:OT, S1:OT-A
805	No	7	7-B	200	McF_6	0	last	singleC	J1:OT-A, J5:OT-A, S1:OT-A, S5:OT-A
806	No	7	7-B	200	McF_6	0	last	wholeT_0.01	J1:DiP-A, J1:OT-A, J5:DiP-A, J5:OT-A, S1:DiP-A, S1:OT-A, S5:DiP-A, S5:OT-A
807	No	7	7-B	200	McF_6	0	last	wholeT_0.5	J1:OT-A, S1:OT-A, S5:OT-A
808	No	7	7-B	200	McF_6	0	unif	singleC	S1:OT, S1:OT-A, S5:OT, S5:OT-A
809	No	7	7-B	200	McF_6	0	unif	wholeT_0.01	J1:OT-A, S1:OT, S1:OT-A, S5:OT, S5:OT-A
810	No	7	7-B	200	McF_6	0	unif	wholeT_0.5	S1:OT, S1:OT-A, S5:OT, S5:OT-A
811	No	7	7-B	200	McF_6	Inf	last	singleC	J1:OT-A, J5:OT-A, S1:OT-A, S5:OT-A
812	No	7	7-B	200	McF_6	Inf	last	wholeT_0.01	J1:OT-A, J5:OT-A, S1:OT-A, S5:OT-A
813	No	7	7-B	200	McF_6	Inf	last	wholeT_0.5	J1:OT-A, S1:OT-A, S5:OT-A

Table 15: *(continued)*

	Conjunction	Drivers	Tree	S.Size	Model	sh	S.Time	S.Type	Best method(s)
814	No	7	7-B	200	McF_6	Inf	unif	singleC	S1:OT, S1:OT-A, S5:OT, S5:OT-A
815	No	7	7-B	200	McF_6	Inf	unif	wholeT_0.01	S1:OT, S1:OT-A, S5:OT, S5:OT-A
816	No	7	7-B	200	McF_6	Inf	unif	wholeT_0.5	J1:OT-A, S1:OT, S1:OT-A, S5:OT, S5:OT-A
817	No	7	7-B	100	Bozic	0	last	singleC	S1:OT-A
818	No	7	7-B	100	Bozic	0	last	wholeT_0.01	S1:OT-A
819	No	7	7-B	100	Bozic	0	last	wholeT_0.5	S1:OT-A
820	No	7	7-B	100	Bozic	0	unif	singleC	S1:CBN, S1:CBN-A, S1:OT, S1:OT-A
821	No	7	7-B	100	Bozic	0	unif	wholeT_0.01	S1:CBN, S1:CBN-A, S1:OT, S1:OT-A
822	No	7	7-B	100	Bozic	0	unif	wholeT_0.5	S1:CBN, S1:CBN-A, S1:OT, S1:OT-A
823	No	7	7-B	100	Bozic	Inf	last	singleC	S1:OT-A
824	No	7	7-B	100	Bozic	Inf	last	wholeT_0.01	S1:CBN-A, S1:OT-A, S5:OT-A
825	No	7	7-B	100	Bozic	Inf	last	wholeT_0.5	S1:OT-A
826	No	7	7-B	100	Bozic	Inf	unif	singleC	S1:CBN-A, S1:OT, S1:OT-A
827	No	7	7-B	100	Bozic	Inf	unif	wholeT_0.01	J1:CBN, J1:CBN-A, J1:OT, J1:OT-A, S1:CBN, S1:CBN-A, S1:OT, S1:OT-A
828	No	7	7-B	100	Bozic	Inf	unif	wholeT_0.5	S1:OT, S1:OT-A
829	No	7	7-B	100	exp	0	last	singleC	S1:OT-A
830	No	7	7-B	100	exp	0	last	wholeT_0.01	S1:OT-A, S5:OT-A
831	No	7	7-B	100	exp	0	last	wholeT_0.5	S1:OT-A
832	No	7	7-B	100	exp	0	unif	singleC	J1:CBN-A, J1:OT, J1:OT-A, S1:CBN, S1:CBN-A, S1:OT, S1:OT-A
833	No	7	7-B	100	exp	0	unif	wholeT_0.01	S1:CBN, S1:CBN-A, S1:OT, S1:OT-A
834	No	7	7-B	100	exp	0	unif	wholeT_0.5	J1:CBN-A, J1:OT, J1:OT-A, S1:CBN, S1:CBN-A, S1:OT, S1:OT-A
835	No	7	7-B	100	exp	Inf	last	singleC	S1:OT-A
836	No	7	7-B	100	exp	Inf	last	wholeT_0.01	S1:OT-A, S5:OT-A
837	No	7	7-B	100	exp	Inf	last	wholeT_0.5	S1:OT-A
838	No	7	7-B	100	exp	Inf	unif	singleC	S1:OT, S1:OT-A
839	No	7	7-B	100	exp	Inf	unif	wholeT_0.01	S1:CBN, S1:CBN-A, S1:OT, S1:OT-A
840	No	7	7-B	100	exp	Inf	unif	wholeT_0.5	S1:CBN, S1:CBN-A, S1:OT, S1:OT-A
841	No	7	7-B	100	McF_4	0	last	singleC	S1:OT-A
842	No	7	7-B	100	McF_4	0	last	wholeT_0.01	S1:OT-A, S5:OT-A
843	No	7	7-B	100	McF_4	0	last	wholeT_0.5	S1:OT-A
844	No	7	7-B	100	McF_4	0	unif	singleC	S1:OT, S1:OT-A
845	No	7	7-B	100	McF_4	0	unif	wholeT_0.01	S1:OT, S1:OT-A
846	No	7	7-B	100	McF_4	0	unif	wholeT_0.5	S1:OT, S1:OT-A
847	No	7	7-B	100	McF_4	Inf	last	singleC	S1:OT-A, S5:OT-A
848	No	7	7-B	100	McF_4	Inf	last	wholeT_0.01	S1:OT-A, S5:OT-A
849	No	7	7-B	100	McF_4	Inf	last	wholeT_0.5	S1:OT-A, S5:OT-A

Table 15: *(continued)*

	Conjunction	Drivers	Tree	S.Size	Model	sh	S.Time	S.Type	Best method(s)
850	No	7	7-B	100	McF_4	Inf	unif	singleC	S1:OT, S1:OT-A
851	No	7	7-B	100	McF_4	Inf	unif	wholeT_0.01	S1:OT, S1:OT-A
852	No	7	7-B	100	McF_4	Inf	unif	wholeT_0.5	S1:OT, S1:OT-A
853	No	7	7-B	100	McF_6	0	last	singleC	J1:OT-A, S1:OT-A, S5:OT-A
854	No	7	7-B	100	McF_6	0	last	wholeT_0.01	J1:OT-A, J5:OT-A, S1:OT-A, S5:OT-A
855	No	7	7-B	100	McF_6	0	last	wholeT_0.5	J1:OT-A, S1:OT-A, S5:OT-A
856	No	7	7-B	100	McF_6	0	unif	singleC	J1:OT-A, S1:OT, S1:OT-A, S5:OT, S5:OT-A
857	No	7	7-B	100	McF_6	0	unif	wholeT_0.01	S1:OT-A, S5:OT, S5:OT-A
858	No	7	7-B	100	McF_6	0	unif	wholeT_0.5	S1:OT, S1:OT-A, S5:OT, S5:OT-A
859	No	7	7-B	100	McF_6	Inf	last	singleC	J1:OT-A, S1:OT-A, S5:OT-A
860	No	7	7-B	100	McF_6	Inf	last	wholeT_0.01	J1:OT-A, J5:OT-A, S1:OT-A, S5:OT-A
861	No	7	7-B	100	McF_6	Inf	last	wholeT_0.5	J1:OT-A, S1:OT-A
862	No	7	7-B	100	McF_6	Inf	unif	singleC	S1:OT, S1:OT-A, S5:OT, S5:OT-A
863	No	7	7-B	100	McF_6	Inf	unif	wholeT_0.01	J1:OT-A, S1:OT-A, S5:OT, S5:OT-A
864	No	7	7-B	100	McF_6	Inf	unif	wholeT_0.5	S1:OT, S1:OT-A, S5:OT, S5:OT-A

5.4 Confidence sets (MCB), FPF, Drivers Unknown

Table 16: Confidence sets (method MCB) when Drivers are Unknown for measure FPF.

	Conjunction	Drivers	Tree	S.Size	Model	sh	S.Time	S.Type	Best method(s)
1	Yes	11	11-A	1000	Bozic	0	last	singleC	J1:DiP, J1:DiP-A, J1:OT-A, J5:DiP, J5:DiP-A, J5:OT, J5:OT-A, S1:OT, S1:OT-A, S5:OT, S5:OT-A
2	Yes	11	11-A	1000	Bozic	0	last	wholeT_0.01	J1:DiP, J1:DiP-A, J5:DiP, J5:DiP-A, S1:DiP, S1:DiP-A, S5:DiP, S5:DiP-A
3	Yes	11	11-A	1000	Bozic	0	last	wholeT_0.5	J1:DiP, J1:DiP-A, J1:OT, J1:OT-A, J5:DiP, J5:DiP-A, J5:OT, J5:OT-A, S1:OT, S1:OT-A, S5:DiP, S5:DiP-A, S5:OT, S5:OT-A
4	Yes	11	11-A	1000	Bozic	0	unif	singleC	J1:DiP, J1:DiP-A, J1:OT, J1:OT-A, J5:CBN, J5:CBN-A, J5:DiP, J5:DiP-A, J5:OT, J5:OT-A, S1:DiP, S1:DiP-A, S1:OT, S1:OT-A, S5:DiP, S5:DiP-A, S5:OT, S5:OT-A
5	Yes	11	11-A	1000	Bozic	0	unif	wholeT_0.01	J5:DiP, J5:DiP-A, J5:OT, J5:OT-A
6	Yes	11	11-A	1000	Bozic	0	unif	wholeT_0.5	J1:CBN, J1:CBN-A, J1:DiP, J1:DiP-A, J1:OT, J1:OT-A, J5:CBN, J5:CBN-A, J5:DiP, J5:DiP-A, J5:OT, J5:OT-A, S1:DiP-A, S1:OT, S1:OT-A, S5:DiP, S5:DiP-A, S5:OT, S5:OT-A
7	Yes	11	11-A	1000	Bozic	Inf	last	singleC	J1:DiP, J1:DiP-A, J1:OT, J1:OT-A, J5:DiP, J5:DiP-A, J5:OT, J5:OT-A, S1:DiP-A, S5:DiP, S5:DiP-A, S5:OT, S5:OT-A
8	Yes	11	11-A	1000	Bozic	Inf	last	wholeT_0.01	J5:DiP, J5:DiP-A, J5:OT, J5:OT-A
9	Yes	11	11-A	1000	Bozic	Inf	last	wholeT_0.5	J1:DiP, J1:DiP-A, J1:OT, J1:OT-A, J5:DiP, J5:DiP-A, J5:OT, J5:OT-A, S5:DiP, S5:DiP-A, S5:OT, S5:OT-A
10	Yes	11	11-A	1000	Bozic	Inf	unif	singleC	J1:DiP, J1:DiP-A, J1:OT, J1:OT-A, J5:DiP, J5:DiP-A, J5:OT, J5:OT-A, S5:DiP, S5:DiP-A, S5:OT, S5:OT-A
11	Yes	11	11-A	1000	Bozic	Inf	unif	wholeT_0.01	J5:DiP, J5:DiP-A, J5:OT, J5:OT-A
12	Yes	11	11-A	1000	Bozic	Inf	unif	wholeT_0.5	J1:DiP, J1:DiP-A, J1:OT, J1:OT-A, J5:DiP, J5:DiP-A, J5:OT, J5:OT-A, S5:DiP, S5:DiP-A, S5:OT, S5:OT-A

Table 16: *(continued)*

	Conjunction	Drivers	Tree	S.Size	Model	sh	S.Time	S.Type	Best method(s)
13	Yes	11	11-A	1000	exp	0	last	singleC	J1:DiP, J1:DiP-A, J1:OT, J1:OT-A, J5:CBN, J5:CBN-A, J5:DiP, J5:DiP-A, J5:OT, J5:OT-A, S1:DiP, S1:DiP-A, S1:OT, S1:OT-A, S5:DiP, S5:DiP-A, S5:OT, S5:OT-A
14	Yes	11	11-A	1000	exp	0	last	wholeT_0.01	J1:DiP, J1:DiP-A, J1:OT, J1:OT-A, J5:DiP, J5:DiP-A, J5:OT, J5:OT-A, S1:OT
15	Yes	11	11-A	1000	exp	0	last	wholeT_0.5	J1:OT, J1:OT-A, J5:CBN, J5:CBN-A, J5:DiP-A, J5:OT, J5:OT-A, S1:DiP-A, S1:OT, S1:OT-A, S5:DiP, S5:DiP-A, S5:OT, S5:OT-A
16	Yes	11	11-A	1000	exp	0	unif	singleC	J1:CBN, J1:CBN-A, J1:DiP, J1:DiP-A, J1:OT, J1:OT-A, J5:CBN, J5:CBN-A, J5:DiP, J5:DiP-A, J5:OT, J5:OT-A, S1:DiP, S1:DiP-A, S1:OT, S1:OT-A, S5:DiP, S5:DiP-A, S5:OT, S5:OT-A
17	Yes	11	11-A	1000	exp	0	unif	wholeT_0.01	J1:DiP, J1:DiP-A, J1:OT, J1:OT-A, J5:CBN, J5:CBN-A, J5:DiP, J5:DiP-A, J5:OT, J5:OT-A, S5:DiP, S5:DiP-A, S5:OT, S5:OT-A
18	Yes	11	11-A	1000	exp	0	unif	wholeT_0.5	J1:CBN, J1:CBN-A, J1:DiP, J1:DiP-A, J1:OT, J1:OT-A, J5:CBN, J5:CBN-A, J5:DiP, J5:DiP-A, J5:OT, J5:OT-A, S1:DiP, S1:DiP-A, S1:OT, S1:OT-A, S5:DiP, S5:DiP-A, S5:OT, S5:OT-A
19	Yes	11	11-A	1000	exp	Inf	last	singleC	J1:DiP, J1:DiP-A, J1:OT, J1:OT-A, J5:DiP, J5:DiP-A, J5:OT, J5:OT-A, S5:DiP, S5:OT
20	Yes	11	11-A	1000	exp	Inf	last	wholeT_0.01	J5:DiP, J5:DiP-A, J5:OT, J5:OT-A, S5:DiP, S5:DiP-A, S5:OT, S5:OT-A
21	Yes	11	11-A	1000	exp	Inf	last	wholeT_0.5	J1:DiP, J1:DiP-A, J1:OT, J1:OT-A, J5:DiP, J5:DiP-A, J5:OT, J5:OT-A, S1:DiP-A, S5:DiP, S5:DiP-A, S5:OT, S5:OT-A
22	Yes	11	11-A	1000	exp	Inf	unif	singleC	J1:DiP, J1:OT, J1:OT-A, J5:CBN, J5:CBN-A, J5:DiP, J5:DiP-A, J5:OT, J5:OT-A, S5:DiP, S5:DiP-A, S5:OT, S5:OT-A

Table 16: *(continued)*

	Conjunction	Drivers	Tree	S.Size	Model	sh	S.Time	S.Type	Best method(s)
23	Yes	11	11-A	1000	exp	Inf	unif	wholeT_0.01	J5:DiP, J5:DiP-A, J5:OT, J5:OT-A, S5:DiP, S5:DiP-A, S5:OT, S5:OT-A
24	Yes	11	11-A	1000	exp	Inf	unif	wholeT_0.5	J1:DiP, J1:DiP-A, J1:OT, J1:OT-A, J5:CBN, J5:CBN-A, J5:DiP, J5:DiP-A, J5:OT, J5:OT-A, S5:DiP, S5:OT, S5:OT-A
25	Yes	11	11-A	1000	McF_4	0	last	singleC	J1:DiP, J1:DiP-A, J1:OT, J1:OT-A, J5:CBN, J5:DiP, J5:DiP-A, J5:OT, J5:OT-A, S1:DiP-A, S1:OT, S1:OT-A, S5:DiP, S5:DiP-A, S5:OT, S5:OT-A
26	Yes	11	11-A	1000	McF_4	0	last	wholeT_0.01	J1:DiP, J1:DiP-A, J1:OT, J1:OT-A, J5:DiP, J5:DiP-A, J5:OT, J5:OT-A, S5:DiP, S5:DiP-A, S5:OT, S5:OT-A
27	Yes	11	11-A	1000	McF_4	0	last	wholeT_0.5	J1:DiP, J1:DiP-A, J1:OT, J1:OT-A, J5:DiP, J5:DiP-A, J5:OT, J5:OT-A, S1:OT, S1:OT-A, S5:DiP, S5:DiP-A, S5:OT, S5:OT-A
28	Yes	11	11-A	1000	McF_4	0	unif	singleC	J1:CBN-A, J1:DiP, J1:DiP-A, J1:OT, J1:OT-A, J5:DiP, J5:DiP-A, J5:OT, J5:OT-A, S1:DiP, S1:DiP-A, S1:OT, S5:DiP, S5:DiP-A, S5:OT, S5:OT-A
29	Yes	11	11-A	1000	McF_4	0	unif	wholeT_0.01	J1:CBN-A, J1:DiP, J1:DiP-A, J1:OT, J1:OT-A, J5:DiP, J5:DiP-A, J5:OT, J5:OT-A, S5:DiP, S5:DiP-A, S5:OT, S5:OT-A
30	Yes	11	11-A	1000	McF_4	0	unif	wholeT_0.5	J1:CBN, J1:CBN-A, J1:DiP, J1:DiP-A, J1:OT, J1:OT-A, J5:DiP, J5:DiP-A, J5:OT, J5:OT-A, S1:DiP, S1:DiP-A, S1:OT, S1:OT-A, S5:CBN-A, S5:DiP, S5:DiP-A, S5:OT, S5:OT-A
31	Yes	11	11-A	1000	McF_4	Inf	last	singleC	J1:DiP-A, J1:OT, J1:OT-A, J5:DiP, J5:DiP-A, J5:OT, J5:OT-A, S1:OT, S5:DiP, S5:DiP-A, S5:OT, S5:OT-A
32	Yes	11	11-A	1000	McF_4	Inf	last	wholeT_0.01	J1:DiP, J1:DiP-A, J1:OT, J1:OT-A, J5:DiP, J5:DiP-A, J5:OT, J5:OT-A, S5:DiP, S5:DiP-A, S5:OT, S5:OT-A
33	Yes	11	11-A	1000	McF_4	Inf	last	wholeT_0.5	J1:DiP, J1:DiP-A, J1:OT, J1:OT-A, J5:DiP, J5:DiP-A, J5:OT, J5:OT-A, S1:DiP-A, S1:OT, S1:OT-A, S5:DiP, S5:DiP-A, S5:OT, S5:OT-A

Table 16: *(continued)*

	Conjunction	Drivers	Tree	S.Size	Model	sh	S.Time	S.Type	Best method(s)
34	Yes	11	11-A	1000	McF_4	Inf	unif	singleC	J1:CBN-A, J1:DiP, J1:DiP-A, J1:OT, J1:OT-A, J5:DiP, J5:DiP-A, J5:OT, J5:OT-A, S1:DiP, S1:DiP-A, S1:OT, S1:OT-A, S5:CBN-A, S5:DiP, S5:DiP-A, S5:OT, S5:OT-A
35	Yes	11	11-A	1000	McF_4	Inf	unif	wholeT_0.01	J1:CBN-A, J1:DiP, J1:DiP-A, J1:OT, J1:OT-A, J5:DiP, J5:DiP-A, J5:OT, J5:OT-A, S5:CBN-A, S5:DiP, S5:DiP-A, S5:OT, S5:OT-A
36	Yes	11	11-A	1000	McF_4	Inf	unif	wholeT_0.5	J1:CBN-A, J1:DiP, J1:DiP-A, J1:OT, J1:OT-A, J5:DiP, J5:DiP-A, J5:OT, J5:OT-A, S1:DiP, S1:DiP-A, S1:OT, S1:OT-A, S5:DiP, S5:DiP-A, S5:OT, S5:OT-A
37	Yes	11	11-A	1000	McF_6	0	last	singleC	J1:DiP, J1:OT, J5:DiP, J5:OT, S1:DiP, S1:OT, S5:DiP, S5:OT
38	Yes	11	11-A	1000	McF_6	0	last	wholeT_0.01	J1:DiP, J1:OT, J5:DiP, J5:OT, S1:DiP, S5:DiP, S5:OT
39	Yes	11	11-A	1000	McF_6	0	last	wholeT_0.5	J1:DiP, J1:OT, J5:DiP, J5:OT, S1:DiP, S5:DiP, S5:OT
40	Yes	11	11-A	1000	McF_6	0	unif	singleC	J1:CBN, J1:CBN-A, J1:DiP, J1:DiP-A, J1:OT, J1:OT-A, J5:CBN, J5:CBN-A, J5:DiP, J5:DiP-A, J5:OT, J5:OT-A, S1:DiP, S1:DiP-A, S1:OT-A, S5:CBN, S5:CBN-A, S5:DiP, S5:DiP-A, S5:OT, S5:OT-A
41	Yes	11	11-A	1000	McF_6	0	unif	wholeT_0.01	J1:CBN, J1:CBN-A, J1:DiP, J1:DiP-A, J1:OT, J1:OT-A, J5:CBN-A, J5:DiP, J5:DiP-A, J5:OT, J5:OT-A, S1:DiP, S1:DiP-A, S5:CBN, S5:CBN-A, S5:DiP, S5:DiP-A, S5:OT, S5:OT-A
42	Yes	11	11-A	1000	McF_6	0	unif	wholeT_0.5	J1:CBN, J1:CBN-A, J1:DiP, J1:DiP-A, J1:OT, J5:CBN, J5:CBN-A, J5:DiP, J5:DiP-A, J5:OT, J5:OT-A, S1:DiP, S1:DiP-A, S1:OT, S1:OT-A, S5:CBN, S5:CBN-A, S5:DiP, S5:DiP-A, S5:OT, S5:OT-A
43	Yes	11	11-A	1000	McF_6	Inf	last	singleC	J1:DiP, J1:OT, J5:DiP, J5:OT, S1:DiP, S5:DiP, S5:OT

Table 16: *(continued)*

	Conjunction	Drivers	Tree	S.Size	Model	sh	S.Time	S.Type	Best method(s)
44	Yes	11	11-A	1000	McF_6	Inf	last	wholeT_0.01	J1:DiP, J1:OT, J5:DiP, J5:OT, S1:DiP, S5:DiP, S5:OT
45	Yes	11	11-A	1000	McF_6	Inf	last	wholeT_0.5	J1:DiP, J1:OT, J5:DiP, J5:OT, S1:DiP, S5:DiP, S5:OT
46	Yes	11	11-A	1000	McF_6	Inf	unif	singleC	J1:CBN-A, J1:DiP, J1:DiP-A, J1:OT, J1:OT-A, J5:CBN-A, J5:DiP, J5:OT, J5:OT-A, S1:DiP, S1:DiP-A, S5:DiP, S5:DiP-A, S5:OT
47	Yes	11	11-A	1000	McF_6	Inf	unif	wholeT_0.01	J1:CBN-A, J1:DiP, J1:DiP-A, J1:OT, J1:OT-A, J5:CBN-A, J5:DiP, J5:DiP-A, J5:OT, J5:OT-A, S1:DiP, S1:DiP-A, S5:CBN-A, S5:DiP, S5:DiP-A, S5:OT, S5:OT-A
48	Yes	11	11-A	1000	McF_6	Inf	unif	wholeT_0.5	J1:CBN-A, J1:DiP, J1:DiP-A, J5:CBN-A, J5:DiP, J5:DiP-A, J5:OT-A, S1:DiP, S1:DiP-A, S5:CBN-A, S5:DiP, S5:DiP-A, S5:OT
49	Yes	11	11-A	200	Bozic	0	last	singleC	J1:DiP, J1:DiP-A, J1:OT, J1:OT-A, J5:DiP, J5:DiP-A, J5:OT, J5:OT-A, S5:DiP, S5:DiP-A, S5:OT, S5:OT-A
50	Yes	11	11-A	200	Bozic	0	last	wholeT_0.01	J1:DiP, J1:DiP-A, J1:OT, J1:OT-A, J5:DiP, J5:DiP-A, J5:OT, J5:OT-A, S5:DiP
51	Yes	11	11-A	200	Bozic	0	last	wholeT_0.5	J1:DiP, J1:DiP-A, J1:OT, J1:OT-A, J5:DiP, J5:DiP-A, J5:OT, J5:OT-A, S1:DiP, S1:DiP-A, S5:DiP, S5:DiP-A, S5:OT, S5:OT-A
52	Yes	11	11-A	200	Bozic	0	unif	singleC	J1:DiP, J1:DiP-A, J1:OT, J1:OT-A, J5:CBN, J5:CBN-A, J5:DiP, J5:DiP-A, J5:OT, J5:OT-A, S1:DiP, S1:DiP-A, S1:OT, S1:OT-A, S5:DiP, S5:DiP-A, S5:OT, S5:OT-A
53	Yes	11	11-A	200	Bozic	0	unif	wholeT_0.01	J5:DiP, J5:DiP-A, J5:OT, J5:OT-A
54	Yes	11	11-A	200	Bozic	0	unif	wholeT_0.5	J1:DiP, J1:DiP-A, J1:OT, J1:OT-A, J5:CBN, J5:CBN-A, J5:DiP, J5:DiP-A, J5:OT, J5:OT-A, S1:DiP, S1:DiP-A, S1:OT, S1:OT-A, S5:DiP, S5:DiP-A, S5:OT, S5:OT-A

Table 16: *(continued)*

	Conjunction	Drivers	Tree	S.Size	Model	sh	S.Time	S.Type	Best method(s)
55	Yes	11	11-A	200	Bozic	Inf	last	singleC	J1:DiP, J1:DiP-A, J5:DiP, J5:DiP-A, J5:OT, J5:OT-A, S5:DiP, S5:DiP-A, S5:OT
56	Yes	11	11-A	200	Bozic	Inf	last	wholeT_0.01	J5:DiP, J5:DiP-A, J5:OT, J5:OT-A
57	Yes	11	11-A	200	Bozic	Inf	last	wholeT_0.5	J1:DiP, J1:DiP-A, J5:DiP, J5:DiP-A, J5:OT, J5:OT-A, S5:DiP, S5:DiP-A, S5:OT, S5:OT-A
58	Yes	11	11-A	200	Bozic	Inf	unif	singleC	J1:DiP, J1:DiP-A, J1:OT, J1:OT-A, J5:DiP, J5:DiP-A, J5:OT, J5:OT-A, S5:DiP, S5:DiP-A, S5:OT, S5:OT-A
59	Yes	11	11-A	200	Bozic	Inf	unif	wholeT_0.01	J5:DiP, J5:OT, J5:OT-A
60	Yes	11	11-A	200	Bozic	Inf	unif	wholeT_0.5	J1:CBN-A, J1:DiP, J1:DiP-A, J1:OT, J1:OT-A, J5:DiP, J5:DiP-A, J5:OT, J5:OT-A, S5:DiP, S5:DiP-A, S5:OT, S5:OT-A
61	Yes	11	11-A	200	exp	0	last	singleC	J1:DiP, J1:DiP-A, J1:OT, J1:OT-A, J5:CBN, J5:CBN-A, J5:DiP, J5:DiP-A, J5:OT, J5:OT-A, S1:DiP, S1:DiP-A, S1:OT, S1:OT-A, S5:DiP, S5:DiP-A, S5:OT, S5:OT-A
62	Yes	11	11-A	200	exp	0	last	wholeT_0.01	J1:DiP, J1:DiP-A, J1:OT-A, J5:DiP, J5:DiP-A, J5:OT, J5:OT-A, S5:DiP, S5:OT, S5:OT-A
63	Yes	11	11-A	200	exp	0	last	wholeT_0.5	J1:DiP, J1:DiP-A, J1:OT, J1:OT-A, J5:CBN, J5:CBN-A, J5:DiP-A, J5:OT, J5:OT-A, S1:DiP, S1:DiP-A, S1:OT, S1:OT-A, S5:DiP, S5:DiP-A, S5:OT, S5:OT-A
64	Yes	11	11-A	200	exp	0	unif	singleC	J1:CBN, J1:CBN-A, J1:DiP, J1:DiP-A, J1:OT, J1:OT-A, J5:CBN, J5:CBN-A, J5:DiP, J5:DiP-A, J5:OT, J5:OT-A, S1:DiP, S1:DiP-A, S1:OT, S1:OT-A, S5:DiP, S5:DiP-A, S5:OT, S5:OT-A
65	Yes	11	11-A	200	exp	0	unif	wholeT_0.01	J1:DiP, J1:DiP-A, J1:OT, J1:OT-A, J5:CBN, J5:CBN-A, J5:DiP, J5:DiP-A, J5:OT, J5:OT-A, S1:DiP, S1:OT, S1:OT-A, S5:DiP, S5:DiP-A, S5:OT, S5:OT-A

Table 16: *(continued)*

	Conjunction	Drivers	Tree	S.Size	Model	sh	S.Time	S.Type	Best method(s)
66	Yes	11	11-A	200	exp	0	unif	wholeT_0.5	J1:CBN, J1:DiP, J1:DiP-A, J1:OT, J1:OT-A, J5:CBN, J5:CBN-A, J5:DiP, J5:DiP-A, S1:DiP, S1:DiP-A, S1:OT, S1:OT-A, S5:DiP, S5:DiP-A, S5:OT, S5:OT-A
67	Yes	11	11-A	200	exp	Inf	last	singleC	J1:DiP, J1:DiP-A, J1:OT, J1:OT-A, J5:DiP, J5:DiP-A, J5:OT, J5:OT-A, S1:DiP, S1:DiP-A, S5:DiP, S5:DiP-A, S5:OT, S5:OT-A
68	Yes	11	11-A	200	exp	Inf	last	wholeT_0.01	J5:DiP, J5:DiP-A, J5:OT, J5:OT-A
69	Yes	11	11-A	200	exp	Inf	last	wholeT_0.5	J1:DiP, J1:DiP-A, J1:OT, J1:OT-A, J5:DiP, J5:DiP-A, J5:OT, J5:OT-A, S1:DiP, S1:DiP-A, S5:DiP, S5:DiP-A, S5:OT, S5:OT-A
70	Yes	11	11-A	200	exp	Inf	unif	singleC	J1:DiP, J1:DiP-A, J1:OT, J1:OT-A, J5:CBN, J5:CBN-A, J5:DiP, J5:DiP-A, J5:OT, J5:OT-A, S1:DiP, S1:DiP-A, S5:DiP, S5:DiP-A, S5:OT, S5:OT-A
71	Yes	11	11-A	200	exp	Inf	unif	wholeT_0.01	J5:DiP, J5:DiP-A, J5:OT, J5:OT-A, S5:OT, S5:OT-A
72	Yes	11	11-A	200	exp	Inf	unif	wholeT_0.5	J1:DiP, J1:DiP-A, J1:OT-A, J5:CBN, J5:DiP, J5:DiP-A, J5:OT, J5:OT-A, S1:DiP-A, S5:DiP, S5:DiP-A, S5:OT, S5:OT-A
73	Yes	11	11-A	200	McF_4	0	last	singleC	J1:DiP, J1:DiP-A, J1:OT, J1:OT-A, J5:DiP, J5:DiP-A, J5:OT, J5:OT-A, S5:DiP, S5:DiP-A, S5:OT, S5:OT-A
74	Yes	11	11-A	200	McF_4	0	last	wholeT_0.01	J1:DiP, J1:DiP-A, J1:OT, J1:OT-A, J5:DiP, J5:DiP-A, J5:OT, J5:OT-A, S5:DiP, S5:OT, S5:OT-A
75	Yes	11	11-A	200	McF_4	0	last	wholeT_0.5	J1:DiP, J1:DiP-A, J1:OT, J1:OT-A, J5:DiP, J5:DiP-A, J5:OT, J5:OT-A, S5:DiP, S5:DiP-A, S5:OT, S5:OT-A
76	Yes	11	11-A	200	McF_4	0	unif	singleC	J1:CBN-A, J1:DiP, J1:DiP-A, J1:OT, J1:OT-A, J5:DiP, J5:DiP-A, J5:OT, J5:OT-A, S1:DiP, S1:DiP-A, S5:DiP, S5:DiP-A, S5:OT, S5:OT-A

Table 16: *(continued)*

	Conjunction	Drivers	Tree	S.Size	Model	sh	S.Time	S.Type	Best method(s)
77	Yes	11	11-A	200	McF_4	0	unif	wholeT_0.01	J1:CBN-A, J1:DiP, J1:DiP-A, J1:OT, J1:OT-A, J5:DiP, J5:DiP-A, J5:OT, J5:OT-A, S1:DiP, S5:DiP, S5:DiP-A, S5:OT, S5:OT-A
78	Yes	11	11-A	200	McF_4	0	unif	wholeT_0.5	J1:CBN-A, J1:DiP, J1:DiP-A, J1:OT, J1:OT-A, J5:DiP, J5:DiP-A, J5:OT, J5:OT-A, S1:DiP, S1:DiP-A, S5:DiP, S5:DiP-A, S5:OT, S5:OT-A
79	Yes	11	11-A	200	McF_4	Inf	last	singleC	J1:DiP, J1:DiP-A, J1:OT, J1:OT-A, J5:DiP, J5:DiP-A, J5:OT, J5:OT-A, S5:DiP, S5:DiP-A, S5:OT, S5:OT-A
80	Yes	11	11-A	200	McF_4	Inf	last	wholeT_0.01	J1:DiP, J1:DiP-A, J1:OT, J1:OT-A, J5:DiP, J5:DiP-A, J5:OT, J5:OT-A
81	Yes	11	11-A	200	McF_4	Inf	last	wholeT_0.5	J1:DiP, J1:DiP-A, J1:OT, J1:OT-A, J5:DiP, J5:OT, J5:OT-A, S1:DiP, S1:DiP-A, S5:DiP, S5:DiP-A, S5:OT, S5:OT-A
82	Yes	11	11-A	200	McF_4	Inf	unif	singleC	J1:CBN-A, J1:DiP, J1:DiP-A, J1:OT, J1:OT-A, J5:DiP, J5:DiP-A, J5:OT, J5:OT-A, S5:CBN-A, S5:DiP-A, S5:OT, S5:OT-A
83	Yes	11	11-A	200	McF_4	Inf	unif	wholeT_0.01	J1:DiP, J1:DiP-A, J1:OT, J1:OT-A, J5:DiP, J5:DiP-A, J5:OT, J5:OT-A, S1:DiP, S1:DiP-A, S5:CBN-A, S5:DiP, S5:OT, S5:OT-A
84	Yes	11	11-A	200	McF_4	Inf	unif	wholeT_0.5	J1:CBN-A, J1:DiP, J1:DiP-A, J1:OT, J5:DiP, J5:DiP-A, J5:OT, J5:OT-A, S1:DiP, S5:DiP, S5:OT, S5:OT-A
85	Yes	11	11-A	200	McF_6	0	last	singleC	J1:DiP, J1:OT, J5:DiP, J5:OT, S1:DiP, S5:DiP
86	Yes	11	11-A	200	McF_6	0	last	wholeT_0.01	J1:DiP, J5:DiP, J5:OT, S1:DiP, S5:DiP, S5:OT
87	Yes	11	11-A	200	McF_6	0	last	wholeT_0.5	J1:DiP, J1:OT, J5:DiP, J5:OT, S1:DiP, S5:DiP, S5:OT
88	Yes	11	11-A	200	McF_6	0	unif	singleC	J1:CBN, J1:CBN-A, J1:OT, J5:CBN, J5:CBN-A, J5:DiP, J5:DiP-A, J5:OT, J5:OT-A, S5:CBN, S5:CBN-A

Table 16: *(continued)*

	Conjunction	Drivers	Tree	S.Size	Model	sh	S.Time	S.Type	Best method(s)
89	Yes	11	11-A	200	McF_6	0	unif	wholeT_0.01	J1:CBN, J1:CBN-A, J1:DiP, J1:DiP-A, J1:OT, J1:OT-A, J5:CBN, J5:CBN-A, J5:DiP, J5:DiP-A, J5:OT, J5:OT-A, S5:CBN, S5:CBN-A, S5:DiP-A, S5:OT, S5:OT-A
90	Yes	11	11-A	200	McF_6	0	unif	wholeT_0.5	J1:CBN, J1:CBN-A, J1:DiP, J5:CBN, J5:CBN-A, J5:DiP, S5:CBN, S5:CBN-A, S5:OT
91	Yes	11	11-A	200	McF_6	Inf	last	singleC	J1:DiP, J1:OT, J5:DiP, J5:OT, S1:DiP, S5:DiP, S5:OT
92	Yes	11	11-A	200	McF_6	Inf	last	wholeT_0.01	J1:DiP, J1:OT, J5:DiP, J5:OT, S1:DiP, S5:DiP, S5:OT
93	Yes	11	11-A	200	McF_6	Inf	last	wholeT_0.5	J1:DiP, J1:OT, J5:DiP, J5:OT, S1:DiP, S5:DiP, S5:OT
94	Yes	11	11-A	200	McF_6	Inf	unif	singleC	J1:CBN-A, J5:CBN, J5:CBN-A, J5:OT, J5:OT-A, S5:CBN, S5:CBN-A
95	Yes	11	11-A	200	McF_6	Inf	unif	wholeT_0.01	J1:CBN-A, J1:OT, J1:OT-A, J5:CBN-A, J5:DiP, J5:DiP-A, J5:OT, J5:OT-A, S5:CBN, S5:CBN-A, S5:OT, S5:OT-A
96	Yes	11	11-A	200	McF_6	Inf	unif	wholeT_0.5	J1:CBN-A, J1:OT, J1:OT-A, J5:CBN, J5:CBN-A, J5:DiP, J5:DiP-A, J5:OT, J5:OT-A, S5:CBN, S5:CBN-A, S5:OT, S5:OT-A
97	Yes	11	11-A	100	Bozic	0	last	singleC	J1:DiP, J1:DiP-A, J5:DiP, J5:DiP-A, J5:OT, J5:OT-A, S1:DiP, S1:DiP-A, S5:DiP, S5:DiP-A, S5:OT, S5:OT-A
98	Yes	11	11-A	100	Bozic	0	last	wholeT_0.01	J1:DiP, J1:DiP-A, J5:DiP, J5:DiP-A, J5:OT, J5:OT-A
99	Yes	11	11-A	100	Bozic	0	last	wholeT_0.5	J1:DiP, J1:DiP-A, J5:DiP, J5:DiP-A, J5:OT, J5:OT-A, S1:DiP, S1:DiP-A, S5:DiP, S5:DiP-A, S5:OT, S5:OT-A
100	Yes	11	11-A	100	Bozic	0	unif	singleC	J1:DiP, J1:DiP-A, J1:OT, J1:OT-A, J5:CBN, J5:CBN-A, J5:DiP, J5:DiP-A, J5:OT, J5:OT-A, S1:DiP, S1:DiP-A, S5:DiP, S5:DiP-A, S5:OT, S5:OT-A
101	Yes	11	11-A	100	Bozic	0	unif	wholeT_0.01	J5:DiP, J5:DiP-A, J5:OT, J5:OT-A, S5:OT

Table 16: *(continued)*

	Conjunction	Drivers	Tree	S.Size	Model	sh	S.Time	S.Type	Best method(s)
102	Yes	11	11-A	100	Bozic	0	unif	wholeT_0.5	J1:DiP, J1:DiP-A, J5:CBN, J5:CBN-A, J5:DiP, J5:DiP-A, J5:OT, S1:DiP, S1:DiP-A, S5:DiP, S5:DiP-A, S5:OT, S5:OT-A
103	Yes	11	11-A	100	Bozic	Inf	last	singleC	J1:DiP, J1:DiP-A, J5:DiP, J5:DiP-A, J5:OT, J5:OT-A, S1:DiP, S5:DiP, S5:DiP-A
104	Yes	11	11-A	100	Bozic	Inf	last	wholeT_0.01	J5:DiP, J5:DiP-A, J5:OT, J5:OT-A
105	Yes	11	11-A	100	Bozic	Inf	last	wholeT_0.5	J1:DiP, J1:DiP-A, J5:DiP, J5:DiP-A, J5:OT, J5:OT-A, S1:DiP, S5:DiP, S5:DiP-A
106	Yes	11	11-A	100	Bozic	Inf	unif	singleC	J1:DiP, J1:DiP-A, J5:DiP, J5:DiP-A, J5:OT, J5:OT-A, S5:DiP, S5:DiP-A, S5:OT, S5:OT-A
107	Yes	11	11-A	100	Bozic	Inf	unif	wholeT_0.01	J5:DiP, J5:DiP-A, J5:OT, J5:OT-A
108	Yes	11	11-A	100	Bozic	Inf	unif	wholeT_0.5	J1:DiP, J1:DiP-A, J5:CBN-A, J5:DiP, J5:DiP-A, J5:OT, J5:OT-A, S5:DiP, S5:DiP-A, S5:OT, S5:OT-A
109	Yes	11	11-A	100	exp	0	last	singleC	J1:DiP, J1:DiP-A, J1:OT, J1:OT-A, J5:CBN, J5:CBN-A, J5:DiP, J5:DiP-A, J5:OT, J5:OT-A, S1:DiP, S1:DiP-A, S5:DiP, S5:DiP-A, S5:OT, S5:OT-A
110	Yes	11	11-A	100	exp	0	last	wholeT_0.01	J1:DiP, J1:DiP-A, J1:OT, J1:OT-A, J5:DiP, J5:DiP-A, J5:OT, J5:OT-A, S1:DiP, S1:DiP-A, S5:DiP, S5:DiP-A, S5:OT, S5:OT-A
111	Yes	11	11-A	100	exp	0	last	wholeT_0.5	J1:DiP, J1:DiP-A, J1:OT, J1:OT-A, J5:CBN, J5:CBN-A, J5:DiP, J5:DiP-A, J5:OT, J5:OT-A, S1:DiP, S1:DiP-A, S1:OT-A, S5:DiP, S5:DiP-A, S5:OT, S5:OT-A
112	Yes	11	11-A	100	exp	0	unif	singleC	J1:CBN, J1:DiP, J1:DiP-A, J1:OT, J1:OT-A, J5:CBN, J5:CBN-A, J5:DiP, J5:DiP-A, J5:OT, J5:OT-A, S1:DiP, S1:DiP-A, S1:OT, S5:DiP, S5:DiP-A, S5:OT, S5:OT-A
113	Yes	11	11-A	100	exp	0	unif	wholeT_0.01	J1:DiP, J1:DiP-A, J5:CBN, J5:CBN-A, J5:DiP, J5:DiP-A, J5:OT, J5:OT-A, S1:DiP, S1:DiP-A, S5:DiP, S5:DiP-A, S5:OT, S5:OT-A

Table 16: *(continued)*

	Conjunction	Drivers	Tree	S.Size	Model	sh	S.Time	S.Type	Best method(s)
114	Yes	11	11-A	100	exp	0	unif	wholeT_0.5	J1:DiP, J1:DiP-A, J5:CBN, J5:CBN-A, J5:DiP, J5:DiP-A, J5:OT, J5:OT-A, S1:DiP, S1:DiP-A, S5:DiP, S5:DiP-A, S5:OT, S5:OT-A
115	Yes	11	11-A	100	exp	Inf	last	singleC	J1:DiP, J1:DiP-A, J5:DiP, J5:DiP-A, J5:OT, J5:OT-A, S1:DiP, S1:DiP-A, S5:DiP, S5:DiP-A, S5:OT, S5:OT-A
116	Yes	11	11-A	100	exp	Inf	last	wholeT_0.01	J5:DiP, J5:DiP-A, J5:OT, J5:OT-A
117	Yes	11	11-A	100	exp	Inf	last	wholeT_0.5	J1:DiP, J1:DiP-A, J5:DiP, J5:DiP-A, J5:OT, J5:OT-A, S1:DiP, S1:DiP-A, S5:DiP, S5:DiP-A, S5:OT, S5:OT-A
118	Yes	11	11-A	100	exp	Inf	unif	singleC	J1:DiP, J1:DiP-A, J5:CBN, J5:CBN-A, J5:DiP, J5:DiP-A, J5:OT, J5:OT-A, S1:DiP, S5:DiP, S5:DiP-A, S5:OT, S5:OT-A
119	Yes	11	11-A	100	exp	Inf	unif	wholeT_0.01	J5:DiP, J5:DiP-A, J5:OT, J5:OT-A, S5:OT-A
120	Yes	11	11-A	100	exp	Inf	unif	wholeT_0.5	J1:DiP, J1:DiP-A, J5:CBN, J5:CBN-A, J5:DiP, J5:DiP-A, J5:OT, J5:OT-A, S1:DiP, S1:DiP-A, S5:DiP, S5:DiP-A, S5:OT, S5:OT-A
121	Yes	11	11-A	100	McF_4	0	last	singleC	J1:DiP, J1:DiP-A, J5:DiP, J5:DiP-A, J5:OT, J5:OT-A, S1:DiP, S1:DiP-A, S5:DiP, S5:DiP-A, S5:OT, S5:OT-A
122	Yes	11	11-A	100	McF_4	0	last	wholeT_0.01	J1:DiP, J1:DiP-A, J5:DiP, J5:DiP-A, J5:OT, J5:OT-A, S1:DiP, S1:DiP-A, S5:DiP, S5:DiP-A, S5:OT, S5:OT-A
123	Yes	11	11-A	100	McF_4	0	last	wholeT_0.5	J1:DiP, J1:DiP-A, J5:DiP, J5:DiP-A, J5:OT, J5:OT-A, S1:DiP, S1:DiP-A, S5:DiP, S5:DiP-A, S5:OT, S5:OT-A
124	Yes	11	11-A	100	McF_4	0	unif	singleC	J1:CBN, J1:DiP, J1:DiP-A, J1:OT, J1:OT-A, J5:DiP, J5:DiP-A, J5:OT, J5:OT-A, S1:DiP, S1:DiP-A, S5:CBN-A, S5:DiP, S5:DiP-A, S5:OT, S5:OT-A
125	Yes	11	11-A	100	McF_4	0	unif	wholeT_0.01	J1:DiP, J1:DiP-A, J5:DiP, J5:DiP-A, J5:OT, J5:OT-A, S5:DiP, S5:DiP-A
126	Yes	11	11-A	100	McF_4	0	unif	wholeT_0.5	J1:CBN-A, J1:DiP, J1:DiP-A, J1:OT-A, J5:DiP, J5:DiP-A, J5:OT, J5:OT-A, S1:DiP, S5:CBN-A, S5:DiP, S5:OT, S5:OT-A

Table 16: *(continued)*

	Conjunction	Drivers	Tree	S.Size	Model	sh	S.Time	S.Type	Best method(s)
127	Yes	11	11-A	100	McF_4	Inf	last	singleC	J1:DiP, J5:DiP, J5:DiP-A, J5:OT, J5:OT-A, S1:DiP, S1:DiP-A, S5:DiP, S5:DiP-A, S5:OT, S5:OT-A
128	Yes	11	11-A	100	McF_4	Inf	last	wholeT_0.01	J1:DiP, J1:DiP-A, J5:DiP, J5:DiP-A, J5:OT, J5:OT-A, S1:DiP, S1:DiP-A, S5:DiP, S5:DiP-A
129	Yes	11	11-A	100	McF_4	Inf	last	wholeT_0.5	J1:DiP, J1:DiP-A, J5:DiP, J5:DiP-A, J5:OT, J5:OT-A, S1:DiP, S1:DiP-A, S5:DiP, S5:DiP-A, S5:OT, S5:OT-A
130	Yes	11	11-A	100	McF_4	Inf	unif	singleC	J1:DiP, J1:DiP-A, J1:OT, J5:DiP, J5:DiP-A, J5:OT, S1:DiP, S1:DiP-A, S5:CBN-A, S5:DiP, S5:DiP-A, S5:OT-A
131	Yes	11	11-A	100	McF_4	Inf	unif	wholeT_0.01	J1:DiP-A, J5:DiP, J5:DiP-A, J5:OT, J5:OT-A, S5:DiP
132	Yes	11	11-A	100	McF_4	Inf	unif	wholeT_0.5	J1:DiP, J1:DiP-A, J5:CBN-A, J5:DiP, J5:DiP-A, J5:OT, J5:OT-A, S5:CBN-A
133	Yes	11	11-A	100	McF_6	0	last	singleC	J1:DiP, J1:OT, J5:DiP, J5:OT, S1:DiP, S5:DiP
134	Yes	11	11-A	100	McF_6	0	last	wholeT_0.01	J1:DiP, J5:DiP, J5:OT, S1:DiP, S5:DiP, S5:OT
135	Yes	11	11-A	100	McF_6	0	last	wholeT_0.5	J1:DiP, J1:OT, J5:DiP, J5:OT, S1:DiP, S5:DiP, S5:OT
136	Yes	11	11-A	100	McF_6	0	unif	singleC	J1:CBN-A, J5:CBN, J5:CBN-A, J5:DiP, J5:OT, J5:OT-A
137	Yes	11	11-A	100	McF_6	0	unif	wholeT_0.01	J1:DiP, J5:CBN, J5:CBN-A, J5:DiP, J5:OT, J5:OT-A
138	Yes	11	11-A	100	McF_6	0	unif	wholeT_0.5	J5:CBN-A, J5:DiP, J5:DiP-A, J5:OT, S5:CBN, S5:CBN-A
139	Yes	11	11-A	100	McF_6	Inf	last	singleC	J1:DiP, J1:OT, J5:DiP, J5:OT, S1:DiP, S5:DiP, S5:OT
140	Yes	11	11-A	100	McF_6	Inf	last	wholeT_0.01	J1:DiP, J5:DiP, J5:OT, S1:DiP, S5:DiP
141	Yes	11	11-A	100	McF_6	Inf	last	wholeT_0.5	J1:DiP, J5:DiP, J5:OT
142	Yes	11	11-A	100	McF_6	Inf	unif	singleC	J1:CBN, J1:CBN-A, J5:CBN-A, J5:DiP, J5:DiP-A, J5:OT, J5:OT-A
143	Yes	11	11-A	100	McF_6	Inf	unif	wholeT_0.01	J1:DiP, J5:CBN-A, J5:DiP, J5:DiP-A, J5:OT, J5:OT-A, S5:CBN, S5:CBN-A
144	Yes	11	11-A	100	McF_6	Inf	unif	wholeT_0.5	J1:CBN-A, J1:DiP, J5:CBN-A, J5:DiP, J5:DiP-A, J5:OT, S5:CBN, S5:CBN-A, S5:OT

Table 16: *(continued)*

	Conjunction	Drivers	Tree	S.Size	Model	sh	S.Time	S.Type	Best method(s)
145	Yes	9	9-A	1000	Bozic	0	last	singleC	J1:DiP, J1:DiP-A, J1:OT, J1:OT-A, J5:DiP, J5:OT, J5:OT-A, S1:OT, S1:OT-A, S5:DiP, S5:DiP-A, S5:OT, S5:OT-A
146	Yes	9	9-A	1000	Bozic	0	last	wholeT_0.01	J1:DiP, J1:DiP-A, J1:OT, J1:OT-A, J5:DiP, J5:DiP-A, J5:OT, J5:OT-A, S1:DiP, S1:DiP-A, S1:OT, S1:OT-A, S5:DiP, S5:DiP-A, S5:OT, S5:OT-A
147	Yes	9	9-A	1000	Bozic	0	last	wholeT_0.5	J1:DiP, J1:DiP-A, J1:OT, J1:OT-A, J5:CBN, J5:CBN-A, J5:DiP, J5:DiP-A, J5:OT, J5:OT-A, S1:OT, S1:OT-A, S5:DiP, S5:DiP-A, S5:OT, S5:OT-A
148	Yes	9	9-A	1000	Bozic	0	unif	singleC	J1:DiP, J1:DiP-A, J1:OT, J1:OT-A, J5:DiP, J5:DiP-A, J5:OT, S1:DiP, S1:DiP-A, S1:OT, S1:OT-A, S5:DiP-A, S5:OT-A
149	Yes	9	9-A	1000	Bozic	0	unif	wholeT_0.01	J1:DiP, J1:DiP-A, J1:OT, J1:OT-A, J5:DiP, J5:DiP-A, J5:OT, J5:OT-A, S1:DiP, S1:DiP-A, S1:OT, S1:OT-A, S5:DiP, S5:DiP-A, S5:OT, S5:OT-A
150	Yes	9	9-A	1000	Bozic	0	unif	wholeT_0.5	J1:CBN, J1:CBN-A, J1:DiP, J1:DiP-A, J1:OT, J1:OT-A, J5:CBN, J5:CBN-A, J5:DiP, J5:DiP-A, J5:OT-A, S1:DiP, S1:DiP-A, S1:OT, S1:OT-A, S5:DiP, S5:DiP-A, S5:OT, S5:OT-A
151	Yes	9	9-A	1000	Bozic	Inf	last	singleC	J1:DiP, J1:DiP-A, J1:OT-A, J5:DiP, J5:DiP-A, J5:OT, J5:OT-A, S5:OT, S5:OT-A
152	Yes	9	9-A	1000	Bozic	Inf	last	wholeT_0.01	J5:DiP, J5:DiP-A, J5:OT, J5:OT-A
153	Yes	9	9-A	1000	Bozic	Inf	last	wholeT_0.5	J1:DiP, J1:DiP-A, J1:OT, J1:OT-A, J5:DiP, J5:DiP-A, J5:OT, J5:OT-A, S5:DiP-A, S5:OT
154	Yes	9	9-A	1000	Bozic	Inf	unif	singleC	J1:DiP, J1:DiP-A, J1:OT, J1:OT-A, J5:DiP, J5:DiP-A, J5:OT, S1:DiP, S1:OT, S5:DiP, S5:DiP-A, S5:OT, S5:OT-A
155	Yes	9	9-A	1000	Bozic	Inf	unif	wholeT_0.01	J5:DiP, J5:DiP-A, J5:OT, J5:OT-A, S5:DiP, S5:DiP-A, S5:OT, S5:OT-A

Table 16: *(continued)*

	Conjunction	Drivers	Tree	S.Size	Model	sh	S.Time	S.Type	Best method(s)
156	Yes	9	9-A	1000	Bozic	Inf	unif	wholeT_0.5	J1:DiP, J1:DiP-A, J1:OT, J1:OT-A, J5:CBN, J5:CBN-A, J5:DiP, J5:DiP-A, J5:OT, J5:OT-A, S1:DiP-A, S1:OT, S1:OT-A, S5:DiP, S5:DiP-A, S5:OT, S5:OT-A
157	Yes	9	9-A	1000	exp	0	last	singleC	J1:DiP, J1:DiP-A, J1:OT, J1:OT-A, J5:CBN, J5:CBN-A, J5:DiP, J5:DiP-A, J5:OT, J5:OT-A, S1:DiP, S1:DiP-A, S1:OT, S1:OT-A, S5:DiP, S5:DiP-A, S5:OT, S5:OT-A
158	Yes	9	9-A	1000	exp	0	last	wholeT_0.01	J1:DiP, J1:DiP-A, J1:OT, J1:OT-A, J5:DiP, J5:DiP-A, J5:OT, J5:OT-A, S1:DiP-A, S1:OT, S1:OT-A, S5:DiP, S5:DiP-A, S5:OT, S5:OT-A
159	Yes	9	9-A	1000	exp	0	last	wholeT_0.5	J1:DiP, J1:DiP-A, J1:OT, J1:OT-A, J5:CBN, J5:CBN-A, J5:DiP, J5:DiP-A, J5:OT-A, S1:DiP, S1:DiP-A, S1:OT, S1:OT-A, S5:DiP, S5:DiP-A, S5:OT, S5:OT-A
160	Yes	9	9-A	1000	exp	0	unif	singleC	J1:CBN, J1:CBN-A, J1:DiP, J1:DiP-A, J1:OT, J1:OT-A, J5:CBN, J5:CBN-A, J5:DiP, J5:DiP-A, J5:OT, J5:OT-A, S1:DiP, S1:DiP-A, S1:OT, S1:OT-A, S5:DiP, S5:DiP-A, S5:OT, S5:OT-A
161	Yes	9	9-A	1000	exp	0	unif	wholeT_0.01	J1:DiP, J1:DiP-A, J1:OT, J1:OT-A, J5:CBN, J5:CBN-A, J5:DiP, J5:DiP-A, J5:OT, J5:OT-A, S1:DiP, S1:DiP-A, S1:OT, S1:OT-A, S5:DiP, S5:DiP-A, S5:OT, S5:OT-A
162	Yes	9	9-A	1000	exp	0	unif	wholeT_0.5	J1:CBN, J1:CBN-A, J1:DiP, J1:DiP-A, J1:OT, J1:OT-A, J5:CBN, J5:CBN-A, J5:DiP, J5:DiP-A, J5:OT, J5:OT-A, S1:DiP, S1:DiP-A, S1:OT, S1:OT-A, S5:DiP, S5:DiP-A, S5:OT, S5:OT-A
163	Yes	9	9-A	1000	exp	Inf	last	singleC	J1:DiP, J1:DiP-A, J1:OT, J1:OT-A, J5:DiP, J5:DiP-A, J5:OT, J5:OT-A, S5:DiP, S5:DiP-A, S5:OT, S5:OT-A
164	Yes	9	9-A	1000	exp	Inf	last	wholeT_0.01	J5:DiP, J5:DiP-A, J5:OT, J5:OT-A

Table 16: *(continued)*

	Conjunction	Drivers	Tree	S.Size	Model	sh	S.Time	S.Type	Best method(s)
165	Yes	9	9-A	1000	exp	Inf	last	wholeT_0.5	J1:DiP, J1:DiP-A, J1:OT, J1:OT-A, J5:DiP, J5:DiP-A, J5:OT, J5:OT-A, S5:DiP, S5:DiP-A, S5:OT, S5:OT-A
166	Yes	9	9-A	1000	exp	Inf	unif	singleC	J1:DiP, J1:DiP-A, J1:OT, J1:OT-A, J5:CBN, J5:CBN-A, J5:DiP, J5:DiP-A, J5:OT, J5:OT-A, S1:DiP, S1:DiP-A, S1:OT, S1:OT-A, S5:DiP, S5:DiP-A, S5:OT, S5:OT-A
167	Yes	9	9-A	1000	exp	Inf	unif	wholeT_0.01	J1:DiP, J1:DiP-A, J1:OT, J1:OT-A, J5:DiP, J5:DiP-A, J5:OT, J5:OT-A, S5:DiP, S5:DiP-A, S5:OT, S5:OT-A
168	Yes	9	9-A	1000	exp	Inf	unif	wholeT_0.5	J1:DiP, J1:DiP-A, J1:OT, J1:OT-A, J5:CBN, J5:CBN-A, J5:DiP, J5:DiP-A, J5:OT, J5:OT-A, S1:DiP, S1:DiP-A, S1:OT, S5:DiP, S5:DiP-A, S5:OT, S5:OT-A
169	Yes	9	9-A	1000	McF_4	0	last	singleC	J1:DiP, J1:DiP-A, J1:OT, J1:OT-A, J5:DiP, J5:DiP-A, J5:OT, J5:OT-A, S1:DiP, S1:DiP-A, S1:OT, S1:OT-A, S5:DiP, S5:DiP-A, S5:OT, S5:OT-A
170	Yes	9	9-A	1000	McF_4	0	last	wholeT_0.01	J1:DiP, J1:DiP-A, J1:OT, J1:OT-A, J5:CBN, J5:DiP, J5:DiP-A, J5:OT, J5:OT-A, S5:DiP, S5:DiP-A, S5:OT, S5:OT-A
171	Yes	9	9-A	1000	McF_4	0	last	wholeT_0.5	J1:DiP, J1:DiP-A, J1:OT, J1:OT-A, J5:DiP, J5:DiP-A, J5:OT, J5:OT-A, S1:DiP, S1:DiP-A, S1:OT, S1:OT-A, S5:DiP-A, S5:OT, S5:OT-A
172	Yes	9	9-A	1000	McF_4	0	unif	singleC	J1:DiP, J1:DiP-A, J1:OT, J1:OT-A, J5:DiP, J5:DiP-A, J5:OT, J5:OT-A, S1:DiP, S1:DiP-A, S1:OT, S1:OT-A, S5:DiP, S5:DiP-A, S5:OT, S5:OT-A
173	Yes	9	9-A	1000	McF_4	0	unif	wholeT_0.01	J1:CBN-A, J1:DiP, J1:DiP-A, J1:OT, J1:OT-A, J5:DiP, J5:DiP-A, J5:OT, J5:OT-A, S1:DiP-A, S5:DiP, S5:DiP-A, S5:OT, S5:OT-A
174	Yes	9	9-A	1000	McF_4	0	unif	wholeT_0.5	J1:DiP, J1:DiP-A, J1:OT, J1:OT-A, J5:DiP, J5:OT, J5:OT-A, S1:DiP, S1:DiP-A, S1:OT, S1:OT-A, S5:DiP, S5:DiP-A, S5:OT, S5:OT-A

Table 16: *(continued)*

	Conjunction	Drivers	Tree	S.Size	Model	sh	S.Time	S.Type	Best method(s)
175	Yes	9	9-A	1000	McF_4	Inf	last	singleC	J1:DiP, J1:DiP-A, J1:OT, J1:OT-A, J5:DiP, J5:DiP-A, J5:OT, J5:OT-A, S1:OT-A, S5:DiP, S5:DiP-A, S5:OT, S5:OT-A
176	Yes	9	9-A	1000	McF_4	Inf	last	wholeT_0.01	J1:DiP, J1:OT, J5:DiP, J5:OT, S1:DiP, S5:DiP, S5:OT
177	Yes	9	9-A	1000	McF_4	Inf	last	wholeT_0.5	J1:DiP, J1:DiP-A, J1:OT, J1:OT-A, J5:DiP, J5:DiP-A, J5:OT, J5:OT-A, S1:DiP, S1:DiP-A, S1:OT, S1:OT-A, S5:DiP, S5:OT, S5:OT-A
178	Yes	9	9-A	1000	McF_4	Inf	unif	singleC	J1:DiP, J1:DiP-A, J1:OT, J1:OT-A, J5:DiP, J5:DiP-A, J5:OT, J5:OT-A, S1:DiP, S1:OT, S1:OT-A, S5:DiP, S5:DiP-A, S5:OT, S5:OT-A
179	Yes	9	9-A	1000	McF_4	Inf	unif	wholeT_0.01	J1:DiP, J1:DiP-A, J1:OT, J1:OT-A, J5:DiP, J5:DiP-A, J5:OT, J5:OT-A, S5:CBN-A, S5:DiP, S5:DiP-A, S5:OT, S5:OT-A
180	Yes	9	9-A	1000	McF_4	Inf	unif	wholeT_0.5	J1:DiP, J1:DiP-A, J1:OT, J1:OT-A, J5:DiP, J5:DiP-A, J5:OT, J5:OT-A, S1:DiP, S1:DiP-A, S1:OT, S1:OT-A, S5:DiP, S5:DiP-A, S5:OT, S5:OT-A
181	Yes	9	9-A	1000	McF_6	0	last	singleC	J1:DiP, J1:OT, J5:DiP, J5:OT, S1:DiP, S5:DiP, S5:OT
182	Yes	9	9-A	1000	McF_6	0	last	wholeT_0.01	J1:DiP, J1:OT, J5:DiP, J5:OT, S1:DiP, S5:DiP, S5:OT
183	Yes	9	9-A	1000	McF_6	0	last	wholeT_0.5	J1:DiP, J1:OT, J5:DiP, J5:OT, S1:DiP, S1:OT, S5:DiP, S5:OT
184	Yes	9	9-A	1000	McF_6	0	unif	singleC	J1:CBN, J1:CBN-A, J1:DiP, J1:DiP-A, J1:OT, J1:OT-A, J5:CBN, J5:CBN-A, J5:DiP, J5:DiP-A, J5:OT, J5:OT-A, S1:CBN-A, S1:DiP, S1:DiP-A, S1:OT, S1:OT-A, S5:CBN, S5:CBN-A, S5:DiP, S5:DiP-A, S5:OT, S5:OT-A
185	Yes	9	9-A	1000	McF_6	0	unif	wholeT_0.01	J1:CBN, J1:CBN-A, J1:DiP, J1:DiP-A, J1:OT, J1:OT-A, J5:CBN, J5:CBN-A, J5:DiP, J5:DiP-A, J5:OT, J5:OT-A, S1:DiP, S5:CBN, S5:CBN-A, S5:DiP, S5:DiP-A, S5:OT, S5:OT-A

Table 16: *(continued)*

	Conjunction	Drivers	Tree	S.Size	Model	sh	S.Time	S.Type	Best method(s)
186	Yes	9	9-A	1000	McF_6	0	unif	wholeT_0.5	J1:CBN, J1:CBN-A, J1:DiP, J1:DiP-A, J1:OT, J1:OT-A, J5:CBN, J5:CBN-A, J5:DiP, J5:DiP-A, J5:OT, J5:OT-A, S1:CBN-A, S1:DiP, S1:DiP-A, S1:OT, S1:OT-A, S5:CBN, S5:CBN-A, S5:DiP, S5:OT, S5:OT-A
187	Yes	9	9-A	1000	McF_6	Inf	last	singleC	J1:DiP, J1:OT, J5:DiP, J5:OT, S1:DiP, S5:DiP, S5:OT
188	Yes	9	9-A	1000	McF_6	Inf	last	wholeT_0.01	J1:DiP, J1:OT, J5:DiP, J5:OT, S5:OT
189	Yes	9	9-A	1000	McF_6	Inf	last	wholeT_0.5	J1:DiP, J1:OT, J5:DiP, J5:OT, S1:DiP, S5:DiP, S5:OT
190	Yes	9	9-A	1000	McF_6	Inf	unif	singleC	J1:CBN, J1:CBN-A, J1:DiP, J1:DiP-A, J1:OT, J1:OT-A, J5:CBN, J5:CBN-A, J5:DiP, J5:DiP-A, J5:OT, J5:OT-A, S1:DiP, S1:OT, S1:OT-A, S5:CBN, S5:CBN-A, S5:DiP, S5:DiP-A, S5:OT, S5:OT-A
191	Yes	9	9-A	1000	McF_6	Inf	unif	wholeT_0.01	J1:CBN, J1:CBN-A, J1:DiP, J1:DiP-A, J1:OT, J1:OT-A, J5:CBN-A, J5:DiP, J5:DiP-A, J5:OT, J5:OT-A, S1:DiP, S1:DiP-A, S5:CBN, S5:CBN-A, S5:DiP, S5:DiP-A, S5:OT, S5:OT-A
192	Yes	9	9-A	1000	McF_6	Inf	unif	wholeT_0.5	J1:CBN, J1:CBN-A, J1:DiP, J1:DiP-A, J1:OT, J1:OT-A, J5:CBN, J5:CBN-A, J5:DiP, J5:DiP-A, J5:OT, J5:OT-A, S1:DiP, S1:DiP-A, S1:OT, S1:OT-A, S5:CBN, S5:CBN-A, S5:DiP, S5:DiP-A, S5:OT, S5:OT-A
193	Yes	9	9-A	200	Bozic	0	last	singleC	J1:DiP, J1:DiP-A, J1:OT, J1:OT-A, J5:DiP-A, J5:OT, J5:OT-A, S1:DiP, S1:OT-A, S5:DiP, S5:DiP-A, S5:OT, S5:OT-A
194	Yes	9	9-A	200	Bozic	0	last	wholeT_0.01	J1:DiP, J1:DiP-A, J1:OT, J1:OT-A, J5:DiP, J5:DiP-A, J5:OT, J5:OT-A, S1:DiP, S1:DiP-A, S5:DiP, S5:DiP-A, S5:OT, S5:OT-A
195	Yes	9	9-A	200	Bozic	0	last	wholeT_0.5	J1:DiP, J1:DiP-A, J1:OT, J1:OT-A, J5:DiP, J5:DiP-A, J5:OT, J5:OT-A, S1:DiP, S1:DiP-A, S5:DiP, S5:DiP-A, S5:OT, S5:OT-A

Table 16: *(continued)*

	Conjunction	Drivers	Tree	S.Size	Model	sh	S.Time	S.Type	Best method(s)
196	Yes	9	9-A	200	Bozic	0	unif	singleC	J1:DiP, J1:DiP-A, J1:OT, J1:OT-A, J5:CBN, J5:CBN-A, J5:DiP, J5:DiP-A, J5:OT, J5:OT-A, S1:DiP, S1:DiP-A, S1:OT, S1:OT-A, S5:DiP, S5:DiP-A, S5:OT, S5:OT-A
197	Yes	9	9-A	200	Bozic	0	unif	wholeT_0.01	J1:DiP, J1:DiP-A, J1:OT, J1:OT-A, J5:DiP-A, J5:OT, J5:OT-A, S5:DiP, S5:DiP-A, S5:OT, S5:OT-A
198	Yes	9	9-A	200	Bozic	0	unif	wholeT_0.5	J1:DiP, J1:DiP-A, J1:OT, J1:OT-A, J5:CBN, J5:CBN-A, J5:DiP, J5:DiP-A, J5:OT, J5:OT-A, S1:DiP-A, S1:OT, S1:OT-A, S5:DiP, S5:DiP-A, S5:OT, S5:OT-A
199	Yes	9	9-A	200	Bozic	Inf	last	singleC	J1:DiP, J1:DiP-A, J5:DiP, J5:DiP-A, J5:OT, J5:OT-A, S5:DiP-A, S5:OT-A
200	Yes	9	9-A	200	Bozic	Inf	last	wholeT_0.01	J5:DiP, J5:DiP-A, J5:OT, J5:OT-A
201	Yes	9	9-A	200	Bozic	Inf	last	wholeT_0.5	J1:DiP, J1:DiP-A, J5:DiP, J5:DiP-A, J5:OT, J5:OT-A, S5:DiP
202	Yes	9	9-A	200	Bozic	Inf	unif	singleC	J1:DiP, J1:DiP-A, J1:OT, J1:OT-A, J5:DiP, J5:DiP-A, J5:OT, J5:OT-A, S5:DiP, S5:DiP-A, S5:OT, S5:OT-A
203	Yes	9	9-A	200	Bozic	Inf	unif	wholeT_0.01	J5:DiP, J5:DiP-A, J5:OT, J5:OT-A, S5:OT, S5:OT-A
204	Yes	9	9-A	200	Bozic	Inf	unif	wholeT_0.5	J1:DiP, J1:DiP-A, J1:OT, J1:OT-A, J5:DiP, J5:DiP-A, J5:OT, J5:OT-A, S5:DiP, S5:DiP-A, S5:OT, S5:OT-A
205	Yes	9	9-A	200	exp	0	last	singleC	J1:DiP, J1:DiP-A, J1:OT, J1:OT-A, J5:CBN, J5:CBN-A, J5:DiP, J5:DiP-A, J5:OT, J5:OT-A, S1:DiP, S1:DiP-A, S1:OT, S1:OT-A, S5:DiP, S5:DiP-A, S5:OT, S5:OT-A
206	Yes	9	9-A	200	exp	0	last	wholeT_0.01	J1:DiP, J1:DiP-A, J1:OT, J1:OT-A, J5:DiP, J5:DiP-A, J5:OT, J5:OT-A, S1:DiP, S1:DiP-A, S1:OT, S5:DiP, S5:DiP-A, S5:OT, S5:OT-A
207	Yes	9	9-A	200	exp	0	last	wholeT_0.5	J1:DiP, J1:DiP-A, J1:OT, J1:OT-A, J5:CBN, J5:CBN-A, J5:DiP, J5:DiP-A, J5:OT, J5:OT-A, S1:DiP, S1:DiP-A, S1:OT, S1:OT-A, S5:DiP, S5:DiP-A, S5:OT, S5:OT-A

Table 16: *(continued)*

	Conjunction	Drivers	Tree	S.Size	Model	sh	S.Time	S.Type	Best method(s)
208	Yes	9	9-A	200	exp	0	unif	singleC	J1:CBN, J1:DiP, J1:DiP-A, J1:OT, J1:OT-A, J5:CBN, J5:CBN-A, J5:DiP, J5:DiP-A, J5:OT, J5:OT-A, S1:DiP, S1:DiP-A, S1:OT, S1:OT-A, S5:DiP, S5:DiP-A, S5:OT, S5:OT-A
209	Yes	9	9-A	200	exp	0	unif	wholeT_0.01	J1:DiP, J1:DiP-A, J1:OT, J1:OT-A, J5:CBN, J5:CBN-A, J5:DiP, J5:DiP-A, J5:OT, J5:OT-A, S1:DiP, S1:DiP-A, S1:OT-A, S5:DiP, S5:DiP-A, S5:OT, S5:OT-A
210	Yes	9	9-A	200	exp	0	unif	wholeT_0.5	J1:CBN, J1:CBN-A, J1:DiP, J1:DiP-A, J1:OT, J1:OT-A, J5:CBN, J5:CBN-A, J5:DiP, J5:DiP-A, J5:OT, J5:OT-A, S1:DiP, S1:DiP-A, S1:OT, S1:OT-A, S5:DiP, S5:DiP-A, S5:OT, S5:OT-A
211	Yes	9	9-A	200	exp	Inf	last	singleC	J1:DiP, J1:DiP-A, J1:OT, J5:DiP, J5:OT, J5:OT-A, S1:DiP, S5:DiP, S5:DiP-A, S5:OT, S5:OT-A
212	Yes	9	9-A	200	exp	Inf	last	wholeT_0.01	J5:DiP, J5:DiP-A, J5:OT, J5:OT-A
213	Yes	9	9-A	200	exp	Inf	last	wholeT_0.5	J1:DiP, J1:DiP-A, J1:OT, J1:OT-A, J5:DiP, J5:DiP-A, J5:OT, J5:OT-A, S1:DiP, S1:DiP-A, S5:DiP, S5:DiP-A, S5:OT, S5:OT-A
214	Yes	9	9-A	200	exp	Inf	unif	singleC	J1:DiP, J1:DiP-A, J1:OT, J1:OT-A, J5:CBN-A, J5:DiP, J5:DiP-A, J5:OT, J5:OT-A, S1:DiP-A, S5:DiP, S5:DiP-A, S5:OT, S5:OT-A
215	Yes	9	9-A	200	exp	Inf	unif	wholeT_0.01	J5:DiP, J5:DiP-A, J5:OT, J5:OT-A, S5:DiP, S5:DiP-A, S5:OT, S5:OT-A
216	Yes	9	9-A	200	exp	Inf	unif	wholeT_0.5	J1:DiP, J1:DiP-A, J1:OT, J1:OT-A, J5:CBN, J5:CBN-A, J5:DiP, J5:DiP-A, J5:OT, J5:OT-A, S1:DiP, S1:DiP-A, S5:DiP, S5:DiP-A, S5:OT, S5:OT-A
217	Yes	9	9-A	200	McF_4	0	last	singleC	J1:DiP, J1:DiP-A, J1:OT, J1:OT-A, J5:DiP, J5:DiP-A, J5:OT, J5:OT-A, S5:DiP, S5:DiP-A, S5:OT, S5:OT-A
218	Yes	9	9-A	200	McF_4	0	last	wholeT_0.01	J1:DiP, J1:DiP-A, J1:OT-A, J5:DiP, J5:DiP-A, J5:OT, J5:OT-A, S1:DiP, S1:DiP-A, S5:DiP, S5:DiP-A, S5:OT, S5:OT-A

Table 16: *(continued)*

	Conjunction	Drivers	Tree	S.Size	Model	sh	S.Time	S.Type	Best method(s)
219	Yes	9	9-A	200	McF_4	0	last	wholeT_0.5	J1:DiP, J1:DiP-A, J1:OT-A, J5:DiP, J5:DiP-A, J5:OT-A, S5:DiP, S5:OT, S5:OT-A
220	Yes	9	9-A	200	McF_4	0	unif	singleC	J1:DiP, J1:DiP-A, J1:OT-A, J5:DiP, J5:DiP-A, J5:OT, J5:OT-A, S1:DiP, S5:DiP, S5:DiP-A, S5:OT, S5:OT-A
221	Yes	9	9-A	200	McF_4	0	unif	wholeT_0.01	J1:DiP, J1:DiP-A, J1:OT, J1:OT-A, J5:DiP, J5:DiP-A, J5:OT, J5:OT-A, S1:DiP, S1:DiP-A, S5:DiP, S5:DiP-A, S5:OT, S5:OT-A
222	Yes	9	9-A	200	McF_4	0	unif	wholeT_0.5	J1:DiP, J1:DiP-A, J1:OT, J1:OT-A, J5:DiP, J5:DiP-A, J5:OT, J5:OT-A, S1:DiP, S1:DiP-A, S5:DiP, S5:DiP-A, S5:OT-A
223	Yes	9	9-A	200	McF_4	Inf	last	singleC	J1:DiP, J1:DiP-A, J5:DiP, J5:DiP-A, J5:OT, J5:OT-A, S5:DiP, S5:OT, S5:OT-A
224	Yes	9	9-A	200	McF_4	Inf	last	wholeT_0.01	J1:DiP, J1:DiP-A, J5:DiP, J5:DiP-A, J5:OT, S1:DiP, S5:DiP, S5:DiP-A, S5:OT
225	Yes	9	9-A	200	McF_4	Inf	last	wholeT_0.5	J1:DiP, J1:DiP-A, J1:OT, J1:OT-A, J5:DiP, J5:DiP-A, J5:OT, J5:OT-A, S1:DiP, S1:DiP-A, S5:DiP, S5:DiP-A, S5:OT, S5:OT-A
226	Yes	9	9-A	200	McF_4	Inf	unif	singleC	J1:DiP, J1:DiP-A, J1:OT, J1:OT-A, J5:DiP, J5:DiP-A, J5:OT, J5:OT-A, S1:DiP-A, S5:DiP, S5:DiP-A, S5:OT, S5:OT-A
227	Yes	9	9-A	200	McF_4	Inf	unif	wholeT_0.01	J1:DiP, J1:DiP-A, J1:OT, J5:DiP, J5:DiP-A, J5:OT, J5:OT-A, S1:DiP, S1:DiP-A, S5:DiP, S5:DiP-A, S5:OT, S5:OT-A
228	Yes	9	9-A	200	McF_4	Inf	unif	wholeT_0.5	J1:DiP, J1:DiP-A, J1:OT, J1:OT-A, J5:DiP, J5:DiP-A, J5:OT, J5:OT-A, S1:DiP, S1:DiP-A, S5:CBN-A, S5:DiP, S5:DiP-A, S5:OT, S5:OT-A
229	Yes	9	9-A	200	McF_6	0	last	singleC	J1:DiP, J5:DiP, J5:OT, S1:DiP, S5:DiP, S5:OT
230	Yes	9	9-A	200	McF_6	0	last	wholeT_0.01	J1:DiP, J5:DiP, J5:OT, S5:DiP, S5:OT
231	Yes	9	9-A	200	McF_6	0	last	wholeT_0.5	J1:DiP, J1:OT, J5:DiP, J5:OT, S1:DiP, S5:DiP, S5:OT

Table 16: *(continued)*

	Conjunction	Drivers	Tree	S.Size	Model	sh	S.Time	S.Type	Best method(s)
232	Yes	9	9-A	200	McF_6	0	unif	singleC	J1:CBN, J1:CBN-A, J1:DiP, J1:DiP-A, J1:OT, J1:OT-A, J5:CBN, J5:CBN-A, J5:DiP, J5:DiP-A, J5:OT, J5:OT-A, S1:DiP, S1:DiP-A, S5:CBN, S5:CBN-A, S5:DiP, S5:DiP-A, S5:OT, S5:OT-A
233	Yes	9	9-A	200	McF_6	0	unif	wholeT_0.01	J1:CBN, J1:CBN-A, J1:DiP, J1:DiP-A, J1:OT, J1:OT-A, J5:CBN, J5:CBN-A, J5:DiP, J5:DiP-A, J5:OT, J5:OT-A, S1:DiP-A, S5:CBN, S5:DiP, S5:DiP-A, S5:OT-A
234	Yes	9	9-A	200	McF_6	0	unif	wholeT_0.5	J1:CBN, J1:CBN-A, J1:DiP, J1:DiP-A, J1:OT, J1:OT-A, J5:CBN, J5:CBN-A, J5:DiP, J5:DiP-A, J5:OT, J5:OT-A, S1:DiP-A, S5:CBN, S5:CBN-A, S5:DiP, S5:DiP-A, S5:OT, S5:OT-A
235	Yes	9	9-A	200	McF_6	Inf	last	singleC	J1:DiP, J5:OT, S5:DiP, S5:OT
236	Yes	9	9-A	200	McF_6	Inf	last	wholeT_0.01	J1:DiP, J5:DiP, J5:OT, S5:DiP, S5:OT
237	Yes	9	9-A	200	McF_6	Inf	last	wholeT_0.5	J1:DiP, J1:OT, J5:DiP, J5:OT, S1:DiP, S5:DiP, S5:OT
238	Yes	9	9-A	200	McF_6	Inf	unif	singleC	J1:CBN, J1:CBN-A, J1:DiP, J1:DiP-A, J1:OT, J1:OT-A, J5:CBN, J5:CBN-A, J5:DiP, J5:DiP-A, J5:OT, J5:OT-A, S1:DiP, S1:DiP-A, S5:CBN, S5:CBN-A, S5:DiP, S5:DiP-A, S5:OT, S5:OT-A
239	Yes	9	9-A	200	McF_6	Inf	unif	wholeT_0.01	J1:CBN, J1:CBN-A, J1:DiP, J1:DiP-A, J1:OT-A, J5:CBN, J5:CBN-A, J5:DiP, J5:DiP-A, J5:OT, J5:OT-A, S1:DiP, S5:CBN, S5:OT, S5:OT-A
240	Yes	9	9-A	200	McF_6	Inf	unif	wholeT_0.5	J1:CBN, J1:CBN-A, J1:DiP, J1:DiP-A, J1:OT, J1:OT-A, J5:CBN, J5:CBN-A, J5:DiP, J5:DiP-A, J5:OT, J5:OT-A, S1:DiP, S1:DiP-A, S5:CBN, S5:CBN-A, S5:DiP, S5:DiP-A, S5:OT, S5:OT-A
241	Yes	9	9-A	100	Bozic	0	last	singleC	J1:DiP, J1:DiP-A, J1:OT, J1:OT-A, J5:DiP, J5:DiP-A, J5:OT, J5:OT-A, S1:DiP, S1:DiP-A, S5:DiP, S5:DiP-A, S5:OT, S5:OT-A
242	Yes	9	9-A	100	Bozic	0	last	wholeT_0.01	J1:DiP, J1:DiP-A, J5:DiP, J5:DiP-A, J5:OT, J5:OT-A, S1:DiP, S1:DiP-A, S5:DiP, S5:DiP-A, S5:OT, S5:OT-A

Table 16: *(continued)*

	Conjunction	Drivers	Tree	S.Size	Model	sh	S.Time	S.Type	Best method(s)
243	Yes	9	9-A	100	Bozic	0	last	wholeT_0.5	J1:DiP, J1:DiP-A, J1:OT, J1:OT-A, J5:DiP, J5:DiP-A, J5:OT, J5:OT-A, S1:DiP, S1:DiP-A, S5:DiP, S5:DiP-A, S5:OT, S5:OT-A
244	Yes	9	9-A	100	Bozic	0	unif	singleC	J1:DiP, J1:DiP-A, J5:CBN, J5:CBN-A, J5:DiP, J5:DiP-A, J5:OT, J5:OT-A, S1:DiP, S1:DiP-A, S5:DiP-A, S5:OT, S5:OT-A
245	Yes	9	9-A	100	Bozic	0	unif	wholeT_0.01	J1:DiP, J1:DiP-A, J1:OT, J5:DiP, J5:DiP-A, J5:OT, J5:OT-A, S1:DiP, S1:DiP-A, S5:DiP, S5:DiP-A, S5:OT, S5:OT-A
246	Yes	9	9-A	100	Bozic	0	unif	wholeT_0.5	J1:DiP, J1:DiP-A, J1:OT, J1:OT-A, J5:CBN, J5:CBN-A, J5:DiP, J5:DiP-A, J5:OT, J5:OT-A, S1:DiP, S1:DiP-A, S5:DiP, S5:DiP-A, S5:OT, S5:OT-A
247	Yes	9	9-A	100	Bozic	Inf	last	singleC	J1:DiP, J1:DiP-A, J5:DiP, J5:DiP-A, J5:OT, J5:OT-A, S1:DiP, S1:DiP-A, S5:DiP, S5:DiP-A
248	Yes	9	9-A	100	Bozic	Inf	last	wholeT_0.01	J5:DiP, J5:DiP-A
249	Yes	9	9-A	100	Bozic	Inf	last	wholeT_0.5	J1:DiP, J1:DiP-A, J5:DiP, J5:DiP-A, J5:OT, J5:OT-A, S1:DiP, S1:DiP-A, S5:DiP, S5:DiP-A
250	Yes	9	9-A	100	Bozic	Inf	unif	singleC	J1:DiP, J1:DiP-A, J5:DiP, J5:DiP-A, J5:OT, J5:OT-A, S1:DiP, S5:DiP, S5:DiP-A, S5:OT, S5:OT-A
251	Yes	9	9-A	100	Bozic	Inf	unif	wholeT_0.01	J5:DiP, J5:DiP-A, J5:OT, J5:OT-A
252	Yes	9	9-A	100	Bozic	Inf	unif	wholeT_0.5	J1:DiP, J1:DiP-A, J5:DiP, J5:DiP-A, J5:OT, J5:OT-A, S5:DiP, S5:DiP-A, S5:OT, S5:OT-A
253	Yes	9	9-A	100	exp	0	last	singleC	J1:DiP, J1:DiP-A, J1:OT, J1:OT-A, J5:CBN, J5:CBN-A, J5:DiP, J5:DiP-A, J5:OT, J5:OT-A, S1:DiP, S1:DiP-A, S5:DiP, S5:DiP-A, S5:OT, S5:OT-A
254	Yes	9	9-A	100	exp	0	last	wholeT_0.01	J1:DiP, J1:DiP-A, J1:OT, J1:OT-A, J5:DiP, J5:DiP-A, J5:OT, J5:OT-A, S1:DiP, S1:DiP-A, S5:DiP, S5:DiP-A, S5:OT, S5:OT-A

Table 16: *(continued)*

	Conjunction	Drivers	Tree	S.Size	Model	sh	S.Time	S.Type	Best method(s)
255	Yes	9	9-A	100	exp	0	last	wholeT_0.5	J1:DiP, J1:DiP-A, J1:OT, J1:OT-A, J5:CBN, J5:CBN-A, J5:DiP, J5:DiP-A, J5:OT, J5:OT-A, S1:DiP, S1:DiP-A, S5:DiP, S5:DiP-A, S5:OT, S5:OT-A
256	Yes	9	9-A	100	exp	0	unif	singleC	J1:DiP, J1:DiP-A, J1:OT, J1:OT-A, J5:CBN, J5:CBN-A, J5:DiP, J5:DiP-A, J5:OT, J5:OT-A, S1:DiP, S1:DiP-A, S1:OT, S1:OT-A, S5:DiP, S5:DiP-A, S5:OT, S5:OT-A
257	Yes	9	9-A	100	exp	0	unif	wholeT_0.01	J1:DiP, J1:DiP-A, J1:OT, J1:OT-A, J5:CBN, J5:CBN-A, J5:DiP, J5:DiP-A, J5:OT, J5:OT-A, S1:DiP, S5:DiP, S5:DiP-A, S5:OT, S5:OT-A
258	Yes	9	9-A	100	exp	0	unif	wholeT_0.5	J1:DiP, J1:DiP-A, J1:OT, J1:OT-A, J5:CBN, J5:CBN-A, J5:DiP, J5:DiP-A, J5:OT, J5:OT-A, S1:DiP, S1:DiP-A, S1:OT, S1:OT-A, S5:DiP, S5:DiP-A, S5:OT, S5:OT-A
259	Yes	9	9-A	100	exp	Inf	last	singleC	J1:DiP, J1:DiP-A, J5:DiP, J5:DiP-A, J5:OT, J5:OT-A, S1:DiP, S1:DiP-A, S5:DiP, S5:DiP-A, S5:OT, S5:OT-A
260	Yes	9	9-A	100	exp	Inf	last	wholeT_0.01	J5:DiP, J5:DiP-A, J5:OT, J5:OT-A
261	Yes	9	9-A	100	exp	Inf	last	wholeT_0.5	J1:DiP, J1:DiP-A, J5:DiP, J5:DiP-A, J5:OT, J5:OT-A, S1:DiP, S1:DiP-A, S5:DiP, S5:DiP-A, S5:OT, S5:OT-A
262	Yes	9	9-A	100	exp	Inf	unif	singleC	J1:DiP, J1:DiP-A, J1:OT-A, J5:CBN-A, J5:DiP, J5:DiP-A, J5:OT, J5:OT-A, S1:DiP, S1:DiP-A, S5:DiP, S5:DiP-A, S5:OT, S5:OT-A
263	Yes	9	9-A	100	exp	Inf	unif	wholeT_0.01	J5:DiP, J5:DiP-A, J5:OT, J5:OT-A, S5:DiP, S5:DiP-A, S5:OT, S5:OT-A
264	Yes	9	9-A	100	exp	Inf	unif	wholeT_0.5	J1:DiP, J1:DiP-A, J5:CBN, J5:CBN-A, J5:DiP, J5:DiP-A, J5:OT, J5:OT-A, S1:DiP, S1:DiP-A, S5:DiP, S5:DiP-A, S5:OT, S5:OT-A
265	Yes	9	9-A	100	McF_4	0	last	singleC	J1:DiP, J1:DiP-A, J5:DiP, J5:DiP-A, J5:OT, J5:OT-A, S1:DiP, S1:DiP-A, S5:DiP, S5:DiP-A, S5:OT, S5:OT-A

Table 16: *(continued)*

	Conjunction	Drivers	Tree	S.Size	Model	sh	S.Time	S.Type	Best method(s)
266	Yes	9	9-A	100	McF_4	0	last	wholeT_0.01	J1:DiP, J1:DiP-A, J5:DiP, J5:DiP-A, J5:OT, J5:OT-A, S1:DiP, S5:DiP, S5:DiP-A, S5:OT, S5:OT-A
267	Yes	9	9-A	100	McF_4	0	last	wholeT_0.5	J1:DiP, J1:DiP-A, J5:DiP, J5:DiP-A, J5:OT, J5:OT-A, S1:DiP, S1:DiP-A, S5:DiP, S5:DiP-A, S5:OT, S5:OT-A
268	Yes	9	9-A	100	McF_4	0	unif	singleC	J1:DiP, J1:DiP-A, J5:DiP, J5:DiP-A, J5:OT, J5:OT-A, S1:DiP, S1:DiP-A, S5:DiP, S5:DiP-A, S5:OT, S5:OT-A
269	Yes	9	9-A	100	McF_4	0	unif	wholeT_0.01	J1:DiP, J1:DiP-A, J5:DiP, J5:DiP-A, J5:OT, J5:OT-A, S5:DiP, S5:DiP-A, S5:OT, S5:OT-A
270	Yes	9	9-A	100	McF_4	0	unif	wholeT_0.5	J1:DiP, J1:DiP-A, J1:OT, J1:OT-A, J5:DiP, J5:DiP-A, J5:OT, J5:OT-A, S1:DiP, S1:DiP-A, S5:DiP, S5:DiP-A, S5:OT, S5:OT-A
271	Yes	9	9-A	100	McF_4	Inf	last	singleC	J1:DiP, J1:DiP-A, J5:DiP, J5:DiP-A, J5:OT, J5:OT-A, S1:DiP-A, S5:DiP, S5:DiP-A, S5:OT, S5:OT-A
272	Yes	9	9-A	100	McF_4	Inf	last	wholeT_0.01	J5:DiP, J5:DiP-A, J5:OT, S1:DiP, S5:DiP, S5:OT
273	Yes	9	9-A	100	McF_4	Inf	last	wholeT_0.5	J1:DiP, J1:DiP-A, J5:DiP, J5:DiP-A, J5:OT, J5:OT-A, S1:DiP, S1:DiP-A, S5:DiP, S5:DiP-A, S5:OT, S5:OT-A
274	Yes	9	9-A	100	McF_4	Inf	unif	singleC	J1:DiP, J1:DiP-A, J1:OT, J1:OT-A, J5:DiP, J5:DiP-A, J5:OT, J5:OT-A, S1:DiP, S1:DiP-A, S5:DiP, S5:DiP-A, S5:OT, S5:OT-A
275	Yes	9	9-A	100	McF_4	Inf	unif	wholeT_0.01	J1:DiP, J1:DiP-A, J5:DiP, J5:DiP-A, J5:OT, J5:OT-A, S5:DiP, S5:DiP-A, S5:OT, S5:OT-A
276	Yes	9	9-A	100	McF_4	Inf	unif	wholeT_0.5	J1:DiP, J1:DiP-A, J1:OT, J1:OT-A, J5:DiP, J5:DiP-A, J5:OT, J5:OT-A, S1:DiP, S1:DiP-A, S5:DiP, S5:DiP-A, S5:OT, S5:OT-A
277	Yes	9	9-A	100	McF_6	0	last	singleC	J1:DiP, J5:DiP, J5:OT, S1:DiP, S5:DiP, S5:DiP-A, S5:OT
278	Yes	9	9-A	100	McF_6	0	last	wholeT_0.01	J1:DiP, J5:DiP, J5:OT, S1:DiP, S5:DiP, S5:OT

Table 16: *(continued)*

	Conjunction	Drivers	Tree	S.Size	Model	sh	S.Time	S.Type	Best method(s)
279	Yes	9	9-A	100	McF_6	0	last	wholeT_0.5	J1:DiP, J5:DiP, J5:OT, S1:DiP, S5:DiP, S5:DiP-A, S5:OT
280	Yes	9	9-A	100	McF_6	0	unif	singleC	J1:CBN, J1:CBN-A, J1:DiP, J1:DiP-A, J1:OT, J5:CBN, J5:CBN-A, J5:DiP, J5:DiP-A, J5:OT, J5:OT-A, S1:DiP, S1:DiP-A, S5:CBN, S5:CBN-A, S5:DiP, S5:DiP-A, S5:OT
281	Yes	9	9-A	100	McF_6	0	unif	wholeT_0.01	J1:DiP, J1:DiP-A, J1:OT-A, J5:CBN, J5:CBN-A, J5:DiP, J5:DiP-A, J5:OT, J5:OT-A, S5:DiP, S5:DiP-A, S5:OT, S5:OT-A
282	Yes	9	9-A	100	McF_6	0	unif	wholeT_0.5	J1:CBN, J1:CBN-A, J1:DiP, J1:DiP-A, J1:OT, J5:CBN, J5:CBN-A, J5:DiP, J5:DiP-A, J5:OT, J5:OT-A, S1:DiP, S5:CBN, S5:CBN-A, S5:DiP, S5:DiP-A, S5:OT, S5:OT-A
283	Yes	9	9-A	100	McF_6	Inf	last	singleC	J1:DiP, J5:DiP, J5:OT, S1:DiP, S5:DiP, S5:OT
284	Yes	9	9-A	100	McF_6	Inf	last	wholeT_0.01	J1:DiP, J5:DiP, J5:OT, S1:DiP, S5:DiP, S5:OT
285	Yes	9	9-A	100	McF_6	Inf	last	wholeT_0.5	J1:DiP, J5:DiP, J5:DiP-A, J5:OT, S1:DiP, S5:DiP, S5:OT
286	Yes	9	9-A	100	McF_6	Inf	unif	singleC	J1:CBN, J1:CBN-A, J1:DiP, J1:DiP-A, J1:OT-A, J5:CBN, J5:CBN-A, J5:DiP, J5:DiP-A, J5:OT, J5:OT-A, S1:DiP, S5:CBN, S5:CBN-A, S5:DiP, S5:DiP-A, S5:OT, S5:OT-A
287	Yes	9	9-A	100	McF_6	Inf	unif	wholeT_0.01	J1:DiP, J5:CBN-A, J5:DiP, J5:DiP-A, J5:OT, J5:OT-A, S5:CBN, S5:CBN-A, S5:DiP, S5:OT, S5:OT-A
288	Yes	9	9-A	100	McF_6	Inf	unif	wholeT_0.5	J1:DiP, J1:OT, J5:CBN, J5:CBN-A, J5:DiP, J5:OT, J5:OT-A, S1:DiP, S1:DiP-A, S5:CBN-A, S5:DiP, S5:DiP-A, S5:OT, S5:OT-A
289	Yes	7	7-A	1000	Bozic	0	last	singleC	J1:CBN-A, J1:DiP, J1:DiP-A, J1:OT, J1:OT-A, J5:CBN, J5:CBN-A, J5:DiP, J5:DiP-A, J5:OT, J5:OT-A, S1:DiP, S1:DiP-A, S1:OT, S1:OT-A, S5:DiP, S5:DiP-A, S5:OT, S5:OT-A
290	Yes	7	7-A	1000	Bozic	0	last	wholeT_0.01	none

Table 16: *(continued)*

	Conjunction	Drivers	Tree	S.Size	Model	sh	S.Time	S.Type	Best method(s)
291	Yes	7	7-A	1000	Bozic	0	last	wholeT_0.5	J1:CBN, J1:DiP, J1:DiP-A, J1:OT, J1:OT-A, J5:CBN, J5:CBN-A, J5:DiP, J5:DiP-A, J5:OT, J5:OT-A, S1:DiP, S1:DiP-A, S1:OT, S1:OT-A, S5:CBN, S5:CBN-A, S5:DiP, S5:DiP-A, S5:OT, S5:OT-A
292	Yes	7	7-A	1000	Bozic	0	unif	singleC	J1:CBN, J1:CBN-A, J1:DiP, J1:OT, J1:OT-A, J5:CBN, J5:CBN-A, J5:DiP, J5:DiP-A, J5:OT, J5:OT-A, S1:DiP, S1:DiP-A, S1:OT, S1:OT-A, S5:CBN, S5:CBN-A, S5:DiP, S5:DiP-A, S5:OT, S5:OT-A
293	Yes	7	7-A	1000	Bozic	0	unif	wholeT_0.01	J5:CBN, J5:CBN-A, J5:DiP, J5:DiP-A, J5:OT, J5:OT-A
294	Yes	7	7-A	1000	Bozic	0	unif	wholeT_0.5	none
295	Yes	7	7-A	1000	Bozic	Inf	last	singleC	J1:DiP, J1:DiP-A, J1:OT, J1:OT-A, J5:DiP, J5:DiP-A, J5:OT, J5:OT-A, S1:DiP-A, S5:DiP, S5:DiP-A, S5:OT, S5:OT-A
296	Yes	7	7-A	1000	Bozic	Inf	last	wholeT_0.01	J5:CBN, J5:CBN-A, J5:DiP, J5:OT
297	Yes	7	7-A	1000	Bozic	Inf	last	wholeT_0.5	J1:DiP, J1:DiP-A, J1:OT, J1:OT-A, J5:DiP, J5:DiP-A, J5:OT, J5:OT-A, S1:DiP, S1:DiP-A, S5:DiP, S5:DiP-A, S5:OT, S5:OT-A
298	Yes	7	7-A	1000	Bozic	Inf	unif	singleC	J1:CBN, J1:CBN-A, J5:CBN, J5:CBN-A, J5:DiP, J5:DiP-A, J5:OT, J5:OT-A, S1:CBN, S1:CBN-A, S5:CBN, S5:CBN-A
299	Yes	7	7-A	1000	Bozic	Inf	unif	wholeT_0.01	J5:CBN, J5:CBN-A, S5:CBN, S5:CBN-A
300	Yes	7	7-A	1000	Bozic	Inf	unif	wholeT_0.5	J1:CBN, J1:CBN-A, J5:CBN, J5:CBN-A, J5:DiP, J5:DiP-A, J5:OT, J5:OT-A, S1:CBN, S1:CBN-A, S5:CBN, S5:CBN-A, S5:DiP, S5:DiP-A, S5:OT, S5:OT-A
301	Yes	7	7-A	1000	exp	0	last	singleC	J1:CBN, J1:CBN-A, J1:DiP, J1:DiP-A, J1:OT, J1:OT-A, J5:CBN, J5:CBN-A, J5:DiP-A, J5:OT, J5:OT-A, S1:DiP, S1:DiP-A, S1:OT, S1:OT-A, S5:CBN, S5:CBN-A, S5:DiP, S5:DiP-A, S5:OT, S5:OT-A

Table 16: *(continued)*

	Conjunction	Drivers	Tree	S.Size	Model	sh	S.Time	S.Type	Best method(s)
302	Yes	7	7-A	1000	exp	0	last	wholeT_0.01	J1:CBN, J1:CBN-A, J1:DiP, J1:OT, J1:OT-A, J5:CBN, J5:CBN-A, J5:DiP, J5:DiP-A, J5:OT, J5:OT-A, S1:CBN-A, S1:DiP, S1:DiP-A, S1:OT, S1:OT-A, S5:DiP, S5:DiP-A, S5:OT, S5:OT-A
303	Yes	7	7-A	1000	exp	0	last	wholeT_0.5	J1:CBN, J1:CBN-A, J1:DiP, J1:DiP-A, J1:OT, J1:OT-A, J5:CBN, J5:CBN-A, J5:DiP, J5:DiP-A, J5:OT, J5:OT-A, S1:DiP, S1:DiP-A, S1:OT, S1:OT-A, S5:CBN, S5:CBN-A, S5:DiP, S5:DiP-A, S5:OT, S5:OT-A
304	Yes	7	7-A	1000	exp	0	unif	singleC	J1:CBN, J1:CBN-A, J1:DiP, J1:OT, J1:OT-A, J5:CBN, J5:CBN-A, J5:DiP, J5:DiP-A, J5:OT, J5:OT-A, S1:CBN, S1:CBN-A, S1:DiP, S1:DiP-A, S1:OT, S1:OT-A, S5:CBN, S5:CBN-A, S5:DiP, S5:DiP-A, S5:OT, S5:OT-A
305	Yes	7	7-A	1000	exp	0	unif	wholeT_0.01	J1:CBN, J1:CBN-A, J1:DiP, J1:DiP-A, J1:OT, J1:OT-A, J5:CBN, J5:CBN-A, J5:DiP, J5:DiP-A, J5:OT, J5:OT-A, S5:CBN, S5:CBN-A, S5:DiP, S5:DiP-A, S5:OT, S5:OT-A
306	Yes	7	7-A	1000	exp	0	unif	wholeT_0.5	none
307	Yes	7	7-A	1000	exp	Inf	last	singleC	J1:DiP, J1:DiP-A, J1:OT, J1:OT-A, J5:CBN, J5:CBN-A, J5:DiP, J5:DiP-A, J5:OT, J5:OT-A, S1:DiP, S1:DiP-A, S5:DiP, S5:DiP-A, S5:OT, S5:OT-A
308	Yes	7	7-A	1000	exp	Inf	last	wholeT_0.01	J1:CBN-A, J5:CBN, J5:CBN-A, J5:DiP, J5:DiP-A, J5:OT, J5:OT-A, S5:CBN, S5:CBN-A, S5:DiP, S5:DiP-A, S5:OT, S5:OT-A
309	Yes	7	7-A	1000	exp	Inf	last	wholeT_0.5	J1:DiP, J1:DiP-A, J1:OT, J1:OT-A, J5:CBN, J5:CBN-A, J5:DiP, J5:DiP-A, J5:OT, J5:OT-A, S1:DiP, S1:DiP-A, S1:OT, S1:OT-A, S5:DiP, S5:DiP-A, S5:OT, S5:OT-A

Table 16: *(continued)*

	Conjunction	Drivers	Tree	S.Size	Model	sh	S.Time	S.Type	Best method(s)
310	Yes	7	7-A	1000	exp	Inf	unif	singleC	J1:CBN, J1:CBN-A, J1:DiP, J1:DiP-A, J1:OT, J1:OT-A, J5:CBN, J5:CBN-A, J5:DiP, J5:DiP-A, J5:OT, J5:OT-A, S1:CBN, S1:DiP-A, S5:CBN, S5:CBN-A, S5:DiP, S5:DiP-A, S5:OT, S5:OT-A
311	Yes	7	7-A	1000	exp	Inf	unif	wholeT_0.01	J1:CBN, J1:CBN-A, J5:CBN, J5:CBN-A, S5:CBN, S5:CBN-A
312	Yes	7	7-A	1000	exp	Inf	unif	wholeT_0.5	J1:CBN, J1:CBN-A, J1:DiP, J1:DiP-A, J1:OT, J1:OT-A, J5:CBN, J5:CBN-A, J5:DiP, J5:DiP-A, J5:OT, J5:OT-A, S1:CBN, S1:CBN-A, S5:CBN, S5:CBN-A, S5:DiP, S5:DiP-A, S5:OT, S5:OT-A
313	Yes	7	7-A	1000	McF_4	0	last	singleC	J1:DiP, J1:DiP-A, J1:OT, J1:OT-A, J5:DiP, J5:DiP-A, J5:OT, S1:DiP, S1:DiP-A, S1:OT-A, S5:DiP
314	Yes	7	7-A	1000	McF_4	0	last	wholeT_0.01	J1:DiP-A, J1:OT, J1:OT-A, J5:DiP, J5:DiP-A, J5:OT, J5:OT-A, S5:OT, S5:OT-A
315	Yes	7	7-A	1000	McF_4	0	last	wholeT_0.5	J1:DiP, J1:DiP-A, J1:OT, J1:OT-A, J5:DiP, J5:DiP-A, J5:OT, J5:OT-A, S1:DiP, S1:DiP-A, S1:OT, S1:OT-A, S5:DiP, S5:DiP-A, S5:OT, S5:OT-A
316	Yes	7	7-A	1000	McF_4	0	unif	singleC	J1:CBN, J1:CBN-A, J1:DiP, J1:DiP-A, J1:OT, J1:OT-A, J5:CBN, J5:CBN-A, J5:DiP, J5:DiP-A, J5:OT-A, S1:CBN, S1:CBN-A, S1:DiP, S1:DiP-A, S1:OT, S1:OT-A, S5:CBN, S5:CBN-A, S5:DiP, S5:DiP-A, S5:OT, S5:OT-A
317	Yes	7	7-A	1000	McF_4	0	unif	wholeT_0.01	J1:CBN, J1:CBN-A, J1:DiP, J1:DiP-A, J1:OT, J1:OT-A, J5:CBN, J5:CBN-A, J5:DiP, J5:DiP-A, J5:OT, J5:OT-A, S1:DiP, S1:DiP-A, S5:CBN, S5:CBN-A, S5:DiP, S5:DiP-A, S5:OT, S5:OT-A
318	Yes	7	7-A	1000	McF_4	0	unif	wholeT_0.5	J1:CBN, J1:CBN-A, J1:DiP, J1:DiP-A, J1:OT, J1:OT-A, J5:CBN, J5:CBN-A, J5:DiP, J5:DiP-A, J5:OT, J5:OT-A, S1:CBN, S1:CBN-A, S1:DiP, S1:DiP-A, S1:OT, S1:OT-A, S5:CBN, S5:DiP, S5:DiP-A, S5:OT, S5:OT-A

Table 16: *(continued)*

	Conjunction	Drivers	Tree	S.Size	Model	sh	S.Time	S.Type	Best method(s)
319	Yes	7	7-A	1000	McF_4	Inf	last	singleC	J1:DiP, J1:DiP-A, J1:OT, J1:OT-A, J5:DiP, J5:DiP-A, J5:OT, J5:OT-A, S1:DiP, S1:DiP-A, S1:OT, S5:DiP, S5:DiP-A, S5:OT, S5:OT-A
320	Yes	7	7-A	1000	McF_4	Inf	last	wholeT_0.01	J1:DiP, J5:DiP, J5:DiP-A, J5:OT, J5:OT-A, S5:DiP, S5:OT, S5:OT-A
321	Yes	7	7-A	1000	McF_4	Inf	last	wholeT_0.5	J1:DiP, J1:DiP-A, J1:OT, J1:OT-A, J5:DiP, J5:DiP-A, J5:OT, J5:OT-A, S1:DiP, S1:DiP-A, S1:OT, S5:DiP, S5:DiP-A, S5:OT, S5:OT-A
322	Yes	7	7-A	1000	McF_4	Inf	unif	singleC	J1:CBN, J1:CBN-A, J1:DiP, J1:DiP-A, J1:OT, J1:OT-A, J5:CBN, J5:CBN-A, J5:DiP, J5:DiP-A, J5:OT, J5:OT-A, S1:CBN, S1:CBN-A, S1:DiP, S1:OT, S1:OT-A, S5:CBN-A, S5:DiP, S5:DiP-A, S5:OT, S5:OT-A
323	Yes	7	7-A	1000	McF_4	Inf	unif	wholeT_0.01	J1:CBN, J1:CBN-A, J1:DiP, J1:DiP-A, J1:OT, J1:OT-A, J5:CBN, J5:CBN-A, J5:DiP, J5:DiP-A, J5:OT, J5:OT-A, S5:CBN, S5:CBN-A, S5:DiP, S5:DiP-A, S5:OT, S5:OT-A
324	Yes	7	7-A	1000	McF_4	Inf	unif	wholeT_0.5	J1:CBN, J1:CBN-A, J1:DiP, J1:OT, J1:OT-A, J5:CBN, J5:CBN-A, J5:DiP, J5:DiP-A, J5:OT, J5:OT-A, S1:CBN, S1:CBN-A, S1:DiP, S1:DiP-A, S1:OT, S1:OT-A, S5:CBN, S5:DiP-A, S5:OT, S5:OT-A
325	Yes	7	7-A	1000	McF_6	0	last	singleC	J1:DiP, J1:OT, J1:OT-A, J5:DiP, J5:OT, J5:OT-A, S1:DiP, S5:DiP, S5:OT, S5:OT-A
326	Yes	7	7-A	1000	McF_6	0	last	wholeT_0.01	J1:DiP, J1:OT, J1:OT-A, J5:DiP, J5:OT, J5:OT-A, S1:DiP, S5:DiP, S5:OT, S5:OT-A
327	Yes	7	7-A	1000	McF_6	0	last	wholeT_0.5	J1:DiP, J1:OT, J1:OT-A, J5:DiP, J5:OT, J5:OT-A, S1:DiP, S5:DiP, S5:OT, S5:OT-A
328	Yes	7	7-A	1000	McF_6	0	unif	singleC	none

Table 16: *(continued)*

	Conjunction	Drivers	Tree	S.Size	Model	sh	S.Time	S.Type	Best method(s)
329	Yes	7	7-A	1000	McF_6	0	unif	wholeT_0.01	J1:CBN, J1:CBN-A, J1:DiP, J1:DiP-A, J1:OT, J1:OT-A, J5:CBN, J5:CBN-A, J5:DiP, J5:DiP-A, J5:OT, J5:OT-A, S5:CBN, S5:CBN-A, S5:DiP, S5:DiP-A, S5:OT, S5:OT-A
330	Yes	7	7-A	1000	McF_6	0	unif	wholeT_0.5	none
331	Yes	7	7-A	1000	McF_6	Inf	last	singleC	J1:DiP, J1:DiP-A, J1:OT, J1:OT-A, J5:DiP, J5:DiP-A, J5:OT, J5:OT-A, S1:DiP, S5:DiP, S5:OT, S5:OT-A
332	Yes	7	7-A	1000	McF_6	Inf	last	wholeT_0.01	J1:DiP, J5:DiP, J5:OT, J5:OT-A, S1:DiP, S5:DiP, S5:OT, S5:OT-A
333	Yes	7	7-A	1000	McF_6	Inf	last	wholeT_0.5	J1:DiP, J1:DiP-A, J1:OT, J1:OT-A, J5:DiP, J5:DiP-A, J5:OT, J5:OT-A, S1:DiP, S1:DiP-A, S5:DiP, S5:DiP-A, S5:OT, S5:OT-A
334	Yes	7	7-A	1000	McF_6	Inf	unif	singleC	J1:CBN, J1:CBN-A, J1:DiP, J1:DiP-A, J1:OT, J1:OT-A, J5:CBN, J5:DiP, J5:DiP-A, J5:OT, J5:OT-A, S1:CBN, S1:CBN-A, S1:DiP, S1:DiP-A, S1:OT, S1:OT-A, S5:CBN, S5:CBN-A, S5:DiP, S5:DiP-A, S5:OT, S5:OT-A
335	Yes	7	7-A	1000	McF_6	Inf	unif	wholeT_0.01	J1:CBN, J1:CBN-A, J1:DiP, J1:DiP-A, J1:OT, J1:OT-A, J5:CBN, J5:CBN-A, J5:DiP, J5:DiP-A, J5:OT, J5:OT-A, S5:CBN, S5:CBN-A, S5:DiP, S5:DiP-A, S5:OT, S5:OT-A
336	Yes	7	7-A	1000	McF_6	Inf	unif	wholeT_0.5	J1:CBN, J1:CBN-A, J1:DiP, J1:DiP-A, J1:OT, J1:OT-A, J5:CBN, J5:DiP, J5:DiP-A, J5:OT, J5:OT-A, S1:CBN, S1:CBN-A, S1:DiP, S1:DiP-A, S1:OT, S1:OT-A, S5:CBN, S5:CBN-A, S5:DiP, S5:DiP-A, S5:OT, S5:OT-A
337	Yes	7	7-A	200	Bozic	0	last	singleC	J1:CBN, J1:CBN-A, J1:DiP, J1:DiP-A, J1:OT, J1:OT-A, J5:CBN, J5:CBN-A, J5:DiP, J5:DiP-A, J5:OT, J5:OT-A, S1:DiP, S1:DiP-A, S1:OT, S5:DiP, S5:DiP-A, S5:OT, S5:OT-A

Table 16: *(continued)*

	Conjunction	Drivers	Tree	S.Size	Model	sh	S.Time	S.Type	Best method(s)
338	Yes	7	7-A	200	Bozic	0	last	wholeT_0.01	J1:CBN-A, J1:DiP, J1:DiP-A, J1:OT, J1:OT-A, J5:CBN, J5:CBN-A, J5:DiP, J5:DiP-A, J5:OT, J5:OT-A, S1:DiP, S1:DiP-A, S5:CBN, S5:CBN-A, S5:DiP, S5:DiP-A, S5:OT, S5:OT-A
339	Yes	7	7-A	200	Bozic	0	last	wholeT_0.5	J1:CBN, J1:CBN-A, J1:DiP, J1:DiP-A, J1:OT, J1:OT-A, J5:CBN, J5:CBN-A, J5:DiP, J5:DiP-A, J5:OT, J5:OT-A, S1:DiP, S1:DiP-A, S5:CBN, S5:CBN-A, S5:DiP, S5:DiP-A, S5:OT, S5:OT-A
340	Yes	7	7-A	200	Bozic	0	unif	singleC	J1:CBN-A, J1:DiP, J1:DiP-A, J1:OT, J1:OT-A, J5:CBN, J5:CBN-A, J5:DiP, J5:DiP-A, J5:OT, J5:OT-A, S1:DiP, S1:DiP-A, S1:OT, S5:CBN, S5:CBN-A, S5:DiP, S5:DiP-A, S5:OT
341	Yes	7	7-A	200	Bozic	0	unif	wholeT_0.01	J5:CBN, J5:CBN-A, J5:DiP, J5:DiP-A, J5:OT, J5:OT-A
342	Yes	7	7-A	200	Bozic	0	unif	wholeT_0.5	J1:CBN-A, J1:DiP, J1:DiP-A, J1:OT, J1:OT-A, J5:CBN, J5:CBN-A, J5:DiP, J5:DiP-A, J5:OT, J5:OT-A, S1:DiP, S1:DiP-A, S1:OT-A, S5:CBN, S5:CBN-A, S5:DiP, S5:DiP-A, S5:OT, S5:OT-A
343	Yes	7	7-A	200	Bozic	Inf	last	singleC	J1:DiP, J1:DiP-A, J5:DiP, J5:DiP-A, J5:OT, J5:OT-A, S5:DiP, S5:DiP-A, S5:OT, S5:OT-A
344	Yes	7	7-A	200	Bozic	Inf	last	wholeT_0.01	J5:CBN-A, J5:DiP, J5:DiP-A, J5:OT
345	Yes	7	7-A	200	Bozic	Inf	last	wholeT_0.5	J1:DiP, J1:DiP-A, J5:DiP, J5:DiP-A, J5:OT, J5:OT-A, S1:DiP, S1:DiP-A, S5:DiP, S5:DiP-A, S5:OT, S5:OT-A
346	Yes	7	7-A	200	Bozic	Inf	unif	singleC	J1:CBN, J1:CBN-A, J5:CBN, J5:CBN-A, J5:DiP, J5:DiP-A, J5:OT, J5:OT-A, S5:CBN, S5:CBN-A
347	Yes	7	7-A	200	Bozic	Inf	unif	wholeT_0.01	J5:CBN, J5:CBN-A, S5:CBN, S5:CBN-A
348	Yes	7	7-A	200	Bozic	Inf	unif	wholeT_0.5	J1:CBN, J1:CBN-A, J5:CBN, J5:CBN-A, J5:DiP, J5:DiP-A, J5:OT, J5:OT-A, S5:CBN, S5:CBN-A, S5:DiP, S5:DiP-A, S5:OT, S5:OT-A

Table 16: *(continued)*

	Conjunction	Drivers	Tree	S.Size	Model	sh	S.Time	S.Type	Best method(s)
349	Yes	7	7-A	200	exp	0	last	singleC	J1:CBN, J1:CBN-A, J1:DiP, J1:DiP-A, J1:OT, J1:OT-A, J5:CBN, J5:CBN-A, J5:DiP, J5:DiP-A, J5:OT, J5:OT-A, S1:DiP, S1:DiP-A, S1:OT, S1:OT-A, S5:CBN, S5:CBN-A, S5:DiP, S5:DiP-A, S5:OT, S5:OT-A
350	Yes	7	7-A	200	exp	0	last	wholeT_0.01	J1:CBN, J1:CBN-A, J1:DiP, J1:DiP-A, J1:OT, J1:OT-A, J5:CBN, J5:CBN-A, J5:DiP, J5:DiP-A, J5:OT, J5:OT-A, S1:CBN, S1:CBN-A, S1:DiP, S1:DiP-A, S5:CBN, S5:DiP, S5:DiP-A
351	Yes	7	7-A	200	exp	0	last	wholeT_0.5	J1:CBN, J1:CBN-A, J1:DiP, J1:DiP-A, J1:OT, J1:OT-A, J5:CBN, J5:CBN-A, J5:DiP, J5:DiP-A, J5:OT, J5:OT-A, S1:DiP, S1:DiP-A, S1:OT, S1:OT-A, S5:CBN, S5:CBN-A, S5:DiP, S5:DiP-A, S5:OT, S5:OT-A
352	Yes	7	7-A	200	exp	0	unif	singleC	none
353	Yes	7	7-A	200	exp	0	unif	wholeT_0.01	J1:CBN, J1:CBN-A, J1:DiP, J1:DiP-A, J1:OT, J1:OT-A, J5:CBN, J5:CBN-A, J5:DiP, J5:DiP-A, J5:OT, J5:OT-A, S1:DiP, S1:DiP-A, S5:CBN, S5:CBN-A, S5:DiP, S5:DiP-A, S5:OT, S5:OT-A
354	Yes	7	7-A	200	exp	0	unif	wholeT_0.5	J1:CBN, J1:CBN-A, J1:DiP, J1:DiP-A, J1:OT, J1:OT-A, J5:CBN, J5:CBN-A, J5:DiP, J5:DiP-A, J5:OT, J5:OT-A, S1:CBN, S1:CBN-A, S1:DiP, S1:DiP-A, S1:OT, S1:OT-A, S5:CBN, S5:CBN-A, S5:DiP-A, S5:OT, S5:OT-A
355	Yes	7	7-A	200	exp	Inf	last	singleC	J1:DiP, J1:DiP-A, J1:OT, J1:OT-A, J5:CBN, J5:CBN-A, J5:DiP, J5:DiP-A, J5:OT, J5:OT-A, S1:DiP, S1:DiP-A, S5:DiP, S5:DiP-A, S5:OT, S5:OT-A
356	Yes	7	7-A	200	exp	Inf	last	wholeT_0.01	J1:DiP, J5:CBN, J5:CBN-A, J5:DiP, J5:DiP-A, J5:OT, S5:CBN-A, S5:DiP, S5:DiP-A
357	Yes	7	7-A	200	exp	Inf	last	wholeT_0.5	J1:DiP, J1:DiP-A, J1:OT, J1:OT-A, J5:CBN, J5:CBN-A, J5:DiP, J5:DiP-A, J5:OT, J5:OT-A, S1:DiP, S1:DiP-A, S5:DiP, S5:DiP-A, S5:OT, S5:OT-A

Table 16: *(continued)*

	Conjunction	Drivers	Tree	S.Size	Model	sh	S.Time	S.Type	Best method(s)
358	Yes	7	7-A	200	exp	Inf	unif	singleC	J1:CBN, J1:CBN-A, J1:DiP, J1:DiP-A, J1:OT-A, J5:CBN, J5:DiP, J5:DiP-A, J5:OT, J5:OT-A, S1:DiP-A, S5:CBN, S5:CBN-A, S5:DiP, S5:DiP-A, S5:OT, S5:OT-A
359	Yes	7	7-A	200	exp	Inf	unif	wholeT_0.01	J5:CBN, J5:CBN-A, J5:DiP-A, J5:OT, J5:OT-A, S5:CBN, S5:CBN-A
360	Yes	7	7-A	200	exp	Inf	unif	wholeT_0.5	J1:CBN, J1:CBN-A, J1:DiP, J1:DiP-A, J1:OT, J1:OT-A, J5:CBN-A, J5:DiP, J5:DiP-A, J5:OT, J5:OT-A, S1:DiP, S1:DiP-A, S5:CBN, S5:CBN-A, S5:DiP, S5:DiP-A, S5:OT, S5:OT-A
361	Yes	7	7-A	200	McF_4	0	last	singleC	J1:DiP, J1:DiP-A, J1:OT, J1:OT-A, J5:DiP, J5:DiP-A, J5:OT, J5:OT-A, S5:DiP, S5:DiP-A, S5:OT, S5:OT-A
362	Yes	7	7-A	200	McF_4	0	last	wholeT_0.01	J1:DiP, J1:DiP-A, J5:DiP, J5:DiP-A, J5:OT, J5:OT-A, S1:DiP, S1:DiP-A, S5:DiP, S5:DiP-A, S5:OT, S5:OT-A
363	Yes	7	7-A	200	McF_4	0	last	wholeT_0.5	J1:DiP, J1:DiP-A, J1:OT, J1:OT-A, J5:DiP, J5:DiP-A, J5:OT, J5:OT-A, S5:DiP, S5:DiP-A, S5:OT
364	Yes	7	7-A	200	McF_4	0	unif	singleC	J1:CBN, J1:CBN-A, J1:DiP, J1:DiP-A, J1:OT, J1:OT-A, J5:CBN, J5:CBN-A, J5:DiP, J5:DiP-A, J5:OT, J5:OT-A, S1:DiP, S1:DiP-A, S5:CBN, S5:CBN-A, S5:DiP, S5:DiP-A, S5:OT, S5:OT-A
365	Yes	7	7-A	200	McF_4	0	unif	wholeT_0.01	J1:CBN, J1:CBN-A, J1:DiP, J1:DiP-A, J1:OT, J1:OT-A, J5:CBN, J5:CBN-A, J5:DiP, J5:DiP-A, J5:OT, J5:OT-A, S1:DiP, S1:DiP-A, S5:CBN, S5:CBN-A, S5:DiP, S5:DiP-A, S5:OT, S5:OT-A
366	Yes	7	7-A	200	McF_4	0	unif	wholeT_0.5	J1:CBN, J1:CBN-A, J1:DiP, J1:DiP-A, J1:OT, J1:OT-A, J5:CBN, J5:CBN-A, J5:DiP, J5:DiP-A, J5:OT, S1:DiP, S1:DiP-A, S5:CBN, S5:CBN-A, S5:DiP, S5:DiP-A, S5:OT, S5:OT-A
367	Yes	7	7-A	200	McF_4	Inf	last	singleC	J1:DiP, J1:DiP-A, J1:OT, J1:OT-A, J5:DiP, J5:DiP-A, J5:OT, J5:OT-A, S1:DiP, S1:DiP-A, S5:DiP, S5:DiP-A, S5:OT, S5:OT-A

Table 16: *(continued)*

	Conjunction	Drivers	Tree	S.Size	Model	sh	S.Time	S.Type	Best method(s)
368	Yes	7	7-A	200	McF_4	Inf	last	wholeT_0.01	J1:DiP, J1:DiP-A, J5:DiP, J5:DiP-A, J5:OT, J5:OT-A, S1:DiP-A, S5:DiP, S5:DiP-A, S5:OT, S5:OT-A
369	Yes	7	7-A	200	McF_4	Inf	last	wholeT_0.5	J1:DiP, J1:DiP-A, J1:OT, J1:OT-A, J5:DiP, J5:DiP-A, J5:OT, J5:OT-A, S5:DiP, S5:DiP-A, S5:OT, S5:OT-A
370	Yes	7	7-A	200	McF_4	Inf	unif	singleC	J1:CBN, J1:CBN-A, J1:DiP, J1:DiP-A, J1:OT, J1:OT-A, J5:CBN, J5:CBN-A, J5:DiP, J5:DiP-A, J5:OT, J5:OT-A, S5:CBN, S5:CBN-A, S5:DiP, S5:DiP-A, S5:OT, S5:OT-A
371	Yes	7	7-A	200	McF_4	Inf	unif	wholeT_0.01	J1:CBN, J1:CBN-A, J1:DiP, J1:DiP-A, J1:OT, J1:OT-A, J5:CBN, J5:CBN-A, J5:DiP, J5:DiP-A, J5:OT, J5:OT-A, S1:DiP, S1:DiP-A, S5:CBN, S5:CBN-A, S5:DiP, S5:DiP-A, S5:OT, S5:OT-A
372	Yes	7	7-A	200	McF_4	Inf	unif	wholeT_0.5	J1:CBN, J1:CBN-A, J1:DiP, J1:DiP-A, J1:OT, J1:OT-A, J5:CBN, J5:CBN-A, J5:DiP, J5:DiP-A, J5:OT, J5:OT-A, S1:DiP, S1:DiP-A, S5:CBN, S5:CBN-A, S5:DiP, S5:DiP-A, S5:OT, S5:OT-A
373	Yes	7	7-A	200	McF_6	0	last	singleC	J1:DiP, J1:OT, J5:DiP, J5:OT, J5:OT-A, S1:DiP, S5:DiP, S5:OT, S5:OT-A
374	Yes	7	7-A	200	McF_6	0	last	wholeT_0.01	J1:DiP, J5:DiP, J5:OT, J5:OT-A, S1:DiP, S5:DiP, S5:OT, S5:OT-A
375	Yes	7	7-A	200	McF_6	0	last	wholeT_0.5	J1:DiP, J1:OT, J1:OT-A, J5:DiP, J5:OT, J5:OT-A, S5:DiP, S5:OT, S5:OT-A
376	Yes	7	7-A	200	McF_6	0	unif	singleC	J1:CBN, J1:CBN-A, J1:DiP, J1:DiP-A, J5:CBN, J5:CBN-A, J5:DiP, J5:DiP-A, S1:DiP, S1:DiP-A, S5:CBN, S5:CBN-A, S5:DiP, S5:DiP-A
377	Yes	7	7-A	200	McF_6	0	unif	wholeT_0.01	J1:CBN, J1:CBN-A, J1:DiP, J1:DiP-A, J1:OT-A, J5:CBN, J5:CBN-A, J5:DiP, J5:DiP-A, J5:OT-A, S5:CBN, S5:CBN-A, S5:DiP, S5:DiP-A
378	Yes	7	7-A	200	McF_6	0	unif	wholeT_0.5	J1:CBN, J1:CBN-A, J1:DiP, J1:DiP-A, J5:CBN, J5:CBN-A, J5:DiP, J5:DiP-A, S1:DiP, S1:DiP-A, S5:CBN, S5:CBN-A, S5:DiP, S5:DiP-A

Table 16: *(continued)*

	Conjunction	Drivers	Tree	S.Size	Model	sh	S.Time	S.Type	Best method(s)
379	Yes	7	7-A	200	McF_6	Inf	last	singleC	J1:DiP, J1:DiP-A, J5:DiP, J5:DiP-A, J5:OT, J5:OT-A, S1:DiP, S5:DiP, S5:DiP-A, S5:OT, S5:OT-A
380	Yes	7	7-A	200	McF_6	Inf	last	wholeT_0.01	J1:DiP, J5:DiP, J5:OT, J5:OT-A, S1:DiP, S5:DiP, S5:OT, S5:OT-A
381	Yes	7	7-A	200	McF_6	Inf	last	wholeT_0.5	J1:DiP, J1:DiP-A, J1:OT, J1:OT-A, J5:DiP, J5:DiP-A, J5:OT, J5:OT-A, S1:DiP, S5:DiP, S5:DiP-A, S5:OT, S5:OT-A
382	Yes	7	7-A	200	McF_6	Inf	unif	singleC	J1:CBN, J1:CBN-A, J1:DiP, J1:DiP-A, J5:CBN, J5:CBN-A, J5:DiP, J5:DiP-A, S1:DiP, S1:DiP-A, S5:CBN, S5:CBN-A, S5:DiP, S5:DiP-A
383	Yes	7	7-A	200	McF_6	Inf	unif	wholeT_0.01	J1:DiP, J1:DiP-A, J5:CBN, J5:CBN-A, J5:DiP, J5:DiP-A, S1:DiP, S1:DiP-A, S5:CBN, S5:CBN-A, S5:DiP, S5:DiP-A
384	Yes	7	7-A	200	McF_6	Inf	unif	wholeT_0.5	J1:CBN, J1:CBN-A, J1:DiP, J1:DiP-A, J5:CBN, J5:CBN-A, J5:DiP, J5:DiP-A, S1:DiP, S1:DiP-A, S5:CBN, S5:DiP, S5:DiP-A
385	Yes	7	7-A	100	Bozic	0	last	singleC	J1:DiP, J1:DiP-A, J5:CBN, J5:CBN-A, J5:DiP, J5:DiP-A, J5:OT, J5:OT-A, S1:DiP, S1:DiP-A, S5:CBN, S5:DiP, S5:DiP-A, S5:OT, S5:OT-A
386	Yes	7	7-A	100	Bozic	0	last	wholeT_0.01	J1:DiP, J1:DiP-A, J5:CBN, J5:CBN-A, J5:DiP, J5:DiP-A, J5:OT, J5:OT-A, S1:DiP, S1:DiP-A, S5:CBN, S5:CBN-A, S5:DiP, S5:DiP-A, S5:OT, S5:OT-A
387	Yes	7	7-A	100	Bozic	0	last	wholeT_0.5	J1:DiP, J1:DiP-A, J5:CBN, J5:CBN-A, J5:DiP, J5:DiP-A, J5:OT, J5:OT-A, S1:DiP, S1:DiP-A, S5:DiP, S5:DiP-A, S5:OT, S5:OT-A
388	Yes	7	7-A	100	Bozic	0	unif	singleC	J1:CBN, J1:CBN-A, J1:DiP, J1:DiP-A, J1:OT, J1:OT-A, J5:CBN, J5:CBN-A, J5:DiP, J5:DiP-A, J5:OT, J5:OT-A, S1:DiP-A, S5:CBN, S5:CBN-A, S5:DiP, S5:DiP-A, S5:OT, S5:OT-A
389	Yes	7	7-A	100	Bozic	0	unif	wholeT_0.01	J5:CBN, J5:CBN-A, J5:DiP, J5:DiP-A, J5:OT, J5:OT-A

Table 16: *(continued)*

	Conjunction	Drivers	Tree	S.Size	Model	sh	S.Time	S.Type	Best method(s)
390	Yes	7	7-A	100	Bozic	0	unif	wholeT_0.5	J1:CBN, J1:CBN-A, J1:DiP, J1:DiP-A, J1:OT, J1:OT-A, J5:CBN, J5:CBN-A, J5:DiP, J5:DiP-A, J5:OT, J5:OT-A, S1:DiP, S1:DiP-A, S5:CBN, S5:CBN-A, S5:DiP, S5:DiP-A, S5:OT, S5:OT-A
391	Yes	7	7-A	100	Bozic	Inf	last	singleC	J1:DiP, J1:DiP-A, J5:DiP, J5:DiP-A, J5:OT, J5:OT-A, S1:DiP, S1:DiP-A, S5:DiP, S5:DiP-A
392	Yes	7	7-A	100	Bozic	Inf	last	wholeT_0.01	J5:CBN, J5:DiP, J5:DiP-A
393	Yes	7	7-A	100	Bozic	Inf	last	wholeT_0.5	J1:DiP, J1:DiP-A, J5:DiP, J5:DiP-A, J5:OT, J5:OT-A, S1:DiP, S1:DiP-A, S5:DiP, S5:DiP-A
394	Yes	7	7-A	100	Bozic	Inf	unif	singleC	J5:CBN, J5:CBN-A, J5:DiP, J5:DiP-A, J5:OT, J5:OT-A, S5:CBN, S5:CBN-A, S5:DiP, S5:DiP-A
395	Yes	7	7-A	100	Bozic	Inf	unif	wholeT_0.01	J5:CBN, J5:CBN-A, J5:OT, J5:OT-A, S5:CBN, S5:CBN-A, S5:OT, S5:OT-A
396	Yes	7	7-A	100	Bozic	Inf	unif	wholeT_0.5	J5:CBN, J5:CBN-A, J5:DiP, J5:DiP-A, J5:OT, J5:OT-A, S5:CBN, S5:CBN-A, S5:DiP, S5:DiP-A, S5:OT, S5:OT-A
397	Yes	7	7-A	100	exp	0	last	singleC	J1:CBN, J1:CBN-A, J1:DiP, J1:DiP-A, J1:OT, J1:OT-A, J5:CBN, J5:CBN-A, J5:DiP, J5:DiP-A, J5:OT, J5:OT-A, S1:DiP, S1:DiP-A, S1:OT, S1:OT-A, S5:CBN, S5:CBN-A, S5:DiP, S5:DiP-A, S5:OT, S5:OT-A
398	Yes	7	7-A	100	exp	0	last	wholeT_0.01	J1:CBN, J1:CBN-A, J1:DiP, J1:DiP-A, J5:CBN, J5:CBN-A, J5:DiP, J5:DiP-A, J5:OT, J5:OT-A, S1:DiP, S1:DiP-A, S5:CBN, S5:DiP, S5:DiP-A
399	Yes	7	7-A	100	exp	0	last	wholeT_0.5	J1:CBN, J1:CBN-A, J1:DiP-A, J1:OT, J5:CBN, J5:DiP, J5:DiP-A, J5:OT, J5:OT-A, S1:DiP, S1:DiP-A, S1:OT, S5:CBN, S5:CBN-A, S5:DiP, S5:DiP-A, S5:OT, S5:OT-A

Table 16: *(continued)*

	Conjunction	Drivers	Tree	S.Size	Model	sh	S.Time	S.Type	Best method(s)
400	Yes	7	7-A	100	exp	0	unif	singleC	J1:CBN, J1:CBN-A, J1:DiP, J1:DiP-A, J1:OT, J1:OT-A, J5:CBN, J5:CBN-A, J5:DiP, J5:DiP-A, J5:OT, J5:OT-A, S1:DiP, S1:DiP-A, S1:OT, S1:OT-A, S5:CBN, S5:CBN-A, S5:DiP, S5:DiP-A, S5:OT, S5:OT-A
401	Yes	7	7-A	100	exp	0	unif	wholeT_0.01	J1:DiP, J1:DiP-A, J5:CBN, J5:CBN-A, J5:DiP, J5:DiP-A, J5:OT, J5:OT-A, S1:DiP, S1:DiP-A, S5:CBN, S5:CBN-A, S5:DiP, S5:DiP-A, S5:OT, S5:OT-A
402	Yes	7	7-A	100	exp	0	unif	wholeT_0.5	J1:CBN, J1:CBN-A, J1:DiP, J1:DiP-A, J1:OT, J1:OT-A, J5:CBN, J5:DiP, J5:DiP-A, J5:OT, J5:OT-A, S1:CBN, S1:CBN-A, S1:DiP, S1:DiP-A, S1:OT, S1:OT-A, S5:CBN, S5:CBN-A, S5:DiP, S5:DiP-A, S5:OT, S5:OT-A
403	Yes	7	7-A	100	exp	Inf	last	singleC	J1:DiP, J1:DiP-A, J1:OT, J1:OT-A, J5:CBN, J5:CBN-A, J5:DiP, J5:DiP-A, J5:OT, J5:OT-A, S1:DiP, S1:DiP-A, S5:DiP, S5:DiP-A, S5:OT, S5:OT-A
404	Yes	7	7-A	100	exp	Inf	last	wholeT_0.01	J5:DiP, J5:DiP-A, S5:DiP
405	Yes	7	7-A	100	exp	Inf	last	wholeT_0.5	J1:DiP, J1:DiP-A, J1:OT, J1:OT-A, J5:CBN, J5:CBN-A, J5:DiP, J5:DiP-A, J5:OT, J5:OT-A, S1:DiP, S1:DiP-A, S5:DiP, S5:DiP-A, S5:OT, S5:OT-A
406	Yes	7	7-A	100	exp	Inf	unif	singleC	J5:CBN, J5:CBN-A, J5:DiP, J5:DiP-A, J5:OT, J5:OT-A, S1:DiP, S5:CBN, S5:CBN-A, S5:DiP, S5:DiP-A, S5:OT, S5:OT-A
407	Yes	7	7-A	100	exp	Inf	unif	wholeT_0.01	J5:CBN, J5:CBN-A, J5:DiP, J5:OT, J5:OT-A, S5:CBN, S5:OT, S5:OT-A
408	Yes	7	7-A	100	exp	Inf	unif	wholeT_0.5	J1:DiP, J1:DiP-A, J5:CBN, J5:CBN-A, J5:DiP, J5:DiP-A, J5:OT, J5:OT-A, S1:DiP, S5:CBN, S5:CBN-A, S5:DiP, S5:DiP-A, S5:OT, S5:OT-A
409	Yes	7	7-A	100	McF_4	0	last	singleC	J1:DiP, J1:DiP-A, J1:OT-A, J5:DiP, J5:DiP-A, J5:OT, J5:OT-A, S1:DiP, S1:DiP-A, S5:DiP, S5:DiP-A, S5:OT, S5:OT-A

Table 16: *(continued)*

	Conjunction	Drivers	Tree	S.Size	Model	sh	S.Time	S.Type	Best method(s)
410	Yes	7	7-A	100	McF_4	0	last	wholeT_0.01	J1:DiP, J1:DiP-A, J5:DiP, J5:DiP-A, J5:OT, J5:OT-A, S1:DiP, S5:DiP, S5:DiP-A, S5:OT, S5:OT-A
411	Yes	7	7-A	100	McF_4	0	last	wholeT_0.5	J1:DiP, J1:DiP-A, J1:OT, J1:OT-A, J5:DiP, J5:DiP-A, J5:OT, J5:OT-A, S1:DiP, S1:DiP-A, S5:DiP, S5:DiP-A, S5:OT, S5:OT-A
412	Yes	7	7-A	100	McF_4	0	unif	singleC	J1:CBN-A, J1:DiP, J1:DiP-A, J5:CBN, J5:CBN-A, J5:DiP, J5:DiP-A, J5:OT, J5:OT-A, S1:DiP, S1:DiP-A, S5:CBN, S5:CBN-A, S5:DiP, S5:DiP-A, S5:OT, S5:OT-A
413	Yes	7	7-A	100	McF_4	0	unif	wholeT_0.01	J1:DiP, J5:CBN, J5:CBN-A, J5:DiP, J5:DiP-A, J5:OT, J5:OT-A, S5:CBN-A, S5:DiP, S5:DiP-A
414	Yes	7	7-A	100	McF_4	0	unif	wholeT_0.5	J1:CBN, J1:CBN-A, J1:DiP, J1:DiP-A, J1:OT, J1:OT-A, J5:CBN, J5:CBN-A, J5:DiP, J5:DiP-A, J5:OT, J5:OT-A, S1:DiP, S1:DiP-A, S5:CBN-A, S5:DiP, S5:DiP-A, S5:OT, S5:OT-A
415	Yes	7	7-A	100	McF_4	Inf	last	singleC	J1:DiP, J1:DiP-A, J1:OT-A, J5:DiP, J5:DiP-A, J5:OT, J5:OT-A, S1:DiP, S1:DiP-A, S5:DiP, S5:DiP-A, S5:OT, S5:OT-A
416	Yes	7	7-A	100	McF_4	Inf	last	wholeT_0.01	J1:DiP, J1:DiP-A, J5:DiP, J5:DiP-A, J5:OT, J5:OT-A, S5:DiP, S5:DiP-A, S5:OT, S5:OT-A
417	Yes	7	7-A	100	McF_4	Inf	last	wholeT_0.5	J1:DiP, J1:DiP-A, J5:DiP, J5:DiP-A, J5:OT, J5:OT-A, S1:DiP, S1:DiP-A, S5:DiP, S5:DiP-A, S5:OT, S5:OT-A
418	Yes	7	7-A	100	McF_4	Inf	unif	singleC	J1:CBN-A, J1:DiP, J1:DiP-A, J5:CBN, J5:CBN-A, J5:DiP, J5:DiP-A, J5:OT, J5:OT-A, S1:DiP, S1:DiP-A, S5:CBN, S5:CBN-A, S5:DiP, S5:DiP-A, S5:OT, S5:OT-A
419	Yes	7	7-A	100	McF_4	Inf	unif	wholeT_0.01	J1:DiP, J5:CBN, J5:CBN-A, J5:DiP, J5:DiP-A, J5:OT, J5:OT-A, S5:CBN, S5:CBN-A, S5:DiP, S5:DiP-A

Table 16: *(continued)*

	Conjunction	Drivers	Tree	S.Size	Model	sh	S.Time	S.Type	Best method(s)
420	Yes	7	7-A	100	McF_4	Inf	unif	wholeT_0.5	J1:CBN, J1:CBN-A, J1:DiP, J1:DiP-A, J1:OT, J1:OT-A, J5:CBN, J5:CBN-A, J5:DiP, J5:DiP-A, J5:OT, J5:OT-A, S1:DiP, S1:DiP-A, S5:CBN, S5:CBN-A, S5:DiP, S5:DiP-A, S5:OT, S5:OT-A
421	Yes	7	7-A	100	McF_6	0	last	singleC	J1:DiP, J1:DiP-A, J5:DiP, J5:DiP-A, J5:OT, J5:OT-A, S1:DiP, S1:DiP-A, S5:DiP, S5:DiP-A, S5:OT, S5:OT-A
422	Yes	7	7-A	100	McF_6	0	last	wholeT_0.01	J1:DiP, J5:DiP, J5:OT, J5:OT-A, S1:DiP, S5:DiP, S5:OT, S5:OT-A
423	Yes	7	7-A	100	McF_6	0	last	wholeT_0.5	J1:DiP, J5:DiP, J5:OT, J5:OT-A, S1:DiP, S5:DiP, S5:DiP-A, S5:OT, S5:OT-A
424	Yes	7	7-A	100	McF_6	0	unif	singleC	J1:CBN, J1:DiP-A, J5:CBN, J5:CBN-A, J5:DiP, J5:DiP-A, S1:DiP, S5:CBN, S5:CBN-A, S5:DiP-A
425	Yes	7	7-A	100	McF_6	0	unif	wholeT_0.01	J1:DiP, J5:CBN, J5:CBN-A, J5:DiP, J5:DiP-A, S5:CBN, S5:CBN-A, S5:DiP, S5:DiP-A
426	Yes	7	7-A	100	McF_6	0	unif	wholeT_0.5	J1:CBN-A, J1:DiP, J5:CBN, J5:CBN-A, J5:DiP, S1:DiP, S5:CBN, S5:CBN-A, S5:DiP, S5:DiP-A
427	Yes	7	7-A	100	McF_6	Inf	last	singleC	J1:DiP-A, J5:DiP, J5:DiP-A, J5:OT, J5:OT-A, S1:DiP, S1:DiP-A, S5:DiP, S5:DiP-A, S5:OT, S5:OT-A
428	Yes	7	7-A	100	McF_6	Inf	last	wholeT_0.01	J5:DiP, J5:OT, J5:OT-A, S1:DiP, S5:DiP, S5:OT, S5:OT-A
429	Yes	7	7-A	100	McF_6	Inf	last	wholeT_0.5	J1:DiP, J5:DiP, J5:DiP-A, J5:OT, J5:OT-A, S1:DiP, S5:DiP, S5:DiP-A, S5:OT, S5:OT-A
430	Yes	7	7-A	100	McF_6	Inf	unif	singleC	J1:DiP, J1:DiP-A, J5:CBN, J5:CBN-A, J5:DiP, J5:DiP-A, S1:DiP, S1:DiP-A, S5:CBN, S5:CBN-A, S5:DiP, S5:DiP-A
431	Yes	7	7-A	100	McF_6	Inf	unif	wholeT_0.01	J1:DiP, J1:DiP-A, J5:CBN, J5:CBN-A, J5:DiP, J5:DiP-A, S1:DiP-A, S5:CBN, S5:CBN-A, S5:DiP, S5:DiP-A
432	Yes	7	7-A	100	McF_6	Inf	unif	wholeT_0.5	J1:CBN, J1:CBN-A, J1:DiP, J1:DiP-A, J5:CBN, J5:CBN-A, J5:DiP, J5:DiP-A, S1:DiP, S1:DiP-A, S5:CBN, S5:CBN-A, S5:DiP, S5:DiP-A

Table 16: *(continued)*

	Conjunction	Drivers	Tree	S.Size	Model	sh	S.Time	S.Type	Best method(s)
433	No	11	11-B	1000	Bozic	0	last	singleC	J1:DiP, J1:DiP-A, J1:OT, J1:OT-A, J5:DiP, J5:DiP-A, J5:OT, J5:OT-A, S1:OT, S1:OT-A, S5:DiP, S5:DiP-A, S5:OT, S5:OT-A
434	No	11	11-B	1000	Bozic	0	last	wholeT_0.01	J1:DiP, J1:DiP-A, J5:DiP, J5:DiP-A, S1:DiP, S1:DiP-A, S5:DiP, S5:DiP-A
435	No	11	11-B	1000	Bozic	0	last	wholeT_0.5	J1:DiP, J1:DiP-A, J1:OT, J1:OT-A, J5:DiP-A, J5:OT, J5:OT-A, S1:OT, S1:OT-A, S5:DiP, S5:DiP-A, S5:OT, S5:OT-A
436	No	11	11-B	1000	Bozic	0	unif	singleC	J1:DiP, J1:DiP-A, J1:OT, J1:OT-A, J5:CBN, J5:CBN-A, J5:DiP, J5:DiP-A, J5:OT, S1:DiP, S1:DiP-A, S1:OT, S1:OT-A, S5:DiP, S5:DiP-A, S5:OT, S5:OT-A
437	No	11	11-B	1000	Bozic	0	unif	wholeT_0.01	J5:DiP, J5:DiP-A, J5:OT, J5:OT-A, S5:OT, S5:OT-A
438	No	11	11-B	1000	Bozic	0	unif	wholeT_0.5	J1:CBN, J1:CBN-A, J1:DiP-A, J1:OT, J1:OT-A, J5:CBN, J5:CBN-A, J5:DiP, J5:DiP-A, J5:OT, J5:OT-A, S1:DiP, S1:DiP-A, S1:OT, S1:OT-A, S5:DiP, S5:DiP-A, S5:OT, S5:OT-A
439	No	11	11-B	1000	Bozic	Inf	last	singleC	J1:DiP, J1:DiP-A, J1:OT, J1:OT-A, J5:DiP, J5:DiP-A, J5:OT, J5:OT-A, S5:DiP, S5:DiP-A, S5:OT, S5:OT-A
440	No	11	11-B	1000	Bozic	Inf	last	wholeT_0.01	J5:DiP, J5:DiP-A, J5:OT, J5:OT-A
441	No	11	11-B	1000	Bozic	Inf	last	wholeT_0.5	J1:DiP, J1:DiP-A, J1:OT, J1:OT-A, J5:DiP, J5:DiP-A, J5:OT, J5:OT-A, S5:DiP, S5:DiP-A, S5:OT, S5:OT-A
442	No	11	11-B	1000	Bozic	Inf	unif	singleC	J1:DiP, J1:DiP-A, J1:OT, J1:OT-A, J5:DiP, J5:DiP-A, J5:OT, J5:OT-A, S5:DiP, S5:DiP-A, S5:OT, S5:OT-A
443	No	11	11-B	1000	Bozic	Inf	unif	wholeT_0.01	J5:DiP, J5:DiP-A, J5:OT, J5:OT-A
444	No	11	11-B	1000	Bozic	Inf	unif	wholeT_0.5	J1:DiP, J1:DiP-A, J1:OT, J1:OT-A, J5:DiP, J5:DiP-A, J5:OT, J5:OT-A, S5:DiP, S5:DiP-A, S5:OT, S5:OT-A
445	No	11	11-B	1000	exp	0	last	singleC	J1:DiP, J1:DiP-A, J1:OT, J1:OT-A, J5:CBN, J5:CBN-A, J5:DiP, J5:DiP-A, J5:OT, J5:OT-A, S1:DiP-A, S1:OT-A, S5:DiP-A, S5:OT, S5:OT-A

Table 16: *(continued)*

	Conjunction	Drivers	Tree	S.Size	Model	sh	S.Time	S.Type	Best method(s)
446	No	11	11-B	1000	exp	0	last	wholeT_0.01	J1:DiP, J1:DiP-A, J1:OT, J1:OT-A, J5:DiP, J5:DiP-A, J5:OT, J5:OT-A, S1:DiP-A, S1:OT, S1:OT-A, S5:DiP-A, S5:OT, S5:OT-A
447	No	11	11-B	1000	exp	0	last	wholeT_0.5	J1:DiP, J1:DiP-A, J1:OT, J1:OT-A, J5:CBN, J5:CBN-A, J5:DiP, J5:DiP-A, J5:OT, J5:OT-A, S1:DiP, S1:DiP-A, S1:OT, S1:OT-A, S5:DiP, S5:DiP-A, S5:OT, S5:OT-A
448	No	11	11-B	1000	exp	0	unif	singleC	J1:CBN, J1:CBN-A, J1:DiP, J1:DiP-A, J1:OT, J1:OT-A, J5:CBN, J5:CBN-A, J5:DiP, J5:DiP-A, J5:OT, J5:OT-A, S1:DiP, S1:DiP-A, S1:OT, S1:OT-A, S5:DiP, S5:DiP-A, S5:OT, S5:OT-A
449	No	11	11-B	1000	exp	0	unif	wholeT_0.01	J1:DiP, J1:DiP-A, J1:OT, J1:OT-A, J5:CBN, J5:CBN-A, J5:DiP, J5:DiP-A, J5:OT, J5:OT-A, S1:DiP-A, S1:OT-A, S5:DiP, S5:DiP-A, S5:OT, S5:OT-A
450	No	11	11-B	1000	exp	0	unif	wholeT_0.5	J1:CBN, J1:CBN-A, J1:DiP, J1:DiP-A, J1:OT, J1:OT-A, J5:CBN, J5:CBN-A, J5:DiP, J5:DiP-A, J5:OT, J5:OT-A, S1:DiP, S1:DiP-A, S1:OT, S1:OT-A, S5:DiP, S5:DiP-A, S5:OT, S5:OT-A
451	No	11	11-B	1000	exp	Inf	last	singleC	J1:DiP, J1:DiP-A, J1:OT, J1:OT-A, J5:DiP, J5:DiP-A, J5:OT, J5:OT-A, S1:DiP, S5:DiP, S5:DiP-A, S5:OT, S5:OT-A
452	No	11	11-B	1000	exp	Inf	last	wholeT_0.01	J5:DiP, J5:DiP-A, J5:OT, S5:DiP, S5:DiP-A, S5:OT-A
453	No	11	11-B	1000	exp	Inf	last	wholeT_0.5	J1:DiP, J1:DiP-A, J1:OT, J1:OT-A, J5:DiP, J5:DiP-A, J5:OT, J5:OT-A, S5:DiP, S5:DiP-A, S5:OT, S5:OT-A
454	No	11	11-B	1000	exp	Inf	unif	singleC	J1:DiP, J1:DiP-A, J1:OT, J1:OT-A, J5:CBN, J5:CBN-A, J5:DiP, J5:DiP-A, J5:OT, J5:OT-A, S5:DiP, S5:OT, S5:OT-A
455	No	11	11-B	1000	exp	Inf	unif	wholeT_0.01	J5:DiP, J5:DiP-A, J5:OT, J5:OT-A, S5:DiP, S5:DiP-A, S5:OT-A

Table 16: *(continued)*

	Conjunction	Drivers	Tree	S.Size	Model	sh	S.Time	S.Type	Best method(s)
456	No	11	11-B	1000	exp	Inf	unif	wholeT_0.5	J1:DiP, J1:DiP-A, J1:OT, J1:OT-A, J5:CBN, J5:CBN-A, J5:DiP, J5:DiP-A, J5:OT, J5:OT-A, S5:DiP, S5:DiP-A, S5:OT, S5:OT-A
457	No	11	11-B	1000	McF_4	0	last	singleC	J1:CBN, J1:DiP, J1:DiP-A, J1:OT, J1:OT-A, J5:CBN, J5:DiP, J5:DiP-A, J5:OT, J5:OT-A, S1:DiP-A, S1:OT, S1:OT-A, S5:DiP, S5:DiP-A, S5:OT, S5:OT-A
458	No	11	11-B	1000	McF_4	0	last	wholeT_0.01	J1:DiP, J1:DiP-A, J1:OT, J1:OT-A, J5:CBN, J5:DiP, J5:DiP-A, J5:OT, J5:OT-A, S1:DiP, S1:DiP-A, S5:DiP, S5:DiP-A, S5:OT, S5:OT-A
459	No	11	11-B	1000	McF_4	0	last	wholeT_0.5	J1:CBN, J1:DiP, J1:DiP-A, J1:OT, J1:OT-A, J5:DiP, J5:DiP-A, J5:OT, J5:OT-A, S1:DiP-A, S1:OT, S1:OT-A, S5:DiP, S5:DiP-A, S5:OT, S5:OT-A
460	No	11	11-B	1000	McF_4	0	unif	singleC	J1:CBN, J1:CBN-A, J1:DiP, J1:DiP-A, J1:OT, J1:OT-A, J5:DiP, J5:DiP-A, J5:OT, J5:OT-A, S1:DiP, S1:DiP-A, S1:OT, S1:OT-A, S5:DiP, S5:DiP-A, S5:OT, S5:OT-A
461	No	11	11-B	1000	McF_4	0	unif	wholeT_0.01	J1:CBN-A, J1:DiP, J1:DiP-A, J1:OT, J1:OT-A, J5:DiP, J5:DiP-A, J5:OT, J5:OT-A, S5:DiP, S5:DiP-A, S5:OT, S5:OT-A
462	No	11	11-B	1000	McF_4	0	unif	wholeT_0.5	J1:CBN-A, J1:DiP, J1:DiP-A, J1:OT, J1:OT-A, J5:DiP, J5:DiP-A, J5:OT, J5:OT-A, S1:DiP, S1:DiP-A, S1:OT, S1:OT-A, S5:CBN, S5:DiP, S5:DiP-A, S5:OT, S5:OT-A
463	No	11	11-B	1000	McF_4	Inf	last	singleC	J1:DiP, J1:DiP-A, J1:OT, J1:OT-A, J5:DiP, J5:DiP-A, J5:OT, J5:OT-A, S5:DiP, S5:DiP-A, S5:OT, S5:OT-A
464	No	11	11-B	1000	McF_4	Inf	last	wholeT_0.01	J1:DiP, J1:DiP-A, J1:OT, J1:OT-A, J5:DiP, J5:DiP-A, J5:OT, J5:OT-A, S1:DiP, S1:DiP-A, S5:DiP, S5:DiP-A, S5:OT, S5:OT-A

Table 16: *(continued)*

	Conjunction	Drivers	Tree	S.Size	Model	sh	S.Time	S.Type	Best method(s)
465	No	11	11-B	1000	McF_4	Inf	last	wholeT_0.5	J1:DiP, J1:DiP-A, J1:OT, J1:OT-A, J5:DiP, J5:DiP-A, J5:OT, J5:OT-A, S1:DiP-A, S1:OT, S1:OT-A, S5:DiP, S5:DiP-A, S5:OT, S5:OT-A
466	No	11	11-B	1000	McF_4	Inf	unif	singleC	J1:CBN-A, J1:DiP, J1:DiP-A, J1:OT, J1:OT-A, J5:DiP, J5:DiP-A, J5:OT, J5:OT-A, S1:DiP, S1:DiP-A, S1:OT-A, S5:DiP, S5:DiP-A, S5:OT, S5:OT-A
467	No	11	11-B	1000	McF_4	Inf	unif	wholeT_0.01	J1:DiP, J1:DiP-A, J1:OT, J1:OT-A, J5:DiP, J5:DiP-A, J5:OT, J5:OT-A, S5:DiP, S5:DiP-A, S5:OT, S5:OT-A
468	No	11	11-B	1000	McF_4	Inf	unif	wholeT_0.5	J1:DiP, J1:DiP-A, J1:OT, J1:OT-A, J5:DiP, J5:DiP-A, J5:OT, J5:OT-A, S1:DiP, S1:DiP-A, S1:OT, S1:OT-A, S5:DiP, S5:DiP-A, S5:OT, S5:OT-A
469	No	11	11-B	1000	McF_6	0	last	singleC	J1:DiP, J1:OT, J5:DiP, J5:OT, S1:DiP, S5:DiP, S5:OT
470	No	11	11-B	1000	McF_6	0	last	wholeT_0.01	J1:DiP, J1:OT, J5:DiP, J5:OT, S1:DiP, S5:DiP, S5:OT
471	No	11	11-B	1000	McF_6	0	last	wholeT_0.5	J1:DiP, J1:OT, J5:DiP, J5:OT, S1:DiP, S1:OT, S5:DiP, S5:OT
472	No	11	11-B	1000	McF_6	0	unif	singleC	J1:CBN, J1:CBN-A, J1:DiP, J1:DiP-A, J1:OT, J1:OT-A, J5:CBN-A, J5:DiP, J5:DiP-A, J5:OT, J5:OT-A, S1:DiP, S1:DiP-A, S5:CBN-A, S5:DiP, S5:DiP-A, S5:OT, S5:OT-A
473	No	11	11-B	1000	McF_6	0	unif	wholeT_0.01	J1:CBN, J1:CBN-A, J1:DiP, J1:DiP-A, J1:OT, J1:OT-A, J5:CBN-A, J5:DiP, J5:DiP-A, J5:OT, J5:OT-A, S1:DiP, S1:DiP-A, S5:DiP, S5:DiP-A, S5:OT, S5:OT-A
474	No	11	11-B	1000	McF_6	0	unif	wholeT_0.5	J1:CBN, J1:CBN-A, J1:DiP, J1:DiP-A, J1:OT, J1:OT-A, J5:CBN, J5:CBN-A, J5:DiP, J5:DiP-A, J5:OT, J5:OT-A, S1:DiP, S1:DiP-A, S5:CBN, S5:DiP, S5:DiP-A, S5:OT, S5:OT-A
475	No	11	11-B	1000	McF_6	Inf	last	singleC	J1:DiP, J1:OT, J5:DiP, J5:OT, S1:DiP, S5:DiP, S5:OT
476	No	11	11-B	1000	McF_6	Inf	last	wholeT_0.01	J1:DiP, J1:OT, J5:DiP, J5:OT, S1:DiP, S5:DiP, S5:OT

Table 16: *(continued)*

	Conjunction	Drivers	Tree	S.Size	Model	sh	S.Time	S.Type	Best method(s)
477	No	11	11-B	1000	McF_6	Inf	last	wholeT_0.5	J1:DiP, J1:OT, J5:DiP, J5:OT, S1:DiP, S5:DiP, S5:OT
478	No	11	11-B	1000	McF_6	Inf	unif	singleC	J1:CBN-A, J1:DiP, J1:DiP-A, J1:OT, J5:CBN-A, J5:DiP, J5:DiP-A, J5:OT, J5:OT-A, S1:DiP, S1:DiP-A, S5:CBN, S5:CBN-A, S5:DiP, S5:DiP-A, S5:OT, S5:OT-A
479	No	11	11-B	1000	McF_6	Inf	unif	wholeT_0.01	J1:CBN-A, J1:DiP, J1:DiP-A, J1:OT, J5:CBN-A, J5:DiP, J5:DiP-A, J5:OT, J5:OT-A, S1:DiP, S1:DiP-A, S5:CBN, S5:DiP, S5:DiP-A, S5:OT, S5:OT-A
480	No	11	11-B	1000	McF_6	Inf	unif	wholeT_0.5	J1:CBN, J1:CBN-A, J1:DiP, J1:DiP-A, J1:OT, J1:OT-A, J5:DiP, J5:DiP-A, J5:OT, S1:DiP, S1:DiP-A, S5:CBN, S5:DiP, S5:DiP-A, S5:OT
481	No	11	11-B	200	Bozic	0	last	singleC	J1:DiP, J1:DiP-A, J1:OT, J1:OT-A, J5:DiP, J5:DiP-A, J5:OT, J5:OT-A, S1:DiP, S5:DiP, S5:DiP-A, S5:OT, S5:OT-A
482	No	11	11-B	200	Bozic	0	last	wholeT_0.01	J1:DiP, J5:DiP
483	No	11	11-B	200	Bozic	0	last	wholeT_0.5	J1:DiP, J1:DiP-A, J1:OT, J1:OT-A, J5:DiP, J5:DiP-A, J5:OT, J5:OT-A, S1:DiP, S1:DiP-A, S5:DiP, S5:DiP-A, S5:OT, S5:OT-A
484	No	11	11-B	200	Bozic	0	unif	singleC	J1:DiP, J1:DiP-A, J1:OT, J1:OT-A, J5:CBN, J5:CBN-A, J5:DiP, J5:DiP-A, J5:OT, J5:OT-A, S1:DiP, S1:DiP-A, S1:OT, S1:OT-A, S5:DiP, S5:DiP-A, S5:OT, S5:OT-A
485	No	11	11-B	200	Bozic	0	unif	wholeT_0.01	J5:DiP, J5:DiP-A, J5:OT, J5:OT-A, S5:OT-A
486	No	11	11-B	200	Bozic	0	unif	wholeT_0.5	J1:CBN-A, J1:DiP, J1:DiP-A, J1:OT, J1:OT-A, J5:CBN, J5:CBN-A, J5:DiP, J5:DiP-A, J5:OT-A, S1:DiP, S1:DiP-A, S1:OT, S5:DiP, S5:DiP-A, S5:OT, S5:OT-A
487	No	11	11-B	200	Bozic	Inf	last	singleC	J1:DiP, J1:DiP-A, J5:DiP, J5:DiP-A, J5:OT, J5:OT-A, S5:DiP, S5:DiP-A, S5:OT
488	No	11	11-B	200	Bozic	Inf	last	wholeT_0.01	J5:DiP, J5:DiP-A, J5:OT, J5:OT-A

Table 16: *(continued)*

	Conjunction	Drivers	Tree	S.Size	Model	sh	S.Time	S.Type	Best method(s)
489	No	11	11-B	200	Bozic	Inf	last	wholeT_0.5	J1:DiP, J1:DiP-A, J5:DiP, J5:DiP-A, J5:OT, J5:OT-A, S5:DiP, S5:DiP-A, S5:OT, S5:OT-A
490	No	11	11-B	200	Bozic	Inf	unif	singleC	J1:CBN-A, J1:DiP, J1:DiP-A, J5:DiP, J5:DiP-A, J5:OT, J5:OT-A, S5:DiP, S5:DiP-A, S5:OT, S5:OT-A
491	No	11	11-B	200	Bozic	Inf	unif	wholeT_0.01	J5:CBN-A, J5:DiP, J5:DiP-A, J5:OT, J5:OT-A
492	No	11	11-B	200	Bozic	Inf	unif	wholeT_0.5	J1:DiP, J1:OT-A, J5:DiP, J5:DiP-A, J5:OT, J5:OT-A, S5:DiP, S5:DiP-A, S5:OT, S5:OT-A
493	No	11	11-B	200	exp	0	last	singleC	J1:DiP, J1:DiP-A, J1:OT, J1:OT-A, J5:CBN, J5:CBN-A, J5:DiP, J5:DiP-A, J5:OT, J5:OT-A, S1:DiP, S1:DiP-A, S1:OT, S1:OT-A, S5:DiP, S5:DiP-A, S5:OT, S5:OT-A
494	No	11	11-B	200	exp	0	last	wholeT_0.01	J1:DiP, J1:DiP-A, J1:OT, J1:OT-A, J5:DiP, J5:DiP-A, J5:OT, J5:OT-A, S1:OT, S1:OT-A, S5:DiP, S5:DiP-A, S5:OT, S5:OT-A
495	No	11	11-B	200	exp	0	last	wholeT_0.5	J1:DiP, J1:DiP-A, J1:OT, J1:OT-A, J5:CBN, J5:CBN-A, J5:DiP, J5:DiP-A, J5:OT, J5:OT-A, S1:DiP, S1:DiP-A, S1:OT, S1:OT-A, S5:DiP, S5:DiP-A, S5:OT, S5:OT-A
496	No	11	11-B	200	exp	0	unif	singleC	J1:CBN, J1:CBN-A, J1:DiP, J1:DiP-A, J1:OT, J1:OT-A, J5:CBN, J5:CBN-A, J5:DiP, J5:DiP-A, J5:OT, J5:OT-A, S1:DiP, S1:DiP-A, S1:OT, S1:OT-A, S5:DiP, S5:DiP-A, S5:OT, S5:OT-A
497	No	11	11-B	200	exp	0	unif	wholeT_0.01	J1:DiP, J1:DiP-A, J1:OT, J1:OT-A, J5:CBN, J5:CBN-A, J5:DiP, J5:DiP-A, J5:OT, J5:OT-A, S1:DiP, S1:DiP-A, S1:OT, S1:OT-A, S5:DiP, S5:DiP-A, S5:OT, S5:OT-A
498	No	11	11-B	200	exp	0	unif	wholeT_0.5	J1:CBN, J1:CBN-A, J1:DiP, J1:DiP-A, J1:OT, J1:OT-A, J5:CBN, J5:CBN-A, J5:DiP, J5:DiP-A, J5:OT, J5:OT-A, S1:DiP, S1:DiP-A, S1:OT, S1:OT-A, S5:DiP, S5:DiP-A, S5:OT, S5:OT-A

Table 16: *(continued)*

	Conjunction	Drivers	Tree	S.Size	Model	sh	S.Time	S.Type	Best method(s)
499	No	11	11-B	200	exp	Inf	last	singleC	J1:DiP, J1:DiP-A, J1:OT-A, J5:DiP, J5:DiP-A, J5:OT, J5:OT-A, S1:DiP, S1:DiP-A, S5:DiP, S5:DiP-A, S5:OT, S5:OT-A
500	No	11	11-B	200	exp	Inf	last	wholeT_0.01	J5:DiP, J5:DiP-A, J5:OT, J5:OT-A
501	No	11	11-B	200	exp	Inf	last	wholeT_0.5	J1:DiP, J1:DiP-A, J1:OT, J1:OT-A, J5:DiP, J5:DiP-A, J5:OT, J5:OT-A, S1:DiP, S1:DiP-A, S5:DiP, S5:DiP-A, S5:OT, S5:OT-A
502	No	11	11-B	200	exp	Inf	unif	singleC	J1:DiP, J1:DiP-A, J1:OT, J1:OT-A, J5:CBN, J5:CBN-A, J5:DiP, J5:DiP-A, J5:OT-A, S5:DiP, S5:DiP-A, S5:OT, S5:OT-A
503	No	11	11-B	200	exp	Inf	unif	wholeT_0.01	J5:DiP, J5:DiP-A, J5:OT, J5:OT-A, S5:OT-A
504	No	11	11-B	200	exp	Inf	unif	wholeT_0.5	J1:DiP, J1:DiP-A, J1:OT, J1:OT-A, J5:CBN, J5:DiP, J5:DiP-A, J5:OT, J5:OT-A, S1:DiP-A, S5:DiP, S5:DiP-A, S5:OT, S5:OT-A
505	No	11	11-B	200	McF_4	0	last	singleC	J1:DiP, J1:DiP-A, J1:OT-A, J5:DiP, J5:DiP-A, J5:OT, J5:OT-A, S5:OT, S5:OT-A
506	No	11	11-B	200	McF_4	0	last	wholeT_0.01	J1:DiP, J1:DiP-A, J1:OT, J1:OT-A, J5:DiP-A, J5:OT, J5:OT-A, S1:DiP, S1:DiP-A, S5:DiP, S5:DiP-A, S5:OT, S5:OT-A
507	No	11	11-B	200	McF_4	0	last	wholeT_0.5	J1:DiP, J1:DiP-A, J1:OT, J1:OT-A, J5:DiP, J5:DiP-A, J5:OT, J5:OT-A, S5:DiP, S5:DiP-A, S5:OT, S5:OT-A
508	No	11	11-B	200	McF_4	0	unif	singleC	J1:CBN, J1:CBN-A, J1:DiP, J1:DiP-A, J1:OT, J1:OT-A, J5:DiP, J5:DiP-A, J5:OT, J5:OT-A, S5:CBN-A, S5:DiP, S5:DiP-A, S5:OT, S5:OT-A
509	No	11	11-B	200	McF_4	0	unif	wholeT_0.01	J1:CBN-A, J1:DiP, J1:DiP-A, J1:OT, J1:OT-A, J5:DiP, J5:DiP-A, J5:OT, J5:OT-A, S1:DiP-A, S5:DiP, S5:DiP-A, S5:OT, S5:OT-A

Table 16: *(continued)*

	Conjunction	Drivers	Tree	S.Size	Model	sh	S.Time	S.Type	Best method(s)
510	No	11	11-B	200	McF_4	0	unif	wholeT_0.5	J1:CBN-A, J1:DiP, J1:DiP-A, J1:OT, J1:OT-A, J5:DiP, J5:DiP-A, J5:OT, J5:OT-A, S1:DiP, S5:CBN-A, S5:DiP, S5:DiP-A, S5:OT-A
511	No	11	11-B	200	McF_4	Inf	last	singleC	J1:DiP, J1:DiP-A, J1:OT, J1:OT-A, J5:DiP, J5:DiP-A, J5:OT, J5:OT-A, S5:OT, S5:OT-A
512	No	11	11-B	200	McF_4	Inf	last	wholeT_0.01	J1:DiP, J1:DiP-A, J5:DiP, J5:DiP-A, J5:OT, J5:OT-A, S1:DiP-A, S5:DiP, S5:DiP-A, S5:OT, S5:OT-A
513	No	11	11-B	200	McF_4	Inf	last	wholeT_0.5	J1:DiP, J1:DiP-A, J1:OT, J1:OT-A, J5:DiP, J5:DiP-A, J5:OT, J5:OT-A, S1:DiP, S1:DiP-A, S5:DiP, S5:DiP-A, S5:OT, S5:OT-A
514	No	11	11-B	200	McF_4	Inf	unif	singleC	J1:CBN-A, J1:DiP, J1:DiP-A, J1:OT, J1:OT-A, J5:DiP, J5:DiP-A, J5:OT, J5:OT-A, S1:DiP, S5:CBN-A, S5:DiP, S5:OT-A
515	No	11	11-B	200	McF_4	Inf	unif	wholeT_0.01	J1:DiP, J1:DiP-A, J1:OT, J1:OT-A, J5:DiP, J5:DiP-A, J5:OT, J5:OT-A, S1:DiP-A, S5:DiP, S5:DiP-A, S5:OT, S5:OT-A
516	No	11	11-B	200	McF_4	Inf	unif	wholeT_0.5	J1:CBN-A, J1:DiP, J1:DiP-A, J1:OT, J1:OT-A, J5:DiP, J5:DiP-A, J5:OT, J5:OT-A, S1:DiP, S5:DiP, S5:DiP-A, S5:OT, S5:OT-A
517	No	11	11-B	200	McF_6	0	last	singleC	J1:DiP, J1:OT, J5:DiP, J5:OT, S1:DiP, S5:DiP, S5:OT
518	No	11	11-B	200	McF_6	0	last	wholeT_0.01	J1:DiP, J1:OT, J5:DiP, J5:OT, S1:DiP, S5:DiP, S5:OT
519	No	11	11-B	200	McF_6	0	last	wholeT_0.5	J1:DiP, J1:OT, J5:DiP, J5:OT, S1:DiP, S5:DiP, S5:OT
520	No	11	11-B	200	McF_6	0	unif	singleC	J1:CBN, J1:CBN-A, J1:DiP-A, J1:OT-A, J5:CBN, J5:CBN-A, J5:DiP, J5:DiP-A, J5:OT, J5:OT-A, S5:CBN-A, S5:OT
521	No	11	11-B	200	McF_6	0	unif	wholeT_0.01	J1:CBN, J1:CBN-A, J1:DiP, J1:DiP-A, J1:OT, J1:OT-A, J5:CBN, J5:CBN-A, J5:DiP, J5:DiP-A, J5:OT, J5:OT-A, S5:DiP, S5:DiP-A, S5:OT, S5:OT-A

Table 16: *(continued)*

	Conjunction	Drivers	Tree	S.Size	Model	sh	S.Time	S.Type	Best method(s)
522	No	11	11-B	200	McF_6	0	unif	wholeT_0.5	J1:CBN, J1:CBN-A, J1:DiP, J1:OT, J1:OT-A, J5:CBN, J5:CBN-A, J5:DiP, J5:OT, J5:OT-A, S5:OT
523	No	11	11-B	200	McF_6	Inf	last	singleC	J1:DiP, J1:OT, J5:DiP, J5:OT, S1:DiP, S5:DiP, S5:OT
524	No	11	11-B	200	McF_6	Inf	last	wholeT_0.01	J1:DiP, J5:DiP, J5:OT, S1:DiP, S5:DiP, S5:OT
525	No	11	11-B	200	McF_6	Inf	last	wholeT_0.5	J1:DiP, J1:OT, J5:DiP, J5:OT, S1:DiP, S5:DiP, S5:OT
526	No	11	11-B	200	McF_6	Inf	unif	singleC	J1:CBN-A, J1:OT, J1:OT-A, J5:CBN-A, J5:DiP, J5:DiP-A, J5:OT, J5:OT-A, S5:CBN-A, S5:OT
527	No	11	11-B	200	McF_6	Inf	unif	wholeT_0.01	J1:CBN-A, J1:DiP, J1:OT, J1:OT-A, J5:CBN-A, J5:DiP, J5:DiP-A, J5:OT, J5:OT-A, S5:OT, S5:OT-A
528	No	11	11-B	200	McF_6	Inf	unif	wholeT_0.5	J1:CBN, J1:CBN-A, J5:CBN-A, J5:DiP, J5:DiP-A, J5:OT, J5:OT-A, S5:CBN, S5:CBN-A
529	No	11	11-B	100	Bozic	0	last	singleC	J1:DiP, J1:DiP-A, J5:DiP, J5:DiP-A, J5:OT, J5:OT-A, S1:DiP, S1:DiP-A, S5:DiP, S5:DiP-A, S5:OT, S5:OT-A
530	No	11	11-B	100	Bozic	0	last	wholeT_0.01	J1:DiP, J1:DiP-A, J5:DiP, J5:DiP-A, J5:OT, J5:OT-A
531	No	11	11-B	100	Bozic	0	last	wholeT_0.5	J1:DiP, J1:DiP-A, J5:DiP, J5:DiP-A, J5:OT, J5:OT-A, S1:DiP, S1:DiP-A, S5:DiP, S5:DiP-A, S5:OT-A
532	No	11	11-B	100	Bozic	0	unif	singleC	J1:DiP, J1:DiP-A, J5:CBN, J5:CBN-A, J5:DiP, J5:DiP-A, J5:OT, J5:OT-A, S1:DiP, S1:DiP-A, S5:DiP, S5:DiP-A, S5:OT, S5:OT-A
533	No	11	11-B	100	Bozic	0	unif	wholeT_0.01	J5:DiP, J5:DiP-A, J5:OT, J5:OT-A, S5:DiP-A, S5:OT, S5:OT-A
534	No	11	11-B	100	Bozic	0	unif	wholeT_0.5	J1:DiP, J1:DiP-A, J5:CBN, J5:CBN-A, J5:DiP, J5:DiP-A, J5:OT, J5:OT-A, S1:DiP, S1:DiP-A, S5:DiP, S5:DiP-A, S5:OT, S5:OT-A
535	No	11	11-B	100	Bozic	Inf	last	singleC	J1:DiP, J5:DiP, J5:DiP-A, J5:OT, J5:OT-A, S5:DiP, S5:DiP-A
536	No	11	11-B	100	Bozic	Inf	last	wholeT_0.01	J5:DiP, J5:DiP-A, J5:OT, J5:OT-A

Table 16: *(continued)*

	Conjunction	Drivers	Tree	S.Size	Model	sh	S.Time	S.Type	Best method(s)
537	No	11	11-B	100	Bozic	Inf	last	wholeT_0.5	J1:DiP, J1:DiP-A, J5:DiP, J5:DiP-A, J5:OT, J5:OT-A, S1:DiP, S1:DiP-A, S5:DiP, S5:DiP-A
538	No	11	11-B	100	Bozic	Inf	unif	singleC	J1:DiP, J5:DiP, J5:DiP-A, J5:OT, J5:OT-A, S5:DiP, S5:DiP-A, S5:OT, S5:OT-A
539	No	11	11-B	100	Bozic	Inf	unif	wholeT_0.01	J5:DiP, J5:DiP-A, J5:OT, J5:OT-A
540	No	11	11-B	100	Bozic	Inf	unif	wholeT_0.5	J1:DiP, J1:DiP-A, J5:DiP, J5:DiP-A, J5:OT, J5:OT-A, S5:DiP, S5:DiP-A, S5:OT, S5:OT-A
541	No	11	11-B	100	exp	0	last	singleC	J1:DiP-A, J1:OT, J1:OT-A, J5:CBN, J5:CBN-A, J5:DiP, J5:DiP-A, J5:OT, J5:OT-A, S1:DiP, S1:DiP-A, S1:OT-A, S5:DiP, S5:DiP-A, S5:OT, S5:OT-A
542	No	11	11-B	100	exp	0	last	wholeT_0.01	J1:DiP, J1:DiP-A, J1:OT, J1:OT-A, J5:DiP, J5:DiP-A, J5:OT, J5:OT-A, S1:DiP, S1:DiP-A, S5:DiP, S5:DiP-A, S5:OT
543	No	11	11-B	100	exp	0	last	wholeT_0.5	J1:DiP, J1:DiP-A, J1:OT, J1:OT-A, J5:CBN, J5:CBN-A, J5:DiP, J5:DiP-A, J5:OT, J5:OT-A, S1:DiP, S1:DiP-A, S1:OT-A, S5:DiP, S5:DiP-A, S5:OT, S5:OT-A
544	No	11	11-B	100	exp	0	unif	singleC	J1:DiP, J1:DiP-A, J1:OT-A, J5:CBN, J5:CBN-A, J5:DiP, J5:DiP-A, J5:OT, J5:OT-A, S1:DiP, S1:DiP-A, S5:DiP, S5:DiP-A, S5:OT, S5:OT-A
545	No	11	11-B	100	exp	0	unif	wholeT_0.01	J1:DiP, J1:DiP-A, J1:OT, J5:CBN, J5:CBN-A, J5:DiP, J5:DiP-A, J5:OT, J5:OT-A, S1:DiP, S1:DiP-A, S5:DiP, S5:DiP-A, S5:OT, S5:OT-A
546	No	11	11-B	100	exp	0	unif	wholeT_0.5	J1:CBN, J1:CBN-A, J1:DiP, J1:OT, J1:OT-A, J5:CBN, J5:CBN-A, J5:DiP, J5:DiP-A, J5:OT, J5:OT-A, S1:DiP, S1:DiP-A, S1:OT, S5:DiP, S5:DiP-A, S5:OT, S5:OT-A
547	No	11	11-B	100	exp	Inf	last	singleC	J1:DiP, J1:DiP-A, J5:DiP, J5:DiP-A, J5:OT, J5:OT-A, S1:DiP, S1:DiP-A, S5:DiP, S5:DiP-A, S5:OT, S5:OT-A
548	No	11	11-B	100	exp	Inf	last	wholeT_0.01	J5:CBN-A, J5:DiP, J5:DiP-A, J5:OT, J5:OT-A

Table 16: *(continued)*

	Conjunction	Drivers	Tree	S.Size	Model	sh	S.Time	S.Type	Best method(s)
549	No	11	11-B	100	exp	Inf	last	wholeT_0.5	J1:DiP, J1:DiP-A, J5:DiP, J5:DiP-A, J5:OT, J5:OT-A, S1:DiP, S1:DiP-A, S5:DiP, S5:DiP-A, S5:OT, S5:OT-A
550	No	11	11-B	100	exp	Inf	unif	singleC	J1:DiP, J1:DiP-A, J5:DiP, J5:DiP-A, J5:OT, J5:OT-A, S1:DiP, S5:DiP, S5:DiP-A, S5:OT, S5:OT-A
551	No	11	11-B	100	exp	Inf	unif	wholeT_0.01	J5:DiP, J5:DiP-A, J5:OT, J5:OT-A, S5:DiP, S5:DiP-A, S5:OT, S5:OT-A
552	No	11	11-B	100	exp	Inf	unif	wholeT_0.5	J1:DiP, J1:DiP-A, J5:CBN, J5:CBN-A, J5:DiP, J5:DiP-A, J5:OT, J5:OT-A, S1:DiP-A, S5:DiP, S5:DiP-A, S5:OT, S5:OT-A
553	No	11	11-B	100	McF_4	0	last	singleC	J1:DiP, J1:DiP-A, J5:DiP, J5:DiP-A, J5:OT, J5:OT-A, S1:DiP, S1:DiP-A, S5:DiP, S5:DiP-A, S5:OT, S5:OT-A
554	No	11	11-B	100	McF_4	0	last	wholeT_0.01	J1:DiP, J1:DiP-A, J5:DiP, J5:DiP-A, J5:OT, J5:OT-A, S1:DiP, S1:DiP-A, S5:DiP, S5:DiP-A, S5:OT
555	No	11	11-B	100	McF_4	0	last	wholeT_0.5	J1:DiP, J1:DiP-A, J5:DiP, J5:DiP-A, J5:OT, J5:OT-A, S1:DiP, S1:DiP-A, S5:DiP, S5:DiP-A, S5:OT, S5:OT-A
556	No	11	11-B	100	McF_4	0	unif	singleC	J1:DiP, J1:DiP-A, J1:OT, J1:OT-A, J5:DiP, J5:DiP-A, J5:OT, J5:OT-A, S1:DiP, S1:DiP-A, S5:DiP, S5:DiP-A, S5:OT, S5:OT-A
557	No	11	11-B	100	McF_4	0	unif	wholeT_0.01	J1:DiP, J1:DiP-A, J5:DiP, J5:DiP-A, J5:OT, J5:OT-A, S5:DiP, S5:DiP-A, S5:OT-A
558	No	11	11-B	100	McF_4	0	unif	wholeT_0.5	J1:CBN-A, J1:DiP, J1:DiP-A, J1:OT, J1:OT-A, J5:DiP-A, J5:OT, J5:OT-A, S1:DiP, S1:DiP-A, S5:DiP, S5:DiP-A, S5:OT, S5:OT-A
559	No	11	11-B	100	McF_4	Inf	last	singleC	J1:DiP, J1:DiP-A, J5:DiP, J5:DiP-A, J5:OT, J5:OT-A, S1:DiP, S5:DiP, S5:DiP-A, S5:OT
560	No	11	11-B	100	McF_4	Inf	last	wholeT_0.01	J1:DiP, J5:DiP, J5:DiP-A, J5:OT, J5:OT-A, S1:DiP-A, S5:DiP, S5:DiP-A, S5:OT-A
561	No	11	11-B	100	McF_4	Inf	last	wholeT_0.5	J1:DiP, J1:DiP-A, J5:DiP, J5:DiP-A, J5:OT, J5:OT-A, S1:DiP, S5:DiP, S5:DiP-A, S5:OT, S5:OT-A

Table 16: *(continued)*

	Conjunction	Drivers	Tree	S.Size	Model	sh	S.Time	S.Type	Best method(s)
562	No	11	11-B	100	McF_4	Inf	unif	singleC	J1:DiP, J1:DiP-A, J1:OT, J5:DiP, J5:DiP-A, J5:OT, J5:OT-A, S1:DiP, S1:DiP-A, S5:DiP, S5:DiP-A, S5:OT, S5:OT-A
563	No	11	11-B	100	McF_4	Inf	unif	wholeT_0.01	J1:DiP, J1:DiP-A, J5:DiP, J5:DiP-A, J5:OT, J5:OT-A, S5:DiP, S5:DiP-A, S5:OT-A
564	No	11	11-B	100	McF_4	Inf	unif	wholeT_0.5	J1:DiP, J1:DiP-A, J5:DiP, J5:DiP-A, J5:OT, S5:DiP-A
565	No	11	11-B	100	McF_6	0	last	singleC	J1:DiP, J1:OT, J5:DiP, J5:OT, S1:DiP
566	No	11	11-B	100	McF_6	0	last	wholeT_0.01	J1:DiP, J5:DiP, J5:OT, S5:DiP
567	No	11	11-B	100	McF_6	0	last	wholeT_0.5	J1:DiP, J1:OT, J5:DiP, J5:OT, S1:DiP, S5:DiP
568	No	11	11-B	100	McF_6	0	unif	singleC	J1:CBN, J1:CBN-A, J5:CBN, J5:CBN-A, J5:DiP, J5:OT, J5:OT-A
569	No	11	11-B	100	McF_6	0	unif	wholeT_0.01	J1:DiP-A, J5:CBN, J5:CBN-A, J5:DiP, J5:DiP-A, J5:OT, J5:OT-A
570	No	11	11-B	100	McF_6	0	unif	wholeT_0.5	J1:CBN-A, J1:DiP, J5:CBN, J5:CBN-A, J5:DiP, J5:DiP-A, J5:OT, J5:OT-A, S5:CBN-A
571	No	11	11-B	100	McF_6	Inf	last	singleC	J1:DiP, J5:DiP, J5:OT, S5:DiP
572	No	11	11-B	100	McF_6	Inf	last	wholeT_0.01	J1:DiP, J5:DiP, S1:DiP, S5:DiP
573	No	11	11-B	100	McF_6	Inf	last	wholeT_0.5	J1:DiP, J5:DiP, J5:DiP-A, J5:OT
574	No	11	11-B	100	McF_6	Inf	unif	singleC	J1:CBN-A, J5:CBN-A, J5:DiP, J5:OT, J5:OT-A, S5:CBN
575	No	11	11-B	100	McF_6	Inf	unif	wholeT_0.01	J5:CBN-A, J5:DiP, J5:DiP-A, J5:OT, J5:OT-A
576	No	11	11-B	100	McF_6	Inf	unif	wholeT_0.5	J5:CBN-A, J5:DiP, J5:OT, J5:OT-A
577	No	9	9-B	1000	Bozic	0	last	singleC	J1:DiP, J1:DiP-A, J1:OT, J1:OT-A, J5:DiP, J5:DiP-A, J5:OT, J5:OT-A, S1:OT, S1:OT-A, S5:DiP, S5:OT, S5:OT-A
578	No	9	9-B	1000	Bozic	0	last	wholeT_0.01	J1:CBN-A, J1:DiP, J1:DiP-A, J1:OT, J1:OT-A, J5:CBN-A, J5:DiP, J5:DiP-A, J5:OT, J5:OT-A, S1:CBN-A, S1:DiP, S1:DiP-A, S1:OT, S1:OT-A, S5:CBN-A, S5:DiP, S5:DiP-A, S5:OT, S5:OT-A

Table 16: *(continued)*

	Conjunction	Drivers	Tree	S.Size	Model	sh	S.Time	S.Type	Best method(s)
579	No	9	9-B	1000	Bozic	0	last	wholeT_0.5	J1:DiP, J1:DiP-A, J1:OT, J1:OT-A, J5:CBN, J5:CBN-A, J5:DiP, J5:DiP-A, J5:OT, J5:OT-A, S1:OT, S1:OT-A, S5:DiP, S5:DiP-A, S5:OT, S5:OT-A
580	No	9	9-B	1000	Bozic	0	unif	singleC	J1:DiP, J1:DiP-A, J1:OT, J1:OT-A, J5:CBN, J5:CBN-A, J5:DiP, J5:DiP-A, J5:OT, J5:OT-A, S1:DiP, S1:DiP-A, S1:OT, S1:OT-A, S5:DiP, S5:DiP-A, S5:OT-A
581	No	9	9-B	1000	Bozic	0	unif	wholeT_0.01	J5:DiP, J5:DiP-A, J5:OT, J5:OT-A, S5:DiP, S5:DiP-A, S5:OT, S5:OT-A
582	No	9	9-B	1000	Bozic	0	unif	wholeT_0.5	J1:CBN, J1:CBN-A, J1:DiP, J1:DiP-A, J1:OT, J1:OT-A, J5:CBN-A, J5:DiP-A, J5:OT, S1:DiP, S1:DiP-A, S1:OT, S1:OT-A, S5:DiP, S5:DiP-A, S5:OT, S5:OT-A
583	No	9	9-B	1000	Bozic	Inf	last	singleC	J1:DiP, J1:DiP-A, J1:OT, J1:OT-A, J5:DiP, J5:DiP-A, J5:OT, J5:OT-A, S1:DiP, S5:DiP, S5:DiP-A, S5:OT, S5:OT-A
584	No	9	9-B	1000	Bozic	Inf	last	wholeT_0.01	J5:DiP, J5:DiP-A
585	No	9	9-B	1000	Bozic	Inf	last	wholeT_0.5	J1:DiP, J1:DiP-A, J1:OT, J1:OT-A, J5:DiP, J5:DiP-A, J5:OT, J5:OT-A, S1:DiP, S1:DiP-A, S5:DiP, S5:DiP-A, S5:OT, S5:OT-A
586	No	9	9-B	1000	Bozic	Inf	unif	singleC	J1:DiP, J1:DiP-A, J1:OT, J1:OT-A, J5:DiP, J5:DiP-A, J5:OT, J5:OT-A, S5:DiP, S5:DiP-A, S5:OT, S5:OT-A
587	No	9	9-B	1000	Bozic	Inf	unif	wholeT_0.01	J1:CBN-A, J5:DiP, J5:DiP-A, J5:OT, J5:OT-A
588	No	9	9-B	1000	Bozic	Inf	unif	wholeT_0.5	J1:DiP, J1:DiP-A, J1:OT, J1:OT-A, J5:CBN, J5:CBN-A, J5:DiP, J5:DiP-A, J5:OT, J5:OT-A, S5:DiP, S5:DiP-A, S5:OT, S5:OT-A
589	No	9	9-B	1000	exp	0	last	singleC	J1:DiP, J1:DiP-A, J1:OT, J1:OT-A, J5:CBN, J5:CBN-A, J5:DiP, J5:DiP-A, J5:OT, J5:OT-A, S1:DiP, S1:DiP-A, S1:OT, S1:OT-A, S5:DiP, S5:DiP-A, S5:OT, S5:OT-A

Table 16: *(continued)*

	Conjunction	Drivers	Tree	S.Size	Model	sh	S.Time	S.Type	Best method(s)
590	No	9	9-B	1000	exp	0	last	wholeT_0.01	J1:DiP, J1:DiP-A, J1:OT, J1:OT-A, J5:DiP, J5:DiP-A, J5:OT, J5:OT-A, S1:OT-A, S5:DiP, S5:DiP-A, S5:OT, S5:OT-A
591	No	9	9-B	1000	exp	0	last	wholeT_0.5	J1:DiP, J1:DiP-A, J1:OT, J5:CBN, J5:CBN-A, J5:DiP, J5:DiP-A, J5:OT, J5:OT-A, S1:DiP, S1:DiP-A, S1:OT, S1:OT-A, S5:DiP, S5:OT, S5:OT-A
592	No	9	9-B	1000	exp	0	unif	singleC	J1:CBN, J1:CBN-A, J1:DiP, J1:DiP-A, J1:OT, J1:OT-A, J5:CBN, J5:CBN-A, J5:DiP, J5:DiP-A, J5:OT, S1:DiP, S1:DiP-A, S1:OT, S1:OT-A, S5:DiP, S5:DiP-A, S5:OT, S5:OT-A
593	No	9	9-B	1000	exp	0	unif	wholeT_0.01	J1:DiP, J1:DiP-A, J1:OT, J1:OT-A, J5:CBN, J5:CBN-A, J5:DiP, J5:DiP-A, J5:OT, J5:OT-A, S1:DiP, S1:DiP-A, S1:OT, S1:OT-A, S5:DiP, S5:DiP-A, S5:OT, S5:OT-A
594	No	9	9-B	1000	exp	0	unif	wholeT_0.5	J1:CBN, J1:CBN-A, J1:DiP, J1:DiP-A, J1:OT, J1:OT-A, J5:CBN, J5:CBN-A, J5:DiP, J5:DiP-A, J5:OT, J5:OT-A, S1:DiP, S1:DiP-A, S1:OT, S1:OT-A, S5:DiP, S5:DiP-A, S5:OT, S5:OT-A
595	No	9	9-B	1000	exp	Inf	last	singleC	J1:DiP, J1:DiP-A, J1:OT, J1:OT-A, J5:DiP, J5:DiP-A, J5:OT, J5:OT-A, S5:DiP, S5:DiP-A, S5:OT, S5:OT-A
596	No	9	9-B	1000	exp	Inf	last	wholeT_0.01	J5:DiP, J5:DiP-A
597	No	9	9-B	1000	exp	Inf	last	wholeT_0.5	J1:DiP, J1:DiP-A, J1:OT, J1:OT-A, J5:DiP, J5:DiP-A, J5:OT, J5:OT-A, S1:DiP-A, S5:DiP, S5:DiP-A, S5:OT, S5:OT-A
598	No	9	9-B	1000	exp	Inf	unif	singleC	J1:DiP, J1:DiP-A, J1:OT, J1:OT-A, J5:CBN, J5:CBN-A, J5:DiP, J5:DiP-A, J5:OT, J5:OT-A, S1:DiP, S1:DiP-A, S1:OT, S5:DiP, S5:DiP-A, S5:OT, S5:OT-A
599	No	9	9-B	1000	exp	Inf	unif	wholeT_0.01	J5:DiP, J5:DiP-A, J5:OT, J5:OT-A, S5:DiP, S5:DiP-A, S5:OT, S5:OT-A

Table 16: *(continued)*

	Conjunction	Drivers	Tree	S.Size	Model	sh	S.Time	S.Type	Best method(s)
600	No	9	9-B	1000	exp	Inf	unif	wholeT_0.5	J1:DiP, J1:DiP-A, J1:OT, J1:OT-A, J5:CBN, J5:CBN-A, J5:DiP, J5:DiP-A, J5:OT, J5:OT-A, S1:DiP, S1:DiP-A, S1:OT, S1:OT-A, S5:DiP, S5:DiP-A, S5:OT, S5:OT-A
601	No	9	9-B	1000	McF_4	0	last	singleC	J1:DiP, J1:DiP-A, J1:OT, J1:OT-A, J5:CBN, J5:DiP, J5:DiP-A, J5:OT, J5:OT-A, S1:DiP-A, S1:OT, S1:OT-A, S5:DiP, S5:DiP-A, S5:OT, S5:OT-A
602	No	9	9-B	1000	McF_4	0	last	wholeT_0.01	J1:DiP, J1:DiP-A, J1:OT, J1:OT-A, J5:CBN, J5:DiP, J5:DiP-A, J5:OT, J5:OT-A, S1:DiP, S1:DiP-A, S5:DiP, S5:DiP-A, S5:OT, S5:OT-A
603	No	9	9-B	1000	McF_4	0	last	wholeT_0.5	J1:DiP, J1:DiP-A, J1:OT, J1:OT-A, J5:DiP, J5:DiP-A, J5:OT, J5:OT-A, S1:DiP, S1:OT, S1:OT-A, S5:DiP, S5:DiP-A, S5:OT, S5:OT-A
604	No	9	9-B	1000	McF_4	0	unif	singleC	J1:DiP, J1:DiP-A, J1:OT, J1:OT-A, J5:DiP, J5:DiP-A, J5:OT, J5:OT-A, S1:DiP, S1:DiP-A, S1:OT, S1:OT-A, S5:DiP, S5:DiP-A, S5:OT, S5:OT-A
605	No	9	9-B	1000	McF_4	0	unif	wholeT_0.01	J1:DiP, J1:DiP-A, J1:OT, J1:OT-A, J5:DiP, J5:DiP-A, J5:OT, J5:OT-A, S1:DiP-A, S5:DiP, S5:DiP-A, S5:OT, S5:OT-A
606	No	9	9-B	1000	McF_4	0	unif	wholeT_0.5	J1:DiP, J1:DiP-A, J1:OT, J1:OT-A, J5:DiP, J5:DiP-A, J5:OT, J5:OT-A, S1:DiP, S1:DiP-A, S1:OT, S1:OT-A, S5:DiP, S5:DiP-A, S5:OT
607	No	9	9-B	1000	McF_4	Inf	last	singleC	J1:DiP, J1:DiP-A, J1:OT, J1:OT-A, J5:DiP, J5:DiP-A, J5:OT, J5:OT-A, S1:OT, S1:OT-A, S5:DiP, S5:DiP-A, S5:OT, S5:OT-A
608	No	9	9-B	1000	McF_4	Inf	last	wholeT_0.01	J1:DiP, J1:DiP-A, J1:OT, J1:OT-A, J5:DiP, J5:DiP-A, J5:OT-A, S1:DiP, S1:DiP-A, S5:DiP, S5:DiP-A, S5:OT, S5:OT-A

Table 16: *(continued)*

	Conjunction	Drivers	Tree	S.Size	Model	sh	S.Time	S.Type	Best method(s)
609	No	9	9-B	1000	McF_4	Inf	last	wholeT_0.5	J1:DiP, J1:DiP-A, J1:OT, J1:OT-A, J5:DiP, J5:DiP-A, J5:OT-A, S1:DiP, S1:DiP-A, S1:OT, S1:OT-A, S5:DiP, S5:DiP-A, S5:OT, S5:OT-A
610	No	9	9-B	1000	McF_4	Inf	unif	singleC	J1:DiP, J1:DiP-A, J1:OT, J1:OT-A, J5:DiP, J5:DiP-A, J5:OT, J5:OT-A, S1:DiP, S1:DiP-A, S1:OT, S1:OT-A, S5:DiP, S5:DiP-A, S5:OT, S5:OT-A
611	No	9	9-B	1000	McF_4	Inf	unif	wholeT_0.01	J1:DiP, J1:DiP-A, J1:OT, J1:OT-A, J5:DiP, J5:DiP-A, J5:OT, J5:OT-A, S1:DiP-A, S5:DiP, S5:DiP-A, S5:OT, S5:OT-A
612	No	9	9-B	1000	McF_4	Inf	unif	wholeT_0.5	J1:DiP, J1:DiP-A, J1:OT, J1:OT-A, J5:DiP, J5:DiP-A, J5:OT, J5:OT-A, S1:DiP, S1:DiP-A, S1:OT, S1:OT-A, S5:DiP, S5:DiP-A, S5:OT, S5:OT-A
613	No	9	9-B	1000	McF_6	0	last	singleC	J1:DiP, J1:OT, J5:DiP, J5:OT, S1:DiP, S5:DiP, S5:OT
614	No	9	9-B	1000	McF_6	0	last	wholeT_0.01	J1:DiP, J1:OT, J5:DiP, J5:OT, S1:DiP, S5:DiP, S5:OT
615	No	9	9-B	1000	McF_6	0	last	wholeT_0.5	J1:DiP, J1:OT, J5:DiP, J5:OT, S1:DiP, S1:OT, S5:DiP, S5:OT
616	No	9	9-B	1000	McF_6	0	unif	singleC	J1:CBN, J1:CBN-A, J1:DiP, J1:DiP-A, J1:OT, J1:OT-A, J5:CBN, J5:CBN-A, J5:DiP, J5:DiP-A, J5:OT, J5:OT-A, S1:DiP, S1:DiP-A, S1:OT, S1:OT-A, S5:CBN, S5:CBN-A, S5:DiP, S5:DiP-A, S5:OT, S5:OT-A
617	No	9	9-B	1000	McF_6	0	unif	wholeT_0.01	J1:CBN, J1:CBN-A, J1:DiP, J1:DiP-A, J1:OT, J1:OT-A, J5:CBN, J5:CBN-A, J5:DiP, J5:DiP-A, J5:OT, J5:OT-A, S1:DiP-A, S5:DiP, S5:DiP-A, S5:OT, S5:OT-A
618	No	9	9-B	1000	McF_6	0	unif	wholeT_0.5	J1:CBN, J1:CBN-A, J1:DiP, J1:DiP-A, J1:OT, J5:CBN-A, J5:DiP, J5:DiP-A, J5:OT, J5:OT-A, S1:DiP, S1:OT, S1:OT-A, S5:CBN-A, S5:DiP, S5:DiP-A, S5:OT, S5:OT-A
619	No	9	9-B	1000	McF_6	Inf	last	singleC	J1:DiP, J1:OT, J5:DiP, J5:OT, S1:DiP, S5:DiP, S5:OT

Table 16: *(continued)*

	Conjunction	Drivers	Tree	S.Size	Model	sh	S.Time	S.Type	Best method(s)
620	No	9	9-B	1000	McF_6	Inf	last	wholeT_0.01	J1:DiP, J1:OT, J5:DiP, J5:OT, S5:DiP, S5:OT
621	No	9	9-B	1000	McF_6	Inf	last	wholeT_0.5	J1:DiP, J1:OT, J5:DiP, J5:OT, S1:DiP, S1:OT, S5:DiP, S5:OT
622	No	9	9-B	1000	McF_6	Inf	unif	singleC	J1:CBN, J1:CBN-A, J1:DiP, J1:DiP-A, J1:OT, J1:OT-A, J5:CBN-A, J5:DiP, J5:DiP-A, J5:OT, J5:OT-A, S1:DiP, S1:DiP-A, S1:OT, S1:OT-A, S5:DiP, S5:DiP-A, S5:OT, S5:OT-A
623	No	9	9-B	1000	McF_6	Inf	unif	wholeT_0.01	J1:CBN-A, J1:DiP-A, J1:OT, J1:OT-A, J5:CBN-A, J5:DiP, J5:DiP-A, J5:OT, J5:OT-A, S5:CBN, S5:DiP, S5:DiP-A, S5:OT, S5:OT-A
624	No	9	9-B	1000	McF_6	Inf	unif	wholeT_0.5	J1:CBN, J1:CBN-A, J1:DiP, J1:DiP-A, J1:OT, J1:OT-A, J5:CBN-A, J5:DiP, J5:DiP-A, J5:OT, J5:OT-A, S1:DiP, S1:DiP-A, S1:OT, S1:OT-A, S5:DiP, S5:DiP-A, S5:OT, S5:OT-A
625	No	9	9-B	200	Bozic	0	last	singleC	J1:DiP, J1:DiP-A, J1:OT, J1:OT-A, J5:DiP, J5:DiP-A, J5:OT, J5:OT-A, S1:DiP, S1:DiP-A, S5:DiP, S5:DiP-A, S5:OT, S5:OT-A
626	No	9	9-B	200	Bozic	0	last	wholeT_0.01	J1:DiP, J1:DiP-A, J1:OT, J1:OT-A, J5:CBN-A, J5:DiP, J5:DiP-A, J5:OT, J5:OT-A, S1:DiP, S5:DiP, S5:DiP-A, S5:OT, S5:OT-A
627	No	9	9-B	200	Bozic	0	last	wholeT_0.5	J1:DiP, J1:DiP-A, J1:OT, J1:OT-A, J5:DiP, J5:DiP-A, J5:OT, J5:OT-A, S1:DiP, S1:DiP-A, S5:DiP, S5:DiP-A, S5:OT, S5:OT-A
628	No	9	9-B	200	Bozic	0	unif	singleC	J1:DiP, J1:DiP-A, J1:OT, J1:OT-A, J5:CBN, J5:CBN-A, J5:DiP, J5:DiP-A, J5:OT, J5:OT-A, S1:DiP, S1:DiP-A, S1:OT, S5:DiP, S5:DiP-A, S5:OT, S5:OT-A
629	No	9	9-B	200	Bozic	0	unif	wholeT_0.01	J1:DiP, J1:DiP-A, J1:OT, J1:OT-A, J5:DiP, J5:DiP-A, J5:OT, J5:OT-A, S5:DiP, S5:DiP-A, S5:OT, S5:OT-A

Table 16: *(continued)*

	Conjunction	Drivers	Tree	S.Size	Model	sh	S.Time	S.Type	Best method(s)
630	No	9	9-B	200	Bozic	0	unif	wholeT_0.5	J1:DiP, J1:DiP-A, J1:OT, J1:OT-A, J5:CBN, J5:CBN-A, J5:DiP, J5:DiP-A, J5:OT, J5:OT-A, S1:DiP, S1:DiP-A, S1:OT, S1:OT-A, S5:DiP, S5:DiP-A, S5:OT, S5:OT-A
631	No	9	9-B	200	Bozic	Inf	last	singleC	J1:DiP, J1:DiP-A, J5:DiP, J5:DiP-A, J5:OT, J5:OT-A, S1:DiP, S5:DiP, S5:DiP-A, S5:OT
632	No	9	9-B	200	Bozic	Inf	last	wholeT_0.01	J5:CBN-A, J5:DiP, J5:DiP-A, J5:OT, J5:OT-A
633	No	9	9-B	200	Bozic	Inf	last	wholeT_0.5	J1:DiP, J1:DiP-A, J1:OT, J1:OT-A, J5:DiP, J5:DiP-A, J5:OT, J5:OT-A, S5:DiP, S5:DiP-A, S5:OT, S5:OT-A
634	No	9	9-B	200	Bozic	Inf	unif	singleC	J1:DiP, J1:DiP-A, J1:OT, J1:OT-A, J5:DiP, J5:DiP-A, J5:OT, J5:OT-A, S5:DiP, S5:DiP-A, S5:OT, S5:OT-A
635	No	9	9-B	200	Bozic	Inf	unif	wholeT_0.01	J5:DiP, J5:OT, J5:OT-A
636	No	9	9-B	200	Bozic	Inf	unif	wholeT_0.5	J1:DiP, J1:DiP-A, J1:OT, J1:OT-A, J5:CBN-A, J5:DiP, J5:DiP-A, J5:OT, J5:OT-A, S5:DiP, S5:DiP-A, S5:OT, S5:OT-A
637	No	9	9-B	200	exp	0	last	singleC	J1:DiP, J1:DiP-A, J1:OT, J1:OT-A, J5:CBN, J5:CBN-A, J5:DiP, J5:DiP-A, J5:OT, J5:OT-A, S1:DiP, S1:DiP-A, S1:OT, S1:OT-A, S5:DiP, S5:DiP-A, S5:OT, S5:OT-A
638	No	9	9-B	200	exp	0	last	wholeT_0.01	J1:DiP, J1:DiP-A, J1:OT, J1:OT-A, J5:DiP, J5:DiP-A, J5:OT, J5:OT-A, S1:DiP, S1:DiP-A, S5:DiP, S5:DiP-A, S5:OT, S5:OT-A
639	No	9	9-B	200	exp	0	last	wholeT_0.5	J1:DiP, J1:DiP-A, J1:OT, J1:OT-A, J5:CBN, J5:CBN-A, J5:DiP, J5:DiP-A, J5:OT, J5:OT-A, S1:DiP, S1:DiP-A, S1:OT, S1:OT-A, S5:DiP, S5:DiP-A, S5:OT, S5:OT-A
640	No	9	9-B	200	exp	0	unif	singleC	J1:CBN, J1:CBN-A, J1:DiP, J1:OT, J1:OT-A, J5:CBN, J5:CBN-A, J5:DiP, J5:DiP-A, J5:OT, J5:OT-A, S1:DiP, S1:DiP-A, S1:OT, S1:OT-A, S5:DiP, S5:DiP-A, S5:OT, S5:OT-A

Table 16: *(continued)*

	Conjunction	Drivers	Tree	S.Size	Model	sh	S.Time	S.Type	Best method(s)
641	No	9	9-B	200	exp	0	unif	wholeT_0.01	J1:DiP, J1:DiP-A, J1:OT, J1:OT-A, J5:CBN, J5:CBN-A, J5:DiP, J5:DiP-A, J5:OT, J5:OT-A, S1:DiP, S1:DiP-A, S1:OT-A, S5:DiP, S5:DiP-A, S5:OT, S5:OT-A
642	No	9	9-B	200	exp	0	unif	wholeT_0.5	J1:CBN, J1:CBN-A, J1:DiP, J1:DiP-A, J1:OT, J1:OT-A, J5:CBN, J5:CBN-A, J5:DiP, J5:DiP-A, J5:OT, J5:OT-A, S1:DiP, S1:DiP-A, S1:OT, S1:OT-A, S5:DiP, S5:DiP-A, S5:OT, S5:OT-A
643	No	9	9-B	200	exp	Inf	last	singleC	J1:DiP, J1:DiP-A, J1:OT, J1:OT-A, J5:DiP, J5:DiP-A, J5:OT, J5:OT-A, S1:DiP, S1:DiP-A, S5:DiP, S5:DiP-A, S5:OT, S5:OT-A
644	No	9	9-B	200	exp	Inf	last	wholeT_0.01	J5:CBN-A, J5:DiP, J5:DiP-A, J5:OT, J5:OT-A
645	No	9	9-B	200	exp	Inf	last	wholeT_0.5	J1:DiP, J1:DiP-A, J1:OT, J1:OT-A, J5:DiP, J5:DiP-A, J5:OT, J5:OT-A, S1:DiP, S1:DiP-A, S5:DiP, S5:DiP-A, S5:OT, S5:OT-A
646	No	9	9-B	200	exp	Inf	unif	singleC	J1:DiP, J1:DiP-A, J1:OT, J1:OT-A, J5:CBN, J5:OT-A, S1:DiP-A, S5:DiP, S5:DiP-A, S5:OT, S5:OT-A
647	No	9	9-B	200	exp	Inf	unif	wholeT_0.01	J5:DiP, J5:DiP-A, J5:OT, J5:OT-A, S5:DiP, S5:OT, S5:OT-A
648	No	9	9-B	200	exp	Inf	unif	wholeT_0.5	J1:DiP, J1:DiP-A, J1:OT, J1:OT-A, J5:CBN-A, J5:DiP, J5:DiP-A, J5:OT, J5:OT-A, S1:DiP, S1:DiP-A, S5:DiP, S5:DiP-A, S5:OT, S5:OT-A
649	No	9	9-B	200	McF_4	0	last	singleC	J1:DiP, J1:DiP-A, J1:OT, J1:OT-A, J5:DiP, J5:DiP-A, J5:OT, J5:OT-A, S5:DiP, S5:DiP-A, S5:OT, S5:OT-A
650	No	9	9-B	200	McF_4	0	last	wholeT_0.01	J1:DiP, J1:DiP-A, J1:OT, J1:OT-A, J5:DiP, J5:DiP-A, J5:OT, J5:OT-A, S1:DiP, S1:DiP-A, S5:DiP, S5:DiP-A, S5:OT, S5:OT-A
651	No	9	9-B	200	McF_4	0	last	wholeT_0.5	J1:DiP, J1:DiP-A, J1:OT, J1:OT-A, J5:DiP, J5:DiP-A, J5:OT, J5:OT-A, S1:DiP-A, S5:DiP, S5:DiP-A, S5:OT, S5:OT-A

Table 16: *(continued)*

	Conjunction	Drivers	Tree	S.Size	Model	sh	S.Time	S.Type	Best method(s)
652	No	9	9-B	200	McF_4	0	unif	singleC	J1:DiP, J1:DiP-A, J1:OT, J1:OT-A, J5:DiP, J5:DiP-A, J5:OT, J5:OT-A, S1:DiP, S1:DiP-A, S5:DiP, S5:DiP-A, S5:OT, S5:OT-A
653	No	9	9-B	200	McF_4	0	unif	wholeT_0.01	J1:DiP, J1:DiP-A, J1:OT, J1:OT-A, J5:DiP, J5:DiP-A, J5:OT, J5:OT-A, S1:DiP, S1:DiP-A, S5:DiP, S5:DiP-A, S5:OT, S5:OT-A
654	No	9	9-B	200	McF_4	0	unif	wholeT_0.5	J1:DiP, J1:DiP-A, J1:OT, J1:OT-A, J5:DiP, J5:DiP-A, J5:OT, J5:OT-A, S1:DiP, S1:DiP-A, S5:DiP, S5:DiP-A, S5:OT, S5:OT-A
655	No	9	9-B	200	McF_4	Inf	last	singleC	J1:DiP, J1:DiP-A, J1:OT, J1:OT-A, J5:DiP, J5:DiP-A, J5:OT, J5:OT-A, S5:DiP, S5:DiP-A, S5:OT, S5:OT-A
656	No	9	9-B	200	McF_4	Inf	last	wholeT_0.01	J1:DiP, J1:DiP-A, J1:OT, J1:OT-A, J5:DiP, J5:DiP-A, J5:OT, J5:OT-A, S5:DiP, S5:DiP-A, S5:OT, S5:OT-A
657	No	9	9-B	200	McF_4	Inf	last	wholeT_0.5	J1:DiP, J1:DiP-A, J1:OT, J1:OT-A, J5:DiP, J5:DiP-A, J5:OT, J5:OT-A, S1:DiP, S1:DiP-A, S5:DiP, S5:DiP-A, S5:OT, S5:OT-A
658	No	9	9-B	200	McF_4	Inf	unif	singleC	J1:DiP, J1:DiP-A, J1:OT, J1:OT-A, J5:DiP, J5:DiP-A, J5:OT, J5:OT-A, S1:DiP, S1:DiP-A, S5:DiP, S5:DiP-A, S5:OT, S5:OT-A
659	No	9	9-B	200	McF_4	Inf	unif	wholeT_0.01	J1:DiP, J1:DiP-A, J1:OT, J1:OT-A, J5:DiP, J5:DiP-A, J5:OT, J5:OT-A, S5:DiP, S5:DiP-A, S5:OT
660	No	9	9-B	200	McF_4	Inf	unif	wholeT_0.5	J1:DiP, J1:DiP-A, J1:OT, J1:OT-A, J5:DiP, J5:DiP-A, J5:OT, J5:OT-A, S1:DiP, S1:DiP-A, S5:DiP, S5:DiP-A, S5:OT, S5:OT-A
661	No	9	9-B	200	McF_6	0	last	singleC	J1:DiP, J1:OT, J5:DiP, J5:OT, S1:DiP, S5:DiP, S5:OT
662	No	9	9-B	200	McF_6	0	last	wholeT_0.01	J1:DiP, J1:OT, J5:DiP, J5:OT, S1:DiP, S5:DiP, S5:OT
663	No	9	9-B	200	McF_6	0	last	wholeT_0.5	J1:DiP, J1:OT, J5:DiP, J5:OT, S1:DiP, S5:DiP, S5:OT

Table 16: *(continued)*

	Conjunction	Drivers	Tree	S.Size	Model	sh	S.Time	S.Type	Best method(s)
664	No	9	9-B	200	McF_6	0	unif	singleC	J1:CBN, J1:CBN-A, J1:DiP-A, J1:OT, J1:OT-A, J5:CBN, J5:CBN-A, J5:DiP, J5:DiP-A, J5:OT, J5:OT-A, S1:DiP, S1:DiP-A, S5:CBN, S5:DiP, S5:DiP-A, S5:OT, S5:OT-A
665	No	9	9-B	200	McF_6	0	unif	wholeT_0.01	J1:CBN-A, J1:DiP, J1:DiP-A, J1:OT, J1:OT-A, J5:CBN, J5:CBN-A, J5:DiP, J5:DiP-A, J5:OT, J5:OT-A, S1:DiP-A, S5:DiP, S5:DiP-A, S5:OT, S5:OT-A
666	No	9	9-B	200	McF_6	0	unif	wholeT_0.5	J1:CBN-A, J1:DiP, J1:DiP-A, J1:OT, J1:OT-A, J5:CBN, J5:CBN-A, J5:DiP, J5:DiP-A, J5:OT, J5:OT-A, S5:CBN-A, S5:DiP, S5:DiP-A, S5:OT, S5:OT-A
667	No	9	9-B	200	McF_6	Inf	last	singleC	J1:DiP, J1:OT, J5:DiP, J5:OT, S1:DiP, S5:DiP, S5:OT
668	No	9	9-B	200	McF_6	Inf	last	wholeT_0.01	J1:DiP, J1:OT, J5:DiP, J5:OT, S1:DiP, S5:DiP, S5:OT
669	No	9	9-B	200	McF_6	Inf	last	wholeT_0.5	J1:DiP, J1:OT, J5:DiP, J5:OT, S1:DiP, S5:DiP, S5:OT
670	No	9	9-B	200	McF_6	Inf	unif	singleC	J1:CBN, J1:CBN-A, J1:DiP, J1:DiP-A, J1:OT, J1:OT-A, J5:CBN-A, J5:DiP, J5:DiP-A, J5:OT, J5:OT-A, S1:DiP, S1:DiP-A, S5:CBN, S5:DiP, S5:DiP-A, S5:OT, S5:OT-A
671	No	9	9-B	200	McF_6	Inf	unif	wholeT_0.01	J1:CBN, J1:CBN-A, J1:DiP, J1:OT, J1:OT-A, J5:DiP, J5:OT, J5:OT-A, S5:OT, S5:OT-A
672	No	9	9-B	200	McF_6	Inf	unif	wholeT_0.5	J1:CBN, J1:CBN-A, J1:DiP, J1:DiP-A, J1:OT, J1:OT-A, J5:CBN-A, J5:DiP, J5:DiP-A, J5:OT, J5:OT-A, S5:CBN, S5:CBN-A, S5:DiP, S5:DiP-A, S5:OT, S5:OT-A
673	No	9	9-B	100	Bozic	0	last	singleC	J1:DiP, J1:DiP-A, J1:OT, J1:OT-A, J5:DiP, J5:DiP-A, J5:OT, J5:OT-A, S1:DiP, S1:DiP-A, S5:DiP, S5:DiP-A, S5:OT, S5:OT-A
674	No	9	9-B	100	Bozic	0	last	wholeT_0.01	J1:DiP, J1:DiP-A, J1:OT, J5:DiP, J5:DiP-A, J5:OT, J5:OT-A, S1:DiP, S1:DiP-A, S5:DiP, S5:DiP-A, S5:OT, S5:OT-A

Table 16: *(continued)*

	Conjunction	Drivers	Tree	S.Size	Model	sh	S.Time	S.Type	Best method(s)
675	No	9	9-B	100	Bozic	0	last	wholeT_0.5	J1:DiP, J1:DiP-A, J1:OT, J1:OT-A, J5:DiP, J5:DiP-A, J5:OT, J5:OT-A, S1:DiP, S1:DiP-A, S5:DiP, S5:OT, S5:OT-A
676	No	9	9-B	100	Bozic	0	unif	singleC	J1:DiP, J1:DiP-A, J1:OT, J1:OT-A, J5:CBN, J5:CBN-A, J5:DiP, J5:DiP-A, J5:OT, J5:OT-A, S1:DiP, S1:DiP-A, S5:DiP, S5:DiP-A, S5:OT, S5:OT-A
677	No	9	9-B	100	Bozic	0	unif	wholeT_0.01	J1:DiP, J1:DiP-A, J5:DiP, J5:DiP-A, J5:OT, J5:OT-A, S1:DiP, S1:DiP-A, S5:DiP, S5:DiP-A, S5:OT, S5:OT-A
678	No	9	9-B	100	Bozic	0	unif	wholeT_0.5	J1:DiP, J1:DiP-A, J5:CBN, J5:CBN-A, J5:DiP, J5:DiP-A, J5:OT, J5:OT-A, S1:DiP, S1:DiP-A, S5:DiP, S5:DiP-A, S5:OT, S5:OT-A
679	No	9	9-B	100	Bozic	Inf	last	singleC	J1:DiP, J1:DiP-A, J5:DiP, J5:DiP-A, J5:OT, J5:OT-A, S1:DiP-A, S5:DiP, S5:DiP-A
680	No	9	9-B	100	Bozic	Inf	last	wholeT_0.01	J5:DiP, J5:DiP-A
681	No	9	9-B	100	Bozic	Inf	last	wholeT_0.5	J1:DiP, J1:DiP-A, J5:DiP, J5:DiP-A, J5:OT, J5:OT-A, S1:DiP, S1:DiP-A, S5:DiP, S5:DiP-A
682	No	9	9-B	100	Bozic	Inf	unif	singleC	J1:DiP, J1:DiP-A, J5:DiP, J5:DiP-A, J5:OT, J5:OT-A, S5:DiP, S5:DiP-A, S5:OT, S5:OT-A
683	No	9	9-B	100	Bozic	Inf	unif	wholeT_0.01	J5:DiP, J5:OT, J5:OT-A
684	No	9	9-B	100	Bozic	Inf	unif	wholeT_0.5	J1:DiP, J1:DiP-A, J5:DiP, J5:DiP-A, J5:OT, J5:OT-A, S5:DiP, S5:DiP-A, S5:OT, S5:OT-A
685	No	9	9-B	100	exp	0	last	singleC	J1:DiP, J1:DiP-A, J1:OT, J1:OT-A, J5:CBN, J5:CBN-A, J5:DiP, J5:DiP-A, J5:OT, J5:OT-A, S1:DiP, S1:DiP-A, S1:OT, S1:OT-A, S5:DiP, S5:DiP-A, S5:OT
686	No	9	9-B	100	exp	0	last	wholeT_0.01	J1:DiP, J1:DiP-A, J1:OT, J1:OT-A, J5:DiP, J5:DiP-A, J5:OT, J5:OT-A, S1:DiP, S1:DiP-A, S5:DiP, S5:DiP-A, S5:OT, S5:OT-A

Table 16: *(continued)*

	Conjunction	Drivers	Tree	S.Size	Model	sh	S.Time	S.Type	Best method(s)
687	No	9	9-B	100	exp	0	last	wholeT_0.5	J1:DiP, J1:DiP-A, J1:OT, J1:OT-A, J5:CBN, J5:CBN-A, J5:DiP, J5:DiP-A, J5:OT, J5:OT-A, S1:DiP, S1:DiP-A, S1:OT-A, S5:DiP, S5:DiP-A, S5:OT
688	No	9	9-B	100	exp	0	unif	singleC	J1:DiP, J1:DiP-A, J1:OT, J1:OT-A, J5:CBN, J5:CBN-A, J5:DiP, J5:DiP-A, J5:OT, J5:OT-A, S1:DiP, S1:DiP-A, S1:OT, S1:OT-A, S5:DiP, S5:DiP-A, S5:OT, S5:OT-A
689	No	9	9-B	100	exp	0	unif	wholeT_0.01	J1:DiP, J1:DiP-A, J1:OT, J1:OT-A, J5:CBN, J5:DiP, J5:DiP-A, J5:OT, J5:OT-A, S1:DiP, S1:DiP-A, S5:DiP, S5:DiP-A, S5:OT, S5:OT-A
690	No	9	9-B	100	exp	0	unif	wholeT_0.5	J1:DiP, J1:DiP-A, J1:OT, J1:OT-A, J5:CBN, J5:CBN-A, J5:DiP-A, J5:OT, J5:OT-A, S1:DiP, S1:DiP-A, S1:OT, S1:OT-A, S5:DiP, S5:DiP-A, S5:OT, S5:OT-A
691	No	9	9-B	100	exp	Inf	last	singleC	J1:DiP, J1:DiP-A, J5:DiP, J5:DiP-A, J5:OT-A, S1:DiP, S1:DiP-A, S5:DiP, S5:DiP-A, S5:OT, S5:OT-A
692	No	9	9-B	100	exp	Inf	last	wholeT_0.01	J5:DiP, J5:DiP-A, J5:OT, J5:OT-A
693	No	9	9-B	100	exp	Inf	last	wholeT_0.5	J1:DiP, J1:DiP-A, J5:DiP, J5:OT, J5:OT-A, S1:DiP-A, S5:DiP, S5:DiP-A, S5:OT, S5:OT-A
694	No	9	9-B	100	exp	Inf	unif	singleC	J1:DiP, J1:DiP-A, J5:CBN, J5:CBN-A, J5:DiP, J5:DiP-A, J5:OT, J5:OT-A, S1:DiP, S1:DiP-A, S5:DiP, S5:DiP-A, S5:OT, S5:OT-A
695	No	9	9-B	100	exp	Inf	unif	wholeT_0.01	J5:DiP, J5:DiP-A, J5:OT, J5:OT-A, S5:OT, S5:OT-A
696	No	9	9-B	100	exp	Inf	unif	wholeT_0.5	J1:DiP, J1:DiP-A, J1:OT, J5:CBN, J5:DiP, J5:DiP-A, J5:OT, J5:OT-A, S1:DiP, S1:DiP-A, S5:DiP, S5:DiP-A, S5:OT, S5:OT-A
697	No	9	9-B	100	McF_4	0	last	singleC	J1:DiP, J1:OT-A, J5:DiP, J5:DiP-A, J5:OT, J5:OT-A, S1:DiP, S1:DiP-A, S5:DiP, S5:DiP-A, S5:OT

Table 16: *(continued)*

	Conjunction	Drivers	Tree	S.Size	Model	sh	S.Time	S.Type	Best method(s)
698	No	9	9-B	100	McF_4	0	last	wholeT_0.01	J1:DiP-A, J5:DiP, J5:DiP-A, J5:OT, J5:OT-A, S1:DiP, S1:DiP-A, S5:DiP, S5:DiP-A, S5:OT, S5:OT-A
699	No	9	9-B	100	McF_4	0	last	wholeT_0.5	J1:DiP, J1:DiP-A, J1:OT, J1:OT-A, J5:DiP, J5:DiP-A, J5:OT, J5:OT-A, S1:DiP, S1:DiP-A, S5:DiP, S5:DiP-A, S5:OT, S5:OT-A
700	No	9	9-B	100	McF_4	0	unif	singleC	J1:DiP, J1:DiP-A, J1:OT-A, J5:DiP, J5:DiP-A, J5:OT, J5:OT-A, S1:DiP, S1:DiP-A, S5:DiP, S5:DiP-A, S5:OT, S5:OT-A
701	No	9	9-B	100	McF_4	0	unif	wholeT_0.01	J1:DiP, J1:DiP-A, J5:DiP, J5:DiP-A, J5:OT, J5:OT-A, S1:DiP, S1:DiP-A, S5:DiP, S5:DiP-A, S5:OT, S5:OT-A
702	No	9	9-B	100	McF_4	0	unif	wholeT_0.5	J1:DiP, J1:DiP-A, J1:OT, J1:OT-A, J5:DiP, J5:DiP-A, J5:OT, J5:OT-A, S1:DiP, S5:DiP, S5:DiP-A, S5:OT, S5:OT-A
703	No	9	9-B	100	McF_4	Inf	last	singleC	J1:DiP, J1:DiP-A, J1:OT, J1:OT-A, J5:DiP, J5:DiP-A, J5:OT, J5:OT-A, S1:DiP, S1:DiP-A, S5:DiP, S5:DiP-A, S5:OT, S5:OT-A
704	No	9	9-B	100	McF_4	Inf	last	wholeT_0.01	J5:DiP, J5:DiP-A, J5:OT, S1:DiP, S5:DiP, S5:DiP-A, S5:OT, S5:OT-A
705	No	9	9-B	100	McF_4	Inf	last	wholeT_0.5	J1:DiP, J1:DiP-A, J5:DiP, J5:DiP-A, J5:OT, J5:OT-A, S1:DiP, S1:DiP-A, S5:DiP, S5:DiP-A, S5:OT, S5:OT-A
706	No	9	9-B	100	McF_4	Inf	unif	singleC	J1:DiP, J1:DiP-A, J1:OT, J1:OT-A, J5:DiP, J5:DiP-A, J5:OT, J5:OT-A, S1:DiP, S1:DiP-A, S5:DiP, S5:OT, S5:OT-A
707	No	9	9-B	100	McF_4	Inf	unif	wholeT_0.01	J1:DiP, J1:DiP-A, J5:DiP, J5:DiP-A, J5:OT, J5:OT-A, S5:DiP, S5:DiP-A, S5:OT, S5:OT-A
708	No	9	9-B	100	McF_4	Inf	unif	wholeT_0.5	J1:DiP, J1:DiP-A, J1:OT, J1:OT-A, J5:DiP, J5:DiP-A, J5:OT, J5:OT-A, S1:DiP, S1:DiP-A, S5:DiP, S5:DiP-A, S5:OT, S5:OT-A

Table 16: *(continued)*

	Conjunction	Drivers	Tree	S.Size	Model	sh	S.Time	S.Type	Best method(s)
709	No	9	9-B	100	McF_6	0	last	singleC	J1:DiP, J1:DiP-A, J1:OT, J5:DiP, J5:DiP-A, J5:OT, S1:DiP, S1:DiP-A, S5:DiP, S5:DiP-A, S5:OT
710	No	9	9-B	100	McF_6	0	last	wholeT_0.01	J5:DiP, J5:OT, S1:DiP, S5:DiP, S5:OT
711	No	9	9-B	100	McF_6	0	last	wholeT_0.5	J1:DiP, J1:OT, J5:DiP, J5:DiP-A, J5:OT, S1:DiP, S1:DiP-A, S5:DiP, S5:DiP-A, S5:OT
712	No	9	9-B	100	McF_6	0	unif	singleC	J1:CBN, J1:CBN-A, J1:DiP, J1:DiP-A, J1:OT, J1:OT-A, J5:CBN-A, J5:DiP, J5:DiP-A, J5:OT, J5:OT-A, S1:DiP, S1:DiP-A, S5:CBN-A, S5:DiP, S5:DiP-A, S5:OT, S5:OT-A
713	No	9	9-B	100	McF_6	0	unif	wholeT_0.01	J1:CBN, J1:DiP, J1:DiP-A, J5:CBN-A, J5:DiP, J5:DiP-A, J5:OT, J5:OT-A, S1:DiP, S5:DiP, S5:DiP-A, S5:OT, S5:OT-A
714	No	9	9-B	100	McF_6	0	unif	wholeT_0.5	J1:CBN, J1:CBN-A, J1:DiP, J1:DiP-A, J1:OT, J1:OT-A, J5:CBN-A, J5:DiP, J5:DiP-A, J5:OT, J5:OT-A, S1:DiP, S5:CBN, S5:CBN-A, S5:DiP, S5:OT, S5:OT-A
715	No	9	9-B	100	McF_6	Inf	last	singleC	J1:DiP, J1:DiP-A, J1:OT, J5:DiP, J5:DiP-A, J5:OT, S1:DiP, S1:DiP-A, S5:DiP, S5:DiP-A, S5:OT
716	No	9	9-B	100	McF_6	Inf	last	wholeT_0.01	J1:DiP, J5:DiP, J5:OT, S1:DiP, S5:DiP, S5:OT
717	No	9	9-B	100	McF_6	Inf	last	wholeT_0.5	J1:DiP, J1:DiP-A, J1:OT, J5:DiP, J5:DiP-A, J5:OT, S1:DiP, S5:DiP, S5:DiP-A, S5:OT
718	No	9	9-B	100	McF_6	Inf	unif	singleC	J1:CBN, J1:CBN-A, J1:DiP, J1:OT, J1:OT-A, J5:CBN-A, J5:DiP, J5:DiP-A, J5:OT, J5:OT-A, S1:DiP, S1:DiP-A, S5:CBN-A, S5:DiP, S5:DiP-A, S5:OT, S5:OT-A
719	No	9	9-B	100	McF_6	Inf	unif	wholeT_0.01	J1:DiP, J5:CBN-A, J5:DiP, J5:DiP-A, J5:OT, J5:OT-A, S1:DiP, S5:CBN-A, S5:DiP, S5:OT, S5:OT-A

Table 16: *(continued)*

	Conjunction	Drivers	Tree	S.Size	Model	sh	S.Time	S.Type	Best method(s)
720	No	9	9-B	100	McF_6	Inf	unif	wholeT_0.5	J1:CBN, J1:CBN-A, J1:DiP, J1:DiP-A, J1:OT, J1:OT-A, J5:CBN-A, J5:DiP, J5:DiP-A, J5:OT, J5:OT-A, S5:CBN, S5:CBN-A, S5:OT, S5:OT-A
721	No	7	7-B	1000	Bozic	0	last	singleC	J1:CBN, J1:CBN-A, J1:DiP, J1:DiP-A, J1:OT, J5:CBN, J5:CBN-A, J5:DiP, J5:DiP-A, J5:OT, J5:OT-A, S1:DiP, S1:DiP-A, S1:OT, S1:OT-A, S5:CBN, S5:DiP, S5:DiP-A, S5:OT, S5:OT-A
722	No	7	7-B	1000	Bozic	0	last	wholeT_0.01	J1:CBN, J1:CBN-A, J1:DiP, J1:DiP-A, J1:OT, J1:OT-A, J5:CBN, J5:CBN-A, J5:DiP, J5:DiP-A, J5:OT, J5:OT-A, S1:CBN, S1:CBN-A, S1:DiP-A, S1:OT, S1:OT-A, S5:CBN, S5:CBN-A, S5:DiP, S5:DiP-A, S5:OT, S5:OT-A
723	No	7	7-B	1000	Bozic	0	last	wholeT_0.5	J1:CBN, J1:CBN-A, J1:DiP, J1:DiP-A, J1:OT, J1:OT-A, J5:CBN, J5:CBN-A, J5:DiP-A, J5:OT, J5:OT-A, S1:DiP, S1:DiP-A, S1:OT, S1:OT-A, S5:CBN, S5:DiP, S5:DiP-A, S5:OT, S5:OT-A
724	No	7	7-B	1000	Bozic	0	unif	singleC	J1:CBN, J1:CBN-A, J1:DiP, J1:DiP-A, J1:OT, J1:OT-A, J5:CBN, J5:CBN-A, J5:DiP, J5:DiP-A, J5:OT, J5:OT-A, S1:CBN-A, S1:DiP, S1:DiP-A, S1:OT-A, S5:CBN, S5:CBN-A, S5:DiP, S5:DiP-A, S5:OT, S5:OT-A
725	No	7	7-B	1000	Bozic	0	unif	wholeT_0.01	J5:CBN, J5:CBN-A, J5:DiP, J5:DiP-A, J5:OT, J5:OT-A
726	No	7	7-B	1000	Bozic	0	unif	wholeT_0.5	J1:CBN-A, J1:DiP, J1:DiP-A, J1:OT, J1:OT-A, J5:CBN, J5:CBN-A, J5:DiP, J5:DiP-A, J5:OT, S1:CBN, S1:CBN-A, S1:DiP, S1:DiP-A, S1:OT, S1:OT-A, S5:CBN, S5:CBN-A, S5:DiP, S5:DiP-A, S5:OT, S5:OT-A
727	No	7	7-B	1000	Bozic	Inf	last	singleC	J1:DiP, J1:DiP-A, J1:OT, J1:OT-A, J5:DiP, J5:DiP-A, J5:OT, J5:OT-A, S5:DiP, S5:DiP-A, S5:OT, S5:OT-A
728	No	7	7-B	1000	Bozic	Inf	last	wholeT_0.01	J5:CBN, J5:CBN-A, J5:DiP, J5:DiP-A, J5:OT, J5:OT-A

Table 16: *(continued)*

	Conjunction	Drivers	Tree	S.Size	Model	sh	S.Time	S.Type	Best method(s)
729	No	7	7-B	1000	Bozic	Inf	last	wholeT_0.5	J1:DiP, J1:DiP-A, J1:OT, J1:OT-A, J5:DiP, J5:DiP-A, J5:OT-A, S5:DiP, S5:DiP-A, S5:OT, S5:OT-A
730	No	7	7-B	1000	Bozic	Inf	unif	singleC	J1:CBN, J1:CBN-A, J5:CBN, J5:CBN-A, J5:DiP, J5:DiP-A, J5:OT, J5:OT-A, S1:CBN, S1:CBN-A, S5:CBN, S5:CBN-A
731	No	7	7-B	1000	Bozic	Inf	unif	wholeT_0.01	J5:CBN, J5:CBN-A, S5:CBN, S5:CBN-A
732	No	7	7-B	1000	Bozic	Inf	unif	wholeT_0.5	J1:CBN, J1:CBN-A, J1:DiP, J1:DiP-A, J1:OT, J1:OT-A, J5:CBN, J5:CBN-A, J5:DiP, J5:DiP-A, J5:OT, J5:OT-A, S1:CBN, S1:CBN-A, S5:CBN, S5:CBN-A, S5:DiP, S5:DiP-A, S5:OT, S5:OT-A
733	No	7	7-B	1000	exp	0	last	singleC	J1:CBN, J1:CBN-A, J1:DiP, J1:DiP-A, J1:OT, J1:OT-A, J5:CBN, J5:CBN-A, J5:DiP, J5:DiP-A, J5:OT, J5:OT-A, S1:DiP, S1:DiP-A, S1:OT, S1:OT-A, S5:CBN, S5:CBN-A, S5:DiP, S5:DiP-A, S5:OT, S5:OT-A
734	No	7	7-B	1000	exp	0	last	wholeT_0.01	J1:CBN, J1:CBN-A, J1:DiP, J1:DiP-A, J1:OT, J1:OT-A, J5:CBN, J5:CBN-A, J5:DiP, J5:DiP-A, J5:OT, J5:OT-A, S1:CBN, S1:CBN-A, S1:DiP, S1:DiP-A, S1:OT, S1:OT-A, S5:CBN, S5:DiP, S5:DiP-A, S5:OT, S5:OT-A
735	No	7	7-B	1000	exp	0	last	wholeT_0.5	J1:CBN, J1:CBN-A, J1:DiP, J1:DiP-A, J1:OT, J1:OT-A, J5:CBN, J5:CBN-A, J5:DiP, J5:DiP-A, J5:OT, J5:OT-A, S1:DiP, S1:OT, S1:OT-A, S5:CBN, S5:CBN-A, S5:DiP, S5:DiP-A, S5:OT, S5:OT-A
736	No	7	7-B	1000	exp	0	unif	singleC	J1:CBN, J1:CBN-A, J1:DiP, J1:DiP-A, J1:OT, J1:OT-A, J5:CBN, J5:CBN-A, J5:DiP, J5:DiP-A, J5:OT, J5:OT-A, S1:CBN, S1:CBN-A, S1:DiP, S1:DiP-A, S1:OT, S1:OT-A, S5:CBN, S5:CBN-A, S5:DiP, S5:DiP-A, S5:OT-A

Table 16: *(continued)*

	Conjunction	Drivers	Tree	S.Size	Model	sh	S.Time	S.Type	Best method(s)
737	No	7	7-B	1000	exp	0	unif	wholeT_0.01	J1:CBN, J1:CBN-A, J1:DiP, J1:DiP-A, J1:OT, J1:OT-A, J5:CBN, J5:CBN-A, J5:DiP, J5:DiP-A, J5:OT, J5:OT-A, S5:CBN, S5:CBN-A, S5:DiP, S5:DiP-A, S5:OT, S5:OT-A
738	No	7	7-B	1000	exp	0	unif	wholeT_0.5	none
739	No	7	7-B	1000	exp	Inf	last	singleC	J1:DiP, J1:OT, J1:OT-A, J5:CBN-A, J5:DiP, J5:DiP-A, J5:OT, J5:OT-A, S1:DiP, S1:DiP-A, S1:OT-A, S5:DiP, S5:DiP-A, S5:OT, S5:OT-A
740	No	7	7-B	1000	exp	Inf	last	wholeT_0.01	J1:CBN, J1:CBN-A, J1:OT, J1:OT-A, J5:CBN, J5:CBN-A, J5:DiP, J5:DiP-A, J5:OT, J5:OT-A, S5:CBN, S5:OT, S5:OT-A
741	No	7	7-B	1000	exp	Inf	last	wholeT_0.5	J1:DiP, J1:DiP-A, J1:OT, J1:OT-A, J5:CBN, J5:CBN-A, J5:DiP, J5:DiP-A, J5:OT, J5:OT-A, S1:DiP, S1:DiP-A, S1:OT, S1:OT-A, S5:DiP, S5:DiP-A, S5:OT, S5:OT-A
742	No	7	7-B	1000	exp	Inf	unif	singleC	J1:CBN, J1:CBN-A, J1:DiP, J1:DiP-A, J1:OT, J1:OT-A, J5:CBN, J5:CBN-A, J5:DiP, J5:DiP-A, J5:OT, J5:OT-A, S1:CBN, S1:CBN-A, S5:CBN, S5:CBN-A, S5:DiP, S5:DiP-A, S5:OT, S5:OT-A
743	No	7	7-B	1000	exp	Inf	unif	wholeT_0.01	J1:CBN, J1:CBN-A, J5:CBN, J5:CBN-A, J5:DiP-A, J5:OT, J5:OT-A, S5:CBN, S5:CBN-A
744	No	7	7-B	1000	exp	Inf	unif	wholeT_0.5	J1:CBN, J1:CBN-A, J1:DiP, J1:DiP-A, J1:OT, J1:OT-A, J5:CBN, J5:CBN-A, J5:DiP, J5:DiP-A, J5:OT, J5:OT-A, S1:CBN, S1:CBN-A, S1:DiP, S5:CBN, S5:CBN-A, S5:DiP, S5:DiP-A, S5:OT
745	No	7	7-B	1000	McF_4	0	last	singleC	J1:DiP, J1:DiP-A, J1:OT, J1:OT-A, J5:DiP, J5:DiP-A, J5:OT, J5:OT-A, S1:DiP, S1:DiP-A, S1:OT, S1:OT-A, S5:DiP, S5:DiP-A, S5:OT, S5:OT-A
746	No	7	7-B	1000	McF_4	0	last	wholeT_0.01	J1:CBN-A, J1:DiP, J1:DiP-A, J1:OT, J1:OT-A, J5:CBN-A, J5:DiP, J5:DiP-A, J5:OT, J5:OT-A, S1:DiP, S1:DiP-A, S5:DiP, S5:DiP-A, S5:OT, S5:OT-A

Table 16: *(continued)*

	Conjunction	Drivers	Tree	S.Size	Model	sh	S.Time	S.Type	Best method(s)
747	No	7	7-B	1000	McF_4	0	last	wholeT_0.5	J1:DiP, J1:DiP-A, J1:OT-A, J5:DiP, J5:DiP-A, J5:OT, J5:OT-A, S1:DiP, S1:DiP-A, S1:OT, S1:OT-A, S5:DiP, S5:DiP-A, S5:OT, S5:OT-A
748	No	7	7-B	1000	McF_4	0	unif	singleC	J1:CBN, J1:CBN-A, J1:DiP, J1:OT, J1:OT-A, J5:CBN, J5:CBN-A, J5:DiP, J5:DiP-A, J5:OT, J5:OT-A, S1:DiP, S1:DiP-A, S1:OT, S1:OT-A, S5:CBN, S5:CBN-A, S5:DiP, S5:DiP-A, S5:OT, S5:OT-A
749	No	7	7-B	1000	McF_4	0	unif	wholeT_0.01	J1:CBN, J1:CBN-A, J1:DiP-A, J1:OT-A, J5:CBN, J5:CBN-A, J5:DiP, J5:DiP-A, J5:OT, J5:OT-A, S1:DiP, S5:CBN, S5:CBN-A, S5:DiP, S5:DiP-A, S5:OT, S5:OT-A
750	No	7	7-B	1000	McF_4	0	unif	wholeT_0.5	J1:CBN, J1:CBN-A, J1:DiP, J1:DiP-A, J1:OT, J1:OT-A, J5:CBN, J5:CBN-A, J5:DiP, J5:DiP-A, J5:OT, S1:DiP, S1:DiP-A, S1:OT, S5:CBN-A, S5:DiP, S5:DiP-A, S5:OT, S5:OT-A
751	No	7	7-B	1000	McF_4	Inf	last	singleC	J1:DiP, J1:DiP-A, J1:OT, J1:OT-A, J5:DiP, J5:DiP-A, J5:OT, J5:OT-A, S1:DiP, S1:DiP-A, S1:OT, S1:OT-A, S5:DiP, S5:DiP-A, S5:OT, S5:OT-A
752	No	7	7-B	1000	McF_4	Inf	last	wholeT_0.01	J1:DiP, J1:DiP-A, J1:OT, J1:OT-A, J5:DiP, J5:DiP-A, J5:OT, J5:OT-A, S5:DiP, S5:DiP-A, S5:OT, S5:OT-A
753	No	7	7-B	1000	McF_4	Inf	last	wholeT_0.5	J1:DiP, J1:DiP-A, J1:OT, J1:OT-A, J5:DiP, J5:DiP-A, J5:OT, J5:OT-A, S1:DiP, S1:DiP-A, S1:OT, S1:OT-A, S5:DiP, S5:DiP-A, S5:OT, S5:OT-A
754	No	7	7-B	1000	McF_4	Inf	unif	singleC	J1:CBN, J1:CBN-A, J1:DiP, J1:OT, J1:OT-A, J5:CBN, J5:CBN-A, J5:DiP, J5:DiP-A, J5:OT, J5:OT-A, S1:DiP, S1:DiP-A, S1:OT, S1:OT-A, S5:CBN, S5:CBN-A, S5:DiP, S5:DiP-A, S5:OT, S5:OT-A

Table 16: *(continued)*

	Conjunction	Drivers	Tree	S.Size	Model	sh	S.Time	S.Type	Best method(s)
755	No	7	7-B	1000	McF_4	Inf	unif	wholeT_0.01	J1:CBN, J1:CBN-A, J1:DiP, J1:DiP-A, J1:OT, J1:OT-A, J5:CBN, J5:CBN-A, J5:DiP, J5:DiP-A, J5:OT, J5:OT-A, S5:CBN, S5:CBN-A, S5:DiP, S5:DiP-A, S5:OT, S5:OT-A
756	No	7	7-B	1000	McF_4	Inf	unif	wholeT_0.5	J1:CBN, J1:CBN-A, J1:DiP, J1:DiP-A, J1:OT, J1:OT-A, J5:CBN-A, J5:DiP-A, J5:OT, S1:DiP, S1:OT, S1:OT-A, S5:CBN, S5:CBN-A, S5:DiP, S5:OT, S5:OT-A
757	No	7	7-B	1000	McF_6	0	last	singleC	J1:DiP, J1:DiP-A, J1:OT, J1:OT-A, J5:DiP, J5:DiP-A, J5:OT, J5:OT-A, S1:DiP, S1:DiP-A, S5:DiP, S5:DiP-A, S5:OT, S5:OT-A
758	No	7	7-B	1000	McF_6	0	last	wholeT_0.01	J1:DiP, J1:DiP-A, J5:DiP, J5:DiP-A, J5:OT, J5:OT-A, S1:DiP, S1:DiP-A, S5:OT, S5:OT-A
759	No	7	7-B	1000	McF_6	0	last	wholeT_0.5	J1:DiP, J1:DiP-A, J1:OT, J1:OT-A, J5:DiP, J5:DiP-A, J5:OT, J5:OT-A, S1:DiP, S1:DiP-A, S1:OT, S1:OT-A, S5:DiP, S5:DiP-A, S5:OT, S5:OT-A
760	No	7	7-B	1000	McF_6	0	unif	singleC	none
761	No	7	7-B	1000	McF_6	0	unif	wholeT_0.01	J1:CBN, J1:CBN-A, J1:DiP, J1:DiP-A, J1:OT, J1:OT-A, J5:CBN, J5:CBN-A, J5:DiP, J5:DiP-A, J5:OT, J5:OT-A, S5:CBN, S5:CBN-A, S5:DiP, S5:DiP-A, S5:OT, S5:OT-A
762	No	7	7-B	1000	McF_6	0	unif	wholeT_0.5	none
763	No	7	7-B	1000	McF_6	Inf	last	singleC	J1:DiP, J1:DiP-A, J1:OT, J1:OT-A, J5:DiP, J5:DiP-A, J5:OT, J5:OT-A, S1:DiP, S1:DiP-A, S5:DiP, S5:DiP-A, S5:OT, S5:OT-A
764	No	7	7-B	1000	McF_6	Inf	last	wholeT_0.01	J1:DiP, J1:DiP-A, J5:DiP, J5:DiP-A, J5:OT, J5:OT-A, S1:DiP, S1:DiP-A, S5:DiP, S5:DiP-A, S5:OT, S5:OT-A
765	No	7	7-B	1000	McF_6	Inf	last	wholeT_0.5	J1:DiP, J1:DiP-A, J1:OT, J1:OT-A, J5:DiP, J5:DiP-A, J5:OT, J5:OT-A, S1:DiP, S1:DiP-A, S1:OT-A, S5:DiP, S5:DiP-A, S5:OT, S5:OT-A
766	No	7	7-B	1000	McF_6	Inf	unif	singleC	none

Table 16: *(continued)*

	Conjunction	Drivers	Tree	S.Size	Model	sh	S.Time	S.Type	Best method(s)
767	No	7	7-B	1000	McF_6	Inf	unif	wholeT_0.01	J1:CBN, J1:CBN-A, J1:DiP, J1:DiP-A, J1:OT, J1:OT-A, J5:CBN, J5:CBN-A, J5:DiP, J5:DiP-A, J5:OT-A, S5:CBN, S5:CBN-A, S5:DiP, S5:DiP-A, S5:OT
768	No	7	7-B	1000	McF_6	Inf	unif	wholeT_0.5	none
769	No	7	7-B	200	Bozic	0	last	singleC	J1:CBN, J1:CBN-A, J1:DiP, J1:DiP-A, J1:OT, J1:OT-A, J5:CBN, J5:CBN-A, J5:DiP, J5:DiP-A, J5:OT, J5:OT-A, S1:DiP, S1:DiP-A, S5:DiP, S5:DiP-A, S5:OT, S5:OT-A
770	No	7	7-B	200	Bozic	0	last	wholeT_0.01	J1:CBN, J1:CBN-A, J1:DiP, J1:DiP-A, J1:OT, J1:OT-A, J5:CBN, J5:CBN-A, J5:DiP, J5:DiP-A, J5:OT, J5:OT-A, S1:DiP, S1:DiP-A, S5:CBN, S5:CBN-A, S5:DiP, S5:DiP-A, S5:OT, S5:OT-A
771	No	7	7-B	200	Bozic	0	last	wholeT_0.5	J1:CBN, J1:CBN-A, J1:DiP, J1:DiP-A, J1:OT, J1:OT-A, J5:CBN, J5:CBN-A, J5:DiP, J5:DiP-A, J5:OT, J5:OT-A, S1:DiP, S1:DiP-A, S1:OT, S5:CBN, S5:DiP, S5:DiP-A, S5:OT, S5:OT-A
772	No	7	7-B	200	Bozic	0	unif	singleC	J1:CBN, J1:CBN-A, J1:DiP, J1:DiP-A, J1:OT, J1:OT-A, J5:CBN, J5:CBN-A, J5:DiP, J5:DiP-A, J5:OT, J5:OT-A, S1:DiP, S1:DiP-A, S1:OT-A, S5:CBN-A, S5:DiP-A, S5:OT, S5:OT-A
773	No	7	7-B	200	Bozic	0	unif	wholeT_0.01	J5:CBN, J5:CBN-A, J5:DiP, J5:DiP-A, J5:OT, J5:OT-A
774	No	7	7-B	200	Bozic	0	unif	wholeT_0.5	J1:CBN, J1:CBN-A, J1:DiP, J1:DiP-A, J1:OT, J1:OT-A, J5:CBN, J5:CBN-A, J5:DiP, J5:DiP-A, J5:OT, J5:OT-A, S1:CBN-A, S1:DiP, S1:DiP-A, S1:OT, S1:OT-A, S5:CBN, S5:CBN-A, S5:DiP, S5:DiP-A, S5:OT, S5:OT-A
775	No	7	7-B	200	Bozic	Inf	last	singleC	J1:DiP, J1:DiP-A, J5:CBN, J5:CBN-A, J5:DiP, J5:DiP-A, J5:OT, J5:OT-A, S5:DiP, S5:DiP-A, S5:OT-A
776	No	7	7-B	200	Bozic	Inf	last	wholeT_0.01	J5:CBN, J5:CBN-A, J5:DiP, J5:OT, J5:OT-A

Table 16: *(continued)*

	Conjunction	Drivers	Tree	S.Size	Model	sh	S.Time	S.Type	Best method(s)
777	No	7	7-B	200	Bozic	Inf	last	wholeT_0.5	J1:DiP, J1:DiP-A, J1:OT-A, J5:CBN, J5:CBN-A, J5:DiP, J5:DiP-A, J5:OT, J5:OT-A, S1:DiP, S5:DiP, S5:DiP-A, S5:OT, S5:OT-A
778	No	7	7-B	200	Bozic	Inf	unif	singleC	J1:CBN, J1:CBN-A, J5:CBN, J5:CBN-A, J5:DiP, J5:DiP-A, J5:OT, J5:OT-A, S5:CBN, S5:CBN-A, S5:DiP, S5:DiP-A, S5:OT-A
779	No	7	7-B	200	Bozic	Inf	unif	wholeT_0.01	J5:CBN, J5:CBN-A, J5:OT, J5:OT-A, S5:CBN, S5:CBN-A, S5:OT, S5:OT-A
780	No	7	7-B	200	Bozic	Inf	unif	wholeT_0.5	J1:CBN, J1:CBN-A, J1:DiP, J5:CBN, J5:CBN-A, J5:DiP, J5:DiP-A, J5:OT, J5:OT-A, S5:CBN, S5:CBN-A, S5:DiP, S5:DiP-A, S5:OT, S5:OT-A
781	No	7	7-B	200	exp	0	last	singleC	J1:CBN, J1:CBN-A, J1:DiP, J1:DiP-A, J1:OT, J1:OT-A, J5:CBN, J5:CBN-A, J5:DiP, J5:DiP-A, J5:OT, J5:OT-A, S1:DiP, S1:DiP-A, S1:OT, S1:OT-A, S5:CBN, S5:CBN-A, S5:DiP, S5:DiP-A, S5:OT, S5:OT-A
782	No	7	7-B	200	exp	0	last	wholeT_0.01	J1:CBN, J1:CBN-A, J1:DiP, J1:DiP-A, J1:OT, J1:OT-A, J5:CBN, J5:CBN-A, J5:DiP, J5:DiP-A, J5:OT, J5:OT-A, S1:CBN, S1:DiP, S1:DiP-A, S5:CBN, S5:CBN-A, S5:DiP, S5:DiP-A
783	No	7	7-B	200	exp	0	last	wholeT_0.5	J1:CBN, J1:CBN-A, J1:DiP, J1:DiP-A, J1:OT, J1:OT-A, J5:CBN, J5:CBN-A, J5:DiP, J5:DiP-A, J5:OT, J5:OT-A, S1:CBN-A, S1:DiP, S1:DiP-A, S1:OT, S1:OT-A, S5:CBN, S5:CBN-A, S5:DiP, S5:DiP-A, S5:OT, S5:OT-A
784	No	7	7-B	200	exp	0	unif	singleC	none
785	No	7	7-B	200	exp	0	unif	wholeT_0.01	J1:CBN, J1:DiP, J1:DiP-A, J1:OT, J1:OT-A, J5:CBN, J5:CBN-A, J5:DiP, J5:DiP-A, J5:OT, J5:OT-A, S1:DiP, S5:CBN, S5:CBN-A, S5:DiP, S5:DiP-A, S5:OT, S5:OT-A
786	No	7	7-B	200	exp	0	unif	wholeT_0.5	none

Table 16: *(continued)*

	Conjunction	Drivers	Tree	S.Size	Model	sh	S.Time	S.Type	Best method(s)
787	No	7	7-B	200	exp	Inf	last	singleC	J1:DiP, J1:DiP-A, J1:OT-A, J5:CBN, J5:CBN-A, J5:DiP, J5:OT, J5:OT-A, S1:DiP, S1:DiP-A, S5:DiP, S5:DiP-A, S5:OT, S5:OT-A
788	No	7	7-B	200	exp	Inf	last	wholeT_0.01	J5:CBN, J5:CBN-A, J5:DiP, J5:DiP-A, J5:OT, J5:OT-A, S5:CBN-A, S5:DiP
789	No	7	7-B	200	exp	Inf	last	wholeT_0.5	J1:DiP, J1:DiP-A, J1:OT, J1:OT-A, J5:CBN, J5:CBN-A, J5:DiP, J5:DiP-A, J5:OT, J5:OT-A, S1:DiP, S1:DiP-A, S5:DiP, S5:DiP-A, S5:OT, S5:OT-A
790	No	7	7-B	200	exp	Inf	unif	singleC	J1:CBN, J1:CBN-A, J1:DiP, J1:DiP-A, J1:OT, J1:OT-A, J5:CBN, J5:CBN-A, J5:DiP, J5:DiP-A, J5:OT, J5:OT-A, S5:CBN, S5:CBN-A, S5:DiP, S5:DiP-A, S5:OT, S5:OT-A
791	No	7	7-B	200	exp	Inf	unif	wholeT_0.01	J5:CBN, J5:CBN-A, J5:DiP-A, J5:OT, J5:OT-A, S5:CBN, S5:CBN-A, S5:OT
792	No	7	7-B	200	exp	Inf	unif	wholeT_0.5	J1:CBN, J1:CBN-A, J1:DiP, J1:DiP-A, J1:OT, J1:OT-A, J5:CBN, J5:CBN-A, J5:DiP, J5:DiP-A, J5:OT, J5:OT-A, S1:CBN, S1:CBN-A, S1:DiP, S1:DiP-A, S5:CBN, S5:CBN-A, S5:DiP, S5:DiP-A, S5:OT, S5:OT-A
793	No	7	7-B	200	McF_4	0	last	singleC	J1:DiP, J1:DiP-A, J1:OT, J1:OT-A, J5:DiP, J5:DiP-A, J5:OT, J5:OT-A, S1:DiP, S1:DiP-A, S5:DiP, S5:DiP-A, S5:OT, S5:OT-A
794	No	7	7-B	200	McF_4	0	last	wholeT_0.01	J1:DiP, J1:DiP-A, J1:OT-A, J5:DiP, J5:DiP-A, J5:OT, J5:OT-A, S1:DiP, S1:DiP-A, S5:DiP-A, S5:OT, S5:OT-A
795	No	7	7-B	200	McF_4	0	last	wholeT_0.5	J1:DiP, J1:DiP-A, J1:OT, J1:OT-A, J5:DiP, J5:DiP-A, J5:OT, J5:OT-A, S1:DiP, S1:DiP-A, S5:DiP, S5:DiP-A, S5:OT, S5:OT-A
796	No	7	7-B	200	McF_4	0	unif	singleC	J1:CBN, J1:CBN-A, J1:DiP, J1:DiP-A, J1:OT, J1:OT-A, J5:CBN, J5:CBN-A, J5:DiP, J5:DiP-A, J5:OT, J5:OT-A, S1:DiP, S5:CBN-A, S5:DiP, S5:DiP-A, S5:OT, S5:OT-A

Table 16: *(continued)*

	Conjunction	Drivers	Tree	S.Size	Model	sh	S.Time	S.Type	Best method(s)
797	No	7	7-B	200	McF_4	0	unif	wholeT_0.01	J1:CBN, J1:CBN-A, J1:DiP, J1:DiP-A, J1:OT, J1:OT-A, J5:CBN, J5:CBN-A, J5:DiP, J5:DiP-A, J5:OT, J5:OT-A, S5:CBN, S5:CBN-A, S5:DiP, S5:DiP-A, S5:OT, S5:OT-A
798	No	7	7-B	200	McF_4	0	unif	wholeT_0.5	J1:CBN, J1:CBN-A, J1:DiP, J1:DiP-A, J1:OT, J5:CBN, J5:CBN-A, J5:DiP, J5:DiP-A, J5:OT, J5:OT-A, S1:DiP, S5:CBN, S5:CBN-A, S5:DiP, S5:DiP-A, S5:OT, S5:OT-A
799	No	7	7-B	200	McF_4	Inf	last	singleC	J1:DiP, J1:DiP-A, J1:OT, J1:OT-A, J5:DiP, J5:DiP-A, J5:OT, J5:OT-A, S1:DiP, S1:DiP-A, S5:DiP, S5:DiP-A, S5:OT, S5:OT-A
800	No	7	7-B	200	McF_4	Inf	last	wholeT_0.01	J1:DiP, J1:DiP-A, J1:OT-A, J5:DiP, J5:DiP-A, J5:OT, J5:OT-A, S1:DiP, S1:DiP-A, S5:DiP, S5:DiP-A, S5:OT, S5:OT-A
801	No	7	7-B	200	McF_4	Inf	last	wholeT_0.5	J1:DiP, J1:DiP-A, J1:OT, J1:OT-A, J5:DiP, J5:DiP-A, J5:OT, J5:OT-A, S1:DiP, S1:DiP-A, S5:DiP, S5:DiP-A, S5:OT-A
802	No	7	7-B	200	McF_4	Inf	unif	singleC	J1:CBN, J1:CBN-A, J1:DiP, J1:DiP-A, J1:OT, J1:OT-A, J5:CBN, J5:CBN-A, J5:DiP, J5:DiP-A, J5:OT, J5:OT-A, S1:DiP, S1:DiP-A, S5:CBN, S5:CBN-A, S5:DiP, S5:DiP-A, S5:OT, S5:OT-A
803	No	7	7-B	200	McF_4	Inf	unif	wholeT_0.01	J1:CBN, J1:CBN-A, J1:DiP, J1:DiP-A, J1:OT, J1:OT-A, J5:CBN, J5:CBN-A, J5:DiP, J5:DiP-A, J5:OT-A, S1:DiP, S5:CBN, S5:CBN-A, S5:DiP, S5:DiP-A, S5:OT
804	No	7	7-B	200	McF_4	Inf	unif	wholeT_0.5	J1:CBN, J1:CBN-A, J1:DiP, J1:DiP-A, J1:OT, J1:OT-A, J5:CBN, J5:CBN-A, J5:DiP, J5:DiP-A, J5:OT, J5:OT-A, S1:DiP, S1:DiP-A, S5:CBN, S5:DiP, S5:DiP-A, S5:OT, S5:OT-A

Table 16: *(continued)*

	Conjunction	Drivers	Tree	S.Size	Model	sh	S.Time	S.Type	Best method(s)
805	No	7	7-B	200	McF_6	0	last	singleC	J1:DiP, J1:DiP-A, J1:OT, J1:OT-A, J5:DiP, J5:DiP-A, J5:OT, J5:OT-A, S1:DiP, S1:DiP-A, S5:DiP, S5:DiP-A, S5:OT, S5:OT-A
806	No	7	7-B	200	McF_6	0	last	wholeT_0.01	J1:DiP, J1:DiP-A, J5:DiP, J5:DiP-A, J5:OT, J5:OT-A, S1:DiP, S1:DiP-A, S5:DiP, S5:DiP-A, S5:OT, S5:OT-A
807	No	7	7-B	200	McF_6	0	last	wholeT_0.5	J1:DiP, J1:DiP-A, J1:OT, J5:DiP, J5:DiP-A, J5:OT, J5:OT-A, S1:DiP, S1:DiP-A, S5:DiP, S5:DiP-A, S5:OT, S5:OT-A
808	No	7	7-B	200	McF_6	0	unif	singleC	J1:CBN, J1:CBN-A, J1:DiP, J1:OT-A, J5:CBN, J5:CBN-A, J5:DiP, J5:DiP-A, J5:OT, J5:OT-A, S1:DiP, S1:DiP-A, S5:CBN, S5:CBN-A, S5:DiP, S5:DiP-A, S5:OT, S5:OT-A
809	No	7	7-B	200	McF_6	0	unif	wholeT_0.01	J1:CBN, J1:CBN-A, J1:DiP, J1:DiP-A, J1:OT, J5:CBN, J5:CBN-A, J5:DiP, J5:OT, J5:OT-A, S1:DiP, S1:DiP-A, S5:CBN-A, S5:DiP, S5:DiP-A, S5:OT, S5:OT-A
810	No	7	7-B	200	McF_6	0	unif	wholeT_0.5	J1:CBN, J1:CBN-A, J1:DiP, J5:CBN, J5:CBN-A, J5:DiP, J5:DiP-A, J5:OT, S1:DiP, S5:CBN, S5:CBN-A, S5:OT, S5:OT-A
811	No	7	7-B	200	McF_6	Inf	last	singleC	J1:DiP, J1:DiP-A, J1:OT, J1:OT-A, J5:DiP, J5:DiP-A, J5:OT, J5:OT-A, S1:DiP, S1:DiP-A, S5:DiP, S5:DiP-A, S5:OT, S5:OT-A
812	No	7	7-B	200	McF_6	Inf	last	wholeT_0.01	J1:DiP, J5:DiP, J5:OT, J5:OT-A, S1:DiP, S5:DiP, S5:OT, S5:OT-A
813	No	7	7-B	200	McF_6	Inf	last	wholeT_0.5	J1:DiP, J1:DiP-A, J1:OT, J1:OT-A, J5:DiP, J5:DiP-A, J5:OT, J5:OT-A, S1:DiP, S5:DiP, S5:DiP-A, S5:OT, S5:OT-A
814	No	7	7-B	200	McF_6	Inf	unif	singleC	J1:CBN, J1:CBN-A, J1:DiP, J1:DiP-A, J1:OT, J1:OT-A, J5:CBN, J5:CBN-A, J5:DiP, J5:DiP-A, J5:OT, S5:CBN, S5:CBN-A, S5:OT, S5:OT-A

Table 16: *(continued)*

	Conjunction	Drivers	Tree	S.Size	Model	sh	S.Time	S.Type	Best method(s)
815	No	7	7-B	200	McF_6	Inf	unif	wholeT_0.01	J1:DiP, J5:CBN, J5:CBN-A, J5:DiP, J5:DiP-A, S1:DiP, S5:CBN, S5:CBN-A, S5:DiP-A
816	No	7	7-B	200	McF_6	Inf	unif	wholeT_0.5	J1:CBN, J1:CBN-A, J1:DiP, J1:DiP-A, J5:CBN, J5:CBN-A, J5:DiP, J5:DiP-A, S5:CBN, S5:CBN-A, S5:DiP, S5:OT-A
817	No	7	7-B	100	Bozic	0	last	singleC	J1:DiP, J1:DiP-A, J5:CBN, J5:CBN-A, J5:DiP, J5:DiP-A, J5:OT, J5:OT-A, S1:DiP, S1:DiP-A, S5:DiP, S5:DiP-A, S5:OT, S5:OT-A
818	No	7	7-B	100	Bozic	0	last	wholeT_0.01	J1:DiP, J1:DiP-A, J5:CBN, J5:CBN-A, J5:DiP, J5:DiP-A, J5:OT, J5:OT-A, S1:DiP, S1:DiP-A, S5:CBN, S5:CBN-A, S5:DiP, S5:DiP-A, S5:OT, S5:OT-A
819	No	7	7-B	100	Bozic	0	last	wholeT_0.5	J1:DiP, J1:DiP-A, J5:CBN, J5:DiP, J5:OT-A, S1:DiP, S1:DiP-A, S5:CBN, S5:CBN-A, S5:DiP, S5:DiP-A, S5:OT, S5:OT-A
820	No	7	7-B	100	Bozic	0	unif	singleC	J1:CBN, J1:DiP, J1:DiP-A, J1:OT, J1:OT-A, J5:CBN, J5:CBN-A, J5:DiP, J5:DiP-A, J5:OT, J5:OT-A, S1:DiP, S1:DiP-A, S5:CBN, S5:CBN-A, S5:DiP, S5:DiP-A, S5:OT, S5:OT-A
821	No	7	7-B	100	Bozic	0	unif	wholeT_0.01	J5:CBN, J5:CBN-A, J5:DiP, J5:DiP-A, J5:OT, J5:OT-A
822	No	7	7-B	100	Bozic	0	unif	wholeT_0.5	J1:CBN, J1:DiP, J1:DiP-A, J1:OT, J1:OT-A, J5:CBN, J5:CBN-A, J5:DiP, J5:DiP-A, J5:OT, J5:OT-A, S1:DiP, S1:DiP-A, S5:CBN, S5:CBN-A, S5:DiP, S5:DiP-A, S5:OT
823	No	7	7-B	100	Bozic	Inf	last	singleC	J1:DiP, J1:DiP-A, J5:CBN, J5:CBN-A, J5:DiP, J5:DiP-A, J5:OT, J5:OT-A, S1:DiP, S1:DiP-A, S5:DiP, S5:DiP-A
824	No	7	7-B	100	Bozic	Inf	last	wholeT_0.01	J5:CBN, J5:CBN-A, J5:DiP
825	No	7	7-B	100	Bozic	Inf	last	wholeT_0.5	J1:DiP, J1:DiP-A, J5:CBN, J5:CBN-A, J5:DiP, J5:DiP-A, J5:OT, J5:OT-A, S1:DiP, S1:DiP-A, S5:DiP, S5:DiP-A
826	No	7	7-B	100	Bozic	Inf	unif	singleC	J5:CBN, J5:CBN-A, J5:DiP, J5:DiP-A, J5:OT, J5:OT-A, S5:CBN, S5:CBN-A, S5:DiP

Table 16: *(continued)*

	Conjunction	Drivers	Tree	S.Size	Model	sh	S.Time	S.Type	Best method(s)
827	No	7	7-B	100	Bozic	Inf	unif	wholeT_0.01	J5:CBN, J5:CBN-A, J5:OT, J5:OT-A, S5:CBN
828	No	7	7-B	100	Bozic	Inf	unif	wholeT_0.5	J5:CBN, J5:CBN-A, J5:DiP, J5:DiP-A, J5:OT, J5:OT-A, S5:CBN, S5:DiP, S5:DiP-A, S5:OT, S5:OT-A
829	No	7	7-B	100	exp	0	last	singleC	J1:CBN, J1:CBN-A, J1:DiP, J1:DiP-A, J1:OT, J1:OT-A, J5:CBN, J5:CBN-A, J5:DiP, J5:DiP-A, J5:OT, J5:OT-A, S1:DiP, S1:DiP-A, S1:OT, S1:OT-A, S5:CBN, S5:CBN-A, S5:DiP, S5:DiP-A, S5:OT, S5:OT-A
830	No	7	7-B	100	exp	0	last	wholeT_0.01	J1:CBN, J1:CBN-A, J1:DiP, J1:DiP-A, J5:CBN, J5:CBN-A, J5:DiP, J5:DiP-A, J5:OT, J5:OT-A, S1:DiP, S1:DiP-A, S5:CBN, S5:CBN-A, S5:DiP, S5:DiP-A
831	No	7	7-B	100	exp	0	last	wholeT_0.5	J1:CBN, J1:CBN-A, J1:DiP, J1:DiP-A, J1:OT, J1:OT-A, J5:CBN, J5:CBN-A, J5:DiP, J5:DiP-A, J5:OT, J5:OT-A, S1:DiP, S1:DiP-A, S1:OT, S5:CBN, S5:CBN-A, S5:DiP, S5:DiP-A, S5:OT, S5:OT-A
832	No	7	7-B	100	exp	0	unif	singleC	J1:CBN, J1:CBN-A, J1:DiP, J1:DiP-A, J1:OT, J1:OT-A, J5:CBN, J5:CBN-A, J5:DiP, J5:DiP-A, J5:OT, J5:OT-A, S1:DiP, S1:DiP-A, S1:OT-A, S5:CBN, S5:CBN-A, S5:DiP, S5:DiP-A, S5:OT
833	No	7	7-B	100	exp	0	unif	wholeT_0.01	J1:DiP, J1:DiP-A, J5:CBN, J5:CBN-A, J5:DiP, J5:DiP-A, J5:OT, J5:OT-A, S1:DiP, S1:DiP-A, S5:CBN, S5:CBN-A, S5:DiP, S5:DiP-A, S5:OT, S5:OT-A
834	No	7	7-B	100	exp	0	unif	wholeT_0.5	J1:CBN-A, J1:DiP, J1:DiP-A, J1:OT, J1:OT-A, J5:CBN, J5:CBN-A, J5:DiP, J5:DiP-A, J5:OT, J5:OT-A, S1:CBN, S1:CBN-A, S1:DiP, S1:DiP-A, S1:OT-A, S5:CBN, S5:CBN-A, S5:DiP, S5:DiP-A, S5:OT, S5:OT-A
835	No	7	7-B	100	exp	Inf	last	singleC	J1:DiP, J1:DiP-A, J1:OT, J1:OT-A, J5:CBN, J5:CBN-A, J5:DiP, J5:DiP-A, J5:OT, J5:OT-A, S1:DiP, S1:DiP-A, S5:DiP, S5:DiP-A, S5:OT, S5:OT-A

Table 16: *(continued)*

	Conjunction	Drivers	Tree	S.Size	Model	sh	S.Time	S.Type	Best method(s)
836	No	7	7-B	100	exp	Inf	last	wholeT_0.01	J5:DiP, J5:DiP-A
837	No	7	7-B	100	exp	Inf	last	wholeT_0.5	J1:DiP, J1:DiP-A, J1:OT, J5:CBN, J5:CBN-A, J5:DiP, J5:DiP-A, J5:OT, J5:OT-A, S1:DiP, S1:DiP-A, S5:DiP, S5:DiP-A, S5:OT, S5:OT-A
838	No	7	7-B	100	exp	Inf	unif	singleC	J1:DiP, J1:DiP-A, J5:CBN, J5:CBN-A, J5:DiP, J5:DiP-A, J5:OT, J5:OT-A, S1:DiP, S1:DiP-A, S5:CBN, S5:CBN-A, S5:DiP, S5:DiP-A, S5:OT, S5:OT-A
839	No	7	7-B	100	exp	Inf	unif	wholeT_0.01	J5:CBN, J5:CBN-A, J5:DiP, J5:DiP-A, J5:OT, J5:OT-A
840	No	7	7-B	100	exp	Inf	unif	wholeT_0.5	J1:DiP, J1:DiP-A, J5:CBN, J5:CBN-A, J5:DiP, J5:DiP-A, J5:OT, J5:OT-A, S1:DiP, S1:DiP-A, S5:CBN, S5:CBN-A, S5:DiP, S5:DiP-A, S5:OT, S5:OT-A
841	No	7	7-B	100	McF_4	0	last	singleC	J1:DiP, J1:DiP-A, J1:OT, J1:OT-A, J5:DiP, J5:DiP-A, J5:OT, J5:OT-A, S1:DiP, S1:DiP-A, S5:DiP, S5:DiP-A, S5:OT, S5:OT-A
842	No	7	7-B	100	McF_4	0	last	wholeT_0.01	J1:DiP, J1:DiP-A, J5:DiP, J5:DiP-A, J5:OT, J5:OT-A, S1:DiP, S5:DiP, S5:DiP-A, S5:OT, S5:OT-A
843	No	7	7-B	100	McF_4	0	last	wholeT_0.5	J1:DiP, J1:DiP-A, J1:OT-A, J5:DiP, J5:DiP-A, J5:OT, J5:OT-A, S1:DiP, S1:DiP-A, S5:DiP, S5:DiP-A, S5:OT, S5:OT-A
844	No	7	7-B	100	McF_4	0	unif	singleC	J1:CBN-A, J1:DiP, J1:DiP-A, J5:CBN, J5:CBN-A, J5:DiP, J5:DiP-A, J5:OT, J5:OT-A, S1:DiP, S1:DiP-A, S5:CBN, S5:CBN-A, S5:DiP, S5:DiP-A
845	No	7	7-B	100	McF_4	0	unif	wholeT_0.01	J1:CBN, J1:CBN-A, J1:DiP, J1:DiP-A, J1:OT, J1:OT-A, J5:CBN, J5:CBN-A, J5:DiP, J5:DiP-A, J5:OT, J5:OT-A, S1:DiP, S1:DiP-A, S5:CBN, S5:DiP, S5:DiP-A, S5:OT, S5:OT-A
846	No	7	7-B	100	McF_4	0	unif	wholeT_0.5	J1:CBN, J1:CBN-A, J1:DiP, J1:DiP-A, J5:CBN, J5:CBN-A, J5:DiP, J5:DiP-A, J5:OT, J5:OT-A, S1:DiP, S1:DiP-A, S5:CBN-A, S5:DiP, S5:DiP-A, S5:OT-A

Table 16: *(continued)*

	Conjunction	Drivers	Tree	S.Size	Model	sh	S.Time	S.Type	Best method(s)
847	No	7	7-B	100	McF_4	Inf	last	singleC	J1:DiP, J1:DiP-A, J1:OT, J1:OT-A, J5:DiP, J5:DiP-A, J5:OT, J5:OT-A, S1:DiP, S1:DiP-A, S5:DiP, S5:DiP-A, S5:OT, S5:OT-A
848	No	7	7-B	100	McF_4	Inf	last	wholeT_0.01	J1:DiP-A, J5:DiP, J5:DiP-A, J5:OT, J5:OT-A, S1:DiP, S1:DiP-A, S5:DiP, S5:DiP-A, S5:OT, S5:OT-A
849	No	7	7-B	100	McF_4	Inf	last	wholeT_0.5	J1:DiP, J1:DiP-A, J5:DiP, J5:DiP-A, J5:OT, J5:OT-A, S1:DiP, S1:DiP-A, S5:DiP, S5:DiP-A, S5:OT, S5:OT-A
850	No	7	7-B	100	McF_4	Inf	unif	singleC	J1:CBN, J1:CBN-A, J1:DiP, J1:DiP-A, J1:OT, J1:OT-A, J5:CBN, J5:CBN-A, J5:DiP, J5:DiP-A, J5:OT, J5:OT-A, S1:DiP, S1:DiP-A, S5:CBN, S5:CBN-A, S5:DiP, S5:DiP-A, S5:OT, S5:OT-A
851	No	7	7-B	100	McF_4	Inf	unif	wholeT_0.01	J1:CBN, J1:CBN-A, J1:DiP, J1:DiP-A, J5:CBN, J5:CBN-A, J5:DiP, J5:DiP-A, J5:OT, J5:OT-A, S1:DiP, S1:DiP-A, S5:CBN-A, S5:DiP, S5:DiP-A, S5:OT, S5:OT-A
852	No	7	7-B	100	McF_4	Inf	unif	wholeT_0.5	J1:CBN, J1:CBN-A, J1:DiP, J1:DiP-A, J5:CBN, J5:CBN-A, J5:DiP, J5:DiP-A, S1:DiP, S1:DiP-A, S5:CBN, S5:DiP, S5:DiP-A
853	No	7	7-B	100	McF_6	0	last	singleC	J1:DiP, J1:DiP-A, J5:DiP, J5:DiP-A, J5:OT, J5:OT-A, S1:DiP, S1:DiP-A, S5:DiP, S5:DiP-A, S5:OT, S5:OT-A
854	No	7	7-B	100	McF_6	0	last	wholeT_0.01	J5:DiP, J5:OT, J5:OT-A, S5:DiP
855	No	7	7-B	100	McF_6	0	last	wholeT_0.5	J1:DiP, J1:DiP-A, J5:DiP, J5:DiP-A, J5:OT, J5:OT-A, S1:DiP, S1:DiP-A, S5:DiP-A, S5:OT, S5:OT-A
856	No	7	7-B	100	McF_6	0	unif	singleC	J1:CBN, J1:CBN-A, J1:DiP, J5:CBN, J5:CBN-A, J5:DiP, J5:DiP-A, S1:DiP, S5:CBN, S5:CBN-A, S5:DiP
857	No	7	7-B	100	McF_6	0	unif	wholeT_0.01	J1:DiP, J1:DiP-A, J5:CBN, J5:CBN-A, J5:DiP, J5:DiP-A, S5:CBN, S5:CBN-A, S5:DiP, S5:DiP-A
858	No	7	7-B	100	McF_6	0	unif	wholeT_0.5	J1:CBN, J1:CBN-A, J5:CBN, J5:CBN-A, J5:DiP, J5:DiP-A, S5:CBN-A

Table 16: *(continued)*

	Conjunction	Drivers	Tree	S.Size	Model	sh	S.Time	S.Type	Best method(s)
859	No	7	7-B	100	McF_6	Inf	last	singleC	J1:DiP, J1:DiP-A, J5:DiP, J5:DiP-A, J5:OT, J5:OT-A, S1:DiP, S1:DiP-A, S5:DiP, S5:DiP-A, S5:OT, S5:OT-A
860	No	7	7-B	100	McF_6	Inf	last	wholeT_0.01	J1:DiP, J5:DiP, J5:OT, J5:OT-A, S1:DiP, S5:DiP
861	No	7	7-B	100	McF_6	Inf	last	wholeT_0.5	J1:DiP-A, J5:DiP, J5:DiP-A, J5:OT, J5:OT-A, S1:DiP, S1:DiP-A, S5:DiP, S5:DiP-A, S5:OT, S5:OT-A
862	No	7	7-B	100	McF_6	Inf	unif	singleC	J1:CBN, J1:CBN-A, J1:DiP, J5:CBN, J5:CBN-A, J5:DiP, J5:DiP-A, S5:CBN, S5:CBN-A, S5:DiP
863	No	7	7-B	100	McF_6	Inf	unif	wholeT_0.01	J1:DiP, J5:CBN, J5:CBN-A, J5:DiP, J5:DiP-A, S5:CBN, S5:CBN-A, S5:DiP, S5:DiP-A
864	No	7	7-B	100	McF_6	Inf	unif	wholeT_0.5	J1:CBN, J1:CBN-A, J1:DiP, J5:CBN, J5:CBN-A, J5:DiP, S5:CBN, S5:CBN-A
