# Supplementary material for: Identifying restrictions in the order of accumulation of mutations during tumor progression: effects of passengers, evolutionary models, and sampling
Source: BMC Bioinformatics. 2015 Feb 12;16:41. doi: 10.1186/s12859-015-0466-7 (PMC4339747; doi:10.1186/s12859-015-0466-7)

Additional file 5. Examples of inferred graphs.

Supplementary material for "Identifying Restrictions in the Order of Accumulation of Mutations during Tumor Progression: Effects of Passengers, Evolutionary Models, and Sampling"

Ramon Diaz-Uriarte
Dept. Biochemistry, Universidad Autónoma de Madrid
Instituto de Investigaciones Biomédicas "Alberto Sols" (UAM-CSIC)
Madrid, Spain

ramon.diaz@iib.uam.es

rdiaz02@gmail.com

http://ligarto.org/rdiaz

Contents

1 Introduction 2

2 Drivers Known 2

3 Drivers Unknown: Filtering = S1 7

4 Drivers Unknown: Filtering = S5 12

5 Drivers Unknown: Filtering = J1 17

6 Drivers Unknown: Filtering = J5 22

1 Introduction

The following figures show examples of inferred graphs, from each of the six methods, for the two scenarios, and under each Model and Graph. The inferred graph shown is one chosen randomly among the 20 possible ones. Across rows (i.e., across Method), the data set is the same, so those correspond to the same data being fitted by different methods.

To minimize the impact of other factors, all figures are for S.Size = 1000, S.Type = whole tumor (0.5), S.Time = last, and sh = Inf.

The original, true graphs, are given in *Additional file 1*. Note that, especially under Filtering = S1, many inferred graphs show nodes that are not really drivers (and that, therefore, should not have been in the graph at all).

2 Drivers Known

Filtering = NP. Model = Bozic

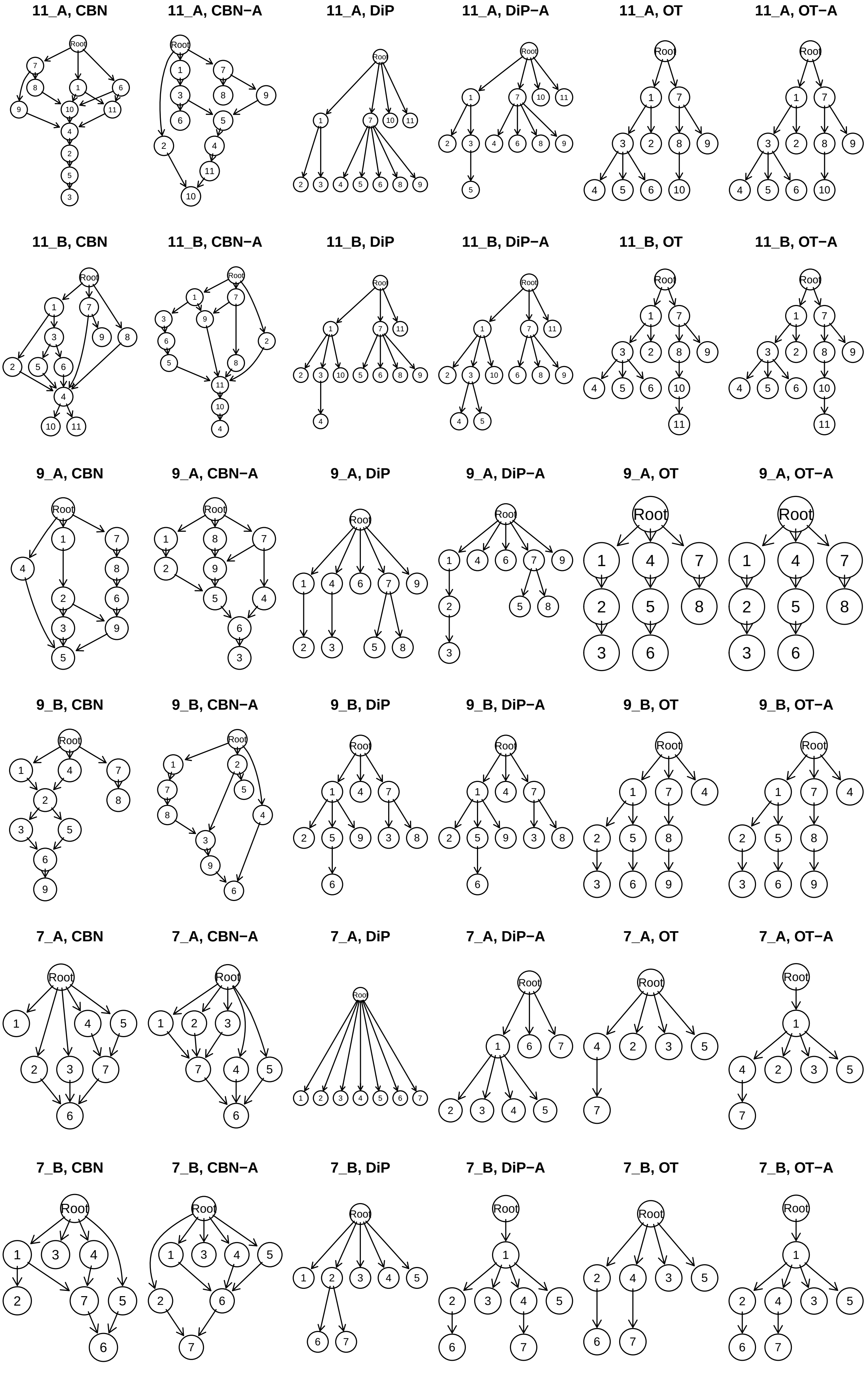

Filtering = NP. Model = exp

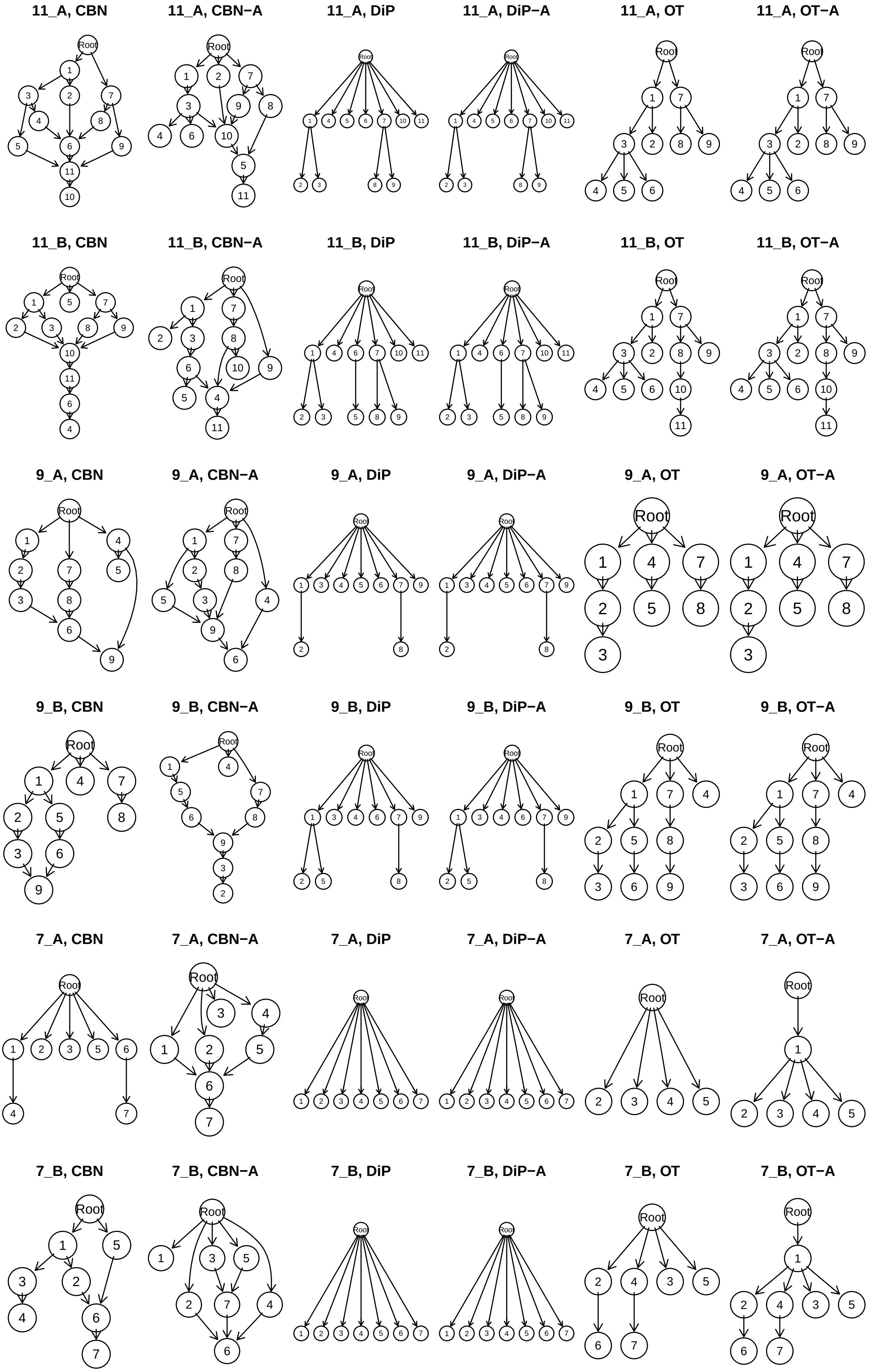

Filtering = NP. Model = McF_4

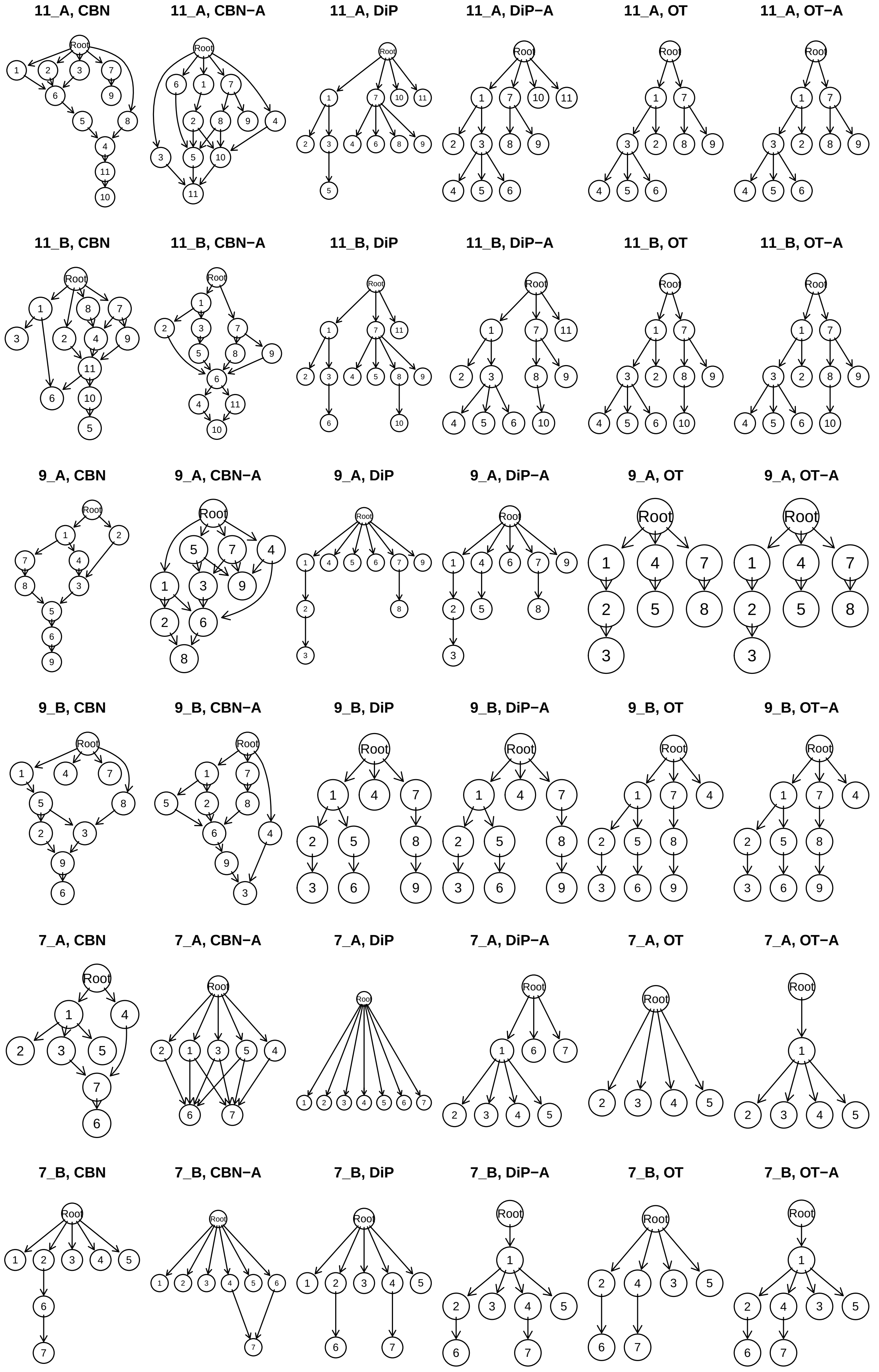

Filtering = NP. Model = McF_6

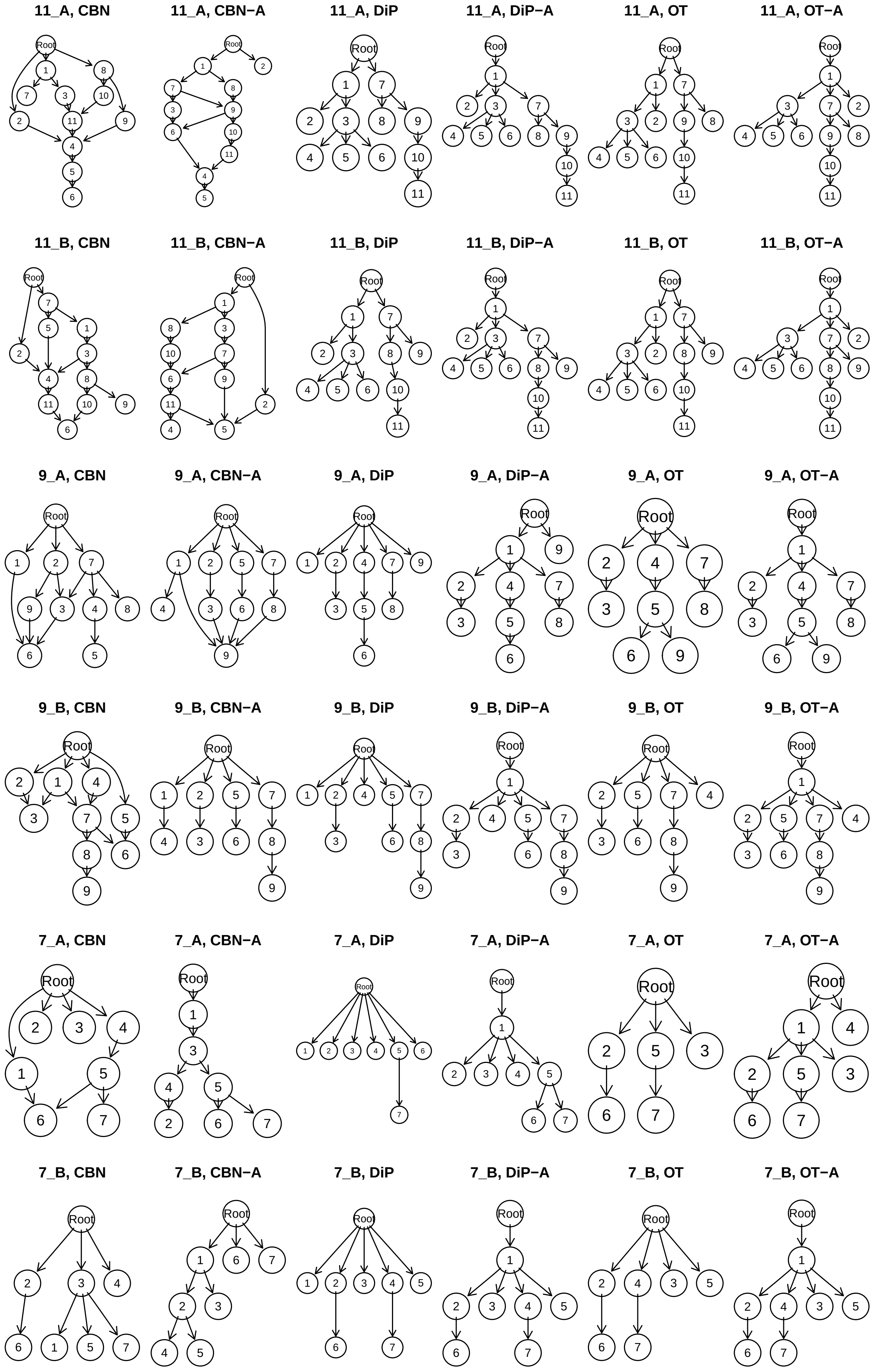

3 Drivers Unknown: Filtering = S1

Filtering = S1. Model = Bozic

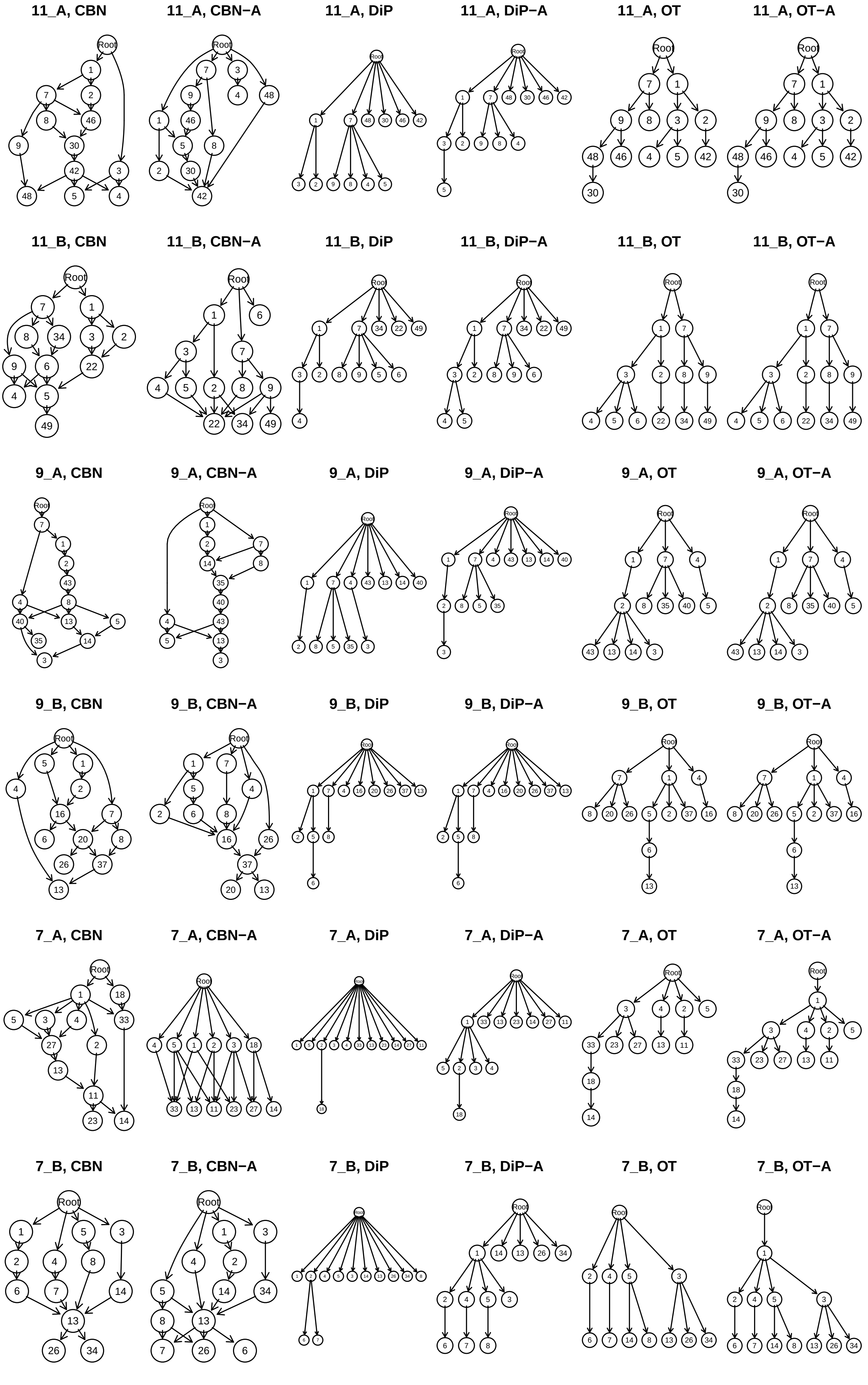

Filtering = S1. Model = exp

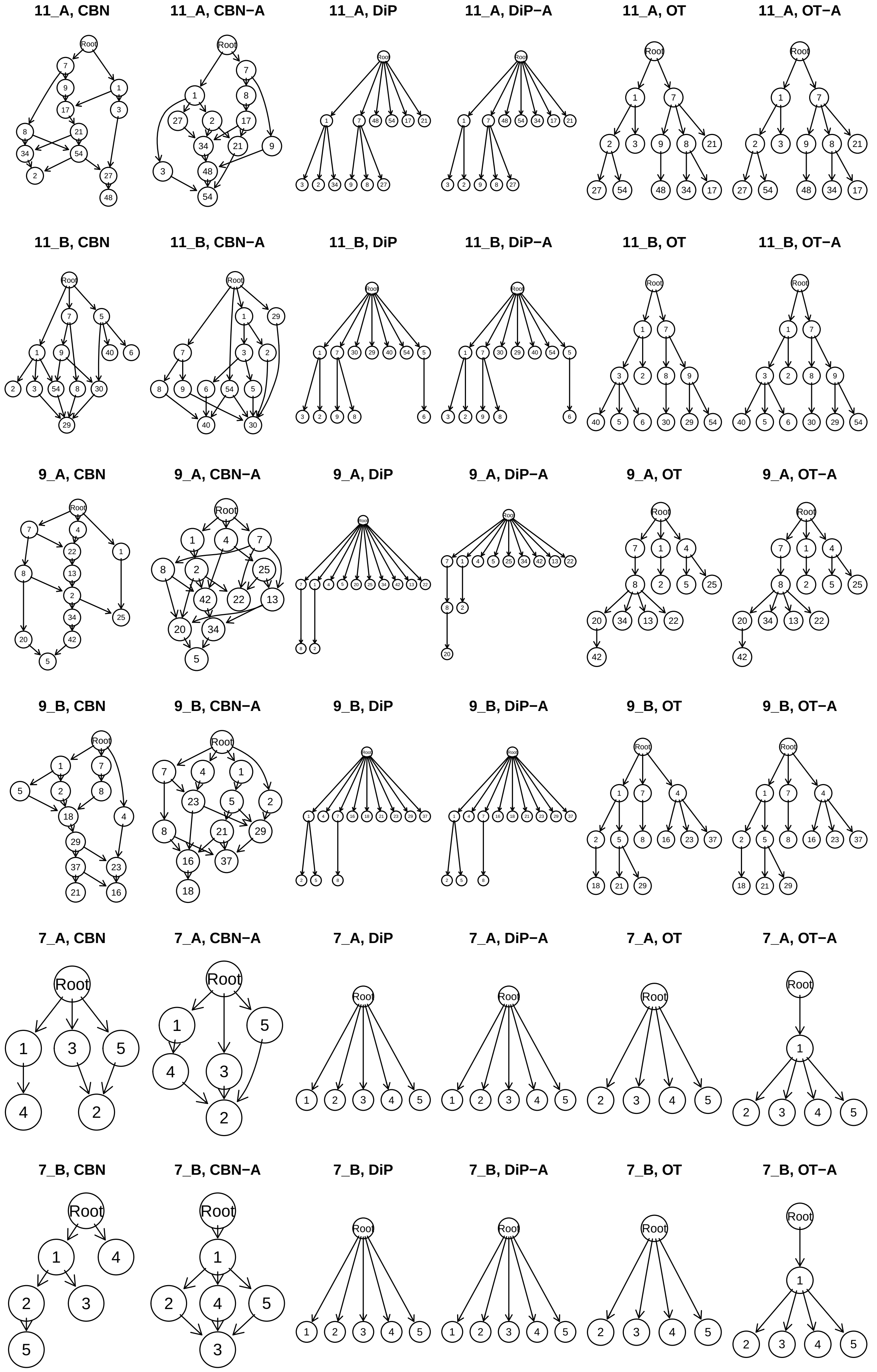

Filtering = S1. Model = McF_4

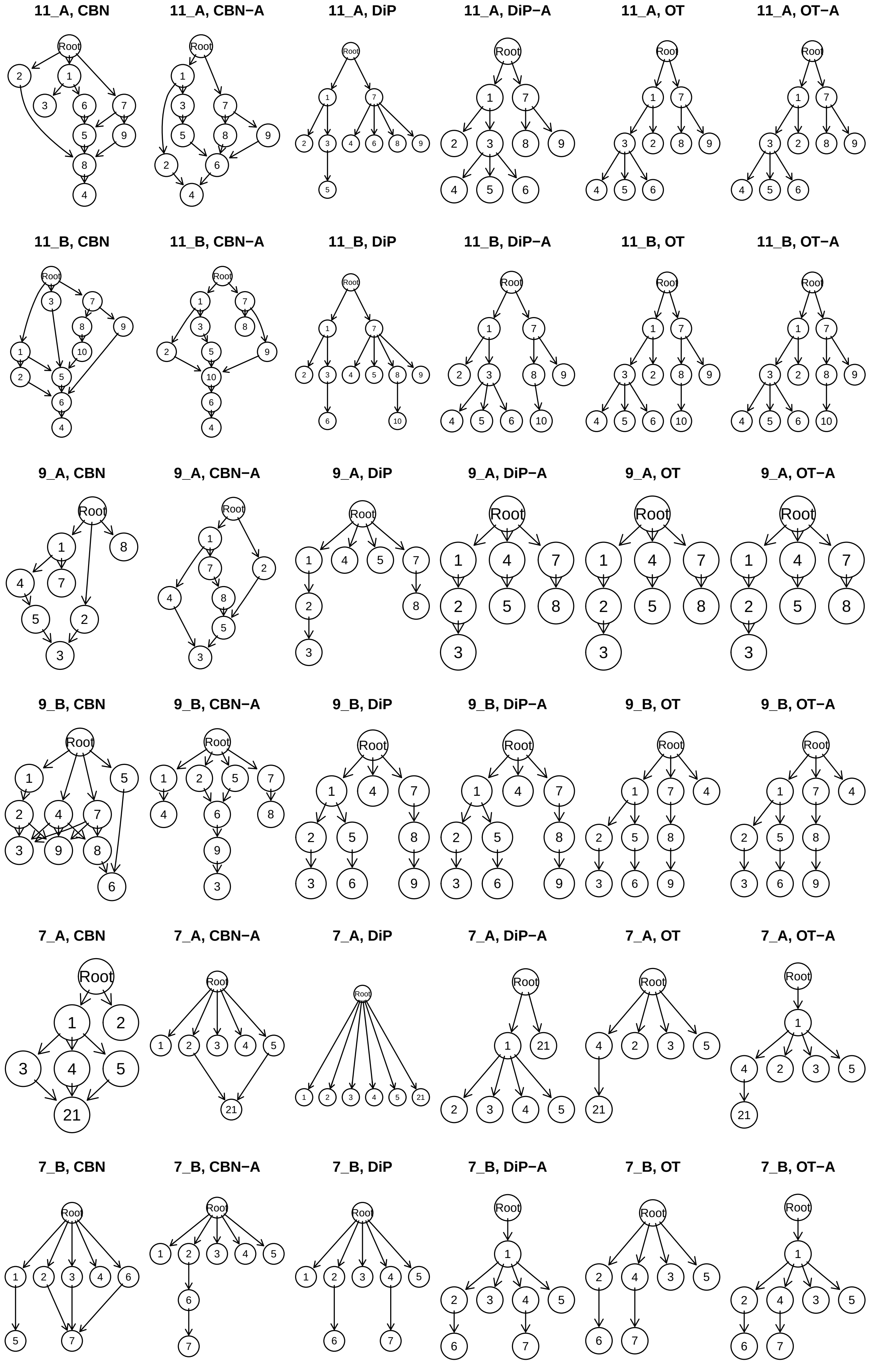

Filtering = S1. Model = McF_6

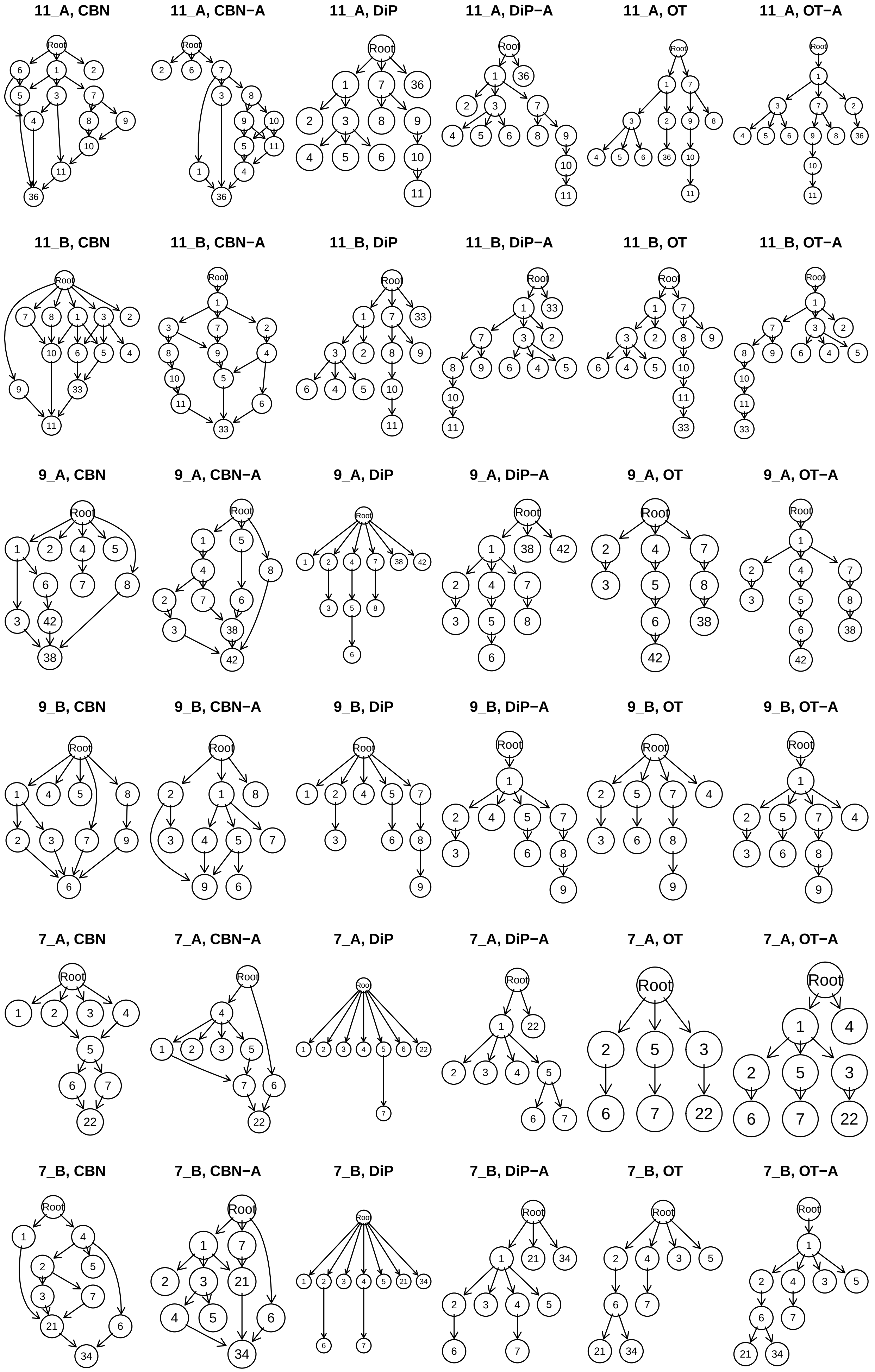

4 Drivers Unknown: Filtering = S5

Filtering = S5. Model = Bozic

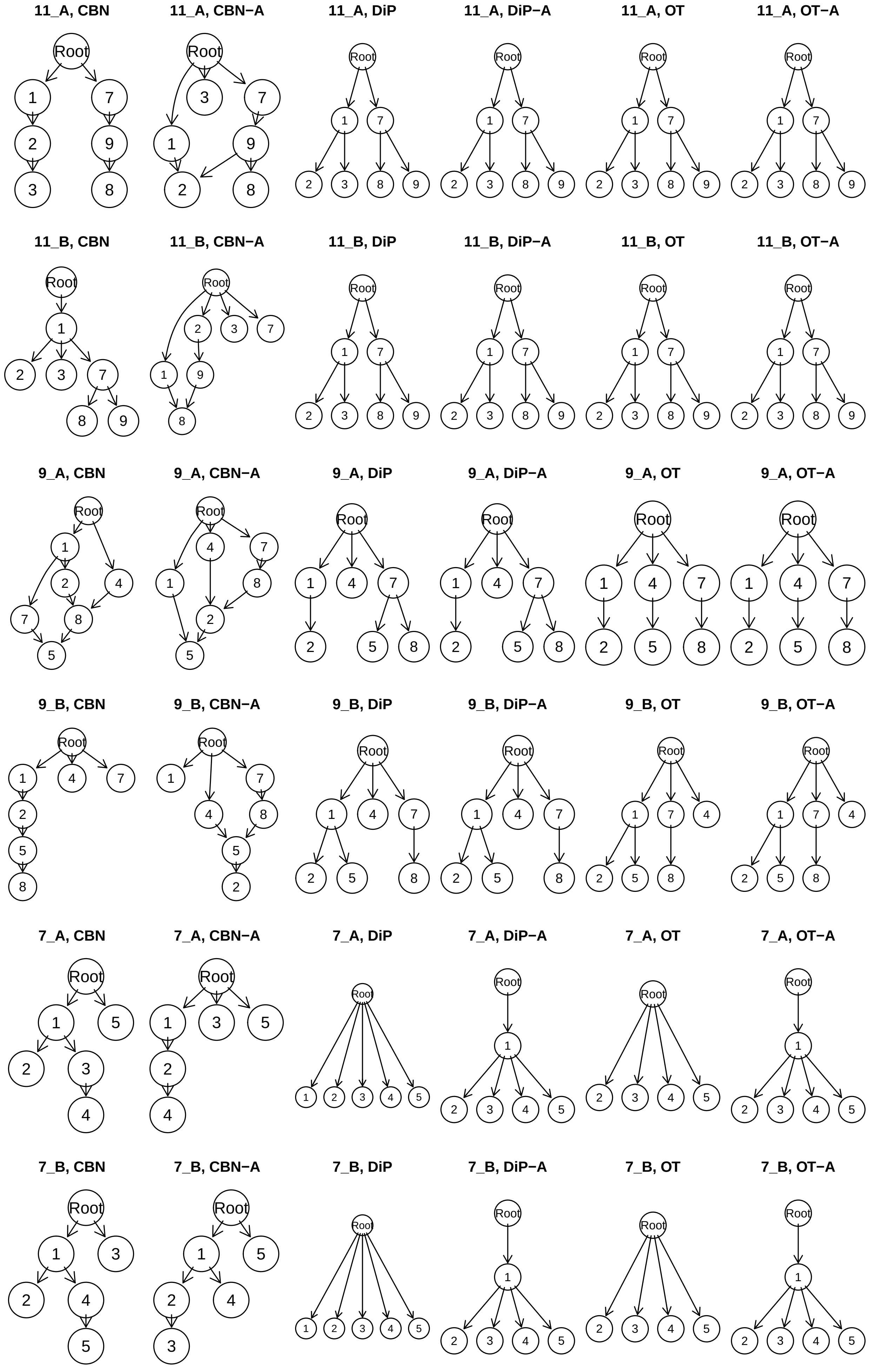

Filtering = S5. Model = exp

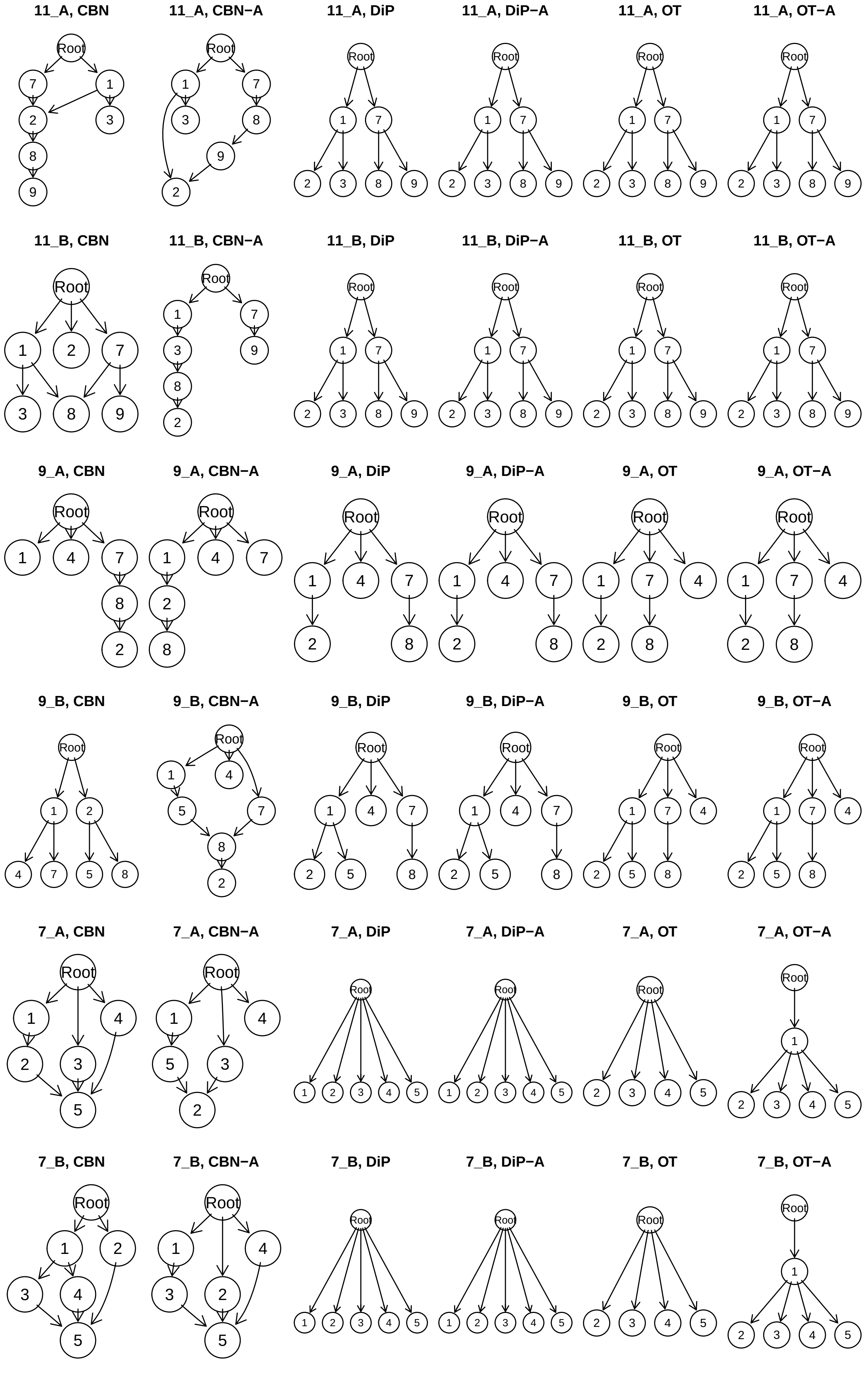

Filtering = S5. Model = McF_4

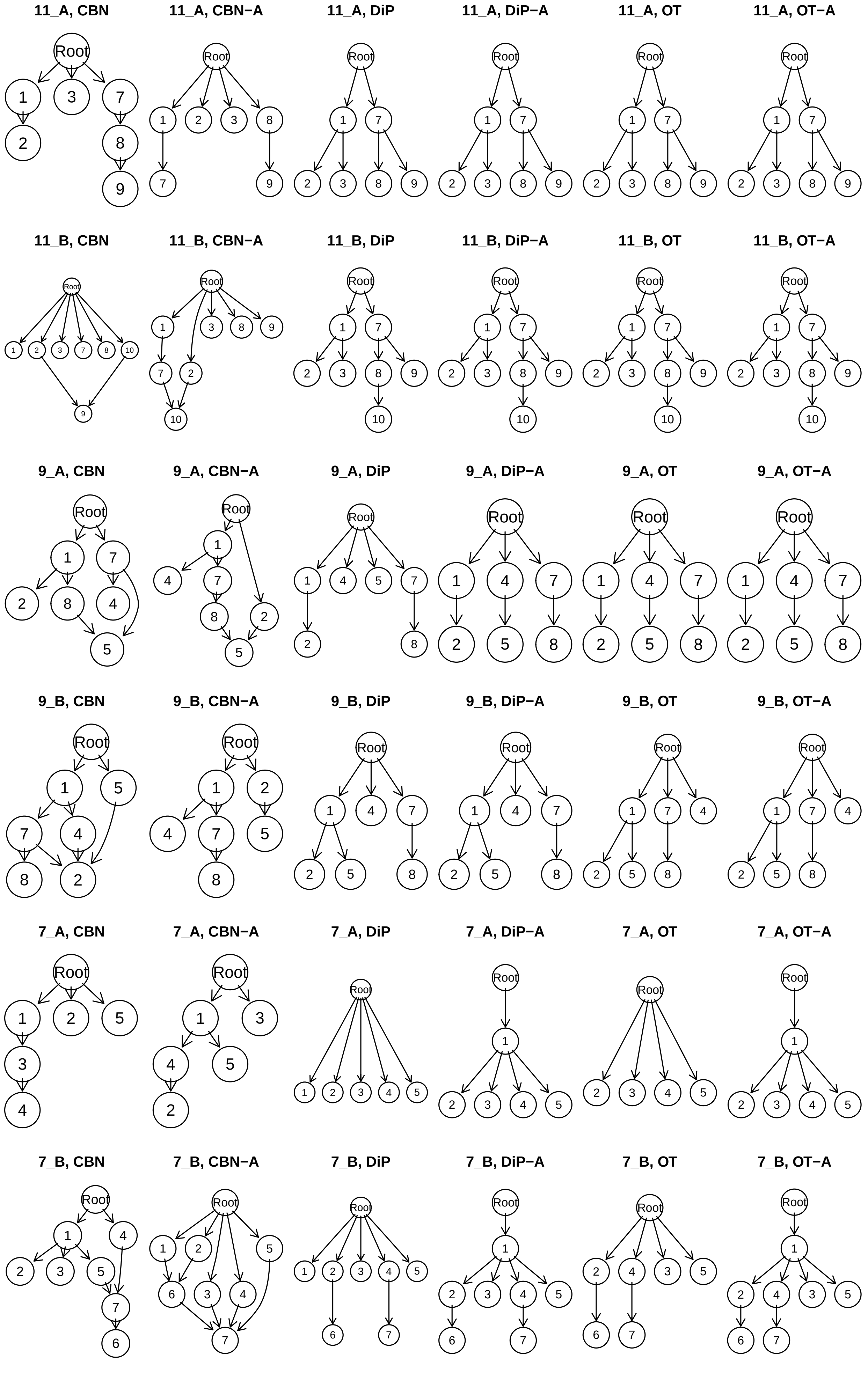

Filtering = S5. Model = McF_6

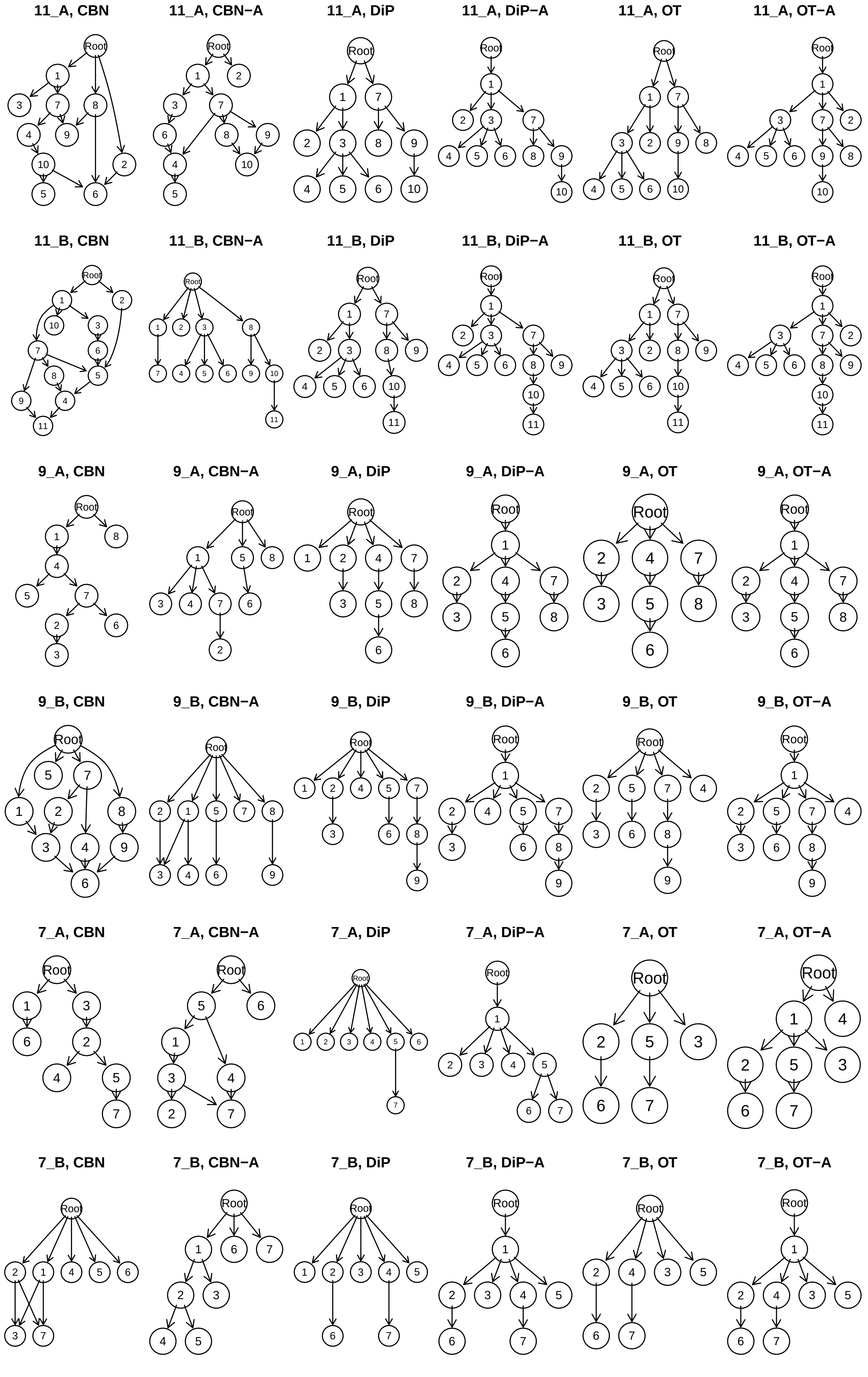

5 Drivers Unknown: Filtering = J1

Filtering = J1. Model = Bozic

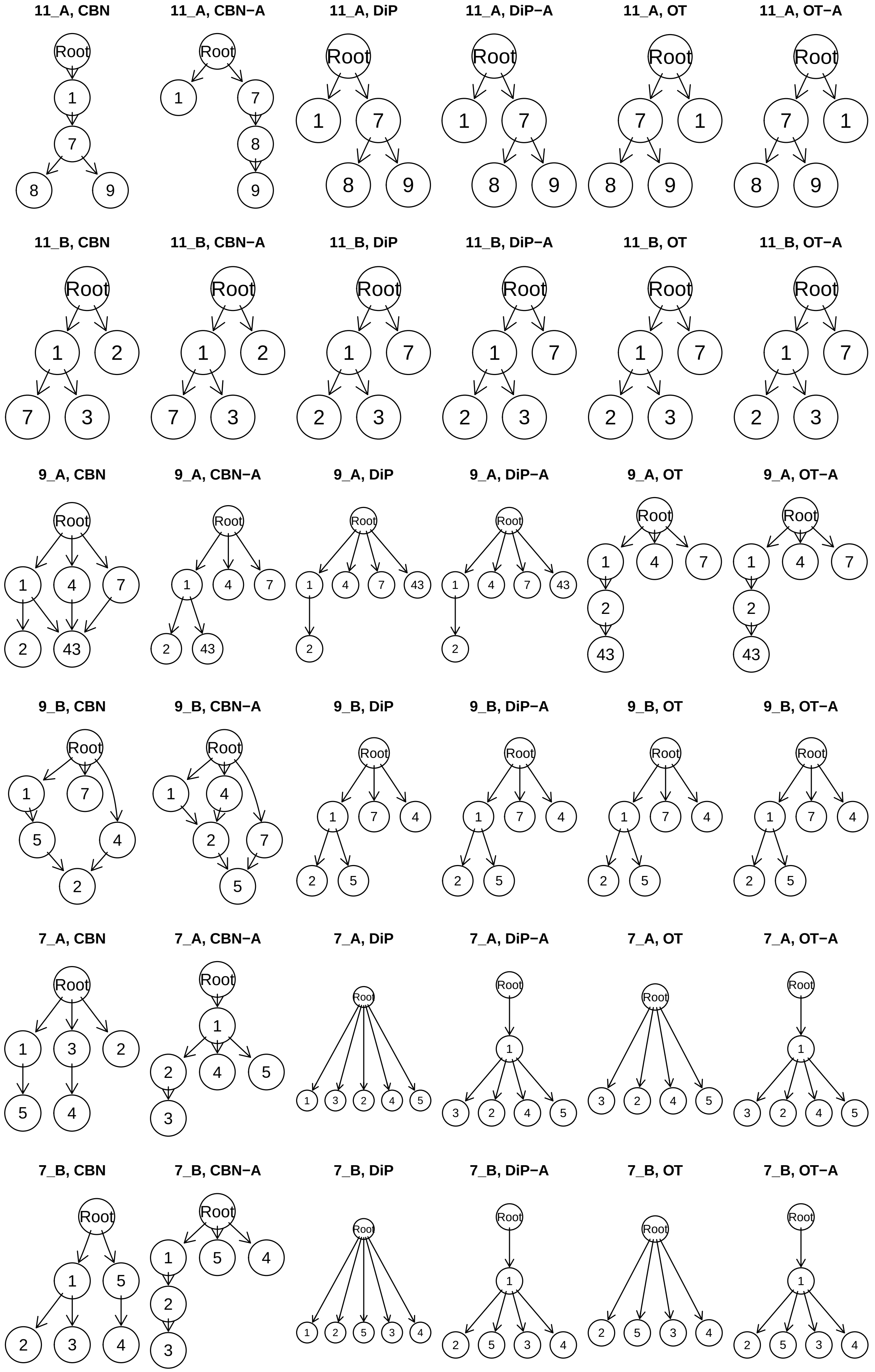

Filtering = J1. Model = exp

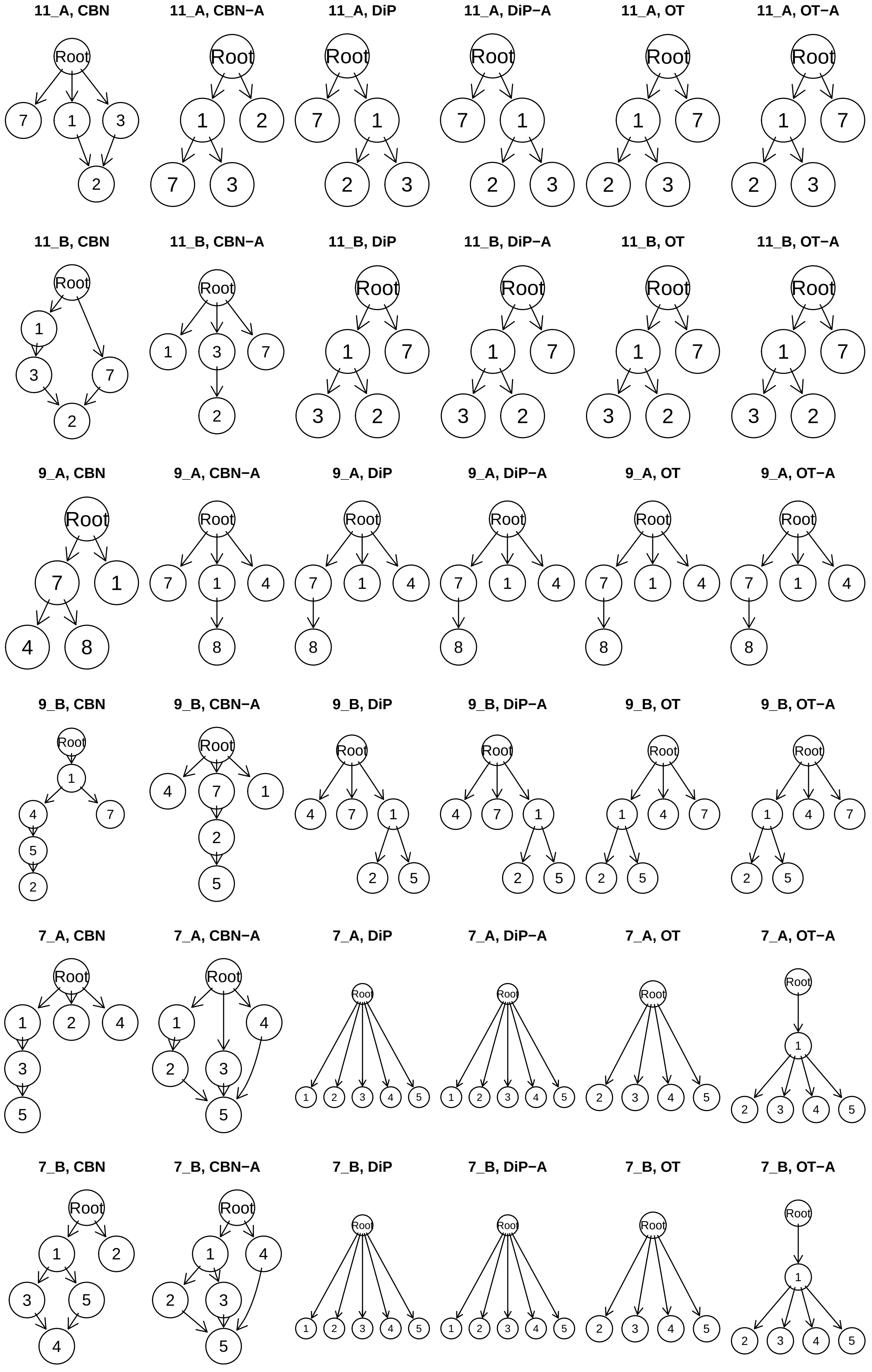

Filtering = J1. Model = McF_4

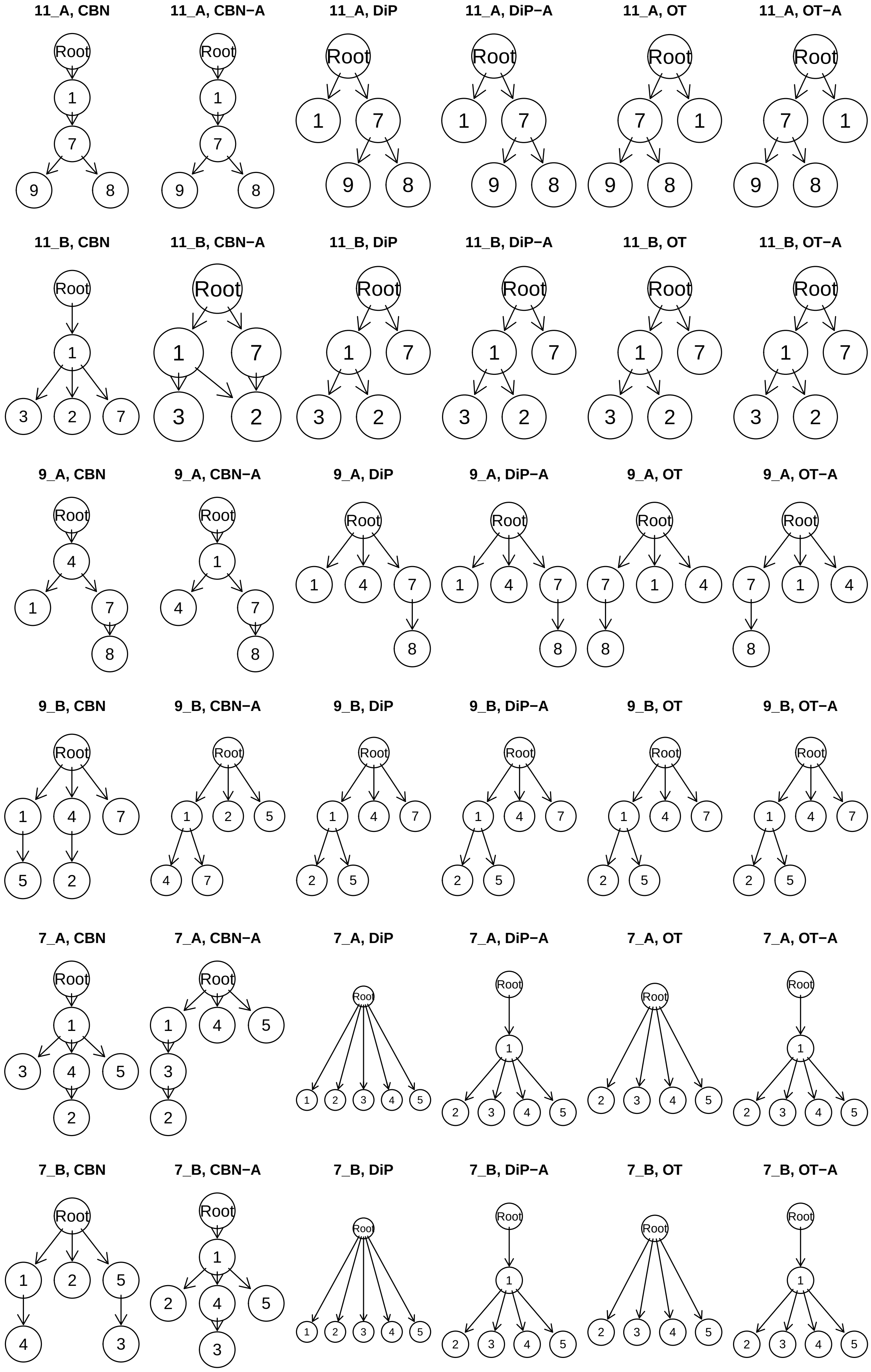

Filtering = J1. Model = McF_6

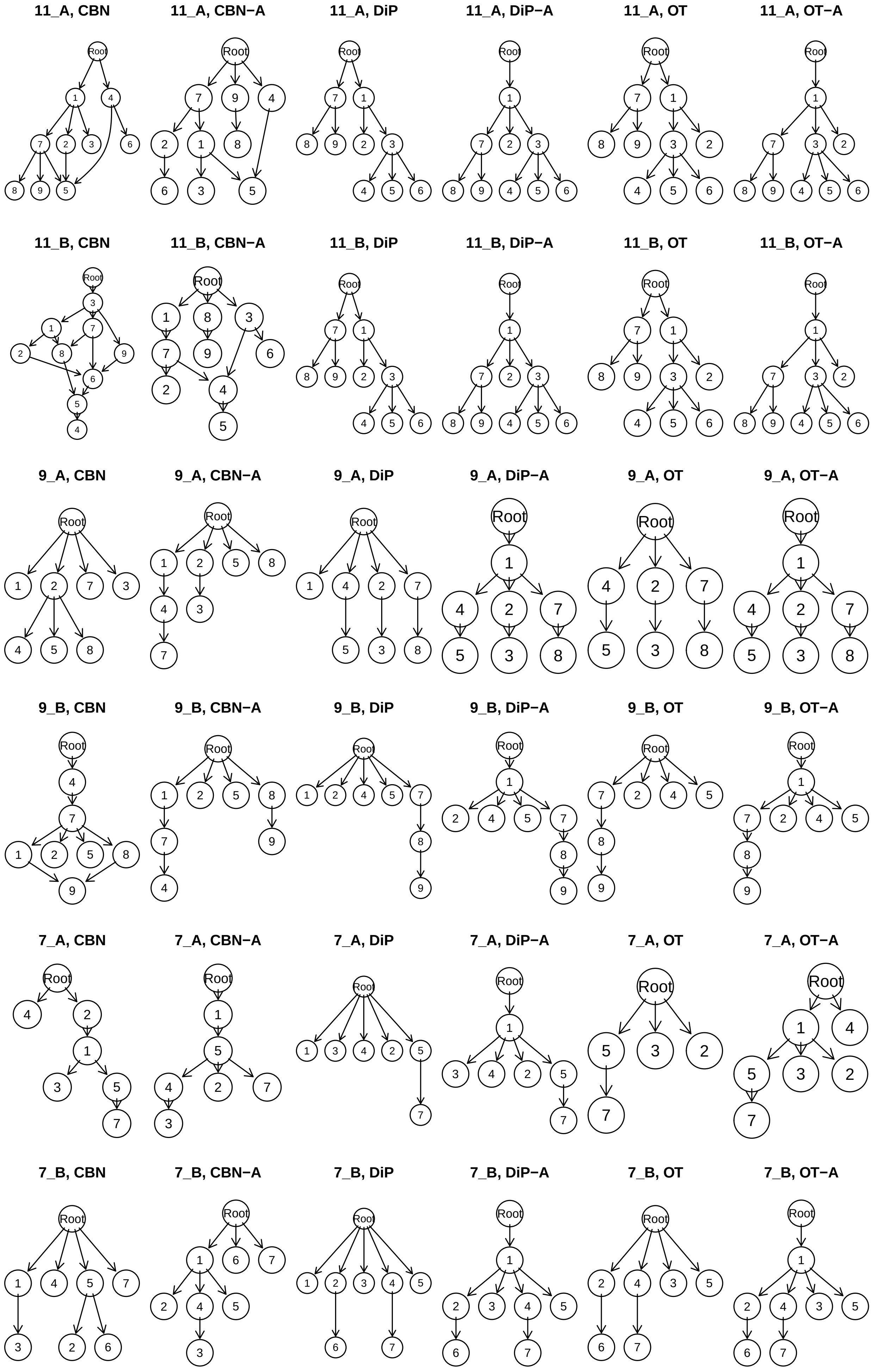

6 Drivers Unknown: Filtering = J5

Filtering = J5. Model = Bozic

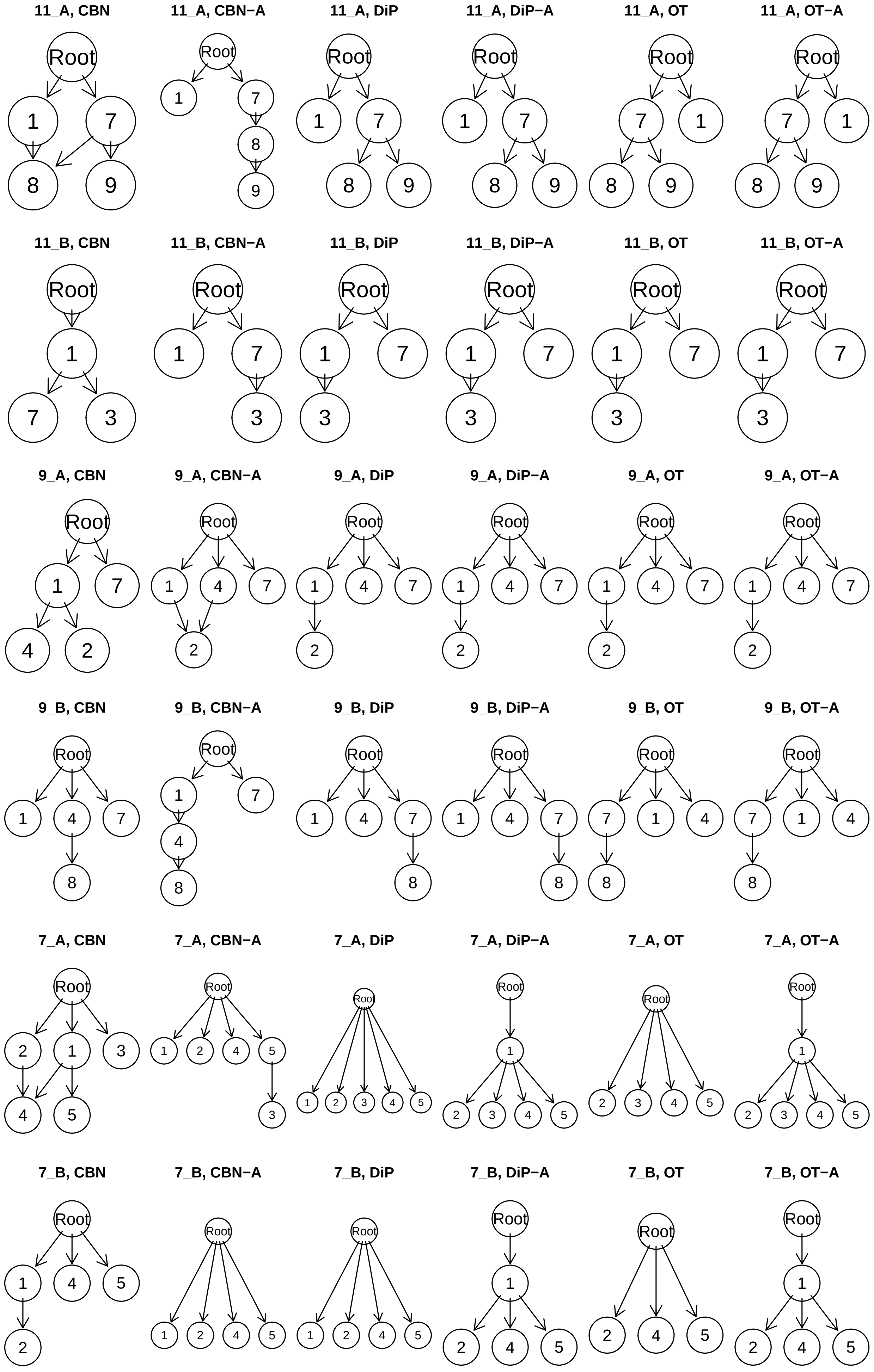

Filtering = J5. Model = exp

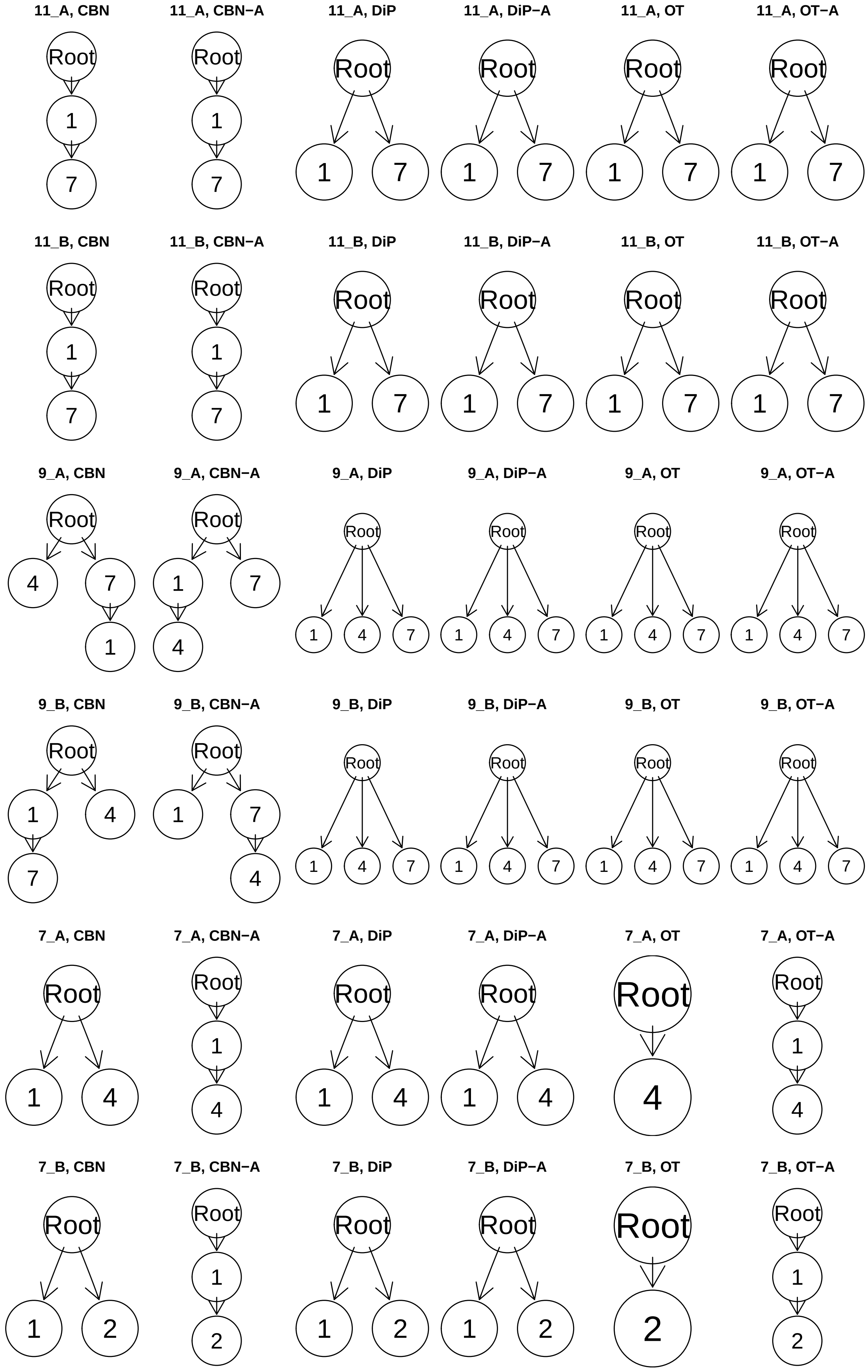

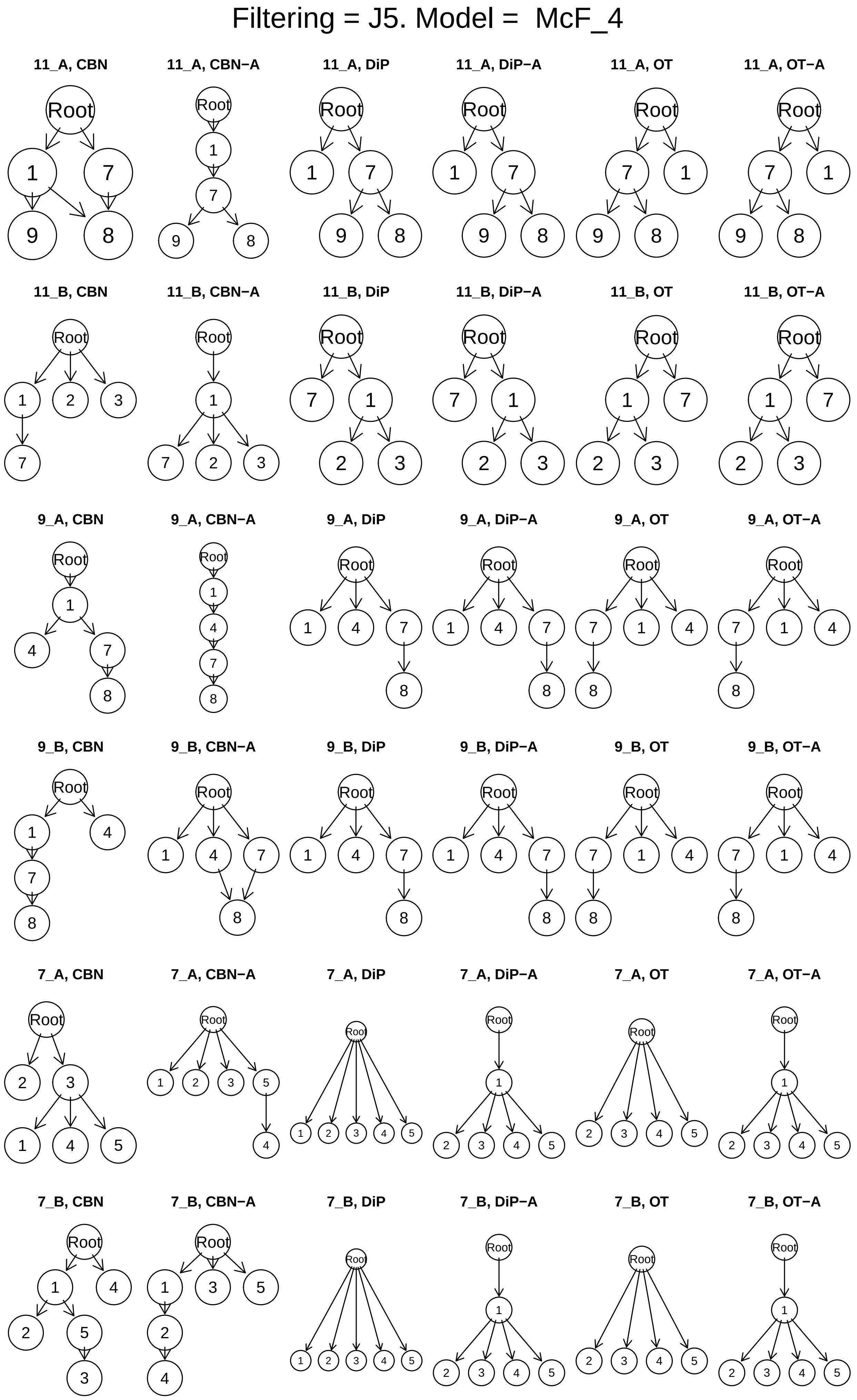
Filtering = J5. Model = McF_4
11_A, CBN
11_A, CBN−A
11_A, DiP
11_A, DiP−A
11_A, OT
11_A, OT−A
11_B, CBN
11_B, CBN−A
11_B, DiP
11_B, DiP−A
11_B, OT
11_B, OT−A
9_A, CBN
9_A, CBN−A
9_A, DiP
9_A, DiP−A
9_A, OT
9_A, OT−A
9_B, CBN
9_B, CBN−A
9_B, DiP
9_B, DiP−A
9_B, OT
9_B, OT−A
7_A, CBN
7_A, CBN−A
7_A, DiP
7_A, DiP−A
7_A, OT
7_A, OT−A
7_B, CBN
7_B, CBN−A
7_B, DiP
7_B, DiP−A
7_B, OT
7_B, OT−A

Filtering = J5. Model = McF_6

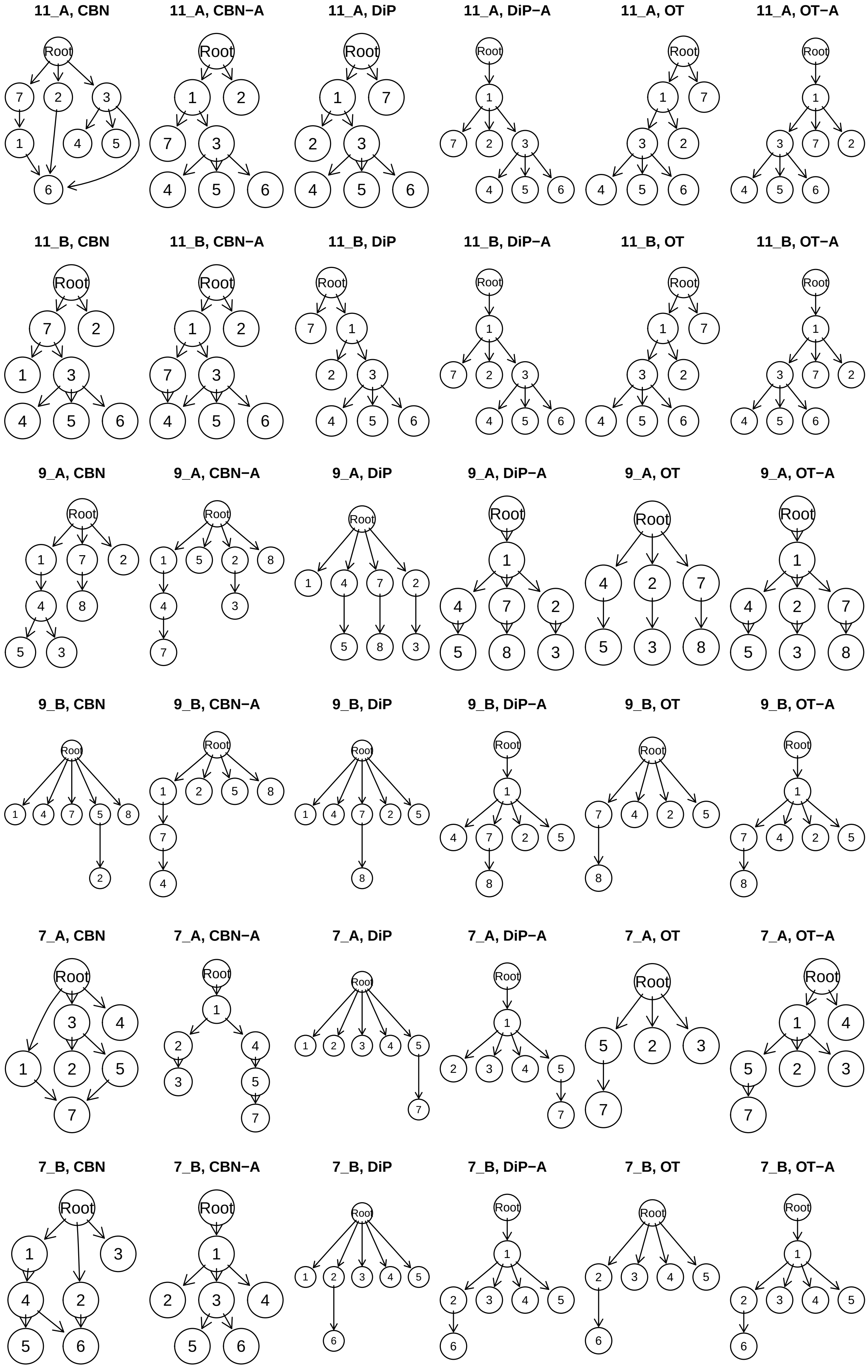

Supplement: Additional file 5 — Examples of inferred graphs. Examples of inferred graphs from each of the six methods for two of the scenarios under each Model and True Graph. [file 12859_2015_466_MOESM5_ESM.pdf]
