# Supplementary material for: Identifying restrictions in the order of accumulation of mutations during tumor progression: effects of passengers, evolutionary models, and sampling
Source: BMC Bioinformatics. 2015 Feb 12;16:41. doi: 10.1186/s12859-015-0466-7 (PMC4339747; doi:10.1186/s12859-015-0466-7)
Supplement: Additional file 6 — Colored tables of median performance for all combinations of factors. Tables that show, for all performance measures, as well as for the number of edges in the transitive closure of the relations, the median values for all combinations of factors. The tables use colors (like a heatmap) to ease locating the best and worst median values. [file 12859_2015_466_MOESM6_ESM.pdf]

Additional file 6. Colored tables of median performance for all combinations of factors.

Supplementary material for "Identifying Restrictions in the Order of Accumulation of Mutations during Tumor Progression: Effects of Passengers, Evolutionary Models, and Sampling"

Ramon Diaz-Uriarte
Dept. Biochemistry, Universidad Autónoma de Madrid
Instituto de Investigaciones Biomédicas "Alberto Sols" (UAM-CSIC)
Madrid, Spain

ramon.diaz@iib.uam.es

rdiaz02@gmail.com

http://ligarto.org/rdiaz

Contents

1 Introduction **3**

2 Drivers Known **4**
2.1 Inferred edges, Drivers Known 4
2.2 Diff, Drivers Known 8
2.3 PFD, Drivers Known 12
2.4 PND, Drivers Known 16
2.5 FPF, Drivers Known 20

3 Drivers Unknown **24**
3.1 Inferred edges, Drivers Unknown 24
3.2 Diff, Drivers Unknown 31
3.3 PFD, Drivers Unknown 38
3.4 PND, Drivers Unknown 45
3.5 FPF, Drivers Unknown 52

List of Figures

1 Drivers Known, number of inferred edges (not counting those from Root), N = 1000 . . . 5
2 Drivers Known, number of inferred edges (not counting those from Root), N = 200 . . . 6
3 Drivers Known, number of inferred edges (not counting those from Root), N = 100 . . . 7
4 Drivers Known, Diff, N = 1000 . . . 9
5 Drivers Known, Diff, N = 200 . . . 10
6 Drivers Known, Diff, N = 100 . . . 11
7 Drivers Known, PFD, N = 1000 . . . 13
8 Drivers Known, PFD, N = 200 . . . 14
9 Drivers Known, PFD, N = 100 . . . 15
10 Drivers Known, PND, N = 1000 . . . 17
11 Drivers Known, PND, N = 200 . . . 18
12 Drivers Known, PND, N = 100 . . . 19
13 Drivers Known, FPF, N = 1000 . . . 21
14 Drivers Known, FPF, N = 200 . . . 22
15 Drivers Known, FPF, N = 100 . . . 23
16 Drivers Unknown, number of inferred edges (not counting those from Root), N = 1000, S.Time = last. . . . 25
17 Drivers Unknown, number of inferred edges (not counting those from Root), N = 1000, S.Time = unif. . . . 26
18 Drivers Unknown, number of inferred edges (not counting those from Root), N = 200, S.Time = last . . . 27
19 Drivers Unknown, number of inferred edges (not counting those from Root), N = 200, S.Time = unif. . . . 28
20 Drivers Unknown, number of inferred edges (not counting those from Root), N = 100, S.Time = last . . . 29
21 Drivers Unknown, number of inferred edges (not counting those from Root), N = 100, S.Time = unif. . . . 30
22 Drivers Unknown, Diff, N = 1000, S.Time = last . . . 32
23 Drivers Unknown, Diff, N = 1000, S.Time = unif. . . . 33
24 Drivers Unknown, Diff, N = 200, S.Time = last . . . 34
25 Drivers Unknown, Diff, N = 200, S.Time = unif. . . . 35
26 Drivers Unknown, Diff, N = 100, S.Time = last . . . 36
27 Drivers Unknown, Diff, N = 100, S.Time = unif. . . . 37
28 Drivers Unknown, PFD, N = 1000, S.Time = last . . . 39
29 Drivers Unknown, PFD, N = 1000, S.Time = unif. . . . 40
30 Drivers Unknown, PFD, N = 200, S.Time = last . . . 41
31 Drivers Unknown, PFD, N = 200, S.Time = unif. . . . 42
32 Drivers Unknown, PFD, N = 100, S.Time = last . . . 43
33 Drivers Unknown, PFD, N = 100, S.Time = unif. . . . 44
34 Drivers Unknown, PND, N = 1000, S.Time = last . . . 46
35 Drivers Unknown, PND, N = 1000, S.Time = unif. . . . 47
36 Drivers Unknown, PND, N = 200, S.Time = last . . . 48
37 Drivers Unknown, PND, N = 200, S.Time = unif. . . . 49
38 Drivers Unknown, PND, N = 100, S.Time = last . . . 50
39 Drivers Unknown, PND, N = 100, S.Time = unif. . . . 51
40 Drivers Unknown, FPF, N = 1000, S.Time = last . . . 53
41 Drivers Unknown, FPF, N = 1000, S.Time = unif. . . . 54
42 Drivers Unknown, FPF, N = 200, S.Time = last . . . 55
43 Drivers Unknown, FPF, N = 200, S.Time = unif. . . . 56
44 Drivers Unknown, FPF, N = 100, S.Time = last . . . 57
45 Drivers Unknown, FPF, N = 100, S.Time = unif. . . . 58

1 Introduction

The following tables (figures, actually) show, for all performance measure., as well as for the number of edges in the transitive closure of the relations (i.e., the # of relations in F for performance measure PFD), the median values for all combinations of factors. Each cell is the median of 20 replicates.

For all figures, lighter colors denote smaller values, and color intensity is normalized by row (i.e., there will be at least one cell that is white and one cell that is dark red in each row). Grey cells denote missing values: i.e., all 20 values for that cell were missing. Missing values can only occur in PFD (since, even if there are no fits, the rest of the performance measures are defined).

For performance measures (Diff, PFD, PND, FPF), therefore, lighter colors denote better performance. For "Inferred edges", lighter colors highlight those cases with few discoveries.

The figures for the Drivers Unknown had to be split into two, for readability: the first half is with S.Time = last and the second with S.Time = unif (this is shown in the figure). Please note that the color intensity is normalized by row **over the two figures**.

2 Drivers Known

2.1 Inferred edges, Drivers Known

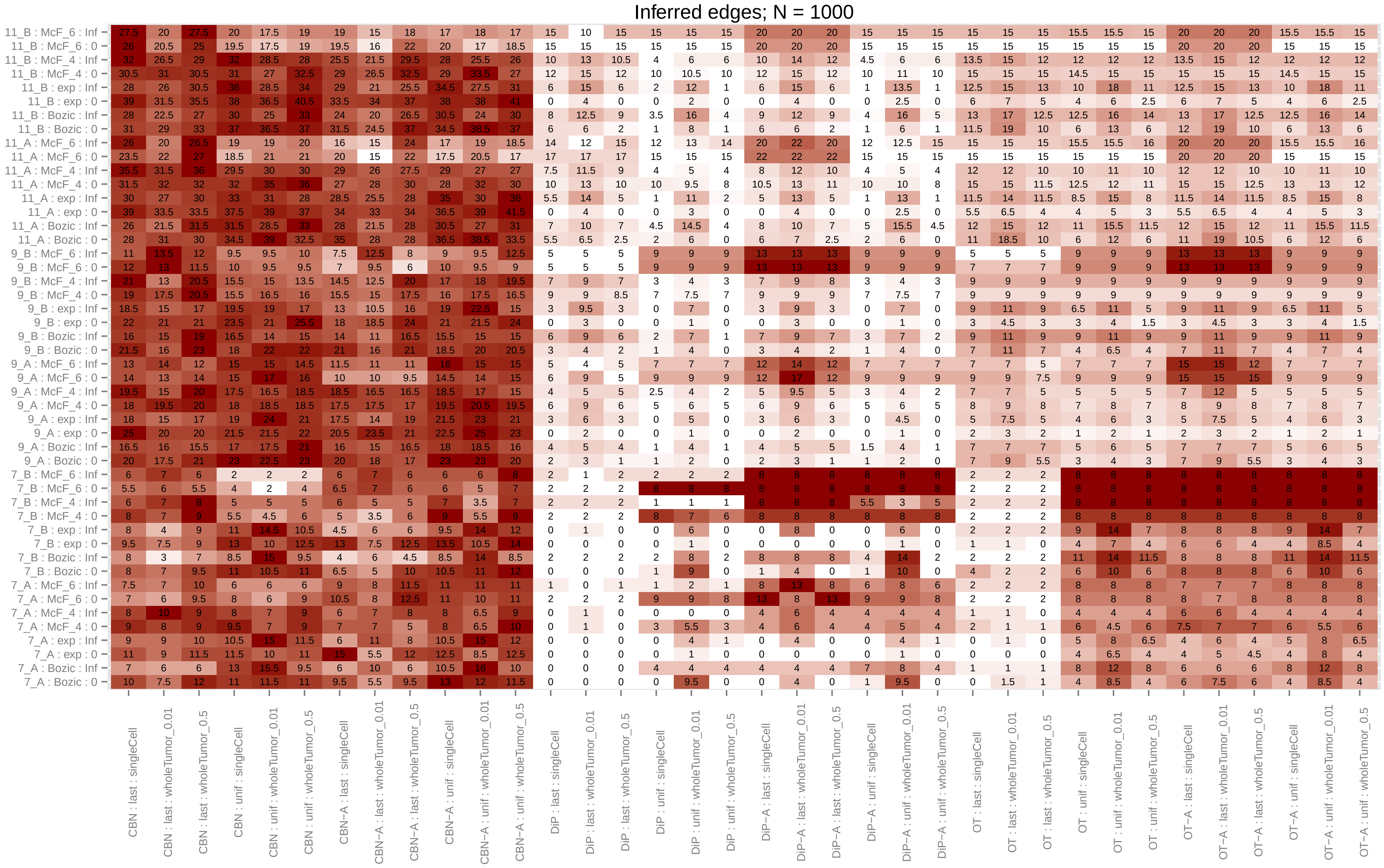

Figure 1: Drivers Known, number of inferred edges (not counting those from Root), N = 1000

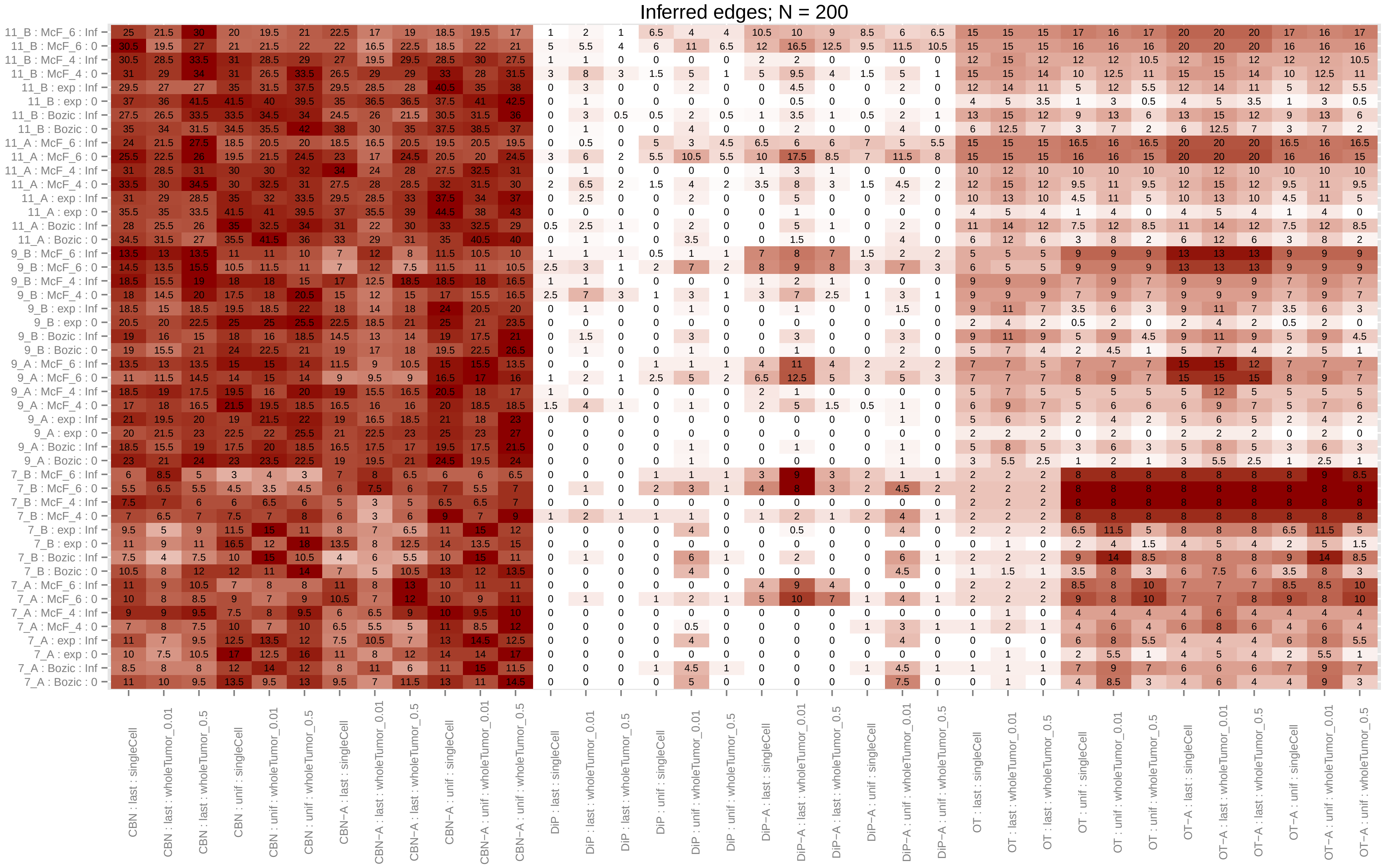

Figure 2: Drivers Known, number of inferred edges (not counting those from Root), N = 200

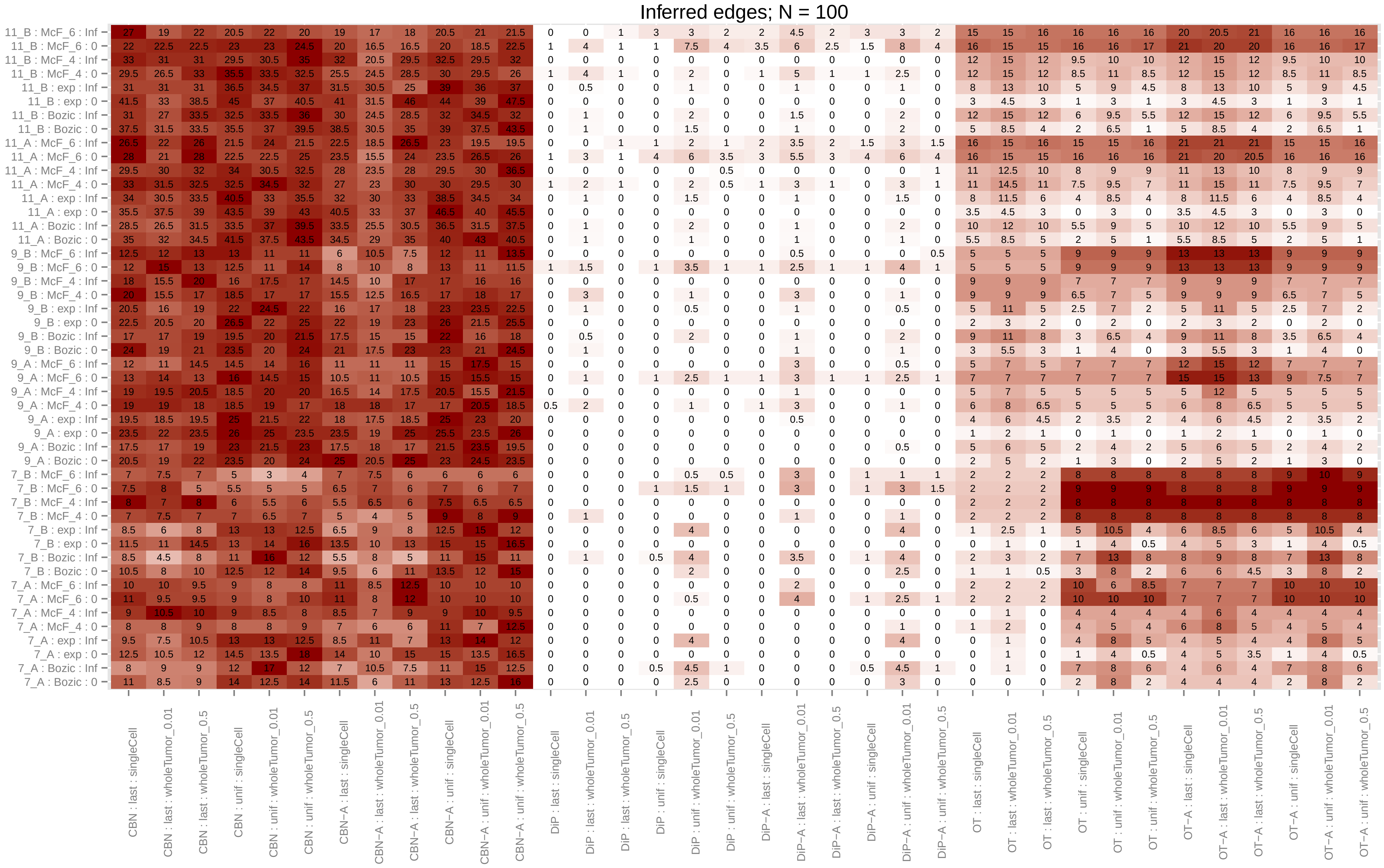

Figure 3: Drivers Known, number of inferred edges (not counting those from Root), N = 100

2.2 Diff, Drivers Known

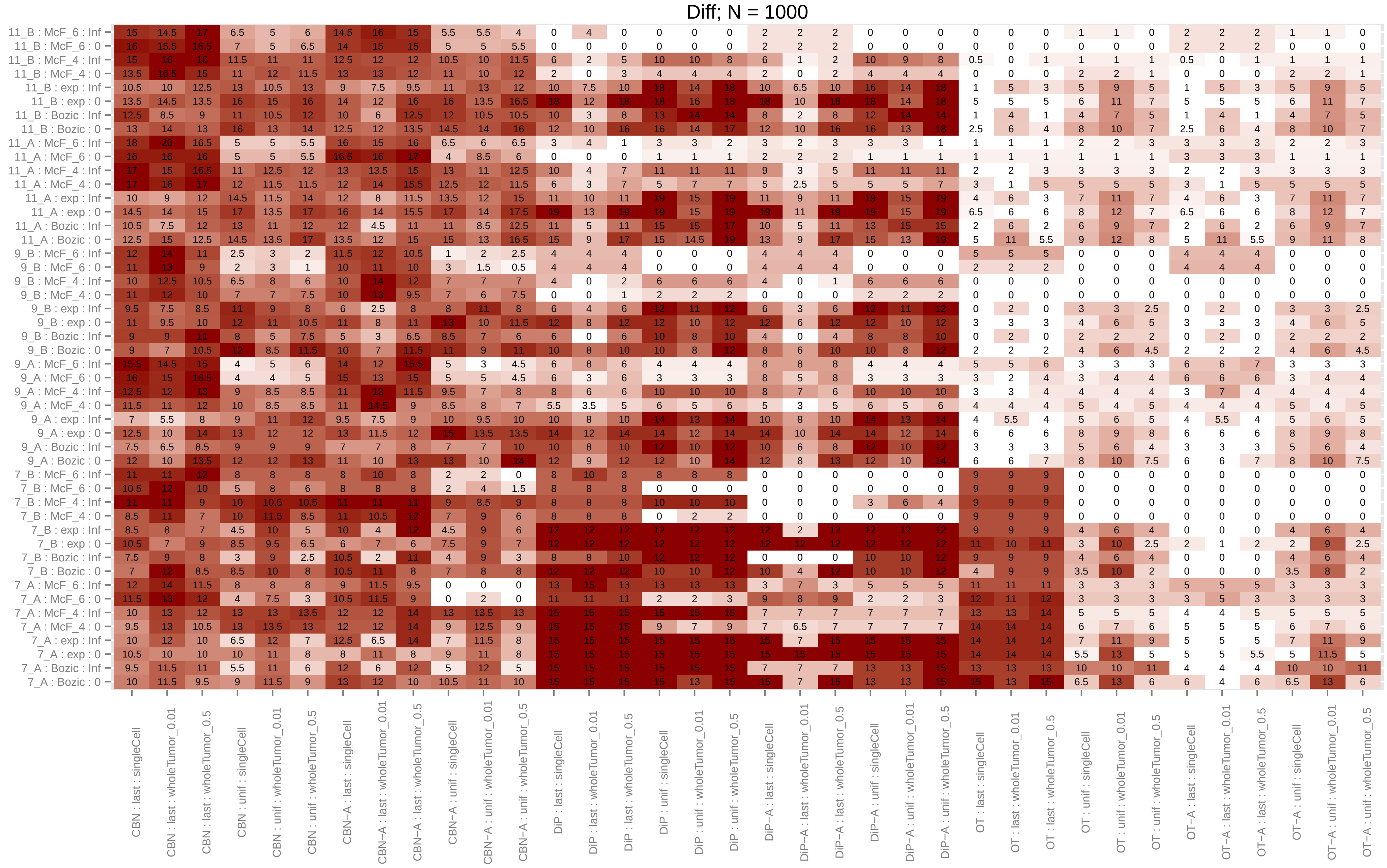

Figure 4: Drivers Known, Diff, N = 1000

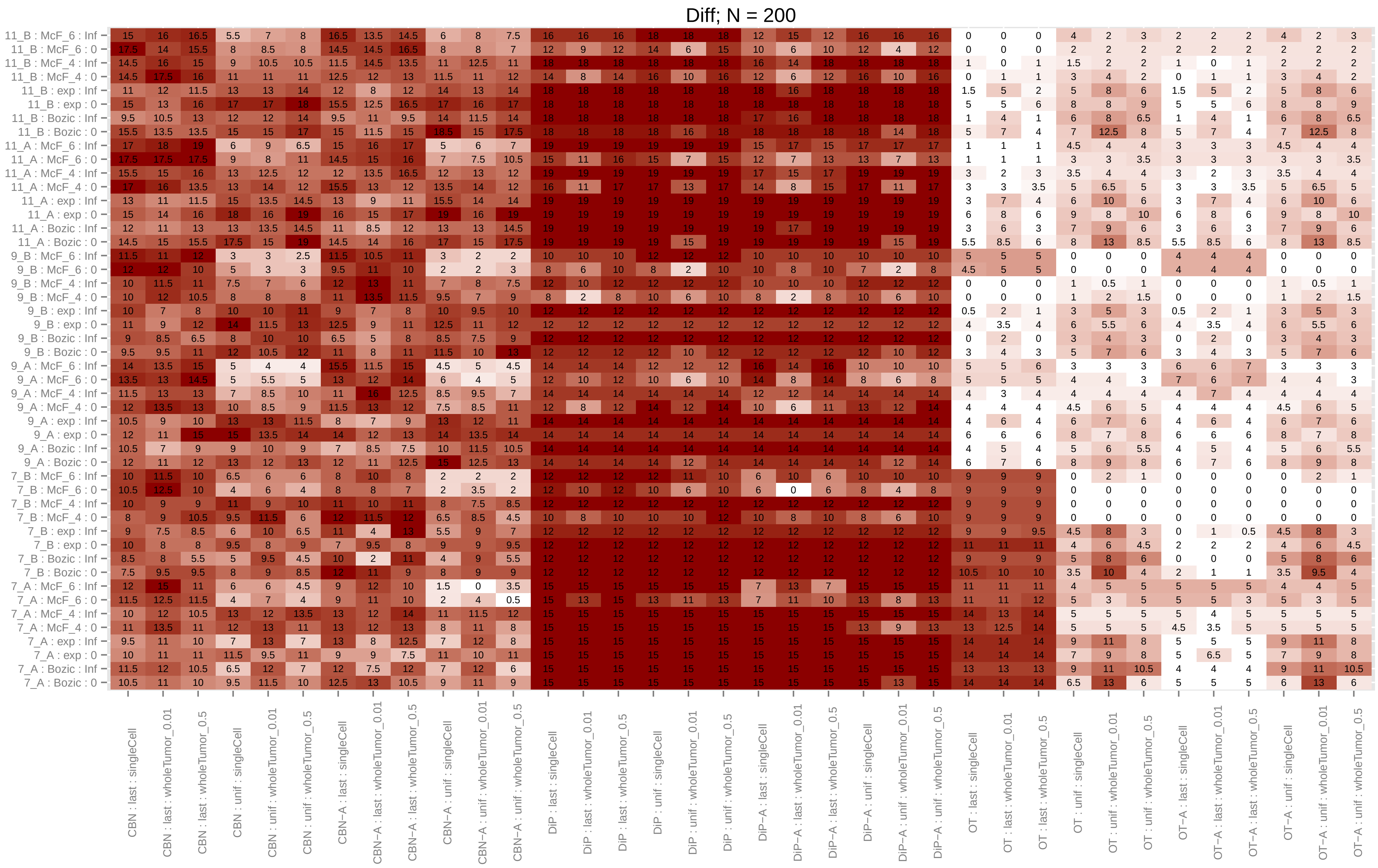

Figure 5: Drivers Known, Diff, N = 200

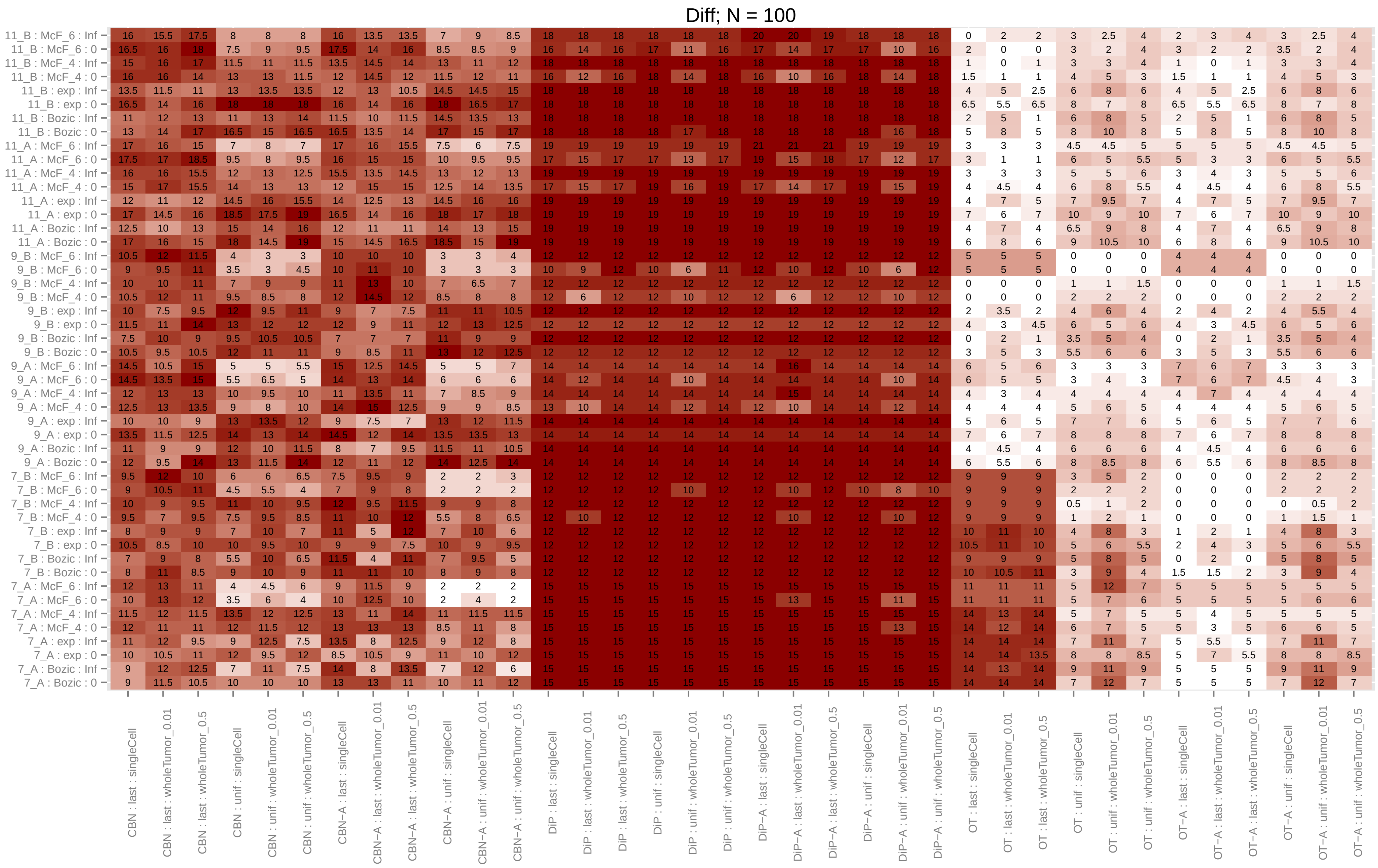

Figure 6: Drivers Known, Diff, N = 100

2.3 PFD, Drivers Known

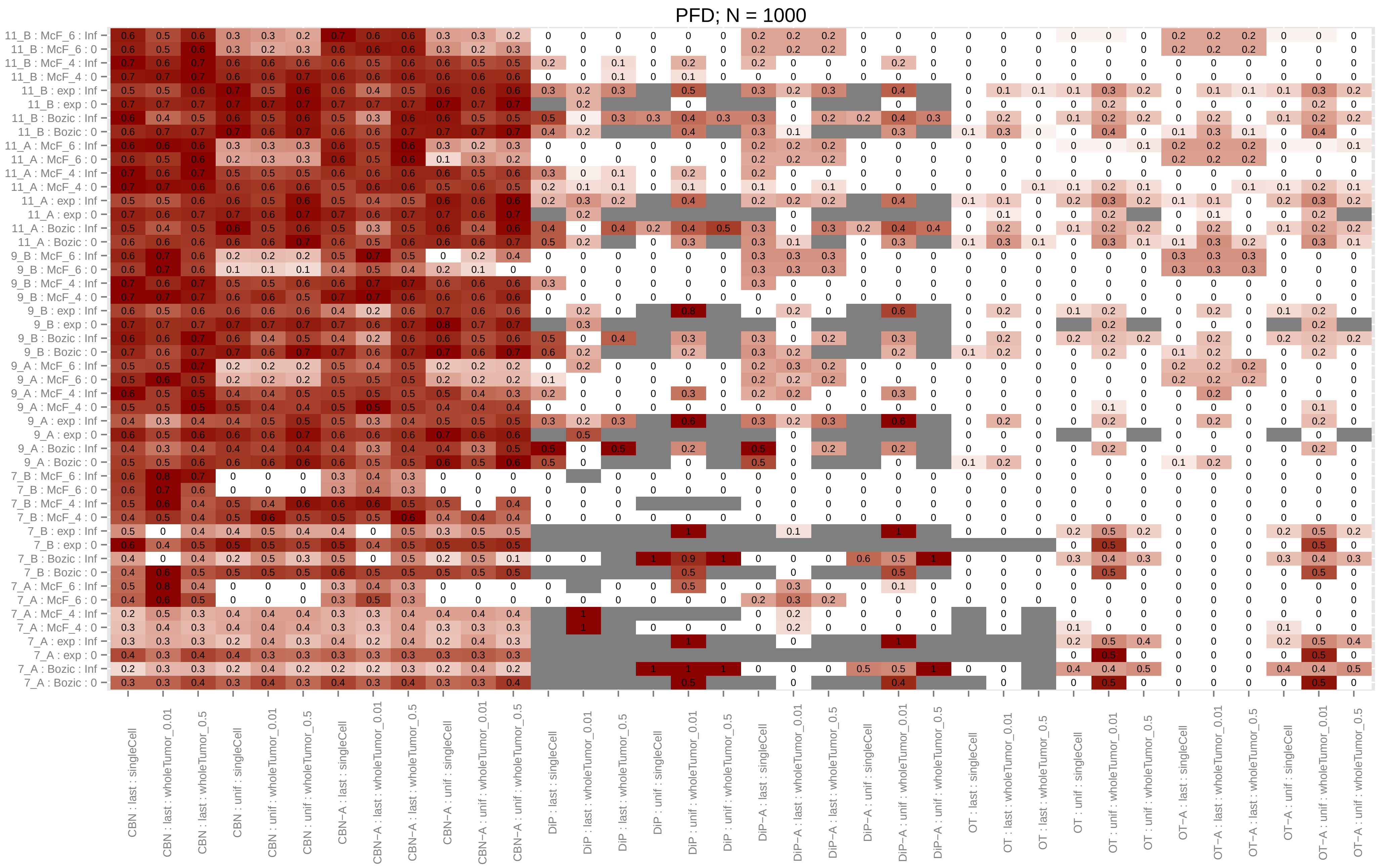

Figure 7: Drivers Known, PFD, N = 1000

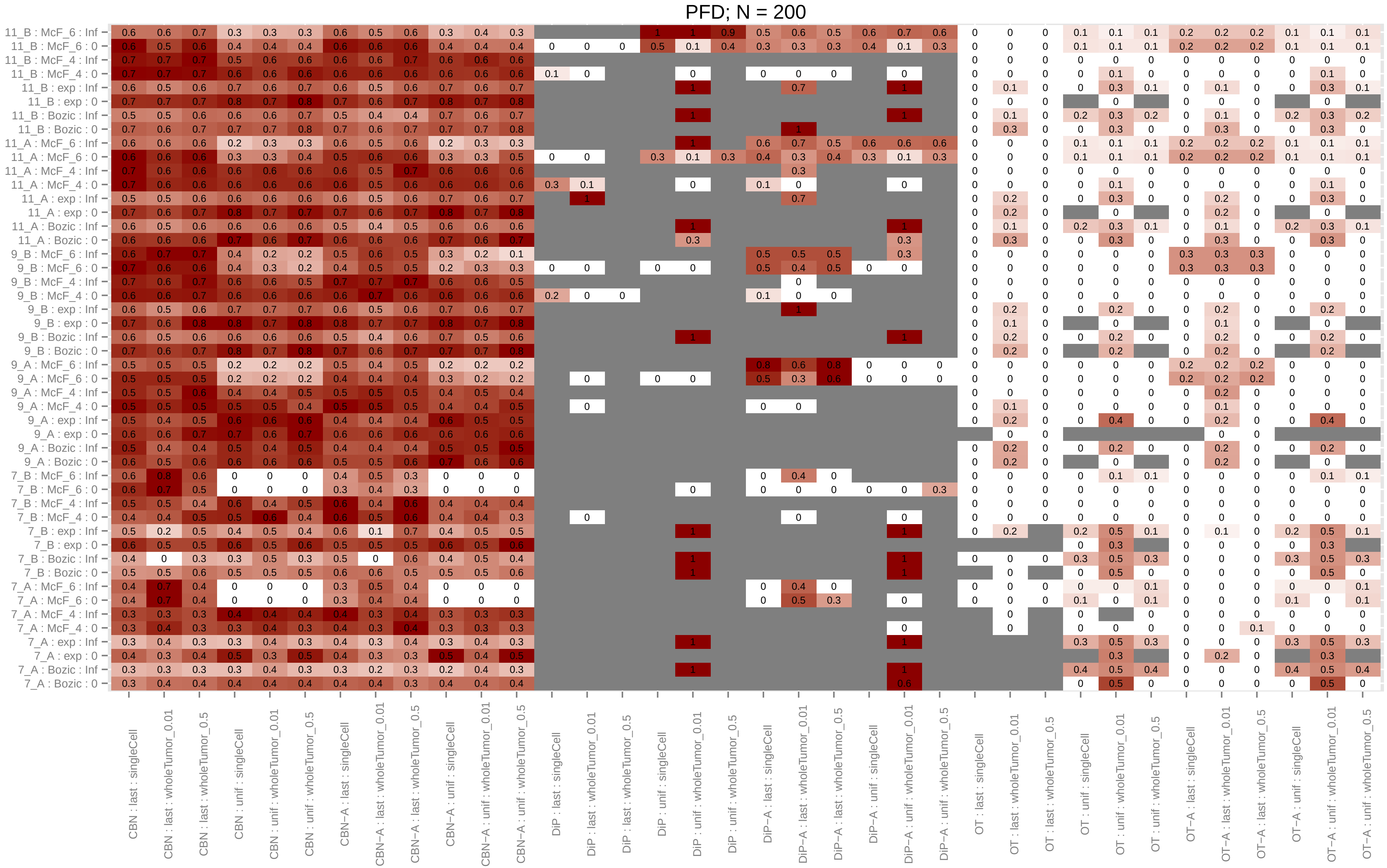

Figure 8: Drivers Known, PFD, N = 200

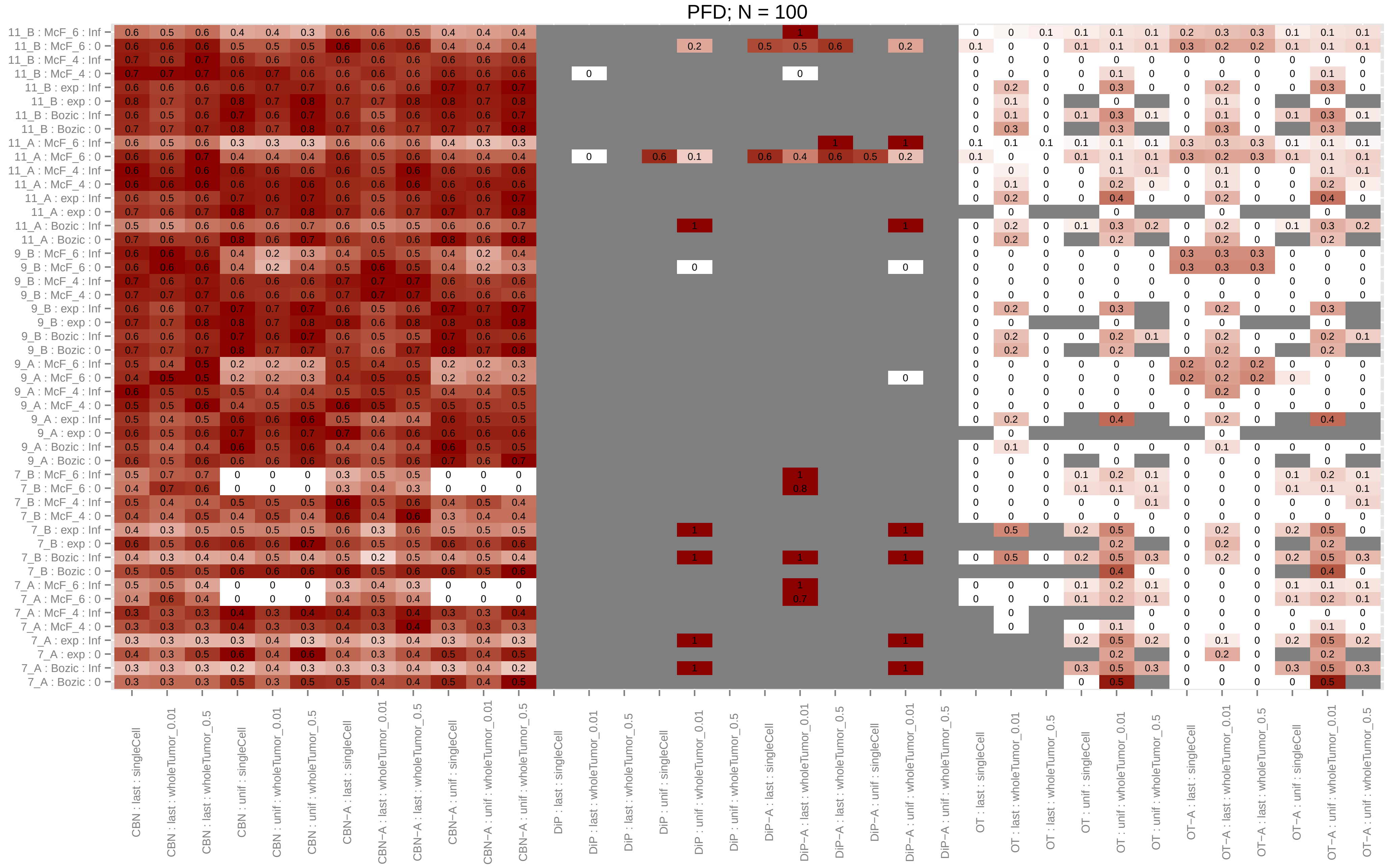

Figure 9: Drivers Known, PFD, N = 100

2.4 PND, Drivers Known

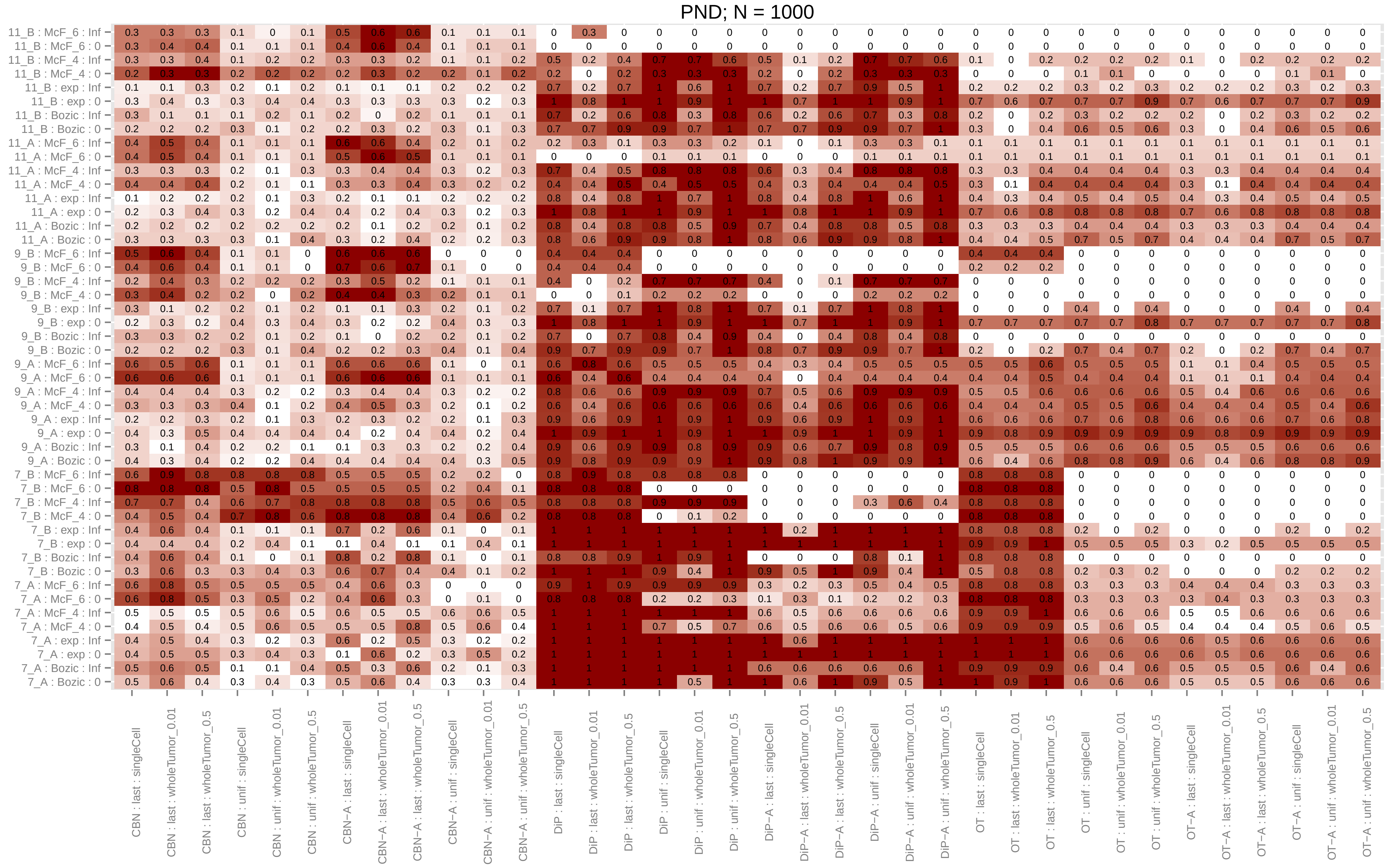

Figure 10: Drivers Known, PND, N = 1000

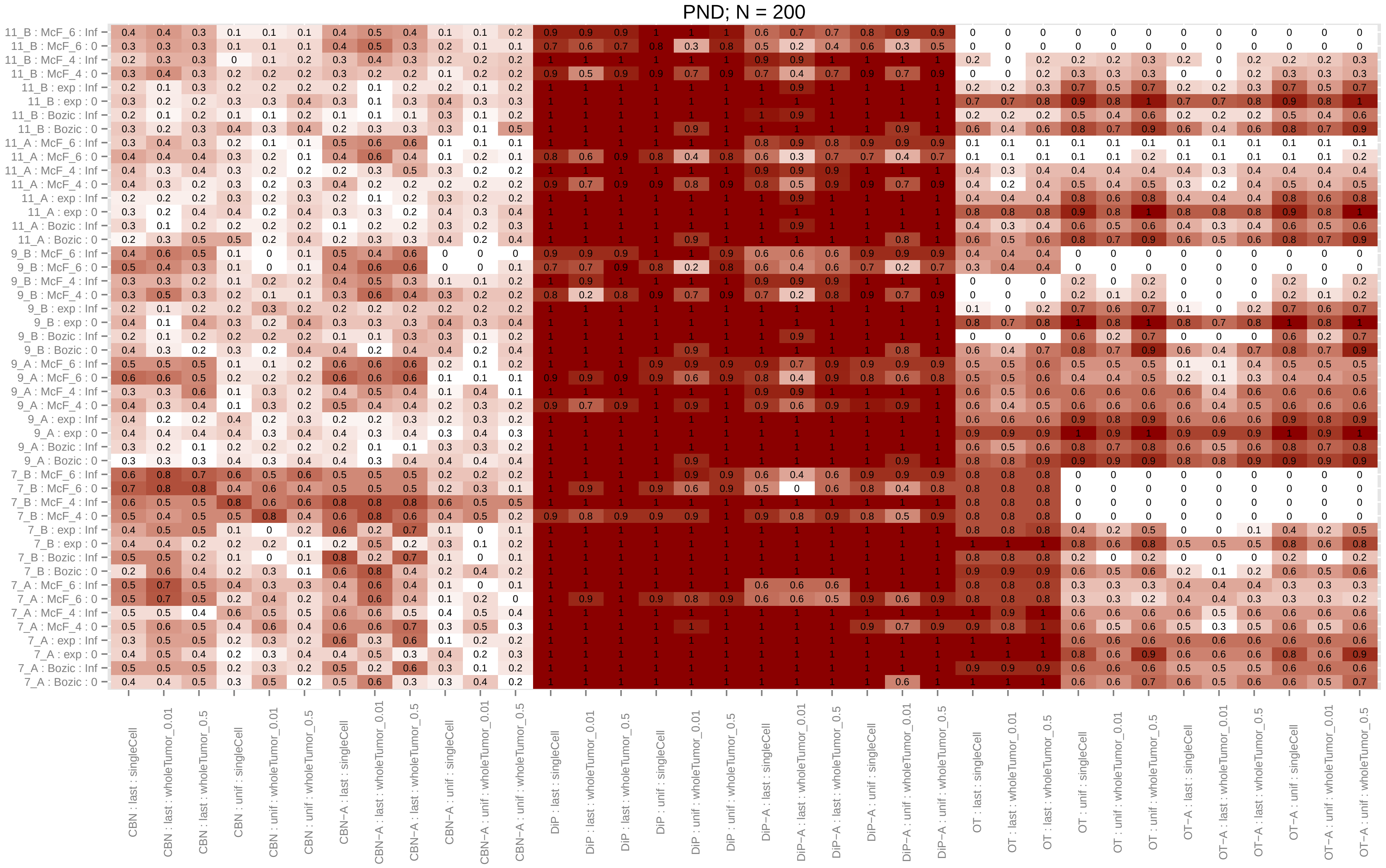

Figure 11: Drivers Known, PND, N = 200

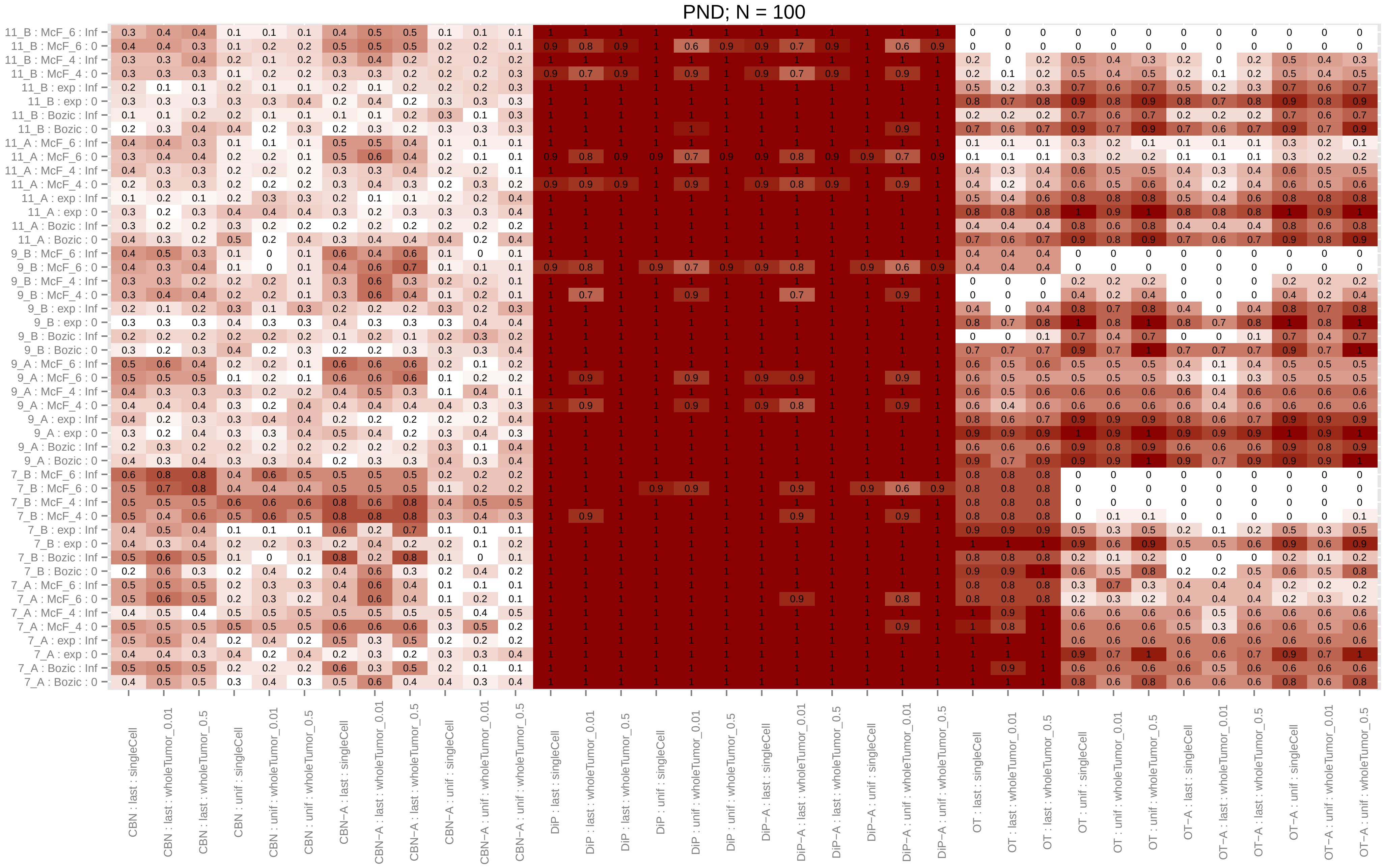

Figure 12: Drivers Known, PND, N = 100

2.5 FPF, Drivers Known

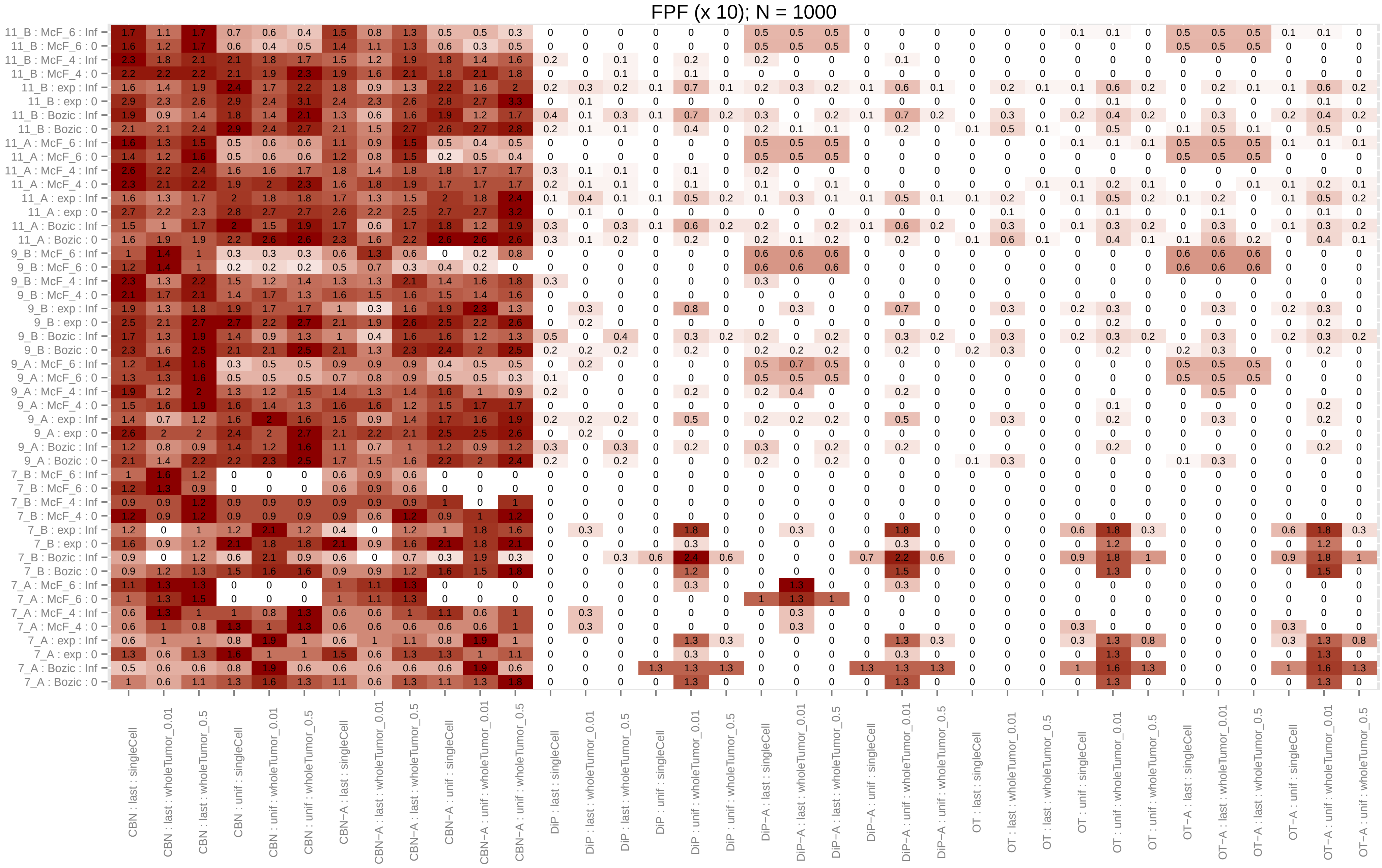

Figure 13: Drivers Known, FPF, N = 1000

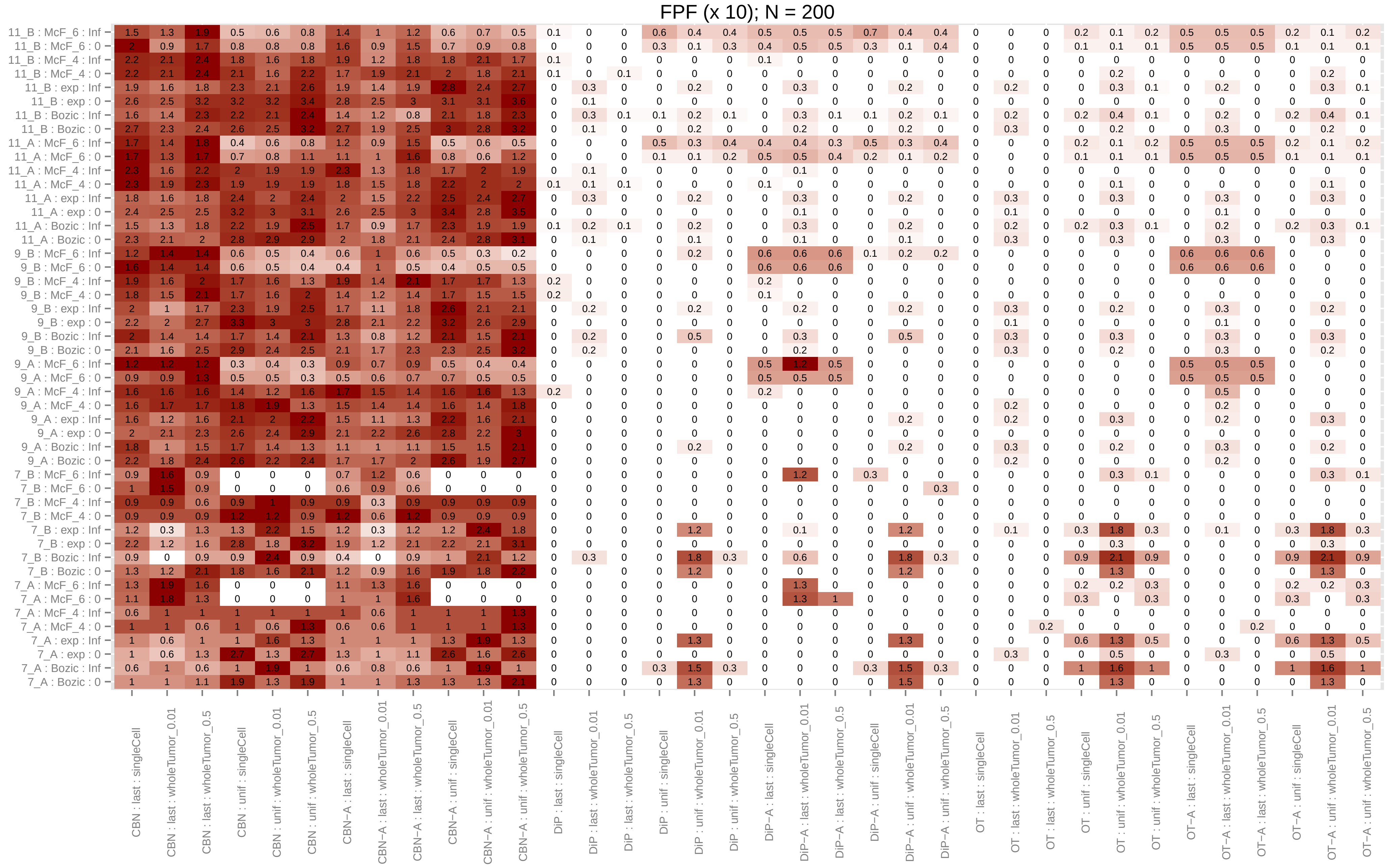

Figure 14: Drivers Known, FPF, N = 200

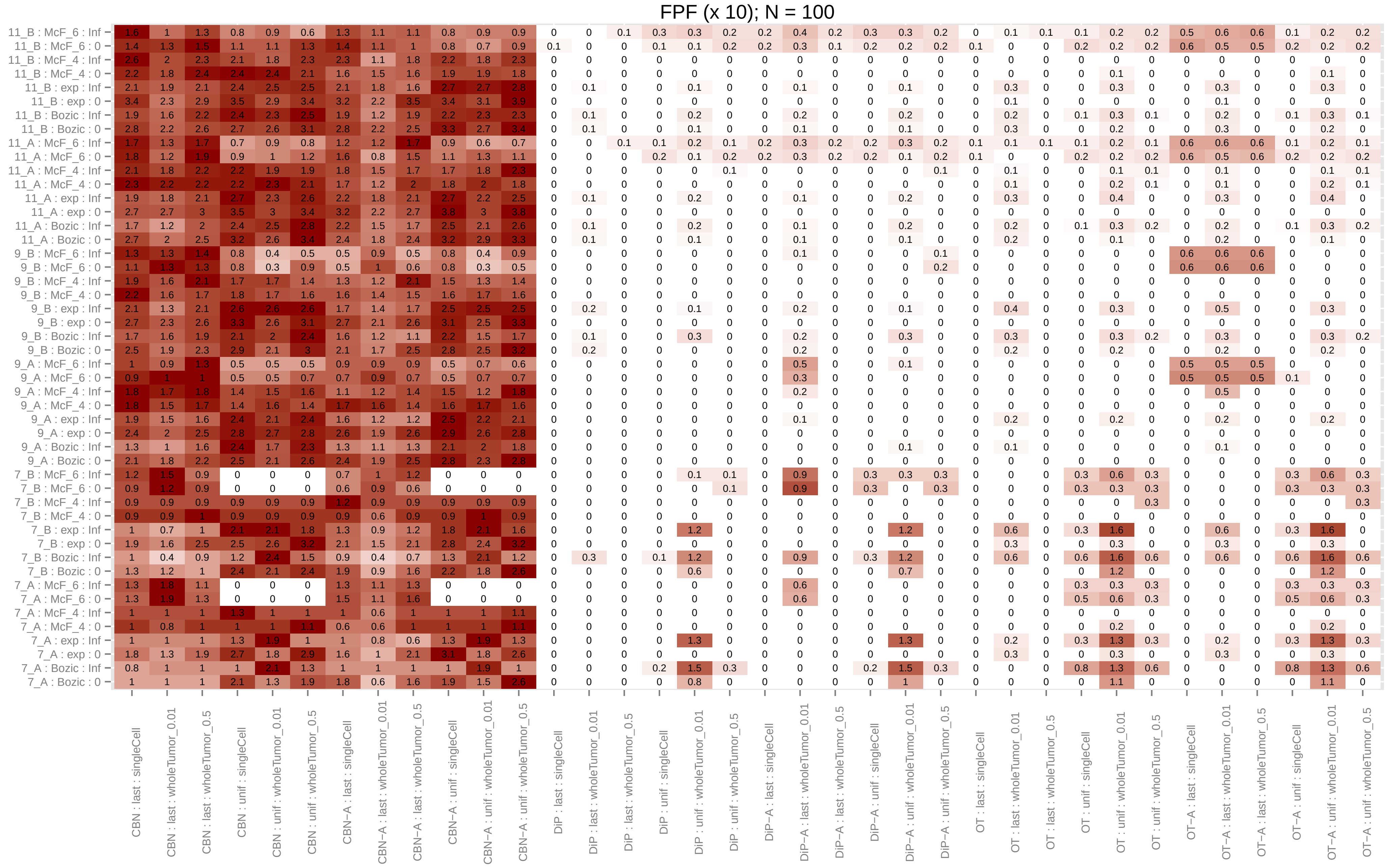

Figure 15: Drivers Known, FPF, N = 100

3 Drivers Unknown

3.1 Inferred edges, Drivers Unknown

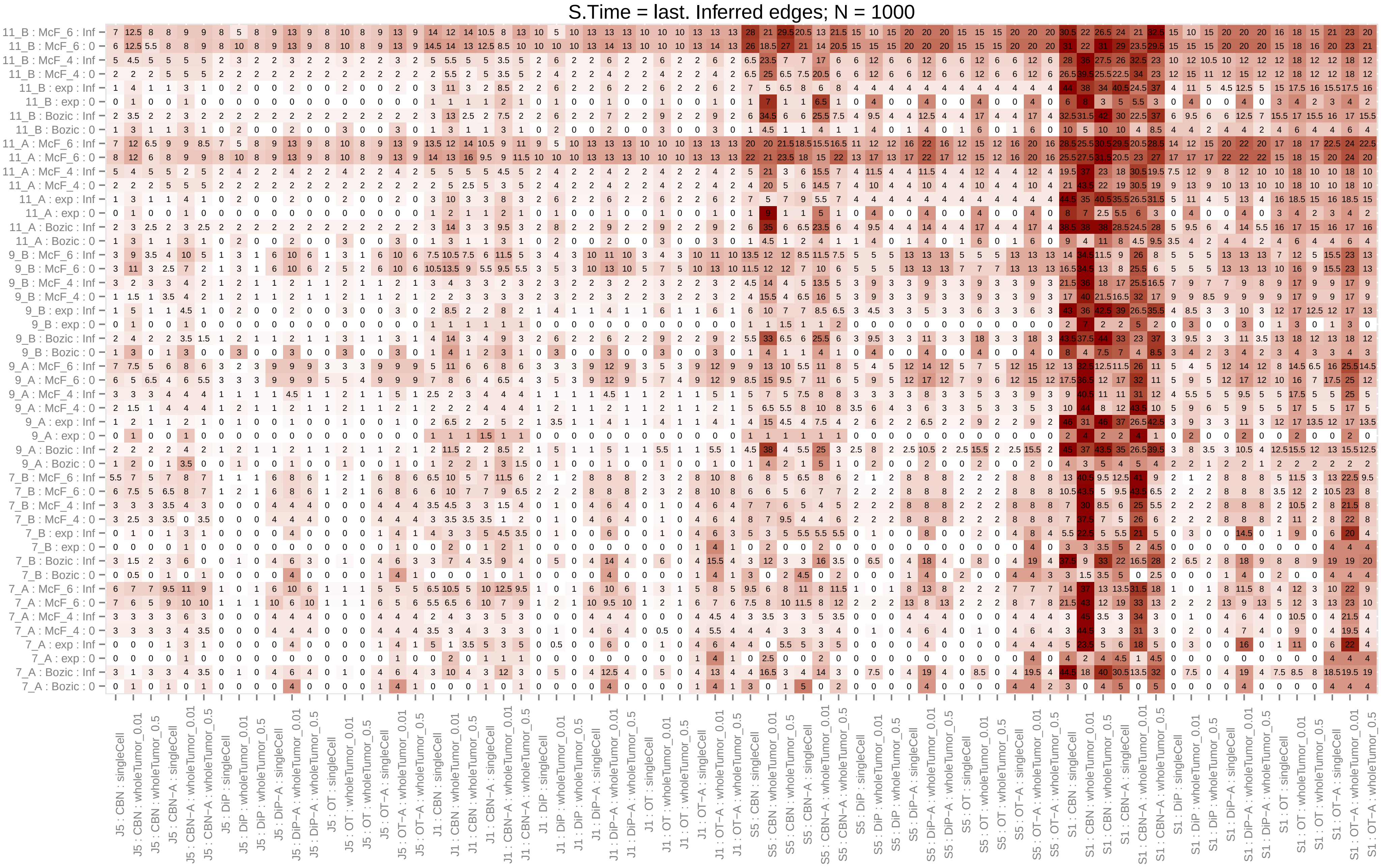

Figure 16: Drivers Unknown, number of inferred edges (not counting those from Root), N = 1000, S.Time = last.

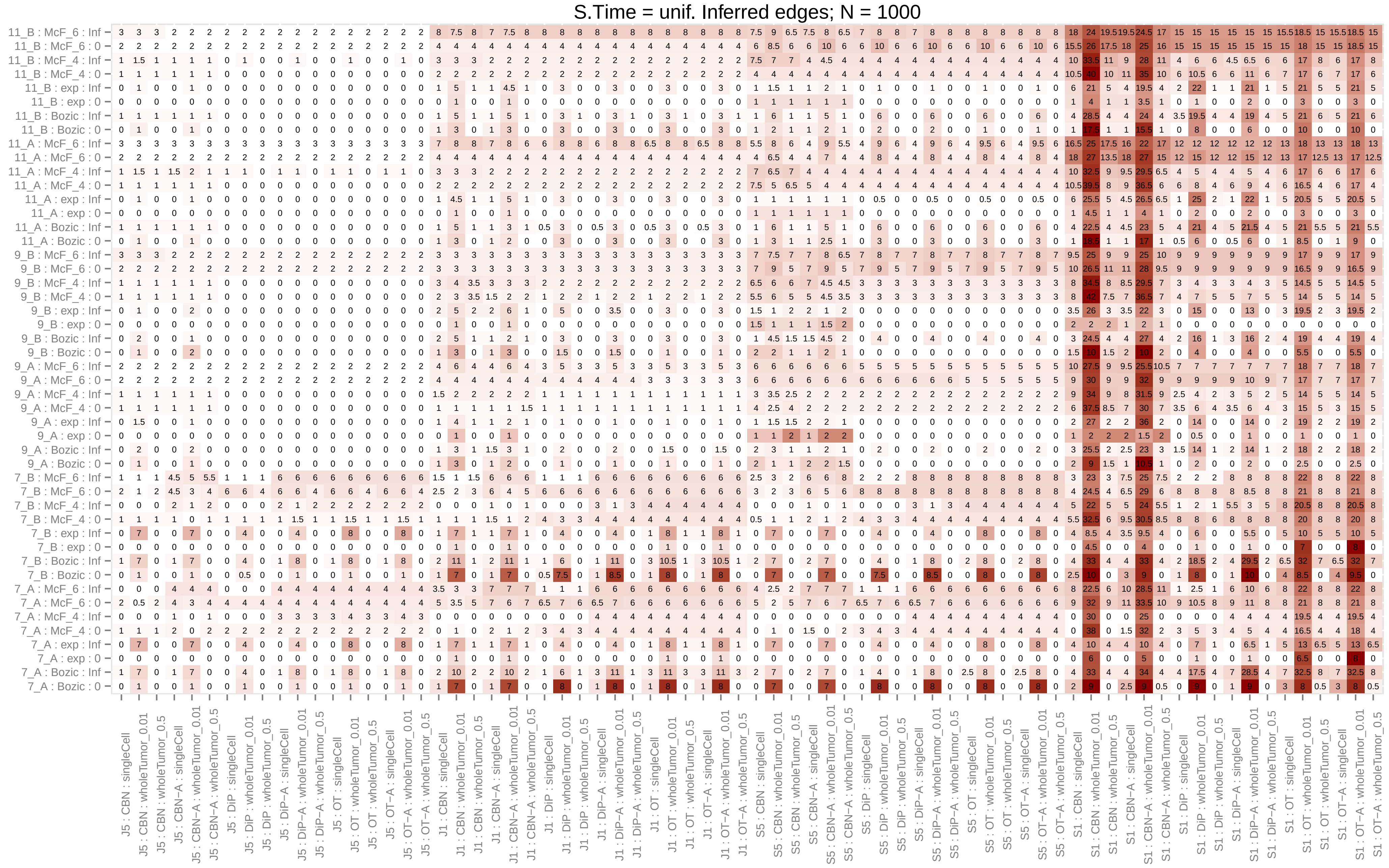

Figure 17: Drivers Unknown, number of inferred edges (not counting those from Root), N = 1000, S.Time = unif.

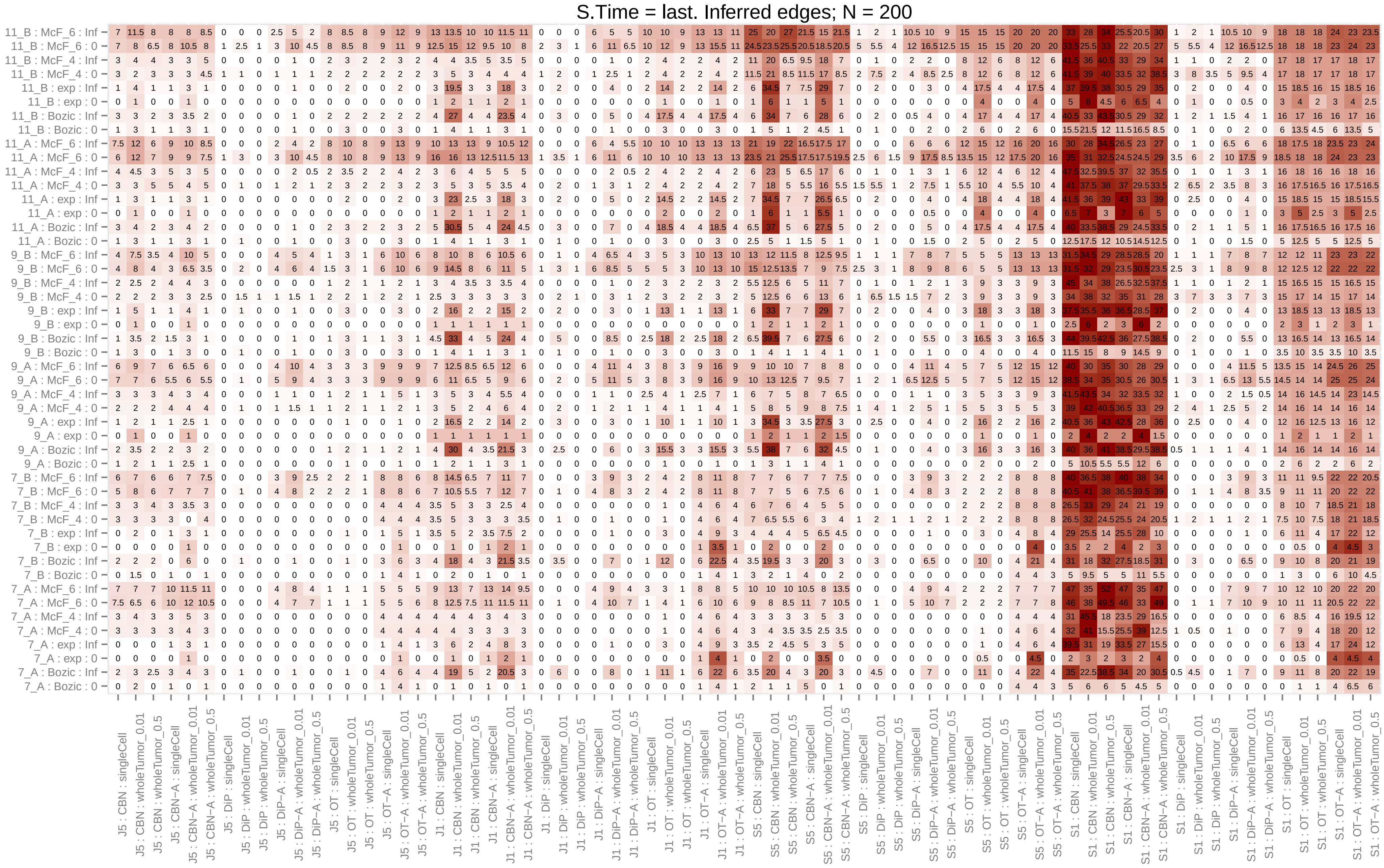

Figure 18: Drivers Unknown, number of inferred edges (not counting those from Root), N = 200, S.Time = last

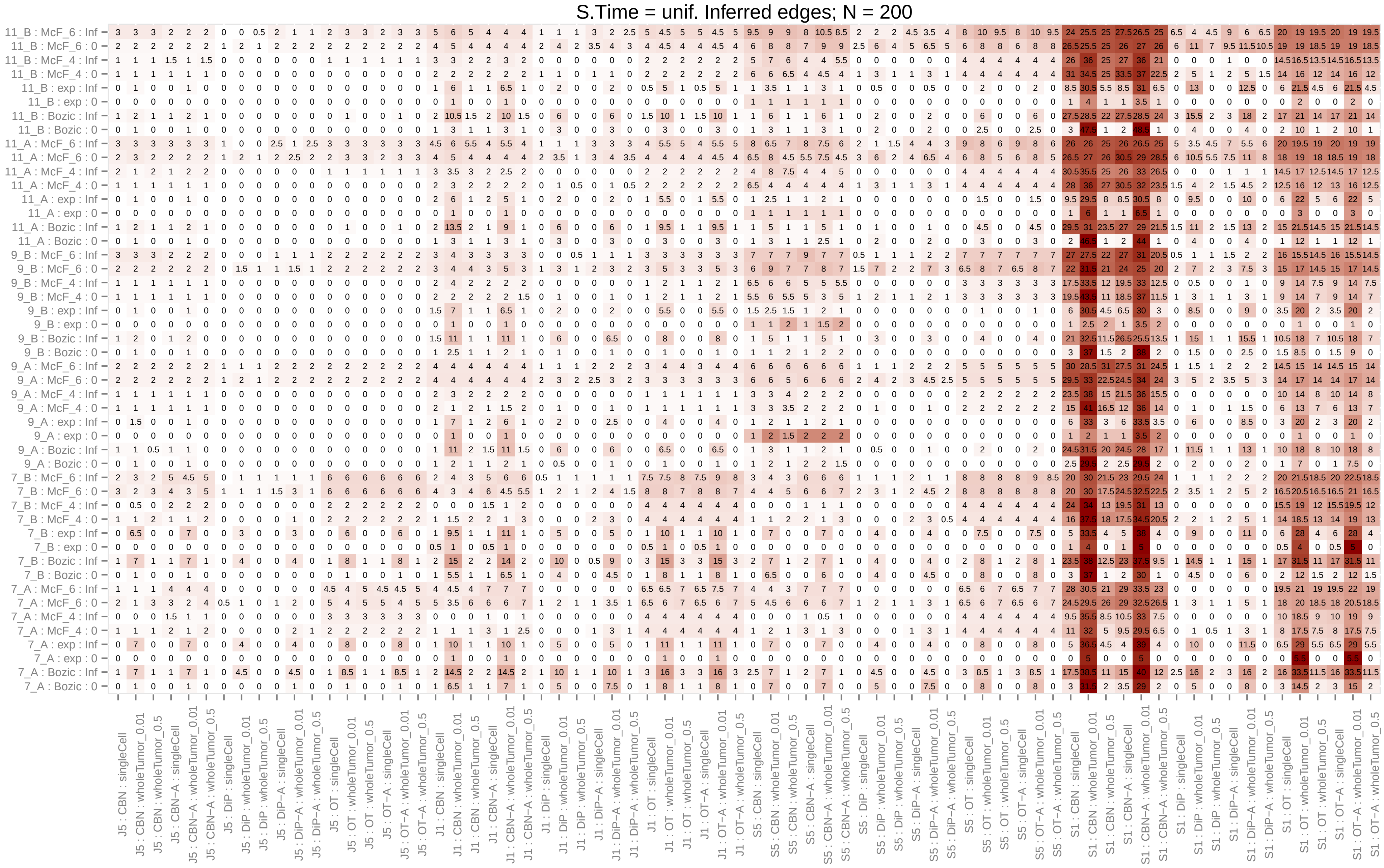

Figure 19: Drivers Unknown, number of inferred edges (not counting those from Root), N = 200, S.Time = unif.

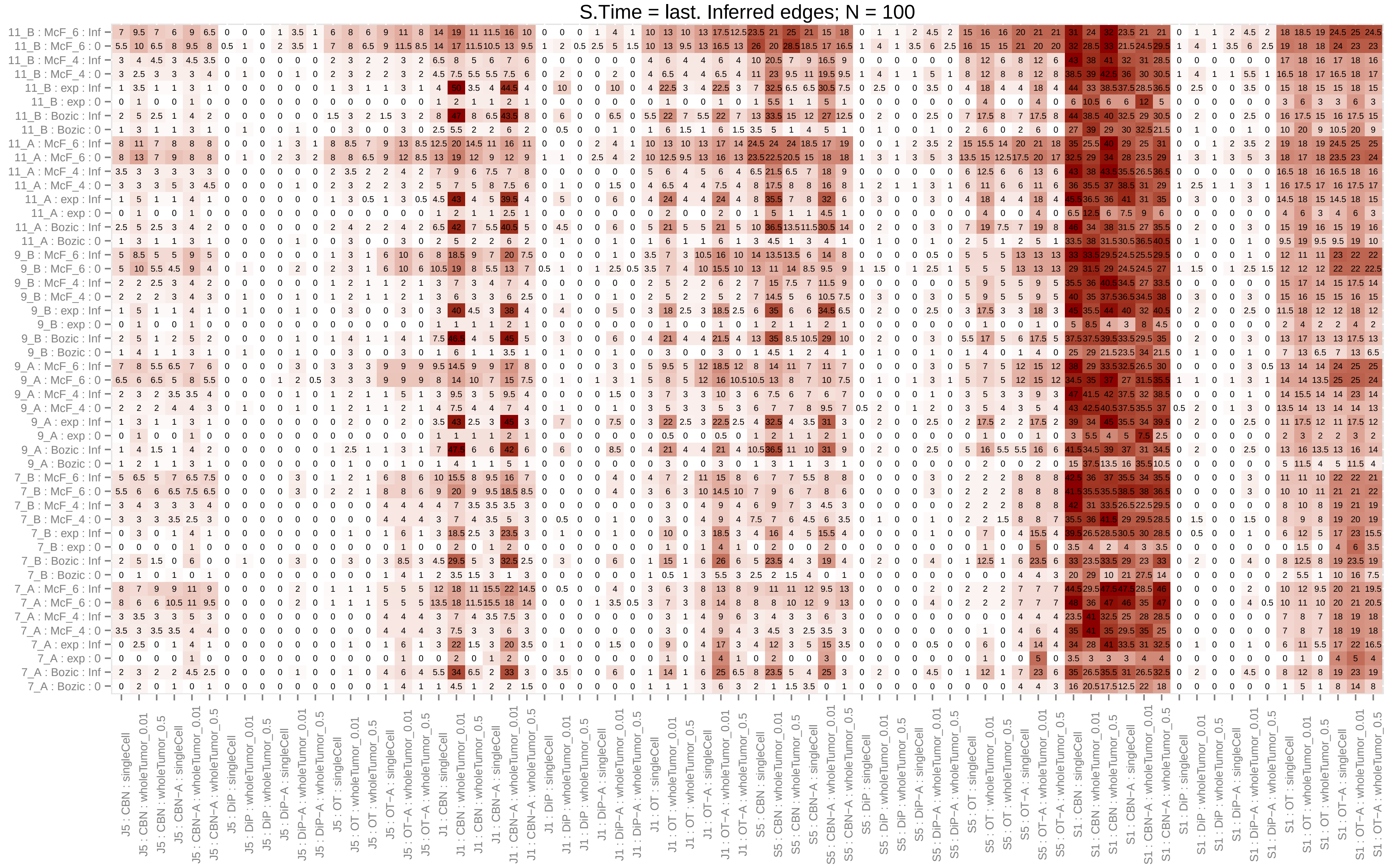

Figure 20: Drivers Unknown, number of inferred edges (not counting those from Root), N = 100, S.Time = last

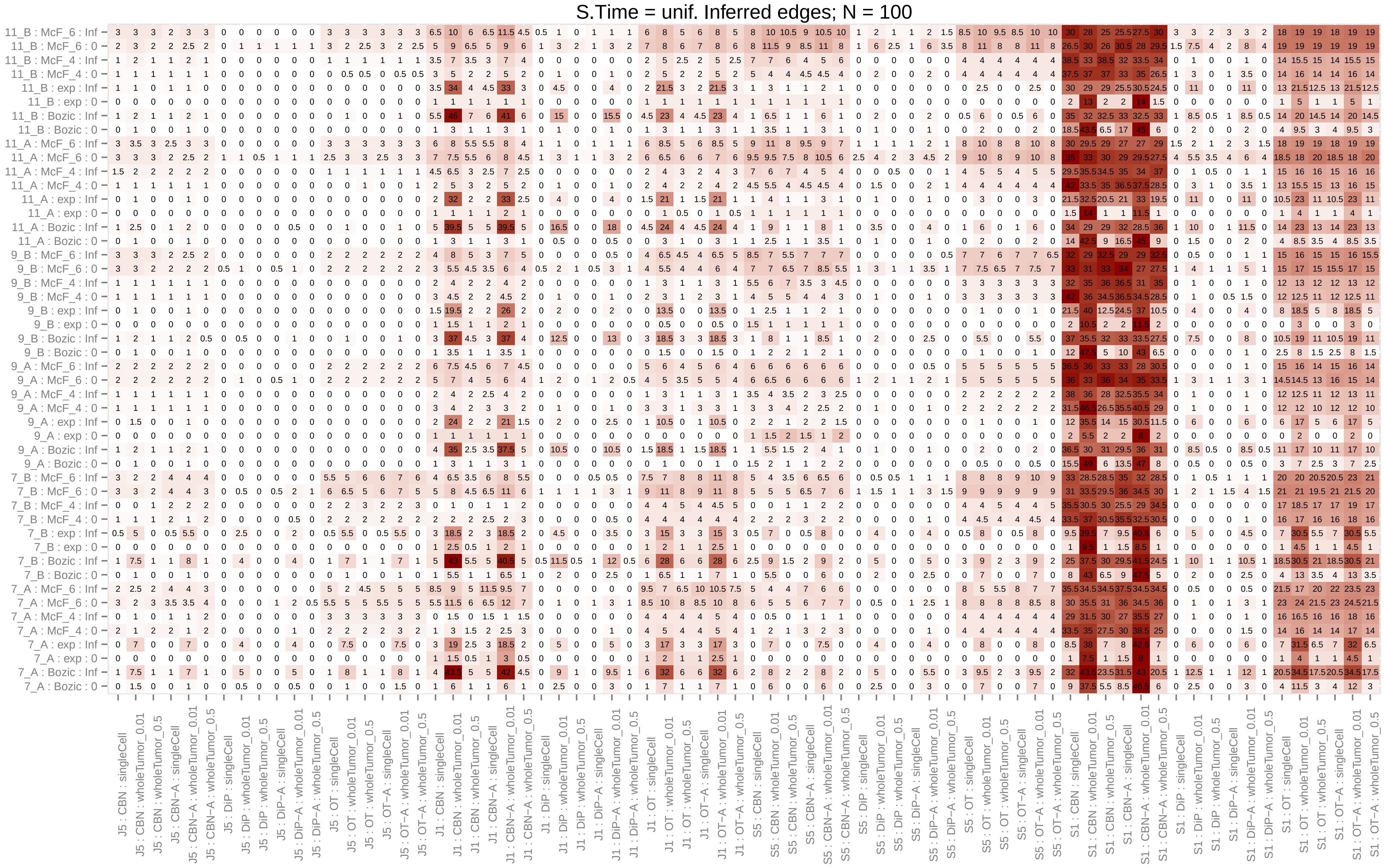

Figure 21: Drivers Unknown, number of inferred edges (not counting those from Root), N = 100, S.Time = unif.

3.2 Diff, Drivers Unknown

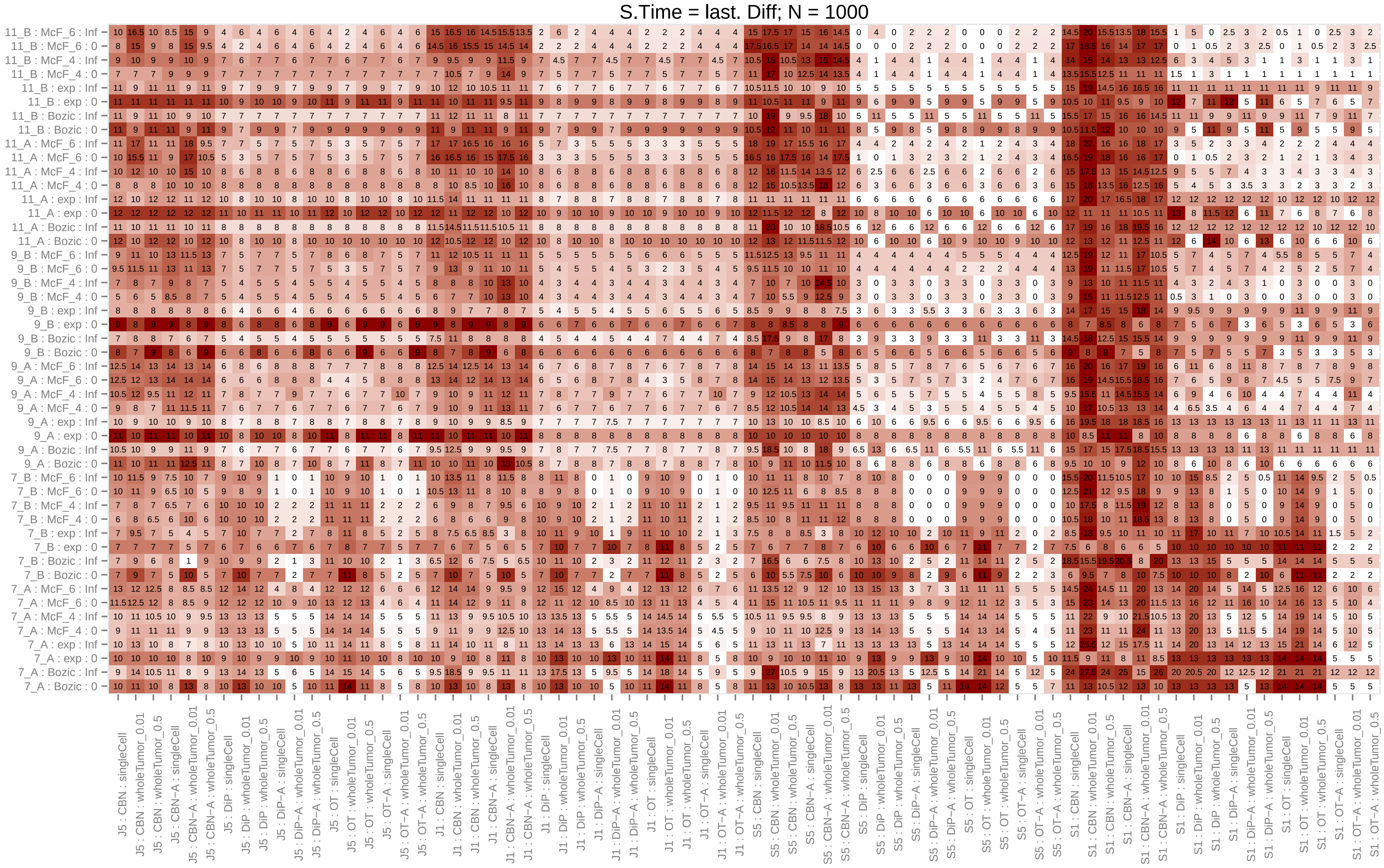

Figure 22: Drivers Unknown, Diff, N = 1000, S.Time = last

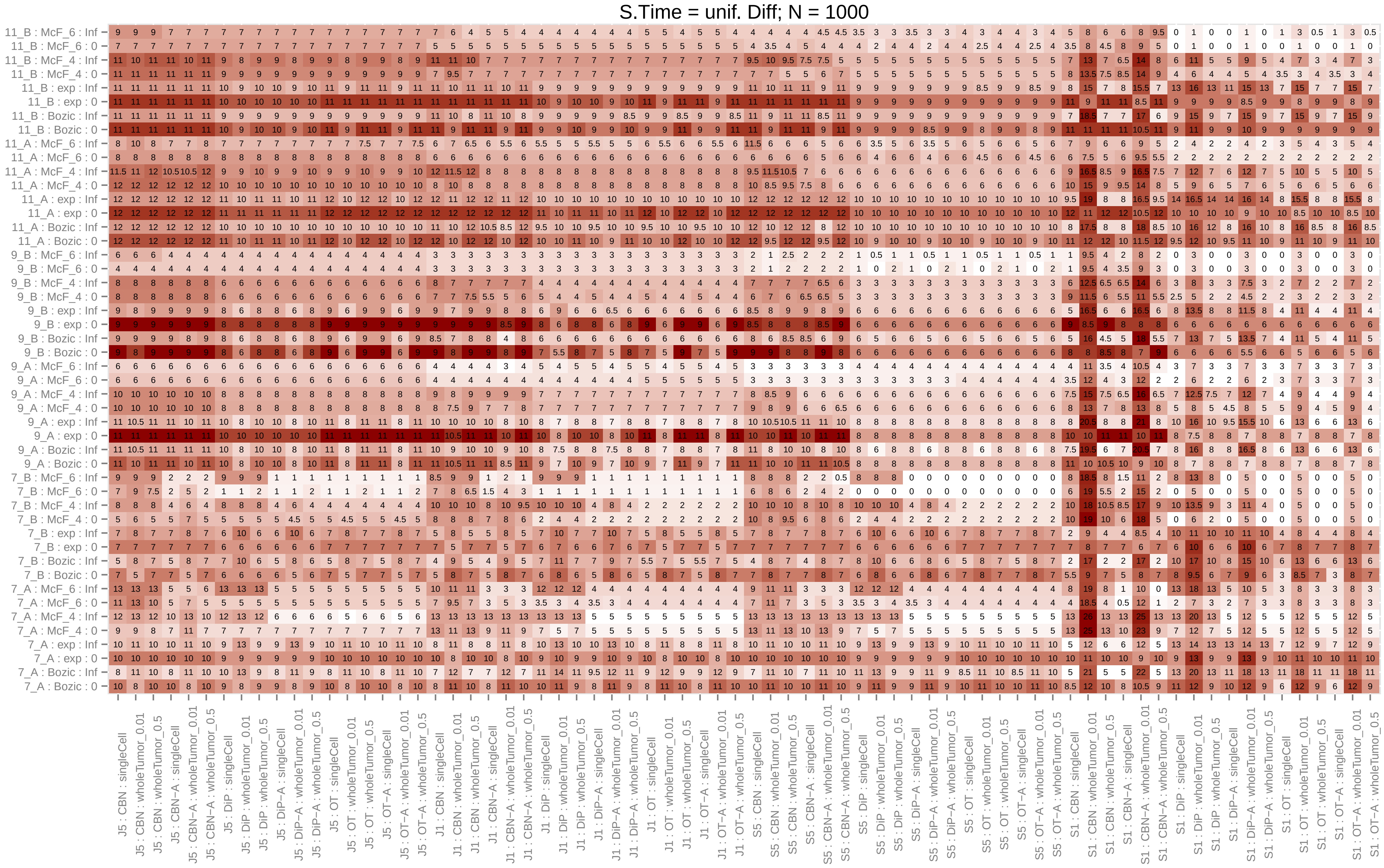

Figure 23: Drivers Unknown, Diff, N = 1000, S.Time = unif.

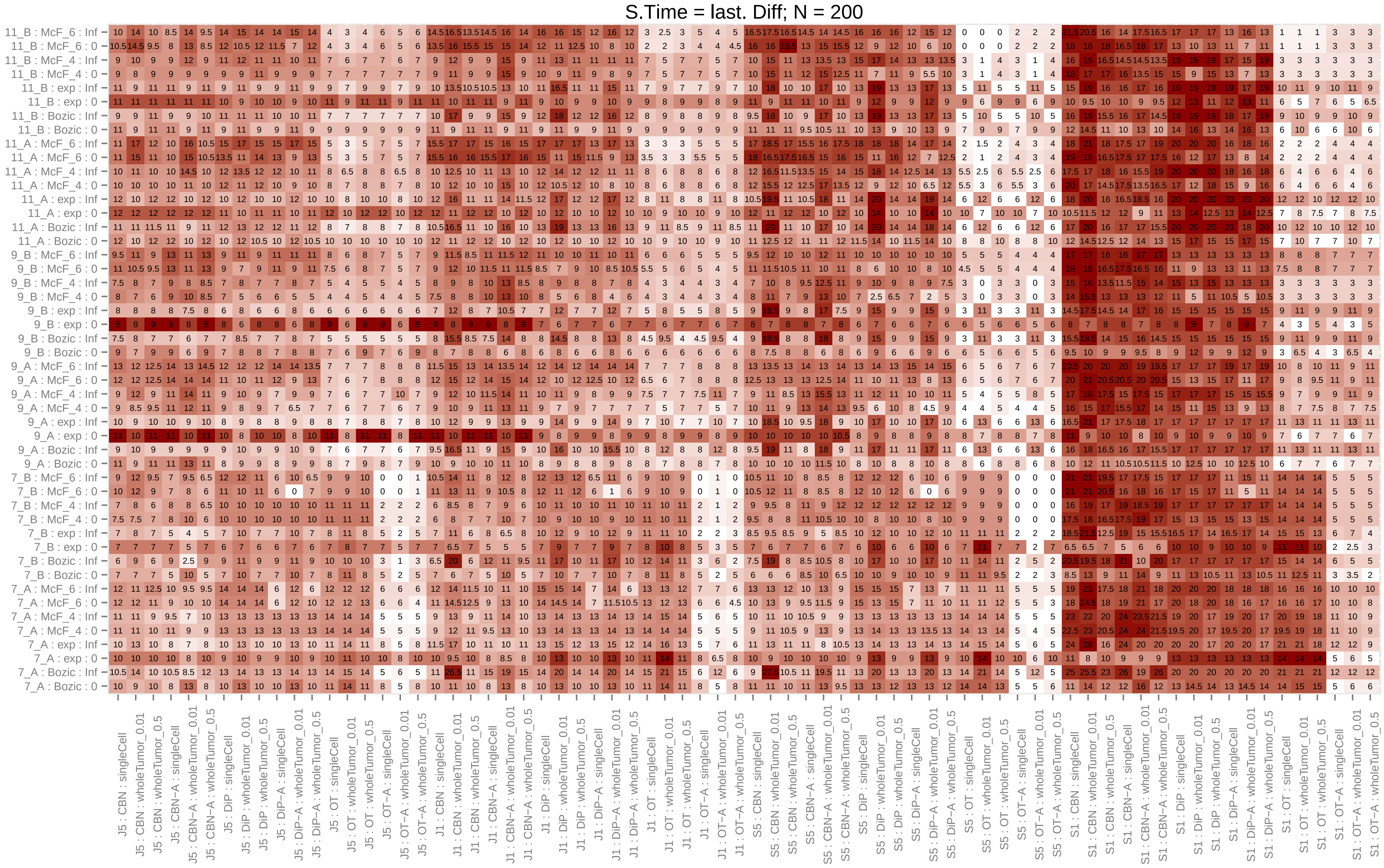

Figure 24: Drivers Unknown, Diff, N = 200, S.Time = last

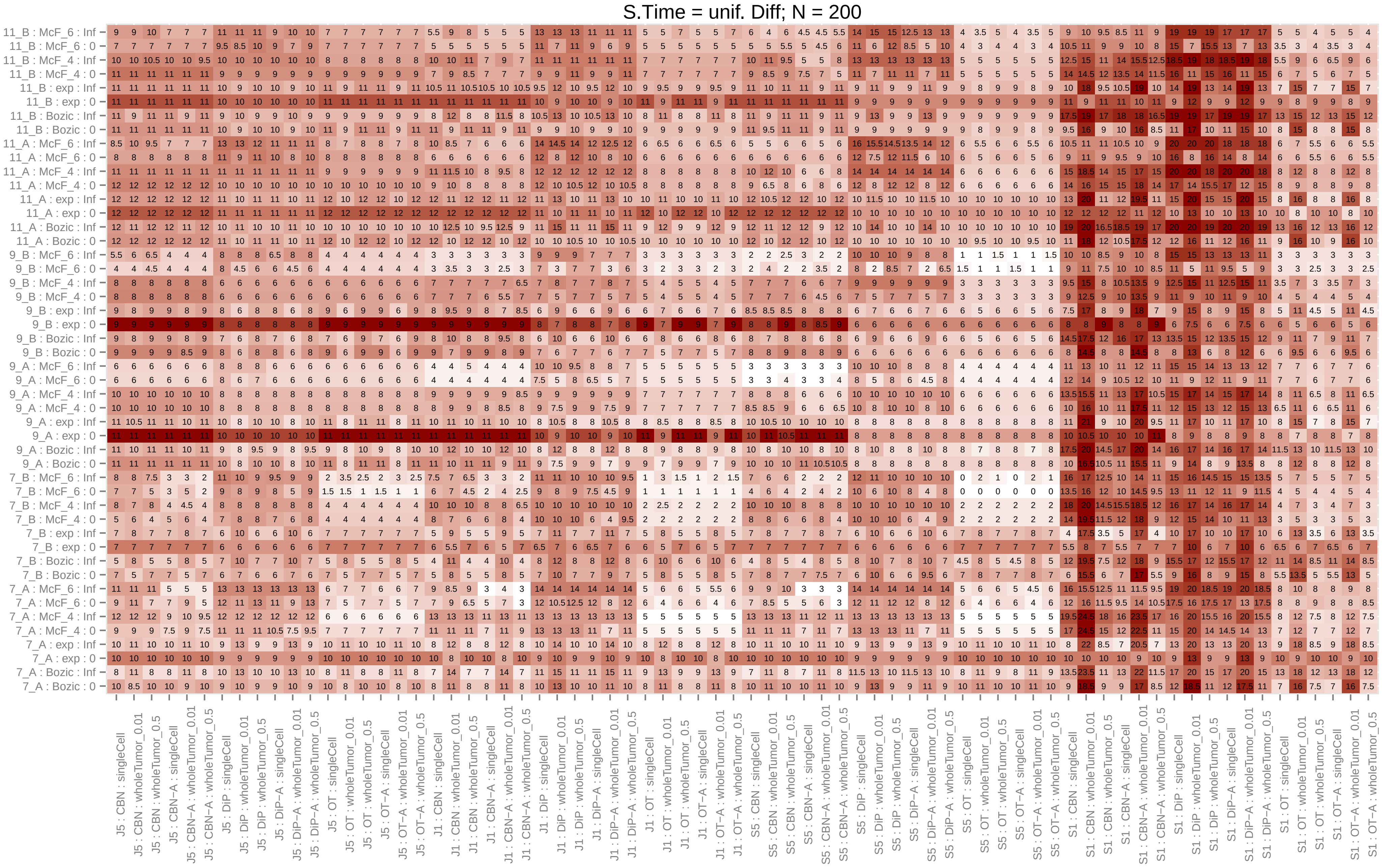

Figure 25: Drivers Unknown, Diff, N = 200, S.Time = unif.

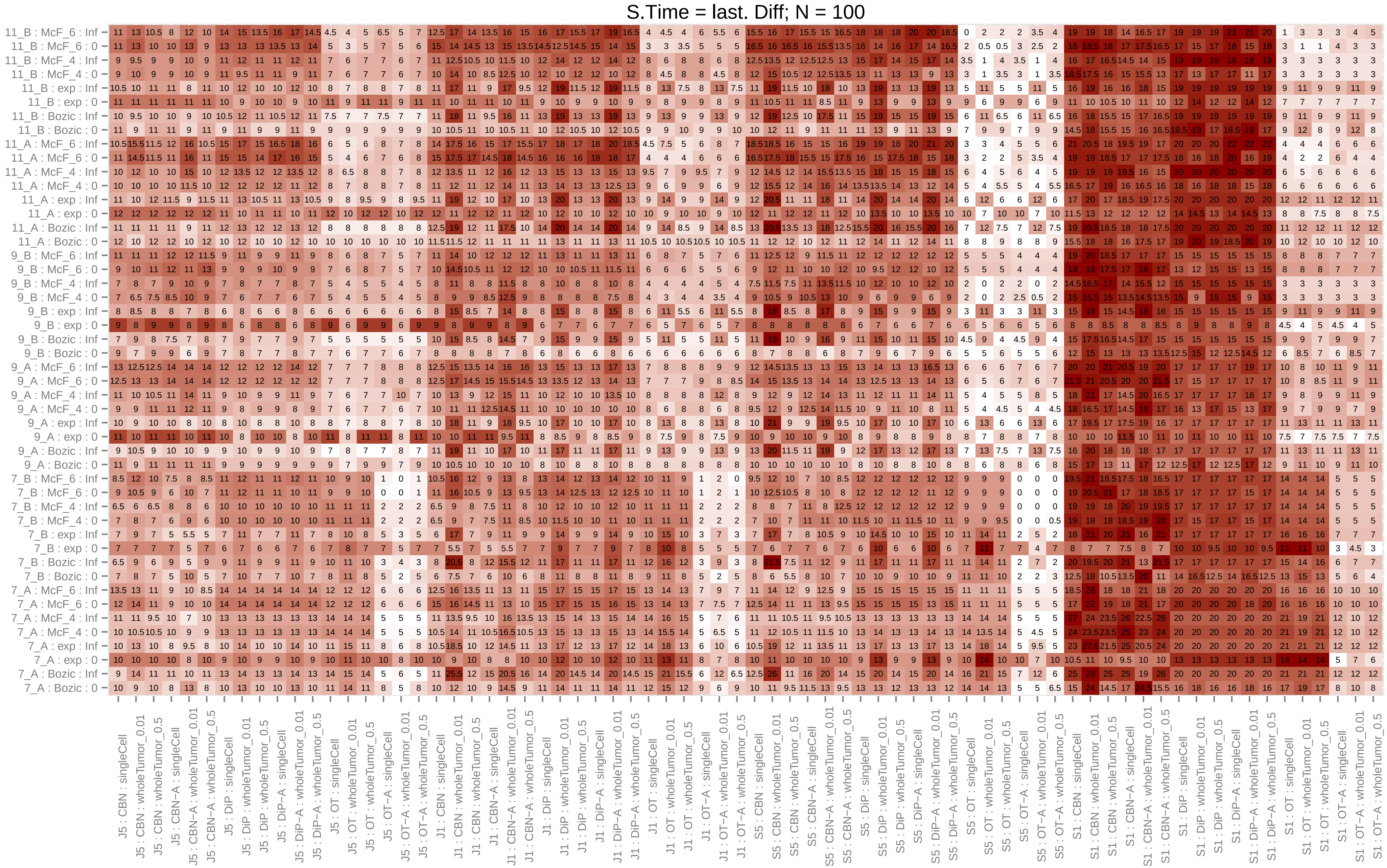

Figure 26: Drivers Unknown, Diff, N = 100, S.Time = last

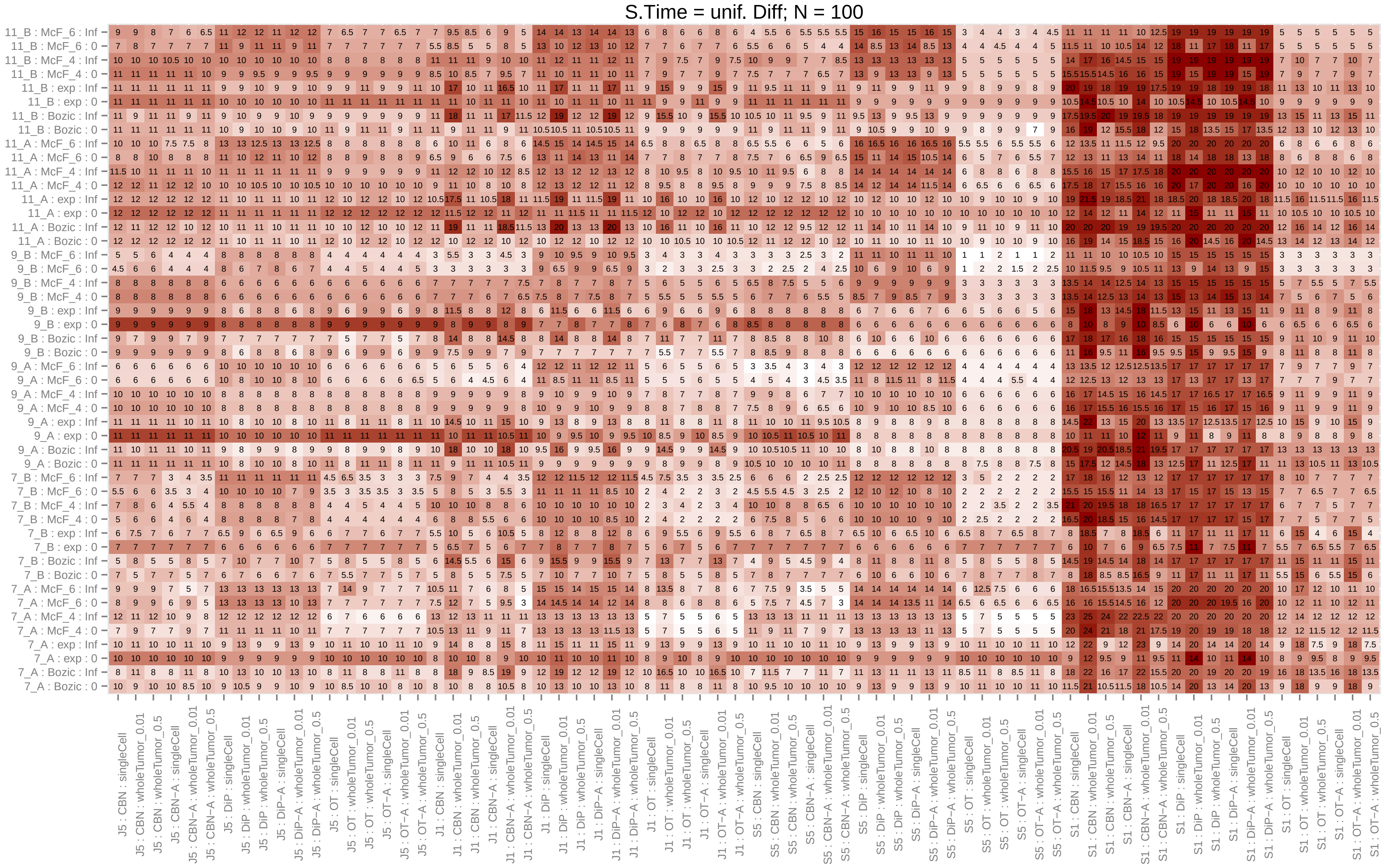

Figure 27: Drivers Unknown, Diff, N = 100, S.Time = unif.

3.3 PFD, Drivers Unknown

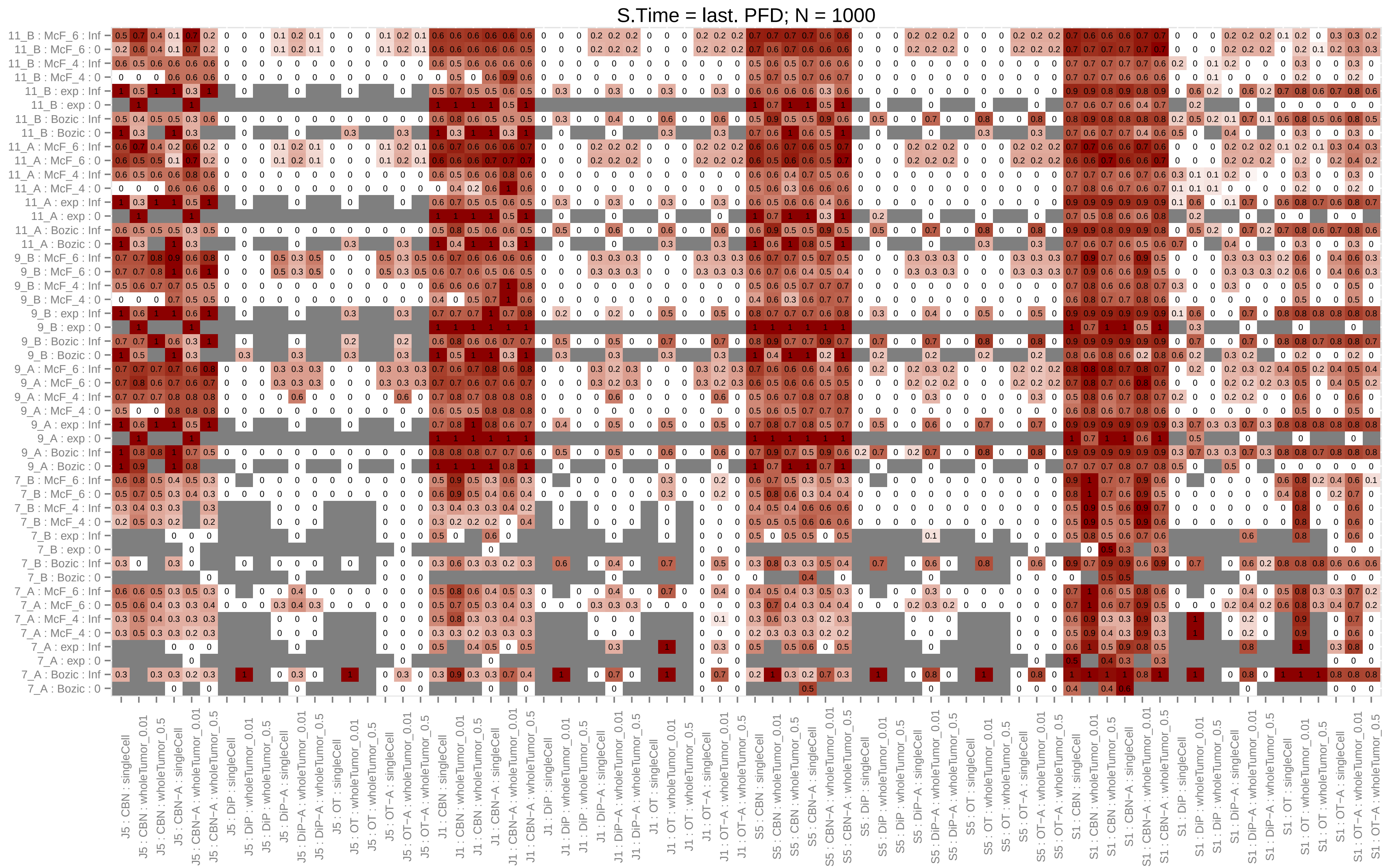

Figure 28: Drivers Unknown, PFD, N = 1000, S.Time = last

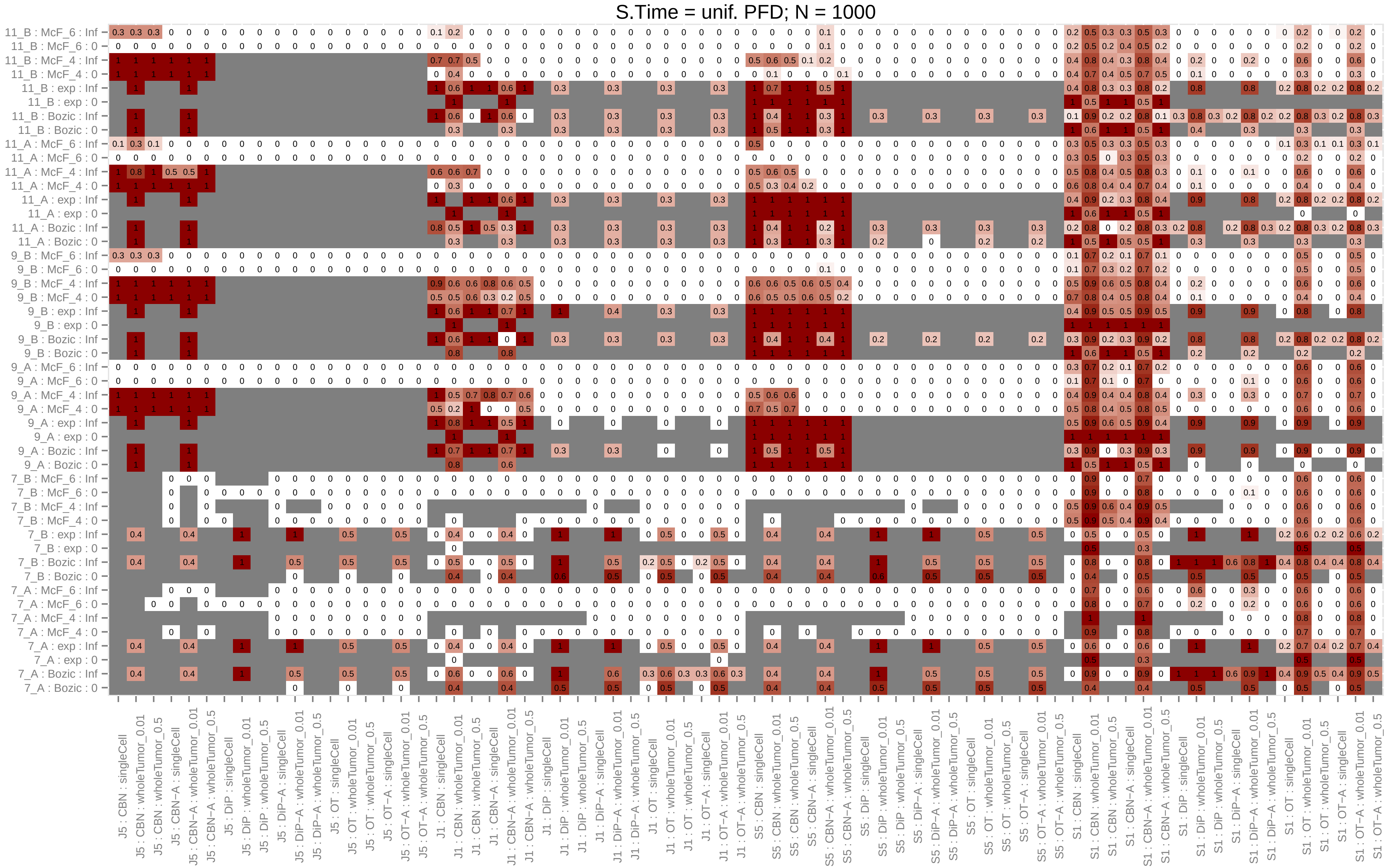

Figure 29: Drivers Unknown, PFD, N = 1000, S.Time = unif.

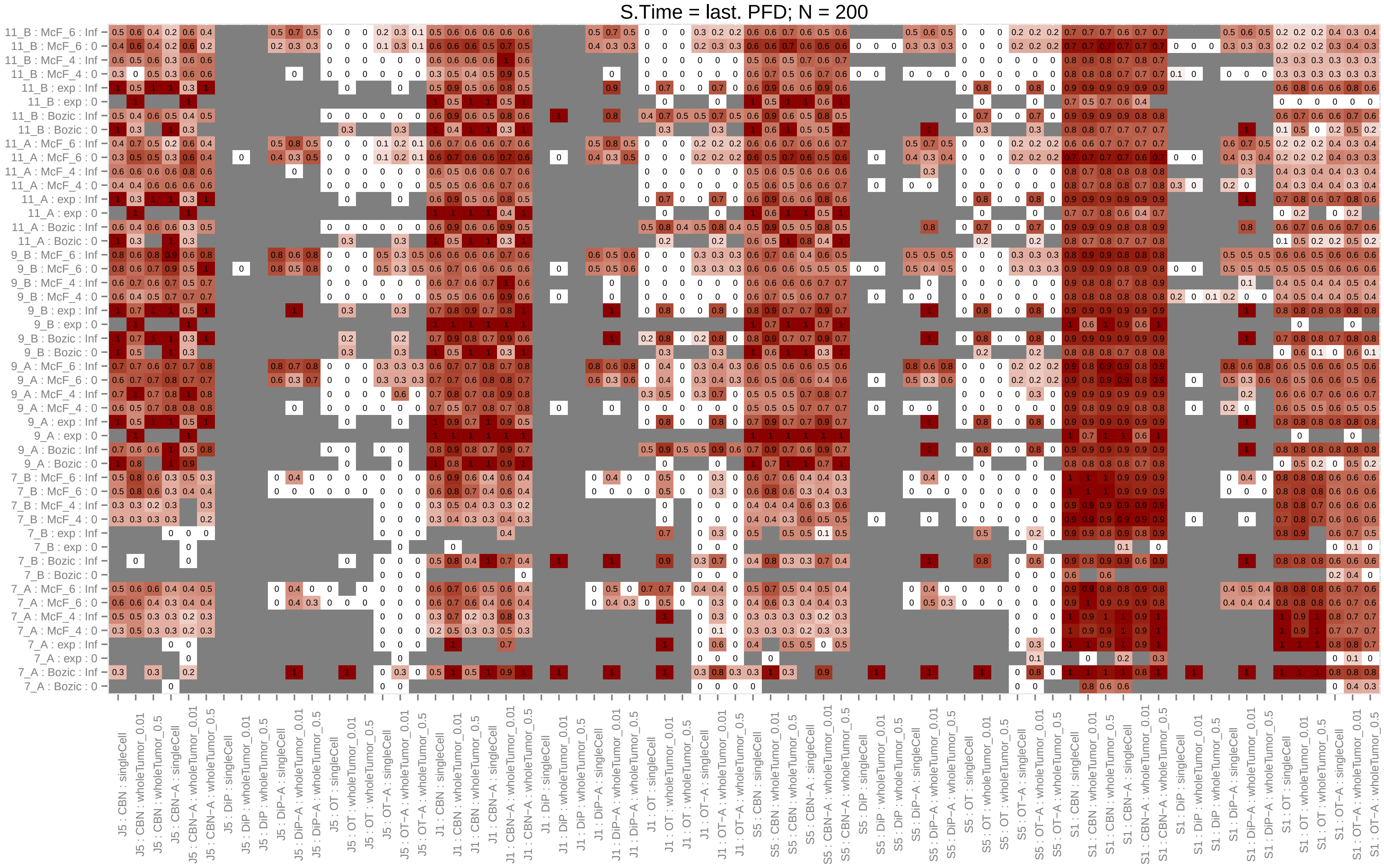

Figure 30: Drivers Unknown, PFD, N = 200, S.Time = last

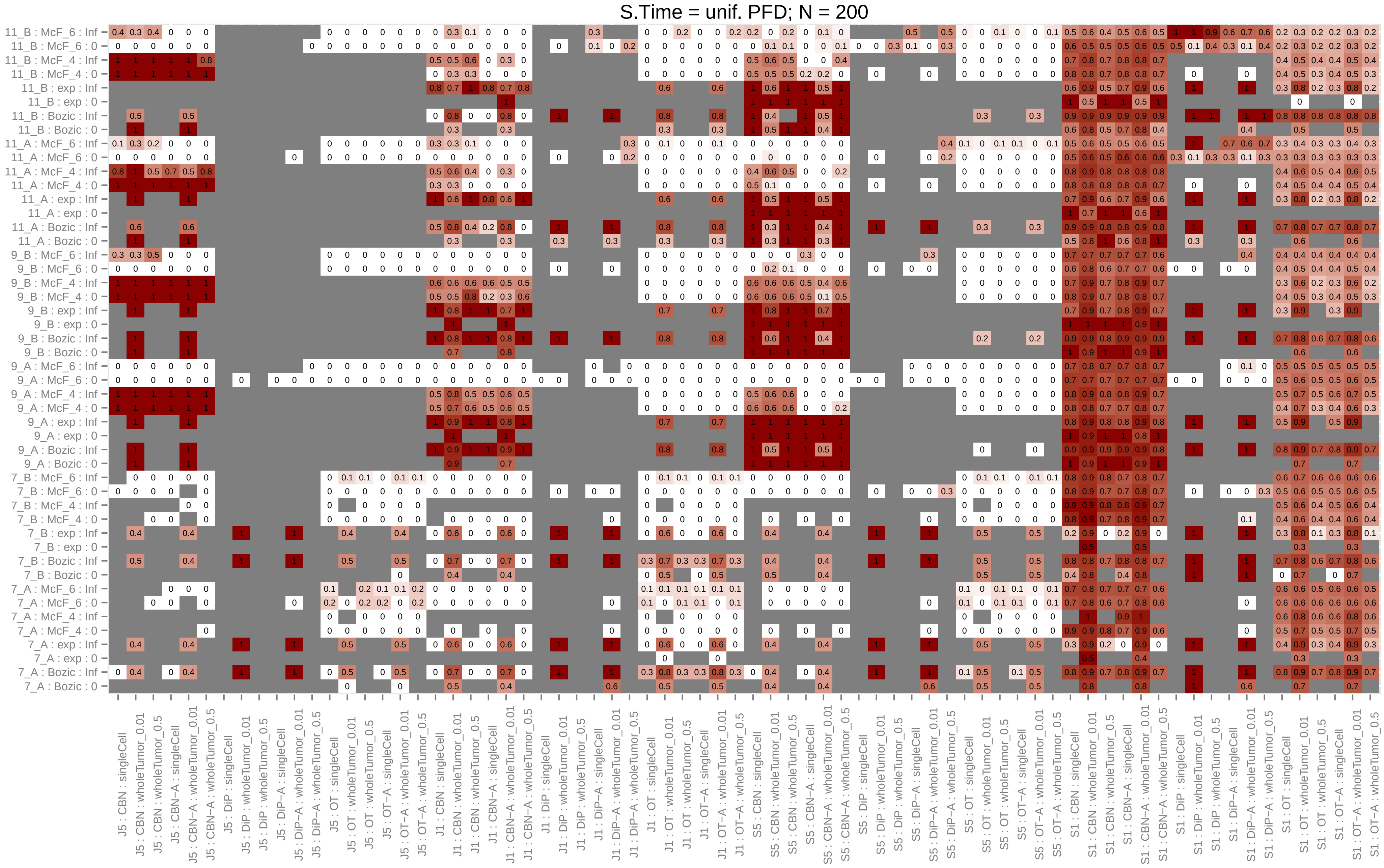

Figure 31: Drivers Unknown, PFD, N = 200, S.Time = unif.

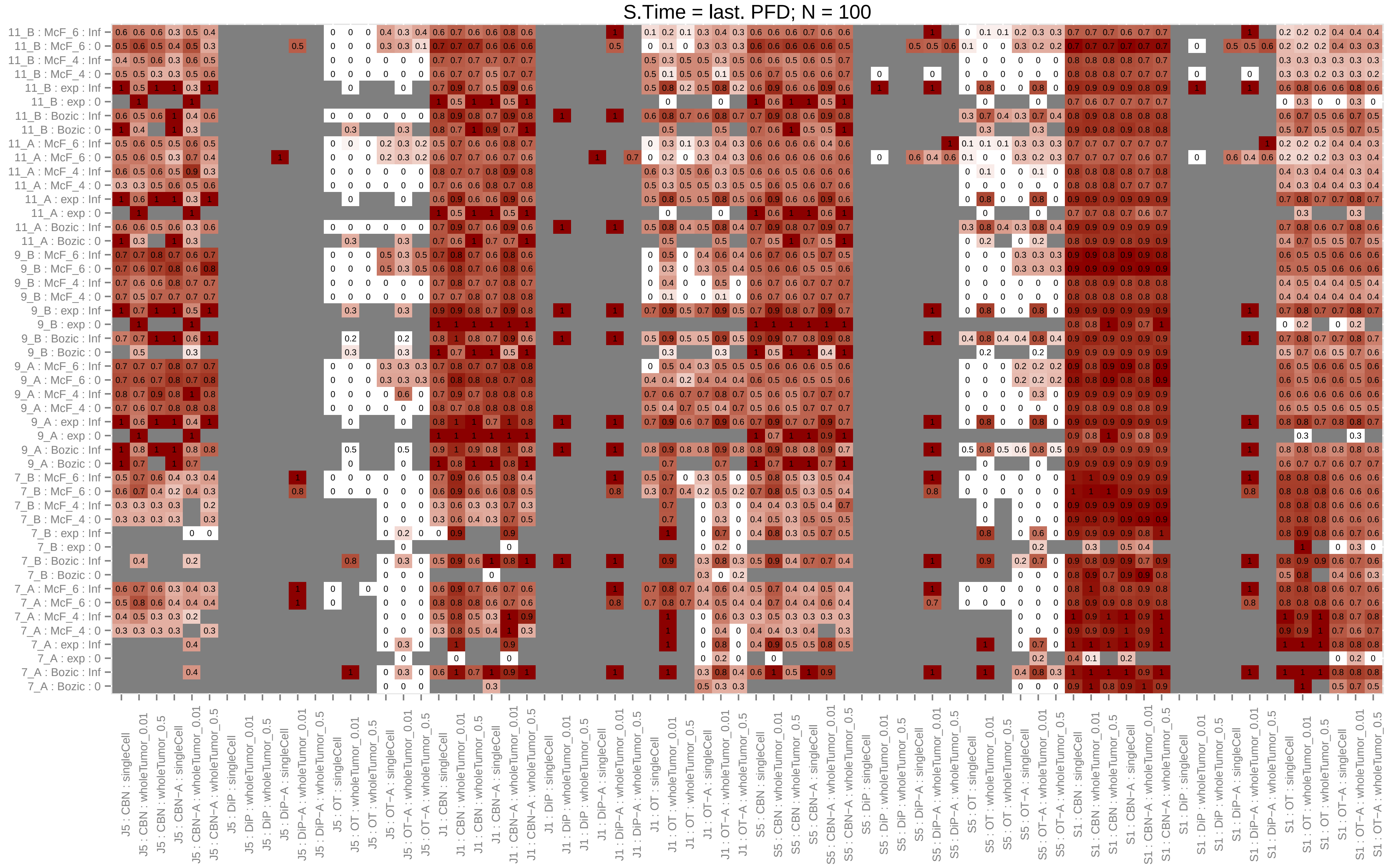

Figure 32: Drivers Unknown, PFD, N = 100, S.Time = last

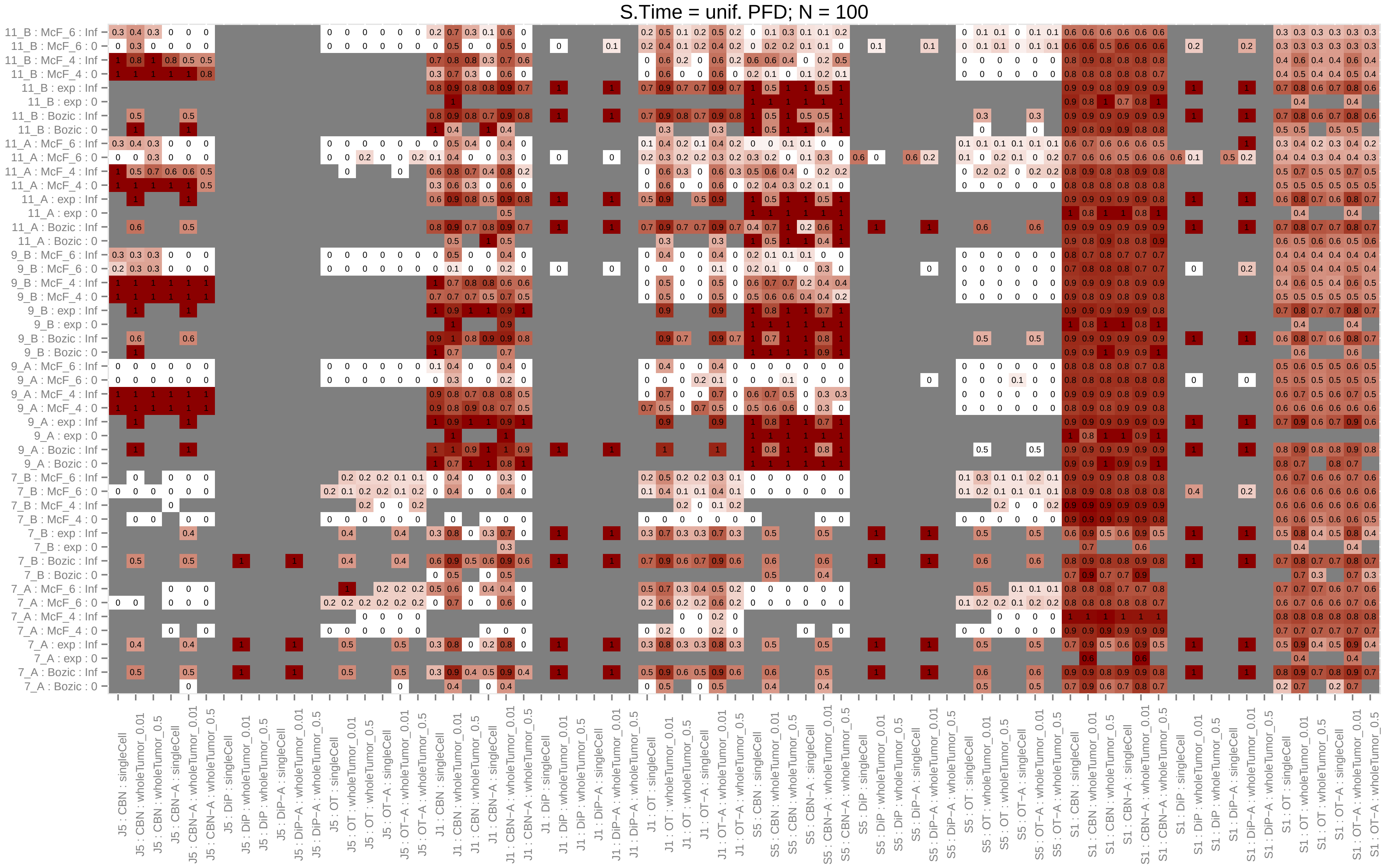

Figure 33: Drivers Unknown, PFD, N = 100, S.Time = unif.

3.4 PND, Drivers Unknown

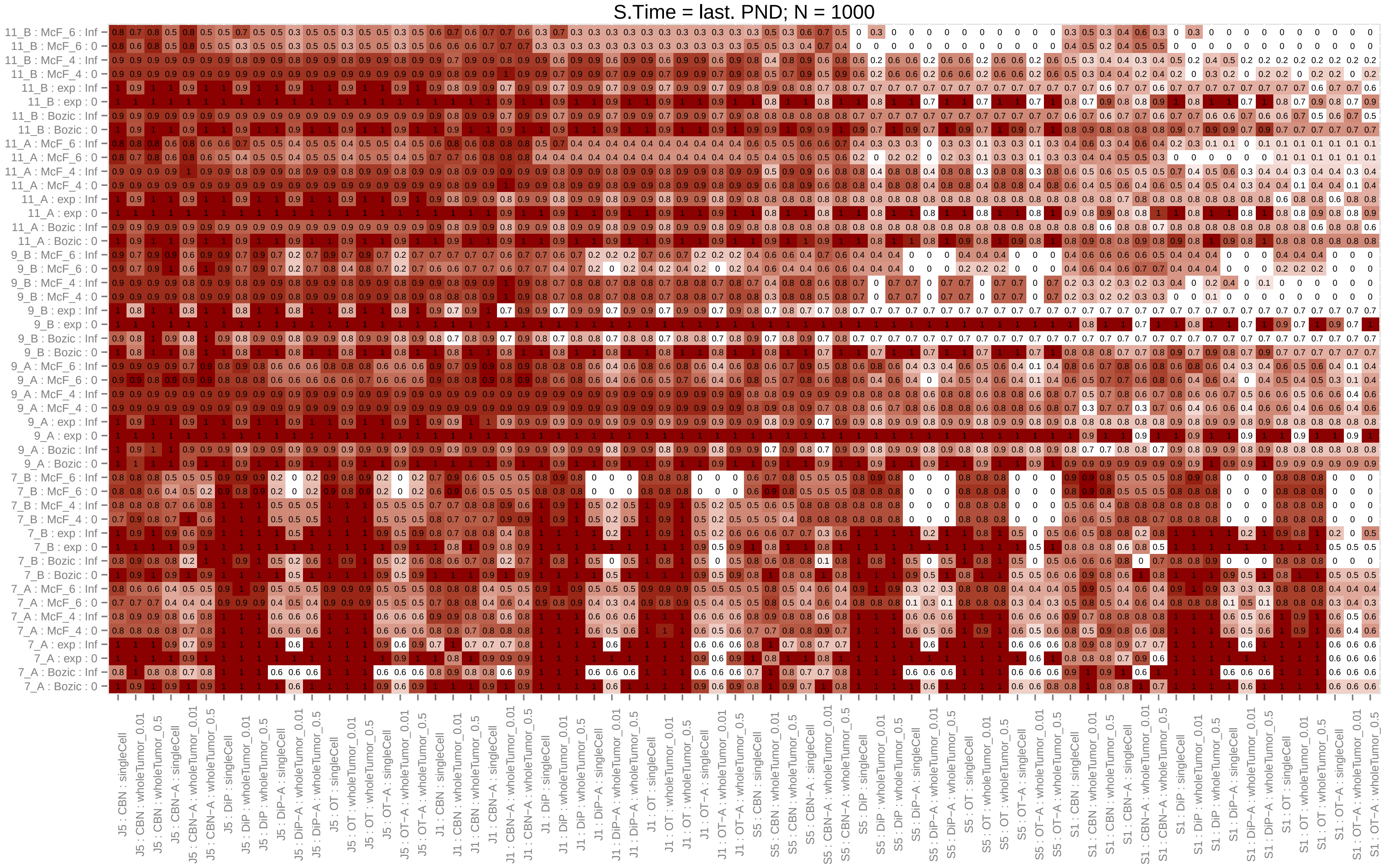

Figure 34: Drivers Unknown, PND, N = 1000, S.Time = last

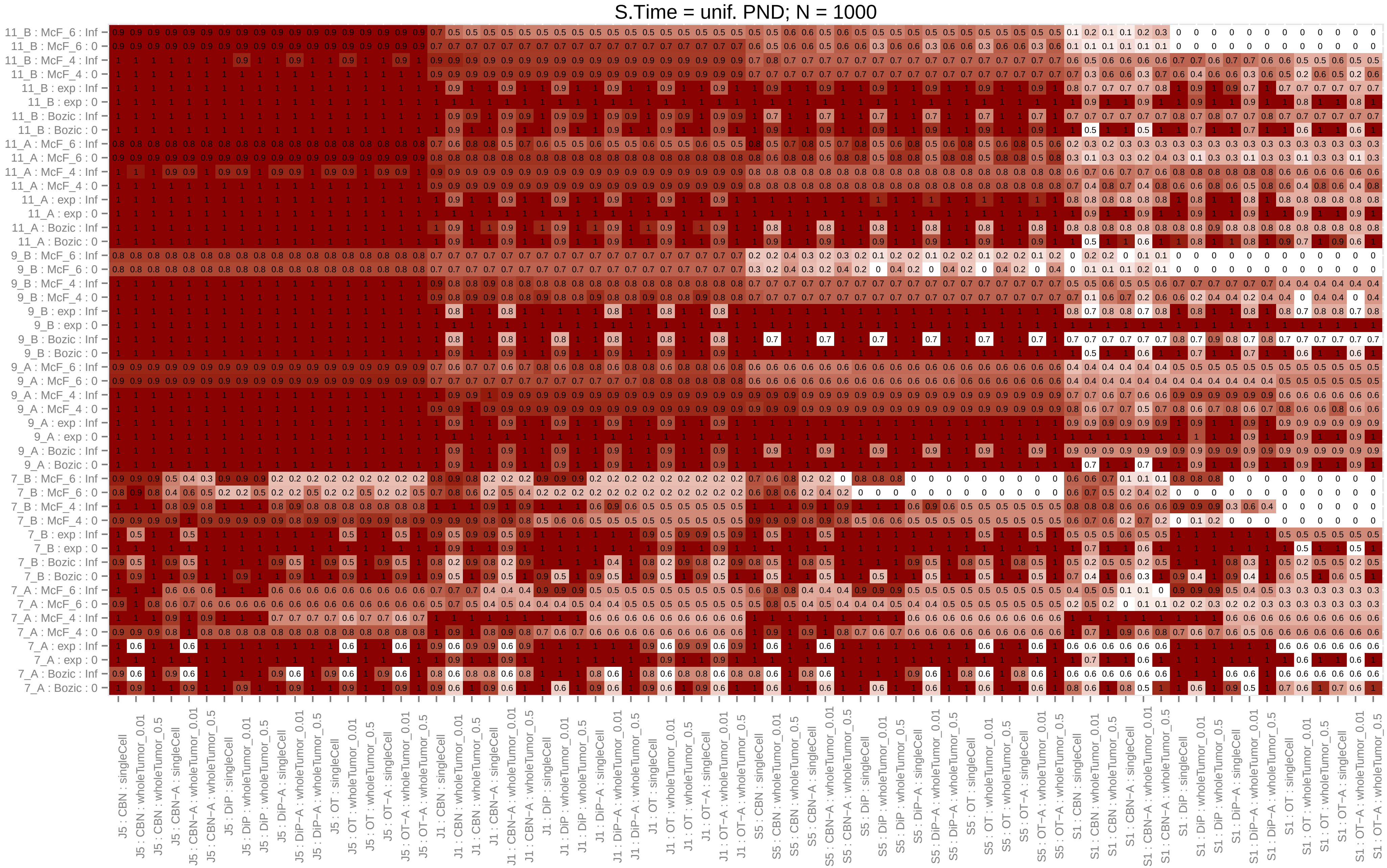

Figure 35: Drivers Unknown, PND, N = 1000, S.Time = unif.

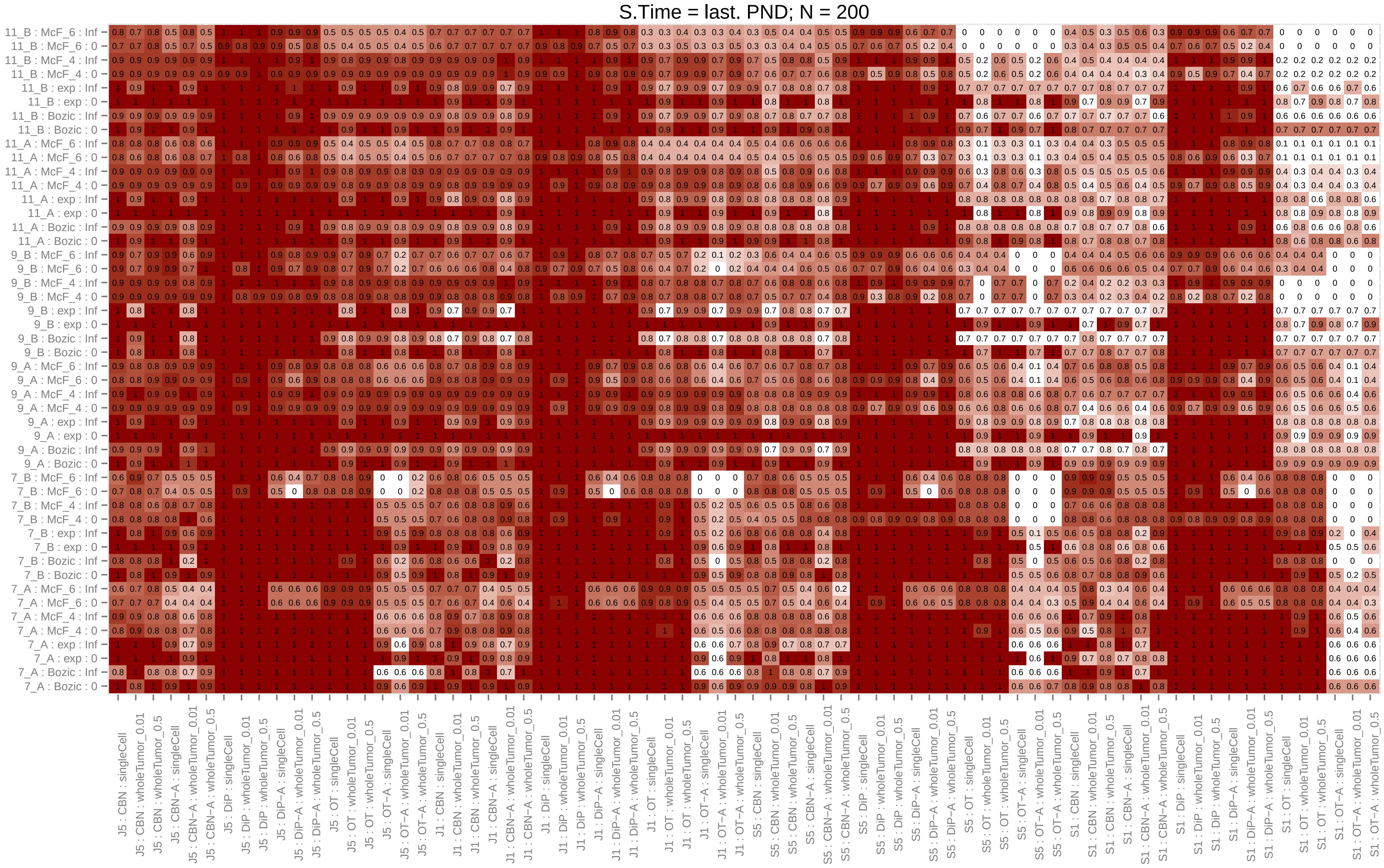

Figure 36: Drivers Unknown, PND, N = 200, S.Time = last

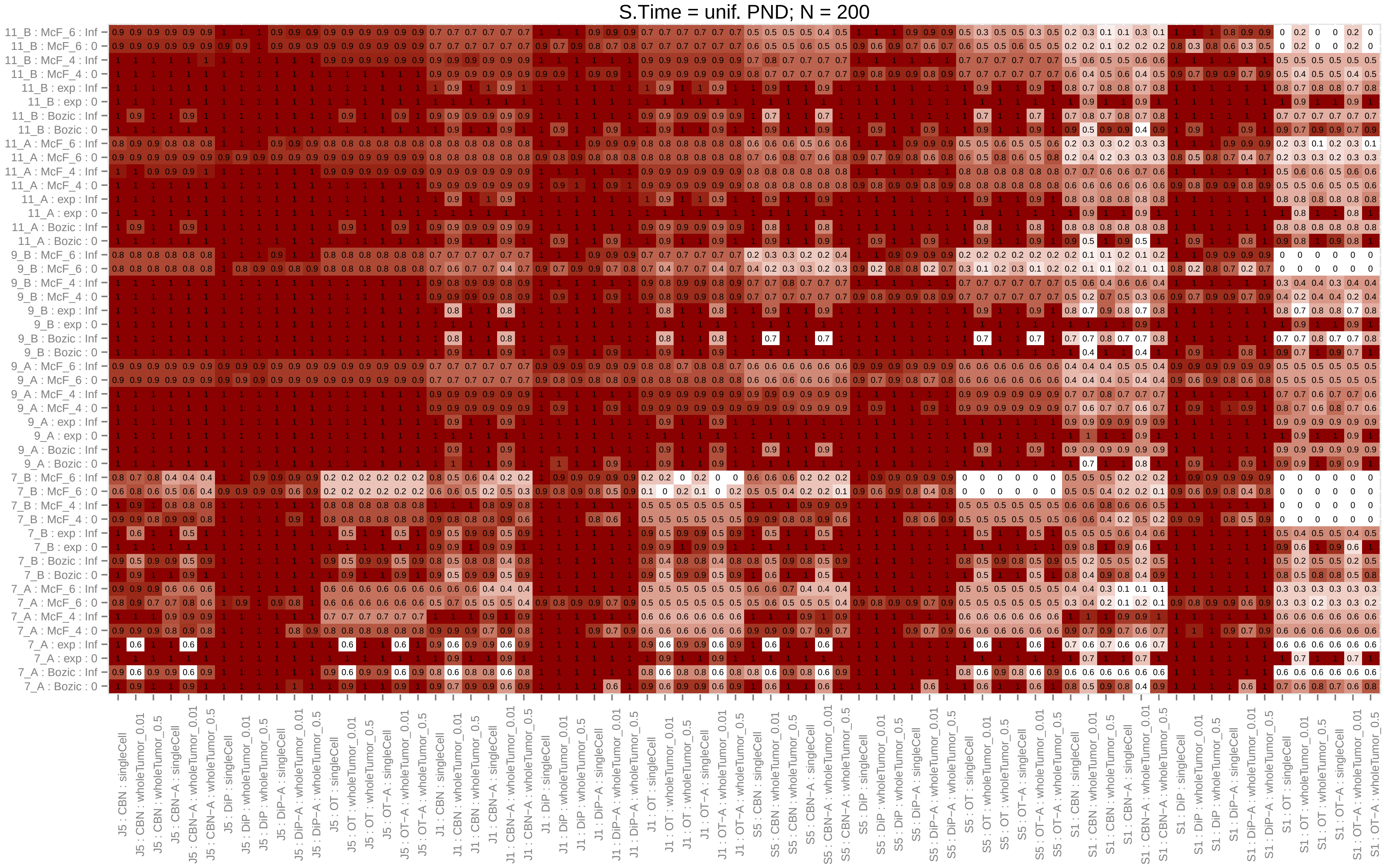

Figure 37: Drivers Unknown, PND, N = 200, S.Time = unif.

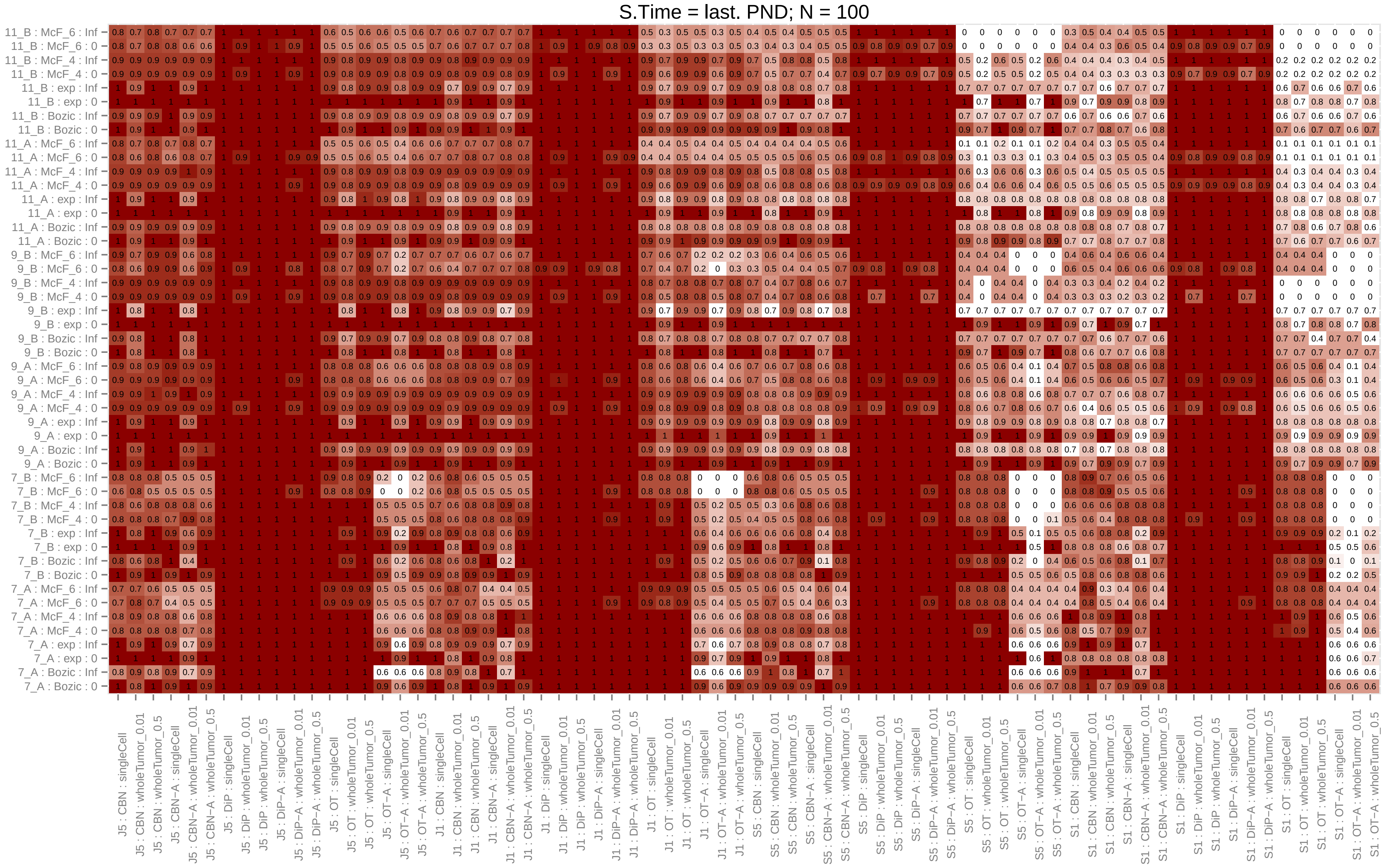

Figure 38: Drivers Unknown, PND, N = 100, S.Time = last

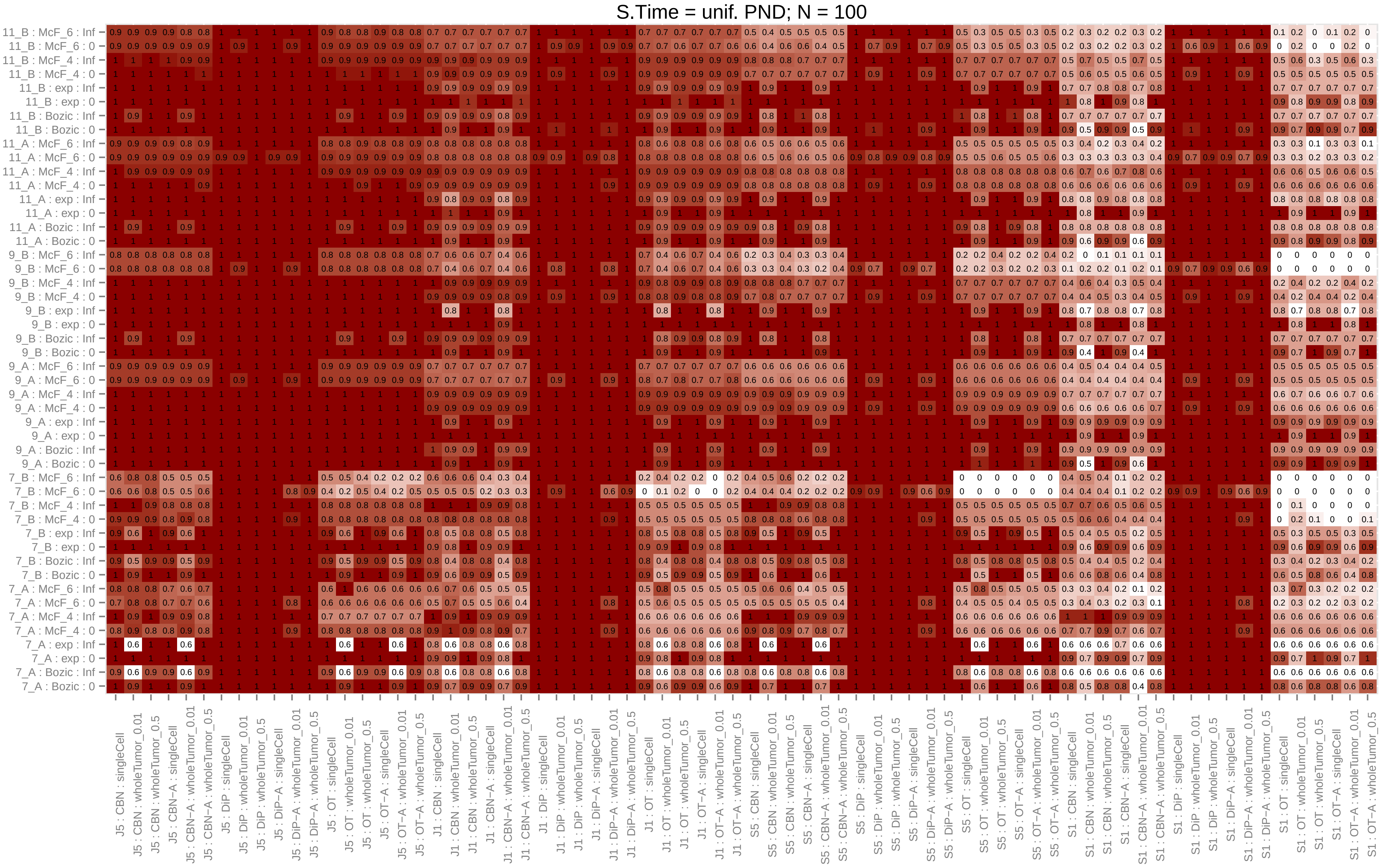

Figure 39: Drivers Unknown, PND, N = 100, S.Time = unif.

3.5 FPF, Drivers Unknown

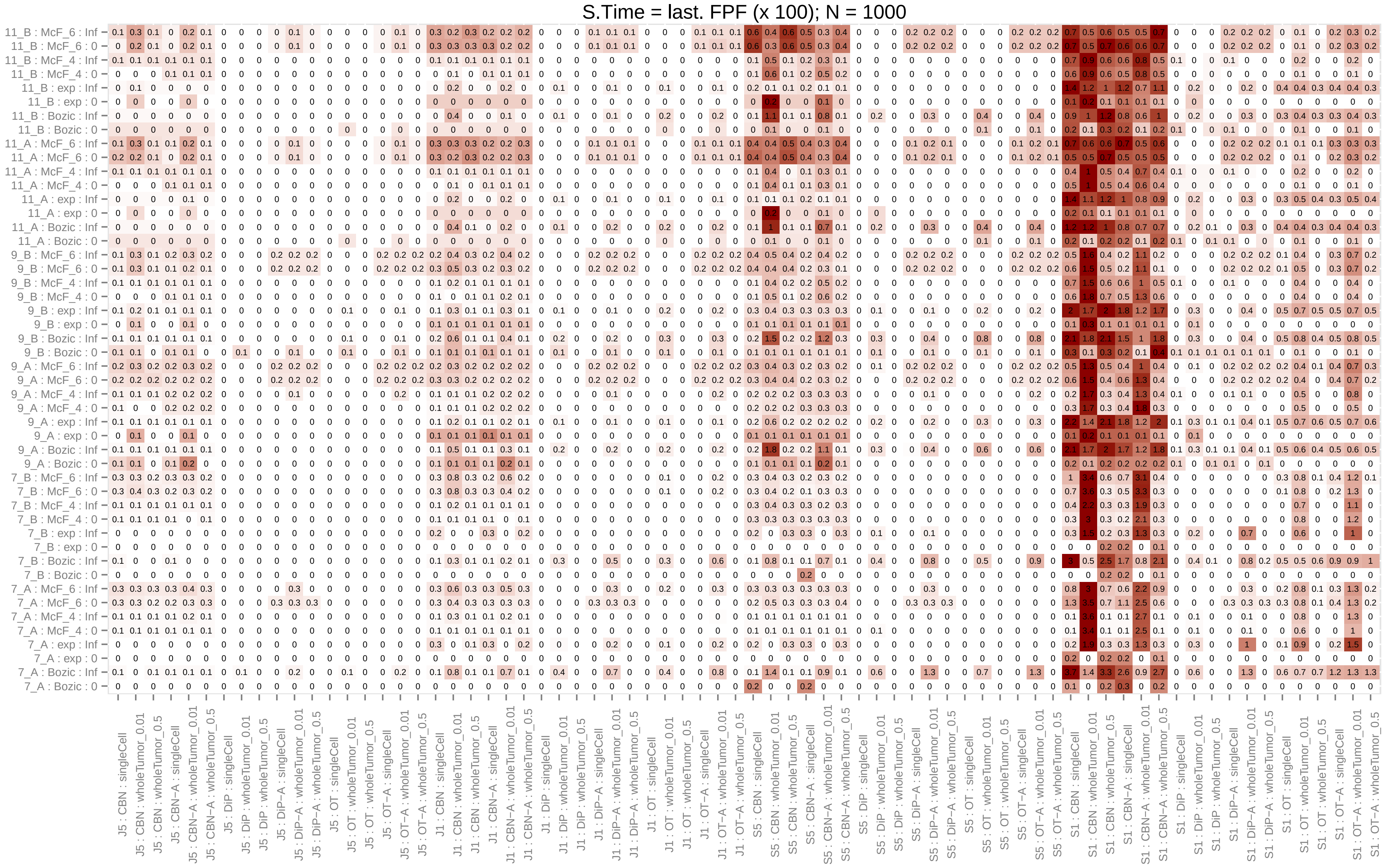

Figure 40: Drivers Unknown, FPF, N = 1000, S.Time = last

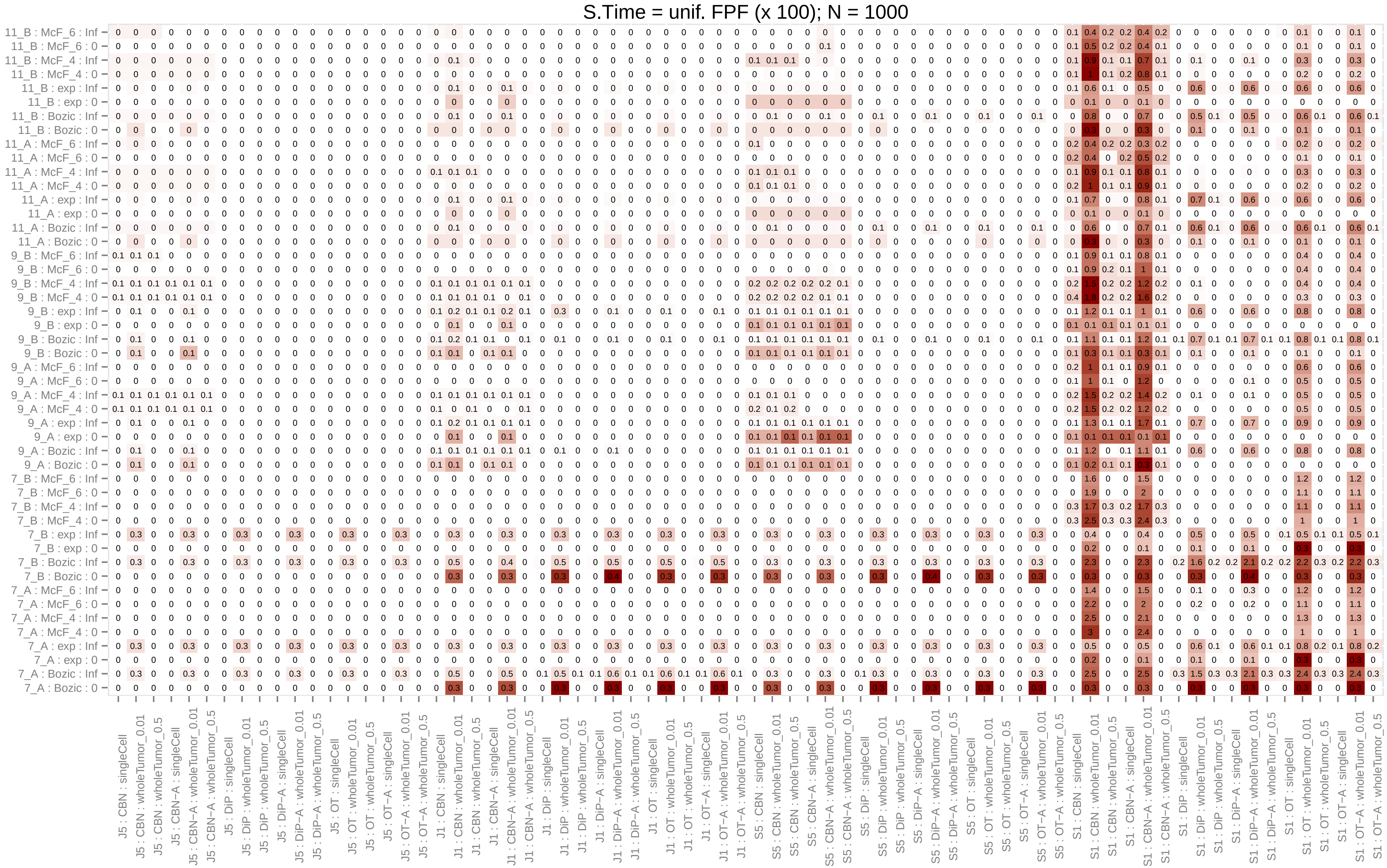

Figure 41: Drivers Unknown, FPF, N = 1000, S.Time = unif.

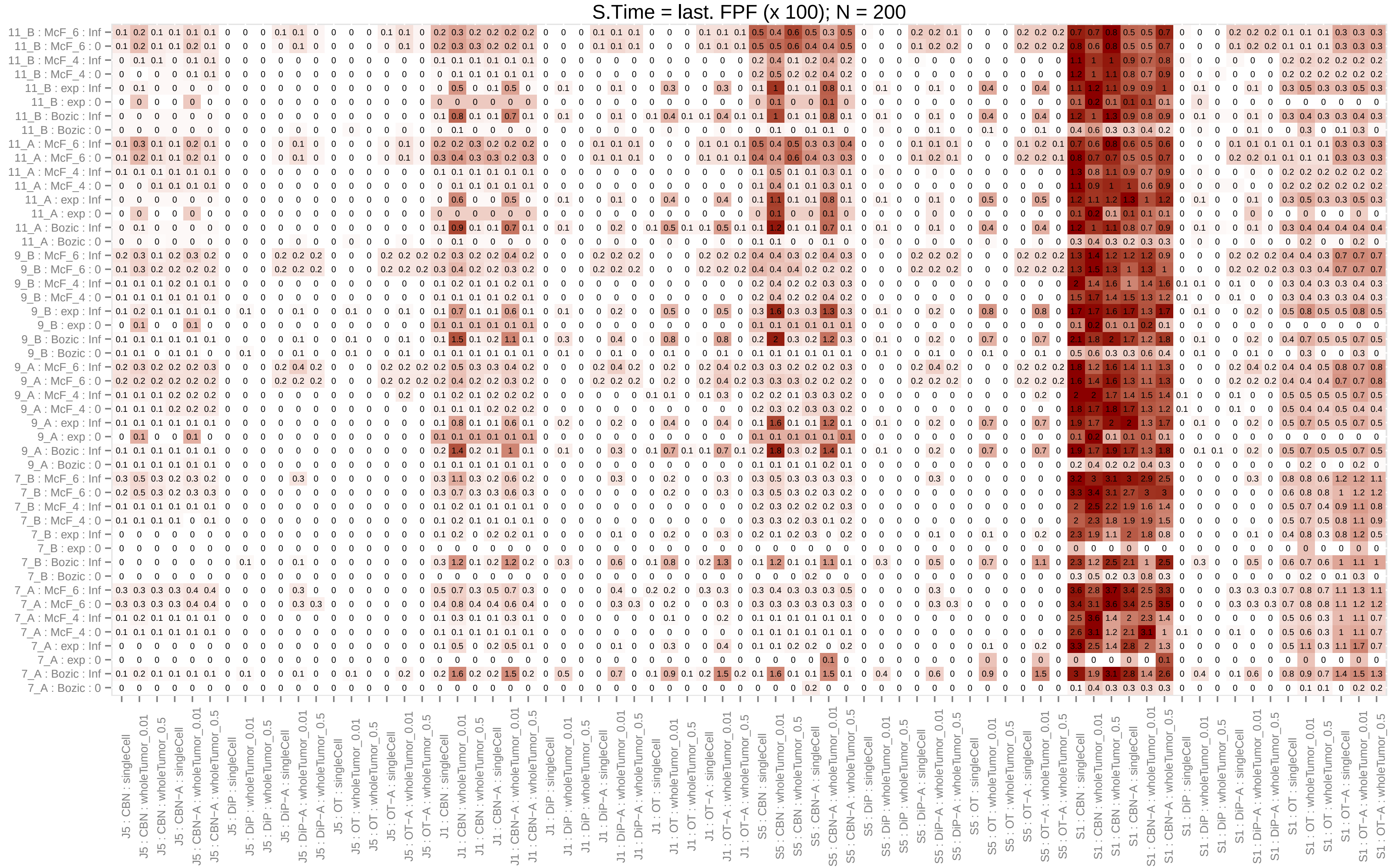

Figure 42: Drivers Unknown, FPF, N = 200, S.Time = last

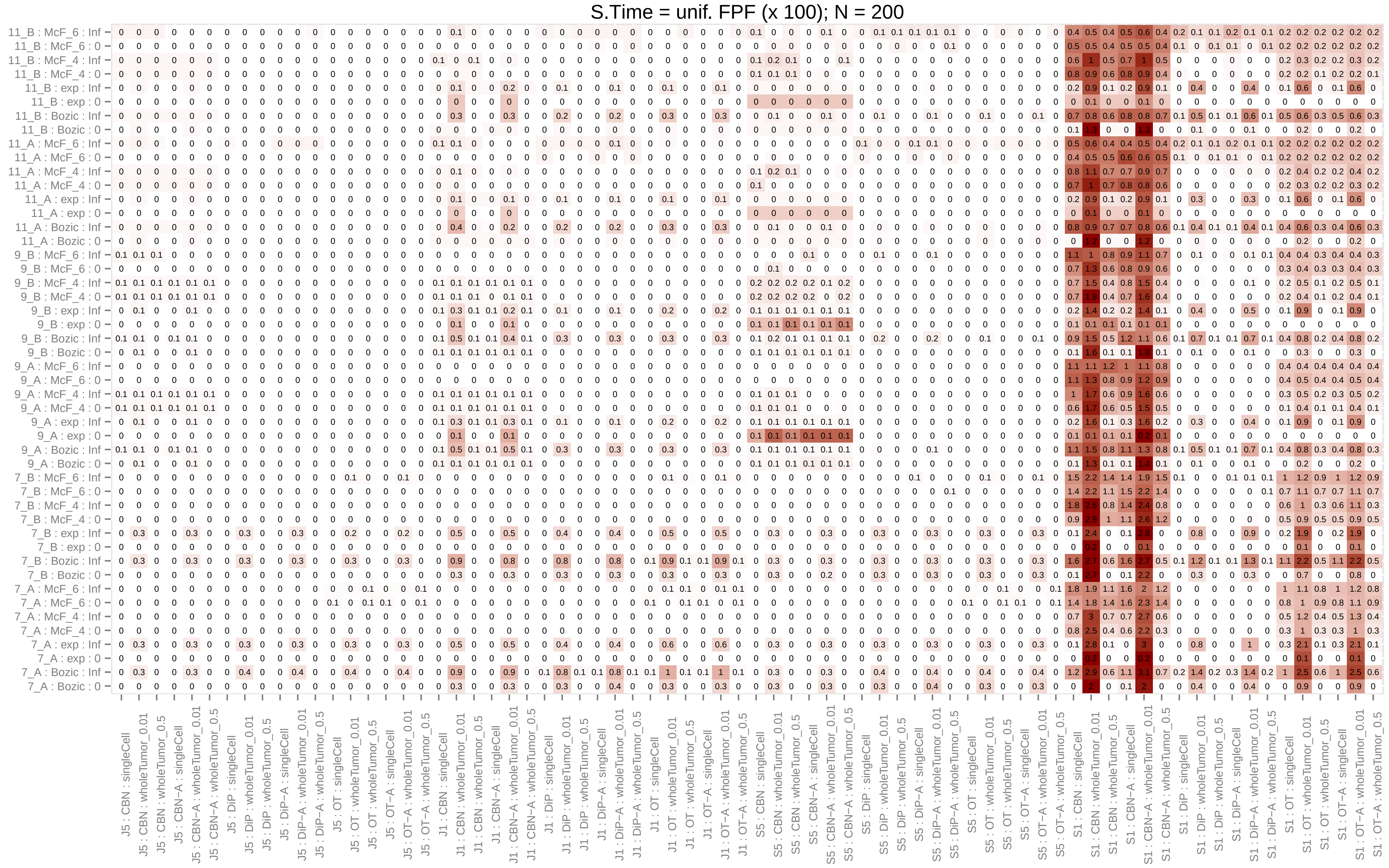

Figure 43: Drivers Unknown, FPF, N = 200, S.Time = unif.

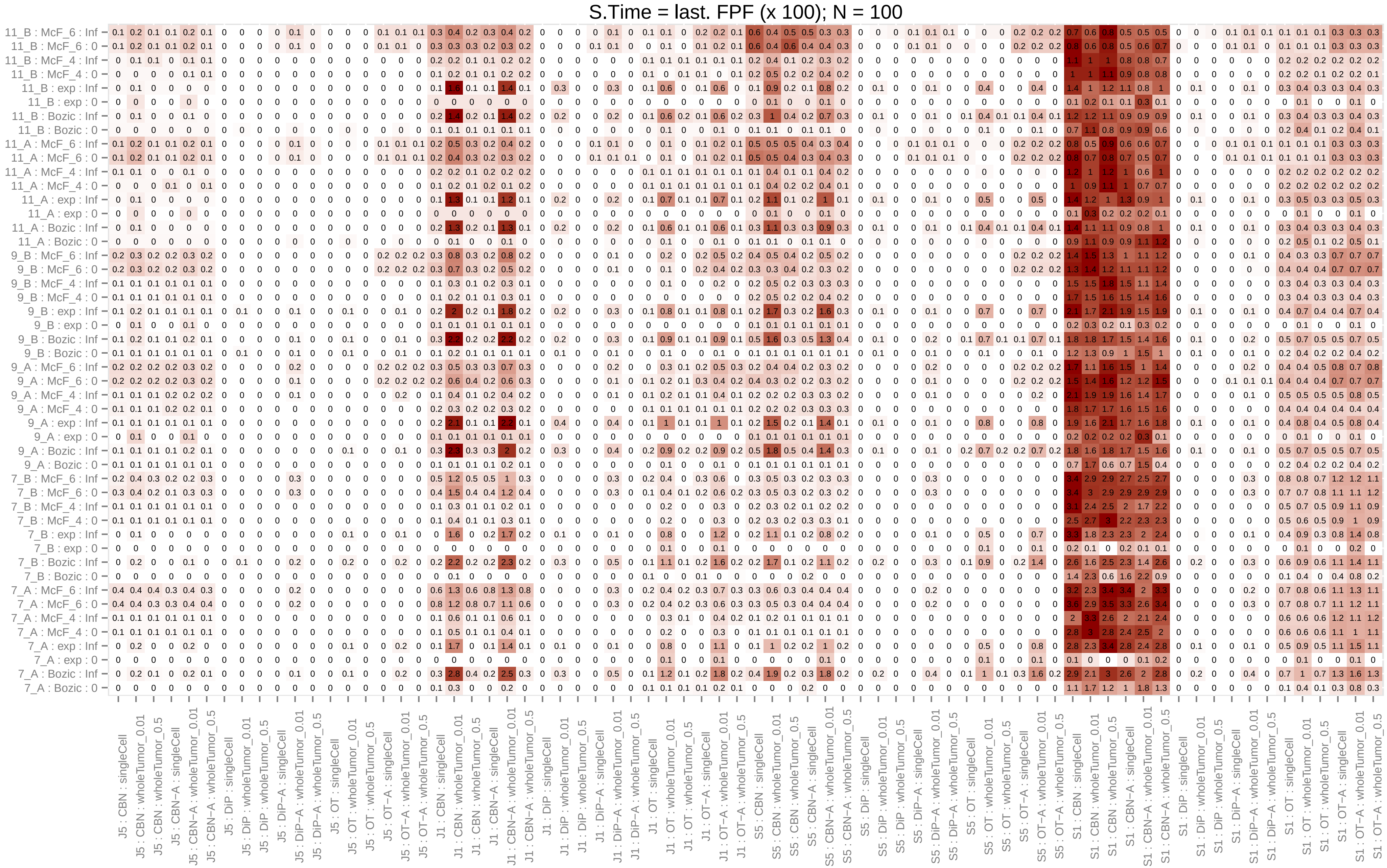

Figure 44: Drivers Unknown, FPF, N = 100, S.Time = last

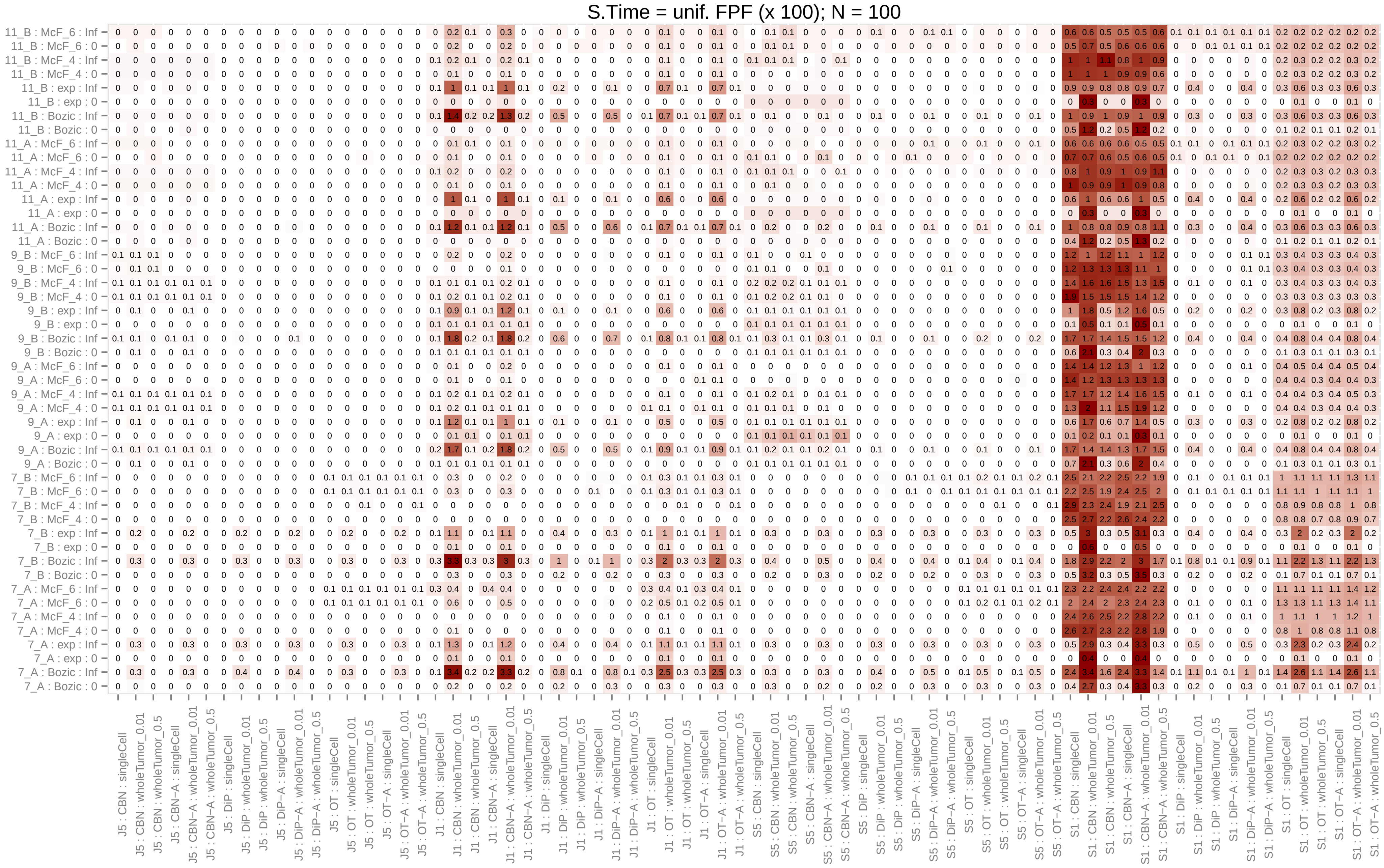

Figure 45: Drivers Unknown, FPF, N = 100, S.Time = unif.
