# Supplementary material for: Identifying restrictions in the order of accumulation of mutations during tumor progression: effects of passengers, evolutionary models, and sampling
Source: BMC Bioinformatics. 2015 Feb 12;16:41. doi: 10.1186/s12859-015-0466-7 (PMC4339747; doi:10.1186/s12859-015-0466-7)
Supplement: Additional file 7 — Estimated conditional probabilities in inferred oncogenetic trees. Figures of reconstructed oncogenetic trees (including inferences using a sample size of 26,000), including the estimates of conditional probabilities. [file 12859_2015_466_MOESM7_ESM.pdf]

Additional file 7. Estimated conditional probabilities in inferred oncogenetic trees.

Supplementary material for "Identifying Restrictions in the Order of Accumulation of Mutations during Tumor Progression: Effects of Passengers, Evolutionary Models, and Sampling"

Ramon Diaz-Uriarte
Dept. Biochemistry, Universidad Autónoma de Madrid
Instituto de Investigaciones Biomédicas "Alberto Sols" (UAM-CSIC)
Madrid, Spain

ramon.diaz@iib.uam.es

rdiaz02@gmail.com

http://ligarto.org/rdiaz

Contents

1 Introduction 3

2 S. Type = single cell 4

3 S. Type = whole tumor, threshold of detection 0.01 8

4 S. Type = single cell with sample size of 26000 12

List of Figures

1 Fitted oncogenetic trees for 11-B. Randomly selected examples of trees reconstructed with method OT, including the estimated transition probabilities, for single cell sampling, sh = Inf, S. Size = 1000, for tree 11-B. 5

2 Fitted oncogenetic trees for 9-B. Randomly selected examples of trees reconstructed with method OT, including the estimated transition probabilities, for single cell sampling, sh = Inf, S. Size = 1000, for tree 9-B. 6

3 Fitted oncogenetic trees for 7-B. Randomly selected examples of trees reconstructed with method OT, including the estimated transition probabilities, for single cell sampling, sh = Inf, S. Size = 1000, for tree 7-B. This figure also illustrates that under S. Time = last, the first node is often missing with OT. 7

4 Fitted oncogenetic trees for 11-B. Randomly selected examples of trees reconstructed with method OT, including the estimated transition probabilities, for whole tumor sampling with threshold 0.01, sh = Inf, S. Size = 1000, for tree 11-B. 9

5 Fitted oncogenetic trees for 9-B. Randomly selected examples of trees reconstructed with method OT, including the estimated transition probabilities, for whole tumor sampling with threshold 0.01, sh = Inf, S. Size = 1000, for tree 9-B. 10

6 Fitted oncogenetic trees for 7-B. Randomly selected examples of trees reconstructed with method OT, including the estimated transition probabilities, for whole tumor sampling with threshold 0.01, sh = Inf, S. Size = 1000, for tree 7-B. None of the edges shown have an observed frequency of occurrence of 1; those are the estimated conditional probabilities. 11

7 Fitted oncogenetic trees for 11-B. Randomly selected examples of trees reconstructed with method OT, including the estimated transition probabilities, for single cell sampling, sh = Inf, S. Size = 26000, for tree 11-B. 13

8 Fitted oncogenetic trees for 9-B. Randomly selected examples of trees reconstructed with method OT, including the estimated transition probabilities, for single cell sampling, sh = Inf, S. Size = 26000, for tree 9-B. 14

9 Fitted oncogenetic trees for 7-B. Randomly selected examples of trees reconstructed with method OT, including the estimated transition probabilities, for single cell sampling, sh = Inf, S. Size = 26000, for tree 7-B. This figure also illustrates that under S. Time = last, the first node is often missing with OT. 15

1 Introduction

The first three figures show reconstructed oncogenetic trees (selected randomly from the set of 20 trees) for graphs 11-B, 9-B and 7-B, and include the estimated conditional probabilities returned by package Oncotree (Szabo and Pappas, 2013). All of the figures correspond to S. type = single cell and $s_h = Inf$, since this is the sampling type and s_h that should produce clone frequencies that are most comparable to those obtainable from simulating directly from the oncogenetic trees themselves. S.Size is 1000, since this is the sample size that produces the best trees and the purpose of these figures is to illustrate variation in fitted conditional probabilities for the best trees.

The next set of three figures are similar to the first ones, but instead of using the results from S. Type = single cell, I have used those from S. Type = whole tumor, with threshold of 0.01.

After those, we repeat the figures for S. Type = single cell, but using all data for each scenario, so instead of showing one randomly selected tree for S. Size = 1000, we put together all data sets, and reconstruct the tree using a total of 26000 samples ($= 20 * (1000 + 200 + 100)$). This allows us to minimize variability in the estimates.

2 S. Type = single cell

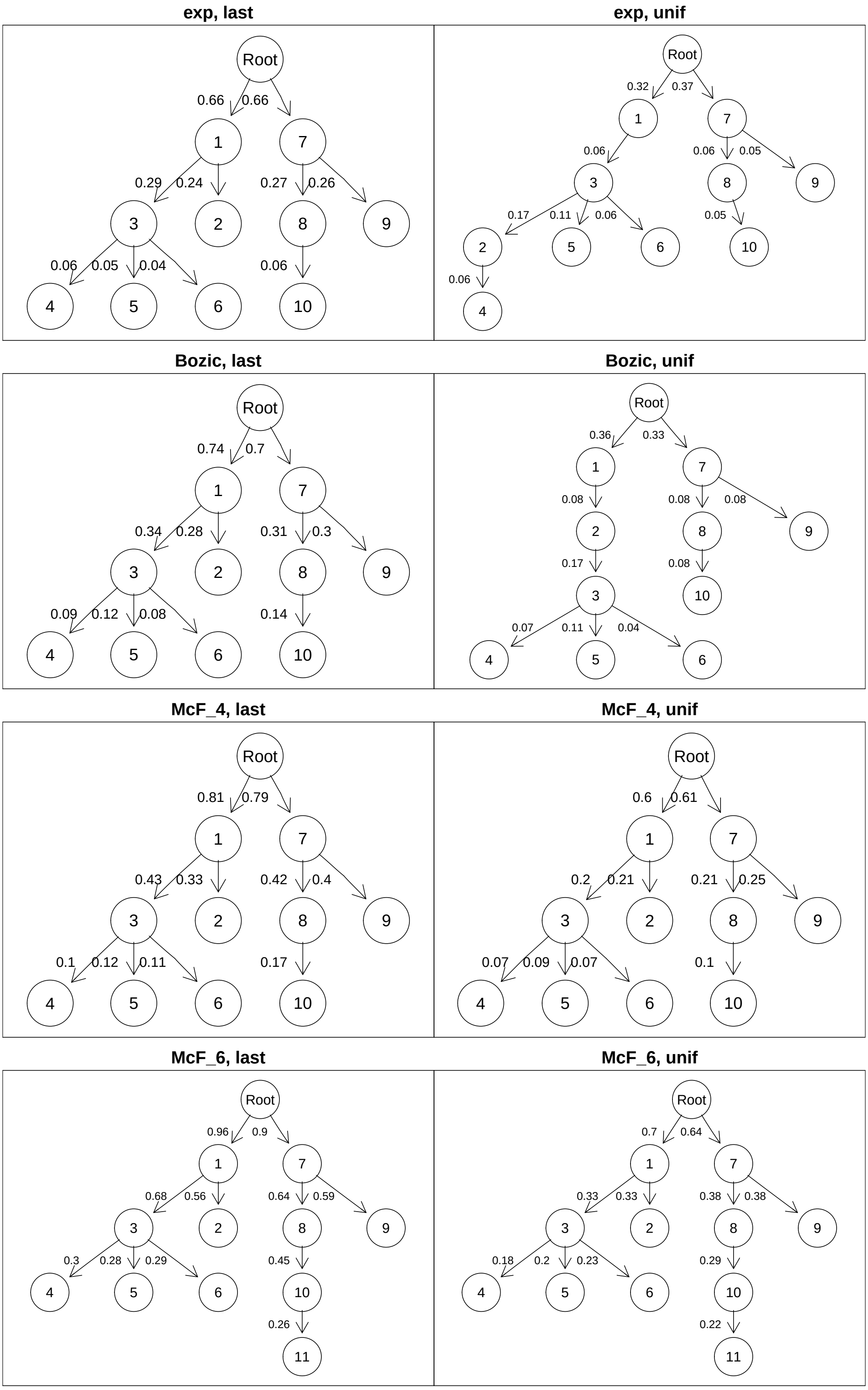

Figure 1: Fitted oncogenetic trees for 11-B. Randomly selected examples of trees reconstructed with method OT, including the estimated transition probabilities, for single cell sampling, sh = Inf, S. Size = 1000, for tree 11-B.

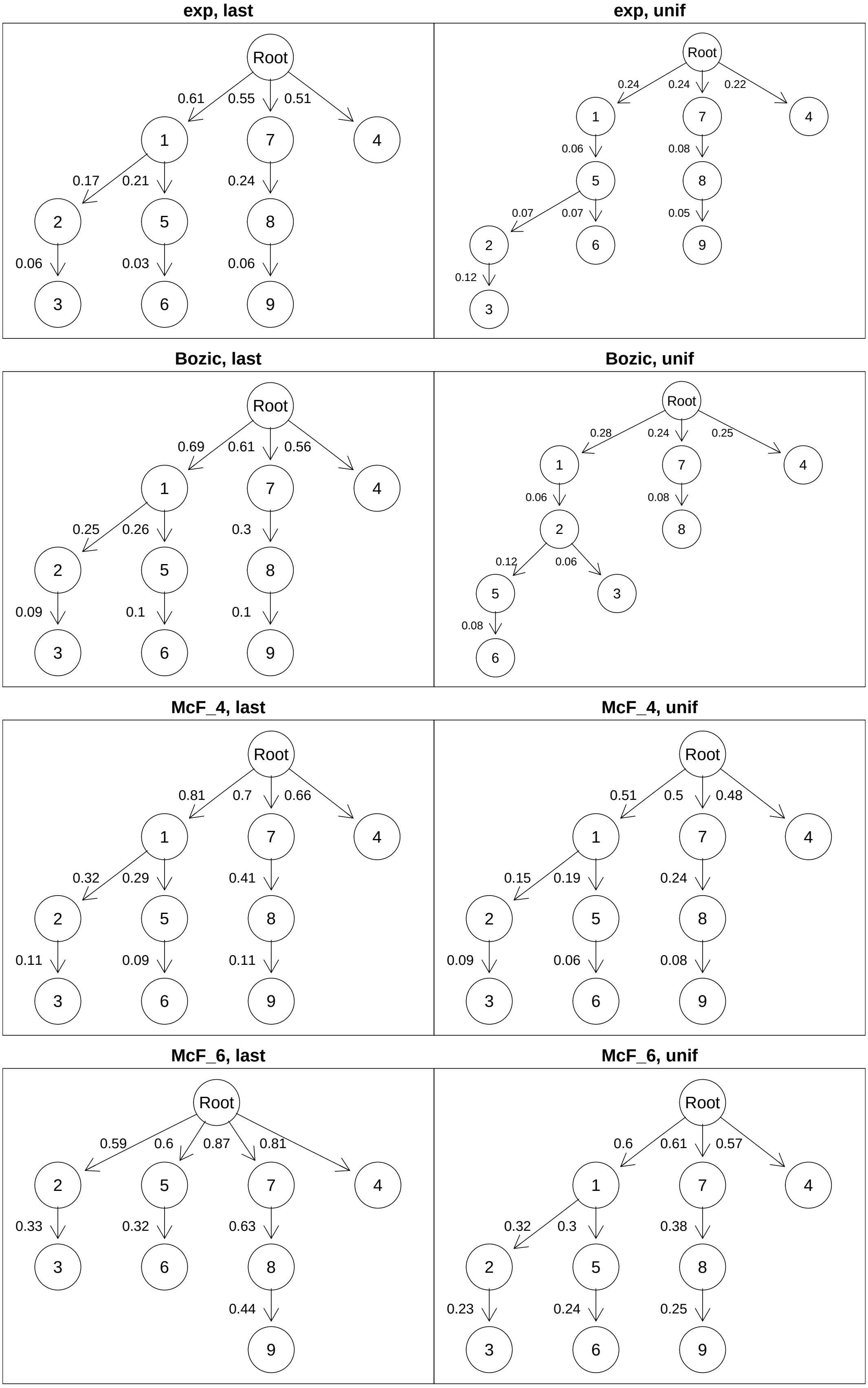

Figure 2: Fitted oncogenetic trees for 9-B. Randomly selected examples of trees reconstructed with method OT, including the estimated transition probabilities, for single cell sampling, sh = Inf, S. Size = 1000, for tree 9-B.

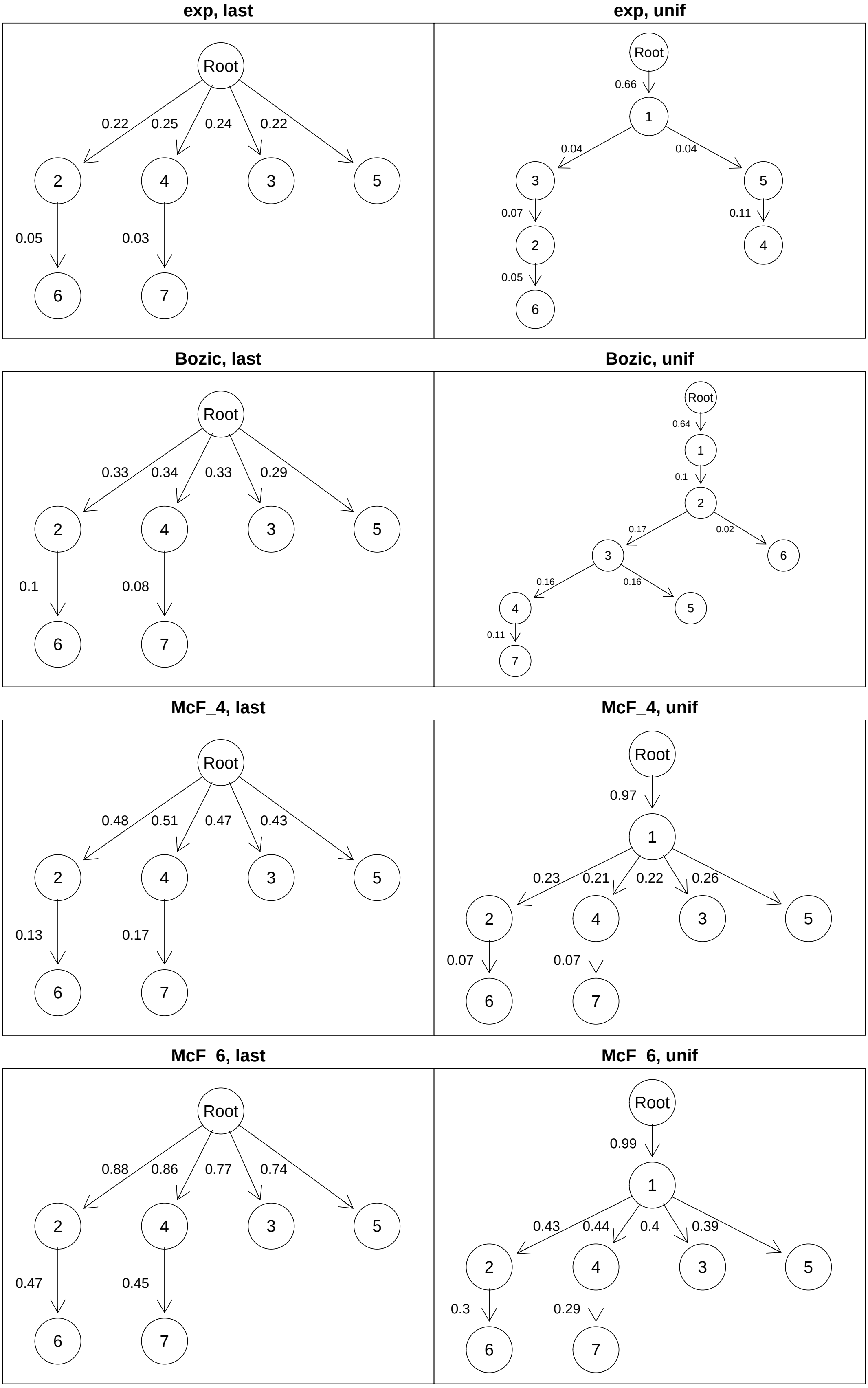

Figure 3: Fitted oncogenetic trees for 7-B. Randomly selected examples of trees reconstructed with method OT, including the estimated transition probabilities, for single cell sampling, sh = Inf, S. Size = 1000, for tree 7-B. This figure also illustrates that under S. Time = last, the first node is often missing with OT.

3 S. Type = whole tumor, threshold of detection 0.01

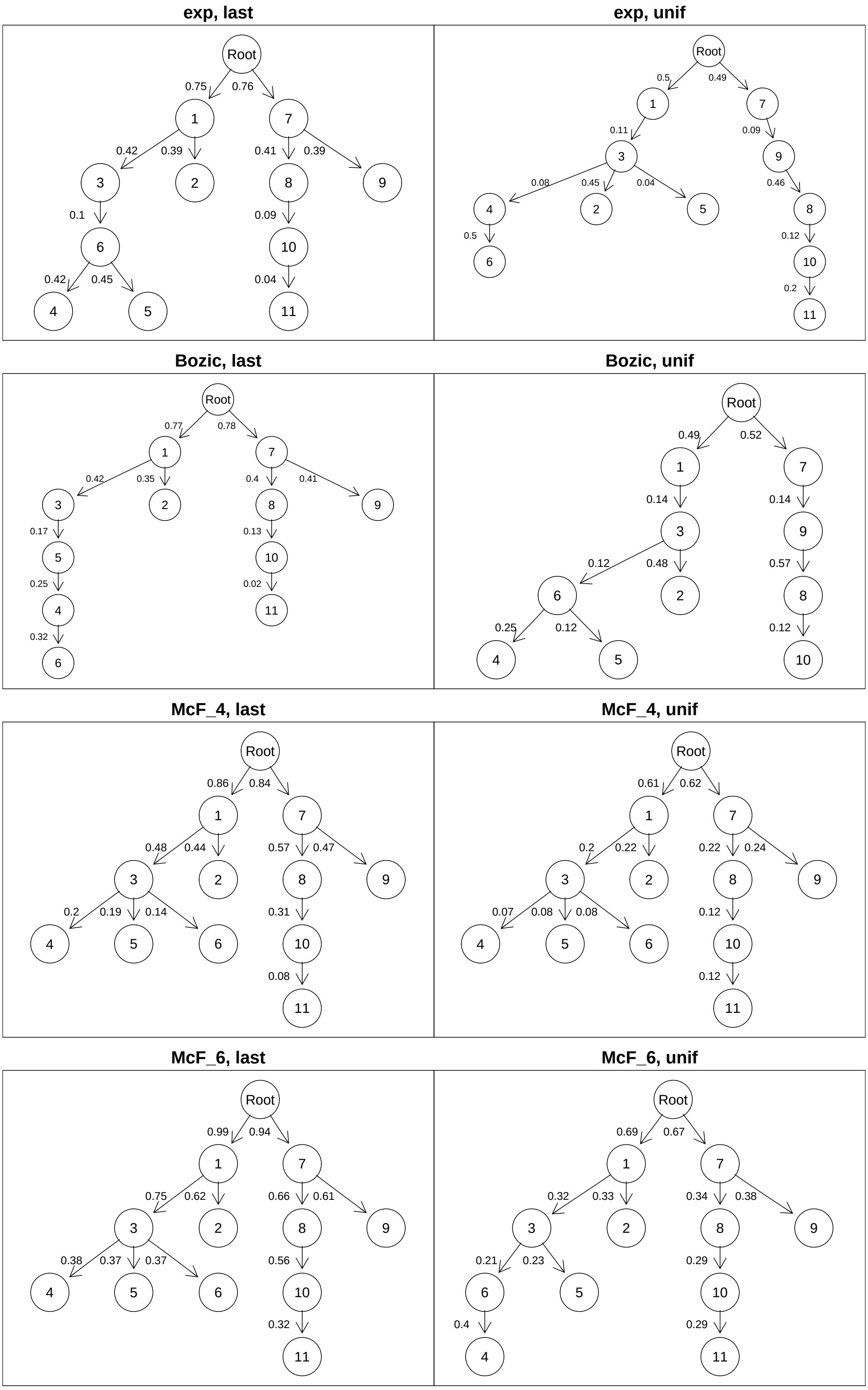

Figure 4: Fitted oncogenetic trees for 11-B. Randomly selected examples of trees reconstructed with method OT, including the estimated transition probabilities, for whole tumor sampling with threshold 0.01, sh = Inf, S. Size = 1000, for tree 11-B.

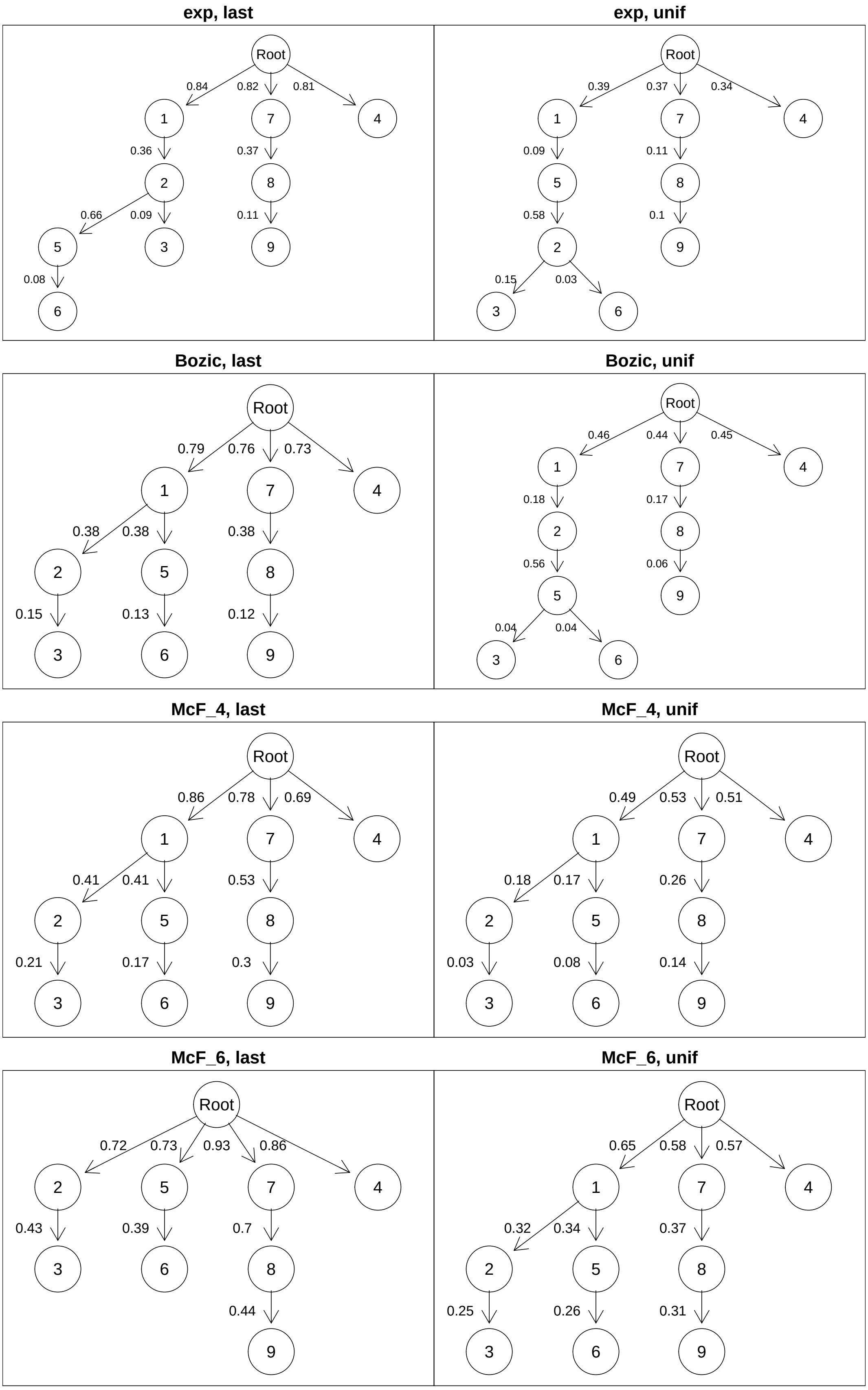

Figure 5: Fitted oncogenetic trees for 9-B. Randomly selected examples of trees reconstructed with method OT, including the estimated transition probabilities, for whole tumor sampling with threshold 0.01, sh = Inf, S. Size = 1000, for tree 9-B.

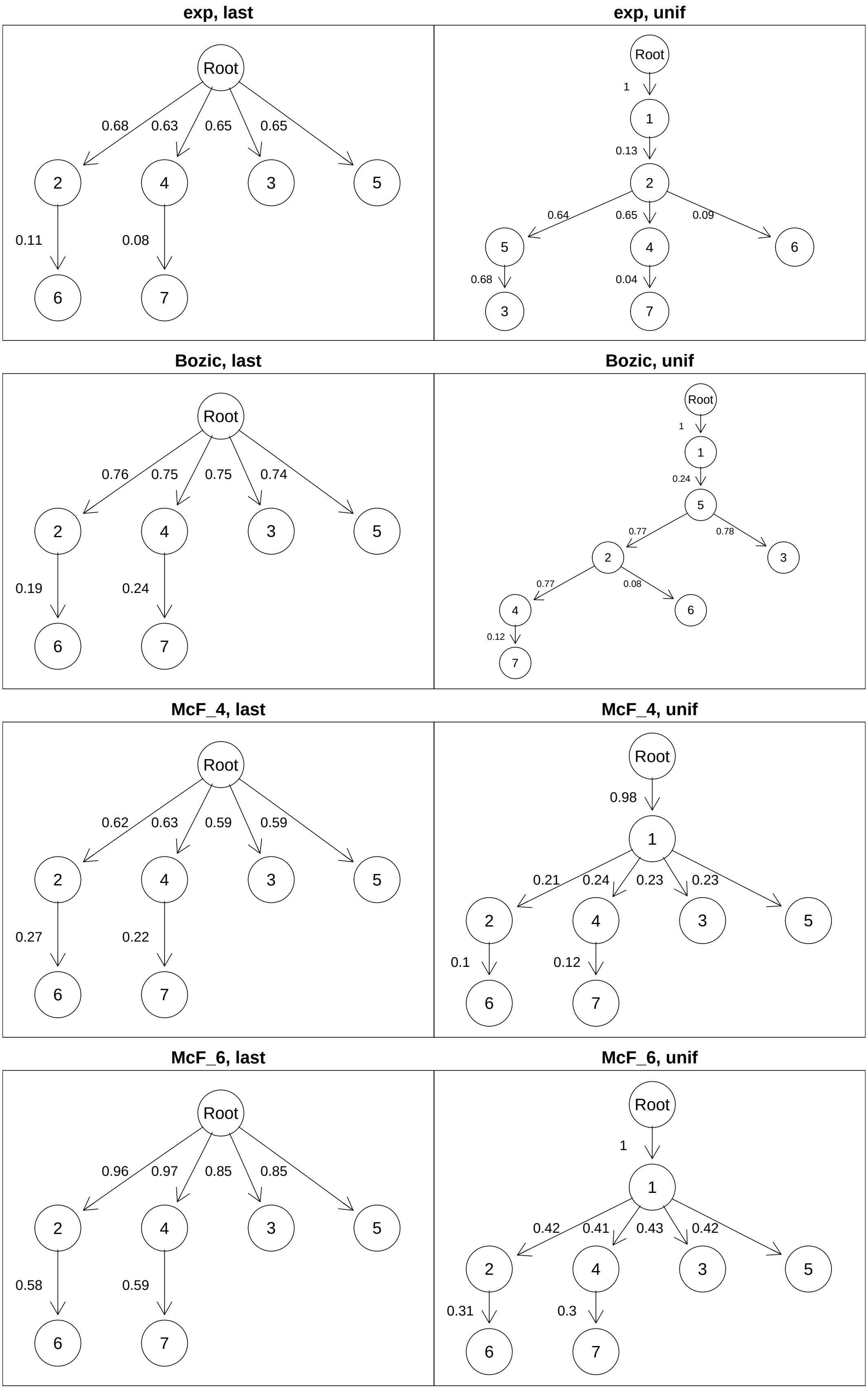

Figure 6: Fitted oncogenetic trees for 7-B. Randomly selected examples of trees reconstructed with method OT, including the estimated transition probabilities, for whole tumor sampling with threshold 0.01, sh = Inf, S. Size = 1000, for tree 7-B. None of the edges shown have an observed frequency of occurrence of 1; those are the estimated conditional probabilities.

4 S. Type = single cell with sample size of 26000

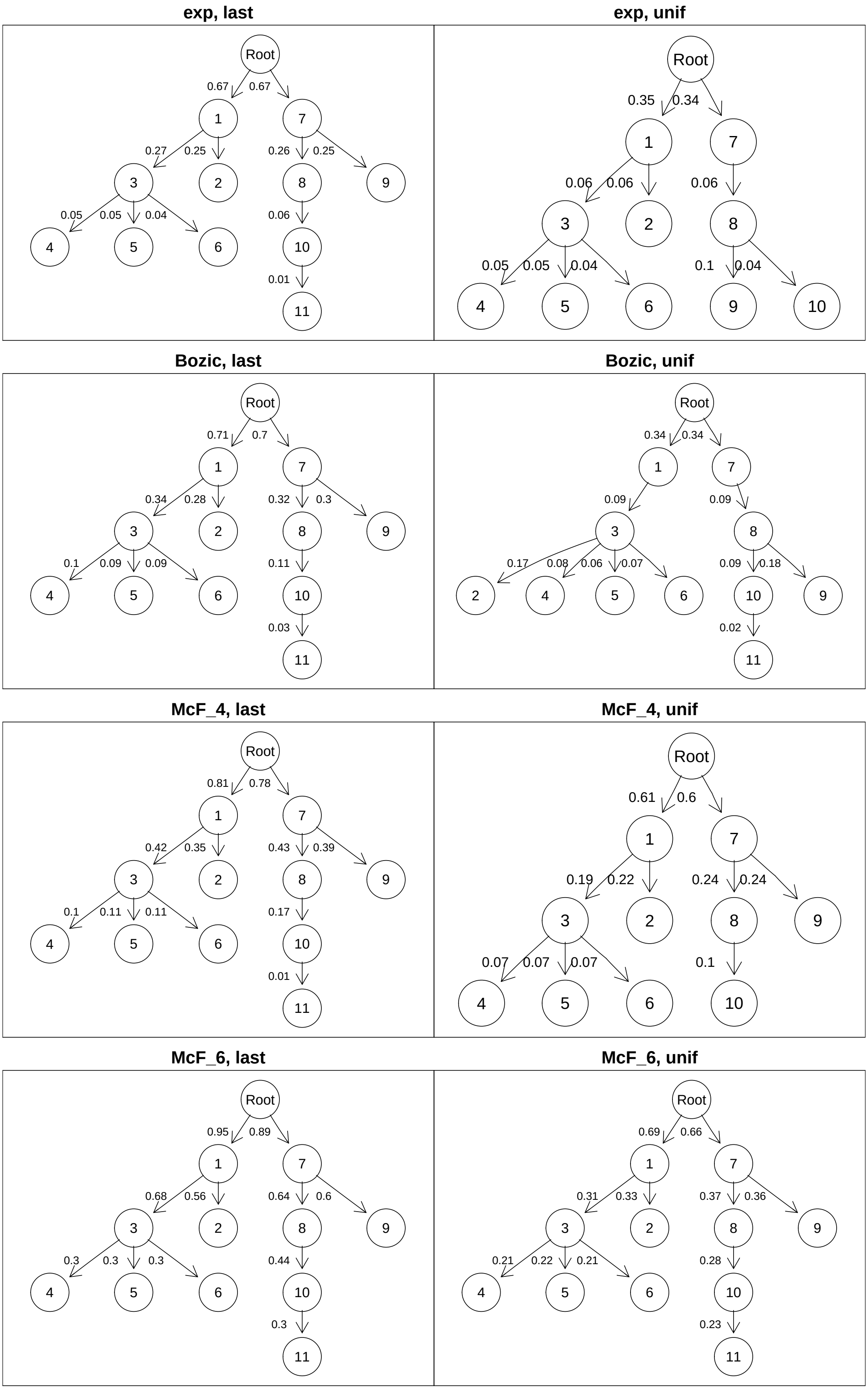

Figure 7: Fitted oncogenetic trees for 11-B. Randomly selected examples of trees reconstructed with method OT, including the estimated transition probabilities, for single cell sampling, sh = Inf, S. Size = 26000, for tree 11-B.

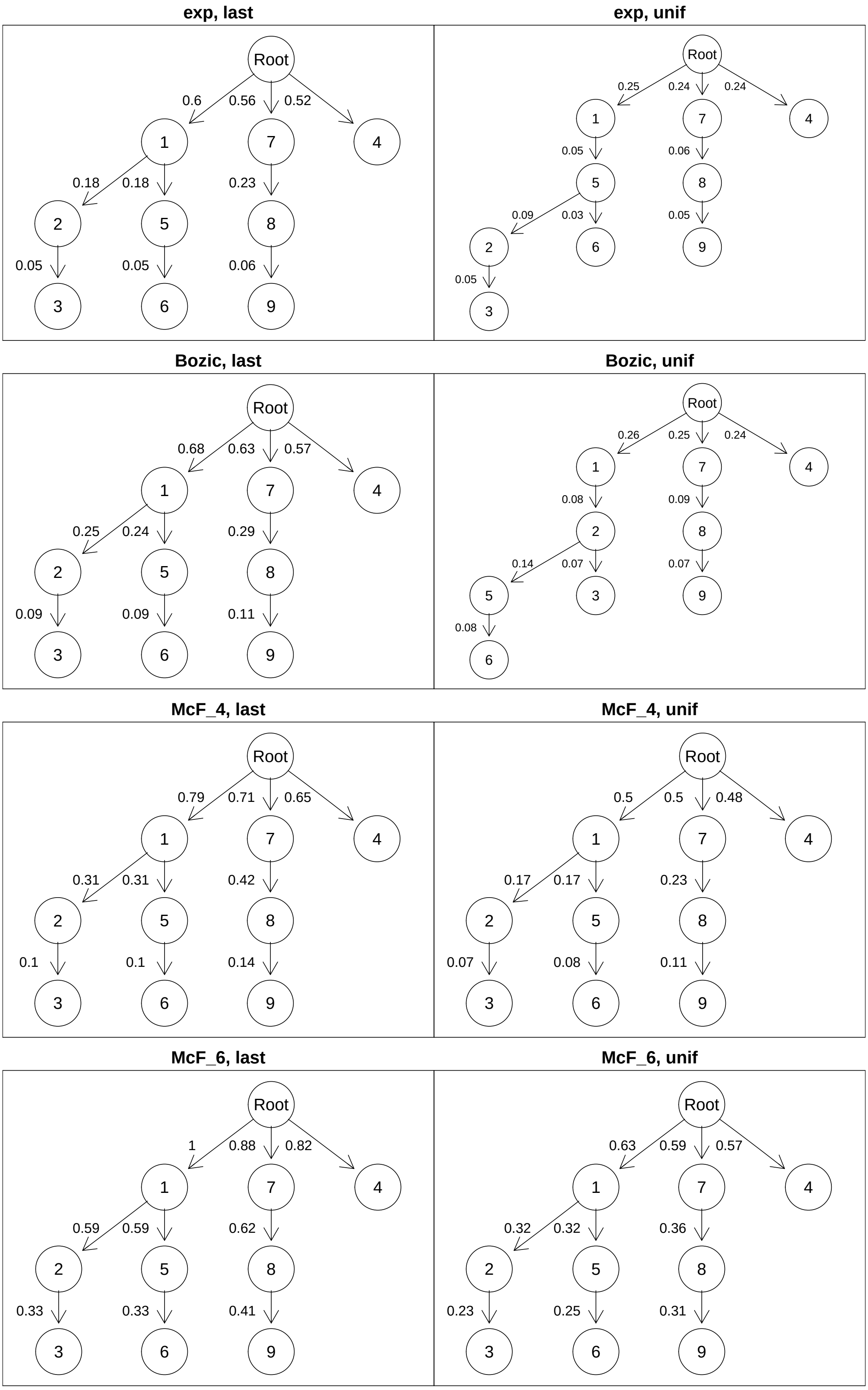

Figure 8: Fitted oncogenetic trees for 9-B. Randomly selected examples of trees reconstructed with method OT, including the estimated transition probabilities, for single cell sampling, sh = Inf, S. Size = 26000, for tree 9-B.

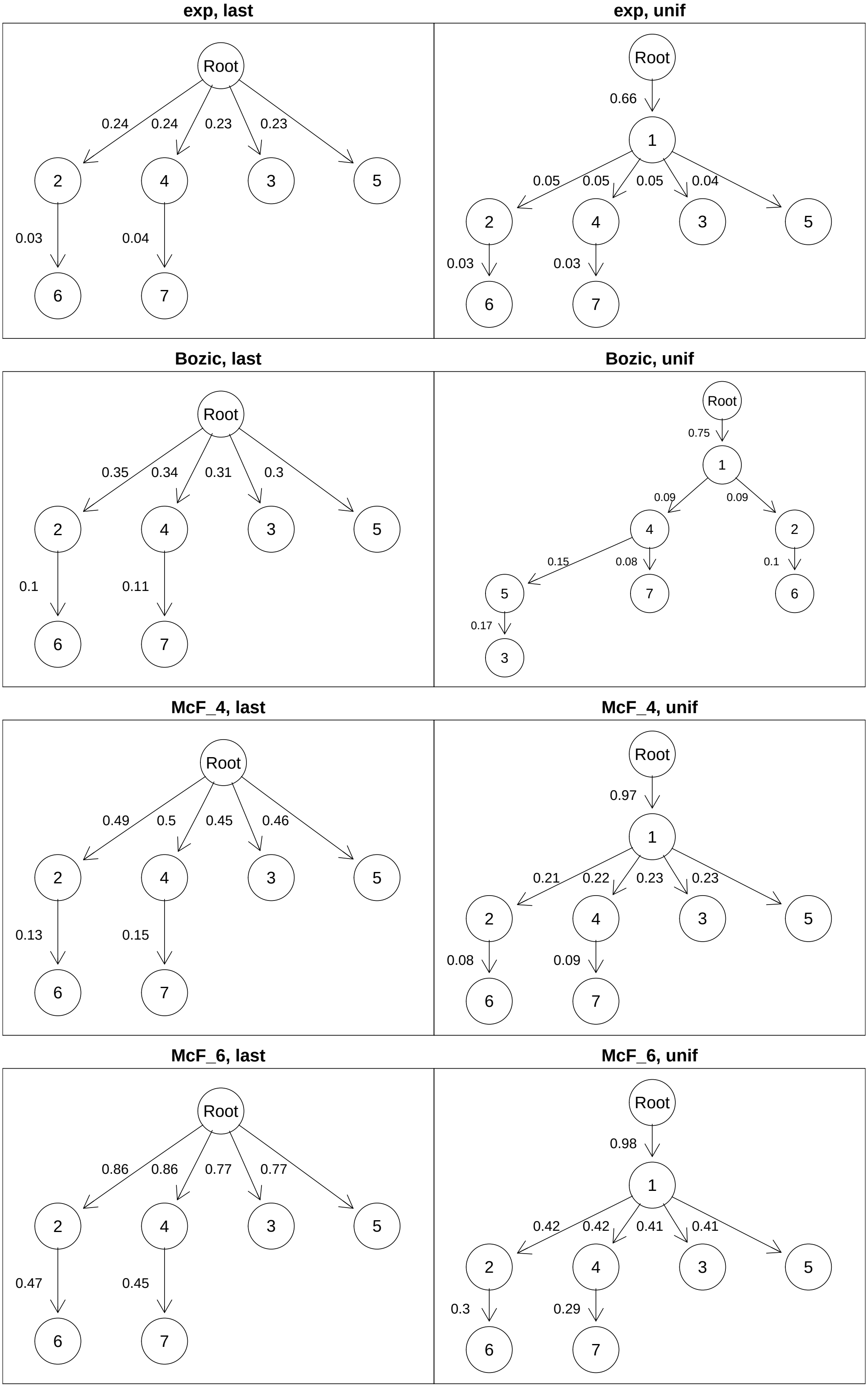

Figure 9: Fitted oncogenetic trees for 7-B. Randomly selected examples of trees reconstructed with method OT, including the estimated transition probabilities, for single cell sampling, sh = Inf, S. Size = 26000, for tree 7-B. This figure also illustrates that under S. Time = last, the first node is often missing with OT.

References

Szabo, A. and Pappas, L. (2013). Oncotree: Estimating oncogenetic trees.
